# Minimalist revision and description of 403 new species in 11 subfamilies of Costa Rican braconid parasitoid wasps, including host records for 219 species

**DOI:** 10.3897/zookeys.1013.55600

**Published:** 2021-02-02

**Authors:** Michael J. Sharkey, Daniel H. Janzen, Winnie Hallwachs, Eric G. Chapman, M. Alex Smith, Tanya Dapkey, Allison Brown, Sujeevan Ratnasingham, Suresh Naik, Ramya Manjunath, Kate Perez, Megan Milton, Paul Hebert, Scott R. Shaw, Rebecca N. Kittel, M. Alma Solis, Mark A. Metz, Paul Z. Goldstein, John W. Brown, Donald L.J. Quicke, C. van Achterberg, Brian V. Brown, John M. Burns

**Affiliations:** 1 The Hymenoptera Institute, 116 Franklin Ave., Redlands, CA, 92373, USA The Hymenoptera Institute Redlands United States of America; 2 Department of Biology, University of Pennsylvania, Philadelphia, PA 19104-6018, USA University of Pennsylvania Philadelphia United States of America; 3 Department of Entomology, University of Kentucky, Lexington, KY 40546-0091, USA University of Kentucky Lexington United States of America; 4 Department of Integrative Biology, University of Guelph and Biodiversity Institute of Ontario, Guelph, Canada University of Guelph Guelph Canada; 5 Academy of Natural Sciences, 1900 Benjamin Franklin Parkway, Philadelphia, PA 19103, USA Academy of Natural Sciences Philadelphia United States of America; 6 Department of Ecosystem Science, University of Wyoming, 1000 East University Avenue, Laramie, Wyoming 82071, USA University of Wyoming Laramie United States of America; 7 Museum Wiesbaden, Hessisches Landesmuseum für Kunst und Natur, Friedrich-Ebert-Allee 2, 65185 Wiesbaden, Germany Hessisches Landesmuseum für Kunst und Natur Wiesbaden Germany; 8 Systematic Entomology Laboratory, Beltsville Agriculture Research Center, Agricultural Research Service, U.S. Department of Agriculture, c/o National Museum Natural History, MRC 168, Smithsonian Institution, P.O. Box 37012, Washington, DC, 20013-7012, USA U.S. Department of Agriculture Washington United States of America; 9 Department of Biology, Faculty of Life Sciences, Chulalongkorn University, Bangkok, Thailand National Museum of Natural History Washington United States of America; 10 Naturalis Biodiversity Center, Postbus 9517, 2300 RA Leiden, The Netherlands Chulalongkorn University Bangkok Thailand; 11 Department of Entomology, Natural History Museum of Los Angeles County, 900 Exposition Boulevard, Los Angeles, CA, 90007, USA Naturalis Biodiversity Center Leiden Netherlands; 12 Division of Entomology, PO Box 37012 12. National Museum of Natural History E515 MRC127, Washington, DC 20013-7012, USA Natural History Museum of Los Angeles County Los Angeles United States of America

**Keywords:** Accelerated taxonomy, Agathidinae, BIN code, BioAlfa, Braconidae, Braconinae, caterpillar, Cheloninae, COI barcode, conservation, DNA barcode, Homolobinae, Hormiinae, Hymenoptera, Ichneumonoidea, Ichneutinae, Lepidoptera, Macrocentrinae, parasitoid host associations, Proteropinae, Rogadinae, Rhysipolinae, tri-trophic interaction, tropical

## Abstract

Three new genera are described: *Michener* (Proteropinae), *Bioalfa* (Rogadinae), and *Hermosomastax* (Rogadinae). Keys are given for the New World genera of the following braconid subfamilies: Agathidinae, Braconinae, Cheloninae, Homolobinae, Hormiinae, Ichneutinae, Macrocentrinae, Orgilinae, Proteropinae, Rhysipolinae, and Rogadinae. In these subfamilies 416 species are described or redescribed. Most of the species have been reared and all but 13 are new to science. A consensus sequence of the COI barcodes possessed by each species is employed to diagnose the species, and this approach is justified in the introduction. Most descriptions consist of a lateral or dorsal image of the holotype, a diagnostic COI consensus barcode, the Barcode Index Number (BIN) code with a link to the Barcode of Life Database (BOLD), and the holotype specimen information required by the International Code of Zoological Nomenclature. The following species are treated and those lacking authorship are newly described here with authorship attributable to Sharkey except for the new species of Macrocentrinae which are by Sharkey & van Achterberg: AGATHIDINAE: *Aerophiluspaulmarshi*, *Mesocoelusdavidsmithi*, *Neothlipsisbobkulai*, *Plesiocoelusvanachterbergi*, *Pneumagathiserythrogastra* (Cameron, 1905), *Therophilusbobwhartoni*, *T.donaldquickei*, *T.gracewoodae*, *T.maetoi*, *T.montywoodi*, *T.penteadodiasae*, *Zacremnopsbrianbrowni*, *Z.coatlicue* Sharkey, 1990, *Zacremnopscressoni* (Cameron, 1887), *Z.ekchuah* Sharkey, 1990, *Z.josefernandezi*, *Zelomorphasarahmeierottoae*. BRACONINAE: *Braconalejandromarini*, *B.alejandromasisi*, *B.alexamasisae*, *B.andresmarini*, *B.andrewwalshi*, *B.anniapicadoae*, *B.anniemoriceae*, *B.barryhammeli*, *B.bernardoespinozai*, *B.carlossanabriai*, *B.chanchini*, *B.christophervallei*, *B.erasmocoronadoi*, *B.eugeniephillipsae*, *B.federicomatarritai*, *B.frankjoycei*, *B.gerardovegai*, *B.germanvegai*, *B.isidrochaconi*, *B.jimlewisi*, *B.josejaramilloi*, *B.juanjoseoviedoi*, *B.juliodiazi*, *B.luzmariaromeroae*, *B.manuelzumbadoi*, *B.marialuisariasae*, *B.mariamartachavarriae*, *B.mariorivasi*, *B.melissaespinozae*, *B.nelsonzamorai*, *B.nicklaphami*, *B.ninamasisae*, *B.oliverwalshi*, *B.paulamarinae*, *B.rafamoralesi*, *B.robertofernandezi*, *B.rogerblancoi*, *B.ronaldzunigai*, *B.sigifredomarini*, *B.tihisiaboshartae*, *B.wilberthbrizuelai*, *Digonogastramontylloydi*, *D.montywoodi*, *D.motohasegawai*, *D.natwheelwrighti*, *D.nickgrishini*. CHELONINAE: *Adeliusadrianguadamuzi*, *A.gauldi* Shimbori & Shaw, 2019, *A.janzeni* Shimbori & Shaw, 2019, *Ascogastergloriasihezarae*, *A.grettelvegae*, *A.guillermopereirai*, *A.gustavoecheverrii*, *A.katyvandusenae*, *A.luisdiegogomezi*, *Chelonusalejandrozaldivari*, *C.gustavogutierrezi*, *C.gustavoinduni*, *C.harryramirezi*, *C.hartmanguidoi*, *C.hazelcambroneroae*, *C.iangauldi*, *C.isidrochaconi*, *C.janecheverriae*, *C.jeffmilleri*, *C.jennyphillipsae*, *C.jeremydewaardi*, *C.jessiehillae*, *C.jesusugaldei*, *C.jimlewisi*, *C.jimmilleri*, *C.jimwhitfieldi*, *C.johanvalerioi*, *C.johnburnsi*, *C.johnnoyesi*, *C.jorgebaltodanoi*, *C.jorgehernandezi*, *C.josealfredohernandezi*, *C.josefernandeztrianai*, *C.josehernandezcortesi*, *C.josemanuelperezi*, *C.josephinerodriguezae*, *C.juanmatai*, *C.junkoshimurae*, *C.kateperezae*, *C.luciariosae*, *C.luzmariaromeroae*, *C.manuelpereirai*, *C.manuelzumbadoi*, *C.marianopereirai*, *C.maribellealvarezae*, *C.markmetzi*, *C.markshawi*, *C.martajimenezae*, *C.mayrabonillae*, *C.meganmiltonae*, *C.melaniamunozae*, *C.michaelstroudi*, *C.michellevanderbankae*, *C.mingfangi*, *C.minorcarmonai*, *C.monikaspringerae*, *C.moniquegilbertae*, *C.motohasegawai*, *C.nataliaivanovae*, *C.nelsonzamorai*, *C.normwoodleyi*, *C.osvaldoespinozai*, *C.pamelacastilloae*, *C.paulgoldsteini*, *C.paulhansoni*, *C.paulheberti*, *C.petronariosae*, *C.ramyamanjunathae*, *C.randallgarciai*, *C.rebeccakittelae*, *C.robertoespinozai*, *C.robertofernandezi*, *C.rocioecheverriae*, *C.rodrigogamezi*, *C.ronaldzunigai*, *C.rosibelelizondoae*, *C.rostermoragai*, *C.ruthfrancoae*, *C.scottmilleri*, *C.scottshawi*, *C.sergioriosi*, *C.sigifredomarini*, *C.stevearonsoni*, *C.stevestroudi*, *C.sujeevanratnasinghami*, *C.sureshnaiki*, *C.torbjornekremi*, *C.yeimycedenoae*, *Leptodrepanaalexisae*, *L.erasmocoronadoi*, *L.felipechavarriai*, *L.freddyquesadai*, *L.gilbertfuentesi*, *L.manuelriosi*, *Phanerotomaalmasolisae*, *P.alvaroherrerai*, *P.anacordobae*, *P.anamariamongeae*, *P.andydeansi*, *P.angelagonzalezae*, *P.angelsolisi*, *P.barryhammeli*, *P.bernardoespinozai*, *P.calixtomoragai*, *P.carolinacanoae*, *P.christerhanssoni*, *P.christhompsoni*, *P.davesmithi*, *P.davidduthiei*, *P.dirksteinkei*, *P.donquickei*, *P.duniagarciae*, *P.duvalierbricenoi*, *P.eddysanchezi*, *P.eldarayae*, *P.eliethcantillanoae*, *P.jenopappi*, *Pseudophanerotomaalanflemingi*, *Ps.albanjimenezi*, *Ps.alejandromarini*, *Ps.alexsmithi*, *Ps.allisonbrownae*, *Ps.bobrobbinsi*. HOMOLOBINAE: *Exasticolusjennyphillipsae*, *E.randallgarciai*, *E.robertofernandezi*, *E.sigifredomarini*, *E.tomlewinsoni*. HORMIINAE: *Hormiusanamariamongeae*, *H.angelsolisi*, *H.anniapicadoae*, *H.arthurchapmani*, *H.barryhammeli*, *H.carmenretanae*, *H.carloswalkeri*, *H.cesarsuarezi*, *H.danbrooksi*, *H.eddysanchezi*, *H.erikframstadi*, *H.georgedavisi*, *H.grettelvegae*, *H.gustavoinduni*, *H.hartmanguidoi*, *H.hectoraritai*, *H.hesiquiobenitezi*, *H.irenecanasae*, *H.isidrochaconi, H.jaygallegosi*, *H.jimbeachi*, *H.jimlewisi*, *H.joelcracrafti*, *H.johanvalerioi*, *H.johnburleyi*, *H.joncoddingtoni*, *H.jorgecarvajali*, *H.juanmatai*, *H.manuelzumbadoi*, *H.mercedesfosterae*, *H.modonnellyae*, *H.nelsonzamorai*, *H.pamelacastilloae*, *H.raycypessi*, *H.ritacolwellae*, *H.robcolwelli*, *H.rogerblancosegurai*, *H.ronaldzunigai*, *H.russchapmani*, *H.virginiaferrisae*, *H.warrenbrighami*, *H.willsflowersi*. ICHNEUTINAE: *Oligoneuruskriskrishtalkai*, *O.jorgejimenezi*, *Paroligoneuruselainehoaglandae*, *P.julianhumphriesi*, *P.mikeiviei*. MACROCENTRINAE: *Austrozelejorgecampabadali*, *A.jorgesoberoni*, *Dolichozelegravitarsis* (Muesebeck, 1938), *D.josefernandeztrianai*, *D.josephinerodriguezae*, *Hymenochaoniakalevikulli*, *H.kateperezae*, *H.katherinebaillieae*, *H.katherineellisonae*, *H.katyvandusenae*, *H.kazumifukunagae*, *H.keithlangdoni*, *H.keithwillmotti*, *H.kenjinishidai*, *H.kimberleysheldonae*, *H.krisnorvigae*, *H.lilianamadrigalae*, *H.lizlangleyae*, *Macrocentrusfredsingeri*, *M.geoffbarnardi*, *M.gregburtoni*, *M.gretchendailyae*, *M.grettelvegae*, *M.gustavogutierrezi*, *M.hannahjamesae*, *M.harisridhari*, *M.hillaryrosnerae*, *M.hiroshikidonoi*, *M.iangauldi*, *M.jennyphillipsae*, *M.jesseausubeli*, *M.jessemaysharkae*, *M.jimwhitfieldi*, *M.johnbrowni*, *M.johnburnsi*, *M.jonathanfranzeni*, *M.jonathanrosenbergi*, *M.jorgebaltodanoi*, *M.lucianocapelli*. ORGILINAE: *Orgilusamyrossmanae*, *O.carrolyoonae*, *O.christhompsoni*, *O.christinemcmahonae*, *O.dianalipscombae*, *O.ebbenielsoni*, *O.elizabethpennisiae*, *O.evertlindquisti*, *O.genestoermeri*, *O.jamesriegeri*, *O.jeanmillerae*, *O.jeffmilleri*, *O.jerrypowelli*, *O.jimtiedjei*, *O.johnlundbergi*, *O.johnpipolyi*, *O.jorgellorentei*, *O.larryspearsi*, *O.marlinricei*, *O.mellissaespinozae*, *O.mikesmithi*, *O.normplatnicki*, *O.peterrauchi*, *O.richardprimacki*, *O.sandraberriosae*, *O.sarahmirandae*, *O.scottmilleri*, *O.scottmorii*, *Stantoniabillalleni*, *S.brookejarvisae*, *S.donwilsoni*, *S.erikabjorstromae*, *S.garywolfi*, *S.henrikekmani*, *S.luismirandai*, *S.miriamzunzae*, *S.quentinwheeleri*, *S.robinkazmierae*, *S.ruthtifferae*. PROTEROPINAE: *Hebichneutestricolor* Sharkey & Wharton, 1994, *Proteropsiangauldi*, *P.vickifunkae*, *Michenercharlesi*. RHYSIPOLINAE: *Pseudorhysipolisluisfonsecai*, *P. mailyngonzalezaeRhysipolisjulioquirosi*. ROGADINAE: *Aleiodesadrianaradulovae*, *A.adrianforsythi*, *A.agnespeelleae*, *A.alaneaglei*, *A.alanflemingi*, *A.alanhalevii*, *A.alejandromasisi*, *A.alessandracallejae*, *A.alexsmithi*, *A.alfonsopescadori*, *A.alisundermieri*, *A.almasolisae*, *A.alvarougaldei*, *A.alvaroumanai*, *A.angelsolisi*, *A.annhowdenae*, *A.bobandersoni*, *A.carolinagodoyae*, *A.charlieobrieni*, *A.davefurthi*, *A.donwhiteheadi*, *A.doylemckeyi*, *A.frankhovorei*, *A.henryhowdeni*, *A.inga* Shimbori & Shaw, 2020, *A.johnchemsaki*, *A.johnkingsolveri*, *A.gonodontovorus* Shimbori & Shaw, 2020, *A.manuelzumbadoi*, *A.mayrabonillae*, *A.michelledsouzae*, *A.mikeiviei*, *A.normwoodleyi*, *A.pammitchellae*, *A.pauljohnsoni*, *A.rosewarnerae*, *A.steveashei*, *A.terryerwini*, *A.willsflowersi*, *Bioalfapedroleoni*, *B.alvarougaldei*, *B.rodrigogamezi*, *Choreborogasandydeansi*, *C.eladiocastroi*, *C.felipechavarriai*, *C.frankjoycei*, *Clinocentrusandywarreni*, *Cl.angelsolisi*, *Cystomastaxalexhausmanni*, *Cy.angelagonzalezae*, *Cy.ayaigarashiae*, *Hermosomastaxclavifemorus* Quicke sp. nov., *Heterogamusdonstonei*, *Pseudoyeliconesbernsweeneyi*, *Stiropiusbencrairi*, *S.berndkerni*, *S.edgargutierrezi*, *S.edwilsoni*, *S.ehakernae*, *Triraphisbillfreelandi*, *T.billmclarneyi*, *T.billripplei*, *T.bobandersoni*, *T.bobrobbinsi*, *T.bradzlotnicki*, *T.brianbrowni*, *T.brianlaueri*, *T.briannestjacquesae*, *T.camilocamargoi*, *T.carlosherrerai*, *T.carolinepalmerae*, *T.charlesmorrisi*, *T.chigiybinellae*, *T.christerhanssoni*, *T.christhompsoni*, *T.conniebarlowae*, *T.craigsimonsi*, *T.defectus* Valerio, 2015, *T.danielhubi*, *T.davidduthiei*, *T.davidwahli*, *T.federicomatarritai*, *T.ferrisjabri*, *T.mariobozai*, *T.martindohrni*, *T.matssegnestami*, *T.mehrdadhajibabaei*, *T.ollieflinti*, *T.tildalauerae*, *Yeliconesdirksteinkei*, *Y.markmetzi*, *Y.monserrathvargasae*, *Y.tricolor* Quicke, 1996. *Y.woldai* Quicke, 1996.

The following new combinations are proposed: *Neothlipsissmithi* (Ashmead), new combination for *Microdussmithi* Ashmead, 1894; *Neothlipsispygmaeus* (Enderlein), new combination for *Microduspygmaeus* Enderlein, 1920; *Neothlipsisunicinctus* (Ashmead), new combination for *Microdusunicinctus* Ashmead, 1894; *Therophilusanomalus* (Bortoni and Penteado-Dias) new combination for *Plesiocoelusanomalus* Bortoni and Penteado-Dias, 2015; *Aerophilusareolatus* (Bortoni and Penteado-Dias) new combination for *Plesiocoelusareolatus* Bortoni and Penteado-Dias, 2015; *Pneumagathiserythrogastra* (Cameron) new combination for *Agathiserythrogastra* Cameron, 1905. *Dolichozelecitreitarsis* (Enderlein), new combination for *Paniscozelecitreitarsis* Enderlein, 1920. *Dolichozelefuscivertex* (Enderlein) new combination for *Paniscozelefuscivertex* Enderlein, 1920. Finally, *Bassusbrooksi* Sharkey, 1998 is synonymized with *Agathiserythrogastra* Cameron, 1905; *Paniscozelegriseipes* Enderlein, 1920 is synonymized with *Dolichozelekoebelei* Viereck, 1911; *Paniscozelecarinifrons* Enderlein, 1920 is synonymized with *Dolichozelefuscivertex* (Enderlein, 1920); and *Paniscozelenigricauda* Enderlein,1920 is synonymized with *Dolichozelequaestor* (Fabricius, 1804). (originally described as *Ophionquaestor* Fabricius, 1804).

## Chapter 1: Introduction and methods

### Introduction

It is the purpose of this article to further refine methods to overcome the taxonomic impediment of ichneumonoid biodiversity. The current treatment deals with eleven subfamilies of Braconidae, i.e., Agathidinae, Braconinae, Cheloninae, Homolobinae, Hormiinae, Ichneutinae, Macrocentrinae, Orgilinae, Proteropinae, Rhysipolinae, and Rogadinae. Although not a thorough review of all species of these subfamilies, we here document a large number of Costa Rican species and provide names and unique identifiers for future keys, biodiversity analyses, conservation (e.g., Janzen 1999a, b; Janzen and Hallwachs 1994, 2011, 2016; Janzen et al. 2017, 2019a, b, 2020a, b), and phylogenetic studies. This paper makes available morphological, biological, and molecular (the mitochondria gene cytochrome oxidase I, the “DNA barcode”) information for 413 species of Costa Rican Braconidae, all but 13 of which are newly described. The discrimination of these species is mostly based on COI barcodes used for Barcode Index Number (BIN) analysis (Ratnasingham and Hebert 2013); however, we use an integrative approach. In 55% of the cases rearing records played a role in species discrimination, and morphology was also employed for a number of species complexes.

In the following paragraphs we justify our method and address criticisms of the Meierotto et al. (2019a) publication (which employed similar methods) proposed in a recent article by Zamani et al. (2020). Their criticisms and their proposed solutions to the taxonomic impediment can be summarized as follows. Many of these are further addressed later in the introduction.

#### Criticisms:

**1. “The method ignores previously described species”**

This is not true, not for the Meierotto et al. (2019a) paper and not for the present paper. In the Meierotto et al. (2019a) paper there was a clear statement that coauthor Sharkey had seen all of the relevant types and that in his opinion none of the species treated were conspecific with these except for *Zelomorphaarizonensis*. The critics suggest that evidence be given to demonstrate that Sharkey had seen the types. Are they asking for letters from curators of the museums that Sharkey visited, or did they simply overlook the statement? Impossible to tell, and way beyond standard taxonomic practice by well-established authorities for a major group of insects. Perhaps they ignored another paper by Meierotto et al. (2019b) in which the presently recognized species of *Zelomorpha* and *Hemichoma* were established by creating dozens of new combinations. Clearly, one of the authors (i.e., MJS) of this latter paper must have viewed the type species, as stated in the paper. In this taxonomically broader treatment also, we have reviewed all of the relevant literature and found a number of previously described species that are mentioned where appropriate.

**2. “Mitochondrial trees often disagree with nuclear species trees, especially in taxa where *Wolbachia* may be altering mtDNA introgression” and “congruence between nuclear and mitochondrial signal should be tested to better reinforce the species units identified”**

This mostly appears to be pointless because we are not trying to recapitulate phylogeny. BOLD automatically screens out sequence uploads for *Wolbachia* (and other contaminants). Indeed, when barcode sequence libraries were surveyed for *Wolbachia*, evidence was found in only 0.16% of cases (Smith et al. 2012a). More independent DNA sequence data is clearly more useful (e.g., Janzen et al. 2017) and there is little doubt that in the years and decades to come the barcode will constitute many markers, if not entire genomes. However, adding more genes is not cost or time effective at present, as the critics themselves point out. Furthermore, part of the first pass taxonomic approach for megadiverse areas/taxa using DNA barcodes includes the creation of a whole genome DNA extract that is available for future phylogenetic work. While Costa Rica has achieved biopolitically public domain status for its CO1 barcodes as presented here (Decree 41767. 2019), the whole genomes are not (yet) full open public access for Costa Rica and most other tropical countries.

**3. “purely DNA-based descriptions will… make the identification of millions of historical specimens impossible”**

As we have made clear in the Meierotto et al. (2019a) paper and here, we are not proposing that comprehensive revisions that include keys and morphological diagnoses be abandoned. Rather, we view barcode-based descriptions as a first pass in an iterative approach to solve the taxonomic impediment of megadiverse and under-taxonomically resourced groups that standard technical and biopolitical approaches have not been able to tackle. For example, if a taxonomist wishes to integrate these elements to *Zelomorpha* or any of the taxa that we review below they will have a great starting point (and resources). When a large number of specimens, from a wide geographic range, are barcoded, effective morphological keys may be written and old museum specimens will regain their value to go along with their barcodes (e.g., Janzen et al. 2017). Nonetheless, until this milestone is achieved, and even then, the old museum specimens have little value, because of the enormous cost in time and budgets to examine and identify them morphologically, e.g., imagine trying to identify in a morphological key one of the more than 3,000 species of Neotropical *Triraphis* 95% of which are currently undescribed.

**4. “it will impair this science [taxonomy] in developing countries which house most of the undiscovered portion of biodiversity, due to high costs and lack of staff and technology”**

This is a point that one of the reviewers of our present manuscript also mentioned. Firstly, high costs, lack of public interest, and lack of staff, to say nothing of lack of biopolitical enthusiasm or budgets, have caused the taxonomic impediment worldwide, not just in developing countries. In Canada, for example, a Canadian Council of Academics report from a decade ago found that previously deep and respected taxonomic resources suffered from decades of lack of investment that resulted in insufficient capacity to describe Canadian biodiversity (Lovejoy et al. 2010). Costa Rica is a relatively poor country that cannot maintain a cohort of taxonomists to describe its biodiversity and yet we here describe more than 400 new species from the country, with many more to come, all public domain and all available to future users at any level if they have barcode access, which they will. The critics may have meant to write that demanding barcodes for species descriptions, which we do, will disenfranchise those who cannot afford the price of barcoding. At the Canadian Centre for Biodiversity Genomics (CBG) the current rates for barcoding vary between 1.02 and 25 US dollars, depending on the quantity of specimens and their age, e.g., old museum specimens are more expensive. Today most taxonomists would be looking at $10 per specimen for a minimum of 95 specimens. That is almost $1,000 and certainly an impediment to many, although not out of the range of routine expenses like salaries, lab infrastructure, and page charges. DNA evidence is necessary to delimit species in megadiverse taxa, as we demonstrate further in the introduction, and the use of which Zamani et al. (2020) promote elsewhere in their article. Our first pass approach is not more expensive than the current standards in comprehensive revisions.

**5. [it will] “drastically affect other related fields of study and, importantly, conservation”**

The authors did not expand on this criticism, but the opposite is true. For example, barcoded species will allow for the identification of metabarcoded specimens. This is a tool that is being used to determine the rareness and distribution of a species and potentially allow for their protection. Our method allows for the rapid naming of species and it is very unlikely that unnamed species will ever be protected, and they will have no chance of being integrated into the socioeconomics of a tropical country, a process that is vital for the conservation of tropical biodiversity (Janzen and Hallwachs 2019b).

**6. “there must be several photographs available, not a single lateral photograph of a single specimen”**

Of course many photographs are desirable as are fully integrated revisions. However, if we are to rid ourselves of the taxonomic impediment, time is a critical factor. Our simple images are meant simply as a voucher for a comparison with newly barcoded specimens, as well as to offer many gestalt traits. For example, we have a number of species in our treatment of Costa Rican fauna below that appear to have conspecific specimens on BOLD that were collected by others in other countries, e.g., Belize and Argentina, i.e., they are in the same BIN. In most of these cases the details on BOLD are “private” (unfortunately owing to other taxonomists’ possessiveness) and so we cannot easily examine the specimens to include them in our revision. However, the proprietors of these specimens can look at our image and conclude either, “yes that looks a lot like my specimen and is probably the same species or not”, and all of our specimens are available for examination from the CBG or from their final deposit in the Canadian National Collection in Ottawa.

**7. “some text highlighting important diagnostic features is valuable… [and] the actual description of the species may be written within minutes when material and expertise are already available”**

This is false. If morphological descriptions and diagnostics could be written in minutes we would include them, and the taxonomic impediment would be just a pebble in the taxasphere’s road. Even the revision of *Alabagrus* by Sharkey et al. (2018), which included barcode data and morphology, could not separate morphologically a number of species that, based on host data and COI sequence data, were clearly different. For megadiverse, understudied taxa like those treated herein, a morphological diagnosis separating tens to hundreds of new species from a few previously morphologically described species is a waste of time. This is because the vast majority of species in these taxa are undescribed and distinguishing a new species from the few described species leaves dozens or hundreds or thousands of species that could fit any simple morphological diagnostic. One of the advantages of the barcode approach is that it acts as a diagnostic that is effective in distinguishing newly described species from almost the entirety of both described and undescribed species yet to be captured. It is impossible to guess at all of the different morphological combinations that may be possessed by the plethora of undescribed species for any particular genus, but the COI barcode is diverse enough across taxa that it is an effective diagnostic even for these unknown species, as has been shown by its reliability for already well-known taxa such as tropical Lepidoptera.

**8. “Simply assigning all BINs taxonomic names.. would indeed complete the inventory of life on Earth extremely quickly (at precisely the same pace as the rate of barcoding) – that we do not dispute. But it would also remove the quantitative and qualitative difference between these preliminary identifiers (based on a single DNA marker) and full taxonomic recognition (based on a more comprehensive diagnosis, ideally supported by multiple lines of evidence including genetic data) that lend taxonomy its value”**

When the authors and the taxasphere have the budgets, the enthusiasm, the time, and the biopolitical permissions to achieve this for the 20+ million tropical species of terrestrial Eukaryota, it will be wonderful. As mentioned earlier, our method is not meant to be the final treatment for a higher taxon. We admire and we practice the integrative approach, and all authors who are taxonomists in this publication regularly publish integrative revisions; this is our standard. But every integration starts with pieces to integrate. The barcode and its associated administrative accounting, along with the specimen itself, and whatever higher taxon information is available, is what we can offer to tropical conservation through sustainable biodevelopment (e.g., Janzen et al. 2020a). We are now offering an alternative method as a taxonomic first pass for megadiverse understudied taxa, the goal being to overcome the taxonomic impediment, thereby allowing the full power of tropical biodiversity value to become truly part of tropical societies. The critics also misrepresent our method. We do not simply assign taxonomic names to BINs. Firstly, ca. 10% of BINs contain multiple species. More rarely, BINs that are presently on BOLD today may coalesce, resulting in one of the BINS disappearing, just as happens when a species is found to bear several classical scientific names (incidentally, often revealed by barcoding). A careful examination of the content of the BINS and their nearest neighbors is necessary. Working with the specimens reared by Dan Janzen, Winnie Hallwachs et al. in the Costa Rican ACG mega-inventory (Janzen and Hallwachs 2016) has been enlightening to discern species boundaries, and to understand the limits of the BIN-only approach, because of the host-caterpillar data that they have. For example, host data, and morphological evidence allowed us to distinguish seven species of *Macrocentrus* within the BINBOLD:ACK7466. This is a low frequency example; for 90% of the species, BINs make a good proxy to species, just as morphological comparisons often do. They are the equivalent of placing look-alikes in a unit tray, later to be sometimes split into a species complex by yet more data, precisely what happens in classical taxonomic practice following naming of museum specimens in a monograph. This in turn generates papers with titles such as “17 new species hiding in 10 long-named gaudy tropical moths (Lepidoptera: Erebidae: Arctiinae)” (Espinoza et al. 2017), each of the 17 having been lumped within the ten classical species in unit trays for decades.

**9. “It would supplant taxonomists with technicians, who need to know nothing of the biology of the units with which they are dealing”**

We wish this were true and imagine the power it would and perhaps will give the general public. Certainly, more people will play a larger part in a barcoding approach. If the criticism were true, we could overcome the taxonomic impediment much more quickly, to say nothing of exporting bioliteracy possibilities to the majority of the world (Janzen and Hallwachs 2019b). Interestingly, many of the co-authors of the present paper fit the technician category. Nonetheless, due to the necessity to identify specimens to genus as well as DNA contamination, multiple species in BINs, and other minor issues, an expert is necessary for the next level of resolution or confirmation.

#### Solutions:

**1. “real revolutions are undoubtedly coming, especially from the fields of machine learning and integrative species delimitation (Solís-Lemus et al. 2015; Favret and Sieracki 2016), and also that it is possible to produce massive, rapidly assembled taxonomic monographs without compromising on quality (Rakotoarison et al. 2017)”**

The ‘good news’ reported by the critics does nothing, and will do nothing, to attack the core of the taxonomic impediment. The visual identification system introduced by Favret and Sieracki (2016) is dependent on preexisting taxonomy for machine training. That is, after a species is described, numerous specimens are investigated, and a machine is trained to recognize the species. BOLD does precisely this by its barcode. The proposed revolution is a fine technology, but unrelated to the taxonomic impediment. Integrative molecular species delimitation proposed by Solis-Lemus et al. (2015) requires far more loci, sequencing, and money than does DNA barcoding and therefore suffers from most of the same criticisms the critics aim at barcoding. The comment that “massive”, rapidly assembled taxonomic monographs (Rakotoarison et al. 2017) can be assembled “without compromising on quality” refers to a revision treating fewer than 50 species, a drop in the bucket compared to our groups. It also is constructed on profuse sequencing, which would be problematic (according to the critics) for labs in developing countries, to say nothing of the general user community.

**2. “a true paradigm shift in taxonomy will come only when there is a revolution in the level of financial investment in taxonomy and the natural history museums that house the described and undescribed reference material of life on Earth”**

The taxasphere has been hoping for this for many decades and it will likely never happen until we show the funding agencies that we can make a dent in the taxonomic impediment without costing billions of dollars, to say nothing of showing the overall public that there is reason to bother. To our knowledge, in the USA (and we presume in many other parts of the world) there are no taxonomists that are federally funded for alpha taxonomic research unless they are also addressing interesting scientific, commercial, or public questions. Trivial funding is available for higher level phylogenetic research, although on a very limited scale. We believe that NSF (USA) and other agencies may fund large scale revisions if the revisor, employing a barcode approach, is treating thousands of species and attacking the taxonomic impediment. Unfortunately, current economic crises are causing many of the countries with appropriate resources to be more interested in funding research that will largely benefit themselves rather than the tropical countries so rich in rapidly disappearing biodiversity.

We continue now by justifying our diagnostic approach. COI barcodes are indispensable for diagnosing the species treated herein, as well as signaling where seemingly trivial morphological variation actually indicates a species boundary, and they are an invaluable tool for studies of most other hyperdiverse arthropod taxa as well. For our taxa, COI barcodes, though not infallible, are a magnitude more effective than morphology, and the evidence presented below demonstrates this. Dozens of additional examples can be found in the following studies: Hebert et al. (2004); Janzen et al. (2005, 2009, 2011, 2012, 2017, 2019b, 2020b); Hajibabaei et al. (2006); Smith et al. (2006–2008, 2012b); Burns et al. (2007, 2008); Chacon et al. (2013); Bertrand et al. (2014); Brown et al. (2014); Fernandez-Triana et al. (2014, 2020); Fleming (2014); Hansson et al. (2015); Phillips-Rodriguez et al. (2014); and Espinoza et al. (2017). Using a morphology-only approach, Leathers and Sharkey (2003) revised the Costa Rican species of *Alabagrus* (Braconidae) with an emphasis on specimens from the La Selva Biological Field Station. Later, using a morphology and COI barcode approach, Sharkey et al. (2018) re-revised Costa Rican *Alabagrus* with an emphasis on specimens reared in the Área de Conservación Guanacaste, only 100‒150 km distant from La Selva, and containing all of the La Selva ecosystems. When the 39 species treated by Leathers and Sharkey were barcoded, 17 were found to have been incorrectly lumped with previously described species that do not even occur in Costa Rica, and five endemic morphology-based Costa Rican species were discovered to be species complexes. The morphology-only approach resulted in an error rate of more than 60%. The following examples illustrate the sources of the errors. The Leathers and Sharkey concept of *Alabagrusenglishi* was found to comprise five distinct species when barcoded. To quote directly from Sharkey et al. (2018). “Many paratypes of *A.englishi*, sensu Leathers and Sharkey (2003), will now key to the following species: *A.johnobryckii*, *A.sarameierottoae*, *A.sarahsharkeyae*, and *A.barbsharanowskiae*.” A second example is *A.pachamama*, which was recorded by Leathers and Sharkey as occurring in Costa Rica. As the name implies, the holotype is from Peru. This “species” turned out to be a complex of five species in Costa Rica, i.e., *A.jackiemillerae*, *A.scottshawi*, *A.fernandezi*, *A.fernandodiasi*, and *A.genemonroei*. Their COI barcode sequences are significantly divergent, and their host caterpillars are conspicuously different species. These facts led the authors to detect subtle morphological differences that distinguish the species, and further, to conclude that *A.pachamama* does not even occur in Costa Rica. These findings suggest that this complexity is common in neotropical ecosystems.

**Figure 1.**
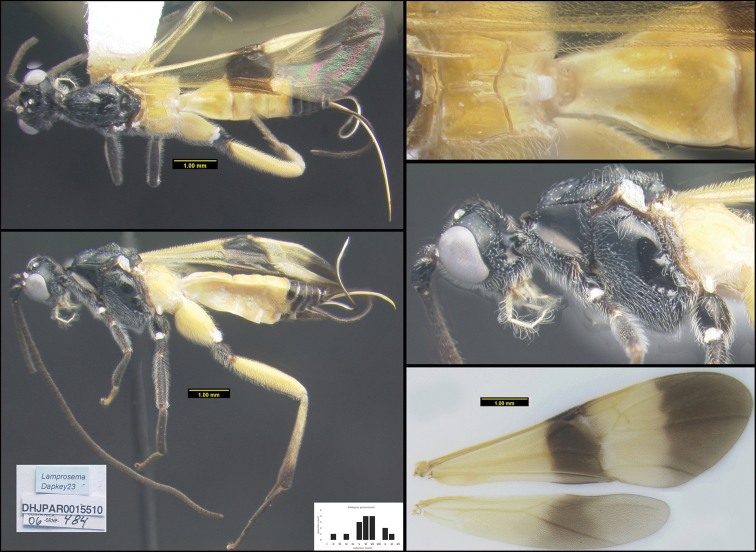
*Alabagrusgenemonroei*, holotype.

**Figure 2.**
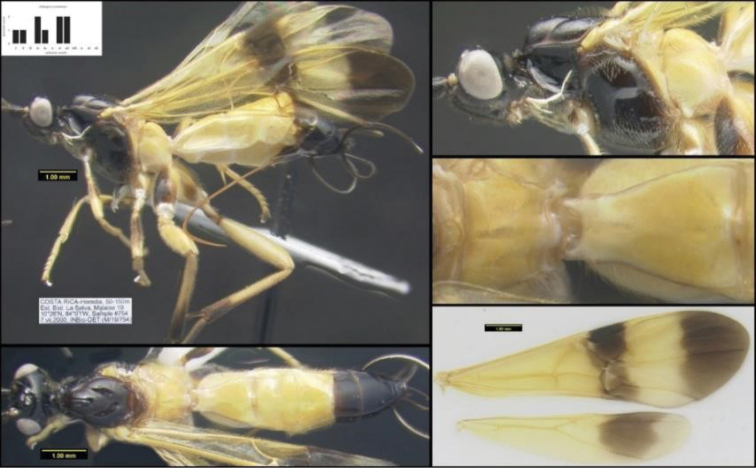
*Alabagrusscottshawi*, holotype.

To understand how morphologically similar the species of the aforementioned complex are, compare the images of *A.scottshawi* and *A.genemonroei* (Figs 1, 2). A casual look reveals that the propodeal sculpture is different, the basal black bands in the forewings are in different positions, and the hind coxae have different colors. Without molecular data, how can one decide if these features reflect intraspecific variation or are evidence of distinct species, especially when there are only a few specimens (and sometimes only one) in hand? To add to this morphological ambiguity, there are three more congeneric species with similar, but slightly different combinations of these character states. It was only after molecular and host data signaled different species that morphological characters were confirmed to distinguish the species of this complex. Even after incorporating COI sequence data, not all species of *Alabagrus* treated in Sharkey et al. (2018) could be separated based on morphological characters; for example, *A.jennyphillipsae*, *A.isidrochaconi*, and *A.jeanfrancoislandryi* are inseparable morphologically (they end in the same terminus in the morphological key) but clearly distinguished by COI data.

In the *Alabagrus* revision (Sharkey et al. 2018) and in the species treatments that follow here, a 2% genetic distance, the conventional threshold for species delimitation using COI barcodes (Jones et al. 2011), was used to cluster putative species (Smith et al. 2012b). Morphology and host data were then employed to test and refine these approximations to species. The 2% divergence results in putative species being coded with Barcode Index Numbers (BINs) (Ratnasingham and Hebert 2013) produced by BOLD (http://www.barcodinglife.org/index.php/Public_BarcodeIndexNumber_Home). The BINs were used to sort specimens just as morphology is traditionally employed to sort specimens into museum unit trays. Morphological data, ecological data, and clustering of barcodes within a BIN were then considered to make final decisions when genetic distances between putative molecular species were near or below the 2% threshold, and commonly, two or more clusters within a BIN were each represented by numerous specimens. In one of the first published examples of “hidden diversity,” that of *Astraptesfulgerator* (Hebert et al. 2004), six species easily distinguished by caterpillar coloration, food plant, and ecology, fell in the same BIN (Janzen et al. 2011) because they were each less than 2% different in their barcodes, yet distinctly differently clustered within the BIN. In the case of *Alabagrus*, COI barcodes were 100% consistent with morphological and host data. By this, we mean that the COI data never lumped specimens that were markedly different morphologically in the same BIN; nor did it lump specimens with divergent host data. Furthermore, “Binning” did not split species that were clearly demarcated by host and morphological data.

COI data are not always diagnostic. In the revision of *Lytopylus* (Braconidae, Agathidinae) (Kang et al. 2017), two of the 32 reared species from Costa Rica did not achieve a 2% COI divergence, yet they had distinct morphologies and host data. The error rate, if COI had been used exclusively for the *Alabagrus* and *Lytopylus* revisions, would be ~ 1%, far better than the grossly underestimated 15% error rate assumed for predominantly morphological revisions of braconids as demonstrated in the database of Yu et al. (2016). Clearly, relying solely on COI barcode sequences will result in some overlooked cryptic species, and we do not promote COI-only species delimitation except where mass samples are being species-counted and therefore small amounts of species lumping is irrelevant (e.g., Janzen et al. 2000b), e.g., it may not matter if a year of Malaise trapping records 8,500 species or 8,516 species if it is being compared with a trap-year recording 3,456‒3,602 species. In the treatment of *Macrocentrus* that follows, seven species fall into the same BIN (BOLD:ACK7466). However, because they are so dissimilar morphologically and separated on the NJ tree, we treat them as distinct species. In this case, because the BIN data are not diagnostic, and will not be until the sample size is large enough to show clustering within the BIN, we provide a morphological key to the seven species. Janzen and Hallwachs’ rearing studies have revealed numerous BINs with multiple species, and they estimate a 10% rate of lumping using BOLD’s 2% genetic distance threshold. For example, in a reared sample of ca. 17,139 reared and Malaise-trapped barcoded specimens of Microgastrinae (Braconidae) from Área de Conservación de Guanacaste (ACG), there are 1,231 BINs, but more detailed examination of morphology and ecology reveals that there are at least 1,346 species in that sample (unpublished). Given the same rate for other major taxa in the inventory (Tachinidae, Ichneumonidae, Hesperiidae), for large samples that have been only BINned (as in a Malaise trap sample), we tentatively add 10% more species to large BIN numbers for a more accurate estimation of the number of species that will be defined with more intense scrutiny by specialists, coupled with more detailed ecological information.

As a final illustration of how ineffective morphology is to differentiate braconid species, we will paraphrase a report published by Kang et al. (2017). Ilgoo Kang (at that time a new graduate student) and Michael Sharkey (with decades of experience in species-level morphological alpha taxonomy) tested their ability to differentiate the species of *Lytopylus* before including COI and host data. They had error rates of 62% and 54%, respectively. All possible types of errors were discovered, i.e., lumping, splitting, and both lumping and splitting (mixing some of the members of two or more species into two or more incorrect species). In contrast, the molecular species concepts matched with the final species delimitations, which were also based on host data, at 96.6%. In light of the evidence presented above, and much more like it in the literature, it seems absurd to us that many in the taxasphere complain about barcode-based species concepts, yet readily accept delineations based solely on morphology. This is not to say that COI-based species discrimination will work for all taxa, but the method undoubtedly is highly superior to morphology for the Braconidae treated here and all indications are that they will be the same for most other species-rich tropical taxa (e.g., MicrogastrinaeBraconidae, Ichneumonidae, Tachinidae).

Hopefully, we have demonstrated the utility of COI barcode data for species circumscription. Now we address the hurdle of species descriptions. Critics claim that the description of a new taxon must include more than an image and COI sequence data, insisting that keys, morphological diagnoses, detailed figures, and the examination of the holotypes of all congeneric species known from the same realm are necessary. Our answer to these criticisms is that there are too many species and too little time. For many species-rich higher taxa, detailed place-based neotropical inventories such as that of ACG (e.g., Janzen et al. 2009, Janzen and Hallwachs 2016) deal with only a fraction of the total local diversity. In these cases, is seems inane to present a morphological key where it is obvious that no key or array of diagnoses will discriminate the described from the hundreds of species yet to be described (e.g., Fernandez-Triana et al. 2014, 2020; Fleming et al. 2014; Hansson et al. 2015). Due to humankind’s assault on the planet, there is an urgency to recognize the multitude of species that are yet to be described, quite simply because by not knowing what they are, where they are, and what they do, humanity has no compulsion to allow them and their wild ecosystems to co-exist (Janzen and Hallwachs 2019b). For years, this urgency of dealing with the taxonomic impediment has been recognized. The predominant solution proposed has been “give us more money and more taxonomists”, a laudable strategy but one doomed to fail as the last three or four decades have demonstrated.

In the following paragraphs we will demonstrate the magnitude of the task of describing the world fauna of one hymenopteran superfamily and demonstrate that the present approach to taxonomy is woefully inadequate regardless of the number of taxonomists employed. The Ichneumonoidea contains the two most species-rich families of Hymenoptera, i.e., Braconidae and Ichneumonidae. As of 2016, the superfamily comprised ca. 44,000 valid species (Yu et al. 2016). The true number of species can only be crudely estimated, but it is conceivable that there are 1,000,000 or more. Estimates of total species richness for this group are quite variable and have increased greatly over recent decades. Rodriguez et al. (2013) used the ratio of described wasp species to Lepidoptera hosts from relatively well studied sites to estimate the total number of species in the braconid subfamily Microgastrinae, which currently has ca. 3,000 described species (Fernandez-Triana et al. 2020). They estimated between 17,000 and 46,000 species of Microgastrinae, but they noted this is likely an under-estimate due to the many undescribed species of Microgastrinae from well-studied sites that were used to make the extrapolations. Extrapolating from Yu et al. (2016), five of every one hundred described ichneumonoid species are microgastrines (Yu et al. 2016). Assuming that this ratio holds true for undescribed species, and that the estimates made by Rodriguez et al. (2013) are sound, there are between 300,000 and at least 900,000 species of Ichneumonoidea.

**Figure 3.**
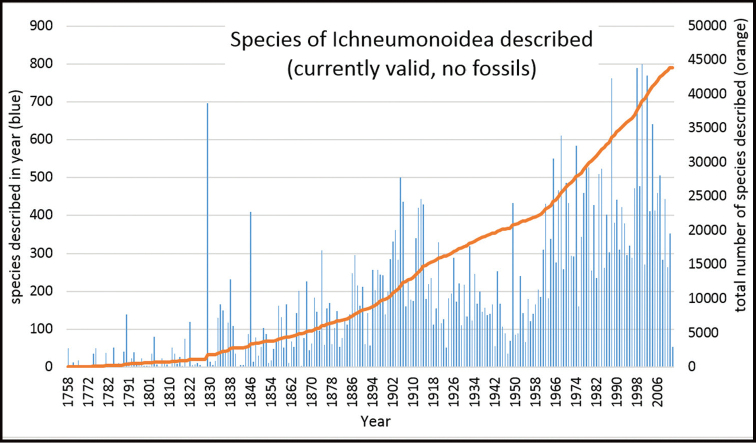
Description rate of Ichneumonoidea species. Data from Taxapad (Yu et al. 2016) (modified from Meierotto et al. 2019a).

Extrapolating again from Yu et al. (2016), from 2000 to 2011, an average of 468 species of ichneumonoids were described per year (Fig. 3). Given the current rate of species description and these estimates of species diversity, all ichneumonoids, that manage to remain extant, would be described somewhere between the years 2561 and 3843, if they were ever actually captured and curated.

Given the number of the species treatments needed to tackle even a small portion of this diversity, classical taxonomic monographic treatments are not the answer. Most of the criticisms offered against our approach stifle or skate over the fundamental purpose of the method. More images, checking types of dubious likelihood of being conspecific with any of the species treated, and producing morphological keys and diagnoses all take time, too much time, and far greater financial resources are required. Worse, they can only be used by the ever-dwindling number of people that society will support to be “experts” in the field, and even when such experts do exist, they and their literature are not available to the global society at large that needs to have reason to know and understand. But there are other more practical reasons not to include some of these elements which we will get to shortly.

With a COI barcode approach, it will be relatively easy to check the identity of a new specimen of braconid from Costa Rica, and in these new stages of method evolution, we are strategically fortunate that > 95% are undescribed and will remain so unless treated differently than in a traditional manner. To identify any specimen, one simply needs to obtain the COI barcode from the specimen and check for a match in BOLD with a barcode or BIN, then compare the images and other information in the BOLD database to confirm or refute the identification. If there is no match, that specimen is then a starting point for further identification or description, and it has its collateral and barcode data already databased with its specimen voucher code. This may not yet be an effective approach today for most braconid taxa from Costa Rica, but it will be effective for all of the genera that are given a preliminary treatment, such as those presented in the chapters that follow. If a Costa Rican specimen is absent from BOLD after the publication of this and further preliminary revisions, it is straightforward for anyone to describe the species and make it available to all through the web. Processing at the Centre for Biodiversity Genomics (CBG) and the output in BOLD allows its placement in a taxonomic hierarchy; e.g., family, subfamily, and usually genus, the latter almost 95% accurate in braconids. Such description can simply follow an example from any of the following chapters. This is why some refer to COI barcoding as the democratization of taxonomy, and perhaps why it frightens some of our more conservative taxonomic practitioners, and especially those who normally work with just a few (today) important species far from the hyperdiverse tropics.

Compare this to the standard approach of identifying a braconid from Costa Rica. One would start by identifying the specimen to subfamily and genus, impossible tasks for most people, even with good keys, and a difficult task for specialists. In the case of Costa Rican braconids, this is also a rather tedious process for a specialist due to the number of new genera being encountered and generated annually. To make this example a little more understandable to the reader, we will not make a reference to the publications required to do this, rather we will let the reader verify the difficulty for themselves by trying to find the references themselves to attempt the subfamilial and generic identification of any braconid specimen from any Neotropical country. Once a generic name is in hand, the identifier will need to proceed to the species level with the knowledge that fewer than 10% of neotropical species are described. Typically, this means obtaining numerous publications, many of which are protected by copyright and expensive to purchase, even if available on the web, and then comparing the specimen in hand with diverse keys and descriptions, in assorted languages (e.g., German for Costa Rican Opiinae). Furthermore, many of the abundant English texts are based on a vocabulary that often does not have unambiguous translations to Spanish or Portuguese. When a tentative identification is made, an examination of the type specimen will almost certainly be needed for confirmation. Most readers understand the impossible barrier that this presents, especially for non-taxonomists from developing countries. Even if a morphological match with a holotype is obtained, the likelihood of cryptic species will make this morphological comparison far from certain.

Morphological keys for masses of hyperdiverse, poorly known species are all but useless, and their exclusion from the following chapters is not an oversight. This is why. For the genera treated herein, we estimate that we have barcoded at most 10–20% of the Costa Rican fauna. Most of the specimens come from caterpillar rearings in Área de Conservación Guanacaste (the size of the footprint of New York City and its suburbs, www.acguanacaste.ac.cr), and for practical reasons, fruit-feeding, stem-boring, and leaf-mining caterpillars have not yet been reared, nor those from canopy-restricted caterpillars. Added to this are specimens from approximately 16 Malaise trap-years of samples from six localities scattered across dry forest, rain forest, and cloud forest (e.g., Janzen and Hallwachs 2020b), a very small number given 120,000 terrestrial hectares of all ages of succession in ACG. In other words, many more species of the genera treated below are yet to be discovered/collected, and the probability of a specimen in hand during further sampling being in the key or other keys (e.g., *Apanteles* by Fernandez-Triana et al. 2014) would be considerably less than 50%. It is our intention to continue with a treatment of all species of Costa Rican braconids as new Malaise trap samples and more reared specimens become available through BioAlfa (Janzen and Hallwachs 2019b), which is an effort to DNA barcode the entire national Eukaryota. If keys were included in these preliminary revisions, addenda would need to be added to the keys on an ongoing basis, creating an even more impossible taxonomic morass for anyone other than a fanatically dedicated taxonomist specialized in Costa Rican Braconidae. There may eventually be some utility in morphological keys for these taxa, but only after the survey nears completion. Even then, the audience will be extremely limited because only Costa Rican species will be surveyed, and all genera are far more widespread, many of these with thousands of undescribed species worldwide, e.g., *Chelonus*, *Triraphis*, and *Aleiodes*. In any case, the effort of making a key at any time is of dubious value because the COI barcode alternative is so much more efficient and accurate, and the arcane vocabulary of entomological keys are impossible for non-specialists to interpret. The above arguments apply equally to morphological diagnoses. Perhaps one day there will be a large ecotourist economy attracting braconid aficionados to Costa Rica, certainly then a morphological key would be warranted, but by then each person will likely have their own personal pocket barcode recorder and can obtain their own identifications through the internet, with the barcode of the specimen in hand as the first couplet in the “key”.

We have often heard the criticism that a barcoding approach to species circumscription will result in duel taxonomic systems, one morphological and the other DNA-based. There is some truth to this statement if an effort is not made to blend the old with the new, and our suggestion is to discontinue revisions of complex faunas based only on morphology, as well as stop imagining that a visual comparison with ancient holotypes allows accurate decisions as to which of multiple species in a cryptic complex actually matches the holotype. Furthermore, once the species have been tagged with interim names, be they codes or human-readable scientific names, they can be studied and grouped morphologically at will.

Our arguments follow. In the generic treatments below every effort has been made to search the literature for species described by specialists who have spent a lifetime doing braconid taxonomy. In some cases, this has resulted in the discovery of published names for our barcoded specimens, e.g., *Adeliusgauldi* and *Adeliusjanzeni*. A recent publication by Shimbori et al. (2019) treated the New World species of Adeliini and included color photos, facilitating identification. Furthermore, the two aforementioned species were both captured in the same areas of Guanacaste Province that are the source of many of our specimens, and where many years ago Janzen used the same kind of Malaise trap to capture them under the guidance of Ian Gauld. The same quality photos are in a revision of *Leptodrepana* by Dadelahi et al. (2018) in which they described 24 species from Costa Rica. Of these, only one species convincingly matched one of our barcoded species, and it too was collected in an area heavily sampled by Janzen and Hallwachs and their team. Papp (2016) described 25 new species of the several thousand undescribed species of *Chelonus* (as *Microchelonus*) from Costa Rica. From his descriptions, and the lack of color photos, it is very difficult to tell if any match our barcoded specimens. A number of his holotypes are from Guanacaste and Alajuela, where the bulk of our specimens were collected, but none are from the Área de Conservación de Guanacaste and it is doubtful that any match those treated here. This lack of matching is all the more probable when you consider that only one of the 24 species of *Leptodrepana* matched. When the Papp holotypes are barcoded, as they eventually will be, some synonyms may be revealed, and our names synonymized. This is a better situation than guessing that one of our species is the same as one of Papp’s. Information is not lost in the long run if species are split. However, if species are lumped, the literature becomes confounded with false data, in this case, host and distributional data being attributed to one species when there are actually two or more species. The Costa Rican braconid fauna is in an ideal position for our approach since few morphology-based revisions have been conducted on it. For areas such as Costa Rica, the solution to the perceived problem of duel taxonomic systems lies in barcoding the holotypes, not stasis until the myriad of new species has been treated at the traditional pace, if ever. These comments apply to any other neotropical country or large region as well.

As a final comment, the strategy employed herein may not be optimal for all taxa and in all regions of the globe. For example, even in Costa Rica, there are taxa for which there are already many described species (e.g., some families of Lepidoptera), and in these groups our approach may result in the description of an unacceptable number of synonyms, so caution has been employed. For example, the tortricid fauna of Costa Rica includes ca. 250 described species, the vast majority of which have not been barcoded. In this group, describing the 300‒400 BINs that currently have not been identified, would almost certainly result in an unacceptable number of synonyms. Also, because the genitalia of Lepidoptera harbor many of the most compelling morphological features for species discrimination and circumscription, considerably greater effort would be required to examine the morphology in these families than in Braconidae. On the other hand, the comparison of barcodes at $10/specimen is vastly cheaper in specialist’s time than is genitalic study of thousands of specimens and every effort should be made to barcode holotypes.

If there is any hope of overcoming the taxonomic impediment it will be achieved with the approach outlined here or something similar to it. The shortcomings of the method are obvious in that it only provides a first pass at the comprehensiveness that we all desire. First passes, such as those presented in the following chapters, will provide the foundations that can be augmented by subsequent generations of revisors as time, money, and determination allow.

### Materials and methods

#### Specimens and generic placement

Approximately half of the species newly described here, and most of the specimens, were collected by rearing wild-caught host caterpillars in ACG in northwestern Costa Rica (Janzen and Hallwachs 2016). Caterpillars were collected by a team of Costa Rican parataxonomists (Janzen and Hallwachs 2011, 2016) as part of the ongoing project to document all ACG non-leaf-mining Lepidoptera larvae, their food plants, and their parasitoids. These caterpillars were databased with collection information, food plant information, and often a photograph, and they were reared to adults. When an adult moth, butterfly, or parasitoid emerged, the specimen was preserved oven-dried or frozen in 95% ethanol, and a leg was sent to the CBG at the University of Guelph, Canada (http://biodiversitygenomics.net; http://ibol.org) for DNA barcoding at a cost ranging from $3–$10 USD per specimen. Analyzed barcodes are deposited with their collateral specimen voucher data in the BOLD public database (www.boldsystems.org). The barcodes and specimens work their way towards whoever is eager to do morphology-based taxonomy, with the associated vouchers’ collateral data. Although the DNA barcodes are public domain (Costa Rican government decree #41767, Janzen and Hallwachs 2020b), as in a public dictionary, the genome associated with that barcoded specimen is currently only available through a permit-based contractual relationship with the government of Costa Rica, just as in any biodiversity prospecting relationship, be it for academic or commercial purposes.

Four sites in ACG are the subject of most of the Malaise-trapped specimens described here. Bosque San Emilio (BSE) is a 100-year old, 10‒20 m tall, secondary successional Pacific dry forest at ca. 300 m elevation in Sector Santa Rosa. Estación San Gerardo (ESG) is 30‒80-year old mid-elevation Caribbean rain forest at ca. 600 m elevation. Derrumbe (Cloud Forest) is a somewhat fragmented old-growth cloud forest at ca. 1400 m elevation near the top of 1500 m Volcán Cacao, a member of Cordillera Guanacaste that separates BSE from ESG. These three sites had one trap apiece that were operated continually, emptied once a week, for a year, and positioned beneath a broken portion of the canopy rather than in full sun or full heavy shade. The fourth site is a complex of seven Malaise traps in an area of ca. 5 km^2^ of forest with a 1.5 km^2^ bare-earth-rock geothermal drilling platform (trap numbers PL12-1-7) in the middle of the area; this forest is mostly composed of old growth at ca. 800 m elevation. The year of sampling at this site ranged from traps nearly insolated at the platform edge to 150 m distant into the shady forest understory. This forest lies at the intersection of ACG dry forest and rainforest at 800 m but slightly below the lower margin of cloud forest that starts at ca. 1000 m. All wild-caught caterpillars from which braconids were reared were found in some variant of the four ecosystems (dry, rain, cloud and interface) and within 20 km of one or more of the Malaise traps. In their first year (sometime between 2011 and 2014, these traps collected ca. 28,000 species of arthropods (excluding spiders, collembola and mites) as based on their BINs (Janzen et al. 2020).

Holotypes of all newly described species are deposited in the insect collection of the Canadian National Collection of Insects, Ottawa (**CNC**). Paratypes and all other specimens are deposited currently for the most part in the Biodiversity Institute of Ontario, at the University of Guelph, 579 Gordon St., Guelph, Ontario, N1G 1Y2, Canada. Others are divided between the CNC and the Hymenoptera Institute Collection (**HIC**), 116 Franklin Ave., Redlands, California, 92373, USA. Specimens deposited in the HIC will eventually be transferred to the CNC, and eventually representative specimens will be donated to the Museo Nacional de Costa Rica, San Jose, Costa Rica.

Identification of specimens to the subfamily level can be achieved using the key by Sharkey (1997a). Exceptions are the Hormiinae and Rhysipolinae, which were included in a large polyphyletic concept of Hormiinae s. l. in that publication. Also, the Ichneutinae and Proteropinae were included under Ichneutinae s. l. in Sharkey (1997a), and here they are separated following Chen and Achterberg (2019). For this reason, a diagnosis is given for these subfamilies in their respective chapters.

Morphological terms largely follow Sharkey and Wharton (1997), and definitions in English may be found at the Hymenoptera Anatomy Ontology Portal (http://portal.hymao.org/projects/32/public/ontology/).

Some host species are still awaiting full identification and are given interim names. For example, *Antaeotricha* Janzen233 is identified to the genus *Antaeotricha* by classical morphology-based criteria and to Janzen233 by barcode and ecological information. However, no formal scientific species name is available until a barcode-match is obtained with an existing holotype or until it is described as new, which may be decades. Equally, *Antaeotricha* radicalisEPR03 is also an interim name based on what the species LOOKS like, but is not, by its barcode, and note that the species epithet is not italicized and contains a number: it is not a scientific name, but temporarily retains the information that this species was called simply its look-alike *A.radicalis* before barcoding and associating it with other ecological data. Also, a name such as gelJanzen01 Janzen407 signifies a caterpillar in the family Gelechiidae for which even a generic name is not obtainable at present. In the future, the barcode, the temporary name, name, and BIN will remain searchable in the Janzen and Hallwachs database as well as in BOLD, GenBank, and any other public sequence repository. Continually updated copies of the Janzen and Hallwachs master database are deposited in the National Museum of Natural History, Washington, DC (**USNM**) and iCLOUD through Amazon, as well as in the Janzen lab and the BioAlfa project in parallel with the Museo Nacional de Costa Rica.

Focus-stacked images of specimens were taken using a JVC digital camera mounted on a Leica microscope and compiled with the program Automontage. Non-distortional image post-photography was done in Adobe Photoshop.

#### DNA extraction and sequencing

Molecular work was carried out at the CBG using their standard protocols. A leg of each specimen was destructively sampled for DNA extraction using a glass fiber protocol (Ivanova et al. 2006). Extracted DNA was amplified for a 658-bp region near the 5’ terminus of the cytochrome *c* oxidase subunit I (COI) gene using standard insect primers LepF1 (5’-ATTCAACCAATCATAAAGATATTGG-3’) and LepR1 (5’-TAAACTTCTGGATGTCCAAAAAATCA-3’) (Ivanova and Grainger 2007). If initial amplification failed, additional amplifications were conducted following the established protocols using internal primer pairs, LepF1-C113R (130 bp) or LepF1-C_ANTMR1D (307 bp) and MLepF1-LepR1 (407 bp) to generate shorter overlapping sequences. Amplified products were sequenced using Sanger technology though the most recent were sequenced by SEQUEL II. Specimens that “failed” barcoding are not included here unless otherwise indicated. When included, they are usually identified by unambiguous morphological and ecological information equally possessed by others from ACG in that species.

#### Databases

Voucher codes are presented for all holotype specimens (and other barcoded individuals) treated herein; and all host caterpillars are individually vouchered to their individual records (yy-SRNP-xxxxx). Codes beginning with DHPARxxxxx are for the parasite (or hyperparasite) specimens reared from the caterpillar. The SRNP voucher codes are from the Janzen and Hallwachs’ database (http://janzen.sas.upenn.edu/caterpillars/database.lasso). Specimen voucher codes beginning with BIOUG are from the BOLD database (http://www.boldsystems.org), and are specimens obtained from ACG Malaise traps. The abundant collateral information obtainable from these two databases complements the species treatments in important ways. A brief introduction to what to look for and how the databases supplement the species treatments follows.

**Figure 4.**
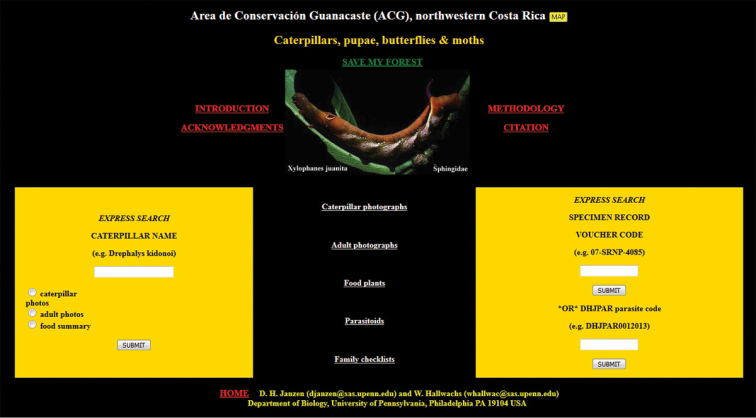
Home page of the Janzen-Hallwachs database.

**Figure 5.**
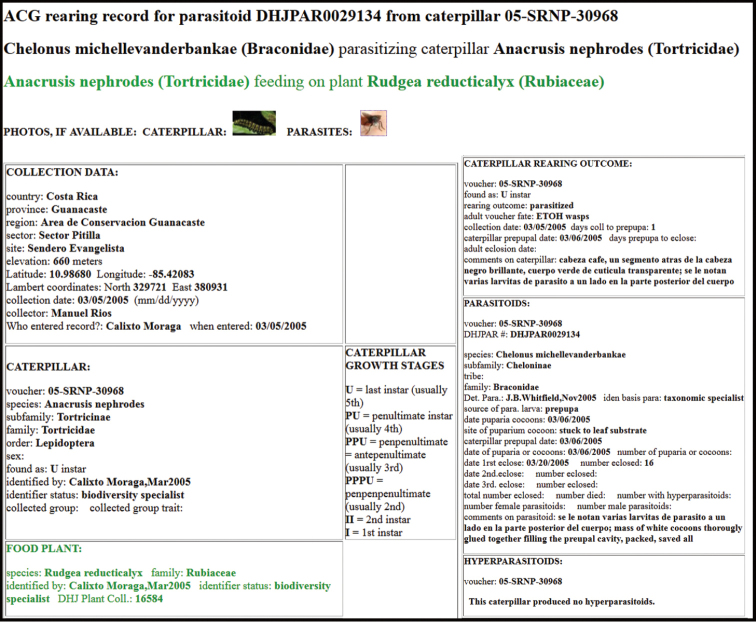
Specimen page on the Janzen-Hallwachs database for voucher DHJPAR0029134, *Chelonusmichellevanderbankae*.

**Figure 6.**
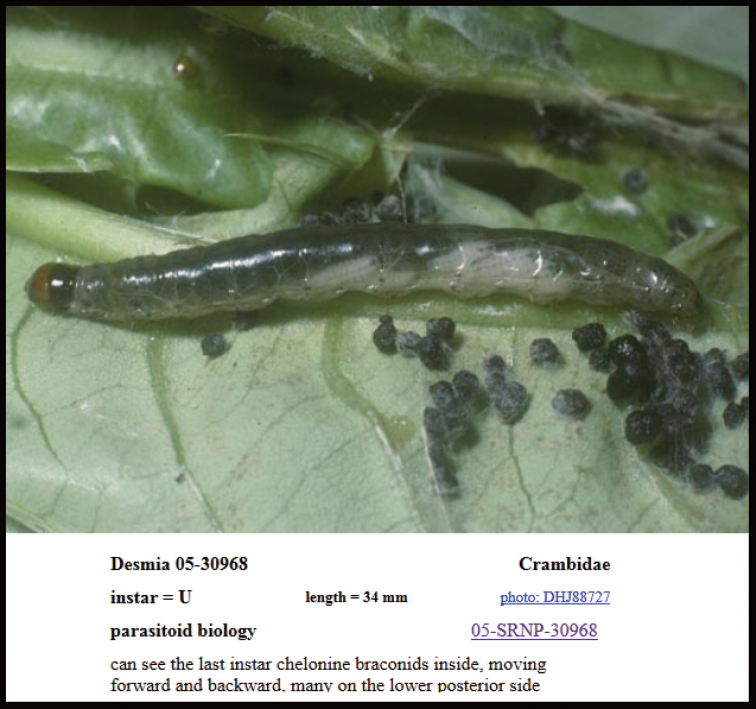
*Anacrucisnephrodes* (Tortricidae) last instar caterpillar parasitized by *Chelonusmichellevanderbankae*, specimen DHJPAR0029134. Note white wasp larvae in the abdomen, visible through the translucent cuticle. The caterpillar has been exposed for the photograph by opening its leaf and silk nest, littered with its own fecal pellets.

**Figure 7.**
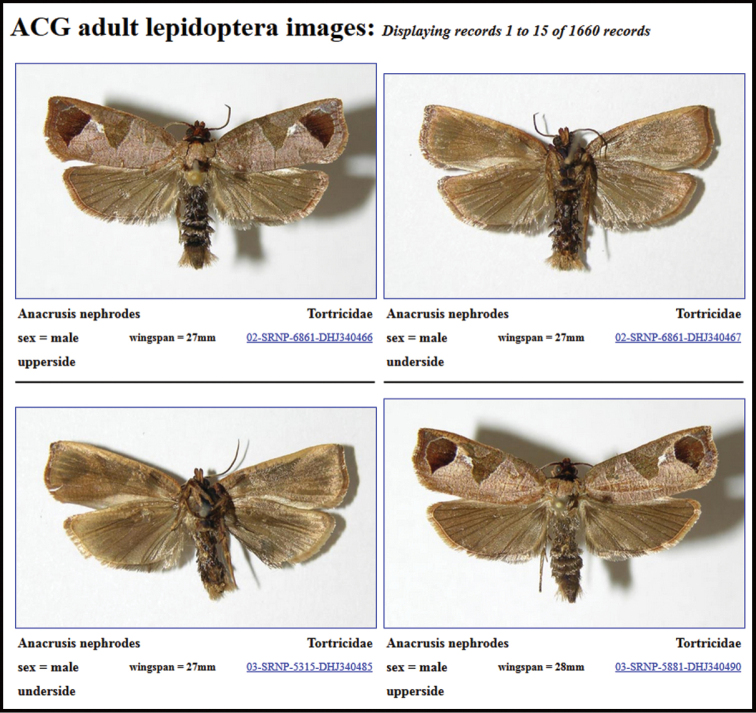
Adults of caterpillar species attacked by *Chelonusmichellevanderbankae*.

As mentioned above, the braconid specimens reared in the ACG have a DHJPAR code and their host caterpillar has a SRNP code. These codes can be entered on the home page of the Janzen and Hallwachs database (Fig. 4). As an example, the information associated with holotype of *Chelonusmichellevanderbankae*, DHJPAR0029134, appears as in Fig. 5, which is cropped to conserve space. The information includes details on the collection locality, host caterpillar and food plant information, dates of caterpillar collection and wasp eclosion, number of specimens emerging from the host (in the case of gregarious species), and notes on any interesting observations. In the case of a gregarious species, each wasp selected from the clutch to be DNA barcoded was given its own unique DHJPARxxxxx code, while its sibs remain tagged with the host yy-SRNP-xxxxxx code on their pin or in their alcohol preservative, a tube that is later deposited in the CNC. The written data can be in Spanish or English, or both. All dates are mm/dd/yyyy. Photos of the host caterpillar are obtained by clicking on the caterpillar image in the upper left of the image in Fig. 5. One of the five images of this parasitized caterpillar is shown in Fig. 6. Finally, if one wishes to see images of the adult of the host caterpillar, these can be found by returning to the home page, clicking on “Adult photographs” and typing the name of the host. In this example, some of the images generated are in Fig. 7.

**Figure 8.**
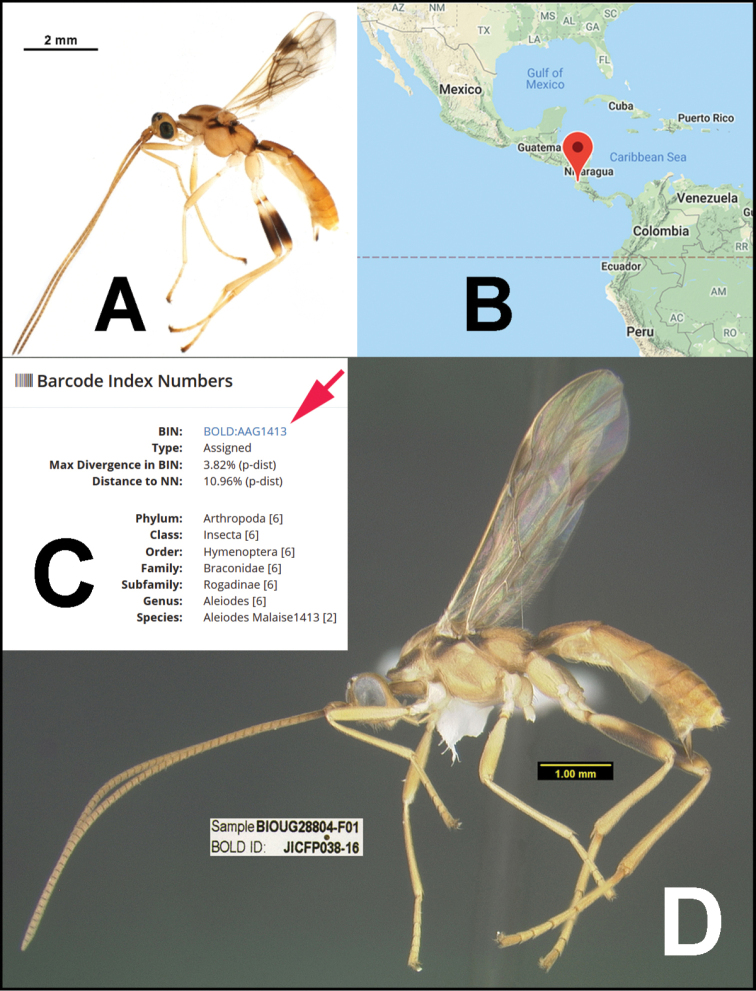
Data for *Aleiodesadrianaradulovae* specimen BIOUG28804-F01**A** image of specimen BIOUG28804-F01as it appears on BOLD**B** distribution map on BOLD (which can be enlarged for more detailed locality) **C**BIN code and other data on BOLD**D** image of specimen BIOUG28804-F01 as it appears in this publication, image taken after point-mounting. Note its interim species epithet in BOLD, which was updated to the “real” code-compliant species epithet *adrianaradulovae* when the name was made public, i.e., this publication.

**Figure 9.**
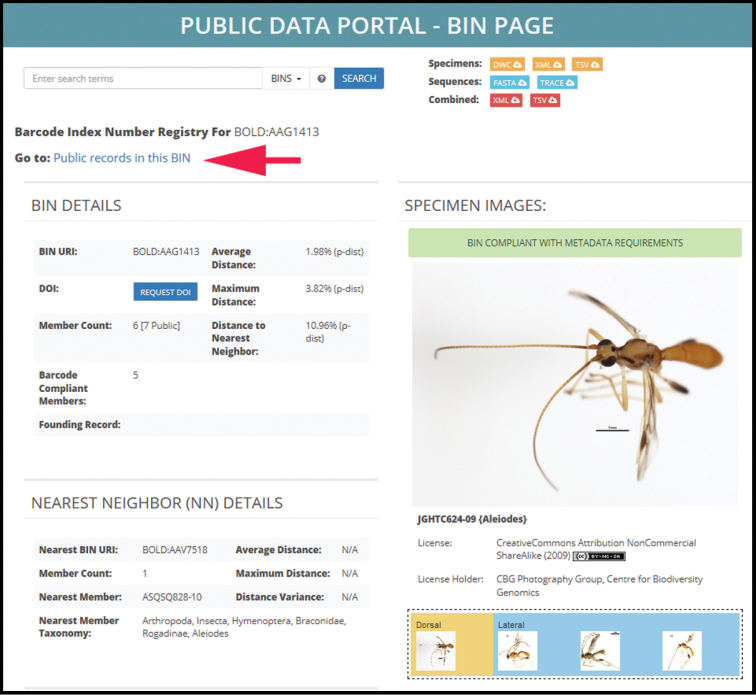
Partial contents of BOLD:AAG1413.

**Figure 10.**
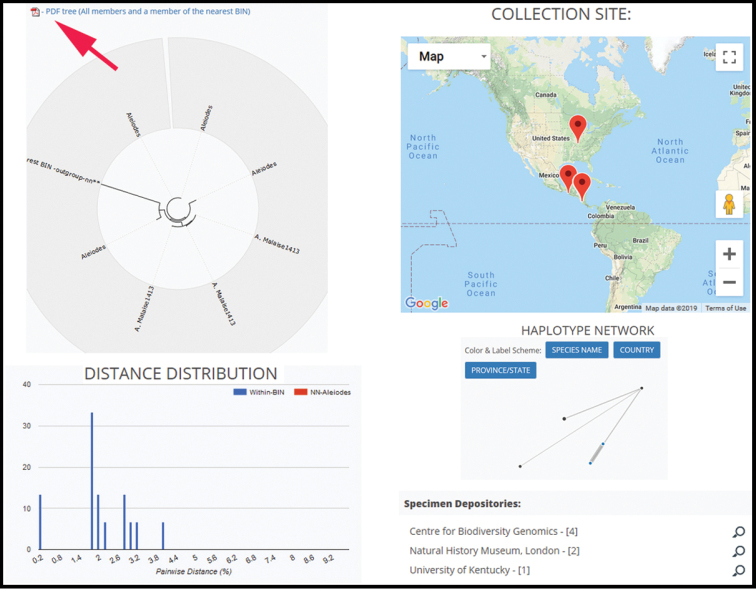
Partial contents of BOLD:AAG1413.

The BOLD website has a wealth of information related to the species that are treated in the chapters below. For example, selecting the link associated with the alphanumeric voucher code of the holotype of *Aleiodesadrianaradulovae*, BIOUG28804-F01, (http://boldsystems.org/index.php/Public_RecordView?processid=JICFP038-16) brings one to the specimen’s page on BOLD. Some of the contents of that page are shown in Fig. 8, including an image of the specimen (Fig. 8A). These images, taken by the Centre for Biodiversity Genomics Photography Group, are produced under significantly different lighting conditions than those in the following chapters and compliment them by showing wing venation more clearly and color more dramatically, even if not as realistically. For example, the image in Fig. 8D is our image of the same specimen as that of Fig. 8A which is from BOLD. Also included in the specimen’s home page is a locality map (Fig. 8B) and a link to the BIN page (Fig. 8C) to which the specimen belongs. Other information included in the specimen page, but not shown in Fig. 8 are locality data, collection data, and the specimen deposition locality. If the link to the specimen’s BIN (indicated with red arrow in Fig. 8) is selected, the BIN page is loaded, the contents of which are shown in Figs 9, 10. This information will increase in kind and bulk as the ACG local and BioAlfa national inventories grow over time; equally, name updates will be added as further ecological information and taxonomic revisions progress. In other words, these are dynamic databases. Over time they will link outward to other databases, such as GenBank and GBIF. While data for a particular specimen will remain tied to that specimen, these databases will contribute to species-based aggregators that will pool this information with that of other specimens from other places.

On the BIN page (Fig. 9) there is an image of a representative specimen from the BIN, and information on the COI barcode data of all members. The “BIN Details” section outlines a basic summary of records included in the BIN, including the number of members, the average genetic distance among them, the maximum genetic distance, and the distance to the closest member of the closest outgroup (Nearest Neighbor) which is normally another BIN. In the example in Fig. 9, BIN “BOLD:AAG1413” has the abnormally large maximum distance of 3.82% among its members and the Nearest Neighbor is almost 11% divergent. The BIN Details section also provides a “BIN DOI” which is a persistent URL that can be used in publications to access the BIN. If the BIN has not yet been assigned a DOI, a button is provided allowing a user to request a DOI.

The “Nearest Neighbor Details” section provides the details on the closest BIN, including its member count and the mean and maximum genetic distances among the members. It also includes the “Process ID” used in the CBG (not to be confused with the specimen voucher code) for the closest member of that BIN as well as the taxonomy for that BIN. In the ‘Specimen Images” section, there are four members of the BIN that have images. Clicking on the thumbnail images will bring them into the large image field above them.

**Figure 11.**
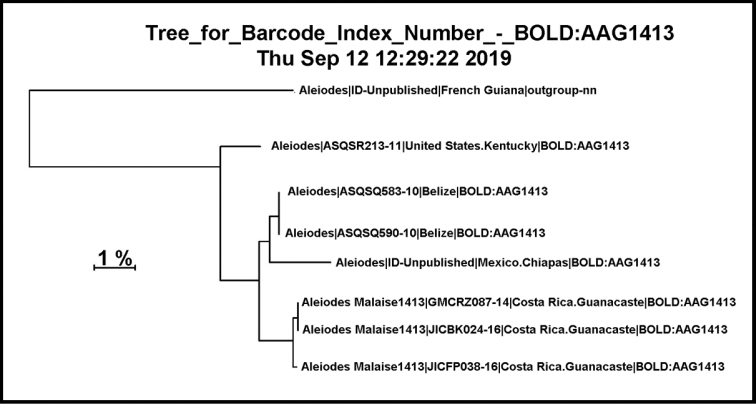
NJ tree of BINBOLD:AAG1413.

Also included in the BIN page (Fig. 10) is a distribution map of all specimens included in the BIN, a haplotype network, a neighbor joining tree that includes the nearest neighbor (outgroup) to the BIN, and a graph of the pairwise distances between the specimens in the BIN. Considering the six specimens from Costa Rica in that BIN, there are no doubts about species integrity; however, adding in the specimens from other countries leads immediately to the strong suspicion that there are one or two more species in that BIN. We have not been able to examine those specimens. Finally, one can download a PDF of the same tree by selecting the link indicted with the red arrow in Fig. 9. The NJ tree generated is shown in Fig. 11, which has been modified to conserve space. These trees have been very informative for the revisions of Costa Rican braconids that follow. In this example (Fig. 11) there are four barcoded specimens outside of our collections, one from Mexico, two from Belize and another from Kentucky. In this work, we describe only the specimens from ACG or Costa Rica as a whole, and, as would be the case for morphological identification and larger series, leave the unexamined individuals with a question mark hovering over them. Unless there are images on BOLD that clearly suggest that non-Costa Rican specimens are different species (we have several cases of this), we remain agnostic. Otherwise, for the present, we know they are either conspecific or potential cryptic species to the Costa Rican specimens. Just as in morphological approaches, many more specimens between these disparate localities need to be collected and barcoded to reach informed conclusions. Experience to date within ACG has been that partitioning of a BIN such as that in Fig. 11 will often lead to three clearly different species as ecological information accumulates and sample sizes for the BIN increase. One will be the holotype and there will be two more to describe, precisely as occurred with the originally named *Udranomiakikkawai* (Hesperiidae) (Janzen et al. 2017). If an older, morphology-based holotype is located that appears to be this species, when that holotype is DNA barcoded it will match just one or none of the Costa Rican BINs. In the case of *U.kikkawai*, the 1906 holotype from Venezuelan rain forest was found to match the specimens from Costa Rican rain forest, and two others, one from dry forest and the other from the intergrade, were described as two new species.

**Figure 12.**
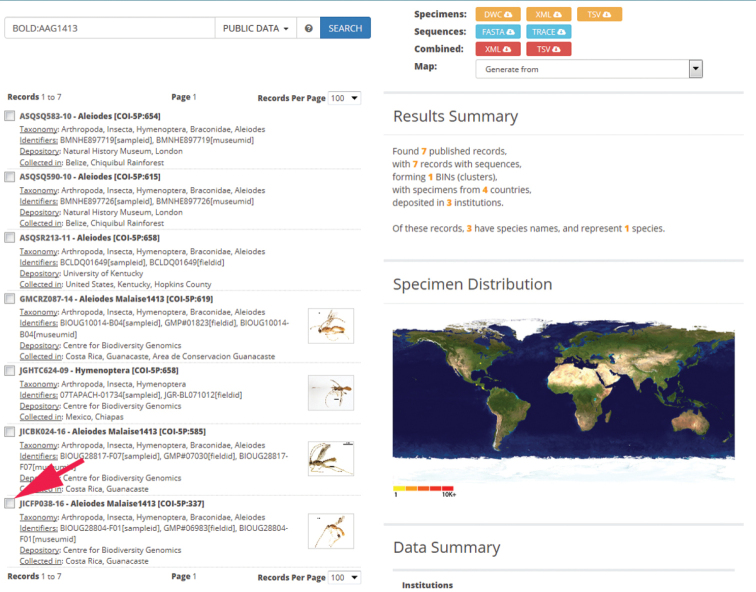
List of specimens in BINBOLD:AAG1413.

In Fig. 9 there is a red arrow pointing to the link “Public records in this BIN”. When this is selected, all of the specimens in the BIN are listed (Fig. 12) with links to public specimen pages with information such as that in Fig. 8.

From the list of the public records as shown in Fig. 12, users have access to download “Specimen Data,” “Sequences,” and “Trace Files” and generate an occurrence map. These options are in the upper right corner and provide various common formats. For example, “Specimen Data” can be downloaded in Darwin Core Format (DWC), XML, or TSV, and there is also the option to download the “Specimen Data” and “Sequence Data” in a combined file with either XML or TSV. Users may wish to download the sequences to use for further analysis such as Maximum Likelihood or Maximum Parsimony trees. We caution, however, that at present, BOLD does not store all of the collateral information for a particular ACG vouchered specimen. For that, the master database at http://janzen.upenn.edu needs to be consulted.

A focus of BOLD Systems is to support collaboration and research networks, and as such, the support team at BOLD helps to connect interested parties with the owners of the specimens with data in BOLD so that they might inquire for further details or gain access to physical specimens, samples, or DNA extracts. Requests for these particular physical objects can be directed to the CBG but for ACG the request should go to DHJ and WH, who will distribute accordingly.

#### Sequence analysis and species determination

Sequences (barcodes) of the Costa Rican specimens were assigned to multi-specimen operational taxonomic units called Barcode Index Numbers (BINs) (Ratnasingham and Hebert 2013) that were generated by BOLD (as in mentioned above, like assigning look-alikes to unit trays visually). For each subfamily treated in the following chapters we produced a NJ tree except for Ichneutinae and Proteropinae which are combined in one tree (Suppl. materials 1–10) containing all records from the New World contained in the BOLD database. This revealed many BINs containing extra-Costa Rican specimens such as displayed in Fig. 11. We comment on each of these in the individual species accounts with respect to their likelihood of being conspecific with their Costa Rican BIN partners. The NJ trees are also useful to identify sister-species and closest neighbors. Link to the BOLD treatments of the holotype and the “species”, as represented by the BIN, are provided for each species. The paratypes are listed as BOLD codes where detailed information and often images can be found for each specimen.

Morphology and host information were compared to the BIN assignments that “package” the members of the NJ tree into putative species. Most specimen groups (most BINs) were supported by all data sources and treated as one species. However, as mentioned previously, in the treatment of *Macrocentrus* that follows here, seven species are lumped into the same BIN (BOLD:ACK7466). This is commonplace in other taxonomic groups; there are many cases where a single BIN has been shown unambiguously to be two or more species by their biology and a deeper genome probe (e.g., Hebert et al. 2004; Janzen et al. 2017). There are several reasons for this phenomenon. First, the BIN algorithm separates groups at ca. a 2% COI sequence divergence (Ratnasingham and Hebert 2013). However, while in the early stages of evolutionary separation, sympatric species can easily have their own distinctive biology and evolutionary trajectories long before their barcodes have attained 2% difference through cumulative mutations. Second, even after long periods of separation, the barcode region, by chance, may not have differentiated to the 2% level. These “shallow splits” in an NJ tree are very difficult to interpret with small sample sizes, but with either large samples and/or ecological data, they become evident flags for the presence of two or more species in the BIN, species that are in turn defined and diagnosed by non-BIN traits as well as the trait of being in that BIN (e.g., Burns et al. 2007, 2008; Bertrand et al. 2014) or by doing a deep genome probe (Janzen et al. 2017). In hyperdiverse tropical taxa, such as many of those treated here, the only practical and reliable method of species-level identification will be by their BIN code (and by a more comprehensive DNA barcode when there are multiple species in a BIN), rather than by attempting species-level identification of specimens one-by-one based on their morphology. This process renders the binominal scientific name and its higher classification, along with all the other more traditional kinds of specimen-based information, to be extremely useful because of its inferential value. However, for a specimen that has been identified by its DNA, whether by a barcode or a more profound examination of its genome, the scientific name is collateral to be curated and used just as are other units of collateral.

#### Species diagnoses

Our species treatments are comprised of a link to the BIN on BOLD, a consensus COI barcode, and a lateral or dorsal image of the specimen. Added to this are certain details such as the type depository and holotype locality information. There are a few exceptions to this generality. In several cases more than one species fall in the same BIN and in these cases a morphological key or morphological diagnoses are given to separate these. To facilitate an ongoing revision of the Neotropical Macrocentrinae we have also included morphological diagnoses and more comprehensive imaging for this taxon.

To comply with the Code of Zoological Nomenclature, a word-based diagnosis or definition is necessary for each new species proposed. In the past this was usually in the form of a morphological key or a suite of morphological characters that distinguish the species from others being treated. In our case consensus barcodes of the COI barcode region were employed. These can be seen as a suite of character states that are universally shared by all members of a species and unique to the species. Consensus barcodes were generated using custom software written by one of us (BVB) implemented at phorid.net/DNAbarcode. Fasta files of barcodes for all specimens of a species were downloaded from BOLD and aligned in AliView (Larsson 2014). A consensus sequence was generated by running the first script on the *DNAbarcode* website, uploading the aligned fasta files. Consensus sequences for multiple species generated in this way were pasted into a new AliView file and realigned. The third script on *DNAbarcode* was then run, using the aligned consensus sequences.

Braconid specimens from the following New World countries appear to be relatively well-sampled in BOLD: Canada, USA, Belize, Argentina, French Guiana, and Mexico. There is a small number of cases where specimens from these countries fall in the same BIN as one of our Costa Rican species. More sampling between these disparate localities, and more genomic and/or morphological and behavioral data will help resolve these species-level cases, which are beyond the scope of this paper. Only rarely are there images of these extra-Costa Rican specimens that suggest that they are not conspecific. These are discussed in more detail under their respective species treatments. To reiterate and confirm: we fully understand that barcodes are not a panacea that will solve all species-limit questions, we simply suggest that they are the optimal available first step.

We attach 10 supplementary chapters for the 11 subfamilies treated here representing ten COI NJ trees, all downloaded from BOLD in November 2020. Each is a snapshot of the dynamic project database at http://djanzen.sas.upenn.edu, select fields of which are routinely updated to BOLD for public use and taxonomic treatment as in this work. The project database is forever dynamic owing to the need for constant updating of the names of species in the ever-shifting taxonomy of plants, hosts and parasites, as new publication and biological relationships emerge. We use the NJ trees as quick visual summaries of sample sizes per species, degrees of specialization of the parasites, flagging of species new to the project, indications of taxonomic puzzles, variability in barcode length, BIN composition (the equivalent of sorting morphological look-alikes into unit trays), and data-checking. Typically, what we consider to be conspecifics are grouped together in their own terminal clade. However, specimens with less than about 550 base pairs are potentially suspect as to their placement in a NJ tree. The shorter the barcode, the more likely it is to be misplaced on the tree, requiring taxonomic placement by other traits, such as morphology or host data or both. It is not uncommon for unrelated specimens with short sequences to group together; this is because the sequences that would group them with their conspecifics are not present and only conserved base pairs, common to many species, are present. Defectively short barcodes are not honored with a BIN code, even though they may group with BINed conspecifics. The arrangement of presumed conspecifics within a BIN into shallowly separated monophyletic groups has high potential for suggesting the presence of a species complex within the BIN, a moderately frequent occurrence (10%). Resolution of such species complexes requires ecological correlates, morphological correlates, and/or a deep genomic probe (e.g., Janzen et al 2017). The appendices contain only the specimens that were successfully barcoded, while all specimens obtained by the inventory are recorded in the project database, irrespective of success in naming.

## Chapter 2: Agathidinae

The key to genera by Sharkey and Chapman (2015) is outdated. We provide a new key that incorporates recent insights into generic limits and includes all New World genera. Agathidines are cosmopolitan and exclusively koinobiont endoparasitoids of caterpillars. They emerge from the host after the caterpillar is full-grown and has begun to spin or has already spun a cocoon. With one exception all are solitary. ACG agathidine genera were treated in the following publications: Sharkey et al. 2011a, Meierotto et al. (2019a), Kang et al. (2017), Janzen et al. (1998), Sharkey et al. (2016), and Sharkey et al. (2018). For the Agathidinae NJ tree, see Suppl. material 1.

### Key to the New World genera of Agathidinae

**Table:** 

1	A. Forewing venation greatly reduced; RS absent and crossvein r present only as a short stub; Neotropical, rare.	*** Mesocoelus ***
–	B. Forewing venation moderately reduced; apical abscissa of RS absent, or mostly so, but crossvein r complete to junction of RS; Neotropical, rare.	**2**
–	C. Forewing venation not significantly reduced; apical abscissa of RS complete or almost complete to wing margin; widespread, common.	**4**
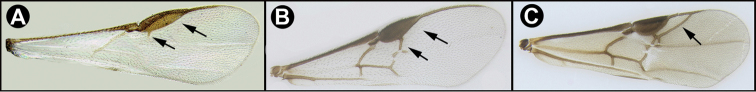		
2(1)	A. Hind wing subbasal (SB) cell 4-sided with vein Cub emanating from an angle in the cell AND/OR AA. Posterior surface of scutellum with a semi-circular or arc-shaped depression (post-scutellar depression)	***Therophilus* (in part)**
–	B. Hind wing subbasal (SB) cell 3-sided. If Cub vein is present, it emanates from a straight vein. BB. Post scutellar depression absent, but rugose sculpture usually present.	**3**
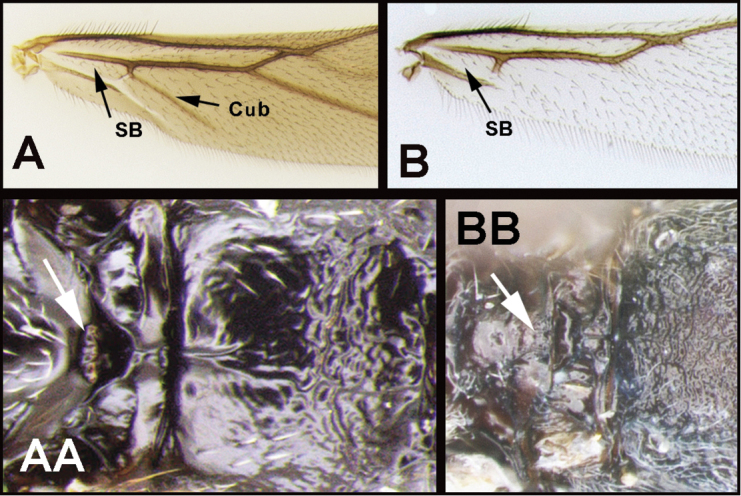		
3(2)	A. Median area of first tergum not raised above lateral portions and granulate or striogranulate. AA. Hind coxal cavities (HCC) open to metasomal foramen or narrowly closed and positioned partly above ventral margin of metasomal foramen (MF); ventral margin of metasomal foramen lacking a straight transverse carina	*** Plesiocoelus ***
–	B. Median area of first tergum raised above lateral portions, sculpture variable but often smooth or smoothly striate. BB. Hind coxal cavities closed and positioned completely below the metasomal foramen; ventral margin of metasomal foramen with a strong, relatively straight transverse carina (TC)	***Aerophilus* (in part)**
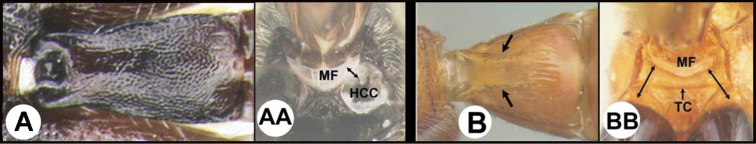		
4(1)	A. Fore tarsal claws bifid	**5**
–	B. Fore tarsal claws simple, with distinct basal lobe.	**9**
–	C. Fore tarsal claws simple, lacking a distinct basal lobe.	**31**
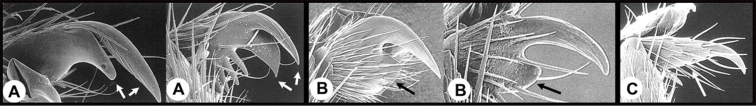		
5	A. Forewing areolet quadrate, not or only slightly narrower anteriorly. AA. Ovipositor as long as or longer than half the length of metasoma	**7**
–	B. Forewing areolet triangular, or if quadrate much narrower anteriorly. BB. Ovipositor shorter than half the length of metasoma.	**6**
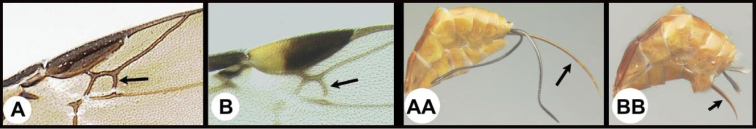		
6(5)	A. Gena expanded into a flange posteriorly; malar space (MS) more than ½ length of eye height (EH); Neotropical, rare	*** Hemichoma ***
–	B. Gena not modified into a flange posteriorly; malar space (MS) less than ½ length of eye height (EH); widespread, common	*** Zelomorpha ***
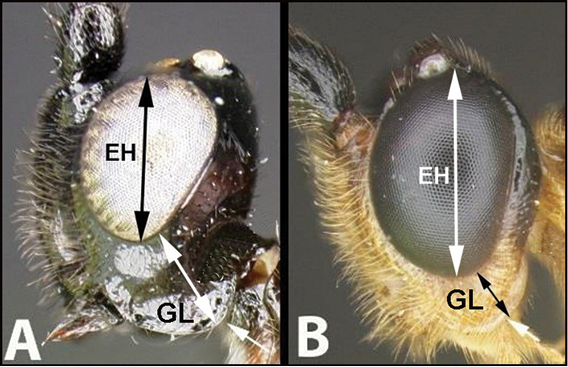		
7(5)	A. Body predominantly orange/yellow. AA. Frons bordered by a carina posteriorly; widespread, common	**8**
–	B. Body predominantly black. BB. Frons not bordered by a carina posteriorly; southern USA through the tropical Neotropics, uncommon	*** Zacremnops ***
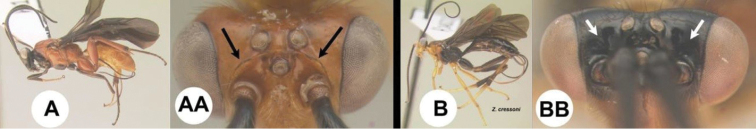		
8(7)	A. Propodeum and hind coxa with granulate sculpture; first metasomal tergum ca. 3 × wider at apex than at base; rare; Neotropical, rare	*** Labagathis ***
–	B. Propodeum and hind coxa lacking granulate sculpture; first metasomal tergum not ca. 2 × wider at apex than at base; widespread, relatively common	*** Cremnops ***
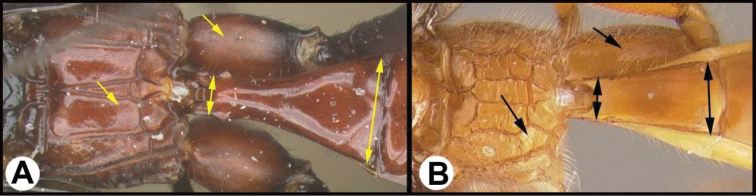		
9(4)	A. Notauli present	**10**
–	B. Notauli absent	**25**
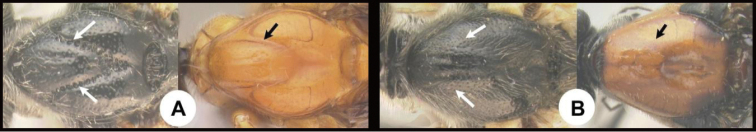		
10(9)	A. Ventral margin of clypeus projecting; width of temple longer than width of eye in lateral view; Nearctic, rare	*** Gelastagathis ***
–	B. Ventral margin of clypeus not projecting; width of temple shorter than width of eye in lateral view; widespread, common	**11**
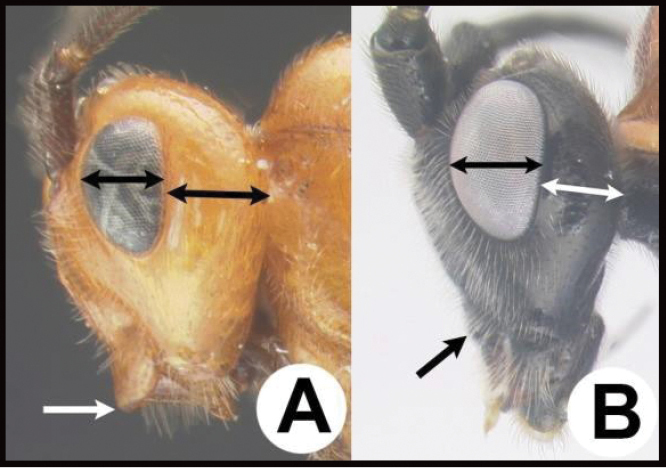		
11(10)	A. Frons bordered by carinae or grooves posteriorly.	**12**
–	B. Frons not bordered by carinae or grooves posteriorly.	**14**
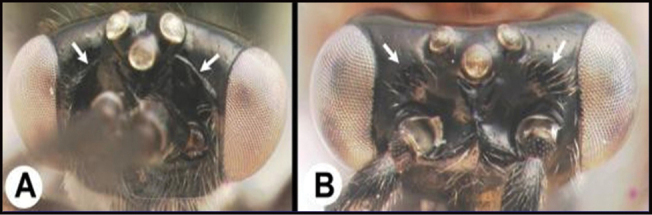		
12(11)	A. Hind coxa with granulate sculpture. AA. Second submarginal cell minute or absent; Neotropical, rare	*** Trachagathis ***
–	B. Hind coxa smooth, lacking granulate sculpture. **BB.** Second submarginal cell of normal dimensions; widespread, common.	**13**
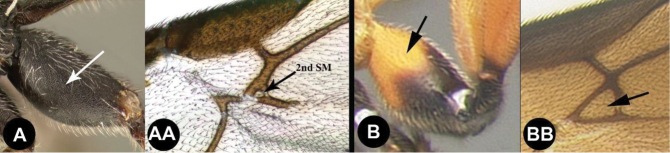		
13(12)	A. First metasomal tergum smooth with two widely spaced converging carinae forming a teardrop-shaped basal area; Neotropical, rare	*** Pharpa ***
–	B. First metasomal tergum usually smooth and convex, or BB. With a median longitudinal carina, or, BBB. Rarely with 2 carinae, in which case the tergum has more extensive sculpture; widespread, common	*** Alabagrus ***
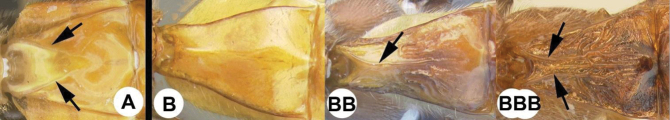		
14(11)	A. Malar space (MS) distinctly greater than ½ eye height (EH); head shape in frontal view elongate, at least as high (measured from ventral margin of clypeus) as wide.	**15**
–	B. Malar space USUALLY (95%) equal to or less than ½ eye height; head shape in frontal view wide, wider than high (measured from ventral margin of clypeus)	**16**
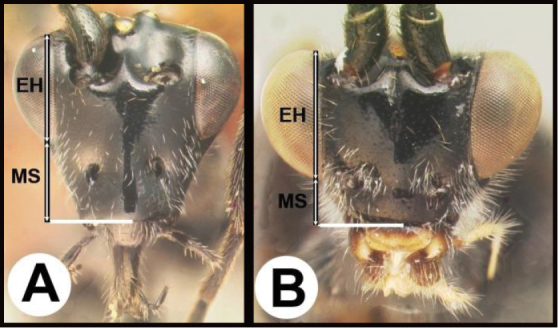		
15(14)	A. Third tergum completely smooth; pair of carinae on first tergum not prominent. AA. Hind coxal cavities (HCC) open to metasomal foramen or narrowly closed and positioned partly above ventral margin of metasomal foramen (MF); common in Nearctic, very rare in Neotropics	***Agathis* (in part)**
–	B. Third tergum usually (95%) partly or completely sculptured, sculpture often confined to narrow line along transverse depression; pair of carinae on first tergum prominent. BB. Hind coxal cavities closed and positioned completely below the metasomal foramen; ventral margin of metasomal foramen with a strong, relatively straight transverse carina (TC); widespread, common	***Aerophilus* (in part)**
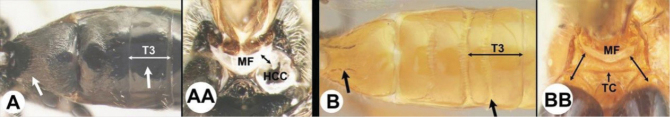		
16(14)	A. Propodeal spiracle elongate, more than 2 × longer than wide; widespread, common	*** Pneumagathis ***
–	B. Propodeal spiracle circular or ovoid, not more than 2 × longer than wide	**17**
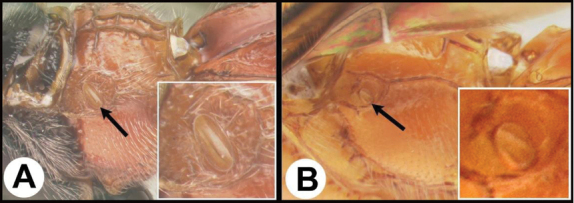		
17(16)	A. Pair of carinae on first tergum NOT prominent. AA. Hind coxal cavities (HCC) open to metasomal foramen or narrowly closed and positioned partly above ventral margin of metasomal foramen (MF).	**18**
–	B. Pair of carinae on first tergum prominent; BB. Hind coxal cavities closed and positioned below the metasomal foramen; ventral margin of metasomal foramen with a strong, relatively straight transverse carina (TC); widespread, common	***Aerophilus* (in part)**
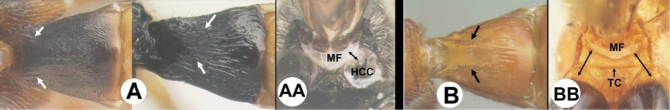		
18(17)	A. First tergum completely smooth, or rarely with some punctures posterolaterally	**20**
–	B. First tergum with sculpture	**19**
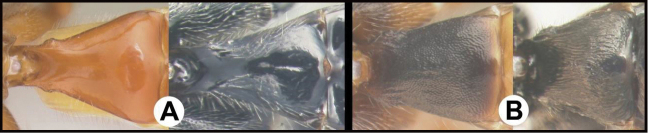		
19(18)	A. Vein Cub of hind wing long and partly tubular, apical margin of subbasal (SB) cell angled; widespread, common.	***Therophilus* (in part)**
–	B. Vein Cub of hind wing weak or absent and never tubular; apical margin of subbasal cell (SB) straight. Nearctic and northern Neotropics (i.e., Mexico and Central America), much less common	***Agathirsia* (in part)**
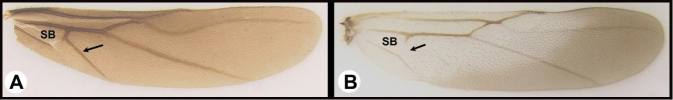		
20(18)	A. Notauli pitted or crenulate	**21**
–	B. Notauli smooth	**24**
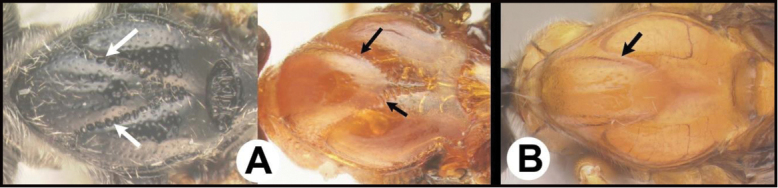		
21(20)	A. Third tergum usually entirely smooth or weakly and partly coriarious (leather-like), if with different sculpture (especially in transverse depressions), then pair of longitudinal carinae on first metasomal tergum weaker than in B or absent. AA. Hind coxal cavities (HCC) open to metasomal foramen or narrowly closed and positioned partly above ventral margin of metasomal foramen (MF)	**22**
–	B. Third tergum usually partly or completely sculptured, sculpture often confined to narrow line along transverse depression; pair of longitudinal carinae on first metasomal tergum present and extending past spiracles. BB. Hind coxal cavities closed and positioned entirely below the metasomal foramen (MF); ventral margin of metasomal foramen with a strong, relatively straight transverse carina (TC); widespread, common	***Aerophilus*** (in part).
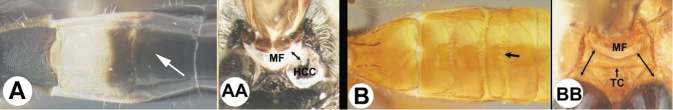		
22(21)	A. First tergum partly or completely granulate; widespread, common	*** Neothlipsis ***
–	B. First tergum otherwise sculptured, usually striate or rugostriate.	**23**
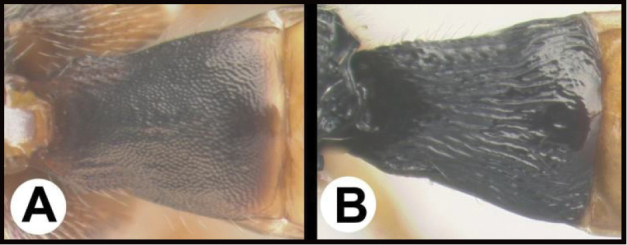		
23(22)	A. Posterior apex of scutellum with a distinct depression in the form of a semicircle or two distinct pits; widespread, common.	***Therophilus* (in part)**
–	B. Posterior apex of scutellum lacking depression, smooth to rugose; common in Nearctic, very rare in Neotropics.	***Agathis* (in part)**
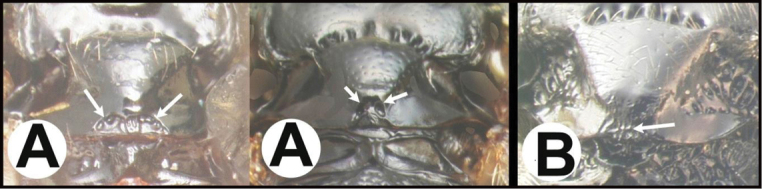		
24(20)	A. Propleuron with a distinct protuberance; gena expanded into an acute angle posteroventrally; Neotropical, rare	*** Zamicrodus ***
–	B. Propleuron flat or weakly convex, lacking a distinct protuberance; genae not expanded and rounded posteroventrally; Neotropical, rare	*** Aphelagathis ***
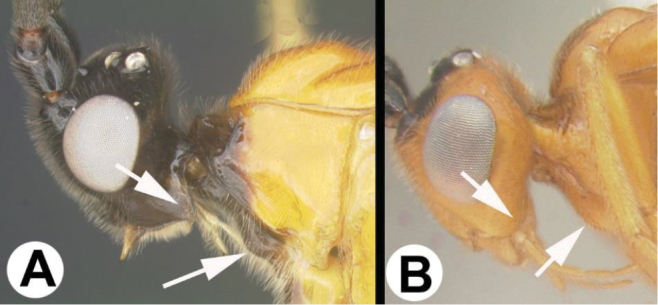		
25(9)	A. Second submarginal cell of forewing quadrate. AA. Cub vein of hind wing present and often tubular; subbasal cell angled distally at junction of Cub; Chile and southern Argentina (Note: Unlike the better known north temperate group of *Earinus*, members of this assemblage are found only in southern and high-altitude South America, and may deserve separate generic status), common	***Earinus* (in part)**
–	B. Not combining the above character states; second submarginal cell usually triangular. BB. Cub vein of hind wing usually absent or not tubular, and subbasal sell not angled distally.	**26**
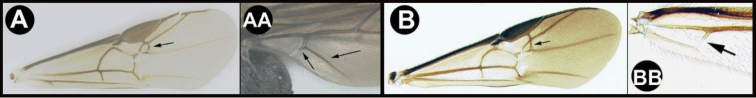		
26(25)	A. Forewing vein RS+M present and complete, though sometimes weaker at midlength; Nearctic, common.	***Earinus* (in part)**
–	B. Forewing vein RS+M completely or mostly absent	**27**
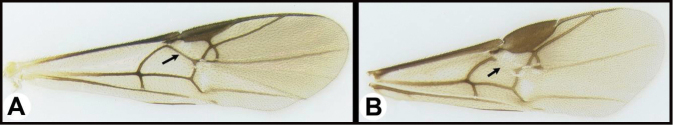		
27(26)	A. Third tergum completely smooth. AA. Hind coxal cavities (HCC) open to metasomal foramen (MF), or narrowly closed such that the ventral part of the metasomal foramen is below the dorsal margin of the hind coxal cavities	**28**
–	B. Third tergum usually (95% of specimens) partly or completely sculptured, sculpture often confined to narrow lines along transverse depressions. BB. Hind coxal cavities closed and positioned completely below the metasomal foramen; ventral margin of metasomal foramen with a strong, relatively straight transverse carina; widespread, common	***Aerophilus* (in part)**
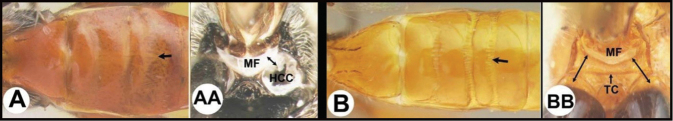		
28(27)	A. Spurious vein, RS2b, well-developed. AA. Ovipositor barely exerted, much shorter than metasoma. Neotropical, very rare	*** Marjoriella ***
–	B. Spurious vein, RS2b, lacking. BB. Ovipositor at least as long as metasoma. Widespread, common	**29**
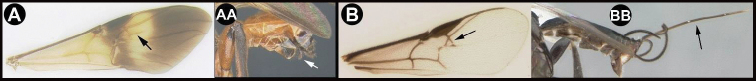		
29(28)	A. Second submarginal cell smaller than its dorsal stem; apical abscissa of RS curving towards fore margin of wing; Neotropical, very rare	*** Smithagathis ***
–	B. Second submarginal cell larger than its dorsal stem; apical abscissa of RS straight	**30**
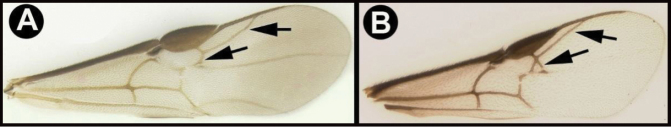		
30(29)	A. Posterolateral corner of gena sharp; propleuron with a protuberance; Neotropical, rare	*** Amputoearinus ***
–	B. Posterolateral corner of gena rounded; propleuron evenly convex, lacking a protuberance; widespread, common.	*** Lytopylus ***
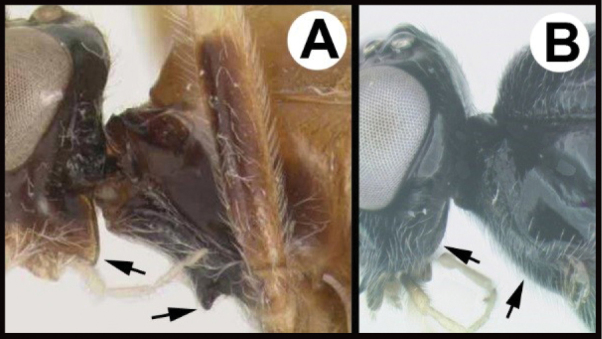		
31(4)	A. Notauli absent, mesoscutum completely smooth; Neotropical, rare	*** Sesioctonus ***
–	B. Notauli present though sometimes only indicated anteriorly or posteriorly; widespread and common in Nearctic, rare in Neotropics.	**32**
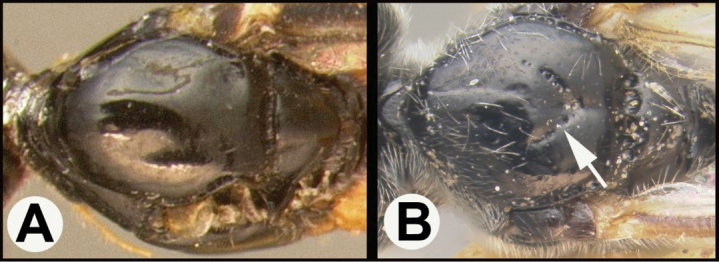		
32(31)	A. First tergum smooth, lacking microsculpture, at most with punctures laterally; Nearctic and Central America, rare	**33**
–	B. First tergum with microsculpture, usually in the form of longitudinal striae or rugae; widespread and common in Nearctic, extremely rare in Neotropics	***Agathis* (in part)**
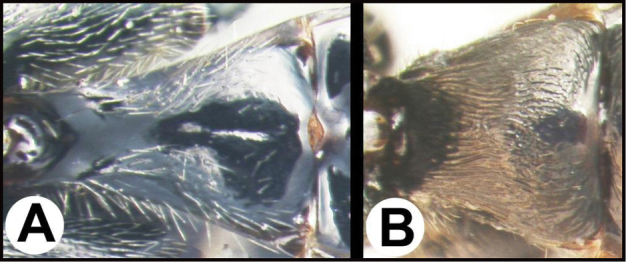		
33(32)	A. Ovipositor barely exerted, shorter than half the length of metasoma; Nearctic and Central America (all species of Crassomicrodus key here)	*** Crassomicrodus ***
–	B. Ovipositor at least as long as half the metasoma, often much longer; Nearctic and Central America, rare (few species key here)	***Agathirsia* (in part)**
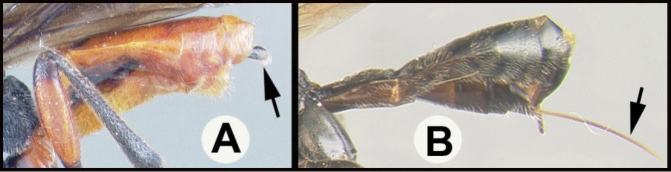		

#### *Aerophilus* Szépligeti, 1902

*Aerophilus* is worldwide in distribution with hundreds (described plus undescribed) of species in the New World. Members are parasitoids of a wide range of caterpillar families. The genus is well represented in both the Nearctic and Neotropical realms. Sharkey et al. (2016) revised the eastern Nearctic species, and Sharkey et al. (2011a) revised and keyed the Costa Rican species. The latter revision employed the name *Lytopylus* in error, and Sharkey et al. (2016) subsequently transferred all of the species described under *Lytopylus* in that paper to *Aerophilus* Szépligeti, 1902.

##### Aerophilus paulmarshi

Taxon classificationAnimaliaHymenopteraBraconidae

Sharkey sp. nov.

http://zoobank.org/0E45721B-53E5-4DD9-8547-8A3C88EEFA0E

Figure 13

###### Diagnostics.

BOLD:ADC6602. Consensus barcode. TATTTTATATTTTATTTTTGGAATTTGATCAGGAATTTTAGGTTTATCAATAAGAATAATTATTCGAATAGAATTAAGATTAGGGGGTAATTTAATTGGTAAT---GATCAAATTTATAATAGAATTGTTTCTGCTCATGCTTTTATTATAATTTTTTTTATAGTAATACCAATTATAATTGGAGGTTTTGGAAATTGATTAGTTCCTTTAATATTAGGGGGACCTGATATAGCTTTTCCTCGTATAAATAATATAAGATTTTGATTATTAATTCCTTCATTATTAATATTAATTTTAAGATCTTTAATTAATATTGGAGTTGGTACAGGATGAACAGTTTATCCTCCTTTATCAATAAATATGAGTCATAGAGGAATATCAGTAGATTTAGCTATTTTTTCTTTACATATTGCAGGAATTTCTTCTATTATAGGAGCAATAAATTTTATTACAACTATTATAAATATATGAATAATCAATGTTAAAATTGATAAAATACCTTTATTAGTATGATCAATTTTTATTTCTGCTATTTTATTATTATTATCATTACCAGTATTAGCTGGAGCTATTACAATATTATTAACTGATCGAAATTTAAATACAAGATTTTTTGATCCATCAGGAGGAGGAGATCC----------------------.

###### Holotype ♀.

Guanacaste, Sector Pailas Dos, PL12-3, 10.7631, -85.6138, 820 meters, Malaise trap, 6/iii/2014. Depository: CNC.

***Host data*.** None.

***Holotype voucher code*.**BIOUG29687-E06.

###### Paratypes.

None.

###### Etymology.

Named in honor of Paul Marsh, eminent braconidologist.

###### Note.

In the key provided by Sharkey et al. (2011a), *A.paulmarshi* runs to *A.robpringlei* (as *Lytopylusrobpringlei*). It differs from the latter in many characters; the most conspicuous is the head color, which is almost entirely black in *A.robpringlei* and pale orange-yellow in *A.paulmarshi*.

**Figure 13.**
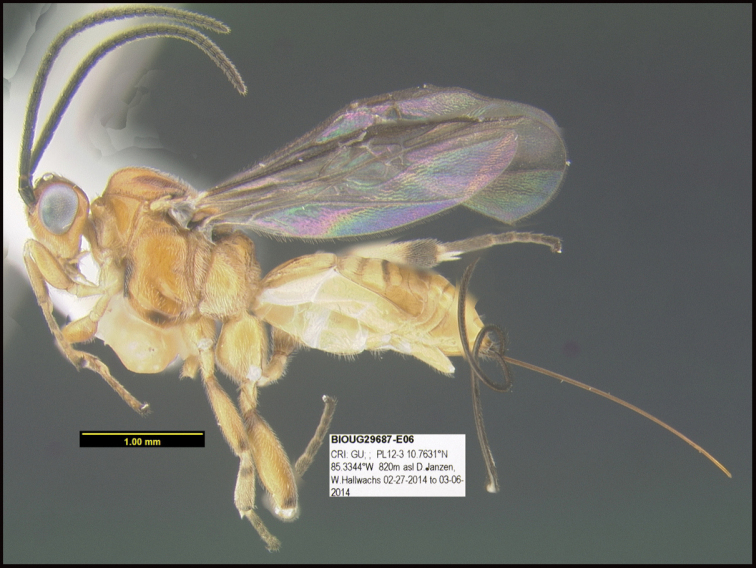
*Aerophiluspaulmarshi*, holotype.

#### *Mesocoelus* Schulz, 1911

Members are widespread but rarely collected in the neotropics. The extremely reduced venation of *Mesocoelus* is rare among agathidines, shared only with the Old World genus *Aneurobracon* (Agathidinae). The two described species are both from the Caribbean (holotypes from St. Vincent and Cuba). Reported hosts are *Chilocampylapsidiella* and *Acrocercops* sp., leaf-miners in the family Gracillariidae. No new host records for the genus are reported here.

##### Mesocoelus davidsmithi

Taxon classificationAnimaliaHymenopteraBraconidae

Sharkey sp. nov.

http://zoobank.org/E854173C-4DEA-4F89-8446-6998B21C18E4

Figure 14

###### Diagnostics.

BOLD:AAV4319. Consensus barcode. AATTTTATATTTTATTTTTGGAGTATGGGCAGGAATTTTAGGGTTATCAATAAGATTAATTATTCGTATAGAATTAAGAGTTATTGGAAATTTTATTGGTAATGATCAGATTTATAATAGGATTGTTACAGCTCATGCATTTATTATAATTTTTTTTATAGTTATACCAATTATAATTGGAGGGTTTGGTAATTGATTAGTTCCATTAATAGTAGGAGGACCTGATATAGCTTTCCCTCGAATAAATAATATAAGATTTTGATTATTAATTCCTTCTTTATTATTATTAATTTTAAGGTCAATAGTTAATGTTGGGGTAGGAACTGGGTGAACAGTTTACCCTCCTTTATCTTTAAATATAAGGCATAGAGGGATTTCTGTTGATTTGGCTATTTTTTCTTTACACATTGCAGGAGTTTCTTCAATTATAGGAGCTATAAACTTTATTACTACTATTTTAAATATATGAATCATTAATATTAAAATTGATAAAATACCTTTATTAGTATGATCTATTTTAATTACTGCAATTTTATTATTATTATCATTACCTGTATTAGCCGGAGCAATTACTATATTGTTAACTGATCGTAATTTAAATACAAGATTTTTTGACCCGACAGGAGGAGGGGATCCTATTTTATATCAACATTTATTT. Of the two described species, *M.davidsmithi* is closest to *M.laeviceps* in that they both lack distinct notauli. To differentiate these two, *M.davidsmithi* has 27 flagellomeres, whereas *M.laeviceps* has between 19 and 22.

###### Holotype ♀.

Heredia, 6 km ENE Vara Blanca, 10.183, -84.12, 2000 meters, 09/iv/2002, Malaise trap. Depository: CNC.

***Host data*.** None.

***Holotype voucher code*.**H1731.

###### Paratypes.

None.

###### Etymology.

The species is named in honor of Dave Smith, eminent symphytologist.

**Figure 14.**
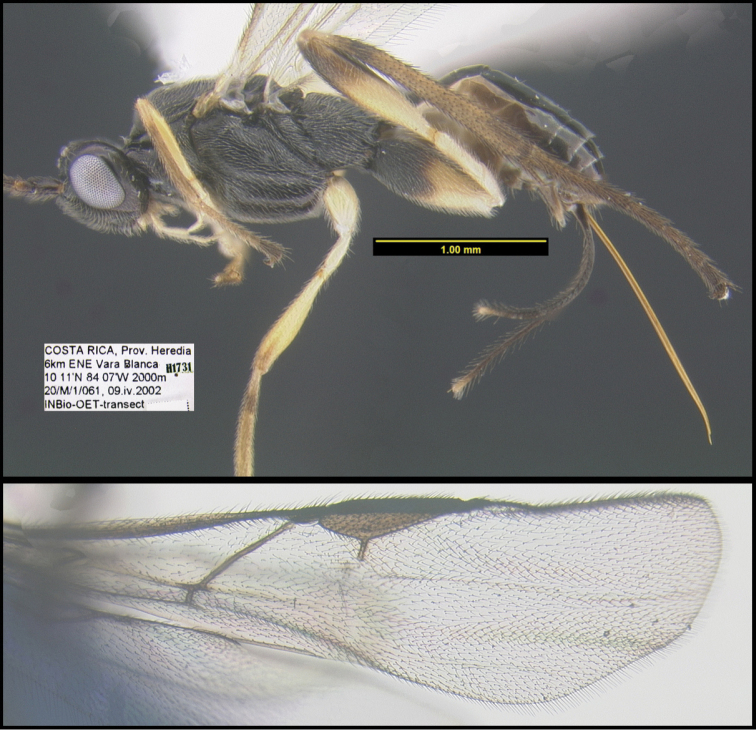
*Mesocoelusdavidsmithi*, holotype.

#### *Neothlipsis* Sharkey, 2011

*Neothlipsis* is widespread in the Nearctic and northern Neotropical region. There may be ca. 70 species including undescribed species. The sole host record is *Sameamultiplicalis* (Guenée) (Crambidae). Sharkey et al. (2011b) included eleven species but omitted the following: *Neothlipsissmithi* (Ashmead), new combination for *Microdussmithi* Ashmead, 1894; *Neothlipsispygmaeus* (Enderlein), new combination for *Microduspygmaeus* Enderlein, 1920; and *Neothlipsisunicinctus* (Ashmead), new combination for *Microdusunicinctus* Ashmead, 1894. All of the holotypes of these species were examined by MJS, and all are found in Mesoamerica including the Caribbean.

##### Neothlipsis bobkulai

Taxon classificationAnimaliaHymenopteraBraconidae

Sharkey sp. nov.

http://zoobank.org/1DEDA50E-D2D7-48E6-9CC2-8E2431ED598C

Figure 15

###### Diagnostics.

BOLD:ADB1901. Consensus barcode. TCTGCTCATGCTTTTATTATAATTTTTTTTATAGTTATACCAATTATAATTGGAGGATTTGGTAATTGATTAGTTCCTTTAATATTAGGAGGTCCTGATATAGCTTTTCCTCGTATAAATAATATAAGATTTTGATTATTAATTCCTTCATTATTAATATTGATTTCTAGATCTATAATTAATATTGGTGTTGGTACAGGTTGAACTGTTTATCCTCCTTTATCTTTAAATTTAAGACATAGTGGAATTTCTGTAGATTTAGCTATTTTTTCTTTACATATTGCAGGAATTTCTTCTATTATGGGAGCAATAAATTTTATTACAACTATTTTAAATATATGAATAATAAATATTAAAATTGATAAAATACCATTATTAATTTGATCTATTTTTATTACAGCTATTTTATTATTATTATCTTTACCAGTATTAGCTGGGGCAATTACAATATTATTAACAGATCGAAATTTAAATACTAGATTTTTTGATCCTACAGGAGGGGGAGATCCA. Similar to *Neothlipsistaeniativentris* (Enderlein, 1920) but differing slightly in color, with the pale yellow on the head of *N.taeniativentris* more extensive.

###### Holotype ♀.

Guanacaste, Sector Pailas Dos, PL12-3, 10.7631, -85.6138, 820 meters, Malaise trap, 5/xii/2013. Depository: CNC.

***Host data*.** None.

***Holotype voucher code*.**BIOUG29112-G02.

###### Paratypes.

BIOUG29646-F07, BIOUG29620-B06, BIOUG29646-E01.

###### Etymology.

This species is named in honor of Bob Kula, renowned braconidologist.

**Figure 15.**
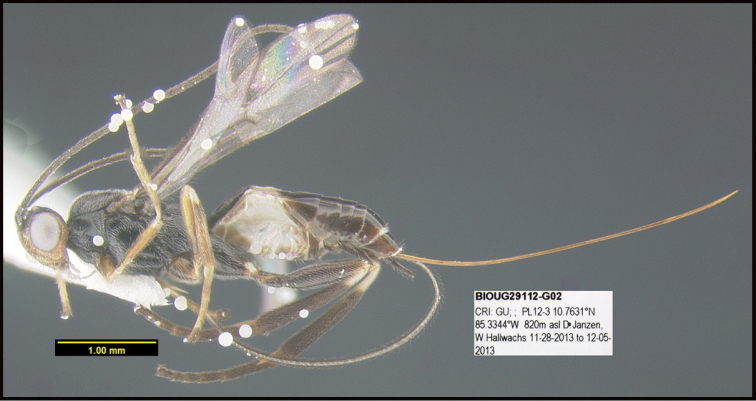
*Neothlipsisbobkulai*, holotype.

#### *Plesiocoelus* van Achterberg, 1990

Members are widespread but rarely collected in the neotropics. It is unlikely that there are more than ten species including undescribed species The holotype of the only described species from the New World, *P.bassiformis*, is from Ecuador. van Achterberg (1990a) also included specimens from Colombia and Honduras under that name. Long and van Achterberg (2016) described a new species from Vietnam. We report the first host records for the genus, *Brenthia* spp. (Lepidoptera: Choreutidae).

Bortoni and Penteado-Dias (2015) described two new species under *Plesiocoelus*, however, they both belong to other genera: *Therophilusanomalus* (Bortoni and Penteado-Dias) new combination for *Plesiocoelusanomalus* Bortoni & Penteado-Dias, 2015; *Aerophilusareolatus* (Bortoni and Penteado-Dias) new combination for *Plesiocoelusareolatus* Bortoni & Penteado-Dias, 2015. See the key to genera above that incorporates these aberrant species with reduced wing venation.

##### Plesiocoelus vanachterbergi

Taxon classificationAnimaliaHymenopteraBraconidae

Sharkey sp. nov.

http://zoobank.org/B61B270B-8ACB-42B4-B820-6C86CAF38514

Figure 16

###### Diagnostics.

BOLD:ABX6701. Consensus barcode. AATTTTATATTTTATTTTCGGAATTTGATCAGGAATTTTGGGRTTATCAATAAGWTTGGTTATTCGAATGGAATTAAGAATTACTAGAAATTTTATTGGTAATGATCAAATTTATAATAGTATTGTT-CTRCTCATGCTTTTATTATAATTTTTTTTATGGTAATACCTATTATGATTGGGGGATTYGGAAATTGATTAATTCCTTTAATATTAGGAGGTCCTGATATAGCTTTYCCTCGAATAAATAATATAAGATTTTGATTATTAATTCCATCTTTATTATTATTAATTTCTAGCTCAATTATTAATGTTGGAGTAGGAACAGGATGAACAGTTTATCCTCCTTTATCATTAAATTTAAGTCATAGTGGAATTTCAGTAGATTTAGCTATTTTTTCTTTACATATTGCTGGAATTTCATCAATTATAGGAGCAATAAATTTTATTACT-CTATTTTAAATATATGRATAATTAAAATTAATGTTGATAAAATAACTTTATTGATTTGRTCAATTTTAATTACAGCAATTTTATTATTATTATCATTACCGGTWTTAGCAGGAGCAATTACAATATTATTAACAGATCGAAATTTAAATACTAGATTTTTTGATCCTTCAGGAGGGGGAGATCCTATTTTATATCAACATTTATTT.

###### Holotype ♀.

Alajuela, Sector Rincon Rain Forest, Jacobo, 10.94076, -85.3177, 461 meters, caterpillar collection date: 28/vii/2013, wasp eclosion date: 8/viii/2013. Depository: CNC.

***Host data*.** Parasitoid of *Brenthia* Janzen02 (Choreutidae) feeding on leaves of *Rhynchosiacalycosa* (Fabaceae).

***Caterpillar and holotype voucher codes*.** 13-SRNP-69969, DHJPAR0053046.

###### Paratypes.

Hosts = *Brenthia* Janzen02, *Brenthia* Janzen05, *Brenthia* Janzen14, *Brenthia* Janzen15. DHJPAR0049867, DHJPAR0048215, DHJPAR0050114, DHJPAR0053040, DHJPAR0053044, DHJPAR0053045, DHJPAR0053033, DHJPAR0045360. Depository: CNC.

###### Etymology.

This species is named in honor of Kees van Achterberg, eminent braconidologist and author of the genus.

**Figure 16.**
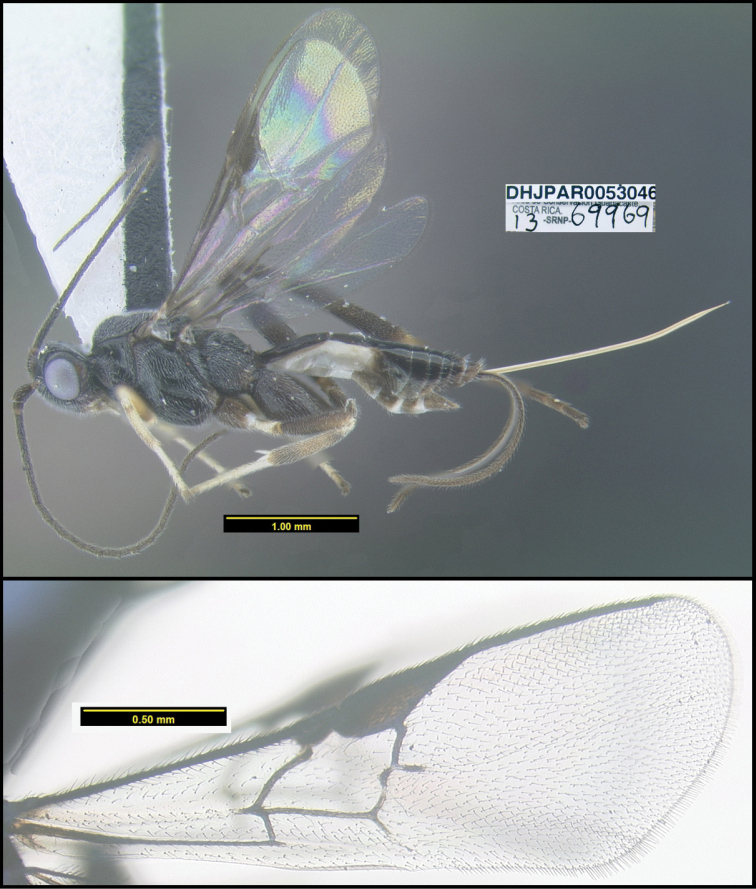
*Plesiocoelusvanachterbergi*, holotype.

#### *Pneumagathis* Sharkey, 2015

Members of *Pneumagathis* are restricted to North and Central America. There appear to be only two species, *P.erythrogastra* (Neotropical) and *P.spiracularis* (Nearctic), both of which are parasitoids of Hesperiidae.

##### Pneumagathis erythrogastra

Taxon classificationAnimaliaHymenopteraBraconidae

(Cameron, 1905) comb. nov.

Figure 17

Agathis erythrogastra Cameron, 1905.
Bassus brooksi Sharkey, 1998, syn. nov.
Pneumagathis brooksi (Sharkey, 1998), in Chapman and Sharkey (2015).

###### Diagnostics.

BOLD:AAV3035. Consensus barcode. GATTTTATATTTTATTTTTGGAATTTGRTCRGGRATATTRGGTTTATCAATAAGTTTAATTATTCGAATAGAATTAAGAATTACAGGAAATTTTATTGGTAATGATCAAATTTATAATTCAATTGTT-CTGCTCATGCTTTTATTATAATTTTTTTTATAGTAATACCAATTATAATTGGAGGATTYGGAAATTGRTTAGTYCCTTTGATATTAGGAGGRCCYGATATAGCTTTTCCDCGAATAAATAATATAAGATTTTGATTATTAATTCCTTCATTAATATTATTAATATTAAGTTCAATTATTAATATTGGRGTAGGTACTGGATGAACAGTTTATCCTCCTTTATCTTTAAATATAAGTCATAGAGGWATATCAGTTGATTTAGCTATTTTTTCTTTACATATTGCTGGAATTTCTTCTATTATAGGAGCAATAAATTTTATTACMACAATTTTAAAYATATGAATTATTAATATTAAAATTGATAAAATACCTTTATTAGTRTGATCAATTTTTATTACTGCAATTTTATTATTATTATCTTTACCAGTTTTAGCTGGAGCTATTACTATATTATTGACTGAYCGAAATTTAAATACAAGATTWTTTGATCCTTCAGGAGGGGGRGATCCAATTTTATATCAACATTTRTTT------------------------------------------------------------------.

###### Notes.

As reported in Janzen et al. (1998), *P.erythrogastra* (as *Bassusbrooksi*) attacks a wide range of skipper caterpillars on broad leafed woody plants in Costa Rica. Sharkey and Chapman (2015) proposed the genus *Pneumagathis*. *P.brooksi* is a junior synonym of *Agathiserythrogastra* Cameron, 1905, which is transferred here to *Pneumagathis*.

**Figure 17.**
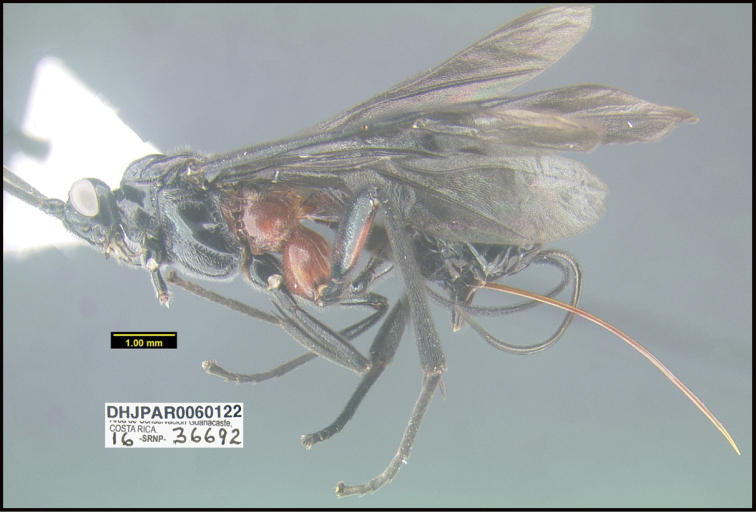
*Pneumagathiserythrogastra*.

#### *Therophilus* Wesmael, 1837

*Therophilus* is a cosmopolitan genus with many hundreds of species (mostly undescribed). It is relatively rare in the neotropics. Published hosts include caterpillars of a wide range of lepidopteran families (Yu et al. 2016). No species have previously been recorded from Costa Rica or neighboring countries.

##### Therophilus penteadodiasae

Taxon classificationAnimaliaHymenopteraBraconidae

Sharkey sp. nov.

http://zoobank.org/A09AAAB1-EB00-43CD-9047-7526F4044AB1

Figure 18

###### Diagnostics.

BOLD:ABZ8830. Consensus barcode. TATTTTATATTTTATTTTTGGGATTTGATCGGGAATTTTAGGGTTATCAATAAGATTATTAATTCGAATAGAGTTAAGTATTGGTGGTAATTTTATTGGTAATGATCAAATTTATAATAGAATTGTTACTGCTCATGCATTTATTATAATTTTTTTTATAGTTATACCAATTATRATTGGTGGTTTTGGTAATTGATTAATTCCTTTAATATTAGGAGGTCCAGATATAGCTTTCCCTCGAATAAATAATATAAGATTTTGATTATTAATTCCTTCATTAACATTATTAATTTTAAGTTCTTTAATTAATATTGGAGTTGGAACTGGATGAACAGTCTATCCTCCTTTATCGTTAAATATAAGACACAGAGGAATATCTGTTGATTTAGCAATTTTTTCTTTACATATTGCTGGTGTTTCTTCAATTATAGGGGCAATAAATTTTATTACAACTATTTTAAATATATGAATTGTAAATATTAAAATTGATAAAATATCTTTATTGGTTTGATCAATTTTAATTACAGCGATTTTATTATTGTTATCTTTACCAGTATTGGCTGGTGCAATTACTATATTATTAACAGATCGAAATTTAAATACAAGATTTTTTGATCCTTCAGGAGGAGGGGATCCAATTTTATATCAGCATTTATTT.

###### Holotype ♀.

Guanacaste, Sector El Hacha, Casa Hacha Vieja, 10.98050, -85.54429, 290 meters, 290 meters, caterpillar collection date: 24/ii/2014, wasp eclosion date: 17/iii/2014. Depository: CNC.

***Host data*.** Parasitoid of *Antaeotricha* Janzen146 (Depressariidae) feeding on *Lonchocarpusoliganthus* (Fabaceae).

***Caterpillar and holotype voucher codes*.** 14-SRNP-55508, DHJPAR0055292.

###### Paratypes.

Hosts = *Antaeotricha* Janzen146 and *Antaeotricha* Janzen366 (Depressariidae). DHJPAR0055209, DHJPAR0055544.

###### Etymology.

The species is named in honor of Angelica Penteado-Dias, eminent Brazilian braconidologist.

###### Note.

The wing venation is very similar to that of *Plesiocoelus*, but the characters given in the key (above), including the shape of the subbasal cell of the hind wing, clearly place this species in *Therophilus*.

**Figure 18.**
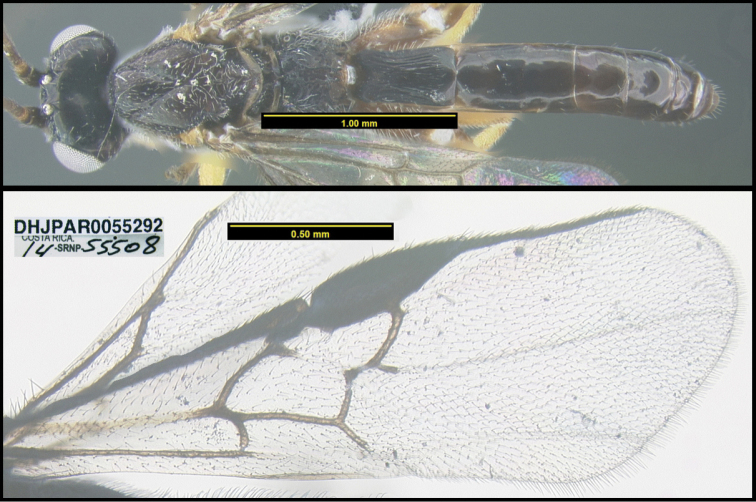
*Therophiluspenteadodiasae*, holotype.

##### Therophilus bobwhartoni

Taxon classificationAnimaliaHymenopteraBraconidae

Sharkey sp. nov.

http://zoobank.org/AD112449-21E0-4A12-9939-74120B7D3CE7

Figure 19

###### Diagnostics.

BOLD:AAY4688. Consensus barcode. AATTTTATATTTTATTTTTGGAATTTGGTCGGGTATTTTGGGATTATCGATAAGTTTATTAATTCGGATGGAATTAAGAGTAGGGGGTAATTTTATTGGAAATGATCAAATTTATAATAGAATTGTTACTGCTCATGCATTTATTATAATTTTTTTTATGGTTATACCAATTATAATTGGGGGATTTGGTAATTGATTAATTCCTTTAATGTTAGGGGGTCCTGATATAGCTTTCCCTCGAATAAATAATATAAGATTTTGATTATTAATTCCCTCATTAATATTATTAATTTTGAGGTCATTAATTAATATTGGAGTTGGGACTGGRTGAACAGTTTACCCTCCTTTATCATTAAATATAAGTCATAGGGGGATATCTGTTGATTTAGCGATTTTTTCTTTGCATATGGCAGGAGTTTCTTCAATTATGGGGGCAATAAATTTTATTACTACAATTTTAAATATATGAATTATAAATATTAAAATTGATAAGATACCTTTATTAGTTTGATCGATTTTAATTACGGCAATTTTGTTATTATTATCATTACCTGTGTTAGCTGGAGCTATTACTATATTATTAACTGACCGAAATTTAAATACAAGATTTTTTGATCCTTCAGGTGGAGGGGATCCAATTTTATATCAACATTTATTT.

###### Holotype ♀.

Guanacaste, Sector Cacao, Sendero Nayo, 10.92446, -85.46953, 1090 meters, caterpillar collection date: 05/ii/2007, wasp eclosion date: 18/iii/2007. Depository: CNC.

***Host data*.** Parasitoid of elachJanzen01 Janzen161 (Depressariidae) feeding on *Viburnumcostaricanum*.

***Caterpillar and holotype voucher codes*.** 07-SRNP-35262, DHJPAR0062169.

###### Paratype.

Host = elachJanzen01 Janzen161: DHJPAR0041864.

###### Etymology.

This species is named in honor of Bob Wharton, eminent braconidologist.

**Figure 19.**
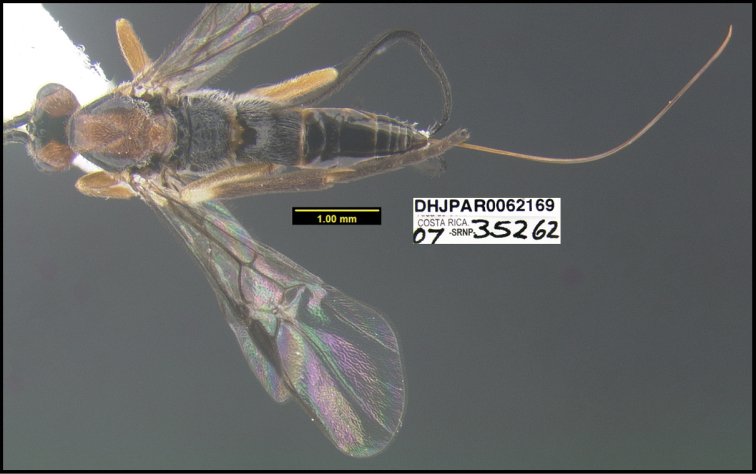
*Therophilusbobwhartoni*, holotype.

##### Therophilus donaldquickei

Taxon classificationAnimaliaHymenopteraBraconidae

Sharkey sp. nov.

http://zoobank.org/ED5FA250-54E1-4F9D-9649-38A28740D637

Figure 20

###### Diagnostics.

BOLD:ADM6790. Consensus barcode. GGGAATGATCAAGTTTATAATAGAATTGTTACTGCGCATGCATTTATTATAATTTTTTTTATGGTGATACCAATTATAATTGGTGGGTTTGGAAATTGATTAGTTCCTTTAATGTTGGGAGGACCTGATATGGCTTTCCCACGAATAAATAATATAAGTTTTTGGTTATTAATTCCATCATTAATATTATTAATTTTGAGTTCTTTAATTAATATTGGGGTTGGAACAGGGTGGACAGTTTATCCTCCATTGTCATTAAATATAAGTCATAGAGGTATATCAGTTGATTTAGCTATTTTTTCTTTACATATAGCCGGTGTTTCTTCAATTATAGGAGCTATAAATTTTATTACTACAATTTTAAATATATGAATAATAACAATTAAAATTGATAAAATACCATTGTTAGTTTGATCTATTTTAATTACAGCAATTTTATTATTGTTATCTTTACCTGTGTTGGCGGGGGCTATTACTATATTATTAACAGATCGAAATTTAAATACAAGATTTTTTGATCCATCAGGTGGTGGTGACCCAATTTTATATCAACATTTATTTTGATTTTTTGGTCATCCTGAAGTTTATATTTTAATTTTACCGGGATTTGGGATTATTTCTCATATT.

###### Holotype ♀.

Heredia, 16 km SSE La Virgen, 1050‒1150 meters, 21/iii/2001, Malaise trap. Depository: CNC.

***Host data*.** None.

***Holotype voucher code*.**H155.

###### Paratypes.

None.

###### Etymology.

The specific epithet is a patronym for Donald Quicke, eminent braconidologist.

**Figure 20.**
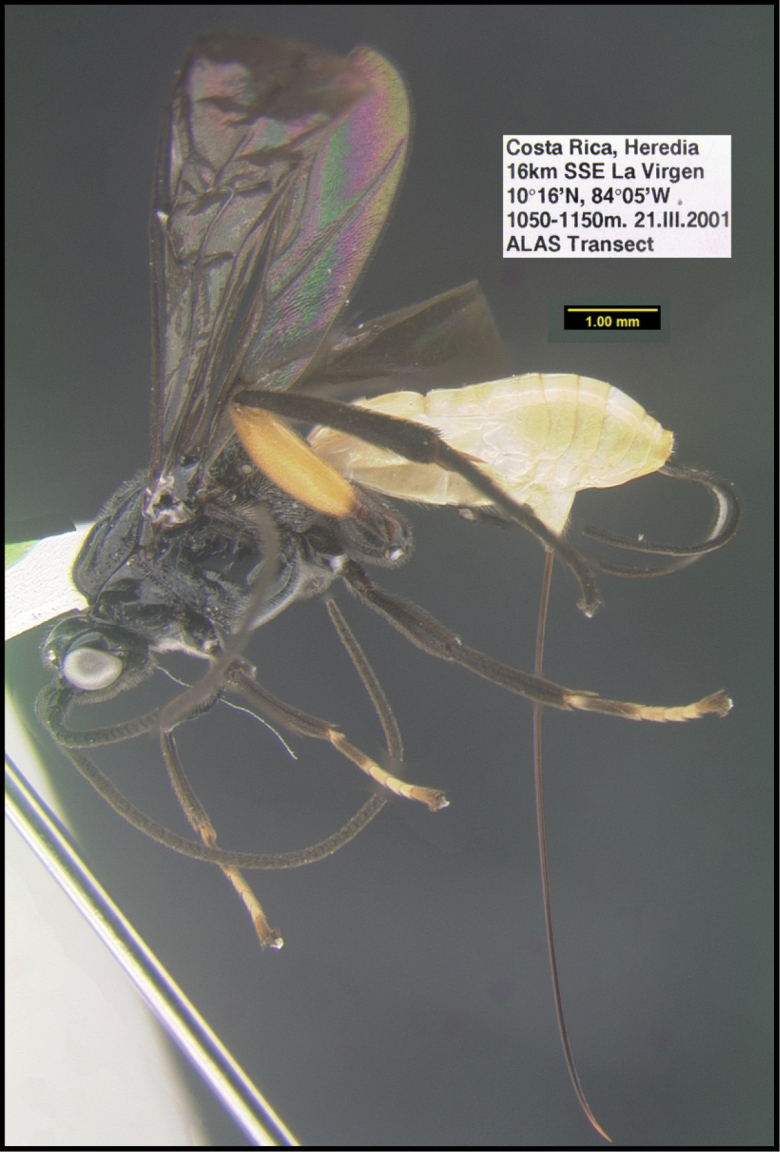
*Therophilusdonaldquickei*, holotype.

##### Therophilus gracewoodae

Taxon classificationAnimaliaHymenopteraBraconidae

Sharkey sp. nov.

http://zoobank.org/34026ED0-F052-4C43-9074-074A42A19563

Figure 21

###### Diagnostics.

BOLD:ADD0457. Consensus barcode. TATTTTATATTTTATTTTTGGAATTTGGTCGGGTATTTTGGGATTATCAATAAGTTTATTAATTCGTATAGAATTAAGAATTGGGGGTAACTTAATTGGTAACGATCAAATTTATAATAGAATTGTTACTGCTCATGCATTTATTATAATTTTTTTTATAGTTATACCAATTATAATTGGAGGTTTTGGTAATTGATTGATTCCTTTAATATTGGGGGGGCCTGATATAGCTTTCCCCCGGATAAATAATATAAGTTTTTGATTATTAATTCCTTCATTAATATTATTAATTTTGAGTTCATTAATTAATATTGGAGTTGGAACAGGATGAACAGTTTATCCTCCTTTATCATTAAATATAAGTCATAGAGGTATATCAGTTGATTTAGCTATTTTTTCTTTACATATAGCTGGTATTTCATCAATTATGGGGGCAATAAATTTTATTACTACAATTTTTAATATATGAATTATAATAATTAAAATTGATAAAATACCATTATTAGTTTGATCTATTTTAATTACAGCAATTTTATTATTATTATCATTACCTGTATTGGCTGGGGCTATTACAATATTATTAACAGATCGAAATTTAAATACAAGATTTTTTGATCCTTCAGGGGGGGGGGA.

###### Holotype ♀.

Guanacaste, Sector Cacao, Sendero Arenales, 10.92471, -85.46738, 1,080 meters, caterpillar collection date: 09/iv/2010, wasp eclosion date: 10/v/2010. Depository: CNC.

***Host data*.** Parasitoid of *Rhodoneuraterminalis* (Thyrididae) feeding on *Hampeaappendiculata* (Malvaceae)

***Caterpillar and holotype voucher codes*.** 10-SRNP-35134, DHJPAR0039519.

###### Paratypes.

None.

###### Etymology.

The species name is a patronym for Grace Wood, renowned entomologist.

**Figure 21.**
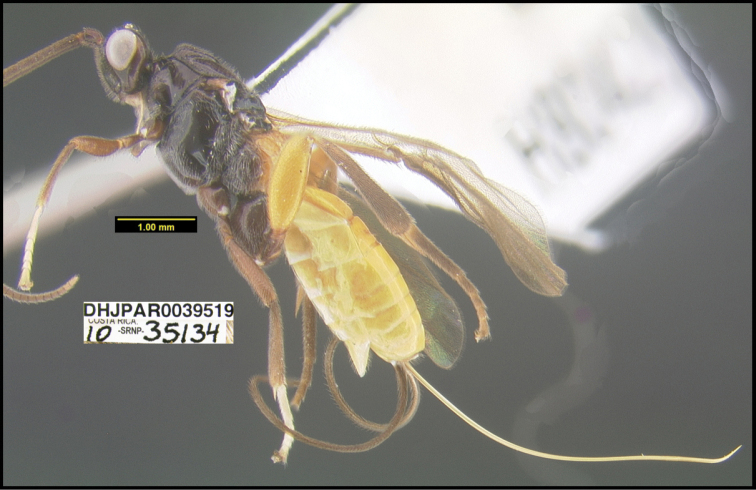
*Therophilusgracewoodae*, holotype.

##### Therophilus maetoi

Taxon classificationAnimaliaHymenopteraBraconidae

Sharkey sp. nov.

http://zoobank.org/375C801E-E40C-407F-84C4-61A0FBDC3702

Figure 22

###### Diagnostics.

BOLD:AAU5369. Consensus barcode. ATTTTTATATTTTATTTTTGGAATTTGATCAGGAATTTTAGGATTATCAATAAGTTTATTAGTTCGAATGGAATTAAGAATTRGTGGTAATTTTATTGGKAATGATCAAATTTATAATAGTATTGTAACTGCACATGCATTTATTATAATTTTTTTTATAGTTATGCCAATYATAATTGGAGGRTTTGGTAATTGGTTAGTACCTTTAATATTAGGAGGTCCTGATATAGCTTTCCCTCGAATAAATAATATAAGGTTTTGATTATTAATYCCTTCATTAATATTATTAATTTTRAGATCATTAATTAATATTGGAGTTGGWACWGGTTGAACAGTATAYCCRCCRTTATCWTTAAATATAAGACATAGGGGTATATCTGTTGATTTRGCTATTTTTTCTTTACATATTGCTGGTATTTCATCTATTATAGGGGCAATAAATTTTATTACAACTATTTTAAATATATGAATTATTAATATTAAAATTGATAAAATRCCTTTATTAGTTTGATCAATTTTAATTACTGCAATTTTATTATTATTATCATTACCTGTTTTAGCTGGAGCTATTACTATATTATTAACAGATCGAAATTTAAATACAAGATTTTTTGATCCTTCAGGAGGTGGTGATCCAATTTTATATCAACATTTATTT.

###### Holotype ♀.

Heredia, 6 km ENE Vara Blanca, 10.183, -84.12, 2000 meters, 21/ii/2002, Malaise trap. Depository: CNC.

***Host data*.** None.

***Holotype voucher code*.**H1168.

###### Paratypes.

H1148, H1150, H1168.

###### Etymology.

The species is named in honor of Kaoru Maetô, renowned Japanese braconidologist.

**Figure 22.**
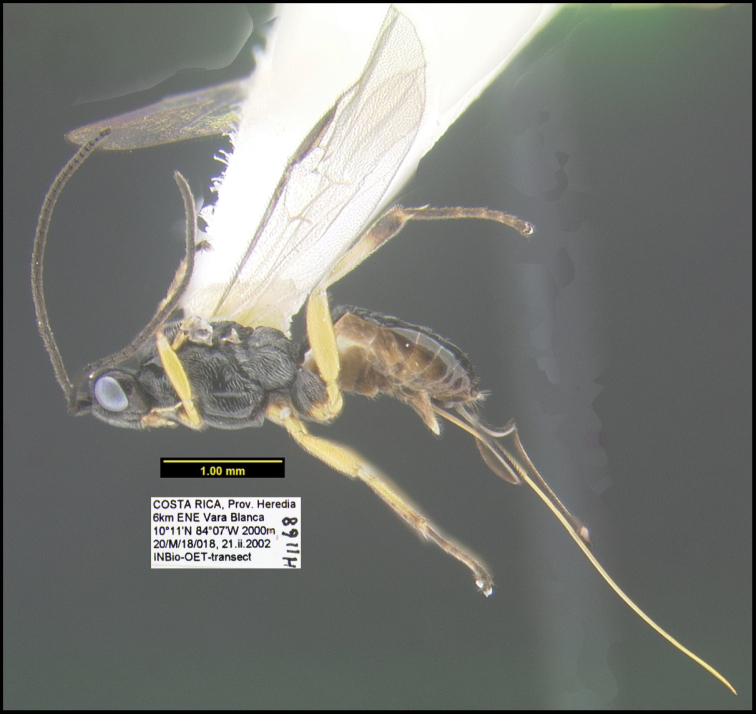
*Therophilusmaetoi*, holotype.

##### Therophilus montywoodi

Taxon classificationAnimaliaHymenopteraBraconidae

Sharkey sp. nov.

http://zoobank.org/CF7708A9-E549-4440-9FDA-4795CD35EA49

Figure 23

###### Diagnostics.

BOLD:ADD0519. Consensus barcode. AATTTTATATTTTATTTTTGGGGTTTGATCAGGAATTGTAGGTTTATCAATAAGATTATTAATTCGAATGGAATTAAGAATTGGTGGTAATTTTATTGGTAATGATCAAATTTATAATAGAATTGTTACAGCTCATGCATTTGTAATAATTTTTTTTATAGTTATACCAATTATGATTGGGGGTTTTGGAAATTGATTAGTTCCTTTAATATTGGGGGGYCCAGATATAGCTTTCCCTCGAATAAATAATATAAGATTCTGGTTATTAATTCCTTCATTAATATTATTAATTTTAAGTTCATTAGTTAATATTGGAGTTGGAACAGGGTGAACAGTTTATCCTCCTTTATCTTTAAATATAAGTCATAGAGGTATATCGGTTGATTTAGCTATTTTTTCTTTACATATTGCAGGGGTTTCATCAATTATAGGGGCTATAAATTTTATTACTACAATTTTAAATATATGAATTATGAATATTAAAATTGATAAAATACCTTTATTAGTGTGATCAATTTTAATTACAGCAATTTTATTATTATTATCATTACCAGTGTTAGCAGGGGCAATTACAATATTATTAACAGATCGTAATTTGAATACAAGATTTTTTGATCCTTCTGGTGGGGGGGATCCAATTTTATATCAACATTTATTT.

###### Holotype ♀.

Alajuela, Sector San Cristobal, Finca San Gabriel, 10.87766, -85.39343, 645 meters, caterpillar collection date: 31/vii/2009, wasp eclosion date: 26/viii/2009. Depository: CNC.

***Host data*.** Parasitoid of *Strepsicrates* Brown25 (Tortricidae) feeding on *Psidiumguajava* (introduced) (Myrtaceae).

***Caterpillar and holotype voucher codes*.** 09-SRNP-4023, DHJPAR0040068.

###### Paratypes.

Host = *Strepsicrates* Brown25: DHJPAR0038338. Depository: CNC.

###### Etymology.

The specific epithet is a patronym for Monty Wood (RIP), renowned tachinidologist.

**Figure 23.**
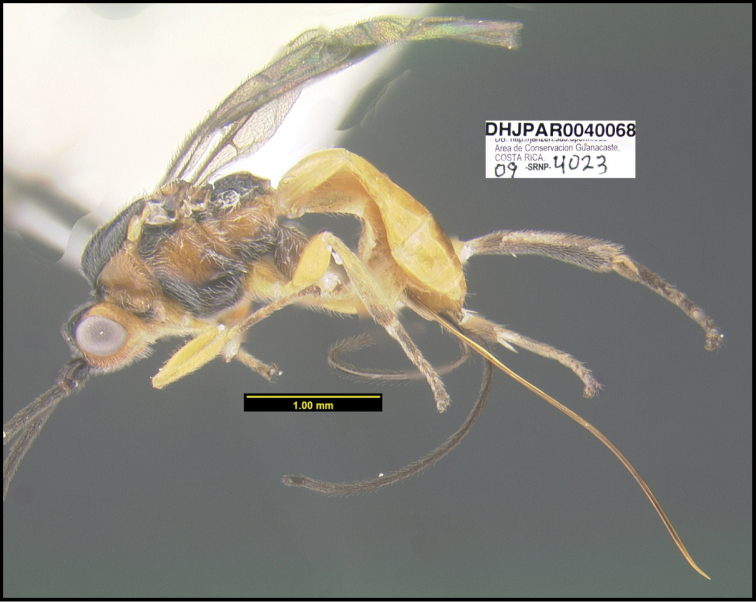
*Therophilusmontywoodi*, holotype.

#### *Zacremnops* Sharkey & Wharton, 1985

Members are widespread in the neotropics. Sharkey (1990) revised the species, and one new species is reported here. There are no previously reported hosts, but here we document four species of *Zacremnops* on crambid caterpillars (Crambidae).

##### Zacremnops brianbrowni

Taxon classificationAnimaliaHymenopteraBraconidae

Sharkey sp. nov.

http://zoobank.org/89E3C0E2-C499-4613-92B8-174FADC853AD

Figure 24

###### Diagnosis.

The COI barcode has only 293 bases and therefore was not given a BIN. This species can be distinguished from all other species of *Zacremnops* by red coloration restricted to the metapleuron (i.e., not on the propodeum) in combination with the melanic hind tarsus.

###### Holotype ♂.

Alajuela, Sector Rincon Rain Forest, Sendero Juntas, 10.90661, -85.28784, 400 meters, caterpillar collection date: 11/viii/2010, wasp eclosion date: 3/ix/2010. Depository: CNC.

***Host data*.** Parasitoid of *Microthyrisprolongalis* (Crambidae) feeding on *Ipomoeabatatas* (Convolvulaceae).

***Caterpillar and holotype voucher codes*.** 10-SRNP-42859, DHJPAR0041189.

###### Paratypes.

None.

###### Etymology.

The species is named in honor of Brian Brown, eminent dipterist (phoridologist).

**Figure 24.**
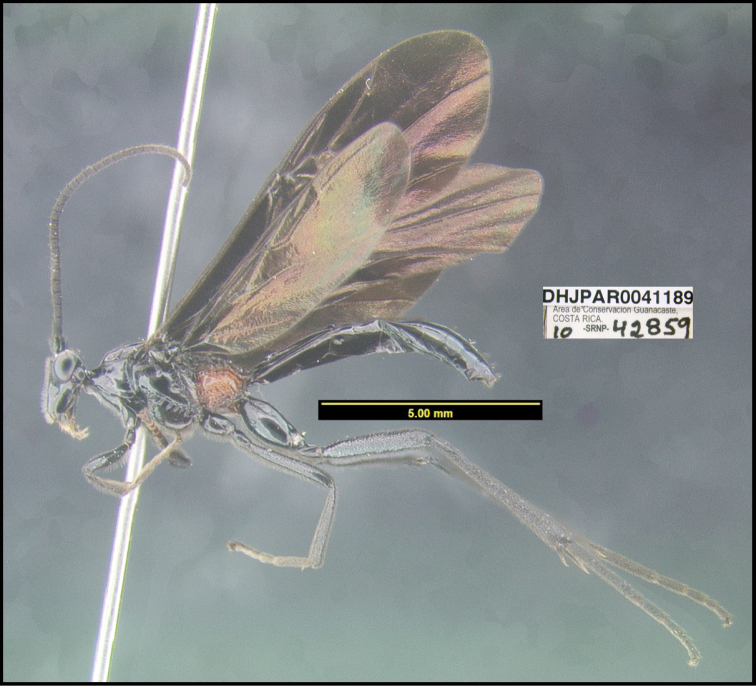
*Zacremnopsbrianbrowni*, holotype.

##### Zacremnops coatlicue

Taxon classificationAnimaliaHymenopteraBraconidae

Sharkey, 1990

Figure 25

###### Diagnostics.

No barcode available. This species can be distinguished from all other species of *Zacremnops* by restriction of the red coloration to the metapleuron and propodeum, in combination with the melanic hind tarsus

***Host data*.** All parasitoids of *Sylleptebelialis* (Crambidae).

###### Material examined.

Several dozen specimens reared from caterpillars collected in lowland Pacific forest, e.g., Guanacaste, Sector Santa Rosa, Sendero Natural, 10.836, -85.613, 290 meters, caterpillar collection date: 11/vi/2001, wasp eclosion date: 11/iv/2002, DHJPAR0015422.

**Figure 25.**
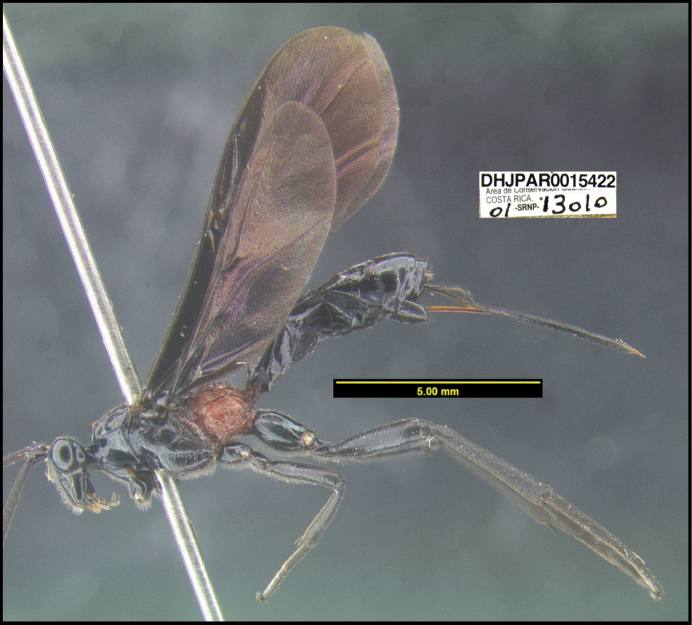
*Zacremnopscoatlicue*.

##### Zacremnops cressoni

Taxon classificationAnimaliaHymenopteraBraconidae

(Cameron, 1887)

Figure 26

###### Diagnosis.

BOLD: AAV3186. *Z.cressoni* is the only known species of *Zacremnops* that has yellow hind tarsi. What appears to be this species is widespread from southern USA to northern Colombia and Venezuela, including the Caribbean.

***Host data***. The sole reared specimen parasitized *Microthyrisprolongalis* (Crambidae) feeding on *Ipomoeabatatas* (Convolvulaceae).

###### Material examined.

Alajuela, Sector Rincon Rain Forest, Palomo, 10.96187, -85.28045, 96 meters, caterpillar collection date: 03/vi/2009, wasp eclosion date: 20/vi/2009, 09-SRNP-67297, DHJPAR0035228.

**Figure 26.**
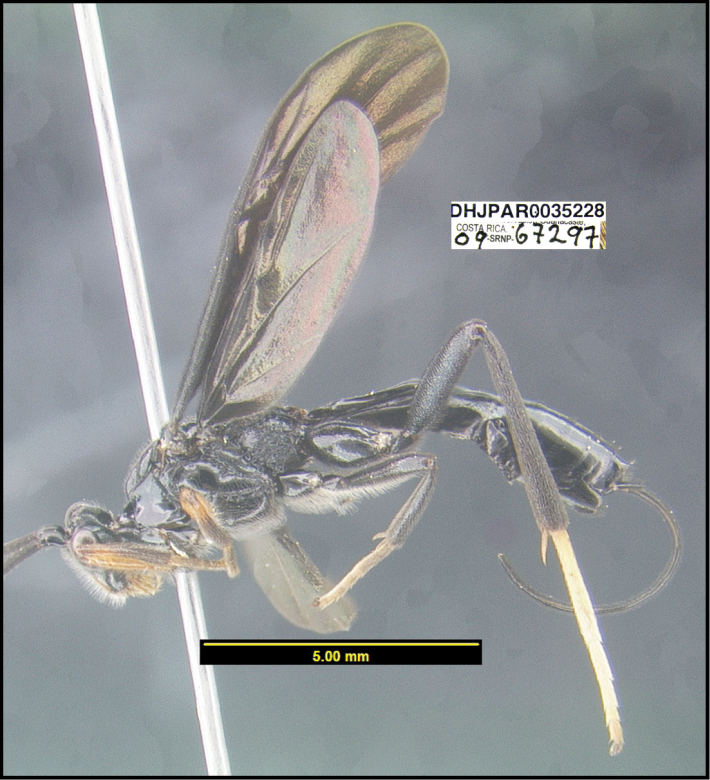
*Zacremnopscressoni*.

##### Zacremnops ekchuah

Taxon classificationAnimaliaHymenopteraBraconidae

Sharkey, 1990

Figure 27

###### Diagnosis.

There are no COI barcodes available for any of the six reared specimens; however, *Z.ekchuah* is the only species of *Zacremnops* that is entirely black. What appears to be this species is widespread in Mexico with southern records in Costa Rican Caribbean mid-elevation rain forest.

***Host data*.** Specimens were reared from spilomeline cambids, i.e., *Microthyrisprolongalis*, *Microthyris* prolongalisDHJ02, *Pantographasuffusalis*, and *Syllepte* amandoDHJ02 (Crambidae).

###### Material examined.

DHJPAR0057410, DHJPAR0052687, DHJPAR0015404, DHJPAR0015405, DHJPAR0015403, and DHJPAR0021168.

**Figure 27.**
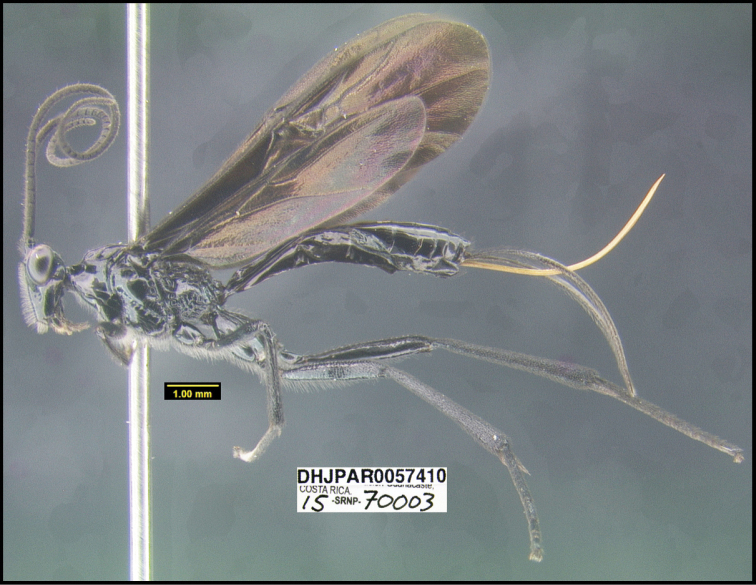
*Zacremnopsekchuah*.

#### *Zamicrodus* Viereck, 1912

Members of this genus are distributed widely across the neotropics. The two described species are from South America. The first host record (Hesperiidae) for the genus is reported here. The genus has not been revised, though there are likely to be fewer than ten species.

##### Zamicrodus josefernandezi

Taxon classificationAnimaliaHymenopteraBraconidae

Sharkey sp. nov.

http://zoobank.org/CD23D014-93CD-4BE2-8B71-9CBFF260713C

Figure 28

###### Diagnostics.

BOLD:ACC5208. Consensus barcode. AATTTTATATTTTATTTTTGGTGTTTGATCAGGAATTTTAGGTTTATCTATAAGAATAATTATTCGAATAGAATTAAGAATTACAAGTAATTTTATTGGAAATGATCAAATTTATAATTCTATTGTCCTGCTCATGCTTTTATTATAATTTTTTTTATAGTAATACCTATTATAATTGGTGGATTTGGAAATTGATTAGTACCATTAATATTAGGAGGACCTGATATAGCTTTTCCTCGAATAAATAATATAAGATTTTGATTATTAATTCCTTCATTATTATTATTAATTATAAGATCTATTATTAATATTGGAGTAGGACTGGATGAACAGTTTATCCTCCTTTATCATTAAATTTAAGTCATAGAGGTATTTCAGTAGATTTAGCTATTTTTTCTTTACATATTGCAGGAATTTCTTCTATTATAGGAGCTATAAATTTTATTACAACAATTTTAAATATATGAATTATTAATATTAAAATTGATAAAATACCCTTATTAGTTTGATCAATTTTTATTACAGCAATTTTATTATTATTATCATTACCAGTTTTAGCTGGAGCTATTACTATATTATTACTGATCGAAATTTAAATACAAGATTTTTTGATCCTTCTGGAGGAGGAGACCCAATTTTATATCAACATTTATTT. The two described species differ from *Z.josefernandezi* in that their mesosomata are partly to completely pale yellow-orange, whereas that of *Z.josefernandezi* is completely black.

###### Holotype ♀.

Guanacaste, Sector Pitilla, Medrano, 11.01602, -85.38053, 380 meters, caterpillar collection date: 9/ii/2012, wasp eclosion date: 8/iii/2012. Depository: CNC.

***Host data*.** Parasitoid of *Quasimellanasethos* (Hesperiidae) feeding on *Rhipidocladumracemiflorum* (Poaceae).

***Caterpillar and holotype voucher codes*.** 12-SRNP-70305, DHJPAR0048733.

09-SRNP-4023, DHJPAR0048733.

###### Paratypes.

Host, *Quasimellanasethos*: DHJPAR0057459, DHJPAR0048728.

###### Etymology.

The specific epithet is a patronym for José Fernandez-Triana, renowned Cuban-Canadian braconidologist.

**Figure 28.**
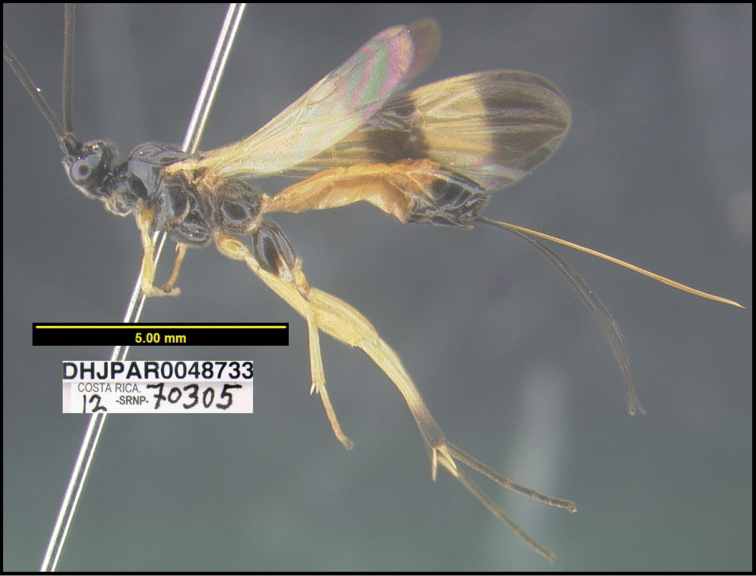
*Zamicrodusjosefernandezi*, holotype.

#### *Zelomorpha* Ashmead, 1900

The Costa Rican species of *Zelomorpha* were revised by Meierotto et al. (2019a) employing barcodes as diagnostic characters. The sole gregarious species of Agathidinae, *Zelomorphagregaria* (Sarmiento & Sharkey, 2004) (originally named *Coccygidiumgregarium*), was inadvertently omitted from that publication. The inclusion of *Z.sarahmeierottoae* brings the total number of Costa Rican species to 17. Hosts include caterpillars in the following families: Erebidae, Euteliidae, Geometridae, Nolidae, Notodontidae, and Noctuidae.

##### Zelomorpha sarahmeierottoae

Taxon classificationAnimaliaHymenopteraBraconidae

Sharkey sp. nov.

http://zoobank.org/46CCD21B-9315-441A-9091-CA75C41AB768

Figure 29

###### Diagnostics.

BOLD:ACG5511. Consensus barcode. TGTTTTATATTTTTTATTTGGTATATGAAGGGGAATTTTAGGATTAAGATTGAGTTTATTGGTTCGATTTGAATTAGGATTAAGAGGAAATTTAATTGGAAGTGATCAAATTTATAATAGGATAGTACTTATCATGCATTAATTATGATTTTTTTTATAGTTATACCTATTATGATTGGAGGATTTGGTAATTGGTTGGTTCCTTTATTGTTAGGAAGACCTGATATAGCTTTCCCTCGAATAAATAATATAAGATTTTGATTATTATTACCTTCATTAATATTATTAATTTTAAGTTCTTTTGTTAATATTGGAGCAGGTACAGGTTGGACTATTTATCCTCCATTATCATTAAATTTTAGACATAGGGGTATGTCGGTTGATTTAATAATTTTTGCTTTGCATATTGCTGGTGTTTCATCAATTATAGGTGCAATTAATTTTATTACTACTATTTTAAATATATGAATAATAAATATTAATATAGATAAAATATCTTTATTTGTTTGATCTATTTTATTAACAGCTATTTTATTATTATTATCTTTGCCAGTTTTAGCTGGAGCTATTACTATATTATTAAGAGATCGAAATTTAAATTCAAGATTTTTTGATCCTACTGGGGGAGGGGATCCTATTTTATATCAACATTTATTT.

###### Holotype ♀.

Guanacaste, Sector Santa Rosa, Bosque San Emilio, 10.8438, -85.6138, Malaise trap, 04/vii/2012. Depository: CNC.

***Host data*.** None.

***Holotype voucher code*.**BIOUG05883-E01.

###### Paratypes.

None.

###### Etymology.

The species is named in honor of Sarah Meierotto, former graduate student of MJS.

**Figure 29.**
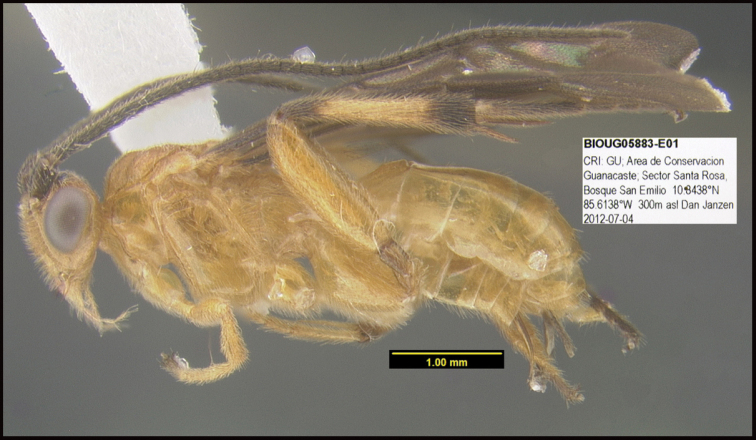
*Zelomorphasarahmeierottoae*, holotype.

## Chapter 3: Braconinae

Braconines are primarily idiobiont parasitoids of Coleoptera and Lepidoptera, but many other insect orders have been used. Below we treat only Costa Rican species. The ten reared species described below are all gregarious parasitoids of caterpillars. Hosts of the Malaise-trapped species could be larval Lepidoptera or the larvae of a number of other insect orders, the most likely being Coleoptera. The key by Quicke (1997) is somewhat dated, and a new key to the New World genera of Braconinae is presented below (constructed by Sharkey, Quicke, and van Achterberg). For the Braconinae NJ tree, see Suppl. material 2.

### Key to the New World genera of Braconinae

The shape of the antennal scape is important for identification of genera. For determination of the relative lengths of the dorsal and ventral sides of the scape, as in couplets 3‒5, 11, 12, and 16, the antennae should be positioned as those of couplet 3, i.e., horizontally.

**Table:** 

1	A. Face with a median projection that is approximately round in cross section; Neotropical, rare	*** Lasiophorus ***
–	B. Face with large, horizontal, dorsally concave plate-like projection; Neotropical, rare	**2**
–	C. Face without a projection; widespread, common	**3**
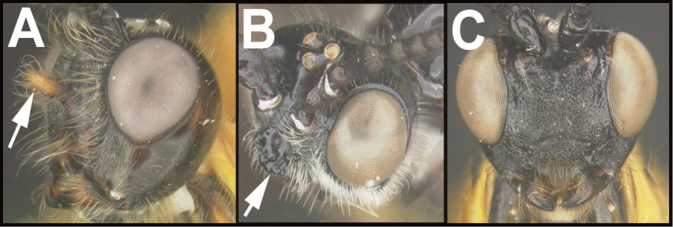		
2(1)	A. First flagellomere with thorn-like projection	*** Cervellus ***
–	B. First flagellomere simple or occasionally slightly flared apically	*** Palabracon ***
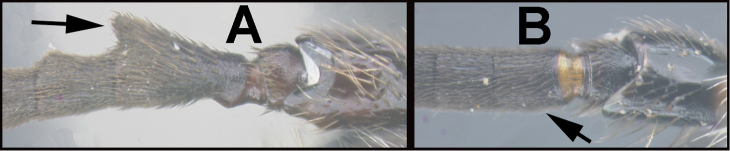		
3(1)	A. Basal flagellomere (1) expanded ventrally	**4**
–	B. Basal flagellomere (1) simple, not expanded ventrally	**5**
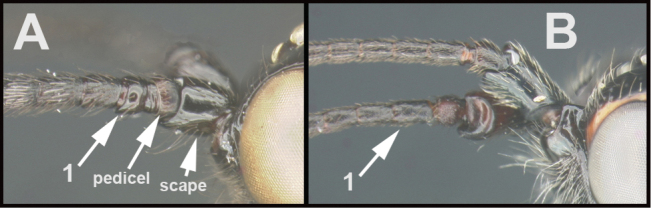		
4 (3)	A. Scape shorter ventrally than dorsally (antenna directed anteriorly); flagellum uniformly dark, without white or cream-colored band at midlength; widespread	*** Coeloides ***
–	B. Scape longer ventrally than dorsally (antenna directed anteriorly); flagellum with white or cream-colored band at midlength; Neotropical	*** Megacoeloides ***
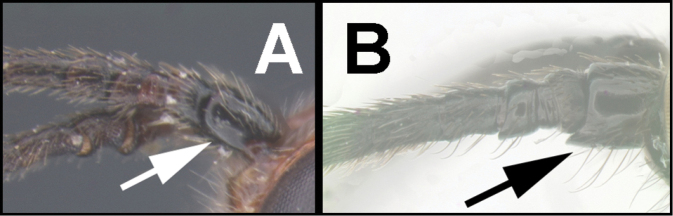		
5(3)	A. Scape with false sub-apical margin, well-separated from the real margin, and scape basally abruptly and concavely narrowing	**6**
–	B. Scape without false sub-apical margin, and scape basally gradually narrowing towards base.	**10**
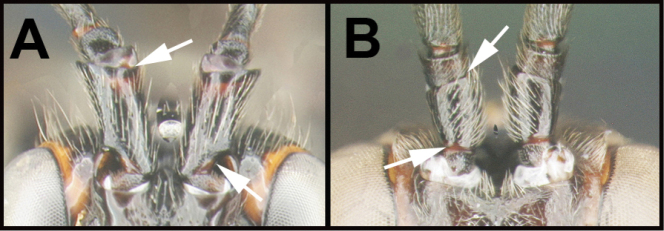		
6(5)	A. Antennal sockets greatly protruding in profile; scape as wide as long; temple wider than eye; Neotropical, rare.	*** Calobracon ***
–	B. Antennal sockets slightly protruding in profile; scape longer than wide; temple shorter than eye.	**7**
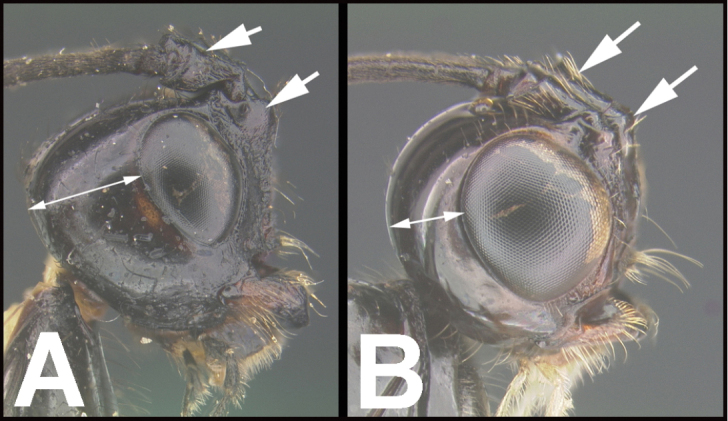		
7(6)	A. Tergum 2 with triangular or (rarely) quadrate raised area narrowed posteriorly; widespread	**8**
–	B Tergum 2 smooth and flat or with a smooth posteriorly widening “pinched-up” area; Neotropical	**9**
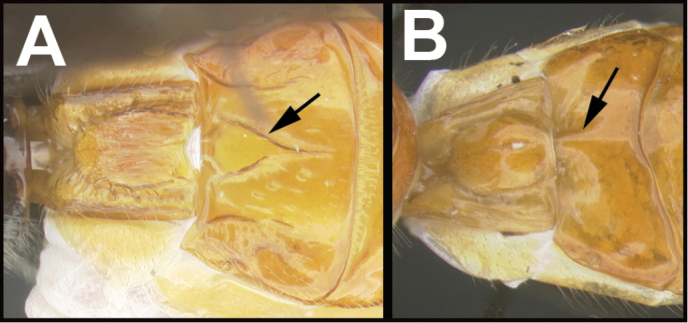		
8(7)	A. Raised median area of tergum 1 with semi-circular emargination postero-laterally; Neotropical	*** Hemibracon ***
–	B. Raised median area of petiole without abrupt emargination postero-laterally; widespread.	*** Atanycolus ***
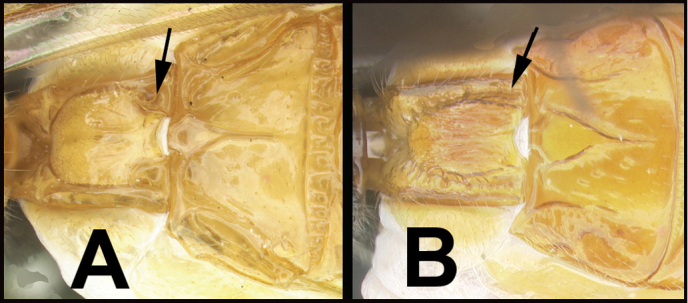		
9(7)	A. Metasomal tergum 2 strongly ‘pinched-up’ antero-medially	*** Gracilibracon ***
–	B. Metasomal tergum 1 not or hardly ‘pinched-up’ antero-medially	***Cyclaulax* (in part)**
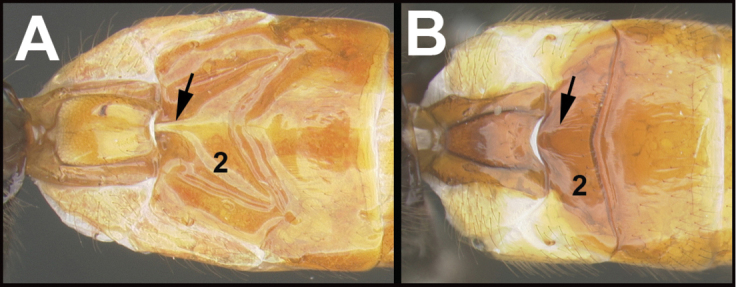		
10(5)	A. Middle part of face clearly demarked from lateral parts by a pair of sub-medial, longitudinal carinae or rugose grooves running ventrally from the antennal sockets. AA. Metasomal tergum 2 without medial, basal, posteriorly-narrowing triangular area.	**11**
–	B. Middle part of face not clearly demarked from lateral parts by longitudinal grooves or carinae. BB. Metasomal tergum 2 often with medial, basal, posteriorly-narrowing triangular area	**16**
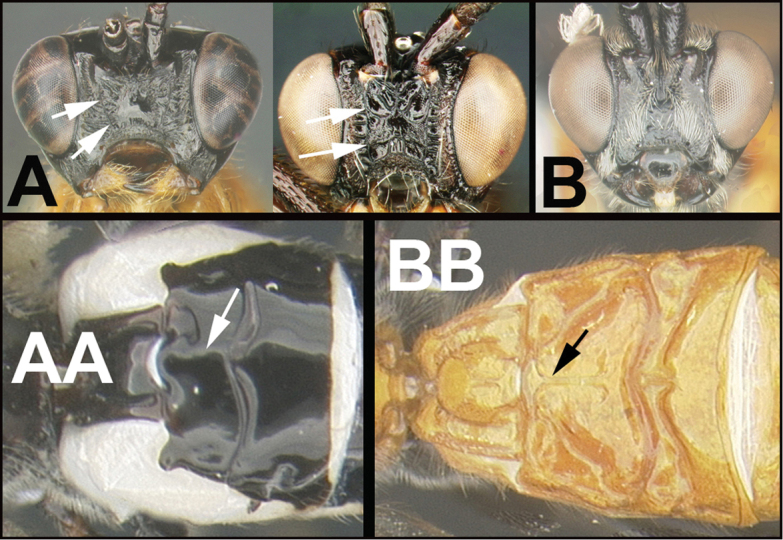		
11(10)	A. Hypopygium truncated in lateral aspect	*** Compsobracon ***
–	B. Hypopygium acutely pointed in lateral aspect	**12**
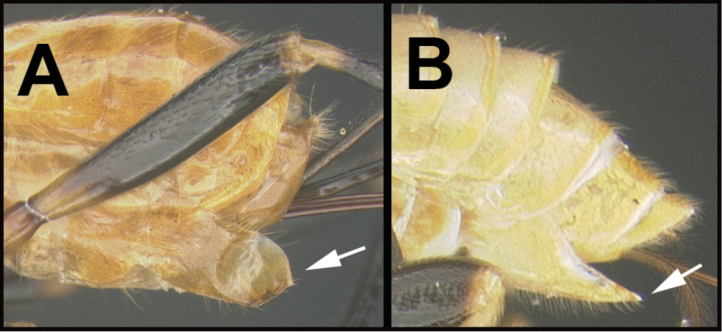		
12(11)	A. Scape shorter ventrally (V) than dorsally (D) (antenna directed anteriorly)	**13**
–	B. Scape longer ventrally (V) than dorsally (D) (antenna directed anteriorly)	**14**
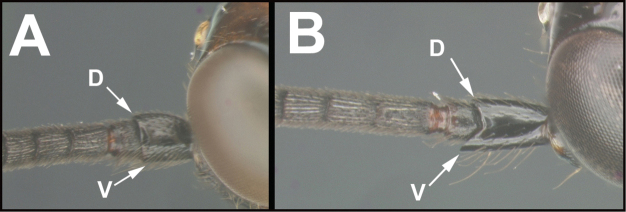		
13(12)	A. Propodeum simple, entirely smooth; second metasomal tergite flat or weakly pinched anteromedially	*** Compsobraconoides ***
–	B. Propodeum with a distinct longitudinal carina posteromedially; second metasomal tergite more pinched-up medio-anteriorly	*** Gozmanycomp ***
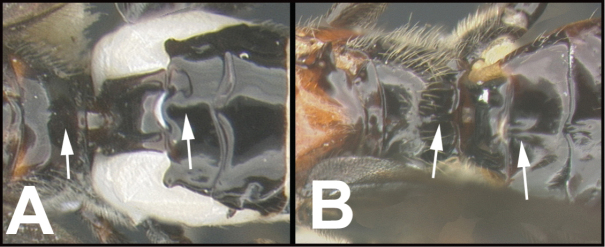		
14(12)	A. Length of tergum 2 more than ½ as long as tergum 3	**15**
–	B. Length of tergum 2 less than ½ length of tergum 3	***Cyclaulax* (in part)**
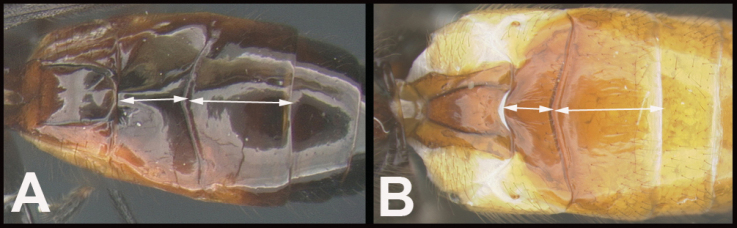		
15(14)	A. Face rugose with a lot of granulate sculpture, often with a raised granulate area medially. AA. Raised median area of petiole rather square in cross-section, lateral margin more or less carinate posteriorly	*** Cyclaulacidea ***
–	B. Face coarsely carinate-rugose medially with little or no granulate sculpture and without a raised granulate area medially. BB. Raised median area of petiole more rounded in cross-section	*** Sacirema ***
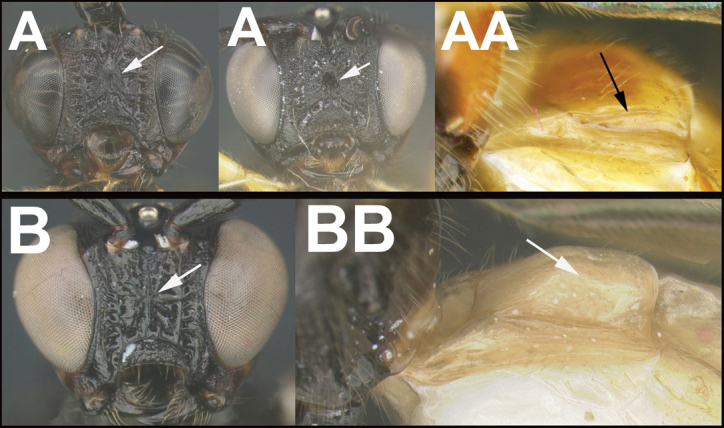		
16(10)	A. Scape not, or only weakly, emarginate apicolaterally; scape shorter ventrally than dorsally (antenna directed anteriorly).	**17**
–	B. Scape apicolaterally emarginate; scape longer ventrally than dorsally (antenna directed anteriorly)	**24**
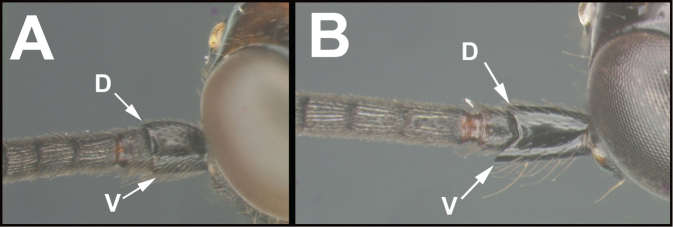		
17(16)	A. Marginal cell of forewing short, vein 3RS reaching wing margin at most 0.7 × distance between apex of stigma and wing tip. AA. Clypeus with pair of long setae arranged in a cluster and often touching apically and/or AAA. Metasomal tergum 4 with characteristic pattern of fine striae that curve away from the midline; widespread.	*** Vipio ***
–	B. Marginal cell of forewing usually longer, vein 3RS reaching wing margin at least 0.8 × distance between apex of stigma and wing tip; if shorter, then BB. Clypeus without pair of long setae arranged in two clusters (clypeal guard setae evenly spaced and not touching apically. BBB. Metasomal tergum 4 without fine striae that curve away from the midline	**18**
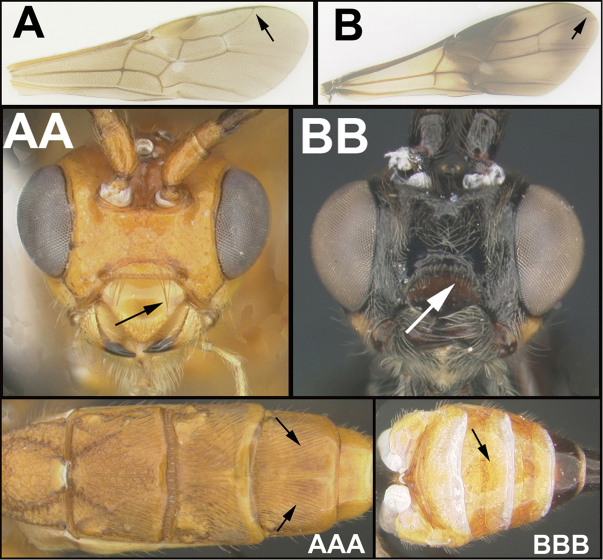		
18(17)	A. Hind tibia strongly compressed laterally, very broad in lateral aspect, with conspicuously long setae. AA. Metasomal tergum 2 with a mid-longitudinal ridge; Neotropical	*** Myosomatoides ***
–	B. Hind tibia usually not strongly compressed laterally, usually without conspicuously long marginal setae. BB. If rather strongly compressed with long marginal setae (most *Myosoma* species) then metasomal tergum 2 lacking mid-longitudinal ridge	**19**
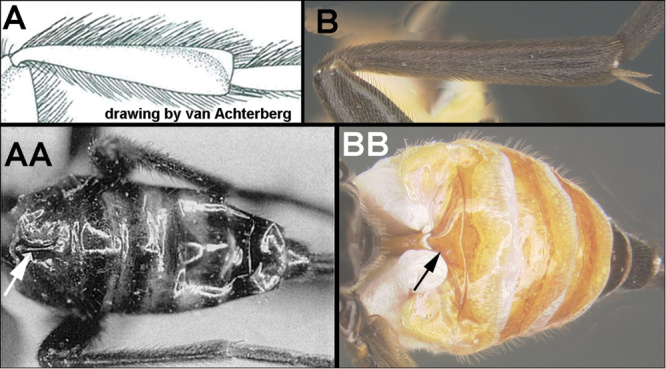		
19(18)	A. Body very strongly depressed dorso-ventrally, mesosoma more than 2.8 × longer than maximally high; Nearctic, rare	*** Chartobracon ***
–	B. Body not or only moderately depressed dorso-ventrally, mesosoma less than 2.3 × longer than maximally high	**20**
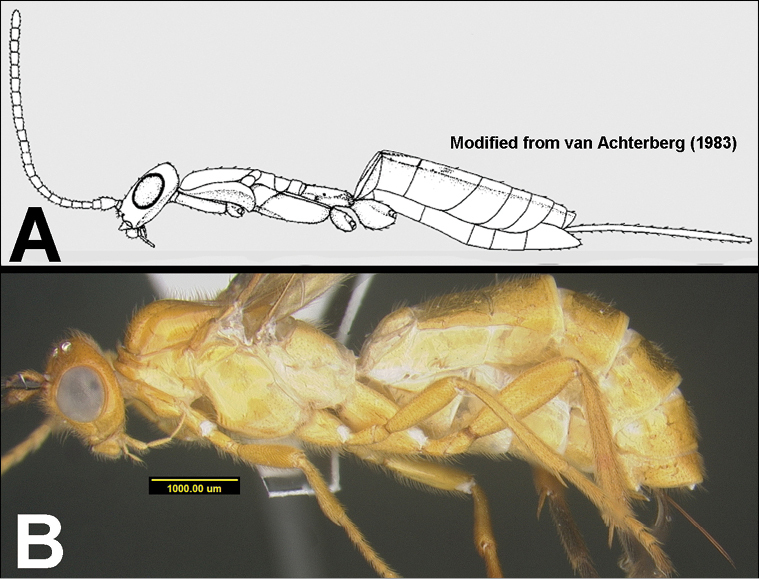		
20(19)	A. Median tergite of petiole very long and narrow, more than 2.0 × longer than maximum width. AA. First tergum usually (in dead specimens) almost vertical and abutting propodeum, and therefore difficult to see	**21**
–	B. Petiole with median tergite not especially elongate, less than 2.0 × longer than maximum width, and first tergum more or less horizontal	**22**
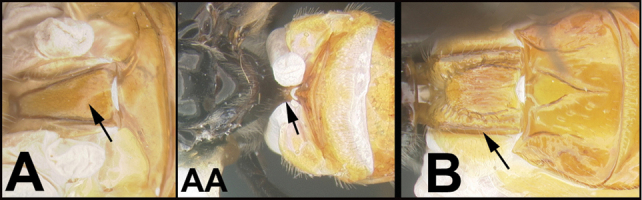		
21(20)	A. Hind femur and tibia with short setae typical of Braconinae; introduced to Florida and a number of Neotropical countries	*** Amyosoma ***
–	B. Hind femur and hind tibia with long black setae; widespread	*** Myosoma ***
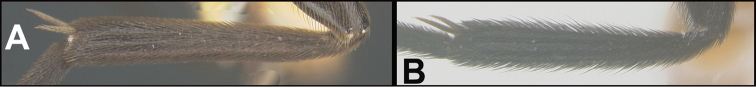		
22(20)	A. Propodeum and metanotum both with a complete lamelliform mid-longitudinal carina. AA. Sternaulus deep and crenulate; Nearctic, rare	*** Lapicida ***
–	B. Propodeum and metanotum both simple, or at most propodeum with a short mid-longitudinal carina posteriorly and/or with metanotum forming a short carina mid-anteriorly. BB. Sternaulus not depressed or if weakly depressed then not crenulate; widespread, common.	**23**
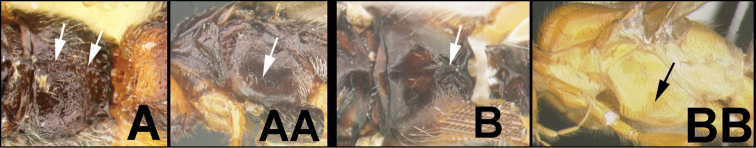		
23(22)	A. Fore wing 3RSa less than 1.5 × length of r (usually less than 1.2 ×); Antenna usually with fewer than 25 flagellomeres; widespread, common	*** Habrobracon ***
–	B. Fore wing 3RSa more than 1.6 × length of r (usually more than 1.8 ×); antenna often with more than 25 flagellomeres; widespread, common	*** Bracon ***
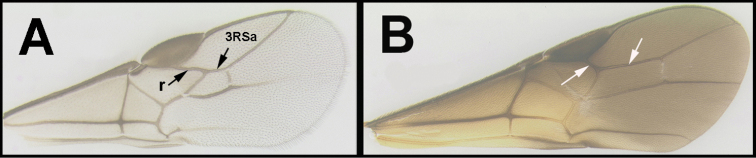		
24(16)	Propodeum coarsely sculptured. A. Forewing marginal cell short, vein 3RS reaching wing margin less than 0.8 × distance between apex of stigma and wing tip	*** Vipiomorpha ***
–	Propodeum smooth and shiny, at most with weak punctures at bases of setae. B. Forewing marginal cell long; vein 3RS reaching wing margin more than 0.8 × distance between apex of stigma and wing tip; forewing RS+M variable, but often distinctly curved or angled posteriorly shortly after arising from 1M	**25**
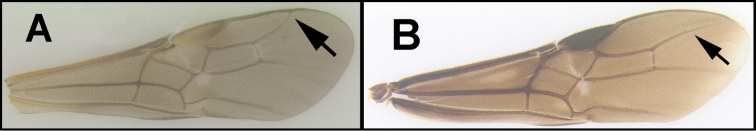		
25(24)	A. Ventral border of clypeus slightly concave in frontal aspect; flagellum with fewer than 50 flagellomeres	*** Alienoclypeus ***
–	B. Ventral border of clypeus slightly convex in frontal aspect; flagellum usually with more than 60 flagellomeres	**26**
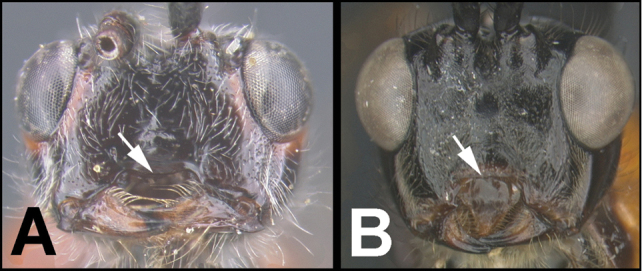		
26(25)	A. Mandibles massive; Neotropical, rare	*** Gnathobracon ***
–	B. Mandibles of normal size; widespread, common	**27**
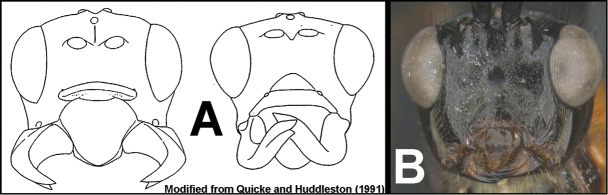		
27(26)	A. With three very long pseudo-ovipositors (more than 5 × longer than body) extending from anal region [true ovipositor (indicated by arrow) much shorter]; Neotropical, very rare	*** Pheloura ***
–	B. Without pseudo-ovipositors	**28**
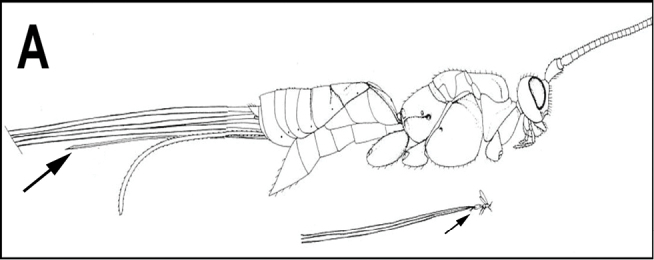		
28(27)	A. Scape very long, more than 2.5 × longer than mid-width; Neotropical, rare	*** Megabracon ***
–	B. Scape much shorter, less than 2.0 × longer than mid-width	**29**
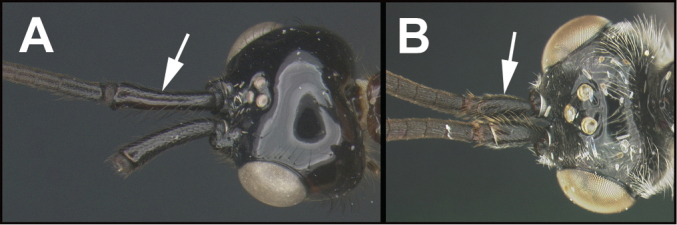		
29(28)	A. Metasomal terga 2‒5 very long and slender, tergum 3 more than 2 × longer than maximum width; posterior margins of terga 3‒5 strongly emarginate and membranous medially, with extremely long internal apodemes; a long, slender wasp; Neotropical, rare	*** Leptobracon ***
–	B. Metasomal terga 2‒5 not as long and slender, tergum 3 less than 2 × longer than maximum width; posterior margins of terga 3‒5 at most weakly emarginate and never with a distinct membranous median zone, and with internal apodemes never more than 2 × longer than medially wide; never as long and slender; widespread, common.	**30**
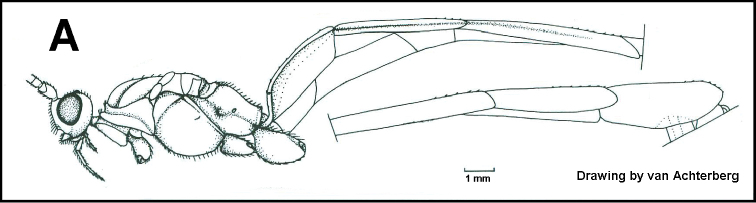		
30(29)	A. Posterior halves of metasomal terga 3‒5 thickly sclerotized and convex in lateral aspect. AA. Metasomal terga coarsely sculptured in most species; widespread, common.	*** Digonogastra ***
–	B. Posterior halves of metasomal terga 3‒5 less thickly sclerotized and straighter in lateral aspect. BB. Metasomal terga mostly smooth; widespread, common.	*** Cyanopterus ***
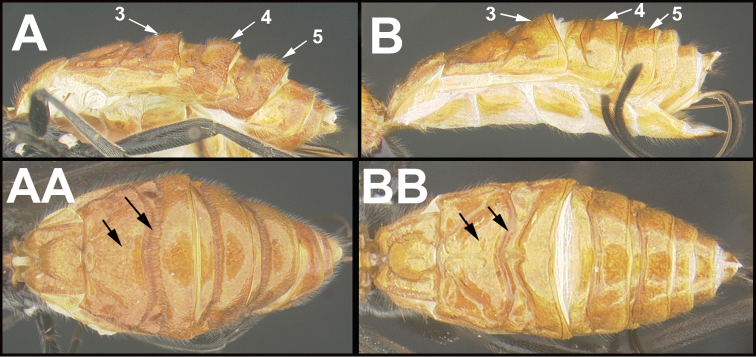		

#### *Bracon* Fabricius, 1804

*Bracon* is an enormous, polyphyletic, cosmopolitan genus with thousands of undescribed species. They are idiobiont ectoparasitoids, attacking hosts in many insect orders, but primarily Coleoptera and Lepidoptera.

##### Bracon alejandromarini

Taxon classificationAnimaliaHymenopteraBraconidae

Sharkey sp. nov.

http://zoobank.org/2E3D9EDE-8B44-4D31-9A86-C0BA04121FD1

Figure 30

###### Diagnostics.

BOLD:ADH4980. Consensus barcode. GGAGTTTTATATTTTTTATTCGGTATATGAGCTGGTATAATTGGTTTATCTATAAGTTTAATTATTCGTTTAGAATTAGGTAYACCAGGAAGTATACTAGGGAATGACCAAATTTATAATAGAATAGTGACTGCTCATGCATTTATTATAATTTTTTTTATAGTTATACCAATTATAATTGGTGGATTTGGAAATTGATTAATTCCTTTAATATTAGGAGCTCCTGATATAGCTTTCCCTCGTTTAAATAATATAAGGTTTTGGTTATTAATTCCTTCATTAACTTTATTATTATTAAGAAGAATTTTAAATATTGGTGTAGGAACAGGATGAACTATATATCCTCCTTTATCTTCAAGTTTAGGCCATAGAGGTATATCAGTTGATTTGGCAATTTTTTCTTTACATTTAGCTGGGGCATCTTCAATTATAGGGGCAATAAATTTTATTACTACTATTTTAAATATACATTTAATAATAATAAAATTAGATCAATTAACTTTATTAATTTGATCTATTTTTATCACAACTATTTTATTATTATTATCTTTACCAGTATTAGCAGGGGCAATTACTATATTATTGACAGAT.

###### Holotype ♀.

Guanacaste, Sector Cacao, Derrumbe, 10.9292, -85.4643, 1220 meters, Malaise trap, 12/iii/2015. Depository: CNC.

***Host data*.** None.

***Holotype voucher code*.**BIOUG32921-A02.

###### Paratype.

BIOUG32997-A06. Depository: CNC.

###### Etymology.

*Braconalejandromarini* is named to honor Alejandro Marin for his efforts to shadow his father, Sigifredo Marin, as an apprentice to the overall managerial aspects of the projects of the Guanacaste Dry Forest Conservation Fund in support of Área de Conservación Guanacaste, and for his enthusiastic willingness to apply his professional medical knowledge to GDFCF staff in Costa Rica.

**Figure 30.**
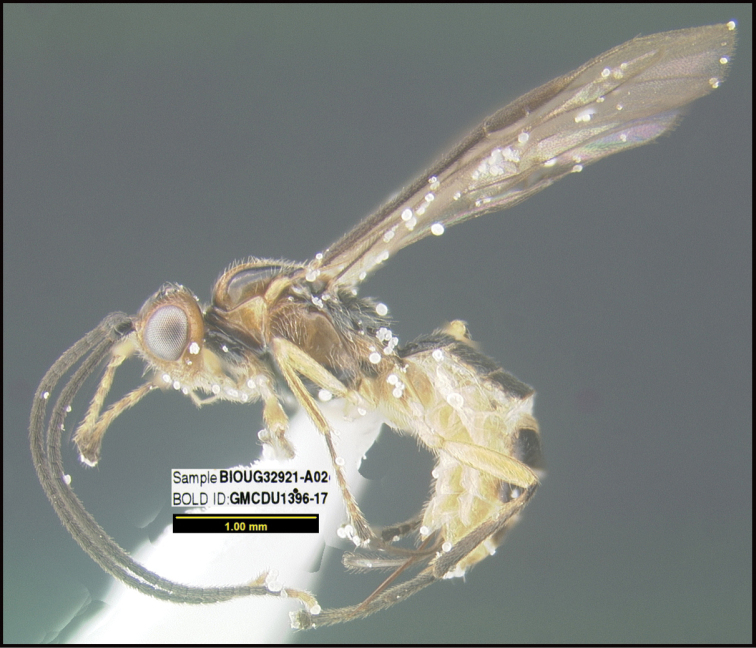
*Braconalejandromarini*, holotype.

##### Bracon alejandromasisi

Taxon classificationAnimaliaHymenopteraBraconidae

Sharkey sp. nov.

http://zoobank.org/CAEAE94C-EA10-41CC-8947-D3F722B6FF12

Figures 31 , 32

###### Diagnostics.

BOLD:AAA5367. Consensus barcode. TGTATTATATTTTTTATTTGGAATATGAGCYGGAATAATTGGTTTATCAATAAGTTTAATTATTCGTTTAGAATTAGGRATACCAGGTAGTTTAYTAGGTAATGATCAAATTTATAATAGTATAGTTACAGCKCATGCTTTTATTATAATTTTTTTTATAGTTATACCAGTAATATTAGGAGGWTTTGGTAATTGATTAGTTCCTTTAATATTAGGTGCTCCTGATATAGCTTTYCCTCGAATAAATAATATAAGATTTTGATTATTAATTCCTTCATTAATTTTATTATTATTAAGAAGAATTTTAAATGTTGGTGTAGGRACAGGCTGAACTATTTATCCTCCTTTATCTTCTATAATAGGTCATAGAGGWATATCTGTRGATTTATCTATTTTYTCTTTACATTTAGCTGGTATTTCTTCTATTATAGGATCGATTAATTTTATTACAACAATTTTAAATATACATTTATTAATATTAAAATTAGATCAATTAACTTTATTTATTTGATCAATTTTTATTACAACTATTTTATTATTATTATCTTTACCTGTATTAGCAGGAGCTATTACTATAYTATTAACTGATCGAAATTTWAATACTTCATTTTTTGATTTTTCTGGAGGTGGGGATCCAATTYTATTTCAACATTTATTT. *Braconalejandromasisi* and *B.tihisiaboshartae* occupy the same BIN and have the same consensus barcode. *Bracontihisiaboshartae* can be differentiated from *B.alejandromasisi* by the color of the metasomal terga: entirely yellow in *B.tihisiaboshartae* and partly black in *B.alejandromasisi*

###### Holotype ♀.

Guanacaste, Sector Cacao, Sendero Nayo, 10.92446, -85.46953, 1090 meters, caterpillar collection date: 22/x/2013, wasp eclosion date: 09/xi/2013, number eclosed 17. Depository: CNC.

***Host data*.** Gregarious parasitoid of *Consulelectra* (Nymphalidae) feeding on leaves of *Piperpsilorhachis* (Piperaceae).

***Caterpillar and holotype voucher codes*.** 13-SRNP-36214, DHJPAR0054650.

###### Note.

*Braconalejandromasisi* and *Bracontihisiaboshartae* occupy the same BIN but are clearly two species, see comments for *B.tihisiaboshartae*.

###### Paratypes.

Hosts = *Consulelectra* and *Consulcecrops* (Nymphalidae) feeding on four species of *Piper* (Piperaceae). 14 specimens, same data as holotype and DHJPAR0029029, DHJPAR0029036, DHJPAR0029031, DHJPAR0034263. Depository: CNC.

###### Etymology.

*Braconalejandromasisi* is named in honor of Alejandro Masis’ persistent high-quality biopoliticking on behalf of ACG in San José, Costa Rica, and especially in Guanacaste Province.

**Figure 31.**
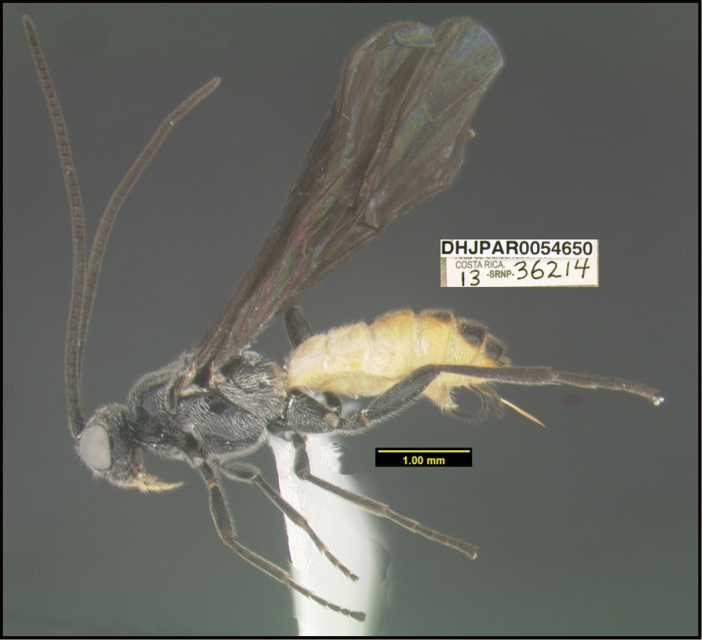
*Braconalejandromasisi*, holotype.

**Figure 32.**
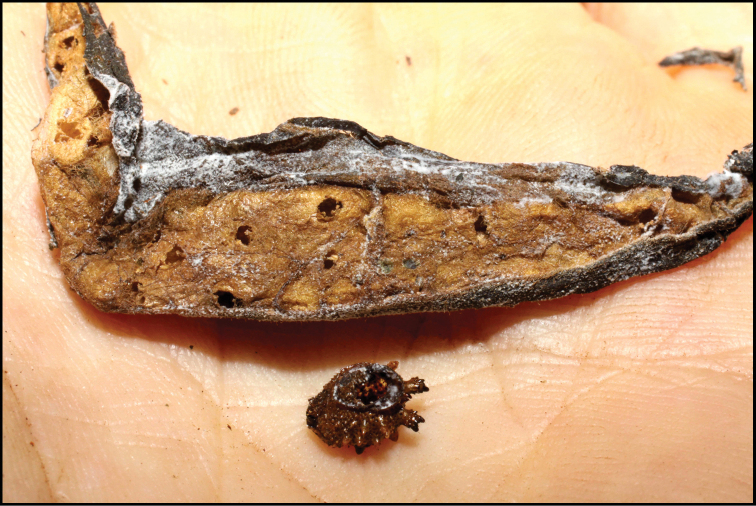
*Braconalejandromasisi* 09-SRNP-20369, dark brown cocoons filling the ultimate instar caterpillar nest of *Consulfabius* (Nymphalidae), with black exit holes cut by the adult wasps.

##### Bracon alexamasisae

Taxon classificationAnimaliaHymenopteraBraconidae

Sharkey sp. nov.

http://zoobank.org/D44BA857-47DF-49AF-85C1-1FB7CFBF4864

Figure 33

###### Diagnostics.

BOLD:ADF2876. Consensus barcode. GTTTTATATTTTTTATTTGGTATATGATCTGGAATATTAGGTTTATCAATAAGGTTAATTATTCGTTTAGAATTAGGTATACCAGGGAGATTATTGGGTAATGATCAAATTTATAATAGTATAGTTACTGCTCATGCTTTTGTAATAATTTTTTTTATGGTTATACCTGTAATAATTGGAGGTTTTGGAAATTGATTATTGCCTTTAATATTAGGAGCTCCAGATATAGCTTTCCCTCGTCTTAATAATATAAGATTTTGGTTATTAATTCCTTCTTTATTTTTATTACTTATAAGAAGAGTTTTAAATGTTGGTGTAGGTACAGGTTGAACAGTTTATCCACCTTTATCTTCTTCTATGGGTCATAGAGGTTTATCTGTTGATTTAGCTATTTTTTCTTTACATATTGCTGGTATTTCTTCTATTTTGGGTGCTATTAATTTTATTACAACTATTTTAAATATACATTTGTATACTTTAAAATTAGATCAGATAACTTTATTAATTTGATCAGTTTTTATTACAGTAATTTTATTATTATTATCTTTACCAGTTTTAGCTGGTGCTATTACTATATTGTTAACTGAT.

###### Holotype ♀.

Guanacaste, Sector Cacao, Derrumbe, 10.9292, -85.4643, 1220 meters, Malaise trap, 6/xi/2014. Depository: CNC.

***Host data*.** None.

***Holotype voucher code*.**BIOUG31518-E04.

###### Paratypes.

None.

###### Etymology.

*Braconalexamasisae* is named to honor Alexa Masis for her cheerful participation in the lives and dedication of the Masis + Boshart family as it has carried out a vital role in the growth and management of the Guanacaste Dry Forest Conservation Fund and Área de Conservación Guanacaste.

**Figure 33.**
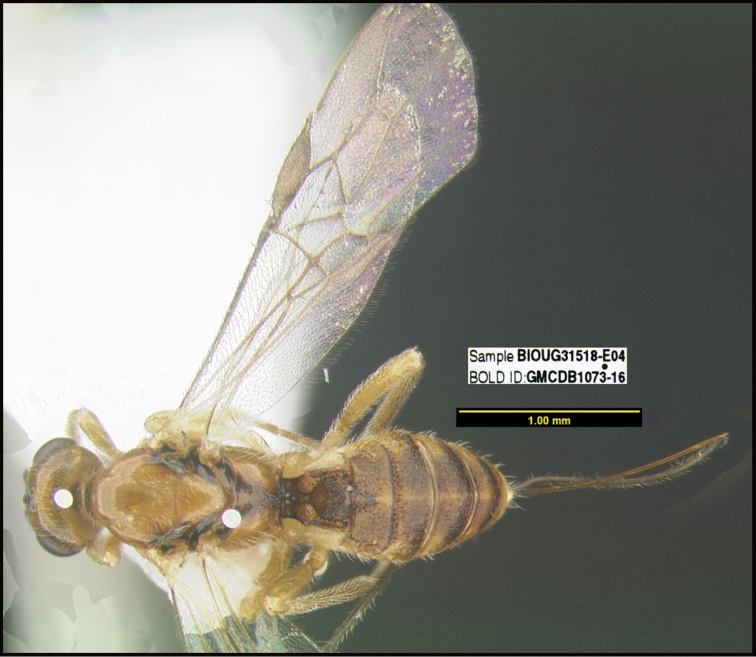
*Braconalexamasisae*, holotype.

##### Bracon andresmarini

Taxon classificationAnimaliaHymenopteraBraconidae

Sharkey sp. nov.

http://zoobank.org/32140FD0-D0EA-4D30-9ACF-CAE7BF00DAEF

Figure 34

###### Diagnostics.

BOLD:ADD6766. Consensus barcode. ATATTATATTTTTTATTTGGATTATGAGCTGGTATATTAGGATTATCTATAAGTTTAATTATTCGTTTAGAATTAGGTATACCTGGTAGATTATTAGGTAATGATCAAATTTATAATAGAATAGTTACTGCCCATGCTTTTGTAATAATTTTTTTTATAGTTATACCAGTAATATTAGGAGGATTTGGAAATTGATTAATTCCGTTAATATTAGGTGCACCAGATATAGCTTTCCCTCGTTTAAATAATATAAGATTTTGATTATTAATTCCTTCATTAATTTTATTAATATTAAGAAGAATTTTAAATATTGGAGTAGGCACAGGATGAACTATATATCCTCCTTTATCATCAAATTTAGGCCACAGAGGTATATCTGTTGATTTAGCAATTTTTTCTTTACATTTAGCCGGAGTATCCTCAATTATAGGCTCAATAAATTTTATTACAACTATTTTAAATATACATTTAGTTATAATAAAATTAGATCAACTAACTTTATTAATTTGATCAATTTTTATTACAACTATTTTATTATTATTATCTTTACCTGTTTTAGCAGGA.

###### Holotype ♀.

Guanacaste, Sector Pailas Dos, PL12-2, 10.7634, -85.335, 824 meters, Malaise trap, 23/i/2014. Depository: CNC.

***Host data*.** None.

***Holotype voucher code*.**BIOUG30922-D05.

###### Paratypes.

None.

###### Etymology.

*Braconandresmarini* is named to honor Andres Marin for his cheerful participation in the lives and business future of the Marin and Romero families as they have carried out its vital role in the growth and management of the Guanacaste Dry Forest Conservation Fund and Área de Conservación Guanacaste.

**Figure 34.**
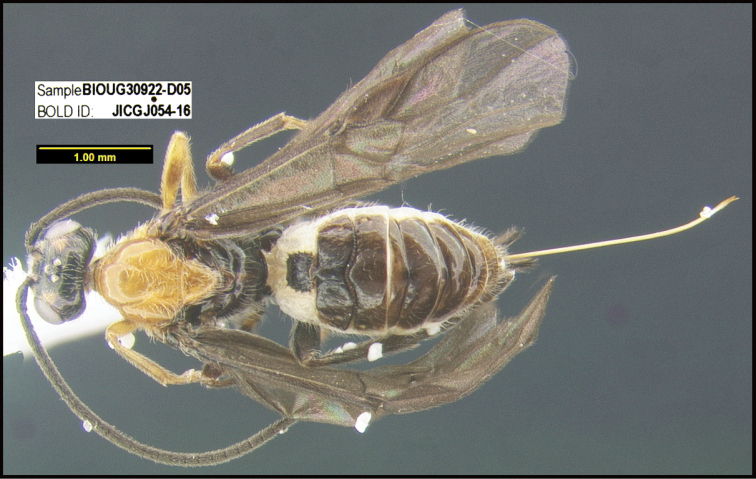
*Braconandresmarini*, holotype.

##### Bracon andrewwalshi

Taxon classificationAnimaliaHymenopteraBraconidae

Sharkey sp. nov.

http://zoobank.org/F951F0BF-BBEB-4DE6-ADFE-414381A22F81

Figure 35

###### Diagnostics.

BOLD:AAY4684. Consensus barcode. AGTTTTATATTTTTTATTTGGTATATGAGCTGGTATAGTAGGTTTATCAATAAGATTAATTATTCGTTTAGAATTAGGTATACCTGGAAGTTTATTAGGTAATGACCAAATTTATAATAGAATAGTTACAGCTCATGCTTTTGTAATAATTTTTTTTATAGTYATACCAGTTATATTAGGTGGATTTGGTAATTGATTAATCCCTTTAATATTAGGAGCCCCTGATATAGCTTTCCCTCGAATAAATAATATAAGTTTTTGGTTGTTAATTCCTTCATTAATTTTATTATTATTAAGAAGAATTTTAAATGTGGGTGTAGGAACAGGATGAACTATATACCCACCTTTATCTTCAAGATTAGGGCATAGAGGTTTATCTGTTGATTTAGCTATTTTTTCTCTACATTTAGCAGGAGTTTCTTCAATTATAGGTTCAATAAATTTTATTACAACTATTTTAAATATACATCTATTAATATTAAAATTAGATCAATTAACTTTATTAGTTTGATCAATTTTTATTACTACTATTTTATTATTGTTATCATTACCTGTTTTAGCAGGAGCTATCACTATATTATTAACTGATCGTAATTTAAATACTTCATTTTTTGATTTTTCAGGAGGTGGGGACCCAATTTTATTCCAACATTTATTT.

###### Holotype ♀.

Guanacaste, Sector Rincon Rain Forest, Zompopera, 10.8884, -85.259, 440 meters, caterpillar collection date: 19/iv/2018, wasp eclosion date: 2/v/2018. Depository: CNC.

***Host data*.***Pirasccatyriotes* (Riodinidae) feeding on *Miconiaargentea* (Melastomataceae).

***Caterpillar and holotype voucher codes*.** 18-SRNP-40675, DHJPAR0062680.

###### Paratypes.

None.

###### Etymology.

*Braconandrewwalshi* is named in honor of Andrew Walsh, son of Karen Sharkey.

**Figure 35.**
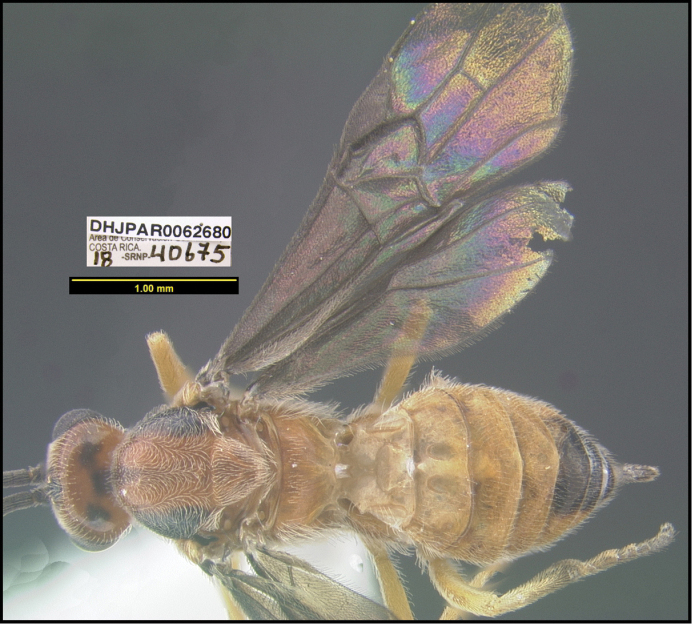
*Braconandrewwalshi*, holotype.

##### Bracon anniapicadoae

Taxon classificationAnimaliaHymenopteraBraconidae

Sharkey sp. nov.

http://zoobank.org/B27AE146-429C-468B-A384-0AE3F7C430AC

Figure 36

###### Diagnostics.

BOLD:ACL5572. Consensus barcode. AATTTTATATTTTTTATTTGGCATATGATCAGGTATAATTGGATTATCTATAAGTTTAATTATTCGATTAGAATTAAGAATACCTGGTAGATTATTAGGTAATGATCAAATTTATAATAGTATAGTAACAGCTCATGCTTTAATAATAATTTTTTTTATAGTTATACCAATTATATTAGGAGGTTTTGGAAATTGATTAATTCCTTTAATATTAGGAGCCCCTGATATAGCTTTCCCACGATTAAATAATATAAGTTTTTGATTATTAATTCCTTCTTTAATTTTATTATTATTAAGAAGAATTTTAAATGTAGGGGTAGGAACAGGTTGAACTATATATCCACCTTTATCATCAAATATGGGACATAGAGGTTTATCTGTTGATTTAGCTATTTTTTCTTTACATTTAGCAGGTATTTCTTCCATTATAGGATCAATTAATTTTATTTCAACTATTTTAAATATACATTTAAAAATATTAAAATTAGATCAATTAACATTATTAATTTGATCAATTTTTATTACAACTATTTTATTATTATTATCATTACCTGTTTTAGCAGGAGCAATTACTATATTATTAACTGATCGAAATTTAAATACTTCTTTTTTTGATTTTTCAGGAGGAGGAGACCCAATTTTATTCCAACATTTATTT.

###### Holotype ♀.

Guanacaste, Sector Santa Rosa, Bosque San Emilio, 10.8438, -85.6138, 300 meters, Malaise trap, 3/ix/2012. Depository: CNC.

***Host data*.** None,

***Holotype voucher code*.**BIOUG08904-B08..

###### Paratypes.

BIOUG09441-F08, BIOUG09739-C05, BIOUG28764-A07, BIOUG28769-G07, BIOUG29282-F10. Depository: CNC.

###### Etymology.

*Braconanniapicadoae* is named to honor Annia Picado for her years of dedicated fly and microlepidopteran processing for the development of the former INBio arthropod collection, now in the Museo Nacional de Costa Rica, and now her furthering the BioAlfa program to DNA barcode all of Costa Rican eukaryote biodiversity.

**Figure 36.**
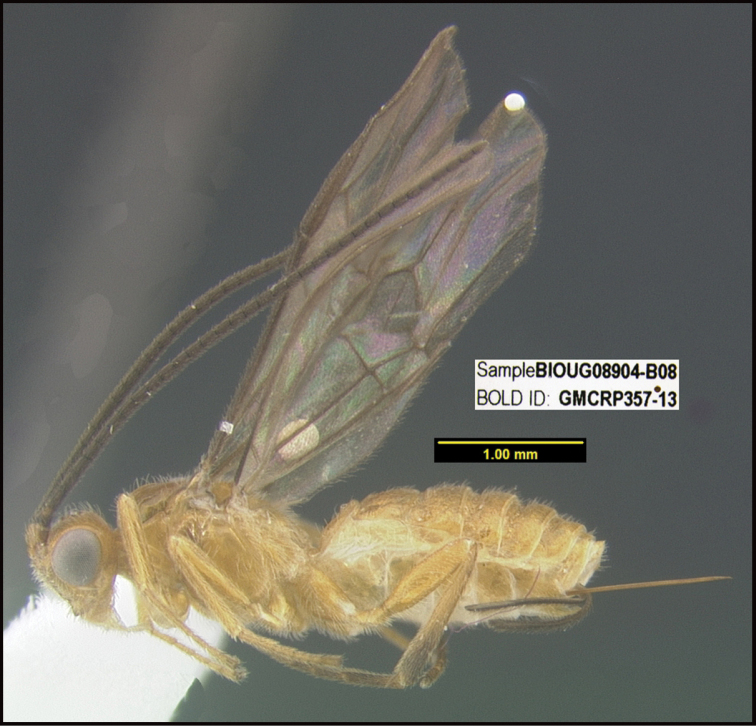
*Braconanniapicadoae*, holotype.

##### Bracon anniemoriceae

Taxon classificationAnimaliaHymenopteraBraconidae

Sharkey sp. nov.

http://zoobank.org/896BF4D4-0B7A-404C-A043-E4CF45B5893F

Figure 37

###### Diagnostics.

BOLD:ADA8088. Consensus barcode. ATTTTATATTTTTTATTTGGTATATGGGCTGGTATAGTTGGTTTATCTATAAGTTTAATTATTCGTTTAGAATTGGGGATACCTGGTAGTTTATTAGGTAATGATCAAATTTATAATAGAATAGTTACAGCTCATGCTTTTGTAATAATTTTTTTTATAGTTATACCAGTTATATTAGGGGGRTTTGGTAATTGATTAATTCCTTTAATATTAGGGTCGCCTGATATAGCATTTCCTCGTTTAAATAATATAAGATTTTGRTTATTAGTTCCTTCATTAATTTTATTATTATTAAGAAGAATTTTAAATGTAGGAGTAGGAACTGGGTGGACAATATATCCCCCTTTATCTTCAAGTTTAGGTCATAGAGGYTTATCTGTTGATTTAGCTATTTTTTCTTTACATTTAGCTGGGGTTTCTTCAATTATAGGTTCAATAAATTTTATTACTACTATTCTTAATATGCATTTATTAATATTAAAATTAGATCARTTGAGTTTATTGATTTGATCAATTTTTATTACTACTATTTTATTATTATTATCTTTACCTGTTTTAGCAGGTGCTATTACTATATTATTAACAGATCGTAATTTAAATACT.

###### Holotype ♀.

Guanacaste, Sector Pailas Dos, PL12-2, 10.7634, -85.335, 824 meters, Malaise trap, 13/ii/2014. Depository: CNC.

***Host data*.** None.

***Holotype voucher code*.**BIOUG28680-H11.

###### Paratypes.

BIOUG28680-G08, BIOUG28770-H05. Depository: CNC.

###### Etymology.

*Braconanniemoriceae* is named to honor Annie Morice for her many years of cheerful office administration on behalf of Área de Conservación Guanacaste.

**Figure 37.**
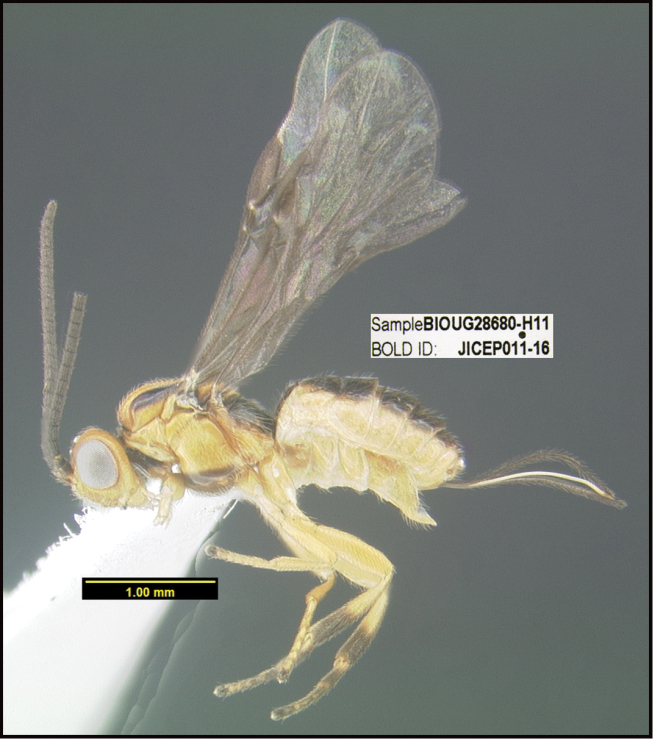
*Braconanniemoriceae*, holotype.

##### Bracon barryhammeli

Taxon classificationAnimaliaHymenopteraBraconidae

Sharkey sp. nov.

http://zoobank.org/68D6A304-BB3D-4227-99FC-8D1D5930D266

Figure 38

###### Diagnostics.

BOLD:ACZ9844. Consensus barcode. TTTTTATTTGGAATTTGATCAGGTTTATTAGGGTTATCAATAAGTTTAATTATTCGTTTAGAATTAGGGACACCTAGAAGTTTAATAATAAATGATCAAATTTATAATAGAATAGTAACATCCCATGCTTTTATTATAATTTTTTTTATAGTAATACCTGTAATATTAGGAGGATTTGGAAATTGACTATTACCTTTAATATTAGGAGCTCCTGATATAGCTTTCCCACGAATAAATAATATAAGATTTTGACTCATTATACCTTCTTTATTTTTGTTATTAATAAGAAGAATTCTGAATGTAGGGGTTGGGACTGGGTGAACTATATACCCTCCATTATCTAGTTCTTTAGGACATAACGGATTATCGGTAGATTTAGCTATTTTTGCTTTACATATAGCTGGAATATCCTCTATTTTAGGATCAATTAATTTTATTACAACTATTTTTAATATACAAATATTAAATTTAAAATTAGATCAATTAACTTTATTTATTTGATCAATTCTTATTACTACTTTTTTATTATTATTATCTTTACCTGTTTTAGCAGGAGCTATTACCATATTACTTACAGATCGT---------------------------------------------------.

###### Holotype ♂.

Guanacaste, Sector San Cristobal, Estación San Gerardo, 10.8801, -85.389, 575 meters, Malaise trap, 3/viii/2015. Depository: CNC.

***Host data*.** None.

***Holotype voucher code*.**BIOUG28152-C12.

###### Paratypes.

None.

###### Etymology.

*Braconbarryhammeli* is named to honor Barry Hammel for his many years of dedicated curatorial and taxonomic efforts for the development of the former INBio plant collection, now in the Museo Nacional de Costa Rica, and now his furthering the BioAlfa program to DNA barcode all of Costa Rican eukaryote biodiversity, with an emphasis on all Costa Rica’s plant species.

**Figure 38.**
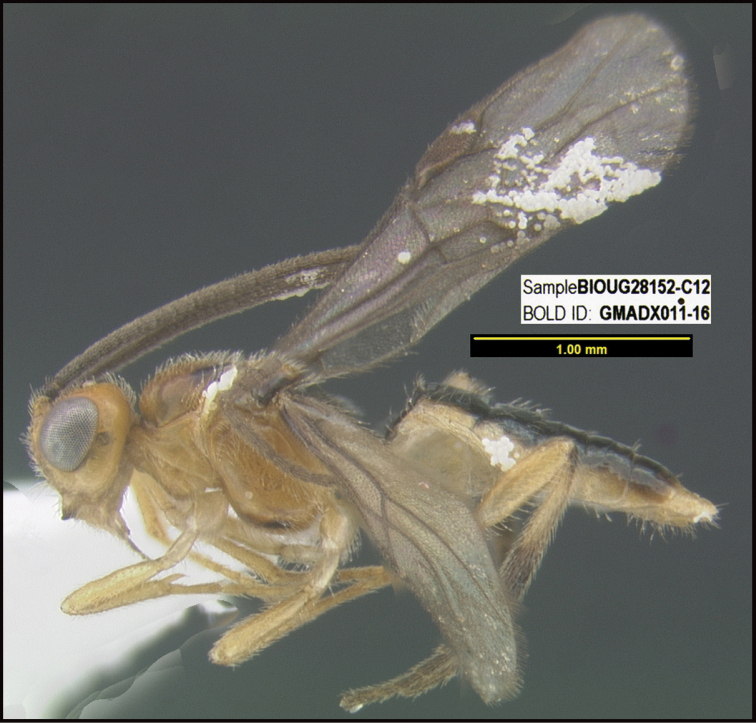
*Braconbarryhammeli*, holotype.

##### Bracon bernardoespinozai

Taxon classificationAnimaliaHymenopteraBraconidae

Sharkey sp. nov.

http://zoobank.org/7D2DA716-0701-49EC-954C-E06447D7AED2

Figure 39

###### Diagnostics.

BOLD:ACG5363. Consensus barcode. TGTTTTATATTTTTTATTTGGGATTTGAGCAGGAATAATTGGATTATCAATAAGATTAATTATTCGGTTAGAATTA---GGAATACCAGGAAATTTATTAAATAATGATCAAATTTATAATAGTATGGTTACTTCTCATGCTTTTGTTATAATTTTTTTTATAGTTATACCAATTATAATTGGAGGATTTGGAAATTGATTAATTCCTTTAATATTAGGGGCTCCTGATATAGCATTCCCCCGTCTAAATAATATAAGATTTTGGTTAATTATTCCTTCAATAATTTTATTATTATTAAGAAGAGTTGTAAATGTAGGTGTAGGTACAGGATGAACAATTTATCCACCTTTATCTTCTAATTTAGGTCACAGAGGGGTTTCAGTAGATATAGCAATTTTTTCTTTACATTTAGCTGGGGTTTCTTCTATTTTAGGGGCAATTAATTTTATTACAACAATTTTAAATATACATTTAAACATTATAAAATTAGATCAATTAACTTTATTAATCTGGTCAATTTTTATTACAACAATTTTATTGCTTTTATCTTTACCAGTATTAGCAGGTGCAATTACTATATTATTAACAGATCGAAATTTAAATACATCTTTTTT-------------------------------------------------------------------.

###### Holotype ♂.

Guanacaste, Sector Santa Rosa, Bosque San Emilio, 10.8438, -85.6138, 300 meters, Malaise trap, 30/vii/2012. Depository: CNC.

***Host data*.** None.

***Holotype voucher code*.**BIOUG05883-A04.

###### Paratypes.

None.

###### Etymology.

*Braconbernardoespinozai* recognizes Bernardo Espinoza for his many years of dedicated curatorial and taxonomic efforts for the enhancement of the former INBio arthropod collection, now in the Museo Nacional de Costa Rica, and now his furthering the BioAlfa program to DNA barcode all of Costa Rican eukaryote biodiversity with an emphasis on Arctiinae.

**Figure 39.**
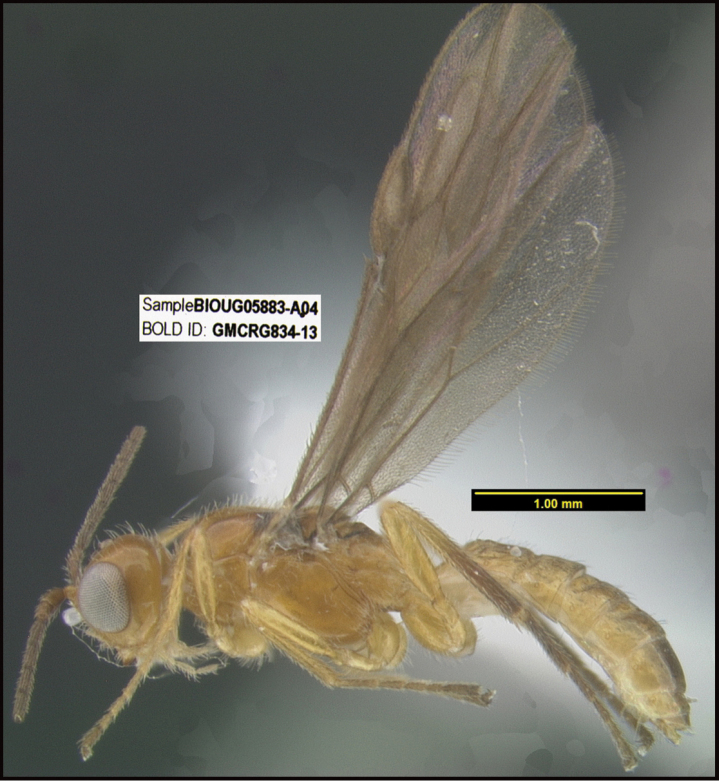
*Braconbernardoespinozai*, holotype.

##### Bracon carlossanabriai

Taxon classificationAnimaliaHymenopteraBraconidae

Sharkey sp. nov.

http://zoobank.org/93F718BE-60A9-459B-96BA-D175305F7F1D

Figure 40

###### Diagnostics.

BOLD:ADA3386. Consensus barcode. GTTTTATATTTTTTATTTGGGATATGAGCTGGTATAGTAGGTTTATCTATAAGATTAATCATTCGTTTAGAATTAGGTATACCTGGTAGTTTACTAGGTAATGATCAAATTTATAATAGAATAGTTACAGCTCATGCTTTTGTAATAATTTTTTTTATAGTTATACCAGTAATAATTGGTGGATTTGGGAATTGATTAATTCCTTTAATATTAGGGGCTCCTGATATAGCTTTTCCACGTTTAAATAATATAAGATTTTGGTTATTAATTCCTTCATTAATTTTATTATTATTAAGAAGAATTTTAAATGTAGGTGTAGGTACTGGTTGAACAATATACCCTCCATTATCTTCAAGATTAGGACATAGGGGTTTATCTGTTGATTTAGCTATTTTTTCTTTACATTTAGCAGGGGTTTCTTCAATTATAGGAGCAATAAATTTTATTACAACTATTTTAAATATACATTTATTAATATTAAAATTAGATCAATTAACTTTATTAATTTGATCTATTTTTATTACAACAATTTTATTATTATTATCTTTACCTGTTTTAGCAGGAGCTATTACTATA---------------------------------------------------------.

###### Holotype ♂.

Guanacaste, Sector San Cristobal, Estación San Gerardo, 10.8801, -85.389, 575 meters, Malaise trap, 14/iv/2014. Depository: CNC.

***Host data*.** None.

***Holotype voucher code*.**BIOUG28246-E07.

###### Paratypes.

None.

###### Etymology.

*Braconcarlossanabriai* is named to honor Carlos Sanabria for his many years of dedicated efforts on behalf of Costa Rica’s Phytosanitary Service (Servicio Fitosanitario del Estado, or SFE), which in turn supports Costa Rica’s new BioAlfa program to DNA barcode the country.

**Figure 40.**
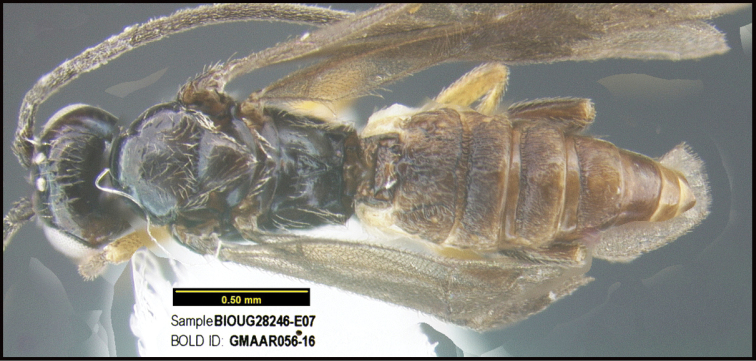
*Braconcarlossanabriai*, holotype.

##### Bracon chanchini

Taxon classificationAnimaliaHymenopteraBraconidae

Sharkey sp. nov.

http://zoobank.org/1D6F07B8-75B8-47E0-BDDD-D61AD13588EB

Figure 41

###### Diagnostics.

BOLD:ACR7966. Consensus barcode. ATTTTATATTTTTTATTTGGAATATGAGCTGGAATATTAGGTATATCTATAAGTTTAATTATTCGATTAGAATTAGGTATACCAGGTAGTTTATTGGGTAATGATCAAATTTATAACAGTATAGTTACTGCTCATGCTTTTGTAATAATTTTTTTTATAGTTATACCAATTATAATTGGAGGGTTTGGGAATTGATTATTACCTTTAATATTAGGAGCCCCTGATATAGCATTCCCTCGTTTGAATAATATAAGGTTTTGATTAATTATCCCTTCTTTAATTTTATTATTAATAAGAAGAATTTTAAATGTAGGTGTTGGAACTGGATGAACAGTATACCCTCCTTTATCTTCTTCTTTAGGACATGGAGGATTATCTATAGATTTAGCTATTTTTTCTTTACATATGGCTGGAATTTCATCTATTTTAGGTGCAATTAATTTTATTACAACTATTTTAAATATGCATTTATTTATTTTGAAGTTGGATCAGTTAACTTTATTAATTTGATCTATTTTTATTACAGTAATTTTATTATTATTATCTTTACCAGTTTTAGCTGGAGCAATCACTATATTATTAACTGAT---------------------------------------------.

###### Holotype ♀.

Guanacaste, Sector Santa Rosa, Bosque San Emilio, 10.8438, -85.6138, 300 meters, Malaise trap, 11/vi/2012. Depository: CNC.

***Host data*.** None.

***Holotype voucher code*.**BIOUG17967-B03.

###### Paratypes.

None.

###### Etymology.

*Braconchanchini* is named to honor Chanchin, an essential member of the fauna of Sector Orosi of ACG, for his dedication to the exploration and conservation of ACG Sector Orosi.

**Figure 41.**
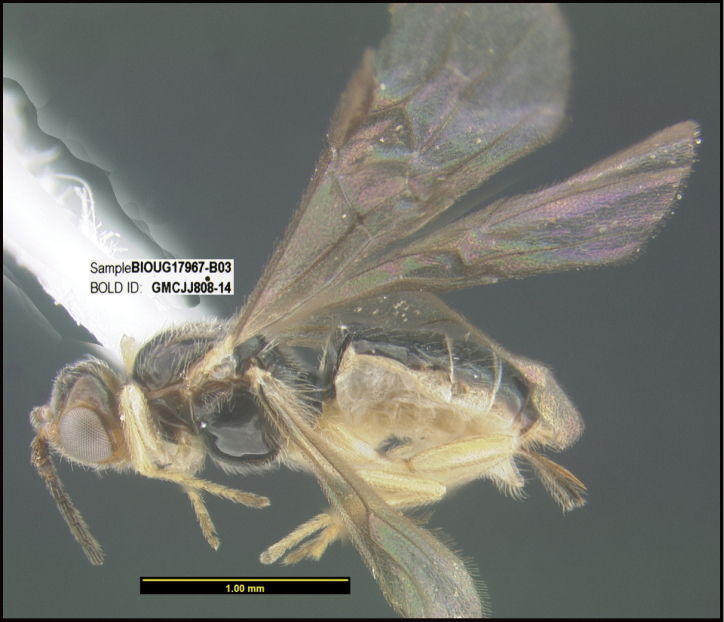
*Braconchanchini*, holotype.

##### Bracon christophervallei

Taxon classificationAnimaliaHymenopteraBraconidae

Sharkey sp. nov.

http://zoobank.org/D6CDAFAB-E699-4479-A6C6-DE2D25A6D401

Figure 42

###### Diagnostics.

BOLD:ADB0826. Consensus barcode. GTTTTATATTTTTTATTTGGTATATGAGCTGGGATAATTGGTTTATCAATAAGTTTAATTATTCGTTTAGAATTAGGCATACCAGGATCTTTATTAAGAAATGATCAAATTTATAATAGAATAGTTACAGCTCATGCTTTTGTTATAATTTTTTTTATAGTTATACCTATTATAATTGGTGGTTTTGGAAATTGATTAATTCCTTTAATATTAGGTTCTCCAGATATAGCTTTCCCTCGTTTAAATAATATGAGATTTTGATTAATTATTCCTGCAATAATTTTATTATTATTAAGGAGAATTTTAAATGTAGGTGTAGGTACTGGTTGAACAATATACCCACCTTTATCTTCTTCATTAGGTCATAGAGGAATTTCAGTTGATTTAGCTATTTTTTCTTTACATTTAGCTGGAGTTTCATCTATTTTAGGTTCAATTAATTTTATTACTACCATTTTAAATATACATTTAAATATTTTAAAGATAGATCAATTAACTTTATTAGTTTGATCAATTTTTATTACAACAATTTTATTACTTTTATCTTTACCTGTTTTAGCAGGT---------------------------------------------------------.

###### Holotype ♂.

Guanacaste, Sector Pailas Dos, PL12-6, 10.7637, -85.333, 853 meters, Malaise trap, 20/iii/2014. Depository: CNC.

***Host data*.** None.

***Holotype voucher code*.**BIOUG29096-E05.

###### Paratypes.

None.

###### Etymology.

*Braconchristophervallei* is named to honor Christopher Valle, ex-fisher in ACG, for his new dedication to the conservation of ACG Sector Marino and the growth and survival of Chanchin as a member of Sector Orosi of ACG.

**Figure 42.**
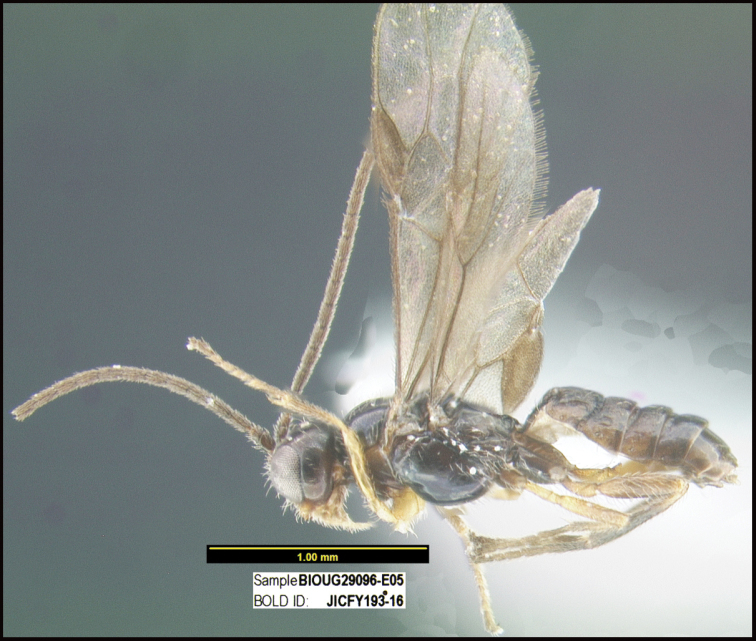
*Braconchristophervallei*, holotype.

##### Bracon erasmocoronadoi

Taxon classificationAnimaliaHymenopteraBraconidae

Sharkey sp. nov.

http://zoobank.org/47FD5F7C-0E33-4C70-ACE8-5CB49C990B46

Figure 43

###### Diagnostics.

BOLD:ADH0668. Consensus barcode. TTTTTATTTGGAATATGAGCCGGAATATTAGGAATATCTATAAGTTTAATTATTCGGTTAGAATTAGGGATACCAGGTAGTTTATTGGGTAATGATCAAATTTATAATAGAATAGTTACTGCTCATGCTTTTGTAATGATTTTTTTTATAGTTATACCAATTATAATTGGAGGATTTGGAAATTGATTACTACCTTTAATATTAGGTGCTCCTGATATAGCATTTCCTCGTTTAAATAATATAAGATTTTGATTAATTATTCCTTCTTTAATTTTATTATTAATAAGAAGAATTTTAAATGTAGGTGTTGGAACTGGTTGAACAGTTTACCCTCCCTTATCTTCTTCTTTAGGTCATAGAGGGCTATCTGTAGATTTAGCTATTTTTTCTTTACATATAGCTGGTATCTCCTCTATTTTAGGAGCAATTAATTTTATCACAACTATTTTAAATATACATTTATTTATTTTAAAATTAGATCAATTGACTTTATTAATTTGATCAATTTTTATCACAGTAATTTTATTACTATTATCTTTACCAGTTTTAGCTGGA-------------------------------------------------------------------.

###### Holotype ♀.

Guanacaste, Sector Cacao, Derrumbe, 10.9292, -85.4643, 1220 meters, Malaise trap, 26/ii/2015. Depository: CNC.

***Host data*.** None.

***Holotype voucher code*.**BIOUG32860-H09.

###### Paratypes.

None.

###### Etymology.

*Braconerasmocoronadoi* is named to honor Erasmo Coronado for his many years of assistance to Sigifredo Marin and other GDFCF and ACG staff, in all aspects of Área de Conservación Guanacaste management.

**Figure 43.**
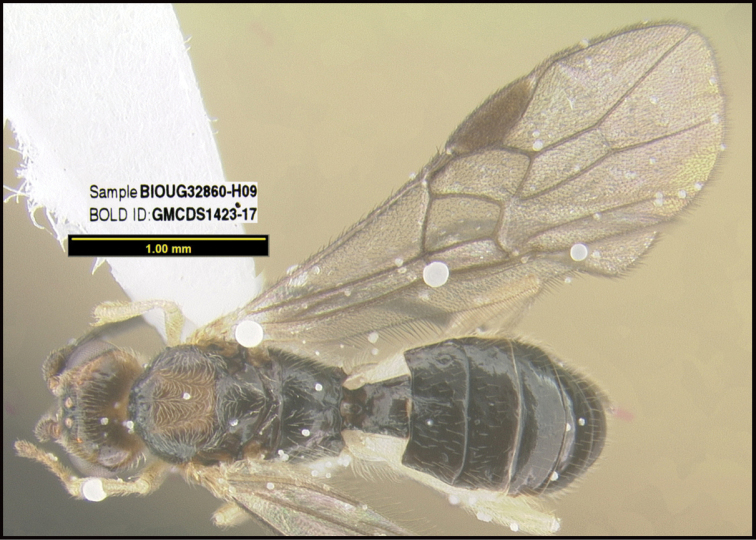
*Braconerasmocoronadoi*, holotype.

##### Bracon eugeniephillipsae

Taxon classificationAnimaliaHymenopteraBraconidae

Sharkey sp. nov.

http://zoobank.org/4A3D13F7-A4A5-4A13-B11A-10A2F17F5D90

Figure 44

###### Diagnostics.

BOLD:ADA9990. Consensus barcode. GTTTTATATTTTTTATTTGGTTTATGATCAGGAATTTTAGGTCTATCAATAAGTTTAATTATTCGATTAGAATTAGGTATACCAGGCAGGATATTAGGTAATGATCAAATTTATAATAGTATTGTAACTGCTCATGCTTTTGTTATAATTTTTTTTATAGTTATACCAATTATAATTGGTGGATTTGGAAATTGGTTATTGCCTTTAATATTAGGAGCTCCTGATATGGCATTYCCTCGTTTAAATAATATAAGATTTTGATTAATTTTCCCTTCTTTAATTTTATTATTAATAAGTAGGATTTTAAATGTAGGAGCAGGTACAGGTTGGACAGTTTATCCTCCTTTATCTTCTTCATTAGGACATAGAGGTTTATCAGTTGATTTAGCTATTTTTTCTTTACATATAGCTGGTGTATCTTCAATTTTAGGAGCAATTAATTTTATCACTACAATTTTAAATATGCATTTAAATACTTTAAARTTAGATCAATTAACTTTAATAATTTGATCAATTTTTATTACTGTAATTTTATTATTGTTATCTTTACCAGTTTTAGCAGGGGCTATTACTATATTATTAACTGATCGA.

###### Holotype ♀.

Guanacaste, Sector Pailas Dos, PL12-9, 10.76, -85.3341, 809 meters, Malaise trap, 6/ii/2014. Depository: CNC.

***Host data*.** None.

***Holotype voucher code*.**BIOUG28680-G05.

###### Paratypes.

BIOUG28680-A07, BIOUG28680-E08, BIOUG29219-D01.

###### Etymology.

*Braconeugeniephillipsae* is named to honor Eugenie (Jenny) Phillips for her many years of dedicated curatorial and taxonomic efforts for the development of the former INBio arthropod collection, now in the Museo Nacional de Costa Rica, and now her administrative development of BioAlfa, the program to DNA barcode all of Costa Rican eukaryote biodiversity.

**Figure 44.**
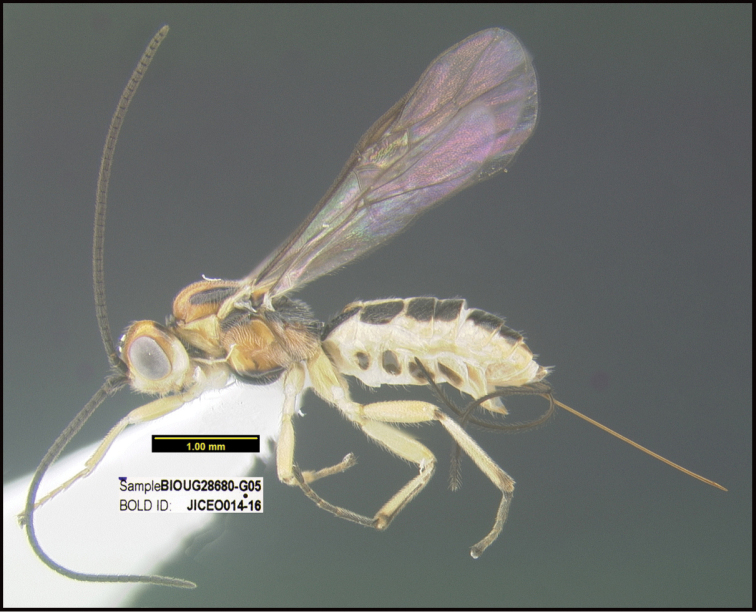
*Braconeugeniephillipsae*, holotype.

##### Bracon federicomatarritai

Taxon classificationAnimaliaHymenopteraBraconidae

Sharkey sp. nov.

http://zoobank.org/3F366019-A277-40D5-A2EB-0239C6B8837D

Figure 45

###### Diagnostics.

BOLD:ACM9419. Consensus barcode. TGTCTTATATTTTTTATTTGGTTTATGAGCTGGAATATTAGGTTTATCTATAAGTTTAATTATTCGATTAGAATTAGGTATACCTGGTAGATTATTAGGTAATGACCAAATTTATAATAGTATAGTTACAGCTCATGCTTTTGTAATAATTTTTTTTATAGTTATACCAGTAATATTAGGAGGATTTGGAAATTGGTTAATTCCTTTAATATTAGGGGCCCCAGATATAGCTTTCCCACGTTTAAATAACATAAGATTTTGATTACTAATTCCTTCATTAATTTTATTATTATTAAGAAGAATTTTAAATATTGGGGTTGGTACAGGTTGGACAATATACCCCCCATTATCATCAAATTTAGGACATAGAGGGATATCTGTTGATTTAGCAATTTTTTCTTTACATTTAGCTGGAATTTCTTCAATTATAGGGTCAATAAATTTTATTACAACTATTTTAAATATACATTTAATTACAATAAAACTAGATCAACTAACTTTATTAGTTTGATCAATTTTTATTACAACTATTTTACTATTATTATCTCTACCTGTTTTAGCAGGGGCTATTACTATACTTTTAACAGATCGTAATTTAAATACTTCTTTTTTCGATTTTTCAGGAGGAGGGGACCCTATTTTATTCCAACATTTATTT.

###### Holotype ♀.

Alajuela, Sector Rincon Rain Forest, Sendero Anonas, 10.90527, -85.27881, 405 meters, caterpillar collection date: 18/iii/2014, wasp eclosion date: 30/iii/2014, 2 wasps eclosed from 2 cocoons. Depository: CNC.

***Host data*.** Gregarious parasitoid of *Tebenna* Janzen02 (Choreutidae) feeding on *Ficuscitrifolia* (Moraceae).

***Caterpillar and holotype voucher codes*.** 14-SRNP-41359, DHJPAR0055286.

**Paratype**: One specimen same data as holotype. Depository: CNC.

###### Etymology.

*Braconfedericomatarritai* is named in honor of Federico Matarrita’s diligent and high-quality management of the ACG web site and guiding the parataxonomists to iteratively contribute to it.

**Figure 45.**
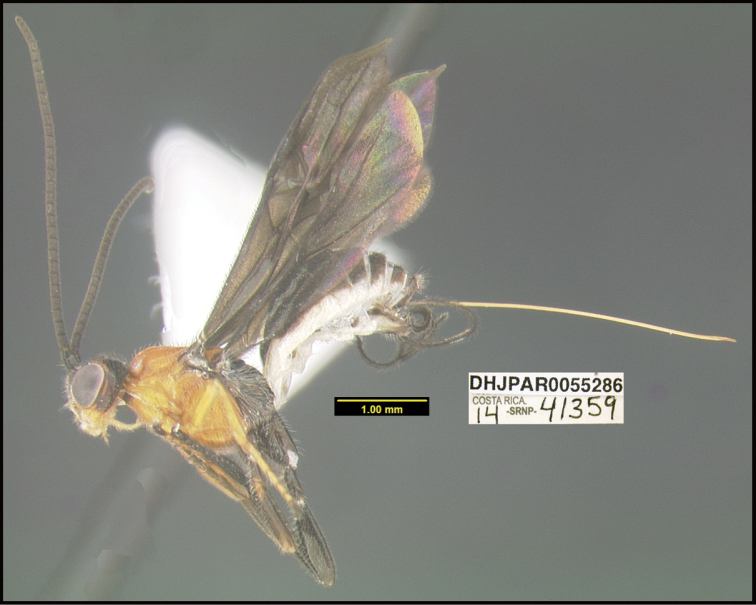
*Braconfedericomatarritai*, holotype.

##### Bracon frankjoycei

Taxon classificationAnimaliaHymenopteraBraconidae

Sharkey sp. nov.

http://zoobank.org/BC84B5AB-1671-4A66-90AD-A8CCDF63BA65

Figure 46

###### Diagnostics.

BOLD:ACJ2744. Consensus barcode. TATTTTATATTTTTTATTTGGTATATGAGCAGGTATAGTAGGTTTATCTATAAGATTAATTATTCGTTTAGAATTAGGTATACCT---GGGAGTTTATTAGGTAATGATCAAATTTATAATAGTATAGTTACAGCTCATGCTTTTGTAATAATTTTTTTTATAGTTATACCTATTATATTAGGTGGGTTTGGAAATTGGTTAATTCCTTTAATATTAGGAGCCCCTGATATAGCTTTCCCTCGATTAAATAATATGAGATTTTGATTATTAATCCCTTCATTAATTTTATTATTATTAAGAAGAATTTTAAATGTTGGTGTAGGTACTGGGTGAACAATATACCCTCCATTATCTTCAAGATTAGGCCATAGAGGTTTATCTGTTGATTTAGCTATTTTTTCTTTACATTTAGCTGGTGTTTCTTCAATTATAGGGTCAATAAATTTTATTACTACAATTTTAAATATACATTTATTAATGTTAAAAATAGATCAATTAACTTTATTAATTTGATCAATTTTTATTACTACTATTTTATTATTATTATCTTTACCAGTATTAGCAGGGGCTATTACAATATTATTAACTGATCGTAACTTAAATACTTCTTTTTTTGACTTTTCTGGCGGAGGGG--------------------------.

###### Holotype ♀.

Guanacaste, Sector Santa Rosa, Bosque San Emilio, 10.8438, -85.6138, 300 meters, Malaise trap, 9/iv/2012. Depository: CNC.

***Host data*.** None.

***Holotype voucher code*.**BIOUG07560-C12.

###### Paratypes.

None.

###### Etymology.

*Braconfrankjoycei* is named to honor Frank Joyce for his many decades of insanely invaluable attention to the biodiversity and sociology on behalf of Parque Nacional Santa Rosa and now Área de Conservación Guanacaste.

**Figure 46.**
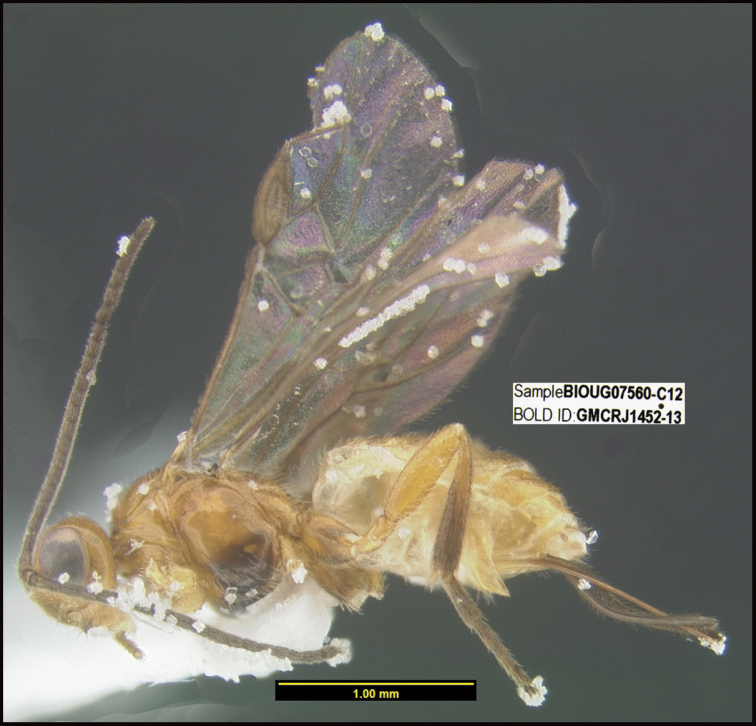
*Braconfrankjoycei*, holotype.

##### Bracon gerardovegai

Taxon classificationAnimaliaHymenopteraBraconidae

Sharkey sp. nov.

http://zoobank.org/625FF1A6-C32E-42BE-8003-64C97E2DE317

Figure 47

###### Diagnostics.

BOLD:ACW3155. Consensus barcode. TTATTAGGGATTTGATCTGGAATTTTAGGATTATCTATAAGATTAATTATTCGGTTAGAACTAGGAACTTCAGGATTTTTATTAGGTAATGATCAAATTTATAATAGATTAGTAACTTCTCATGCTTTTATTATAATTTTTTTTATAGTTATACCTATTATACTAGGAGGATTTGGAAATTGATTAATCCCTTTAATATTGGGGGCTCCTGATATAGCATTCCCTCGAATAAATAATATAAGGTTTTGACTTCTTATTCCTTCATTAATATTATTAATTTTAAGAAGAATCTTAAATGTAGGAGTTGGCACAGGGTGAACAATATATCCTCCTTTATCTTCTTCTATAGGTCATAGAGGATTATCTACAGATTTAGCAATTTTTTCTTTACATTTAGCAGGAGCATCCTCAATTTTAGGAGCTATTAATTTTATTACAACAATTTTTAATATAAAATTAAATTCAATAAAATTAGATCAATTAACTTTATTAATTTGATCTATTTTAATTACTACAATTTTATTATTATTATCTTTACCTGTGTTAGCTGGAGCTATTACTATATTATTAACGGAT.

###### Holotype ♀.

Guanacaste, Sector San Cristobal, Estación San Gerardo, 10.8801, -85.389, 575 meters, Malaise trap, 2/xii/2013. Depository: CNC.

***Host data*.** None.

***Holotype voucher code*.**BIOUG22966-G03.

###### Paratype.

BIOUG27938-F06. Depository: CNC.

###### Etymology.

*Bracongerardovegai* is named to honor Gerardo Vega (RIP) for his many years of dedicated and enthusiastic field assistance to Janzen and Hallwachs’ field ecological studies in Costa Rica’s Parque Nacional Corcovado and Parque Nacional Santa Rosa.

**Figure 47.**
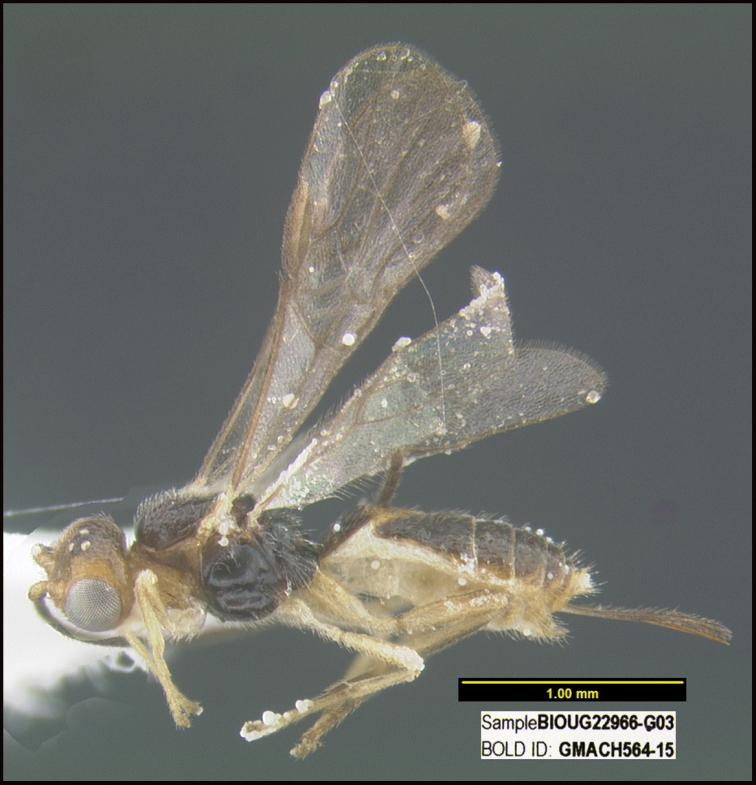
*Bracongerardovegai*, holotype.

##### Bracon germanvegai

Taxon classificationAnimaliaHymenopteraBraconidae

Sharkey sp. nov.

http://zoobank.org/20AB4DA8-ADFF-41D7-AA1F-564796FD611D

Figure 48

###### Diagnostics.

BOLD:ADL8270. Consensus barcode. TTATTAGGGATTTGATCTGGAATTTTAGGATTATCTATAAGATTAATTATTCGGTTAGAACTAGGAACTTCAGGATTTTTATTAGGTAATGATCAAATTTATAATAGATTAGTAACTTCTCATGCTTTTATTATAATTTTTTTTATAGTTATACCTATTATACTAGGAGGATTTGGAAATTGATTAATCCCTTTAATATTGGGGGCTCCTGATATAGCATTCCCTCGAATAAATAATATAAGGTTTTGACTTCTTATTCCTTCATTAATATTATTAATTTTAAGAAGAATCTTAAATGTAGGAGTTGGCACAGGGTGAACAATATATCCTCCTTTATCTTCTTCTATAGGTCATAGAGGATTATCTACAGATTTAGCAATTTTTTCTTTACATTTAGCAGGAGCATCCTCAATTTTAGGAGCTATTAATTTTATTACAACAATTTTTAATATAAAATTAAATTCAATAAAATTAGATCAATTAACTTTATTAATTTGATCTATTTTAATTACTACAATTTTATTATTATTATCTTTACCTGTGTTAGCTGGAGCTATTACTATATTATTAACGGAT.

###### Holotype ♂.

Guanacaste, Sector Cacao, Derrumbe, 10.9292, -85.4643, 1220 meters, Malaise trap, 4/vi/2015. Depository: CNC.

***Host data*.** None.

***Holotype voucher code*.**BIOUG36580-A08.

###### Paratypes.

None.

###### Etymology.

*Bracongermanvegai* is named to honor German Vega for his many years of administrative and taxonomic efforts in the Museo Nacional de Costa Rica.

**Figure 48.**
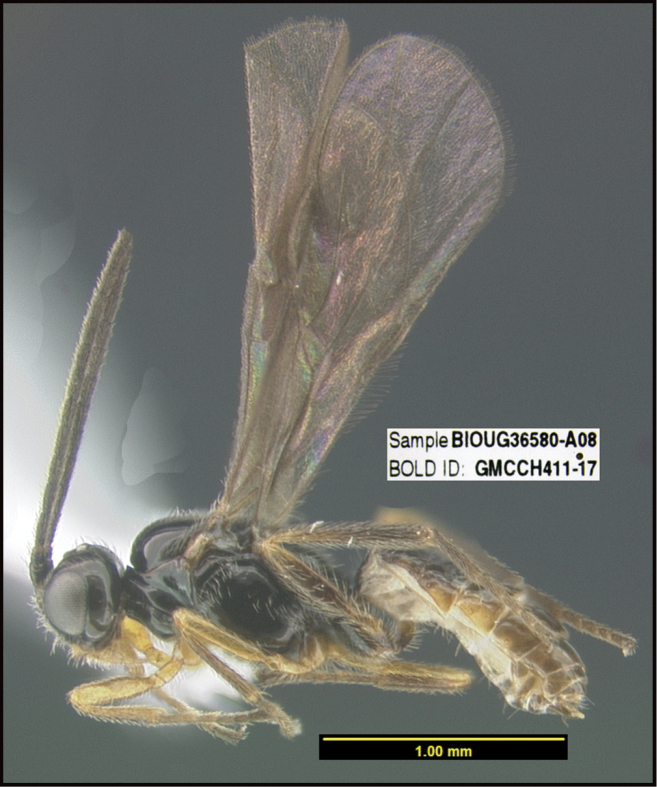
*Bracongermanvegai*, holotype.

##### Bracon isidrochaconi

Taxon classificationAnimaliaHymenopteraBraconidae

Sharkey sp. nov.

http://zoobank.org/5D43849F-37FC-4CB9-8D6B-4EAE482FF773

Figure 49

###### Diagnostics.

BOLD:ADA7614. Consensus barcode. GTTTTATATTTTTTATTTGGAATATGAGCTGGTATAGTTGGTTTATCAATAAGAATAATTATTCGTTTAGAATTAGGTATACCTGGATTTTTATTAATAAATGATCAAATTTATAATAGAATAGTAACTGCTCATGCTTTTGTTATAATTTTTTTTATAGTTATACCAATTATAATTGGTGGATTTGGGAATTGATTAGTTCCTTTAATATTAGGTTCTCCAGATATAGCTTTCCCTCGTTTAAATAATATAAGATTTTGATTAATTATCCCAGCAATAATTTTATTATTATTAAGAAGAATTTTAAATGTTGGTGTAGGTACAGGTTGAACAATTTACCCTCCTTTATCTTCTTCTTTAGGACATAGAGGAATTTCAGTTGATTTAGCTATTTTTTCTTTACATTTAGCTGGTATTTCATCTATTTTAGGTTCAATTAATTTTATTACAACAATTTTAAATATACATTTAAATGTATTAAAGATAGATCAATTAACTTTATTTGTTTGGTCAATTTTTATTACTACAATTTTATTATTATTATCTTTACCTGTTTTAGCTGGAGCTATTACAATATTA------------------------------------------------------.

###### Holotype ♀.

Guanacaste, Sector Pailas Dos, PL12-9, 10.76, -85.3341, 809 meters, Malaise trap, 23/i/2014. Depository: CNC.

***Host data*.** None.

***Holotype voucher code*.**BIOUG28721-H09.

###### Paratypes.

None.

###### Etymology.

*Braconisidrochaconi* is named to honor Isidro Chacon for his many years of dedicated curatorial and taxonomic efforts for the advancement of the former INBio arthropod collection, now in the Museo Nacional de Costa Rica, and now his furthering the BioAlfa program to DNA barcode all of Costa Rican eukaryote biodiversity with an emphasis on Notodontidae, butterflies, Bombycoidea, and Noctuoidea.

**Figure 49.**
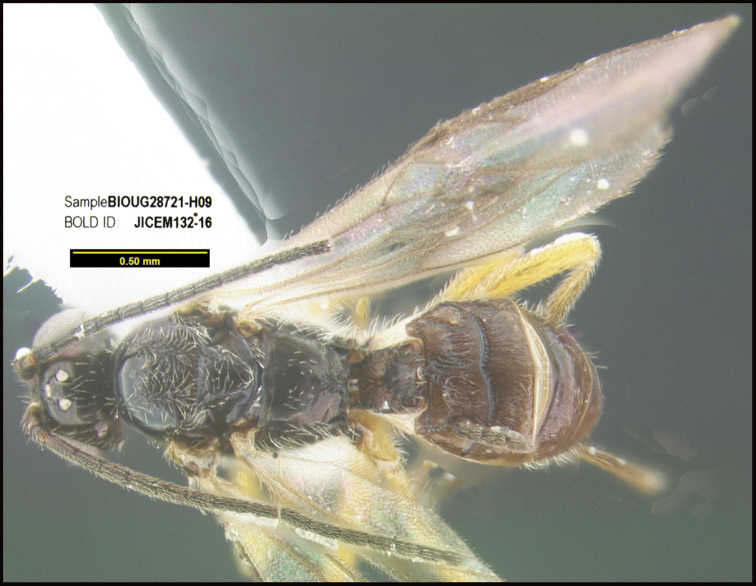
*Braconisidrochaconi*, holotype.

##### Bracon jimlewisi

Taxon classificationAnimaliaHymenopteraBraconidae

Sharkey sp. nov.

http://zoobank.org/BCAEFC06-5469-44AA-9B8F-6F9BB5204248

Figure 50

###### Diagnostics.

BOLD:ADL5861. Consensus barcode. TGTTTTATATTTTTTATTTGGGGTTTGATCTGGATTTTTAGGTTTATCTATAAGATTAATTATTCGTATAGAATTGAGTATACCAGGAAGATTATTAAGTAATGATCAAATTTATAATAGTTTAGTAACTGCTCATGCTTTTGTTATAATTTTTTTTATAGTTATACCAGTAATAATTGGAGGTTTTGGAAATTGATTAATTCCTTTAATATTAGGGGCTCCTGATATAGCTTTCCCTCGATTAAATAATATAAGATTTTGGTTAATTATTCCTTCATTAATACTATTAATTTTAAGAAGAATTTTAAATGTTGGGGTAGGTACAGGTTGAACAATATACCCCCCTTTATCTTCTTCTTTAGGGCATAGAGGTAATTCAACTGATTTAGCTATTTTTTCTTTACATATAGCTGGAATTTCTTCAATTTTAGGGGCTATTAATTTTATTACAACTATTTTTAATATAAAATTATTTTTTTTAAAATTTGATCAATTAACTTTATTTATTTGATCAATTTTAATTACAACAATTTTATTATTGTTATCTTTACCAGTTTTAGCTGGGGCAATCACAATA---------------------------------------------.

###### Holotype ♀.

Guanacaste, Sector Cacao, Derrumbe, 10.9292, -85.4643, 1220 meters, Malaise trap, 9/vii/2015. Depository: CNC.

***Host data*.** None.

***Holotype voucher code*.**BIOUG36967-F06.

###### Paratypes.

None.

###### Etymology.

*Braconjimlewisi* is named to honor Jim Lewis for his many years of dedicated volunteer curatorial and taxonomic efforts for the construction of the former INBio arthropod collection, now in the Museo Nacional de Costa Rica, and now his furthering the BioAlfa program to DNA barcode all of Costa Rican eukaryote biodiversity, with an emphasis on Hemiptera.

**Figure 50.**
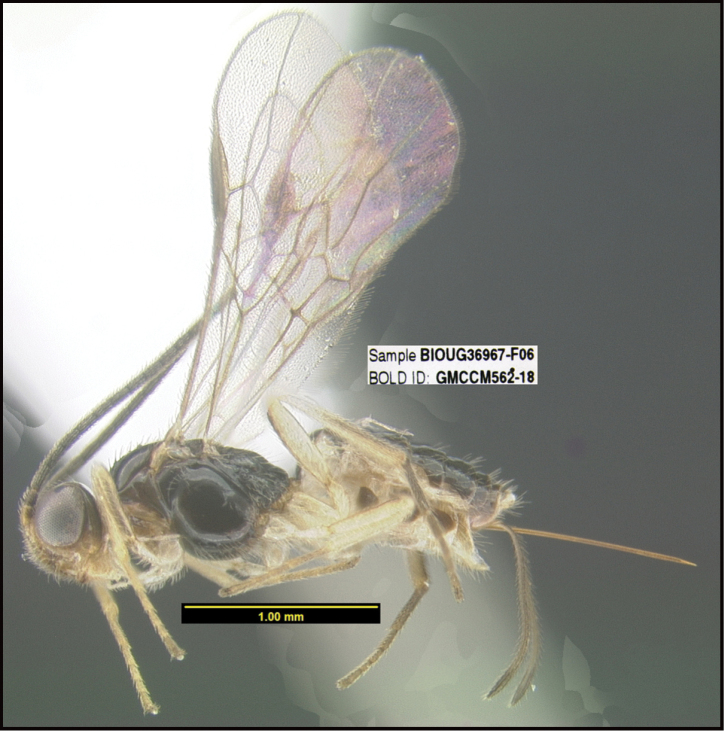
*Braconjimlewisi*, holotype.

##### Bracon josejaramilloi

Taxon classificationAnimaliaHymenopteraBraconidae

Sharkey sp. nov.

http://zoobank.org/AB4F8A19-38C0-4D52-AB61-BC298878188B

Figures 51 , 52

###### Diagnostics.

BOLD:ACK7911. Consensus barcode. TATTTTATATTTTTTATTTGGTATATGAGCTGGTATGTTAGGTTTATCAATAAGAATAATTATTCGGTTAGAATTAGGTATACCAGGAAGATTACTAGGTAATGATCAAATTTATAATAGTATAGTTACTGCTCATGCATTTGTTATAATTTTTTTTATAGTTATACCAATTATATTAGGAGGATTTGGAAATTGATTAGTTCCTTTAATATTAGGAGCTCCTGATATAGCTTTCCCTCGTATAAATAATATAAGATTTTGATTAATTATTCCTTCTTTAGTTTTATTATTATTAAGAAGAATTTTAAATATTGGGGCAGGAACAGGATGAACTATATATCCTCCTTTATCTTCTCATTTAGGTCATAGAGGTATATCAGTTGATTTAGCTATTTTTTCTTTACATATAGCAGGGATTTCATCAATTTTAGGATCTATTAATTTTATTACAACAATTTTAAATATACATTTATTTACTTTAAAATTAGATCAATTAACTTTATTAGTTTGATCAATATTTATTACTACAATTTTATTATTATTATCTTTACCAGTTTTAGCAGGGGGTATTACAATATTATTAACAGACCGTAAYCTAAATACTTCATTTTTTGATTTTTCTGGAGGAGGAGATCCTATTTTATTCCAACATTTATTT.

###### Holotype ♀.

Guanacaste, Sector Pitilla, Pasmompa, 440 meters, 11.01926, -85.40997, caterpillar collection date: 21/ii/2012, wasp eclosion date: 08/iii/2012, 11 wasps eclosed from 15 cocoons. Depository: CNC.

***Host data*.** Gregarious parasitoid (Fig. 52) of *Leremaliris* (Hesperiidae) feeding on leaves of *Homolepisaturensis* (Poaceae).

***Caterpillar and holotype voucher codes*.** 12-SRNP-30473, DHJPAR0049094.

###### Paratypes.

Hosts = *Leremaliris* and *Morysvalerius* (Hesperiidae). DHJPAR0049094, DHJPAR0028977, DHJPAR0058913.

###### Etymology.

*Braconjosejaramilloi* is named in honor of José Jaramillo of Área de Conservación Guanacaste in recognition of his 30 years of managing ACG mechanics, telecommunication, computer, and internet processing for ACG and its web site.

**Figure 51.**
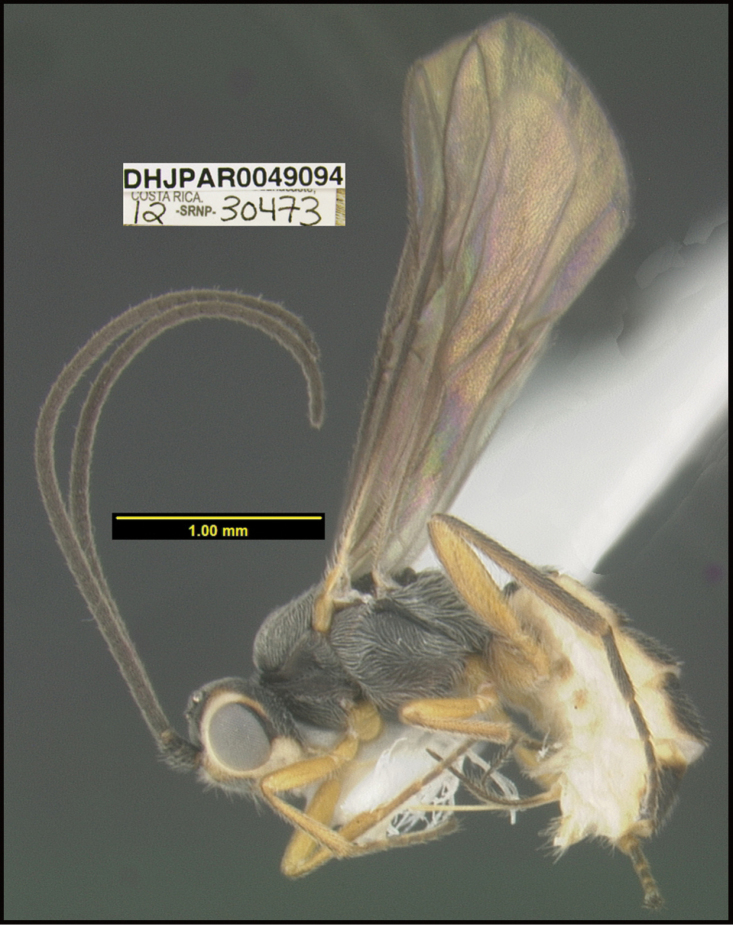
*Braconjosejaramilloi*, holotype.

**Figure 52.**
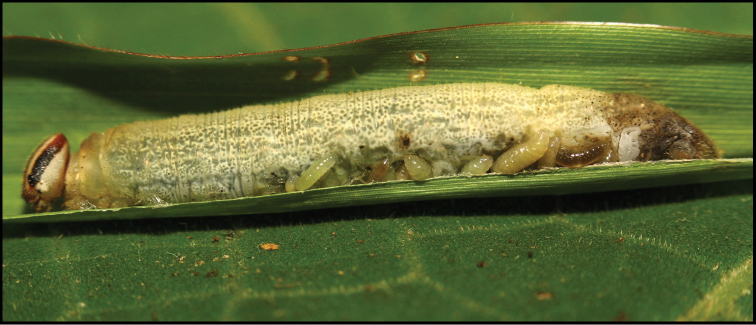
*Braconjosejaramilloi* (12-SRNP-30473) ultimate instar wasp larvae below their host ultimate instar caterpillar *Leremaliris* (Hesperiidae); as a group they will spin a roof-in-common followed by their individual closely packed cocoons below that roof, adjacent to the cadaver of the caterpillar.

##### Bracon juanjoseoviedoi

Taxon classificationAnimaliaHymenopteraBraconidae

Sharkey sp. nov.

http://zoobank.org/ABC4DA51-51DA-4322-968B-E6C54DE67754

Figure 53

###### Diagnostics.

BOLD:ADB0539. Consensus barcode. ATTTTATATTTTTTATTTGGTATATGAGCTGGAATATTAGGTTTATCAATAAGATTAATTATTCGATTAGAATTAGGAATACCAGGAAGATTATTAGGAAATGATCAAATTTATAATAGAATAGTGACAGCTCATGCATTTGTTATAATTTTTTTTATAGTTATACCAATTATAATTGGAGGATTTGGAAATTGATTATTACCTTTAATATTAGGGGCTCCTGATATAGCTTTTCCTCGTCTTAATAATATAAGATTTTGATTAATTATTCCGGCTTTAATTTTATTACTAATAAGAAGAATTTTAAATGTAGGTGTAGGTACTGGTTGAACAGTTTATCCTCCTTTATCTTCTTCTTTAGGTCATAGAGGTTTATCTGTTGATTTAGCTATTTTTTCTTTACATATTGCTGGTATTTCTTCAATTTTAGGGGCAATTAATTTTATTACAACAATTTTAAATATACATTTATATAAATTAAAATTAGATCAATTAACTTTATTAACTTGATCAATTTTTATTACAGTAATTCTTTTACTTTTATCTTTACCAGTTTTAGCTGGAGCTATTACTATACTTTTAACTGATCGA------------------------------.

###### Holotype ♀.

Guanacaste, Sector Pailas Dos, PL12-2, 10.7634, -85.335, 824 meters, Malaise trap, 13/ii/2014. Depository: CNC.

***Host data*.** None.

***Holotype voucher code*.**BIOUG28680-H09.

###### Paratypes.

None.

###### Etymology.

*Braconjuanjoseoviedoi* is named to honor Juan José Oviedo for his new efforts on behalf of Costa Rica’s Phytosanitary Service (Servicio Fitosanitario del Estado, or SFE), which in turn supports Costa Rica’s new BioAlfa program to DNA barcode the country.

**Figure 53.**
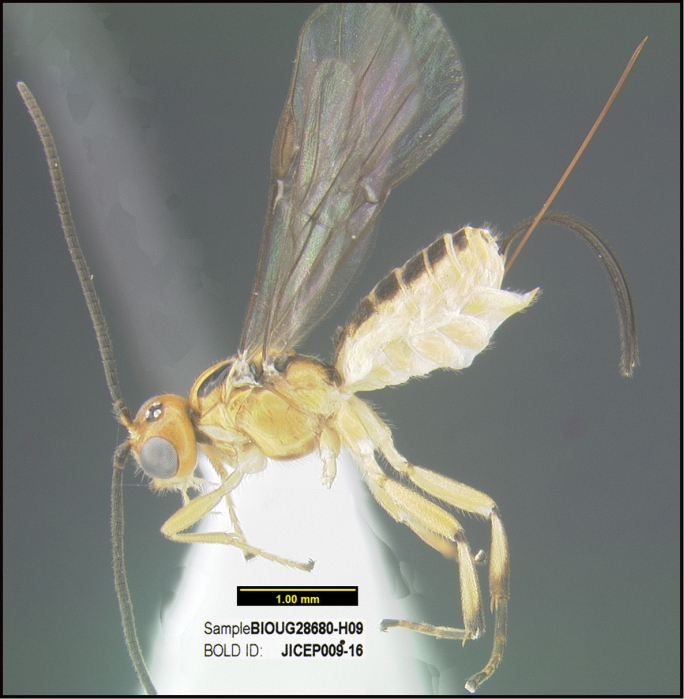
*Braconjuanjoseoviedoi*, holotype.

##### Bracon juliodiazi

Taxon classificationAnimaliaHymenopteraBraconidae

Sharkey sp. nov.

http://zoobank.org/47151893-CF98-415F-9D15-F2596E66AF92

Figure 54

###### Diagnostics.

BOLD:ACG3370. Consensus barcode. TATTTTATATTTTTTATTCGGAATATGAGCAGGTATATTAGGATTATCTATAAGAATAATTATTCGATTAGAATTAGGRATACCTGGGACTTTATTAGGCAATGATCAAATTTATAATAGAATAGTGACAGCTCATGCATTTGTTATAATTTTTTTTATAGTTATACCAATTATACTAGGGGGTTTCGGTAATTGATTAATTCCTTTAATATTAGGAGCCCCAGACATAGCTTTCCCTCGAATAAATAATATAAGATTTTGATTAATTATTCCTTCTTTAATTTTATTATTATTAAGAAGAATTTTAAATGTAGGGGTAGGTACTGGATGAACTGTTTATCCTCCCTTATCTTCTTCTTTAGGTCATAGAGGCATATCTGTTGATTTAGCAATTTTTTCATTACATTTAGCAGGTATTTCTTCAATTTTAGGTTCAATTAATTTTATTACAACTATTTTAAATATAAAATTACATACAATAAAATTAGATCAATTAACTTTATTAATTTGATCAATTTTTATTACTACAATTTTATTATTATTATCTTTACCAGTTYTAGCGGGAGCTATTACAATATTATTAACTGATCGAAATTTAAATACTTCATTTTTTGATTTTTCTGGGGGAGGTGACCCTATTTTATTCCAACATTTATTT.

###### Holotype ♀.

Guanacaste, Sector Santa Rosa, Bosque San Emilio, 10.8438, -85.6138, 300 meters, Malaise trap, 29/x/2012. Depository: CNC.

***Host data*.** None.

***Holotype voucher code*.**BIOUG09074-C11.

###### Paratypes.

BIOUG09074-B05, BIOUG09074-B02. Depository: CNC.

###### Etymology.

*Braconjuliodiazi* is named to honor Julio Diaz for his many years of dedicated guidance and administration of the ACG Protection and Fire Control Program.

**Figure 54.**
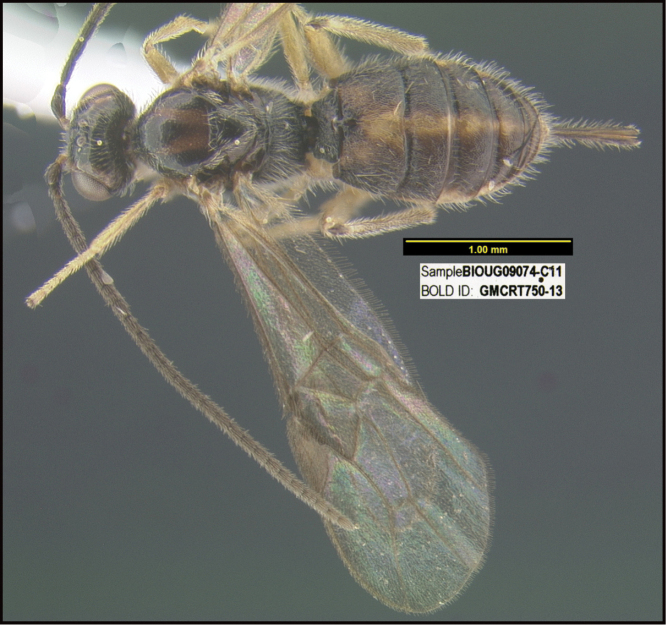
*Braconjuliodiazi*, holotype.

##### Bracon luzmariaromeroae

Taxon classificationAnimaliaHymenopteraBraconidae

Sharkey sp. nov.

http://zoobank.org/56DBE55B-8273-435C-A7C6-DA99BD00598E

Figure 55

###### Diagnostics.

BOLD:ADF0526. Consensus barcode. AATTTTATATTTTTTATTTGGAATATGATCTGGAATAATTGGTTTATCTATAAGTTTAATTATTCGTTTAGAATTAGGAATACCTGGAAGATTATTGAGAAATGACCAAATTTATAATAGAATAGTTACAGCTCATGCTTTAGTAATAATTTTTTTTATAGTTATACCAATTATATTAGGAGGATTTGGTAATTGATTAATTCCTTTAATATTAGGAGCTCCTGATATAGCTTTCCCTCGATTAAATAATATAAGATTTTGATTATTAATTCCTTCTTTAATTTTATTATTATTAAGAAGAATTTTAAATGTTGGTGTTGGAACAGGATGAACTTTATACCCTCCTTTATCTTCAAATTTAGGACATAATGGAGTATCTGTAGATTTATCTATTTTTTCTTTACATTTAGCTGGTATTTCATCAATTATAGGTTCATTAAATTTTATTACTACTATTTTAAATATACATTTATTAATATTAAAATTAGATCAATTAACTTTATTAATTTGATCAATTTTTATTACAACTATTTTATTATTATTATCTTTACCTGTTTTAGCAGGAGCTATTACTATATTATTAACTGATCGTAATTTAAATACTTCATTTTTTGATTTTTCAGGAGGTGGTGACCCAATTCTATTTCAACATTTATTT.

###### Holotype ♂.

Alajuela, Sector Rincon Rain Forest, Casa Keyner, 10.95644, -85.26611, 121 meters, caterpillar collection date: 13/viii/2016, wasp eclosion date: 23/viii/2016, six wasps eclosed from six cocoons. Depository: CNC.

***Host data*.** Gregarious parasitoid of *Vettiusaurelius* (Hesperiidae) feeding on mature leaves of *Lasiacissorghoidea* (Poaceae).

***Caterpillar and holotype voucher codes*.** 16-SRNP-46268, DHJPAR0060193.

###### Paratypes.

Five specimens, same data as holotype but still coded with their caterpillar code 16-SRNP-46268 (DHJPAR codes not assigned). Depository: CNC.

###### Etymology.

*Braconluzmariaromeroae* is named in recognition of Luz Maria Romero’s 30+ years of facilitating ACG, developing its Biological Education Program, and coaching the parataxonomists to improve their databases and species pages on the ACG web site.

**Figure 55.**
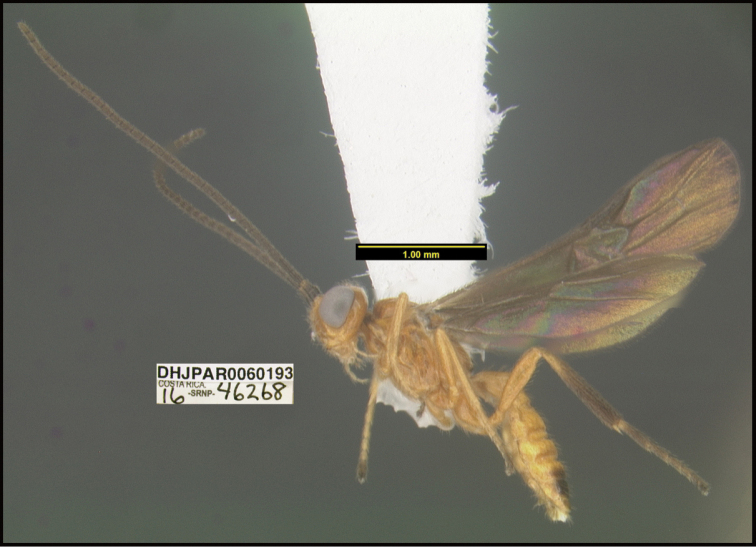
*Braconluzmariaromeroae*, holotype.

##### Bracon manuelzumbadoi

Taxon classificationAnimaliaHymenopteraBraconidae

Sharkey sp. nov.

http://zoobank.org/3ADD20C5-CF2D-43E5-9D30-2CB0B83F2DA3

Figure 56

###### Diagnostics.

BOLD:ADH5864. Consensus barcode. GTGTTTTATATTTTTTGTTTGGTATATGGTCTGGGATATTAGGGATATCTATAAGATTAATTATTCGATTAGAATTAGGAATACCCGGTAGATTATTAGGGAATGATCAACTTTATAATAGTATGGTAACAGCTCATGCTTTTGTAATAATTTTTTTTATAGTAATACCTGTAATAATTGGGGGTTTTGGCAATTGATTATTACCTTTAATATTAGGGTCCCCTGATATAGCTTTCCCTCGTCTTAATAATATAAGATTTTGATTGATTATTCCTTCATTAATTTTGTTATTAATAAGAAGAATTTTGAATGTAGGTGTAGGTACTGGTTGAACTGTATATCCTCCTTTATCTTCTTCTTTAGGACATAGGGGGATATCTGTTGATTTAGCTATTTTTTCTTTACATATTGCTGGAGTATCCTCAATTTTAGGAGCAATTAATTTTATTAGAACTATTTTAAATATACATTTATTTATATTAAAATTGGATCAATTAACTTTGTTAATTTGATCAATTTTTATTACTGTTATTTTATTATTATTATCTTTACCAGTTTTAGCTGGTGCAATTACTATATTATTAACTGAT.

###### Holotype ♀.

Guanacaste, Sector Cacao, Derrumbe, 10.9292, -85.4643, 1220 meters, Malaise trap, 2/iv/2015. Depository: CNC.

***Host data*.** None.

***Holotype voucher code*.**BIOUG33120-A12.

###### Paratypes.

None.

###### Etymology.

*Braconmanuelzumbadoi* is named to honor Manuel Zumbado for his many years of dedicated curatorial and taxonomic efforts for the construction of the former INBio arthropod collection, now in the Museo Nacional de Costa Rica, and now his furthering the BioAlfa program to DNA barcode all of Costa Rican eukaryote biodiversity, with an emphasis on Diptera.

**Figure 56.**
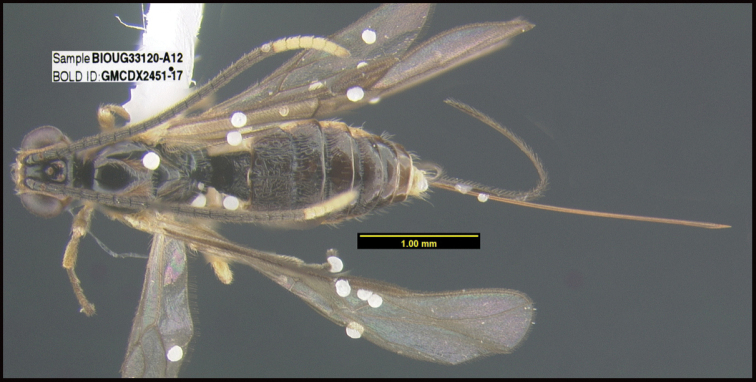
*Braconmanuelzumbadoi*, holotype.

##### Bracon marialuisariasae

Taxon classificationAnimaliaHymenopteraBraconidae

Sharkey sp. nov.

http://zoobank.org/1312E3D0-1A85-4310-B52F-415EF5CA7412

Figure 57

###### Diagnostics.

BOLD:ADA9695. Consensus barcode. GTTTTATATTTTTTGTTTGGGATATGATCTGGTATAATTGGTTTATCAATAAGTTTAATTATTCGTTTAGAATTGGGTATACCAGGTAGATTATTAGGCAATGATCAGATTTATAATAGTATAGTTACTGCTCATGCTTTTGTTATAATTTTTTTTATAGTTATGCCTGTAATATTAGGAGGGTTTGGGAATTGATTAATTCCTTTAATGTTGGGTGCTCCTGATATAGCTTTCCCTCGTATAAATAATATAAGGTTTTGGTTATTAATTCCTTCATTGGTTTTATTATTACTAAGAAGAATTTTAAATGTTGGTGTTGGGACTGGGTGAACTATATACCCTCCTTTATCTTCAAGATTAGGTCATAGTGGTTTGTCAGTTGATTTAGCTATTTTTTCTTTACATTTAGCTGGGGTATCTTCAATTATAGGGGCAATTAATTTTATTACAACTATTTTAAATATACATTTATTTATATTAAAATTAGATCAATTAACTTTGTTAATTTGATCAATTTTTATCACAACTATTTTATTATTATTATCATTACCTGTTTTAGCTGGAGCTATTACTATGTTATTAACTGATCGTAAT---------------------------.

###### Holotype ♂.

Guanacaste, Sector Pailas Dos, PL12-2, 10.7634, -85.335, 824 meters, Malaise trap, 23/i/2014. Depository: CNC.

***Host data*.** None.

***Holotype voucher code*.**BIOUG28680-C06.

###### Paratypes.

None.

###### Etymology.

*Braconmarialuisariasae* is named to honor Maria Luisa Arias for her decades of administrative effort on behalf of Parque Nacional Santa Rosa and now Área de Conservación Guanacaste.

**Figure 57.**
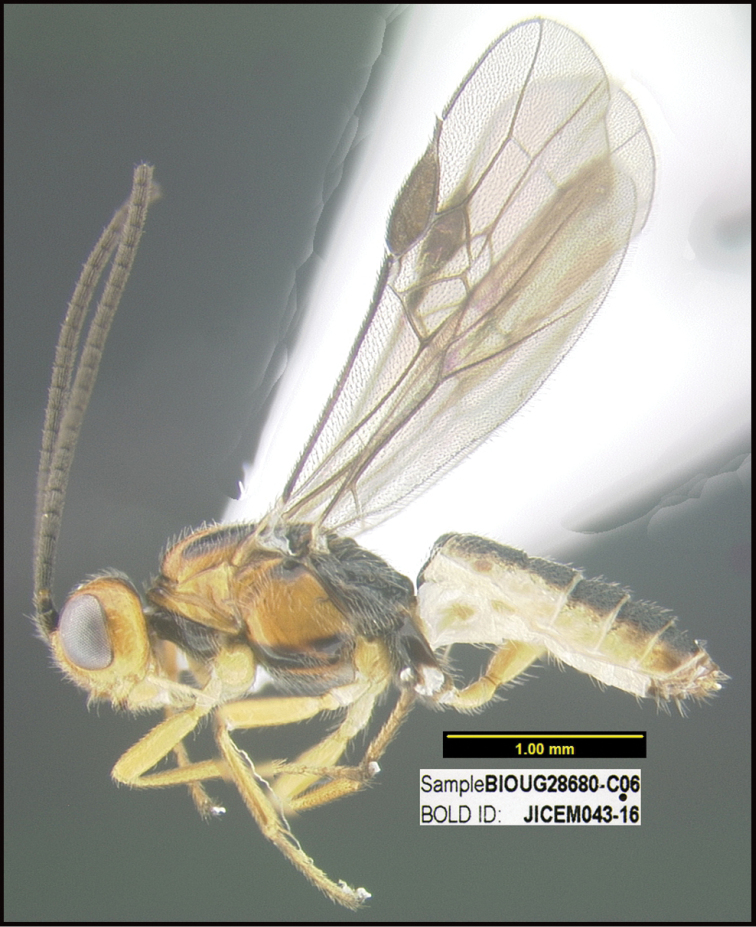
*Braconmarialuisariasae*, holotype.

##### Bracon mariamartachavarriae

Taxon classificationAnimaliaHymenopteraBraconidae

Sharkey sp. nov.

http://zoobank.org/3BE747E2-E77A-4696-A376-C2081E31DDCD

Figure 58

###### Diagnostics.

BOLD:AAV6295. Consensus barcode. AATTTTATATTTCTTATTTGGYATATGAGCAGGTATAGTAGGYYTATCAATAAGTTTAATTATTCGATTAGAATTAGGKATACCTGGRTCATTATTAGGWAATGATCAAATTTATAATAGAATAGTKACAGCTCATGCTYTARTWATAATTTTTTTTATAGTTATACCAGTAATATTAGGAGGTTTTGGTAATTGATTAATTCCTTTAATATTAGGWGCTCCTGATATAGCYTTCCCTCGATTAAATAATATAAGTTTTTGRTTAYTAATYCCTTCATTAATTTTATTAATATTAAGAAGAATTTTAAATGTAGGTGTAGGTACAGGTTGAACTTTATAYCCTCCATTATCYTCAAATTTAGGACATAGAGGDTTATCTGTTGATTTAGCTATTTTTTCTTTACATTTAGCTGGTGTATCTTCAATTATAGGTTCAATAAATTTTATTACTACTATTTTAAATATACATTTATTAATATTAAAATTAGATCAATTAACTTTATTAATTTGATCAATTTTTATTACAACTATTTTATTATTATTATCTTTACCYGTWTTAGCAGGWGCAATTACAATATTATTAACTGATCGAAATTTAAATACTTCATTTTTTGATTTTTCAGGWGGAGGAGAYCCAATTYTATTYCAACATTTATTT. Terminal flagellomeres black, concolorous with basal flagellomeres.

###### Holotype ♀.

Alajuela, Sector Rincon Rain Forest, Selva, 10.92291, -85.31877, 410 meters, caterpillar collection date: 05/viii/2017, wasp eclosion date: 21/viii/2017; nine wasps eclosed from 11 cocoons. Depository: CNC.

***Host data*.** Gregarious parasitoid of *Drephalys* Burns01 (Hesperiidae) feeding on mature leaves of *Vochysiaguatemalensis* (Vochysiaceae).

***Caterpillar and holotype voucher codes*.** 17-SRNP-80872, DHJPAR0061668.

###### Paratypes.

Eight specimens, same data as holotype. Depository: CNC.

###### Other specimens.

There are three specimens from French Guiana (e.g., ASNUR330-11) and one from Argentina (BIOUG13458-C11) in the same BIN, none of which we consider to be conspecific with the Costa Rican specimens. Images on BOLD show that the French Guiana specimens have the terminal flagellomeres yellow. There are no images for the Argentinian specimen, but its locality makes it unlikely to be conspecific.

###### Etymology.

*Braconmariamartachavarriae* is named in honor of Maria Marta Chavarria in recognition of her persistent efforts as co-coordinator of ACG research; intense education efforts to adults, teenagers, and schoolchildren about ACG biology both terrestrial and marine; maintenance and data management of ACG weather data; ACG biopolitics; and rescue of key ACG animals.

**Figure 58.**
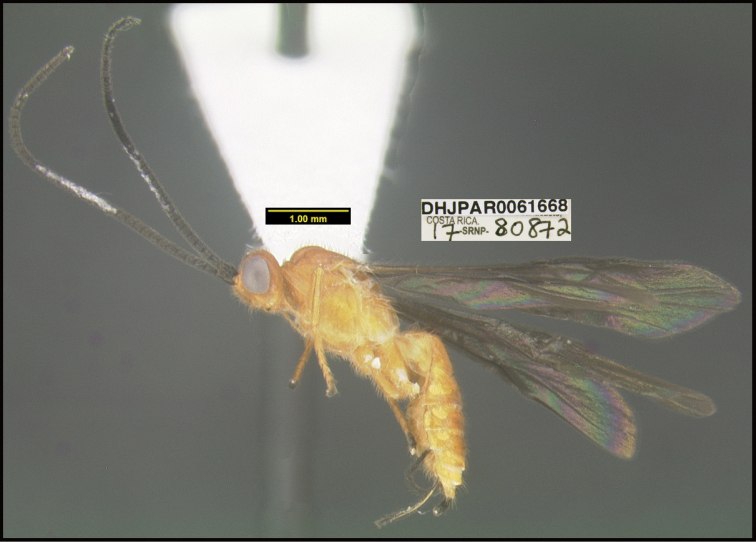
*Braconmariamartachavarriae*, holotype.

##### Bracon mariorivasi

Taxon classificationAnimaliaHymenopteraBraconidae

Sharkey sp. nov.

http://zoobank.org/99C8F6F3-0260-4DBF-AB07-6E7639A34D97

Figure 59

###### Diagnostics.

BOLD:ADA3193. Consensus barcode. GTATTATATTTTTTATTTGGTATATGATCTGGCATTTTAGGTTTATCAATAAGTTTAATAATTCGATTGGAATTGGGGACGCCAGGTAGATTGTTAGGTAATGATCAAATTTATAATAGAATGGTGACAGCTCATGCTTTTGTAATAATTTTTTTTATAGTTATACCAGTTATAGTTGGAGGTTTTGGAAATTGATTATTACCTTTAATATTAGGATCTCCAGATATAGCATTTCCTCGATTAAATAATATAAGGTTTTGACTAATTATTCCTTCTTTAATTCTTTTATTAATAAGAAGGATTTTAAATGTAGGAGTTGGTACGGGATGAACAGTTTATCCTCCTTTATCTTCTTCTTTAGGTCATAGAGGTTTATCTATAGATTTAGCTATTTTTTCTCTTCATATAGCAGGAATTTCTTCAATTTTAGGTGCTATTAATTTTATTTCTACAATTTTTAATATACATTTATATAATTTAAAATTAGATCAATTAGTTTTATTAATTTGGTCTATTTTTATTACTGCTGTTTTATTATTGTTGTCATTACCTGTTTTGGCAGGGGCTATTACAATACTTTTAACAGAT------------------------------------------.

###### Holotype ♂.

Guanacaste, Sector San Cristobal, Estación San Gerardo, 10.8801, -85.389, 575 meters, Malaise trap, 17/ii/2014. Depository: CNC.

***Host data*.** None.

***Holotype voucher code*.**BIOUG28290-E07.

###### Paratypes.

None.

###### Etymology.

*Braconmariorivasi* is named to honor Mario Rivas for his support in ACG being able to purchase forested properties in the formation of ACG, and for the idea of Malaise trapping his pitaya plantations on behalf of BioAlfa.

**Figure 59.**
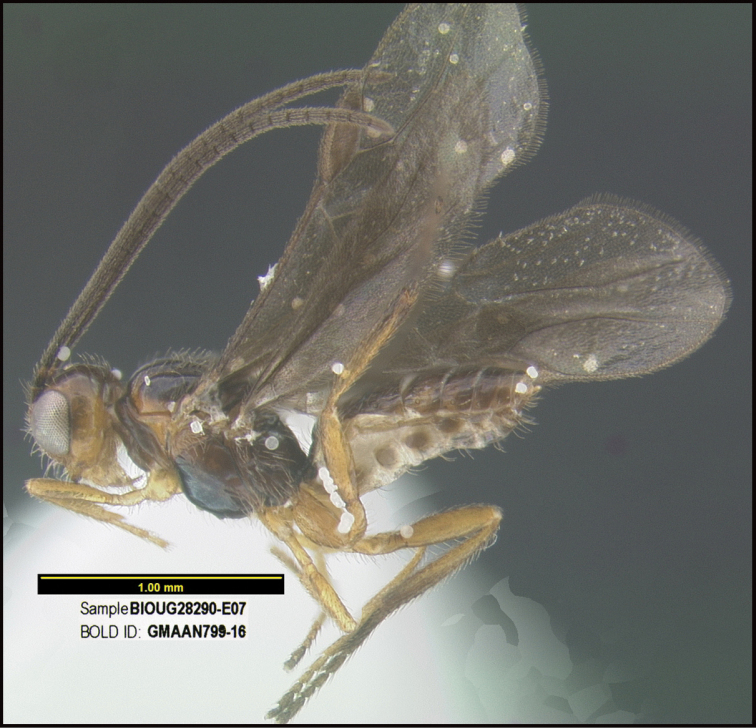
*Braconmariorivasi*, holotype.

##### Bracon melissaespinozae

Taxon classificationAnimaliaHymenopteraBraconidae

Sharkey sp. nov.

http://zoobank.org/B7CB7C8C-4661-49EF-95C4-01591C397952

Figure 60

###### Diagnostics.

BOLD:ACJ4722. Consensus barcode. AATTTTATATTTTTTATTTGGGATATGGTCTGGAATAATTGGTTTATCTATAAGTTTAATTATTCGATTAGAATTGAGAATACCTGGTAGATTATTAGGTAATGATCAAATTTATAATAGTATAGTAACAGCTCATGCTTTAATAATAATTTTTTTTATAGTTATACCAATTATATTAGGAGGTTTTGGGAATTGATTAATTCCTTTAATGTTAGGAGCTCCTGATATAGCTTTCCCACGTTTAAATAATATAAGTTTTTGGTTATTAATCCCCTCTTTAATTTTATTATTATTAAGAAGAATTTTAAATGTAGGGGTAGGAACAGGTTGAACTATATATCCACCTTTATCATCAAATATAGGACATAGAGGTTTATCTGTTGATTTAGCTATTTTTTCTTTACATTTAGCAGGTATTTCTTCAATTATAGGATCAATTAATTTTATTTCAACTATTTTAAATATACATTTAAAAATATTAAAATTAGATCAATTAACATTATTAATTTGATCAATTTTTATTACAACTATTTTATTATTGTTATCATTACCTGTTTTAGCGGGAGCAATTACTATATTATTAACTGATCGAAATTTAAATACTTCTTTTTTTGATTTTTCAGGAGGAGGTGATCCAATTTTATTTCAACATTTATTT.

###### Holotype ♀.

Guanacaste, Sector Santa Maria, Light Site Trail, Volcán Santa Maria, 10.75772, -85.30598, 840 meters, caterpillar collection date: 29/viii/2012, wasp eclosion date: 10/ix/2012, 16 wasps eclosed. Depository: CNC.

***Host data*.** Gregarious parasitoid of unknown caterpillar in leaf nest on *Smilaxspinosa* (Smilacaeae).

***Caterpillar and holotype voucher codes*.** 12-SRNP-13169, DHJPAR0051204.

###### Paratypes.

15 specimens, same data as holotype, still labelled with 12-SRNP-13169. Depository: CNC.

###### Etymology.

*Braconmelissaespinozae* is named in honor of Melissa Espinoza’s proactive and growing abilities as a science reporter for all things ACG for the ACG web site (www.acguanacaste.ac.cr).

**Figure 60.**
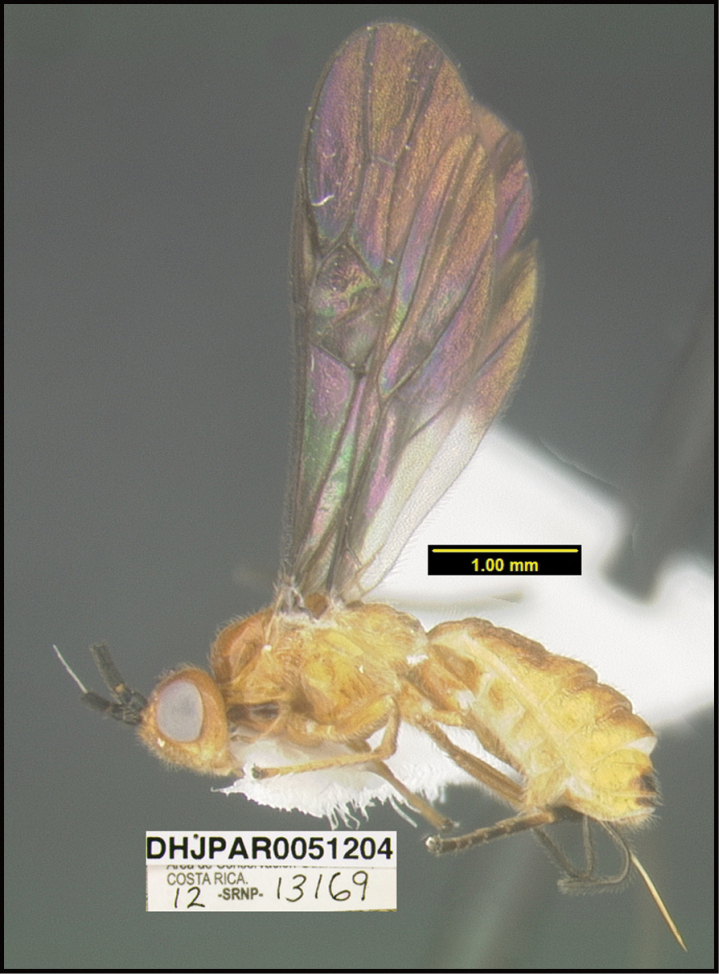
*Braconmelissaespinozae*, holotype.

##### Bracon nelsonzamorai

Taxon classificationAnimaliaHymenopteraBraconidae

Sharkey sp. nov.

http://zoobank.org/26E8A237-1A2C-46CC-A876-8062E4525228

Figure 61

###### Diagnostics.

BOLD:ADB4596. Consensus barcode. ATTTTATATTTTTTATTTGGGATATGAGCTGGGATAATTGGTTTATCAATAAGTTTAATTATTCGATTAGAATTAGGGATACCTGGAAGTTTATTAGGTAATGACCAAATTTATAATAGTATAGTTACTGCTCATGCTTTTATTATAATTTTTTTTATAGTTATACCTGTAATATTAGGAGGATTTGGAAATTGATTAATTCCTTTAATATTAGGAGCTCCTGATATAGCTTTCCCTCGTTTAAATAATATAAGATTTTGATTATTAATCCCTTCTTTAATTTTATTAATTTTGAGAAGAATTTTAAATATTGGGGTAGGCACAGGCTGAACTATATACCCCCCTTTATCGTCTAGATTAGGTCATAGAGGAGTATCTGTTGATTTAGCAATTTTTTCATTACATTTAGCAGGAATCTCTTCTATTATAGGAGCAATAAATTTTATTACCACTATTTTAAATATACATTTATTAATATTAAAATTAGACCAATTAACCTTATTAATCTGATCAATTTTTATTACAACTATTTTATTATTACTATCTTTACCAGTATTAGCTGGAGCTATCACTATACTATTAACAGATCGAAACCTAAAC---------------------------------.

###### Holotype ♂.

Guanacaste, Sector Cacao, Derrumbe, 10.9292, -85.4643, 1220 meters, Malaise trap, 22/v/2014. Depository: CNC.

***Host data*.** None.

***Holotype voucher code*.**BIOUG29002-G10.

###### Paratypes.

None.

###### Etymology.

*Braconnelsonzamorai* is named to honor Nelson Zamora for his many years of dedicated curatorial and taxonomic efforts for the growth of the former INBio plant collection, now in the Museo Nacional de Costa Rica, and his furthering the BioAlfa program to DNA barcode all of Costa Rican eukaryote biodiversity, with an emphasis on all of Costa Rica’s plant species.

**Figure 61.**
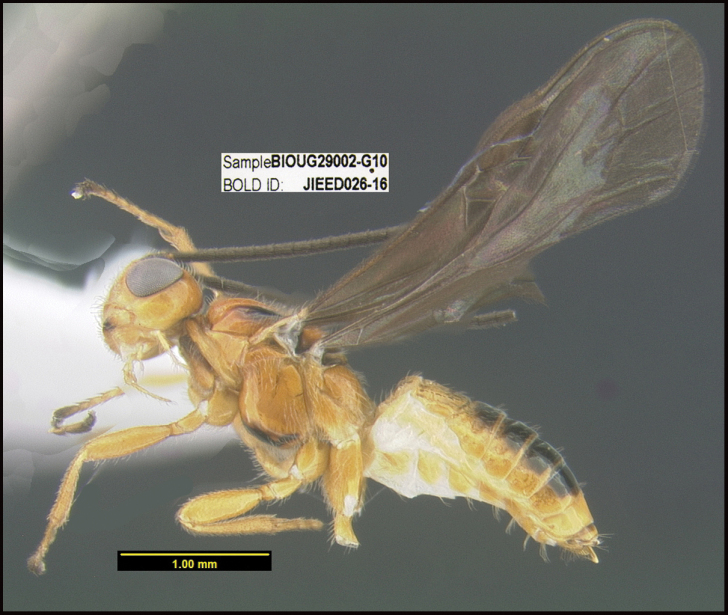
*Braconnelsonzamorai*, holotype.

##### Bracon nicklaphami

Taxon classificationAnimaliaHymenopteraBraconidae

Sharkey sp. nov.

http://zoobank.org/18DDD25E-1899-4827-AD86-FC3CA8767925

Figure 62

###### Diagnostics.

BOLD:ACP6341. Consensus barcode. AATTTTATATTTTTTATTTGGTATATGATCTGGTATAATTGGTTTATCAATAAGATTAATTATTCGTTTAGAATTAGGTATACCTGGAAGATTATTAAGAAATGATCAAATTTATAATAGAATAGTCACAGCCCATGCTTTAGTAATGATTTTTTTTATAGTTATACCAATTATATTAGGAGGTTTTGGAAATTGGTTAATTCCTTTAATATTAGGTGCTCCTGATATAGCTTTCCCTCGAATAAATAATATAAGATTTTGATTATTAATTCCTTCTTTAATTTTGTTATTATTAAGAAGAATTTTAAATGTAGGTGTAGGAACAGGATGGACATTATATCCTCCTTTATCTTCAAATTTAGGTCATAGAGGTTTATCTGTGGATTTATCTATTTTTTCTATTCATTTAGCTGGAATTTCATCAATTATAGGTTCATTAAATTTTATTACTACAATTTTAAATATACATTTATTAATATTAAAATTAGATCAATTAACTTTATTAATTTGATCAATTTTTATTACAACTATTTTATTATTATTATCTTTACCTGTTTTGGCAGGAGCTATTACTATATTATTAACTGATCGTAATTTAAATACTTCATTTTTTGATTTTTCAGGAGGTGGGGATCCAATTTTATTTCAACATTTATTT.

###### Holotype ♀.

Alajuela, Sector Rincon Rain Forest, Palomo, 10.96187, -85.28045, 96 meters, caterpillar collection date: 24/iv/2014, wasp eclosion date: 4/v/2014. Depository: CNC.

***Host data*.***Damasimmacula* (Hesperiidae) feeding on *Geonomacongesta* (Arecaceae).

***Holotype voucher code*.**DHJPAR0055350.

###### Paratypes.

None.

###### Etymology.

*Braconnicklaphami* is named in honor of Nick Lapham’s long-appreciated contributions to publicity for ACG and GDFCF, and now BioAlfa.

**Figure 62.**
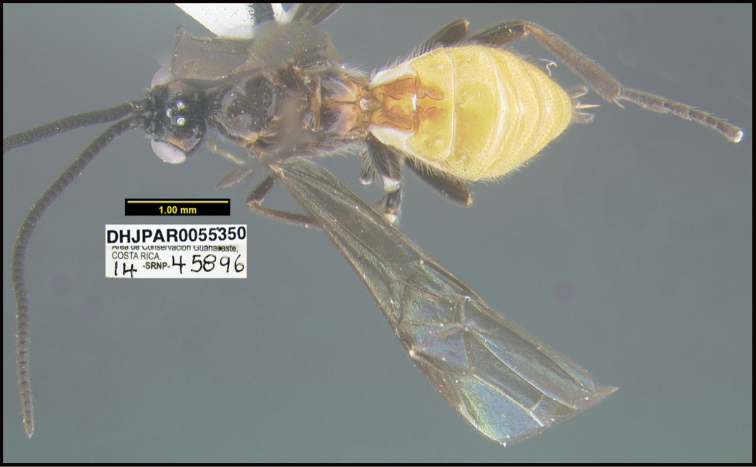
*Braconnicklaphami*, holotype.

##### Bracon ninamasisae

Taxon classificationAnimaliaHymenopteraBraconidae

Sharkey sp. nov.

http://zoobank.org/B622D924-8955-4563-8F87-0BAB42A782CE

Figure 63

###### Diagnostics.

BOLD:ADA4862. Consensus barcode. ATTTTATATTTTTTATTTGGAATATGATCAGGAATAATTGGTCTATCAATAAGATTAATTATTCGGTTAGAATTAAGAATACCTGGTAGATTATTAGGTAATGACCAAATTTATAATAGTATAGTAACAGCTCATGCTTTAATAATAATTTTTTTTATAGTTATACCAATTATATTAGGAGGATTTGGAAATTGATTAATTCCTTTAATATTAGGAGCTCCTGATATAGCTTTCCCACGTTTAAATAATATAAGTTTTTGACTATTAATTCCTTCTTTAATTTTATTATTATTAAGAAGAATTTTGAATATAGGAGTAGGTACAGGTTGAACTTTATACCCACCTTTATCTTTAAGAATTGGTCATAGAGGTTTATCTGTTGATTTAGCTATTTTTTCTTTACATTTAGCAGGTATTTCTTCAATTATAGGATCAATTAATTTTATTTCAACTATTTTAAATATACATTTAAAATCATTAAAATTAGATCAATTAACATTATTAATTTGATCAATTTTTATTACAACTATTTTATTATTATTATCATTACCTGTTTTAGCAGGAGCAATTACTATA---------------------------------------------------------.

###### Holotype ♀.

Guanacaste, Sector San Cristobal, Estación San Gerardo, 10.8801, -85.389, 575 meters, Malaise trap, 9/xii/2013. Depository: CNC.

***Host data*.** None.

***Holotype voucher code*.**BIOUG28011-B01.

###### Paratypes.

None.

###### Etymology.

*Braconninamasisae* is named to honor Nina Masis for her cheerful participation in the lives and dedication of the Masis + Boshart family as it carried out its vital role in the growth and management of the Guanacaste Dry Forest Conservation Fund and Área de Conservación Guanacaste.

**Figure 63.**
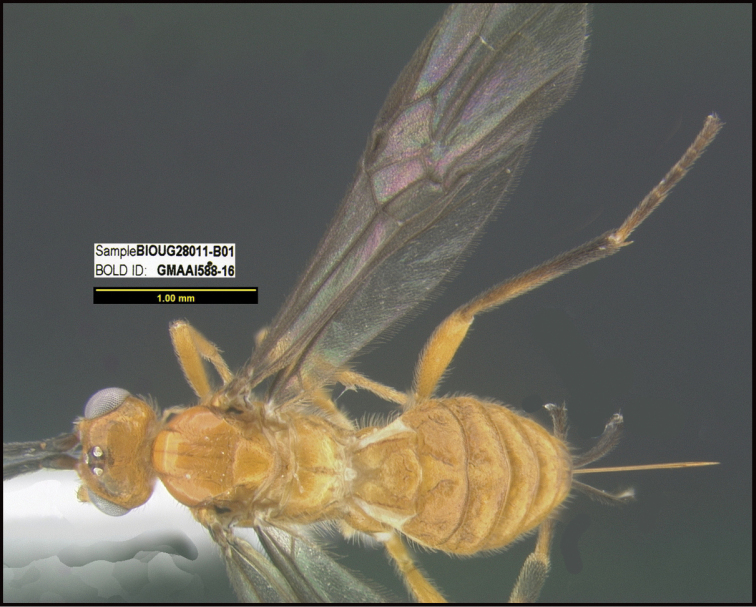
*Braconninamasisae*, holotype.

##### Bracon oliverwalshi

Taxon classificationAnimaliaHymenopteraBraconidae

Sharkey sp. nov.

http://zoobank.org/82D85FD6-06AA-441F-A9CB-F04A7D30D0B6

Figure 64

###### Diagnostics.

BOLD:ABV9910. Consensus barcode. AATTCTTTATTTTTTATTTGGTATATGAGCAGGAATATTAGGTTTATCAATAAGATTAATTATTCGTTTAGAACTAGGAATACCAGGTAGATTATTAGGGAATGACCAAATTTATAATAGAATAGTTACAGCTCATGCTTTTGTTATAATTTTTTTTATAGTTATACCAGTAATAGTTGGAGGGTTTGGGAATTGATTATTACCTTTAATATTAAGGGCCCCTGATATAGCTTTCCCACGTTTAAATAATATAAGATTTTGGTTATTAATTCCTTCTTTATTTTTATTATTAATAAGAAGAGTATTAAATGTAGGTGTTGGTACTGGATGAACAATATATCCTCCTTTGTCTTCTTCTTTAGGTCATAGGGGTATATCAGTTGATTTAGCTATTTTTTCTTTACATATTGCGGGTATTTCATCAATTTTAGGGGCTATAAATTTTATTTCAACTATTTTTAATATACATTTATTAACTTTAAAATTAGATCAATTAACTTTATTTATTTGATCAATTTTTATTACAACTTTGTTATTATTATTATCTTTACCAGTATTAGCTGGGGCTATTACCATATTATTAACTGATCGAAATTTAAATACTTCTTTTTTTGATTTTTCTGGTGGAGGTGACCCAATTTTATTTCAACATTTATTT.

###### Holotype ♀.

Guanacaste, Sector Cacao, Sendero Cima, 10.933, -85.457, 1460 meters, Malaise trap, 9/iii/2009. Depository: CNC.

***Host data*.** None.

***Holotype voucher code*.**DHJPAR0046065.

###### Paratypes.

None.

###### Etymology.

*Braconoliverwalshi* is named in honor of Oliver Walsh, son-in-law of Karen Sharkey.

**Figure 64.**
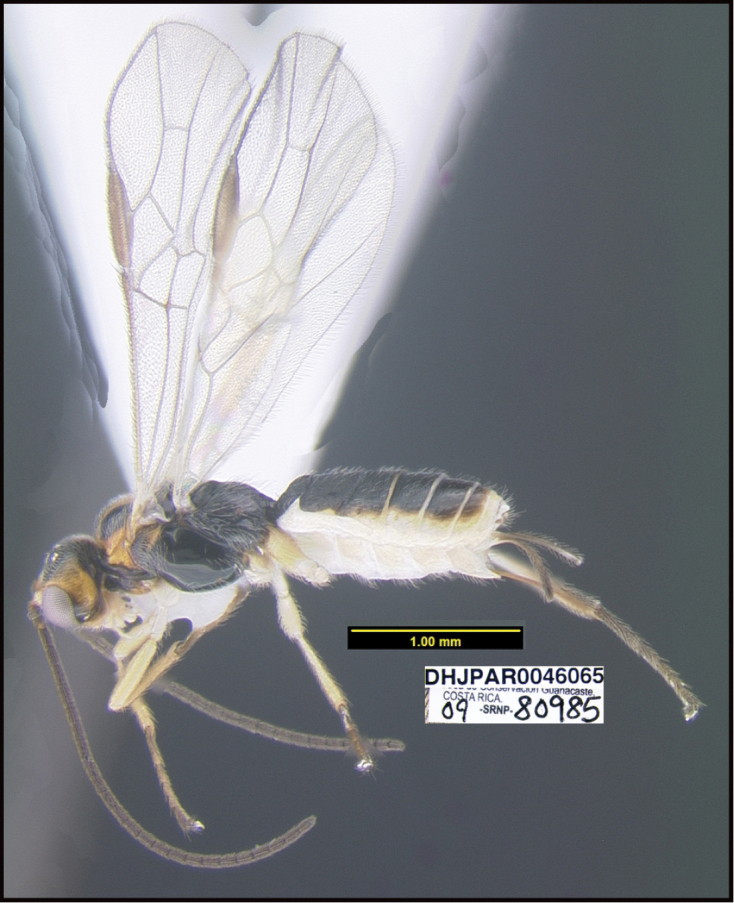
*Braconoliverwalshi*, holotype.

##### Bracon paulamarinae

Taxon classificationAnimaliaHymenopteraBraconidae

Sharkey sp. nov.

http://zoobank.org/941A0A53-E106-41AC-A724-5C14844F3DBC

Figure 65

###### Diagnostics.

BOLD:ADB2848. Consensus barcode. GTTTTATATTTTTTATTTGGTATATGGGCAGGAATAGTAGGATTATCAATAAGATTAATTATTCGATTAGAATTAGGTATGCCTGGCAGATTACTGGGGAATGATCAAATTTATAATAGGATAGTAACAGCTCATGCTTTTGTAATAATTTTTTTTATAGTAATACCAGTTATATTAGGGGGGTTTGGCAATTGATTAATTCCTTTAATATTGGGTGCTCCAGATATGGCTTTCCCTCGATTAAATAATATAAGATTTTGGTTATTAATTCCTTCTTTAATTTTATTATTATTAAGAAGAATTTTAAATGTAGGGGTAGGAACTGGTTGAACAATATACCCTCCTTTATCTTCTAATTTAGGACATAGGGGATTGTCAGTTGATTTAGCTATTTTTTCTCTACATTTAGCTGGGGCATCTTCAATTATAGGGTCTATAAATTTTATTACTACAATTTTAAATATACATTTAATAATATTAAAATTAGATCAATTAACTTTATTAATTTGGTCAATTTTTATTACTACTATTTTATTATTATTATCTTTACCAGTTTTAGCTGGAGCTATTACTATATTATTAACT------------------------------------------.

###### Holotype ♀.

Guanacaste, Sector Cacao, Derrumbe, 10.9292, -85.4643, 1220 meters, Malaise trap, 9/vii/2015. Depository: CNC.

***Host data*.** None.

***Holotype voucher code*.**BIOUG28824-F05.

###### Paratypes.

None.

###### Etymology.

*Braconpaulamarinae* is named to honor Paula Marin for her cheerful participation in the lives and nutritional health of the Marin and Romero families as they have carried out its vital role in the growth and management of the Guanacaste Dry Forest Conservation Fund and Área de Conservación Guanacaste.

**Figure 65.**
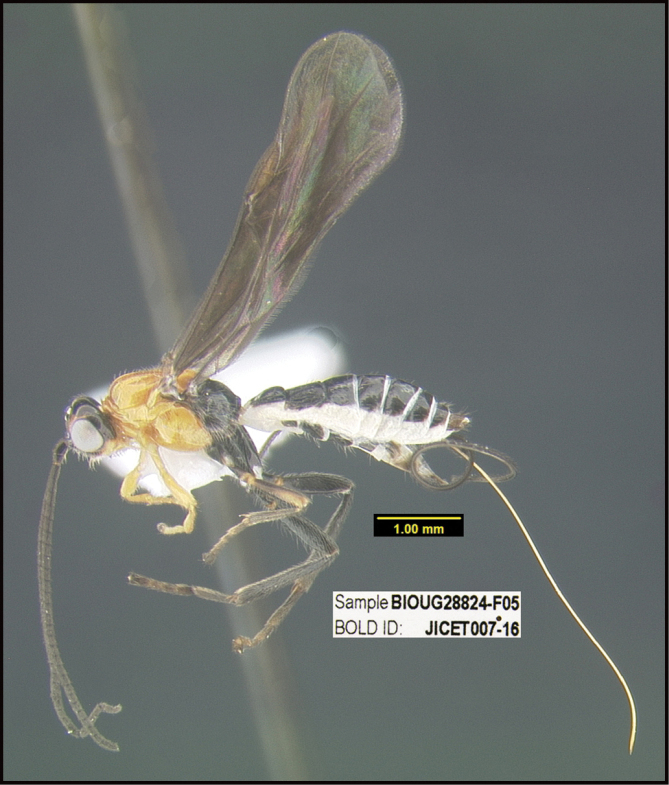
*Braconpaulamarinae*, holotype.

##### Bracon rafamoralesi

Taxon classificationAnimaliaHymenopteraBraconidae

Sharkey sp. nov.

http://zoobank.org/83AEB11C-410C-420E-BC2E-F7BDE89794F0

Figure 66

###### Diagnostics.

BOLD:ADB5058. Consensus barcode. ATTTTATATTTTTTATTTGGTATATGATCAGGTATAGTTGGTTTATCAATAAGGTTAATTATTCGATTAGAATTAGGTTTACCTGGGAGTTTATTAGGTAATGATCAAATTTATAATAGAATAGTTACTGCTCATGCTTTTATTATAATTTTTTTTATAGTTATACCTGTTATATTAGGGGGGTTTGGTAATTGATTAATTCCTTTAATATTAGGAGCCCCAGATATAGCTTTCCCTCGTTTAAATAATATAAGATTTTGGTTATTATTTCCTTCTTTAATTTTATTATTATTGAGAAGAATTTTAAATGTTGGTGTAGGCACTGGTTGAACAATATATCCTCCTTTATCATCTAGGTTAGGTCATAGAGGTTTATCTGTTGATTTAGCAATTTTTTCTTTACATTTAGCTGGGGTTTCTTCTATTTTAGGTTCAATAAATTTTATTACAACAATTTTAAACATGCATTTATTAATATTAAAGTTAGATCAATTGACTTTATTAATTTGGTCAATTTTTATTACAACTATTTTATTATTATTGTCTTTACCTGTTTTGGCTGGTGCTATTACTATATTATTAACAGAT------------------------------------------------.

###### Holotype ♂.

Guanacaste, Sector Pailas Dos, PL12-3, 10.7631, -85.3344, 820 meters, Malaise trap, 19/xii/2013. Depository: CNC.

***Host data*.** None.

***Holotype voucher code*.**BIOUG29288-B07.

###### Paratypes.

None.

###### Etymology.

*Braconrafamoralesi* is named to honor Rafa Morales, an essential member of the fauna of Sector Orosi of ACG, for his dedication to exploration and management for conservation of ACG Sector Orosi and Estación Biologica Maritza within it.

**Figure 66.**
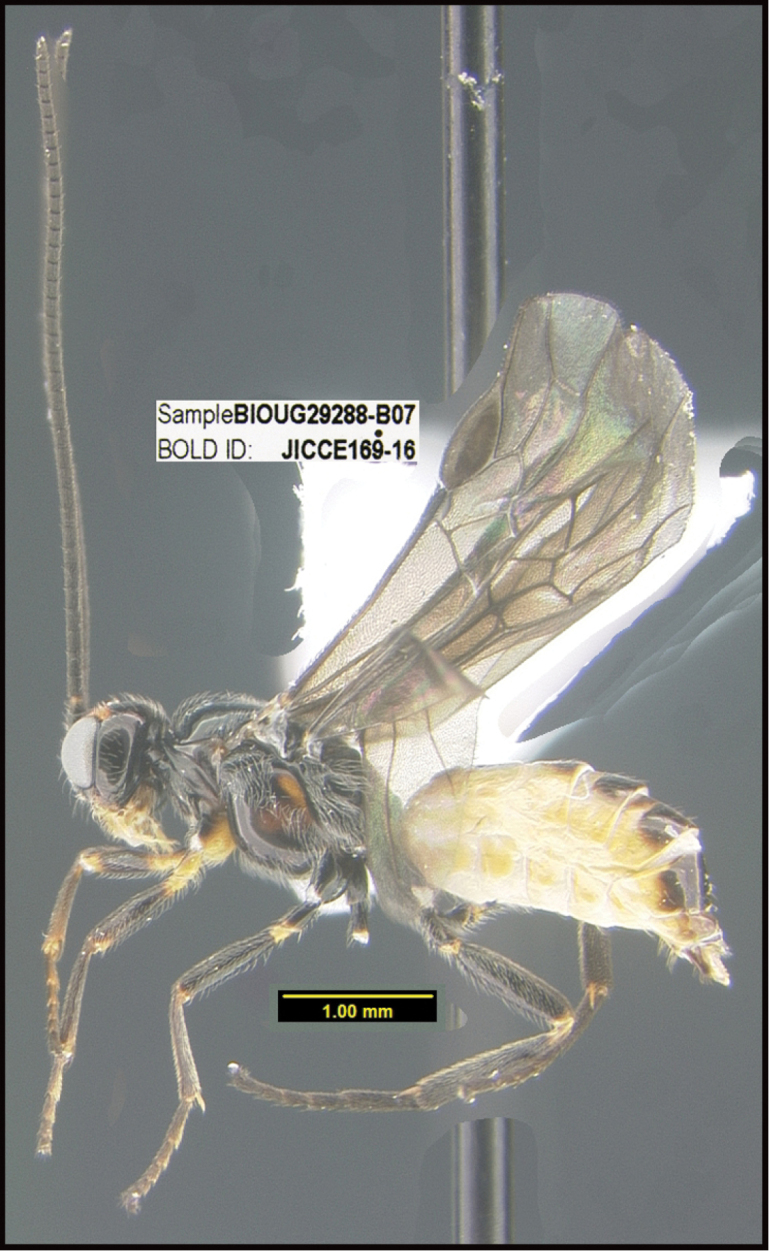
*Bracnrafamoralesi*, holotype.

##### Bracon robertofernandezi

Taxon classificationAnimaliaHymenopteraBraconidae

Sharkey sp. nov.

http://zoobank.org/E73B1115-BC62-41C6-B5CE-2F8D1F70FE61

Figure 67

###### Diagnostics.

BOLD:ADC8245. Consensus barcode. GTTTTATATTTTTTATTTGGGATATGAGCTGGCATGCTGGGGTTATCAATAAGATTGATTATTCGATTAGAATTAGGCATACCAGGAAGAATATTAGGTAATGATCAAATTTATAATAGTATAGTTACTGCGCATGCTTTTATTATAATTTTTTTTATGGTTATACCTATTATAATTGGTGGGTTTGGAAATTGATTACTACCATTAATATTAGGGGCCCCAGATATGGCATTTCCTCGATTAAATAATATAAGATTTTGATTAATTATTCCTGCTTTAATTATATTATTAATAAGTAGAATTTTAAATGTTGGTGTAGGAACTGGTTGAACTGTTTATCCTCCTTTATCTTCTTCATTGGGGCATAGGGGGATATCTGTTGATTTAGCTATTTTTTCATTACATATAGCTGGAATTTCTTCAATTTTAGGTGCAATTAATTTTATTACTACTATTTTTAATATACAACTATATATTTTAAAATTAGACCAATTAACTTTATTAATTTGATCAATTTTTATCACAGTTGTCTTGTTATTATTATCATTACCAGTTTTAGCAGGAGCTATTACTATATTATTAACTGATCGT------------------------.

###### Holotype ♀.

Guanacaste, Sector Pailas Dos, PL12-2, 10.7634, -85.335, 824 meters, Malaise trap, 30/x/2014. Depository: CNC.

***Host data*.** None.

***Holotype voucher code*.**BIOUG30497-D09.

###### Paratypes.

None.

###### Etymology.

*Braconrobertofernandezi* is named to honor Roberto Fernandez for his previous efforts on behalf of environmental planning for ICE, the National Electric Company, and now his work for BioAlfa the program to DNA barcode all of Costa Rican eukaryote biodiversity.

**Figure 67.**
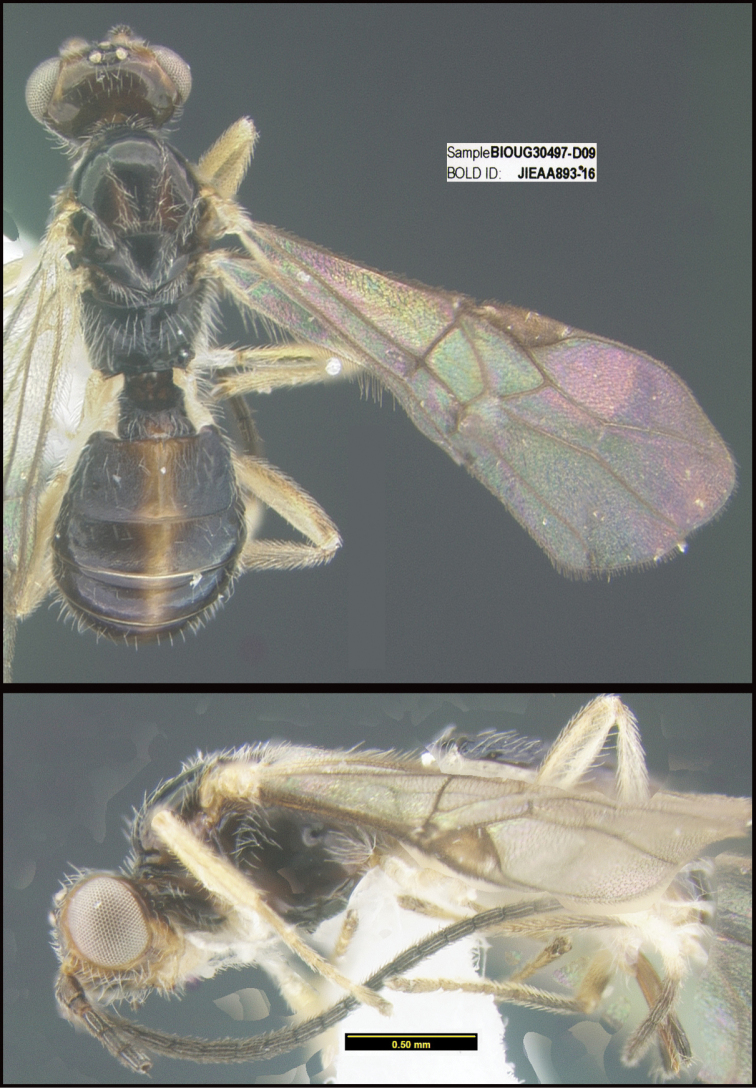
*Braconrobertofernandezi*, holotype.

##### Bracon rogerblancoi

Taxon classificationAnimaliaHymenopteraBraconidae

Sharkey sp. nov.

http://zoobank.org/25BCDF32-AD39-4A13-A507-C1AEB255BC34

Figures 68 , 69

###### Diagnostics.

BOLD:AAA5369. Consensus barcode. AATTTTATATTTTTTATTTGGTTTATGATCTGGTATAGTTGGATTATCTATAAGTTTAATTATTCGTTTAGAATTAGGTATACCTGGAAGATTATTAAGTAATGACCAAATTTATAATAGAATAGTTACAGCTCATGCTTTAGTAATAATTTTTTTTATAGTTATACCAATTATATTAGGAGGATTTGGTAATTGATTAATTCCTTTAATATTAGGTGCTCCTGATATAGCTTTCCCTCGATTAAATAATATAAGATTTTGATTATTAATTCCTTCTTTAATTTTATTATTATTAAGAAGAATTTTAAATGTTGGAGTAGGAACAGGTTGAACTTTATACCCTCCTTTATCTTCAAATTTAGGTCATAATGGTATATCTGTAGATTTATCTATTTTTTCTTTACATTTAGCTGGTATTTCATCAATTATAGGTTCATTAAATTTTATTACTACTATTTTAAATATACATTTATTAACATTAAAATTAGATCAATTAACTTTATTAATTTGATCAATTTTTATTACAACTATTTTATTATTATTATCTTTACCTGTTTTAGCAGGRGCTATTACTATATTATTAACTGATCGTAATTTAAATACTTCATTTTTTGATTTTTCAGGAGGTGGAGACCCAATTTTATTTCAACATTTATTT.

###### Holotype ♀.

Alajuela, Sector Rincon Rain Forest, Palomo, 10.96187, -85.28045, 96 meters, caterpillar collection date: 05/vii/2012, wasp eclosion date: 17/vii/2012, 8 wasps eclosed. Depository: CNC.

***Host data*.** Gregarious parasitoid of *Leremaliris* (Hesperiidae) feeding on leaves of *Paspalumfasciculatum* (Poaceae) (Fig. 69).

***Caterpillar and holotype voucher codes*.** 12-SRNP-68015, DHJPAR0049816.

###### Paratypes.

Hosts = *Synaptesalenus*, *Parphorusstorax*, *Morys* lydeDHJ01, *Leremaliris* (all Hesperiidae, Hesperiinae). 11 specimens, same data as holotype and DHJPAR0029034, DHJPAR0052239, DHJPAR0051799, DHJPAR0029037, DHJPAR0051186, DHJPAR0029035, DHJPAR0049816, DHJPAR0058162, DHJPAR0058165, DHJPAR0058166. Depository: CNC.

###### Etymology.

*Braconrogerblancoi* is named to honor Sr. Roger Blanco, former parataxonomist, and Research Program Coordinator, and now the Subdirector of ACG, in recognition of three decades of intense support and facilitation of ACG germination and growth.

**Figure 68.**
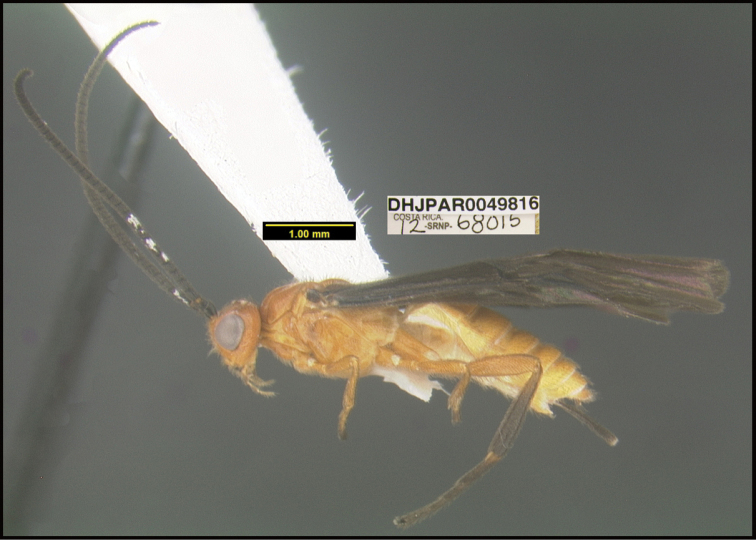
*Braconrogerblancoi*, holotype.

**Figure 69.**
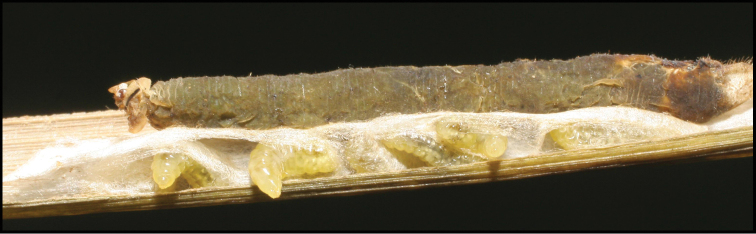
*Braconrogerblancoi* (DHJPAR0029037) mass of white cocoons spun by the wasp larvae below their roof-in-common and end-to-end cocoons adjacent to the ultimate instar of a *Morys* lydeDHJ01 (Hesperiidae, Hesperiinae) caterpillar cadaver in its rolled grass leaf nest (06-SRNP-43580); prepupal larvae exposed by tearing away one wall of the leaf-silk caterpillar nest.

##### Bracon ronaldzunigai

Taxon classificationAnimaliaHymenopteraBraconidae

Sharkey sp. nov.

http://zoobank.org/96760F41-A15A-4D36-A47A-3548DB1E9DC0

Figure 70

###### Diagnostics.

BOLD:ACR7910. Consensus barcode. GTATTATATTTTTTATTTGGAATATGGGCAGGTATATTAGGTTTATCAATAAGTATAATTATTCGTTTAGAATTAGGYAYAGTTGGAAGTTTATTAATAAATGATCAAATTTATAATAGTATTGTTACTTCTCATGCTTTTGTAATAATTTTTTTTATAGTTATGCCTGTAATAGTTGGGGGTTTTGGAAATTGATTATTACCTTTAATGTTAGGATCTCCTGATATAGCTTTTCCTCGGTTAAATAATATAAGATTTTGATTACTTATTCCTTCTTTAATTATATTATTGTTTAGTAGAGTTTTAAATATTGGTGTGGGGACTGGGTGAACTATATATCCTCCTTTATCTTCTTTATTAGGTCATGGTGGATTGTCAGTAGATTTAGCTATTTTTTCTTTACATATTGCAGGAGTTTCATCAATTTTAGGGGCTATTAATTTTATTTCAACTATTTTAAATATATTTTTATATACTTTAAAATTAGATCAATTAACTTTGTTGATTTGATCAATTTTTATTACAGCTATTTTGTTATTATTATCTTTACCTGTTTTAGCAGGCGCTATTACAATACTATTAACAGATCGAAATTTAAAT.

###### Holotype ♀.

Guanacaste, Sector San Cristobal, Estación San Gerardo, 10.8801, -85.389, 575 meters, Malaise trap, 19/v/2014. Depository: CNC.

***Host data*.** None.

***Holotype voucher code*.**BIOUG25142-A09.

###### Paratype.

BIOUG18005-A01. Depository: CNC.

###### Etymology.

*Braconronaldzunigai* is named to honor Ronald Zuniga for his many years of dedicated curatorial and taxonomic efforts for the advancement of the former INBio arthropod collection, now in the Museo Nacional de Costa Rica, and his furthering the BioAlfa program to DNA barcode all of Costa Rican eukaryote biodiversity, with an emphasis on Hymenoptera.

**Figure 70.**
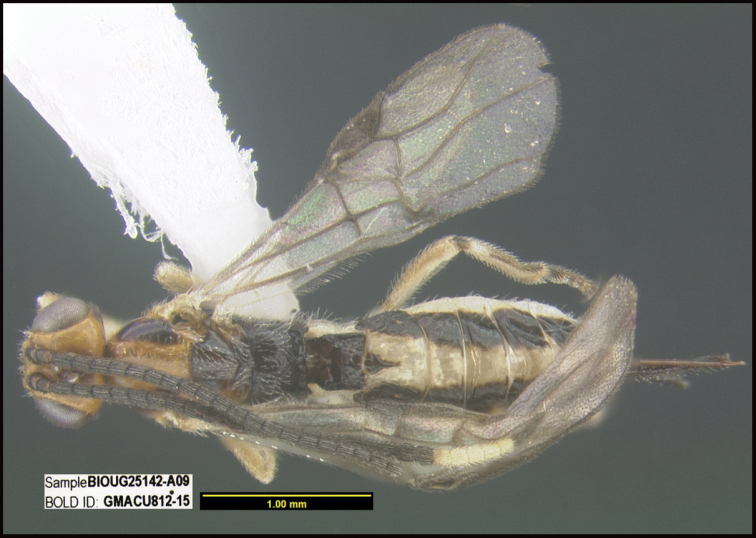
*Braconronaldzunigai*, holotype.

##### Bracon sigifredomarini

Taxon classificationAnimaliaHymenopteraBraconidae

Sharkey sp. nov.

http://zoobank.org/8475D396-33AA-4B22-8250-E1D185CF4D70

Figure 71

###### Diagnostics.

BOLD:ACG5126. Consensus barcode. TATTTTATATTTTTTATTTGGAATATGAGCTGGGATATTAGGTATATCTATAAGATTAATTATTCGTTTAGAATTAGGGGTACCAGGTAGCTTATTAGGAAATGATCAAATTTATAATAGGATAGTTACTGCTCATGCTTTTGTAATAATTTTTTTTATAGTTATACCAATTATAATTGGAGGATTTGGAAATTGATTATTACCTTTAATATTAGGGGCTCCTGATATAGCATTCCCTCGTTTAAATAATATAAGGTTTTGATTGATTATTCCTTCTTTAATTTTATTATTAATAAGAAGAATTTTAAATGTAGGAGTTGGTACTGGTTGAACAGTTTACCCTCCTTTATCTTCATCTTTAGGACATAGAGGGCTATCTGTAGATTTAGCAATTTTTTCTTTACATATAGCTGGTATTTCATCTATTTTAGGTGCAATTAATTTTATTACAACTATTTTAAATATACATTTATTTATTTTGAAATTAGATCAATTAACTTTATTAATTTGATCAATTTTTATTACAGTTATTTTATTATTATTATCTTTACCAGTTTTAGCTGGGGCAATTACTATATTATTAACTGACCGAAATTTAAATACATCTTTTTTTGATTTTTCTGGAGGAGGGGATCCAATTTTATTCCAACATTTATTT.

###### Holotype ♂.

Guanacaste, Sector Santa Rosa, Bosque San Emilio, 10.8438, -85.6138, 300 meters, Malaise trap, 30/vii/2012. Depository: CNC.

***Host data*.** None.

***Holotype voucher code*.** BIOUG05422-G01.

###### Paratypes.

None.

###### Etymology.

*Braconsigifredomarini* is named to honor Sigifredo Marin for his decades of managerial soul and energy and effort on behalf of Parque Nacional Santa Rosa, Área de Conservación Guanacaste, and then the Guanacaste Dry Forest Conservation Fund projects in and around ACG.

**Figure 71.**
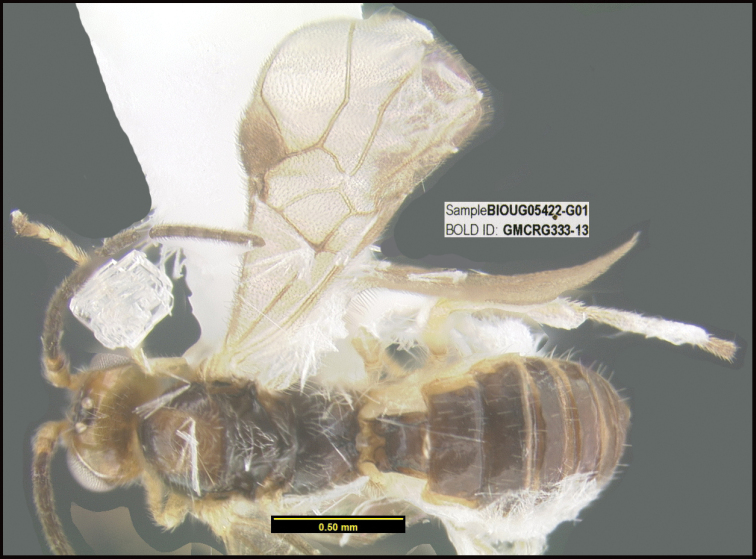
*Braconsigifredomarini*, holotype.

##### Bracon tihisiaboshartae

Taxon classificationAnimaliaHymenopteraBraconidae

Sharkey sp. nov.

http://zoobank.org/4877A43F-55D1-4003-80FA-A1E1216E0B7F

Figures 72 , 73

###### Diagnostics.

BOLD:AAA5367. Consensus barcode. TGTATTATATTTTTTATTTGGAATATGAGCYGGAATAATTGGTTTATCAATAAGTTTAATTATTCGTTTAGAATTAGGRATACCAGGTAGTTTAYTAGGTAATGATCAAATTTATAATAGTATAGTTACAGCKCATGCTTTTATTATAATTTTTTTTATAGTTATACCAGTAATATTAGGAGGWTTTGGTAATTGATTAGTTCCTTTAATATTAGGTGCTCCTGATATAGCTTTYCCTCGAATAAATAATATAAGATTTTGATTATTAATTCCTTCATTAATTTTATTATTATTAAGAAGAATTTTAAATGTTGGTGTAGGRACAGGCTGAACTATTTATCCTCCTTTATCTTCTATAATAGGTCATAGAGGWATATCTGTRGATTTATCTATTTTYTCTTTACATTTAGCTGGTATTTCTTCTATTATAGGATCGATTAATTTTATTACAACAATTTTAAATATACATTTATTAATATTAAAATTAGATCAATTAACTTTATTTATTTGATCAATTTTTATTACAACTATTTTATTATTATTATCTTTACCTGTATTAGCAGGAGCTATTACTATAYTATTAACTGATCGAAATTTWAATACTTCATTTTTTGATTTTTCTGGAGGTGGGGATCCAATTYTATTTCAACATTTATTT. *Bracontihisiaboshartae* and *B.alejandromasisi* occupy the same BIN. *Bracontihisiaboshartae* can be differentiated from *B.alejandromasisi* by the color of the metasomal terga: entirely yellow in *B.tihisiaboshartae* and partly black in *B.alejandromasisi*. The two can also be differentiated based on their host caterpillars and style of cocoons, see note below.

###### Holotype ♀.

Guanacaste, Sector Mundo Nuevo, Quebrada Tibio Perla, 10.76261, -85.42979, 330 meters, caterpillar collection date: 17/xi/2013, wasp eclosion date: 1/xii/2013, 26 wasps eclosed. Depository: CNC.

**Figure 72.**
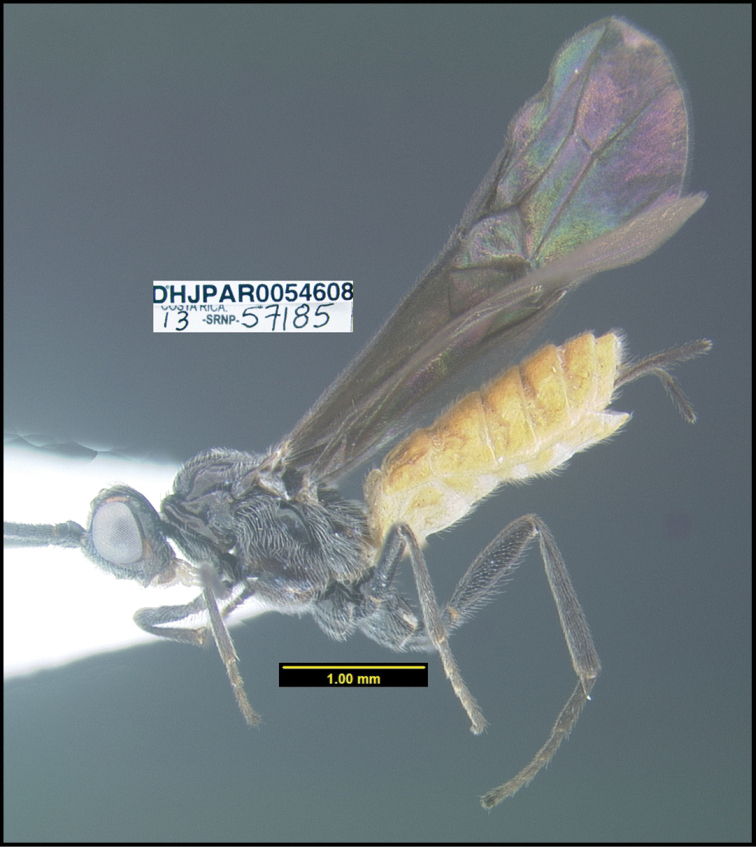
*Bracontihisiaboshartae*, holotype.

***Host data*.** Gregarious parasitoid of *Memphisboisduvali* (Nymphalidae) feeding on *Mespilodaphneveraguensis* (Lauraceae) (Fig. 72)

***Caterpillar and holotype voucher codes*.** 13-SRNP-57185, DHJPAR0054608.

###### Paratype.

Host = *Memphispithyusa*: DHJPAR0040072. Depository: CNC.

###### Note.

**Figure 73.**
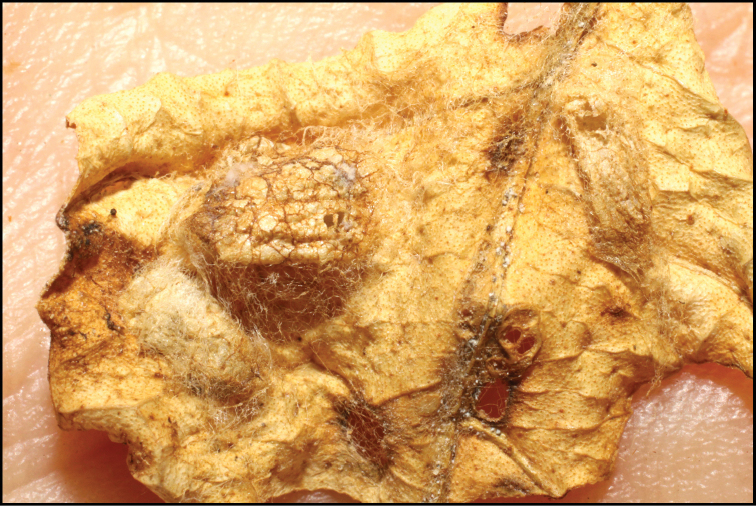
*Bracontihisiaboshartae* (09-SRNP-56390-DHJ474143.jpg) white-golden cocoons piled together in two masses on the leaf, rather than filling the nest cavity as the case with *Braconalejandromasisi*; wasp exit hole is in the mass on the left.

*Braconalejandromasisi* and *Bracontihisiaboshartae* are placed in the same BIN, but their COI barcodes differ by ca. 1%, and each is reared from different genera of caterpillars (*Consul*, *Memphis*) feeding on very different plants (*Piper*, *Croton*). The cocoons of the former are dark brown and tightly fill the caterpillar nest (Fig. 32), whereas the cocoons of the later are white-golden and form an irregular mass in a much looser leaf roll nest of the host caterpillar, not filling it (Fig. 73). Experience has shown that with this level of difference within ACG ecosystems and hosts, larger sample sizes demonstrate that this species-level taxonomic hypothesis is almost certainly correct.

###### Etymology.

*Bracontihisiaboshartae* is name in honor of Tihisia Boshart in specific recognition of her rapid and high-quality rendering of artwork for Hymenoptera and Lepidoptera patronyms for major Costa Rican decision-makers helping ACG.

##### Bracon wilberthbrizuelai

Taxon classificationAnimaliaHymenopteraBraconidae

Sharkey sp. nov.

http://zoobank.org/50707DEB-073E-45C8-AC51-D24B20D256E8

Figure 74

###### Diagnostics.

BOLD:ADB1539. Consensus barcode. ATTTTATACTTCTTATTCGGAATATGAGCTGGAATAATTGGTTTATCTATAAGATTAATTATTCGGTTAGAATTAGGTATACCTGGGAGATTATTAAAAAATGACCAAATTTATAATAGAATAGTCACAGCCCATGCTTTTATTATAATTTTTTTTATAGTTATACCTGTAATATTAGGAGGGTTTGGAAATTGATTAACCCCTTTAATATTAGGTGCTCCTGATATAGCTTTCCCTCGATTAAATAATATAAGATTTTGATTATTAATTCCTTCTTTAATTTTATTATTATTAAGAAGAATTTTAAATGTTGGGGTAGGAACAGGTTGAACTATATACCCCCCATTATCATCAAATTTAGGACACAGAGGTTTATCAGTTGATTTAGCTATTTTTTCCTTACATTTAGCAGGAATTTCTTCTATTATAGGGGCAATAAATTTTATTTCTACTATTTTAAATATACATTTATTTACATTAAAAATAGATCAATTAACTTTATTTGTTTGATCTATTTTTATCACAACTATTTTATTACTATTATCTTTACCAGTTTTAGCTGGTGCTATCACTATATTATTAACAGAT------------------------------------------------.

###### Holotype ♀.

Guanacaste, Sector Pailas Dos, PL12-2, 10.7634, -85.335, 824 meters, Malaise trap, 2/i/2014. Depository: CNC.

***Host data*.** None.

***Holotype voucher code*.**BIOUG29474-A02.

###### Paratypes.

None.

###### Etymology.

*Braconwilberthbrizuelai* is named to honor Wilberth Brizuela for his cheerful and always-ready restaurant at Nuevo Zelandia, that has supported many ACG staff and visitors.

**Figure 74.**
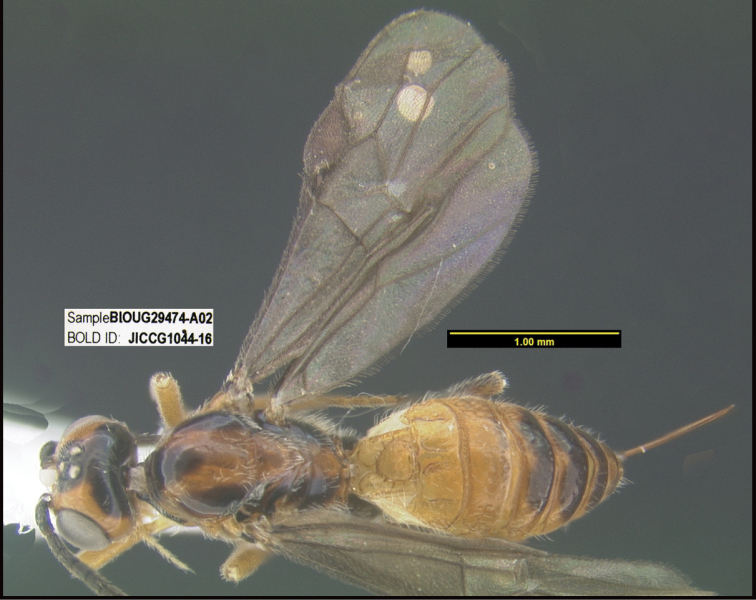
*Braconwilberthbrizuelai*, holotype.

#### *Digonogastra* Viereck, 1912

*Digonogastra* is a large and species rich genus restricted to the New World. Members attack larval Lepidoptera and Coleoptera. The generic assignment of many species of *Digonogastra* from *Cyanopterous* is difficult or impossible because the precise limits of the two are not well defined at present.

##### Digonogastra montylloydi

Taxon classificationAnimaliaHymenopteraBraconidae

Sharkey sp. nov.

http://zoobank.org/3C16F085-9C41-472D-89AD-8BA242602046

Figure 75

###### Diagnostics.

BOLD:ADD0356. Consensus barcode. ATTTTATATTTTTTATTTGGGATGTGATCTGGAATAGTAGGGTTATCAATAAGTTTAATTATTCGATTAGAATTAGGAGTTCCTGGAAGGTTATTAGGTAATGATCAAATTTATAATAGAATAGTTACTGCTCATGCTTTTGTAATAATTTTTTTTATAGTTATACCAATTATATTAGGTGGATTTGGAAATTGGTTAATTCCTTTAATATTAGGTGCTCCTGACATAGCTTTCCCTCGAATAAATAATATAAGATTTTGATTATTAATTCCATCTTTATTAATATTATTGTTGAGAGGAATTTTAAATGTGGGTGTAGGTACTGGATGAACAATTTATCCTCCATTATCTTCATTTTTGGGACATGGAGGGATTTCTGTTGATTTAGCTATTTTTTCTTTACATTTGGCTGGTGTTTCTTCAATTATAGGATCAATTAATTTTATTACAACAATTTTAAATATACGATTATTTTTTTTGAAATTAGATCAATTAACTTTATTTATTTGATCAATTTTTATTACTACAATTTTATTATTATTATCTTTACCAGTTTTAGCTGGGGGAATTACTATATTATTAACAGATCGTAATTTAAATACAACTTTTTTTGATTTTTCTGGTGGAGGAGATCCAATTTTATTTCAACATTTA.

###### Holotype ♀.

Guanacaste, Sector Pitilla, Estación Quica, 10.99697, -85.39666, 470 meters, caterpillar collection date 28/iv/2008, wasp eclosion date: 30/iv/2008. Depository: CNC.

***Host data*.***Oiketicuskirbyi* (Psychidae) feeding on *Byrsonimacrassifolia* (Malpighiaceae). The gregarious wasp larvae spin their cocoons inside the bagworm silk and twig nest; the paratypes are sibs of the holotype.

***Caterpillar and holotype voucher codes*.** 08-SRNP-70260, DHJPAR0028324.

###### Paratypes.

Host = same as holotype: DHJPAR0028320, DHJPAR0028321, DHJPAR0028322, DHJPAR0028323. Depository: CNC.

###### Etymology.

*Digonogastramontylloydi* is named in honor of Monty Lloyd’s long-appreciated contributions to publicity for ACG, GDFCF, and now, BioAlfa.

**Figure 75.**
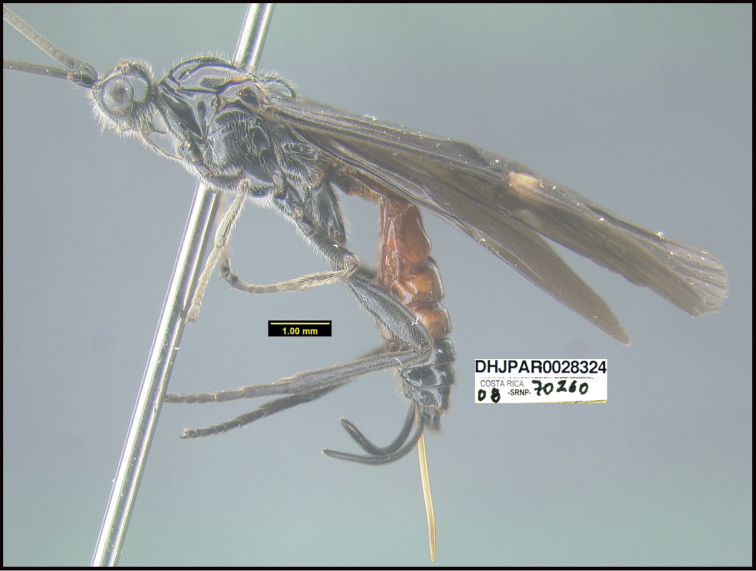
*Digonogastramontylloydi*, holotype.

##### Digonogastra montywoodi

Taxon classificationAnimaliaHymenopteraBraconidae

Sharkey sp. nov.

http://zoobank.org/0BE3373C-9A72-461F-A0B9-295C9F8F0B18

Figure 76

###### Diagnostics.

BOLD:ADJ0327. Consensus barcode. TATATTATATTTTTTATTTGGTATATGAGCTGGAATAATTGGTTTGTCTATAAGATTAATTATTCGTTTAGAGTTAGGTATACCGGGTAGAATATTAAATAATGATCAAATTTATAATAGAATAGTTACTGCTCATGCTTTTATTATAATTTTTTTTATGGTTATACCTATAATAGTAGGTGGATTTGGGAATTGATTAACACCTTTAATATTAGGGGCTCCTGATATGGCTTTCCCACGAATAAATAATATAAGATTTTGGTTATTGGTTCCTTCAATTTTATTATTAATATTAAGAAGAATTATAAATATTGGAGTAGGTACTGGATGAACAATATATCCTCCTTTATCTTCTTTATTAGGACATAGTGGAATTTCAGTTGATTTAGCAATTTTTTCTTTACATTTAGCGGGGGTTTCTTCAATTATAGGTTCAATTAATTTTATTTCAACAATTTTAAATATACGTTTATTTTATTTAAAATTAGATCAATTAACTTTATTTATTTGATCAATTTTTATTACAACAATTTTGTTATTATTATCTTTACCTGTTTTGGCGGGGGGTATTACTATGTTATTAACTGATCGTAATTTAAATTCTACATTTTTTGATTTTTCTGGAGGAGGAGATCCAATTTTATTTCAACATTTATTT.

###### Holotype ♀.

Guanacaste, Sector Pailas, Catarata Borinquen, 10.817721, -85.390465, 945 meters, 29/i/2017, light-trapped. Depository: CNC.

***Host data*.** None.

***Holotype voucher code*.**DHJPAR0061034.

###### Paratypes.

None.

###### Other material.

A specimen from Panama is in the same BIN and is likely conspecific.

###### Etymology.

*Digonogastramontywoodi* is named in honor of Monty Wood’s (RIP) long-appreciated contributions to publicity for ACG, GDFCF, and now, BioAlfa.

**Figure 76.**
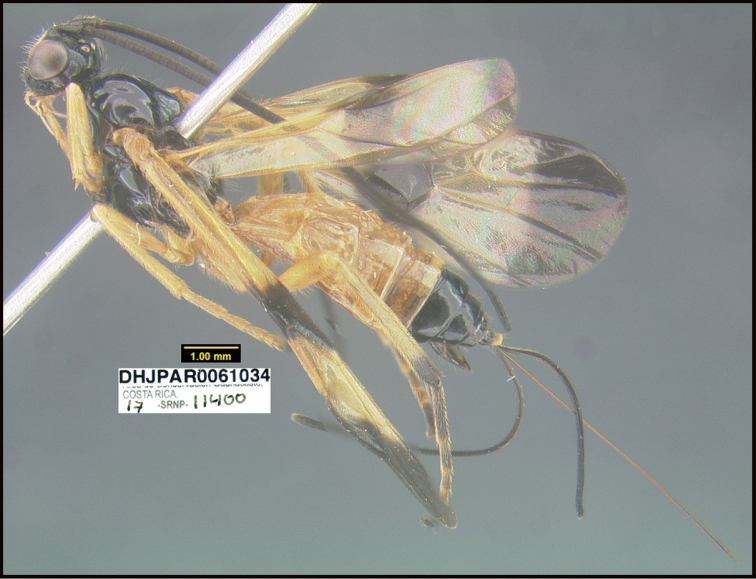
*Digonogastramontywoodi*, holotype.

##### Digonogastra motohasegawai

Taxon classificationAnimaliaHymenopteraBraconidae

Sharkey sp. nov.

http://zoobank.org/D2C90F51-7D9F-4C1C-997D-28F4A4AAEAD5

Figure 77

###### Diagnostics.

BOLD:ADJ4657. Consensus barcode. GATATTGTATTTTTTATTTGGTATATGGGCTGGGATAGTTGGTTTATCAATAAGATTAATTATTCGTTTAGAGTTGGGGATACCTGGAAGGATGTTAGGTAATGATCAAATTTATAATAGAATAGTGACGGCACATGCTTTTGTAATAATTTTTTTTATAGTTATACCAGTAATATTGGGGGGGTTTGGAAATTGGTTAATTCCATTAATGTTAGGGGCTCCTGATATAGCTTTCCCTCGAATAAATAATATAAGGTTTTGATTACTTATTCCTTCAATTTTATTATTATTATTAAGAAGAATTTTAAATATTGGRGTAGGTACAGGGTGAACTGTTTATCCTCCTTTATCTTCATCTTTAGGACATAGAGGGGTATCAGTTGATTTAGCTATTTTTTCTTTACATTTAGCTGGTGTTTCTTCTATTATAGGGTCAATTAATTTTATTACAACAATTTTAAATATACGTTTATTTTTTTTAAAATTAGATCAATTAACTTTATTTATTTGATCAATTTTTATTACAACAATTTTATTGTTATTATCTTTACCTGTTTTGGCAGGGGGAATTACAATACTATTAACAGATCGAAATTTAAATACAACATTTTTTGATTTTTCTGGGGGAGGGGATCCTGTTTTGTTTCAACATTTATTT.

###### Holotype ♀.

Guanacaste, Sector Pailas, Catarata Borinquen, 10.817721, -85.390465, 945 meters, 29/i/2017, light-trapped. Depository: CNC.

***Host data*.** None.

***Holotype voucher code*.**DHJPAR0061037.

###### Paratypes.

DHJPAR0061039. Depository: CNC.

###### Etymology.

*Digonogastramotohasegawai* is named in honor of Moto Hasegawa’s long-appreciated contributions to publicity for ACG, GDFCF, and now, BioAlfa.

**Figure 77.**
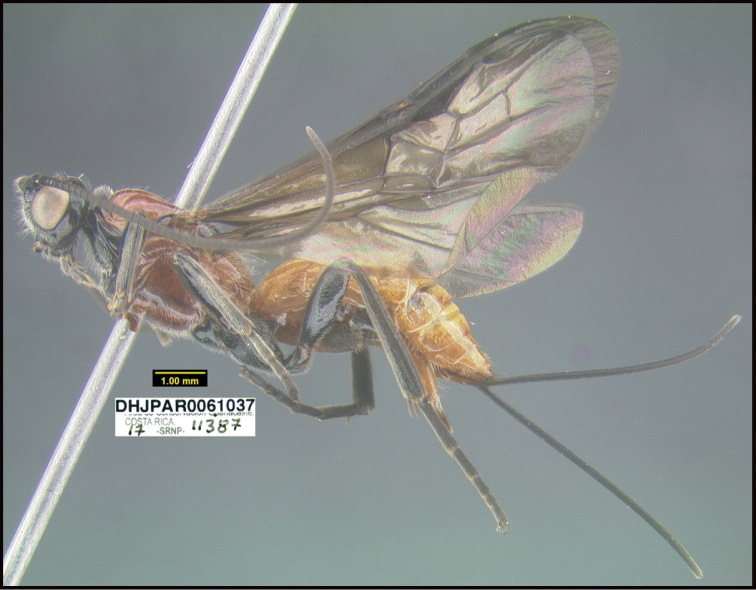
*Digonogastramotohasegawai*, holotype.

##### Digonogastra natwheelwrighti

Taxon classificationAnimaliaHymenopteraBraconidae

Sharkey sp. nov.

http://zoobank.org/72B00897-B561-47A8-B2A0-BA7BC73DDE83

Figure 78

###### Diagnostics.

BOLD:ADM7321. Consensus barcode. AATATTGTATTTTTTATTTGGTATGTGAGCTGGTATAGTTGGATTATCAATAAGGTTGATTATTCGTTTAGAATTAGGTATACCAGGGAGTTTATTAGGTAATGATCAGATTTATAATAGTATAGTAACTGCTCATGCTTTTATTATAATTTTTTTTATGGTTATACCTATTATATTAGGGGGATTTGGGAATTGATTAATTCCTTTAATATTGGGGGCCCCAGATATAGCTTTTCCTCGAATAAATAATATAAGATTTTGATTACTTATTCCTTCTATTTTGTTGTTATTATTAAGGAGATTTATAAATATTGGGGTTGGTACAGGATGGACAGTTTATCCTCCTTTATCTTCTTCTTTGGGGCATAGGGGTATTTCAGTTGATTTAGCAATTTTTTCTTTACATTTAGCTGGTATTTCTTCAATTATGGGGTCAATTAATTTTATTTCTACTATTTTAAATATACATTTATTTTTTTTAAAATTAGATCAGTTAACTTTGTTTATTTGATCAATTTTTATTACAACAATTTTATTGTTATTATCTTTACCTGTTTTAGCTGGGGGTATTACTATATTATTAACGGATCGTAATTTAAATACAACTTTTTTTGATTTTTCGGGGGGGGGAGATCCTATTTTATTTCAACATTTATTT.

###### Holotype ♀.

Guanacaste, Sector Pailas, PDL#5, 10.7627, -85.334, 825 meters, 4/i/2014, light-trapped. Depository: CNC.

***Host data*.** None.

***Holotype voucher code*.**DHJPAR0062413.

###### Paratypes.

None.

###### Etymology.

*Digonogastranatwheelwrighti* is named in honor of Nat Wheelwright’s long-appreciated contributions to publicity for ACG, GDFCF, and now, BioAlfa.

**Figure 78.**
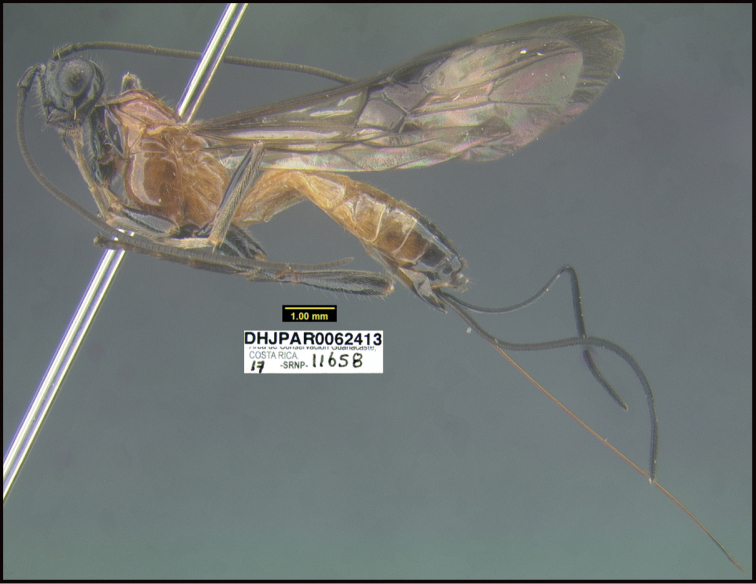
*Digonogastranatwheelwrighti*, holotype.

##### Digonogastra nickgrishini

Taxon classificationAnimaliaHymenopteraBraconidae

Sharkey sp. nov.

http://zoobank.org/DB0F73B8-9C0F-4D51-AFB8-19FC44B991F0

Figure 79

###### Diagnostics.

BOLD:ADJ3490. Consensus barcode. GGTATTATATTTTTTATTTGGGATGTGATCAGGAATATTAGGTTTATCAATAAGAATAATTATTCGTTTAGAATTAGGGATACCAGGAAGAATATTAGGTAATGATCAAATTTATAATAGAATAGTTACTATTCATGCTTTTATTATAATTTTTTTTATAGTTATACCAATTATATTAGGTGGATTTGGTAATTGATTAATTCCATTAATATTAGGGGCACCTGATATGGCTTTCCCTCGAATAAATAATATAAGATTTTGATTAATTATTCCTTCTTTAATGTTATTATTATTAAGAAGGGTAATAAATGTTGGAGTAGGAACTGGTTGAACTATATATCCTCCTTTATCATCATTTTTAGGTCATGGAGGAATATCAGTTGATTTATCAATTTTTTCTTTACATTTGGCTGGAATTTCTTCAATTATGGGTGCAATTAATTTTATTACAACTATTTTAAATATACGTTTATTTTTTTTAAAGTTAGATCAGTTAACTTTATTTATTTGATCAATTTTTATTACTACAATTTTATTATTATTATCATTACCAGTTTTAGCTGGTGGAATTACAATATTATTAACTGATCGTAATTTAAATACTACATTTTTTGATTTTTCTGGAGGGGGGGATCCAATTTTATTTCAACATTTATTT.

###### Holotype ♀.

Guanacaste, Sector Pailas, Catarata Borinquen, 10.817721, -85.390465, 945 meters, 29/i/2017, light-trapped. Depository: CNC.

***Host data*.** None.

***Holotype voucher code*.**DHJPAR0061042.

###### Paratypes.

None.

###### Etymology.

*Digonogastranickgrishini* is named in honor of Nick Grishin’s long-appreciated contributions to publicity for ACG, GDFCF, and BioAlfa.

**Figure 79.**
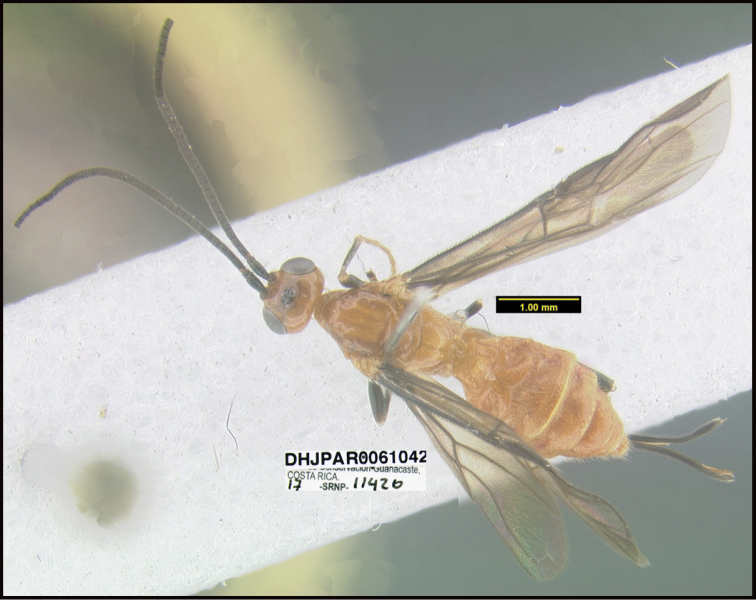
*Digonogastranatwheelwrighti*, holotype.

## Chapter 4: Cheloninae

Cheloninae have been viewed as solitary, koinobiont, egg-larval parasitoids, i.e., lay one egg in the egg of the host and delaying development until the host caterpillar is nearing maturity (van Achterberg 1990b; Shaw and Huddleston 1991; Shaw 1997; Hanson and Gauld 2006). Contrary to this generalization, of the 60 species reared by the inventory, *Chelonusmotohasegawai* [2 records] (Fig. 81), *Chelonusmichellevanderbankae* [13 records] (Figs 82, 83), and *Chelonusnataliaivanovae* [3 records] (Fig. 84) are gregarious. It is not known if these species are injecting multiple eggs into an egg of the host, or if the wasp eggs are polyembryonic. The question is complicated because oviposition behavior of Cheloninae is based on few records (Shaw and Huddleston 1991). It is not even known if all chelonines are egg-larval parasitoids. For example, there are no rearing records for Adeliini and most of the less common chelonine genera such as *Pseudophanerotoma* and *Ascogaster*.

Quicke et al. (2012) noted that when gene trees of the barcode region of COI are the product of species-rich samples, they are impressionably accurate in recapitulating the assumed phylogeny. Four of the six included genera are effectively monophyletic in the COI neighbor-joining tree (Suppl. material 3), and we obtained the same result with a multilocus tree that included extensive outgroups (not illustrated). Only two species cause the lack of monophyly for *Pseudophanerotoma*: *Pseudophanerotomaallisonbrownae* and *Pseudophanerotomabobrobbinsi*. These are both nested within *Phanerotoma* in the tree (Suppl. material 3). *P.bobrobbinsi* is well nested within *Phanerotoma* even though it has all of the other characteristics of *Pseudophanerotoma*, other than being somewhat unusual in its dark coloration (Fig. 205). *Pseudophanerotomaallisonbrownae* is placed as the sister to all *Phanerotoma* and its generic affiliation is somewhat questionable. Unlike all other members of *Pseudophanerotoma*, the second submarginal cell of the forewing does not intersect vein 1RS+M. Instead, it meets the junction of 1RS+M and crossvein cu-a. Furthermore, the origin of 1RS+M is close to vein M rather than high in the parastigma. However, unlike all members of *Phanerotoma*, *Pseudophanerotomaallisonbrownae* has more than 21 flagellomeres. We prefer to rely on this single character due to its clear definition and lack of intermediates. With these comments in mind, the New World genera may be identified using the key below.

All reared Cheloninae reported here are parasitoids of wild-caught leaf rolling and webbing “Microlepidoptera” caterpillars and none from “macrocaterpillars” even though there are 550+ species of leaf-sheltering or leaf-nest Hesperiidae caterpillars in the rearing inventory (Janzen et al. 2011). The 66 reared species are derived from a sample of ca. 1,170 caterpillars among 719,000 “wild-caught then lab-reared” caterpillars of ca. 5,000 species over 40 years (1978‒2018 inclusive) in all ACG ecosystems and habitats. However, if we restrict the summaries to the leaf-rolling and webbing “small” caterpillars in the ACG inventory (Crambidae, Gelechiidae, Depressariidae, Thyrididae, Oecophoridae, Tortricidae, Choreutidae, Immidae, Gracillariidae), the available sample is ca. 107,000 caterpillars of ca. 2,700 species. Because chelonine parasitization is both obvious and restricted, we can offer these generalizations, but further inventory is required to offer this for other subfamilies

Dan Boyce (1936) observed that a species of *Ascogaster* pupated within the pupal chamber of the host, and that has become the universal truth applied to all chelonines (Hanson and Gauld 2006). The ACG rearing experience has confirmed this for all species of Cheloninae reared by the inventory, though the host pupal chamber ranges from a silk cocoon on a leaf surface to between two leaf surfaces silked together by the caterpillar. It is quite commonplace for the feeding caterpillar to live between two leaves lightly silked together, which then become the roof and floor of the chamber into which the wasp larva spins its cocoon after emerging from the prepupal caterpillar (or an earlier stage, whichever the case may be).

In some cases where there are many records for a single species of reared Cheloninae, this is because the host caterpillars are often gregarious and live in a sloppy mass of silked-together leaves. Presumably, the female moth lays a large mass of 50‒200 eggs, and when a female chelonine wasp finds it, it has the opportunity to oviposit into many eggs. The consequence is that the eclosing wasps will be sibs (e.g., *Pseudophanerotomaalexsmithi*, *Chelonussujeevanratnasinghami*, *Chelonusscottshawi*, *Chelonusgustavogutierrezi*).

**Figure 80.**
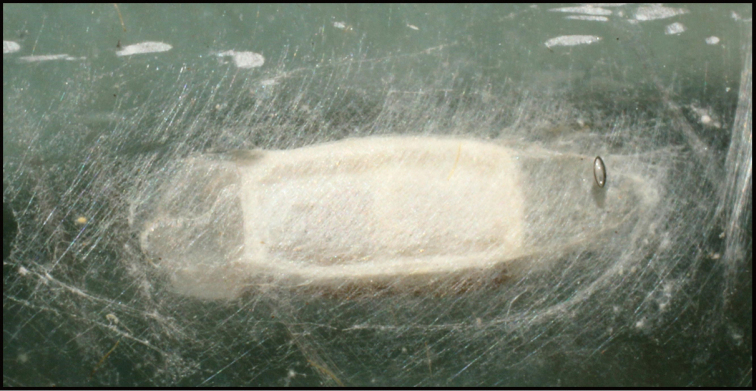
Cocoon of *Chelonusrodrigogamezi* (DHJPAR0050074).

The ACG rearing inventory has found that an identification trait of most chelonine cocoons is that they are squared at both apices, as shown in Fig. 80. This is diagnostic for most reared ACG members of the subfamily, with the occasional exception such as *Chelonusmichellevanderbankae* (Fig. 83).

All *Adelius* and most *Ascogaster* and *Phanerotoma* were captured with Malaise traps and rarely reared. In part, this is because they may be parastioids of caterpillars that are miners in leaf tissue, rather than exposed in a leaf-silk webbing. For practical reasons, leaf-mining caterpillars are rarely reared in the ACG, remaining for further development of the inventory. For the Cheloninae NJ tree, see Suppl. material 3.

**Figure 81.**
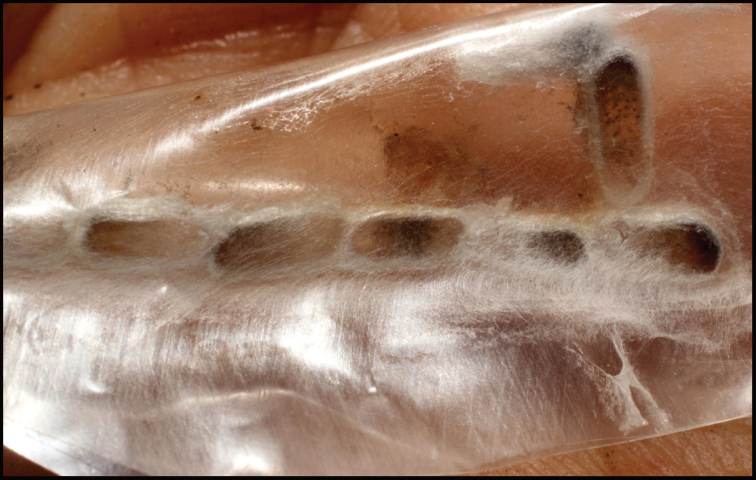
Cocoons of *Chelonusmotohasegawai* in the remains of the host caterpillar pupal chamber spun between sides of a plastic rearing bag (DHJPAR0042920).

**Figure 82.**
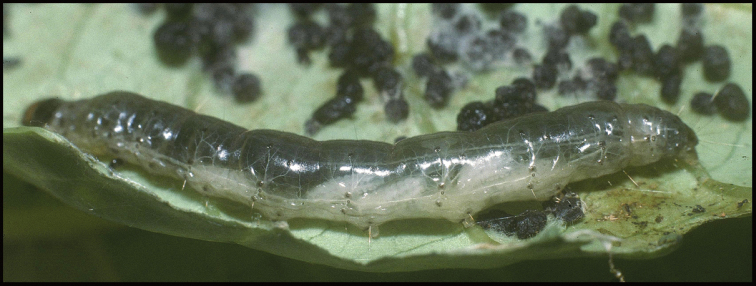
Multiple white larvae of *Chelonusmichellevanderbankae* (DHJPAR0029134) inside its translucent host caterpillar (05-SRNP-30968).

**Figure 83.**
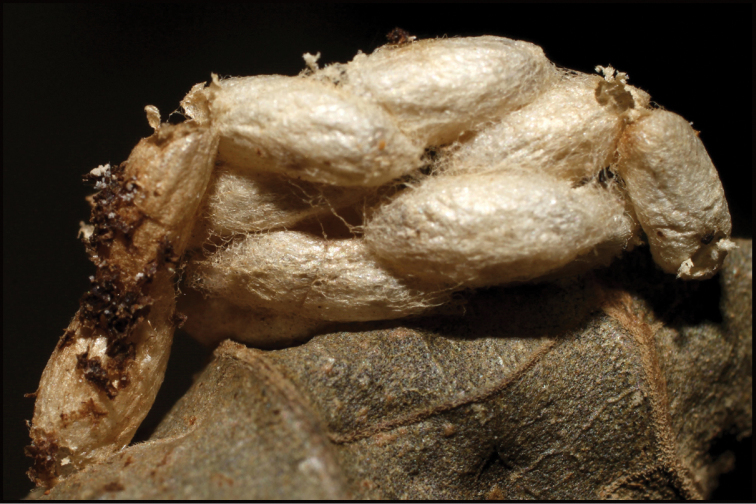
Cocoons of *Chelonusmichellevanderbankae*, (DHJPAR0039447); all larvae emerged from a single caterpillar (see Fig. 82).

**Figure 84.**
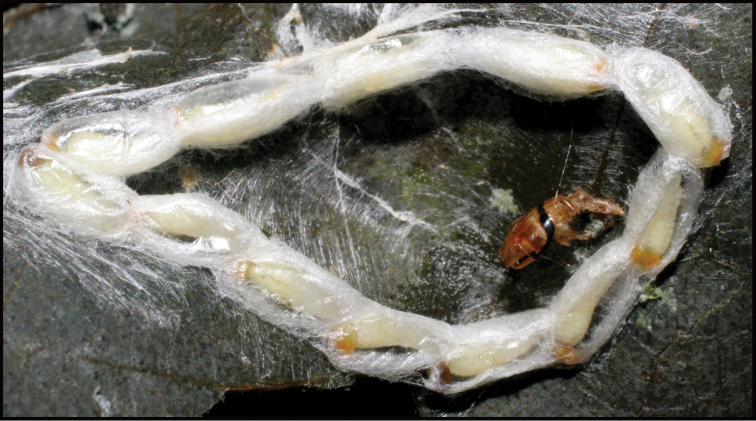
Prepupae of *Chelonusnataliaivanovae* (DHJPAR0035559) inside the pupal chamber of a single caterpillar of its host (*Anacrusisturrialbae* 09-SRNP-44104) after the leaf ceiling was lifted off. The head and thorax capsule of the caterpillar are inside the ring of prepupae in the cocoons that they have spun; a yellow meconial pellet is at the posterior end of each cocoon.

### Key to New World genera of Cheloninae

**Table:** 

1	A. RS of forewing not reaching wing margin; small specimens usually under 2 mm	**2**
–	B. RS of forewing reaching wing margin; specimens usually longer than 2 mm	**3**
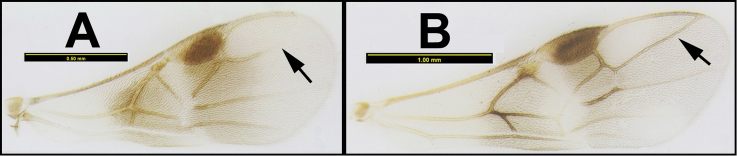		
2 (1)	A. First two metasomal terga sculptured; Nearctic	*** Paradelius ***
–	B. First two metasomal terga not or only weakly sculptured; widespread	*** Adelius ***
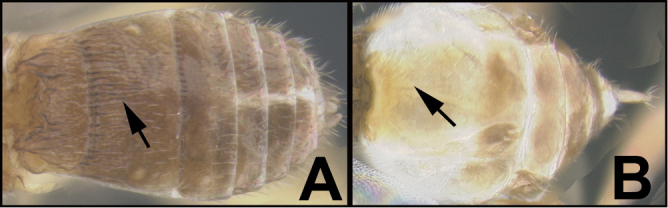		
3(1)	A. Metasomal carapace with two traverse depressions on	**4**
–	B. Metasomal carapace without two traverse depressions	**10**
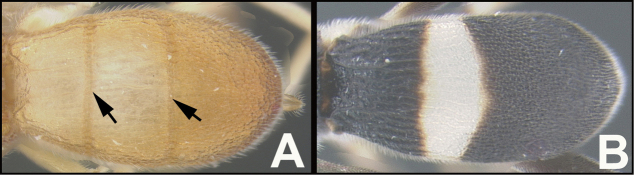		
4(3)	A. Forewing crossvein cu-a and vein M meeting or almost so; occipital carina incomplete dorsally; Chile, rare.	*** Leptochelonus ***
–	B. Forewing crossvein cu-a originating far distal to M; occipital carina complete dorsally; widespread, common.	**5**
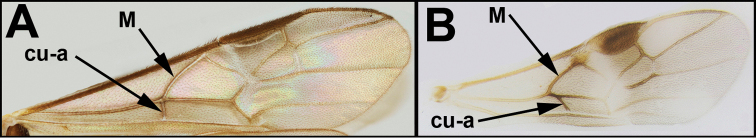		
5(4)	A. Antenna with 23 antennal articles, including scape (S) and pedicel (P). AA. 1RS+M vein well distant from vein M and intersecting parastigma (PS)	**6**
–	B. Antenna with more than 23 antennal articles including scape (S) and pedicel (P). BB. 1RS+M vein intersecting vein M or parastigma (PS) very close to vein M	**8**
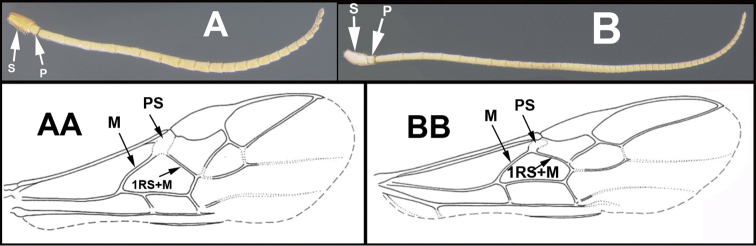		
6(5)	A. Clypeus without teeth or rounded protuberances; Neotropical, rare	*** Phanerotomoides ***
–	B. Clypeus with two or more teeth or rounded protuberances; widespread, common	**7**
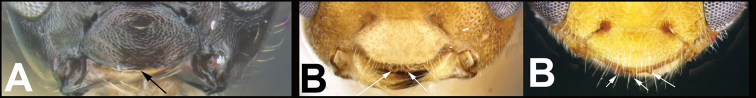		
7(6)	A. Metasomal carapace decurved apically and covering all segments; widespread, common.	*** Phanerotoma ***
–	B. Metasomal carapace relatively flat apically and not covering all segments; Neotropical, rare	*** Huseyinia ***
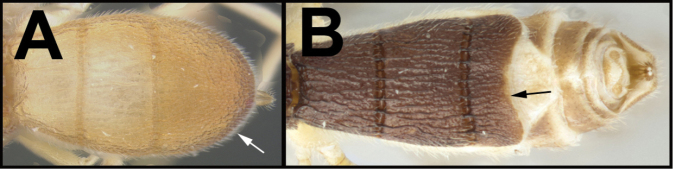		
8(5)	A. Clypeus ca. as long as wide and without teeth or rounded protuberances; Neotropical, rare.	*** Furcidentia ***
–	B. Clypeus distinctly wider than long and with teeth or rounded protuberances (the latter sometimes very weak)	**9**
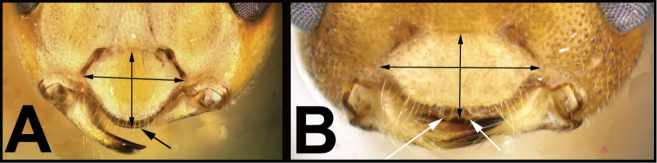		
9(8)	A. Latero-posterior margin of carapace with two large teeth; Neotropical; rare	*** Dentigaster ***
–	B. Latero-posterior margin of carapace without pair of large teeth; widespread; relatively common	*** Pseudophanerotoma ***
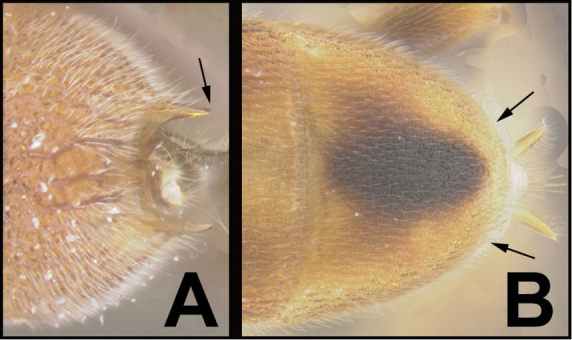		
10(3)	A. Forewing 1-SR+M absent	*** Chelonus ***
–	B. Forewing 1-SR+M present	**11**
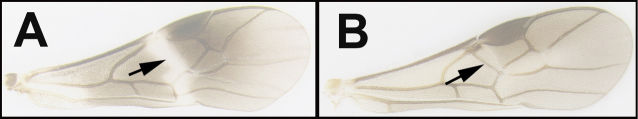		
11(10)	A. Ocelli forming an isosceles triangle	*** Ascogaster ***
–	B. Ocelli forming an equilateral triangle	*** Leptodrepana ***
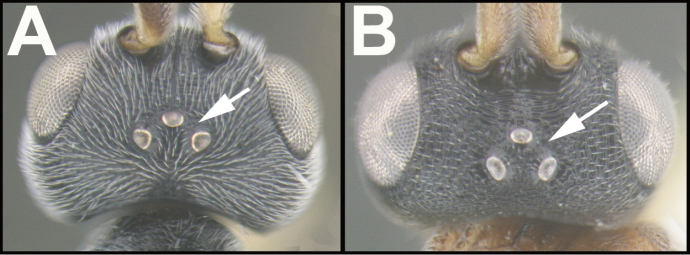		

#### *Adelius* Haliday, 1833

*Adelius* is worldwide in distribution. Hosts are leaf-mining Lepidoptera, e.g., Nepticulidae. Shimbori et al. (2019) revised the New World species of *Adelius* and included 19 species, seven of which are from Costa Rica.

##### Adelius adrianguadamuzi

Taxon classificationAnimaliaHymenopteraBraconidae

Sharkey sp. nov.

http://zoobank.org/647A5D50-F635-482C-AC34-B6A6796C4E87

Figure 85

###### Diagnostics.

BOLD:ADF7277. Consensus barcode. ATTTTATATTTTATATTTGGTATTTGATCAGGTGGTTTAGGTTTATCACTAAGAATAATAATTCGTATAGAATTAAGAACAGTAGGAAGTTATTTGGGTAATGATCAAATTTATAATAGAATTGTAACTCTTCATGCTTTTGTGATAATTTTTTTTATAGTTATACCAATTATAATTGGTGGATTTGGTAATTGGTTAATTCCATTAATAGTAATAAGACCTGATATATCATTTCCTCGAATAAATAATATGAGATTTTGATTATTAATTCCTTCTTTATTTTTATTGATTATGAGAAGATTTGTAAATGTTGGTGTAGGTACAGGATGAACAGTTTATCCTCCTTTATCTTTAATTATAGGTCATGGTGGTGTAGCTGTAGATTTATCAATTTTTTCTTTACACTTAGCTGGGATTTCATCAATTATGGGTGCTATAAATTTTATTGTTACAATTTTTAATAGATCAATATTAATAAATATATTTAATAAAATTTCTTTATTTTGTTGATCTGTTTTTATTACAGCTTTTTTATTGCTATTATCTTTACCAGTATTAGCTGGGGCTATTACTATATTATTAACTGAT----------------------------. Similar to *A.excelsus* Bortoni and Shimbori, but differs slightly in color, and the hind tibia is much more swollen in *A.adrianguadamuzi*.

###### Holotype ♀.

Guanacaste, Sector Cacao, Derrumbe, 10.9292, -85.4643, 1220 meters, 18‒25/x/2014, Malaise trap. Depository: CNC.

***Host data*.** None.

***Holotype voucher code*.**BIOUG31661-B07..

###### Paratypes.

None.

###### Note.

The holotype and all but one paratype of *A.excelsus* are from high elevation localities in Colombia. The paratype of *A.excelsus* from Costa Rica is likely to be a member of *A.adrianguadamuzi* in the opinion of MJS.

###### Etymology.

*Adeliusadrianguadamuzi* is named to honor Sr. Adrian Guadamuz of GDFCF and ACG for his many years as a dedicated plant parataxonomist, and Malaise and mouse live trapper for ACG.

**Figure 85.**
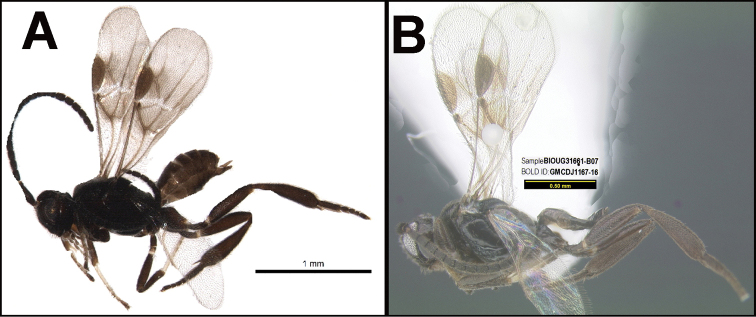
*Adeliusadrianguadamuzi*, holotype **A**BOLD image **B** mounted image.

##### Adelius gauldi

Taxon classificationAnimaliaHymenopteraBraconidae

Shimbori & Shaw, 2019

###### Diagnostics.

BOLD:ACJ2315. Image available on BOLD.

***Host data*.** None.

###### Material examined.

♀, Guanacaste, Sector Santa Rosa, Bosque San Emilio, 10.8438, -85.6138, 300 meters, 16‒23/iv/2012, Malaise trap. BIOUG07619-D09, BIOUG18398-G03.

##### Adelius janzeni

Taxon classificationAnimaliaHymenopteraBraconidae

Shimbori & Shaw, 2019

###### Diagnostics.

BOLD:ACJ2270. Image available on BOLD.

***Host data*.** None, Malaise trap.

###### Material examined.

♀, Guanacaste, Sector Santa Rosa, Bosque San Emilio, 10.8438, -85.6138, 300 meters, 16‒23/iv/2012, Malaise trap. BIOUG07614-F11.

#### *Ascogaster* Wesmael, 1835

No described species of *Ascogaster* are documented from Costa Rica, and only two species are recorded from the Neotropics, i.e., Peru and Uruguay, though there are probably a few hundred species undescribed. Numerous lepidopteran families serve as hosts. Here we add records from Tortricidae and Depressariidae, neither of which is a new family record.

##### Ascogaster gloriasihezarae

Taxon classificationAnimaliaHymenopteraBraconidae

Sharkey sp. nov.

http://zoobank.org/40E59D9E-D1CA-43DF-8187-D3C4DE6FE121

Figure 86

###### Diagnostics.

BOLD:ADA4144. Consensus barcode. ATTTTATATTTTATTTTTGGAATTTGATCAGGTATATTTGGTTTATCTTTAAGTCTAATTATTCGAATAGAATTAAGGTCGGTTACAGCTTATTTAGGTAACGATCAAATTTATAACAGGTTAGTTACAATACATGCTTTTATTATAATTTTTTTTATAGTTATACCAATTATAATTGGTGGTTTTGGAAATTGATTAGTTCCTTTAATATTAGGTGGTCCTGATATATCATTTCCTCGAATAAATAATATAAGTTTTTGATTATTAATTCCTTCACTTTTTTTATTGATTATAAGAAGTTTTGTAAATGTAGGTGTGGGTACAGGATGAACAGTTTATCCTCCTTTATCATTAATTATTGGGCATGGTGGGGTATCAGTTGATATTAGAATTTTTTCTTTACATATAGCTGGAATATCTTCTGTAATAGGTGCATTAAATTTTATTGTAACAATTATAAATATATGAATTGGAGTAAAATATATAGATAAATTGTCTTTATTCACTTGATCAGTTTTTATTACAGCTATTTTATTATTACTATCCTTGCCTGTTTTAGCTGGTGCAATTACTATATTATTAACTGATCGAAATTTTAAT---------------------------------.

###### Holotype ♂.

Alajuela, Sector San Cristobal, Estación San Gerardo, 10.8801, -85.389, 575 meters, 16‒23/xii/2013, Malaise trap. Depository: CNC.

***Host data*.** None.

***Holotype voucher code*.**BIOUG28068-C02.

###### Paratypes.

None.

###### Etymology.

*Ascogastergloriasihezarae* is named to honor Sra. Gloria Sihezar of GDFCF and ACG for three decades of outstanding parataxonomist inventory of ACG biodiversity.

**Figure 86.**
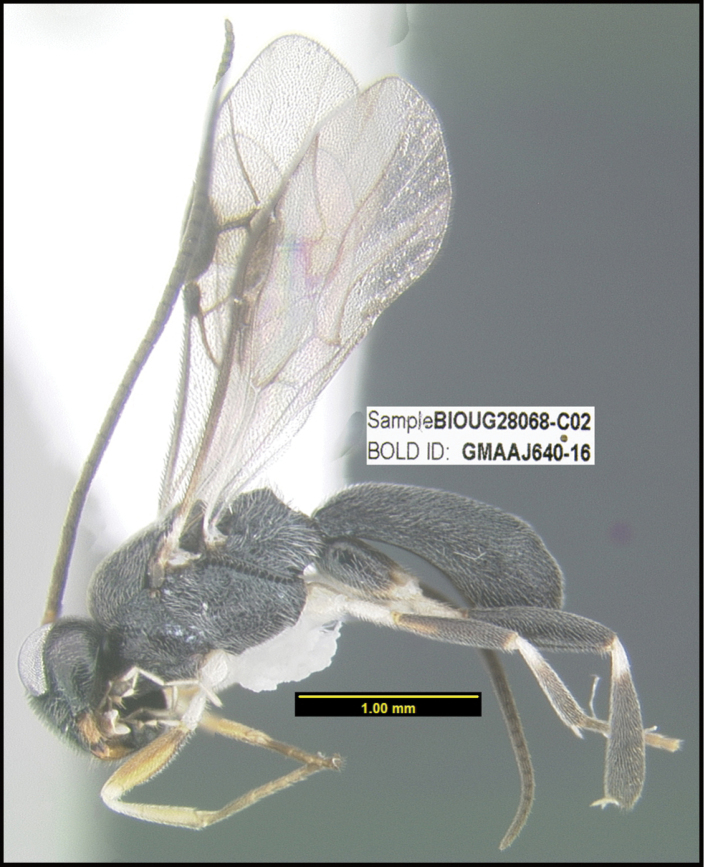
*Ascogastergloriasihezarae*, holotype.

##### Ascogaster grettelvegae

Taxon classificationAnimaliaHymenopteraBraconidae

Sharkey sp. nov.

http://zoobank.org/D75F27FE-F2B1-4AFD-BD59-0E59B1783315

Figure 87

###### Diagnostics.

BOLD:ABX8525. Consensus barcode. TATTTGGACTATCTTTAAGTTTAATTATTCGAATAGAATTAAGTTCTATTACTTCTTATTTAGGTAATGATCAGATTTATAATAGAGTAGTTACTCTACATGCATTTATTATAATTTTTTTTATAGTTATACCTATTATAATTGGCGGTTTTGGAAATTGGTTGGTTCCTTTAATGTTAGGAGGACCAGATATATCATTTCCTCGAATAAATAATTTAAGATTTTGATTGTTAGTACCTTCAATTATTTTATTAATTAATAGAAGTTTTATTAATGTAGGGGTAGGAACAGGATGAACTGTATATCCGCCTTTATCTCTTATAATTGGCCATAGAGGAGCTTCTGTAGATTTAAGTATTTTTTCTTTGCATTTAGCTGGAATATCCTCTATTATAGGGGCTGTAAATTTTATTGTTACTATTTTAAATATAAGTTTTAGATTTAAAAATATAGATAAATTTCCTTTGTTTGTGTGATCAATTATAATTACTGCAATTTTATTACTTCTATCTTTACCTGTCTTAGCAGGTGCAATTACTATATTATTAACAGATCGAAATTTAAATACTAGATTTTTTGACCCCTCAGGGGGCGGTGATCCAATTTTATATCAACATTTATTT.

###### Holotype ♂.

Guanacaste, Sector San Cristobal, Tajo Angeles, 10.865, -85.415, 540 meters, caterpillar collection date: 13/i/2011, wasp eclosion date: 05/ii/2011. Depository: CNC.

***Host data*.***Episimusortygia* (Tortricidae) feeding on *Vismiabaccifera* (Hypericaceae).

***Caterpillar and holotype voucher codes*.** 11-SRNP-180, DHJPAR0043010.

###### Paratypes.

None.

###### Etymology.

*Ascogastergrettelvegae* is named to honor Sra. Grettel Vega of SINAC for her support of BioAlfa as Director of SINAC, Costa Rica’s National System of Conservation areas.

**Figure 87.**
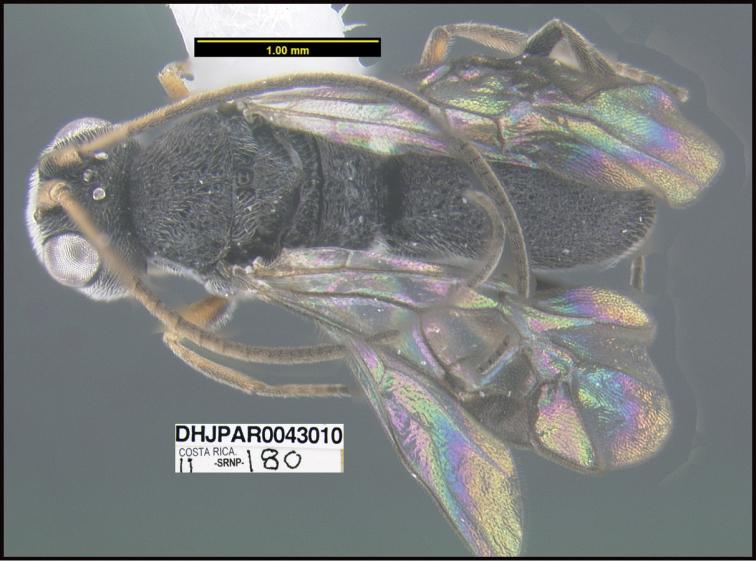
*Ascogastergrettelvegae*, holotype.

##### Ascogaster guillermopereirai

Taxon classificationAnimaliaHymenopteraBraconidae

Sharkey sp. nov.

http://zoobank.org/AC1B5BAE-45B0-4461-A606-5346E33BA829

Figure 88

###### Diagnostics.

BOLD:ACJ4048. Consensus barcode. AATTTTATATTTTATATTTGGAATTTGATCAGGAATAATTGGATTATCTTTAAGTTTATTAATTCGTATAGAATTAAGTTCAGTTTCTTCTTATTTAGGTAATGATCAAATTTATAATAGAATTGTAACTATACATGCTTTTATTATAATTTTTTTTATAGTTATACCTATTATAATTGGAGGCTTCGGAAATTGATTAGTTCCTTTAATATTAGGGGCTCCTGATATATCTTTCCCTCGATTGAATAATTTGAGATTTTGATTATTAATTCCTTCACTTTTTTTTTTATTAATTGGTGGTATTTTGAATTCAGGAGTTGGAACTGGATGAACAGTTTATCCACCGTTGTCATTAGGTATTTATCATAGAGGTATTTCTGTAGATTTAAGTATTTTTTCTTTACATATAGCTGGAATATCTTCAATTTTAGGGGCCTTAAATTTTATTATTACTATTTATTGTATATGGTTAGGTTCAAAAAATATGGATAAGTTATCTTTATTTGTTTGATCAGTAATAATTACTGCATTTTTATTAATTACATCTTTACCAGTTCTAGCAGGCGCAATTACTATATTATTAACTGATCGTAATTTAAATACTAGATTTTTTGATCCTTCTGGTGGTGGTGATCCTGTTTTATATCAACATTTATTT.

###### Holotype ♂.

Alajuela, Sector San Cristobal, Rio Blanco Abajo, 10.90037 -85.37254, 500 meters, caterpillar collection date: 27/ix/2012, wasp eclosion date: 15/x/2012.

***Host data*.***Mictopsichia* Janzen330 (Tortricidae) feeding on *Marcgravianepenthoides* (Marcgraviaceae).

***Caterpillar and holotype voucher codes*.** 12-SRNP-5277, DHJPAR0051332.

###### Paratype.

Host = *Mictopsichia* Janzen330: DHJPAR0051327.

###### Etymology.

*Ascogasterguillermopereirai* is named to honor Sr. Guillermo Pereira of GDFCF and ACG for his many decades as a dedicated inventory parataxonomist, Malaise and mouse live-trapper, and specimen processor for ACG.

**Figure 88.**
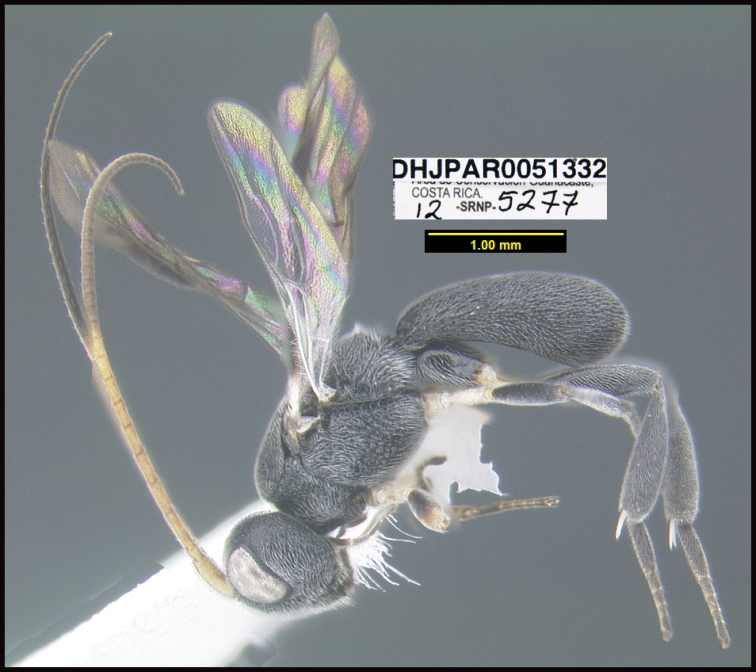
*Ascogasterguillermopereirai*, holotype.

##### Ascogaster gustavoecheverrii

Taxon classificationAnimaliaHymenopteraBraconidae

Sharkey sp. nov.

http://zoobank.org/BFE71690-5F48-4078-BDB1-78A58E988C6E

Figure 89

###### Diagnostics.

BOLD:AAL0574. Consensus barcode. TATTTTATATTTTATATTTGGAATTTGATCTGGTATAGTTGGTTTATCCTTAAGTTTATTAATTTGTATAGAATTAAGATTAGTTTCTTCTTATTTAGGTAATGATCAAATTTATAATAGAGTAGTTACTATACATGCTTTTATTATAATTTTTTTTATAGTTATACCAATTATAATTGGCGGTTTCGGAAATTGATTAGTTCCTTTAATATTAGGAGCTCCTGATATGTCTTTTCCTCGTTTAAATAATTTAAGTTTTCGACTTTTGGTTCCTTCTCTATTTTATTCAATATTAAGAGGTATTTTAAATACTGGGAATGGTACAGGATGAACAGTATATCCACCATTATCATTAGGAATATATCATAGAGGTATTTCTGTAGATCTTAGAATTTTTTCTTTACATATAGCTGGTATATCATCAATTTTAGGAGCTTTAAATTTTATTATTACTATTTATTGTATATGAATTGGTTTTAAAAATATAAATAAATTATCATTATWTGTTTGATCTGTATTAATTACTACTTTTTTATTAATTACATCTTTACCTGTATTAGCAGAAGCTATTACTATATTATTAACTGATCGTAATTTAAATACTAGATTTTTTGATCCATCTGGTGGAGGAGATCCTGTATTATATCAACATTT.

###### Holotype ♀.

Alajuela, Sector San Cristobal, Sendero Huerta, 10.93, -85.372, 527 meters, caterpillar collection date: 18/xi/2013, wasp eclosion date: 12/xii/2013. Depository: CNC.

***Host data*.***Megalotacrassana* (Tortricidae) feeding on *Crotonschiedeanus* (Euphorbiaceae).

***Caterpillar and holotype voucher codes*.** 13-SRNP-6613, DHJPAR0054560.

###### Paratypes.

Hosts = *Megalotaochreoapex*, *Megalotaspinulosa*, *Megalotasimpliciana*, *Megalotadelphinosema*, *Megalotacrassana*. DHJPAR0042852, DHJPAR0042856, DHJPAR0045414, DHJPAR0046941, DHJPAR0049003, DHJPAR0053678, DHJPAR0054566, DHJPAR0062572. Depository: CNC.

###### Etymology.

*Ascogastergustavoecheverrii* is named to honor Sr. Gustavo Echeverri for his thousands of hectares donated to ACG and financial support of the ACG Biological Education Program.

**Figure 89.**
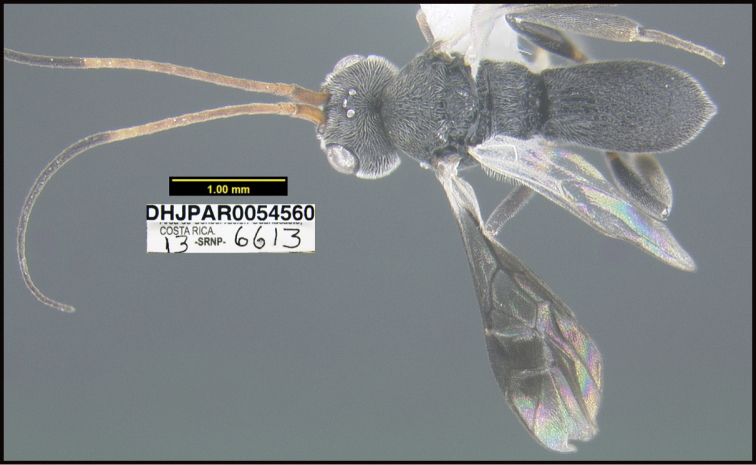
*Ascogastergustavoecheverrii*, holotype.

##### Ascogaster katyvandusenae

Taxon classificationAnimaliaHymenopteraBraconidae

Sharkey sp. nov.

http://zoobank.org/92F7806C-A676-43EF-8A14-DDA750B864F

Figure 90

###### Diagnostics.

BOLD:AAK1026. Consensus barcode. AATTTTGTATTTTTTATTTGGCATTTGATCCGGTGTATTTGGGTTGTCTTTAAGATTAATTATTCGAATAGAATTAAGTTCAGTCACTTCTTATTTAGGTAATGATCAGATTTATAATAGTATTGTCACCCTTCATGCATTTATTATAATTTTTTTTATAGTTATACCAATTATAATTGGAGGTTTTGGAAATTGATTAGTTCCTTTAATGTTAGGGGGTCCAGATATATCATTCCCTCGTATAAATAATTTAAGATTTTGATTATTGGTTCCTTCAATTATTTTACTAATTAATAGAAGTTTTATTAATGTTGGGGTAGGGACAGGATGAACTGTTTATCCACCTTTATCTCTTATTATTGGACATAGAGGAGCTTCTGTAGATATAAGAATTTTTTCTTTACATTTAGCTGGTATATCTTCAATTATAGGGGCTGTAAATTTTATTGTTACTATTTTAAACATAAGATTTAGGTTTAAAAATATGGATAAATTTCCTTTATTTGTTTGATCAATTATAATTACTGCAATTTTATTATTATTATCTTTACCTGTTTTAGCAGGTGCAATTACTATATTATTAACAGATCGAAATTTAAATACAAGTTTTTTTGACCCTTCAGGGGGGGGAGACCCAATTTTATATCAGCATTTATTT.

###### Holotype ♂.

Guanacaste, Sector San Cristobal, Tajo Angeles, 10.865, -85.415, 540 meters, caterpillar collection date: 18/viii/2010, wasp eclosion date: 31/viii/2010. Depository: CNC.

***Host data*.***Megalotaspinulosa* (Tortricidae) feeding on *Crotonschiedeanus* (Euphorbiaceae).

***Caterpillar and holotype voucher codes*.** 10-SRNP-4345, DHJPAR0055824.

###### Paratype.

Host = *Amorbiadecerptana*: DHJPAR0035314. Depository: CNC.

###### Etymology.

*Ascogasterkatyvandusenae* is named to honor Mrs. Katy Van Dusen of Monteverde and Cuajiniquil for her decades of support of ACG activities with birds and marine projects, and chief collaborator with Frank Joyce.

**Figure 90.**
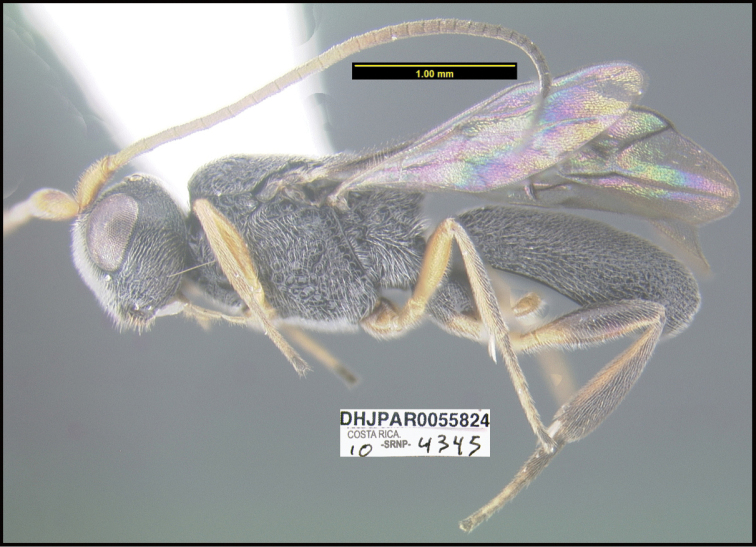
*Ascogasterkatyvandusenae*, holotype.

##### Ascogaster luisdiegogomezi

Taxon classificationAnimaliaHymenopteraBraconidae

Sharkey sp. nov.

http://zoobank.org/4536F2C5-83FB-41FF-8D49-19D61C2DB7F0

Figure 91

###### Diagnostics.

BOLD:ACC1221. Consensus barcode. AATTTTGTATTTTTTATTTGGTATTTGATCAGGTATAATTGGTTTATCTTTAAGTTTATTAATTCGTATAGAATTAAGATCAGTTTCTTCTTATTTAGGAAATGATCAAATTTATAATAGGGTAGTAACAATGCATGCTTTTATCATAATTTTTTTTATAGTTATACCTATTATAATTGGTGGTTTTGGGAATTGATTAGTTCCTTTAATATTAGGATCTCCTGATATATCTTTTCCTCGATTAAATAATTTAAGTTTTTGATTATTAATTCCTTCTTTATTTTTTTTATTAATTAGAGGTATTTTAAATATTGGAGTTGGTACAGGATGAACTGTTTATCCACCATTATCATTAGGTATATTTCATAGAGGAATTTCAGTTGATTTAAGAATTTTCTCTTTACATATAGCAGGCATATCTTCTATTTTAGGAGCTTTAAATTTTATTATTACTATTTTTTGTATATGATTTGGATTTAAATATATAGATAAATTATCTTTATTTATTTGATCAGTAGTAATTACTGCATTTTTATTAATTACATCTTTACCCGTTTTAGCAGGTGCTATTACAATATTATTAACAGATCGAAATTTAAATACTAGTTTTTTTGACCCTTCTGGTGGAGGTGACCCAATTTTATATCAACATTTGTTT.

###### Holotype ♀.

Alajuela, Sector San Cristobal, Sendero Huerta, 10.93, -85.372, 527 meters, caterpillar collection date: 09/vi/2012, wasp eclosion date: 04/vii/2012. Depository: CNC.

***Host data*.***Stenoma* Janzen414 (Depressariidae) feeding on *Ocoteaatirrensis* (Lauraceae).

***Caterpillar and holotype voucher codes*.** 12-SRNP-2366, DHJPAR0049931.

###### Paratypes.

None.

###### Etymology.

*Ascogasterluisdiegogomezi* is named to honor Sr. Luis Diego Gomez (RIP) for his strong biopolitical support of Janzen and Hallwachs in the initiation of their Costa Rican biodiversity research adventures in the 1960s–1970s.

**Figure 91.**
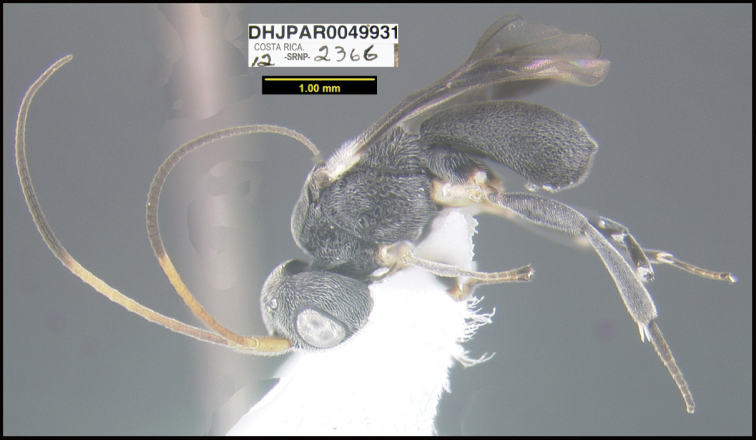
*Ascogasterluisdiegogomezi*, holotype.

#### *Chelonus* Panzer, 1806

*Chelonus* is worldwide in distribution with ca. 1000 described species and many more undescribed. Hosts include a wide range of lepidopterous families. Here we follow Kittel et al. (2015) and include *Microchelonus* in *Chelonus*. Papp (2016) described 25 species of *Microchelonus* and included a key to the species occurring in central and South America. Of these, all but one occurs in Costa Rica or Honduras, and it is likely that some of the species described below may turn out to be junior synonyms; which we will discover when, and if, the holotypes are barcoded. Judging by the similarly sized revision of *Leptodrepana* by Dadelahi et al. (2018), synonyms would be few. The species descriptions in Papp (2016) lack photographic images, and although the line drawings and descriptions are detailed, they do not provide enough diagnostic power for accurate identification. Though the types could be borrowed, this would dramatically retard the rate of description and still result in uncertainty. The types of Papp’s species are deposited in the following collections: Department of Entomology, Utah State University, Logan (USA); Hungarian Natural History Museum, Budapest (Hungary); USNM = United States National Museum of Natural History, Washington (USA); and Zoological Institute and Museum, The University, Lund (Sweden).

##### Chelonus alejandrozaldivari

Taxon classificationAnimaliaHymenopteraBraconidae

Sharkey sp. nov.

http://zoobank.org/055B5F06-2534-40FF-B504-2D129C0B0E55

Figure 92

###### Diagnostics.

BOLD:ACO0288. Consensus barcode. TACATTATATTTTATTTTTGGTATTTGATCTGGAATATTTGGTTTATCTTTAAGTTTACTTATTCGAATAGAATTAAGGATAGGAGGTAGATTATTATTAAATGATCAATTATATAATAGTTTAGTAACATTRCATGCTTTTGTAATAATTTTTTTTATAGTTATACCTGTTATAATTGGAGGYTTTGGTAATTGATTAATTCCTTTAATATTAGGATTACCTGATATAGCTTTCCCTCGTATAAATAATATAAGATTTTGGTTATTAATTCCTTCATTAACTTTATTAATTTTAAGGGGATTTATTAATATAGGGGTAGGTACAGGTTGAACTGTTTATCCTCCTTTATCTTCTTTAATTGGTCATGGAGGAATTTCTGTTGATTTATCTATTTTTTCTTTACATTTAGCAGGAGCTTCTTCTATTATAGGCGCAATTAATTTTATTGTTACTATTTTAAATACTTGAATATTAAGTAAATATTTAGATAAGTTTCCTTTATTTTCATGATCTGTTTTTATTACTGCTATTTTATTATTATTATCATTRCCAGTATTAGCYGGTGCAATTACTATATTATTAAGAGATCGTAATTTAAATACTAGATTTTTTGATCCTTCTGGTGGAGGRGAYCCTGTTTTATATCAACATTTATTT.

###### Holotype ♂.

Guanacaste, Pailas Dos, PL12-3, 10.7631, -85.3344, 820 meters, 05-18/xii/2014, Malaise trap PL12-3A. Depository: CNC.

***Host data*.** None.

***Holotype voucher code*.**BIOUG44692-B02.

###### Paratypes.

BIOUG43804-H09, BIOUG44551-E11. Depository: CNC.

###### Etymology.

*Chelonusalejandrozaldivari* is named to honor Dr. Alejandro Zaldivar-Riveron for his research in braconid phylogeny and taxonomy.

**Figure 92.**
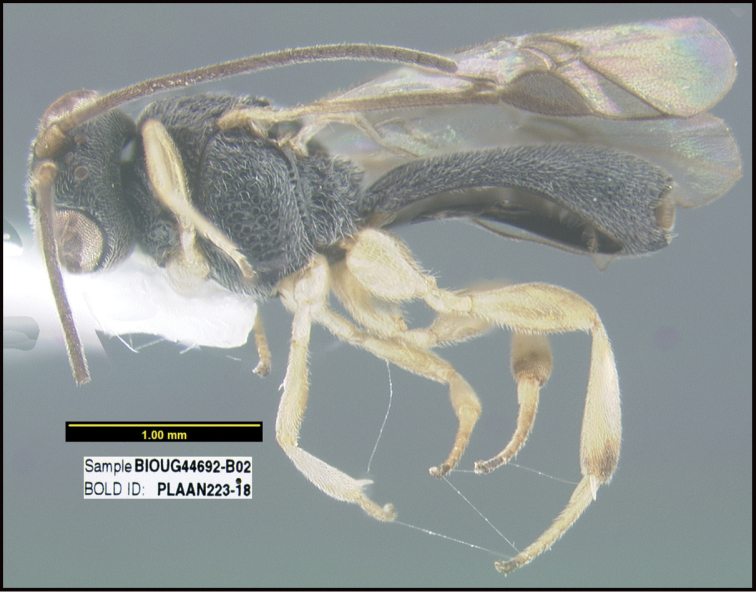
*Chelonusalejandrozaldivari*, holotype.

##### Chelonus gustavogutierrezi

Taxon classificationAnimaliaHymenopteraBraconidae

Sharkey sp. nov.

http://zoobank.org/509D89B0-0D2D-4B8B-9F02-C04931C583BD

Figure 93

###### Diagnostics.

BOLD:AAA6603. Consensus barcode. AATATTATATTTTATATTTGGAATTTGATGTGGRATAATAGGATTGTCATTAAGAGTAWTAATTCGTATAGAATTAAGAAGAGTAATAAGATTATTTTATAATGATCAATTATATAATAGAATTGTTACTATGCATGCTTTTATTATAATTTTTTTTATAGTTATACCATTAATAATTGGTGGATTTGGRAATTGATTAATTCCACTAATATTAGGATTATCAGATATAATTTTTCCTCGAATAAATAATATAAGATTTTGATTGTTACTACCTTCAATTATTTTATTAATTATAGGTGGATTTGTTAATATAGGAGCAGGTACAGGATGAACAGTTTATCCACCTTTATCATTATTAATAGGTCATAGAGGAATCTCAGTAGATTTATCTATTTTTTCTTTACATTTAGCTGGTATATCTTCAATTATAGGTTCAATTAATTTTATTGTTACTATTATTAATACATGATTAAATATAAAATATATGGATAAATATCCTTTATTTGTTTGATCAGTATTTATTACTACTATTTTATTATTATTATCTTTGCCAGWTTTGGCTGGAGCAATTACTATATTATTAAGTGATCKAAATTTAAATACTAGTTTTTTTGATCCTTCAGGTGGTGRTGATCCAGTATTATATCAACAYTTATTT.

###### Holotype ♂.

Alajuela, Sector Rincon Rain Forest, Flecha, 10.947, -85.315, 491 meters, caterpillar collection date: 15/ix/2009, wasp eclosion date: 27/ix/2009. Depository: CNC.

***Host data*.***Omiodescuniculalis* (Crambidae) feeding on *Gliricidiasepium* (introduced) (Fabaceae), and a variety of other woody species.

***Caterpillar and holotype voucher codes*.** 09-SRNP-80021: DHJPAR0037990.

###### Paratypes.

Host = *Omiodescuniculalis*, Pyralidae 09-SRNP-71481: DHJPAR0037185, DHJPAR0037164, DHJPAR0029095, DHJPAR0029096, DHJPAR0029097, DHJPAR0029099, DHJPAR0029100, DHJPAR0029084, DHJPAR0029083, DHJPAR0029081, DHJPAR0029080, DHJPAR0029092, DHJPAR0029091, DHJPAR0029090, DHJPAR0029089, DHJPAR0029101, DHJPAR0037985. Depository: CNC.

###### Etymology.

*Chelonusgustavogutierrezi* is named to honor Dr. Gustavo Gutierrez of the Universidad de Costa Rica for his enthusiastic Universidad de Costa Rica support for BioAlfa development.

**Figure 93.**
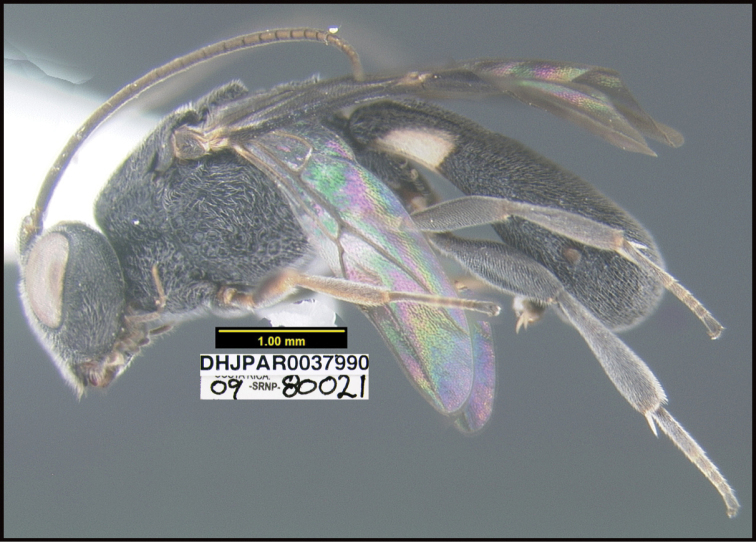
*Chelonusgustavogutierrezi*, holotype.

##### Chelonus gustavoinduni

Taxon classificationAnimaliaHymenopteraBraconidae

Sharkey sp. nov.

http://zoobank.org/5F5D038F-0679-43E2-8451-26A7DC2AC374

Figure 94

###### Diagnostics.

BOLD:ADA0356. Consensus barcode. GTATTATATTTTATTTTTGGTATTTGATCTGGAGTTTTAGGATTATCATTAAGATTAATTTTACGAATAGAATTAAGAGTGTTAGGAAGGTTATTAAAAAATGATCAATTATATAATAGGGTAGTTACTTTACATGCTTTTGTAATAATTTTTTTTATGGTTATACCAGTAATAATTGGAGGATTTGGAAATTGATTAGTTCCATTAATATTAGGATTACCAGATATAGCTTTTCCACGAATAAATAATATAAGATTTTGGTTATTAATTCCTTCATTAATAATGTTATTATTGAGAAGATTTGTAAATATAGGTGTAGGTACAGGATGAACGGTTTATCCACCATTATCTTCATTAATAGGACATGGTGGTATTTCAGTAGATTTATCAATTTTTTCTTTACATTTAGCAGGTGCATCTTCTATTATGGGGGCAATTAATTTTATTGTAACGGTAATAAATACTAATTTTAAGATTGGGTTTATAGATAAATTTCCATTATTTGTTTGATCAGTTTTAATTACGGCTATTTTATTATTATTATCATTGCCAGTATTAGCCGGTGCTATTACTATATTATTA.

###### Holotype ♂.

Guanacaste, Sector San Cristobal, Estación San Gerardo, 10.8801, -85.389, 575 meters, 22‒29/vi/2015, Malaise trap. Depository: CNC.

***Host data*.** None.

***Holotype voucher code*.**BIOUG28148-H10.

###### Paratypes.

None.

###### Etymology.

*Chelonusgustavoinduni* is named to honor Sr. Gustavo Induni of SINAC for his enthusiastic SINAC support for BioAlfa development.

**Figure 94.**
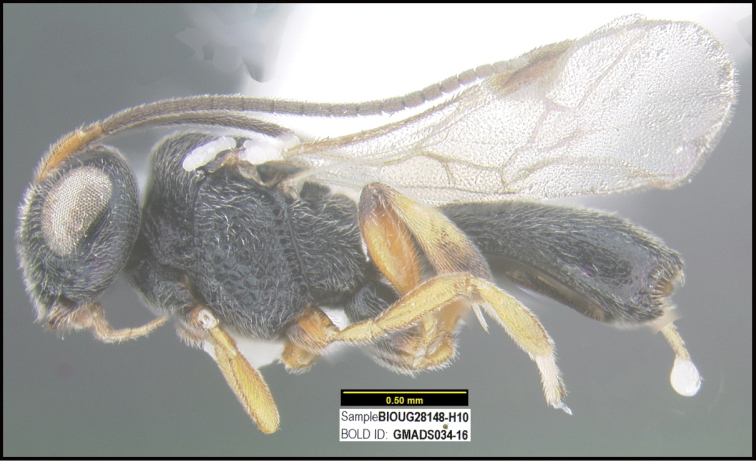
*Chelonusgustavoinduni*, holotype.

##### Chelonus harryramirezi

Taxon classificationAnimaliaHymenopteraBraconidae

Sharkey sp. nov.

http://zoobank.org/2CA5A1E2-5B44-4A98-90F5-83EE43600D54

Figure 95

###### Diagnostics.

BOLD:AAE2698. Consensus barcode. AATATTATATTTTATTTTTGGAATTTGATGKGGAATGTTAGGTTTATCATTAAGAGTTATAATTCGTATAGAATTAAGAAGGACATTAAGTTTATTCTATAATGATCAATTATATAATAGAATTGTTACTATACATGCTTTTATTATAATTTTTTTTATAGTTATACCATTAATAATTGGAGGGTTTGGAAATTGATTAATTCCTTTAATATTAGGTTTATCAGATATAATTTTTCCTCGTATAAATAATATAAGATTTTGATTATTAATTCCTTCAATTATTTTATTAATTATAGGGGGTTTTGTAAATACAGGGGCAGGTACAGGATGAACAGTTTATCCTCCTTTATCATTATTGATAGGTCATAGAGGGGTATCAGTAGATTTATCAATCTTTTCTTTACATTTAGCTGGAGCATCATCTATTATGGGTTCTATTAATTTTATTGTAACTATTATTAATACTTGGTTGAATATAAAATATATAGATAAATATCCTTTATTTGTTTGATCTGTTTTTATTACCACAATTTTGTTATTATTATCTTTACCAGTTTTAGCTGGGGCAATTACTATATTATTAAGAGATCGAAATTTAAATACTAGATTTTTTGATCCTTCAGGGGGTGGAGAYCCAGTATTATATCAACATTTATTT.

###### Holotype ♂.

Alajuela, Sector Rincon Rain Forest, Quebrada Bambu, 10.93, -85.252, 109 meters, caterpillar collection date: 14/ii/2013, wasp eclosion date: 01/iii/2013. Depository: CNC.

***Host data*.***Omiodeshumeralis* (Crambidae) feeding on *Ingasapindoides* (Fabaceae).

***Caterpillar and holotype voucher codes*.** 13-SRNP-75542, DHJPAR0051927.

###### Paratypes.

Hosts = *Omiodeshumeralis*. DHJPAR0023700, DHJPAR0020624, DHJPAR0020623, DHJPAR0020802, DHJPAR0038012, DHJPAR0038013, DHJPAR0038014, DHJPAR0020804, DHJPAR0046938, DHJPAR0051326, DHJPAR0051328, DHJPAR0051330, DHJPAR0055229, DHJPAR0054564, DHJPAR0054938. Depository: CNC.

###### Etymology.

*Chelonusharryramirezi* is named to honor Sr. Harry Ramirez of GDFCF and ACG for his many decades as a dedicated inventory parataxonomist, Malaise and mouse live-trapper, and specimen processor for ACG.

**Figure 95.**
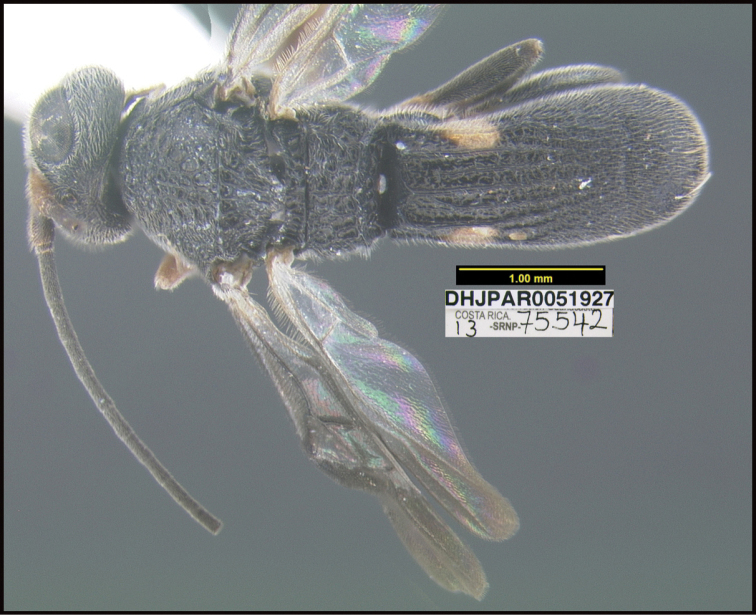
*Chelonusharryramirezi*, holotype.

##### Chelonus hartmanguidoi

Taxon classificationAnimaliaHymenopteraBraconidae

Sharkey sp. nov.

http://zoobank.org/747BBCFD-2013-449B-96DF-926A6A0E8CA0

Figure 96

###### Diagnostics.

BOLD:AAD1994. Consensus barcode. AATATTATATTTTATTTTTGGAATTTGGTGTGGAATAATAGGATTATCCTTAAGAGTTATGATTCGTATAGAGTTAAGAAGAACAATAAGATTATTTTGTAATGATCAATTATATAATAGAATTGTTACTATACATGCTTTTATTATAATTTTTTTTATAGTTATACCATTAATAATTGGGGGGTTTGGAAATTGATTAATYCCTTTAATATTAGGTTTATCAGATATAATTTTTCCTCGTATAAATAATATAAGATTTTGATTATTAATCCCTTCAATTATTTTATTAATTATAGGAGGATTTGTAAATATAGGTGCAGGTACAGGATGAACAATTTATCCGCCTTTATCATTATTAATAGGTCATAGTGGGATTTCAGTAGATTTATCAATTTTTTCTTTACATTTAGCTGGGGCATCATCTATTATAGGTTCAATTAATTTTATTGTGACTATTATTAATACTTGATTAAATATAAAGTATATGGATAAATATCCTTTATTTGTTTGATCTGTTTTTATTACTACAATTTTATTATTATTATCTTTRCCTGTTTTAGCTGGGGCAATTACTATACTATTAAGAGATCGAAATTTAAATACTAGATTTTTTGATCCTTCAGGGGGTGGGGACCCAGTATTATACCAACATTTATTT.

###### Holotype ♀.

Alajuela, Sector Rincon Rain Forest, Palomo, 10.962, -85.28, 96 meters, caterpillar collection date: 19/i/2011, wasp eclosion date: 10/ii/2011. Depository: CNC.

***Host data*.***Omiodes* Janzen05 (Crambidae) feeding on *Entadagigas* (Fabaceae).

***Caterpillar and holotype voucher codes*.** 11-SRNP-67022, DHJPAR0042543.

###### Paratypes.

Hosts = *Omiodes* Janzen03, spiloBioLep01 BioLep577, *Piletosomathialis*, *Omiodes* Janzen05, *Phostriacyrisalis*. DHJPAR0029154, DHJPAR0029166, DHJPAR0035222, DHJPAR0035307, DHJPAR0035308, DHJPAR0035312, DHJPAR0035313, DHJPAR0036398, DHJPAR0046801, DHJPAR0046947, DHJPAR0052896, DHJPAR0053677, DHJPAR0053686, DHJPAR0054439, DHJPAR0054570, DHJPAR0054571, DHJPAR0056957, DHJPAR0056958, DHJPAR0056963, DHJPAR0056968, DHJPAR0056969. Depository: CNC.

###### Etymology.

*Chelonushartmanguidoi* is named to honor Sr. Hartman Guido of ICE for his years of crucial support for the ACG-ICE-GDFCF bioinventory project of PL12 geothermal site at Pailas II on the boundary of ACG.

**Figure 96.**
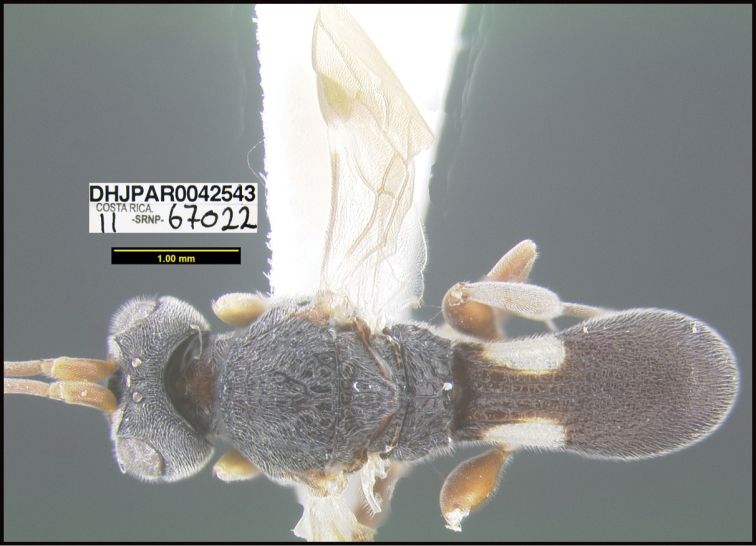
*Chelonushartmanguidoi*, holotype.

##### Chelonus hazelcambroneroae

Taxon classificationAnimaliaHymenopteraBraconidae

Sharkey sp. nov.

http://zoobank.org/4D48BB03-76E3-4A6D-B32C-8DF5D74C71EE

Figure 97

###### Diagnostics.

BOLD:AAE9128. Consensus barcode. TATATTATATTTTATTTTTGGAGTTTGAAGTGGGATAATAGGTTTATCATTAAGAGTTATAATTCGTATAGAATTAAGGAGTGTTATAAGATTATTTAGTAATGATCAGTTATATAATAGGATTGTTACAATACATGCCTTTGTAATAATTTTTTTTATAGTTATACCTTTAATAATTGGAGGATTTGGAAATTGATTAGTTCCTTTAATATTAGGATTATCTGATATAATTTTCCCTCGAATAAATAATATAAGATTTTGATTATTAATTCCTTCACTTATTTTATTAATTATAGGTGGATTTGTTAATATAGGTGCTGGGACAGGATGAACAGTTTATCCTCCTTTATCATTATTAATAGGTCATAGAGGAATTTCAGTAGATTTATCTATTTTTTCTTTACATTTAGCTGGAATATCATCAATTATAGGTTCAATTAATTTTATTGTTACTATTATAAATACTTGATTACATGTAAAATATATAGATAAATATCCATTATTTGTTTGATCTGTATTTATTACAACTATTTTATTATTGTTATCATTGCCAGTTTTAGCAGGAGCAATTACTATATTATTAAGTGATCGAAATTTAAATACAAGATTTTTTGATCCATCAGGTGGAGGTGATCCAGTATTATATCAACATTTATTT.

###### Holotype ♀.

Alajuela, Sector San Cristobal, Sendero Huerta, 10.93, -85.372, 527 meters, caterpillar collection date: 27/vii/2012, wasp eclosion date: 25/viii/2012. Depository: CNC.

***Host data*.**Crambidae: *Prenesta* scyllalisDHJ02 (Crambidae) feeding on *Allomarkgrafiaplumeriiflora* (Apocynaceae).

***Caterpillar and holotype voucher codes*.** 12-SRNP-3201, DHJPAR0049930.

###### Paratypes.

Host = *Prenesta* scyllalisDHJ02: DHJPAR0030990, DHJPAR0030989, DHJPAR0030988, DHJPAR0022199. Depository: CNC.

###### Etymology.

*Chelonushazelcambroneroae* is named to honor Sra. Hazel Cabronero for her many years as a as a dedicated inventory parataxonomist and specimen preparer in BioLep for the ACG bioinventory.

**Figure 97.**
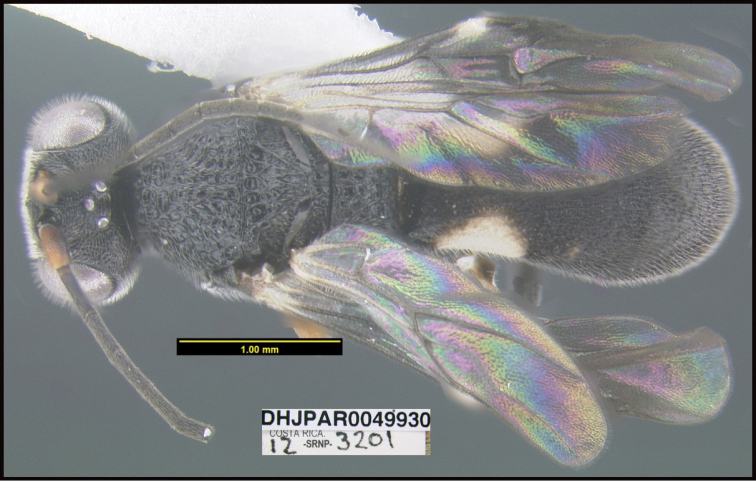
*Chelonushazelcambroneroae*, holotype.

##### Chelonus iangauldi

Taxon classificationAnimaliaHymenopteraBraconidae

Sharkey sp. nov.

http://zoobank.org/81CDE458-F370-46E0-ABE6-EBC9DFF3009E

Figure 98

###### Diagnostics.

BOLD:AAD4709. Consensus barcode. AATATTATATTTTATTTTTGGAGTTTGAAGAGGRATAATAGGGTTATCTTTAAGAGTAATAATTCGTATAGAATTAAGAAGAGTAATAAGATTATTTTATAATGATCAATTATATAATAGAATTGTAACGATACATGCTTTTATTATAATTTTTTTTATAGTTATACCATTAATAGTAGGAGGATTTGGAAATTGATTAATTCCTTTAATATTAGGATTATCTGATATAATTTTTCCTCGAATAAATAATATAAGATTTTGATTATTAATTCCTTCAATTATTTTATTAATTATAGGTAGATTTGTTAATATAGGAGCTGGAACAGGGTGAACAGTATATCCTCCATTATCATTATTAGTAAGACATAGAGGAATTTCTGTAGATTTATCTATTTTTTCTTTACATTTAGCTGGAATATCATCAATTATAGGTTCAATTAATTTTATTGTTACTATTTTAAATACTTGAATATATAAAAAATATATAGATAAATATCCATTATTTGTGTGATCTATTTTTATTACAACAATTTTATTATTATTATCATTACCAGTTTTGGCTGGTGCAATTACTATATTATTAAGTGATCGAAATTTAAATACAAGATTTTTTGATCCATCAGGAGGAGGAGATCCAGTATTATACCAACATTTATTT.

###### Holotype♀.

Alajuela, Sector San Cristobal, Quebrada Garcia, 10.861, -85.426, 495 meters, caterpillar collection date: 20/vii/2009, wasp eclosion date: 11/x/2009. Depository: CNC.

***Host data*.***Prenesta* scyllalisDHJ01 (Crambidae) feeding on *Forsteroniaspicata* (Apocynaceae).

***Caterpillar and holotype voucher codes*.** 09-SRNP-4322, DHJPAR0037165.

###### Paratypes.

Host = *Prenesta* scyllalisDHJ01: DHJPAR0037160, DHJPAR0037183, DHJPAR0036726, DHJPAR0037168, DHJPAR0037175, DHJPAR0037169, DHJPAR0040017. Depository: CNC.

###### Etymology.

*Chelonusiangauldi* is named to honor Dr. Ian Gauld (RIP) for his decades of highly enthusiastic support for all things taxonomic and bioinventory of Costa Rica in general and ACG specifically, with special emphasis on wasps in the family Ichneumonidae.

**Figure 98.**
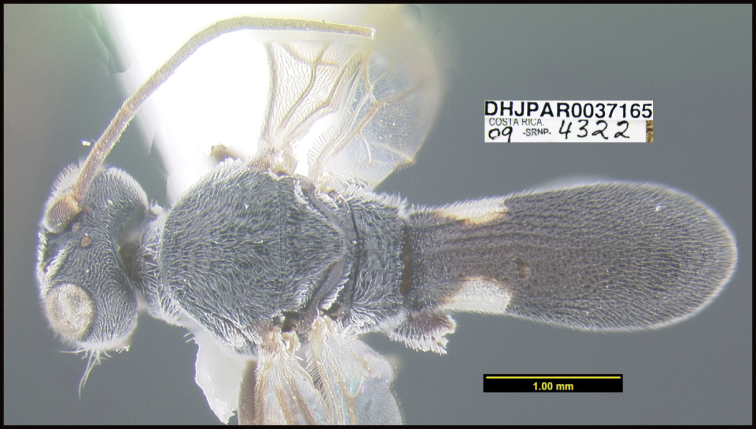
*Chelonusiangauldi*, holotype.

##### Chelonus isidrochaconi

Taxon classificationAnimaliaHymenopteraBraconidae

Sharkey sp. nov.

http://zoobank.org/0B58ED94-6FBE-4F68-907A-604D45333A33

Figure 99

###### Diagnostics.

BOLD:AAC8260. Consensus barcode. AATATTATATTTTGTATTTGGAATTTGAAGAGGTATAATTGGGYTATCATTAAGAGTTATAATTCGTATAGAATTAAGTAGGGTAATAAGATTATTTTCTAAYGATCAATTGTATAATAGAATTGTGACTATACATGCTTTTATTATAATTTTTTTTATAGTAATACCTTTRATAATTGGRGGTTTTGGAAATTGGYTAATTCCTTTAATATTAGGATTATCTGATATAATTTTTCCTCGAATAAATAATATAAGATTTTGGTTATTAATTCCTTCAATTATTTTATTAATTATAGGCGGATTTGTAAATATAGGAGCTGGGACAGGATGAACAGTGTATCCYCCTTTATCTTTATTAATAGGTCATAGAGGGGTGTCAGTTGATTTATCTATTTTTTCTTTACATTTAGCTGGGGCTTCATCAATTATAGGKTCAATTAATTTTATTGTTACTATTATAAATACTTGGTTACATATTAAGTATATAGATAAATAYCCATTATTTGTTTGATCTGTATTTATTACAACTATTCTTTTATTATTATCATTACCAGTTTTAGCTGGAGCAATTACTATATTATTAAGTGATCGAAATTTAAATACAAGATTTTTTGAYCCTTCAGGAGGGGGRGAYCCTGTATTGTATCAACATTTATTT.

###### Holotype ♀.

Alajuela, Sector San Cristobal, Estación San Gerardo, 10.88, -85.389, 575 meters, caterpillar collection date: 26/v/2012, wasp eclosion date: 16/vi/2012. Depository: CNC.

***Host data*.***Parasteniaretractalis* (Crambidae) feeding on *Trichostigmaoctandrum* (Phytolaccaceae).

***Caterpillar and holotype voucher codes*.** 12-SRNP-2158, DHJPAR0049008.

###### Paratypes.

Host = *Parasteniaretractalis*: DHJPAR0037159, DHJPAR0037174, DHJPAR0037181, DHJPAR0037166, DHJPAR0037172, DHJPAR0037173, DHJPAR0037162, DHJPAR0037176, DHJPAR0037179, DHJPAR0048983, DHJPAR0048984, DHJPAR0048986, DHJPAR0048987, DHJPAR0048988. Depository: CNC.

###### Etymology.

*Chelonusisidrochaconi* is named to honor Sr. Isdro Chacon of BioAlfa for his decades of dedicated support and action for the taxonomy and biodiversity of Costa Rica in general and specifically INBio, Costa Rica’s Instituto Nacional de Biodiversidad and the Museo Nacional de Costa Rica.

**Figure 99.**
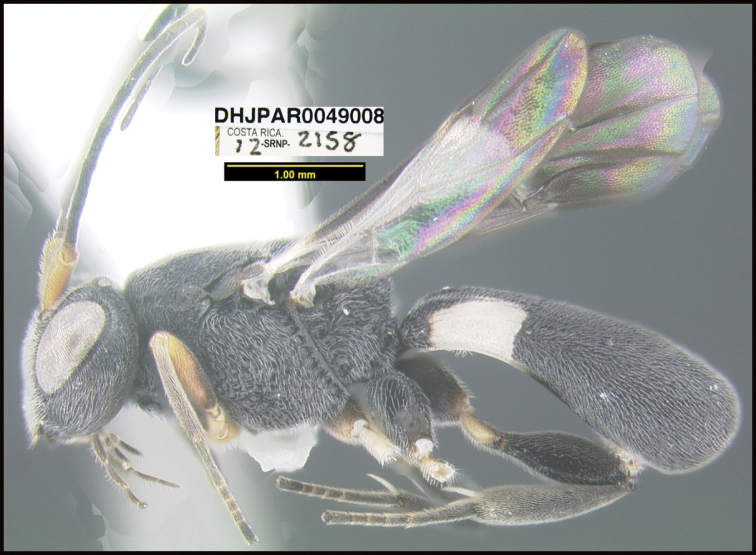
*Chelonusisidrochaconi*, holotype.

##### Chelonus janecheverriae

Taxon classificationAnimaliaHymenopteraBraconidae

Sharkey sp. nov.

http://zoobank.org/456F0E51-57E4-45D8-AB2D-DDCBE03FB7A0

Figure 100

###### Diagnostics.

BOLD:AAL5555. Consensus barcode. GTAATAGGCTTATCTTTAAGTGTAATAATTCGTATAGAATTAAGAAGTGTAATAAGATTATTTTATAATGATCAATTATATAATAGAATTGTAACTATACATGCTTTTATTATAATTTTTTTTATAGTTATGCCTTTAATAATTGGGGGGTTTGGAAATTGATTAATTCCTTTAATGTTAGGTTTATCTGATATAATTTTTCCTCGAATAAATAATATAAGATTTTGATTATTAATTCCTTCAATTATTTTATTAATTATAGGAGGATTTGTTAATATAGGGGCTGGCACAGGATGAACAGTTTATCCGCCATTATCATTATTAATAGGTCATAGTGGTGTTTCAGTAGATTTATCTATTTTTTCTTTACATTTGGCAGGAGCCTCATCTATTATAGGTTCAATTAATTTTATTGTGACTATTATAAATACTTGGATGTATTATAAATACATAGATAAATATCCATTATTTGTTTGATCAGTATTTATTACAACTATTTTATTATTATTATCATTACCAGTTTTAGCTGGTGCAATTACTATATTATTAAGAGACCGAAATTTGAACACAAGATTTTTTGATCCATCAGGGGGGGGGGG.

###### Holotype ♀.

Alajuela, Sector Rincon Rain Forest, San Lucas, 10.918, -85.303, 320 meters, caterpillar collection date: 02/ii/2010, wasp eclosion date: 20/ii/2010. Depository: CNC.

***Host data*.** crambidJanzen01 Janzen27 (Crambidae) feeding on *Calathealutea* (Marantaceae).

***Caterpillar and holotype voucher codes*.** 10-SRNP-40463, DHJPAR0039179.

###### Paratypes.

None.

###### Etymology.

*Chelonusjanecheverriae* is named to honor Sra. Jane Echeverri for her lifetime support of Sr. Gustavo Echeverri in their high-quality management of Costa Rican farms and their financial support for decades of ACG’s Programa de Educación Biológica for the school children in the schools surrounding ACG.

**Figure 100.**
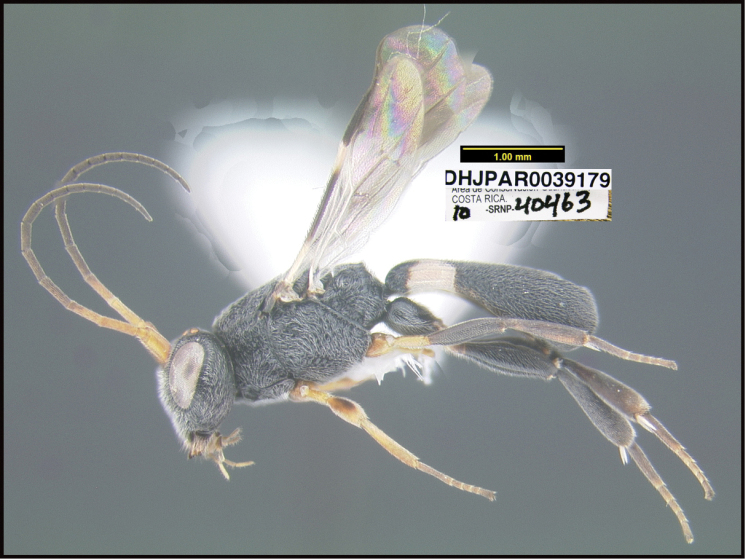
*Chelonusjanecheverriae*, holotype.

##### Chelonus jeffmilleri

Taxon classificationAnimaliaHymenopteraBraconidae

Sharkey sp. nov.

http://zoobank.org/4E814E27-51E7-4E8F-B84E-9787B9C873EB

Figure 101

###### Diagnostics.

BOLD:ACF0845. Consensus barcode. AATATTATATTTTTTATTTGGAATTTGAAGTGGGATAATAGGTTTATCTTTAAGTGTAATAATTCGTATAGAATTAAGAAGTGTAATAAGATTATTTTATAATGATCAATTATATAATAGAATTGTAACTATACATGCTTTTATTATAATTTTTTTTATAGTTATGCCTTTAATAATTGGGGGATTTGGAAATTGATTAATCCCTTTAATATTAGGATTATCTGATATAATTTTTCCTCGAATAAATAATATAAGATTTCGATTATTAATTCCTTCAATTATTTTATTAATTATAGGAGGATTCGTTAATATAGGGGCTGGTACGGGATGAACAGTTTATCCTCCATTATCATTATTAATAGGTCATAGTGGAGTTTCAGTAGATTTATCTATTTTTTCTTTACATTTGGCAGGGGCTTCATCTATTATAGGTTCAATTAATTTTATTGTGACTATTATAAATACTTGGTTACATTATAAATATATAGATAAATATCCATTATTTGTTTGATCAGTATTTATTACAACTATTTTATTATTATTATCATTACCAGTTTTAGCTGGTGCAATTACTATATTATTAAGAGATCGAAATTTAAATACAAGATTTTTTGATCCATCAGGGGGAGGAGACCCAGTATTATATCAGCATTTATTT.

###### Holotype ♂.

Guanacaste, Sector Del Oro, Quebrada Trigal, 11.027, -85.495, 290 meters, caterpillar collection date: 04/viii/2009, wasp eclosion date: 18/viii/2009. Depository: CNC.

***Host data*.***Phostriaoajacalis* (Crambidae) feeding on *Merremiaumbellata* (Convolvulaceae).

***Caterpillar and holotype voucher codes*.** 09-SRNP-22201, DHJPAR0040375.

###### Paratypes.

Host = *Eulepte* Solis15: DHJPAR0042859, DHJPAR0042862. Depository: CNC.

###### Etymology.

*Chelonusjeffmilleri* is named to honor Dr. Jeff Miller for his enthusiastic and productive interest in bringing the ACG caterpillars and their moths and butterflies into public view through book publications and public presentations.

**Figure 101.**
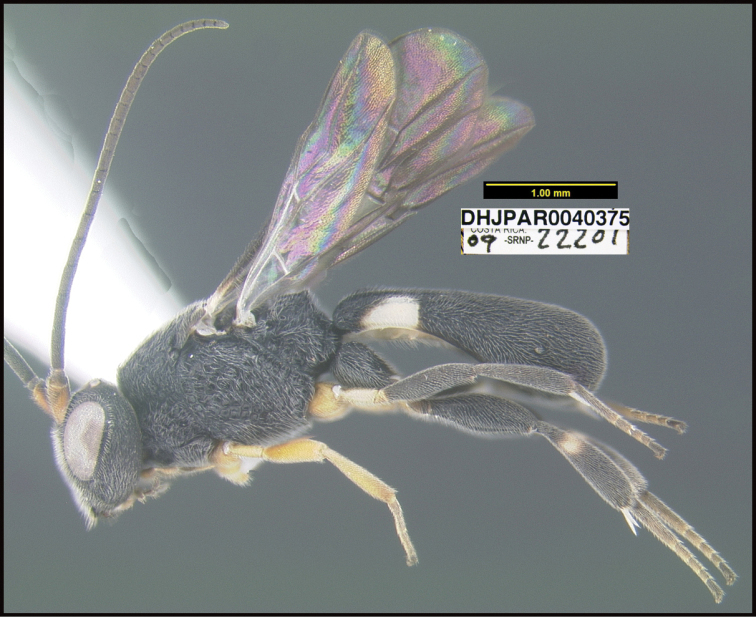
*Chelonusjeffmilleri*, holotype.

##### Chelonus jennyphillipsae

Taxon classificationAnimaliaHymenopteraBraconidae

Sharkey sp. nov.

http://zoobank.org/C3C57E01-AA1B-4E55-A265-47CDDF468030

Figure 102

###### Diagnostics.

BOLD:ACL6996. Consensus barcode. AATGTTATATTTTTTATTTGGAATTTGGAGTGGGATAATAGGTTTATCTTTGAGTGTAATAATTCGTATAGAATTAAGAAGTGTAATAAGATTATTTTATAATGATCAATTATATAATAGAATTGTAACTATACATGCTTTTATTATAATTTTTTTTATAGTTATGCCTTTAATAATTGGGGGATTTGGGAATTGGTTAATCCCTTTAATATTAGGATTATCTGATATAATTTTTCCTCGAATAAATAATATAAGATTTTGGTTACTAATTCCTTCAATTATTTTATTAATTATAGGAGGATTTGTTAATATGGGGGCTGGTACGGGATGAACAGTTTATCCTCCATTATCATTATTAATAGGTCATAGTGGAGTTTCAGTAGATTTATCTATTTTTTCTTTACATTTGGCAGGGGCTTCATCTATTATAGGTTCAATTAATTTTATTGTGACTATTATAAATACTTGATTGCATTTTAAATATATAGATAAATATCCATTATTTGTTTGATCAGTATTTATTACAACTATTTTATTATTATTATCATTACCAGTTTTAGCTGGTGCAATTACTATATTATTAAGAGATCGAAATTTAAATACAAGATTTTTTGATCCATCAGGGGGAGGGGATCCAGTATTATACCAGCATTTATTT.

###### Holotype ♀.

Alajuela, Sector Rincon Rain Forest, Malaguenya, 10.956, -85.284, 221 meters, caterpillar collection date: 14/x/2013, wasp eclosion date: 30/x/2013. Depository: CNC.

***Host data*.***Eulepte* Janzen12 (Crambidae) feeding on *Vitexcooperi* (Lamiaceae).

***Caterpillar and holotype voucher codes*.** 13-SRNP-79751, DHJPAR0053682.

###### Paratypes.

None.

###### Etymology.

*Chelonusjennyphillipsae* is named to honor Dr. Jenny Phillips of BioAlfa for her decades of enthusiastic taxonomic and administrative support of INBio, parataxonomists, and all matters biodiversity of Costa Rica coupled with intensely developing BioAlfa of Costa Rica.

**Figure 102.**
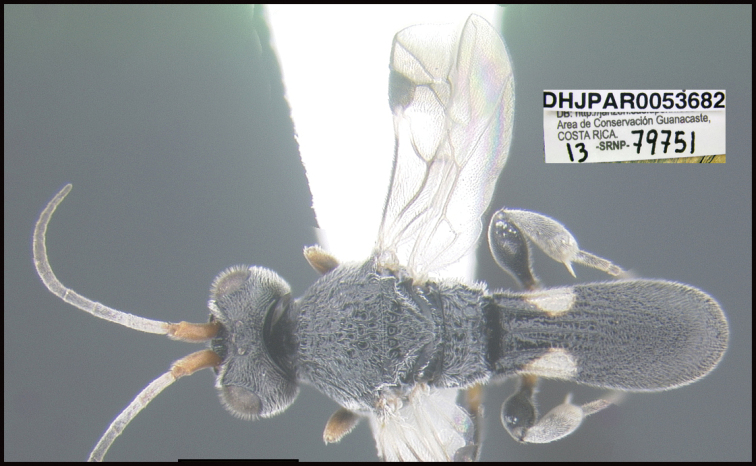
*Chelonusjennyphillipsae*, holotype.

##### Chelonus jeremydewaardi

Taxon classificationAnimaliaHymenopteraBraconidae

Sharkey sp. nov.

http://zoobank.org/84B46263-B836-4162-B57F-CE88A37EFE72

Figure 103

###### Diagnostics.

BOLD:AAB1653. Consensus barcode. AATATTATATTTTTTATTTGGGATTTGAAGTGGAATAATAGGTYTATCTTTAAGTGTAATAATTCGTATAGAATTAAGAAGTGTAATRAGATTATTTTATAATGATCAATTATATAATAGAATTGTAACTATACATGCTTTTATTATAATTTTTTTTATAGTTATGCCTTTAATAATTGGGGGATTTGGAAATTGATTAATCCCTTTAATATTAGGATTATYTGATATAATTTTTYCTCGAATAAATAATATAAGATTTTGATTATTAATTCCTTCAATTATTTTATTAATTATAGGAGGATTTGTTAATATAGGGGCTGGWACAGGGTGAACAGTTTATCCYCCATTATCATTATTAATAGGTCATAGTGGAGTTTCAGTAGATTTATCTATTTTTTCTTTACATTTGGCAGGGGYTTCATCTATTATAGGTTCAATTAATTTTATTGTGACTATTATAAATACTTGRYTACATTATAAATATATAGATAAATATCCGTTATTTGTTTGGTCAGTATTTATTACAACTATTTTATTATTATTATCATTACCAGTTTTAGCTGGTGCAATTACTATATTATTAAGAGATCGAAAYTTAAATACAAGATTTTTTGATCCATCAGGGGGAGGAGAYCCAGTATTATATCAGCATTTATTT.

###### Holotype ♂.

Guanacaste, Sector Pitilla, Bullas, 10.987, -85.385, 440 meters, caterpillar collection date: 29/v/2014, wasp eclosion date: 15/vi/2014. Depository: CNC.

***Host data*.***Syllepismarialis* (Crambidae) feeding on *Allophyluspsilospermus* (Sapindaceae).

***Caterpillar and holotype voucher codes*.** 14-SRNP-70940, DHJPAR0055376.

###### Paratypes.

Hosts = Crambidae: *Asturodesfimbriauralis*, *Asturodesjunkoshimurae*, *Ceratociliasixolalis*, *Desmia* benealisDHJ02, *Desmia* Janzen03, *Desmia* ploralisDHJ10, *Desmia* Solis100, *Eulepte* Solis15, *Herpetogrammasalbialis*, *Herpetogramma* Solis10, *Leucochromodes* melusinalisDHJ02, *Microthyris* prolongalisDHJ02, *Omiodesfulvicauda*, *Patania* Solis03, *Patania* Solis04, *Phostriacyrisalis*, *Psara* obscuralisDHJ01, *Salbiacassidalis*, spiloBioLep01 BioLep311, spiloJanzen01 Janzen14DHJ02, *Syllepismarialis*. DHJPAR0029069, DHJPAR0029070, DHJPAR0029117, DHJPAR0035309, DHJPAR0035311, DHJPAR0035315, DHJPAR0036400, DHJPAR0037182, DHJPAR0037862, DHJPAR0037979, DHJPAR0037981, DHJPAR0037982, DHJPAR0037989, DHJPAR0039121, DHJPAR0039502, DHJPAR0040364, DHJPAR0040368, DHJPAR0042084, DHJPAR0042093, DHJPAR0042096, DHJPAR0042857, DHJPAR0042860, DHJPAR0045402, DHJPAR0045403, DHJPAR0045408, DHJPAR0046798, DHJPAR0046935, DHJPAR0048091, DHJPAR0048092, DHJPAR0048093, DHJPAR0048094, DHJPAR0048097, DHJPAR0048104, DHJPAR0048169, DHJPAR0048973, DHJPAR0050072, DHJPAR0051337, DHJPAR0052887, DHJPAR0052891, DHJPAR0053674, DHJPAR0053681, DHJPAR0055370, DHJPAR0056949, DHJPAR0058149, DHJPAR0062180, DHJPAR0062181, DHJPAR0062182, DHJPAR0062183, DHJPAR0062184, DHJPAR0062185, DHJPAR0062186, DHJPAR0062187, DHJPAR0062188, DHJPAR0062189, DHJPAR0062190, DHJPAR0062191. Depository: CNC.

###### Other material.

A specimen registered in BOLD (NHM749343) from Belize shares the same BIN and is likely conspecific. It was not examined and is not part of the type series.

###### Etymology.

*Chelonusjeremydewaardi* is named to honor Dr. Jeremy deWaard for his many years laboring in execution and administration of the field and laboratory actions performed by the Centre for Biodiversity Genomics to rapidly and accurately DNA barcode tens of thousands of Costa Rican insects.

**Figure 103.**
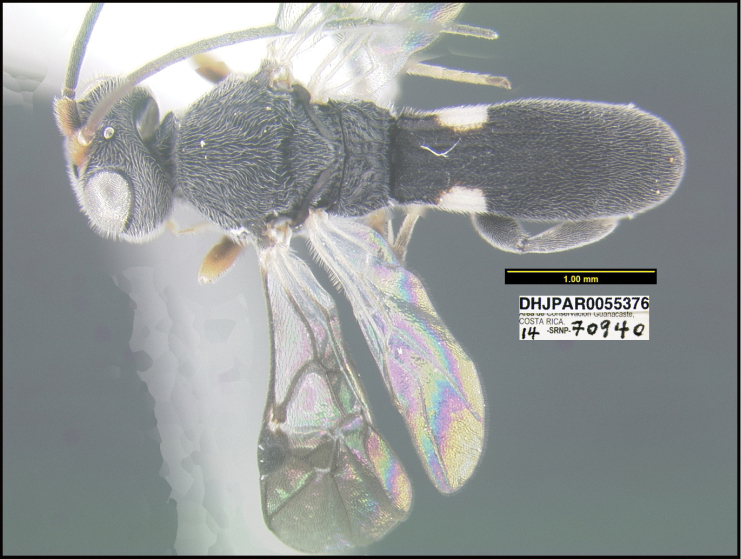
*Chelonusjeremydewaardi*, holotype.

##### Chelonus jessiehillae

Taxon classificationAnimaliaHymenopteraBraconidae

Sharkey sp. nov.

http://zoobank.org/467DEF33-1830-4D9D-BB45-CB4B8207B0D4

Figure 104

###### Diagnostics.

BOLD:AAW4588. Consensus barcode. TATATTATATTTTATATTTGGAATTTGATGTGGGATATTAGGGTTATCTTTAAGATTAATGATTCGTATGGAATTAAGAAGAGTAATAAGTTTATTTTTTAATGATCAATTATATAATAGAATTGTTACTATACATGCTTTTGTAATAATTTTTTTTATAGTAATACCAGTAATAATTGGGGGGTTTGGTAATTGATTAGTTCCTTTAATATTAGGGTTATCTGATATAATTTTTCCACGAATGAATAACATAAGTTTTTGGTTATTAATTCCTTCAATTATTTTATTAATTATAGGAGGATTTGTAAATATAGGGGCAGGTACTGGGTGGACAGTTTATCCACCATTATCTTTATTAATAGGTCATAGAGGGGTATCTGTTGATTTATCAATTTTTTCTTTACATTTAGCAGGTATATCATCAATTATAGGTTCAATTAATTTTATTGTTACTATTGTTAATACATGATTATTAATTAATTATATAGATAAATATCCTTTATTTGTATGGTCAGTGTTTATTACTACAATTTTATTATTATTATCTTTACCGGTTTTAGCTGGTGCAATTACTATATTATTAAGAGATCGTAATTTAAATACTAGATTTTTTGATCCTTCAGGAGGAGGGGATCCAGTTTTATACCAACATTTATTT.

###### Holotype ♂.

Alajuela, Sector Rincon Rain Forest, Sendero Albergue Crater, 10.849, -85.328, 980 meters, caterpillar collection date: 10/viii/2010, wasp eclosion date: 31/viii/2010. Depository: CNC.

***Host data*.** gelJanzen01 Janzen349 (Gelechiidae) feeding on *Mikaniabanisteriae* (Asteraceae).

***Caterpillar and holotype voucher codes*.** 10-SRNP-4459, DHJPAR0042088.

###### Paratypes.

None.

###### Etymology.

*Chelonusjessiehillae* is named to honor Mrs. Jesse Hill for her decades of support for ACG in general and specifically the Parataxonomist Program conducting the field aspects of the bioinventory of all of ACG.

**Figure 104.**
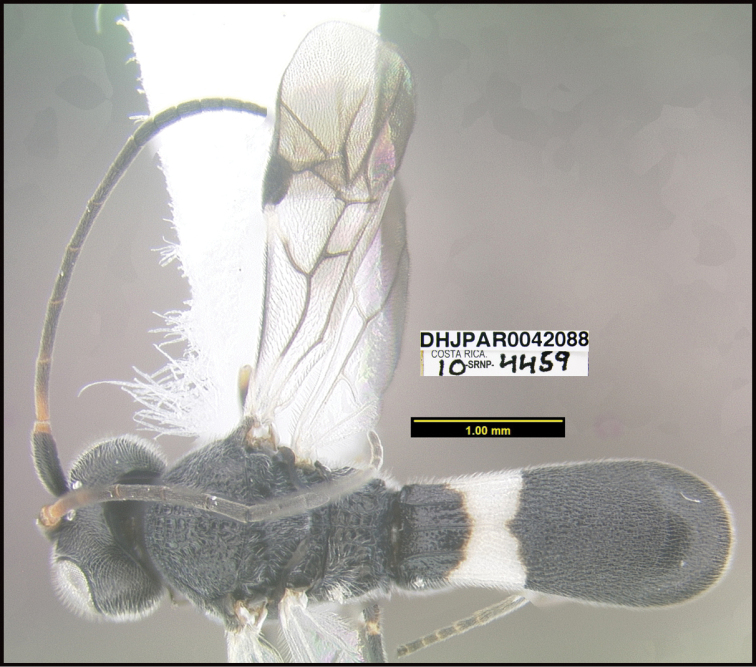
*Chelonusjessiehillae*, holotype.

##### Chelonus jesusugaldei

Taxon classificationAnimaliaHymenopteraBraconidae

Sharkey sp. nov.

http://zoobank.org/73C6675C-BC13-47D0-8E0E-BD34271B5D5B

Figure 105

###### Diagnostics.

BOLD:ABY3312. Consensus barcode. TATAATATATTTTGTATTTGGAATTTGATGTGGGATATTAGGTTTATCTTTGAGATTAATAATTCGTATAGAATTAAGAAGAGTAATAAGTTTATTTTATAATGATCAATTATATAATAGAATTGTTACTATACATGCTTTTGTAATAATTTTTTTTATAGTAATACCTATTATAATTGGAGGTTTTGGAAATTGATTAATTCCTTTAATATTAGGTTTATCAGATATAATTTTTCCTCGAATAAATAATATAAGATTTTGATTATTACTTCCTTCAATTATTTTATTAATTATAGGAGGATTTGTAAATATAGGAGCAGGTACTGGTTGAACAGTTTATCCACCATTATCTTTATTAATAGGTCATAGTGGAATTTCTGTTGATTTATCAATTTTTTCTTTACATTTAGCAGGTATATCTTCTATTATAGGTTCAATTAATTTTATTGTTACTATTATTAATACATGAATATTAATAAATTATATAGATAAATATCCTTTATTTGTATGATCAGTATTTATTACTACAATTTTATTATTATTATCTTTACCAGTTTTGGCTGGTGCTATTACAATGTTATTAAGAGATCGAAATTTAAATACAAGATTTTTTGATCCATCAGGAGGAGGTGATCCAGTGTTATATCAACATTTATTT.

###### Holotype ♂.

Guanacaste, Sector El Hacha, Estación Los Almendros, 11.032, -85.528, 290 meters, caterpillar collection date: 01/iv/2011, wasp eclosion date: 08/v/2011. Depository: CNC.

***Host data*.***Herpetogrammaphaeopteralis* (Crambidae) feeding on *Sclerialatifolia* (Cyperaceae).

***Caterpillar and holotype voucher codes*.** 10-SRNP-4459, DHJPAR0048100.

###### Paratypes.

None.

###### Etymology.

*Chelonusjesusugaldei* is named to honor Sr. Jesus Ugalde for his many years of dedicated administrative support to the national biodiversity inventory under way by INBio, the National Biodiversity Institute of Costa Rica, the collection from which now resides in the Museo Nacional de Costa Rica.

**Figure 105.**
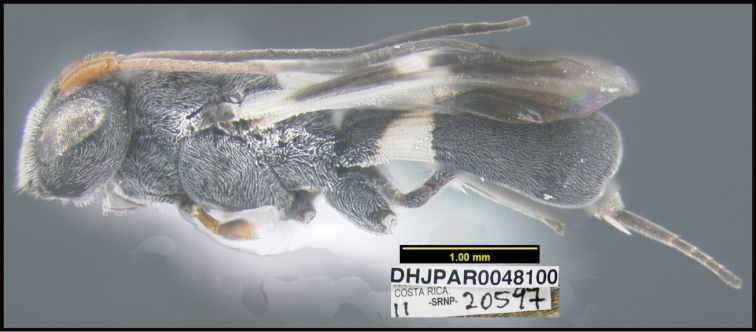
*Chelonusjesusugaldei*, holotype.

##### Chelonus jimlewisi

Taxon classificationAnimaliaHymenopteraBraconidae

Sharkey sp. nov.

http://zoobank.org/9FA4CF2C-4CDC-48AB-999A-4326FDAD203B

Figure 106

###### Diagnostics.

BOLD:AAF2610. Consensus barcode. TATATTATATTTTTTATTTGGAATTTGATCAGGCATATTAGGATTATCTTTAAGTTTAATAATTCGTATAGAACTAAGTAGGGTTATAAGATTATTTTATAATGATCAGTTATATAATAGGATTGTAACTATACATGCATTTGTAATAATTTTTTTTATAGTTATACCAGTAATAATTGGTGGATTTGGRAATTGATTAATTCCTTTAATATTAGGCTTATCTGATATAATTTTCCCTCGAATAAATAACATAAGATTTTGATTATTATTACCTTCAATTTTTTTATTAATTATAGGAGGATTTGTTAATATGGGGGCAGGAACTGGTTGAACRGTTTATCCACCATTATCTCTAATAATGGGACATAGGGGAATTTCTGTAGATTTATCAATTTTTTCTTTACATTTAGCAGGCATATCTTCAATTATAGGATCAATTAATTTTATTGTAACTATTATTAATACTTGATTGATTTATAATTATATGGATAAATATCCTTTATTTGTATGATCAGTATTTATTACTACAATTTTATTATTATTATCTTTACCAGTATTAGCTGGGGCTATTACTATATTATTAAGAGATCGAAATTTAAATACTAGGTTTTTTGATCCTTCAGGGGGAGGAGACCCAGTTTTATATCAACATTTRTTT.

###### Holotype ♀.

Alajuela, Sector Rincon Rain Forest, Palomo, 10.962, -85.28, 96 meters, caterpillar collection date: 16/x/2012, wasp eclosion date: 01/xi/2012. Depository: CNC.

***Host data*.***Neoleucinodes* Janzen02 (Crambidae) feeding on *Heliconiairrasa* (Heliconiaceae).

***Caterpillar and holotype voucher codes*.** 12-SRNP-68630, DHJPAR0051336.

###### Other material.

Several specimens that were not examined but that are in BOLD (e.g., DHJPAR0054551) and in the Janzen and Hallwachs database are recorded from *Neoleucinodesalegralis* (Crambidae). Depository: CNC.

###### Etymology.

*Chelonusjimlewisi* is named to honor Dr. Jim Lewis for his years of dedicated taxonomic and curational support, especially with Hemiptera, of the INBio collection now deposited in the Museo Nacional de Costa Rica.

**Figure 106.**
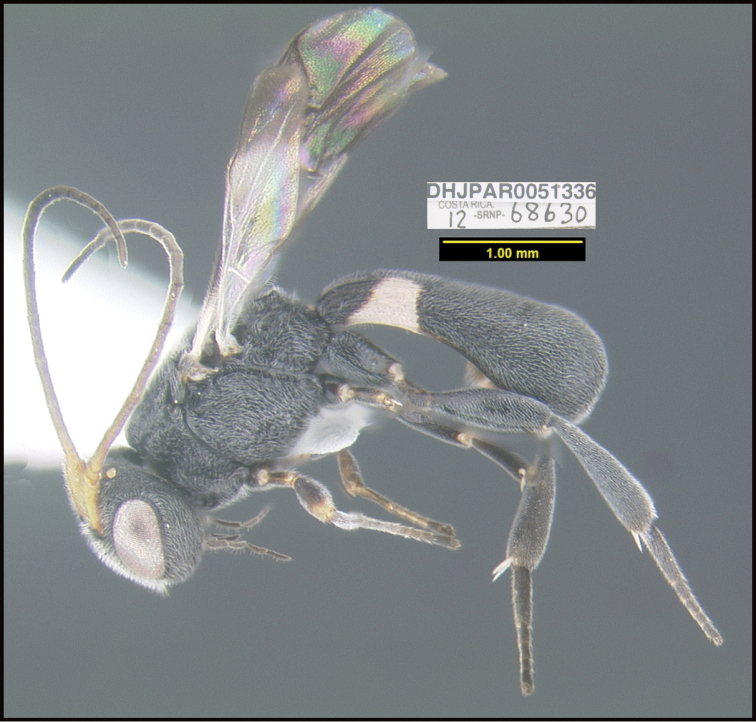
*Chelonusjimlewisi*, holotype.

##### Chelonus jimmilleri

Taxon classificationAnimaliaHymenopteraBraconidae

Sharkey sp. nov.

http://zoobank.org/1A8A19E5-54B3-4EAD-917A-95AE052A98B2

Figure 107

###### Diagnostics.

BOLD:ADM8275. Consensus barcode. TATATTATATTTTTTATTTGGAATTTGATCTGGTATATTAGGATTATCTTTAAGATTAATAATTCGTATAGAATTAAGAAGAGTAATAAGATTATTTTATAATGATCAATTATATAATAGTATTGTTACTATACATGCATTTGTAATAATTTTTTTTATGGTTATACCAGTAATAATTGGGGGATTTGGGAATTGGTTAGTTCCTTTAATATTAGGATTATCTGATATAATTTTTCCTCGAATAAATAATTTAAGATTTTGGTTGTTAGTGCCTTCAATTATTTTATTAATTATAGGTGGATTTGTAAATATGGGTGCAGGAACTGGTTGAACAGTTTATCCTCCATTATCTTTATTAATAGGTCATAGAGGAATTTCTGTTGATTTATCAATTTTTTCTTTACATTTGGCTGGTATATCTTCAATTATAGGTTCAATTAATTTTATTGTAACTATTATTAATACTTGATTAATAATTAATTATATAGATAAATATCCTTTATTTGTATGATCAGTATTTATTACTACAATTTTATTATTATTATCTTTACCAGTGTTAGCTGGTGCTATTACAATATTATTAAGAGATCGTAATTTAAATACTAGATTTTTTGATCCTTCAGGAGGGGGAGATCCAGTTTTATATCAGCATTTATTT.

###### Holotype ♀.

Alajuela, Sector Rincon Rain Forest, Palomo, 10.9619, -85.2804, 96 meters, caterpillar collection date: 02/xi/2017, wasp eclosion date: 07/xii/2017. Depository: CNC.

***Host data*.***Antaeotricha* Janzen78 (Depressariidae) feeding on *Hylaeanthehoffmannii* (Marantaceae).

***Caterpillar and holotype voucher codes*.** 17-SRNP-46525, DHJPAR0062576.

###### Paratypes.

Host = *Antaeotricha* Janzen78: DHJPAR0062577, DHJPAR0062578. Depository: CNC.

###### Etymology.

*Chelonusjimmilleri* is named to honor Dr. Jim Miller of the American Museum of Natural History for his years of enthusiasm for taxonomic and biodiversity support for understanding the Notodontidae of Costa Rica and those also working on its taxonomy.

**Figure 107.**
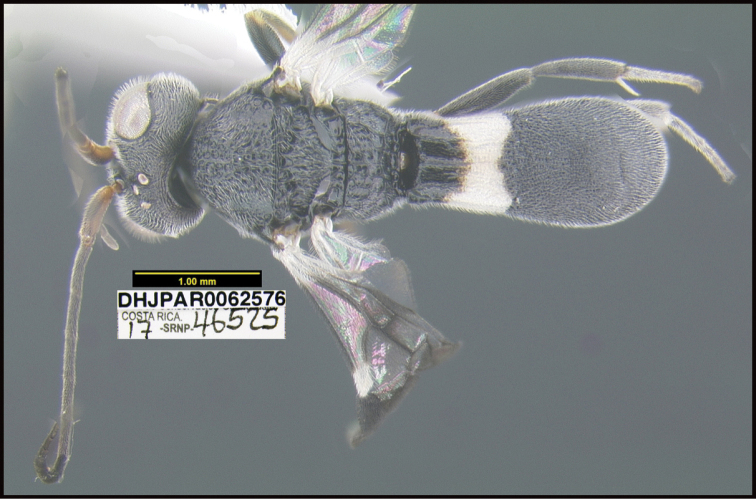
*Chelonusjimmilleri*, holotype.

##### Chelonus jimwhitfieldi

Taxon classificationAnimaliaHymenopteraBraconidae

Sharkey sp. nov.

http://zoobank.org/ADABAC0C-CAA1-4E9A-8FCE-8F64CC02AE9C

Figure 108

###### Diagnostics.

BOLD:ACC1273. Consensus barcode. TATATTATATTTTATATTTGGAATATGATGTGGGATTTTAGGTTTATCTTTAAGATTGATGATTCGGATAGAGTTAAGAAGTGTAATGAGTTTATTTTCTAATGATCAATTATATAATAGGATTGTCACTATACATGCTTTTGTAATAATTTTTTTTATGGTTATACCAATTATAATTGGCGGATTTGGAAATTGGTTAATTCCATTAATATTAGGATTATCAGATATGATTTTTCCTCGGATAAATAATATAAGATTTTGATTATTGGTTCCTTCAATTATATTATTAATTATAAGAGGATTTGTGAATATGGGAGCAGGGACTGGGTGGACTGTGTATCCTCCTTTGTCGTTGTTGATAGGTCATAGAGGTATTTCAGTAGATTTATCTATTTTTTCTTTGCATTTGGCAGGAGTTTCTTCAATTATAGGCTCAATTAATTTTATTGTAACTATTGTTAGTACTTGAATAAGTTTAAAATATATAGATAAATATTCGTTATTTGTTTGATCTGTGTTTATTACTACTATTTTATTATTATTATCTTTACCAGTGTTAGCTGGGGCAATTACTATATTATTAAGTGATCGAAATTTAAATACAAGTTTTTTTGATCCTTCTGGTGGGGGAGATCCTGTATTATATCAACATTTATTT.

###### Holotype ♀.

Guanacaste, Sector Pitilla, Medrano, 11.016, -85.381, 380 meters, caterpillar collection date: 22/viii/2012, wasp eclosion date: 13/ix/2012. Depository: CNC.

***Host data*.***Prenestascyllalis* (Crambidae) feeding on *Forsteroniaspicata* (Apocynaceae).

***Caterpillar and holotype voucher codes*.** 12-SRNP-72051, DHJPAR0050070.

###### Paratype.

Host = *Prenesta* scyllalisDHJ01: DHJPAR0050073. Depository: CNC.

###### Etymology.

*Chelonusjimwhitfieldi* is named to honor Dr. Jim Whitfield of the University of Illinois for his decades of mentorship to DHJ about Braconidae taxonomy and stimulus for their collection and rearing from ACG, while carrying out many braconid taxonomic projects for ACG specimens.

**Figure 108.**
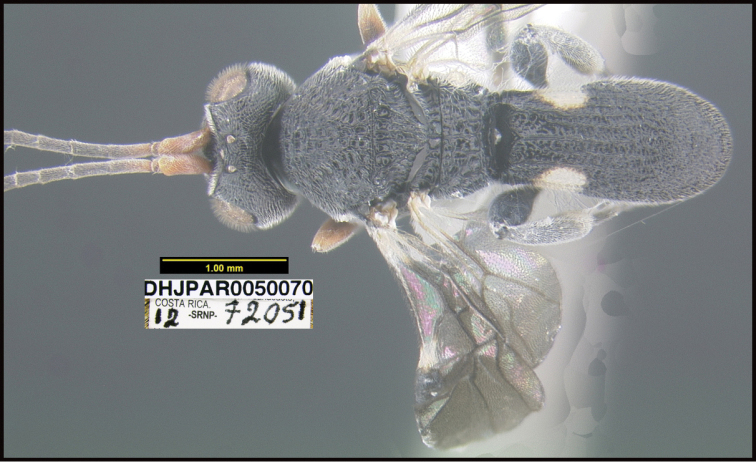
*Chelonusjimwhitfieldi*, holotype.

##### Chelonus johanvalerioi

Taxon classificationAnimaliaHymenopteraBraconidae

Sharkey sp. nov.

http://zoobank.org/1BED0947-BBCE-4227-ACEC-DE685A0D37B9

Figure 109

###### Diagnostics.

BOLD:AAF2611. Consensus barcode. TATATTATATTTTATATTTGGAATTTGGKSCGGCATATTAGGYTTATCATTAAGATTAATAATTCGTATAGAATTAAGAAGTGTAATAAGATTATTTTATAATGATCAATTGTATAATAGAATTGTAACAATACATGCTTTTGTTATAATTTTTTTTATAGTTATGCCAGTGATAATTGGAGGATTTGGTAATTGGTTGGTTCCTTTAATATTAGGGTTATCAGATATAATTTTTCCTCGTATAAATAATATAAGATTTTGGTTACTAATTCCTTCAATTATTTTATTAATTATGGGTGGTTTTGTAAATATAGGGGCAGGTACAGGTTGGACAGTTTATCCACCTTTATCTCTATTAAGAAGACATAGAGGAATTTCGGTGGATTTATCTATTTTTTCTTTGCATTTAGCAGGGGCTTCTTCAATTATAGGGTCTATTAATTTTATTATTACTATTATTAATACATGGATAGTGTTAAGTAACATAGATAAATATTCTTTATTTGTTTGATCTGTTTTTATTACAACAATTTTATTGTTATTATCTTTGCCTGTTTTAGCAGGGGCTATYACTATATTATTAAGTGACCGAAATTTAAATACTAGTTTTTTTGATCCTTCTGGAGGGGGTGAYCCAGTGTTGTACCAACATTTATTT.

###### Holotype ♂.

Guanacaste, Sector San Cristobal, Tajo Angeles, 10.865, -85.415, 540 meters, caterpillar collection date: 19/viii/2010, wasp eclosion date: 13/ix/2010. Depository: CNC.

***Host data*.***Ceratocilia* Janzen02 (Crambidae) feeding on *Cosmibuenagrandiflora* (Rubiaceae).

***Caterpillar and holotype voucher codes*.** 10-SRNP-4589, DHJPAR0042090.

###### Paratypes.

Hosts = *Ceratocilia* Janzen02, *Phostrialatiapicalis*, *Pilocrocispurpurascens* (all Crambidae). DHJPAR0029142, DHJPAR0036388, DHJPAR0036728, DHJPAR0039119, DHJPAR0042087, DHJPAR0042092, DHJPAR0048106, DHJPAR0052096, DHJPAR0055381, DHJPAR0055397, DHJPAR0055398, DHJPAR0055951, DHJPAR0056967, DHJPAR0057408, DHJPAR0057409. Depository: CNC.

###### Etymology.

*Chelonusjohanvalerioi* is named to honor Sr. Johan Valerio of ICE for his many years of mentorship of DHJ for the ACG-ICE-GDFCF project to biomonitor PL12 in Pailas II and the new ICE road crossing Sector Mundo Nuevo of ACG.

**Figure 109.**
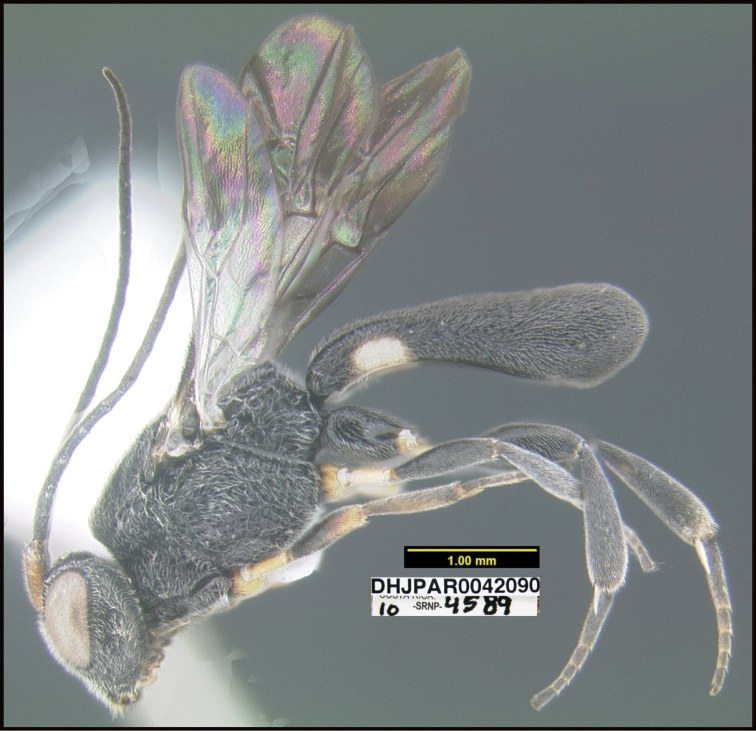
*Chelonusjohanvalerioi*, holotype.

##### Chelonus johnburnsi

Taxon classificationAnimaliaHymenopteraBraconidae

Sharkey sp. nov.

http://zoobank.org/96E3D1A8-5991-4FC3-87A4-1F347776A42A

Figure 110

###### Diagnostics.

BOLD:ACJ4065. Consensus barcode. AATATTATATTTTATTTTTGGTATATGGGCAGGTATAATAGGTTTATCATTAAGAATAATGATTCGAATGGAATTAAGAAGTGTTATGAGATTATTTTTTAGGGATCAATTATATAATAGATTAGTAACTATACATGCATTTATTATAATTTTTTTTATAGTTATACCTATTATAATTGGAGGATTTGGAAATTGGTTGGTGCCATTGATATTGGGTTTATCAGATATAATTTTTCCTCGAATAAATAATATAAGATTTTGATTATTAATTCCATCTTTATTTTTATTAATTATAGGTGGATTTGTAAATACAGGGGCTGGTACAGGATGAACTGTTTATCCTCCATTATCTTTATTAATAGGTCATGGAGGAGTTTCAGTTGATTTATCAATTTTTTCTTTACATTTAGCTGGTGCATCATCTATTATAGGATCTATAAATTTTATTGTTACTATTTTAAATACATGAATAAAATTAAAGTATATAGATAAATTTTCATTATTTATTTGGTCAGTATTAATTACTACTATTTTATTACTTTTGTCATTGCCTGTATTAGCTGGGGCAATTACAATATTGTTAAGAGATCGTAATTTAAATACTAGATTTTTTGATCCTTCAGGAGGGGGGGACCCTGTATTATATCAACATTTATTT.

###### Holotype ♂.

Alajuela, Sector Rincon Rain Forest, Palomo, 10.962, -85.28, 96 meters, caterpillar collection date: 02/i/2013, wasp eclosion date: 05/ii/2013. Depository: CNC.

***Host data*.***Spilomelapantheralis* (Crambidae) feeding on *Celtisiguanaea* (Ulmaceae).

***Caterpillar and holotype voucher codes*.** 13-SRNP-67036, DHJPAR0051324.

###### Paratypes.

None.

###### Etymology.

*Chelonusjohnburnsi* is named to honor Dr. John Burns of the Smithsonian Institution for his decades of taxonomic projects and mentorship of DHJ and WH about the Hesperiidae of ACG, as well as happily accepting their DNA barcoding as part of their taxonomic discovery.

**Figure 110.**
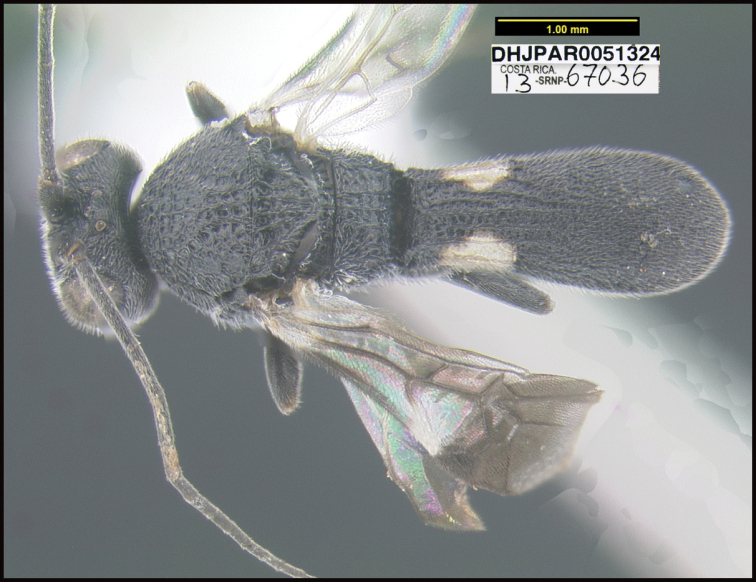
*Chelonusjohnburnsi*, holotype.

##### Chelonus johnnoyesi

Taxon classificationAnimaliaHymenopteraBraconidae

Sharkey sp. nov.

http://zoobank.org/563844C1-75BF-4A4C-B3A3-5A22BAF1B25B

Figure 111

###### Diagnostics.

BOLD:ACF7386. Consensus barcode. AATATTATATTTTATTTTTGGTATTTGATGTGGAGTTTTAGGTTTATCTTTAAGTTTATTAATTCGTATGGAATTAAGAAGGGTAATAAGTTTATTTATAAATGATCAATTGTATAATAGAGTTGTTACTATGCATGCTTTTGTAATAATTTTTTTTATGGTTATACCAGTTATAGTTGGTGGGTTTGGAAATTGATTAGTACCTTTAATATTAGGGCTATCTGATATAATTTTTCCTCGAATAAATAATATAAGTTTTTGATTATTATTTCCTTCAATTATTTTATTAGTTATAGGTGGATTTATTAATATGGGGGTTGGAACAGGGTGAACTGTTTATCCCCCATTATCTTTAATGATGGGTCATAGGGGGATTTCCGTTGATTTATCAATTTTTTCTTTACATTTGGCTGGGATATCGTCAATTATAGGTTCTATTAATTTTATTGTTACAACAATTAATACATGATTAAAGGTTACTTATATAGATAAATATTCATTATTTATTTGATCTATTTTTATTACTACAATTTTATTGTTATTATCTTTACCTGTCTTAGCTGGGGCAATTACTATATTATTAAGTGATCGAAATTTAAATACTAGATTTTTTGATCCTTCAGGAGGAGGAGA--------.

###### Holotype ♀.

Guanacaste, Sector Santa Rosa, Bosque San Emilio, 10.8438, -85.6138, 300 meters, 11-18/vi/2012, Malaise trap. Depository: CNC.

***Host data*.** None.

***Holotype voucher code*.**BIOUG05181-G09.

###### Paratypes.

None.

###### Etymology.

*Chelonusjohnnoyesi* is named to honor Dr. John Noyes of The Natural History Museum, London, for his decades of enthusiastic support and actions with the taxonomy of Encyrtidae wasps of Costa Rica in general, and specifically for INBio and ACG.

**Figure 111.**
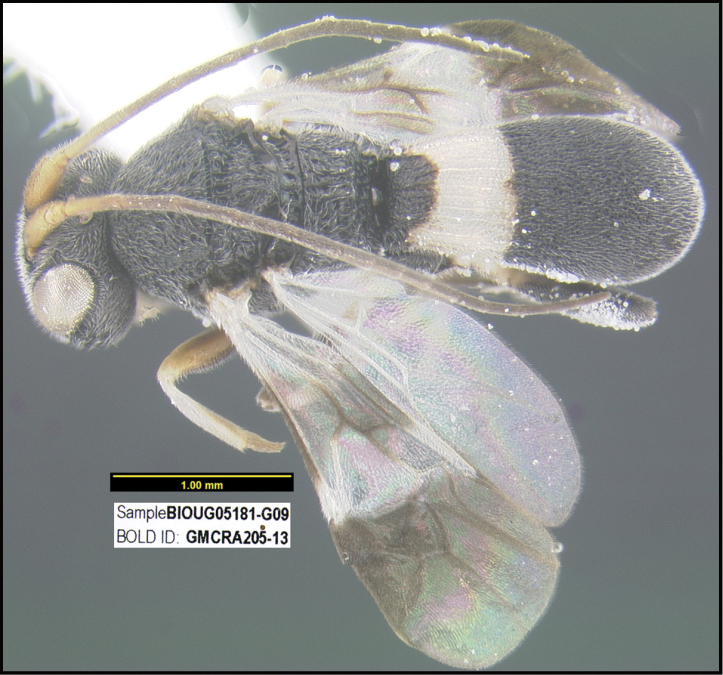
*Chelonusjohnnoyesi*, holotype.

##### Chelonus jorgebaltodanoi

Taxon classificationAnimaliaHymenopteraBraconidae

Sharkey sp. nov.

http://zoobank.org/65FC430A-82DB-4308-98AF-B285744E72E9

Figure 112

###### Diagnostics.

BOLD:ACK4767. Consensus barcode. AATTCTTTATTTCATTTTTGGGATTTGATCTGGTGTAATAGGGTTATCTTTAAGTTTATTAATTCGTATGGAAYTAAGGAGAGTAATAAGATTAYTTATAAATGATCAATTATATAATAGTATTGTTACTATACATGCTTTTATTATAATTTTTTTTATGGTTATACCTGTAATAGTTGGGGGATTCGGAAATTGATTAATTCCTTTAATATTAGGTTTATCTGATATAATTTTTCCTCGTATAAATAATTTAAGATTTTGATTACTGATTCCATCAATTATTTTATTAYTATTAGGAGGYTTTACTAATACAGGGGCTGGAACAGGATGAACTGTGTATCCTCCATTATCTTTAATAATAGGACATAGAGGAATTTCAGTTGATTTGTCAATYTTTTCTTTGCATTTAGCTGGGGCTTCTTCTATTATAGGATCTATTAATTTTATTGTAACAACAATTAATACATGATTAAAAATAACTAAYATAGATAAATATTCTTTATTTGTATGATCTATTTTTATCACTACAATTTTACTTTTATTATCTTTACCAGTTTTAGCYGGGGCAATTACTATATTATTAAGAGATCGAAATTTAAATACTAGGTTTTTTGATCCTTCTGGTGGTGGGGACCCTGTATTATACCAGCATTTATTT.

###### Holotype ♀.

Guanacaste, Pailas Dos, PL12-2, 10.7634, -85.335, 824 meters, 10‒17/iv/2014, Malaise trap PL12-2A. Depository: CNC.

***Host data*.** None.

***Holotype voucher code*.**BIOUG30966-F11.

###### Paratypes.

Both Malaise-trapped. BIOUG08913-A09, BIOUG28821-H05. Depository: CNC.

###### Etymology.

*Chelonusjorgebaltodanoi* is named to honor Sr. Jorge Baltodano (RIP) for his many years as a neighboring collaborator with ACG and for accepting the challenge of being the first President of the Comité Local for ACG; part of his ranch is now a permanent part of ACG thanks to his foresight and spirit for conservation.

**Figure 112.**
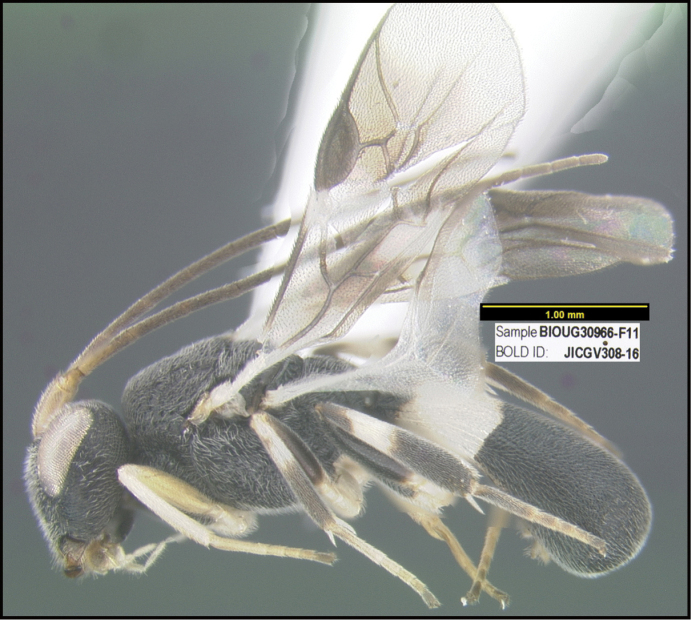
*Chelonusjorgebaltodanoi*, holotype.

##### Chelonus jorgehernandezi

Taxon classificationAnimaliaHymenopteraBraconidae

Sharkey sp. nov.

http://zoobank.org/A865A598-3741-487C-9D9C-2F0050652CF1

Figure 113

###### Diagnostics.

BOLD:ADB2931. Consensus barcode. ATTCTTTATTTCATTTTTGGGATTTGATCTGGTRTAATAGGGTTATCTTTAAGTTTACTAATTCGTATGGAACTAAGAAGAGTAATAAGGTTACTTATAAATGATCAATTATATAATAGTATCGTTACTATGCATGCTTTTATTATAATTTTTTTTATGGTTATGCCTGTAATAGTTGGTGGATTTGGTAATTGATTAATTCCTTTAATATTAGGTTTATCTGATATAATTTTCCCCCGTATAAATAATTTAAGATTTTGATTATTGATTCCGTCAATTATTTTATTATTATTAGGAGGTTTTATTAATAYAGGGGCTGGAACAGGATGAACTGTGTATCCTCCATTATCTTTAATAATAGGACATAGAGGAATTTCAGTTGATTTGTCAATTTTTTCTTTGCATTTAGCTGGAGYTTCTTCTATTATAGGATCTGTTAATTTTATTGTAACAACAATTAATACATGATTAAAAATAACTAATATAGATAAATATTCTTTATTTGTATGATCTATTTTTATTACCACAATTTTACTTTTATTATCTTTACCAGTTTTAGCTGGGGCAATCACTATATTATTAAGAGACCGAAATTTAAATACTAGGTTTTTT.

###### Holotype ♀.

Guanacaste, Pailas Dos, PL12-3, 10.7631, -85.3344, 820 meters, 05‒12/xii/2013, Malaise trap PL12-3A. Depository: CNC.

***Host data*.** None.

***Holotype voucher code*s**: BIOUG29282-F08.

###### Paratype.

Malaise-trapped. BIOUG29803-C11. Depository: CNC.

###### Etymology.

*Chelonusjorgehernandezi* is named to honor Sr. Jorge Hernandez of GDFCF and ACG for his many years as a dedicated inventory parataxonomist for ACG.

**Figure 113.**
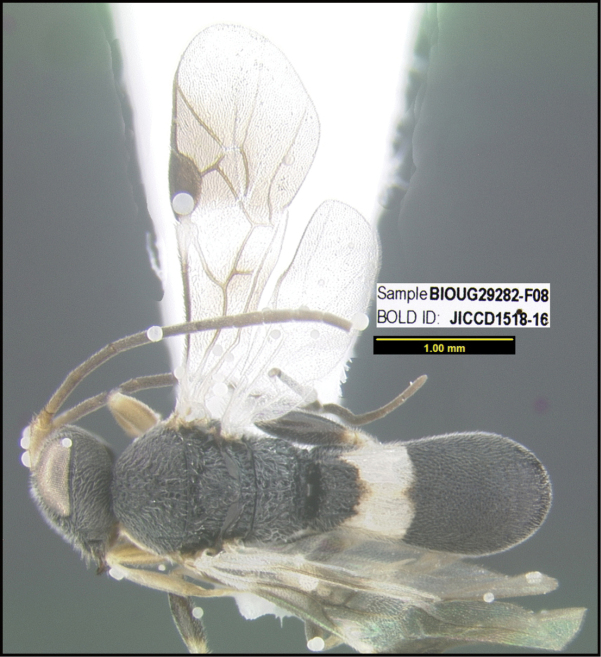
*Chelonusjorgehernandezi*, holotype.

##### Chelonus josealfredohernandezi

Taxon classificationAnimaliaHymenopteraBraconidae

Sharkey sp. nov.

http://zoobank.org/A3ECBC91-E1A6-4FCD-B7E4-DEE0D3D65D2D

Figure 114

###### Diagnostics.

BOLD:ADC6444. Consensus barcode. AATTCTTTATTTTATTTTTGGAATTTGATCTGGTATAATAGGGTTATCTTTAAGTTTATTAATTCGTATGGAATTA---AGAAGAGTAACAAGGTTATTTATAAATGATCAATTATATAATAGTATTGTTACTATACATGCTTTTGTAATAATTTTTTTTATAGTTATACCTGTAATAGTAGGTGGATTTGGAAATTGATTAATTCCTTTAATATTAGGTTTGTCCGATATAATTTTTCCTCGTATGAATAATTTAAGATTTTGATTATTGATTCCATCAATTATTTTATTAATAATAGGAGGTTTTATTAATATAGGGGCTGGAACGGGATGAACTGTTTATCCCCCATTATCATTAATAATAGGACATAGGGGAATTTCAGTTGATTTATCAATTTTTTCTTTACATTTAGCTGGGGCTTCTTCTATTATAGGGTCAATTAATTTTATTGTTACAGTGATTAATACATGATTAAAAATAACTAATATAGATAAATATTCTTTATTTGTTTGATCTATTTTTATCACTACAATTTTACTTTTATTATCTTTACCAGTTTTAGCTGGAGCTATTACAATATTATTAAGAGACCGAAATTTAAATACTAGATTTTTTGATCCTTCGGGTGGGGGAGATCC-------.

###### Holotype ♀.

Guanacaste, Pailas Dos, PL12-9, 10.76, -85.3341, 809 meters, 15‒22/v/2014, Malaise trap PL12-9A. Depository: CNC.

***Host data*.** None.

***Holotype voucher code*.**BIOUG29216-H07.

###### Paratypes.

None.

###### Etymology.

*Chelonusjosealfredohernandezi* is named to honor Sr. Jose Alfredo Hernandez of CONAGEBIO for his deep understanding and advising for the Costa Rican government permits for BioAlfa bioinventory.

**Figure 114.**
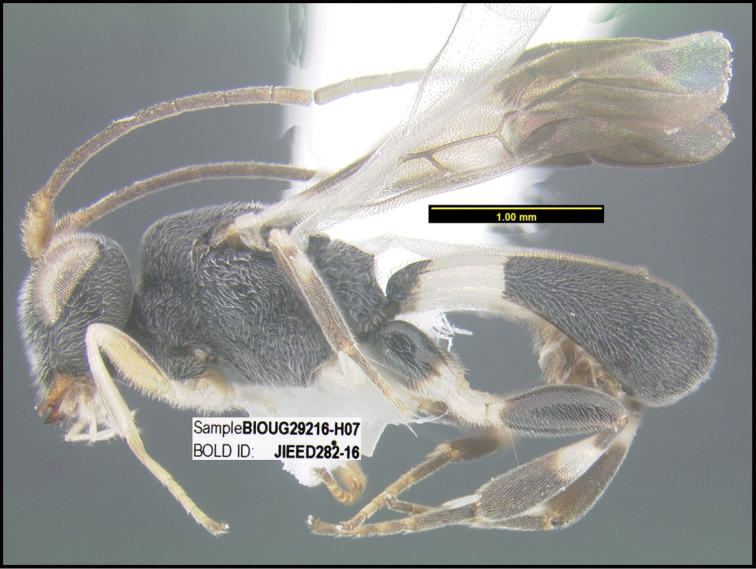
*Chelonusjosealfredohernandezi*, holotype.

##### Chelonus josefernandeztrianai

Taxon classificationAnimaliaHymenopteraBraconidae

Sharkey sp. nov.

http://zoobank.org/9702D736-3794-4079-9EBA-7B2B6060ADC7

Figure 115

###### Diagnostics.

BOLD:ACJ1335. Consensus barcode. AATTTTGTATTTTATTTTTGGAATATGATCAGGGATAATAGGATTATCATTAAGGTTATTAATTCGAATAGAATTAAGTATTGTGGGTAGATTATTTATAAATGATCAGTTATATAATAGAATTGTAACAATACATGCATTTATTATAATTTTTTTTATAGTCATACCAATTATAATTGGTGGTTTTGGGAATTGATTAATTCCTTTAATATTAGGATTACCAGATATAATTTTTCCTCGAATAAATAATATAAGATTTTGATTATTAATTCCTTCAATTATTTTATTAATTATAGGGGGATTTGTAAATATAGGAGTTGGAACTGGTTGAACTGTATATCCACCTTTATCTTTATTAATAAGACATAGAGGTATTTCAGTAGATTTATCAATTTTTTCTTTGCATTTAGCTGGGATATCTTCAATTATAGGGTCTATTAATTTTATTGTTACAATTTTAAATACATGAATAAATAAAAAAATGATAGATAAATTTCCTTTATTTGTTTGATCAGTATTTATTACAACAATTTTATTATTATTATCTTTACCAGTTTTAGCTGGAGCAATTACAATGTTGTTAAGTGATCGAAATTTAAATACAAGATTTTTTGATCCTTCTGGTGGAGGAGATCCAGTATTATATCAACATTTATTT------.

###### Holotype ♂.

Guanacaste, Sector Santa Rosa, Bosque San Emilio, 10.8438, -85.6138, 300 meters, 02‒09/iv/2012, Malaise trap. Depository: CNC.

***Host data*.** None.

***Holotype voucher code*.**BIOUG07458-A09.

###### Paratypes.

None.

###### Etymology.

*Chelonusjosefernandeztrianai* is named to honor Dr. José Fernandez-Triana for his many years of mentoring and taxonomic applications to the bioinventory of the Microgastrinae, Braconidae, of ACG.

**Figure 115.**
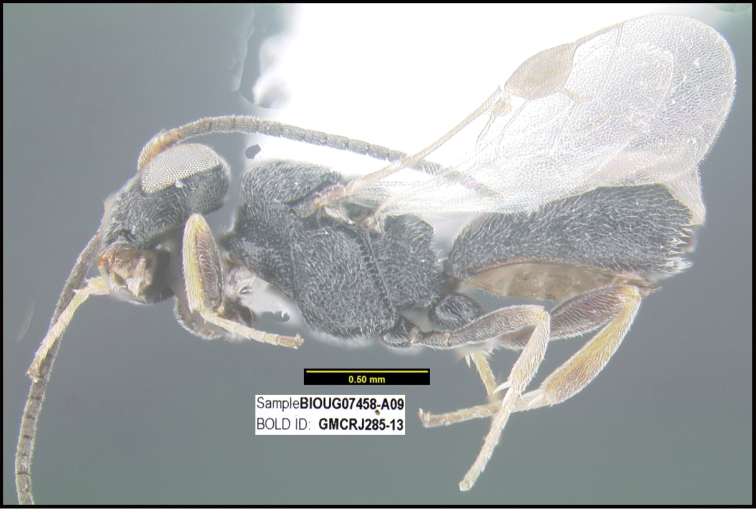
*Chelonusjosefernandeztrianai*, holotype.

##### Chelonus josehernandezcortesi

Taxon classificationAnimaliaHymenopteraBraconidae

Sharkey sp. nov.

http://zoobank.org/1B4DB395-27E9-4E3D-B9FC-7E9B95D0F216

Figure 116

###### Diagnostics.

BOLD:ACR6579. Consensus barcode. ATATTATATTTTATTTTTGGTATATGATCTGGAATATTAGGTTTATCATTAAGTTTATTAATTCGAATAGAATTAAGAATTACGGGTAGATTATTTATAAATGATCAATTATATAATAGAATTGTAACTATACATGCTTTTATTATAATTTTTTTTATGGTTATACCAATTATAATTGGTGGTTTTGGGAATTGACTAATTCCACTAATATTAGGTTTACCTGATATGATTTTTCCTCGAATAAATAATATAAGTTTTTGATTATTAATACCTTCGTTATTATTGTTATTATTAGGAGGATTTGTAAATATAGGAGTAGGAACAGGTTGAACAGTTTATCCACCATTATCTTTATTAATAGGTCATAGTGGAATTTCAGTTGATTTATCAATTTTTTCTTTACATTTAGCTGGGGCATCTTCAATTATAGGATCAATTAATTTTATTGTAACAATTATTAATACATGAATAAATAATAAATTTATAGATAAATTTCCATTATTTGTTTGATCAGTATTTATTACTACAATTTTGTTATTATTATCTTTACCAGTATTAGCTGGTGCAATTACAATATTATTAAGTGATCGAAATTTAAAT.

###### Holotype ♀.

Guanacaste, Pailas Dos, PL12-3, 10.7631, -85.3344, 820 meters, 13‒20/ii/2014, Malaise trap PL12-3A. Depository: CNC.

***Host data*.** None.

***Holotype voucher code*.**BIOUG29681-C10.

###### Paratype.

Malaise-trapped. BIOUG17842-H09. Depository: CNC.

###### Etymology.

*Chelonusjosehernandezcortesi* is named to honor Sr. José Hernandez of GDFCF and ACG for his many years as a dedicated inventory parataxonomist for ACG.

**Figure 116.**
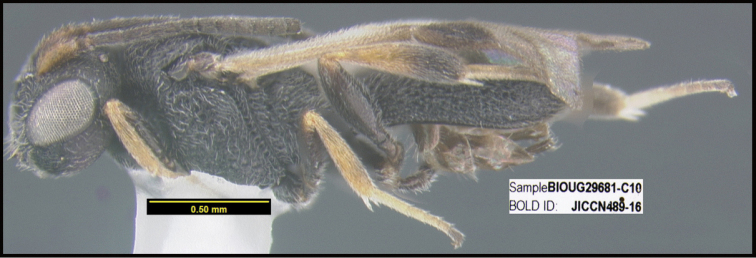
*Chelonusjosehernandezcortesi*, holotype.

##### Chelonus josemanuelperezi

Taxon classificationAnimaliaHymenopteraBraconidae

Sharkey sp. nov.

http://zoobank.org/2F9DB29F-7FEB-45A2-8F31-2F0B0EB0C17E

Figure 117

###### Diagnostics.

BOLD:ACZ8757. Consensus barcode. AATATTATATTTTTTATTTGGTTTATGAAGAGGAATTTTAGGTTTGTCATTAAGTTTGTTAATTCGTATAGAATTAAGAATAGTTGGTAGATTATTTATAAATGATCAGCTATATAATAGAATTGTAACTATACATGCTTTTATTATAATTTTTTTTATAGTTATACCTGTAATAATTGGAGGATTTGGAAATTGATTAGTTCCTTTAATATTAGGGTTATCAGATATAATTTTTCCACGAATAAATAATATAAGATTTTGATTGTTAATTCCTTCTATTTTGTTATTAATTATAGGAGGATATGTGGGAACAGGGGTAGGTACTGGTTGAACTGTTTATCCACCTTTATCTTTATTAATTGGCCATAGTGGAATTTCTGTAGATTTATCTATTTTTTCTTTACATTTAGCTGGAGTATCATCAATTATGGGATCTGTAAATTTTATTGTTACTATTATAAATACTTGAATAGAAAAAATAAACATGGATAAATATTCTTTATTTATTTGATCTGTTTTTATTACTACTATTTTATTATTATTATCTTTACCTGTGTTAGCAGGTGCTATTACTATATTATTAAGTGATCGTAATTTGAATACAAGATTTTTTGATCCTTCAGGTGGGGGGGATCCAGTATTATATCAACATTTATTT.

###### Holotype ♀.

Guanacaste, Sector San Cristobal, Estación San Gerardo, 10.88, -85.389, 575 meters, 29/xii/2014‒05/i/2015, Malaise trap. Depository: CNC.

***Host data*.** None.

***Holotype voucher code*.**BIOUG28039-F05.

###### Paratypes.

All Malaise-trapped. BIOUG27759-B05, BIOUG28037-E12, BIOUG28039-G01. Depository: CNC.

###### Etymology.

*Chelonusjosemanuelperezi* is named to honor Sr. Jose Manuel Perez of GDFCF and ACG for his many years as a dedicated inventory parataxonomist for ACG.

**Figure 117.**
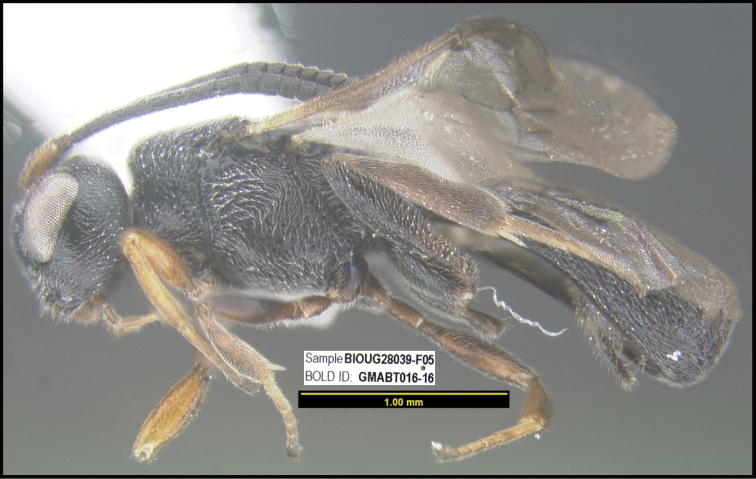
*Chelonusjosemanuelperezi*, holotype.

##### Chelonus josephinerodriguezae

Taxon classificationAnimaliaHymenopteraBraconidae

Sharkey sp. nov.

http://zoobank.org/2BC7B303-AC7A-4ACC-BF11-B435C889C995

Figure 118

###### Diagnostics.

BOLD:ADG0772. Consensus barcode. ATATTATATTTCATTTTTGGTTTATGAAGCGGAATTTTAGGTTTATCATTAAGTTTATTAATTCGAATAGAATTAAGAATAGTAGGAAGTTTATTTTTAAATGATCAATTATATAATAGAATTGTAACAATACATGCTTTTATTATAATTTTTTTTATAGTTATACCAATTATAATTGGTGGATTTGGGAATTGATTAGTTCCATTAATATTAGGATTATCTGATATAATTTTTCCACGAATAAATAATATAAGATTTTGATTATTAATTCCTTCAATTTTATTATTAATTTTAGGAAGATATGTAGGTACTGGAGCAGGAACAGGATGAACTGTATATCCACCATTATCTTTATTAATAGGTCATAGAGGAATATCTGTAGATTTATCAATTTTTTCTTTACATTTAGCTGGAATTTCTTCTATTATAGGTTCAATTAATTTTATTGTGACAATTATAAATACTTGAATATTAAAAAAAAATATAGATAAATATTCATTATTTATTTGATCTGTTTTTATTACAACTATTTTATTGTTATTATCTTTACCTGTATTAGCTGGTGCAATTACAATATTATTAAGTGATCGTAAT----------------------------.

###### Holotype ♀.

Guanacaste, Sector Cacao, Derrumbe, 10.9292, -85.4643, 1220 meters, 25/xii/2014‒01/i/2015, Malaise trap. Depository: CNC.

***Host data*.** None.

***Holotype voucher code*.**BIOUG31680-G05.

###### Paratypes.

None.

###### Etymology.

*Chelonusjosephinerodriguezae* is named to honor Dr. Josephine Rodriguez of the University of Virginia’s College at Wise, for her intense collaboration and taxonomy of ACGMicrogastrinae as part of the ACG ongoing Biodiversity Inventory.

**Figure 118.**
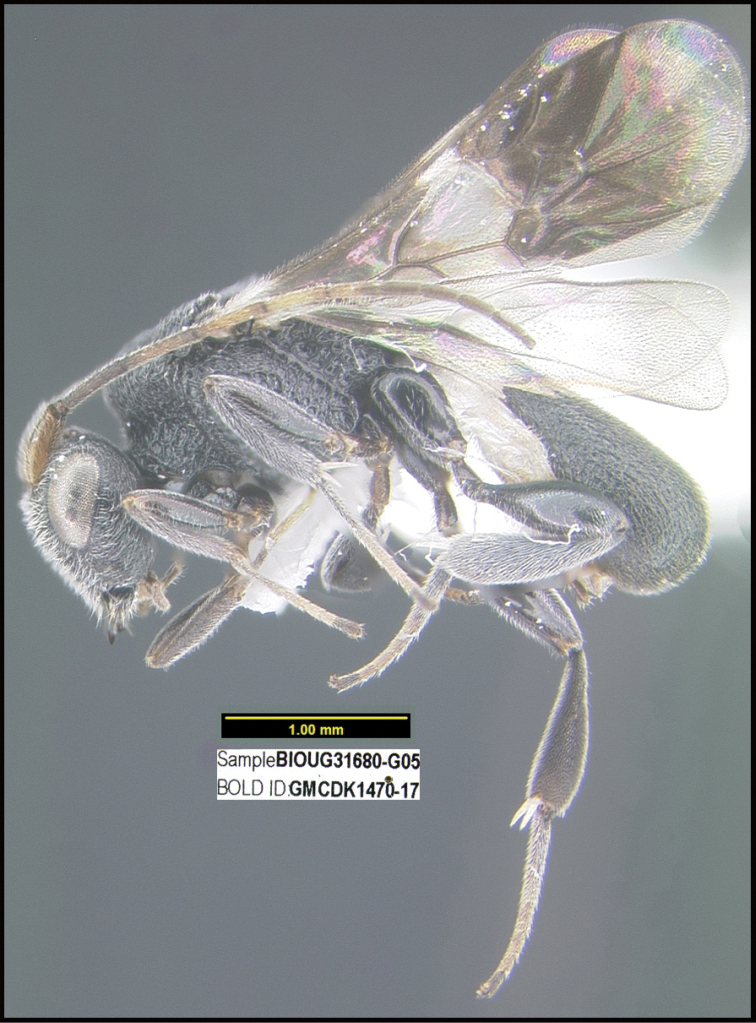
*Chelonusjosephinerodriguezae*, holotype.

##### Chelonus juanmatai

Taxon classificationAnimaliaHymenopteraBraconidae

Sharkey sp. nov.

http://zoobank.org/D888F34C-1362-499D-BE02-4FFAC8EF1724

Figure 119

###### Diagnostics.

BOLD:ABY3313. Consensus barcode. AATATTATATTTTTTATTTGGTTTATGAAGAGGAATTTTAGGTTTGTCATTAAGTTTGTTAATTCGTATAGAATTAAGAATAGTTGGTAGATTATTTATAAATGATCAGCTATATAATAGAATTGTAACTATACATGCTTTTATTATAATTTTTTTTATAGTTATACCTGTAATAATTGGAGGATTTGGAAATTGATTAGTTCCTTTAATATTAGGGTTATCAGATATAATTTTTCCACGAATAAATAATATAAGATTTTGATTGTTAATTCCTTCTATTTTGTTATTAATTATAGGAGGATATGTGGGAACAGGGGTAGGTACTGGTTGAACTGTTTATCCACCTTTATCTTTATTAATTGGCCATAGTGGAATTTCTGTAGATTTATCTATTTTTTCTTTACATTTAGCTGGAGTATCATCAATTATGGGATCTGTAAATTTTATTGTTACTATTATAAATACTTGAATAGAAAAAATAAACATGGATAAATATTCTTTATTTATTTGATCTGTTTTTATTACTACTATTTTATTATTATTATCTTTACCTGTGTTAGCAGGTGCTATTACTATATTATTAAGTGATCGTAATTTGAATACAAGATTTTTTGATCCTTCAGGTGGGGGGGATCCAGTATTATATCAACATTTATTT.

###### Holotype ♂.

Alajuela, Sector San Cristobal, Finca San Gabriel, 10.878, -85.393, 645 meters, caterpillar collection date: 13/viii/2011, wasp eclosion date: 29/viii/2011. Depository: CNC.

***Host data*.** immidjanzen01 Janzen23 (Immidae) feeding on *Pentagoniadonnell-smithii* (Rubiaceae).

***Caterpillar and holotype voucher codes*.** 11-SRNP-3192, DHJPAR0048103.

###### Paratypes.

None.

###### Etymology.

*Chelonusjuanmatai* is named to honor Sr. Juan Mata for his outstanding laboratory photography contributions to the INBio, MN, and BioAlfa taxonomy publications from BioAlfa and his interest in Mantodea.

**Figure 119.**
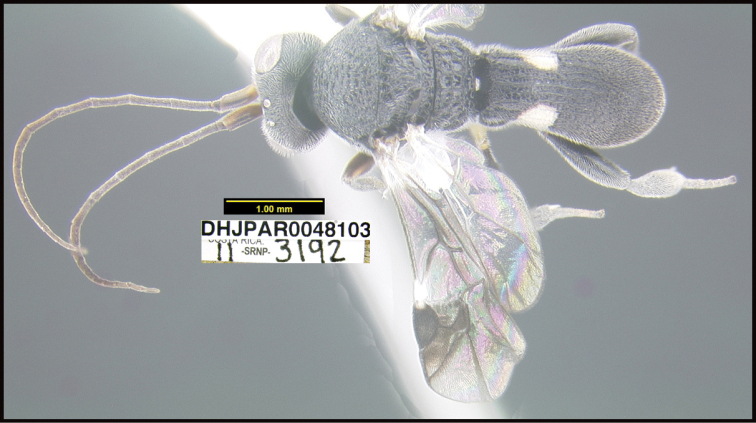
*Chelonusjuanmatai*, holotype.

##### Chelonus junkoshimurae

Taxon classificationAnimaliaHymenopteraBraconidae

Sharkey sp. nov.

http://zoobank.org/D4EF9F0A-F329-4130-934E-9D41DF6A8E24

Figure 120

###### Diagnostics.

BOLD:AAF2633. Consensus barcode. AATATTATATTTTATTTTTGGTATATGATGTGGTATACTTGGATTATCATTAAGTATATTAATTCGAATAGAATTAAGTATAGTTTCAAGTTTATTAATAAATGATCAATTATATAATAGAATTGTTACAATACATGCATTTGTAATAATTTTTTTTATGGTTATACCARTTATAATTGGTGGATTTGGGAATTGGTTAATTCCATTAATATTAGGATTATCAGATATAATATTTCCACGAATAAATAATATAAGATTTTGATTGTTAGTACCGTCAATTATATTATTAATTTTAAGAGGTTTTATTAATACTGGTGTTGGAACTGGATGAACAGTTTATCCCCCATTATCTTTATTAATAGGTCATGGGGGAATTTCTGTAGATTTATCAATTTTTTCTTTACATTTAGCAGGGGCTTCATCAATTATAGGTTCAATTAATTTTATTGTTACAGTAATAAATTCTTGAATAAAATTAAGTTATATAGATAAATTTGCTTTATTTGTTTGATCAGTTTTTATTACAACAATTTTATTATTATTATCTTTACCGGTATTAGCAGGAGCTATTACTATATTATTAAGGGATCGAAATTTAAATACAAGATTTTTTGATCCTTCAGGAGGAGGAGATCCTGTATTATATCAACATTTATTT.

###### Holotype ♂.

Guanacaste, Sector Orosi, Maderos, 11.005, -85.475, 510 meters, caterpillar collection date: 19/vi/2010, wasp eclosion date: 07/vii/2010. Depository: CNC.

***Host data*.***Diaphania* hyalinataDHJ02 (Crambidae) feeding on *Guraniamakoyana* (Cucurbitaceae).

***Caterpillar and holotype voucher codes*.** 10-SRNP-21385, DHJPAR0040366.

###### Paratypes.

Hosts = *Diaphania* hyalinataDHJ01, *Diaphania* hyalinataDHJ02. BIOUG17755-H07, DHJPAR0021626, DHJPAR0029175, DHJPAR0029176, DHJPAR0039504, DHJPAR0040367, DHJPAR0040373, DHJPAR0040374. Depository: CNC.

###### Etymology.

*Chelonusjunkoshimurae* is named to honor Sra. Junko Shimura of the Convention for Biological Diversity for her biopolitical support of BioAlfa and conservation of tropical biodiversity.

**Figure 120.**
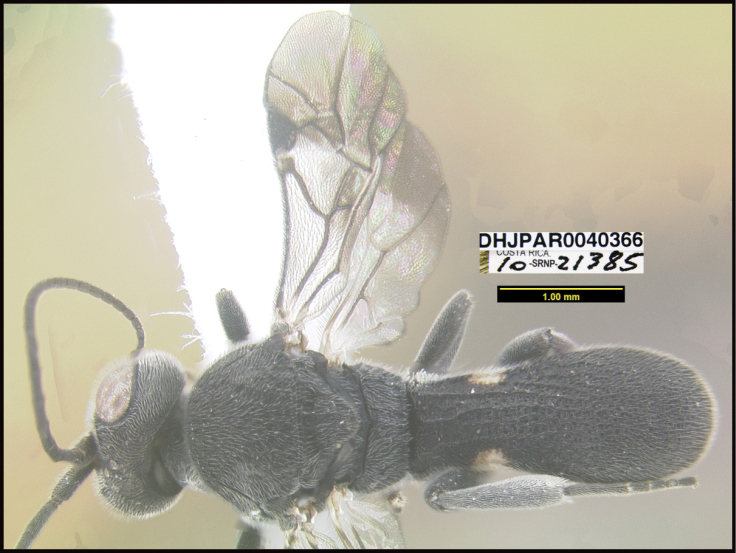
*Chelonusjunkoshimurae*, holotype.

##### Chelonus kateperezae

Taxon classificationAnimaliaHymenopteraBraconidae

Sharkey sp. nov.

http://zoobank.org/897CDE06-4307-45D2-9B80-5E347CF359AB

Figure 121

###### Diagnostics.

BOLD:ACR8387. Consensus barcode. TATTTTATGTTTGGTATATGATCAGGTATATTAGGTTTATCTTTAAGATTAATTATTCGAATAGAATTAGGAACTTTAAAAACTTTATTGTTTAATGATCAATTATATAATAGATTAGTTACTTTACATGCTTTTGTAATAATTTTTTTTATGGTTATGCCAGTAATAATTGGTGGTTTTGGAAATTGATTAATTCCTTTAATATTAGGTTTATCAGATATATTATTTCCACGTATAAATAATATAAGTTTTTGATTATTAGTGCCATCAATGATTTTATTATTAATAAGTGGTTTTGTAAATGTTGGAGTTGGTACAGGTTGAACGGTTTATCCTCCTTTATCTTCTTTAATTGGTCATAGGGGTGTATCGGTTGATATATCGATTTTTTCTTTACATTTAGCAGGGATATCATCAATTATGGGAGCAATTAATTTTATTGTAACTGTTATAAATACTTGATTAAAATATAATTTTATAGATAAATTTCCTTTATTTGTTTGATCAGTGTATATTACAGCTATTTTATTATTATTGTCTTTACCAGTTTTAGCAGGGGCTATTACTATATTGTTAAGGGAT---------------------------------------------------.

###### Holotype ♀.

Guanacaste, Sector Santa Rosa, Bosque San Emilio, 10.8438, -85.6138, 300 meters, 04‒11/vi/2012, Malaise trap. Depository: CNC.

***Host data*.** None.

***Holotype voucher code*.**BIOUG17967-C10.

###### Paratypes.

None.

###### Etymology.

*Chelonuskateperezae* is named to honor Ms. Kate Perez for her many years laboring in execution and administration of the field and laboratory actions performed by the Centre for Biodiversity Genomics to rapidly and accurately DNA barcode tens of thousands of Costa Rican insects from Malaise traps installed by ACG and BioAlfa.

**Figure 121.**
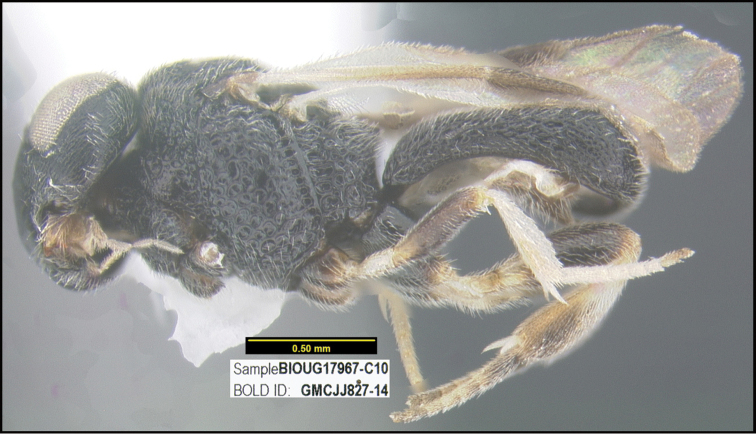
*Chelonuskateperezae*, holotype.

##### Chelonus luciariosae

Taxon classificationAnimaliaHymenopteraBraconidae

Sharkey sp. nov.

http://zoobank.org/44660355-15FF-48BC-8BDE-3786E8A31516

Figure 122

###### Diagnostics.

BOLD:ACM2853. Consensus barcode. TACTTTATATTTAATTTTTGGTATATGATCAGGTATATTTGGTTTATCTTTAAGTTTATTAATTCGTTTAGAATTAAGTGGATTAATAAGATTTTTTAATAATGATCAATTATATAATAGAATAATTACTTTACATGCTTTTATTATAATTTTTTTTATAGTAATACCAATTATAATAGGTGGTTTTGGAAATTGATTAATTCCAATAATATTGGGTTTAATAGATATAGCTTTTCCTCGATTAAATAATTTAAGGTTTTGATTATTAATTCCTTCAATTATATTATTAGTTATAAGTAGATTTGTAAATAGTGGTTCTGGTACAGGTTGAACAGTTTATCCTCCATTATCTTCATTAATAGGTCATGGGGGGGTATCAGTAGATATAACTATTTTTTCTTTACATTTAGCAGGTGTGTCTTCAATTTTAAGATCAATTAATTTTATTGTAACTATTTTAAATAGTTGAATAAATGTAAAGTATATAGATAAATTTCCTTTATTTATTTGATCTGTTTTTCTTACAGCAATTTTATTATTATTATCTTTACCTGTATTAGCAGGGGCAATTACTATATTATTAAGAGATCGAAATATAAATACTAGATTTTTTGATTCTTCAGGAGGAGGTGATCCTATTTTGTATCAGCATTTATTT.

###### Holotype ♂.

Alajuela, Sector San Cristobal, Jardin Estrada, 10.865, -85.397, 722 meters, caterpillar collection date: 30/xii/2013, wasp eclosion date: 24/i/2014. Depository: CNC.

***Host data*.** chryBioLep01 BioLep174 (Pyralidae) feeding on *Mansoahymenaea* (Fabaceae).

***Caterpillar and holotype voucher codes*.** 13-SRNP-7596, DHJPAR0054557.

###### Paratypes.

Host = chryBioLep01: BioLep174, DHJPAR0054549, DHJPAR0054550, DHJPAR0054552, DHJPAR0054555, DHJPAR0054559, DHJPAR0054561, DHJPAR0054563, DHJPAR0054567, DHJPAR0054782. Depository: CNC.

###### Etymology.

*Chelonusluciariosae* is named to honor Sra. Lucia Rios of GDFCF and ACG for her many years as a dedicated inventory parataxonomist for ACG.

**Figure 122.**
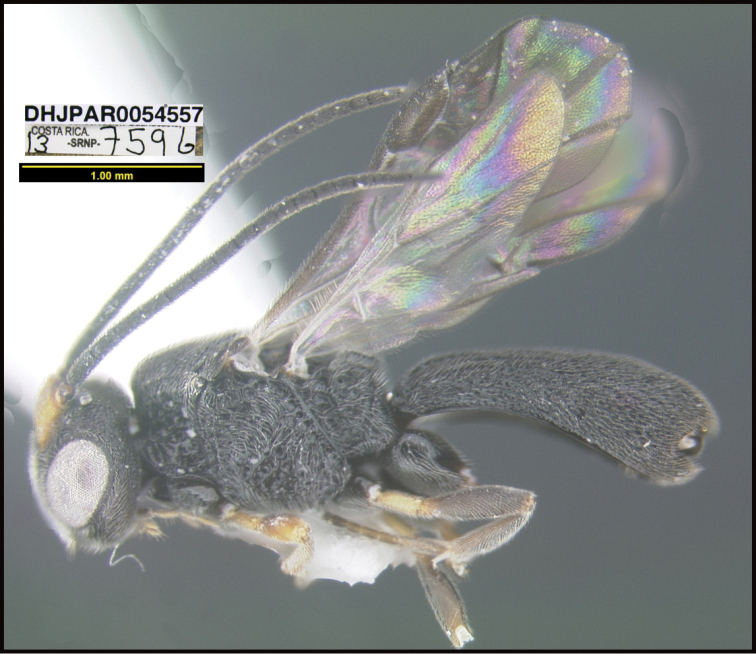
*Chelonusluciariosae*, holotype.

##### Chelonus luzmariaromeroae

Taxon classificationAnimaliaHymenopteraBraconidae

Sharkey sp. nov.

http://zoobank.org/5E315E19-5A3A-4092-BD8A-88E5DD2784D2

Figure 123

###### Diagnostics.

BOLD:AAM1444. Consensus barcode. GACATTATATTTTTTATTTGGTATATGATCAGGGTTTTTTGGTTTATCATTAAGAATATTAATTCGTATAGAATTAAGTTCTTTAGGTAGATTATTAATAAATGATCAATTGTATAATAGAATTGTAACTATGCATGCTTTTATTATAATTTTTTTTATAGTTATACCAATTATAATTGGTGGATTTGGTAATTGGATAGTTCCTTTAATGTTAGGTATATGTGATATAATATTTCCTCGAATAAATAATATAAGATTTTGGATATTAATACCTTCTTTAATAATATTAATTTTAGGAAGGTTAGTAAATATGGGTGTTGGGACAGGATGAACAGTTTATCCTCCTTTATCTTTATTAATAGGACATGGGGGGATYTCTTTAGATTTATCAATTTTTTCTTTACATTTAGCAGGGATTTCTTCAATTTTAGGTTCTATTAATTTTATTGTAACTATTTTAAATACTTGRATAGAAAAAAAATTTTTGGATATATTTCCTTTATTTATTTGATCAGTATTTATTACAACAATTTTATTATTATTATCATTACCAGTATTAGCTGGCGCAATTACTATACTTTTAAGTGATCGTAATTTAAATACAAGATTTTTTGATCCTGCTGGTGGAGGGGATCCTGTATTATATCAACATTTATTT.

###### Holotype ♂.

Alajuela, Sector San Cristobal, Tajo Angeles, 10.865, -85.415, 540 meters, caterpillar collection date: 12/viii/2011, wasp eclosion date: 28/viii/2011. Depository: CNC.

***Host data*.***Dichomeris* Janzen703 (Gelechiidae) feeding on *Koanophyllonhylonoma* (Asteraceae).

***Caterpillar and holotype voucher codes*.** 11-SRNP-3178, DHJPAR0048167.

###### Paratypes.

Hosts = *Dichomeris* Janzen703, *Dichrorampha* Janzen322. DHJPAR0037974, DHJPAR0037975. Depository: CNC.

###### Etymology.

*Chelonusluzmariaromeroae* is named to honor Sra. Luz Maria Romero for her decades of conducting and developing the ACG Biological Education Program, followed by more than a decade of mentoring the parataxonomists as to how to better manage and polish their field databases and their Species Pages on the ACG web site.

**Figure 123.**
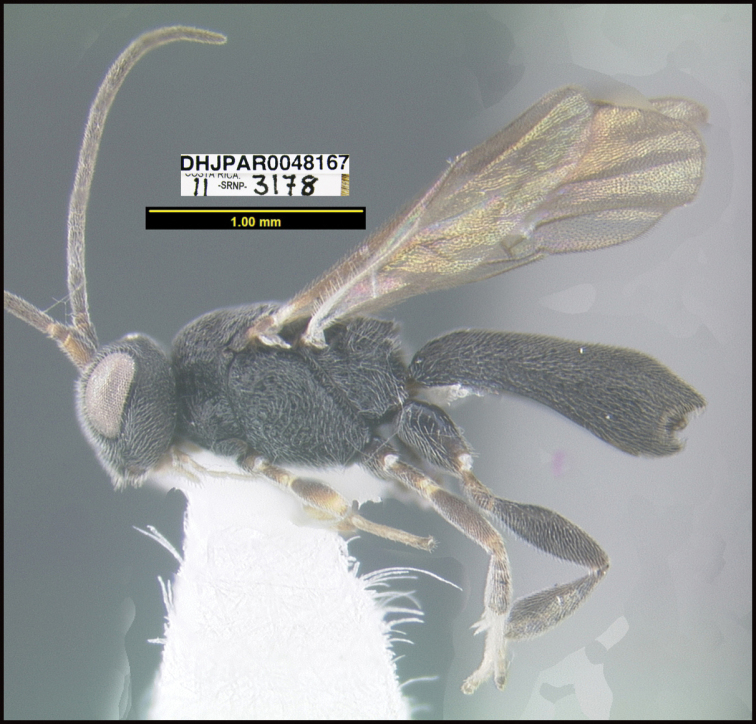
*Chelonusluzmariaromeroae*, holotype.

##### Chelonus manuelpereirai

Taxon classificationAnimaliaHymenopteraBraconidae

Sharkey sp. nov.

http://zoobank.org/0D813931-7C78-4E1D-B03C-0B14E77A159D

Figure 124

###### Diagnostics.

BOLD:ACP7116. Consensus barcode. AACATTATATTTTTTATATGGAATATGATCTGGATTTTTTGGTCTATCACTAAGTATATTAATTCGAATAGAATTAAGATCTTTAGGAAGATTTTTATTAAATGATCAATTATATAATAGAATTGTTACAATACATGCATTTATTATAATTTTTTTTATAGTTATACCTATTATAGTAGGTGGTTTTGGAAATTGAATAGTTCCTATAATATTAGGTTTACCAGATATAATTTTTCCACGAATAAATAATTTAAGATTTTGATTATTAATTCCATCATTAATTTTATTAATTTTAAGTAGTTTAGTTAATTCAGGAGTTGGTACAGGTTGAACCGTTTATCCACCTTTATCATTATTAATAGGTCATAGAGGAATTTCAGTTGATTTATCAATTTTTTCTTTACATTTAGCTGGAGCATCTTCAATTTTAGGATCAATTAATTTTATTGTAACTACATTAAATACTTGAATAAAAAAAAATATTATAGATACTTATCCATTATTTATTTGATCAATTTTTATTACTACAATTTTATTATTATTATCTTTACCTGTTTTAGCTGGTGCAATTACAATATTATTAAGAGATCGAAATTTAAATACAAGATTTTTTGACCCAGCAGGTGGGGGAGATCCTGTACTTTATCAACATTTATTT.

###### Holotype ♂.

Alajuela, Sector Rincon Rain Forest, Sendero Juntas, 10.907, -85.288, 400 meters, caterpillar collection date: 30/iv/2014, wasp eclosion date: 12/v/2014. Depository: CNC.

***Host data*.** gelJanzen01 Janzen501 (Gelechiidae) feeding on *Gouaniapolygama* (Rhamnaceae).

***Caterpillar and holotype voucher codes*.** 14-SRNP-42146, DHJPAR0055499.

###### Paratypes.

None.

###### Etymology.

*Chelonusmanuelpereirai* is named to honor Sr. Manuel Pereira of GDFCF and ACG for his many years as a dedicated inventory parataxonomist for ACG.

**Figure 124.**
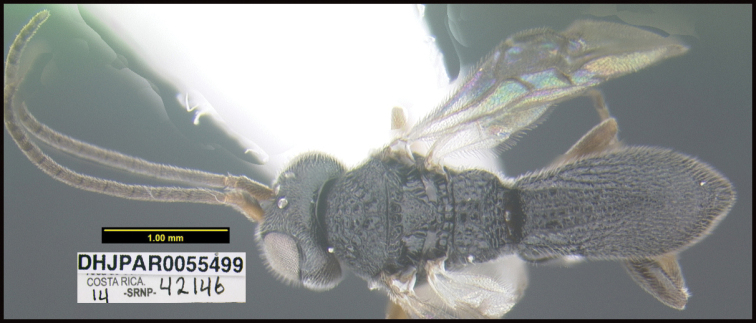
*Chelonusmanuelpereirai*, holotype.

##### Chelonus manuelzumbadoi

Taxon classificationAnimaliaHymenopteraBraconidae

Sharkey sp. nov.

http://zoobank.org/8A153768-F490-4F0B-BAA3-26E031C68307

Figure 125

###### Diagnostics.

BOLD:ACW6597. Consensus barcode. GTTTTATATTTTATTTTTGGAATATGGAGGGGGGTTTTGGGTTTGTCTTTAAGATTAATTTTACGAATAGAATTAAGTGTTTTAGGTAGATTAATTAAAAATGATCAATTATATAATAGTGTAGTAACTATACATGCTTTTGTAATAATTTTTTTTATGGTTATACCAGTTATAATTGGTGGTTTTGGATTAGTTCCATTAATGTTGGGTTTGCCAGATATAGCATTTCCTCGAATGAATAATATAAGATTTTGATTATTAATTCCTTCATTATTATTATTATTATTAGGGGGGTTTATTAATAGAGGAGTGGGAACAGGGTGAACTGTATATCCTCCATTATCGTCATTAATTGGTCACGGTGGAATTTCTGTAGATTTATCAATTTTTTCTTTACATTTAGCAGGGATATCTTCAATTATAGGAGCTATTAATTTTATTGTAACAGTAATAAATATAAGTTTTGATTTAAAATGTATAGATAAATATTCATTATTTGTATGGTCAGTTTTGATTACAGCGGTTTTATTGTTGTTATCATTACCTGTTTTGGCTGGTGCAATTACTATATTATTAAGTGAT.

###### Holotype ♀.

Guanacaste, Sector San Cristobal, Estación San Gerardo, 10.8801, -85.3889, 575 meters, 23‒30/xii/2013, Malaise trap. Depository: CNC.

***Host data*.** None.

***Holotype voucher code*.**BIOUG23153-E04.

###### Paratypes.

None.

###### Etymology.

*Chelonusmanuelzumbadoi* is named to honor Sr. Manuel Zumbado as an outstanding parataxonomist turned curator of Diptera in the INBio-MN insect collection and now part of the BioAlfa national biodiversity inventory.

**Figure 125.**
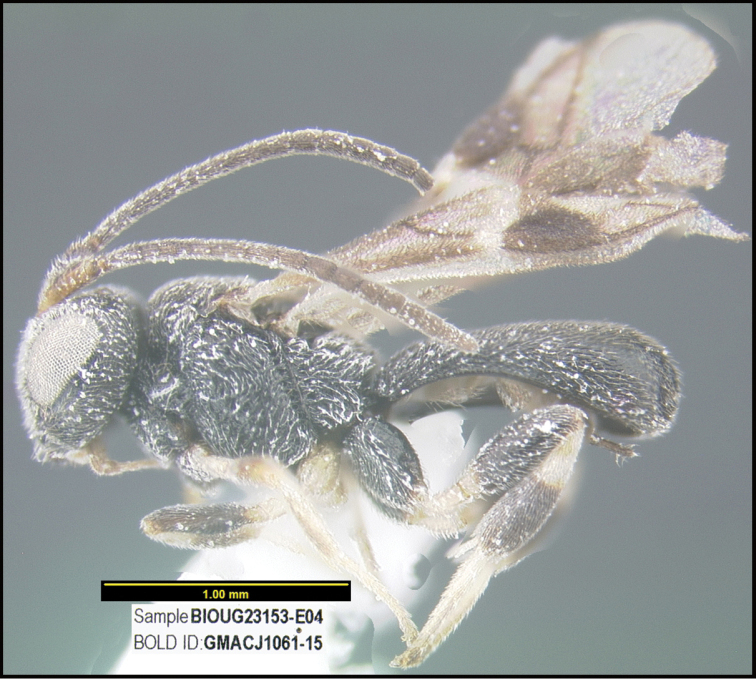
*Chelonusmanuelzumbadoi*, holotype.

##### Chelonus marianopereirai

Taxon classificationAnimaliaHymenopteraBraconidae

Sharkey sp. nov.

http://zoobank.org/2AAA7D76-F632-4409-B00A-E2001FBF8492

Figure 126

###### Diagnostics.

BOLD:ADC0083. Consensus barcode. TTTATTTTTGGAATTTGAGCAGGAGTTTTAGGTTTATCTTTAAGATTAATTTTACGAATAGAATTAAGTGTATTAGGAAGCTTATTAAAAAATGATCAATTATATAATAGTGTGGTTACTATACATGCTTTTGTAATAATTTTTTTTATGGTGATACCAATTATGATTGGGGGATTTGGTAATTGATTAATTCCATTAATATTAGGATTACCTGATATAGCTTTTCCACGAATAAATAATATAAGATTTTGATTATTAATTCCTTCATTAATATTATTGTTATTAAGAAGATTTGTAAATACAGGTGTAGGAACTGGATGAACAGTTTATCCACCATTATCATCATTAATAGGTCATGGAGGAATTTCCGTGGATTTATCAATTTTTTCTTTACATTTAGCAGGTATATCTTCAATTATAGGAGCAATTAATTTTATTGTTACAGTTTTAAATACAAATTTTGATATAAATTATATAGACAAATTTCCATTATTTGTATGATCTGTATTAATTACAGCAATTTTATTATTATTGTCATTACCTGTTTTAGCTGGTGCAATTACTATA------------------------------------------------------------------.

###### Holotype ♀.

Guanacaste, Pailas Dos, PL12-3, 10.7631, -85.3344, 820 meters, 06‒13/xi/2014, Malaise trap PL12-3A. Depository: CNC.

***Host data*.** None.

***Holotype voucher code*s**: BIOUG30255-D09.

###### Paratypes.

None.

###### Etymology.

*Chelonusmarianopereirai* is named to honor Sr. Mariano Pereira formerly of GDFCF and ACG for his many years as a dedicated inventory parataxonomist for ACG.

**Figure 126.**
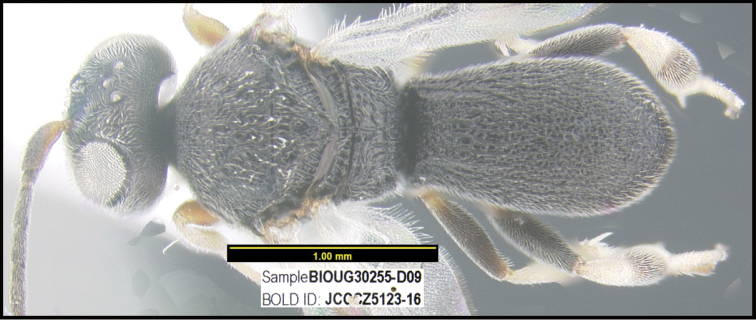
*Chelonusmarianopereirai*, holotype.

##### Chelonus maribellealvarezae

Taxon classificationAnimaliaHymenopteraBraconidae

Sharkey sp. nov.

http://zoobank.org/9DC0539E-EA83-4F4A-B66C-E77640C240D3

Figure 127

###### Diagnostics.

BOLD:AAM1066. Consensus barcode. AGTATTATATTTTATTTTTGGKATATGATCGGGGGTTTTGGGTTTATCTYTAAGGTTAATAATTCGTATAGAATTRAGTATATTAGGTAGTATATTTATAAATGATCAATTATATAATAGAATTGTTACTATACATGCTTTTATTATAATTTTTTTTATAGTTATACCAATTATAATTGGAGGATTTGGAAATTGATTAGTACCATTAATATTAGGTTTACCAGATATAATTTTTCCTCGAATAAATAATATAAGATTTTGATTAYTAATTCCTTCTTTATTATTATTAATTATAAGAAGTTTYGTAAATACAGGTGTAGGAACTGGATGAACAGTTTATCCTCCATTATCTTTATTAATAGGTCATAGGGGTATTTCAGTAGATTTATCAATTTTTTCTTTACATTTAGCAGGTATRTCTTCAATTATRGGGTCAATTAATTTTATTGTTACTGTYTTAAATACATGAATAAATAATAAATTAATAGATAARTTTTCTTTATTTGTTTGATCRATTTTTATTACAACAATTTTATTATTGTTATCTTTACCTGTTTTAGCGGGRGCTATTACTATATTAYTAAGGGATCGAAATTTAAATACTAGATTTTTTGATCCTTCTGGAGGGGGGGATCCTATTTTATATCAACATTTATTT.

###### Holotype ♂.

Guanacaste, Sector Mundo Nuevo, Vado Miramonte, 10.772, -85.434, 305 meters, caterpillar collection date: 18/xii/2009, wasp eclosion date: 31/xii/2009. Depository: CNC.

***Host data*.***Antaeotricha* Janzen221 (Depressariidae) feeding on *Desmodiumprocumbens* (Fabaceae).

***Caterpillar and holotype voucher codes*.** 09-SRNP-58092, DHJPAR0038214.

###### Paratype.

Host = elachJanzen01 Janzen38: DHJPAR0051929.

###### Etymology.

*Chelonusmaribellealvarezae* is named to honor Sra. Maribelle Alvarez of CONAGEBIO for her collaboration and support with the entire biodiversity permitting needs for BioAlfa and other ACG Biodiversity inventory.

**Figure 127.**
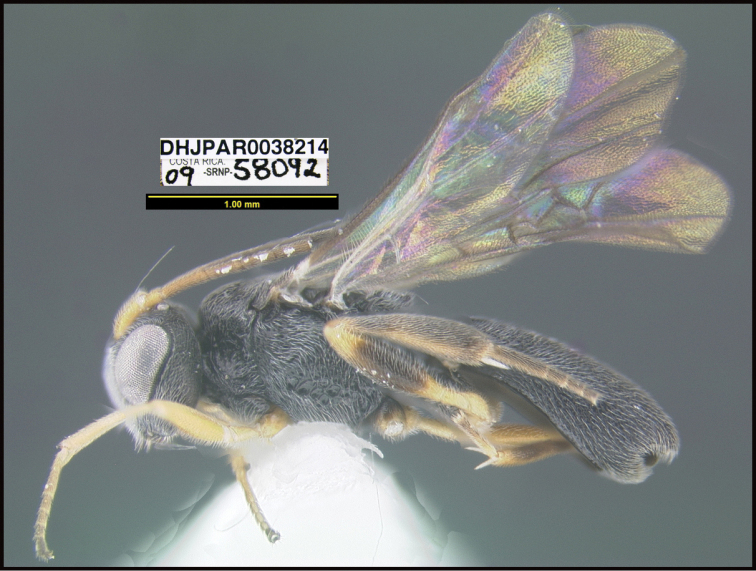
*Chelonusmaribellealvarezae*, holotype.

##### Chelonus markmetzi

Taxon classificationAnimaliaHymenopteraBraconidae

Sharkey sp. nov.

http://zoobank.org/E36B52F1-B99C-4B7A-8650-8FBA3BC914B8

Figure 128

###### Diagnostics.

BOLD:AAA4444. Consensus barcode. TATATTATATTTTATKTTTGGTATATGGGCTGGATTTTTGGGATTATCATTAAGATTATTAATTCGGATAGAATTGAGAGTGCTAGGYAGAGTATTTACTAATGAYCAATTATATAATAGAATTGTAACTATACATGCTTTTGTAATAATTTTTTTTATGGTTATACCAATTATGATTGGTGGATTTGGRAATTGGTTGGTACCTTTGATATTAGGATTACCGGATATAATTTTTCCTCGAATAAATAATATAAGTTTTTGATTATTAATTCCATCTTTAATATTATTAATATTAGGGGGATTTGTCAATATAGGGGTAGGTACTGGTTGAACAGTTTACCCCCCATTATCTTTATTAATAGGTCATAGAGGAGTTTCTGTTGATTTATCAATTTTTTCTTTACATATAGCGGGAGTYTCATCTATTATAGGATCRGTAAATTTTATTGTAACTATTTTAAATACTTGAATAAAAAATGATTTTATAGATAAATTTCCTTTATTTATTTGATCTGTATTTATTACTACTATTTTATTATTATTATCGTTACCWGTATTAGCAGGGGCTATTACTATATTATTAAGTGAYCGTAATTTAAATACAAGATTTTTTGATCCATCAGGTGGGGGGGATCCAATTTTATATCAACATTTATTT.

###### Holotype ♀.

Guanacaste, Sector Mundo Nuevo, Estación La Perla, 10.767, -85.433, 325 meters, caterpillar collection date: 18/v/2011, wasp eclosion date: 31/v/2011. Depository: CNC.

***Host data*.***Herpetogrammaphaeopteralis* (Crambidae) feeding on *Axonopusfissifolius* (Poaceae).

***Caterpillar and holotype voucher codes*.** 11-SRNP-5549, DHJPAR0045350.

###### Paratypes.

Hosts = *Herpetogrammaphaeopteralis*. DHJPAR0045335, DHJPAR0045355, DHJPAR0055996, DHJPAR0055997, DHJPAR0056052, BIOUG17653-H06, BIOUG18660-H04. Depository: CNC.

###### Other material.

Specimens from Mexico (BIOUG19755-A08, BIOUG20526-D01) share the same BIN on BOLD and may be conspecific. They were not examined.

###### Etymology.

*Chelonusmarkmetzi* is named to honor Dr. Mark Metz of the Smithsonian Institution and USDA for his open-mindedness in exploring and conducting the biopolitics of micromoth taxonomy and patronyms for ACG and BioAlfa.

**Figure 128.**
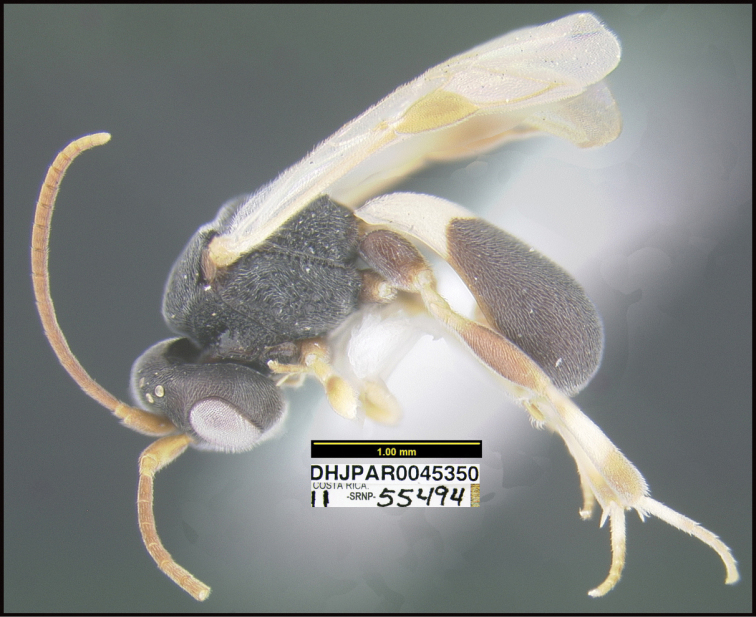
*Chelonusmarkmetzi*, holotype.

##### Chelonus markshawi

Taxon classificationAnimaliaHymenopteraBraconidae

Sharkey sp. nov.

http://zoobank.org/02143B0E-E380-4706-B6B3-F736FBCCD763

Figure 129

###### Diagnostics.

BOLD:ACL6642. Consensus barcode. GATACTTTATTTTATTTTTGGTATTTGATCAGGAATATTAGGTTTATCTTTGAGAATAATTATTCGTTTAGAGTTAATTTATGTTGGGAGATTTTTAACAAATGATCAGTTATATAATAATATTGTAACTATACATGCATTTATTATAATTTTTTTTATAGTAATGCCAGTAATAATTGGGGGGTTTGGAAATTGATTGGTTCCTTTAATATTAGGATTATCAGATATATCTTTTCCTCGTATAAATAATATAAGATTTTGATTATTAATTCCTTCAATTTTAATAATGTTAATAAGGGGGTTTGTAAATATGGGTGCAGGAACTGGATGAACAGTGTATCCTCCTTTATCTTTAAGTATGGGTCATAGTGGTATTTCTGTAGATATAGTAATTTTTTCTTTACATTTAGCTGGGATATCTTCTATTATGGGTGCTATTAATTTTATTACTACTGTAATGAATACTTGAATAAATGTAAAATTAATAGATAAATTTCCTCTATTTATTTGATCTGTTTATATTACAGCAATTTTATTATTGTTATCATTGCCTGTGTTGGCAGGAGCAATTACTATATTATTAAGAGATCGTAATATAAATACAAGATTTTTTGATTCATCTGGTGGTGGAGATCCTGTATTATATCAACATTTATTT.

###### Holotype ♀.

Alajuela, Sector Rincon Rain Forest, Quebrada Escondida, 10.899, -85.275, 420 meters, caterpillar collection date: 01/vii/2013, wasp eclosion date: 04/ix/2013. Depository: CNC.

***Host data*.***Stenoma* Janzen142 (Depressariidae) feeding on *Calyptrogynetrichostachys* (Arecaceae).

***Caterpillar and holotype voucher codes*.** 13-SRNP-42923, DHJPAR0053672.

###### Paratypes.

None.

###### Etymology.

*Chelonusmarkshawi* is named to honor Dr. Mark Shaw of National Museums Scotland for his many decades of exploring the natural history and taxonomy of European parasitic Hymenoptera.

**Figure 129.**
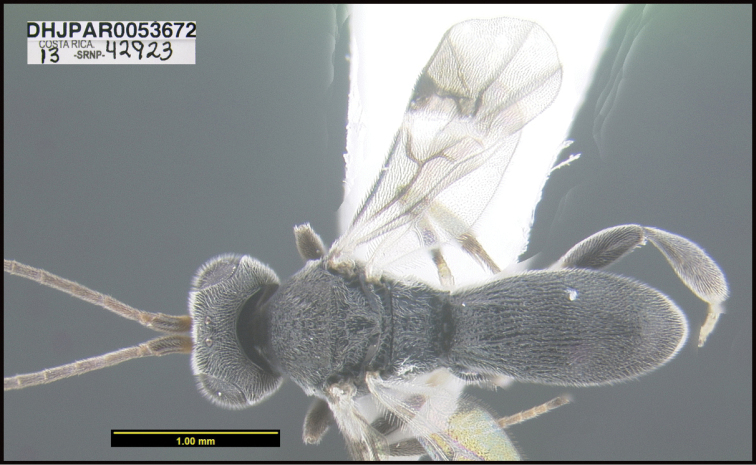
*Chelonusmarkshawi*, holotype.

##### Chelonus martajimenezae

Taxon classificationAnimaliaHymenopteraBraconidae

Sharkey sp. nov.

http://zoobank.org/850545F0-13F0-4268-A7CB-B7F7D75AE078

Figure 130

###### Diagnostics.

BOLD:ADA0878. Consensus barcode. GTATTATATTTTTTATTTGGATTATGATCAGGTGTAATAGGTTTATCATTAAGAATAATTATTCGTTTTGAGTTAATATATCCTGGAGCATTTATAGGAAATGATCAATTATATAATAGAGTTGTTACTATACATGCATTTGTAATGATTTTTTTTATAGTTATACCGGTAATAATTGGAGGATTTGGAAATTGATTAATTCCTTTAATATTAGGTTTACCGGATATAGCTTTTCCACGAATAAATAATATAAGGTTTTGATTATTAATACCTTCAATATTATTYTTAATTATAGGTAGATTATTAAATAGAGGCACTGGTACTGGATGAACCGTTTATCCACCTTTATCTTTAATTATAGGTCATAGTGGTATTTCAGTTGATTTTACAATTTTTTCTTTACATTTGGCTGGAATATCATCAATTATAGGTGCTTTAAATTTTATTGTTACTATTATTAATACTTGAATAAGAAAAATTTATATAGATAAATTTTCTTTRTTTGTATGATCAGTATTTATTACAGCAATTTTGTTATTAATATCTTTACCTGTTTTAGCTGGGGCAATTACTATATTATTAAGTGATCGTAATATTAAT.

###### Holotype ♂.

Alajuela, Sector San Cristobal, Estación San Gerardo, 10.8801, -85.389, 575 meters, 25/viii‒01/ix/2014, Malaise trap. Depository: CNC.

***Host data*.** None.

***Holotype voucher code*.**BIOUG27764-E10.

###### Paratypes.

None.

###### Etymology.

*Chelonusmartajimenezae* is named to honor Sra. Marta Jimenez, former director of CONAGEBIO, for her welcoming and exploratory attitude about Costa Rican national biodiversity inventory preview of BioAlfa.

**Figure 130.**
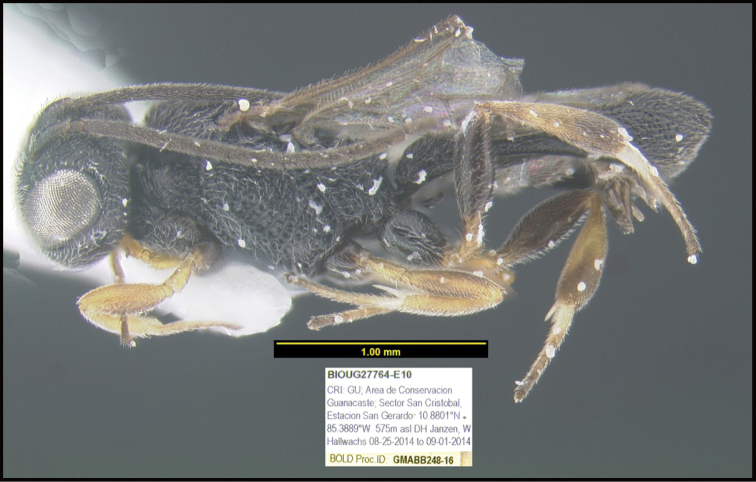
*Chelonusmartajimenezae*, holotype.

##### Chelonus mayrabonillae

Taxon classificationAnimaliaHymenopteraBraconidae

Sharkey sp. nov.

http://zoobank.org/EE46CACA-428A-4F16-8217-61FCC46A261D

Figure 131

###### Diagnostics.

BOLD:ADB0889. Consensus barcode. GTATTATATTTTTTATTTGGATTATGATCAGGTGTAATAGGTTTATCATTAAGAATAATTATTCGTTTTGAGTTAATATATCCTGGAGCATTTATAGGAAATGATCAATTATATAATAGAGTTGTTACTATACATGCATTTGTAATGATTTTTTTTATAGTTATACCGGTAATAATTGGAGGATTTGGAAATTGATTAATTCCTTTAATATTAGGTTTACCGGATATAGCTTTTCCACGAATAAATAATATAAGGTTTTGATTATTAATACCTTCAATATTATTYTTAATTATAGGTAGATTATTAAATAGAGGCACTGGTACTGGATGAACCGTTTATCCACCTTTATCTTTAATTATAGGTCATAGTGGTATTTCAGTTGATTTTACAATTTTTTCTTTACATTTGGCTGGAATATCATCAATTATAGGTGCTTTAAATTTTATTGTTACTATTATTAATACTTGAATAAGAAAAATTTATATAGATAAATTTTCTTTRTTTGTATGATCAGTATTTATTACAGCAATTTTGTTATTAATATCTTTACCTGTTTTAGCTGGGGCAATTACTATATTATTAAGTGATCGTAATATTAAT.

###### Holotype ♀.

Guanacaste, Pailas Dos, PL12-9, 10.76, -85.3341, 809 meters, 03‒10/vii/2014, Malaise trap PL12-9A. Depository: CNC.

***Host data*.** None.

***Holotype voucher code*.**BIOUG29390-E08.

###### Paratypes.

All Malaise-trapped. BIOUG28728-G11, BIOUG28876-C11, BIOUG29391-H05, BIOUG29395-G08, BIOUG29397-E02, BIOUG29501-H09, BIOUG29509-E11. Depository: CNC.

###### Etymology.

*Chelonusmayrabonillae* is named to honor Sra. Mayra Bonilla of Costa Rica for her unflagging support of the Stroud-Bonilla family for decades of development and management of ACG.

**Figure 131.**
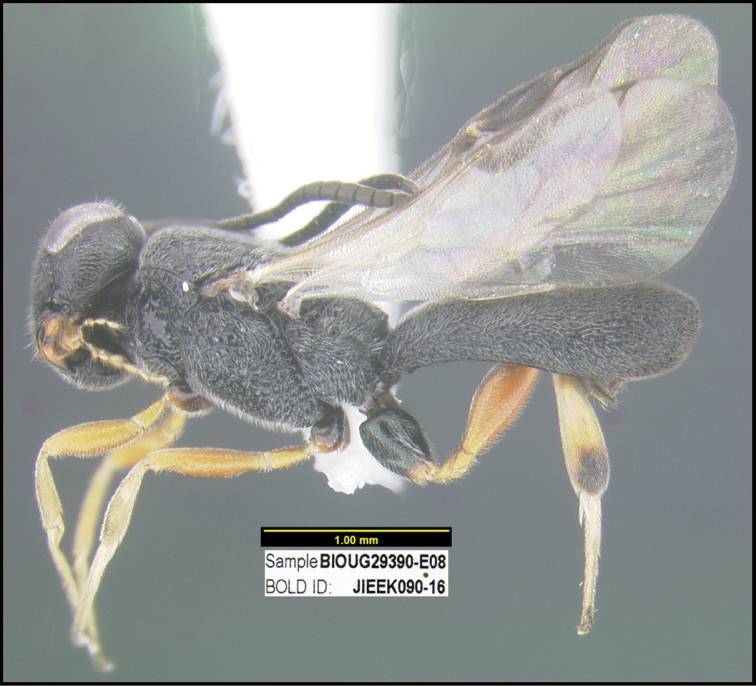
*Chelonusmayrabonillae*, holotype.

##### Chelonus meganmiltonae

Taxon classificationAnimaliaHymenopteraBraconidae

Sharkey sp. nov.

http://zoobank.org/3787BB74-CBA8-4608-BE52-4FA3D75FEE1C

Figure 132

###### Diagnostics.

BOLD:ADA8853. Consensus barcode. ATATTATATTTTATTTTTGGAATTTGGTCTGGTATTTTGGGTTTATCATTAAGTATAATAATTCGAATAGAATTAAATTGTACTGGAAGTTTGTTTATTAATGATCAATTATATAATAGATTAGTAACTATACATGCGTTTGTAATAATTTTTTTTATGGTTATACCAGTAATAATTGGTGGTTTTGGAAATTGATTAATTCCTTTAATATTAGGATTATCTGATATAGCTTTTCCTCGAATAAATAATATGAGTTTTTGATTATTAATTCCTTCAATTTTTATATTAATTATTAGTAGATTTGTAAATATAGGTGTTGGTACTGGTTGGACTGTATATCCACCTTTATCTTCATTATTAGGTCATAGTGGTGTATCTGTTGATATAGCTATTTTTTCTTTACATTTAGCTGGAATATCATCAATTATAGGTGCTATTAATTTTATTGTTACTATTTTAAATACATGAATAATAAATTTATTAATAGATAAATTTCCATTATTTGTTTGATCAGTATTTATTACTGCTTTATTATTATTATTATCATTACCTGTATTAGCTGGTGCAATTACTATATTATTAAGAGATCGTAAT---------------------------------------.

###### Holotype ♂.

Guanacaste, Pailas Dos, PL12-1, 10.7642, -85.335, 828 meters, 20‒27/ii/2014, Malaise trap PL12-1A. Depository: CNC.

***Host data*.** None.

***Holotype voucher code*.**BIOUG28769-G03.

###### Paratypes.

None.

###### Etymology.

*Chelonusmeganmiltonae* is named to honor Sra. Megan Milton for her many years laboring in execution and administration of the field and laboratory actions performed by the Centre for BioDiversity Genomics to rapidly and accurately DNA barcode tens of thousands of Costa Rican insects found in ACG.

**Figure 132.**
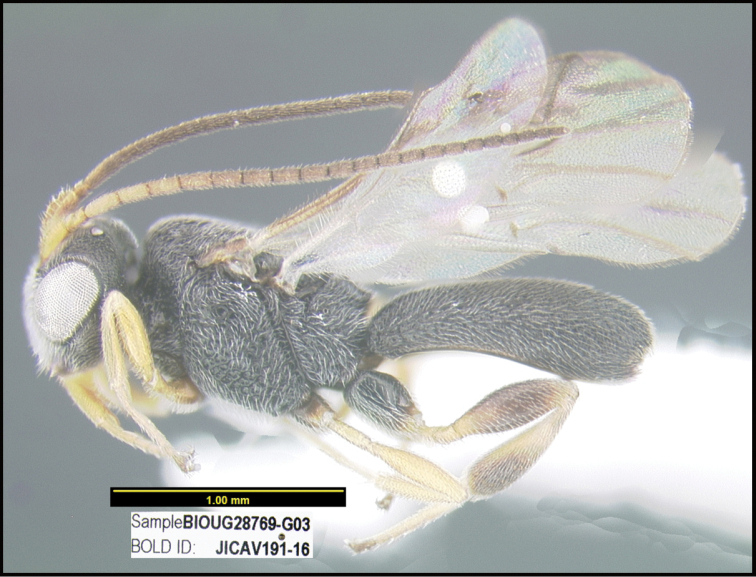
*Chelonusmeganmiltonae*, holotype.

##### Chelonus melaniamunozae

Taxon classificationAnimaliaHymenopteraBraconidae

Sharkey sp. nov.

http://zoobank.org/7B616FF6-D3C8-4AD1-A877-4431420D70C4

Figure 133

###### Diagnostics.

BOLD:AAM1107. Consensus barcode. AATTTTATACTTTATTTTTGGAATATGATCAGGTATATTAGGTTTATCTTTAAGTATAATAATTCGTTTAGAATTAGCTTTTACTGGAAGTTTATTTATTAATGATCAATTATATAATTTATTAGTAACTTTACATGCTTTTGTAATAATTTTTTTTATAGTAATGCCATTAATAATTGGAGGATTTGGAAATTGATTAATTCCTTTAATATTAGGTTTACCTGATATAGCTTTTCCTCGAATGAATAATATAAGATTTTGACTATTAATTCCTTCAATTTTTTTATTAATTATAAGAAATTTTGTTAATATAGGAGTAGGTACTGGATGAACAGTATATCCTCCATTATCTTCTTTAATAGGTCATAGAGGTATTTCTGTTGATTTGGCTATTTTTTCTTTACATTTGGCAGGAATATCTTCTATTATAGGAGCTATTAATTTTATTGTAACTGTAATTAATWCTTGAATAAAAAATTTATATATAGATAAATTATCTTTATTTGTTTGATCTGTTTTTATTACTGCAATTTTATTATTATTATCATTACCTGTATTAGCAGGAGCTATTACTATATTATTAAGAGATCGTAATATAAATACAAGATTTTTTGATTCATCTGGGGGAGGAGATCCTATTTTATATCAACATTTATTT.

###### Holotype ♂.

Guanacaste, Sector Pitilla, Sendero Mismo, 10.988, -85.42, 680 meters, caterpillar collection date: 10/iii/2010, wasp eclosion date: 25/iii/2010. Depository: CNC.

***Host data*.** gelJanzen01 Janzen758 (Gelechiidae) feeding on *Coccolobaporphyrostachys* (Polygonaceae).

***Caterpillar and holotype voucher codes*.** 10-SRNP-30674, DHJPAR0039043.

###### Paratypes.

Hosts = gelJanzen01 Janzen758. DHJPAR0039052, DHJPAR0039064, DHJPAR0039065. Depository: CNC.

###### Etymology.

*Chelonusmelaniamunozae* is named to honor Sra. Melania Muñoz of CONAGEBIO for her collaboration and support with the entire biodiversity permitting needs for BioAlfa and other ACG Biodiversity inventory.

**Figure 133.**
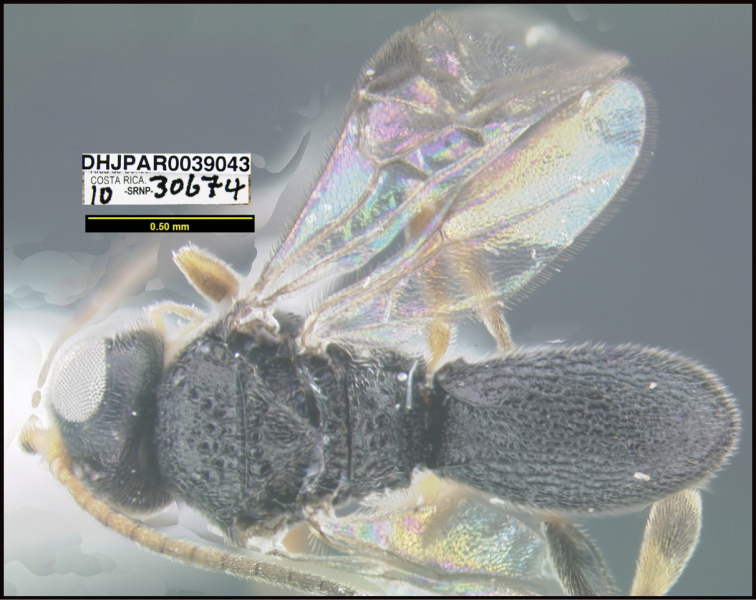
*Chelonusmelaniamunozae*, holotype.

##### Chelonus michaelstroudi

Taxon classificationAnimaliaHymenopteraBraconidae

Sharkey sp. nov.

http://zoobank.org/4BAD763C-520B-488D-8F94-66D03001EB02

Figure 134

###### Diagnostics.

BOLD:ACR3626. Consensus barcode. TATATTATATTTTATTTTTGGAATATGATCTGGAATATTTGGTTTATCATTGAGTATAATTATTCGTTTAGAATTAATTTATCCTGGAAGT---TTATTAATGAATGATCAATTGTATAATAGG------GTAATTACTATACATGCTTTTGTAATAATTTTTTTTATAGTTATACCTATTATAATTGGGGGTTTTGGAAATTGATTAATTCCTTTAATGTTGGGTTTACCGGATATAGCATTTCCTCGTATAAATAATATAAGATTTTGATTATTAATCCCTTCATTAATTATATTAATTATAGGAGGTTTTATTAATATAGGAGCAGGTACTGGTTGAACTATTTATCCTCCATTATCATCATTAATAGGACATAGTGGAATATCTGTAGATTTATCTATTTTTTCTTTGCATTTAGCTGGAATATCTTCAATTATAGGTGCTATTAATTTTATTGTTACAGTATTAAATACTTGAATAGATATT-AAATTTATAGATAAATATCCATTATTTGTATGATCAGTATTTATTACAGCTTTTTTATTATTATTATCATTACCTGTATTAGCAGGAGCAATTACTATATTATTAAGGGATCGAAATATAAATACAAGATTTTTTGATTCTTCTGGAGGGGGAGATCCAGTTTTATATCAACATTTATTT.

###### Holotype ♂.

Guanacaste, Sector Pitilla, Medrano, 11.016, -85.381, 380 meters, caterpillar collection date: 11/vi/2014, wasp eclosion date: 26/vi/2014. Depository: CNC.

***Host data*.** gelJanzen01 Janzen485 (Gelechiidae) feeding on *Rinoreadeflexiflora* (Violaceae).

***Caterpillar and holotype voucher codes*.** 14-SRNP-71020, DHJPAR0056003.

###### Paratypes.

None.

###### Etymology.

*Chelonusmichaelstroudi* is named for Sr. Michael Stroud for his highly meritorious behavior of collaborating with his parents in their efforts to support ACG as a whole and biodiversity development specifically.

**Figure 134.**
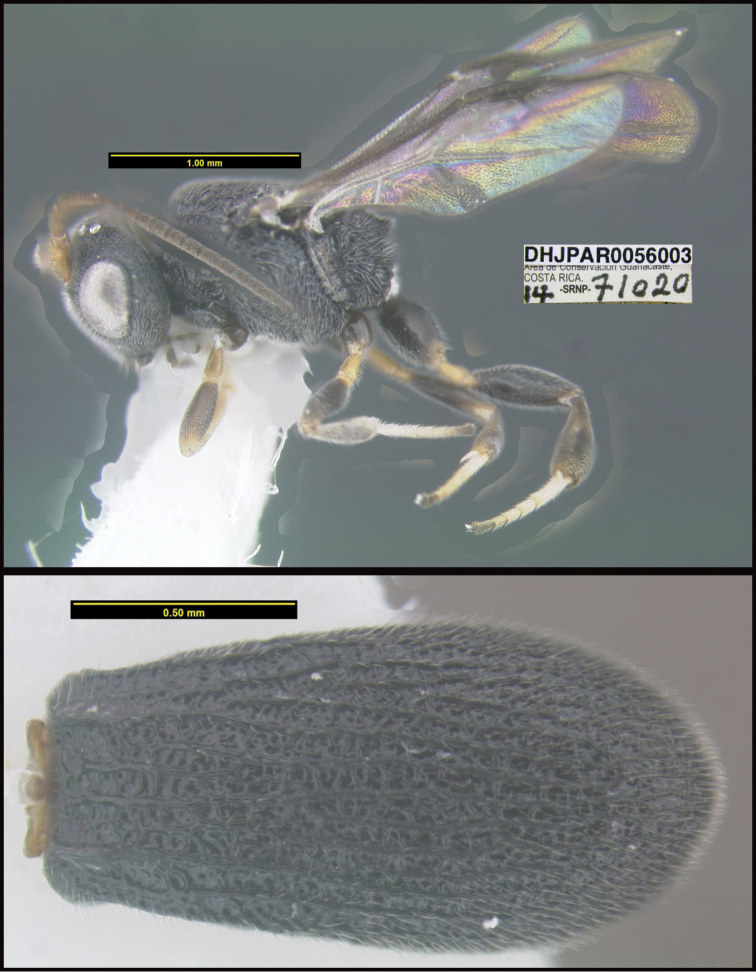
*Chelonusmichaelstroudi*, holotype.

##### Chelonus michellevanderbankae

Taxon classificationAnimaliaHymenopteraBraconidae

Sharkey sp. nov.

http://zoobank.org/F4DE854C-9490-45C0-AEE4-D706B9291E79

Figure 135

###### Diagnostics.

BOLD:AAT8838. Consensus barcode. TGTTTTATATTTTATTTTTGGAATATGATCAGGTATATTAGGATTATCTTTAAGATTATTAATTCGTATGGAATTGAGAATAACTGGAAGATTATTAATAAATGATCAATTATATAATAGTATTGTTACATTACATGCTTTTATTATAATTTTTTTTATAGTTATACCAATTATAATTGGTGGATTTGGTAATTGATTAATTCCTTTAATATTAGGGTYACCTGATATAGCTTTTCCWCGAATAAATAATATAAGATTTTGGTTATTAATTCCGTCAATTTTTTTATTGATTTTAGGTGGATTTGTTAATATAGGAGCTGGGACTGGTTGAACAGTTTATCCTCCATTATCTTTATTAATTGGTCATAGTGGAGCTTCAGTGGATATATCAATTTTTTCATTACATTTGGCTGGAATATCTTCAATTATAGGAGCAATTAATTTTATTGTAACAGTAATTAATACATGAATAGATATAAAATTAATAGATAAATTTTCTTTATTTGTTTGATCAGTATTTATTACTGCAATTTTATTATTGTTGTCTTTGCCTGTATTAGCTGGTGCTATTACTATATTATTAAGAGATCGTAATTTAAATACAAGATTTTTTGATCCATCTGGTGGGGGGGATCCTGTTTTATATCAGCATTTATTT.

###### Holotype ♀.

Guanacaste, Sector Pitilla, Sendero Mismo, 10.988, -85.42, 680 meters, caterpillar collection date: 16/ii/2010, wasp eclosion date: 08/iii/2010. Depository: CNC.

***Host data*.** Gregarious parasitoid of *Anacrusisnephrodes* (Tortricidae) feeding on *Zygiapalmana* (Fabaceae).

***Caterpillar and holotype voucher codes*.** 10-SRNP-30487, DHJPAR0038977.

###### Paratypes.

Host = *Anacrusisnephrodes*: DHJPAR0029134, DHJPAR0029135, DHJPAR0039447, DHJPAR0040018, DHJPAR0040021, DHJPAR0040406, DHJPAR0047069, DHJPAR0049118, DHJPAR0050964, DHJPAR0055415. Depository: CNC.

###### Etymology.

*Chelonusmichellevanderbankae* is named for Dr. Michelle Van Der Bank of South Africa in recognition of her broad efforts to introduce DNA barcoding into the process of understanding South African biodiversity for its conservation.

**Figure 135.**
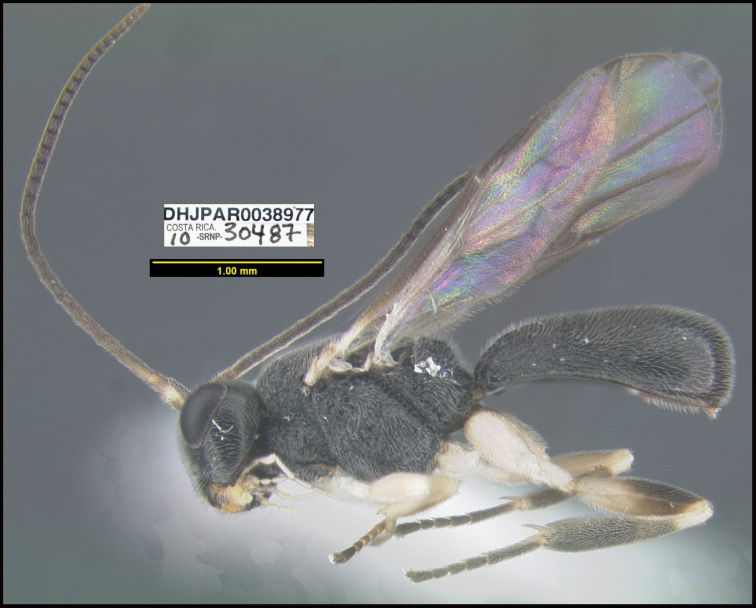
*Chelonusmichellevanderbankae*, holotype.

##### Chelonus mingfangi

Taxon classificationAnimaliaHymenopteraBraconidae

Sharkey sp. nov.

http://zoobank.org/D03A34F0-3F6A-482A-A279-9525BE55A3D9

Figure 136

###### Diagnostics.

BOLD:ADA5459. Consensus barcode. ATTTTATATTTTTTATTTGGTATATGGTCAGGAATATTAGGATTATCTTTAAGAATATTAATTCGTATAGAATTAAGGTTTTCTGGAAGGTTATTTTTAAATGATCAATTATATAATAGGATTGTTACTATACATGCTTTTGTTATAATTTTTTTTATAGTGATACCAATTATAATTGGGGGATTTGGAAATTGATTAATTCCTTTAATATTAGGATTACCTGATATAGCTTTTCCTCGAATAAATAATATAAGATATTGATTATTAATTCCTTCTTTATTAATATTAATAATAAGTGGATTTATTAATATAGGAGTAGGTACTGGATGAACTATTTATCCTCCTTTATCATTATTAATAGGTCATGGAGGTATTTCTGTGGATATATCAATTTTTTCTTTACATTTAGCTGGGATATCTTCTATTATAGGAGCTATTAATTTTATTATTACTATTATTAATACTTGAATAGATATTAAATTTATAGATAAGTTTCCTTTATTTGTTATGTCAGTTTTTATTACTGCTATTTTATTATTATTATCATTACCAGTATTAGCAGGGGCTATTACTATATTATTAAGAGATCGTAAT------------------------------------.

###### Holotype ♂.

Guanacaste, Sector San Cristobal, Estación San Gerardo, 10.8801, -85.389, 575 meters, 24‒31/iii/2014, Malaise trap. Depository: CNC.

***Host data*.** None.

***Holotype voucher code*.**BIOUG28344-E03.

###### Paratypes.

None.

###### Etymology.

*Chelonusmingfangi* is named for Mr. Ming Fang of Japan and Hawaii for his magnanimous collaboration with Mrs. Jessie Hill in supporting the growth and survival of the ACG parataxonomist program and its operations.

**Figure 136.**
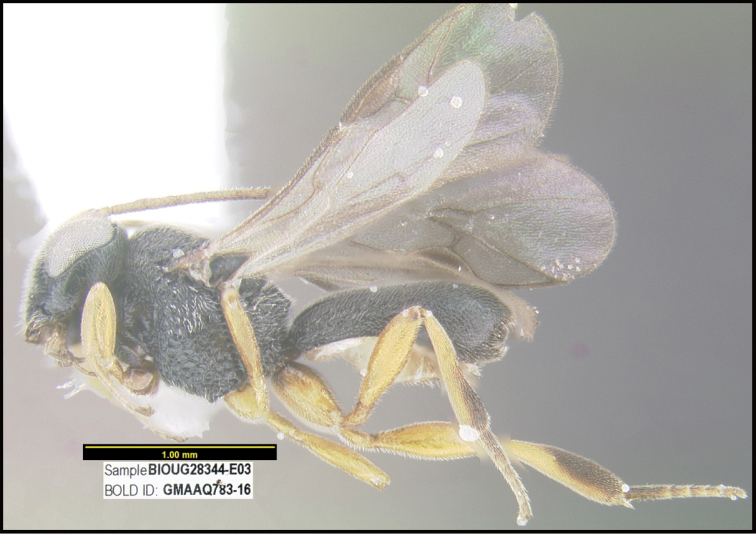
*Chelonusmingfangi*, holotype.

##### Chelonus minorcarmonai

Taxon classificationAnimaliaHymenopteraBraconidae

Sharkey sp. nov.

http://zoobank.org/1B96D92D-BE87-49BA-8A25-B863FACB6790

Figure 137

###### Diagnostics.

BOLD:ADB0320. Consensus barcode. ATTTTATATTTTTTATTTGGTATATGATCTGGTATATTAGGATTATCATTAAGTATATTAATTCGTATAGAATTAAGATTTTCTGGTAGGTTATTTATAAATGATCAATTATATAATAGAATTATTACTTTACATGCTTTTGTTATAATTTTTTTTATAGTTATACCTATTATAATTGGTGGATTTGGTAATTGATTAGTTCCTTTAATATTAGGTTTACCTGATATAGCTTTTCCTCGAATAAATAATATAAGATTTTGATTATTAATTCCTTCATTATTTATATTATTAATAAGAGGATTTATTAATATAGGTGTAGGTACAGGTTGAACAGTTTATCCACCTTTATCATTATTAATTGGTCATGGTGGAATTTCTGTTGATATATCAATTTTTTCTTTACATTTAGCTGGTATATCATCAATTATAGGTGCAATTAATTTTATTGTTACTATTATTAATACATGATTAAATATTAATTATATAGATAAATTTTCTTTATTTGTAATATCAGTTTTTATTACTGCAATTTTATTATTATTATCTTTACCAGTTTTAGCAGGTGCTATTACTATATTATTAAGGGATCGTAATATAAATACAAGATTTTTT.

###### Holotype ♀.

Guanacaste, Pailas Dos, PL12-3, 10.7631, -85.3344, 820 meters, 05‒12/xii/2013, Malaise trap PL12-3A. Depository: CNC.

***Host data*.** None.

***Holotype voucher code*.**BIOUG29282-H09.

###### Paratypes.

All Malaise-trapped. BIOUG28697-B07, BIOUG29112-C06, BIOUG29112-F08, BIOUG29415-F01. Depository: CNC.

###### Etymology.

*Chelonusminorcarmonai* is named to honor Sr. Minor Carmona of GDFCF and ACG for his many years as a dedicated inventory parataxonomist for ACG.

**Figure 137.**
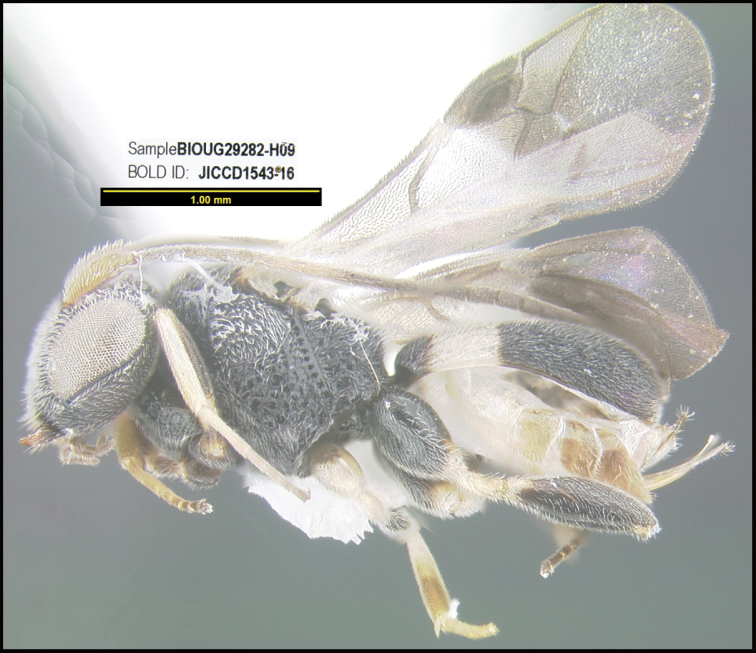
*Chelonusminorcarmonai*, holotype.

##### Chelonus monikaspringerae

Taxon classificationAnimaliaHymenopteraBraconidae

Sharkey sp. nov.

http://zoobank.org/095F7A77-3C26-4B51-82A9-436A6080BC5F

Figure 138

###### Diagnostics.

BOLD:ACU3782. Consensus barcode. ATATTATATTTTATATTTGGAATATGATCTGGTATATATGGTTTGTCTTTAAGTTTATTAATTCGTATGGAATTAAGGATAGCAGGTAGGTTATTTGTGAATGATCAATTATACAATAGAGTTGTTACTTTACATGCTTTTATTATAATTTTTTTTATAGTTATGCCTATTATAATTGGTGGTTTTGGAAATTGATTAATTCCATTAATATTAGGTTTACCTGATATAGCATTTCCTCGTATAAATAATATAAGATTTTGGTTATTGATTCCATCTTTAATTATGTTAACATTAAGAGGATTTGTTAATATAGGGGCAGGTACTGGATGAACTGTTTATCCTCCTTTATCTTTATTAATTGGTCACGGGGGTATTTCTGTTGATATATCAATTTTTTCTTTACATTTAGCAGGGATATCTTCTATTATAGGGGCTATTAATTTTATTGTTACTATTATTAATACATGAATAAATAATAAATTTATAGATAAATTTTCTTTATTTGTTTGATCAGTTTTTATTACTGCTATTTTATTATTATTATCTTTACCTGTATTAGCAGGTGCTATTACTATATTATTAAGTGATCGTAAT------------------------------------------.

###### Holotype ♀.

Alajuela, Sector San Cristobal, Estación San Gerardo, 10.88, -85.389, 575 meters, 19‒26/viii/2013, Malaise trap. Depository: CNC.

***Host data*.** None.

***Holotype voucher code*.**BIOUG19587-A04.

###### Paratypes.

None.

###### Etymology.

*Chelonusmonikaspringerae* is named in honor of Dr. Monika Springer of the Universidad de Costa Rica for her welcoming reaction to the concept of DNA barcoding Costa Rican aquatic insects for their identification.

**Figure 138.**
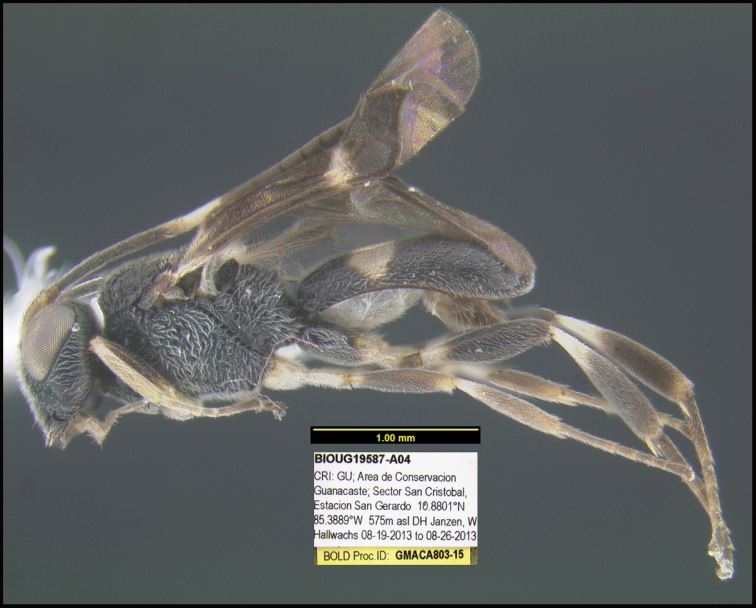
*Chelonusmonikaspringerae*, holotype.

##### Chelonus moniquegilbertae

Taxon classificationAnimaliaHymenopteraBraconidae

Sharkey sp. nov.

http://zoobank.org/4A270B26-3A4E-4563-A1C9-E987702F4E9B

Figure 139

###### Diagnostics.

BOLD:AAJ0370. Consensus barcode. TATATTATATTTTATTTTTGGAATATGGTCAGGAATATTAGGATTGTCGTTGAGTTTATTGATTCGTATAGAATTAAGGTTAACAGGTAGGTTATTTTTAAATGATCAACTTTATAATAGAGTAGTAACTATACATGCTTTTATTATAATTTTTTTTATAGTTATACCTATTATAATTGGTGGATTTGGAAATTGATTGGTTCCATTAATGTTAGGTTTACCTGATATAGCATTTCCTCGAATAAATAATATAAGATTTTGATTGTTAATTCCTGCTATTATTTTGATGATTTTAAGAGGGTTTGTTAATATAGGAGCTGGTACTGGTTGAACAGTTTATCCTCCTTTATCTTCTTTGATAGGACATGGTGGAATTTCTGTTGATATATCAATTTTTTCTTTACATTTAGCTGGAGCTTCATCAATTATAGGTGCTATTAATTTTATTGTTACAGTAATTAATACTTGAATAAATATTAAATTTATAGATAAATTTTCTTTATTTGTTTGATCAGTATTTATTACTGCTATTTTATTATTATTATCTTTACCTGTATTAGCGGGTGCTATTACTATATTATTAAGAGATCGTAATATAAATACAAGATTTTTTGATCCTTCTGGTGGTGGAGATCCAGTTTTATATCAACATTTATTT.

###### Holotype ♂.

Guanacaste, Sector Pitilla, Estación Quica, 10.997, -85.397, 470 meters, caterpillar collection date: 09/viii/2009, wasp eclosion date: 31/viii/2009. Depository: CNC.

***Host data*.** Gregarious parasitoid of *Antaeotrichaspurca* (Depressariidae) feeding on *Cassiagrandis* (introduced) (Fabaceae).

***Caterpillar and holotype voucher codes*.** 09-SRNP-71792, DHJPAR0040022.

###### Paratypes.

Hosts = *Omiodeshumeralis* (Crambidae) elachJanzen01 Janzen235 (Depressariidae), elachJanzen01 Janzen38 (Depressariidae). DHJPAR0020801, DHJPAR0020805, DHJPAR0042517, DHJPAR0051323. Depository: CNC.

###### Etymology.

*Chelonusmoniquegilbertae* is named in honor of Mrs. Monique Gilbert of the GDFCF office in Vermont who is Chief Development Officer for GDFCF as well as “general fixer of many important things”.

**Figure 139.**
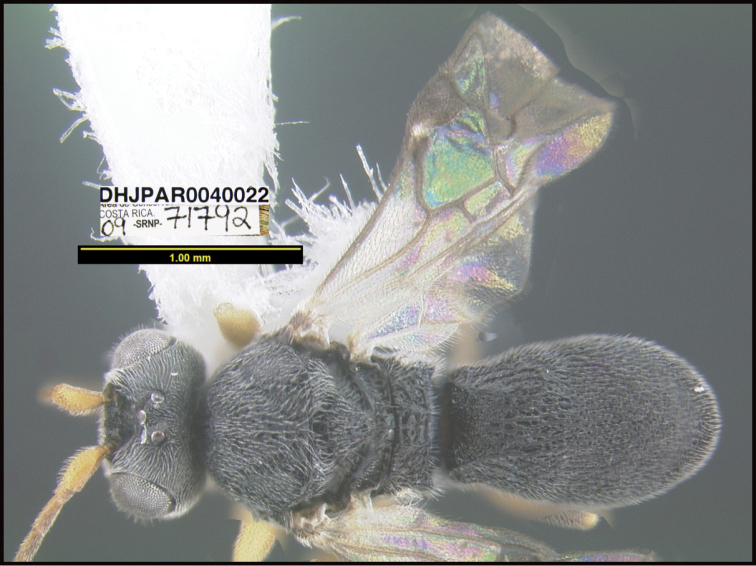
*Chelonusmoniquegilbertae*, holotype.

##### Chelonus motohasegawai

Taxon classificationAnimaliaHymenopteraBraconidae

Sharkey sp. nov.

http://zoobank.org/67B8F20E-B8BC-40C0-9ACD-142EDB3C8A8E

Figure 140

###### Diagnostics.

BOLD:AAW3555. Consensus barcode. TATTTTATATTTTATATTTGGTATATGAGCAGGTATATTAGGTTTATCATTAAGTTTATTAATTCGTATAGAATTAAGATTAAGTGGTAGTTTATTTATAAATGATCAATTATATAATAGTGTAGTTACTTTACATGCTTTTATTATAATTTTTTTTATAGTTATACCAATTATAATTGGTGGGTTTGGAAATTGATTAGTTCCTTTAATATTAGGATTACCTGATATAGCATTTCCTCGAATAAATAATATAAGATTTTGATTATTAGTTCCTTCTTTATTTTTAATATTATTAAGTAGAATAGTTAATATAGGTGTAGGAACTGGATGAACAGTTTATCCTCCTTTATCTTCATTAGTTGGTCATGGAGGAATTGCAGTAGATATATCAATTTTTTCTTTACATATAGCTGGGGCATCTTCTATTATAGGAGCAATTAATTTTATTGTTACTATTTTAAATTCTTGAATTGAAACTAAATTTATCGATAAATTTCCTTTATTTGTATGATCAGTATTTATTACTGCAATTTTATTATTGTTATCTTTACCTGTATTAGCTGGTGCAATTACAATGTTATTAAGAGATCGTAATTTAAATACAAGATTTTTTGATCCTTCAGGGGGAGGAGACCCAGTTTTATATCAACATTTATTT.

###### Holotype ♀.

Guanacaste, Sector Cacao, Estación Cacao, 10.927, -85.468, 1150 meters, caterpillar collection date: 07/i/2011, wasp eclosion date: 04/ii/2011. Depository: CNC.

***Host data*.** Gregarious parasitoid of *Anacrusisellensatterleeae* (Tortricidae) feeding on *Rondeletiacostaricensis* (Rubiaceae).

***Caterpillar and holotype voucher codes*.** 11-SRNP-35011, DHJPAR0042920.

###### Paratype.

Host = *Ardeutica* Brown002 (Tortricidae): DHJPAR0030992. Depository: CNC.

###### Etymology.

*Chelonusmotohasegawai* is named for Dr. Motohiro Hasegawa of JICA, Japan in recognition of his many years of facilitating and causing the integration of Costa Rican SINAC activities with ICE activities through his outstanding example for the Pailas II Geothermal Development Project beginning in 2013.

**Figure 140.**
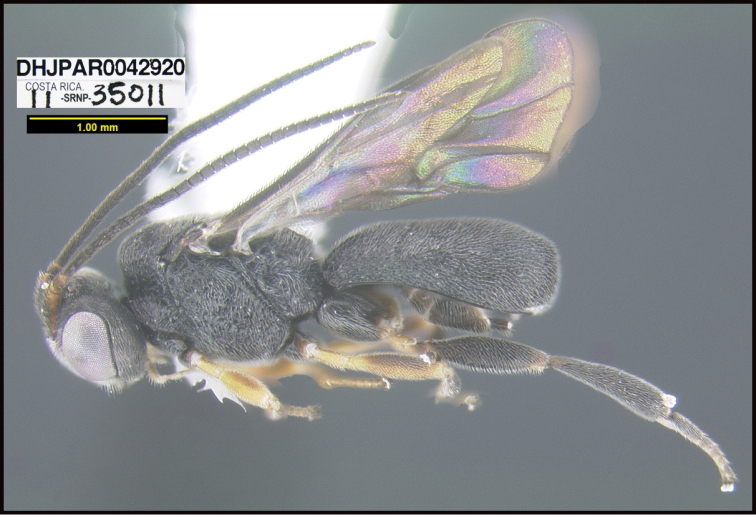
*Chelonusmotohasegawai*, holotype.

##### Chelonus nataliaivanovae

Taxon classificationAnimaliaHymenopteraBraconidae

Sharkey sp. nov.

http://zoobank.org/DBB3275F-091B-42EA-BBB7-77BF7BC114FD

Figure 141

###### Diagnostics.

BOLD:AAF2632. Consensus barcode. TATATTATATTTTATTTTTGGAATATGGTCAGGTATATTAGGGTTATCTTTAAGATTAATAATTCGAATAGAATTGAGATTAAGGGGAAGATTATTTATAAATGATCAATTATATAATAGAATTGTTACTTTACATGCTTTTGTTATAATTTTTTTTATAGTAATACCAATTATAATTGGGGGATTTGGAAATTGRTTAGTTCCTTTAATATTAGGGYTRCCAGATATAGCTTTTCCACGTATAAATAATATAAGATTTTGATTATTAATTCCTTCACTTTTTTTAATAATTTTAAGGAGGTTTATTAATATAGGTGTAGGGACAGGTTGAACAGTTTATCCTCCATTATCATCTTTAATTGGTCATGGTGGTATTTCAGTTGATATATCAATTTTTTCTTTACATTTAGCAGGYATATCTTCAATTATAGGAGCAATTAATTTTATTGTAACTATTTTAAATACATGAATTAATAATAAATTTATGGATAAATTCCCTTTATTTGTTTGATCTGTATTTATTACTGCAATTTTATTATTGTTATCTTTACCTGTATTAGCTGGGGCTATTACTATATTATTAAGTGATCGTAATTTAAATACTAGATTTTTTGATCCTTCTGGAGGGGGGGATCCTGTATTGTATCAGCATTTWTTT.

###### Holotype ♂.

Guanacaste, Sector Pitilla, Bullas, 10.987, -85.385, 440 meters, caterpillar collection date: 28/iv/2011, wasp eclosion date: 13/v/2011. Depository: CNC.

***Host data*.** Gregarious parasitoid of *Antaeotricha* Janzen140DHJ03 (Depressariidae) feeding on *Zygialongifolia* (Fabaceae).

***Caterpillar and holotype voucher codes*.** 11-SRNP-65195, DHJPAR0042960.

###### Paratypes.

Hosts = *Omiodeshumeralis* (Crambidae), *Spilomelapantheralis* (Crambidae), *Antaeotricha* BioLep38 (Depressariidae), *Antaeotricha* ianthinaEPR01 (Depressariidae), *Antaeotricha* Janzen140DHJ03 (Depressariidae), *Antaeotricha* Janzen401 (Depressariidae), *Antaeotrichaspurca* (Depressariidae). *Amorbiaproductana* (Tortricidae), *Amorbiarevolutana* (Tortricidae), *Anacrusisnephrodes* (Tortricidae), *Anacrusisturrialbae* (Tortricidae), *Megalotavulgaris* (Tortricidae), *Strepsicrates* Brown37 (Tortricidae). DHJPAR0029133, DHJPAR0034268, DHJPAR0035316, DHJPAR0035415, DHJPAR0035559, DHJPAR0039111, DHJPAR0040019, DHJPAR0040020, DHJPAR0045406, DHJPAR0049124, DHJPAR0049786, DHJPAR0052897, DHJPAR0053679, DHJPAR0055461, DHJPAR0056713. Depository: CNC.

###### Etymology.

*Chelonusnataliaivanovae* is named to honor Dr. Natalia Ivanova for her many years laboring in execution and administration of the laboratory actions performed by the Centre for Biodiversity Genomics to rapidly and accurately DNA barcode tens of thousands of Costa Rican insects.

**Figure 141.**
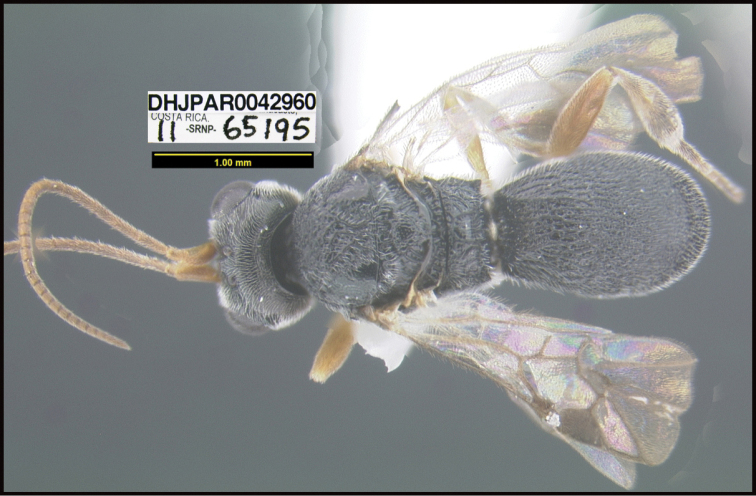
*Chelonusnataliaivanovae*, holotype.

##### Chelonus nelsonzamorai

Taxon classificationAnimaliaHymenopteraBraconidae

Sharkey sp. nov.

http://zoobank.org/9102140F-F0DA-473D-B49C-FDE454EB7666

Figure 142

###### Diagnostics.

BOLD:ADB4135. Consensus barcode. ATTTTATATTTTATTTTTGGTATATGATCTGGAATATTAGGATTATCATTAAGATTATTAATTCGTTTAGAATTAGGTATATTAGGTAGTTTATTAATAAATGATCAATTATATAATAGAATAGTTACTTTACATGCATTTGTAATAATTTTTTTTATAGTTATACCTATTATAATTGGAGGTTTTGGTAATTGATTAATTCCTTTAATGTTAGGTTTACCAGATATAGCATTTCCTCGAATGAATAATATAAGATTTTGATTATTAATTCCATCTATATTTATATTGTTAATAGGGGGATTTGTTAATGCTGGTGCTGGAACTGGTTGAACAGTTTATCCACCTTTATCTTCAATTATAGGACATAGGGGGGTATCTGTTGATATATCAATTTTTTCATTACATTTGGCTGGGATATCATCTATTATAGGTGCAATTAATTTTATTATTACTTCAATAAATACTTGAATATTAAATAAATATATTGATAAATTTCCTTTATTTGTGTGATCGGTTTTAATTACAGCTATTTTATTATTATTATCATTACCTGTATTAGCAGGGGCTATTACTATGCTTTTAAGTGATCGTAATTTAAATACA------------------------------.

###### Holotype ♀.

Guanacaste, Pailas Dos, PL12-9, 10.76, -85.3341, 809 meters, 19‒26/vi/2014, Malaise trap PL12-9A. Depository: CNC.

***Host data*.** None.

***Holotype voucher code*.**BIOUG29298-C10.

###### Paratypes.

None.

###### Etymology.

*Chelonusnelsonzamorai* is named to honor Dr. Nelson Zamora for his decades of taxonomic research and field surveys for the plants of Costa Rica, invaluable for the ecological understanding of ACG and the Costa Rica as a whole.

**Figure 142.**
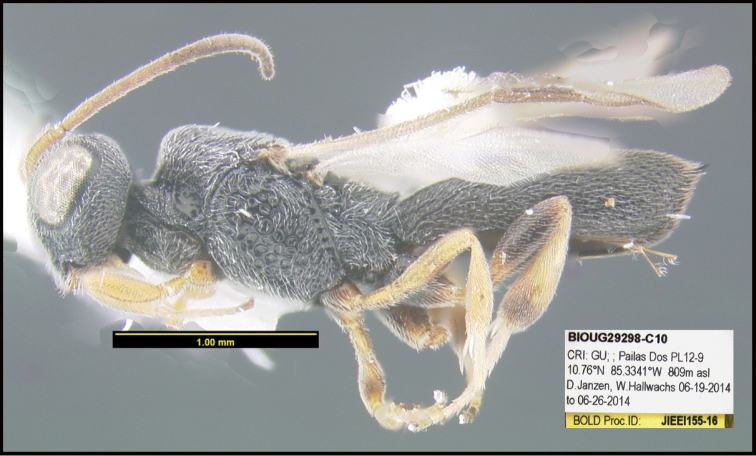
*Chelonusnelsonzamorai*, holotype.

##### Chelonus normwoodleyi

Taxon classificationAnimaliaHymenopteraBraconidae

Sharkey sp. nov.

http://zoobank.org/D118BBA8-3F6E-4E0A-BCA5-359486C9A962

Figure 143

###### Diagnostics.

BOLD:AAW3564. Consensus barcode. GATTTTGTATTTTATATTTGGGATATGAGCTGGAATATTAGGTTTATCATTAAGAATATTGATCCGAATAGAGTTGAGTGTAATTGGAAGATTAATATTAAATGATCAATTGTATAATAGAATTGTAACTTTGCATGCTTTTGTAATAATTTTTTTTATGGTGATACCAATTATAATTGGTGGTTTTGGAAATTGATTAGTTCCATTAATAATTGGTTTACCTGATATAGCTTTTCCTCGAATAAATAATATAAGTTATTGATTGTTGATTCCTTCTTTATTTTTATTATTATCAAGGGGATTTGTAAATACAGGAGTAGGTACTGGTTGGACAGTTTATCCTCCTTTATCTTCACTAACGGGTCATGGGGGTGTTTCTGTTGATTTGTCTATTTTTTCATTACATTTAGCTGGTTCTTCTTCTATTATAGGTGCTATTAATTTTATTGTTACAAGRTTGAATACTTGATTGAGTATTTATTATATGGATAAGATTTCTTTATTTGTTTGATCTGTATTAATTACTGCTATTTTATTATTGTTATCCTTACCGGTRTTAGCGGGTGCTATTACTATACTTTTAAGAGACCGAAATTTAAATACTAGATTTTTTGATCCTTCTGGTGGGGGTGACCCAGTATTATATCAACATTTATTT.

###### Holotype ♂.

Alajuela, Sector San Cristobal, Sendero Huerta, 10.93, -85.372, 527 meters, caterpillar collection date: 22/ii/2013, wasp eclosion date: 28/iii/2013. Depository: CNC.

***Host data*.***Stenoma* BioLep82 (Depressariidae) feeding on *Lueheaseemannii* (Malvaceae).

***Caterpillar and holotype voucher codes*.** 13-SRNP-880, DHJPAR0051920.

###### Paratypes.

Host = *Stenoma* BioLep82: DHJPAR0036402, DHJPAR0048089, DHJPAR0048090, DHJPAR0048196. Depository: CNC.

###### Etymology.

*Chelonusnormwoodleyi* is named to honor Dr. Normal Woodley of the Smithsonian Institution and USDA for his decades of Tachinidae fly taxonomy for ACG.

**Figure 143.**
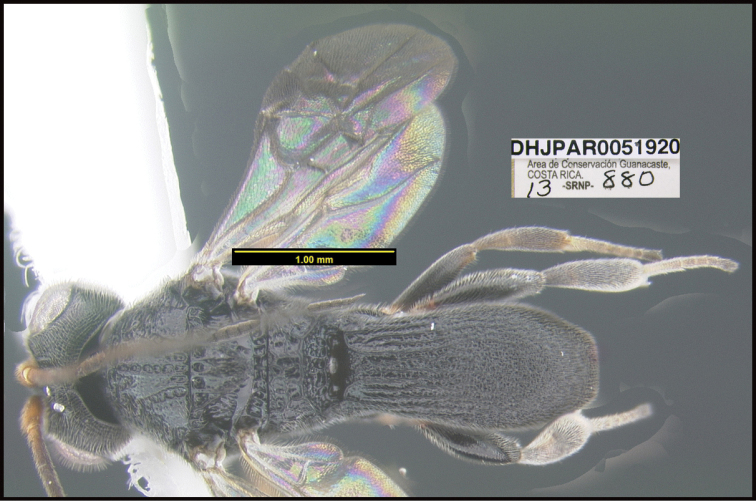
*Chelonusnormwoodleyi*, holotype.

##### Chelonus osvaldoespinozai

Taxon classificationAnimaliaHymenopteraBraconidae

Sharkey sp. nov.

http://zoobank.org/1CF9C9DC-F60A-4925-B275-B132541DDF38

Figure 144

###### Diagnostics.

BOLD:ABU8005. Consensus barcode. TGTATTATATTTTATTTTTGGTATATGATCTGGGATATTAGGTTTATCACTAAGTATATTAATTCGAATAGAATTAAGTTTGGTAGGTAGATTATTAATAAATGATCAGTTATATAATAGAATTGTTACTTTACATGCCTTTGTTATAATTTTTTTTATAGTTATACCAATTATAATTGGTGGATTTGGTAATTGATTAATTCCTTTAATATTAGGTTTACCTGATATAGCATTTCCACGTATAAATAATATAAGATATTGACTATTAATTCCTTCTTTATTTATATTATTATTAAGTGGATTTGTTAATATAGGGGTAGGTACTGGATGAACAGTTTATCCTCCTTTATCTTTATTGATTGGTCATGGGGGAATTTCTGTAGATTTATCTATTTTTTCTYTACATTTAGCTGGAATATCTTCTATTATAGGGGCYATTAATTTTATTACTACTAGATTAAATACTTGAATTAATAATAAGTATATGGATAAATTYCCTTTATTTGTTTGGTCAGTATTAATTACTGCTGTTTTATTATTATTGTCYTTACCTGTATTGGCAGGTGCTATTACTATATTATTAAGGGATCGAAATTTAAATACCAGATTTTTTGATCCATCTGGTGGGGGGGATCCAGTTTTATATCAGCATTTATTT.

###### Holotype ♂.

Alajuela, Sector Rincon Rain Forest, Sendero Venado, 10.897, -85.27, 420 meters, caterpillar collection date: 24/iv/2011, wasp eclosion date: 21/v/2011. Depository: CNC.

***Host data*.***Stenoma* Janzen230 (Depressariidae) feeding on *Xylopiafrutescens* (Annonaceae).

***Caterpillar and holotype voucher codes*.** 11-SRNP-41837, DHJPAR0042858.

###### Paratype.

Host = *Stenoma* Janzen230: DHJPAR0052184. Depository: CNC.

###### Etymology.

*Chelonusosvaldoespinozai* is named to honor Sr. Osvaldo Espinoza of GDFCF and ACG for his many years as a dedicated inventory parataxonomist for ACG.

**Figure 144.**
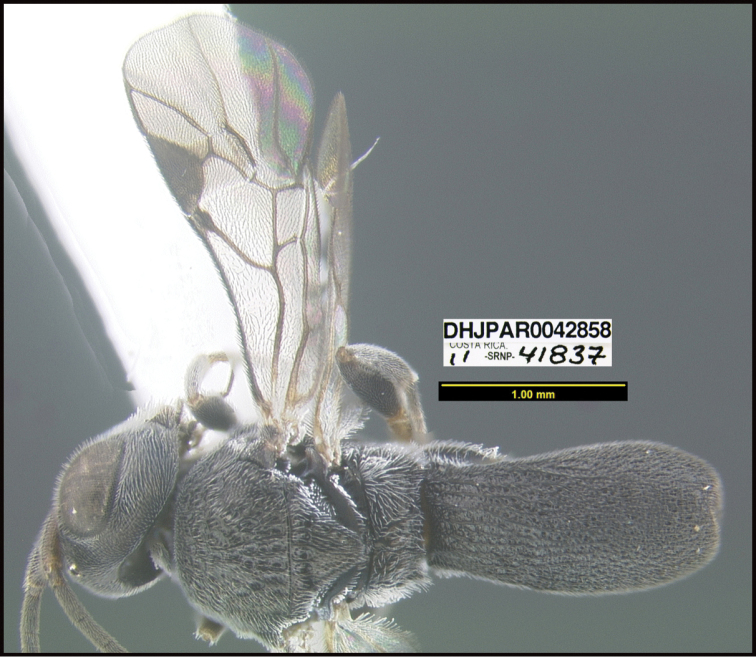
*Chelonusosvaldoespinozai*, holotype.

##### Chelonus pamelacastilloae

Taxon classificationAnimaliaHymenopteraBraconidae

Sharkey sp. nov.

http://zoobank.org/257D7007-E999-4561-AAC0-AADED3913464

Figure 145

###### Diagnostics.

BOLD:AAK1568. Consensus barcode. GATATTATATTTTATTTTTGGTATATGATCGGGAATTTTAGGTTTATCTTTGAGAATATTAATTCGTATAGAGTTAAGATTAAGAGGAAGTTTGTTAATAAATGATCAGTTATATAATAGAATTGTTACTTTACATGCTTTTATTATAATTTTTTTTATGGTTATACCAATTATAATTGGGGGTTTTGGAAATTGATTAGTTCCTTTAATGTTAGGAGTTCCAGATATAGCATTTCCACGAATAAATAATATAAGATATTGGTTATTAATTCCGTCTTTATTTATATTAATTATAAGAAGATTTGTTAATGTAGGTGTAGGTACGGGTTGAACAGTTTATCCACCATTATCTTTATTAATTGGTCATGGTGGAGTTTCTGTAGATATATCAATTTTTTCTTTACATTTAGCTGGGATATCTTCAATTATAGGTGCTATTAATTTTATTGTTACTGGGGCAAATAGATGAATAAAGAATAAATTTATAGATAAATATCCATTATTTGTATGGTCAGTATTGATTACTGCGTTATTATTACTATTATCTTTGCCAGTATTGGCGGGAGCTATTACTATATTATTAAGAGATCGTAATATAAATACTAGGTTTTTTGATCCTTCAGGTGGTGGGGATCCAGTATTATATCAACATTTGTTT.

###### Holotype ♂.

Alajuela, Sector Rincon Rain Forest, Sendero Venado, 10.897, -85.27, 420 meters, caterpillar collection date: 29/vii/2011, wasp eclosion date: 26/viii/2011. Depository: CNC.

***Host data*.***Antaeotricha* BioLep91 (Depressariidae) feeding on *Tachigalicostaricensis* (Fabaceae).

***Caterpillar and holotype voucher codes*.** 11-SRNP-43517, DHJPAR0045401.

###### Paratypes.

None.

###### Etymology.

*Chelonuspamelacastilloae* is named for Sra. Pamela Castillo, Costa Rica’s Vice-Minister for the Environment in the Ministry of the Environment and Energy (MINAE) and for her welcoming response to BioAlfa for Costa Rica.

**Figure 145.**
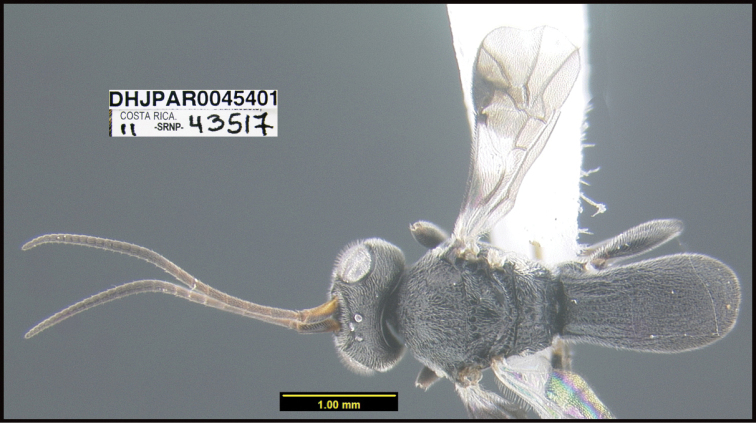
*Chelonuspamelacastilloae*, holotype.

##### Chelonus paulgoldsteini

Taxon classificationAnimaliaHymenopteraBraconidae

Sharkey sp. nov.

http://zoobank.org/54BEFAD8-22E1-4AEC-8A9E-A4D6D2094E13

Figure 146

###### Diagnostics.

BOLD:AAE9140. Consensus barcode. GGTGTTATATTTTATTTTTGGGATATGGGCCGGAGTATTGGGCTTATCATTAAGTATATTAATTCGAATGGAATTAAGGGTGAGAGGTAGGTTATTTATAAATGATCAGTTATATAATAGAATTGTGACTTTACATGCTTTTGTTATAATTTTTTTTATAGTTATGCCAATTATAATTGGGGGGTTTGGGAATTGGYTGATTCCTTTAATATTAGGGGTTCCGGATATGGCTTTTCCTCGAATGAATAATATAAGTTATTGGTTATTAATTCCTTCTTTATTTATATTGGTAGTTAGGAGGTTTATTAATATGGGGGTAGGTACGGGATGAACCGTTTATCCTCCTTTATCATTATTAATTGGTCATGGGGGYGTGTCAGTGGATATATCAATTTTTTCTTTACATTTAGCTGGTGCGTCTTCAATTATAGGGGCGATTAATTTTATTGTGACAGGGGTTAATACGTGGATAAGTAATAAATTAATAGATAAGTTCCCTTTATTTGTGTGATCAGTAATAATTACTGCGGTATTATTGCTATTGTCTTTACCAGTTTTAGCTGGGGCAATTACAATGTTGTTAAGGGATCGTAATTTAAATACAAGATTTTTCGATCCTYMRGGGGGGGGGGRTCCTGTATTATATCAGCATTTATTT.

###### Holotype ♀.

Guanacaste, Sector San Cristobal, Tajo Angeles, 10.865, -85.415, 540 meters, caterpillar collection date: 23/ii/2011, wasp eclosion date: 23/iii/2011. Depository: CNC.

***Host data*.** elachBioLep01 BioLep754 (Depressariidae) feeding on *Tapirirabrenesii* (Anacardiaceae).

***Caterpillar and holotype voucher codes*.** 11-SRNP-830, DHJPAR0042851.

###### Paratypes.

Hosts = *Stenoma* BioLep86 (Depressariidae), elachBioLep01 BioLep754 (Depressariidae). DHJPAR0035526, DHJPAR0035527, DHJPAR0035528, DHJPAR0039120, DHJPAR0042855, DHJPAR0046940, DHJPAR0048102, DHJPAR0048980, DHJPAR0051921, DHJPAR0051923, DHJPAR0051928, DHJPAR0056954, DHJPAR0056976. Depository: CNC.

###### Etymology.

*Chelonuspaulgoldsteini* is named for Dr. Paul Goldstein of the Smithsonian Institution and USDA in recognition of his intense care and curation and taxonomic processing of the thousands of species of DNA barcoded ACG moths being deposited in the collections of the National Museum of Natural History.

**Figure 146.**
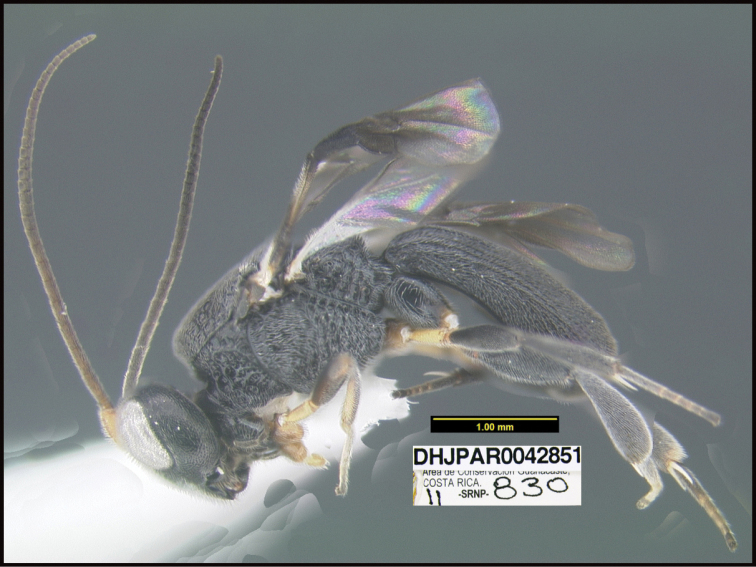
*Chelonuspaulgoldsteini*, holotype.

##### Chelonus paulhansoni

Taxon classificationAnimaliaHymenopteraBraconidae

Sharkey sp. nov.

http://zoobank.org/ECF20A71-B9F7-4A17-832D-0B8319D6011B

Figure 147

###### Diagnostics.

BOLD:ACB1254. Consensus barcode. TGTATTATATTTTATTTTTGGTATATGATGTGGGGTTTTGGGTTTGTCTATAAGGGTATTAATTCGTATAGAATTAAGAATATCTGGAAGATTATTGTTAAATGATCAATTATATAATAGAATTGTGACTTTACATGCTTTTATTATAATTTTTTTTATGGTTATACCAATTATGATTGGGGGATTTGGAAATTGATTAGTTCCTTTAATATTAGGGTTACCTGATATAGCATTTCCTCGAATAAATAATATAAGATATTGATTATTAATTCCATCTTTATTTTTGTTATTAATAAGTGGATTTATTAATGTGGGGGTAGGTACTGGGTGAACAGTTTATCCTCCATTATCTTTATTAATTGGACATGGAGGAATTTCAGTAGATATATCAATTTTTTCATTACATTTAGCTGGGGTTTCATCAATTATAGGTGCAATTAATTTTATTACTACAATTATAAATATATGATTAAAAATAAAATTTATAGATAAATTTCCTTTATTTGTATGATCAGTTTTGATTACTGCATTTTTATTATTATTATCTTTACCAGTTTTGGCAGGTGCTATTACTATATTATTAAGTGATCGTAATATAAATACAAGATTTTTTGATCCTTCAGGTGGGGGGGATCCAATTTTATATCAACATTTATTT.

###### Holotype ♂.

Alajuela, Sector Rincon Rain Forest, Quebrada Bambu, 10.93, -85.252, 109 meters, caterpillar collection date: 27/x/2012, wasp eclosion date: 14/xi/2012. Depository: CNC.

***Host data*.***Antaeotricha* Janzen204 (Depressariidae) feeding on *Miconiaxalapensis* (Melastomataceae).

***Caterpillar and holotype voucher codes*.** 12-SRNP-77452, DHJPAR0051343.

###### Paratypes.

Hosts = *Antaeotricha* Janzen204. DHJPAR0048978, DHJPAR0049326, DHJPAR0061491. Depository: CNC.

###### Etymology.

*Chelonuspaulhansoni* is named for Dr. Paul Hanson of the Universidad de Costa Rica in recognition of his decades of cheerful entomological and taxonomic interaction with the taxonomists and biodiversity of ACG and INBio.

**Figure 147.**
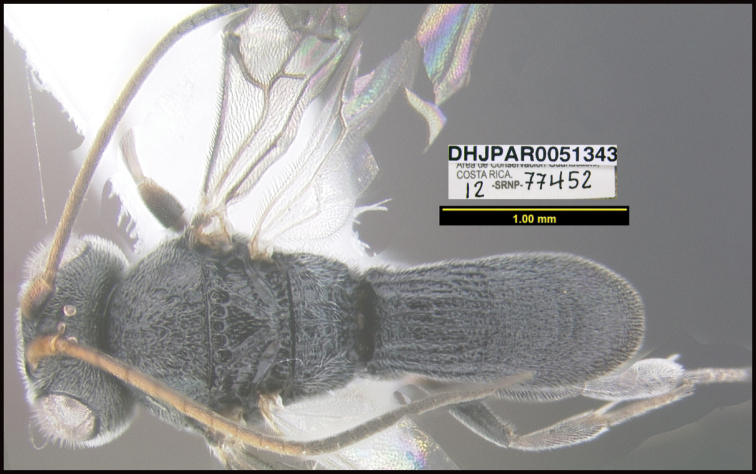
*Chelonuspaulhansoni*, holotype.

##### Chelonus paulheberti

Taxon classificationAnimaliaHymenopteraBraconidae

Sharkey sp. nov.

http://zoobank.org/08C6D536-1D5C-4AFE-884C-79CDD52A8766

Figure 148

###### Diagnostics.

BOLD:AAN2554. Consensus barcode. TATATTATATTTTATTTTTGGTATATGATGTGGTATATTGGGGTTGTCTTTAAGTATATTGATTCGTATAGAGTTAAGAATATCAGGTAGACTTTTATTAAATGATCAATTATATAATAGAATTGTAACTYTACATGCTTTTATTATAATTTTTTTTATAGTTATACCTGTAATAATTGGGGGATTTGGTAATTGATTAATTCCTTTAATATTAGGATTACCTGATATGGCTTTTCCTCGAATAAACAATATAAGTTAYTGATTATTAATTCCTTCATTATTTATACTAATAATAAGAGGATTTATTAATACAGGAGTTGGAACCGGTTGAACTGTTTATCCTCCATTATCATTATTAATTGGACATGGGGGGATTTCTGTAGATATATCAATTTTTTCTTTACATTTAGCAGGGGCTTCTTCAATTATGGGGGCAATTAATTTTATTACTACTATTATAAATATGTGATTAGTAAAAAGTTTTATGGATAAATATCCTTTATTTGTATGATCAGTTTTAATTACTGCAGTTTTGTTATTATTATCTCTTCCTGTGTTAGCAGGGGCTATTACTATATTATTAAGAGATCGTAATATAAATACTAGATTTTTTGATCCTTCAGGAGGGGGGGATCCTATTTTATATCAACATTTGTTT.

###### Holotype ♀.

Alajuela, Sector San Cristobal, Quebrada Garcia, 10.861, -85.426, 495 meters, caterpillar collection date: 27/ii/2011, wasp eclosion date: 22/iii/2011. Depository: CNC.

***Host data*.** elachBioLep01 BioLep754 (Depressariidae) feeding on *Tapirirabrenesii* (Anacardiaceae).

***Caterpillar and holotype voucher codes*.** 11-SRNP-869, DHJPAR0042850.

###### Paratypes.

Hosts = *Antaeotricha* Janzen233 (Depressariidae), elachBioLep01 BioLep754 (Depressariidae), elachBioLep01 BioLep55 (Depressariidae), *Stenoma* BioLep86 (Depressariidae). DHJPAR0039503, DHJPAR0042854, DHJPAR0042861, DHJPAR0042863, DHJPAR0048105, DHJPAR0048972. Depository: CNC.

###### Etymology.

*Chelonuspaulheberti* is named for Dr. Paul Hebert founder and major motor of the Centre for Biodiversity Genomics at the University of Guelph, Canada, and his invention of the DNA barcoding concept for identification of all species anywhere anytime by anyone.

**Figure 148.**
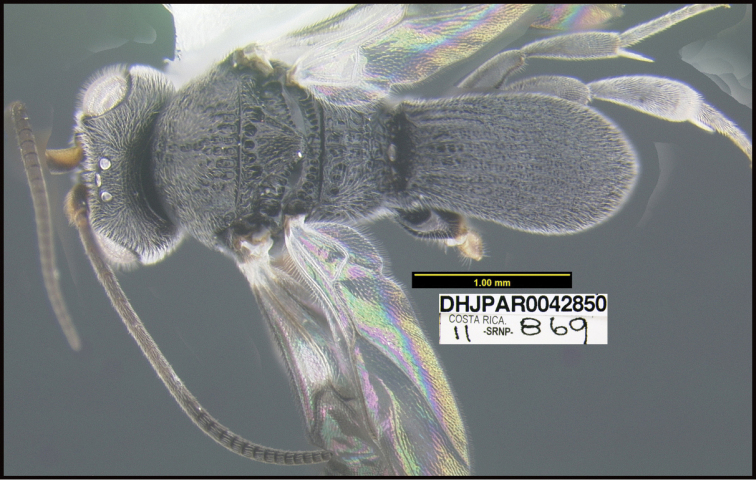
*Chelonuspaulheberti*, holotype.

##### Chelonus petronariosae

Taxon classificationAnimaliaHymenopteraBraconidae

Sharkey sp. nov.

http://zoobank.org/209DE698-C073-48B8-91EC-1D0BFF4ADA

Figure 149

###### Diagnostics.

BOLD:ACB1255. Consensus barcode. TATATTATATTTTATTTTTGGTATATGATGTGGAGTTTTAGGTTTATCTTTAAGTATATTAATTCGAATAGAATTAAGGGTATCTGGTAGATTATTATTAAATGATCAATTATATAATAGTATTGTAACCCTACATGCCTTTATTATAATTTTTTTTATAGTTATACCAGTAATAATTGGTGGATTTGGAAATTGATTAGTACCTTTAATATTAGGTTTACCTGATATAGCATTTCCTCGAATAAATAATATAAGATATTGATTATTAATTCCTTCATTATTTATATTATTAATGAGGGGATTTATTAATATGGGGGTAGGTACTGGTTGAACAGTTTATCCCCCTTTATCATTATTGATTGGTCATGGAGGTATTTCTGTAGATATATCAATTTTTTCATTACATTTAGCAGGGGCTTCTTCAATTATAGGAGCTATTAATTTTATTACTACTATTATAAATATATGAATAAATAGGAGATTTATAGATAAATATCCATTATTTGTATGATCAGTATTAATTACAGCGTTTTTATTATTATTATCTTTACCGGTATTAGCAGGAGCTATTACTATATTATTAAGGGATCGTAATATAAATACAAGATTTTTTGATCCTTCAGGTGGAGGTGACCCAATTTTATATCAACATTTATTT.

###### Holotype ♀.

Alajuela, Brasilia, Moga, 11.012, -85.349, 320 meters, caterpillar collection date: 13/iii/2012, wasp eclosion date: 04/iv/2012. Depository: CNC.

***Host data*.***Antaeotricha* Janzen146 (Depressariidae) feeding on *Lonchocarpusguatemalensis* (Fabaceae).

***Caterpillar and holotype voucher codes*.** 12-SRNP-65223, DHJPAR0048976.

###### Paratypes.

None.

###### Etymology.

*Chelonuspetronariosae* is named to honor Sra. Petrona Rios of GDFCF and ACG for her many years as a dedicated inventory parataxonomist for ACG.

**Figure 149.**
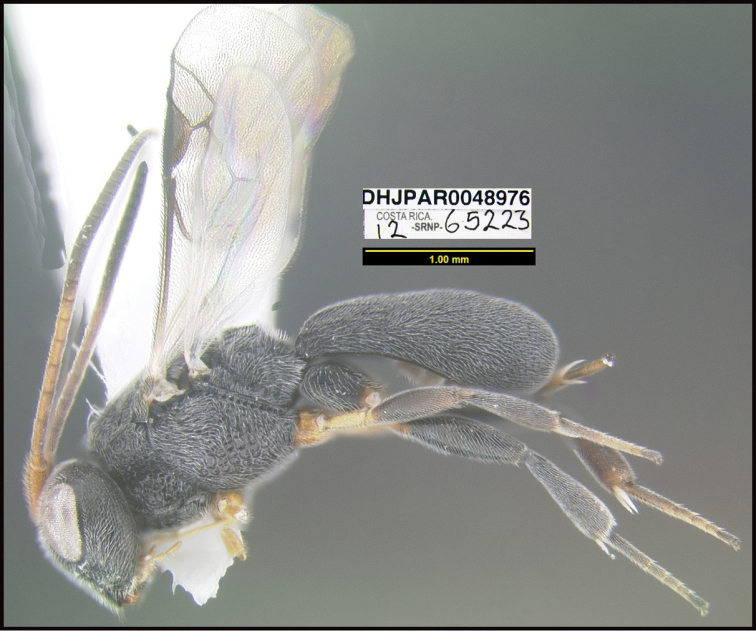
*Chelonuspetronariosae*, holotype.

##### Chelonus ramyamanjunathae

Taxon classificationAnimaliaHymenopteraBraconidae

Sharkey sp. nov.

http://zoobank.org/40C69855-B931-44B8-A203-57C850D16096

Figure 150

###### Diagnostics.

BOLD:ABA9309. Consensus barcode. AACATTATATTTTATTTTTGGTATATGATGTGGAGTTTTAGGTTTATCTTTAAGTATACTAATTCGAATAGAATTAAGTATATCTGGGAGATTATTATTAAATGATCAGTTATATAATAGTATTGTAACTTTACATGCTTTTATTATAATTTTTTTTATGGTTATGCCGGTAATAATTGGGGGATTTGGTAATTGATTAGTACCTTTAATATTAGGTTTACCTGATATAGCATTTCCTCGGATGAATAATATAAGATATTGGTTATTAATTCCTTCATTATTTATATTATTAATAAGTGGTTTTATTAATATAGGGGTGGGGACAGGTTGAACGGTTTATCCTCCTTTATCTTTGTTAATTGGTCATGGGGGTATTTCAGTAGATATATCAATTTTTTCATTACATTTAGCTGGGGCTTCTTCAATTATAGGTGCTATTAATTTTATTACTACAATTTTAAATATATGAATAAAAAGAAAATTTATGGATAAATTTCCTTTATTTGTTTGATCTGTATTAATTACTGCATTTTTACTTTTGTTATCATTACCTGTTTTGGCAGGGGCTATTACTATATTATTAAGTGATCGAAATATAAATACTAGATTTTTTGATCCTTCAGGGGGAGGTGATCCAATTTTATACCAACATTTATTT.

###### Holotype ♀.

Alajuela, Sector Rincon Rain Forest, Malaguenya, 10.956, -85.284, 221 meters, caterpillar collection date: 07/vii/2011, wasp eclosion date: 25/vii/2011. Depository: CNC.

***Host data*.***Antaeotricha* Janzen321 (Depressariidae) feeding on *Combretumfruticosum* (Combretaceae).

***Caterpillar and holotype voucher codes*.** 11-SRNP-67386, DHJPAR0045410.

###### Paratypes.

Hosts = *Antaeotricha* Janzen126, *Antaeotricha* Janzen146, *Antaeotricha* Janzen321. DHJPAR0040016, DHJPAR0045411, DHJPAR0045412, DHJPAR0045413, DHJPAR0048971, DHJPAR0048975, DHJPAR0055366, DHJPAR0055369. Depository: CNC.

###### Etymology.

*Chelonusramyamanjunathae* is named to honor Ms. Ramya Manjunatha for her many years of detailed, dedicated, gatekeeper and analytical role for all of the barcodes and BOLD updates into the Centre for Biodiversity Genomics.

**Figure 150.**
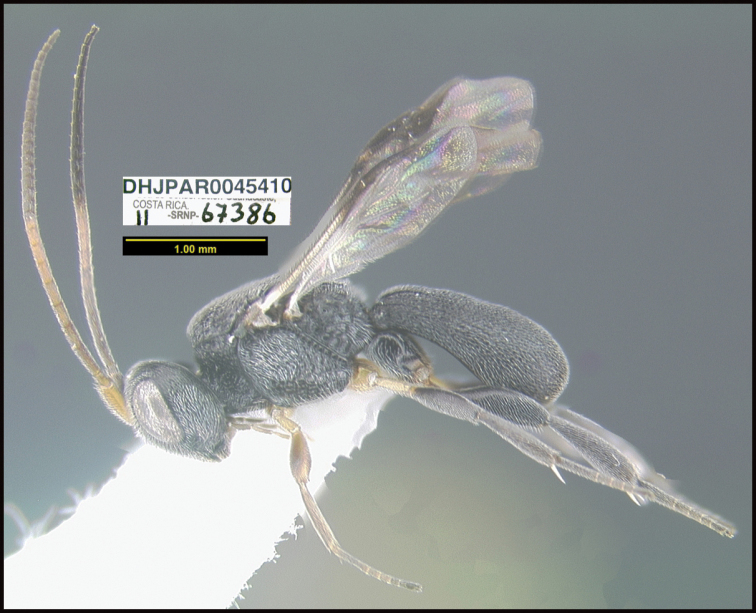
*Chelonusramyamanjunathae*, holotype.

##### Chelonus randallgarciai

Taxon classificationAnimaliaHymenopteraBraconidae

Sharkey sp. nov.

http://zoobank.org/88CF8C4D-5A67-47CB-B9E0-EAC703E9A7C7

Figure 151

###### Diagnostics.

BOLD:ADR1783. Consensus barcode. AACATTATATTTTATTTTTGGGATATGATGTGGAGTTTTAGGTTTATCTTTAAGTATACTAATTCGAATGGAATTAAGTGTATCTGGGAGGTTATTATTAAATGATCAGTTATATAATAGTATTGTAACTTTACATGCTTTTATTATAATTTTTTTTATGGTTATGCCGGTAATAATTGGGGGATTTGGTAATTGATTAGTACCTTTAATATTAGGTTTACCTGATATAGCATTTCCTCGTATGAATAATATAAGATATTGGTTATTAATTCCTTCATTATTTATATTATTAATAAGGGGTTTTATTAATATAGGGGTGGGGACAGGTTGAACGGTTTATCCCCCTTTATCTTTATTAATTGGTCATGGGGGTATTTCAGTAGATATATCAATTTTTTCATTACATTTAGCTGGGATTTCTTCAATTATAGGTGCTATTAATTTTATTACTACAATTTTAAATATATGAATAAAAAGAAAATTTATGGATAAATTTCCTTTATTTGTTTGGTCTGTATTAGTTACTGCATTTTTACTTTTATTATCATTACCTGTTTTGGCAGGGGCTATTACTATATTATTAAGTGACCGAAATATAAATACTAGATTTTTTGATCCTTCAGGGGGAGGTGACCCAATTTTATATCAGCATTTATTT.

###### Holotype ♀.

Guanacaste, Sector San Cristobal, Sendero Huerta, 10.9305, -85.3722, 527 meters, caterpillar collection date: 25/v/2018, wasp eclosion date: 14/vi/2018. Depository: CNC.

***Host data*.***Antaeotricha* Janzen146 (Depressariidae) feeding on *Lonchocarpusguatemalensis*.

***Caterpillar and holotype voucher codes*.** 18-SRNP-926, DHJPAR0062693.

###### Paratypes.

None.

###### Etymology.

*Chelonusrandallgarciai* is named to honor Sr. Randall Garcia for his decades of essential administrative roles in the founding of ACG and subsequent founding and ongoing life of INBio to the present day.

**Figure 151.**
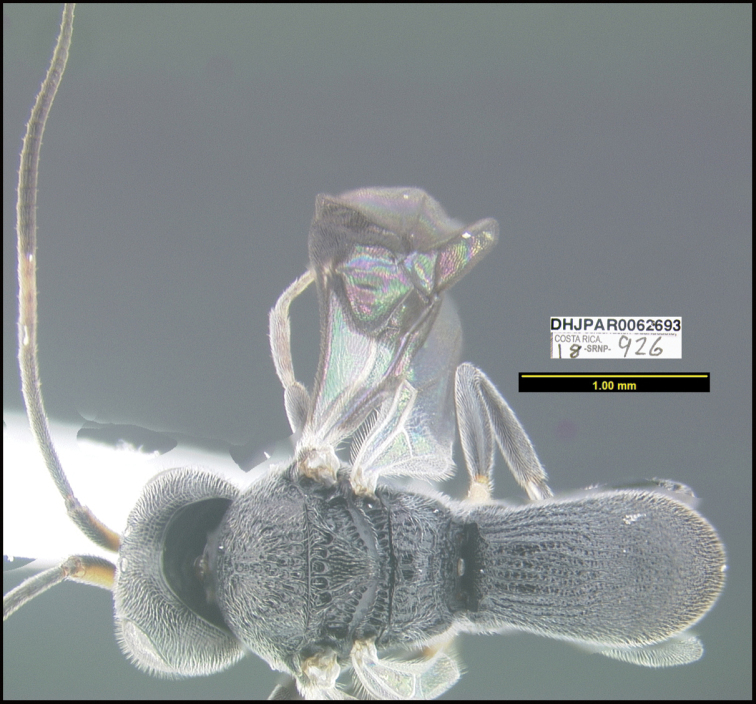
*Chelonusrandallgarciai*, holotype.

##### Chelonus rebeccakittelae

Taxon classificationAnimaliaHymenopteraBraconidae

Sharkey sp. nov.

http://zoobank.org/28D344D0-F5D2-4CBF-AB66-CD2929DD078E

Figure 152

###### Diagnostics.

BOLD:ACB1121. Consensus barcode. GTGGAGTTTTGGGATTATCTTTAAGGATATTAATTCGTATGGAGTTAAGAATATCTGGAAGGTTATTGTTGAATGATCAGTTATATAATAGAATCGTTACTTTACATGCTTTTATTATAATTTTTTTTATAGTTATGCCTGTAATAATTGGTGGGTTTGGTAATTGATTAATTCCTTTAATATTAGGGTTGCCTGATATAGCTTTTCCACGTATAAATAATATAAGTTATTGATTATTAATTCCTTCATTATTTATATTATTAATAAGGGGATTTATTAATATAGGAGTAGGAACTGGTTGAACGGTTTATCCTCCATTATCATTATTAATTGGACATGGAGGTATTTCAGTAGATATATCAATTTTTTCTTTACATTTGGCTGGGGCTTCTTCTATTATAGGGGCTATTAATTTTATTACTACAATTATAAATATATGAGTAATTAAAAGATTTATAGATAAATATCCTTTATTTGTATGATCAGTATTTATTACTGCATTTTTATTATTATTATCATTACCAGTATTGGCTGGGGCTATTACTATATTATTAAGAGATCGTAATATAAATACAAGATTTTTTGATCCTTCAGGGGGGGGGGA-------------------------.

###### Holotype ♀.

Guanacaste, Sector El Hacha, Estación Los Almendros, 11.032, -85.528, 290 meters, caterpillar collection date: 20/ii/2012, wasp eclosion date: 10/v/2012. Depository: CNC.

***Host data*.***Antaeotricha* Janzen224 (Depressariidae) feeding on *Microdesmiaarborea* (Chrysobalanaceae).

***Caterpillar and holotype voucher codes*.** 12-SRNP-20443, DHJPAR0048982.

###### Paratypes.

None.

###### Etymology.

*Chelonusrebeccakittelae* is named for Dr. Rebecca Kittel, visiting the Smithsonian Institution and contributing her efforts to identify Cheloninae braconids reared from the ACG biodiversity inventory in the 2019’s and before.

**Figure 152.**
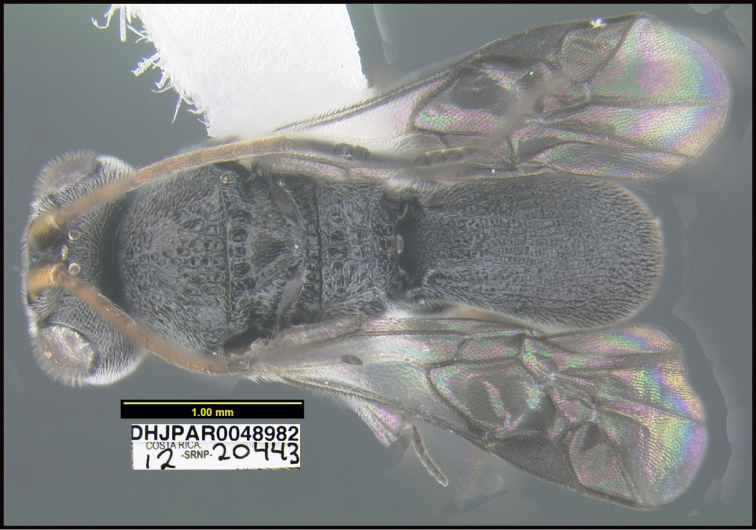
*Chelonusrebeccakittelae*, holotype.

##### Chelonus robertoespinozai

Taxon classificationAnimaliaHymenopteraBraconidae

Sharkey sp. nov.

http://zoobank.org/590136BB-61BE-4C97-88F9-F7BC978941B2

Figure 153

###### Diagnostics.

BOLD:AAD4678. Consensus barcode. GATATTATATTTTATTTTTGGCATATGGTGTGGAGTATTAGGATTATCTTTAAGTATATTAATTCGAATAGAATTAAGGATATCTGGTAGTTTATTAATAAATGATCAATTATATAATAGTATTGTAACTTTACATGCTTTTATTATAATTTTTTTTATAGTTATACCTGTTATGATTGGAGGATTTGGTAATTGATTAATTCCTTTAATATTAGGATTACCTGATATAGCTTTYCCTCGAATGAATAATATAAGTTATTGATTATTAATTCCTTCATTATTTTTATTATTAATAAGAGGATTTATTAATATAGGGGTTGGTACTGGATGAACAGTTTATCCTCCATTATCATTATTAATTGGGCATGGGGGAATTTCAGTAGATATATCAATTTTTTCTTTACATTTAGCTGGGGCATCTTCAATTATAGGTGCTATTAATTTTATTACTACAATTATAAATATATGGGTGGTTAGGAGGTTTATAGATAAATATCCTTTATTTGTGTGGTCGGTGTTAATTACTGCCTTTTTATTATTATTATCTTTACCTGTATTAGCAGGGGCTATTACTATATTATTAAGTGATCGTAATATGAATACAAGATTTTTTGATCCTTCAGGAGGGGGGGATCCAATTTTATATCAACATTTATTT.

###### Holotype ♀.

Alajuela, Brasilia, Moga, 11.012, -85.349, 320 meters, caterpillar collection date: 24/x/2011, wasp eclosion date: 18/xi/2011. Depository: CNC.

***Host data*.** elachJanzen01 Janzen211 (Depressariidae) feeding on *Miconiaargentea* (Melastomataceae).

***Caterpillar and holotype voucher codes*.** 11-SRNP-66100, DHJPAR0046939.

###### Paratypes.

Host = *Ategumialotanalis* (Crambidae): DHJPAR0029174, DHJPAR0029177. Depository: CNC.

###### Etymology.

*Chelonusrobertoespinozai* is named to honor Sr. Roberto Espinoza of GDFCF and ACG for his many years as a dedicated inventory parataxonomist for ACG.

**Figure 153.**
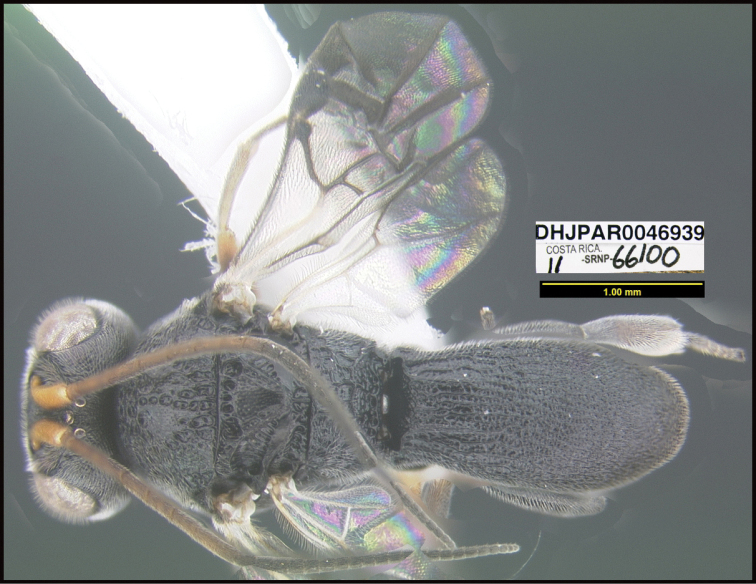
*Chelonusrobertoespinozai*, holotype.

##### Chelonus robertofernandezi

Taxon classificationAnimaliaHymenopteraBraconidae

Sharkey sp. nov.

http://zoobank.org/B90F0801-30A2-441C-998D-D05788ABF0C1

Figure 154

###### Diagnostics.

BOLD:ACJ5332. Consensus barcode. AATATTATATTTTATTTTTGGTATATGGTGTGGAGTTTTAGGGTTATCTTTGAGAATATTAATTCGGATAGAATTAAGTATATCTGGAAGTTTATTATTAAATGATCAATTATATAATAGTATCGTAACTTTACATGCTTTTATTATAATTTTTTTTATGGTTATACCTGTTATAATTGGTGGATTTGGTAATTGATTAATTCCTTTAATGTTAGGATTACCTGATATAGCTTTTCCTCGAATAAATAATATAAGTTATTGATTATTGATTCCTTCATTATTTATACTATTAATAAGGGGATTTGTTAATATAGGAGTTGGTACTGGTTGAACAGTTTATCCTCCTTTATCCTTATTAATTGGGCATGGAGGTATTTCAGTAGATATATCAATTTTTTCTTTACATTTAGCTGGTGCTTCATCAATTATAGGTGCTATTAATTTTATTACTACAGTTATAAATATATGGGTGGTTAGGAGATTTATAGATAAATATCCTTTATTTGTATGATCAGTATTAATTACTGCATTTTTATTATTATTATCATTACCTGTATTAGCGGGGGCTATTACTATGTTATTGAGTGATCGTAATATAAATACAAGATTTTTTGATCCCTCAGGGGGGGGAGACCCAATTTTATATCAACATTTATTT.

###### Holotype ♂.

Alajuela, Sector Rincon Rain Forest, Vado Rio Francia, 10.901, -85.289, 400 meters, caterpillar collection date: 26/vii/2013, wasp eclosion date: 13/viii/2013. Depository: CNC.

***Host data*.***Antaeotricha* Janzen110 (Depressariidae) feeding on *Ardisiacompressa* (Primulaceae).

***Caterpillar and holotype voucher codes*.** 13-SRNP-42816, DHJPAR0052883.

###### Paratypes.

None.

###### Etymology.

*Chelonusrobertofernandezi* is named to honor Sr. Roberto Fernandez of GDFCF and BioAlfa for his years of environmental planning for ICE followed by his current facilitation of the interaction between BioAlfa and ICE and other sectors of Costa Rican society while administrating the field aspects of the SINAC participation in BioAlfa Malaise-trapping of national parks.

**Figure 154.**
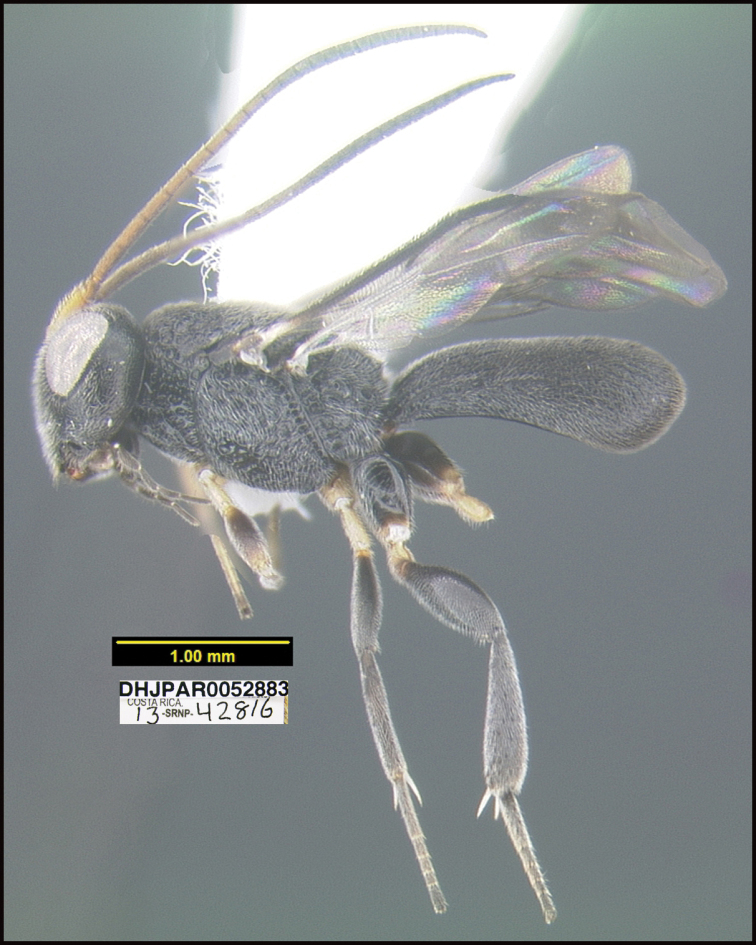
*Chelonusrobertofernandezi*, holotype.

##### Chelonus rocioecheverriae

Taxon classificationAnimaliaHymenopteraBraconidae

Sharkey sp. nov.

http://zoobank.org/E71E6775-0D50-4133-BC63-10EE45F80893

Figure 155

###### Diagnostics.

BOLD:ACB1122. Consensus barcode. AATATTATATTTTATTTTTGGAATATGGTGTGGAGTTTTAGGGTTATCTTTAAGTATTTTAATTCGAATAGAATTAAGTATAACTGGAAGATTATTTATAAATGATCAATTATATAATAGAATTGTAACTTTGCATGCTTTTATTATAATTTTTTTTATAGTTATACCCGTTATGATTGGTGGATTTGGTAATTGATTAATTCCTTTGATATTAGGGTTACCTGATATGGCATTTCCACGAATGAATAATATAAGTTATTGATTATTAATTCCTTCATTATTTATATTATTAATAAGAGGATTTATTAATATAGGAGTAGGTACAGGATGAACAGTTTATCCTCCTTTATCTTTATTAATTGGGCATGGTGGTATTTCCGTAGATATATCAATTTTTTCTTTACATTTAGCTGGGGCTTCTTCAATTATAGGTGCTATTAATTTTATTACTACGATTATAAATATATGAATAAATAAAAAGTTTATAGATAAATATCCTTTATTTGTGTGATCAGTATTAATTACTGCATTTTTATTATTATTATCTTTACCTGTATTAGCAGGAGCTATTACTATATTATTAAGAGATCGTAATATAAATACGAGATTTTTTGATCCTTCAGGAGGGGGTGATCCAATTTTATATCAACATTTATTT.

###### Holotype ♀.

Alajuela, Brasilia, Moga, 11.012, -85.349, 320 meters, caterpillar collection date: 13/iii/2012, wasp eclosion date: 01/iv/2012. Depository: CNC.

***Host data*.***Antaeotricha* Janzen146 (Depressariidae).

***Caterpillar and holotype voucher codes*.** 12-SRNP-65224, DHJPAR0048970.

###### Paratypes.

None.

###### Etymology.

*Chelonusrocioecheverriae* is named to honor Srta. Rocio Echeverri of Guanacaste and Proparques (NGO) for her lifelong enthusiasm for integrating Costa Rican conservation with its socio-economy.

**Figure 155.**
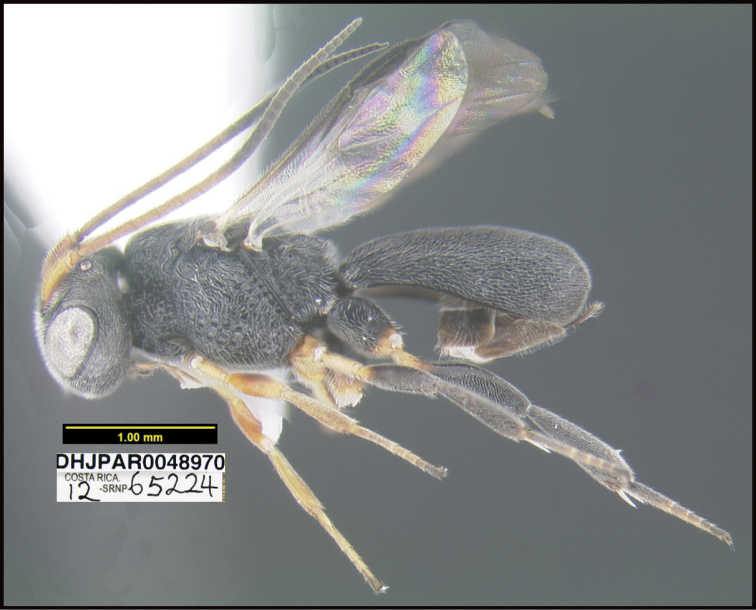
*Chelonusrocioecheverriae*, holotype.

##### Chelonus rodrigogamezi

Taxon classificationAnimaliaHymenopteraBraconidae

Sharkey sp. nov.

http://zoobank.org/EBEE6078-ABE5-478B-AA04-6A5554E75D26

Figure 156

###### Diagnostics.

BOLD:AAJ0362. Consensus barcode. AATATTATATTTTATTTTTGGGATATGATGTGGGGTTTTAGGRTTRTCTYTAAGTATACTAATTCGAATGGAATTAAGAATAACTGGGAGGTTATTTATAAATGATCAATTATATAATAGAATTGTAACTTTACATGCTTTTATTATAATTTTTTTTATGGTTATGCCTGTYATGATTGGYGGATTTGGTAATTGATTAATTCCYYTAATGTTGGGTTTACCTGATATAGCATTYCCTCGAATGAATAATATAAGTTATTGATTATTAATYCCYTCATTATTTATATTATTAATAAGGGGATTTATTAATATAGGTGTGGGYACTGGATGAACAGTTTATCCYCCTTTATCTTTATTAATTGGTCATGGGGGTATTTCAGTAGATATRTCAATTTTTTCTTTACATTTAGCTGGGGCTTCTTCAATTATAGGTGCTATTAATTTTATTACTACYATTATAAATATATGAATAAATAAGAAATTTATAGATAAATATCCTTTATTTGTATGATCTGTATTAATTACAGCATTTTTATTATTATTATCTTTRCCTGTATTGGCAGGGGCTATTACTATATTATTAAGTGATCGTAATATGAATACAAGRTTTTTTGATCCYTCAGGAGGAGGAGAYCCAATTTTATAYCAACAYTTWWTT.

###### Holotype ♂.

Alajuela, Sector Rincon Rain Forest, Sendero Juntas, 10.907, -85.288, 400 meters, caterpillar collection date: 03/vi/2010, wasp eclosion date: 19/vi/2010. Depository: CNC.

***Host data*.***Antaeotricha* Janzen88 (Depressariidae).

***Caterpillar and holotype voucher codes*.** 10-SRNP-41977, DHJPAR0040365.

###### Paratypes.

Hosts = *Antaeotricha* Janzen88. DHJPAR0029168, DHJPAR0040359, DHJPAR0040360, DHJPAR0040361, DHJPAR0040362, DHJPAR0040363, DHJPAR0040370, DHJPAR0042097, DHJPAR0046937, DHJPAR0048098, DHJPAR0048099, DHJPAR0050074, DHJPAR0050121, DHJPAR0051329, DHJPAR0051335, DHJPAR0051341, DHJPAR0052880, DHJPAR0052884, DHJPAR0052885, DHJPAR0052888, DHJPAR0052892, DHJPAR0052893, DHJPAR0052894, DHJPAR0052895, DHJPAR0053675, DHJPAR0053680, DHJPAR0054554, DHJPAR0054558, DHJPAR0054562, DHJPAR0054565, DHJPAR0055230, DHJPAR0055358, DHJPAR0055359, DHJPAR0055361, DHJPAR0055362, DHJPAR0055374, DHJPAR0055375, DHJPAR0055378, DHJPAR0055383, DHJPAR0055385, DHJPAR0055394, DHJPAR0055401, DHJPAR0055993, DHJPAR0055994, DHJPAR0055999, DHJPAR0056001, DHJPAR0056349, DHJPAR0056352, DHJPAR0056951, DHJPAR0056953, DHJPAR0056956, DHJPAR0056959, DHJPAR0056971, DHJPAR0062574, DHJPAR0062575.

###### Etymology.

*Chelonusrodrigogamezi* is named to honor Dr. Rodrigo Gámez for his founding role for ACG both logistically and philosophically, and then the same for INBio and its enormous impact on Costa Rica and the international world of conservation through national biodevelopment.

**Figure 156.**
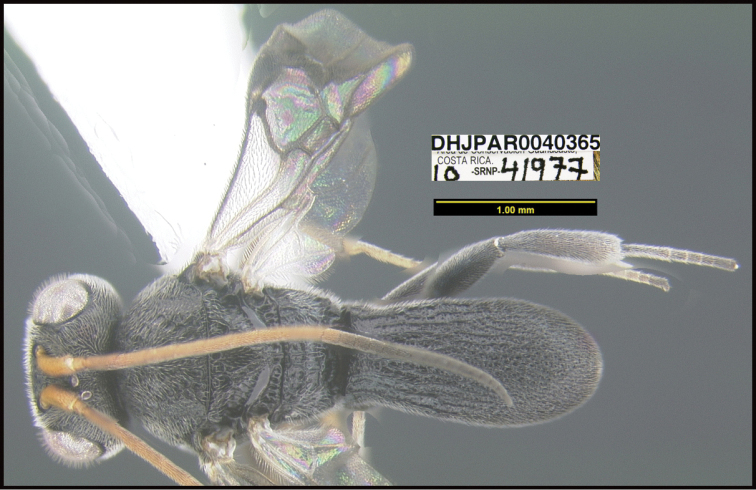
*Chelonusrodrigogamezi*, holotype.

##### Chelonus ronaldzunigai

Taxon classificationAnimaliaHymenopteraBraconidae

Sharkey sp. nov.

http://zoobank.org/1BBCE9DF-1873-44B2-9EA7-50E2E2C4CFFA

Figure 157

###### Diagnostics.

BOLD:AAK1016. Consensus barcode. AATACTATATTTTATTTTTGGAATATGATGTGGAGTTTTAGGATTATCATTAAGTATATTAATTCGAATAGAATTAAGAATAACTGGAAGATTATTTATAAATGATCAGTTATATAATAGTATTGTGACTTTACATGCTTTTATTATAATTTTTTTTATGGTTATACCTGTTATAATTGGTGGATTTGGTAATTGATTAATTCCTTTAATATTAGGATTACCTGATATAGCATTTCCTCGAATGAATAATATAAGTTATTGATTATTAATTCCTTCATTATTTATATTATTGATAAGGGGTTTTATTAATATAGGAGTTGGTACTGGATGAACAGTTTATCCCCCATTATCATTATTAATTGGGCATGGAGGTATTTCAGTGGATATATCAATTTTTTCTTTACATTTAGCGGGGGCTTCCTCAATTATAGGTGCTATTAATTTTATTACTACGATTATAAATATGTGGATAATTAAAAGATTTATAGATAAATATCCTTTATTTGTATGATCAGTATTAATTACTGCATTTTTATTATTATTATCTTTACCTGTATTGGCAGGGGCTATTACTATATTATTAAGTGATCGTAATATAAATACAAGATTTTTTGATCCTTCTGG----------------------------------.

###### Holotype ♀.

Alajuela, Sector San Cristobal, Puente Palma, 10.916, -85.379, 460 meters, caterpillar collection date: 16/ix/2009, wasp eclosion date: 07/x/2009. Depository: CNC.

***Host data*.***Antaeotricha* Janzen134 (Depressariidae) feeding on *Calophyllumbrasiliense* (Calophyllaceae).

***Caterpillar and holotype voucher codes*.** 09-SRNP-4814, DHJPAR0037167.

###### Paratypes.

None.

###### Etymology.

*Chelonusronaldzunigai* is named to honor Sr. Ronald Zuñiga for his decades of taxonomic curation and support of the Hymenoptera portion of the INBio and Museo Nacional insect collection, and now the same role for BioAlfa for Costa Rica in general and ACG specifically.

**Figure 157.**
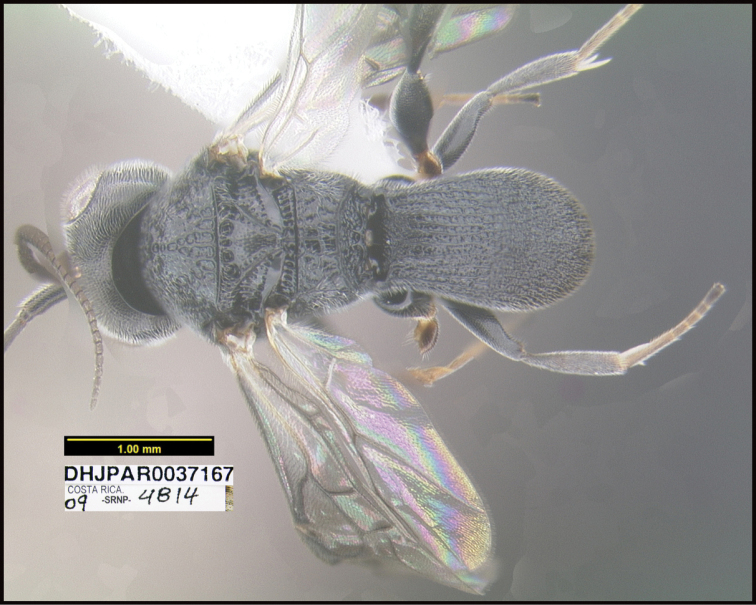
*Chelonusronaldzunigai*, holotype.

##### Chelonus rosibelelizondoae

Taxon classificationAnimaliaHymenopteraBraconidae

Sharkey sp. nov.

http://zoobank.org/88D2E819-0B1C-46B2-A961-6FFB5D551F60

Figure 158

###### Diagnostics.

BOLD:AAM1446. Consensus barcode. AATATTATATTTTATTTTTGGAATATGGTGTGGAGTTTTAGGATTATCATTAAGTATATTAATTCGAATAGAATTAAGAATAACTGGAAGATTATTTATAAATGATCAGTTATATAATAGGATTGTGACTTTACATGCTTTTATTATAATTTTTTTTATGGTTATACCTGTAATAATTGGTGGATTTGGTAATTGATTAATTCCTTTAATGTTAGGATTACATGATATAGCATTTCCTCGAATGAATAATATAAGTTATTGATTATTAATTCCTTCATTATTTATATTATTGATAAGGGGATTTATTAATATAGGGGTTGGTACTGGATGAACAGTTTATCCTCCATTGTCATTATTAATTGGGCATGGAGGTATTTCAGTGGATATATCAATTTTTTCTTTACATTTGGCGGGGGCTTCTTCAATTATAGGTGCCATTAATTTTATTACTACGATTATAAATATGTGGATAATTAAAAGATTTATAGATAAATATCCTTTATTTGTATGATCAGTATTAATTACTGCATTTTTATTATTATTATCTTTACCTGTATTGGCGGGGGCTATTACTATATTATTAAGTGATCGTAATATAAATACAAGATTTTTTGACCCTTCTGGTGGGGGGGATCCAATTTTGTATCAACATTTATTT.

###### Holotype ♀.

Alajuela, Sector San Cristobal, Puente Palma, 10.916, -85.379, 460 meters, caterpillar collection date: 17/x/2012, wasp eclosion date: 21/xi/2012. Depository: CNC.

***Host data*.***Antaeotricharenselariana* (Depressariidae) feeding on *Pterocarpusrohrii* (Fabaceae).

***Caterpillar and holotype voucher codes*.** 12-SRNP-4584, DHJPAR0051334.

###### Paratype.

Host = *Antaeotricha* Janzen146 (Depressariidae): DHJPAR0038004. Depository: CNC.

###### Etymology.

*Chelonusrosibelelizondoae* is named honor of Sra. Rosibel Elizondo for her decades of being an enthusiastic and dedicated biodiversity teacher of 4-5-6^th^ grade students living near ACG, through the ACG Programa de Educación Biológica.

**Figure 158.**
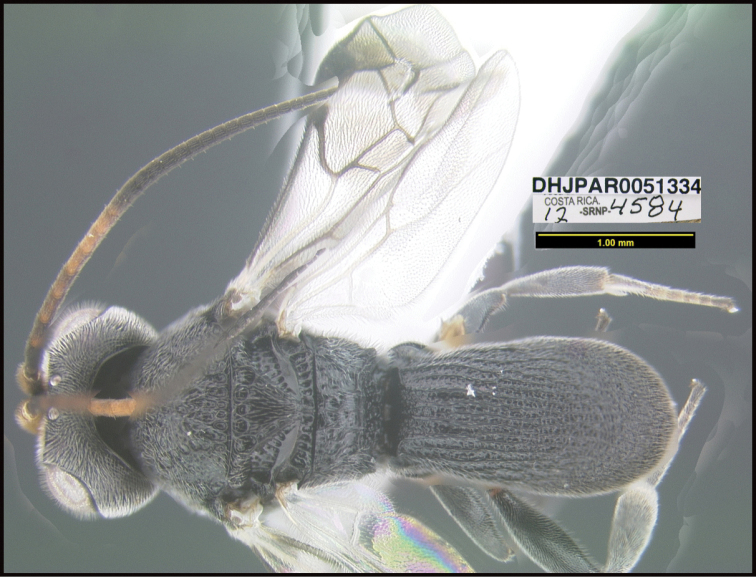
*Chelonusrosibelelizondoae*, holotype.

##### Chelonus rostermoragai

Taxon classificationAnimaliaHymenopteraBraconidae

Sharkey sp. nov.

http://zoobank.org/CE8C68A1-E60B-43AE-AE72-7655D7F9DB32

Figure 159

###### Diagnostics.

BOLD:ACJ3551. Consensus barcode. AATATTATATTTTATTTTTGGAATATGGTGTGGAGTTTTGGGATTATCATTAAGTATATTAATTCGAATAGAATTAAGAATAACYGGAAGATTATTTATAAATGATCAGTTGTATAATAGGATTGTRACTTTACATGCTTTTATCATAATTTTTTTTATGGTTATACCTGTTATAATTGGTGGATTTGGTAATTGATTAATTCCATTAATGTTAGGATTACCTGATATAGCATTTCCTCGAATGAATAATATAAGTTATTGATTATTAATTCCTTCATTATTTATATTATTGATAAGAGGATTTATYAATATAGGAGTTGGTACTGGATGAACAGTTTATCCTCCATTATCATTATTAATTGGACATGGGGGTATTTCGGTAGATATATCAATTTTTTCTTTGCATTTAGCGGGAGCTTCTTCAATTATAGGTGCCATTAATTTTATTACTACTATTATAAATATGTGGATAATTAAAAGATTTATAGATAAATATCCTTTATTTGTATGATCAGTATTAATTACTGCATTTTTAYTATTATTATCTTTACCGGTATTAGCAGGAGCTATTACTATATTATTAAGTGATCGTAATATAAATACAAGATTTTTTGATCCTTCTGGGGGGGGAGATCCAATTTTGTATCAACATCTATTT.

###### Holotype ♀.

Alajuela, Sector San Cristobal, Sendero Huerta, 10.93, -85.372, 527 meters, caterpillar collection date: 08/ii/2013, wasp eclosion date: 01/iii/2013. Depository: CNC.

***Host data*.***Antaeotricha* stigmatiasDHJ01 (Depressariidae) feeding on *Tetrapterystinifolia* (Malpighiaceae).

***Caterpillar and holotype voucher codes*.** 13-SRNP-616, DHJPAR0051922.

###### Paratypes.

Host = *Antaeotricha* stigmatiasDHJ01: DHJPAR0051925, DHJPAR0051926, DHJPAR0053673, DHJPAR0053685, DHJPAR0056000. Depository: CNC.

###### Etymology.

*Chelonusrostermoragai* is named to honor Sr. Roster Moraga of GDFCF and ACG for his many years as a dedicated inventory parataxonomist for ACG.

**Figure 159.**
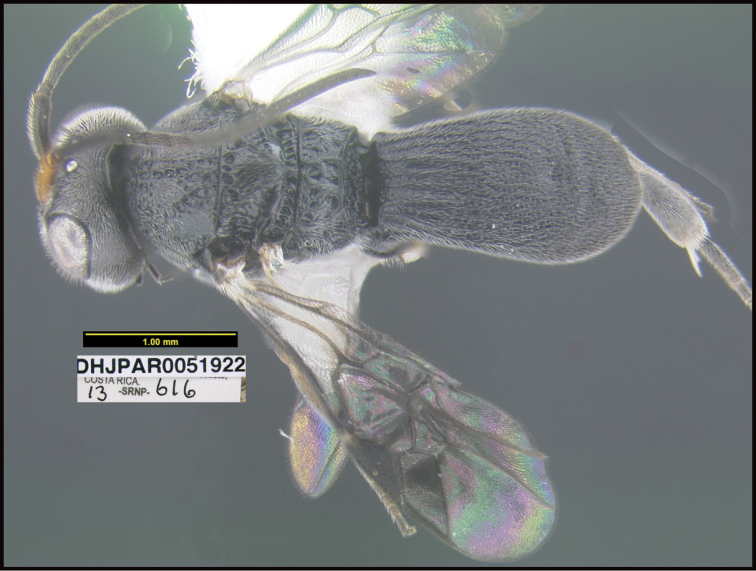
*Chelonusrostermoragai*, holotype.

##### Chelonus ruthfrancoae

Taxon classificationAnimaliaHymenopteraBraconidae

Sharkey sp. nov.

http://zoobank.org/659469DE-E73C-4C4E-BA35-11FD39BDE3B7

Figure 160

###### Diagnostics.

BOLD:AAW4589. Consensus barcode. AATATTATATTTTATTTTTGGRATATGGTGTGGRGTTTTAGGRTTATCTTTAAGTGTATTAATTCGAATAGAATTAAGAATAACTGGAAGGTTATTTATAAATGATCAGTTATATAATAGGATTGTAACTTTACATGCTTTTATTATAATTTTTTTTATGGTTATACCTGTAATGATTGGKGGGTTTGGTAATTGATTAATTCCCTTAATGTTAGGATTACCTGACATAGCATTTCCTCGTATGAATAATATAAGTTATTGATTAYTAATTCCTTCATTATTTATATTATTAATAAGGGGATTTATTAATATAGGAGTTGGGACTGGATGAACAGTTTATCCTCCATTATCATTATTAATTGGYCATGGRGGTATTTCAGTAGATATATCAATTTTTTCATTACATTTAGCYGGGGCTTCTTCAATTATAGGTGCTATTAATTTTATTACTACTATTATGAATATATGAATAATTAAGAGATTTATGGATAAATATCCTTTATTTGTATGATCAGTGTTAATTACTGCATTTTTGTTATTATTATCTTTACCTGTTTTGGCWGGAGCTATTACTATATTATTAAGTGATCGTAATATAAATACAAGATTTTTTGATCCCTCAGGAGGGGGGGATCCAATTTTATATCAACATTTATTT.

###### Holotype ♂.

Alajuela, Sector San Cristobal, Sendero Huerta, 10.93, -85.372, 527 meters, caterpillar collection date: 27/i/2013, wasp eclosion date: 27/i/2013. Depository: CNC.

***Host data*.***Antaeotricha* similisEPR02 (Depressariidae) feeding on *Ingaoerstediana* (Fabaceae).

***Caterpillar and holotype voucher codes*.** 12-SRNP-5796, DHJPAR0051094.

###### Paratypes.

Hosts = *Antaeotricha* BioLep46, *Antaeotricha* Janzen13, *Antaeotricha* Janzen290, *Antaeotricha* Janzen31, *Antaeotricha* similisEPR02, *Antaeotricha* similisEPR03, *Cerconota* Janzen82 (Depressariidae), *Stenoma* Janzen09 (Depressariidae). DHJPAR0042089, DHJPAR0042091, DHJPAR0050136, DHJPAR0051344, DHJPAR0055363, DHJPAR0055399, DHJPAR0056353, DHJPAR0056354, DHJPAR0056950, DHJPAR0056962.DHJPAR0056965. Depository: CNC.

###### Etymology.

*Chelonusruthfrancoae* is named to honor Srta. Ruth Franco of GDFCF and ACG for her many years as a dedicated inventory parataxonomist for ACG.

**Figure 160.**
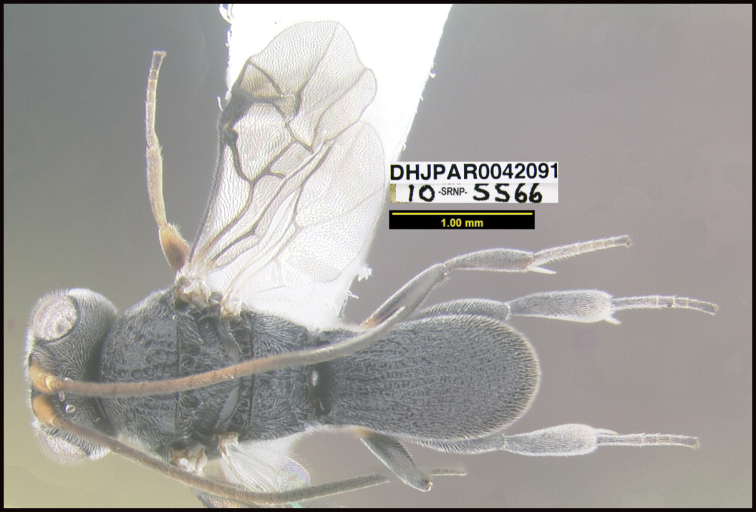
*Chelonusruthfrancoae*, holotype.

##### Chelonus scottmilleri

Taxon classificationAnimaliaHymenopteraBraconidae

Sharkey sp. nov.

http://zoobank.org/E8DB235A-5916-4FFE-B517-E37BAE4761A2

Figure 161

###### Diagnostics.

BOLD:ABY5286. Consensus barcode. AATATTATATTTTATTTTTGGAATATGGTGTGGAGTTTTAGGATTATCTTTAAGTATATTAATTCGAATAGAATTAAGAATAACTGGGAGGCTATTTATAAATGATCAGTTATATAATAGAATTGTGACCTTACATGCTTTTATTATAATTTTTTTTATGGTTATACCTGTAATGATTGGTGGATTTGGTAATTGATTAATTCCTTTAATGTTAGGATTACCTGATATAGCATTTCCTCGTATGAATAATATAAGTTATTGATTATTAATTCCTTCATTATTTATATTATTGATAAGGGGATTTATTAATATAGGAGTTGGAACTGGATGAACAGTTTATCCTCCATTATCATTATTAATTGGTCATGGGGGTATTTCTGTAGATATATCAATTTTTTCTTTACATTTAGCTGGTGCTTCTTCAATTATAGGTGCTATTAATTTTATTACTACAATTATAAATATATGAATAATTAAAAGGTTTATAGATAAATATCCTTTATTTGTATGATCAGTATTAATTACTGCATTTTTATTATTATTATCTTTACCTGTGTTGGCTGGGGCTATTACTATATTATTAAGTGATCGTAATATAAATACAAGATTTTTTGATCCCTCAGGAGGAGGGGATCCAATTTTATATCAGCATTTATTT.

###### Holotype ♂.

Guanacaste, Sector Pitilla, Estación Quica, 10.997, -85.397, 470 meters, caterpillar collection date: 22/vi/2010, wasp eclosion date: 09/vii/2010. Depository: CNC.

***Host data*.***Antaeotricha* radicalisEPR03 (Depressariidae) feeding on *Miconiatrinervia* (Melastomataceae).

***Caterpillar and holotype voucher codes*.** 10-SRNP-71898, DHJPAR0040369.

###### Paratypes.

Hosts = *Antaeotricha* Janzen04, *Antaeotricha* Janzen204, *Antaeotrichamarmorea*, *Antaeotricharadicalis*, *Antaeotricha* radicalisEPR02, *Antaeotricha* radicalisEPR03. DHJPAR0035310, DHJPAR0039122, DHJPAR0045285, DHJPAR0048095, DHJPAR0048096, DHJPAR0051325, DHJPAR0051339, DHJPAR0051345, DHJPAR0053670, DHJPAR0055384, DHJPAR0055388, DHJPAR0055389, DHJPAR0061492. Depository: CNC.

###### Etymology.

*Chelonusscottmilleri* is named to honor Dr. Scott Miller of the Smithsonian Institution for his decades of peripheral and direct support of the establishment of ACG and the permanent deposit of the ACG inventory voucher specimens, both DNA barcoded and not, as well as facilitating their collateral information integration with that of older specimens in the same Smithsonian collections.

**Figure 161.**
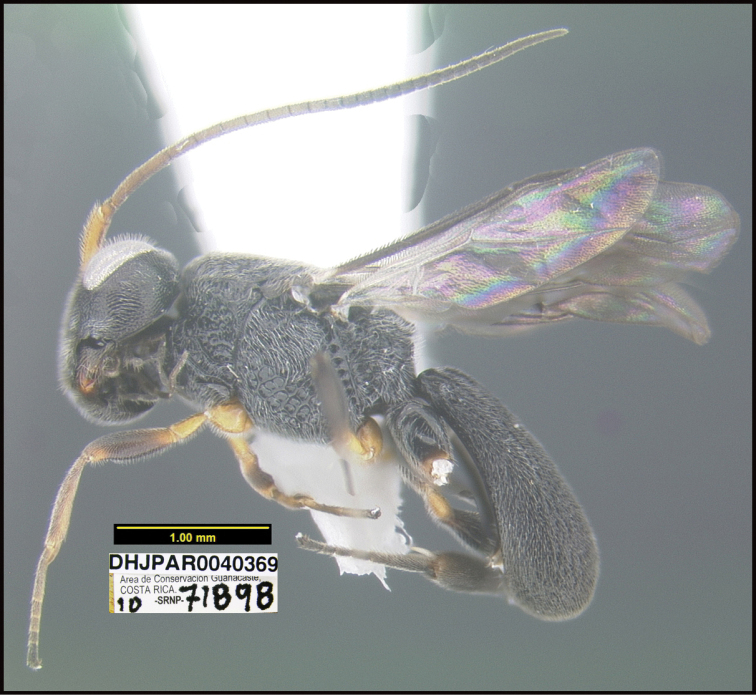
*Chelonusscottmilleri*, holotype.

##### Chelonus scottshawi

Taxon classificationAnimaliaHymenopteraBraconidae

Sharkey sp. nov.

http://zoobank.org/BBE86EA2-FF1B-4E61-96C2-D59C8D089E88

Figure 162

###### Diagnostics.

BOLD:ABX5499. Consensus barcode. AATATTATATTTTATTTTTGGAATATGATGTGGAGTTTTAGGRTTATCTTTAAGTATRTTAATTCGAATAGAATTAAGAATAACTGGAAGATTATTTATAAATGATCAGTTATATAATAGAATTGTRACTTTRCAYGCTTTTATTATAATTTTTTTTATGGTTATACCTGTAATGATTGGTGGRTTTGGTAATTGATTRATTCCTTTAATGYTAGGATTACCTGATATAGCATTYCCTCGTATGAATAATATAAGTTAYTGATTATTAATYCCTTCATTATTTATATTATTGATAAGGGGRTTTATTAATATAGGAGTTGGAACTGGATGAACAGTTTATCCTCCATTGTCATTATTAATTGGTCATGGRGGTATTTCTGTAGATATATCAATTTTTTCTTTACATTTAGCTGGTGCTTCTTCAATTATAGGTGCTATTAATTTTATTACTACAATTATAAATATATGAATAATTAAGAGATTTATAGATAAATAYCCTTTATTTGTATGATCAGTATTAATTACTGCAYTTTTGTTATTATTATCTTTACCTGTGTTGGCTGGGGCTATTACCATATTATTAAGTGAYCGTAATATAAATACAAGATTTTTTGAYCCCTCAGGAGGAGGGGAYCCAATTTTATATCAACATTTATTT.

###### Holotype ♂.

Alajuela, Sector Rincon Rain Forest, Quebrada Guarumo, 10.904, -85.284, 400 meters, caterpillar collection date: 3/iii/2011, wasp eclosion date: 30/iii/2011. Depository: CNC.

***Host data*.***Antaeotricha* Janzen146 (Depressariidae) feeding on *Lonchocarpusoliganthus* (Fabaceae).

***Caterpillar and holotype voucher codes*.** 11-SRNP-41083, DHJPAR0042853.

###### Paratypes.

Hosts = *Antaeotricha* Janzen146, *Antaeotricha* Janzen24, *Antaeotricha* Janzen245, *Antaeotricha* Janzen40, *Antaeotricha* Janzen49, *Antaeotricharenselariana*. DHJPAR0036381, DHJPAR0036382, DHJPAR0036383, DHJPAR0036384, DHJPAR0036386, DHJPAR0036387, DHJPAR0036389, DHJPAR0036393, DHJPAR0036395, DHJPAR0036403, DHJPAR0037988, DHJPAR0037992, DHJPAR0037995, DHJPAR0039505, DHJPAR0048979, DHJPAR0048981, DHJPAR0048992, DHJPAR0049007, DHJPAR0049018, DHJPAR0051924, DHJPAR0052182, DHJPAR0052183, DHJPAR0052185, DHJPAR0052186, DHJPAR0052187, DHJPAR0052280, DHJPAR0054573, DHJPAR0055360, DHJPAR0055365, DHJPAR0055372, DHJPAR0055377, DHJPAR0055379, DHJPAR0055380, DHJPAR0055386, DHJPAR0055387, DHJPAR0055391, DHJPAR0055392, DHJPAR0055393, DHJPAR0055395, DHJPAR0055396, DHJPAR0055991, DHJPAR0056955, DHJPAR0062573, DHJPAR0062697. Depository: CNC.

###### Etymology.

*Chelonusscottshawi* is named to honor Dr. Scott Shaw of the University of Wyoming for his enthusiastic taxonomizing of ACGBraconidae in the ACG biodiversity inventory.

**Figure 162.**
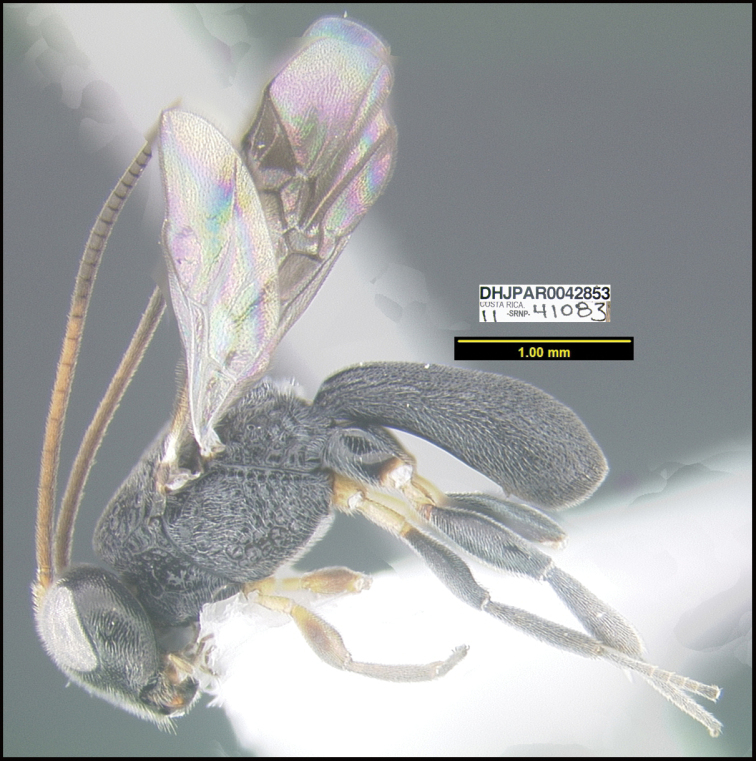
*Chelonusscottshawi*, holotype.

##### Chelonus sergioriosi

Taxon classificationAnimaliaHymenopteraBraconidae

Sharkey sp. nov.

http://zoobank.org/FE0A159A-0A25-49A5-A2C4-218F1E30A36C

Figure 163

###### Diagnostics.

BOLD:ACA6835. Consensus barcode. AATATTATATTTTATTTTTGGTATATGATCTGGTATATTTGGTTTATCATTAAGTTTATTAATTCGTATAGAATTGAGAATTTCTGGAACTTTATTTGGTAATGATCAATTATATAATAGTATTGTAACTTTRCATGCATTTATTATAATTTTTTTTATAGTTATRCCAGTTATAATTGGTGGTTTTGGAAATTGATTAGTTCCATTAATATTAGGATTACCAGATATAGCTTTTCCTCGTATAAATAATATAAGGTTTTGATTATTAATACCTTCATTATTTATATTAATTATAAGAGGATTTGTAAATATAGGAGTAGGAACTGGTTGAACTGTTTATCCTCCRTTATCTTTATTAATAGGACATGGTGGAGTTTCGGTAGATATATCAATTTTTTCTTTACATTTAGCTGGTATATCTTCAATTATAGGAGCTATTAATTTTATTGTTACTATTATGAATATATGAATAAGAATAGTTTTTTTTGATAAATATCCTTTATTTGTATGATCAGTATTAATTACTGCGGTATTATTATTATTATCATTACCAGTTTTAGCTGGTGCTATTACTATATTATTAAGAGATCGAAACTTAAATACAAGATTTTTTGATCCTTCAGGKGGWGGTGAYCCAGTATTATATCAACATTTATTT. Distribution restricted to Central America.

###### Holotype ♂.

Guanacaste, Sector Santa Rosa, Bosque San Emilio, 10.8438, -85.6138, 300 meters, 25/ii‒11/iii/2013, Malaise trap. Depository: CNC.

***Host data*.** None.

***Holotype voucher code*.**BIOUG10752-A09.

###### Paratypes.

None.

###### Etymology.

*Chelonussergioriosi* is named to honor Sr. Sergio Rios of GDFCF and ACG for his many years as a dedicated inventory parataxonomist for ACG.

###### Note.

A specimen from Collier County, Florida, United States (BOLD code BIOUG02575-C01) shares the same BIN, but the color of the hind leg is dramatically different making it unlikely to be conspecific. The hind femur and tibia of the Florida specimen are pale yellow, whereas the tibia of *C.sergioriosi* is brown. More specimens from the two localities will need to be barcoded and examined to confirm the consistency of this difference.

**Figure 163.**
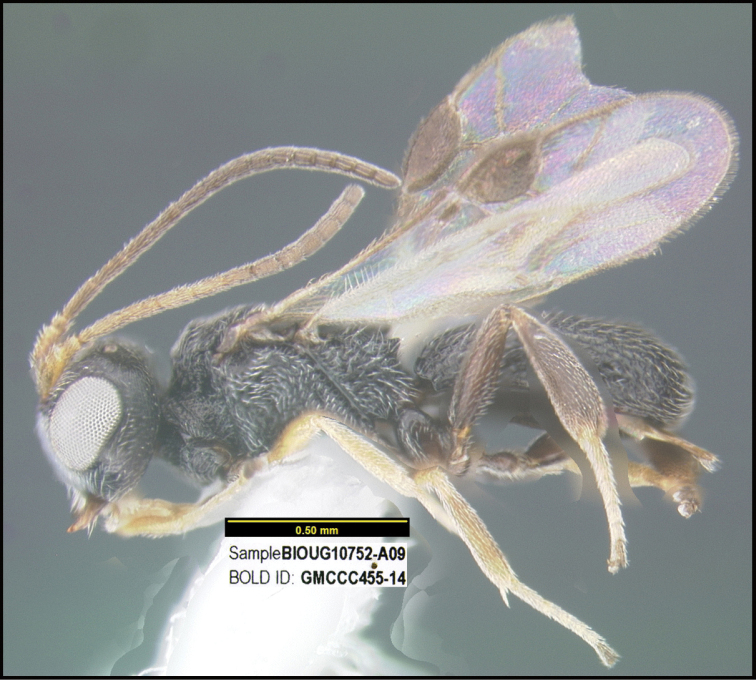
*Chelonussergioriosi*, holotype.

##### Chelonus sigifredomarini

Taxon classificationAnimaliaHymenopteraBraconidae

Sharkey sp. nov.

http://zoobank.org/C10EA736-7D53-4FFB-A8E7-B233EDC2DFA0

Figure 164

###### Diagnostics.

BOLD:AAM1445. Consensus barcode. AATTTTATATTTTATTTTTGGTATTTGAAGAGGTATATTTGGTTTATCTTTAAGAATAATAATTCGTTTAGAATTATCATTTACTGGTAATTTAATAATAAATGATCAATTATATAATAGTATTGTTACTATACATGCTTTTATTATGATTTTTTTTTTAGTTATACCAGTTATAATTGGTGGTTTTGGTAATTGATTAATTCCATTAATATTAGGTTTATGTGATATATGTTTTCCTCGAATGAATAATATAAGATTTTGATTATTAATACCTTCWTTATTTATATTAATTATAAGAGGATTTGTAAATACAGGTGTAGGTACAGGTTGGACTTTGTATCCTCCATTATCYTTATTAATAGGTCATAGGGGAATTTCAGTAGATATATCTATTTTTTCTTTACATTTAGCTGGAATATCTTCAATTATAGGAGCTATTAATTTTATTGTAACAATTGTTAAYACTTGGATAATAAAAGTTTATATAGATAAAATTTCATTATTTGTTTGGTCAGTTTTAATTACTGCTGTATTATTATTATTATCTTTACCTGTTTTAGCAGGAGGTATTACTATATTATTAAGTGATCGGAATTTTAATACAACATTTTTTGATTCTTCAGGAGGTGGTGATCCTATTTTGTATCAACATTTATTT.

***Host data*.***Euacidaliacertissa* (Geometridae) feeding on *Leucaenamulticapitula* (Fabaceae).

###### Holotype ♂.

Alajuela, Sector Rincon Rain Forest, Estación Llanura, 10.933, -85.253, 135 meters, caterpillar collection date: 28/xi/2009, wasp eclosion date: 19/xii/2009. Depository: CNC.

***Host data*.** None.

***Caterpillar and holotype voucher codes*.** 09-SRNP-76655, DHJPAR0037978.

###### Partypes.

None.

###### Etymology.

*Chelonussigifredomarini* is named to honor Sr. Sigifredo Marin for many decades of intense conservation administration of ACG itself followed by the same intelligent care and development for GDFCF field projects and the members of the ACG Parataxonomist Program.

**Figure 164.**
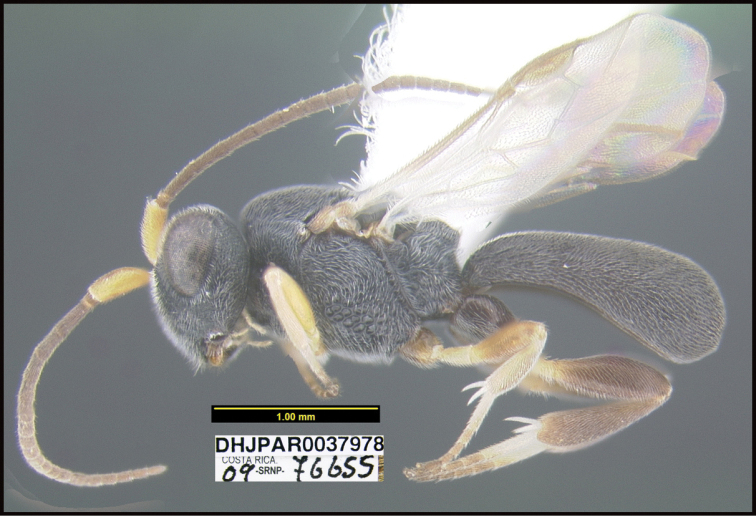
*Chelonussigifredomarini*, holotype.

##### Chelonus stevearonsoni

Taxon classificationAnimaliaHymenopteraBraconidae

Sharkey sp. nov.

http://zoobank.org/6D1B0ABD-898E-4647-9C8E-4921B93C2BCE

Figure 165

###### Diagnostics.

BOLD:AAW3558. Consensus barcode. TGTTTTATATTTTATTATAGGTATATGATCTGGTGTATTTGGATTGTCATTAAGTATACTTATTCGTTTAGAATTATCTTTTCCTGGAAGTTTAATAATAAATGATCAATTATATAATAGTTTAATTACTATACATGCTTTTATTATAATTTTTTTTATAGTTATACCAGTTATAATTGGTGGTTTTGGTAATTGATTAGTTCCATTAATATTAGGTTTATGTGATATATGTTTTCCTCGAATAAATAATATAAGATTTTGATTATTGGTTCCTTCTTTAATTTTATTAATTTCAAGTTCTTTTATTAATACTGGGGTTGGTACTGGATGAACTGTTTATCCTCCATTATCTTTATTAATAGGTCATAGAGGTATTTCAGTAGATTTATCTATTTTTTCTCTTCATTTGGCTGGTATATCATCAATTATAGGAGCAATTAATTTTATTGTAACTGTAATTAATACTTGAATATTAAAAAATTATATAGATAAATTATCTTTATTTGTATGATCTGTAATAATTACAGCTGTATTATTATTATTATCTTTACCAGTATTAGCTGGTGCTATTACTATATTATTAAGAGATCGAAATATAAATACAACATTTTTTGATTCTTCAGGTGGYGGAGATCCTGTATTGTATCAACATTTATTT.

###### Holotype ♀.

Guanacaste, Sector Pitilla, Gazo, 10.98, -85.386, 485 meters, caterpillar collection date: 18/iii/2014, wasp eclosion date: 06/iv/2014. Depository: CNC.

***Host data*.***Leucirisfimbriaria* (Geometridae) feeding on *Mimosawatsonii* (Fabaceae).

***Caterpillar and holotype voucher codes*.** 14-SRNP-70585, DHJPAR0055364.

###### Paratypes.

Host = *Leucirisfimbriaria*: DHJPAR0016436, DHJPAR0029120, DHJPAR0042849. Depository: CNC.

###### Etymology.

*Chelonusstevearonsoni* is named to honor Mr. Steve Aronson of Britt Coffee and NGO Proparques for his essential support to ACG internet connectivity and subsequent development of forest walkways for handicapped visitors.

**Figure 165.**
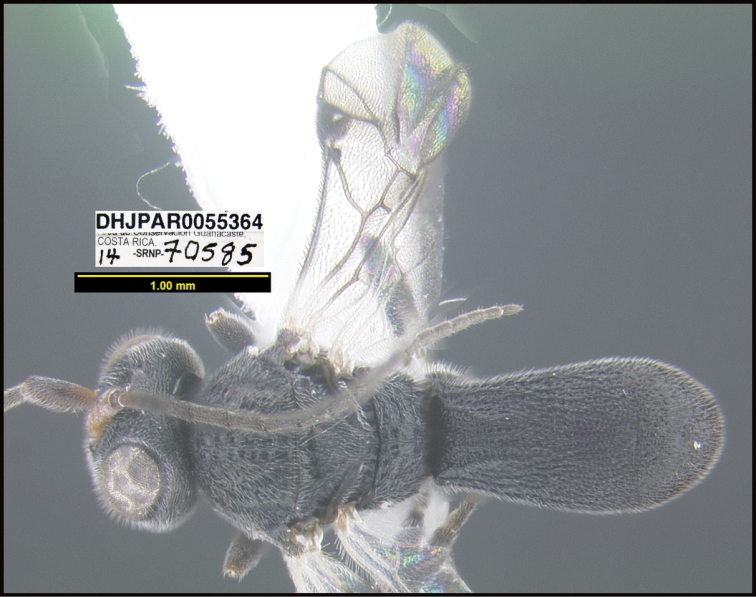
*Chelonusstevearonsoni*, holotype.

##### Chelonus stevestroudi

Taxon classificationAnimaliaHymenopteraBraconidae

Sharkey sp. nov.

http://zoobank.org/CD59760D-5EFD-4654-8E07-022040344CA4

Figure 166

###### Diagnostics.

BOLD:AAW3557. Consensus barcode. TATTTTATATTTTATTTTTGGCATATGATGTGGTATATTCGGATTATCTTTAAGTATATTAATTCGTATAGAATTAGGTACTTTAAAAAGATTATTTATAAATGATCAGTTATATAAYGGRTTAGTTACTATACATGCATTTATTATAATTTTTTTTATAGTTATACCAATTATAATTGGTGGATTTGGTAATTGATTAGTTCCATTAATATTAGGATTACCTGATATATCTTTTCCTCGTATAAATAATATAAGTTATTGATTATTGATTCCTTCATTATTTATATTAATTATAAGAAGTTTTGTAAATATGGGGGTTGGTACAGGTTGAACTGTRTATCCACCATTATCTTTATTAATGGGACATAGGGGAGTTTCTGTAGATATAGCTATTTTTTCTTTACATTTAGCGGGRGCTTCATCTATTATAGGTGCAATTAATTTTATTATTACAAGGTTAAATACTTATATAAAATTAAGTTATATAGAAAAATTTCCTTTATTTGTTTGATCAGTATTAATTACTGCAGTATTATTATTATTATCTTTACCAGTATTAGCGGGGGCTATTACAATATTGTTAAGAGATCGAAATATAAATACTAGATTTTTTGATCCAGCAGGAGGAGGGGATCCATTATTGTATCAACATTTATTT.

###### Holotype ♂.

Alajuela, Sector San Cristobal, Sendero Huerta, 10.93, -85.372, 527 meters, caterpillar collection date: 03/viii/2014, wasp eclosion date: 23/viii/2014. Depository: CNC.

***Host data*.***Antaeotricha* Janzen13 (Depressariidae) feeding on *Ingaoerstediana* (Fabaceae).

***Caterpillar and holotype voucher codes*.** 14-SRNP-3582, DHJPAR0056351.

###### Paratypes.

Host = *Antaeotricha* BioLep46, *Antaeotricha* Janzen290, *Antaeotricha* similisEPR01: DHJPAR0020803, DHJPAR0056002, DHJPAR0056004. Depository: CNC.

###### Etymology.

*Chelonusstevestroudi* is named to honor Mr. Steve Stroud of Escazu, Costa Rica for his decades of support, both financial and psychological, for all things ACG and the subsequent spin-out of BioAlfa, and its exploration of private ecotourism at Hacienda Baru.

**Figure 166.**
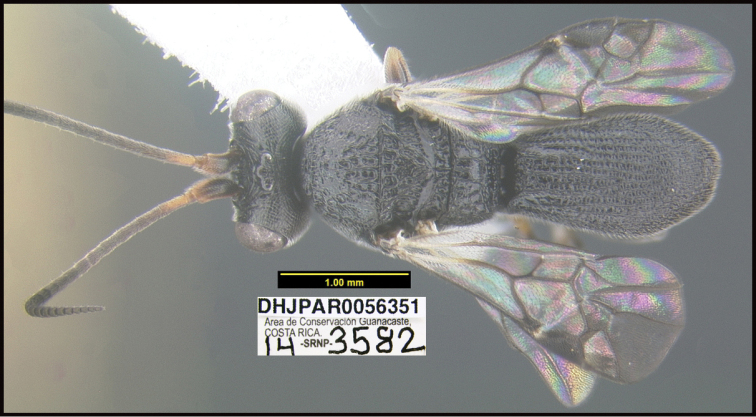
*Chelonusstevestroudi*, holotype.

##### Chelonus sujeevanratnasinghami

Taxon classificationAnimaliaHymenopteraBraconidae

Sharkey sp. nov.

http://zoobank.org/BC52D59F-4D5E-41DB-B559-B6F335CD6CF8

Figure 167

###### Diagnostics.

BOLD:ABW9828. Consensus barcode. TGTATTATATTTTATTTTTGGGATATGGTCTGGTATATTAGGTTTATCTTTAAGTATATTAATTCGTATAGAATTAGTATATCCTGGTAGATTATTAATAAATGATCAATTATATAATAGATTAGTTACTATACATGCATTTATTATAATTTTTTTTATAGTGATACCTATTATAATTGGTGGATTTGGAAATTGATTAATTCCTTTAATGTTAGGATTACCTGATATAATTTTTCCTCGTATAAATAATATAAGATTTTGATTATTAATTCCTTCTTTAATAATATTAATTTTAAGAATATTAGTTAATATGGGGGTAGGGACAGGATGAACAGTTTATCCTCCTTTATCTTCATTAATAGGTCATAGGGGAGTAAGTGTAGACATAGCTATTTTTTCTTTGCATTTAGCTGGGATATCATCAATTATAGGAGCTATTAATTTTATAGTAACTGTGTTAAATAGATGGATACATTATAGTTATATAGATGTAATACCTTTATTTGTTTGATCAGTTTTTATTACGGCAATTTTATTATTATTATCATTACCTGTATTAGCCGGTGCTATTACTATATTATTAAGAGATCGTAATATAAATACTACATTTTTTGATCCTACGGGAGGGGGAGACCCAATTTTATATCAACATTTATTT.

###### Holotype ♂.

Alajuela, Brasilia, Gallinazo, 11.018, -85.372, 360 meters, caterpillar collection date: 09/i/2012, wasp eclosion date: 24/i/2012. Depository: CNC.

***Host data*.***Rhobonda* gaurisanaDHJ04 (Choreutidae) feeding on *Ficusdonnell-smithii* (Moraceae).

***Caterpillar and holotype voucher codes*.** 12-SRNP-65012, DHJPAR0046942.

###### Paratypes.

Host = *Rhobonda* gaurisanaDHJ04: DHJPAR0046943, DHJPAR0046944, DHJPAR0046945, DHJPAR0046946, DHJPAR0047222, DHJPAR0047223, DHJPAR0047229, DHJPAR0047230. Depository: CNC.

###### Etymology.

*Chelonussujeevanratnasinghami* is named to honor Dr. Sujeevan Ratnasingham of the Centre for Biodiversity Genomics at the University of Guelph for designing and executing the intellectual and logistic guts of BOLD at the CBG, without which CBG would have no purpose.

**Figure 167.**
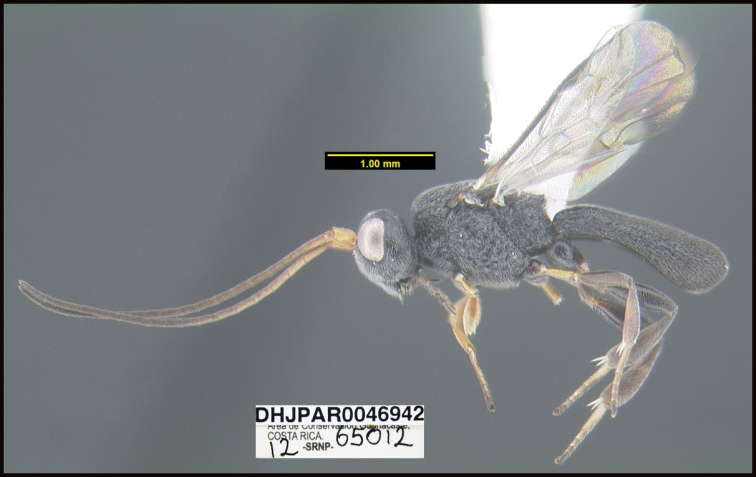
*Chelonussujeevanratnasinghami*, holotype.

##### Chelonus sureshnaiki

Taxon classificationAnimaliaHymenopteraBraconidae

Sharkey sp. nov.

http://zoobank.org/566B6104-153F-429C-80ED-529F05E812EE

Figure 168

###### Diagnostics.

BOLD:AAW3556. Consensus barcode. TATATTATATTTTATTTTTGGTATATGAAGAGGTATACTAGGTTTATCTTTAAGTTTATTAATTCGTATAGAATTAAGATTTAGGGGAAGTTTATTTCAAAATGATCAATTATATAATAGAATTGTAACTATACATGCTTTTGTAATAATTTTTTTTATAGTTATACCAATTATAGTAGGGGGTTTTGGAAATTGATTGATTCCTTTAATATTAGGTATACCTGATATAATATTTCCTCGAATAAATAATATAAGATTTTGATTATTAATTCCTTCATTGTTAATATTAATTTTTGGGGGATTTATTAATATAGGTGTAGGTACTGGATGAACATTATATCCTCCATTATCATTATTAATGAGACATGGAGGAATGTCAGTAGATATTTCAATTTTTTCGTTACATTTAGCTGGTATATCTTCAATTATAGGATCAATTAATTTTATTGTAACTATTATAATAGTATGAATTGAAAAAATATATATAGATAAATTTTCTTTATTTTTATGATCTATTTTAATTACTACAATTTTATTGTTATTATCTTTACCTGTATTGGCTGGTGCTATTACAATATTATTAAGAGATCGTAATATAAATACTAGATTTTTTGATCCCGCTGGGGGCGGGGATCCAGTATTGTATCAACATTTATTT.

###### Holotype ♀.

Guanacaste, Sector Pitilla, Medrano, 11.016, -85.381, 380 meters, caterpillar collection date: 05/ix/2014, wasp eclosion date: 27/ix/2014. Depository: CNC.

***Host data*.***Microscahedialis* (Thyrididae) feeding on *Alchornealatifolia* (Euphorbiaceae).

***Caterpillar and holotype voucher codes*.** 14-SRNP-71641, DHJPAR0056350.

###### Paratype.

Host = *Rhodoneuraterminalis* (Thyrididae): DHJPAR0029162. Depository: CNC.

###### Etymology.

*Chelonussureshnaiki* is named to honor Dr. Suresh Naik for his sterling role in the sequencing and information management roles of the Centre for Biodiversity Genomics of the University of Guelph.

**Figure 168.**
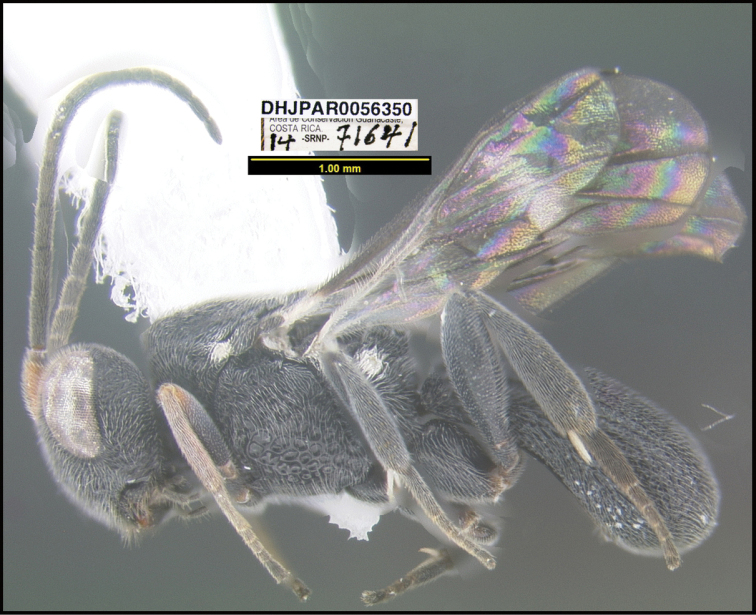
*Chelonussureshnaiki*, holotype.

##### Chelonus torbjornekremi

Taxon classificationAnimaliaHymenopteraBraconidae

Sharkey sp. nov.

http://zoobank.org/8DC2985C-3A9C-4D28-92B3-FDAFDFC598BE

Figure 169

###### Diagnostics.

BOLD:ACZ0402. Consensus barcode. AATATTATATTTTTTATTTGGAATTTGAAGTGGAGTTATAGGTTTGTCTTTAAGTATAATAATTCGTATAGAATTGAGAAGTGTAATAAGATTATTTTATAATGATCAGTTATATAATAGAATTGTTACTATACATGCTTTTATTATAATTTTTTTTATAGTAATACCTTTAATAATTGGGGGATTTGGAAATTGATTAATTCCATTGATGTTAGGATTGTCTGATATAATTTTTCCTCGAATAAATAATATAAGATTTTGGTTATTAATTCCTTCAATTATTTTATTAATTATAGGGGGATTTGTAAATATAGGGTCTGGAACAGGGTGAACAATTTATCCTCCATTATCACTATTAATAGGCCATAGGGGAGTTTCAGTAGATTTATCTATTTTTTCTTTACACTTAGCAGGTATATCATCTATTATAGGTTCAATTAATTTTATTGTTACTATTATAAATACTTGATTACACATTAAATATATAGATAAATATCCTTTATTTGTTTGATCTGTATTTATTACAGTTATTTTATTATTAATATCATTACCAGTTTTAGCTGGGGCAATTACTATATTATTAAGTGATCGAAATTTAAATACAAGATTTTTTGATCCATCAGGAGGGGGAGATCCGGTATTGTATCAACATTTATTT.

###### Holotype ♂.

Alajuela, Sector Rincon Rain Forest, Cafecito, 10.94404, -85.31738, 455 meters, caterpillar collection date: 10/x/2014, wasp eclosion date: 26/x/2014. Depository: CNC.

***Host data*.** pyrausJanzen01 Janzen26 (Crambidae) feeding on *Baccharistrinervis* (Asteraceae).

***Caterpillar and holotype voucher codes*.** 14-SRNP-81300, DHJPAR0056964.

###### Paratypes.

Hosts = pyrausJanzen01 Janzen26, *Aponia* Janzen469 (Crambidae). DHJPAR0045404, DHJPAR0060594, DHJPAR0061493. Depository: CNC.

###### Etymology.

*Chelonustorbjornekremi* is named to honor Dr. Torbjorn Ekrem of the NTNU University of Museum in Norway for his eager and high quality administrative and science participation in the iBOL international meetings in South Africa and in Trondheim, Norway, and especially in support of BioAlfa and the ACG-ICE project to biomonitor the Pailas II geothermal site.

**Figure 169.**
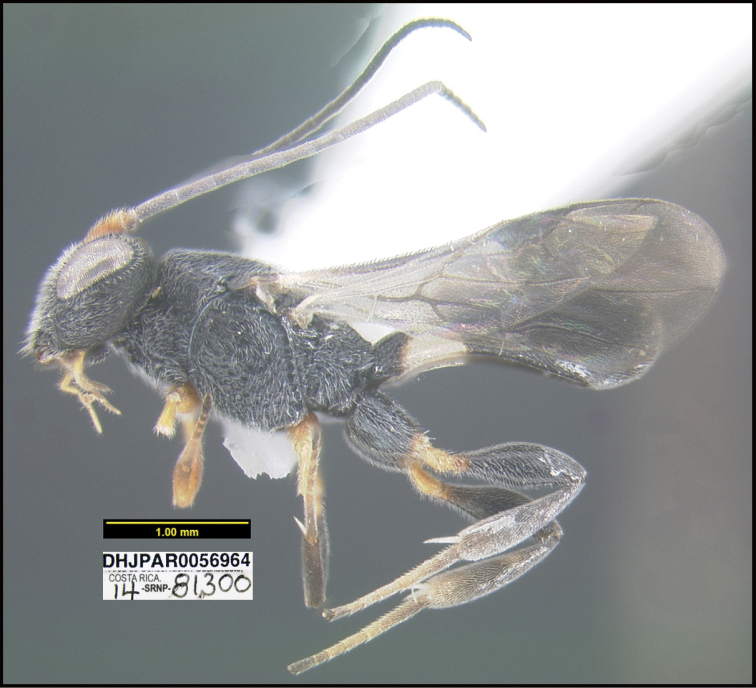
*Chelonustorbjornekremi*, holotype.

##### Chelonus yeimycedenoae

Taxon classificationAnimaliaHymenopteraBraconidae

Sharkey sp. nov.

http://zoobank.org/F174E937-8073-4E3D-A9E3-B0EE0D15EC93

Figure 170

###### Diagnostics.

BOLD:ADW6358. Consensus barcode. TATTATATTTCATATTTGGTATATGATCTGGTATATTTGGATTATCATTAAGTTTATTAATTCGAATAGAATTAAGAGTATCGGGTAGTTTATTTATAAATGATCAATTATATAATAGAATTGTTACTTTACATGCATTTATTATAATTTTTTTTATAGTTATGCCAATTATAATTGGTGGATTTGGAAATTGATTAATTCCTTTAATATTGGGTTTACCTGATATATTATTTCCACGAATAAATAATATAAGTTTTTGATTATTAATACCTTCATTAATTATATTAATATTGAGTAGATTTGTAAATATAGGAGTAGGTACAGGTTGAACTATTTATCCTCCATTATCTTTATTAATTGGTCATGGTGGAATTTCAGTGGATATATCAATTTTTTCTTTACATTTAGCTGGAATATCTTCAATTATAGGTGCTATTAATTTTATAGTTACAATTATTAATACATGAATAAGTAATAATTATATAGATAAATTTTCTTTATTTGTTTGATCAGTTTTTATTACTGCAATTTTATTATTATTATCTTTACCTGTTTTAGCTGGTGCTATTACTATATTATTAAGAGATCGAAATATAAATACAAGATTTTTTGATCCTTCAGGTGGGGGTGATCCAATTTTATATCAGCATTTAT------------------------------------------.

###### Holotype ♂.

Guanacaste, Pailas Dos, PL12-3, 10.7631, -85.3344, 820 meters, 05‒18/xii/2014, Malaise trap PL12-3A. Depository: CNC.

***Host data*.** None.

***Holotype voucher code*.**BIOUG44134-G02.

###### Paratypes.

None.

###### Etymology.

*Chelonusyeimycedenoae* is named to honor Srta. Yeimy Cedeño of SINAC in recognition of her energetic high-quality representation of Costa Rica and BioAlfa in the CBD meeting in Egypt in 2018.

**Figure 170.**
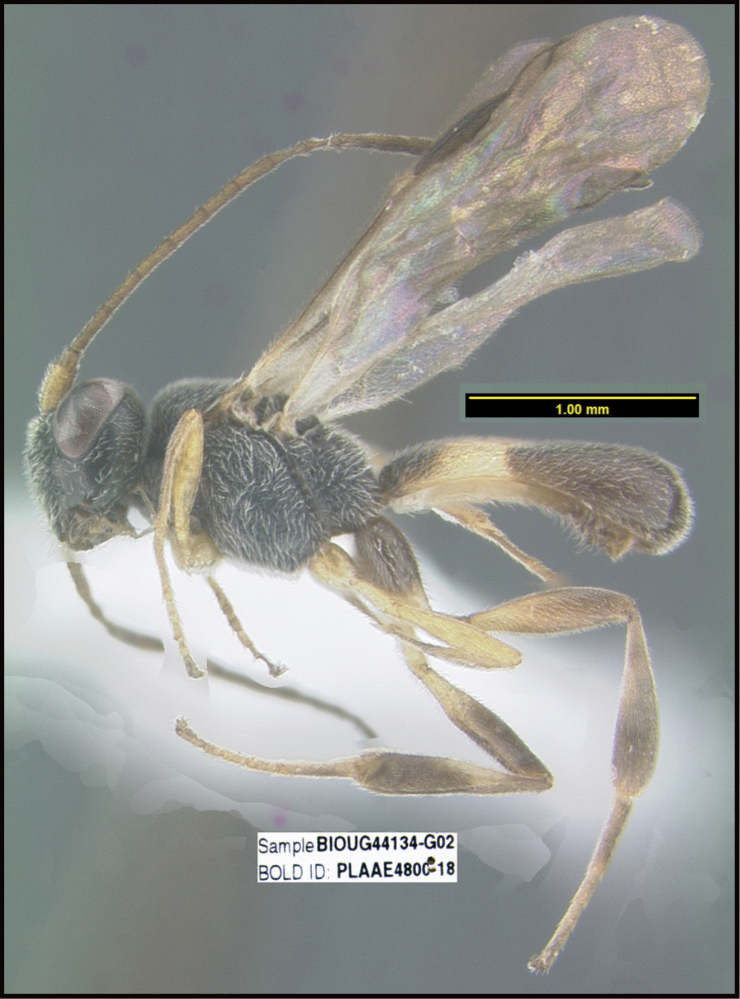
*Chelonusyeimycedenoae*, holotype.

#### *Leptodrepana* Shaw, 1983

*Leptodrepana* species are found throughout the New World. Dadelahi and Shaw (2018) described the first 24 Costa Rican species of the genus. Only one of these, *L.alexisae*, was captured in our Malaise traps and none was reared. Its identification was based on morphological inspection rather than its barcode. Hosts are unknown for the genus, and we do not treat any reared species here. This strongly suggests that the hosts are leaf-miners since these were not included in the Janzen and Hallwachs rearing program.

##### Leptodrepana alexisae

Taxon classificationAnimaliaHymenopteraBraconidae

Dadelahi & Shaw, 2018

Figure 171

###### Diagnostics.

BOLD:ABV9918. Consensus barcode. GGTATTTTATATTTTATTTTTGGTATATGATCTGGCATATTTGGTTTATCATTAAGTTTAATAATTCGAATAGAGTTAAGTTCTTTAACATCTTTTATAGGTAATGATCAAATTTATAATAGAATTGTTACTATACATGCATTTATTATAATTTTTTTTATGGTTATACCAATTATAATTGGTGGTTTTGGAAATTGATTAATTCCATTAATATTAGGGGGTCCAGATATAGCATTTCCTCGTATAAATAATATAAGATTTTGACTATTAATTCCATCATTATTAATATTAATTATGAGAAGGTTAGTAAATGTTGGTGTTGGGACAGGATGAACAGTTTATCCTCCTTTATCTTTATTAATAGGTCATGGGGGAATTTCTGTAGATTTAAGAATTTTTTCTTTACATTTAGCGGGAGCTTCATCAATTATAGGTGCAATTAATTTTATTGTTACAATTATGAATATATGATTAGGTATAAAATATATAGATAAAATTTCTTTATTTAGATGATCAGTTTTAATTACTGCTATTTTATTATTATTATCATTACCTGTTTTAGCTGGGGCAATTACAATATTATTAACAGATCGAAATTTAAATACAAGATTTTTTGATCCGGCAGGAGGTGGGGATCCAATTTTGTATCAACATTTATTT.

###### Imaged specimen.

♀: Guanacaste Province, Sector Cacao, Sendero Arenales, 10.92471, -85.46738, 1080 meters, University of Wyoming Insect Museum, Laramie, Wyoming.

***Host data*.** None.

***Voucher code of imaged specimen conspecific with the holotype*.**BIOUG33274-F09.

###### Other material.

All Malaise-trapped: DHJPAR0045916, DHJPAR0045947, DHJPAR0046006, DHJPAR0046011, DHJPAR0046014, DHJPAR0046015, DHJPAR0046029, DHJPAR0047295, DHJPAR0047294, DHJPAR0047288, DHJPAR0047301, DHJPAR0047309, DHJPAR0047318, DHJPAR0047319, DHJPAR0047323, DHJPAR0047325, DHJPAR0047327, DHJPAR0047332, BIOUG36488-A02, BIOUG36495-H04.

**Figure 172.**
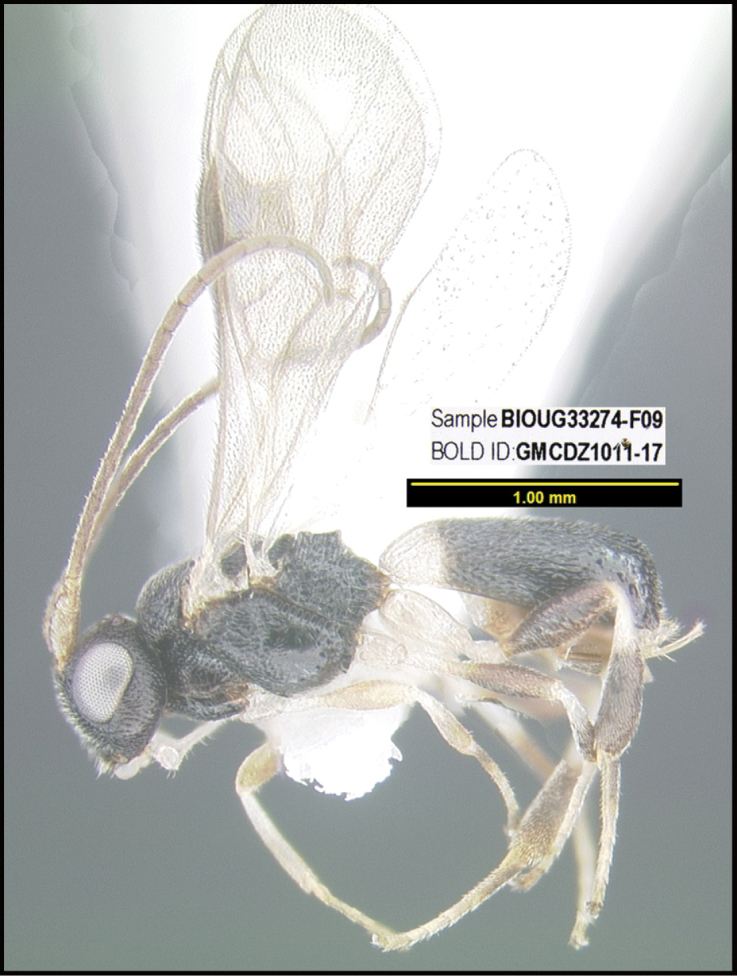
*Leptodrepanaerasmocoronadoi*, holotype **A** image on BOLD**B** mounted image, head missing.

##### Leptodrepana erasmocoronadoi

Taxon classificationAnimaliaHymenopteraBraconidae

Sharkey sp. nov.

http://zoobank.org/3ADDCFC4-0A04-4DF4-8E2B-8F6A74E039A3

Figure 172

###### Diagnostics.

BOLD:ADA3192. Consensus barcode. TTTATTTTTGGAATATGATCAGGAATATTTGGATTATCATTAAGATTATTAATTCGTATAGAATTAAGTTCTATCACAACTTATATTGGAAATGATCAGATTTATAATAGAATTGTTACTATACATGCTTTTATTATAATTTTTTTTATAGTTATACCTGTAATAATTGGGGGATTTGGAAATTGATTAGTACCTTTAATAATAGGTGGTCCTGACATATCTTTCCCTCGTATAAATAATATAAGATTTTGATTATTATTACCTTCATTAATTTTATTAATTATAAGGAGTTTAGTAAATGTAGGTGTTGGTACTGGTTGAACAGTTTATCCTCCTTTATCTTTATTAATGGGTCATGGAGGTATTTCAGTAGATTTAAGAATTTTTTCTTTACATTTAGCTGGAGCTTCTTCAATTATAGGAGCTATTAATTTTATTGTAACAATTTTAAATATATGATTTAGTAAAAAATATATAGATAAGATTTCTTTATTTAGGTGATCAGTATTTATTACAGCAATTTTATTATTATTATCATTACCAGTATTAGCTGGAGCAATTACAATATTATTAACAGATCGAAATTTAAATACA------------------------------------.

###### Holotype ♀.

Alajuela, Sector San Cristobal, Estación San Gerardo, 10.8801, -85.389, 575 meters, 24‒31/iii/2014, Malaise trap. Depository: CNC.

***Host data*.** None.

***Holotype voucher code*.**BIOUG28344-E11.

###### Paratypes.

None.

###### Etymology.

*Leptodrepanaerasmocoronadoi* is named to honor Sr. Erasmo Coronado of Liberia, Costa Rica for his years of high-quality service in the GDFCF field administration of the GDFCF field projects.

**Figure 171.**
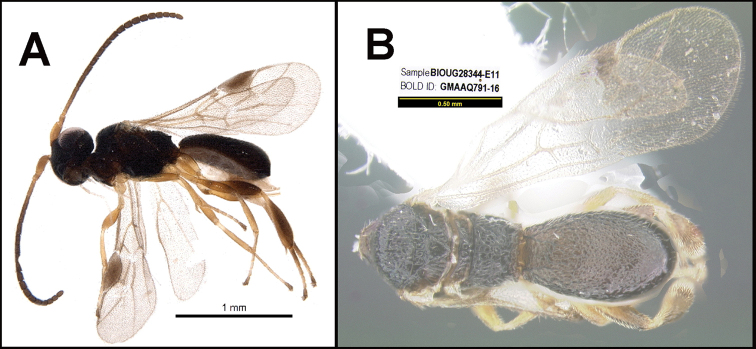
*Leptodrepanaalexisae*.

##### Leptodrepana felipechavarriai

Taxon classificationAnimaliaHymenopteraBraconidae

Sharkey sp. nov.

http://zoobank.org/DF89B051-E22C-4919-972D-0E6A1AFCAB42

Figure 173

###### Diagnostics.

BOLD:ADB5898. Consensus barcode. ATTTTATATTTTATTTTTGGTTTATGATCAGGAATATTTGGGTTGTCATTGAGGTTAATGATTCGAATAGAATTAAGTTCAGTGGGTACTTATATAGGTAATGATCAAATTTATAATAGGATTGTAACAATACATGCATTTATCATAATTTTTTTTATAGTTATACCAGTTATAATTGGTGGATTTGGAAATTGATTGATTCCTTTGATGTTGGGGGGGCCTGATATATCTTTTCCTCGAATGAATAATATAAGATTTTGGTTGTTGGTACCATCATTATTTATATTAATTTTAAGAAGTTTAGTAAATACTGGGGTAGGGACTGGGTGAACAGTTTATCCTCCTTTATCTTTATTAATAGGTCATGGAGGAATTTCAGTTGATTTAAGAATTTTTTCATTACATTTAGCGGGGGCATCATCTATTATAGGGGCTATTAATTTTATTGTAACTATTATAAATATATGGTTAGGGTTTAAATTTTTAGATAAAATTTCTTTATTTAGGTGATCAGTGTTAATTACTGCAATTTTATTATTATTATCTTTACCAGTTTTAGCTGGAGCAATTACAATGTTATTGACA------------------------------------------.

###### Holotype ♂.

Guanacaste, Pailas Dos, PL12-3, 10.7631, -85.3344, 820 meters, 03‒10/iv/2014, Malaise trap PL12-3A. Depository: CNC.

***Host data*.** None.

***Holotype voucher code*.**BIOUG29764-B11.

###### Paratypes.

None.

###### Etymology.

*Leptodrepanafelipechavarriai* is named to honor Sr. Felipe Chavarria of Santa Rosa, ACG, for his decades of detail accurate handling of GDFCF accounting for GDFCF field projects, taking very high-quality images of ACG biodiversity, and being a core role in ACG and INBio Biodiversity Prospecting projects.

**Figure 173.**
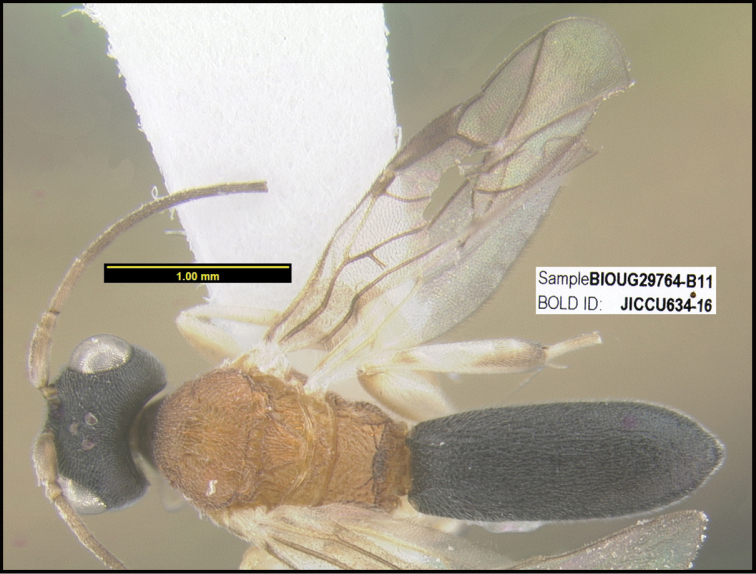
*Leptodrepanafelipechavarriai*, holotype.

##### Leptodrepana freddyquesadai

Taxon classificationAnimaliaHymenopteraBraconidae

Sharkey sp. nov.

http://zoobank.org/DDCC1A20-85C1-4A30-85A2-5CD6A5F4CE19

Figure 174

###### Diagnostics.

BOLD:ADD4610. Consensus barcode. TTATATTTTATTTTTGGAATATGGTCTGGGATGTTTGGGTTATCATTAAGATTGTTAATTCGAATAGAATTAAGTTCATTAACTTCATATATGGGGAATGATCAAATTTATAATAGAGTTGTAACTATACATGCATTTATTATAATTTTTTTTATGGTGATACCAATTATAATTGGTGGATTTGGAAATTGATTAATTCCATTAATATTAGGTGGTCCTGATATAGCGTTTCCACGAATAAATAATATAAGGTTTTGGTTATTAATTCCTTCATTATTTTTATTAATTTTAAGAAGATTAGTAAATGTAGGTGTTGGAACTGGATGAACAGTTTATCCGCCTTTATCTTTATTAATAGGGCATAGGGGAATTTCAGTGGATTTAAGAATTTTTTCTTTACATTTAGCTGGAGCATCTTCAATTATGGGAGCAATTAATTTTATTGTAACAATTATAAATATATGAATAGGTGTAAATTATATAGATAAAATTTCTTTATTTAGATGATCAGTATTAATTACAGCTATTTTATTATTATTATCTTTGCCAGTTTTAGCAGGTGCTATTACAATATTATTAACTGAT---------------------------------------------------.

###### Holotype ♀.

Guanacaste, Pailas Dos, PL12-2, 10.7634, -85.335, 824 meters, 10‒17/iv/2014, Malaise trap PL12-2A. Depository: CNC.

***Host data*.** None.

***Holotype voucher code*.**BIOUG30966-F04.

###### Paratypes.

None.

###### Etymology.

*Leptodrepanafreddyquesadai* is named to honor Sr. Freddy Quesada of GDFCF and ACG for his many years as a dedicated inventory parataxonomist for ACG.

**Figure 174.**
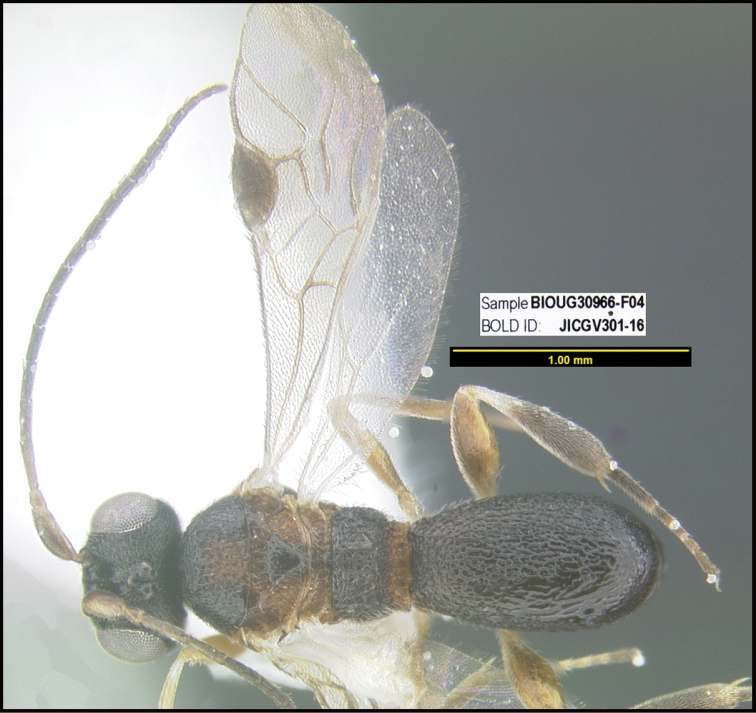
*Leptodrepanafreddyquesadai*, holotype.

##### Leptodrepana gilbertfuentesi

Taxon classificationAnimaliaHymenopteraBraconidae

Sharkey sp. nov.

http://zoobank.org/EF09ACDF-488B-46FB-90EE-67F197B2A7F1

Figure 175

###### Diagnostics.

BOLD:ACF7385. Consensus barcode. AATTTTATATTTTATTTTTGGAATATGATCAGGTTTATTTGGTTTATCATTAAGATTATTAATTCGTATAGAATTAAGTTCAGTTTCA---AGATATTTAGGTAATGATCAAATTTATAATAGTGTAGTTACAATACATGCATTTATTATAATTTTTTTTATAGTTATACCAATTATAATTGGTGGTTTTGGAAATTGATTAATTCCGTTAATGTTAGGAGGTCCAGATATAGCTTTTCCTCGTATAAATAATATAAGATTTTGGTTATTGGTTCCTTCTTTATTTTTATTGATTTTAAGAAGTTTAGTTAATGTTGGTGTTGGTACGGGTTGAACAGTTTATCCTCCTTTATCTTTGTTAATAGGTCATGGTGGAATTTCTGTAGATTTAAGAATTTTTTCTTTACATTTAGCTGGTGCTTCCTCTATTATAGGAGCTATTAATTTTATTGTAACAATTATTAATATATGAATAGGA---ATAAAATTTATAGATAAAATTTCTTTATTTAGATGATCTGTTTTAATTACTGCAATTTTATTATTATTATCTTTACCTGTTTTAGCAGGTGCTATTACTATATTGTTAACTGATCGAAATTTAAATACTAGATTTTTTGATCCTGCAGGTGGAGGGGATCCAATTTTGTATCAACATTTATTT.

###### Holotype ♀.

Guanacaste, Sector Santa Rosa, Bosque San Emilio, 10.8438, -85.6138, 300 meters, 11‒18/vi/2012, Malaise trap. Depository: CNC.

***Host data*.** None.

***Holotype voucher code*.**BIOUG05181-B07.

###### Paratypes.

None.

###### Etymology.

*Leptodrepanagilbertfuentesi* is named to honor Sr. Gilbert Fuentes of OTS/OET San Jose office for his diligent librarian efforts with ACG and other Costa Rican biodiversity publications.

**Figure 175.**
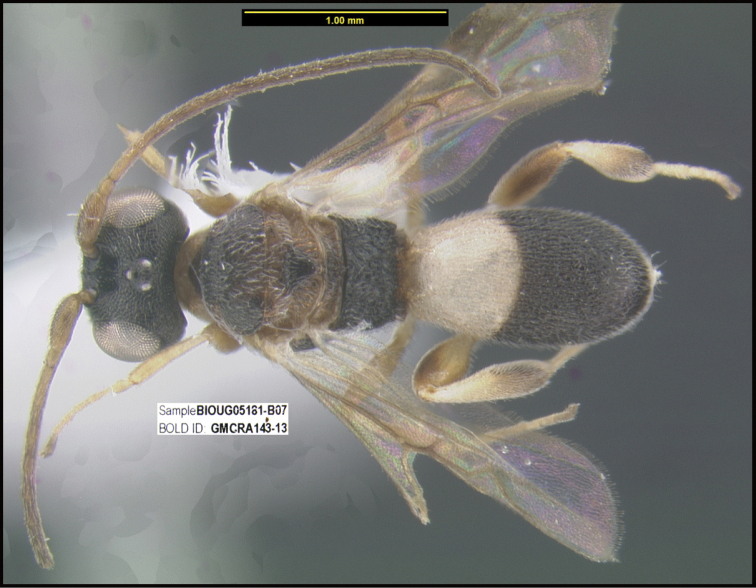
*Leptodrepanagilbertfuentesi*, holotype.

##### Leptodrepana manuelriosi

Taxon classificationAnimaliaHymenopteraBraconidae

Sharkey sp. nov.

http://zoobank.org/89A0AC1F-6C36-462D-A215-CF483DD9B7C6

Figure 176

###### Diagnostics.

BOLD:ACR7934. Consensus barcode. ATAAAATTTATAGATAAAATTTCTTTATTTAGATGATCTGTTTTAATTACTGCAATTTTATTATTATTATCTTTACCTGTTTTAGCAGGTGCTATTACTATATTGTTAACTGATCGAAATTTAAATACTAGATTTTTTGATCCTGCAGGTGGAGGGGATCCAATTTTGTATCAACATTTATTTATTTTATATTTTATTTTTGGAATATGATCTGGGATATTTGGTTTATCATTAAGTTTAATTATTCGAATAGAATTAAGATCAGTAACATCTTTTTTAGGAAATGATCAAATTTATAATAGAGTAGTAACAATGCATGCATTTATTATAATTTTTTTTATAGTTATACCAATTATAATTGGGGGATTTGGAAATTGATTAATTCCTTTGATATTAGGAGGTCCTGATATAGCTTTTCCACGAATAAATAATATAAGATTTTGATTATTAATTCCTTCATTGTGTATATTAATTTTAAGAAGATTAGTTAATATAGGAGTAGGGACAGGTTGAACAGTATATCCTCCTTTATCTTTATTAATAGGACATGGAGGGATTTCAGTAGATTTAGGGATTTTTTCATTACATTTAGCAGGAGCTTCATCAATTATAGGGGCAATTAATTTTATTGTAACAATTATAAATATATGATTAGGAATAAAATATATAGATAAGATTTCATTATTTAGATGATCAGTTTTAATTACTGCAATTCTATTATTATTATCTTTACCTGTATTAGCA------------------------------------------------------------------------.

###### Holotype ♀.

Guanacaste, Sector Santa Rosa, Bosque San Emilio, 10.8438, -85.6138, 300 meters, 04‒11/vi/2012, Malaise trap. Depository: CNC.

***Host data*.** None.

***Holotype voucher code*.**BIOUG18005-B12.

###### Paratypes.

None.

###### Etymology.

*Leptodrepanamanuelriosi* is named to honor Sr. Manuel Rios of GDFCF and ACG for his many years as a dedicated inventory parataxonomist.

**Figure 176.**
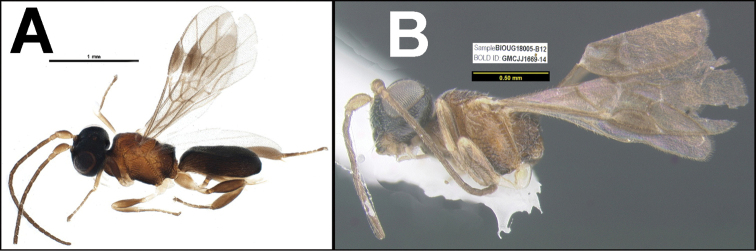
*Leptodrepanamanuelriosi*, holotype **A** image on BOLD**B** mounted image.

#### *Phanerotoma* Wesmael, 1838

There are 37 species recorded from the Neotropical region but none from Costa Rica (Yu et al. 2016). Eleven species are recorded from Panama, mostly described by Zettel (1990), and some of the species described here may be revealed as junior synonyms if and when the holotypes are barcoded. Judging from the similarly sized revision of *Leptodrepana* by Dadelahi et al. (2018), synonyms are unlikely. Recorded hosts are mostly from Pyralidae and Tortricidae. Only two of the 23 species treated here are reared, both from species of Pyralidae. The lack of host records, given the species-richness of the genus in Costa Rica, suggests that many may use leaf-miners as hosts, since this group has not been a focus of the Janzen and Hallwachs rearing program.

##### Phanerotoma almasolisae

Taxon classificationAnimaliaHymenopteraBraconidae

Sharkey sp. nov.

http://zoobank.org/E9E20D15-EBC6-456C-9A38-826A5A4C8B7E

Figure 177

###### Diagnostics.

BOLD:ADB0692. Consensus barcode. ATATTATATTTTTTATTTGGAATATATTCAGGATTTTTTGGATTATCTTTTAGAATTATAATTCGATTAGAATTAAGAGTCTTAGGACAAATAATAGGAAATGATCAAATTTATAATATAATAGTAACAATTCATGCATTTGTTATAATTTTTTTTATAGTTATACCTATTATAATTGGAGGATTTGGAAATTGATTAATTCCTTTAATATTGGGAGGTCCTGATATAAGATTTCCTCGAATGAATAATATAAGATTTTGATTATTAATTCCTTCTTTATTATTATTAATTATATCAAGATTTATTAATATAGGGGTAGGTACAGGTTGAACAGTTTATCCTCCTTTATCATTAATTATAGGACATGGGGGTGTATCTGTAGATTTAGTAATTTTTTCTTTACATTTGGCTGGAATTTCTTCAATTTTAGGTGCAATTAATTTTATTAGAACTATTTTAAATATATGATATAATATAAAAGAACTAGAAAAATTATCTTTATTTAGATGATCTGTTGCTATTACTGCTTTATTGTTATTATTATCTTTACCAGTTTTAGCAGGTGCAATTACTATATTA---------------------------------------------------.

###### Holotype ♀.

Guanacaste, Pailas Dos, PL12-6, 10.7637, -85.333, 853 meters, 20‒27/iii/2014, Malaise trap PL12-6A. Depository: CNC.

***Host data*.** None.

***Holotype voucher code*.**BIOUG29157-B02.

###### Paratypes.

None.

###### Etymology.

*Phanerotomaalmasolisae* is named to honor Dr. Alma Solis of the Smithsonian and USDA for her decades of diligent and enthusiastic support for Costa Rican biodiversity development and taxonomy in INBio, ACG, and her home office in the USA, with special emphasis on Pyraloidea microlepidopteran identifications.

**Figure 177.**
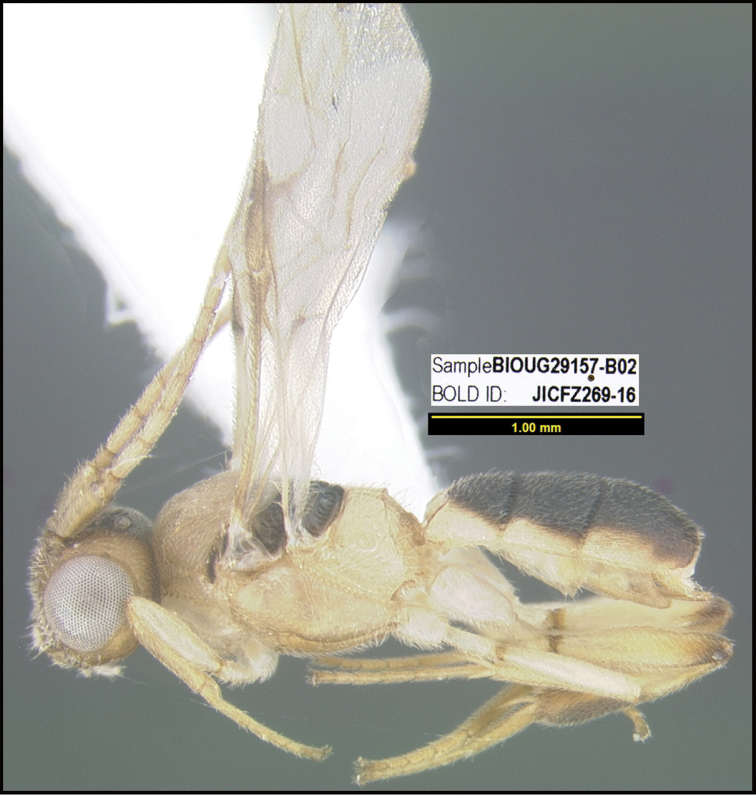
*Phanerotomaalmasolisae*, holotype.

##### Phanerotoma alvaroherrerai

Taxon classificationAnimaliaHymenopteraBraconidae

Sharkey sp. nov.

http://zoobank.org/C3A75DCE-FBD5-422C-B9D1-99DBE25017CF

Figure 178

###### Diagnostics.

BOLD:AAL8247. Consensus barcode. GAGTTTTATATTTTTTATTTGGTATTTATTCAGGGTTTTTAGGTTTATCTTTAAGAATAATAATTCGTTTAGAATTAAGAGTGTTAGGGGRGATAYTAGGTAATGATCAAATTTATAATACAATTGTTACTATTCATGCTTTTATTATAATTTTTTTTATAGTAATACCAATTATAATTGGAGGRTTTGGRAATTGATTAGTRCCTTTAATATTAGGTGGTCCTGATATAAGRTTYCCTCGAATAAATAATATAAGATTTTGGTTATTAATTCCTTCATTAATTTTAATAATTTTATCTAGTTTTGTMAATGTTGGGGCTGGTACTGGATGAACARTYTAYCCTCCTTTRTCATTATTAGTTGGKCATGGGGGGGTTTCAGTTGATTTAGTAATTTTTTCTTTACATTTGGCAGGYRTTTCTTCAATTTTAGGGGCAATTAATTTTATTAGTACAGTTTTAAATATATGAGTTGGGTATAAAATGATAGATAAATTATCTTTRTTTATTTTTTCTGTAGTAATTACAGCATTATTATTATTATTATCTTTACCTGTTTTAGCTGGGGCYATTACAATRTTATTATTGGATCGAAATTTAAATACTAGATTTTTTGATCCTAGAGGMGGTGGGGATCCAGTTTTGTATCAACATTTATTT.

###### Holotype ♂.

Guanacaste, Sector Santa Rosa, Bosque San Emilio, 10.8438, -85.6138, 300 meters, 21‒28/i/2013, Malaise trap. Depository: CNC.

***Host data*.** None.

***Holotype voucher code*.**BIOUG18290-F04.

###### Paratypes.

All Malaise-trapped. BIOUG07918-F10, BIOUG09739-D11, BIOUG09739-C01, BIOUG08348-E03, BIOUG09739-H08, BIOUG10016-F02, BIOUG10017-G06, BIOUG10112-B02, BIOUG10112-B03, BIOUG10112-E11, BIOUG10247-E11, BIOUG17575-A10, BIOUG18289-H10, BIOUG18290-A04, BIOUG18290-D02, BIOUG18290-F04, BIOUG18290-F11, BIOUG18397-C11, BIOUG18398-B05, BIOUG18430-E07, BIOUG18499-D10, BIOUG18499-F05, BIOUG18499-F06. Depository: CNC.

###### Other material.

Two specimens from the same BIN were found on BOLD; one from Oklahoma (BOLD voucher 09BBHYM-914) and the other from southern Florida (BOLD voucher 10BBHYM-1201). We doubt that they are conspecific with the Costa Rican specimens based on their distributions, but they were not examined and deserve closer scrutiny.

###### Etymology.

*Phanerotomaalvaroherrerai* is named to honor Sr. Alvaro Herrera for his long patience and efforts in the administration of the debt swap purchase of 22A of Sector Rincon Rain Forest in ACG in 2014–2015.

**Figure 178.**
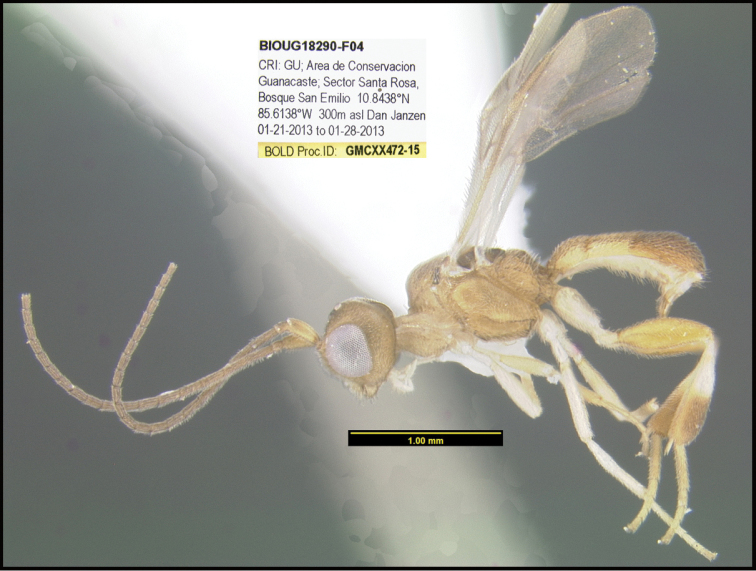
*Phanerotomaalvaroherrerai*, holotype.

##### Phanerotoma anacordobae

Taxon classificationAnimaliaHymenopteraBraconidae

Sharkey sp. nov.

http://zoobank.org/3279D75C-BF9D-4772-ACB1-753380B2DCAE

Figure 179

###### Diagnostics.

BOLD:ACJ2167. Consensus barcode. GAGTTTTATATTTTTTATTTGGTATTTATTCTGGGKTTTTAGGTTTRTCTTTAAGAATAATAATTCGATTGGAATTAAGAGTWTTAGGGGAAATATTAGGTAATGAYCAAATTTATAATACAATTGTAACTATTCATGCTTTTATTATAATTTTTTTTATAGTYATACCAATTATAATTGGTGGTTTTGGTAATTGATTAGTWCCTTTAATATTAGGTGGACCTGATATAAGATTYCCTCGAATAAATAATATAAGATTTTGATTATTAATTCCTTCATTAATTTTAATAATTTTATCTAGTTTTGTAAATGTAGGGGCTGGAACTGGATGRACAGTTTATCCTCCTTTATCATTATTAGTTGGTCATGGTGGTATTTCAGTTGATTTAGTAATTTTTTCTTTACATTTGGCAGGTATTTCTTCAATTTTAGGTGCAATTAATTTTATTAGTACTGTWTTAAATATATGGGTTGRTTATAAATTGATAGATAAATTATCTTTATTTATTTTTTCTGTAGTAATTACAGCATTGTTATTGTTATTATCTTTACCTGTATTAGCTGGAGCAATTACAATGTTATTGTTGGATCGAAATTTAAATACAAGATTTTTTGACCCAAGAGGTGGAGGAGATCCAATTTTATATCAACATTTATTT.

###### Holotype ♀.

Guanacaste, Sector Santa Rosa, Bosque San Emilio, 10.8438, -85.6138, 300 meters, 16‒23/iv/2012, Malaise trap. Depository: CNC.

***Host data*.** None.

***Holotype voucher code*.**BIOUG07616-D05.

###### Paratypes.

All Malaise-trapped. BIOUG07616-F09, BIOUG07781-H06, BIOUG07918-H09, BIOUG08266-F05, BIOUG10016-G07, BIOUG10016-H06, BIOUG10017-H03, BIOUG10247-H02, BIOUG10753-B11, BIOUG17335-B07, BIOUG17335-E04, BIOUG17335-H08, BIOUG17346-E10, BIOUG17684-B01, BIOUG17684-B12, BIOUG17684-D11, BIOUG18290-C10, BIOUG18290-D04, BIOUG18290-E12, BIOUG18290-G07, BIOUG18290-H09, BIOUG18397-C12, BIOUG18398-B04, BIOUG18398-D11, BIOUG18398-F09, BIOUG18398-G07, BIOUG18498-H02, BIOUG18499-A12, BIOUG18499-H09, BIOUG18661-G06, BIOUG18664-E01.

###### Etymology.

*Phanerotomaanacordobae* is named to honor Sra. Ana Cordoba of GDFCF and ACG for her many years as a dedicated inventory parataxonomist for ACG.

**Figure 179.**
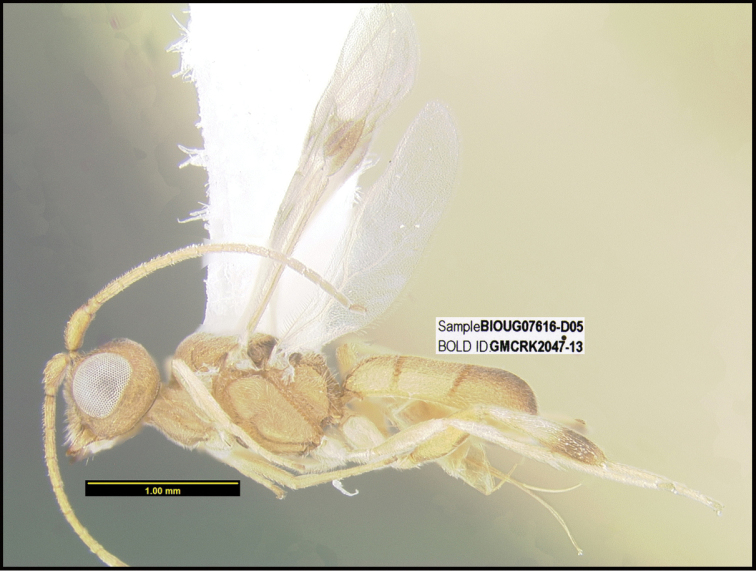
*Phanerotomaanacordobae*, holotype.

##### Phanerotoma anamariamongeae

Taxon classificationAnimaliaHymenopteraBraconidae

Sharkey sp. nov.

http://zoobank.org/87CAC79F-2097-4F30-A5C6-04AB0B0314CC

Figure 180

###### Diagnostics.

BOLD:ADA6322. Consensus barcode. AGTTTTATATTTTTTATTTGGTATTTATTCAGGATTTTTRGGDYTATCTTTAAGAATAATAATTCGTTTAGAATTAAGAGTATTAGGRGAAATATTAGGTAATGATCAAATTTATAATACAATTGTAACTATYCATGCTTTTATTATAATTTTTTTTATAGTTATACCAATTATAATTGGTGGGTTTGGAAATTGATTAGTACCTTTAATATTAGGTGGTCCTGATATAAGGTTTCCTCGAATAAATAATATAAGTTTTTGATTATTAATTCCTTCATTAATTTTAATAATTTTGTCTAGRTTTGTAAATGTAGGAGCTGGAACTGGGTGAACAGTTTAYCCTCCTTTATCATTATTARTTGGRCATGGTGGTGTTTCAGTTGATTTGGTAATTTTTTCTTTACATTTAGCRGGTRTTTCTTCAATTTTAGGKGCAATTAATTTTATTAGAACGGTTTTAAATATATGAATTGGTTATAAAATAATGGATAAATTGTCTTTATTTATTTTTTCTGTAGTGATTACAGCATTATTRTTATTATTATCTTTACCTGTTTTAGCTGGGGCAATTACAATATTATTATTGGATCGAAATTTAAATACAAGATTTTT.

###### Holotype ♀.

Guanacaste, Pailas Dos, PL12-9, 10.76, -85.3341, 809 meters, 06‒13/ii/2014, Malaise trap PL12-9A. Depository: CNC.

***Host data*.** None.

***Holotype voucher code*.**BIOUG28728-G08.

###### Paratypes.

All Malaise-trapped. BIOUG28388-H02, BIOUG28667-D07, BIOUG28651-A02, BIOUG28674-H11, BIOUG28743-A02, BIOUG28678-G12, BIOUG28721-A12, BIOUG28770-H07, BIOUG28770-H10, BIOUG28770-H11, BIOUG28724-C04, BIOUG28728-A09, BIOUG28728-G09, BIOUG28728-G10, BIOUG28729-D07, BIOUG28846-B09, BIOUG28823-E08, BIOUG28825-B01, BIOUG28777-A04, BIOUG28779-B01. Depository: CNC.

###### Etymology.

*Phanerotomaanamariamongeae* is named to honor Srta. Ana Maria Monge for her enthusiastic support for the integration of the NGO BioAlfa into the SINAC government bureaucracy.

**Figure 180.**
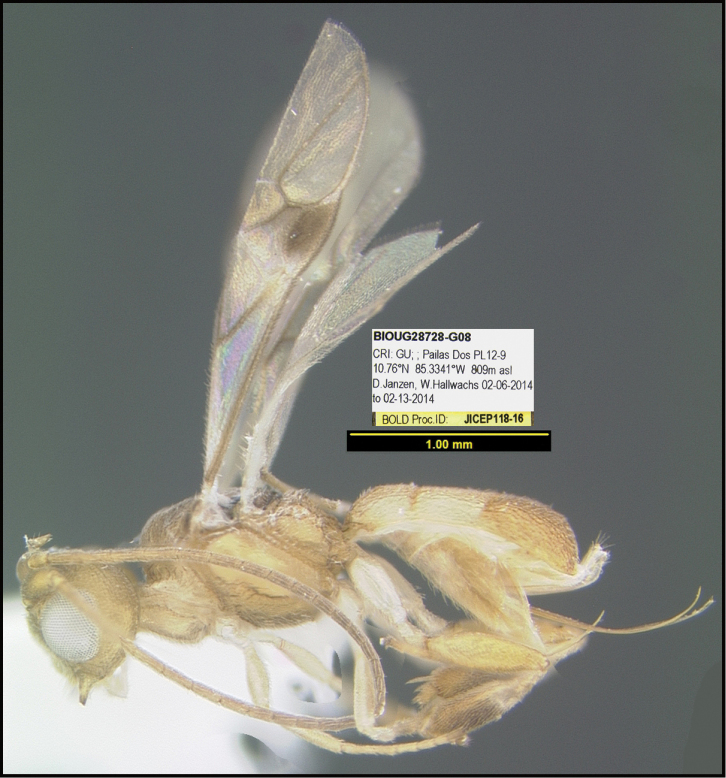
*Phanerotomaanamariamongeae*, holotype.

##### Phanerotoma andydeansi

Taxon classificationAnimaliaHymenopteraBraconidae

Sharkey sp. nov.

http://zoobank.org/CA8F53F2-12E7-49B7-9D16-7957061D1060

Figure 181

###### Diagnostics.

BOLD:ADA7613. Consensus barcode. GTATTATATTTTTTATTTGGTATTTATTCAGGATTTTTAGGTTTATCATTAAGAATAATAATTCGATTAGAATTAAGAGTTTTAGGTGAAATGTTAGGGAATGATCAGGTTTATAATACAATTGTTACAATACATGCTTTTGTAATAATTTTTTTTATAGTTATACCAATTATAATTGGAGGTTTTGGGAATTGATTAGTACCTTTAATATTAGGTGGTCCTGATATAAGTTTTCCTCGAATAAATAATATAAGATTTTGATTGTTAATTCCTTCTTTAATTTTAATAATTTTATCAAGATTTGTAAATGTTGGTGCTGGTACAGGATGAACAGTTTATCCTCCTTTATCATTATTAATTGGTCATGGTGGTATTTCAGTTGATTTAGTAATTTTTTCTTTACATTTGGCAGGTATTTCATCAATTTTAGGTGCTATTAATTTTATTAGAACAGTTTTAAATATATGAATTAGTTTAAAAATAATAGATAAATTAACATTATTTATTTTTTCTGTAATAATTACAGCAATATTATTATTGTTGTCATTACCTGTWTTGGCTGGAGCAATTACAATATTATTATTAGATCGAAATATAAAT.

###### Holotype ♀.

Guanacaste, Pailas Dos, PL12-6, 10.7637, -85.333, 853 meters, 01‒08/v/2014, Malaise trap PL12-6A. Depository: CNC.

***Host data*.** None.

***Holotype voucher code*.**BIOUG29322-E10.

###### Paratypes.

All Malaise-trapped. BIOUG28394-F08, BIOUG28673-C05, BIOUG28764-F10, BIOUG28766-E08, BIOUG28722-F01, BIOUG28728-H01, BIOUG28823-E07, BIOUG28830-H02, BIOUG29059-D04, BIOUG29060-G09, BIOUG29064-E01, BIOUG29140-A12, BIOUG29146-E10, BIOUG29322-E08, BIOUG29322-E10, BIOUG29377-F12, BIOUG29403-F11, BIOUG29406-G10, BIOUG29388-H08, BIOUG29398-F04, BIOUG29530-G09, BIOUG29511-G04, BIOUG29725-C09, BIOUG29991-F01, BIOUG30959-G11. Depository: CNC.

###### Etymology.

*Phanerotomaandydeansi* is named to honor Dr. Andy Deans of Pennsylvania State University for his intense taxonomic development of ACGMicrogastrinae wasps and his later contributions as a museum director.

**Figure 181.**
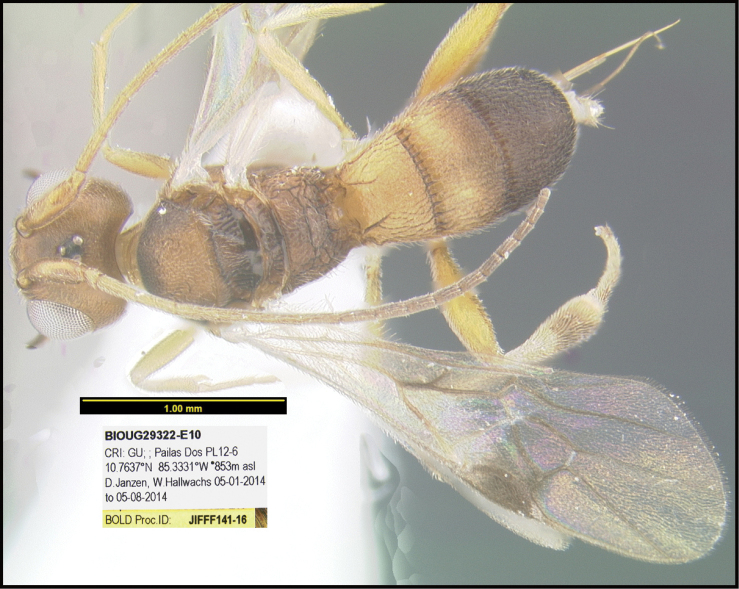
*Phanerotomaandydeansi*, holotype.

##### Phanerotoma angelagonzalezae

Taxon classificationAnimaliaHymenopteraBraconidae

Sharkey sp. nov.

http://zoobank.org/44A35082-2B56-44FC-BE21-843A96D159BE

Figure 182

###### Diagnostics.

BOLD:ADB3156. Consensus barcode. GTATTATATTTTTTGTTTGGAATTTATTCAGGAATTTTAGGTTTATCATTAAGTATAATAATTCGATTAGAATTAAGAGTTTTGGGGGAAATATTAGGTAATGATCAAGTTTATAATATAATAGTAACTATTCATGCTTTTATTATAATTTTTTTTATAGTTATACCAATTATAATTGGGGGATTTGGAAATTGGTTAGTACCTTTAATATTAGGAGGTCCTGATATAAGATTTCCTCGTATAAATAATATAAGATTTTGGTTATTAATTCCATCTTTAATATTATTAATTTTATCAAGATTTGTAAATATAGGAGCTGGAACAGGATGAACTATTTACCCTCCATTATCATTAATATTAGGTCATGGAGGAGTTTCTGTAGATTTAGTAATTTTTTCATTACATTTAGCAGGTATTTCATCAATTTTAGGTGCAATTAATTTTATTAGAACAGTTTTAAATATATGATTTATAAAAAGTATAATAGATAAATTGTCTTTATTTATTTTTTCAGTAGTTATTACTGCATTATTATTATTATTATCATTACCGGTTTTAGCTGGAGCTATTACTATATTATTAATAGATCGTAATTTAAAT.

###### Holotype ♀.

Guanacaste, Pailas Dos, PL12-3, 10.7631, -85.3344, 820 meters, 08‒15/v/2014, Malaise trap PL12-3A. Depository: CNC.

***Host data*.** None.

***Holotype voucher code*.**BIOUG29845-C10.

###### Paratypes.

All Malaise-trapped. BIOUG29320-A09, BIOUG29862-C07, BIOUG30984-H06. Depository: CNC.

###### Etymology.

*Phanerotomaangelagonzalezae* to honor Dr. Angela Gonzalez, Directora of CONAGEBIO, for her constant encouragement and facilitation of the growth and development of BioAlfa for Costa Rica.

**Figure 182.**
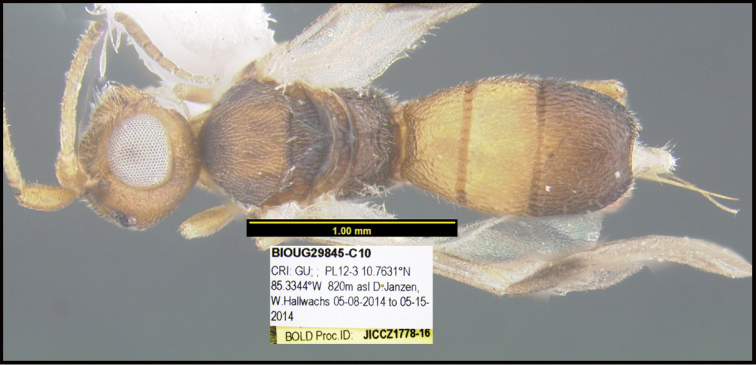
*Phanerotomaangelagonzalezae*, holotype.

##### Phanerotoma angelsolisi

Taxon classificationAnimaliaHymenopteraBraconidae

Sharkey sp. nov.

http://zoobank.org/4EF4BC86-D742-4524-95B2-9C0DB4768031\

Figure 183

###### Diagnostics.

BOLD:AAG8211. Consensus barcode. TGTATTRTATTTTTTWTTTGGAATTTATTCAGGTATTTTTGGTTTRTCATTAAGTATAATAATTCGATTAGAATTAAGAGTTTTAGGAGAARTRTTAGGTAATGATCAAGTTTATAATACAATTGTWACAATTCATGCTTTTATTATAATTTTTTTTATGGTTATACCARTTATAATTGGTGGTTTTGGWAATTGATTARTTCCTTTAATATTAGGAGGTCCYGATATAAGRTTTCCTCGAATAARTAATATAAGATTTTGRTTATTAATTCCTTCTTTATTAATATTAATTTTATCAAGTTTTRTTAATGTAGGTGYTGGAACAGGTTGAACTRTWTAYCCTCCTTTRTCTTTAYTRGTAGGTCATGGAGGTGTTTCRGTTGATATAGTAATTTTTTCTTTACATTTAGCAGGAATTTCWTCAATTTTRGGTGCTATTAATTTTATTAGAACAGTTTTAAATATATGGTTTGATTATRAAATAATAGATAAAYTGTCTTTATTTATTTTTTCTGTAKTTATTACAGCATTATTATTATTATYATCYTTACCTGTTTTAGCTGGTGCAATTACDATATTATTRTTAGATCGAAATTTAAAKACAAGATTTTTTGATCCAAGAGGAGGTGGGGATCCTATTTTATAYCAACATTTATTT. Restricted to Central America.

###### Holotype ♀.

Guanacaste, Pailas Dos, PL12-3, 10.7631, -85.3344, 820 meters, 12‒19/xii/2013, Malaise trap PL12-3A. Depository: CNC.

***Host data*.** None.

***Holotype voucher code*.**BIOUG29415-C09.

###### Paratype.

BIOUG07751-A11. Depository: CNC.

###### Other material.

BIOUG07751-A11 (Malaise-trapped) from Honduras was examined and considered conspecific, and it is included as a paratype. There are dozens of specimens in the same BIN that are from widespread locations across Canada and the USA. Several of these were examined and they differ significantly in color from the Mesoamerican specimens and are not considered conspecific. See BOLD for details.

###### Etymology.

*Phanerotomaangelsolisi* is named to honor Mr. Angel Solis of BioAlfa for his decades of enthusiastic taxonomic and administrative support of INBio, parataxonomists, and all matters biodiversity of Costa Rica coupled with intensely developing BioAlfa of Costa Rica.

**Figure 183.**
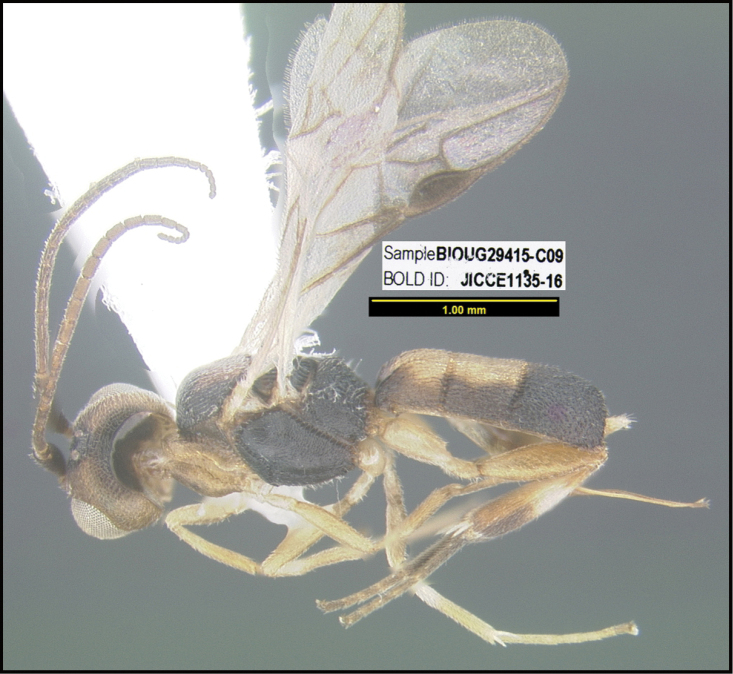
*Phanerotomaangelsolisi*, holotype.

##### Phanerotoma barryhammeli

Taxon classificationAnimaliaHymenopteraBraconidae

Sharkey sp. nov.

http://zoobank.org/20727B66-1867-44F3-A71D-22C19A9F7C06

Figure 184

###### Diagnostics.

BOLD:ADG7956. Consensus barcode. AGTATTATATTTTTTATTTGGTATTTATTCAGGTGTATTAGGTTTATCTTTAAGTATAATAATTCGATTAGAATTGAGGGTTTTAGGAGAAATGTTAGGTAATGATCAAGTTTATAATACTATTGTTACTATTCATGCTTTTATTATAATTTTTTTTATAGTTATACCAATTATAATTGGTGGGTTTGGTAATTGGTTAGTTCCATTAATATTAGGGGGACCTGATATAAGATTCCCTCGTATGAATAATATAAGGTTTTGGTTATTAGTACCTTCATTAATTTTATTAGTTTTATCAAGATTTGTAAATATAGGGGTTGGTACAGGATGAACAGTTTACCCTCCATTATCTTTAACATTAGGACATGGAGGTATTTCAGTAGATTTAGTAATTTTTTCTTTACATTTAGCAGGTATTTCTTCAATTTTGGGGGCGATTAATTTTATTAGTACAGTATTAAATATATGATTTAATTATAAAATGATAGATAAATTATCTTTATTTATTTTTTCTGTAGTAATTACAGCATTATTATTATTATTATCTTTACCTGTTTTAGC.

###### Holotype ♂.

Guanacaste, Sector Cacao, Derrumbe, 10.9292, -85.4643, 1220 meters, 19‒26/ii/2015, Malaise trap. Depository: CNC.

***Host data*.** None.

***Holotype voucher code*.**BIOUG32861-A06.

###### Paratypes.

None.

###### Etymology.

*Phanerotomabarryhammeli* is named to honor Dr. Barry Hammel of the Missouri Botanical Garden and the Museo Nacional de Costa Rica for his decades of direct mentoring and plant taxonomy for the plants of Costa Rica.

**Figure 184.**
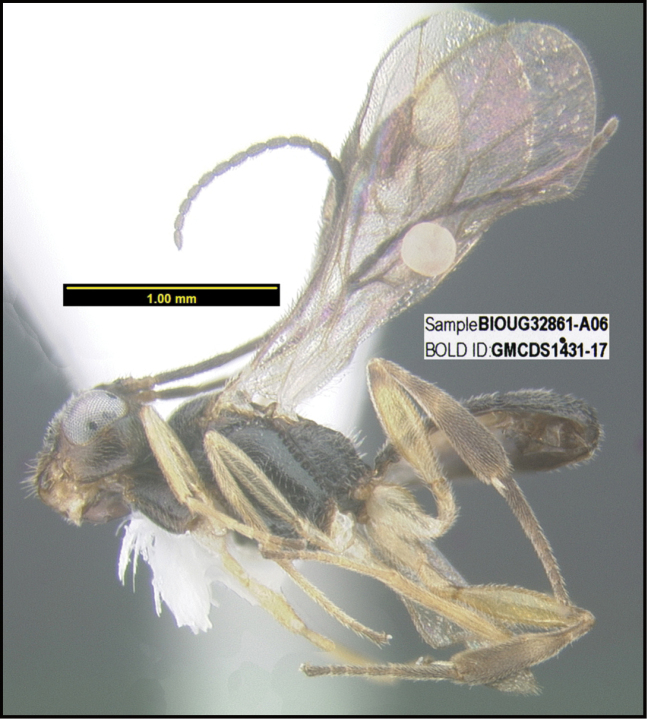
*Phanerotomabarryhammeli*, holotype.

##### Phanerotoma bernardoespinozai

Taxon classificationAnimaliaHymenopteraBraconidae

Sharkey sp. nov.

http://zoobank.org/7A4AFFC3-D95B-43D3-91A8-3BDDA2CD2EE2

Figure 185

###### Diagnostics.

BOLD:ACJ8069. Consensus barcode. AGTTTTATATTTTTTGTTAGGTATATATTCTGGAATAATAGGTTTATCATTAAGTTTATTAATTCGTTTGGAATTAAGTGTTTTAGGA------TGTATAATAGGTAATGATCAAGTTTACAATATAATTGTTACTATTCATGCATTTATTATAATTTTTTTTATAGTTATACCAATTATAATTGGTGGATTTGGAAATTGATTAGTTCCATTAATATTAGGTGGACCTGATATAAGTTTTCCTCGAATAAATAATATAAGTTTTTGGTTATTAATTCCTTCTTTAATTTTATTAATAATATCAAGTTTTGTAAATGTTGGTGCTGGAACTGGTTGAACTGTTTATCCACCATTATCATTAATAATTGGTCATGGAGGAATTTCTGTTGATTTAGTAATTTTTTCTTTACATTTGGCTGGAATTTCTTCAATTTTAGGTGCTATTAATTTTATTTCTACTGTATTAAATATATGATTTAATTTAAAAATATTAGAAAAAATATCTTTATTTATTTGATCTGTTGTTATTACTGCTTTATTATTATTATTATCTTTACCTGTTTTAGCAGGTGCTATTACTATATTATTAATAGATCGAAATTTAAATACAAGATTTTTTGATCCAAGAGGAGGTGGTGATCCTGTGTTATATCAACATTTATTT.

###### Holotype ♀.

Guanacaste, Sector Santa Rosa, Bosque San Emilio, 10.8438, -85.6138, 300 meters, 04/vi/2012, Malaise trap. Depository: CNC.

***Host data*.** None.

***Holotype voucher code*s.**BIOUG08356-H07.

###### Paratypes.

None.

###### Etymology.

*Phanerotomabernardoespinozai* is named to honor Sr. Bernardo Espinoza for his decades of high-quality taxonomic actions for the curation and taxonomy of the INBio and now Museo Nacional insect collection, with special emphasis on BioAlfa and the Arctiidae of ACG and Costa Rica in overall.

**Figure 185.**
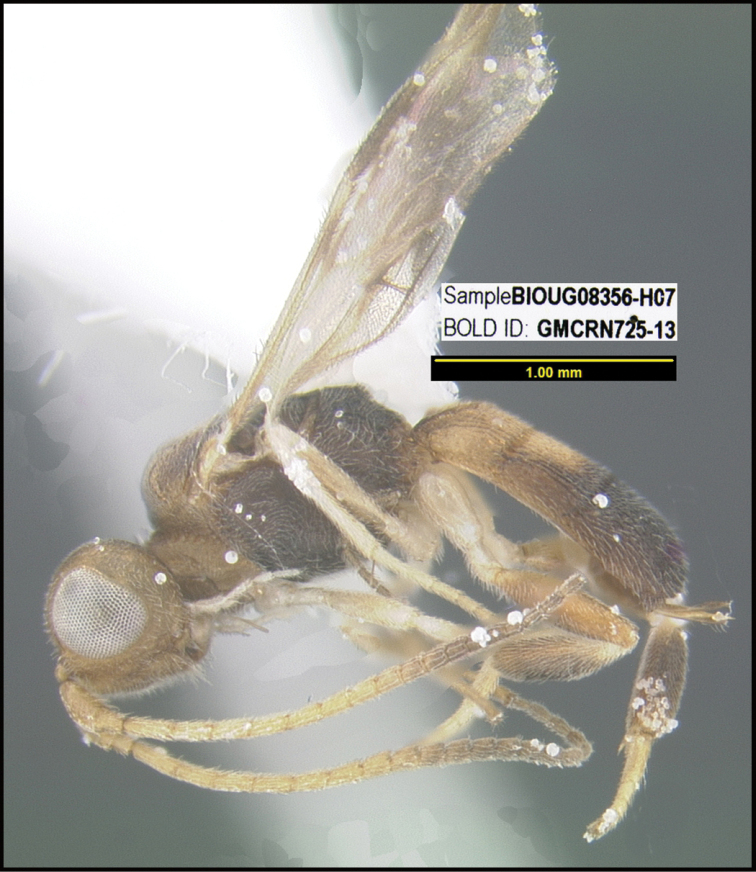
*Phanerotomabernardoespinozai*, holotype.

##### Phanerotoma calixtomoragai

Taxon classificationAnimaliaHymenopteraBraconidae

Sharkey sp. nov.

http://zoobank.org/3EBB00F5-4300-4B7C-AEAF-BAD2A0E0F1C8

Figure 186

###### Diagnostics.

BOLD:ABV9880. Consensus barcode. AATTTTATATTTTTTATTTGGAATGTATTCAGGAATAATAGGTTTATCATTAAGTTTATTAATTCGTTTAGAATTAAGAGTTTTAGGAAATATATTAGGTAATGATCAAGTTTATAATATAATTGTTACTATTCATGCTTTTATTATAATTTTTTTTATAGTAATACCTATTATAATTGGAGGTTTTGGAAATTGGTTAATTCCTTTAATATTAGGAGGTCCTGATATAAGATTTCCTCGGATGAATAATATAAGGTTTTGATTATTAGTTCCTTCTTTAATTTTATTGAGTATGTCAAGATTTATTAATATAGGGGTTGGAACAGGTTGAACAGTTTATCCTCCTTTATCTTTAATAATTGGACATGGTGGGGTTTCCGTTGATTTAGTTATTTTTTCTTTACATTTAGCTGGAATTTCTTCAATTTTAGGGGCTATTAATTTTATTAGAACTATTTTAAATATATGAATTAATCTTAAAATAATAGATAAATTATCTTTATTTATTTGATCTGTTATGATTACTGTTTTATTATTGTTATTATCTTTACCAGTTTTAGCAGGAGCTATTACTATATTATTAATAGATCGAAATCTTAATACAAGATTTTTTGATCCAAGAGGAGGGGGGGATCCAGTATTATATCAGCATTTATTT.

###### Holotype ♀.

Guanacaste, Sector Cacao, Sendero Cima, 10.933, -85.457, 1460 meters, 20/x/2008, Malaise trap. Depository: CNC.

***Host data*.** None.

***Holotype voucher code*.**DHJPAR0045952.

###### Paratypes.

None.

###### Etymology.

*Phanerotomacalixtomoragai* is named to honor Sr. Calixto Moraga of GDFCF and ACG for his many years as a dedicated inventory parataxonomist for ACG.

**Figure 186.**
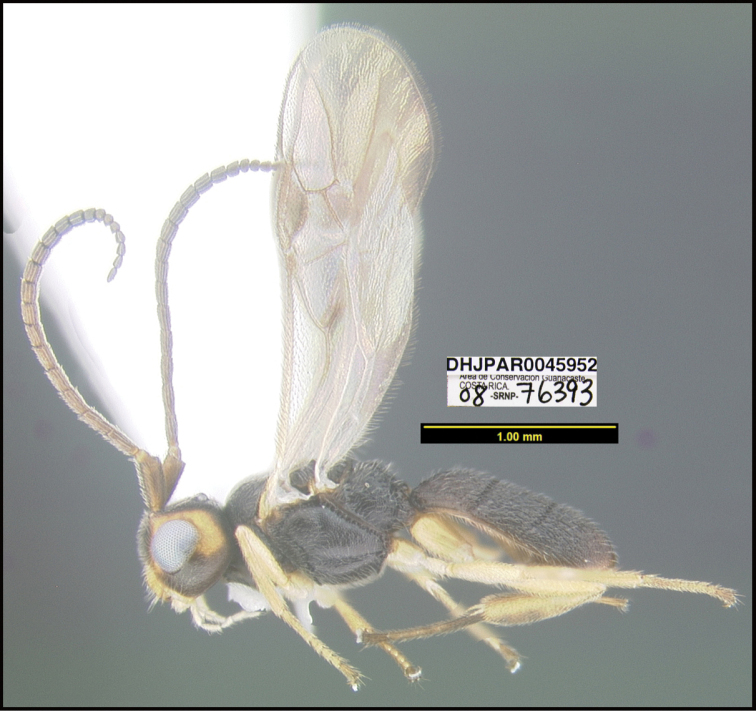
*Phanerotomacalixtomoragai*, holotype.

##### Phanerotoma carolinacanoae

Taxon classificationAnimaliaHymenopteraBraconidae

Sharkey sp. nov.

http://zoobank.org/802AF82F-83EC-4362-AD29-E73939990644

Figure 187

###### Diagnostics.

BOLD:ADB0776. Consensus barcode. TTATTATATTTTTTATTTGGTATATATTCTGGAATAATAGGATTATCTTTAAGATTATTAATTCGTTTAGAATTAAGGGTTTTAGGTAATATATTAGGGAATGATCAAATTTATAATATAGTTGTTACTATTCATGCTTTTATTATAATTTTTTTTATAGTAATACCAGTTATAATTGGTGGTTTTGGAAATTGATTAATTCCTTTAATGTTAGGAGGTCCTGATATAAGATTTCCTCGAATAAATAATATAAGTTTTTGATTGTTAATTCCTTCATTAATGTTATTAAGATTATCAAGATTTATTAATATAGGTGCTGGAACTGGTTGAACTATTTACCCTCCTTTATCCTTAATAATTGGTCATGGAGGATTTTCTGTTGATATAGTAATTTTTTCATTACATTTAGCTGGAATTTCATCAATTTTAGGAGCAATTAATTTTATCACTACAGTTTTAAATATATGGTTTAATATAAAAATAATAGATAAATTATCTTTATTTATTTGATCAGTAATTATTACTGCTTTATTATTATTATTATCTTTACCTGTATTAGCAGGAGCAATTACAATATTATTAATAGATCGTAAT---------------------------------------------.

###### Holotype ♂.

Guanacaste, Pailas Dos, PL12-9, 10.76, -85.3341, 809 meters, 16‒23/i/2014, Malaise trap PL12-9A. Depository: CNC.

***Host data*.** None.

***Holotype voucher code*.**BIOUG28722-E11.

###### Paratypes.

None.

###### Etymology.

*Phanerotomacarolinacanoae* is named to honor Sra. Carolina Cano of GDFCF and ACG for her many years as a dedicated inventory parataxonomist for ACG.

**Figure 187.**
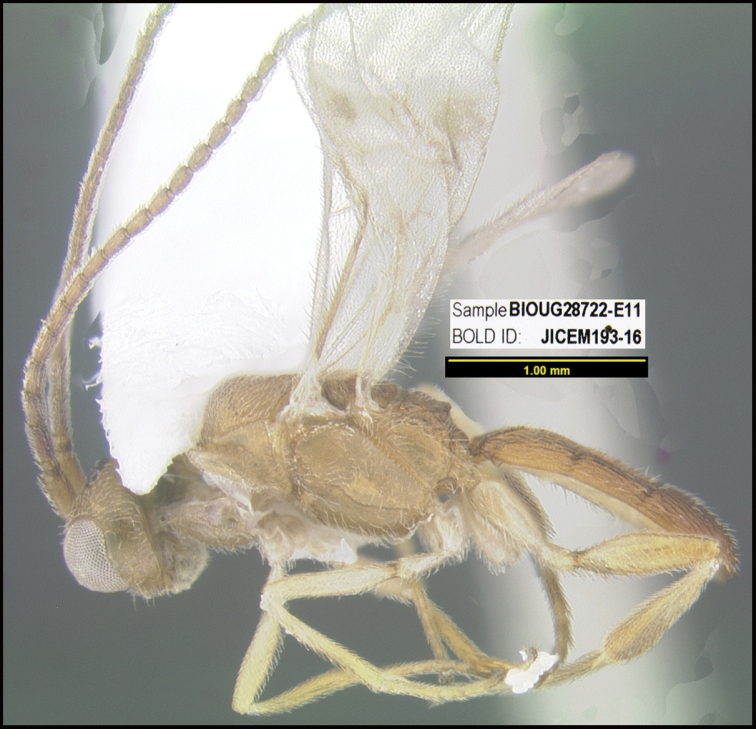
*Phanerotomacarolinacanoae*, holotype.

##### Phanerotoma christerhanssoni

Taxon classificationAnimaliaHymenopteraBraconidae

Sharkey sp. nov.

http://zoobank.org/70A14DC8-2B5E-45DF-BEDC-8ED7975F0360

Figure 188

###### Diagnostics.

BOLD:ACL3879. Consensus barcode. TGTTTTATATTTTTTATTTGGAATGTATTCAGGAATAATAGGTTTATCTTTAAGATTATTAATTCGTTTGGAATTAAGTGTATTAGGGTCAATATTAGGTAATGATCAAATTTATAATATAGTTGTTACAGTTCATGCTTTTATTATAATTTTTTTTATAGTTATACCTATTATGATTGGTGGTTTTGGAAATTGATTAGTTCCTTTAATATTAGGAGGACCTGATATAAGGTTTCCTCGAATAAATAATATAAGATTTTGATTGTTAATTCCTTCATTAATATTATTATGTTTATCTAGATTTATTAATATAGGTGCTGGTACAGGTTGAACTGTTTATCCTCCTTTATCTTTAATAATTGGTCATGGGGGAATTTCAGTAGATTTAGTAATTTTTTCATTACATTTAGCAGGAATTTCATCAATTTTAGGTGCTATTAATTTTATTAGAACTGTGTTGAATATGTGATTTAAATTGAAAATAATAGATAAATTATCATTATTTATTTGATCAGTTATAATTACTGCTTTATTGTTATTGTTATCTTTACCTGTATTGGCAGGTGCAATTACTATATTATTAATAGATCGTAATTTAAATACAAGTTTTTTTGATCCTAGAGGTGGTGGTGATCCAATTTTATATCAACATTTATTT.

###### Holotype ♀.

Guanacaste, Sector Santa Rosa, Bosque San Emilio, 10.8438, -85.6138, 300 meters, 31/xii/2012‒07/i/2013, Malaise trap. Depository: CNC.

***Host data*.** None.

***Holotype voucher code*.**BIOUG09826-F06.

###### Paratypes.

All Malaise-trapped. BIOUG10016-H08, BIOUG17462-A10, BIOUG17575-C11, BIOUG17616-B12, BIOUG18290-F03, BIOUG18397-B07. Depository: CNC.

###### Etymology.

*Phanerotomachristerhanssoni* is named to honor Dr. Christer Hansson of Lund University, Sweden, for his huge contribution to the taxonomy of the Eulophidae of Costa Rica overall, and ACG specifically.

**Figure 188.**
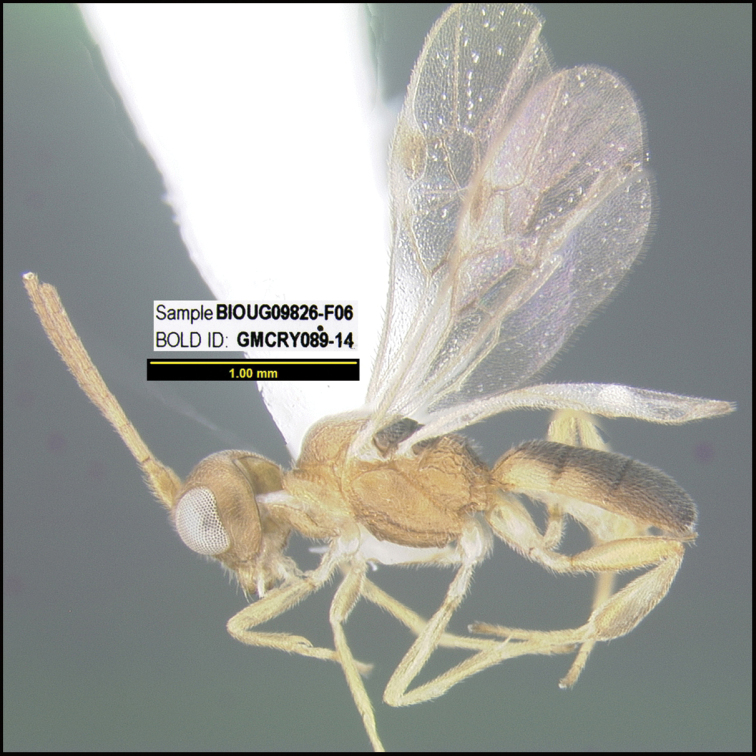
*Phanerotomachristerhanssoni*, holotype.

##### Phanerotoma christhompsoni

Taxon classificationAnimaliaHymenopteraBraconidae

Sharkey sp. nov.

http://zoobank.org/9BD9D022-74E7-4BAC-A7C9-262B2E2DD74E

Figure 189

###### Diagnostics.

BOLD:ACL5703. Consensus barcode. TGTTTTATATTTTTTATTTGGAATATATTCAGGTATGTTAGGTTTATCATTAAGTTTATTRATTCGTTTGGAATTAAGTGTTTTAGGATCTATATTAGGTAATGATCAAATTTATAATATAATTGTTACTRTTCATGCTTTTATTATAATTTTTTTTATAGTTATACCAATTATAATTGGTGGKTTTGGAAATTGATTAGTTCCTTTAATATTAGGGGGTCCTGATATAAGTTTTCCTCGAATAAATAATATAAGATTTTGATTATTAATTCCTTCATTAATATTATTATGTTTATCAAGATTTATTAATATAGGAGCTGGAACAGGATGAACAGTGTATCCACCTTTATCTTTAATAATTGGTCATGGAGGAATTTCAGTAGATTTAGTAATTTWTTCKTTACATTTGGCTGGAATTTCTTCTATTTTAGGTGCAATTAATTTTATTAGAACTGTTTTAAATATRTGATTTTTATTAAAAATAATAGATAAATTATCATTRTTTATTTGATCAGTAATWATTACTGCTTTATTATTATTATTATCTTTACCTGTATTAGCRGGTGCTATTACAATATTATTAATGGATCGAAATTTAAATACAAGRTTTTTTGATCCTAGAGGTGGTGGAGATCCAATTTTATATCAACATTTATTT.

###### Holotype ♀.

Guanacaste, Sector Santa Rosa, Bosque San Emilio, 10.8438, -85.6138, 300 meters, 17‒24/xii/2012, Malaise trap. Depository: CNC.

***Host data*.** None.

***Holotype voucher code*.**BIOUG09739-B06.

###### Paratypes.

All Malaise-trapped. BIOUG05346-A11, BIOUG08356-G03, BIOUG05422-D07, BIOUG08904-A11, BIOUG08905-F02, BIOUG08905-F11, BIOUG08911-A03, BIOUG09435-D11, BIOUG09435-D12, BIOUG09739-B06, BIOUG09739-F12,BIOUG09827-A02, BIOUG10017-D06, BIOUG17494-A10, BIOUG17494-C11, BIOUG17569-F01, BIOUG17569-F07, BIOUG17575-E05, BIOUG17575-E09, BIOUG18290-E11, BIOUG18290-F08, BIOUG18397-B01, BIOUG18551-A08. Depository: CNC.

###### Etymology.

*Phanerotomachristhompsoni* is named to honor Dr. Chris Thompson of the Smithsonian Institution for his decades and continuing intense enthusiasm for the taxonomy of Costa Rican Syrphidae flies and subsequent development of the INBio collections and training of INBio curators, including their current financial support.

**Figure 189.**
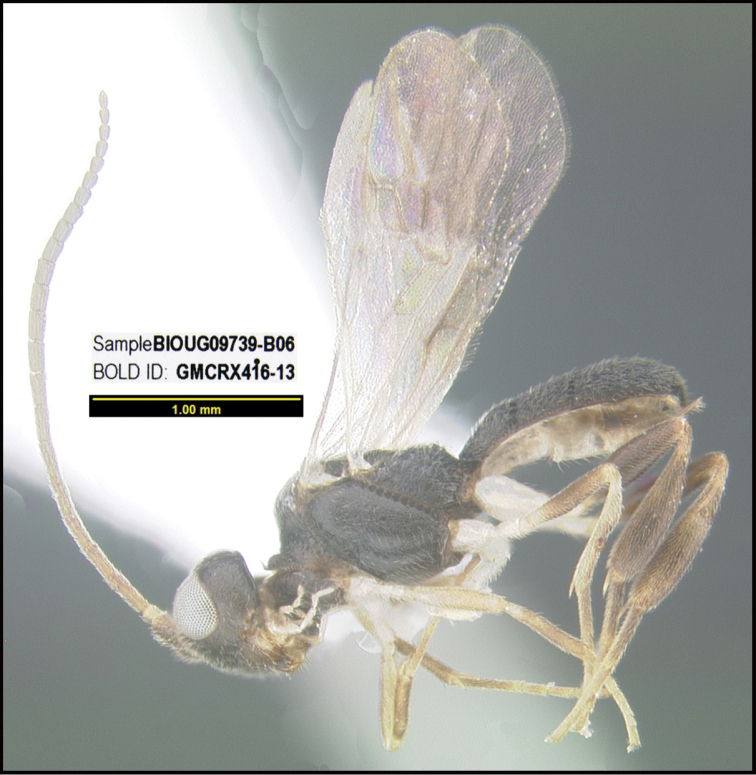
*Phanerotomachristhompsoni*, holotype.

##### Phanerotoma davesmithi

Taxon classificationAnimaliaHymenopteraBraconidae

Sharkey sp. nov.

http://zoobank.org/38A42B33-C0E3-4FD4-894A-74E2655E0CA4

Figure 190

###### Diagnostics.

BOLD:ADB4617. Consensus barcode. GTTTTATATTTTATATTTGGAATATATTCAGGAATGTTAGGTTTATCATTGAGATTATTAATTCGTTTAGAATTGAGTGTTTTAGGYTCTATATTGGGTAATGATCAAATTTATAATATAATTGTTACTATTCATGCTTTTATTATAATTTTTTTTATAGTTATACCAATTATAATTGGTGGTTTTGGAAATTGATTAGTGCCTTTAATATTAGGTGGTCCTGATATAAGTTTTCCTCGTATAAATAATATAAGATTTTGATTATTAATTCCTTCATTAATATTATTATGTTTATCTAGATTTATTAATATAGGAGCTGGAACAGGATGAACYGTTTATCCTCCATTATCTTTAATAATTGGACATGGTGGAATTTCTGTAGATTTAGTAATTTTTTCGTTACATTTAGCAGGAATTTCTTCAATTTTAGGTGCTATTAATTTTATTAGAACTGTTTTAAATATATGATTTAAATTAAAAATAATAGATAAATTATCTTTATTTATTTGATCAGTAATTATTACTGCTTTATTATTGTTATTATCTTTACCTGTATTAGCAGGTGCAATTACTATATTATTAATGGATCGAAATTTAAAT.

###### Holotype ♀.

Guanacaste, Pailas Dos, PL12-6, 10.7637, -85.333, 853 meters, 05‒12/vi/2014, Malaise trap PL12-6A. Depository: CNC.

***Host data*.** None.

***Holotype voucher code*s**: BIOUG29436-A09.

###### Paratypes.

All Malaise-trapped. BIOUG29437-F01, BIOUG29584-C07, BIOUG29964-B02, BIOUG29992-B03, BIOUG29999-D12, BIOUG30041-B07, BIOUG30497-D05, BIOUG30507-F05. Depository: CNC.

###### Etymology.

*Phanerotomadavesmithi* is named to honor Dr. Dave Smith of the Smithsonian Institution for his enthusiastic taxonomic treatments of ACG sawflies.

**Figure 190.**
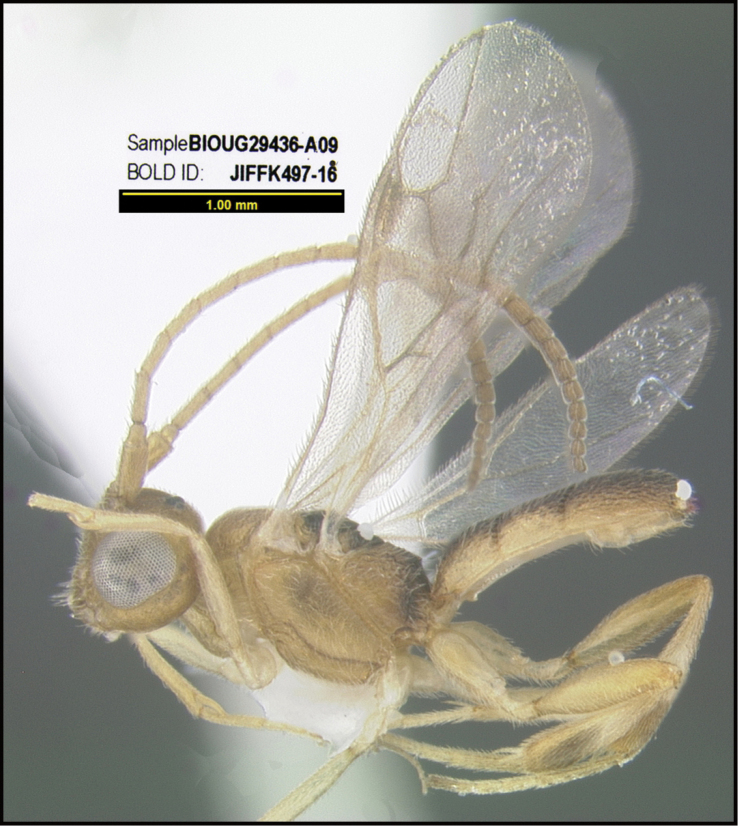
*Phanerotomadavesmithi*, holotype.

##### Phanerotoma davidduthiei

Taxon classificationAnimaliaHymenopteraBraconidae

Sharkey sp. nov.

http://zoobank.org/75606537-A4EC-467F-9DAE-F5C36EB5A8C7

Figure 191

###### Diagnostics.

BOLD:ACL9696. Consensus barcode. TATTTTATATTTTATTTTTGGTATATATTCAGGTATATTAGGATTATCTTTAAGAATAATAATTCGTTTAGAATTAAGAGTTTTAGGATCATTATTAGGTAATGATCAAATTTATAATATAATAGTTACTAGACATGCTTTTATTATGATTTTTTTTATAGTTATACCAATTATGATTGGAGGTTTTGGAAATTGATTAATTCCTTTAATATTAGGGGGTCCTGATATAAGTTTTCCTCGTATAAATAATATAAGTTTTTGATTATTAATTCCTTCTTTATTTTTGTTAGTTTTATCAAGATATATTAATATAGGGGCAGGTACAGGTTGAACTGTTTATCCTCCATTATCTTTAATAATAGGTCATGGAGGTATTTCAGTTGATTTAGTAATTTTTTCTTTACATTTAGCTGGAGTTTCTTCAATTTTAGGAGCTATTAATTTTATTACAACAGTTTTAAATATGTGATTTAATATTAAAATATTAGATAAGTTAAGTTTATTTATTTGGTCTGTATTAATTACTGCATTATTATTATTATTATCTTTACCTGTATTAGCAGGGGCAATTACAATATTATTAATAGATCGTAATTTAAATACAAGATTTTTTGATCCTAGAGGAGGTGGGGATCCTGTATTATATCAACATTTATTT.

###### Holotype ♀.

Guanacaste, Sector Santa Rosa, Bosque San Emilio, 10.8438, -85.6138, 300 meters, 11‒18/ii/2013, Malaise trap. Depository: CNC.

***Host data*.** None.

***Holotype voucher code*.**BIOUG10247-H11.

###### Paratypes.

None.

###### Etymology.

*Phanerotomadavidduthiei* is named to honor Dr. David Duthie, retired from the CBD in Montreal, for his enthusiastic support for the action and philosophy of ACG from its very beginning in 1985 through to the present day.

**Figure 191.**
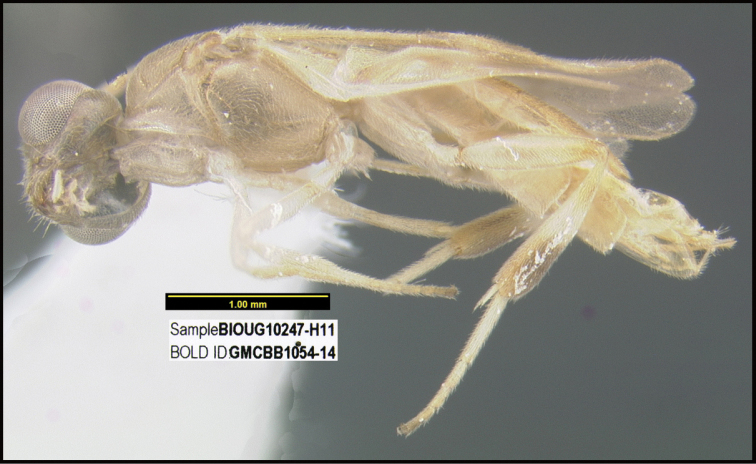
*Phanerotomadavidduthiei*, holotype.

##### Phanerotoma dirksteinkei

Taxon classificationAnimaliaHymenopteraBraconidae

Sharkey sp. nov.

http://zoobank.org/9658754F-D3AD-4858-85DD-059237800CC8

Figure 192

###### Diagnostics.

BOLD:ACA7259. Consensus barcode. GAATTTTRTATTTTTTGTTTGGTATATATTCAGGAATTTTAGGTTTATCTTTAAGRATAATAATTCGTTTAGAATTGAGGGTTTTRGGTTCAATATTAGGTAATGATCAGATTTATAATATGATAGTGACTAGGCATGCATTTATTATAATTTTTTTTATAGTTATACCTATTATAATTGGTGGATTTGGTAATTGGTTGGTTCCTTTAATATTGGGGGGTCCTGATATAAGATTYCCTCGAATAAATAATATAAGATTTTGATTATTAATTCCATCTTTATTTTTATTAATTTTGTCTAGRTTTATTAATATAGGGGCAGGTACTGGTTGAACTRTTTATCCTCCATTATCTTTGATAATGGGTCATGGAGGAATTTCAGTTGATTTAGTAATTTTTTCTTTACATTTAGCTGGTATTTCATCTATTTTAGGAGCTATTAATTTTATTAGTACAGTTTTAAATATATGATTTAGAGTTAAAATATTGGATAAAATAAGTTTATTTATTTGGTCAGTGGTTATTACTGCTTTATTATTATTATTGTCATTACCTGTTTTGGCAGGGGCTATTACAATATTATTAATAGATCGAAATTTAAATACTAGATTTTTTGATCCTAGTGGTGGGGGAGATCCTGTTTTATATCAACATTTATTT.

###### Holotype ♂.

Guanacaste, Sector Santa Rosa, Bosque San Emilio, 10.8438 -85.6138, 300 meters, 11‒18/iii/2013, Malaise trap. Depository: CNC.

***Host data*.** None.

***Holotype voucher code*s**: BIOUG18661-F05.

###### Paratypes.

All Malaise-trapped. BIOUG09442-B05, BIOUG09738-H03, BIOUG09740-A03, BIOUG09740-D01, BIOUG09826-A02, BIOUG09826-A08, BIOUG09827-A01, BIOUG09827-B03, BIOUG10017-A10, BIOUG13945-A09, BIOUG13945-B10, BIOUG13945-D01, BIOUG17520-A12, BIOUG17550-F01, BIOUG17566-G05, BIOUG17607-G02, BIOUG17608-B03, BIOUG17967-C02, BIOUG18290-D05, BIOUG18290-H03, BIOUG18330-D05, BIOUG18499-E11, BIOUG18660-H05, BIOUG18661-A05, BIOUG18661-B01, BIOUG18661-E02, BIOUG18661-F04, BIOUG18664-C06, BIOUG18664-H09, BIOUG18665-C07, BIOUG18665-F09, BIOUG18665-G01, BIOUG18748-E11, BIOUG19974-F05, BIOUG29209-A05, BIOUG29212-G12, BIOUG29320-A11, BIOUG29320-B09, BIOUG29322-E03, BIOUG29322-E04, BIOUG29322-F05, BIOUG29327-C06, BIOUG29327-C10, BIOUG29401-C04, BIOUG29401-C07, BIOUG29403-F09, BIOUG29403-F10, BIOUG29403-F12, BIOUG29490-E11, BIOUG29831-C08, BIOUG30887-G08, BIOUG02644-C08. Depository: CNC.

###### Other material.

Two specimens, one from Munroe County in southern Florida and one from Honduras (BIOUG02644-C08 and BIOUG19974-F05), are in the same BIN in BOLD. They are morphologically similar to Costa Rican specimens and are included as paratypes.

###### Etymology.

*Phanerotomadirksteinkei* is named to honor Dr. Dirk Steinke for his outstanding performance as an educational outreach member of the Centre for Biodiversity Genomics at the University of Guelph.

**Figure 192.**
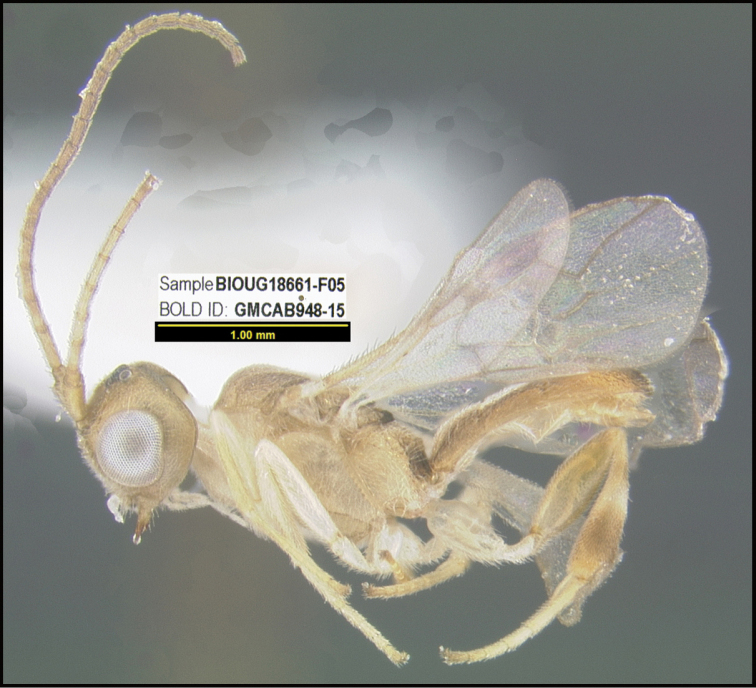
*Phanerotomadirksteinkei*, holotype.

##### Phanerotoma donquickei

Taxon classificationAnimaliaHymenopteraBraconidae

Sharkey sp. nov.

http://zoobank.org/CECC5DC9-3F55-43E8-8692-34EFEDB91E13

Figure 193

###### Diagnostics.

BOLD:ACS0581. Consensus barcode. AATTTTATATTTTTTATTTGGTATGTATTCAGGAGTATTAGGGTTGTCATTAAGAATAATAATTCGTTTAGAATTGAGGGTTTTAGGATCTATATTGGGTAATGATCAAATTTATAATATAATGGTTACTAGACATGCTTTTATTATAATTTTTTTTATGGTTATACCAATTATAATTGGGGGTTTTGGGAATTGGTTAGTTCCTTTAATATTAGGAGGTCCTGATATAAGTTTTCCTCGAATAAATAATATAAGGTTTTGATTATTAATTCCATCATTATTTTTATTAGTTTTATCTAGATATATTAATATAGGAGCAGGAACTGGTTGAACTGTATACCCTCCTTTATCTTTAATAATGGGTCATGGTGGAATTTCGGTTGATTTAGTAATTTTTTCATTACATTTAGCTGGAATTTCTTCAATTTTAGGTGCTATTAATTTTATTAGAACAGTTTTGAATATATGATTTAGAATAAAAATGTTGGATAAATTAAGTTTATTTATTTGATCTGTAGTAATTACTGCTTTATTATTATTATTATCTTTACCTGTATTAGCAGGTGCTATTACTATATTATTAATGGATCGAAATTTAAATACTAGATTTTTTGA----------------.

###### Holotype ♀.

Guanacaste, Sector Santa Rosa, Bosque San Emilio, 10.8438, -85.6138, 300 meters, 18‒25/ii/2013, Malaise trap. Depository: CNC.

***Host data*.** None.

***Holotype voucher code*.**BIOUG18510-C09.

###### Paratypes.

None.

###### Etymology.

*Phanerotomadonquickei* is named to honor Dr Don Quicke of Chulalongkorn University, Pathumwan, Thailand, for his intense interest in the taxonomy of global Braconidae wasp biodiversity.

**Figure 193.**
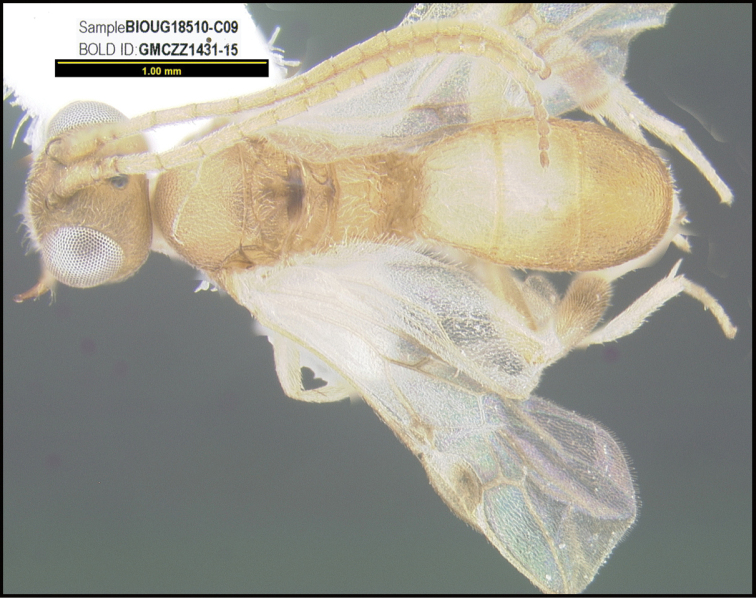
*Phanerotomadonquickei*, holotype.

##### Phanerotoma duniagarciae

Taxon classificationAnimaliaHymenopteraBraconidae

Sharkey sp. nov.

http://zoobank.org/032FDC28-E2C8-456B-8A84-348431130AC7

Figure 194

###### Diagnostics.

BOLD:ADB2375. Consensus barcode. ATTTTATATTTTTTATTTGGAATATATTCAGGCATTATAGGTTTATCTTTAAGAATAATAATTCGTTTGGAATTAAGAGTTTTAGGTTCTATGTTAGGAAATGATCAAATTTATAATATATTTGTTACAAGTCATGCTTTTATTATAATTTTTTTTATAGTAATACCTATTATAATTGGAGGGTTTGGGAATTGATTAGTTCCATTAATATTAGGTGGGCCTGATATAAGTTTTCCTCGAATAAATAATATAAGATTTTGATTATTAGTTCCTTCTTTATTATTATTAATTTTGTCTAGATATACTAATATAGGAGCAGGGACTGGTTGAACAGTGTATCCTCCTTTATCTTTAATAATGGGTCATGGAGGAATTTCTGTTGATTTAGTAATTTTTTCTTTACATTTAGCTGGTATTTCTTCTATTATGGGGGCTATTAATTTTATTAGAACAATTTTAAATATATGATTTGATATTAAAATATTAGATAAATTGAGATTATTTATTTGATCTGTAATAATTACTGCTTTATTATTACTGTTATCATTACCTGTATTAGCTGGAGCTATTACAATATTATTAATGGAT------------------------------------------------.

###### Holotype ♀.

Guanacaste, Pailas Dos, PL12-3, 10.7631 -85.3344, 820 meters, 19‒26/xii/2013, Malaise trap PL12-3A. Depository: CNC.

***Host data*.** None.

***Holotype voucher code*.**BIOUG29456-B07.

###### Paratypes.

None.

###### Etymology.

*Phanerotomaduniagarciae* is named to honor Sra. Dunia Garcia of GDFCF and ACG for her many years as a dedicated inventory parataxonomist for ACG.

**Figure 194.**
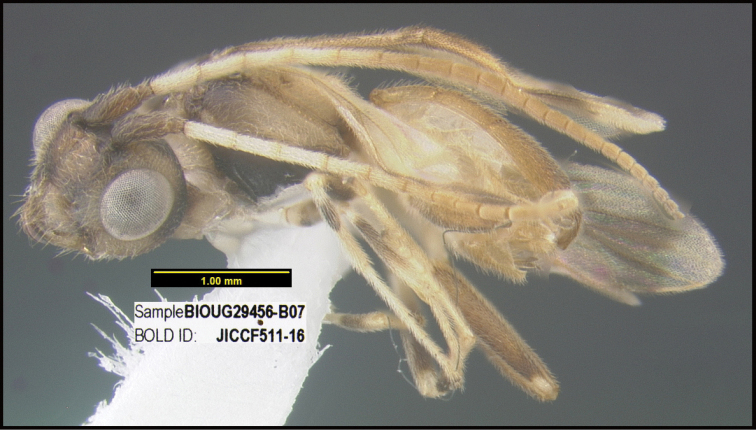
*Phanerotomaduniagarciae*, holotype.

##### Phanerotoma duvalierbricenoi

Taxon classificationAnimaliaHymenopteraBraconidae

Sharkey sp. nov.

http://zoobank.org/CC1C59BE-D90E-4E10-A290-1DB8D4089FED

Figure 195

###### Diagnostics.

BOLD:ADD5784. Consensus barcode. ATTTTATATTTTTTATTTGGTATATATTCAGGCATTATAGGGTTGTCTTTAAGAATGATAATTCGTTTGGAATTAAGAGTATTAGGTTCAATATTAGGAAATGATCAAATTTATAATATATTTGTTACAAGACATGCTTTTATTATAATTTTTTTTATAGTTATACCAATTATAATTGGAGGATTTGGTAATTGGTTAGTACCATTAATATTAGGTGGACCTGATATAAGTTTTCCTCGAATAAATAATATAAGATTTTGATTATTAATTCCTTCTTTATTTTTATTAATTTTGTCTAGTTATACAAATATAGGTGCAGGTACTGGTTGAACAGTTTATCCACCTTTATCTTTAATAATAGGTCATGGGGGAATTTCTGTTGATTTAGTAATTTTTTCTTTACATTTAGCTGGAATTTCTTCTATTATAGGGGCAATTAATTTTATTAGAACTGTTTTAAATATATGATTTAGAATAAAAATGTTAGATAAGTTAAGTTTATTTATTTGATCAGTTGTTATTACTGCTTTGTTATTGTTATTATCTTTACCTGTATTAGCTGGAGCTATTACAATATTATTA---------------------------------------------.

###### Holotype ♀.

Guanacaste, Pailas Dos, PL12-2, 10.7634, -85.335, 824 meters, 15‒22/v/2014, Malaise trap PL12-2A. Depository: CNC.

***Host data*.** None.

***Holotype voucher code*.**BIOUG30941-E12.

###### Paratypes.

None.

###### Etymology.

*Phanerotomaduvalierbricenoi* is named to honor Sr. Duvalier Briceño of GDFCF and ACG for his many years as a dedicated inventory parataxonomist for ACG.

**Figure 195.**
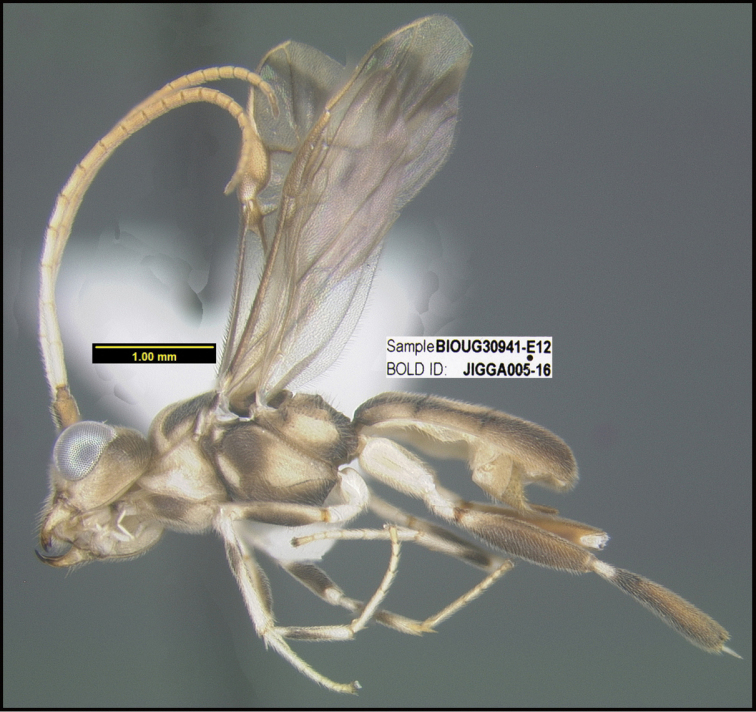
*Phanerotomaduvalierbricenoi*, holotype.

##### Phanerotoma eddysanchezi

Taxon classificationAnimaliaHymenopteraBraconidae

Sharkey sp. nov.

http://zoobank.org/8934F0A7-9B84-472E-A60D-0903EB832A10

Figure 196

###### Diagnostics.

BOLD:AAW3563. Consensus barcode. AATTTTATATTTTTTATTTGGAATATATTCTGGRGTTATTGGTTTATCATTAAGTATAATAATTCGTTTAGAATTAAGAGTATTAGGATCTATATTAGGAAATGATCAAATTTATAATATATTTGTTACTAGACATGCTTTTATTATAATTTTTTTTATGGTTATACCTATTATAATTGGTGGTTTTGGAAATTGATTGGTTCCTTTAATGTTAGGGGGTCCTGATATAAGTTTCCCTCGAATAAATAATATRAGATTTTGRTTATTAGTTCCTTCTTTAATRTTATTAATTTTGTCTAGTTATACTAATATAGGGGCRGGTACTGGATGAACTGTTTATCCTCCTTTATCTTTAATAATAGGTCATGGGGGTATTTCTGTTGATTTAGTAATTTTTTCTTTACATTTAGCTGGTATTTCTTCAATTATAGGAGCTATTAATTTTATTAGTACAGTTTTAAATATATGATTTAGAATTAAAATATTAGATAAATTAAGATTATTTATTTGGTCAGTTGTAATTACTGCTTTATTATTATTATTATCTTTACCTGTATTAGCTGGGGCTATTACAATACTATTAATAGATCGAAATTTAAATACTAGGTTTTTTGATCCAAGAGGTGGGGGTGATCCWG-TTTATATCAGCATTTATTT.

###### Holotype ♀.

Guanacaste, Sector Mundo Nuevo, Estación La Perla, 10.76737, -85.43313, 325 meters, caterpillar collection date: 09/i/2011, wasp eclosion date: 23/ii/2011. Depository: CNC.

***Host data*.** Gregarious parasitoid on *Oryctometopiafossulatella* (Pyralidae) feeding on *Semialariummexicanum* (Celastraceae).

***Caterpillar and holotype voucher codes*.** 11-SRNP-55189, DHJPAR0045461, two parasitoids, both with this voucher code, emerged from the host caterpillar.

###### Paratypes.

Host = *Oryctometopiafossulatella*: BIOUG18005-A02, DHJPAR0034287. Depository: CNC.

###### Etymology.

*Phanerotomaeddysanchezi* is named to honor Sr. Eddy Sanchez for his generous and supportive understanding of the integration of ACG conservation goals with the ICE goals of constructing a geothermal development site on the margin of ACG as a Natural World Heritage Site.

**Figure 196.**
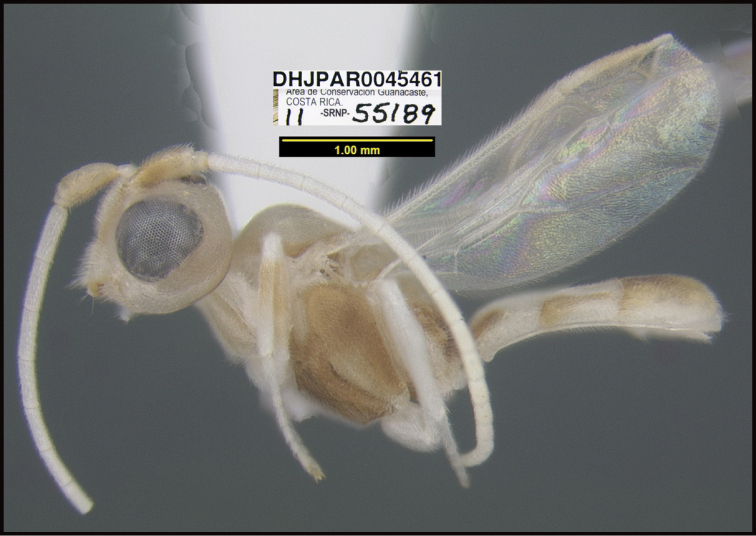
*Phanerotomaeddysanchezi*, holotype.

##### Phanerotoma eldarayae

Taxon classificationAnimaliaHymenopteraBraconidae

Sharkey sp. nov.

http://zoobank.org/94F7A958-A610-44ED-BE85-294BE0E008B3

Figure 197

###### Diagnostics.

BOLD:ACJ4087. Consensus barcode. TGTTTTATATTTTTTGTTTGGAATATATTCTGGAATTTTAGGTTTATCTTTAAGTATAATAATTCGTTTAGAATTA---AGAGTTTTAGGATCAATATTAGGTAATGATCAAATTTATAATATAATAGTTACTAGACATGCTTTTATTATAATTTTTTTTATAGTTATACCTATTATAATTGGAGGATTTGGGAATTGGTTAGTTCCTTTAATATTAGGTGGACCTGATATAAGATTTCCTCGAATAAATAATATAAGATTTTGGTTATTAGTTCCTTCTTTATTTTTATTAATTATATCTAGATATATTAATATAGGTGTAGGAACAGGATGAACTGTTTATCCTCCATTATCTTTAATAATAGGTCATGGTGGAATTTCAGTAGATTTAGTTATTTTTTCTTTACATTTAGCTGGAATTTCTTCAATTTTAGGTGCAATTAATTTTATTAGAACAGTAATAAATATATGATTTAGAATAAAAATATTAGATAAATTAAGATTATTTATTTGATCTGTAGTTATTACTGCTTTATTGTTATTATTATCTTTACCTGTTTTAGCAGGTGCTATTACTATATTATTAATAGATCGAAATTTAAATACAAGATTTTTTGATCCAAGAGGAGGTGGAGATCCTGTATTGTATCAACATTTATTT.

###### Holotype ♀.

Guanacaste, Sector Santa Rosa, Bosque San Emilio, 10.8438, -85.6138, 300 meters, 07/v/2012, Malaise trap. Depository: CNC.

***Host data*.** None.

***Holotype voucher code*.**BIOUG07919-D03.

###### Paratypes.

None.

###### Etymology.

*Phanerotomaeldarayae* is named to honor Sra. Elda Araya of GDFCF and ACG for her many years as a dedicated inventory parataxonomist for ACG.

**Figure 197.**
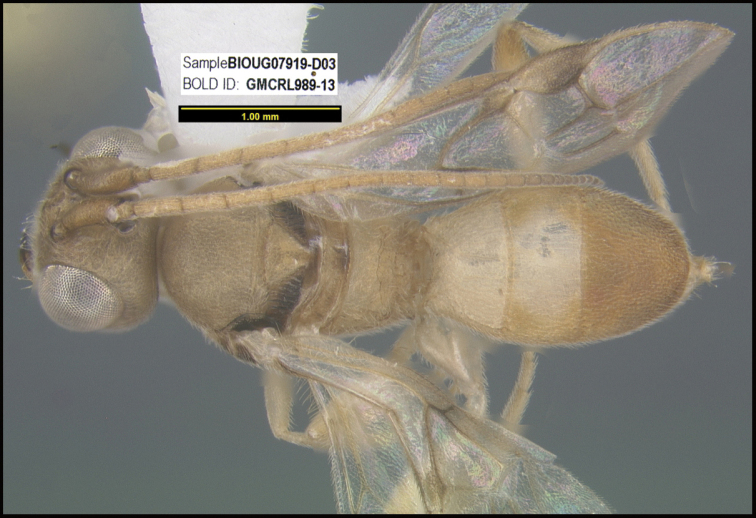
*Phanerotomaeldarayae*, holotype.

##### Phanerotoma eliethcantillanoae

Taxon classificationAnimaliaHymenopteraBraconidae

Sharkey sp. nov.

http://zoobank.org/2026BB1D-CAE5-413F-A00D-691252954ACB

Figure 198

###### Diagnostics.

BOLD:ADE1674. Consensus barcode. ---------------------------TTCTGGAGTTTTAGGTTTGTCATTAAGAATAATAATTCGTTTAGAATTAAGTGTTTTAGGATCAATATTAGGTAATGATCAAATTTATAATATAATAGTTACTAGACATGCTTTTGTAATGATTTTTTTTATAGTAATACCTATTATAATTGGAGGTTTTGGGAATTGGTTGGTTCCTTTAATATTAGGTGGACCTGATATAAGATTTCCTCGGATAAATAATATAAGATTTTGATTATTGATTCCTTCTTTATTTTTATTAATTTTGTCTAGTTATATTAATATAGGGGCAGGTACAGGATGGACTGTTTATCCTCCATTATCTTTAATAATAGGTCATGGTGGAATTTCTGTAGATTTAGTTATTTTTTCTTTACATTTAGCTGGAATTTCTTCTATTTTAGGTGCTATTAATTTTATTAGAACAGTTTTTAATATATGATTTAGAATAAAAATATTAGATAAATTAAGATTGTTTATTTGATCTGTAGTAATTACTGCTTTATTATTATTATTATCTTTACCTGTTTTGGCTGGTGCAATTACTATATTATTAATAGATCGAAATTTAAATACAAGGTTTTTTGATCCAAGAGGGGGGGGGGATCCTGTATTATATCAACATTTATTT.

###### Holotype ♀.

Guanacaste, Sector Rincon Rain Forest, Camino Rio Francia, 10.90425, -85.28651, 410 meters, caterpillar collection date: 05/iv/2016, wasp eclosion date: 02/v/2016. Depository: CNC.

***Host data*.** phyBioLep01 BioLep17 (Pyralidae) feeding on *Stryphnodendronmicrostachyum* (Fabaceae).

***Caterpillar and holotype voucher codes*.** 16-SRNP-40256, DHJPAR0059458.

###### Paratypes.

None.

###### Etymology.

*Phanerotomaeliethcantillanoae* is named to honor Sra. Elieth Cantillano of GDFCF and ACG for her many years as a dedicated inventory parataxonomist for ACG.

**Figure 198.**
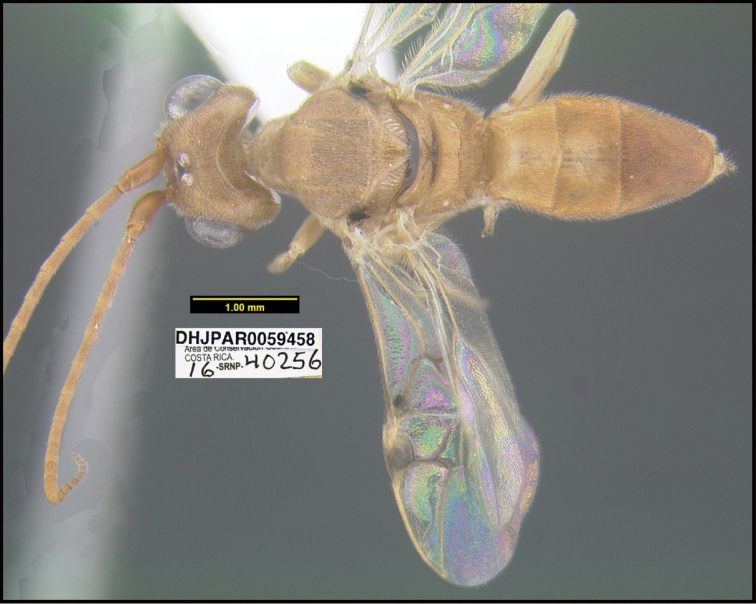
*Phanerotomaeliethcantillanoae*, holotype.

##### Phanerotoma jenopappi

Taxon classificationAnimaliaHymenopteraBraconidae

Sharkey sp. nov.

http://zoobank.org/6C9C9B21-0B9A-45B5-9921-5D2D3A8E54D7

Figure 199

###### Diagnostics.

BOLD:ADY2296. Consensus barcode. TTTATATTTTATATTTGGTTTATATTCTGGATTTTTAGGATTATCTTTAAGTTTAATAATTCGTTTGGAATTAAGAGTTTTAGGAGTTATATTAGGTAATGATCAAATTTATAATATAATAGTTACAGTTCATGCATTTATTATAATTTTTTTTATAGTAATACCAATTATAATTGGAGGATTTGGAAATTGATTAATTCCATTAATGTTAGGAGGTCCTGATATAAGATTTCCTCGAATAAATAATATAAGATTTTGATTATTAATTCCTTCTTTAATTTTAATAATTATTTCAAGATTTATTAATATAGGAGCAGGAACAGGTTGAACAGTTTATCCTCCATTATCGTTAATAATGGGACATGGTGGAATTTCTGTGGATTTAGTAATTTTTTCTTTACATTTGGCAGGAATTTCATCTATTTTAGGGGCAATTAATTTTATTAGTACTGTTATAAATATATGGTTTATAATTAAAATATTAGAAAAATTATCTTTATTTATTTGGTCTGTATTAATTACTGCTTTATTATTATTATTATCTTTACCAGTTTTAGCTGGTGCTATTACAATATTATTAATAGATCGTAATTTAAATACTAGTTTTTTTGATCCTAGTGGAGGTGGGGATCCAATTTTATATCAACATTTATT.

###### Holotype ♂.

Guanacaste, Sector Pailas Dos, PL12-3, 10.7637, -85.3331, 853 meters, Malaise trap, 19/iii/2015. Depository: CNC.

***Host data*.** None.

***Holotype voucher code*.**BIOUG46564-D07.

###### Paratypes.

None.

###### Etymology.

*Phanerotomajenopappi* is named to honor Dr. Jenő Papp (RIP) of Hungary for his significant contributions in understanding the Braconidae of the world, and especially Palearctic Microgastrinae.

**Figure 199.**
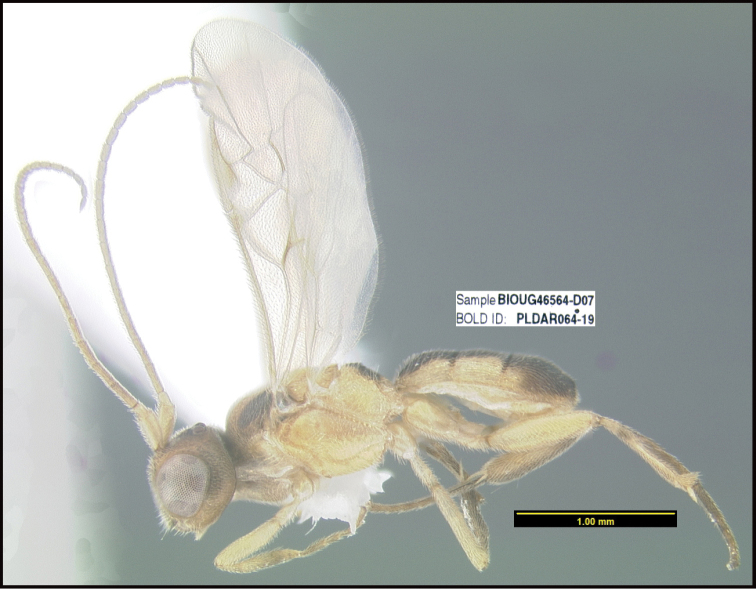
*Phanerotomajenopappi*, holotype.

#### *Pseudophanerotoma* Zettel, 1990

The genus is restricted to the New World and contains 14 described species. Eight species are recorded from the Neotropical region (Kittel 2018) but none from Costa Rica. Two species are recorded from Panama, and it is possible these may be conspecific with one or two of those described below but, judging by the similarly sized revision of *Leptodrepana* by Dadelahi et al. (2018), in which no synonyms were found, synonyms are unlikely.

##### Pseudophanerotoma alanflemingi

Taxon classificationAnimaliaHymenopteraBraconidae

Sharkey sp. nov.

http://zoobank.org/6379A89C-BF25-42B0-ABD9-E3CBD426AA69

Figure 200

###### Diagnostics.

BOLD:ACL3198. Consensus barcode. TATTTTATATTTTATATTTGGTATATATTCTGGTGTGTTAGGTTTGTCTTTAAGTTTATTAATTCGAATAGAATTAAGATGTTTAGGTAGATTATTAGGTAATGATCAAATTTATAATATAGTAGTAACAATTCATGCTTTTATTATAATTTTTTTTATAGTTATACCAGTTATAATTGGTGGATTTGGTAATTGATTAATTCCTTTAATAATTGGTGGGCCTGATATATCTTTTCCTCGTATAAATAATATAAGATTTTGATTATTAATTCCTTCATTATTTTTATTACTTATATCAAGTTTTGTAAATATAGGAGTAGGAACTGGTTGAACAGTTTATCCTCCTTTATCTTTAATGATAGGTCATGGTGGTATATCWGTAGATTTAGCTATTTATTCATTACATTTAGCTGGAATTTCATCAATTATAGGAGCAGTAAATTTTATTACAACAATTTTAAATATATGAAATTTAAATTTTATAGATAAATTATCTTTATTTAGTTGATCTGTTATTATTACAGCTTTATTATTATTATTATCATTACCTGTATTAGCAGGTGCTATTACTATATTATTAAGAGATCGTAATTTAAATACTAGATTTTTTGATCCAAGTGGAGGGGGGGATCCTGTTTTATATCAACATTTATTT.

###### Holotype ♀.

Guanacaste, Pailas Dos, PL12-9, 10.76, -85.3341, 809 meters, 16‒23/i/2014, Malaise trap PL12-9A. Depository: CNC.

***Host data*.** None.

***Holotype voucher code*.**BIOUG28722-F02.

###### Paratype.

Malaise-trapped. BIOUG09826-G03. Depository: CNC.

###### Etymology.

*Pseudophanerotomaalanflemingi* is named to honor Mr. Alan Fleming of the Canadian National Collection and GDFCF for his extreme devotion and production with the taxonomy of ACGTachinidae flies.

**Figure 200.**
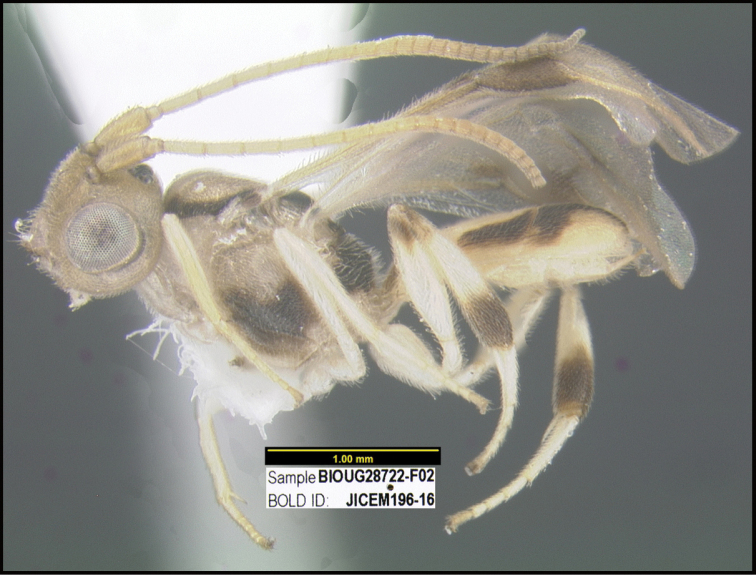
*Pseudophanerotomaalanflemingi*, holotype.

##### Pseudophanerotoma albanjimenezi

Taxon classificationAnimaliaHymenopteraBraconidae

Sharkey sp. nov.

http://zoobank.org/F359A3A2-1703-4F6B-B06E-9B01BA206D7D

Figure 201

###### Diagnostics.

BOLD:AAV8843. Consensus barcode. AGTTTTATATTTTATATTTGGTATATATTCAGGTTTTATTGGTTTATCATTAAGATTGTTAATTCGGATAGAATTAAGATGTTTAGGGGGATTAATAGGTAATGATCAAATTTATAATATAATTGTAACAATTCATGCTTTTGTAATAATTTTTTTTATAGTTATACCAATTATAATTGGAGGATTTGGAAATTGATTAGTTCCATTAATAATTGGTGGACCTGATATATCTTTCCCACGAATAAATAATATAAGATTTTGATTATTAGTTCCTTCCTTATTTTTATTAATATTTTCAAGTTTTGTTAATATAGGGGTTGGGACAGGTTGAACAGTTTAYCCTCCATTATCATCAATTACTGGACATAGGGGTATATCTGTAGATATAGCTATTTATTCTTTACATTTAGCTGGTGCGTCTTCAATTATAGGGGCAGTAAATTTTATTACAACTATTTTAAATATATGAAATTTTAAATTATTAGATAAAATAYCATTATTTGTATGATCAGTTTTTATTACAGCTTTATTATTATTATTATCTTTACCTGTCTTAGCTGGTGCAATTACAATGTTATTAAGAGATCGAAATTTGAATACAAGATTTTTTGATCCAAGAGGTGGAGGTGACCCTATTTTATATCAACATTTATTT.

###### Holotype ♂.

Alajuela, Sector San Cristobal, Tajo Angeles, 10.86472, -85.41531, 540 meters, caterpillar collection date: 22/viii/2011, wasp eclosion date: 09/ix/2011. Depository: CNC.

***Host data*.***Episimusortygia* (Tortricidae) feeding on *Vismiabaccifera* (Hypericaceae).

***Caterpillar and holotype voucher codes*.** 11-SRNP-3257, DHJPAR0048279.

###### Paratypes.

Host = *Episimusortygia*: DHJPAR0040083, DHJPAR0055207. Depository: CNC.

###### Etymology.

*Pseudophanerotomaalbanjimenezi* is named to honor Sr. Alban Jimenez for his intense and dedicated efforts in the ACG Programa de Educación Biologica.

**Figure 201.**
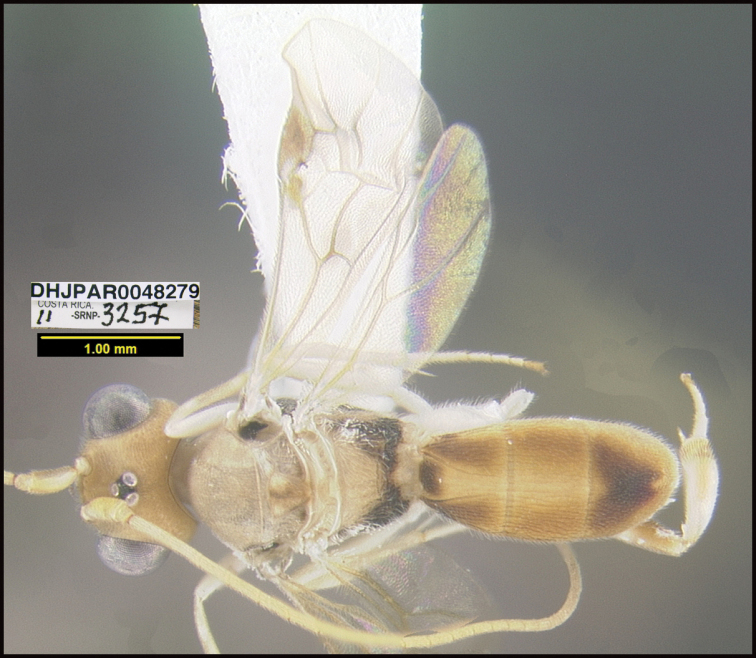
*Pseudophanerotomaalbanjimenezi*, holotype.

##### Pseudophanerotoma alejandromarini

Taxon classificationAnimaliaHymenopteraBraconidae

Sharkey sp. nov.

http://zoobank.org/92069F69-FE11-4471-A3EC-C0C39CD2B39E

Figure 202

###### Diagnostics.

BOLD:ACE1984. Consensus barcode. AATTTTATATTTTATATTTGGAATATATTCTGGWATRTTAGGRTTATCATTAAGTTTATTAATTCGAATAGAGTTAAGATGTTTAGGAAGATTATTAGGAAATGATCAAATTTATAATATAATTGTAACAATTCATGCTTTTATTATAATTTTTTTTATAGTTATACCAGTAATAATTGGTGGRTTTGGAAATTGATTAGTACCTTTAATAATAGGTGGACCAGATATATCTTTYCCACGAATAAATAATATAAGATTTTGATTATTRATTCCTTCTTTAATATTATTAATTTTATCAAGTTTYGTTAATATAGGAGTTGGGACAGGWTGAACAGTTTAYCCWCCTTTATCATTAGTATTAGGRCATGGGGGTATATCAGTTGATATAGCAATTTATTCATTACATTTAGCTGGAATTTCATCAATTATAGGTGCTATTAATTTTATTTCTACAATTATYAATATATGAAGTTTAAAATTATTTGATAAAATATCTTTATTTAGYTGATCAGTAATTATTACAGCTTTTTTATTATTAATGTCATTACCAGTATTAGCTGGTGCAATTACRATATTATTAAGGGATCGAAATTTAAATACAAGATTTTTTGATCCAAGTGGAGGAGGGGATCCAATTTTATACCAACATTTATTT.

###### Holotype ♀.

Guanacaste, Pailas Dos, PL12-9, 10.76, -85.3341, 809 meters, 05‒12/xii/2013, Malaise trap PL12-9A. Depository: CNC.

***Host data*.** None.

***Holotype voucher code*.**BIOUG28669-E05.

###### Paratypes.

All Malaise-trapped. BIOUG04583-E05, BIOUG25658-C12, BIOUG28388-B02, BIOUG29507-E10, BIOUG29577-D11, BIOUG04583-E05, BIOUG25658-C12. Depository: CNC.

###### Note.

Two specimens from Honduras, BIOUG04583-E05 and BIOUG25658-C12, are in the same BIN and are likely conspecific, though they were not examined.

###### Etymology.

*Pseudophanerotomaalejandromarini* is named to honor Dr. Alejandro Marin for his medical attention to ACG GDFCF parataxonomists and administrative support to Sigifredo Marin, the Field Director of GDFCF field projects.

**Figure 202.**
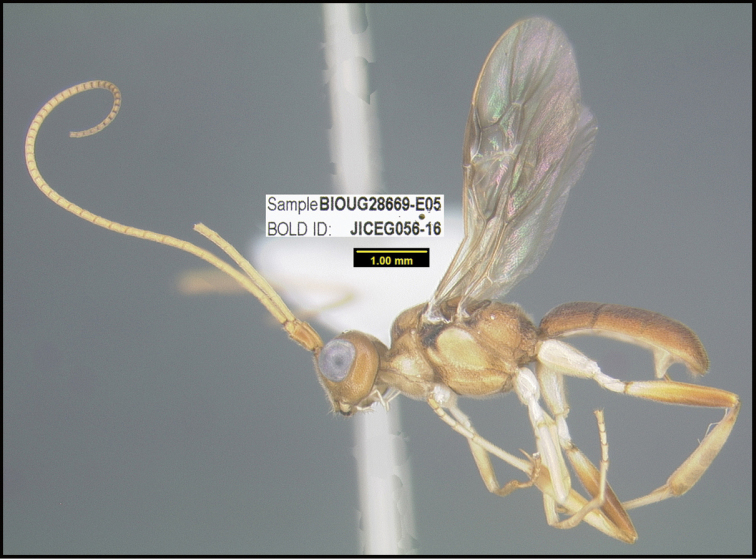
*Pseudophanerotomaalejandromarini*, holotype.

##### Pseudophanerotoma alexsmithi

Taxon classificationAnimaliaHymenopteraBraconidae

Sharkey sp. nov.

http://zoobank.org/20FA4B08-604D-47F9-BF8F-F1271981C134

Figure 203

###### Diagnostics.

BOLD:ACB1516. Consensus barcode. TATTTTATATTTTATATTTGGTATATATTCTGGTATATTAGGGTTATCATTAAGTTTATTAATTCGTATAGAATTAAGGTGTTTAGGTAGGTTATTAGGRAATGATCAAATTTATAATATAATTGTTACAATTCATGCTTTTATTATAATTTTTTTTATAGTTATACCTGTTATAATTGGAGGATTCGGAAATTGATTGGTACCTTTAATAATTGGTGGACCAGATATATCTTTTCCTCGAATAAATAATATAAGATTTTGACTATTAYTACCTTCTTTATTTTTATTAATTATATCAAGATTTGTAAATATAGGAGTTGGTACAGGATGAACTGTTTATCCTCCATTGTCATTAATAATTGGTCATGGTGGTATATCAGTTGATATAGCTATTTATTCATTACATTTAGCTGGTATTTCTTCTATTATAGGAGCAATTAATTTTATTACAACAATTACAAATATATGGAATTTTAAATTGTTTGATAAAATATCYTTGTTTAGATGGTCAGTAATTATTACAGCTTTATTACTTTTATTATCATTACCAGTTTTGGCTGGTGCTATTACAATATTATTAAGAGATCGAAATTTAAATACAAGATTTTTTGATCCAAGGGGTGGTGGGGATCCAATTTTATACCAACATTTGTTT.

###### Holotype ♀.

Alajuela, Sector Rincon Rain Forest, Palomo, 10.96187, -85.28045, 96 meters, caterpillar collection date: 05/iii/2012, wasp eclosion date: 20/iii/2012. Depository: CNC.

***Host data*.***Cosmorrhynchaalbistrigulana* (Tortricidae) feeding on *Dialiumguianense* (Fabaceae).

***Caterpillar and holotype voucher codes*.** 12-SRNP-67407, DHJPAR0049445.

###### Paratypes.

Hosts = *Cosmorrhynchaalbistrigulana*, *Cosmorrhyncha* brown001. DHJPAR0049430, DHJPAR0049441, DHJPAR0049443, DHJPAR0049444, DHJPAR0049450, DHJPAR0049452, DHJPAR0049453, DHJPAR0049456, DHJPAR0049458, DHJPAR0049459, DHJPAR0049460, DHJPAR0049461, DHJPAR0049462, DHJPAR0049463, DHJPAR0049466, DHJPAR0049473, DHJPAR0055194, DHJPAR0055300. Depository: CNC.

###### Etymology.

*Pseudophanerotomaalexsmithi* is named to honor Dr. Alex Smith of the University of Guelph in recognition of his dedicated logistic, taxonomic, and ecological support for all things ACG, with special emphasis on the biology of the volcano elevational gradients in biodiversity.

**Figure 203.**
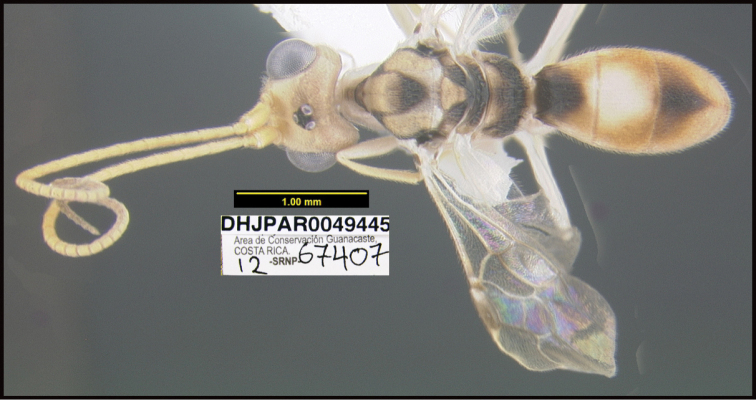
*Pseudophanerotomaalexsmithi*, holotype.

##### Pseudophanerotoma allisonbrownae

Taxon classificationAnimaliaHymenopteraBraconidae

Sharkey sp. nov.

http://zoobank.org/5F165CD0-94C8-46C8-BD94-195675B52147

Figure 204

###### Diagnostics.

BOLD:ACJ2201. Consensus barcode. AGTATTATATTTTATTTTTGGTATTTATGCAGGAGTAGTAGGTTTGTCAATAAGAATAATAATTCGATTAGAATTAAGAGTTTTAGGATCATTAATAGGAAATGATCAAATTTATAATGTATTAGTGACAAGACATGCTTTTGTAATAATTTTTTTTATAGTTATACCAATTATAATTGGAGGATTTGGAAATTGATTAATTCCTTTAATATTAGGAGCTCCTGATATAAGTTTTCCTCGAATAAATAATATAAGATTTTGATTATTGGTACCTTCATTATATTTTTTAATAATATCAATATTTATTAATATAGGAGCAGGAACTGGATGAACAGTATATCCTCCTTTGTCAATAACTATAAGGCATAGAGGGGTTTGTGTAGATTTAGTAATTTTTTCTTTACATTTAGCTGGGATTTCATCAATTTTAGGTTCAATTAATTTTATTAGAACTATTTTTAATATATGAATTGATTATAAATTAATAGATAAATTGAGTTTATTTATTATTTCAGTATTAGTAACTGCTTTTTTATTGTTATTGTCTTTACCAGTATTAGCAGGTGCAATTACAATATTATTAAGAGATCGAAATTTGAATACAAGATTTTTTGATCCTAGAGGAGGTGGTGATCCTGTTTTATATCAACATTTATTT.

###### Holotype ♂.

Guanacaste, Sector Santa Rosa, Bosque San Emilio, 10.8438, -85.6138, 300 meters, 16‒23/iv/2012, Malaise Trap, DH Janzen and W Hallwachs. Depository: CNC.

***Host data*.** None.

***Holotype voucher code*.**BIOUG07616-H01.

###### Paratype.

BIOUG07918-H11.

###### Etymology.

*Pseudophanerotomaallisonbrownae* is named in honor of Allison Brown, formerly of the Centre for Biodiversity Genomics, for her intensely accurate specimen processing of ACG insects for international taxonomists and for her tolerance of their quirks.

**Figure 204.**
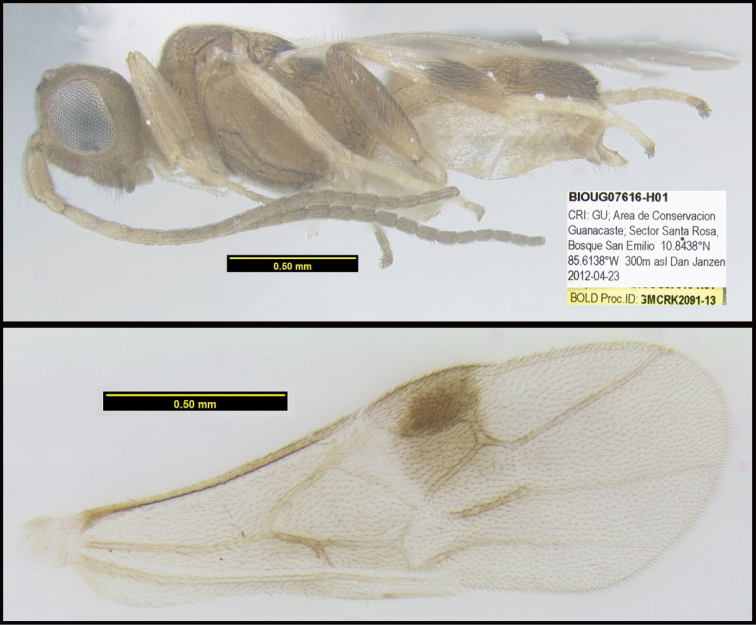
*Pseudophanerotomaallisonbrownae*, holotype.

##### Pseudophanerotoma bobrobbinsi

Taxon classificationAnimaliaHymenopteraBraconidae

Sharkey sp. nov.

http://zoobank.org/900E1AC2-D1AA-4EE7-A6CC-4DE1A3F0F90E

Figure 205

###### Diagnostics.

BOLD:ADB6487. Consensus barcode. TTTTTTTTTGGTATTTATTGTGGAATAATAGGTTTATCATTAAGAATATTAATTCGATTAGAATTAAGAGTTTTAGGGTCTTTATTAGGAAATGATCAAATTTATAATGTAATAGTTACTAGTCATGCATTTATTATAATTTTTTTTATGGTTATACCCATTATGATTGGTGGGTTTGGTAATTGATTAGTACCATTAATATTGGGTGGYCCAGATATAAGATTTCCTCGAATAAATAATATAAGATTTTGATTATTAATTCCTTCATTAATATTATTAATTATATCAAGGTTTGTTAATATAGGTGCAGGAACTGGTTGAACTGTTTATCCACCATTATCATTAATAGTTGGACATAGAGGAATTTCTGTTGATTTAGTAATTTTTTCTTTACATTTAGCTGGTATATCTTCTATTTTAGGGGCTATTAATTTTATTAGGACAGTTTTAAATATATGAATTAGTATAAAAATATTGGATAAATTATCTTTATTTATTTGATCAGTTATAATTACTGCTTTATTATTATTATTATCTTTACCAGTTTTAGCAGGTGCTATTACTATATTATTAAGAGATCGAAATTTAAATACA.

###### Holotype ♀.

Guanacaste, Pailas Dos, PL12-3, 10.7631, -85.3344, 820 meters, 13‒20/ii/2014, Malaise trap PL12-3A. Depository: CNC.

***Host data*.** none.

***Holotype voucher code*.** BIOUG29681-H06.

###### Paratypes.

BIOUG33275-D10, BIOUG33411-D01. Depository: CNC.

###### Etymology.

*Pseudophanerotomabobrobbinsi* is named in honor of Bob Robbins of the Smithsonian Institution for his decades of taxonomic support for ACGLycaenidae and the inclusion of ACG voucher specimens in the National Museum of Natural History in Washington, D.C.

**Figure 205.**
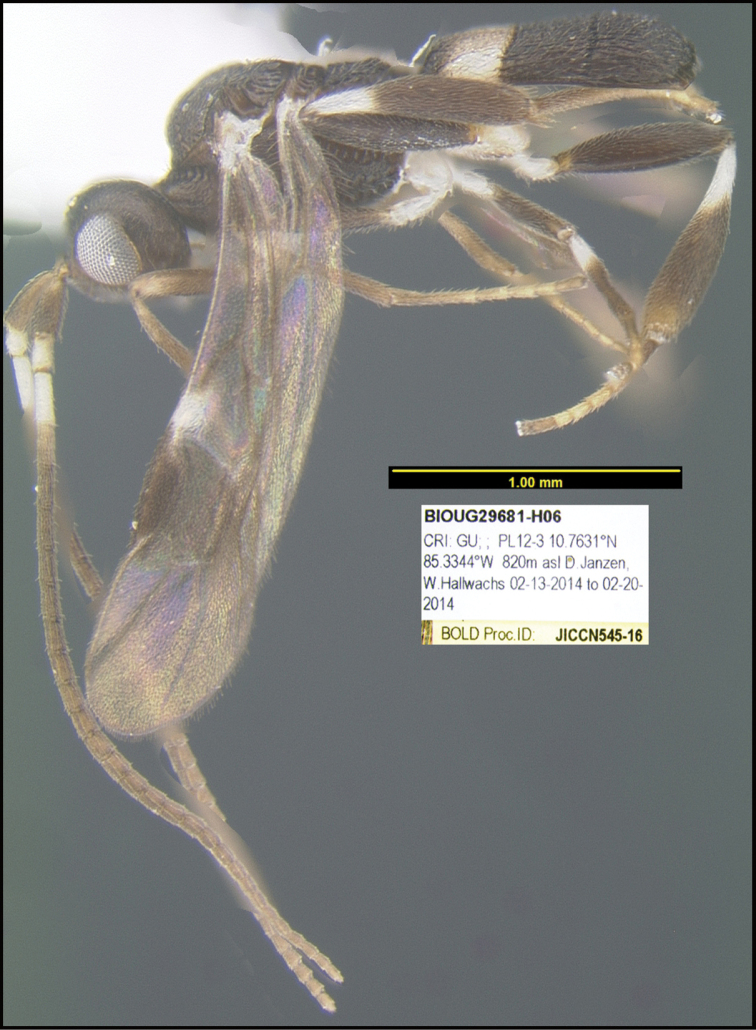
*Pseudophanerotomabobrobbinsi*, holotype.

## Chapter 5: Homolobinae

Homolobinae is a small subfamily containing three genera, two of which occur in the New World. The subfamily is cosmopolitan with several widespread species. The world fauna was revised by van Achterberg (1979). Homolobines are koinobiont endoparasitoids of Lepidoptera, and the rearing records have been reviewed by van Achterberg (1979). To date, all records are of solitary parasitoids, but we report herein the first gregarious species. Ovipositors are short and notched in New World genera and are thought to be used against exposed, nocturnal-feeding hosts. The two New World genera can be distinguished using the key that follows.

### Key to the New World genera of Homolobinae

**Table:** 

1	A. Forewing (RS+M)a curved. AA. Inner side of hind basitarsus with apical comb.	*** Exasticolus ***
–	B. Forewing vein (RS+M)a straight or weakly sinuate. BB. Inner side of hind tibia without a well-developed apical comb.	*** Homolobus ***
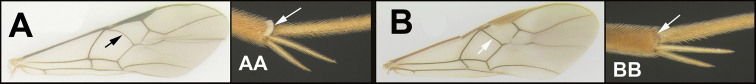		

#### *Exasticolus* van Achterberg, 1979

*Exasticolus* is restricted to the New World, and the most recent key to species is found in López-Martínez et al. (2011). The five new species described here increase the total number of recognized species to eleven. Two previously described species have known hosts, i.e., *E.nigriceps* Enderlein was reared from a larva of *Gloveriaballovi* (Lasiocampidae) (van Achterberg, 1979), and *E.fuscicornis* (Cameron) has been reported as a parasitoid of *Spodopterafrugiperda* (Noctuidae) and *Leuciris* sp. (Geometridae) (Penteado-Dias et al. 2006). To date, all records have been of solitary parasitoids, and we report here the first gregarious species. For the Homolobinae NJ tree, see Suppl. material 4.

##### Exasticolus jennyphillipsae

Taxon classificationAnimaliaHymenopteraBraconidae

Sharkey sp. nov.

http://zoobank.org/CA8EF190-AA53-4B9C-A233-071F1818517E

Figures 206 , 207

###### Diagnostics.

BOLD:AAW1602. Consensus barcode. GTTTTATATTTTTTATTGGGAATTTGATCAGGAATTTTAGGTATATCAATAAGATTTTTAATTCGAATAGAATTAATAATACCAGGTAGATTTTTAAGAAATGATCAAATTTATAATAGTATCGTTACTAGACATGCTTTTATTATAATTTTTTTTTTAGTTATACCAATAATAATTGGAGGATTTGGAAATTGATTAATTCCTTTAATATTAGGATGTCCTGATATGGCTTTCCCTCGAATAAATAATATAAGATTTTGATTATTAATTCCTTCATTAATTTTATTATTATTAAGAAGTATGGTAAATTTAGGAGTAGGTACTGGTTGAACAGTTTATCCTCCTTTGTCATTAAATTTAAGTCATAGGGGTATATCTGTAGATATGGCTATTTTTTCTTTACATTTAGCTGGAATTTCTTCTATTATAGGAGCAATTAATTTTATTACAACAATTTTAAATATACGTTCTAATATAGTTTTTATGGATAAAATTCCTTTATTTGTTTGATCAGTATTTATTACTGTAATTTTACTATTATTGTCTTTACCTGTTTTAGCAGGAGCTATTACTATGTTATTGACTGATCGAAATTTAAATACATCTTTTTTTGATCCTTGTGGTGGAGGGG--------------------------.

Similar to *E.xmatkuilensis* López-Martínez, Delfin-González and van Achterberg 2011 but differing in the ratio of forewing veins r/3RSa, which is 0.9‒1.1 in *E.xmatkuilensis* and 1.3 in this species.

###### Holotype ♀.

Guanacaste, Sector Cacao, Sendero Cima, 10.93328, -85.45729, 1460 meters, collection date: 30/vi/2008, wasp eclosion date: 29/vii/2008. Depository: CNC.

***Host data*.** noto 08-SRNP-35960 (Notodontidae) feeding on *Vacciniumpoasanum* (Ericaceae).

***Caterpillar and holotype voucher codes*.** 08-SRNP-35960, DHJPAR0028118.

###### Paratypes.

None.

###### Etymology.

*Exasticolusjennyphillipsae* is named in honor of Jenny (Eugenie) Phillips of San José, Costa Rica in recognition of her extreme patience in dealing with the foundational bureaucracy of the startup of the BioAlfa process to facilitate bioliteracy in Costa Rica.

**Figure 206.**
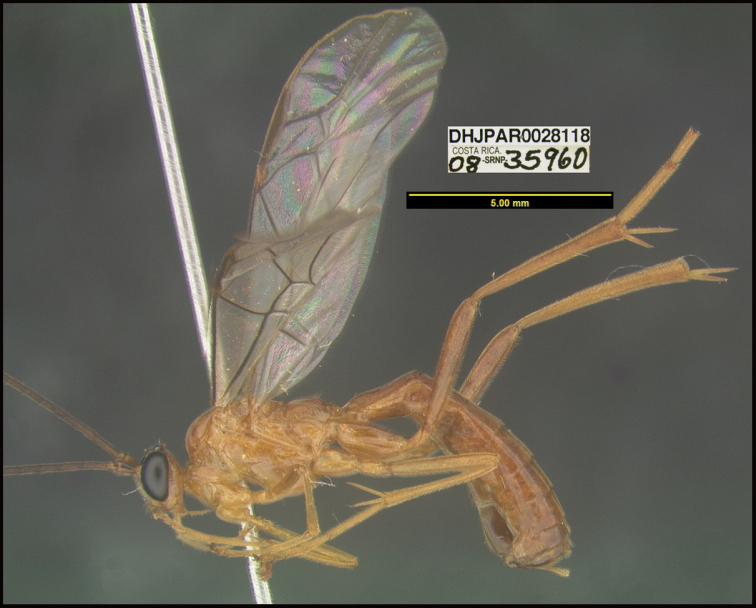
*Exasticolusjennyphillipsae*, holotype.

**Figure 207.**
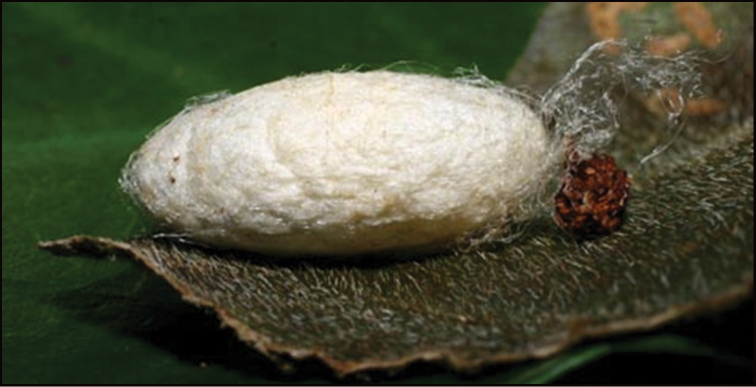
*Exasticolusjennyphillipsae*, cocoon. A snow-white cocoon of *Exasticolusjennyphillipsae* was discovered in the silk and leaf pupal chamber of 08-SRNP-35960, a medium large Notodontidae caterpillar. However, without an associated adult moth, the identity of the host is unknown at present. As the cocoon ages, it turns golden brown in color, but long after the wasp has emerged through the circular hole cut by the wasp in the end of the cocoon.

##### Exasticolus randallgarciai

Taxon classificationAnimaliaHymenopteraBraconidae

Sharkey sp. nov.

http://zoobank.org/BC655BD2-2348-4CF0-B58E-B666FFA8C839

Figure 208

###### Diagnostics.

BOLD:ACM4427. Consensus barcode. TATTTTATATTTTTTATTAGGTATTTGAGCAGGGATTTTAGGAATATCTATGAGAATTTTAATTCGTATAGAATTAATGATACCTGGGAGATTTTTAAGTAATGATCAGATTTATAATAGGATAGTAACTAGACATGCTTTTATTATAATTTTTTTTTTAGTTATACCAATAATAATTGGGGGATTTGGAAATTGATTAATTCCTTTAATGTTAGGATGTCCAGATATAGCTTTCCCTCGTATAAATAATATAAGATTTTGATTATTAATTCCTTCTTTAATTTTATTAATATTAAGAAATATAGTAAATTTAGGAGTAGGTACTGGTTGAACTGTATACCCTCCTTTATCTTTAAATTTAAGTCATAGGGGGATATCTGTAGATATGGCTATTTTTTCTTTACATTTAGCAGGTATTTCTTCAATTATAGGAGCTATTAATTTTATTACAACAATTTTAAATATACGATCAGAAGGAATTTTTATAGATAAAATTCCTTTATTTGTTTGATCTGTTTTTATTACAGTAATTTTATTATTATTATCTTTACCTGTTTTAGCTGGGGCCATTACTATATTATTGACTGATCGAAATTTAAATACATCATTTTTTGATCCTTGTGGTGGGGGAGATCCAATTTTATATCAACATTTATTT. Similar to *E.xmatkuilensis* López-Martínez, Delfin-González & van Achterberg, 2011 but differing in the degree of sculpture on the frons, which is relatively smooth in *E.xmatkuilensis* and with more carinae in this species. The ocelli are much smaller in *E.xmatkuilensis*, and the black color restricted to the stemmaticum in *E.xmatkuilensis* is more extensive in *E.randallgarciai*.

###### Holotype ♀.

Guanacaste, Sector Pitilla, Sendero Nacho, 10.98445, -85.42481, 710 meters, caterpillar collection date: 11/xi/2013, wasp eclosion date: 15/i/2014. Depository: CNC.

***Host data*.***Leptostales* angulataDHJ01 (Geometridae) feeding on *Sabiceapanamensis* (Rubiaceae).

***Caterpillar and holotype voucher codes*.** 13-SRNP-31747, DHJPAR0054461.

###### Paratypes.

None.

###### Etymology.

*Exasticolusrandallgarciai* is named in honor of Randall Garcia of former INBio, San José, Costa Rica, in recognition of his decades of facilitating ACG germination and growth from its beginning in 1986 to the present day.

**Figure 208.**
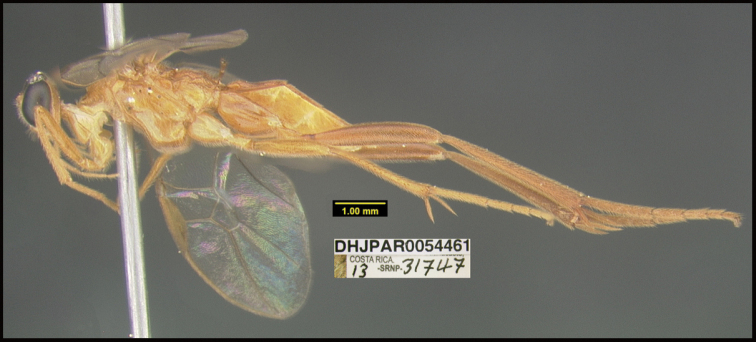
*Exasticolusrandallgarciai*, holotype.

##### Exasticolus robertofernandezi

Taxon classificationAnimaliaHymenopteraBraconidae

Sharkey sp. nov.

http://zoobank.org/FCA0D6FA-E9DA-4511-B2AE-CA87CA7B2E0C

Figures 209 , 210

###### Diagnostics.

BOLD:AAA5869. Consensus barcode. AATTTTATATTTTATTTTGGGTATTTGATCAGGTATTTTAGGTATATCAATAAGATTATTAGTACGAATRGARTTGATAGTTCCTGGAAAATTTTTAGGTAAYGATCAAATTTATAATAGAATTGTTACTGGCATGCATTTTTATAATTTTTTTTTTAGTTATACCAATAATAATTGGTGGATTTGGAAATTGATTAATTCCTTTAATATTAGGATGTCCAGATATAGCTTTTCCTCGTATAAATAATATAAGGTTTTGGTTATTAATTCCTTCTTTATTATTATTAATTATTAGAAGATTAATAAATGTTGGGTTGGAACTGGGTGAACAGTTTATCCTCCGTTATCTTTAAATATAAGTCATAGAGGAATATCTGTAGATATAGCAATTTTTTCATTACATTTGGCAGGAATTTCTTCTATTATAGGAGCAATTAATTTTATTACAACTATTTTAAATATACGTTCAAGTTTAATTTATATAGATAAAATTTCTTTATTAACTTGATCAGTATTTATTACAGTRATTTTATTATTATTATCTTTACCAATTTTAGCAGGAGCTATTACTATATTATTAACTGATCGTAATTTAAATACTTCTTTTTTTGATCCGTGKGGAGGTGGAGATCCAATTTTATATCAACATTTATTT. Close to *Exasticolusnigriceps* (Enderlein, 1920) but differing in the much larger ocelli of *Exasticolusrobertofernandezi*.

###### Holotype ♀.

Guanacaste, Sector Horizontes, Vado Esteron, 10.763, -85.56, 95 meters, caterpillar collection date 20/vii/1999, wasp eclosion 21/viii/1999. Depository: CNC.

***Host data*.** Gregarious parasitoid of *Ormeticasicilia* (Erebidae, Arctiinae) feeding on leaves of *Ingavera* (Fabaceae); 6 wasps eclosed 21/viii/1999 and 4 eclosed 23/viii/1999 from 10 beige wasp cocoons spun next to the cadaver inside the moth cocoon.

***Caterpillar and holotype voucher codes*.** 03-SRNP-9821, DHJPAR0029331.

###### Paratypes.

As recorded for each of the vouchers below, all are hairy, medium-sized Arctiinae (Erebidae) caterpillars. All 15 caterpillar cocoons have gregarious parasitoids that spin their white cocoons inside the caterpillar cocoon after emerging from the prepupal caterpillar. Hosts = *Lophocampa* modestaDHJ01, *Lophocampamaroniensis*, *Ormeticaataenia*, *Ormeticasicilia*, *Vivienneategyra*. DHJPAR0021125, DHJPAR0022194, DHJPAR0029333, DHJPAR0029334, DHJPAR0029335, DHJPAR0021107, DHJPAR0021108, DHJPAR0021124, DHJPAR0021126, DHJPAR0021127, DHJPAR0021128, DHJPAR0021133, DHJPAR0016437, DHJPAR0016438, DHJPAR0016440, DHJPAR0016441, DHJPAR0022099, DHJPAR0022100, DHJPAR0022101, DHJPAR0022102, DHJPAR0022103, DHJPAR0022104, DHJPAR0022105, DHJPAR0022107, DHJPAR0022108, DHJPAR0022109, DHJPAR0022114, DHJPAR0023536, DHJPAR0023537, DHJPAR0016439, DHJPAR0023538, DHJPAR0023539, DHJPAR0023540, DHJPAR0023541, DHJPAR0023542, DHJPAR0023543, DHJPAR0023544, DHJPAR0023545, DHJPAR0023546, DHJPAR0029394, DHJPAR0029395, DHJPAR0029396, DHJPAR0029397, DHJPAR0029399, DHJPAR0037963, DHJPAR0023343, DHJPAR0023344, DHJPAR0023345, DHJPAR0023346, DHJPAR0023347, DHJPAR0023348, DHJPAR0023349, DHJPAR0023350, DHJPAR0036312, DHJPAR0036313, DHJPAR0036314, DHJPAR0036315, DHJPAR0036316, DHJPAR0029330, DHJPAR0029331, DHJPAR0029401, DHJPAR0029402, DHJPAR0055433, DHJPAR0029185, DHJPAR0029327, DHJPAR0029328, DHJPAR0029329. DHJPAR0029400. Depository: CNC.

###### Etymology.

*Exasticolusrobertofernandezi* is named in honor of Roberto Fernandez Ugalde of Cartago, Costa Rica, in recognition of his persistent efforts to plan minimal environmental impacts for geothermal and hydroelectric projects by Costa Rica’s National Electric Company (ICE).

**Figure 209.**
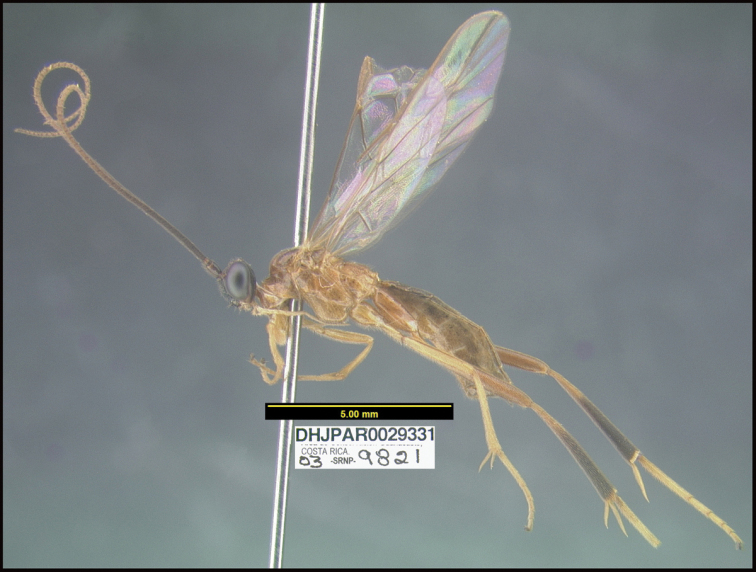
*Exasticolusrobertofernandezi*, holotype.

**Figure 210.**
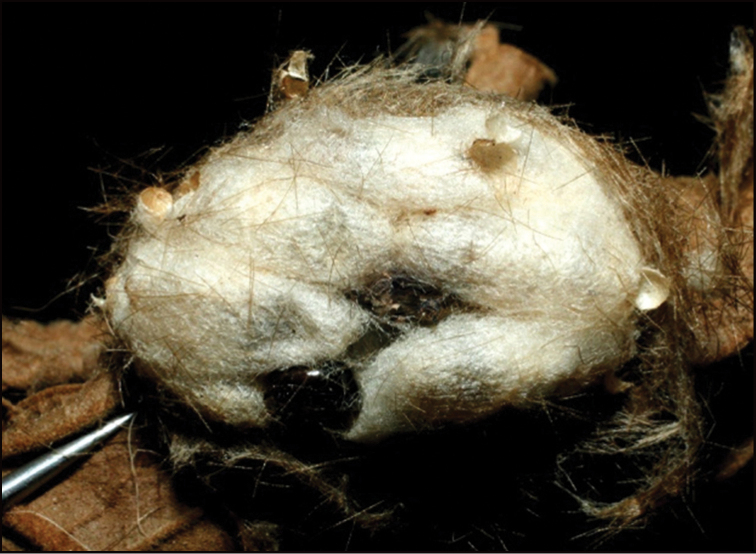
White cocoons of *Exasticolusrobertofernandezi* spun inside of the erebid arctiine moth cocoon (*Lophocampamodesta*), after exiting from the prepupal cadaver (08-SRNP-20003-DHJ462244.jpg).

##### Exasticolus sigifredomarini

Taxon classificationAnimaliaHymenopteraBraconidae

Sharkey sp. nov.

http://zoobank.org/4F3B93FB-F303-4534-BEFC-8D8FD9E1BD01

Figure 211

###### Diagnostics.

BOLD:ACM4428. Consensus barcode. AATTTTATATTTTTTATTGGGAATTTGATCAGGAATTTTAGGTATGTCTATAAGAGTTTTAATTCGTATAGAATTAATAATACCAGGTAGATTTTTAAGTAATGATCAAATTTATAATAGTATTGTTACTAGACATGCTTTTATTATAATTTTTTTTTTAGTTATACCAATAATAATTGGAGGATTTGGAAATTGATTAATTCCTTTAATATTAGGATGTCCTGATATAGCTTTTCCTCGAATAAATAATATAAGATTTTGATTATTAATTCCTTCATTAATTTTATTATTATTAAGAAGTATAGTAAATTTGGGTGTAGGGACAGGTTGAACAGTTTAYCCTCCTTTGTCATTAAATTTAAGTCATAGAGGAATATCTGTGGATATAGCTATTTTTTCTTTACATTTGGCTGGAATTTCATCTATTATAGGAGCAATTAATTTTATTACAACAATTTTAAATATACGTTCTAATATAATTTTTATAGATAAAATTCCTTTATTTGTTTGATCAGTATTTATTACTGTAATTTTATTGTTATTATCTTTACCTGTTTTAGCAGGGGCTATTACTATATTATTGACTGATCGAAATTTAAATACATCTTTTTTTGATCCTTGTGGTGGGGGGGACCCAATTTTGTATCAACATTTATTT. Similar to *E.fuscicornis* (Cameron, 1887) but differing in the ratio of forewing veins r/3RSa, which is 1.4–1.5 in *E.fuscicornis* and 1.1 in *Exasticolussigifredomarini*.

###### Holotype ♀.

Alajuela, Sector Rincon Rain Forest, Sendero Aura, 10.96536, -85.32386, 432 meters, caterpillar collection date: 02/vi/2017, wasp eclosion date: 29/vi/2017. Depository: CNC.

***Host data*.***Malocampamatralis* (Notodontidae) feeding on *Pseudolmediamollis* (Moraceae).

***Caterpillar and holotype voucher codes*.** 17-SRNP-26903 DHJPAR0061471.

###### Paratypes.

Hosts = *Malocampamatralis* and *Sericochroafelderi* (Notodontidae). DHJPAR0029336, DHJPAR0054460. Depository: CNC.

###### Etymology.

*Exasticolussigifredomarini* is named to honor Sigifredo Marin of Liberia, Costa Rica, in emphatic recognition of his decades of effort to support the germination and growth of Area de Conservación Guanacaste and the projects of the Guanacaste Dry Forest Conservation Fund.

**Figure 211.**
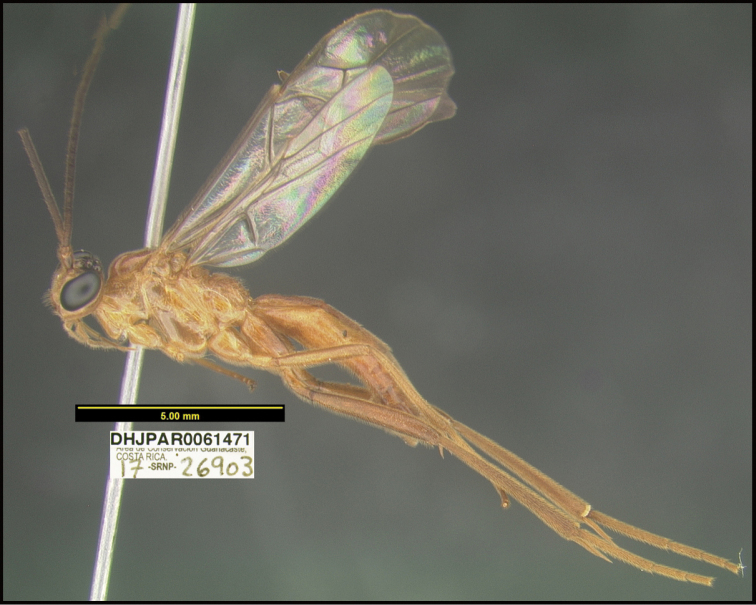
*Exasticolussigifredomarini*, holotype.

##### Exasticolus tomlewinsoni

Taxon classificationAnimaliaHymenopteraBraconidae

Sharkey sp. nov.

http://zoobank.org/71C8EE67-464C-4A33-A0E7-5EA6C7C3FBE1

Figure 212

###### Diagnostics.

BOLD:ADB0948. Consensus barcode. ATTTTGTATTTTTTATTAGGAATTTGATCAGGAATTTTAGGAATATCTATAAGAATTTTAATTCGAATAGAGTTAATAATACCAGGTAGGTTTTTAAGTAATGATCAAATTTATAATAGTATAGTTACTAGACATGCTTTTATTATAATTTTTTTTTTAGTTATACCTATAATAATTGGGGGGTTTGGTAATTGATTAATTCCTTTAATATTAGGATGTCCTGATATAGCTTTCCCTCGTATAAATAATATAAGATTTTGGTTATTAATTCCTTCATTAATTTTATTATTATTAAGAAGTATAGTAAATTTAGGAGTAGGAACTGGTTGAACGGTTTATCCTCCTTTATCATTAAATTTAAATCATGGRGGGATGTCTGTAGATATAGCTATTTTTTCTTTACATTTGGCTGGAATTTCTTCTATTATAGGTGCTATTAATTTTATTACAACAATTTTAAATATACGATCTGATATAATTTTTATAGATAAAATTCCTTTATTTGTTTGATCAGTATTTATTACAGTAATTTTATTATTATTATCTTTACCYGTATTAGCTGGTGCAATTACTATATTATTAACTGATCGA. Similar to *E.xmatkuilensis* López-Martínez, Delfin-González & van Achterberg (2011) but differing in that the basal antennal flagellomeres and the area lateral to the ocelli are black in specimens of *Exasticolustomlewinsoni* and pale in *E.xmatkuilensis*.

###### Holotype ♀.

Guanacaste, Sector Cacao, Pailas Dos, 10.7612, -85.3353, 791 meters, Malaise trap, 5/xii/2013. Depository: CNC.

***Host data*.** None.

***Holotype voucher code*.**BIOUG28731-E04.

###### Paratypes.

BIOUG28713-H02, BIOUG28408-A02, BIOUG29043-D07, BIOUG28817-F06, BIOUG28731-F03. Depository: CNC.

###### Etymology.

*Exasticolustomlewinsoni* is named to acknowledge the participation and guidance of Tom Lewinson at the international NSF-funded planning meeting for the All Taxa Biodiversity Inventory (ATBI) of Terrestrial Systems, and contributing his wisdom to the planning that was the founding of Costa Rica’s national BioAlfa today.

**Figure 212.**
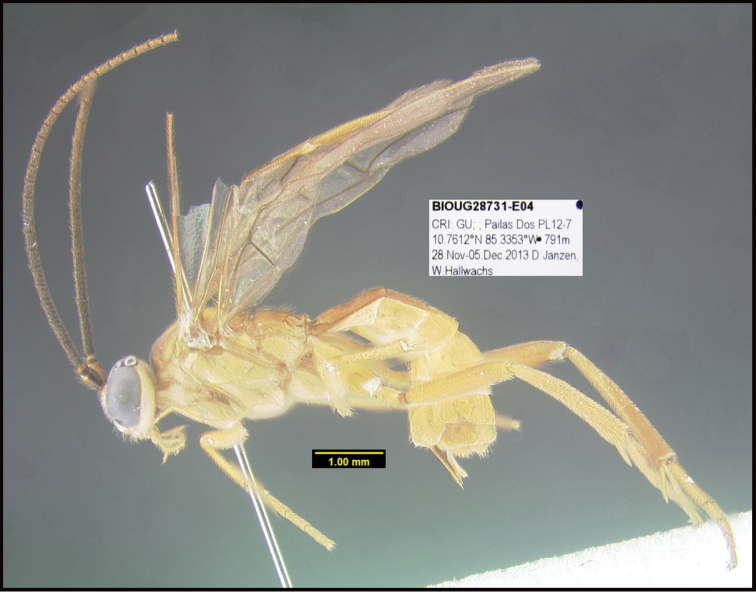
*Exasticolustomlewinsoni*, holotype.

## Chapter 6: Hormiinae

There are only two New World genera of Hormiinae as strictly defined, *Hormius* and *Parahormius*. These can be differentiated with the key below. The subfamily may be distinguished from all other New World braconids by the possession of the following suite of character states: oral cavity cyclostome (= concave dorsally); metasomal terga, except first tergum, membranous medially and weakly sclerotized laterally; occipital carina complete; second submarginal cell 5-sided; stigma normal, not linear; RS vein of forewing reaching margin of wing; and Cu vein of forewing straight, not bent towards the posterior margin of the wing. For the Hormiinae NJ tree, see Suppl. material 5.

### Key to the New World genera of Hormiinae

**Table:** 

1	A. Epicnemial carina present	*** Hormius ***
–	B. Epicnemial carina absent	*** Parahormius ***
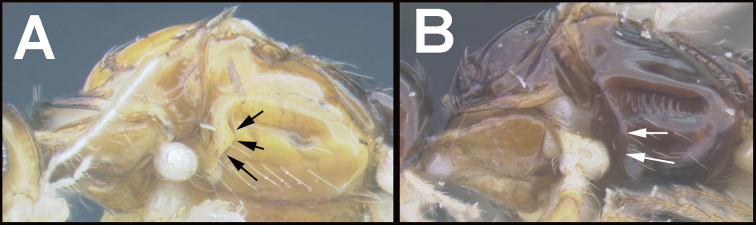		

#### *Hormius* Nees, 1819

No species of *Hormius* was previously recorded from Costa Rica. The few described Neotropical species are mostly from Brazil, Paraguay, and Cuba. Based on distribution, the only likely candidate to be conspecific with any of the species described here is *Hormiusdeletus* Wharton, 1993, and it is distinctive from all listed species here in having the r-m crossvein of the forewing lacking. The few New World species with recorded life-histories are gregarious ectoparasitoids of Gelechiidae, Tortricidae, and possibly Coleophoridae. Here we describe species that are parasitoids of the following families: Hesperiidae, Crambidae, Depressariidae, Gelechiidae, Pyralidae, and Tortricidae. In some of the cases below, there is just one parasite from a given caterpillar host. In these instances, the wasp may be solitary, or it may be that only one of multiple offspring survived.

##### Hormius anamariamongeae

Taxon classificationAnimaliaHymenopteraBraconidae

Sharkey sp. nov.

http://zoobank.org/2633E730-6663-4FB5-80EA-5683ED0EB740

Figure 213

###### Diagnostics.

BOLD:AAW4506. Consensus barcode. AATTTTATATTTTTTATTTGGAATATGGGCTGGTATAGTTGGTTTATCAATAAGATTAATTATTCGTTTGGAATTAGGAATACCAGGGAGTTTATTAGGTAATGATCAGATTTATAATAGAATAGTGACTGCTCATGCATTTATTATGATTTTTTTTATAGTTATACCAATTATAATTGGGGGGTTTGGAAATTGATTAATTCCTTTAATATTAGGGTCTCCTGATATAGCCTTTCCTCGAATAAATAATATAAGATTTTGATTATTAGTTCCTTCATTAATATTATTAGTTTTTAGTGGTGTTTTAAATATTGGGGTTGGTACAGGGTGGACTATATATCCTCCTTTATCTTCTTTAATTGGTCATAGAGGAATTTCAGTAGATTTAGCTATTTTTTCTTTACATTTAGCTGGTATTTCTTCAATTATAGGGGCTATTAATTTTATTTCTACAATTTTTAATATAAGTTTAAATTATATGAAAATAGATCAAATTAATTTATTAATTTGATCTATTTTAATTACTGCAATTTTATTATTATTATCTCTTCCTGTTTTAGCAGGGGCTATTACTATATTATTAACTGATCGTAATTTAAATACAACATTTTTTGATTTTTCTGGAGGTGGGGATCCTATTTTATTTCAACATTTATTT.

###### Holotype ♀.

Alajuela, Sector Rincon Rain Forest, Jacobo, 10.94076, -85.3177, 461 meters, caterpillar collection date: 09/i/2011, wasp eclosion date: 23/i/2011, one of seven wasps that emerged from the same host caterpillar. Depository: CNC.

***Host data*.** Gregarious parasitoid of *Rhectocraspeda* Solis05 (Crambidae) feeding on *Solanumjamaicense* (Solanaceae).

***Caterpillar and holotype voucher codes*.** 11-SRNP-69067, DHJPAR0042073.

###### Paratypes.

Hosts = *Rhectocraspeda* Solis05, and *Rhectocraspediaperiusalis*. 6 specimens, same data as holotype, and DHJPAR0056686. Depository: CNC.

###### Etymology.

*Hormiusanamariamongeae* is named in honor of Ana Maria Monge in recognition of her enthusiastic participation in the BioAlfa process for Costa Rica, as a government employee.

**Figure 213.**
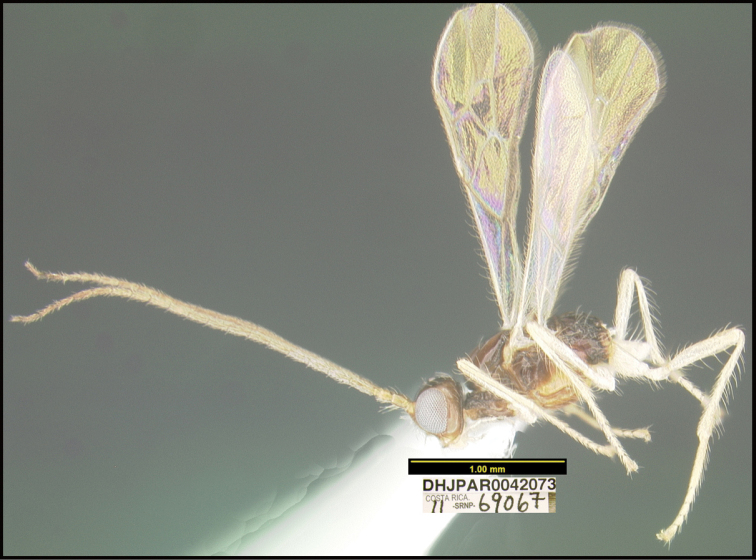
*Hormiusanamariamongeae* holotype.

##### Hormius angelsolisi

Taxon classificationAnimaliaHymenopteraBraconidae

Sharkey sp. nov.

http://zoobank.org/B44959DA-F723-4759-B4BD-6D4213EF1609

Figure 214

###### Diagnostics.

BOLD:AAA5364. Consensus barcode. ATTATTATATTTTTTATTTGGGATATGATCTGGTATAATTGGTTTATCTATAAGTTTAATTATTCGTTTAGAATTAGGAATACCTGGTAGATTATTGAGTAATGATCAAATTTATAATAGTATAGTAACCGCTCATGCTTTTATTATAATTTTTTTTATAGTTATACCTATTATAATTGGGGGATTTGGAAATTGATTAGTTCCTTTAATATTAGGATCTCCTGATATGGCTTTTCCTCGAATAAATAATATAAGGTTTTGATTATTAATCCCTTCTTTAATATTATTAATTTTTAGAAGAATTTTAAATGTAGGAGTAGGGACGGGTTGAACTATATATCCACCTTTATCTTCTTTAATTGGTCATGGGGGAATTTCTGTTGATTTAGCTATTTTTTCTTTACATTTGGCTGGTATTTCTTCAATTATAGGAGCTATTAATTTTATTTCAACTATTTTAAATATAAATTTATATAATATAAAATTGGATCAAATTAATTTATTAATTTGATCTATTTTAATTACTGCTATTTTATTATTATTATCTTTACCTGTTTTAGCAGGGGCTATTACTATATTATTAACTGATCGTAATTTAAATACAACTTTTTTTGATTTTTCTGGTGGTGGGGATCCTATTTTATTTCAACATTTATTT.

###### Holotype ♀.

Guanacaste Sector Pitilla, Pasmompa, 11.01926, -85.40997, 440 meters, caterpillar collection date: 10/viii/2005, wasp eclosion date: 28/viii/2005, one of 27 wasps that emerged from the same host caterpillar. Depository: CNC.

***Host data*.***Parphorusdecora* (Hesperiidae) feeding on leaves of *Olyralatifolia* (Poaceae).

***Caterpillar and holotype voucher codes*.** 05-SRNP-33249, DHJPAR0029016.

###### Paratypes.

Host = *Parphorusdecora*: 26 specimens with same data as holotype, and DHJPAR0029009, DHJPAR0038938, DHJPAR0029007, DHJPAR0060215. Depository: CNC.

###### Etymology.

*Hormiusangelsolisi* is named in honor of Angel Solis in recognition of his decades of diligent effort with the taxonomy of ACG and Costa Rican Coleoptera.

**Figure 214.**
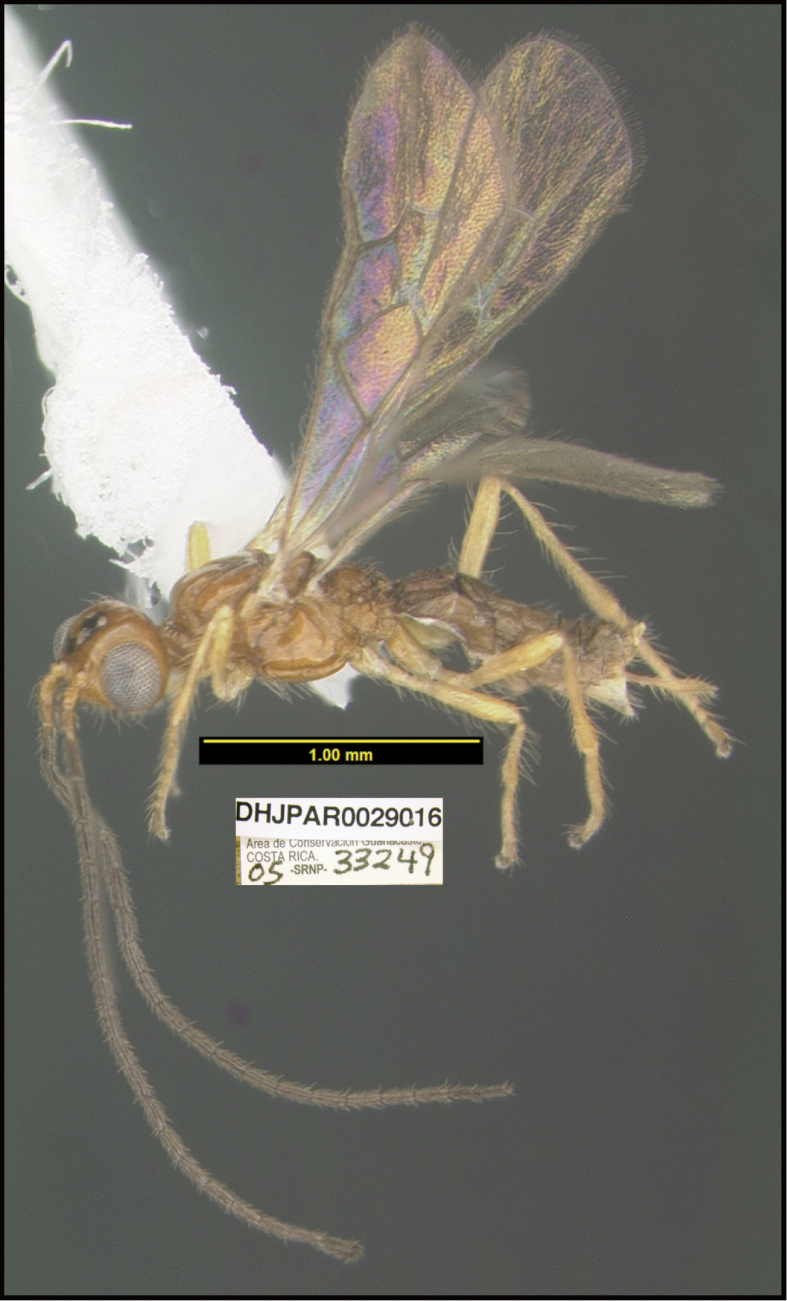
*Hormiusangelsolisi* holotype.

##### Hormius anniapicadoae

Taxon classificationAnimaliaHymenopteraBraconidae

Sharkey sp. nov.

http://zoobank.org/02BDD1DC-3BAD-4A59-B4E5-5538E7178820

Figures 215 , 216

###### Diagnostics.

BOLD:AAU2092. Consensus barcode. TGGTCTGGGATAATTGGGCTTTCAATAAGATTAATTATTCGAATAGAATTAGGTATACCTGGAAGTTTATTAGGTAATGATCAAATTTATAATAGTATTGTTACTGCTCATGCTTTTATTATAATTTTTTTTATAGTTATACCAATTATAATTGGAGGATTTGGAAATTGATTAATTCCTTTAATATTAGGGTCTCCTGATATAGCTTTTCCACGAATAAATAATATAAGGTTTTGATTATTGATTCCTTCTTTATTTTTATTAATTTTTAGGGGTTTATTAAATGTTGGGGTAGGAACGGGTTGAACAATATATCCCCCCTTGTCTTCTTTAATTGGTCATAATGGTGTATCTGTTGATTTAGCTATTTTTTCTTTGCATTTAGCTGGTATTTCTTCTATTATAGGTGCTGTAAATTTTATTTCAACTATTTTTAATATAAATTTATATAATATAAAATTTGATCAAATTAATTTATTAATTTGATCAATTTTGATTACTGCATTATTATTATTATTGTCTTTACCTGTATTAGCTGGAGCTATTACAATATTATTAACTGATCGTAATTTAAATACAACATTTTTTGATTTTTCTGGTGGTGGGGACCCAATTTTATTTCAACATTTATTT.

###### Holotype ♀.

Alajuela, Sector San Cristobal. Sendero Huerta, 10.93050, -85.37223, 527 meters, caterpillar collection date: 16/viii/2009, wasp eclosion date: 29/viii/2009, one of five wasps that emerged from the host caterpillar. Depository: CNC.

***Host data*.** Gregarious parasitoid of *Amorbiaproductana* (Tortricidae) feeding on leaves of *Hyptisobtusiflora* (Lamiaceae).

***Caterpillar and holotype voucher codes*.** 09-SRNP-4249, DHJPAR0040024.

###### Paratypes.

4 specimens, same data as holotype. Depository: CNC.

###### Etymology.

*Hormiusanniapicadoae* is named in honor of Annia Picado of San José, Costa Rica for her long years sorting Malaise trap specimens, identifying Diptera, and now dissecting genitalia of Microlepidoptera in the former INBio collections.

**Figure 215.**
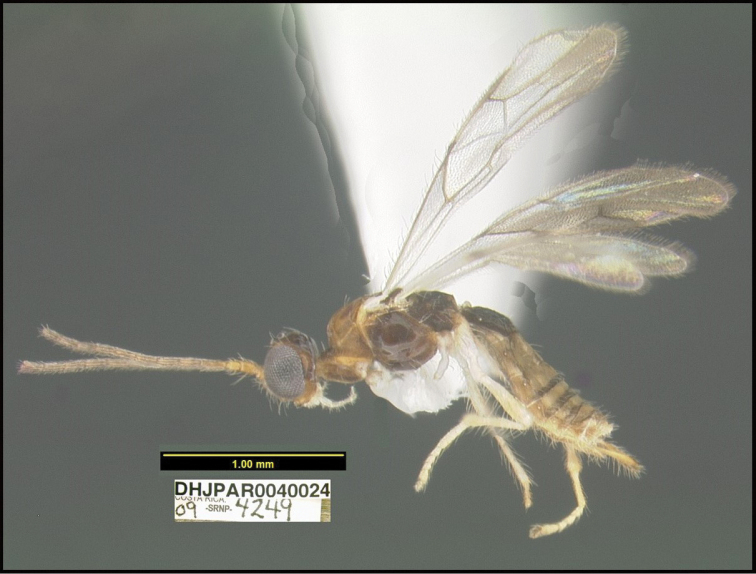
*Hormiusanniapicadoae* holotype.

**Figure 216.**
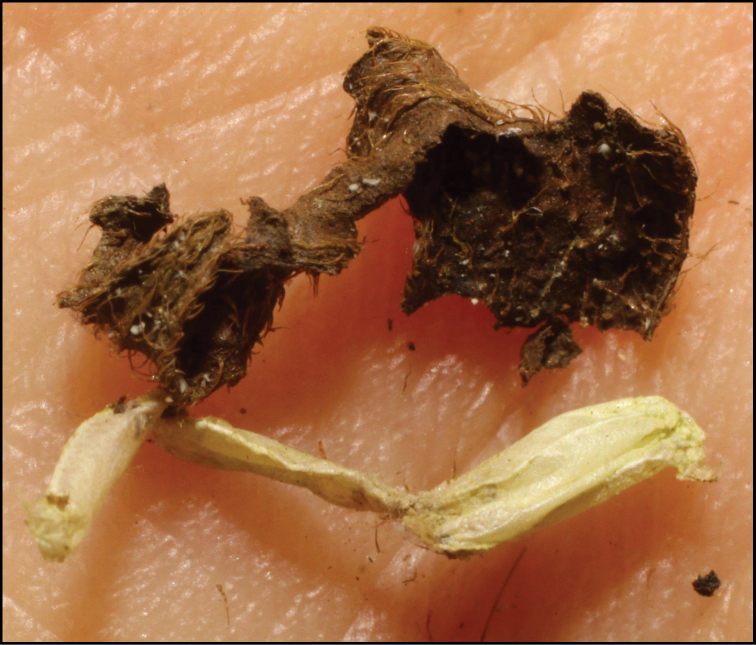
Five tightly packed elongate cocoons of *Hormiusanniapicadoae* (DHJPAR0040024).

##### Hormius arthurchapmani

Taxon classificationAnimaliaHymenopteraBraconidae

Sharkey sp. nov.

http://zoobank.org/2F71BF67-6203-4492-A82F-484B62D87BD1

Figure 217

###### Diagnostics.

BOLD:ADA7345. Consensus barcode. ATTTTATATTTTTTATTTGGGATATGGTCAGGGATATTAGGTTTATCAATAAGGATAATTGTTCGTTTAGAATTAGGGATACCCGGTAGATTGTTAGGTAATGATCAGATTTATAATAGAATAGTAACTGCTCATGCATTTATTATAATTTTTTTTATAGTAATACCAATTATAATTGGAGGGTTTGGAAATTGATTAGTTCCTTTAATATTAGGGTCTCCTGATATAGCTTTTCCTCGTATAAATAATATAAGGTTTTGATTATTAATTCCTTCATTGATATTATTAATTTTTAGAGGGGTGTTGAATGTTGGAGTCGGGACAGGATGAACTATTTATCCTCCATTGTCTTCTTTAATTGGTCATAGAGGAATTTCAGTTGATTTGGCTATTTTTTCTTTACATTTAGCAGGTGCTTCTTCAATTATAGGGGCAATTAATTTTATTACTACTATTTTAAATATAAATTTATATATAAAAATAGATCAAATTAGTTTATTAATTTGATCTATTATGATTACGGCAATTTTATTATTATTATCTTTGCCAGTTTTAGCTGGAGCTATTACAATACTTTTAACTGATCGAAATTTGAATACT.

###### Holotype ♀.

Guanacaste, Sector Pailas Dos, PL12-3, 10.7631, -85.3344, 820 meters, Malaise trap, 8/v/2014. Depository: CNC.

***Host data*.** None.

***Holotype voucher code*.**BIOUG29831-H04.

###### Paratypes.

BIOUG29863-E06, BIOUG29862-H08. Depository: CNC.

###### Etymology.

*Hormiusarthurchapmani* recognizes the participation of Arthur Chapman at the international NSF-funded planning meeting for the All Taxa Biodiversity Inventory (ATBI) of Terrestrial Systems, and the contribution of his wisdom to the planning that was the founding of Costa Rica’s national BioAlfa today.

**Figure 217.**
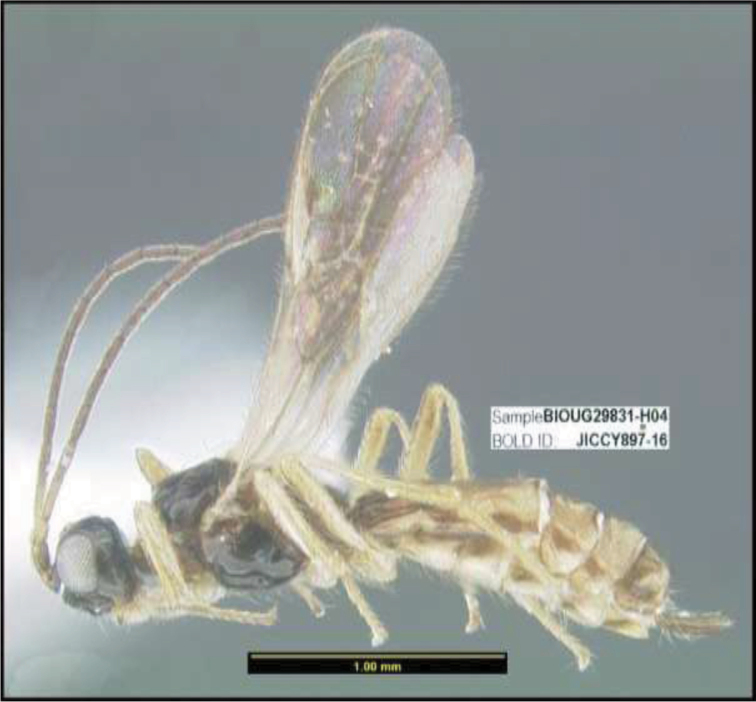
*Hormiusarthurchapmani*, holotype.

##### Hormius barryhammeli

Taxon classificationAnimaliaHymenopteraBraconidae

Sharkey sp. nov.

http://zoobank.org/8348A1DC-BEA2-489E-8A26-C57824D4F4C7

Figures 218 , 219

###### Diagnostics.

BOLD:AAM1049. Consensus barcode. AGTATTATATTTTTTATTTGGGATATGAGCTGGTATAATTGGTTTATCAATAAGATTAATTATTCGTTTAGAGTTAGGTATACCAGGTAGTTTATTAGGTAATGATCAAATTTATAATAGTATAGTTACAGCTCATGCTTTTATTATAATTTTTTTTATAGTTATACCAATTATGATTGGGGGTTTTGGTAATTGATTAGTTCCTTTAATATTAGGTTCACCTGATATAGCATTTCCACGAATAAATAAYATAAGATTTTGATTATTAGTTCCTTCTTTAATATTATTAATTTTTAGTGGATTATTAAATATTGGGGTAGGTACTGGATGAACTATGTATCCACCTTTATCTTCTTTAATTGGTCATGGGGGAATTTCAGTTGATTTAGCAATTTTTTCATTACATTTGGCTGGTATTTCTTCAATTATAGGGGCTATTAATTTTATTTCAACTATTTTAAATATAAATTTATATTATATAAAATTTGATCAAATTAGTTTATTGATTTGATCAATTTTAATTACTGCTGTATTATTATTATTATCTTTGCCTGTTTTAGCTGGGGCAATTACTATATTGTTGACTGATCGTAATTTAAATACAACTTTTTTTGATTTTTCTGGTGGAGGTGACCCTATTTTATTTCAACATTTATTT.

###### Holotype ♀.

Alajuela, Sector Rincon Rain Forest, Jacobo, 10.94076, -85.3177, 461 meters, caterpillar collection date: 07/v/2013, wasp eclosion date: 17/v/2013, one of eight wasps that emerged from the same host caterpillar. Depository: CNC.

***Host data*.** Gregarious parasitoid of *Monoloxis* flavicintalisDHJ02 (Pyralidae) feeding on upper surface of leaves of *Lacistemaaggregatum* (Lacistemataceae).

***Caterpillar and holotype voucher codes*.** 13-SRNP-69652, DHJPAR0052245.

###### Paratypes.

Hosts = *Monoloxis* flavicintalis*DHJ02* and *Cordiapanamensis* (Cordiaceae). 7 specimens, same data as holotype, and DHJPAR0038037. Depository: CNC.

###### Etymology.

*Hormiusbarryhammeli* is named in honor of Barry Hammel in recognition of his persistent high quality and essential taxonomic efforts for the flora of Costa Rica, working in tandem with Nelson Zamora.

**Figure 218.**
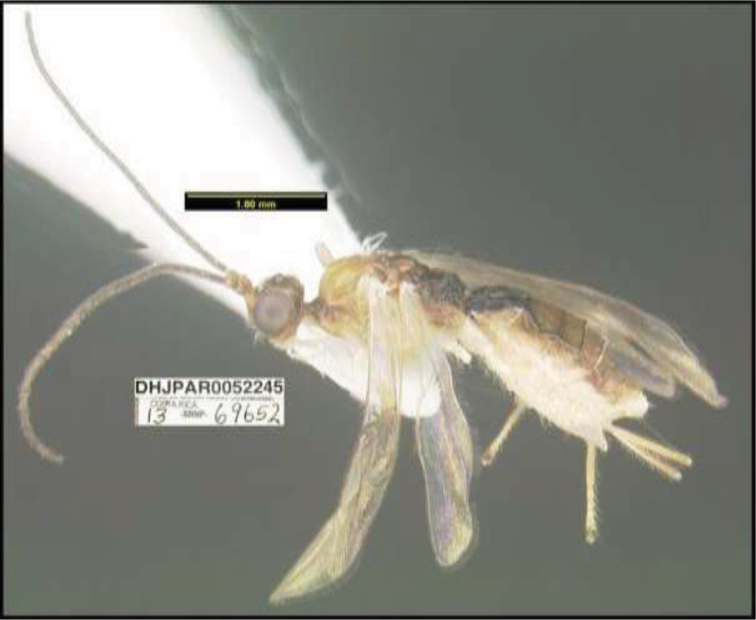
*Hormiusbarryhammeli* holotype.

**Figure 219.**
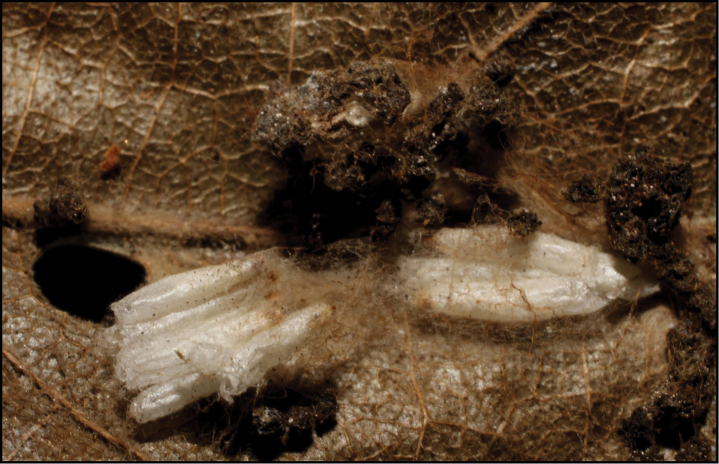
Multiple white cocoons (09-SRNP-23352-DHJ495931.jpg) of *Hormiusbarryhammeli* (DHJPAR0038037) glued to the silk spun on the leaf surface of the nest of the host caterpillar, *Monoloxis* flavicintalisDHJ04 (Pyralidae). The hole through the leaf on the left is the escape hole used by the caterpillar when disturbed.

##### Hormius carloswalkeri

Taxon classificationAnimaliaHymenopteraBraconidae

Sharkey sp. nov.

http://zoobank.org/1BDB961A-6B58-4607-8DB6-64AC2AFAE1B9

**Figure 220.**
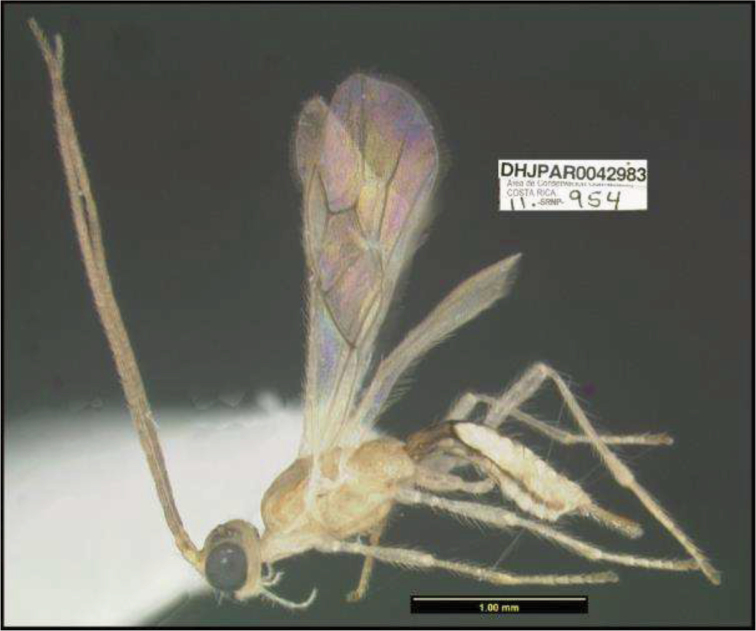
*Hormiuscarloswalkeri* holotype.

**Figure 221.**
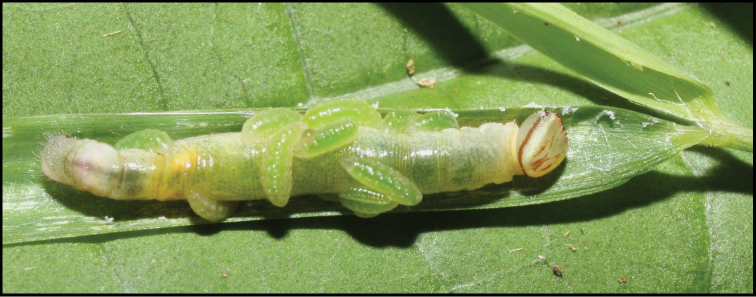
Penultimate instar larvae of *Hormiuscarloswalkeri* (DHJPAR0049061) feeding on living last instar *Tigasissimplex* (Hesperiidae).

**Figure 222.**
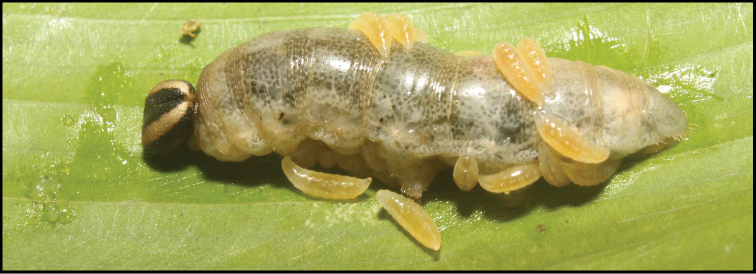
Two last instar larvae of *Hormiuscarloswalkeri* (DHJPAR0057466) feeding on last instar (living) caterpillar of *Cynea Irma* (Hesperiidae).

**Figure 223.**
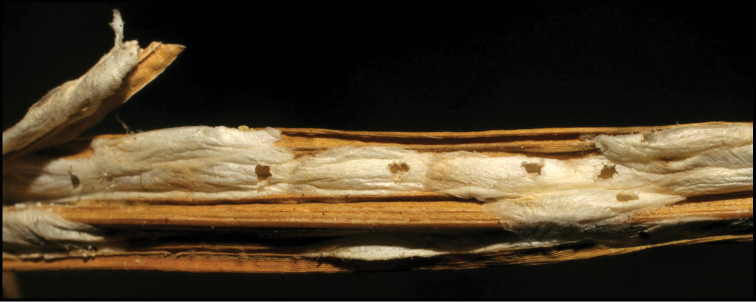
Cocoons of *Hormiuscarloswalkeri* (DHJPAR0029010) that fed on last instar caterpillar of *Joannajoanna* (Hesperiidae) in the caterpillar’s rolled grass leaf nest.

Figures 220 , 221 , 222 , 223

###### Diagnostics.

BOLD:ACK8132. Consensus barcode. ATTATTATATTTTTTATTTGGGATGTGATCAGGAATAATTGGTTTATCAATAAGATTGATTATTCGTTTAGAATTAGGGATACCTGGAAGATTATTAGGTAATGATCAAATTTATAATAGAATAGTTACTGCTCATGCTTTTATTATAATTTTTTTTATAGTTATACCTATTATAATTGGTGGTTTTGGAAATTGATTAATTCCTTTAATATTGGGATCTCCCGATATAGCTTTTCCTCGTATAAATAATATAAGTTTTTGATTATTAATTCCTTCATTAATAATATTAATTTTTAGAGGGATTTTAAATGTTGGAGTTGGTACAGGTTGAACAATATATCCTCCTTTATCTTCACTTATTGGACATAGAGGAATTTCTGTTGATTTAGCAATTTTTTCTTTACATTTAGCTGGTATTTCTTCAATTATAGGTGCTATTAATTTTATTTCTACTATTTTAAATATAAATTTATATTCATTAAAATTAGATCAAATTAATTTATTAATTTGATCAATTTTAATTACTGCTATTTTATTATTATTATCTTTACCTGTTTTAGCAGGAGCAATTACTATATTATTAACTGATCGTAATTTAAATACAACTTTTTTTGATTTTTCAGGAGGAGGAGATCCAATTTTATTTCAACATTTATTT.

###### Holotype ♀.

Guanacaste, Sector Pitilla, Pasmompa, 11.01926, -85.40997, 440 meters, caterpillar collection date: 27/iii/2006, wasp eclosion date: 13/iv/2006, one of 32 wasps that emerged from the same host caterpillar. Depository: CNC.

***Host data*.** Gregarious parasitoid of *Vettiusaurelius* (Hesperiidae) feeding on *Lasiacisprocerrima* (Poaceae).

***Caterpillar and holotype voucher codes*.** 06-SRNP-31447, DHJPAR0029027.

###### Paratypes.

Hosts = *Joannajoanna*, *Vettiusaurelius*, *Cynea Irma*, *Vehiliusvetula*, *Justinianorda*, *Tigasissimplex* (all Hesperiidae). 31 specimens, same data as holotype and DHJPAR0029010, DHJPAR0057466, DHJPAR0047098, DHJPAR0047119, DHJPAR0047127, DHJPAR0049061, DHJPAR0049084, DHJPAR0053764. Depository: CNC.

###### Etymology.

*Hormiuscarloswalkeri* is named in recognition of Carlos Walker’s many years of mentoring the financial arrangement between ACG, GDFCF, and IEDO (formerly Mesoamerica) for the power line crossing Sector Mundo Nuevo of ACG.

##### Hormius carmenretanae

Taxon classificationAnimaliaHymenopteraBraconidae

Sharkey sp. nov.

http://zoobank.org/58BA9FDE-6E83-48D2-A51B-8AED6E9AF5C3

Figures 224 , 225

###### Diagnostics.

BOLD:ABA7266. Consensus barcode. AGTTTTATATTTTTTATTTGGAATATGGGCTGGTATAGTTGGTTTATCAATAAGTTTAATTATTCGATTAGAATTAGGAATACCAGGTAGATTATTGGGTAATGATCAAATTTATAATAGAATAGTTACTGCACATGCTTTTATTATAATTTTTTTTATAGTTATACCAATTATAATTGGAGGATTTGGTAATTGATTAGTTCCTTTAATAATAGGAGCTCCTGATATAGCTTTCCCTCGAATAAATAATATAAGATTTTGATTATTAATTCCTTCTTTAATATTATTGATTTTTAGGGGAGTATTAAATATTGGGGTTGGAACTGGTTGAACTATATATCCTCCTTTATCTTCTTTAATTGGTCATGGTGGTATTTCTGTTGATTTAACAATTTTTTCTTTACATTTGGCTGGAATTTCTTCAATTATAGGAGCTATTAATTTTATTTCTACAATTTTAAATATAAATTTATATTATATAAAATTTGATCAAATTAGTTTATTAATTTGATCAATTTTAATTACTGCTATTTTATTATTATTATCTTTACCTGTTTTAGCTGGGGCTATTACAATATTATTAACAGATCGTAATTTGAATACTACTTTTTTTGATTTTTCTGGGGGAGGGGATCCAATTTTATTTCAACATTTATTT.

###### Holotype ♀.

Alajuela, Sector Rincon Rain Forest, Camino Albergue Oscar, 10.87741, -85.32363, 560 meters, caterpillar collection date: 06/iii/2011, wasp eclosion date: 28/iii/2011, 1 wasp eclosed. Depository: CNC.

***Host data*.** Apparently solitary parasitoid of phyBioLep01 BioLep757 (Pyralidae) feeding on *Crotonbillbergianus* (Euphorbiaceae).

***Caterpillar and holotype voucher codes*.** 11-SRNP-954, DHJPAR0042983.

###### Paratypes.

None.

###### Etymology.

*Hormiuscarmenretanae* is named for Carmen Retana in recognition of her long years of guiding the Minister of Environment’s administrative office of the Ministerio del Ambiente y Energia.

**Figure 224.**
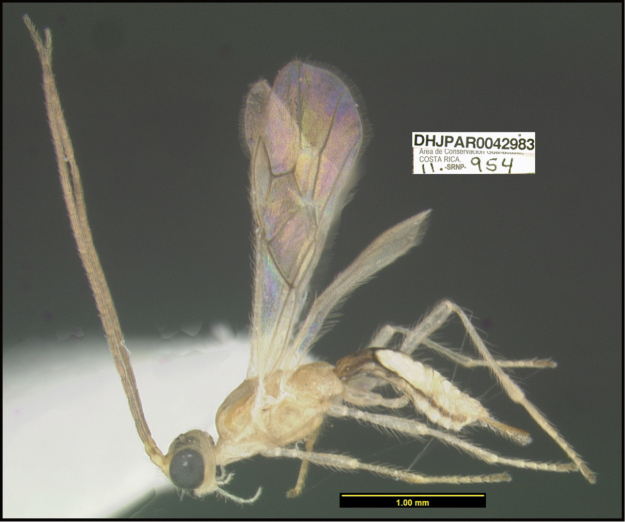
*Hormiuscarmenretanae* holotype.

**Figure 225.**
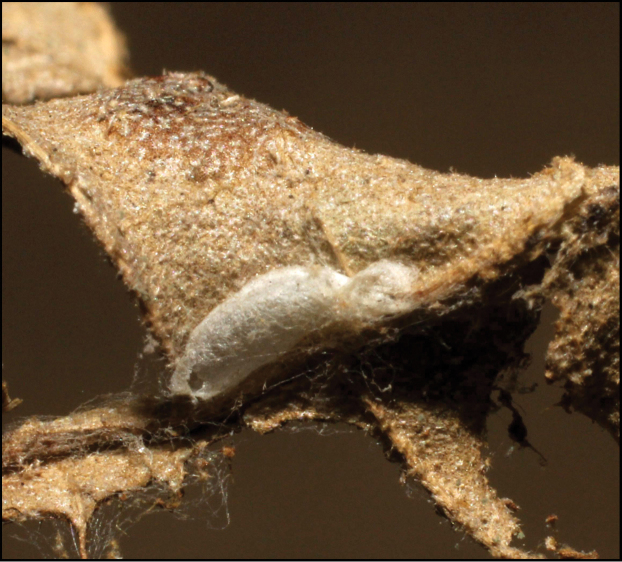
Single cocoon of *Hormiuscarmenretanae* of wasp DHJPAR0042983 spun on leaf surface of nest of phyBioLep01 BioLep757.

##### Hormius cesarsuarezi

Taxon classificationAnimaliaHymenopteraBraconidae

Sharkey sp. nov.

http://zoobank.org/8C1B959C-7B6B-4079-9F7D-C576836D0458

Figures 226 , 227

###### Diagnostics.

BOLD:ACK7796. Consensus barcode. AATTTTGTATTTTTTATTTGGAATATGAGCAGGTATAGTTGGGTTATCAATAAGATTAATTATTCGGTTAGAATTAGGGATACCAGGAAGATTATTAGGTAATGATCAAATTTATAATAGRATAGTTACTGCACATGCATTTGTTATAATTTTTTTTATAGTTATACCAATTATAATTGGAGGATTTGGAAATTGATTAATTCCTTTAATATTAGGTTCACCTGATATAGCTTTYCCTCGTATAAATAATATAAGATTTTGATTATTAGTTCCYTCATTAATATTATTAATTTTTAGTGGTTTATTAAATATTGGRGTGGGTACAGGATGGACAATGTATCCTCCATTATCTTCATTAATTGGACATGGAGGTATTTCTGTTGATTTAGCTATTTTTTCTTTACATTTAGCTGGTATTTCTTCAATTATGGGGGCTATTAATTTTATTTCAACAATTTTTAATATAAATTTGTATTATTTAAAATTGGATCAGATTAATTTATTAATTTGATCAATTTTAATTACTGCTGTTTTATTAYTATTATCTCTACCTGTTTTAGCTGGAGCAATTACAATATTATTAACTGATCGTAATTTAAATACAACATTTTTTGATTTTTCMGGAGGRGGTGAYCCAATTTTATTTCAACATTTATTT.

###### Holotype ♀.

Sector Pitilla, Ingas, 11.00311, -85.42041, 580 meters, caterpillar collection date: 15/ii/2012, wasp eclosion date: 29/ii/2012, one of three wasps that emerged from the same host caterpillar. Depository: CNC.

***Host data*.** Gregarious parasitoid of *Herpetogrammasalbialis* (Crambidae) feeding on *Zexmeniavirgulta* (Asteraceae).

***Caterpillar and holotype voucher codes*.** 12-SRNP-30368, DHJPAR0049093.

###### Paratypes.

Hosts = *Herpetogrammasalbialis*. 2 specimens, same data as holotype and DHJPAR0047041, DHJPAR0047116, DHJPAR0039420, DHJPAR0039421, DHJPAR0031136, DHJPAR0038115, DHJPAR0038262, DHJPAR0053699, DHJPAR0029000, DHJPAR0029006, DHJPAR0029013, DHJPAR0035321, DHJPAR0035390, DHJPAR0041698, DHJPAR0041709, DHJPAR0041724. Depository: CNC.

###### Etymology.

*Hormiuscesarsuarezi* is named in recognition of Cesar Suarez’s continued management support for the IEDO powerline crossing Sector Mundo Nuevo of ACG.

**Figure 226.**
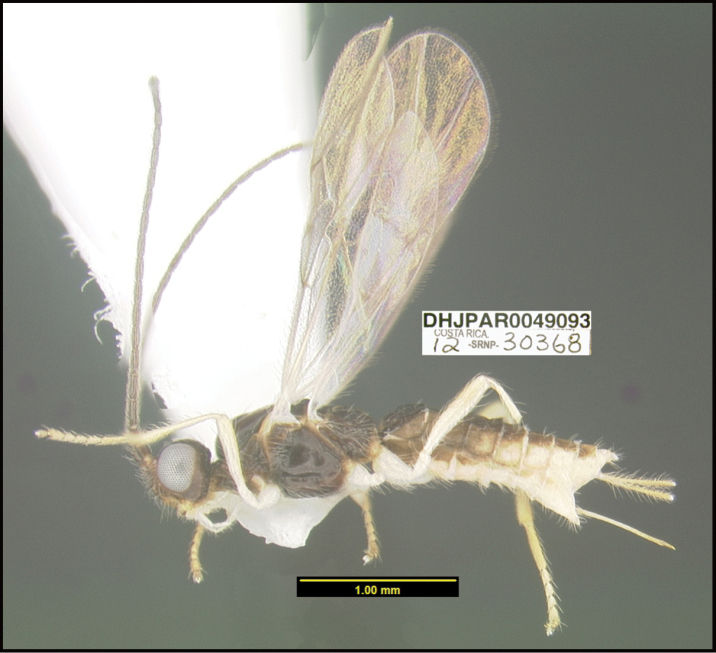
*Hormiuscesarsuarezi* holotype.

**Figure 227.**
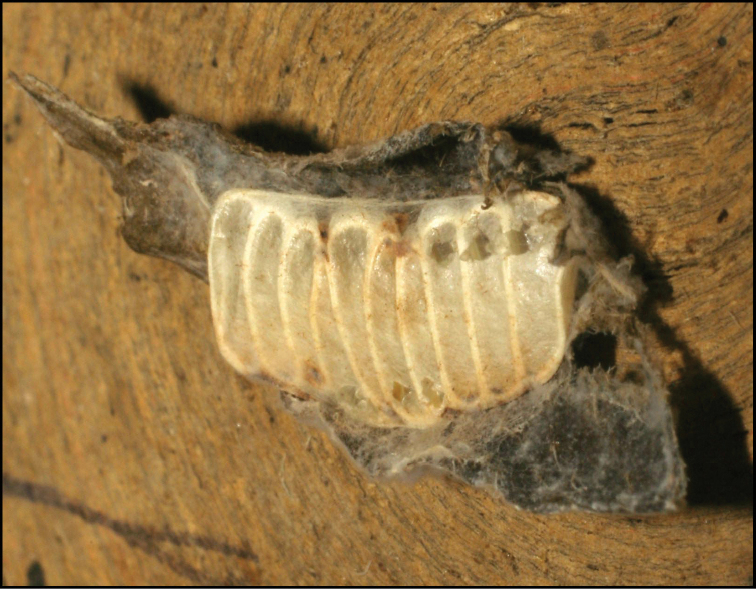
Cocoons of *Hormiuscesarsuarezi* (DHJPAR0029013) glued to the wall of the leaf nest of the last instar caterpillar of *Herpetogrammasalbialis* (Crambidae).

##### Hormius danbrooksi

Taxon classificationAnimaliaHymenopteraBraconidae

Sharkey sp. nov.

http://zoobank.org/410540D6-39C8-4FF2-B9E9-288F8DA6CB3D

Figure 228

###### Diagnostics.

BOLD:ADY0841. Consensus barcode. TTTATATTTTTTATTTGGAATATGATCTGGAATAATTGGTTTATCAATAAGATTAATTATTCGTTTAGAATTAGGTATACCAGGGAGTTTATTAGGTAATGATCAAATTTATAATACAATAGTTACAGCTCATGCTTTTATTATAATTTTTTTTATAGTTATACCAATTATAATCGGAGGTTTTGGAAATTGATTAGTTCCTTTAATATTAGGTTCTCCAGATATAGCTTTTCCTCGGATAAATAATATAAGTTTTTGGTTATTAGTTCCTTCTTTAATATTATTAATTTTTAGTGGTTTATTGAATATTGGTGTTGGAACAGGTTGAACAATATATCCTCCATTATCTTCTTTAATTGGACATAGAGGAATTTCTGTTGATTTGGCTATTTTTTCATTACATTTGGCTGGTATTTCATCAATTATAGGTGCAATTAATTTTATTTCAACTATTTTAAATATAAATTTATATTATATAAAATTAGATCAGATTAGTTTATTAATTTGATCAATTTTAATTACTGCTATTTTATTATTATTGTCTTTACCCGTTTTAGCTGGTGCAATTACTATATTATTAACTGATCGTAATTTAAATACTACATTTTTTGATTTTTCTGGTGGAGGTGATCCAATTTTATTTCAACATTTAT.

###### Holotype ♀.

Guanacaste, Sector Pailas Dos, PL12-4, 10.7629, -85.3341, 820 meters, Malaise trap, 20/xi/2014. Depository: CNC.

***Host data*.** None.

***Holotype voucher code*.**BIOUG46727-F07.

###### Paratypes.

None.

###### Etymology.

*Hormiusdanbrooksi* is named in recognition of the attendance and guidance of Dan Brooks at the international NSF-funded planning meeting for the All Taxa Biodiversity Inventory (ATBI) of Terrestrial Systems, and contributing his wisdom to the planning that was the founding of Costa Rica’s national BioAlfa today.

**Figure 228.**
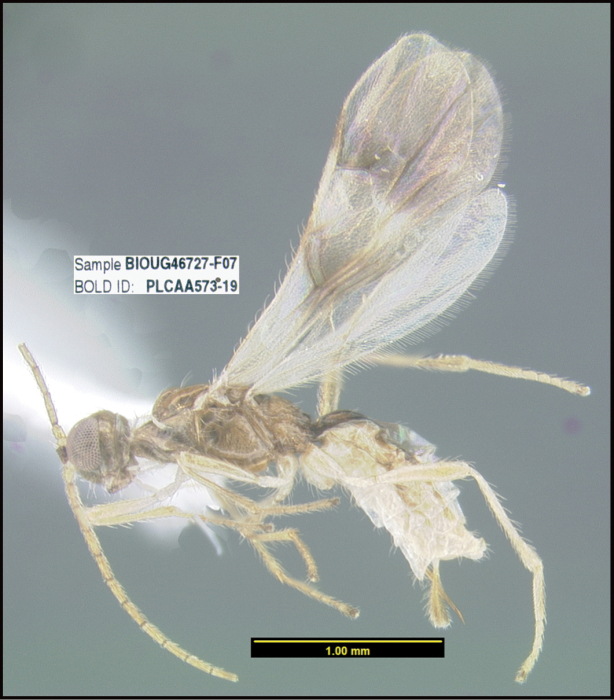
*Hormiusdanbrooksi*, holotype.

##### Hormius eddysanchezi

Taxon classificationAnimaliaHymenopteraBraconidae

Sharkey sp. nov.

http://zoobank.org/CC62D6EF-FE3C-442E-A1B2-DD9120314F79

**Figure 229.**
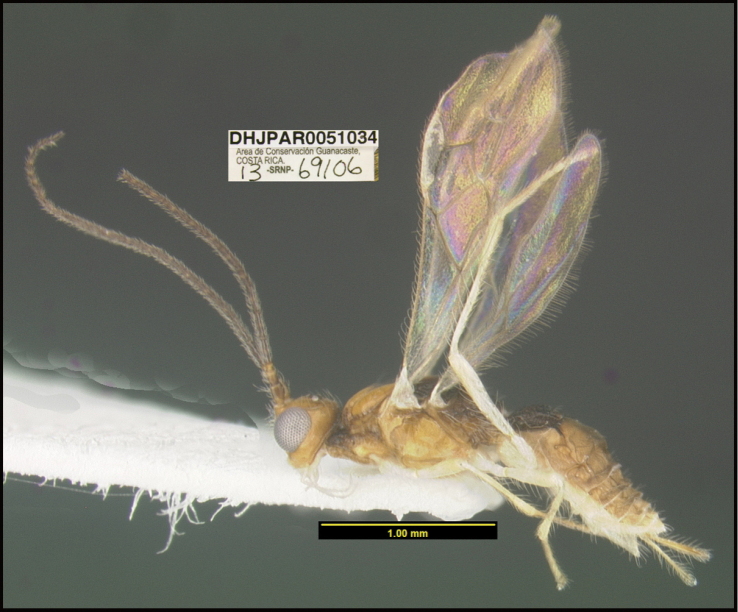
*Hormiuseddysanchezi* holotype.

Figure 229 , 230 , 231

###### Diagnostics.

BOLD:ACC0955. Consensus barcode. AATTTTATATTTTTTTTTTGGAATATGGGCTGGTATAGTTGGCTTATCTATAAGTTTAATTATTCGTTTAGAATTAGGGATACCTGGTAGATTATTAGGTAATGATCAAATTTATAATAGAATAGTTACTGCTCATGCATTTATTATAATTTTTTTTATAGTTATACCAATTATAATTGGAGGTTTYGGAAATTGATTAATTCCTTTAATATTAGGATCTCCTGATATAGCTTTYCCTCGAATAAATAACATAAGATTTTGATTATTAGTACCTTCTTTAATATTATTAGTTTTTAGAGGAATTTTAAATATTGGGGTAGGAACAGGTTGAACAATATACCCACCATTATCTTCATTAATTGGTCATAGAGGAATTTCAGTTGATATAGCTATTTTTTCATTACATTTAGCTGGTATTTCTTCAATTATAGGAGCTATTAATTTTATTTCTACTATTTTTAATATAAAATTAAATTATATAAAAATAGATCAAATTAATTTATTAATTTGATCTATTTTAATTACAGCAATTTTATTATTATTATCTTTRCCCGTTTTAGCCGGTGCAATTACTATATTATTAACTGATCGTAATTTAAATACAACTTTTTTTGATTTTTCTGGAGGAGGGG.

###### Holotype ♀.

Rincon Rain Forest, Jacobo, 10.94076, -85.3177, 461 meters, caterpillar collection date: 14/i/2013, wasp eclosion date: 30/i/2013, one of two wasps that emerged from the same host caterpillar. Depository: CNC.

***Host data*.** Gregarious parasitoid of crambidBioLep01 BioLep77 (Crambidae) feeding on *Lepidaploatortuosa* (Asteraceae).

***Caterpillar and holotype voucher codes*.** 13-SRNP-69106, DHJPAR0051034.

###### Paratypes.

Hosts = *Herpetogrammasalbialis* crambidBioLep01 BioLep77 (both Crambidae). DHJPAR0056927, DHJPAR0050031. Depository: CNC.

###### Etymology.

*Hormiuseddysanchezi* is named in honor of Eddy Sanchez in recognition of his bureaucratic bravery of allowing the government’s PL12 geothermal project to be biomonitored by ACG.

**Figure 230.**
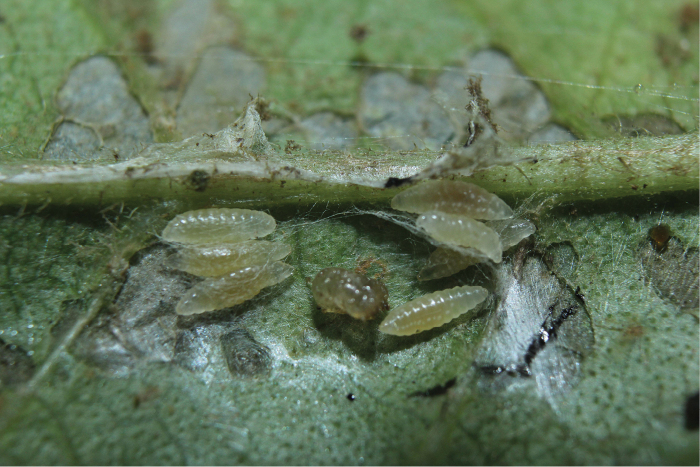
Last instar larvae of *Hormiuseddysanchezi* (DHJPAR0056927) a few hours after spinning their in-common over-layer of silk, now peeled back to show the larvae.

**Figure 231.**
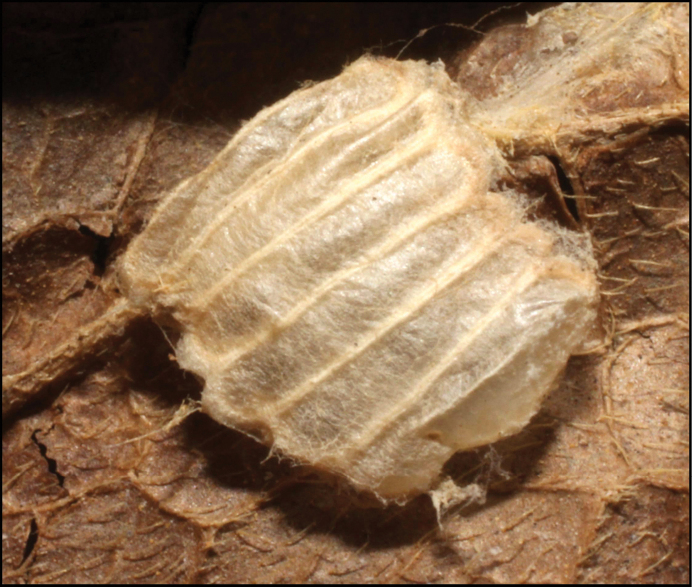
Cocoons of *Hormiuseddysanchezi* (DHJPAR0050031) spun side by side under a common layer of silk, glued to the leaf of the caterpillar nest.

##### Hormius erikframstadi

Taxon classificationAnimaliaHymenopteraBraconidae

Sharkey sp. nov.

http://zoobank.org/7A9F97B2-EFAF-421B-BD7E-A544153AD473

Figure 232

###### Diagnostics.

BOLD:ACG5362. Consensus barcode. TATTTTATATTTTTTATTTGGAATATGATCAGGAATATTAGGTTTATCAATAAGATTAATTATTCGTTTAGAATTA---GGGATACCTGGTAGATTATTAGGTAATGATCAAATTTATAATAGGATAGTTACTGCTCATGCTTTTATTATAATTTTTTTTATAGTTATACCAATTATGATTGGTGGATTTGGGAATTGGTTAATTCCTTTAATGTTAGGGTCTCCTGATATAGCTTTCCCTCGAATAAATAATATAAGGTTTTGATTATTAATTCCTTCATTAATATTATTAATTTTTAGGGGGTTATTAAATATTGGGGTTGGGACAGGATGAACTGTTTATCCCCCATTATCTTCTTTAATTGGGCATGGGGGGATTTCTGTGGATTTAGCAATTTTTTCTTTACATTTGGCTGGTGTTTCATCAATTATGGGGGCAATTAATTTTATTACTACTATTTTAAATATAAATTTATATATA---AAAATAGATCAAATTAATTTATTAATTTGATCTATTATAATTACGGTAATTTTGTTATTATTATCATTACCAGTTTTGGCTGGTGCTATTACTATACTTTTAACTGATCGAAATTTAAATACAACTTTTTT-------------------------------------------------------------------.

###### Holotype ♀.

Guanacaste, Sector Santa Rosa Bosque San Emilio, 10.8438, -85.6138, 300 meters, Malaise trap, 30/vii/2012. Depository: CNC.

***Host data*.** None.

***Holotype voucher code*.**BIOUG05883-A02.

###### Paratypes.

None.

###### Etymology.

*Hormiuserikframstadi* is named in honor of Erik Framstad for his participation in the international NSF-funded planning meeting for the All Taxa Biodiversity Inventory (ATBI) of Terrestrial Systems, and contributing his wisdom to the planning that was the founding of Costa Rica’s national BioAlfa today.

**Figure 232.**
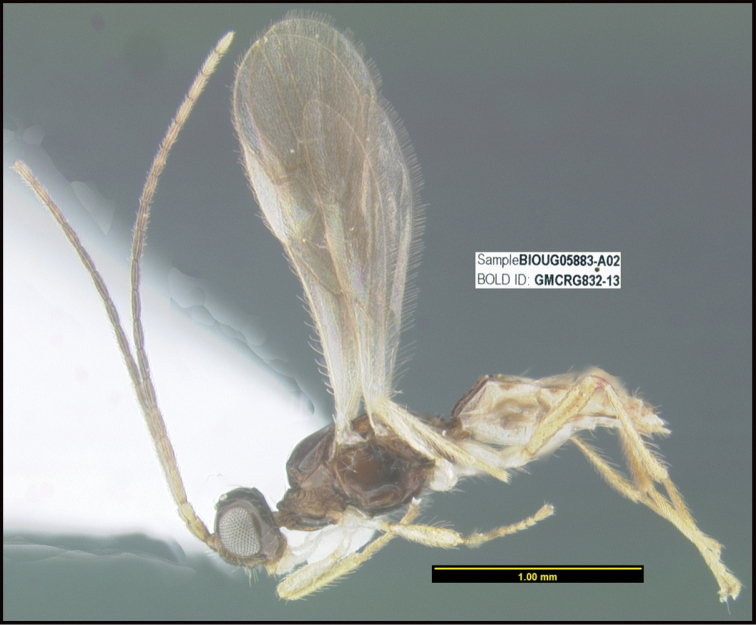
*Hormiuserikframstadi*, holotype.

##### Hormius georgedavisi

Taxon classificationAnimaliaHymenopteraBraconidae

Sharkey sp. nov.

http://zoobank.org/94E505DF-594E-461E-8C9A-815F5E395B44

Figure 233

###### Diagnostics.

BOLD:ACG5375. Consensus barcode. TGTTTTATATTTTTTATTTGGAATATGATCAGGTATATTAGGTTTATCAATAAGATTGATTGTTCGTTTAGARTTAGGGATACCTGGTAGTTTATTAGGTAATGATCAAATTTATAATTCTATAGTTACAGCTCATGCATTTATTATAATTTTTTTTATAGTTATACCTATTATGATTGGAGGTTTTGGTAATTGATTAATTCCTTTAATATTAGGATCTCCTGATATAGCTTTTCCTCGAATAAATAATATGAGGTTTTGATTATTAATTCCTTCTTTAATATTATTAATTTTTAGAGGTTTATTAAATATTGGGGTTGGTACAGGTTGAACTATTTATCCTCCTTTATCTTCATTAATTGGTCATGGAGGTATTTCTGTTGATTTAGCTATTTTTTCTTTACATTTAGCTGGTATTTCTTCTATTATAGGGGCTATTAATTTTATTACTACTATTTTTAATATGAATTTATATATAAAAATAGATCAAATTAGTTTATTAATTTGATCTATTATGATTACAGCTATTTTATTATTATTGTCTTTACCTGTATTAGCTGGAGCTATTACTATATTATTAACTGATCGAAATTTAAATACAACTTTTTT.

###### Holotype ♀.

Guanacaste, Sector Pailas Dos, PL12-6, 10.7637, -85.333, 853 meters, Malaise trap, 20/ii/2014. Depository: CNC.

***Host data*.** None.

***Holotype voucher code*.**BIOUG29020-B06.

###### Paratypes.

BIOUG29015-D12, BIOUG05883-C01. Depository: CNC.

###### Etymology.

*Hormiusgeorgedavisi* is named in honor of George Davis attending the international NSF-funded planning meeting for the All Taxa Biodiversity Inventory (ATBI) of Terrestrial Systems, and contributing his wisdom to the planning that was the founding of Costa Rica’s national BioAlfa today.

**Figure 233.**
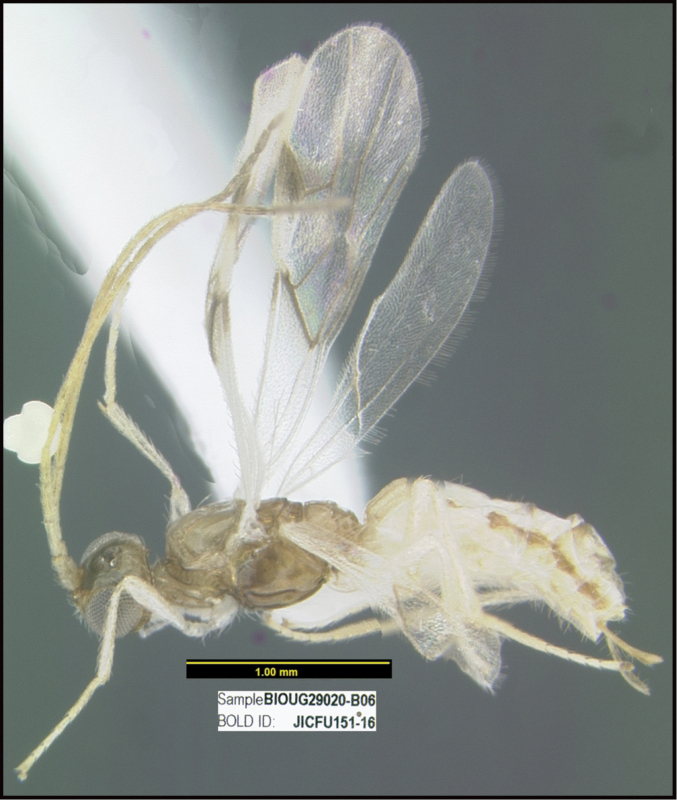
*Hormiusgeorgedavisi*, holotype.

##### Hormius grettelvegae

Taxon classificationAnimaliaHymenopteraBraconidae

Sharkey sp. nov.

http://zoobank.org/F7DD9E7D-1981-4827-8F09-A4B3739AFDC5

Figure 234

###### Diagnostics.

BOLD:AAM1094. Consensus barcode. AGTTTTATATTTTTTATTTGGAATATGAGCTGGTATAATTGGTTTATCAATAAGTTTAATTATTCGTTTAGAATTAGGAATACCGGGTAGGTTATTAGGAAATGATCAAATTTATAATAGTATAGTAACTGCTCATGCTTTTATTATAATTTTTTTTATAGTTATACCTATTATAATTGGAGGTTTTGGAAATTGATTAGTTCCTTTAATATTAGGTTCTCCTGATATGGCTTTTCCACGTATAAATAATATAAGGTTTTGATTATTAATTCCTTCTTTGATATTATTAATTTTTAGAGGAGTATTAAATATTGGRGTAGGAACTGGATGAACTATATATCCTCCATTATCTTCATTGATTGGACATAGAGGTATTTCAGTTGATTTAGCAATTTTCTCTTTACATTTAGCTGGAATTTCATCAATTATAGGAGCAATTAATTTTATTTCTACAATTTTAAATATAAATTTGTATAATATAAAATTTGATCAAATTAGATTATTAATTTGATCTATTTTAATTACTGCAATTTTACTTTTATTATCTTTACCTGTTTTRGCGGGTGCAATTACTATATTATTAACAGATCGAAATTTAAATACAACTTTTTTTGAYTTTTCGGGGGGAGGTGATCCTATTTTATTTCAACATTTATTT.

###### Holotype ♀.

Alajuela, Sector Rincon Rain Forest, Tamarindo, 10.94259, -85.31333, 478 meters, caterpillar collection date: 10/iii/2010, wasp eclosion date: 28/iii/2010, one wasp eclosed from two cocoons in the host caterpillar. Depository: CNC.

***Host data*.** Solitary parasitoid of *Antaeotricharibbei* (Depressariidae) feeding on *Guarearhopalocarpa* (Meliaceae).

***Caterpillar and holotype voucher codes*.** 10-SRNP-69369, DHJPAR0038964.

###### Paratypes.

DHJPAR0058966 reared from *Gonionota* Janzen116 (Depressariidae). Depository: CNC.

###### Etymology.

*Hormiusgrettelvegae* is named in honor of Grettel Vega in recognition of her enthusiasm for the integration of Costa Rica’s SINAC with BioAlfa of Costa Rica.

**Figure 234.**
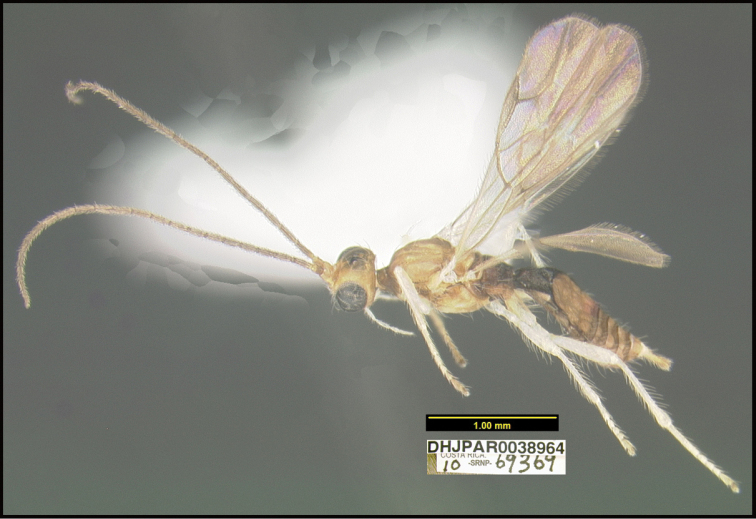
*Hormiusgrettelvegae* holotype.

##### Hormius gustavoinduni

Taxon classificationAnimaliaHymenopteraBraconidae

Sharkey sp. nov.

http://zoobank.org/F9710F43-3BA7-40B1-B8D3-C716AB5BBE10

Figure 235

###### Diagnostics.

BOLD:ABA7247. Consensus barcode. AGTTTTATATTTTTTATTTGGAATATGATCTGGAATGGTTGGGTTATCTATAAGTTTAATTATTCGATTAGAGTTAGGGATACCAGGGAGATTATTGGGGAATGATCAGATTTATAATAGTATAGTAACCGCTCATGCTTTTATTATAATTTTTTTTATAGTTATACCTATTATAATTGGGGGATTTGGTAATTGATTAATTCCTTTAATAATAGGATCTCCTGATATAGCTTTTCCTCGAATAAACAATATAAGCTTTTGGTTATTGATTCCTTCATTGATATTATTAATTTTTAGTGGTATATTAAATATTGGTGTTGGAACTGGTTGAACAATATATCCTCCTTTATCTTCTTTAATTGGGCATGGAGGTATTTCTGTTGATTTGGCAATTTTTTCTTTACATTTAGCTGGTATTTCTTCAATTATAGGAGCTATTAATTTTATTACAACAATTATAAATATAAATTTATATTATATTAAATTTGACCAAATTAGTTTATTAATTTGGTCAATTTTAATTACTGCTATTTTATTATTATTATCTTTACCTGTTTTAGCTGGGGCTATTACAATATTGTTAACAGATCGTAATTTAAATACTACCTTTTTTGATTTTTCTGGAGGAGGGGATCCAATTTTATTTCAACATTTATTT.

###### Holotype ♀.

Guanacaste, Sector Pitilla, Estación Quica, 10.99697, -85.39666, 470 meters, caterpillar collection date: 22/i/2010, wasp eclosion date: 10/ii/2010 one of two wasps that emerged from the same host caterpillar. Depository: CNC.

***Host data*.** Gregarious parasitoid of elachJanzen01 Janzen764 (Depressariidae) feeding on *Microgrammapercussa* (Polypodiaceae).

***Caterpillar and holotype voucher codes*.** 10-SRNP-70422, DHJPAR0038344.

###### Paratypes.

Hosts = elachJanzen01 Janzen764. 1 specimen same data as holotype and DHJPAR0043056. Depository: CNC.

###### Etymology.

*Hormiusgustavoinduni* is named in honor of Gustavo Induni in recognition of his enthusiastic participation in the BioAlfa process for Costa Rica, as a government employee.

**Figure 235.**
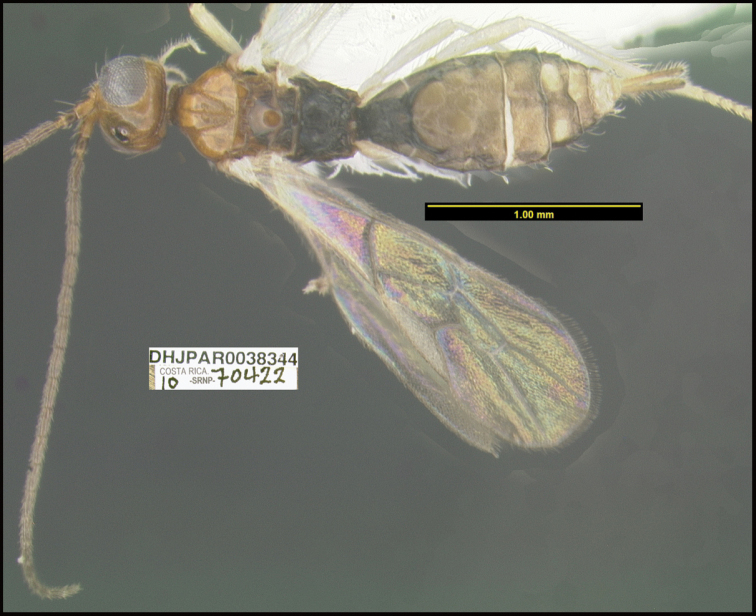
*Hormiusgustavoinduni* holotype.

##### Hormius hartmanguidoi

Taxon classificationAnimaliaHymenopteraBraconidae

Sharkey sp. nov.

http://zoobank.org/ABA8E98B-C6A5-499F-AEC5-27681AD153B9

Figures 236 , 237

###### Diagnostics.

BOLD:ACJ2468. Consensus barcode. TGTTTTATATTTTATATTTGGAATATGATCTGGAATAGTTGGTTTATCAATGAGATTAATTATTCGTTTAGAGTTGGGGATACCTGGAAGTTTATTAGGAAATGATCAAATTTATAATAGTATGGTTACATCTCATGCTTTTATTATAATTTTTTTTATGGTTATACCTATTATGATTGGAGGGTTTGGAAATTGGTTAGTTCCTTTAATATTAGGGTCTCCTGATATGGCTTTTCCTCGAATAAATAATATGAGATTTTGATTATTGGTTCCTTCTTTAATATTATTAATTTTTAGTGGATTATTAAATATTGGGGTTGGAACAGGTTGAACTATATATCCACCTTTATCTTCATTAATTGGTCATGGAGGAATTTCTGTTGATTTAGCTATTTTTTCTTTACATTTAGCTGGTATTTCTTCAATTATAGGAGCTATTAATTTTATTTCAACAATTTTAAATATAAATTTGTATTATATAAAATTGGATCAGATTAATTTATTAATTTGATCTATTTTAATTACTGCTATTTTATTATTATTATCTTTACCTGTATTAGCAGGGGCTATTACTATATTATTAACTGATCGTAATTTAAATACAACATTTTTTGATTTTTCAGGTGGAGGAGATCCAATTTTATTTCAACATTTATTT.

###### Holotype ♀.

Sector Rincon Rain Forest, Camino Rio Francia, 10.90425, -85.28651, 410 meters, caterpillar collection date: 14/xii/2012, wasp eclosion date: 11/i/2013, one of seven wasps that emerged from the same host caterpillar. Depository: CNC.

***Host data*.** Gregarious parasitoid of *Dichomeris* Janzen703 (Gelechiidae) feeding on leaves of *Lepidaploatortuosa* (Asteraceae).

***Caterpillar and holotype voucher codes*.** 12-SRNP-87112, DHJPAR0050996.

###### Paratypes.

6 specimens, same data as holotype. Depository: CNC.

###### Etymology.

*Hormiushartmanguidoi* is named in honor of Hartman Guido in recognition of his long-standing support for the biomonitoring of ICE’s biomonitoring of PL12 geothermal project.

**Figure 236.**
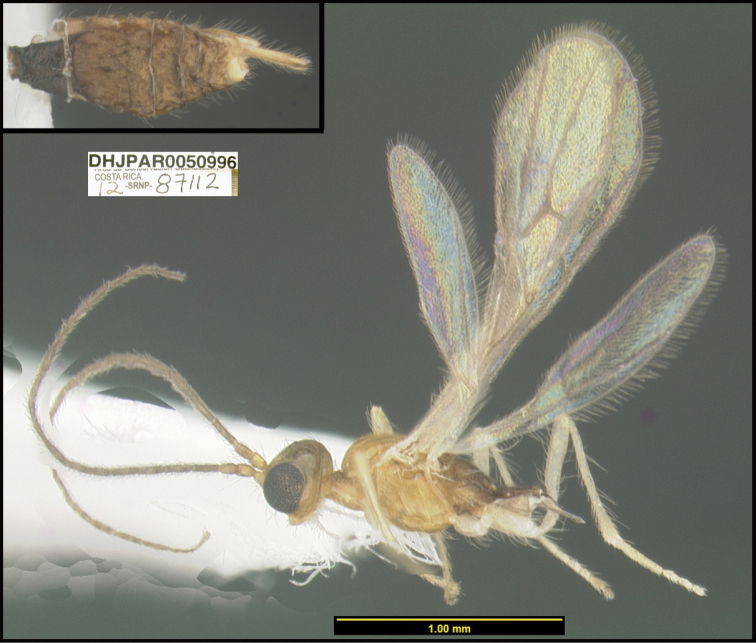
*Hormiushartmanguidoi* holotype.

**Figure 237.**
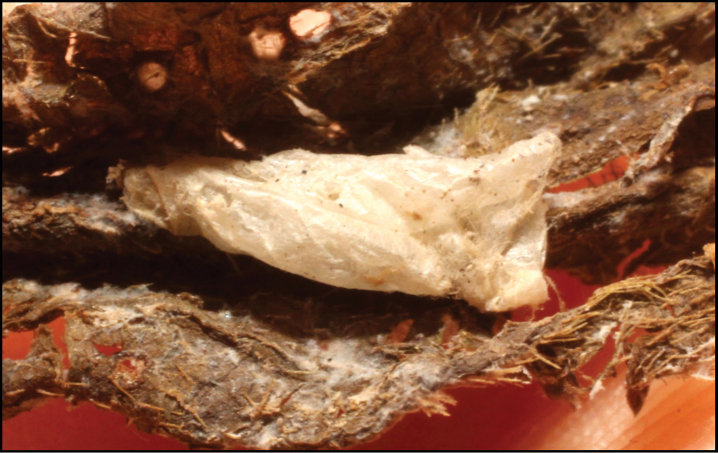
Mass of tightly clumped cocoons of *Hormiushartmanguidoi* (DHJPAR0050996) inside the lightly silked leaves of the cocoon of *Dichomerus* Janzen703.

##### Hormius hectoraritai

Taxon classificationAnimaliaHymenopteraBraconidae

Sharkey sp. nov.

http://zoobank.org/82476464-10D8-42D5-A1F7-D222D97E540E

Figure 238

###### Diagnostics.

BOLD:ADY6062. Consensus barcode. ATTATATTTTATCTTTGGAATATGGTCAGGTATATTAGGGTTATCAATAAGTTTAATTATTCGAATAGAGTTGGGGATACCAGGAAGGTTGTTAGGTAATGATCAAATTTATAATACGATGGTGACAGCTCATGCATTTATTATAATTTTTTTTATAGTTATACCAATTATGATTGGGGGGTTTGGAAATTTTTTAATTCCTTTAATATTAGGGGCTCCTGATATAGCTTTCCCTCGAATAAATAATATAAGGTTTTGATTATTAATTCCTTCTTTAATTTTATTAATTTTAAGAAGGTTATTAAATACAGGGGTAGGTACAGGTTGAACAATATATCCTCCTTTATCTTTATTATTAGGACATGGAGGTATTTCTGTAGATTTATCAATTTTTTCTTTACATTTAGCAGGAATTTCATCAATTATGGGGGCAATTAATTTTATTACAACTATTTTTAATATAAATTTATTTACAATTAAAATAGATCAAATTATATTATTAGTTTGATCTGTAATAATTACTGCTTTTTTATTATTATTATCATTACCTGTATTAGCGGGGGCTATCACAATATTATTAACAGATCGTAATTTAAATACAAGATTTTTTGATTTTTCAGGAGGGGGGGATCCTATTTTATTTCAACATTTATT.

###### Holotype ♀.

Guanacaste, Sector Pailas Dos, PL12-3, 10.7631, -85.3344, 820 meters, Malaise trap, 5/ii/2015. Depository: CNC.

***Host data*.** None.

***Holotype voucher code*.**BIOUG44643-B02.

###### Paratypes.

None.

###### Etymology.

*Hormiushectoraritai* is named in honor of Hector Arita attending the international NSF-funded planning meeting for the All Taxa Biodiversity Inventory (ATBI) of Terrestrial Systems, and contributing his wisdom to the planning that was the founding of Costa Rica’s national BioAlfa today.

**Figure 238.**
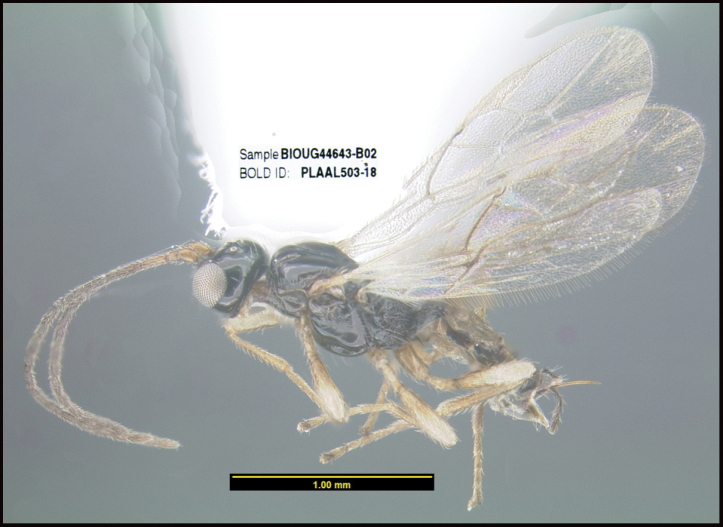
*Hormiushectoraritai*, holotype.

##### Hormius hesiquiobenitezi

Taxon classificationAnimaliaHymenopteraBraconidae

Sharkey sp. nov.

http://zoobank.org/D599128A-DA41-4DCA-B6E3-4D9A2E7F4F18

Figure 239

###### Diagnostics.

BOLD:ACJ1489. Consensus barcode. AGTTTTATATTTTTTATTTGGTATATGATCTGGAATAATTGGATTATCAATAAGTTTAATTATTCGATTAGAATTAGGTATACCAGGTAGTTTATTAGGAAATGACCAAATTTATAATAGAATAGTAACCGCTCATGCTTTTATTATAATTTTTTTTATAGTCATACCAATTATAATTGGAGGTTTTGGAAATTGATTAATTCCTTTAATAATAGGATCTCCTGATATAGCTTTTCCTCGAATAAATAATATAAGGTTTTGATTATTAATTCCTTCATTAATAATATTAGTTTTTAGGGGTTTATTAAATATTGGAGTTGGAACTGGTTGAACTATATATCCTCCTTTGTCTTCATTAATTGGACAYAGAGGTATTTCTGTTGATTTGGCAATTTTTTCTTTACATTTAGCTGGAATTTCTTCAATTATAGGAGCTATTAATTTTATTTCTACAATTATAAATATAAATTTATATTATATAAAATTTGATCAGATTAGATTATTAATTTGATCAATTTTAATTACTGCTATTTTATTATTGTTATCATTACCTGTTTTAGCTGGGGCTATTACAATATTATTAACAGATCGTAATTTGAATACAACTTTTTTTGATTTTTCTGGAGGAGGTGATCCAATTTTATTTCAACATTTATTT.

###### Holotype ♀.

Guanacaste, Sector Santa Rosa Bosque San Emilio, 10.8438, -85.6138, 300 meters, Malaise trap, 23/iv/2012. Depository: CNC.

***Host data*.** None.

***Holotype voucher code*.**BIOUG07617-B11.

###### Paratypes.

BIOUG07616-G03, BIOUG07458-G07. Depository: CNC.

###### Etymology.

*Hormiushesiquiobenitezi* is named in honor of Hesiquio Benitez attending the international NSF-funded planning meeting for the All Taxa Biodiversity Inventory (ATBI) of Terrestrial Systems, and contributing his wisdom to the planning that was the founding of Costa Rica’s national BioAlfa today.

**Figure 239.**
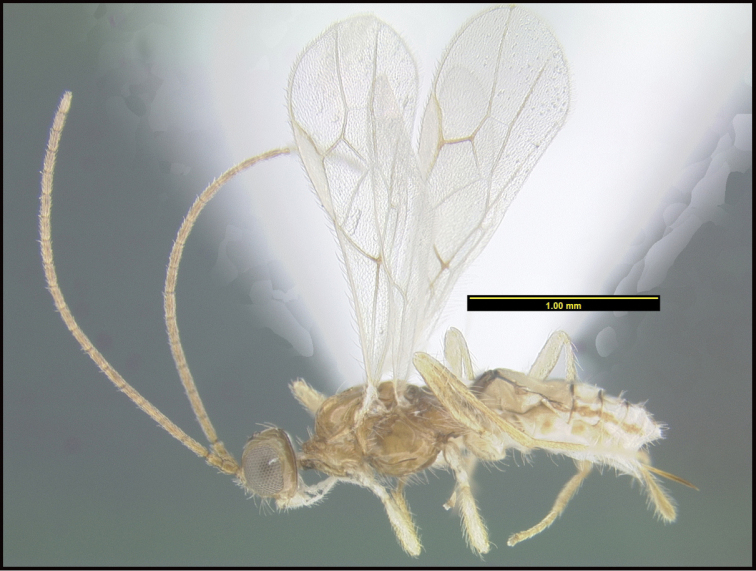
*Hormiushesiquiobenitezi*, holotype.

##### Hormius irenecanasae

Taxon classificationAnimaliaHymenopteraBraconidae

Sharkey sp. nov.

http://zoobank.org/DA4FC36C-9779-4981-B486-697903800D97

Figures 240 , 241

###### Diagnostics.

BOLD:ACB2774. Consensus barcode. AGTTTTATATTTTTTATTTGGAATGTGAGCTGGAATAGTTGGTTTATCAATGAGTTTAATTATTCGTTTAGAATTAGGTATACCAGGTAGATTATTAGGTAATGATCAAATTTATAATAGTATAGTYACTGCTCATGCTTTTATTATAATTTTTTTTATAGTTATACCAATTATAATTGGGGGWTTTGGAAATTGATTAGTTCCATTAATAATAGGTGCYCCTGATATAGCTTTTCCTCGAATAAATAATATAAGTTTTTGATTATTAATTCCTTCTTTAATATTATTAATTTTTAGGGGATTATTAAATATTGGRGTTGGTACTGGTTGAACAATATATCCTCCTTTATCTTCTTTAATTGGRCATGGAGGAATTTCTGTTGATTTAGCAATTTTTTCTTTACATTTAGCTGGAATTTCTTCAATTATAGGAGCTATTAATTTTATTTCAACAATTTTAAATATAAATTTATATTATATAAAATTTGATCAGATTAGATTATTAATTTGATCAATTTTAATTACTGCTATTTTATTATTATTATCWTTACCTGTTTTRGCTGGGGCTATTACAATATTATTAACAGATCGTAATTTAAATACTACTTTTTTTGATTTTTCAGGAGGGGGGGACCCAATTTTATTTCAACATTTATTT.

###### Holotype ♀.

Sector Rincon Rain Forest, Rio Francia Arriba, 10.89666, -85.29003, 400 meters, caterpillar collection date: 04/iii/2012, wasp eclosion date: 17/iii/2012, 1 wasp eclosed.

***Host data*.** Apparently solitary parasitoid of gelJanzen01 Janzen235 (Gelechiidae) feeding on *Crotonschiedeanus* (Euphorbiaceae). Depository: CNC.

***Caterpillar and holotype voucher codes*.** 12-SRNP-40926, DHJPAR0049383.

###### Paratypes.

Host = *Asturodes* fimbriauralisDHJ02 (Crambidae): DHJPAR0052293. Depository: CNC.

###### Etymology.

*Hormiusirenecanasae* is named for Irene Cañas in recognition of her broad-minded attitude about integrating Costa Rican wild biodiversity conservation with industrial geothermia.

**Figure 240.**
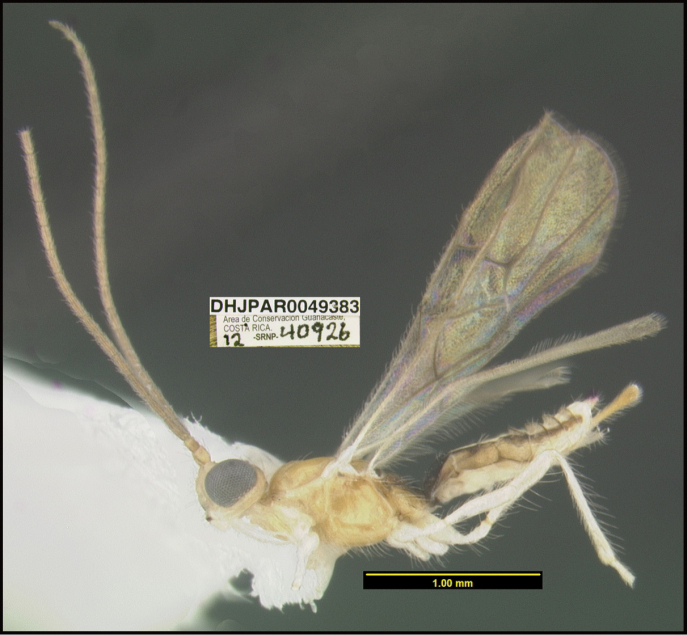
*Hormiusirenecanasae* holotype.

**Figure 241.**
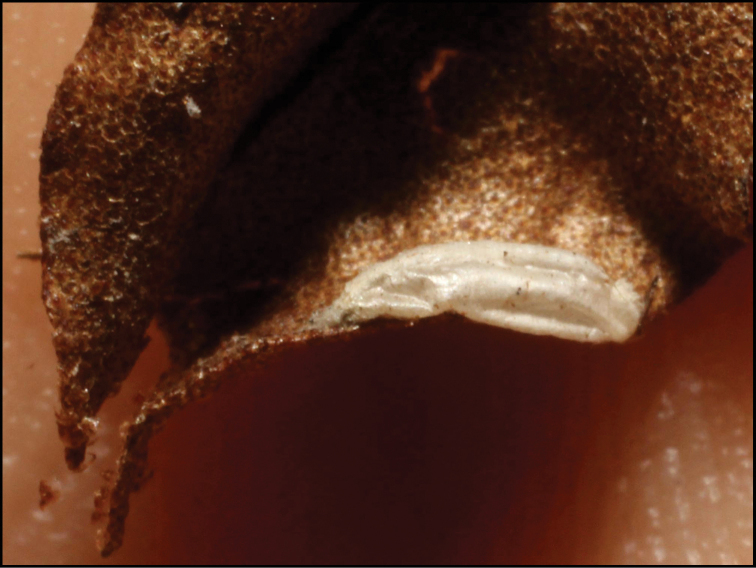
Single cocoon of *Hormiusirenecanasae* of wasp DHJPAR0049383 glued to the leaf nest of its host caterpillar.

##### Hormius isidrochaconi

Taxon classificationAnimaliaHymenopteraBraconidae

Sharkey sp. nov.

http://zoobank.org/99BB873D-19B6-43F9-ACF4-BA639E6581F6

Figures 242 , 243

###### Diagnostics.

BOLD:AAT8859. Consensus barcode. ATTATTATATTTTTTATTTGGGATATGAGCTGGAATAGTTGGTTTATCAATAAGTTTAATTATTCGTTTAGAATTAGGTATACCTGGGAGATTATTAGGTAATGATCAAATTTATAATAGAATCGTTACTGCACATGCTTTTGTAATAATTTTTTTTATAGTTATACCAATTATAATTGGAGGTTTTGGAAATTGATTAGTTCCTTTAATATTAGGTTCACCTGATATAGCTTTTCCACGAATAAATAATATAAGATTTTGGTTATTAGTTCCTTCTTTATTTATATTAATTTTTAGAGGTTTATTAAATGTGGGAGTTGGTACGGGTTGAACTATATATCCTCCTTTATCTTCATTGATTGGTCATGGGGGAGTGTCTGTTGATTTGGCTATTTTTTCTTTACATTTAGCTGGAATTTCTTCTATTATGGGGGCAGTAAATTTTATTTCAACAATTTTAAATATAAAATTATTTAATATAAAATTTGATCAAATTAATTTATTAATTTGATCAATTTTAATTACTGCTATTTTATTATTATTATCTTTACCTGTTTTGGCAGGGGCTATTACTATATTATTAACTGATCGAAATTTAAATACAACATTTTTTGATTTTTCTGGTGGTGGTGATCCAATTTTATTTCAACATTTATTT.

###### Holotype ♀.

Guanacaste, Sector El Hacha, Sendero Bejuquilla, 11.03004, -85.52699, 280 meters, caterpillar collection date: 18/vi/2009, wasp eclosion date: 30/vi/2009. Depository: CNC.

***Host data*.** Gregarious parasitoid of *Antaeotricha* BioLep38 (Depressariidae) feeding on *Arrabidaeachica* (Bignoniaceae).

***Caterpillar and holotype voucher codes*.** 09-SRNP-21577, DHJPAR0041628.

###### Paratypes.

None.

###### Etymology.

*Hormiusisidrochaconi* is named to honor Sr. Isidro Chacon of San José, Costa Rica in recognition of his 40 years of cataloguing and taxonomizing the Lepidoptera of Costa Rica, and supporting the founding and development of INBio, the National Biodiversity Institute of Costa Rica.

**Figure 242.**
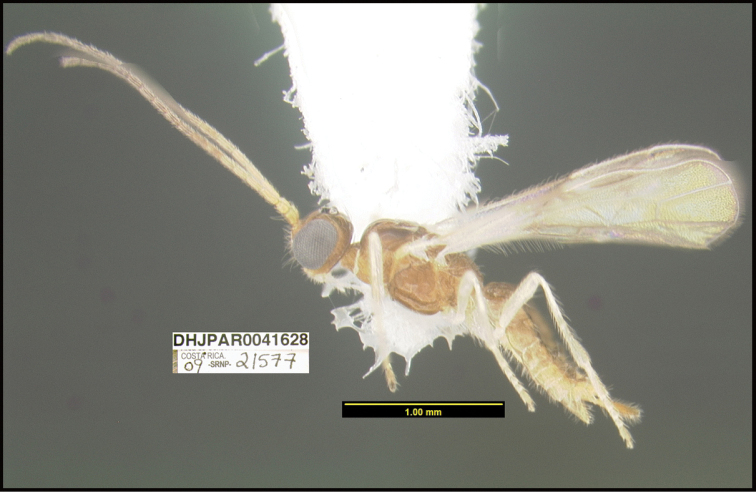
*Hormiusisidrochaconi* holotype.

**Figure 243.**
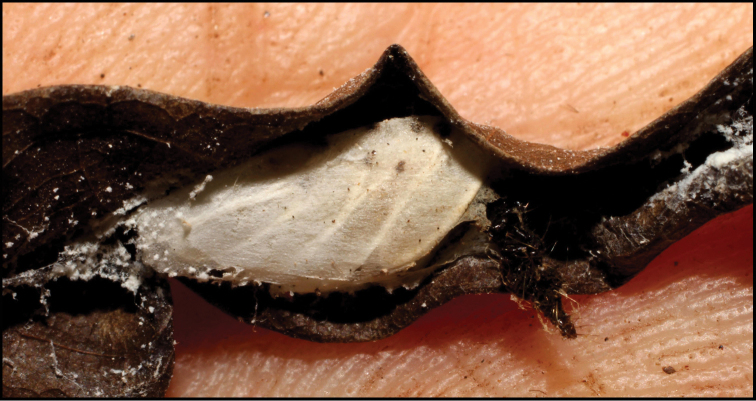
Typical *Hormius* cocoons of *Hormiusisidrochaconi*, tightly adjacent and angled, sandwiched between two leaf surfaces in the caterpillar pupal chamber next to the caterpillar cadaver (09-SRNP-21577).

##### Hormius jaygallegosi

Taxon classificationAnimaliaHymenopteraBraconidae

Sharkey sp. nov.

http://zoobank.org/214A1D23-845A-4131-AFFB-EFE23C3538F0

Figures 244 , 245

###### Diagnostics.

BOLD:ACK6504. Consensus barcode. AGTTTTATATTTTTTATTTGGTATGTGATCTGGAATAGTTGGTTTGTCAATAAGTTTAATTATTCGATTGGAATTAGGTATACCAGGTAGATTATTAGGTAATGATCAAATTTATAATAGAATAGTTACTGCTCATGCTTTTATTATAATTTTTTTTATAGTTATACCTATTATAATTGGTGGATTTGGAAATTGGTTAGTTCCTTTAATAATAGGGTCTCCTGATATAGCTTTTCCACGAATAAATAATATAAGTTTTTGATTATTAATTCCTTCATTAATATTATTAATTTTTAGTGGTTTATTAAATATTGGGGTTGGAACTGGGTGAACAATATATCCTCCTTTATCTTCTCTTATTGGTCATAGAGGAATTTCTGTTGATTTAGCAATTTTTTCTTTACATTTAGCAGGTATTTCTTCAATTATAGGAGCAATTAATTTTATTTCTACAATTTTAAATATAAATTTATATTATATAAAGTTTGATCAAATTAGTTTATTAATTTGGTCTATTTTAATTACTGCTATTTTATTATTATTATCTTTACCTGTTTTAGCGGGAGCTATTACTATATTATTAACAGATCGAAATTTAAATACTACTTTTTTTGATTTTTCTGGAGGTGGGGATCCAATTTTATTTCAACATTTATTT.

###### Holotype ♀.

Guanacaste Sector El Hacha, Sendero Bejuquilla, 11.03004, -85.52699, 280 meters, caterpillar collection date: 17/xii/2003, wasp eclosion date: 08/i/2004, one of 11 wasps that emerged from the same host caterpillar. Depository: CNC.

***Host data*.** Gregarious parasitoid of *Cliniodesopalalis* (Crambidae) feeding on *Daphnopsisamericana* (Thymelaeaceae).

***Caterpillar and holotype voucher codes*.** 03-SRNP-38276, DHJPAR0029001.

###### Paratypes.

Host = *Cliniodesopalalis*: 10 specimens, same data as holotype and DHJPAR0062198, DHJPAR0029014, DHJPAR0029015, DHJPAR0029023, DHJPAR0029025. Depository: CNC.

###### Etymology.

*Hormiusjaygallegosi* is named for Jay Gallegos in recognition of Mesoamerica (IEDO) moral and financial support for the IEDO electric power line highly positive interaction with Sector Mundo Nuevo of ACG.

**Figure 244.**
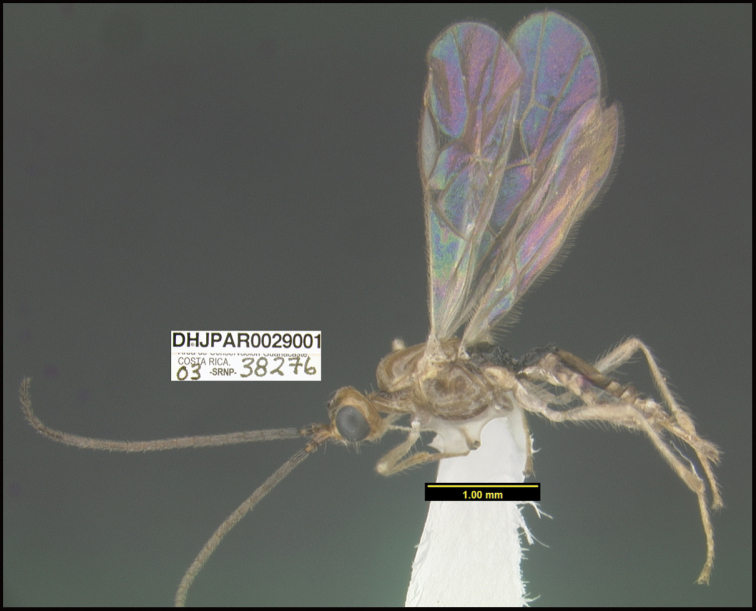
*Hormiusjaygallegosi* holotype.

**Figure 245.**
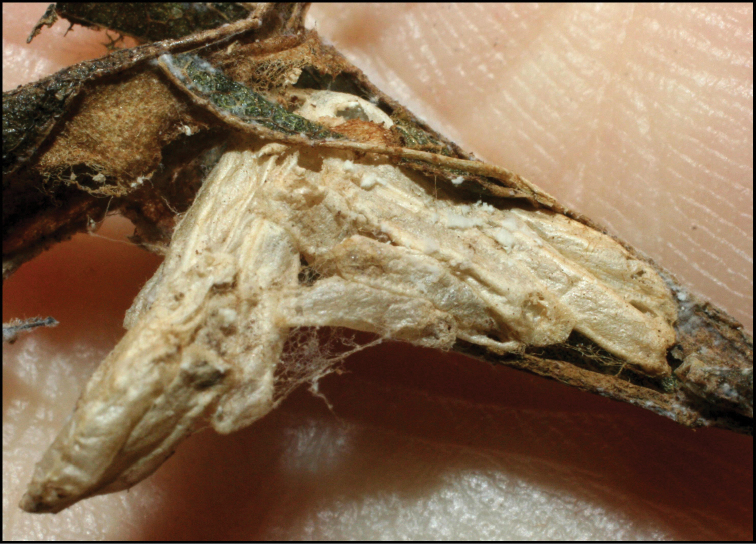
Irregular mass of cocoons of *Hormiusjaygallegosi* (DHJPAR0062198) filling the rolled leaf nest of *Clinodesopalalis*.

##### Hormius jimbeachi

Taxon classificationAnimaliaHymenopteraBraconidae

Sharkey sp. nov.

http://zoobank.org/B5C574B4-17A7-4107-A802-A3A4D4DDF6A8

Figure 246

###### Diagnostics.

BOLD:ADB3326. Consensus barcode. TTTTTATTTGGTATATGGTCTGGAATATTAGGGTTATCAATAAGATTAATTATTCGTTTAGAGTTAGGTATGCCTGGGAGATTATTAGGTAATGATCAAATTTATAATAGAATAGTAACAGCTCATGCATTTGTTATAATTTTTTTTATAGTGATACCAATTATAATTGGAGGATTTGGAAATTGGTTAATTCCTTTAATATTAGGGTCACCTGATATGGCTTTTCCTCGAATAAATAATATAAGGTTTTGATTATTAGTTCCTTCTTTAATATTATTAATTTTTAGGGGATTATTAAATATTGGGGTTGGTACAGGTTGAACTATTTATCCTCCTTTATCTTCTTTAATTGGTCATAGAGGAATTTCAGTTGATTTAGCAATTTTTTCTTTACACTTAGCAGGGGCATCTTCTATTATAGGGGCAATTAATTTTATTACAACTATTTTGAATATAAATTTATATATAAAAATAGATCAAATTAGTTTATTAATTTGATCAATTATAATTACGGCTATTTTATTATTATTATCATTACCTGTATTAGCT------------------------------------------------------------------------------------.

###### Holotype ♀.

Guanacaste, Sector Pailas Dos, PL12-7, 10.7612, -85.3353, 791 meters, Malaise trap, 5/vi/2014. Depository: CNC.

***Host data*.** None.

***Holotype voucher code*.**BIOUG29355-F10.

###### Paratypes.

None.

###### Etymology.

*Hormiusjimbeachi* is named in honor of Jim Beach attending the international NSF-funded planning meeting for the All Taxa Biodiversity Inventory (ATBI) of Terrestrial Systems, and contributing his wisdom to the planning that was the founding of Costa Rica’s national BioAlfa today.

**Figure 246.**
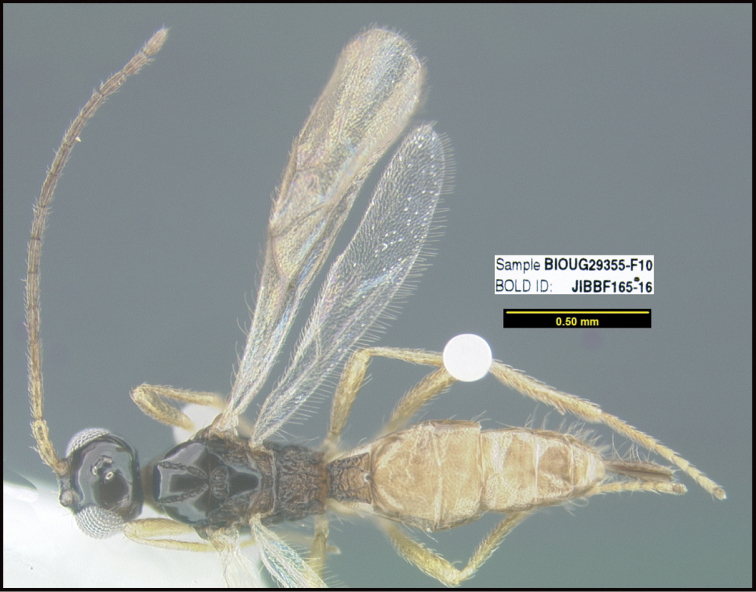
*Hormiusjimbeachi*, holotype.

##### Hormius jimlewisi

Taxon classificationAnimaliaHymenopteraBraconidae

Sharkey sp. nov.

http://zoobank.org/AD9446B8-1288-4EDC-AF23-6F76EC7BC9A7

Figures 247 , 248

###### Diagnostics.

BOLD:AAK5352. Consensus barcode. RRKTTTATATTTTTTATTTGGAATGTGAGCTGGRATAATTGGTTTATCAATGAGTTTAATTATTCGTTTAGAATTAGGAATACCAGGAAGATTRTTGGGTAATGATCAAATTTATAATAGTATAGTAACTGCYCATGCTTTTATTATAATTTTTTTTATAGTTATACCAATTATAATTGGGGGATTTGGTAATTGATTGGTYCCTTTAATAATAGGRGCYCCTGATATGGCTTTYCCTCGAATAAATAATATAAGTTTTTGATTATTAATTCCTTCTTTAATATTATTAATTTTTAGAGGTTTATTGAATATTGGGGTTGGTACTGGTTGAACAATATATCCACCTTTATCTTCATTGATTGGTCATGGAGGAATTTCTGTTGATTTAGCAATTTTTTCTTTACATTTAGCTGGAATTTCTTCAATTATRGGAGCAATTAATTTTATTTCAACAATTTTAAATATAAATTTATATTATATAAAATTTGATCAAATTAGATTATTAATTTGATCAATTTTAATTACTACTATTTTATTATTATTATCATTACCTGTTTTAGCTGGAGCTATTACAATATTATTAACAGATCGTAATTTAAATACTACTTTTTTTGATTTTTCAGGAGGGGGGGATCCAATTTTATTTCAGCATTTATTT.

###### Holotype ♀.

Guanacaste, Sector Santa Rosa, Area Administrativa, 10.83764, -85.61871, 295 meters, caterpillar collection date: 03/xi/1995, wasp eclosion date: 18/xi/1995, one of two wasps that emerged from the same host caterpillar. Depository: CNC.

***Host data*.** Gregarious parasitoid of *Salbiacassidalis* (Crambidae) feeding on *Lasiacissorghoidea* (Poaceae).

***Caterpillar and holotype voucher codes*.** 95-SRNP-10855, DHJPAR0030617.

###### Paratypes.

Hosts = *Salbiacassidalis*, *Orphanostigmahaemorrhoidalis* (Crambidae). 1 specimen same data as holotype, and DHJPAR0030615, DHJPAR0038122, DHJPAR0042956, DHJPAR0047155, DHJPAR0051229. Depository: CNC.

###### Etymology.

*Hormiusjimlewisi* is named in recognition of Jim Lewis’ long years of curating the Hemiptera and other insects of the former INBio national insect inventory.

**Figure 247.**
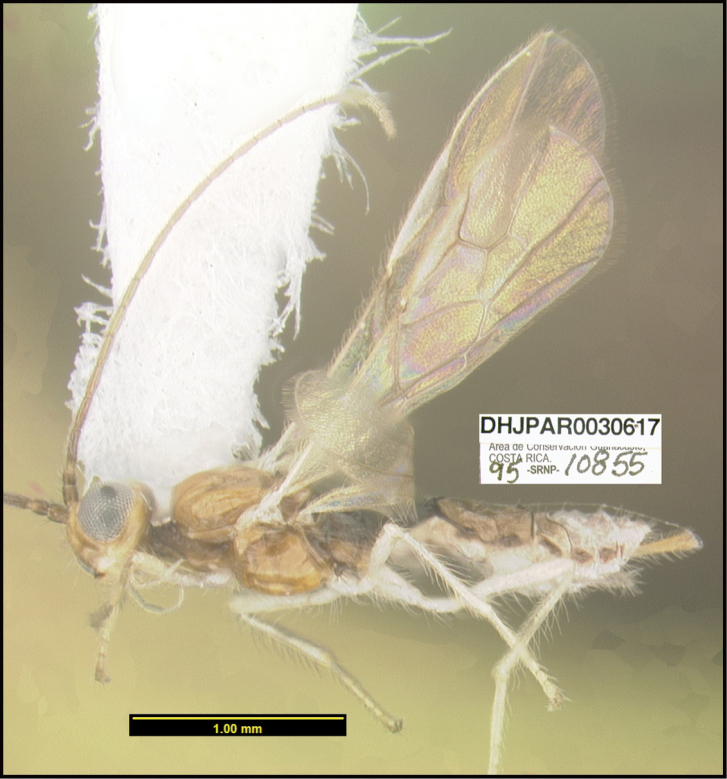
*Hormiusjimlewisi* holotype.

**Figure 248.**
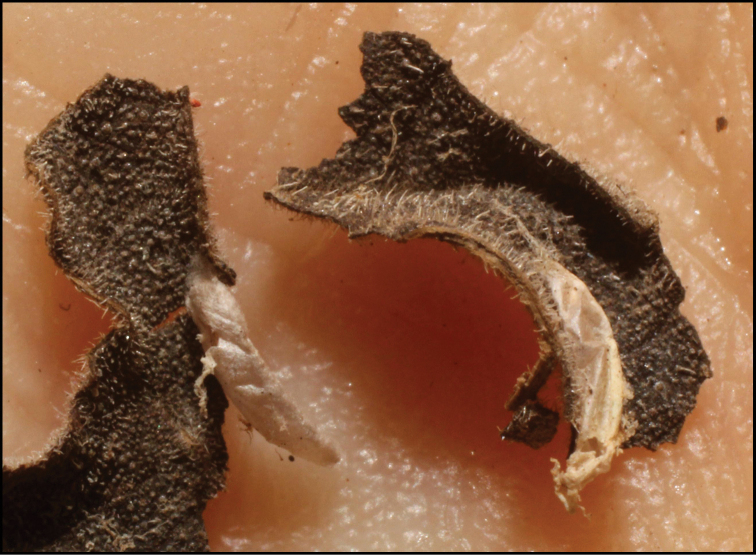
Two white scattered cocoons of *Hormiusjimlewisi* (DHJPAR0047155) glued to the leaf fragments of the larval nest.

##### Hormius joelcracrafti

Taxon classificationAnimaliaHymenopteraBraconidae

Sharkey sp. nov.

http://zoobank.org/BA3B94CB-C04E-44DC-A7FF-8BA71C29BA93

Figure 249

###### Diagnostics.

BOLD:ACX9208. Consensus barcode. ATTTTATATTTTTTATTAGGTATATGATCTGGTATATTAGGTTTATCAATAAGATTAATTATTCGTTTAGAGTTAGGAATACCTGGAAGATTGTTAGGGAATGATCAGATTTATAATAGAATAGTTACTGCACATGCATTTATTATAATTTTTTTTATAGTTATACCAATTATAATTGGTGGATTTGGGAATTGGTTAGTTCCTTTAATATTAGGTTCACCTGATATAGCTTTTCCTCGAATAAATAATATAAGGTTTTGATTATTGATTCCTTCATTAATATTATTAATTTTTAGTAGTTTATTAAATATTGGGGTTGGTACAGGTTGAACTATTTATCCTCCTTTGTCTTCATTAATTGGTCATAGGGGGATTTCAGTTGATATAGCAATTTTTTCTTTACATTTAGCGGGTGTATCTTCTATTATAGGGGCAATTAATTTTATTACTACTATTTTAAATATAAATTTATATATAAAAATAGATCAGATTAGTTTATTAATTTGATCTATTATAATTACAGCAATTTTATTATTATTATCTTTACCAGTTTTGGCTGGAGCTATTACAATACTTTTAACTGATCGGAAT---------------------------------------------.

###### Holotype ♀.

Guanacaste, Sector San Cristobal, Estación San Gerardo, 10.8801, -85.389, 575 meters, Malaise trap, 24/ii/2014. Depository: CNC.

***Host data*.** None.

***Holotype voucher code*.**BIOUG24700-H07.

###### Paratypes.

None.

###### Etymology.

*Hormiusjoelcracrafti* is named in honor of the participation of Joel Cracraft in the international NSF-funded planning meeting for the All Taxa Biodiversity Inventory (ATBI) of Terrestrial Systems, and contributing his wisdom to the planning that was the founding of Costa Rica’s national BioAlfa today.

**Figure 249.**
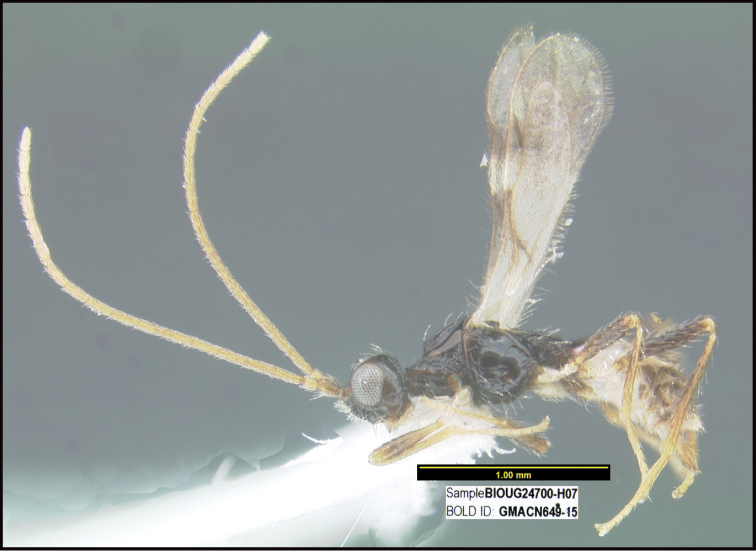
*Hormiusjoelcracrafti*, holotype.

##### Hormius johanvalerioi

Taxon classificationAnimaliaHymenopteraBraconidae

Sharkey sp. nov.

http://zoobank.org/3ED79B6C-D600-4BBA-BC5F-9685E240D355

Figure 250

###### Diagnostics.

BOLD:ACC1525. Consensus barcode. TGTTTTATATTTTTTATTTGGAATATGATCTGGTATAATTGGTTTATCAATAAGATTAATTATTCGTTTAGAATTAGGGATACCGGGAAGATTATTGGGGAATGATCAAATTTATAATAGTATAGTTACAGCTCATGCTTTTATTATAATTTTTTTTATAGTTATACCTATTATAATTGGAGGGTTTGGGAATTGGTTAATTCCATTGATATTAGGATCTCCTGATATAGCTTTTCCTCGAATAAATAATATAAGATTTTGACTATTAATTCCTTCTTTAATATTATTAGTTTTTAGAGGTATTTTAAATATTGGTGTTGGTACAGGTTGAACAATATATCCTCCTTTGTCTTCATTAATTGGTCATGGTGGAATTTCTGTTGATTTAGCTATTTTTTCATTACATTTAGCTGGTATTTCTTCAATTATAGGAGCTATTAATTTTATTTCAACTATTTTAAATATAAATTTATATTATATAAAGTTAGATCAAATTAGATTATTAATTTGATCTATTTTAATTACTGCTATTTTATTATTATTGTCTTTACCTGTTTTAGCTGGTGCAATTACTATATTATTAACTGATCGTAATTTAAATACAACATTTTTCGATTTTTCTGGTGGGGGGGATCCAATTTTATTTCAACATTTATTT.

###### Holotype ♀.

Guanacaste, Sector Pitilla, Sendero Laguna, 10.9888, -85.42336, 680 meters, caterpillar collection date: 22/ix/2012, wasp eclosion date: 06/x/2012, one of five wasps that emerged from the same host caterpillar. Depository: CNC.

***Host data*.** Gregarious parasitoid of *Mictopsichia* Janzen340 (Tortricidae) feeding on *Marcgravianervosa* (Marcgraviaceae).

***Caterpillar and holotype voucher codes*.** 12-SRNP-31369, DHJPAR0050059.

###### Paratypes.

Four specimens, same data as holotype. Depository: CNC.

###### Etymology.

*Hormiusjohanvalerioi* is named in honor of Johan Valerio for his direct role in environmental management of ICE projects in Sector Mundo Nuevo of ACG.

**Figure 250.**
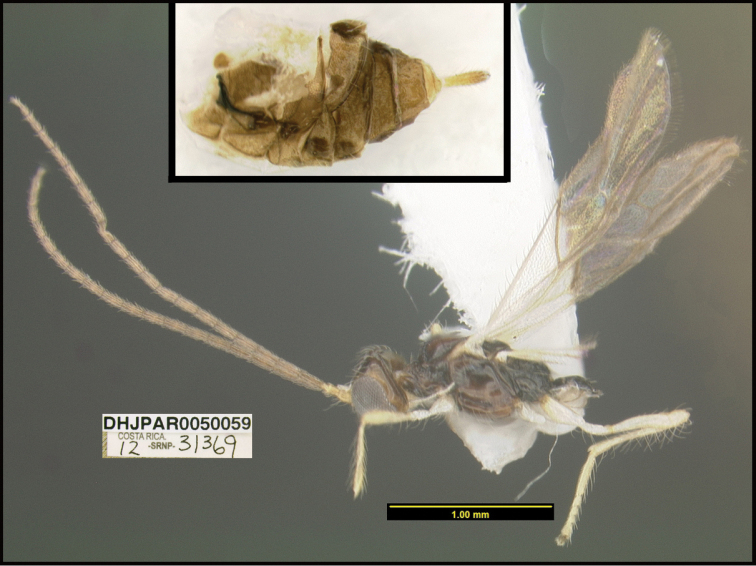
*Hormiusjohanvalerioi*, holotype.

##### Hormius johnburleyi

Taxon classificationAnimaliaHymenopteraBraconidae

Sharkey sp. nov.

http://zoobank.org/EFACFD4C-4B47-4AE0-A110-44A57E9D5183

Figure 251

###### Diagnostics.

BOLD:ADA5377. Consensus barcode. TATTTTATATTTTTTATTTGGAATGTGATCAGGAATATTAGGTTTATCAATAAGATTAATTATTCGTTTAGAATTAGGGATACCTGGTAGATTATTAGGTAATGATCAAATTTATAATAGGATAGTTACTGCTCATGCTTTTATTATAATTTTTTTTATAGTTATGCCAATTATGATTGGGGGATTTGGAAATTGATTAATTCCTTTAATATTAGGATCACCTGATATAGCTTTCCCTCGAATAAATAATATAAGGTTTTGATTATTAATTCCTTCATTAATATTATTAATTTTTAGAGGATTATTAAATATTGGGGTTGGGACGGGATGAACTGTTTATCCTCCATTATCTTCTTTAATTGGTCATAGGGGGATTTCTGTAGATTTAGCAATTTTTTCTTTGCATTTGGCTGGTGCTTCATCAATTATAGGGGCAATTAATTTTATTACTACTATTTTAAATATGAATTTATATATAAAAATAGATCAAATTAATTTATTAATTTGATCTATTATAATTACGGCAATTTTATTATTATTATCATTACCAGTTTTGGCTGGGGCTATTACTATACTTTTAACTGATCGAAATTTAAATACAACTTTTTTTGATTTTTCTGGGGGGGGAGATCCTATTTTGTTTCAACATTTATTT.

###### Holotype ♂.

Alajuela, Sector San Cristobal. Sendero Huerta, 10.88, -85.389, 575 meters, Malaise trap, 28/iv/2014. Depository: CNC.

***Host data*.** None.

***Holotype voucher code*.**BIOUG28377-A03.

###### Paratype.

BIOUG28377-D02. Depository: CNC.

###### Etymology.

*Hormiusjohnburleyi* is named in honor of John Burley attending the international NSF-funded planning meeting for the All Taxa Biodiversity Inventory (ATBI) of Terrestrial Systems, and contributing his wisdom to the planning that was the founding of Costa Rica’s national BioAlfa today.

**Figure 251.**
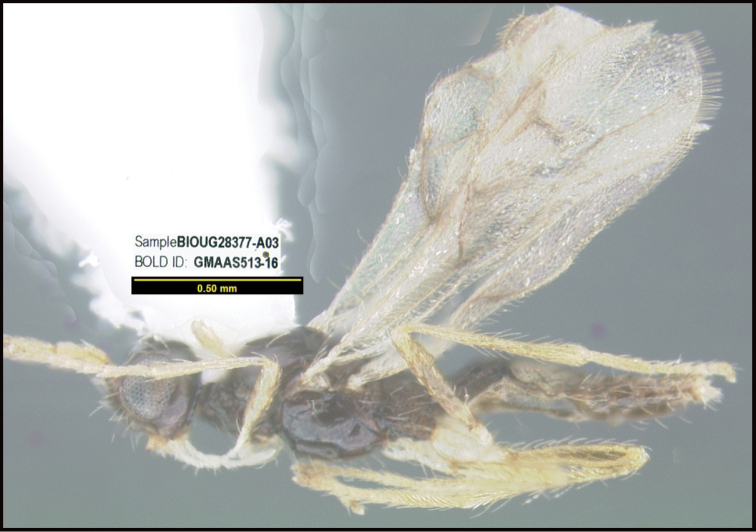
*Hormiusjohnburleyi*, holotype.

##### Hormius joncoddingtoni

Taxon classificationAnimaliaHymenopteraBraconidae

Sharkey sp. nov.

http://zoobank.org/6BD70EC2-D34F-4774-B6B7-D9E48B2584D3

Figure 252

###### Diagnostics.

BOLD:ADA9980. Consensus barcode. AATTTTATATTTTTTATTTGGTATATGATCAGGTATATTAGGTTTATCAATAAGTTTGATTGTTCGTTTAGAGTTAGGAATACCTGGAAGAATATTAGGGAATGATCAAATTTATAATAATATAGTTACTGCTCATGCTTTTATYATAATTTTTTTTATAGTTATACCAATTATAATTGGGGGGTTTGGAAATTGATTAATTCCTTTAATATTAGGGTCTCCTGATATGGCTTTTCCACGAATAAATAATATAAGATTTTGATTATTAATTCCTTCATTAATATTATTAATTTTTAGTGGRTTATTAAATATTGGGGTTGGGACAGGATGAACTATTTATCCTCCATTATCTTCTTTAATTGGACATAGAGGTATTTCTGTTGATTTAGCTATTTTTTCTTTACATTTAGCTGGTGCTTCTTCAATTATAGGGGCAATTAATTTTATTACTACTATTTTAAATATAAATTTATATATAAAAATAGATCAAATTAATTTATTAATTTGATCTATTATAATTACAGCAATTTTATTATTGTTGTCTTTACCAGTTTTAGCTGGTGCYATTACTATACTTTTAACTGATCGAAATTTAAAT.

###### Holotype ♀.

Guanacaste, Sector Pailas Dos, PL12-9, 10.76, -85.3341, 809 meters, Malaise trap, 16/x/2014. Depository: CNC.

***Host data*.** None.

***Holotype voucher code*.**BIOUG29696-G02.

###### Paratypes.

BIOUG29696-F02, BIOUG29696-E09. Depository: CNC.

###### Etymology.

*Hormiusjoncoddingtoni* is named in honor of Jon Coddington for his participation in the international NSF-funded planning meeting for the All Taxa Biodiversity Inventory (ATBI) of Terrestrial Systems, and contributing his wisdom to the planning that was the founding of Costa Rica’s national BioAlfa today.

**Figure 252.**
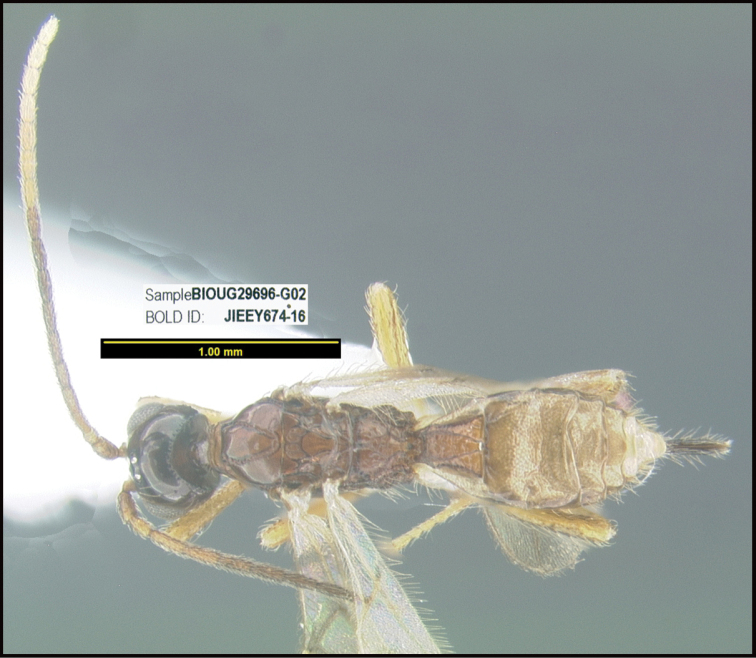
*Hormiusjoncoddingtoni*, holotype.

##### Hormius jorgecarvajali

Taxon classificationAnimaliaHymenopteraBraconidae

Sharkey sp. nov.

http://zoobank.org/39209A1A-40EC-4844-B100-E84809DA8EF0

Figures 253 , 254

###### Diagnostics.

BOLD:AAW4502. Consensus barcode. AGTTTTATATTTTTTATTTGGTATATGATCTGGAATATTAGGGTTATCAATAAGATTAATTATTCGATTAGAATTAGGTATACCTGGAAGATTATTAGGTAATGATCAAATTTATAATAGTATAGTAACTGCTCATGCATTTATTATAATTTTTTTTATAGTTATACCAATTATAATTGGAGGTTTTGGAAATTGATTAATTCCTTTAATATTAGGGTCTCCTGATATAGCTTTTCCTCGTATAAATAATATAAGTTTTTGATTATTAGTTCCTTCTTTAATATTATTAATTTTTAGAGGAATTTTAAATGTAGGGGTTGGTACTGGATGAACTATATATCCTCCTTTATCATCTCTTATTGGTCATAGGGGTATTTCAGTTGATTTAGCTATTTTTTCTTTACATTTAGCTGGTATATCTTCTATTATAGGAGCTATTAATTTTATTTCAACTATTTTAAATATAAAATTATATTATRTAAAATTAGATCAAATTAATTTATTAATTTGATCAATTTTAATTACTGCAATTTTATTATTRTTATCTTTACCTGTATTAGCTGGAGCTATTACAATATTATTAACTGATCGTAATTTAAATACAACTTTTTTTGATTTTTCTGGTGGAGGAGACCCTATTTTATTTCAACATTTATTT.

###### Holotype ♀.

Alajuela, Sector Rincon Rain Forest, Quebrada Escondida, 10.89928, -85.27486, 420 meters, caterpillar collection date: 19/vii/2011, wasp emergence date: 29/vii/2011, one of two wasps that emerged from the same host caterpillar. Depository: CNC.

***Host data*.** Sometimes gregarious parasitoid of *Desmiaoctomaculalis* (Crambidae) feeding on *Notoplurauliginosa* (Rubiaceae).

***Caterpillar and holotype voucher codes*.** 11-SRNP-43374, DHJPAR0055451.

###### Paratypes.

Hosts = *Desmiaploralis*, *Desmiaoctomaculalis*. 1 specimen same data as holotype, and DHJPAR0041689, DHJPAR0051039, DHJPAR0054838, DHJPAR0064146, DHJPAR0064159, DHJPAR0051039, DHJPAR0038269, DHJPAR0038357, DHJPAR0064159 (four specimens do not show in the BOLDBIN because they have relatively short barcodes, but by their host caterpillars and tight clustering in the NJ tree, they are unambiguously *Hormiusjorgecarvajali*). Depository: CNC.

###### Etymology.

*Hormiusjorgecarvajali* is named in honor of Jorge Carvajal, the decades-long logistics facilitator for INBio and now, BioAlfa in its temporary residence in the INBio facilities.

**Figure 253.**
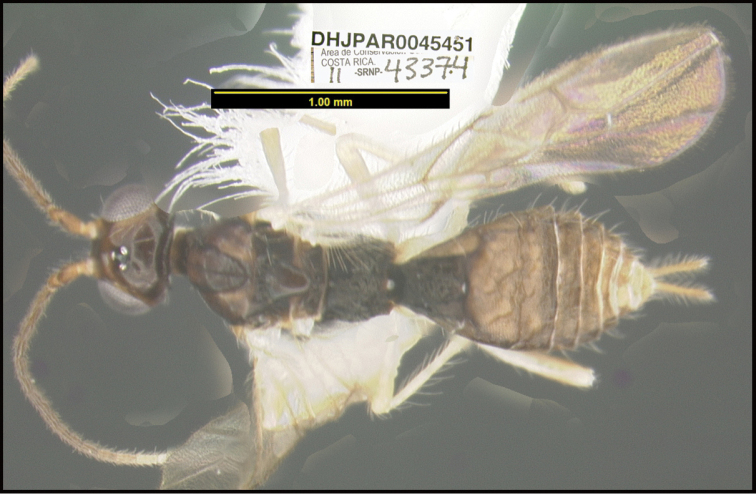
*Hormiusjorgecarvajali*, holotype.

**Figure 254.**
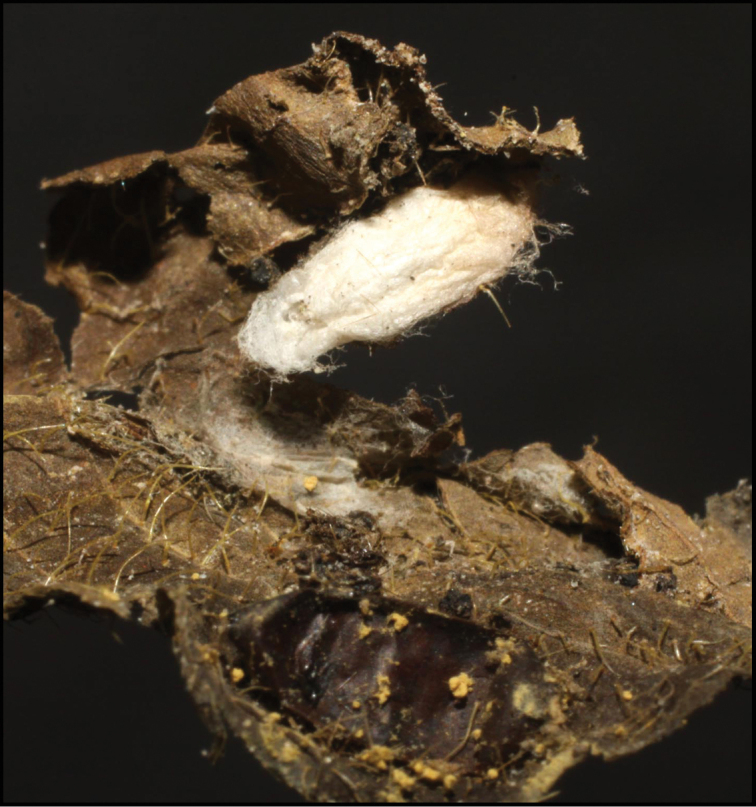
Single white cocoon of *Hormiusjorgecarvajali* (DHJPAR0038357), a variably gregarious species with clutches ranging from one to four parasitoid larvae per caterpillar.

##### Hormius juanmatai

Taxon classificationAnimaliaHymenopteraBraconidae

Sharkey sp. nov.

http://zoobank.org/9670E02D-2E91-43FF-8C07-33998B1598CD

Figures 255 , 256

###### Diagnostics.

BOLD:AAT9025. Consensus barcode. AGTTTTATATTTTTTATTTGGAATRTGATCTGGAATAATTGGTTTATCAATAAGATTAATTATTCGTTTAGAATTAGGAATACCTGGAAGTTTATTAGGTAATGATCAAATTTATAATAGAATAGTTACAGCTCATGCTTTTATTATAATTTTTTTTATAGTTATACCTATTATRATTGGGGGATTTGGAAATTGATTAGTTCCATTAATATTAGGATCACCTGATATRGCTTTTCCTCGAATAAATAATATAAGATTTTGATTATTAGTTCCTTCWTTAATGTTATTAATTTTTAGAGGTATATTAAATATTGGGGTTGGAACAGGTTGAACAATATATCCTCCTTTATCTTCATTAATTGGTCATGGAGGAATTTCTGTTGATTTAGCTATTTTTTCATTACATTTAGCTGGTATTTCTTCTATTATAGGGGCTATTAATTTTATTTCAACAATTTTAAATATAAATTTATATTATATAAAGTTAGATCAAATTAGTTTATTAATTTGATCTATTTTAATTACTGCTATTTTRTTATTATTATCTTTACCAGTTTTAGCTGGAGCTATTACTATATTATTAACTGATCGAAATTTAAATACAACATTTTTTGATTTTTCTGGAGGAGGAGATCCAATTTTATTTCAACATTTATTT.

###### Holotype ♀.

Guanacaste, Sector Mundo Nuevo, Mamones, 10.77074, -85.42874, 365 meters, caterpillar collection date: 10/xii/2010, wasp eclosion date: 28/xii/2010, one of five wasps that emerged from the same host caterpillar. Depository: CNC.

***Host data*.** Gregarious parasitoid of *Palpusia* Solis25 (Crambidae) feeding on *Guettardamacrosperma* (Rubiaceae).

***Caterpillar and holotype voucher codes*.** 10-SRNP-57322, DHJPAR0041552.

###### Paratypes.

Hosts = *Palpusia* Solis25 and *Asturodesfimbriauralis* (Crambidae). 4 specimens, same data as holotype, and DHJPAR0050993. Depository: CNC.

###### Etymology.

*Hormiusjuanmatai* is named to honor Juan Mata of San José, Costa Rica and the former INBio insect collection in Santo Domingo de Heredia, and for his diligent skill in creating images for publications from the INBio collections.

**Figure 255.**
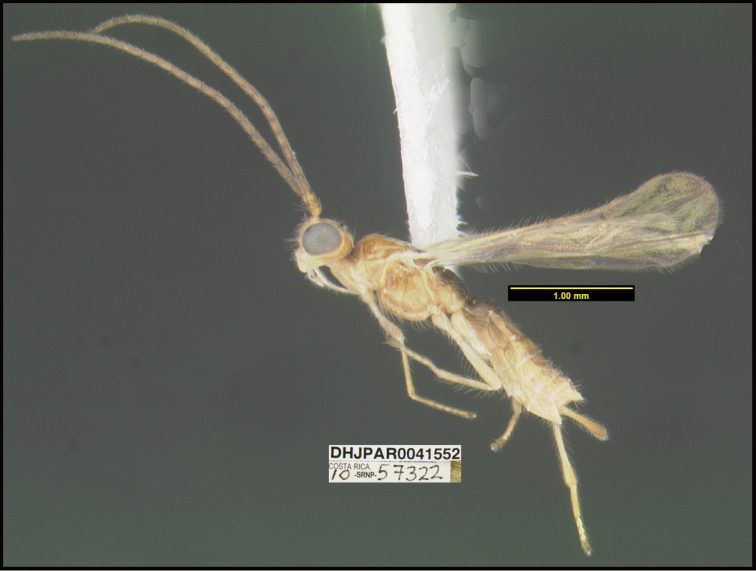
*Hormiusjuanmatai*, holotype.

**Figure 256.**
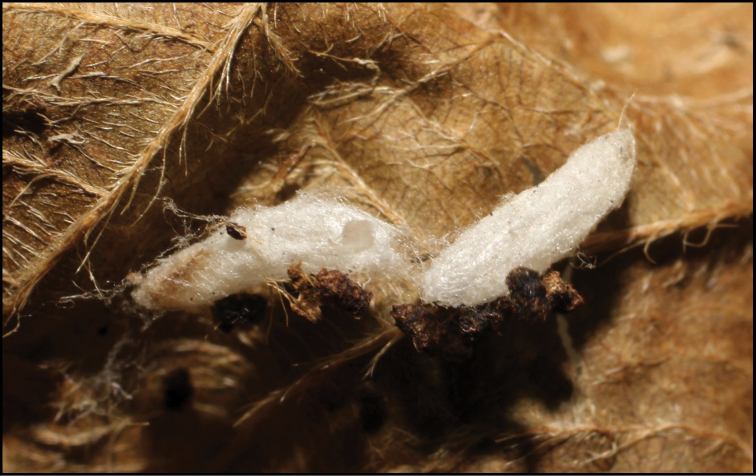
Two separated cocoons of *Hormiusjuanmatai* (DHJPAR0041552) that were tightly but irregularly clustered in the pupal chamber of the ultimate instar caterpillar.

##### Hormius manuelzumbadoi

Taxon classificationAnimaliaHymenopteraBraconidae

Sharkey sp. nov.

http://zoobank.org/D496C3F5-A0C8-445D-8B40-6F382123398B

Figures 257 , 258

###### Diagnostics.

BOLD:AAD0121. Consensus barcode. TGTTTTATATTTTTTATTTGGAATGTGGTCTGGAATAATTGGTTTATCAATAAGATTAATTATTCGTTTAGAATTAGGAATACCTGGAAGTTTATTAGGTAATGATCAAATTTATAATAGTATAGTTACAGCTCATGCTTTTATTATAATTTTTTTTATAGTTATACCTATTATGATTGGAGGTTTTGGAAATTGATTAGTTCCTTTAATATTAGGGTCTCCTGATATGGCTTTTCCTCGAATAAATAATATAAGTTTTTGGTTATTAATTCCTTCTTTAATATTATTGATTTTTAGAGGTATATTAAATATTGGAGTTGGAACAGGTTGAACAATATATCCTCCTTTATCTTCATTAATTGGTCATGGTGGAATTTCTGTTGATTTAGCTATTTTTTCTTTACATTTAGCTGGTATTTCTTCAATTATAGGAGCTATTAATTTTATTTCAACTATTTTAAATATAAATTTATATAATATAAAATTGGATCAAATTAGTTTATTGATTTGATCTATTTTAATTACTGCTATTTTATTATTATTATCTTTACCAGTGTTAGCTGGAGCTATTACTATATTATTAACTGATCGTAATTTAAATACTACTTTTTTTGATTTTTCTGGTGGAGGAGATCCTATTTTATTTCAACATTTATTT.

###### Holotype ♀.

Alajuela, Sector Rincon Rain Forest, Camino Porvenir, 10.90383, -85.25964, 383 meters, caterpillar collection date: 04/v/2012, wasp eclosion date: 24/v/2012, one of 18 wasps that emerged from the same host caterpillar. Depository: CNC.

***Host data*.** Gregarious parasitoid of *Stenoma* Janzen20 (Depressariidae) feeding on leaves of *Miconiaxalapensis* (Melastomataceae).

***Caterpillar and holotype voucher codes*.** 12-SRNP-41874, DHJPAR0049109.

###### Paratypes.

Hosts = elachJanzen01 Janzen01 (Depressariidae) and *Stenoma* Janzen20. 17 specimens, same data as holotype, and DHJPAR0035338, DHJPAR0049128, DHJPAR0049476. Depository: CNC.

###### Etymology.

*Hormiusmanuelzumbadoi* is named in honor of Manuel Zumbado in recognition of his decades of curating Diptera in the Costa Rican national insect collection.

**Figure 257.**
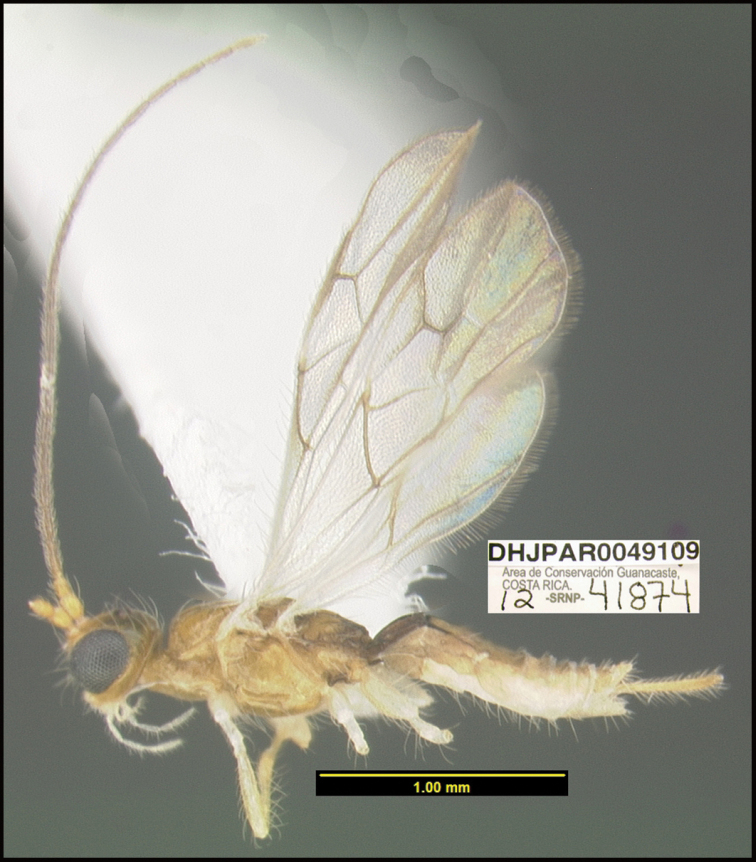
*Hormiusmanuelzumbadoi* holotype.

**Figure 258.**
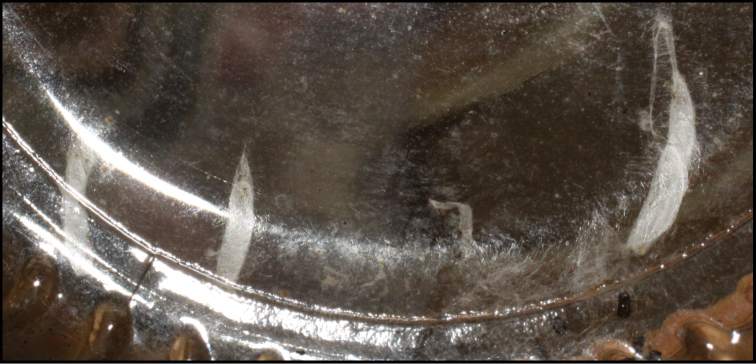
Four white scattered cocoons of *Hormiusmanuelzumbadoi* (DHJPAR0049109) glued to the wall of the caterpillar rearing container.

##### Hormius mercedesfosterae

Taxon classificationAnimaliaHymenopteraBraconidae

Sharkey sp. nov.

http://zoobank.org/526D53F3-A757-4C04-ADEE-AF2568270D66

Figure 259

###### Diagnostics.

BOLD:ACG3568. Consensus barcode. TATTTTATATTTTTTATTTGGAATATGAGCAGGAATATTGGGTTTATCAATAAGATTAATTGTTCGTTTAGAGTTAGGAATACCTGGTAGATTATTAGGTAATGATCAAATTTATAATAGAATAGTTACTGCTCATGCTTTTATTATAATTTTTTTTATAGTTATACCAATTATAATTGGTGGATTTGGAAATTGATTAATTCCTTTAATATTAGGATCTCCTGATATAGCTTTTCCTCGAATAAATAATATAAGTTTTTGATTATTAATTCCTTCATTATTATTATTAATTTTTAGAGGATTATTAAATATTGGGGTTGGAACAGGATGAACTGTTTATCCTCCATTGTCTTCTTTAATTGGTCATAGTGGAATTTCTGTTGATTTAGCAATTTTTTCTTTRCATTTAGCCGGTGCTTCTTCAATTATAGGAGCAATTAATTTTATTACTACTATTTTGAATATAAATTTATATATAAAGATAGATCAAATTAATTTATTGATTTGATCTATTATAATTACGGCGATTTTATTATTATTATCATTACCGGTTTTGGCTGGTGCTATTACTATACTTTTAACTGATCGAAATTTAAATACAACTTTTTTTGATTTTTCGGGAGGAGGAGATCCAATTTTGTTTCAACATTTATTT.

###### Holotype ♀.

Guanacaste, Sector Santa Rosa, Bosque San Emilio, 10.8438, -85.6138, 300 meters, Malaise trap, 29/x/2012. Depository: CNC.

***Host data*.** None.

***Holotype voucher code*.**BIOUG09074-A02.

###### Paratypes.

BIOUG09074-A10, BIOUG09074-A08. Depository: CNC.

###### Etymology.

*Hormiusmercedesfosterae* is named in honor of Mercedes Foster for her participation in the international NSF-funded planning meeting for the All Taxa Biodiversity Inventory (ATBI) of Terrestrial Systems, and contributing her wisdom to the planning that was the founding of Costa Rica’s national BioAlfa today.

**Figure 259.**
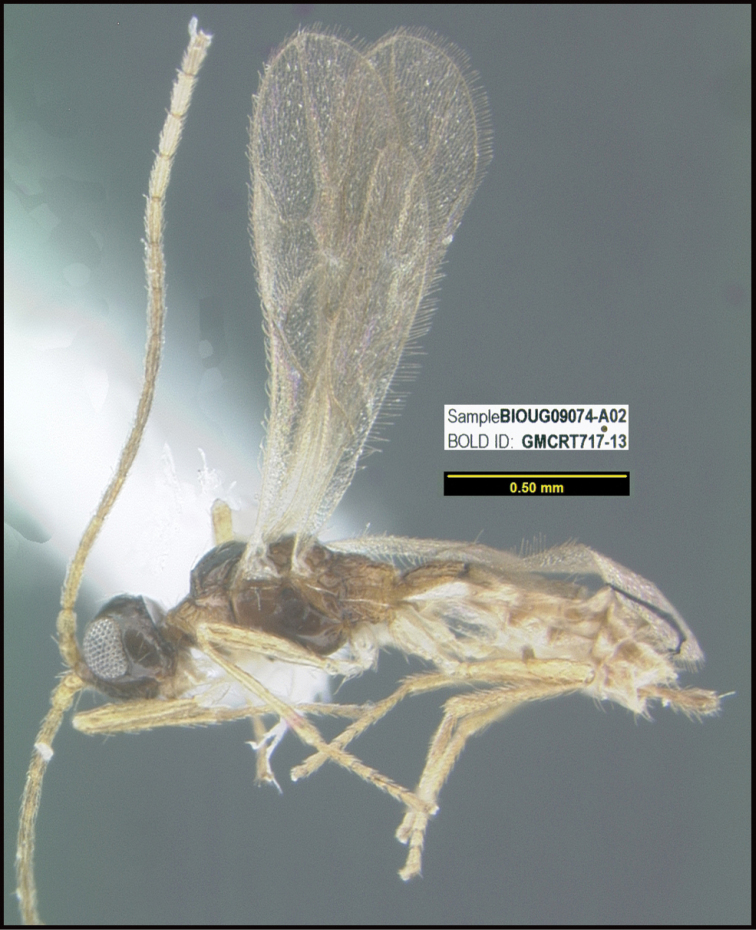
*Hormiusmercedesfosterae*, holotype.

##### Hormius modonnellyae

Taxon classificationAnimaliaHymenopteraBraconidae

Sharkey sp. nov.

http://zoobank.org/A1BDDA86-9B8E-413D-BA44-D85C274E84CF

Figure 260

###### Diagnostics.

BOLD:ADA9991. Consensus barcode. TATTTTATATTTTTTATTTGGAATATGAGCAGGAATATTGGGTTTATCAATAAGATTAATTGTTCGTTTAGAGTTAGGAATACCTGGTAGATTATTAGGTAATGATCAAATTTATAATAGGATAATTACTGCTCATGCTTTCATTATAATTTTTTTTATAGTTATACCAATTATAATTGGTGGATTTGGAAATTGATTAATTCCTTTAATATTAGGGTCTCCTGATATAGCTTTTCCTCGAATAAATAATATAAGTTTTTGATTATTAATTCCTTCATTAGTATTATTAATTTTTAGAGGATTATTAAATATTGGAGGTGGAACAGGATGAACTATTTATCCTCCATTGTCTTCTTTAATTGGTCATACTGGAATTTCTATTGATTTAGCAATTTTTTCTTTACATTTAGCTGGTGCTTCTTCGATTATAGGAGCAATTAATTTTATTACTACTATTTTGAATATAAATTTATATATAAAAATAGATCAAATCAATTTATTGATTTGATCTATTATAATTACGGCGATTTTATTATTATTGTCATTACCGGTTTTAGCTGGTGCTATTACTATACTTTTAACTGATCGAAATTTAAATACA.

###### Holotype ♂.

Guanacaste, Sector Pailas Dos, PL12-3, 10.7631, -85.3344, 820 meters, Malaise trap, 23/x/2014. Depository: CNC.

***Host data*.** None.

***Holotype voucher code*.**BIOUG30201-B10.

###### Paratypes.

BIOUG30201-B07, BIOUG30201-A12. Depository: CNC.

###### Etymology.

*Hormiusmodonnellyae* is named in honor of Mo Donnelly attending the international NSF-funded planning meeting for the All Taxa Biodiversity Inventory (ATBI) of Terrestrial Systems, and contributing her wisdom to the planning that was the founding of Costa Rica’s national BioAlfa today.

**Figure 260.**
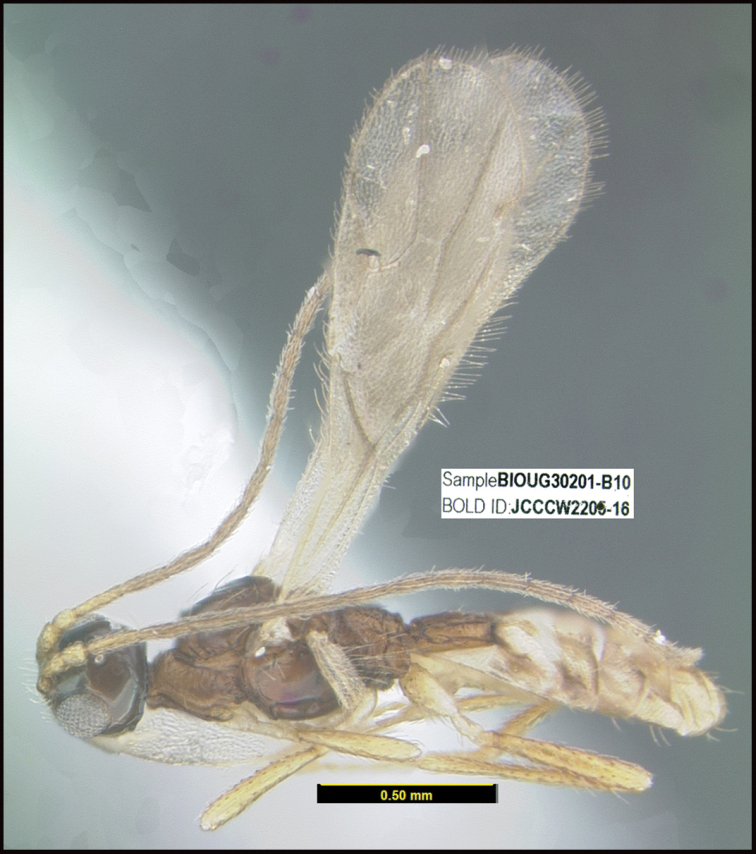
*Hormiusmodonnellyae*, holotype.

##### Hormius nelsonzamorai

Taxon classificationAnimaliaHymenopteraBraconidae

Sharkey sp. nov.

http://zoobank.org/6354E5D1-DCD7-45E7-A32F-7EAAE3E2C000

Figures 261 , 262

###### Diagnostics.

BOLD:AAM1047 Consensus barcode. TTATCAATAAGTTTAATTATTCGATTAGAATTAGGAATACCAGGTAGATTATTAGGTAATGATCAGATTTATAATAGTATAGTAACCGCTCATGCTTTTATTATAATTTTTTTTATAGTTATACCAATTATAATTGGGGGATTTGGAAATTGATTAGTACCTTTAATAATAGGTTCTCCTGATATAGCTTTCCCTCGAATAAATAATATAAGTTTTTGATTATTAATCCCCTCTCTTATATTGTTGATTTTTAGAGGTTTATTAAATATTGGGGTTGGGACTGGTTGGACAATTTATCCTCCTTTGTCTTCATTAATTGGTCATAGAGGAATTTCTGTTGATTTAGCAATTTTTTCTTTACATTTAGCTGGAATTTCTTCAATTATAGGAGCTATTAATTTTATTTCAACAATTTTAAATATAAATTTATATTATATAAAATTTGATCAGATTAGATTATTAATTTGATCTATTTTAATTACTGCTATTTTATTATTATTGTCTTTACCTGTTTTAGCTGGAGCTATTACAATATTATTGACGGATCGAAATTTAAATACAACTTTTTTTGA-------------------------------------------.

###### Holotype ♀.

Alajuela, Sector Rincon Rain Forest, Estación Llanura, 10.93332, -85.25331, 135 meters, caterpillar collection date: 12/vi/2009, wasp eclosion date: 25/vii/2009, one of nine wasps that emerged from the same host caterpillar. Depository: CNC.

***Host data*.** Gregarious parasitoid of *Stenoma* Janzen07feeding on *Vismiabillbergiana* (Hypericaceae).

***Caterpillar and holotype voucher codes*.** 09-SRNP-44582, DHJPAR0035445.

###### Paratypes.

Eight specimens, same data as holotype. Depository: CNC.

###### Etymology.

*Hormiusnelsonzamorai* is named in recognition of Nelson Zamora’s decades of cheerfully and energetically providing high quality identifications for the foods of the caterpillar hosts of the parasitoids of ACG.

**Figure 261.**
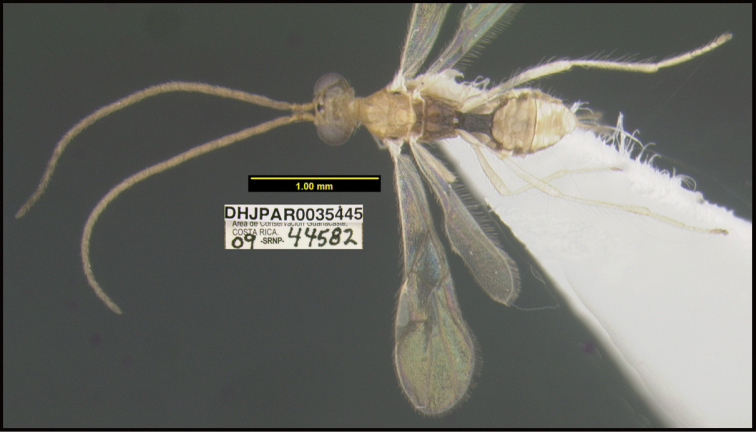
*Hormiusnelsonzamorai* holotype.

**Figure 262.**
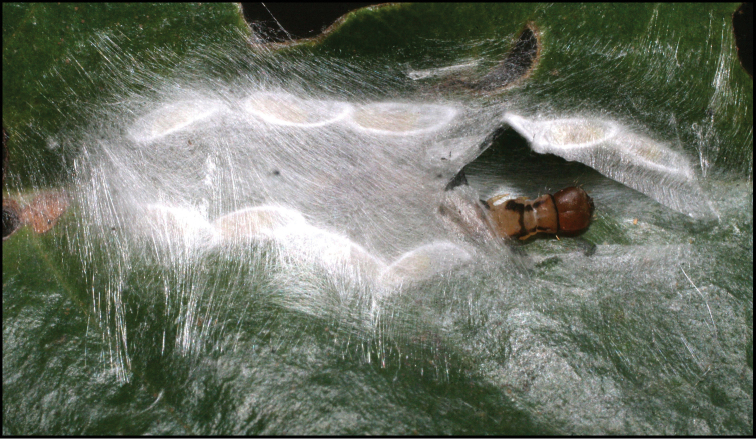
Nine neatly arrayed white cocoons of *Hormiusnelsonzamorai* (DHJPAR0035445) that were glued to the leaf wall of the larval nest, over the cadaver, revealed by stripping off one wall of the collectively spun cover of the cocoons.

##### Hormius pamelacastilloae

Taxon classificationAnimaliaHymenopteraBraconidae

Sharkey sp. nov.

http://zoobank.org/23A4FB89-3E30-48EA-B4F9-0B49B3D0BA00

Figures 263 , 264

###### Diagnostics.

BOLD:AAM1076. Consensus barcode. AATTCTTTATTTTTTATTTGGTATATGAGCAGGAATATTAGGTTTATCAATAAGATTAATTATTCGTTTAGAACTAGGAATACCAGGTAGATTATTAGGGAATGACCAAATTTATAATAGAATAGTTACAGCTCATGCTTTTGTTATAATTTTTTTTATAGTTATACCAGTAATAGTTGGAGGGTTTGGGAATTGATTATTACCTTTAATATTAAGGGCCCCTGATATAGCTTTCCCACGTTTAAATAATATAAGATTTTGGTTATTAATTCCTTCTTTATTTTTATTATTAATAAGAAGAGTATTAAATGTAGGTGTTGGTACTGGATGAACAATATATCCTCCTTTGTCTTCTTCTTTAGGTCATAGGGGTATATCAGTTGATTTAGCTATTTTTTCTTTACATATTGCGGGTATTTCATCAATTTTAGGGGCTATAAATTTTATTTCAACTATTTTTAATATACATTTATTAACTTTAAAATTAGATCAATTAACTTTATTTATTTGATCAATTTTTATTACAACTTTGTTATTATTATTATCTTTACCAGTATTAGCTGGGGCTATTACCATATTATTAACTGATCGAAATTTAAATACTTCTTTTTTTGATTTTTCTGGTGGAGGTGACCCAATTTTATTTCAACATTTATTT.

###### Holotype ♀.

Alajuela, Sector Rincon Rain Forest, Estación Llanura, 10.93332, -85.25331, 135 meters, caterpillar collection date: 11/i/2010, wasp eclosion date: 22/i/2010, one of three wasps that emerged from the same host caterpillar. Depository: CNC.

***Host data*.** Gregarious parasitoid of *Omiodes* humeralisDHJ07 (Crambidae) feeding on leaves of *Ingaoerstediana* (Fabaceae).

***Caterpillar and holotype voucher codes*.** 10-SRNP-75038, DHJPAR0038272.

###### Paratypes.

Two specimens, same data as holotype. Depository: CNC.

###### Etymology.

*Hormiuspamelacastilloae* is named to honor Pamela Castillo in recognition of her direct support of the integration of the BioAlfa process into National System of Conservation Areas (SINAC) of the government of Costa Rica.

**Figure 263.**
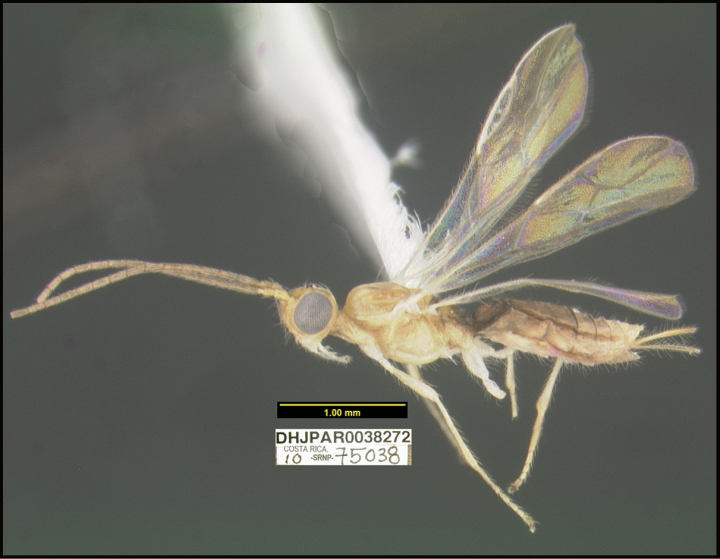
*Hormiuspamelacastilloae* holotype.

**Figure 264.**
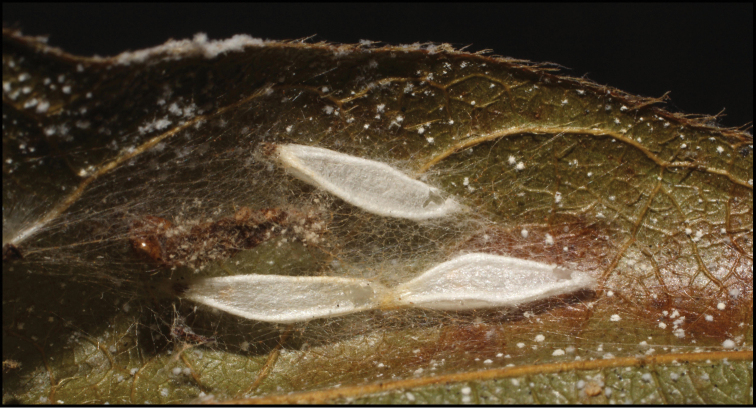
Three separate cocoons of *Hormiuspamelacastilloae* (DHJPAR0038272) that were glued between the overlapping leaves of the nest of the host caterpillar *Omiodes* humeralisDHJ07 (10-SRNP-75038).

##### Hormius raycypessi

Taxon classificationAnimaliaHymenopteraBraconidae

Sharkey sp. nov.

http://zoobank.org/13017926-274D-42F4-A104-2CDABDDB6F7E

Figure 265

###### Diagnostics.

BOLD:ADB7824. Consensus barcode. TTATTATATTTTATTTTTGGAATATGATCAGGTATAGTTGGGTTAGCTATAAGTTTAATTGTACGTTTAGAATTAGGAATACCTGGTAGTTTATTAGGTAATGATCAAATTTATAATAGTATAGTTACAGCGCATGCATTTATTATAATTTTTTTTATAGTGATACCTATTATAATTGGAGGGTTTGGAAATTTTTTAGTTCCTTTAATATTAGGGTCTCCTGATATAGCATTTCCTCGTATAAACAATATAAGGTTTTGATTATTAATTCCTTCATTAATTTTATTAATTTTGAGAGGGATTTTGAATGTAGGAGTGGGGACTGGATGAACTATATATCCTCCATTATCTTCATTAGTAGGTCATAGAGGTATTTCTGTAGATTTATCTATTTTTTCTTTACATTTAGCAGGTATTTCTTCAATTATAGGAGCTATTAATTTTATTACAACTATTTTTAATATAAGTTTATTTTCAATTAAAATAGATCAAATTATGTTATTGATTTGATCTGTGATAATTACTGCTTTTTTATTATTATTATCTTTACCCGTATTAGCGGGAGCAATTACAATATTATTAACTGAT---------------------------------------------.

###### Holotype ♀.

Guanacaste, Sector Pailas Dos, PL12-3, 10.7631, -85.3344, 820 meters, Malaise trap, 30/i/2014. Depository: CNC.

***Host data*.** None.

***Holotype voucher code*.**BIOUG29646-F01.

###### Paratypes.

None.

###### Etymology.

*Hormiusraucypessi* is named in honor of Ray Cypess attending the international NSF-funded planning meeting for the All Taxa Biodiversity Inventory (ATBI) of Terrestrial Systems, and contributing his wisdom to the planning that was the founding of Costa Rica’s national BioAlfa today.

**Figure 265.**
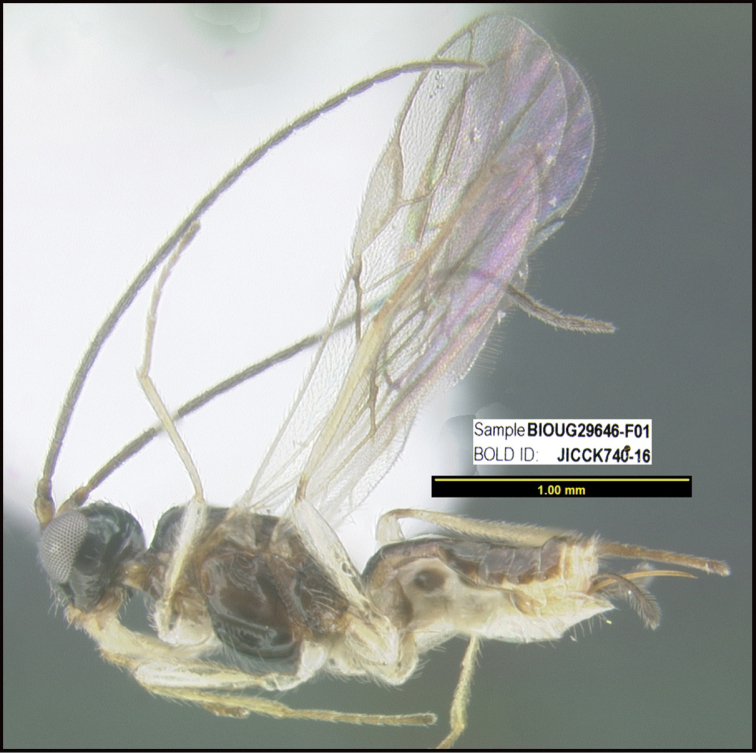
*Hormiusraycypessi*, holotype.

##### Hormius ritacolwellae

Taxon classificationAnimaliaHymenopteraBraconidae

Sharkey sp. nov.

http://zoobank.org/9062A52A-DC7A-4CAE-ACFA-3CC2EBAA508C

Figure 266

###### Diagnostics.

BOLD:ADB4713. Consensus barcode. GTTTTATATTTTTTATTTGGTATATGATCTGGAATAATTGGTTTATCAATAAGTTTAATTATTCGTTTAGAATTAGGGATACCTGGTAGATTATTAGGGAATGATCAAATTTATAATAGAATAGTGACAGCTCATGCTTTTATTATAATTTTTTTTATAGTTATACCTATTATAATTGGAGGATTTGGAAATTGGTTGATTCCATTAATATTGGGATCTCCCGATATAGCTTTTCCTCGAATAAATAATATAAGATTTTGATTATTAATTCCTTCTTTATTATTATTAATTTTTAGAAGAATTTTAAATATTGGTGTTGGAACAGGTTGAACAATATATCCTCCTTTATCTTCATTAATTGGTCATGGTGGAATTTCTGTTGATTTAGCTATTTTTTCATTACATTTGGCTGGTATTTCTTCAATTATAGGAGCTATTAATTTTATTTCAACTATTTTAAATATAAATTTATATTATATAAAATTAGATCAAATTAGATTATTAATTTGATCTATTTTAATTACCGCTATTTTATTGTTATTATCTTTACCTGTTTTGGCTGGTGCAATTACTATATTATTAACTGAT------------------------------.

###### Holotype ♀.

Guanacaste, Sector Pailas Dos, PL12-3, 10.7631, -85.3344, 820 meters, Malaise trap, 9/i/2014. Depository: CNC.

***Host data*.** None.

***Holotype voucher code*.**BIOUG29536-F03.

###### Paratypes.

None.

###### Etymology.

*Hormiusritacolwellae* is named in honor of Rita Colwell attending the international NSF-funded planning meeting for the All Taxa Biodiversity Inventory (ATBI) of Terrestrial Systems, and contributing her wisdom to the planning that was the founding of Costa Rica’s national BioAlfa today.

**Figure 266.**
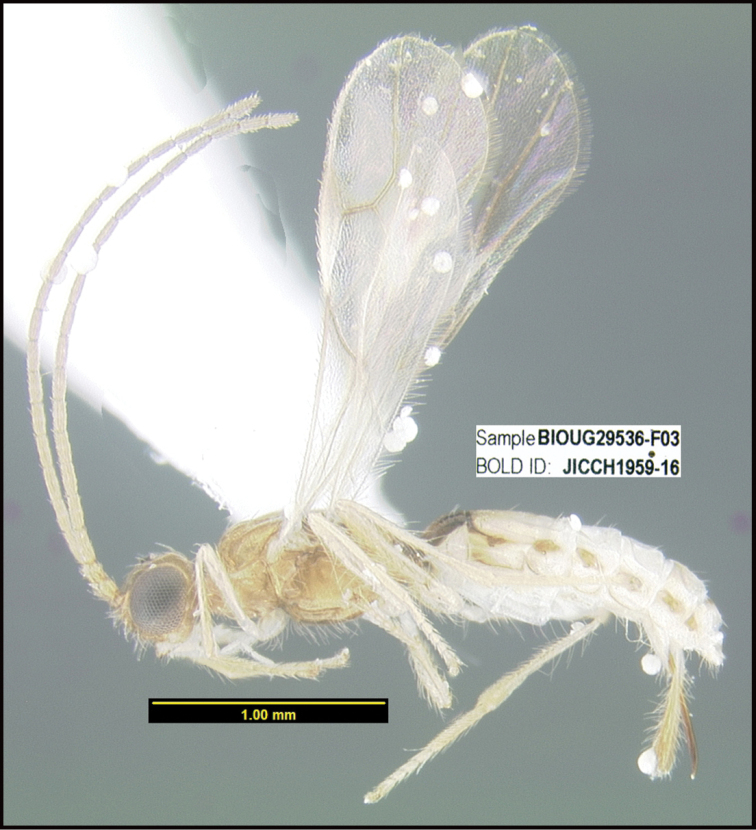
*Hormiusritacolwellae*, holotype.

##### Hormius robcolwelli

Taxon classificationAnimaliaHymenopteraBraconidae

Sharkey sp. nov.

http://zoobank.org/F50AB977-FBED-4D06-9FB7-B88450641ABD

Figure 267

###### Diagnostics.

BOLD:ADZ9590. Consensus barcode. TATTTTTTATTTGGAATATGATCAGGAATATTAGGTTTATCAATAAGATTAATTATTCGTTTAGAATTAGGGATACCTGGTAGATTATTAGGTAATGATCAAATTTATAATAGGATAGTTACTGCTCATGCTTTTATTATAATTTTTTTTATAGTTATACCAATTATGATTGGTGGATTTGGAAATTGATTAATTCCTTTAATATTAGGGGCACCTGATATAGCTTTTCCTCGAATAAATAATATAAGGTTTTGATTATTAATTCCTTCATTAATATTATTAATTTTTAGGGGGTTATTAAATATTGGGGTTGGGACAGGATGAACTGTTTATCCTCCATTATCTTCTTTAATTGGACATAGGGGGATTTCTGTAGATTTAGCAATTTTTTCTTTACATTTAGCTGGTGCTTCATCAATTATAGGAGCAATTAATTTTATTACTACTATTTTAAATATAAATTTATATATAAAAATAGATCAAATTAATTTATTAATTTGATCTATTATAATTACGGCAATTTTATTATTATTATCATTACCAGTTTTGGCTGGT------------------------------------------------------------------.

###### Holotype ♀.

Guanacaste, Sector San Cristobal, Estación San Gerardo, 10.8801, -85.389, 575 meters, Malaise trap, 25/viii/2014. Depository: CNC.

***Host data*.** None.

***Holotype voucher code*.**BIOUG27759-A09.

###### Paratypes.

None.

###### Etymology.

*Hormiusrobcolwelli* is named in honor of Rob Cowell attending the international NSF-funded planning meeting for the All Taxa Biodiversity Inventory (ATBI) of Terrestrial Systems, and contributing his wisdom to the planning that was the founding of Costa Rica’s national BioAlfa today.

**Figure 267.**
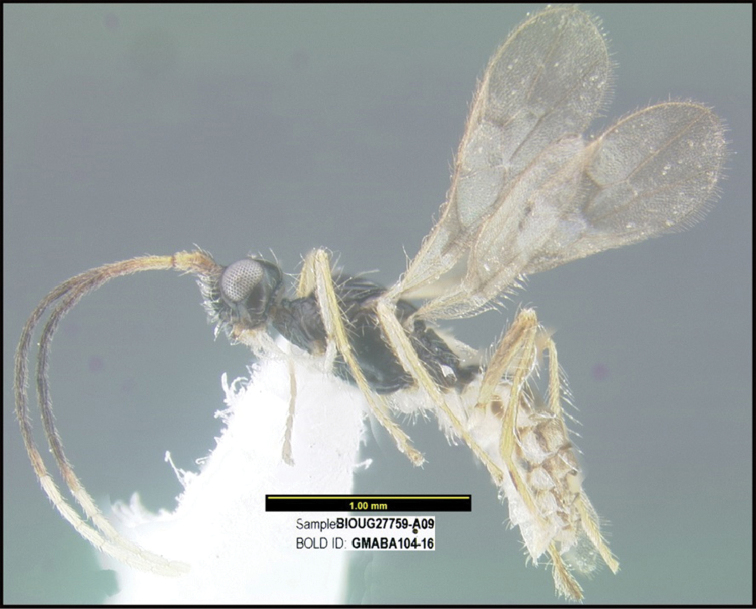
*Hormiusrobcolwelli*, holotype.

##### Hormius rogerblancosegurai

Taxon classificationAnimaliaHymenopteraBraconidae

Sharkey sp. nov.

http://zoobank.org/5C3124C0-F4AF-40EF-9A9B-5B71E72B11E5

Figure 268

###### Diagnostics.

BOLD:ADA9724. Consensus barcode. GTATTATATTTTATTTTTGGTATATGGGCTGGAATAGTGGGKTTATCAATGAGGTTAATTGTTCGTATAGAATTAGGTATGCCTGGGAGGTTATTAGGYAATGATCAAATTTATAATAGAATAGTTACTGCTCATGCATTTATTATAATTTTTTTTATAGTTATACCTATTATGATTGGGGGATTTGGTAAYTTTTTAATTCCTTTAATATTAGGGGCTCCTGATATGGCTTTCCCTCGTATAAATAATATAAGTTTTTGATTGTTGATTCCTTCRTTAATTTTATTAATTTTAAGGGGAATTTTAAATACTGGGGTGGGGACAGGGTGAACTATATATCCYCCTTTGTCTTCATTATTGGGSCATGGGGGRGTTTCTGTAGATTTAGCAATTTTYTCTTTACATTTAGCTGGGGTYTCTTCAATCATAGGGGCTATTAATTTTATTACAACTATTTTTAATATAAATTTATTTTCAATTAAAATAGATCAAATTATATTGTTAATTTGGTCAGTGATGATTACTGCTTTTTTATTAYTGTTATCTTTACCAGTATTGGCAGGGGCTATTACAATATTATTAACAGATCGTAATTTAAATACTAGGTTTTTT.

###### Holotype ♀.

Guanacaste, Sector Pailas Dos, PL12-3, 10.7631, -85.3344, 820 meters, Malaise trap, 26/xii/2013. Depository: CNC.

***Host data*.** None.

***Holotype voucher code*.**BIOUG29456-G12.

###### Paratypes.

BIOUG28748-C02, BIOUG29537-H06. Depository: CNC.

###### Etymology.

*Hormiusrogerblancosegurai* is named in honor of Roger Blanco Segura attending the international NSF-funded planning meeting for the All Taxa Biodiversity Inventory (ATBI) of Terrestrial Systems, and contributing his wisdom to the planning that was the founding of Costa Rica’s national BioAlfa today.

**Figure 268.**
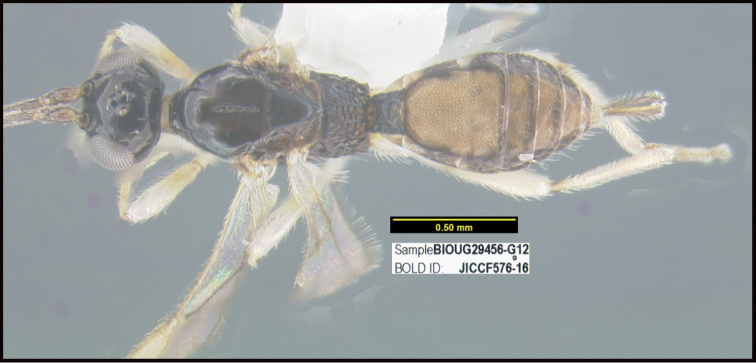
*Hormius.rogerblancosegurai*, holotype.

##### Hormius ronaldzunigai

Taxon classificationAnimaliaHymenopteraBraconidae

Sharkey sp. nov.

http://zoobank.org/0B78F9A1-0598-4979-9029-9553D9D7D69E

Figures 269 , 270

###### Diagnostics.

BOLD:AAA5366. Consensus barcode. AATTTTATATTTTTTATTTGGTATATGATCTGGTATAGTTGGTTTATCAATAAGATTAATTATTCGGTTAGAATTAGGAATACCAGGAAGATTATTAGGTAATGATCAAATTTATAACAGTATAGTAACCGCTCATGCTTTTATTATAATTTTTTTTATAGTTATACCAATTATAATTGGCGGATTCGGAAATTGATTAATTCCTTTAATAATAGGGTCTCCTGATATAGCTTTCCCTCGAATAAATAATATAAGTTTTTGGTTATTAGTTCCTTCATTAATGTTATTAATTTTTAGTGGGTTATTAAATATTGGAGTGGGGACTGGGTGAACTATATATCCTCCATTATCTTCATTAATTGGACATAGTGGGATTTCAGTTGATTTAGCTATTTTTTCTTTACATTTAGCAGGGGTTTCATCAATTATGGGGGCTATTAATTTTATTTCTACAATTTTAAATATAAATTTATATAATATGAAATTTGATCAAATTAGTTTATTAATTTGGTCTATTTTAATTACTGCAATTTTACTTTTATTATCTCTTCCCGTTTTAGCAGGTGCAATTACTATGTTATTAACAGATCGGAATTTAAATACAACTTTTTTTGATTTTTCTGGAGGGGGTGACCCTATTTTATTTCAACATTTATTT.

###### Holotype ♀.

Guanacaste, Sector San Cristobal, Tajo Angeles, 10.86472, -85.41531, 540 meters, caterpillar collection date: 19/x/2009, wasp eclosion date: 11/xi/2009, one of three wasps that emerged from the same host caterpillar. Depository: CNC.

***Host data*.** Gregarious parasitoid of *Antaeotrichafascicularis* (Depressariidae) feeding on *Pouteriaviridis* (Sapotaceae)

***Caterpillar and holotype voucher codes*.** 09-SRNP-5487, DHJPAR0038086.

###### Paratypes.

Host = *Chlamydastistryphon* (Depressariidae), 2 wasps, same data as holotype, and DHJPAR0029024. Depository: CNC.

###### Etymology.

*Hormiusronaldzunigai* is named for Ronald Zuñiga in recognition of his decades of curating Hymenoptera of Costa Rica.

**Figure 269.**
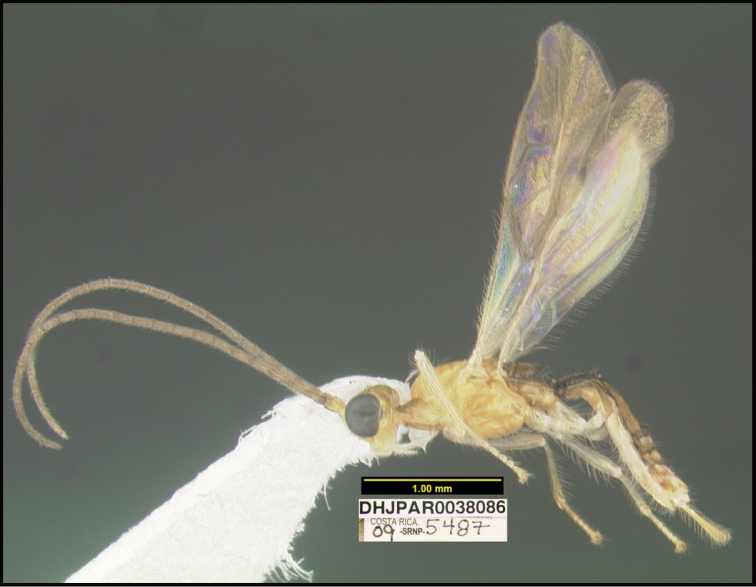
*Hormiusronaldzunigai*, holotype.

**Figure 270.**
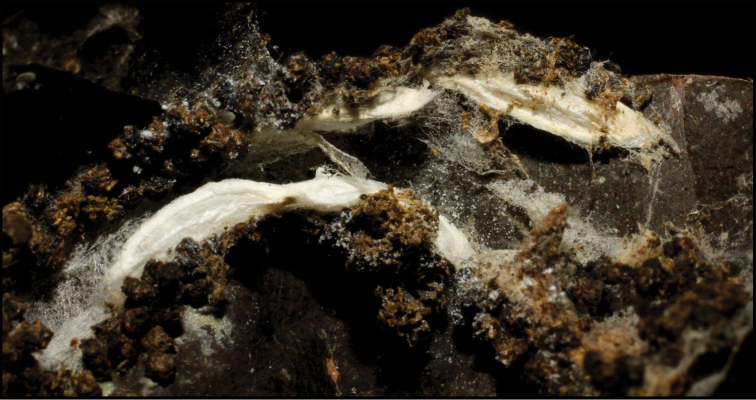
Dispersed white cocoons of *Hormiusronaldzunigai* (DHJPAR0038086) in the remains of the caterpillar nest of leaves spun together with light silk.

##### Hormius russchapmani

Taxon classificationAnimaliaHymenopteraBraconidae

Sharkey sp. nov.

http://zoobank.org/AC9649BB-E0CF-440E-AB14-95FF31674EDF

Figure 271

###### Diagnostics.

BOLD:ACG4356. Consensus barcode. GTATTTTATATTTTTTATTAGGAATATGGTCTGGAATATTAGGATTATCAATAAGTTTAATTGTTCGGTTAGAATTAGGTATACCAGGAAGATTATTAGGGAATGATCAAATTTATAATAGAATAGTAACTGCACATGCATTTATTATAATTTTTTTTATAGTGATACCAATTATAATTGGTGGATTTGGAAATTGATTAGTTCCTTTAATATTAGGGGTGCCTGATATAGCTTTTCCTCGAATAAATAATATAAGTTTTTGATTATTAATTCCTTCATTAATGTTATTGATTTTTAGAAGTTTATTAAATATTGGGGTTGGTACTGGTTGAACTATTTATCCTCCTTTATCTTCTTTAATTGGTCATAGAGGAATTTCAGTTGATTTAGCAATTTTTTCATTACATTTAGCAGGTGCTTCATCAATTATAGGAGCAATTAATTTTATTACTACTATTTTAAATATAAATTTATATATAAAAATAGATCAAATTAGTTTATTAATTTGATCTATTATAATTACAGCTATTTTATTATTGTTATCTTTACCAGTTTTAGCTGGAGCTATTACTATACTTTTAACTGATCGAAATTTGAATACTACTTTTTTTGATTTTTCTGGGGGAGGGGATCCTATTTTATTTCAACATTTATTT.

###### Holotype ♀.

Guanacaste, Sector Santa Rosa Bosque San Emilio, 10.8438, -85.6138, 300 meters, Malaise trap, 25/vi/2012. Depository: CNC.

***Host data*.** None.

***Holotype voucher code*.**BIOUG05195-E11.

###### Paratype.

BIOUG10677-B04. Depository: CNC.

###### Etymology.

*Hormiusrusschapmani* is named in honor of Russ Chapman attending the international NSF-funded planning meeting for the All Taxa Biodiversity Inventory (ATBI) of Terrestrial Systems, and contributing his wisdom to the planning that was the founding of Costa Rica’s national BioAlfa today.

**Figure 271.**
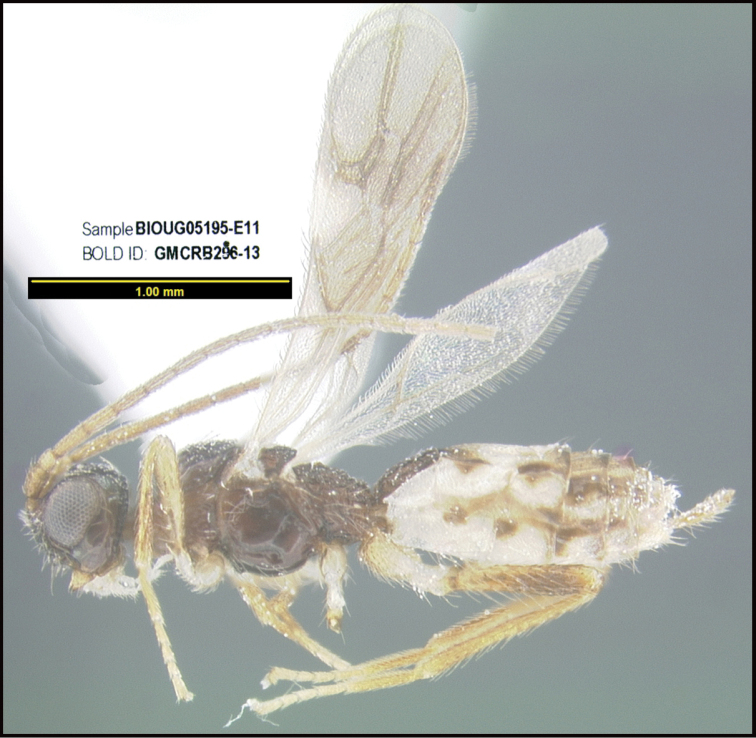
*Hormiusrusschapmani*, holotype.

##### Hormius virginiaferrisae

Taxon classificationAnimaliaHymenopteraBraconidae

Sharkey sp. nov.

http://zoobank.org/20FD829F-C5EF-430E-9914-AD7DBF17BDED

Figure 272

###### Diagnostics.

BOLD:ADB9401. Consensus barcode. GTATTATATTTTTTGTTTGGAATATGAGCTGGTATATTAGGGTTATCAATAAGTTTAATTGTTCGGTTAGAATTAGGTATACCTGGGAGTTTATTAGGTAATGATCAAATTTATAATAGAATAGTTACAGCTCATGCTTTTATTATAATTTTTTTTATAGTGATACCAATTATAATTGGTGGGTTTGGAAATTGATTGGTTCCTTTAATATTGGGGTCTCCTGATATAGCTTTCCCTCGTATAAATAATATAAGTTTTTGGTTATTGATTCCTTCATTAATATTATTAATTTTTAGGGGTGTTTTAAATACGGGCGTCGGCACTGGATGAACAGTTTATCCTCCATTATTTCATTAATTGGTCACAGAGGTATTTCAGTTGATTTAGCAATTTTTTCTTTACATTTAGCTGGTGCTTCATTATTATAGGGGCTATTAATTTTATTACTACTATTTTAAATATAAATTTATATATAAAAATAGATCAAATTAGTTTATTAATTTGGTCAATTATGATTACGGCTGTTTTATTATTATTATCTTTACCTGTGTTAGCAGGAGCTATCACGATACTTTTAACTGAT------------------------------------------------.

###### Holotype ♀.

Guanacaste, Sector Pailas Dos, PL12-3, 10.7631, -85.3344, 820 meters, Malaise trap, 23/x/2014. Depository: CNC.

***Host data*.** None.

***Holotype voucher code*.**BIOUG30103-A11.

###### Paratypes.

None.

###### Etymology.

*Hormiusvirginiaferrisae* is named in honor of Virginia Ferris attending the international NSF-funded planning meeting for the All Taxa Biodiversity Inventory (ATBI) of Terrestrial Systems, and contributing her wisdom to the planning that was the founding of Costa Rica’s national BioAlfa today.

**Figure 272.**
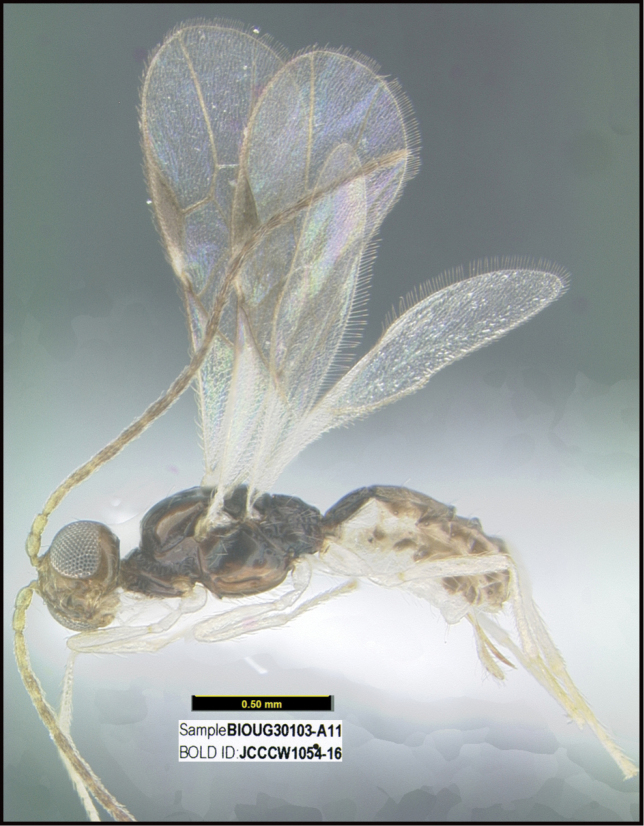
*Hormiusvirginiaferrisae*, holotype.

##### Hormius warrenbrighami

Taxon classificationAnimaliaHymenopteraBraconidae

Sharkey sp. nov.

http://zoobank.org/60669C74-8C05-4CFA-B6FB-D3E7FBF0D6C2

Figure 273

###### Diagnostics.

BOLD:ACS0289. Consensus barcode. TTTTTATTTGGGATGTGATCTGGAATGTTAGGTTTATCAATAAGTTTAATTGTTCGTTTAGAATTAGGAATACCAGGAAGTTTATTAGGTAATGATCAAATTTATAATAGAATAGTTACAGCTCATGCTTTTATTATAATTTTTTTTATAGTAATGCCAATTATAATTGGGGGGTTTGGAAATTGATTAATTCCTTTAATATTAGGTTCACCTGATATAGCTTTTCCTCGAATAAATAATATGAGGTTTTGACTTTTAGTTCCTTCTTTAATATTATTAATTTTTAGAGGATTTTTAAATACTGGAGTAGGTACAGGTTGAACAATTTATCCTCCTTTATCTTCTTTAATTGGTCATGGTGGAATTTCTGTAGATTTGGCAATTTTTTCTTTACATCTTGCTGGAATTTCTTCAATTATAGGTGCAATTAATTTTATTACAACTATTTTGAATATAAATTTATATTTAAAAATAGATCAAATTAGTTTATTAATTTGATCAATTATAATTACAGCTATTTTATTATTATTATCTTTACCTGTTTTAGCAGGTGCTATTACTATATTATTGACAGATCGT------------------------------.

###### Holotype ♂.

Guanacaste, Sector Santa Rosa Bosque San Emilio, 10.8438, -85.6138, 300 meters, Malaise trap, 28/v/2012. Depository: CNC.

***Host data*.** None.

***Holotype voucher code*.**BIOUG18764-G08.

###### Paratypes.

None.

###### Etymology.

*Hormiuswarrenbrighami* is named in honor of Warren Brigham attending the international NSF-funded planning meeting for the All Taxa Biodiversity Inventory (ATBI) of Terrestrial Systems, and contributing his wisdom to the planning that was the founding of Costa Rica’s national BioAlfa today.

**Figure 273.**
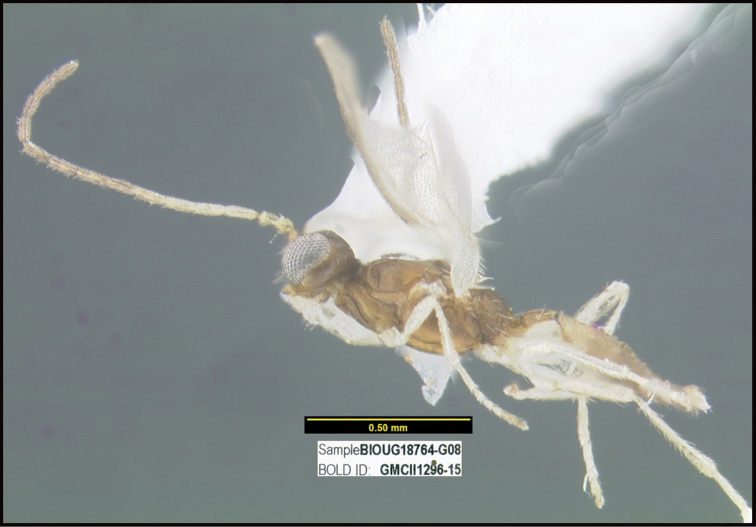
*Hormiuswarrenbrighami*, holotype.

##### Hormius willsflowersi

Taxon classificationAnimaliaHymenopteraBraconidae

Sharkey sp. nov.

http://zoobank.org/60016047-6715-4C95-AC27-64878FB5F302

Figure 274

###### Diagnostics.

BOLD:ADB9850. Consensus barcode. TTTTTATTTGGAATATGATCTGGAATAGTTGGTTTATCTATAAGTTTAATTATTCGTTTGGAATTAGGAATACCAGGTAGATTATTAGGAAATGATCAAATTTATAATAGTATAGTAACCGCTCATGCTTTTATTATAATTTTTTTTATAGTAATACCTATTATAATTGGGGGATTTGGAAATTGATTAATTCCTTTAATAATAGGGGCTCCTGATATAGCTTTTCCTCGAATAAATAATATAAGTTTTTGATTATTAATTCCTTCTTTAATATTATTAGTTTTTAGTGGTTTATTAAATATTGGTGTTGGAACTGGTTGAACAATATATCCTCCTTTATCATCTTTAATTGGACATGGAGGAATTTCTGTTGATTTAGCAATTTTTTCTTTACATTTGGCTGGTATTTCTTCAATTATGGGAGCAATTAATTTTATTTCTACAATTTTAAATATAAATTTATATTATATAAAATTTGATCAAATTAGTTTATTAATTTGATCAATTTTAATTACTGCTATTTTATTATTATTATCTTTACCTGTTTTAGCTGGG---------------------------------------------------------------------------------.

###### Holotype ♀.

Guanacaste, Sector Pailas Dos, PL12-3, 10.7631, -85.3344, 820 meters, Malaise trap, 28/viii/2014. Depository: CNC.

***Host data*.** None.

***Holotype voucher code*.**BIOUG29991-F05.

###### Paratypes.

None.

###### Etymology.

*Hormiuswillsflowersi* is named in honor of Wills Flowers attending the international NSF-funded planning meeting for the All Taxa Biodiversity Inventory (ATBI) of Terrestrial Systems, Philadelphia, USA, and contributing his wisdom to the planning that was the founding of Costa Rica’s national BioAlfa today.

**Figure 274.**
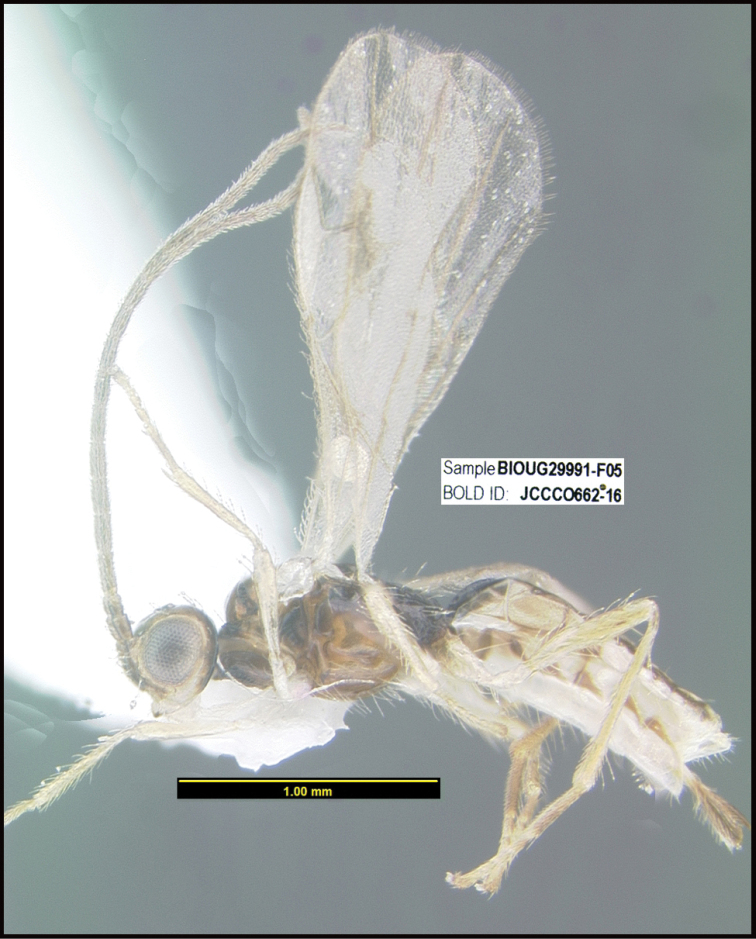
*Hormiuswillsflowersi*, holotype.

## Chapter 7: Ichneutinae

The subfamily Ichneutinae is treated here as comprising the tribes Ichneutini Foerster, 1862 and Muesbeckiini Mason, 1969. The tribe Proteropini van Achterberg, 1976 was removed from Ichneutinae and elevated to subfamily rank by Chen and van Achterberg (2019). This separation is the result of numerous phylogenetic analyses that do not resolve the Ichneutinae s. l. as monophyletic, e.g., Sharanowski et al. (2011). A diagnosis for Ichneutinae follows: mouthparts normal, not exodont nor cyclostome; occipital carina absent; vein M of forewing sharply curved where it meets the parastigma; vein R of forewing not reaching wing margin or intersecting margin well before the apex of the wing. There are five New World genera including *Pseudichneutes* Belokobylskij, 1996, which is here reported in the New World for the first time. All members are solitary koinobiont endoparasites. Ichneutini (*Ichneutes* and *Pseudichneutes*) attack tenthredinid sawflies (Belokobylskij 1996; Ohnesorge 1960); the biology of the genera is reviewed by Shaw and Huddleston (1991). Muesbeckiini (*Lixpixys*, *Oligoneurus*, *Paroligoneurus*) attack leaf-mining Lepidoptera; all three genera are all known from the Neotropical realm, but none has been previously recorded from Costa Rica. For the Ichneutinae NJ tree, see Suppl. material 6.

### Key to the New World genera of Ichneutinae

**Table:** 

1	A. Venation reduced, forewing RS not reaching wing margin; widespread	**2**
–	B. Venation complete, or nearly so, forewing RS reaching wing margin; Nearctic	**4**
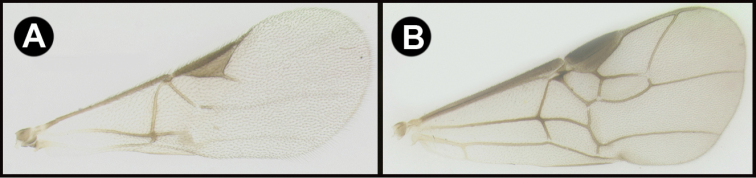		
2(1)	A. Apical flagellomere narrower with a sharper apex. AA. Ovipositor longer, longer than first metasomal tergite; Neotropical	*** Lispixys ***
–	B. Apical flagellomere thicker, with a blunt apex. BB. Ovipositor shorter, barely exerted; widespread	**3**
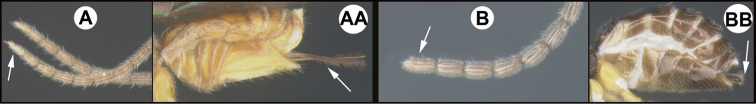		
3(2)	A. Hind wing 1A incomplete, clearly separated from cu-a. AA. Apical flagellomeres of female with a pair of ventral placodes widely separated; widespread	*** Paroligoneurus ***
–	B. Hind wing 1A complete, meeting or nearly meeting cu-a. BB. Apical flagellomeres of female with evenly spaced placodes; widespread	*** Oligoneurus ***
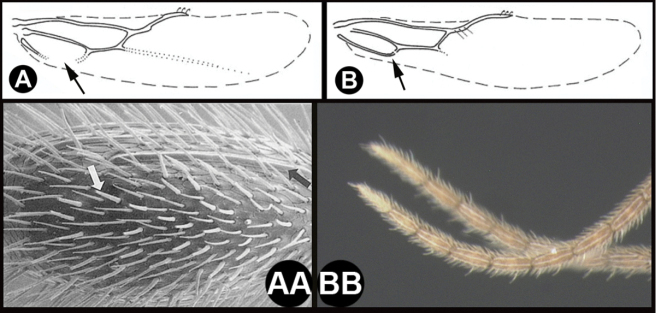		
4(1)	A. Apical abscissa of forewing RS curved towards parastigma; crossvein r emanating near midlength of parastigma; crossvein 2cu-a absent or barely indicated leaving the subdiscal cell open; Canada, first record for the New World, rare	*** Pseudichneutes ***
–	B. Apical abscissa of forewing RS straight or curved towards apex of wing; crossvein r emanating basal to midlength of parastigma; crossvein 2cu-a long and distinct, closing the first subdiscal cell; Nearctic, common	*** Ichneutes ***
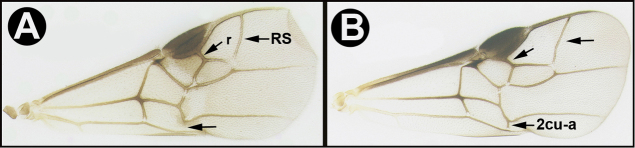		

#### *Oligoneurus* Szépligeti, 1902

Members of the genus are recorded from the Holarctic, Oriental, and Neotropical realms. There are 13 described species of which two are Neotropical (Brazil, Peru). The two published host records are on Cosmopterigidae and Gracillariidae (both Lepidoptera). Here we report a new host family, Crambidae.

##### Oligoneurus jorgejimenezi

Taxon classificationAnimaliaHymenopteraBraconidae

Sharkey sp. nov.

http://zoobank.org/1B46F60E-BEBC-4037-A552-6BF927B2636F

Figure 275

###### Diagnostics.

BOLD:ADB9923. Consensus barcode. ATATTATATTTTATTTTCGGTATATGATCAGGTATATTTGGTTTATCTTTAAGATTATTAATTCGATTAGAATTAGGAAATTTAGGAAATTTTATTGGAAATGATCAAATTTATAATAGAATTGTTACTTCTCATGCATTTATTATAATTTTTTTTATAGTTATACCAATTATAATTGGAGGATTTGGAAATTGATTAATTCCTTTAATATTAGGGGGACCAGATATATGTTTTCCTCGTTTAAATAATATAAGATTTTGATTATTAATTCCTTCAATAATATTTATAATTATTAGAAGATTTGTTGGAAGTGGATGTGGCACAGGATGAACTGTATATCCTCCATTATCTTCATTGATTGGTCATTCTAGTTTATCGGTAGATTTTGGAATTTTTTCTTTACATTTAGYAGGAGTTTCTTCTATTTTAGGATCAATAAATTTTATTTCAACAATTTTAAATATACGATCTTTAAAATTTACTATAGAAAAAATTTCTTTATTTTGTTGAGCTGTATTAATTACAACAATTTTATTATTATTATCATTACCTGTATTAGCTGGTGCAATTACTATATTATTAACAGAT.

###### Holotype ♂.

Guanacaste, Sector Pailas Dos, PL12-3, 10.7631, -85.3344, 820 meters, Malaise trap, 25/ix/2014. Depository: CNC.

***Host data*.** None.

***Holotype voucher code*.**BIOUG30059-F11.

###### Paratype.

BIOUG30154-F02. Depository: CNC.

###### Etymology.

*Oligoneurusjorgejimenezi* is named in honor of Jorge Jimenez attending the international NSF-funded planning meeting for the All Taxa Biodiversity Inventory (ATBI) of Terrestrial Systems, and contributing his wisdom to the planning that was the founding of Costa Rica’s national BioAlfa today.

**Figure 275.**
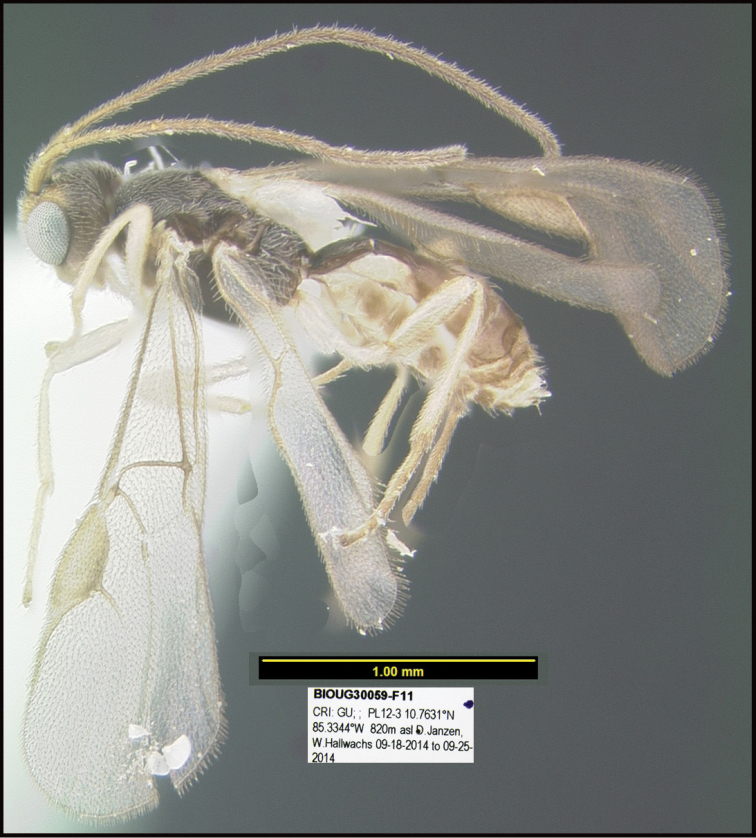
*Oligoneurusjorgejimenezi*, holotype.

##### Oligoneurus kriskrishtalkai

Taxon classificationAnimaliaHymenopteraBraconidae

Sharkey sp. nov.

http://zoobank.org/16FD6B75-414C-4CD8-AF3A-D4AB7A45ACFF

Figure 276

###### Diagnostics.

BOLD:ABA7267. Consensus barcode. TATTTTATATTTTATTTTTGGTTTATGATCAGGAATAATGGGATTATCTTTAAGAATTTTAATCCGTATAGAATTAAGTGCATTAGGACAATTAATTGGTAATGATCAAATTTATAATAGAATTGTAACTTCACATGCTTTTGTGATAATTTTTTTTATAGTAATACCTGTAATAATTGGGGGATTTGGAAATTGGTTGATTCCTTTAATATTAGGAAGACCTGATATATGTTTCCCTCGATTAAATAATATAAGGTTTTGACTATTAGTTCCTTCTGTCCTCTTTATAATCATTAGAAGATTCGTAAGAAATGGATGTGGTACAGGATGAACAGTTTATCCACCTTTATCATTATTAATTGGACATTCAGGCATCTCTGTAGATTTTAGAATTTTTTCTTTACATTTAGCTGGTGTTTCTTCTATTTTAGGATCAATAAATTTTATTTCAACAATTTTGAATATACGATCTATAAAATTTAAAATAGAAAATATTTCATTATTATGCTGATCTGTAATAATTACTACAATTTTACTTTTATTATCATTGCCTGTTTTAGCGGGAGCAATTACGATATTATTAACAGATCGAAATTTAAATACAACATTTTTTGATCCTTCTGGAGGCGGAGACCCAGTTTTATATCAACATTTATTT.

###### Holotype ♀.

Guanacaste, Sector San Cristobal, Tajo Angeles, 10.86472, -85.41531, 540 meters, caterpillar collection date: 8/ii/2011, wasp eclosion date: 20/iii/2011. Depository: CNC.

***Host data*.***Desmia* benealisDHJ02 (Crambidae) feeding on *Drymoniamacrophylla* (Gesneriaceae).

***Caterpillar and holotype voucher codes*.** 11-SRNP-521, DHJPAR0043136.

###### Paratypes.

**None.**

###### Etymology.

*Oligoneuruskriskrishtalkai* is named in honor of Kris Krishtalka attending the international NSF-funded planning meeting for the All Taxa Biodiversity Inventory (ATBI) of Terrestrial Systems, and contributing his wisdom to the planning that was the founding of Costa Rica’s national BioAlfa today.

**Figure 276.**
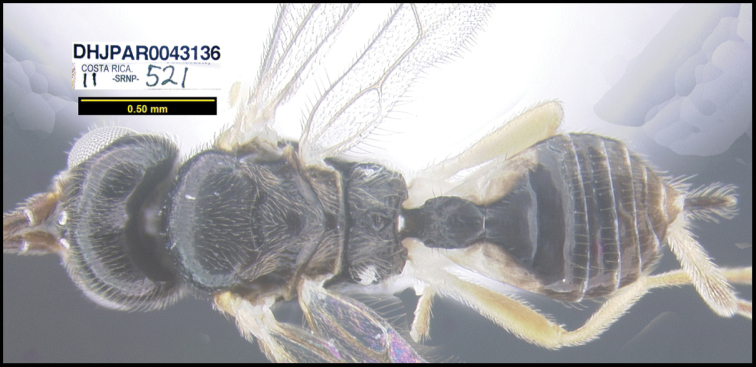
*Oligoneuruskriskrishtalkai*, holotype.

#### *Paroligoneurus* Muesebeck, 1931

Members of the genus are found worldwide. There are 18 described species of which six are described from Mexico and one from Ecuador. The sole published host record is on Nepticulidae (Lepidoptera) (Belokobylskij 1986). No new host records are recorded here.

##### Paroligoneurus elainehoaglandae

Taxon classificationAnimaliaHymenopteraBraconidae

Sharkey sp. nov.

http://zoobank.org/26B935B4-7A7B-419E-9532-F2BCF404B5A5

Figure 277

###### Diagnostics.

BOLD:ADA8821. Consensus barcode. ATTTTATATTTTTTATTTGGTATATGAGCTGGAATATTTGGTTTGTCAATAAGTTTAATTATTCGTTTAGAGTTGGGTATGTTGGGGTCTTTTATTGGTAGAGATCAAATTTATAATAGAATAGTTACTTCTCATGCTTTTGTAATAATTTTTTTTATAGTTATACCAATTATAATTGGCGGATTTGGAAATTGATTAATTCCTTTAATATTATCAGCTCCAGATATAGCCTTTCCTCGTATGAATAATATGAGATTTTGATTATTAATTCCTTCTTTAATTATATTAATTTTAAGGGGGTTAATAAATAGGGGAGCAGGTACAGGGTGAACAGTTTATCCTCCTTTATCTTTATTAATTGGTCATGGGGGCATATCAGTTGATATATGTATTTTTTCTTTACATTTAGCAGGTGCTTCTTCAATTATAGGTGCAATTAATTTTATTACTACTATTTTAAATATACGTTCTATTTATTTAACTATAGATAAAATTTCTTTATTAAGATGATCAGTTTTAATTACTGCAATTTTGTTATTATTATCATTACCTGTTTTAGCTGGGGCTATTACTATACTTTTAACA---------------------------------------------.

###### Holotype ♀.

Guanacaste, Sector Pailas Dos, PL12-1, 10.7642, -85.335, 828 meters, Malaise trap, 2/x/2013. Depository: CNC.

***Host data* . None.**

***Holotype voucher code*.**BIOUG28589-E03.

###### Paratypes.

None.

###### Etymology.

*Paroligoneuruselainehoaglandae* is named in honor of Elaine Hoagland attending the international NSF-funded planning meeting for the All Taxa Biodiversity Inventory (ATBI) of Terrestrial Systems, and contributing her wisdom to the planning that was the founding of Costa Rica’s national BioAlfa today.

**Figure 277.**
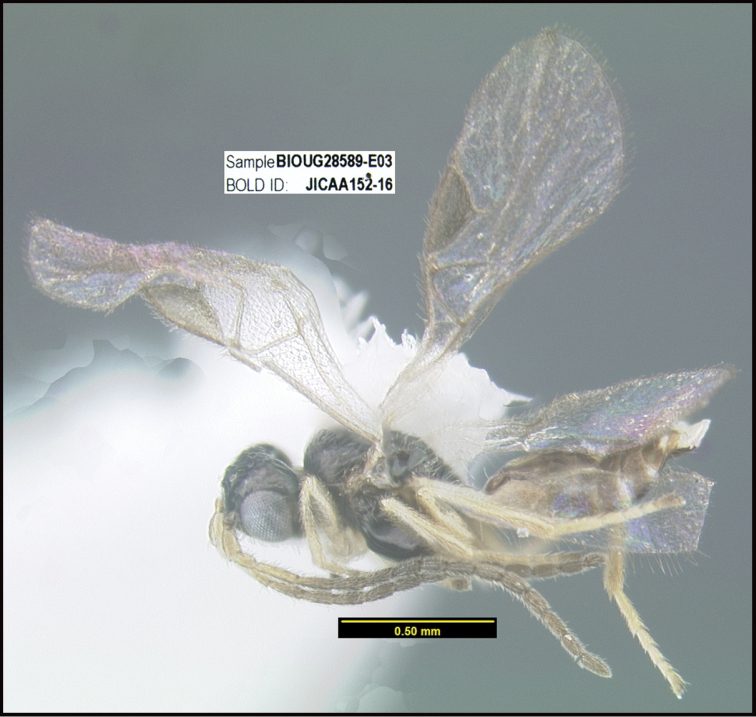
*Paroligoneuruselainehoaglandae*, holotype.

##### Paroligoneurus julianhumphriesi

Taxon classificationAnimaliaHymenopteraBraconidae

Sharkey sp. nov.

http://zoobank.org/192E4691-5422-4FA7-BD33-DF1579FF0C3D

Figure 278

###### Diagnostics.

BOLD:ADF4538. Consensus barcode. AATTTTATATTTTTTATTTGGTATATGAGCTGGAATATTTGGTTTGTCAATAAGTTTAATTATTCGTTTAGAGTTGGGTATGTTGGGGTCTTTTATTGGTAGAGATCAGATTTATAATAGAATAGTTACTTCTCATGCTTTTGTAATAATTTTTTTTATAGTTATACCAATTATAATTGGTGGATTTGGAAATTGATTAATTCCTTTAATATTATCAGCTCCAGATATAGCTTTTCCTCGTATAAATAATATGAGATTTTGATTATTAATTCCTTCTTTAATTATATTAATTTTAAGGGGGCTAATGAATAGGGGAGCAGGYACAGGGTGAACAGTTTATCCTCCTTTATCTTTATTAATTGGTCATGGGGGTATATCAGTTGATATATGTATTTTTTCTTTACATTTAGCAGGTGCTTCTTCGATTATAGGTGCAATTAATTTTATTACTACTATTTTAAATATACGTTCTATTTATTTAACTATAGATAAAATTTCTTTATTAAGATGATCAGTTTTAATTACTGCAATTTTGTTATTATTATCATTACCTGTTTTAGCTGGGGCTATTACTATACTTTTAACAGATCGTAATTTGAATACT.

###### Holotype ♀.

Guanacaste, Sector Cacao, Derrumbe, 10.9292, -85.4643, 1220 meters, Malaise trap, 30/x/2014. Depository: CNC.

***Host data*.** None.

***Holotype voucher code*.**BIOUG31504-H06.

###### Paratypes.

BIOUG31533-C04, BIOUG31533-E09. Depository: CNC.

###### Etymology.

*Paroligoneurusjulianhumphriesi* is named in honor of Julian Humphries attending the international NSF-funded planning meeting for the All Taxa Biodiversity Inventory (ATBI) of Terrestrial Systems, and contributing his wisdom to the planning that was the founding of Costa Rica’s national BioAlfa today.

**Figure 278.**
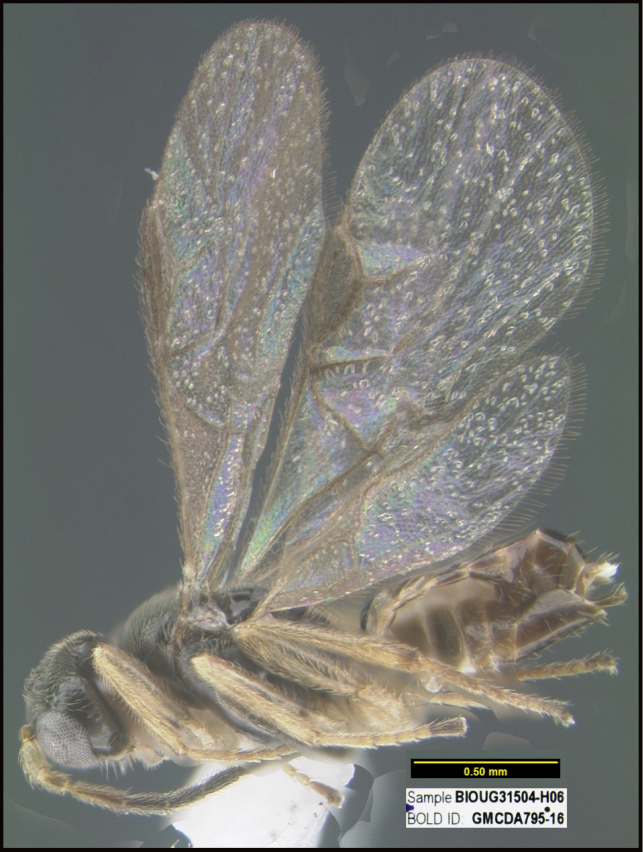
*Paroligoneurusjulianhumphriesi*, holotype.

##### Paroligoneurus mikeiviei

Taxon classificationAnimaliaHymenopteraBraconidae

Sharkey sp. nov.

http://zoobank.org/0F369AE8-CFA6-42A5-AB87-148F2A6C1EAB

Figure 279

###### Diagnostics.

BOLD:ADB7695. Consensus barcode. GGAATTTTATATTTTTTATTTGGTATATGGGCTGGAATATTTGGTTTGTCAATAAGTTTAATTATTCGTTTAGAGTTGGGTATGTTGGGATCTTTTATTGGTAGAGATCAAATTTATAATAGAATAGTTACTTCTCATGCTTTTGTAATAATTTTTTTTATAGTTATACCAATTATAATTGGTGGATTTGGAAATTGATTAATTCCTTTAATATTATCAGCTCCAGATATAGCATTTCCTCGTATAAATAATATAAGATTTTGATTATTAATTCCTTCTTTAATTATATTAATTTTAAGGGGATTAATAAATAGGGGTGCAGGTACGGGGTGAACAGTTTATCCYCCCTTATCTTTATTAATTGGTCATGGGGGTATATCAGTTGATATATGTATTTTTTCTTTACATTTAGCAGGKGCKTCTTCAATTATAGGTGCTATTAATTTTATTACTACTATTTTTAATATACGCTCTATTTATTTAACTATAGATAAAATTTCTTTATTAAGATGATCAGTTTTAATTACTGCAATTTTATTATTATTATCATTACCTGTTTTAGCTGGGGCTATTACTATACTTTTAACAGATCGTAATTTGAATACTAGTTTTTTTGATCCTGC-GGAGGGG--.

###### Holotype ♀.

Guanacaste, Sector Cacao, Derrumbe, 10.9292, -85.4643, 1220 meters, Malaise trap, 5/ii/2015. Depository: CNC.

***Host data*.** None.

***Holotype voucher code*.**BIOUG33120-D06.

###### Paratypes.

BIOUG31723-D02, BIOUG32722-B01, BIOUG32747-H05, BIOUG32722-C04, BIOUG31532-E01, BIOUG32722-B01. Depository: CNC.

###### Etymology.

*Paroligoneurusmikeiviei* is named in honor of Mike Ivie attending the international NSF-funded planning meeting for the All Taxa Biodiversity Inventory (ATBI) of Terrestrial Systems, and contributing his wisdom to the planning that was the founding of Costa Rica’s national BioAlfa today.

**Figure 279.**
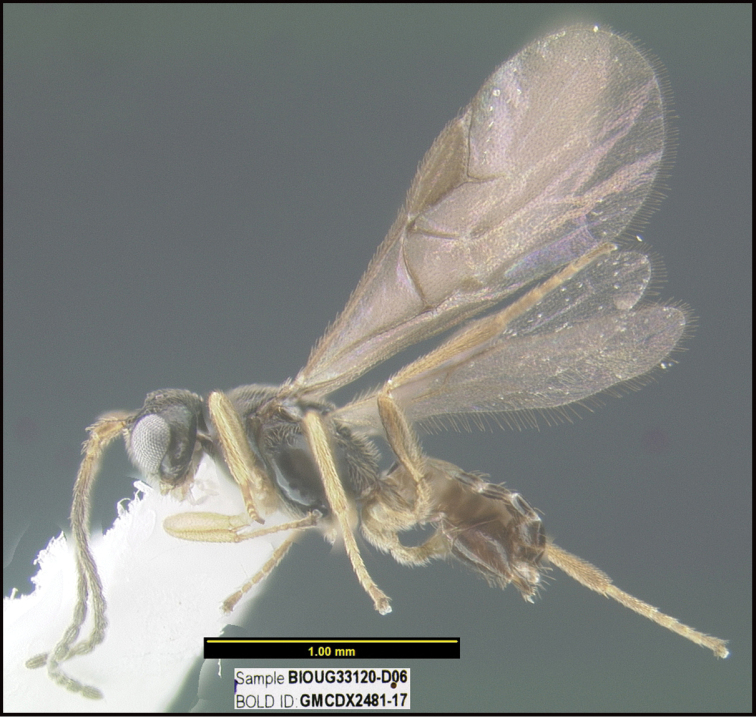
*Paroligoneurusmikeiviei*, holotype.

## Chapter 8: Macrocentrinae

The New World genera of Macrocentrinae may be distinguished using the key that follows. Interestingly, no species have been recorded from Costa Rica (Yu et al. 2016). Members of all genera are koinobiont endoparasitoids of caterpillars from a wide range of families; most are solitary, but several gregarious parasitoids are known. Notodontidae is recorded here as a host for the first time. For the Macrocentrinae NJ tree, see Suppl. material 7.

### Key to the New World genera of Macrocentrinae

**Table:** 

1	A. Petiole with laterope shallow or absent; petiole flat or convex basal-medially	**2**
–	B. Petiole with laterope deep; petiole nearly always slightly concave basal-medially	**3**
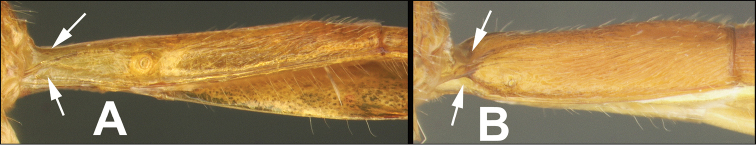		
2 (1)	A. Ovipositor long, ovipositor sheath at least as long as metasoma. AA. Length of inner (longest) spur of hind tibia 0.4–0.6 × length of hind basitarsus	*** Hymenochaonia ***
–	B. Ovipositor short, ca. equal to apical height of metasoma. BB. Length of inner (longest) spur of hind tibia usually (80%) 0.6–0.8 × length of hind basitarsus; if shorter thenapex of the spur lacking setae	*** Dolichozele ***
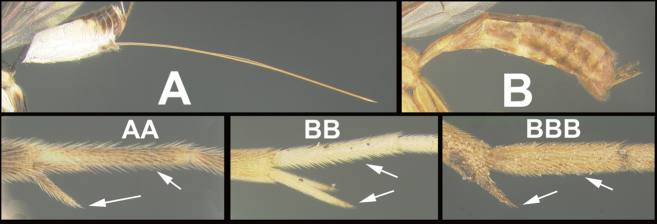		
3(1)	A. Ovipositor long, ovipositor sheath nearly always at least as long as metasoma. AA. Length of inner (longest) spur of hind tibia 0.3–0.5 × length of hind basitarsus	*** Macrocentrus ***
–	B. Ovipositor short, ca. equal to apical height of metasoma. BB. Length of inner (longest) spur of hind tibia 0.5–0.8 × length of hind basitarsus	*** Austrozele ***
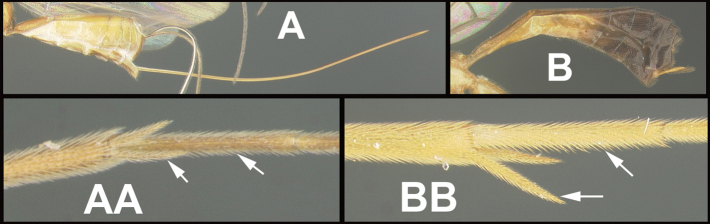		

#### *Austrozele* Roman, 1910

Worldwide in distribution, but most species found in the Palaeotropical region. Recorded hosts are Geometridae and Noctuidae; Notodontidae is recorded as a host for the first time in this work. No species have been recorded from Costa Rica. The types of all five *Austrozele* species previously recorded from the Neotropical region (Colombia, Mexico, Brazil, and Guyana) under *Austrozele* were described in *Paniscozele* Enderlein, 1920, a junior synonym of *Austrozele* Roman, 1911. These were examined by CvA and all belong to *Dolichozele* Viereck. For details and new combinations see the latter genus. As a result, we report here the genus as new for the Neotropical region.

##### Austrozele jorgecampabadali

Taxon classificationAnimaliaHymenopteraBraconidae

Sharkey & van Achterberg sp. nov.

http://zoobank.org/F6AD230B-1669-4C13-9838-A3ED0E1CE6B4

Figure 280

###### Diagnostics.

BOLD:AAE4116. Consensus barcode. AATTTTATATTTTTTATTTGGGATATGATCAGGAATAATTGGATTATCAATAAGAATTATTATTCGAATAGAATTAAGTCAATCTGGATCAATAATTGGAAATGATCAAATTTACAATAGATTTGTTACTACCCATGCTTTCATTATAATTTTTTTTATAGTAATACCTACTATATTAGGGGGGTTTGGAAATTGATTAATYCCATTAATATTAGGAAGAGTTGATATAGCTTTCCCACGAATAAATAATATAAGATTTTGATTATTAATTCCTTCTTTATYATTATTATTATTAAGAGGATTTATAAATATTGGAGTTGGTACTGGATGAACTGTTTAYCCTCCATTATCTTTAAATATTAGACATATAGGAATATCTGTTGATATAGCTATTTTTTCTTTACATTTAGCAGGAATTTCTTCTATTATAGGATCAATTAATTTTATTGTTACTATTATAAATATACGTAATTATGGAGTAATTATAGAAAAAATTAGTTTATTATGTTGATCAATTTTAATTACAGTAATTTTATTATTATTATCATTGCCTGTTTTAGCAGGAGCAATTACTATATTATTAACTGATCGAAATTTAAATACTACTTTTTTTGATCCTGCAGGAGGGGGAGATCCAATTTTATATCAACATTTATTT. Morphologically very similar to *A.jorgesoberoni* because of posteriorly slightly widened first metasomal tergite, glabrous apical half of subbasal cell of forewing combined with a faint sclerome and dark brown posterior half of metasoma. Differs by having the diameter of the lateral ocellus ca. equal to its distance from the eye (vs. 1.2–1.3 × in *A.jorgesoberoni*), vein cu-a of forewing straight and vertical (bent posteriorly and reclivous) and base of first tergite wider in lateral view (vs. slenderer).

###### Holotype ♀.

Alajuela, Sector San Cristobal, Sendero Palo Alto, 570 meters, 10.88186, -85.38221, caterpillar collection date: 20/viii/2004, wasp eclosion date: 14/ix/2004. Depository: CNC.

***Host data*.***Dunamajanewaldronae* (Notodontidae) feeding on *Chamaedoreadammeriana* (Arecaceae).

***Caterpillar and holotype voucher codes*.** 04-SRNP-4200, DHJPAR0009366.

###### Paratypes.

Host = *Dunamajanewaldronae*: DHJPAR0009367, DHJPAR0009370, DHJPAR0029346, DHJPAR0030311, DHJPAR0030381, DHJPAR0061211. Depository: CNC.

###### Etymology.

*Austrozelejorgecampabadali* is named to honor Sr. Jorge Campabadal as the first Costa Rican administrator for the Organization for Tropical Studies in its founding in Costa Rica.

**Figure 280.**
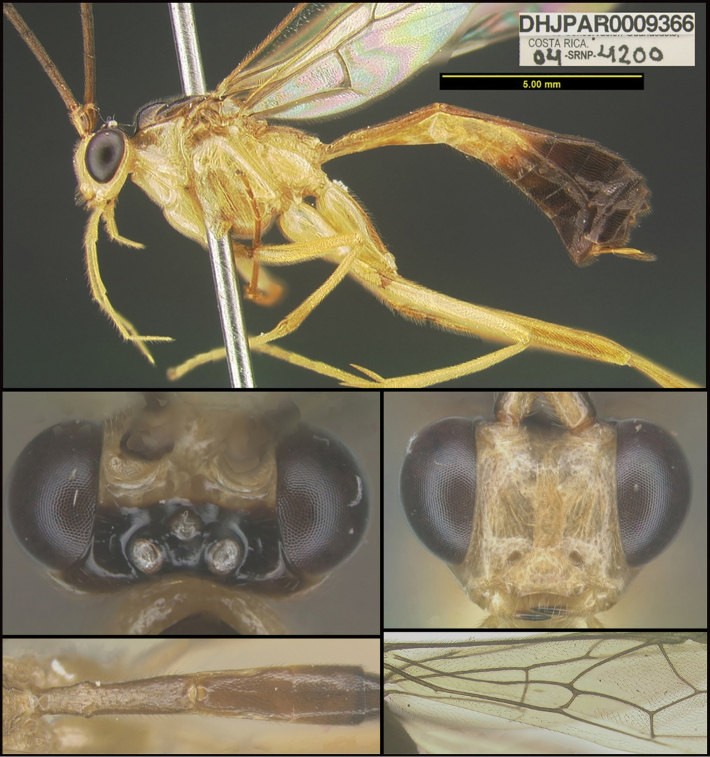
*Austrozelejorgecampabadali*, holotype.

##### Austrozele jorgesoberoni

Taxon classificationAnimaliaHymenopteraBraconidae

Sharkey & van Achterberg sp. nov.

http://zoobank.org/4432706E-3F83-4D2E-B452-DF97163D9D6C

Figure 281

###### Diagnostics.

BOLD:AAF0520. Consensus barcode. AATTTTATATTTTTTATTTGGAATATGAGCGGGAATAATTGGGTTATCAATAAGAATTATTATTCGTATAGAATTAAGTCAACCTGGATCATTAATTGGAAATGATCAAATTTATAATAGATTTGTTACCACCCATGCTTTCATTATAATTTTTTTTATAGTAATGCCTATTATATTGGGGGGATTTGGTAATTGATTAATTCCATTAATATTAGGAAGAGTTGATATAGCTTTYCCACGAATGAATAATATAAGATTTTGATTACTAATTCCTTCTTTATTATTATTATTATTAAGAGGATTTATAAATATTGGAGTTGGTACTGGATGAACTGTTTACCCTCCCTTATCTTTAAATATTAGACATATAGGGATATCAGTTGATATAGCTATTTTTTCTTTACATTTAGCAGGAATTTCTTCTATTATAGGGTCAATTAATTTTATTGTTACAATTTTGAATATACGTAATTACGGARTAATTATAGAAAAAATTAGTTTATTATGTTGATCAATTTTAGTTACAGTAATTTTATTATTATTATCATTACCTGTTTTAGCAGGAGCTATTACTATATTATTAACTGATCGAAATTTAAATACTACTTTTTTTGACCCTGCGGGAGGGGGGGATCCAGTTTTATATCAACATTTATTT. For morphological differences see diagnosis of *A.jorgecampabadali*.

###### Holotype ♀.

Guanacaste, Sector Pitilla, Sendero Naciente, 700 meters, 10.98705, -85.42816, caterpillar collection date: 04/xi/2013, wasp eclosion date: 18/xii/2013. Depository: CNC.

***Host data*.***Acrotomodes* bolaDHJ01 (Geometridae) feeding on *Pimentaguatemalensis* (Myrtaceae).

***Caterpillar and holotype voucher codes*.** 13-SRNP-31637, DHJPAR0054444.

###### Paratypes.

Host = *Pyrinia* Janzen02: DHJPAR0009365, DHJPAR0029343, DHJPAR0029348, DHJPAR0042749, DHJPAR0054443, DHJPAR0054445, DHJPAR0054446, DHJPAR0054447, DHJPAR0054453, DHJPAR0063839. Depository: CNC.

###### Etymology.

*Austrozelejorgesoberoni* is named to honor Dr. Jorge Soberon of the University of Kansas for his seminal role in the founding and development of Mexico’s CONABIO.

**Figure 281.**
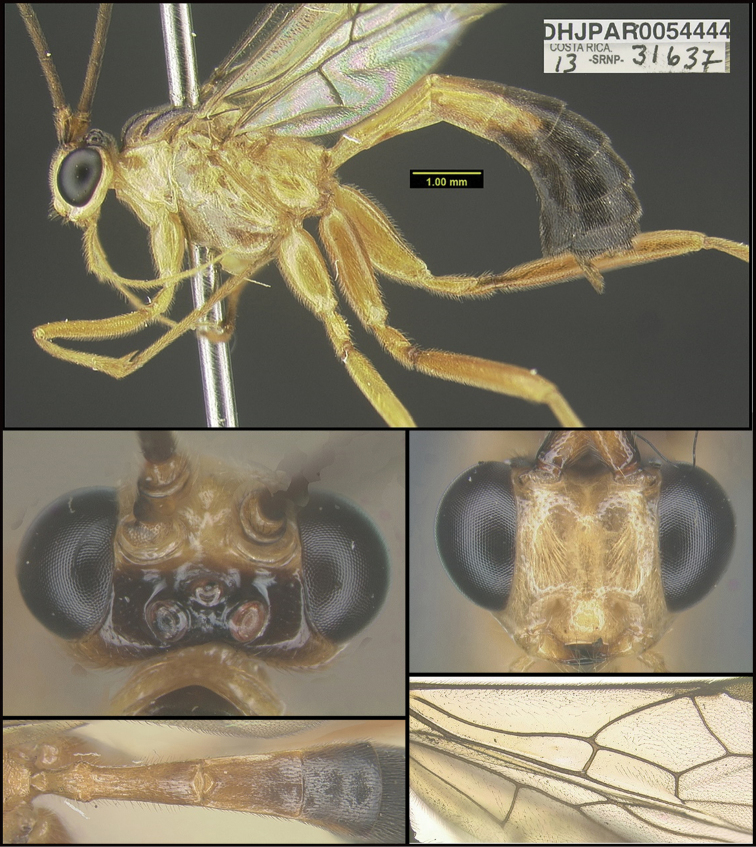
*Austrozelejorgesoberoni*, holotype.

#### *Dolichozele* Viereck, 1911

*Dolichozele* is exclusively New World in distribution. Of the twelve recorded species only *D.gravitarsis* has host data. It is reported as a parasitoid of *Lophocampaargentata* and *Lophocampaingens* (Erebidae, as Arctiidae; Marsh 1979). Here we report a new host family, Noctuidae. There are ten named Neotropical species; the species named by Enderlein (1920) were examined by CvA and his results are as follows:

*Austrozelecitreitarsis* (Enderlein, 1920) *Paniscozele*. Valid species in *Dolichozele* Viereck, 1911, comb nov.

*Austrozelecarinifrons* (Enderlein, 1920) *Paniscozele*. New junior synonym of *Dolichozelefuscivertex* (Enderlein, 1920).

*Austrozelefuscivertex* (Enderlein, 1920) *Paniscozele*. Valid species in *Dolichozele* Viereck, 1911, comb nov.

*Austrozelegriseipes* (Enderlein, 1920) *Paniscozele*. New junior synonym of *Dolichozelekoebelei* Viereck, 1911.

*Austrozelenigricauda* (Enderlein,1920) *Paniscozele*. New junior synonym of *Dolichozelequaestor* (Fabricius, 1804). (originally described as *Ophionquaestor* Fabricius, 1804).

##### Dolichozele josefernandeztrianai

Taxon classificationAnimaliaHymenopteraBraconidae

Sharkey & van Achterberg sp. nov.

http://zoobank.org/13077531-0A1C-4F2B-A11C-C68661A54A98

Figure 282

###### Diagnostics.

BOLD:ACK6060. Consensus barcode. ATTTTATATTTTTTATTCGGAATATGAGCAGGTATAATTGGTTTATCAATAAGAATCATTATTCGAATCGAATTAAGTCAATCAGGATCATTTATTAGTAATGATCAAATTTATAATAGATTTGTTACTGCTCATGCTTTTATTATAATTTTTTTTATAGTTATACCTATTATAATTGGGGGATTTGGAAATTGATTAATTCCTTTAATATTAGGAAGAGTTGATATAGCTTTCCCTCGAATAAATAATATAAGATTTTGATTATTAATTCCTTCATTATTATTATTATTAATAAGAGGATTTATAAATATTGGAGTGGGAACTGGATGAACAGTTTATCCTCCTTTATCATTAAATATTAGTCATAGAGGAATATCAGTTGATATAGCTATTTTTTCTTTACATTTAGCTGGTATATCATCAATTATAGGATCTATTAATTTTATTGTAACTATTTTAAATATACGAAATTATGGAGTTATTTTAGAAAAAATTAGATTATTAAGATGATCAATTTTAATTACTGTAATTTTATTATTATTATCTTTACCTGTTTTAGCAGGAGCAATTACAATATTATTAACAGATCGAAATTTAAATACTACTTTTTTTGATCCTTCTGGGGGGGGAGATCCTGTATTATACCAACATTTATTT. Differs from other species by its brown hind tarsus, vein cu-a of forewing bent posteriorly and subbasal cell of forewing without a faint sclerome.

###### Holotype ♀.

Guanacaste, Sector Pitilla, Colocho, 375 meters, 11.02367, -85.41884, caterpillar collection date: 25/vii/2005, wasp eclosion date: 02/x/2005. Depository: CNC.

***Host data*.***Goniohelia* Poole02 (Erebidae) feeding on *Senegaliamultipinnata* (Fabaceae).

***Caterpillar and holotype voucher codes*.** 05-SRNP-33496, DHJPAR0028979.

###### Paratypes.

None.

###### Etymology.

*Dolichozelejosefernandeztrianai* is named to honor Dr. José Fernandez-Triana for his many years of mentoring and taxonomic applications to the bioinventory of the Microgastrinae, Braconidae, of ACG.

**Figure 282.**
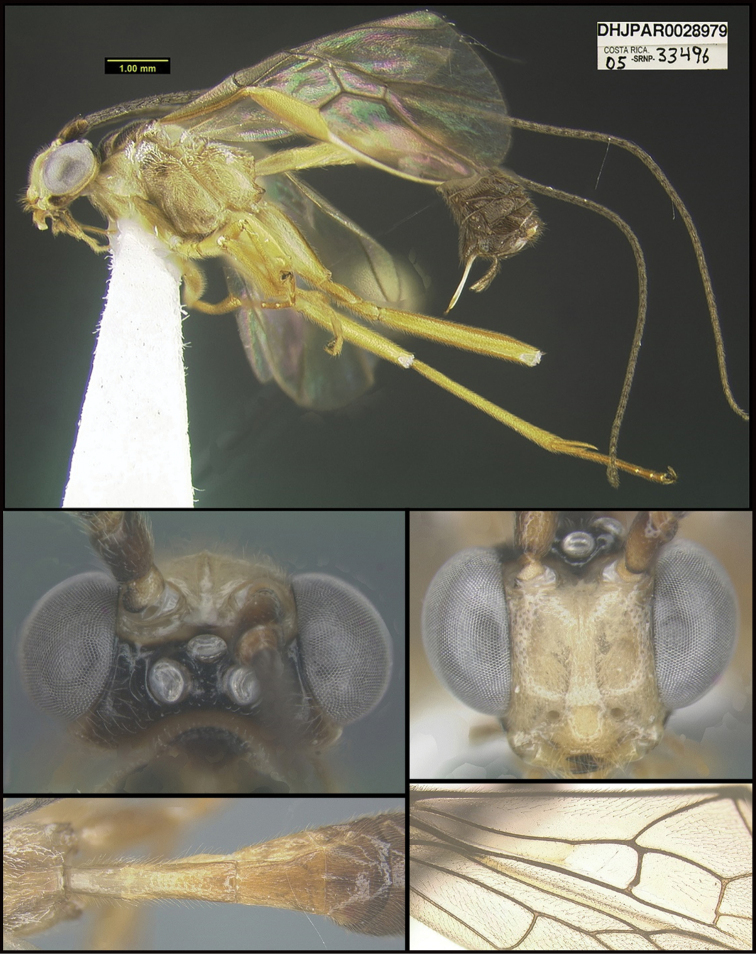
*Dolichozelejosefernandeztrianai*, holotype.

##### Dolichozele josephinerodriguezae

Taxon classificationAnimaliaHymenopteraBraconidae

Sharkey & van Achterberg sp. nov.

http://zoobank.org/F3161064-737B-4361-ACD2-F4BF0DCC4F89

Figure 283

###### Diagnostics.

BOLD:AAF3799. Consensus barcode. AATTTTATATTTTATATTTGGAATATGATCAGGAATAATTGGTTTATCGATAAGAATTATTATTCGAATAGAATTAAGACAATCAGGATCATTTATTTGTAATGATCAAATTTATAATAGATTTGTAACTGCACATGCTTTTATTATAATTTTTTTTATAGTTATACCAATTATGATTGGTGGATTTGGAAATTGATTAATTCCTTTAATATTAGGTAGAGTTGATATAGCTTTTCCTCGAATAAATAATATAAGGTTTTGATTATTAATTCCTTCATTATTATTATTATTAATGAGTGGATTTATAAATATTGGAGTAGGAACAGGTTGAACTGTTTATCCTCCTTTATCTTTAAATATTAGTCAAAGAGGAATATCAGTTGATATGGCTATTTTTTCTTTACATTTAGCTGGAGTATCATCAATTATAGGGTCAATTAATTTTATTGTAACTATTTTAAATATACGAAATTATGGAATTATTATAGAAAAAATTAGATTATTATGTTGATCAATTTTAATTACTGTAATTTTATTATTAATATCATTACCTGTATTAGCAGGAGCAATTACTATATTATTAACAGATCGAAATTTAAATACTACTTTTTTTGATCCTGCAGGAGGAGGAGATCCTGTATTATATCAACATTTATTT. The new species shares with *D.fuscivertex* (Enderlein) the dark brown band on the vertex, but differs by the comparatively robust first tergite (distinctly widened posteriorly and approx. 3 × longer than its apical width (parallel-sided in *D.fuscivertex* and approx. 5 × longer than its apical width), inner hind claw of female different from outer hind claw (similar in *D.fuscivertex*) and subbasal cell of forewing with faint nearly round sclerome (absent in *D.fuscivertex*).

###### Holotype ♀.

Guanacaste, Sector Pailas, Estación Pailas, 785 meters, 10.76969, -85.34080, caterpillar collection date: 01/viii/1998, wasp eclosion date: 3/ix/1998. Depository: CNC.

***Host data*.***Bagisaraalbicosta* (Noctuidae) feeding on *Crotonmorifolius* (Euphorbiaceae).

***Caterpillar and holotype voucher codes*.** 98-SRNP-11256, DHJPAR0029357.

###### Paratypes.

Host = *Bagisaraalbicosta*: DHJPAR0029416, DHJPAR0029417, DHJPAR0029415, DHJPAR0029356, DHJPAR0021564, DHJPAR0021114. Depository: CNC.

###### Note.

males and females differ strongly in color.

###### Etymology.

*Dolichozelejosephinerodriguezae* is named to honor Dr. Josephine Rodriguez of the University of Virginia’s College at Wise, for her intense collaboration and taxonomy of ACGMicrogastrinae as part of the ACG ongoing Biodiversity Inventory.

**Figure 283.**
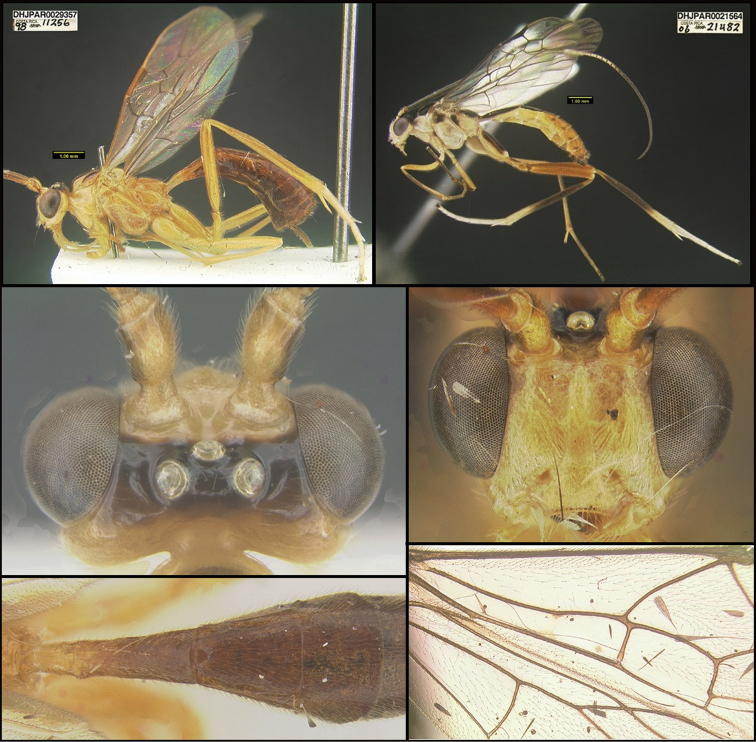
*Dolichozelejosephinerodriguezae*: holotype female except male paratype (upper right); image added to show sexual dimorphism.

##### Dolichozele gravitarsis

Taxon classificationAnimaliaHymenopteraBraconidae

(Muesebeck, 1938)

Figure 284

###### Diagnostics.

BOLD:AAI9864. Consensus barcode. ATTTTATATTTTTTATTTGGAATATGATCAGGAATAATTGGTTTATCAATAAGAATTATTATTCGAATAGAATTRAGACAATCAGGAATATTTATTGGTAATGATCAAATTTATAATAGATTTGTAACAGCTCATGCATTTATTATAATTTTTTTTATAGTCATACCAATTATAATTGGTGGATTTGGAAATTGAYTAATTCCTTTAATATTAGGAAGWRTTGATATAGCTTTTCCACGAATAAATAATATAAGATTTTGATTATTAATTCCATCATTATTTTTATTATTAATAAGAGGATTYATAAATATTGGTRTTGGAACTGGTTGAACTGTATATCCTCCTTTATCATTAAATGTAAGTCATAGAGGAATATCTGTTGATATAGCTATTTTTTCTCTTCATTTAGCWGGAATTTCTTCAATYATAGGATCAATTAATTTTATTGTTACTATTTTAAATATACGTAATTATGGAATCATTATAGAAAAAATTAGATTATTATGTTGATCAATTTTAATTACTGTTATTTTATTATTAATRTCTTTACCAGTTTTRGCTGGAGCAATTACTATATTATTAACAGATCGAAATTTAAATACTACATTTTTTGATCCTGCTGGAGGAGGTGATCCTGTATTATAYCAACATTTATTT. Both sexes have a strongly bent forewing vein M+Cu1 and comparatively short hind tibial spurs; the females have a robust and yellowish hind tarsus, which is ivory or whitish and slenderer in males.

###### Figured specimen.

♂, Alajuela, Sector Rincon Rain Forest, Camino Albergue Oscar, 10.877, -85.324, 560 meters, caterpillar collection date 3/iii/2008, wasp eclosion date 26/v/2008.

***Host data*.***Turuptianaobliqua* (Erebidae) feeding on *Nectandrahihua* (Lauraceae).

***Caterpillar and figured male voucher codes*.**DHJPAR0027736.

###### Additional specimens.

Host = *Hypocrisias* Espinoza01 DHJPAR0027736, DHJPAR0029351, DHJPAR0029352, DHJPAR0029353, DHJPAR0029355. Depository: CNC.

###### Notes.

The species is widespread from British Columbia, Canada, through the western USA, Mexico, and Meso America. One specimen from British Columbia (BIOUG22381-E09) is in the same BIN.

**Figure 284.**
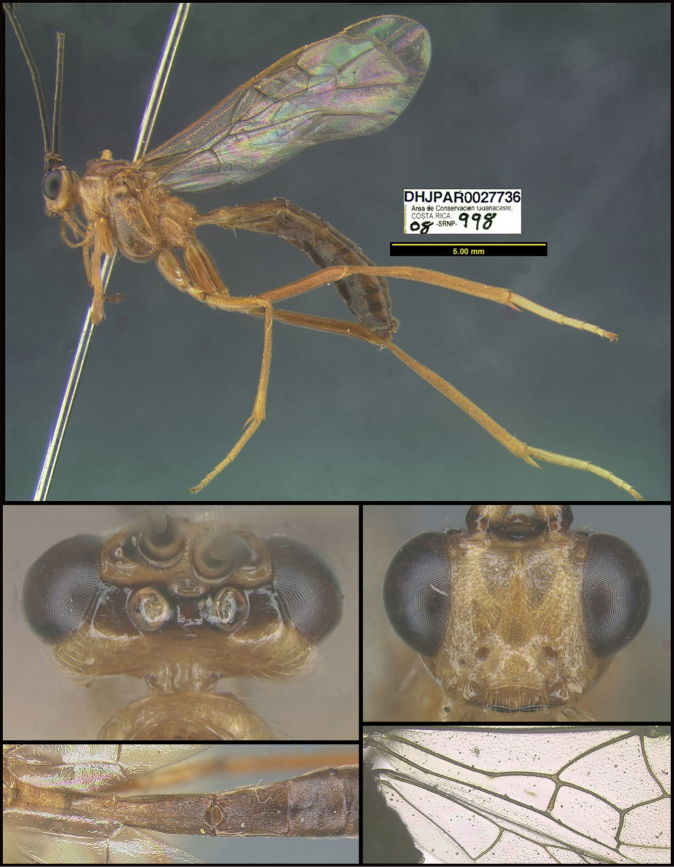
*Dolichozelegravitarsis*.

#### *Hymenochaonia* Dalla Torre, 1901

*Hymenochaonia* is exclusively New World in distribution although *H.delicata* (Cresson, 1872) has been introduced (apparently unsuccessfully) to Italy for biocontrol purposes. *Hymenochaoniadelicata* has been recorded as a parasitoid of many caterpillar species including members of Depressariidae, Gelichiidae, Noctuidae, Pyralidae, and Tortricidae. It cannot be ruled out that more than one species of parasitoid were treated in these published reports (see Yu et al. 2016) that rely on morphological identification without examination of the type. Other species of *Hymenochaonia* are reported as parasitoids of Pyralidae and Tortricidae. The types of four previously described Neotropical species are from Cuba, Guyana, and Brazil have been examined by CvA. The 13 species proposed here more than doubles the number of known species.

##### Hymenochaonia kalevikulli

Taxon classificationAnimaliaHymenopteraBraconidae

Sharkey & van Achterberg sp. nov.

http://zoobank.org/30B0051E-4CCD-4922-8056-EA3ACB85DF5F

Figure 285

###### Diagnostics.

BOLD:AAP9411. Consensus barcode. AATATTATATTTTATTTTTGGAATATGGGCAGGTATAATTGGTTTATCAATAAGATTAATTATTCGWATAGAATTAGGTCAATCAGGRAGTTTAATTGCTAATGATCAAATTTAYAATAGRGTAGTAACAGCTCATGCATTTATTATAATTTTTTTTATAGTTATACCAATTATAATTGGRGGATTTGGTAATTGATTAATTCCTTTAATATTAGGGRGGGTTGATATAGCTTTCCCACGTATAAATAATATAAGATTTTGGTTATTAATTCCTTCAATTATTTTATTATTAATAAGAGGATTTTTGAATATTGGGGTTGGTACTGGTTGAACTATTTATCCTCCTTTATCTTTAAATATTAGACATTTAGGAGTTTCTTTAGATATAACTATTTTTTCTTTACATTTAGCTGGAATTTCTTCTATTATAGGGGCTATAAATTTTATTGTAACAATTTTAAATATACGTAATTCTGGAGTATTAATAGATAAAATTAGATTATTATGTTGATCAATTTTAATTACTGCTATTTTATTATTATTGTCTTTACCAGTGTTAGCAGGGGCAATTACAATATTATTAACTGAYCGAAATTTAAATACTTCTTTTTTTGATCCTTCAGGAGGGGGGGACCCAATTTTATAYCAGCATTTATTT. This species and *H.keithwillmotti* sp. n. share a long and widened vein 1Cua of forewing, antenna (except blackish base) pale yellowish or whitish and distinctly curved vein cu-a of forewing. *Hymenochaoniakalevikulli* differs by having vein 1Cua of forewing longer than vein cu-a, tarsal claws simple, hind tibia white basally and remainder black, subbasal cell of forewing largely glabrous and apical half of metasoma black.

###### Holotype ♀.

Alajuela, Sector Rincon Rain Forest, Sendero Albergue Crater, 10.84886, -85.3281, 980 meters, caterpillar collection date: 06/xii/2010, wasp eclosion date: 7/x/2010. Depository: CNC.

***Host data*.***Antaeotricha* caprimulgaEPR01 (Depressariidae) feeding on *Gonocalyxpterocarpus* (Fabaceae).

***Caterpillar and holotype voucher codes*.** 10-SRNP-3034, DHJPAR0040587.

###### Paratypes.

Hosts = *Antaeotricha* caprimulgaEPR01 and *Antaeotricha* caprimulgaEPR02. DHJPAR0040484, DHJPAR0040567, DHJPAR0040571 DHJPAR0040572, DHJPAR0040574, DHJPAR0040578, DHJPAR0040579, DHJPAR0040580, DHJPAR0040584, DHJPAR0040585, DHJPAR0040591, DHJPAR0040595. Depository: CNC.

###### Etymology.

*Hymenochaoniakalevikulli* is named to honor Dr. Kalevi Kull for his efforts to translate and publicize ACG biodiversity biodevelopment in the Estonian language.

**Figure 285.**
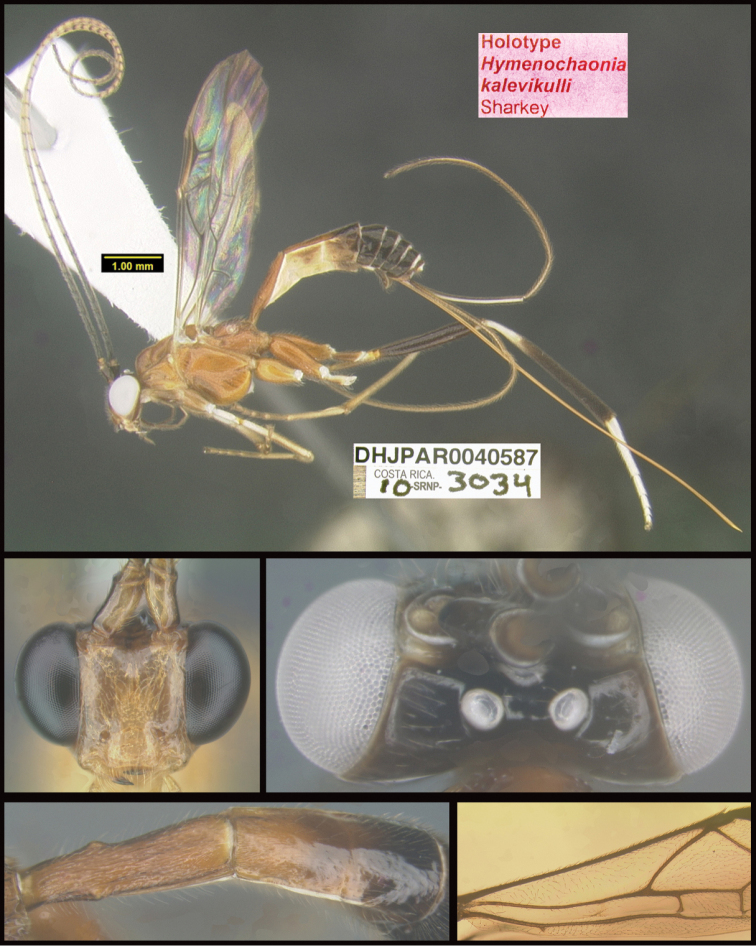
*Hymenochaoniakalevikulli*, holotype.

##### Hymenochaonia kateperezae

Taxon classificationAnimaliaHymenopteraBraconidae

Sharkey & van Achterberg sp. nov.

http://zoobank.org/AD8DF9AB-82C3-43F2-9CC1-BE14B63D688A

Figure 286

###### Diagnostics.

BOLD:AAA7061. Consensus barcode. GGTTTTATATTTTATATTTGGGTTATGGGCGGGTATAATTGGKATATCAATAAGATTAGTTATTCGTTTAGAATTAGGTCAATCAGGGGTATTAATTGGTAATGAYCAAATTTATAATAGATTTGTAACTGCCCATGCTTTTGTTATAATTTTTTTTATAGTTATACCTATTATAATTGGGGGGTTTGGTAATTGATTAATTCCTTTAATATTAGGAAGGGTTGATATAGCATTTCCTCGGATAAATAATATGAGATTTTGATTATTAATTCCTTCAATTAATTTATTAGTTTTTAGGGGATTTATAAATATTGGGGTAGGTACTGGGTGAACTGTTTATCCCCCTTTATCATTAAATATTAGACATATAGGTATTTCTGTGGATATAGCAATTTTTTCTTTACATTTAGCAGGGGTTTCTTCTATTATAGGAGCTGTAAATTTTATTGTAACTATTATAAATATACGAAATAATAAAGTATTTATAGATAAAATTAGATTATTATGTTGATCTATTTTAATTACTGYAATTTTATTATTATTGTCTTTACCAGTTTTAGCYGGGGCAATTACAATATTATTAACTGATCGTAATTTAAATACATCTTTTTTTGACCCAGCAGGTGGAGGGGATCCTGTTTTATACCAGCATTTATTT. The long vein 2-SR+M of forewing (ca. as long as vein m-cu), ovipositor sheath ca. 1.2 × as long as body and simple tarsal claws are shared with the *H.texana* (Muesebeck, 1932) from USA and Mexico. However, the new species has vein 1Cua distinctly wider than vein 2-Cu1 (similar in *H.texana*), POL ca. equal to diameter of posterior ocellus (ca. 2 × diameter of posterior ocellus in *H.texana*), vertex laterally pale yellowish (dark brown in *H.texana*) and vein cu-a of forewing inclivous (vertical in *H.texana*).

###### Holotype ♀.

Guanacaste, Sector San Cristobal, Tajo Angeles, 10.86472, -85.41531, 540 meters, caterpillar collection date: 23/vii/2011, wasp eclosion date: 15/viii/2011. Depository: CNC.

***Host data*.** elachBioLep01 BioLep754 (Depressariidae) feeding on *Tapirirabrenesii* (Anacardiaceae).

***Caterpillar and holotype voucher codes*.** 11-SRNP-2902, DHJPAR0045062.

###### Paratypes.

Host = *Stenoma* BioLep86: DHJPAR0035531, DHJPAR0035533. Depository: CNC.

###### Etymology.

*Hymenochaoniakateperezae* is named to honor Ms. Kate Perez for her many years laboring in execution and administration of the field and laboratory actions performed by the Centre for Biodiversity Genomics to rapidly and accurately DNA barcode tens of thousands of Costa Rican insects from Malaise traps installed by ACG and BioAlfa.

**Figure 286.**
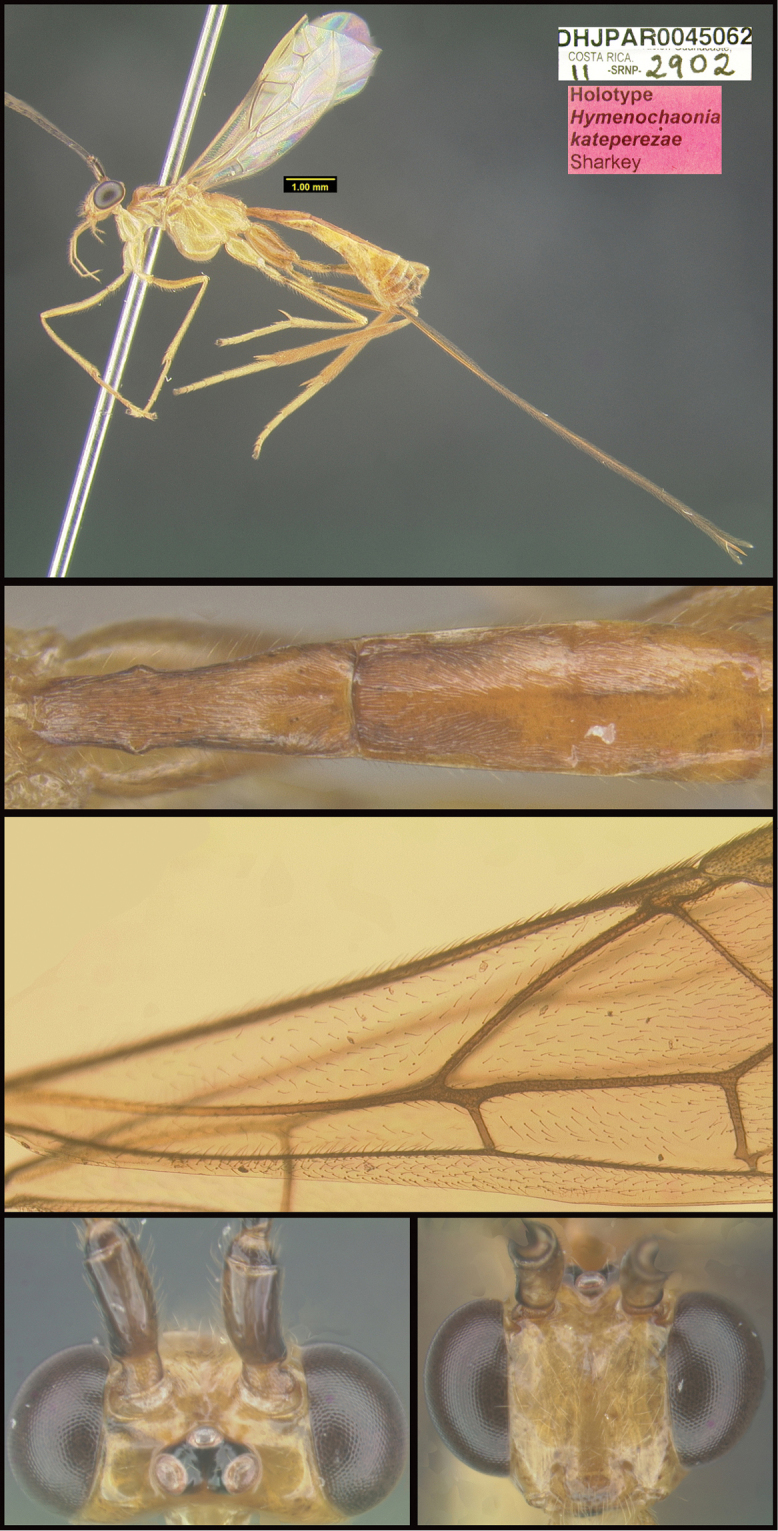
*Hymenochaoniakateperezae*, holotype.

##### Hymenochaonia katherinebaillieae

Taxon classificationAnimaliaHymenopteraBraconidae

Sharkey & van Achterberg sp. nov.

http://zoobank.org/C8603A01-D272-42BE-8892-86B12DE3B76D

Figure 287

###### Diagnostics.

BOLD:ACK7901. Consensus barcode. WATATTATATTTTTTATTTGGRATTTGATCWGGGATAATTGGTTTATCAATAAGTTTAATTATTCGTTTAGAGTTAGGTCAATCTGGTTCATTAATTGGTAATGATCAAATTTATAATAGTATAGTTACTTCTCATGCTTTTATTATAATTTTTTTTATAGTWATACCAATTATAATTGGAGGATTTGGRAATTGATTAATTCCTTTAATATTAGGTAGAATTGATATAGCTTTYCCTCGAATAAATAATATAAGATTTTGRTTAYTRATTCCTTCAATTAAATTATTAATTTTAAGGGGATTTATAAATATTGGRGTWGGTACTGGTTGAACAGTTTAYCCYCCTTTATCATTAAATATAAGACATATAGGYATTTCTGTAGATATATCWATTTTTTCTTTACATTTGGCGGGTATTTCTTCTATTATAGGKTCAATTAATTTTATTGTWACTATTTTAAATATACGAAATTATGGARTTTTAATAGATAAAATTAGTTTATTATGTTGATCTATTTTAATTACTGTTATTTTATTATTATTKTCATTRCCTGTRTTGGCTGGTGCAATYACAATATTATTRACTGATCGTAATTTAAATACATCTTTTTTTGATCCTTCAGGRGGTGGRGAYCCTGTTTTATATCAACATTTATTT. Similar to *H.kateperezae* sp. nov. because of the long vein 2-SR+M of forewing (ca. as long as vein m-cu) and moderately widened and medium-sized vein 1Cua of forewing, but differs by having a brownish stripe in the subbasal cell of forewing (absent in *H.kateperezae*), vein m-cu of forewing much longer than vein 3-Cu1 (slightly longer in *H.kateperezae*), hind tarsus brownish yellow (ivory in *H.kateperezae*), tarsal claws with lamella (absent in *H.kateperezae*) and vein cu-a of forewing subvertical (inclivous in *H.kateperezae*). The long vein 2-SR+M of forewing (ca. as long as vein m-cu), simple tarsal claws and distinctly widened marginal cell of hind wing are shared with the *H.texana* (Muesebeck, 1932) from USA and Mexico. However, the new species has vein 1Cua distinctly wider than vein 2-Cu1 (similar in *H.texana*), claws with ventral lamella (with no lamella in *H.texana*), ovipositor sheath ca. as long as body (distinctly longer than body in *H.texana*) and first metasomal tergite hardly widened posteriorly (distinctly widened in *H.texana*).

###### Holotype ♀.

Alajuela, Sector San Cristobal, Corrales Viejos, 10.89974, -85.38085, 495 meters, caterpillar collection date: 8/xi/2003, wasp eclosion date: 18/xii/2003. Depository: CNC.

***Host data*.***Quadraformaobliqualis* (Pyralidae) feeding on *Myrciasplendens* (Myrtaceae)

***Caterpillar and holotype voucher codes*.** 03-SRNP-34090, DHJPAR0029337.

###### Paratypes.

Hosts = *Quadraformaobliqualis*, *Pococerasadotha*, *Carthara* abruptaDHJ01, *Carthara* abruptaDHJ02, *Chloropaschiapegalis*, *Cartharaabrupta*, *Chloropaschiagranitalis*, *Accinctapubes* albifasciataDHJ03, *Accinctapubes* albifasciataDHJ01, *Chloropaschiamennusalis*, *Chloropaschia* Janzen3596, *Tancoacrinite*, *Deuterollytamajuscule*, *Chloropaschia* Janzen05 (all Pyralidae). DHJPAR0009297, DHJPAR0009298, DHJPAR0029180, DHJPAR0028980, DHJPAR0009658, DHJPAR0009767, DHJPAR0028996, DHJPAR0029369, DHJPAR0030262, DHJPAR0030263, DHJPAR0030264, DHJPAR0030265, DHJPAR0037792, DHJPAR0037865, DHJPAR0037866, DHJPAR0037867, DHJPAR0037868, DHJPAR0037869, DHJPAR0037870, DHJPAR0037871, DHJPAR0010512, DHJPAR0040229, DHJPAR0040230, DHJPAR0040231, DHJPAR0040232, DHJPAR0040235, DHJPAR0040236, DHJPAR0040237, DHJPAR0040238, DHJPAR0040240, DHJPAR0040609, DHJPAR0041130, DHJPAR0041131, DHJPAR0041133, DHJPAR0041134, DHJPAR0041135, DHJPAR0041136, DHJPAR0041137, DHJPAR0041209, DHJPAR0041210, DHJPAR0041211, DHJPAR0041212, DHJPAR0041213, DHJPAR0041214, DHJPAR0041216, DHJPAR0041217, DHJPAR0041218, DHJPAR0041219, DHJPAR0041220, DHJPAR0041221, DHJPAR0042391, DHJPAR0042392, DHJPAR0045055, DHJPAR0045072, DHJPAR0045378, DHJPAR0045813, DHJPAR0045819, DHJPAR0045821, DHJPAR0045829, DHJPAR0046725, DHJPAR0046726, DHJPAR0046727, DHJPAR0046728, DHJPAR0046729, DHJPAR0048080, DHJPAR0048082, DHJPAR0048084, DHJPAR0048085, DHJPAR0050913, DHJPAR0050918, DHJPAR0054448, DHJPAR0054449, DHJPAR0054450, DHJPAR0054451, DHJPAR0054452, DHJPAR0055123, DHJPAR0062983, DHJPAR0048088. Depository: CNC.

###### Etymology.

*Hymenochaoniakatherinebaillieae* is named to honor science reporter Ms. Katherine Baillie for her perceptive efforts at the University of Pennsylvania to popularize the ACG process and bioinventory.

**Figure 287.**
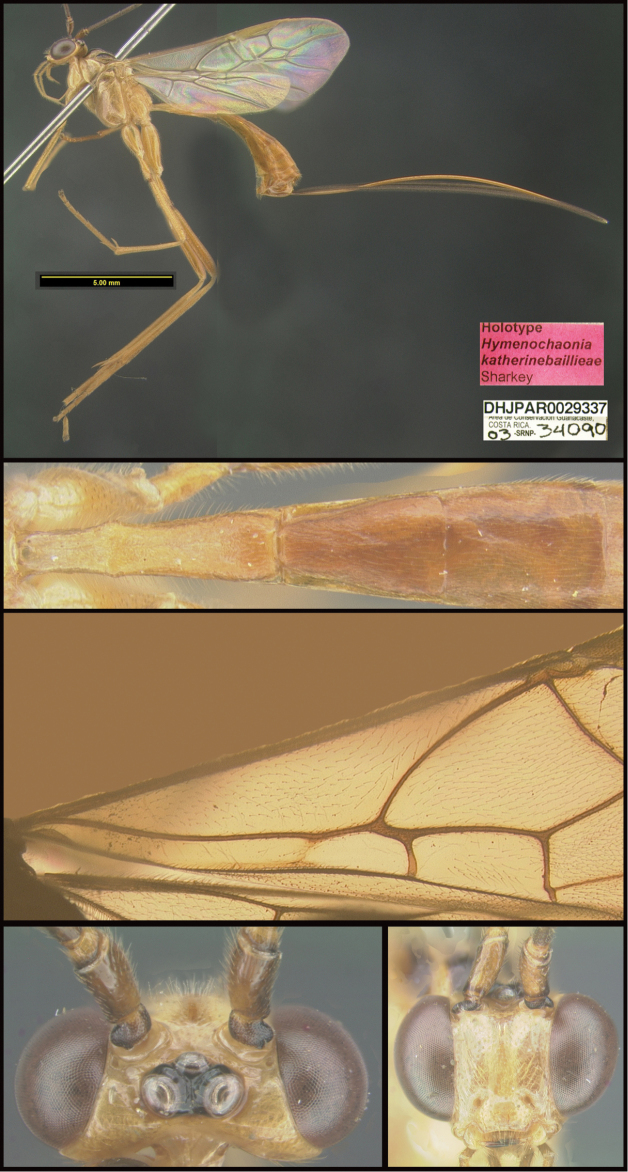
*Hymenochaoniakatherinebaillieae*, holotype.

##### Hymenochaonia katherineellisonae

Taxon classificationAnimaliaHymenopteraBraconidae

Sharkey & van Achterberg sp. nov.

http://zoobank.org/4DB4268F-028B-42DB-BCED-6876461FB5CA

Figure 288

###### Diagnostics.

BOLD:ADF5149. Consensus barcode. GTATACTTTATTTTTTATTTGGTATGTGATCAGGAATATTGGGGYTATCTATAAGTGTGTTAATTCGTTTAGAATTAGGATGTTCTGGCACTCTTATTAATAATGATCAAATTTATAATAGATTTGTTACTGCTCATGCTTTTATTATAATTTTTTTTATGGTTATACCTATTATAATTGGGGGGTTTGGTAATTGATTAGTTCCTTTAATGTTGGGGAGTGTTGATATAGCTTTCCCTCGAATAAATAATATGAGATTTTGATTATTAATTCCTTCTATTAATATATTGTTATTAAGGGGTTTTATTAATATTGGGGTAGGTACTGGTTGAACAGTTTATCCTCCTTTATCTTTAAATGTAAGTCATATAGGTATTTCTGTTGATATAGCTATTTTTTCTTTACATTTAGCGGGTATTTCTTCAATTATAGGGGCTATTAATTTTATTGTTAGAATTTTAAATATACGAAATTATGGAATTTTAATAGAAAAAATTAGGTTAATATGTTGATCTATTTTAATTACTGCTATTTTATTATTACTTTCATTGCCTGTATTGGCTGGTGCTATTACAATGTTGTTAACTGAT. An easily recognizable species because of the robust (ca. 2 × as long as wide apically) and black first metasomal tergite, the simple tarsal claws and the dark brown stigma.

###### Holotype ♀.

Guanacaste, Sector Cacao, Derrumbe, 10.9292, -85.4643, 1220 meters, 20/xi/2014, Malaise trap. Depository: CNC.

***Host data*.** None.

***Holotype voucher code*.**BIOUG31549-B03.

###### Paratypes.

All Malaise-trapped: BIOUG33120-C06, BIOUG36548-H01. Depository: CNC.

###### Etymology.

*Hymenochaoniakatherineellisonae* is named to honor Ms. Katherine Ellison for her support for Dr. Gretchen Daily’s efforts to integrate wild biodiversity with national socioeconomics.

**Figure 288.**
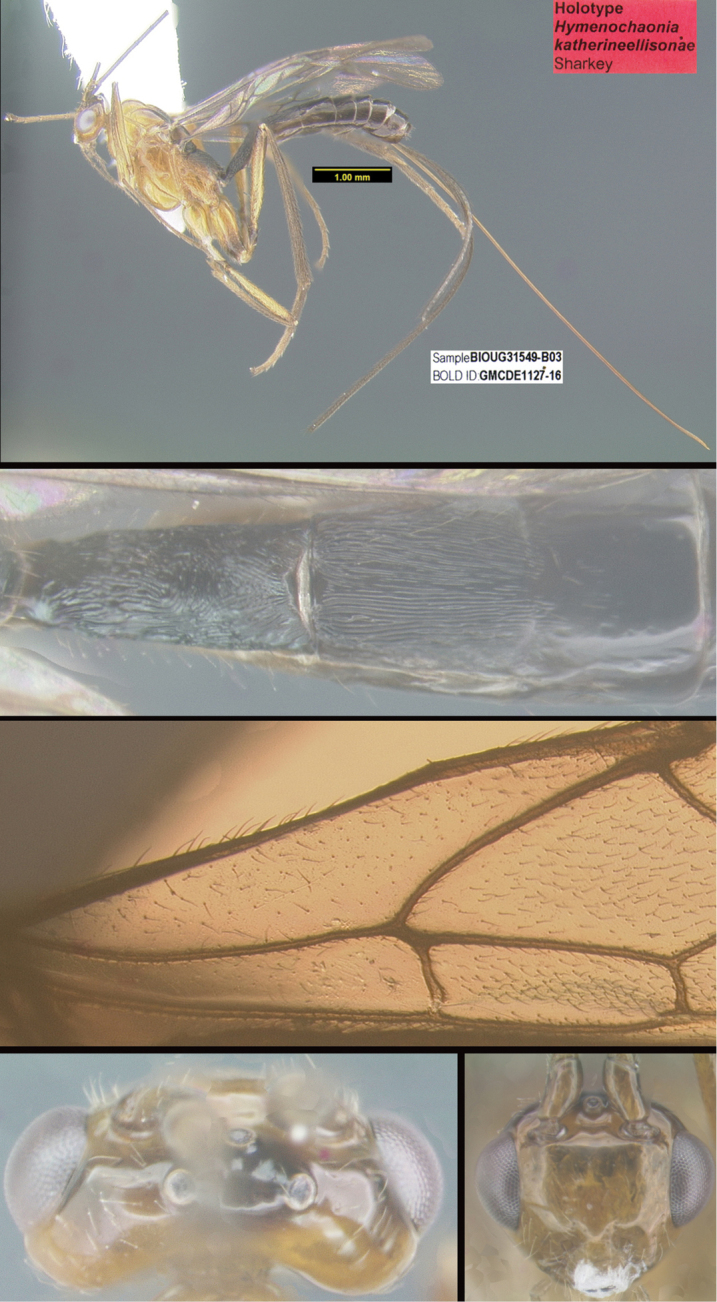
*Hymenochaoniakatherineellisonae*, holotype.

##### Hymenochaonia katyvandusenae

Taxon classificationAnimaliaHymenopteraBraconidae

Sharkey & van Achterberg sp. nov.

http://zoobank.org/D7B7EC4D-EC48-48D8-B7BD-DFF393CEF41A

Figure 289

###### Diagnostics.

BOLD:AAL5547. Consensus barcode. AATATTATATTTTTTATTTGGGATATGATCAGGAATAATTGGTTTGTCAATAAGTTTAATTATTCGATTGGAATTAGGTCAATCAGGTACATTAATTGGTAATGATCAAATTTATAATAGGTTTGTTACTTCACATGCATTTATTATAATTTTTTTCATAGTTATACCAATTATAATTGGAGGGTTTGGGAATTGATTAATCCCTTTAATATTAGGAAGAGTTGATATAGCTTTCCCTCGAATAAATAATATAAGATTTTGGTTATTAATTCCTTCTTTAATAATGTTAATTTTAAGAGGATTTGTAAATATTGGGGTGGGTACTGGTTGAACAGTTTACCCCCCTTTATCATTAAATATTAGACATATAGGAATTTCTGTAGATATAGCTATTTTTTCTTTACATTTAGCAGGAATTTCATCAATTATAGGGGCTATTAATTTTATTGTAACAATTATAAATATACGAAATTATGGAGTAATAATAGATAAAATTAGATTATTAAGTTGATCAATTTTAATCACTACTATTTTGTTATTATTATCATTACCTGTATTGGCTGGTGCTATTACTATATTATTAACTGATCGAAATTTAAATACGTCTTTTTTTGACCCTTCAGGAGGTGGGGATCCAATTTTATATCAACATTTATTT. An easily recognizable species because of the unique oval shape of vein cu-a of forewing; in addition, the vein is shorter than normal (subequal to length of vein 1Cua), the first metasomal tergite is very slender and the face is bicolored.

###### Holotype ♀.

Alajuela, Sector Rincon Rain Forest, Sendero Albergue Crater, 10.84886, -85.3281, 980 meters, caterpillar collection date: 1/iii/2010, wasp eclosion date: 29/iii/2010. Depository: CNC.

***Host data*.***Pilocrocispurpurascens* (Crambidae) feeding on *Pentagoniadonnell-smithii* (Rubiaceae).

***Caterpillar and holotype voucher codes*.** 10-SRNP-1080, DHJPAR0038863.

###### Paratypes.

Host = *Pilocrocispurpurascens* (Crambidae): DHJPAR0036780, DHJPAR0037835, DHJPAR0039536, DHJPAR0040227, DHJPAR0041215, DHJPAR0042732, DHJPAR0052091, DHJPAR0052092, DHJPAR0052093, DHJPAR0057264, DHJPAR0057303. Depository: CNC.

###### Etymology.

*Hymenochaoniakatyvandusenae* is named to honor Mrs. Katy Van Dusen of Monteverde and Cuajiniquil for her decades of support of ACG activities with birds and marine projects, and chief collaborator with Frank Joyce.

**Figure 289.**
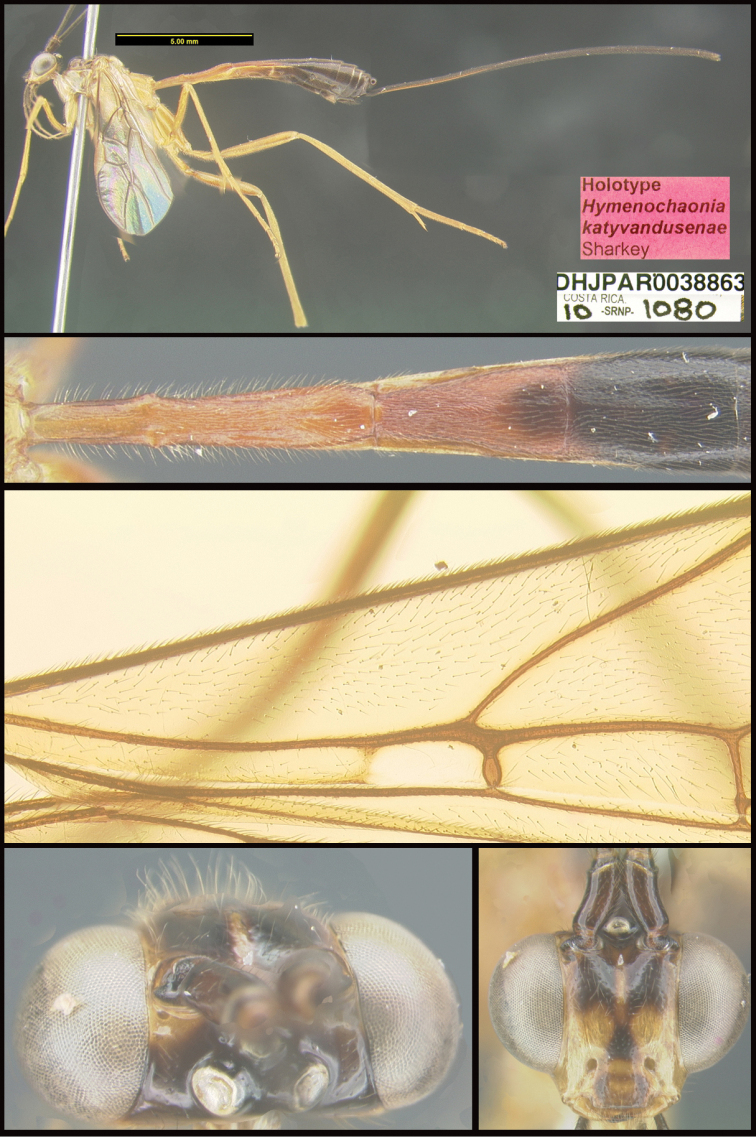
*Hymenochaoniakatyvandusenae*, holotype.

##### Hymenochaonia kazumifukunagae

Taxon classificationAnimaliaHymenopteraBraconidae

Sharkey & van Achterberg sp. nov.

http://zoobank.org/68362CB0-A909-4CAD-B881-1F54F35C1D2E

Figure 290

###### Diagnostics.

BOLD:AAX3464. Consensus barcode. GTTTTATATTTTTTATTTGGGATATGATCAGGAATAATTGGTTTATCTATAAGAATAATTATTCGTTTAGAATTAGGGCAATC---AGGGACTTTAATTAATAATGATCAAATTTATAATAGATTTGTAACTTCACATGCATTTATTATAATTTTTTTTATAGTTATACCAATTATAATCGGGGGGTTTGGAAATTGGTTAATTCCTTTAATATTAGGTAGGGTAGATATAGCTTTCCCTCGGATGAATAATATAAGATTTTGATTATTAATTCCATCATTAATAATATTGATTTTAAGGGGATTTATAAATATTGGGGTAGGTACGGGATGAACAGTATATCCTCCTTTATCTTTGAATGTAAGGCATATAGGGGTTTCAGTTGATATAGCTATTTTTTCTTTACATTTAGCTGGTATTTCCTCTATTATGGGGGCTATAAATTTTATTATTACTATTTTAAATATACGAAATTATGGGGTATTAATAGATAAAATTAGATTATTATGTTGATCAGTTTTAATTACTGCAATTTTATTATTATTATCATTGCCAGTTTTAGCAGGAGCTATTACAATATTATTAACTGATCGAAATTTAAATACATCTTTTTTTGATCCATCAGGAGGGGGGGATCCAATTTTGTATCAACATTTATTT. Both *H.keithlangdoni* and *H.kazumifukunagae* have the ovipositor sheath and antenna of ♀ medially white or ivory combined with a strongly widened vein 1Cua of forewing which is shorter than curved vein cu-a. Differs from *H.keithlangdoni* by the longer ovipositor sheath (approx. 1.3 × longer than body) and largely dark head and mesosoma.

###### Holotype ♀.

Guanacaste, Sector Del Oro, Quebrada Serrano, 11.00025, -85.45614, 585 meters, caterpillar collection date: 19/vii/2007, wasp eclosion date: 10/viii/2007. Depository: CNC.

***Host data*.** tortricid 07-SRNP-22617 (Tortricidae) feeding on *Hampeaappendiculata* (Malvaceae).

***Caterpillar and holotype voucher codes*.** 07-SRNP-22618, DHJPAR0021643.

###### Paratypes.

None.

###### Etymology.

*Hymenochaoniakazumifukunagae* is named to honor Mrs. Kazuma Fukunaga of Japan for her valiant efforts with the Japan Children’s Rainforest to seek funding for the initiation of ACG land purchase.

**Figure 290.**
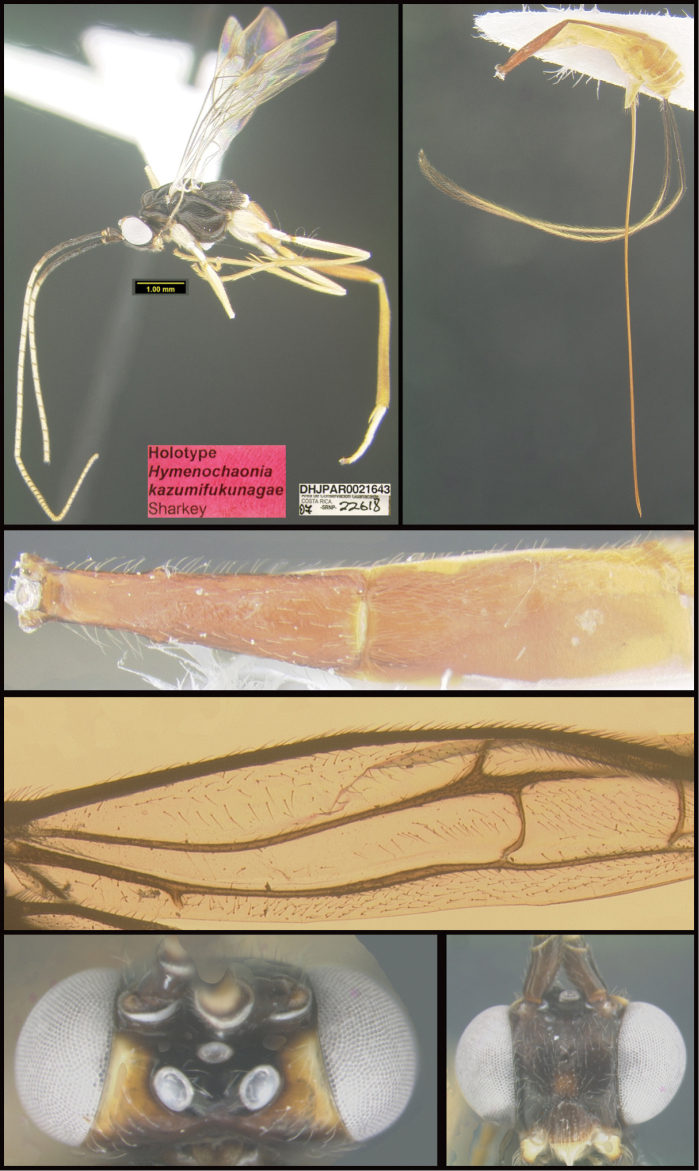
*Hymenochaoniakazumifukunagae*, holotype.

##### Hymenochaonia keithlangdoni

Taxon classificationAnimaliaHymenopteraBraconidae

Sharkey & van Achterberg sp. nov.

http://zoobank.org/BEB26BEC-9AE7-41BD-A266-B27AF8A33C35

Figure 291

###### Diagnostics.

BOLD:AAC3252. Consensus barcode. TATATTATATTTTTTATTTGGAATATGATCGGGAATAATTGGTTTATCAATAAGTATAATTATTCGTTTAGAATTAGGTCAATCAGGAACTTTAATTAATAATGATCAAATTTATAATAGATTTGTAACTTCTCATGCATTTATTATAATTTTTTTTATAGTTATACCAATTATAATTGGAGGGTTTGGAAATTGATTAATTCCATTAATATTAGGAAGAGTTGATATGGCTTTCCCTCGAATAAATAATATAAGATTTTGATTGTTAATTCCTTCATTAATAATATTAATTTTAAGAGGATTTATAAATATTGGAGTTGGTACAGGATGAACAGTTTATCCACCTTTATCTTTAAATGTAAGTCATATAGGAATTTCAGTTGATATAGCTATTTTTTCTTTACATTTAGCTGGAATTTCATCTATTATGGGAGCTATTAATTTTATTGTTACTATTTTAAATATACGAAATTATGGGGTGTTAATAGATAAAATTAGTTTATTATGTTGATCAATTTTAATTACAGCTATTTTATTATTATTATCATTACCAGTTTTAGCTGGAGCTATTACTATATTATTAACTGATCGAAATTTAAATACATCTTTTTTTGATCCTTCAGGGGGGGGGGATCCAATTTTGTATCAACATTTATTT. Both *H.keithlangdoni* and *H.kazumifukunagae* have the ovipositor sheath and antenna of ♀ medially white or ivory combined with a strongly widened vein 1Cua of forewing which is shorter than curved vein cu-a. Differs from *H.kazumifukunagae* by the shorter ovipositor sheath (approx. as long as body) and largely ivory head, mesopleuron and metapleuron.

###### Holotype ♀.

Guanacaste, Sector Cacao, Sendero Segundo, 10.92679, -85.45332, 1180 meters, caterpillar collection date: 30/vii/2007, wasp eclosion date: 1/ix/2007. Depository: CNC.

***Host data*.** gelJanzen01 Janzen356 (Depressariidae) feeding on *Hampeaappendiculata* (Malvaceae)

***Caterpillar and holotype voucher codes*.** 07-SRNP-36311, DHJPAR0021647.

###### Paratypes.

Hosts = *Conchylodessalamisalis* (Crambidae) *Dichomeris* Janzen169 (Gelechiidae). DHJPAR0040592, DHJPAR0040597. Depository: CNC.

###### Etymology.

*Hymenochaoniakeithlangdoni* is named to honor Mr. Keith Langdon of the US National Park Service for his initiation of the ongoing biodiversity inventory of Great Smoky Mountains National Park.

**Figure 291.**
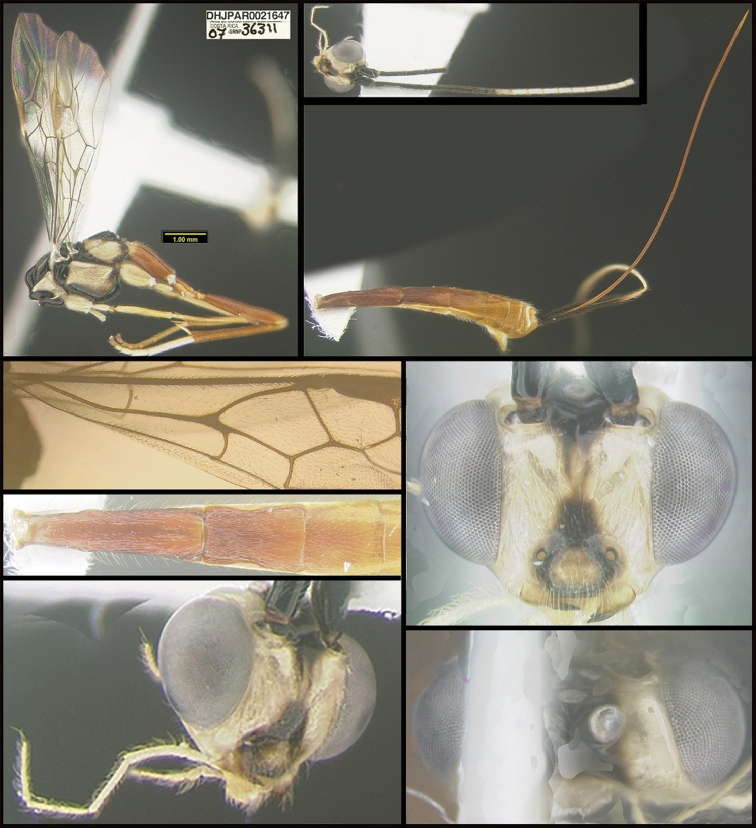
*Hymenochaoniakeithlangdoni*, holotype.

##### Hymenochaonia keithwillmotti

Taxon classificationAnimaliaHymenopteraBraconidae

Sharkey & van Achterberg sp. nov.

http://zoobank.org/C293B1D1-5506-4E93-8910-03178374185D

Figure 292

###### Diagnostics.

BOLD:AAP9457. Consensus barcode. TATATTATATTTTATATTTGGAATATGGTCAGGGATAATTGGTTTATCAATAAGAATAATTATTCGTTTAGAAYTAGGTCAATCTGGTACTTTAATTAATAATGATCAAATTTATAATAGATTTGTTACTTYTCATGCATTTATTATAATTTTTTTTATAGTTATACCAATTATAATTGGAGGGTTTGGTAATTGATTGATTCCTTTAATATTAGGTAGGGTAGATATAGCTTTYCCTCGAATAAATAATATAAGATTTTGGTTATTAATTCCATCTTTAATAATATTAATTTTAAGGGGATTTATAAATATTGGTGTAGGAACTGGTTGGACTGTTTATCCTCCTTTATCTTTAAATGYAAGTCATATAGGAATTTCAGTAGATATAGCAATTTTTTCATTACATTTAGCGGGWATTTYTTCTATTTTAGGTGCCATTAATTTTATTATTACTATTATAAATATACGAAATTATGGAGTATTAATAGATAAAATTAGATTATTATCTTGATCAATTTTAATTACAGCAATTTTATTATTATTATCATTACCAGTTTTAGCAGGTGCTATTACTATATTATTAACTGATCGGAATTTAAATACATCTTTTTTTGATCCTTCAGGGGGGGGGGRTCCTATTTTATATCAACATTTATTT. This species and *H.kalevikulli* sp. nov. share a long and widened vein 1Cua of forewing, antenna (except blackish base) pale yellowish or whitish and distinctly curved vein cu-a of forewing. *Hymenochaoniakeithwillmotti* differs by having vein 1Cua of forewing approx. as long as vein cu-a, tarsal claws with ventral lamella, hind tibia brownish yellow, subbasal cell of forewing setose medially and apical half of metasoma yellowish.

###### Holotype ♀.

Alajuela, Sector Rincon Rain Forest, Rio Francia Arriba, 10.89666, -85.29003, 400 meters, caterpillar collection date: 13/ix/2011, wasp eclosion date: 2/x/2011. Depository: CNC.

***Host data*.** gelJanzen01 Janzen407 (Gelechiidae) feeding on *Clibadiumpittieri* (Asteraceae).

***Caterpillar and holotype voucher codes*.** 11-SRNP-44212, DHJPAR0048081.

###### Paratypes.

Host = gelJanzen01 Janzen461 (Gelechiidae), elachJanzen01 Janzen272 (Depressaridae), *Dichomeris* Janzen703 (Gelechiidae), gelJanzen01 Janzen407 (Gelechiidae): DHJPAR0040613, DHJPAR0040570, DHJPAR0040601, DHJPAR0040603, DHJPAR0040606, DHJPAR0040607, DHJPAR0041403, DHJPAR0042758, DHJPAR0043246, DHJPAR0048083, DHJPAR0048086, DHJPAR0048087. Depository: CNC.

###### Etymology.

*Hymenochaoniakeithwillmotti* is named to honor Dr. Keith Willmott for his deep identification and publication efforts for Costa Rican butterflies in general, and ACG species in particular.

**Figure 292.**
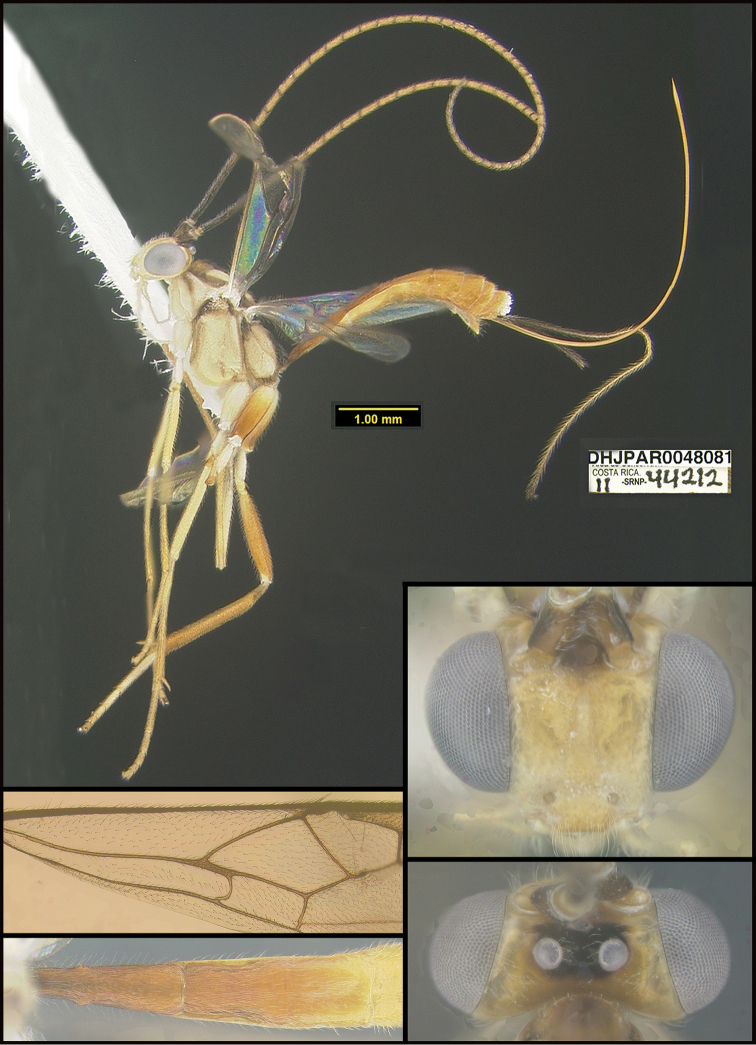
*Hymenochaoniakeithwillmotti*, holotype.

##### Hymenochaonia kenjinishidai

Taxon classificationAnimaliaHymenopteraBraconidae

Sharkey & van Achterberg sp. nov.

http://zoobank.org/80AB4E2E-9C95-47BE-9F19-8220D975C913

Figure 293

###### Diagnostics.

BOLD:ACG8190. Consensus barcode. TATATTATATTTTATATTTGGGATATGATCAGGGTTAATTGGTTTATCAATAAGATTAATTATTCGAATAGAGTTAGGTCAATCTGGTTTATTTATTGGTAATGATCAAATTTATAATAGTATAGTTACTGCACATGCTTTTATTATAATTTTTTTTATAGTTATACCAATTATAATTGGGGGTTTTGGTAATTGATTAATTCCTTTAATATTAGGAAGTGTGGATATGGCTTTCCCTCGGATAAATAATATAAGATTTTGGTTATTATTCCCTTCAATTATAATATTAATTTTTAGAGGTTTTATAAATATTGGTGTTGGTACTGGTTGAACTGTTTATCCTCCTTTATCTATAAATATTAGACATATAGGTATATCAGTTGATATAGCTATTTTTTCTCTTCATTTAGCTGGGATTTCTTCAATTATAGGTTCTATTAATTTTATTGTTACAATTATAAATATACGAAATTATATAGTGTTAATAGATAAAATTAGTTTATTTTGTTGATCAGTATTAATTACAGTTATTTTATTGTTATTATCTTTGCCTGTTTTAGCTGGTGCAATTACAATATTATTAACTGATCGAAATTTAAATACTTCTTTTTTTGATCCTGCTGGAGGGGGGGATCCAGTTTTATATCAACATTTATTT. Very similar to *H.pallida* (Cresson, 1865) because of the pale colored body and stigma, slender first metasomal tergite and simple tarsal claws. The new species differs by having the first subdiscal cell of forewing approx. 1.5 × longer than wide (approx. 2.5 × in *H.pallida*), vein cu-a of forewing curved (straight) and mesosoma with dark brown pattern (entirely brownish yellow).

###### Holotype ♂.

Guanacaste, Sector Santa Rosa, Bosque San Emilio, 10.8438, -85.6138, 300 meters, 4/vii/2012, Malaise trap. Depository: CNC.

***Host data*.** None.

***Holotype voucher code*.**BIOUG06141-G06.

###### Paratypes.

None.

###### Etymology.

*Hymenochaoniakenjinishidai* is named to honor Dr. Kenji Nishida for his intense efforts to read and understand Costa Rican biodiversity.

**Figure 293.**
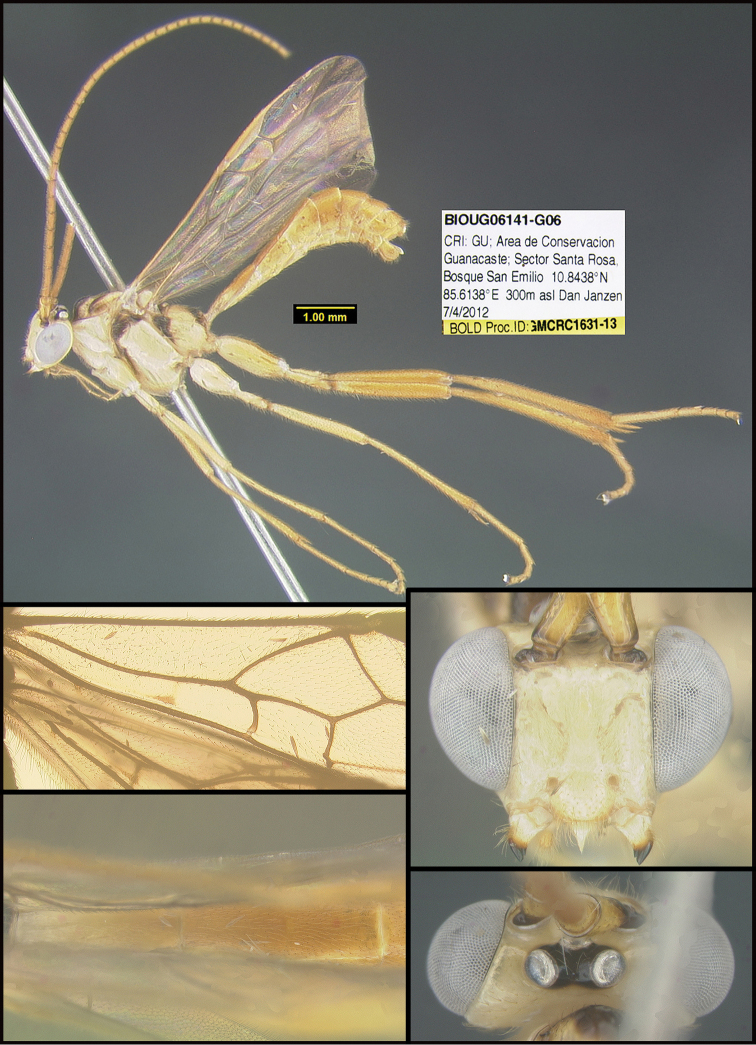
*Hymenochaoniakenjinishidai*, holotype.

##### Hymenochaonia kimberleysheldonae

Taxon classificationAnimaliaHymenopteraBraconidae

Sharkey & van Achterberg sp. nov.

http://zoobank.org/0BC2030D-E975-419E-BED7-2952D883CD9F

Figure 294

###### Diagnostics.

BOLD:AAB4677. Consensus barcode. AATATTATATTTTTTATTTGGGTTATGATCTGGAATAATTGGGTTATCAATTAGATTAATTATTCGTATAGAACTAAGACAATCAGGGGTTTTATTAAATAATGATCAAATTTATAATAGGTTAGTTACTATGCATGCTTTTATTATAATTTTTTTTATAGTTATACCAATTATAATTGGTGGATTTGGAAATTGGTTAATTCCTTTAATGTTGGGTAGAGTGGATATGGCTTTTCCTCGTATAAATAATATAAGATTTTGGTTATTAATTCCTTCATTAATATTATTAATTTTAAGAGGTTTTATAAATATTGGGGTTGGAACTGGATGAACAGTGTATCCTCCTCTTTCATTAAATGTTAGACATATAGGTATTTCTGTAGATATAGCTATTTTTTCTTTACATTTAGCTGGAATTTCTTCAATTATAGGAGCTATTAATTTTATTAGAACAATTTTAAATATACGTAATTATGGGGTTTTAATAGATAAAATTAGATTAATATGTTGATCTATTTTAATTACTGCTATTTTATTATTATTATCTTTACCTGTATTGGCAGGAGCTATCACAATATTATTAACAGATCGTAATTTAAATACTTCTTTTTTTGATCCTYCGGGAGGGGGGGACCCTATTTTATATCAACATTTATTT. The new species shares with *H.maculicoxa* (Enderlein, 1920) the elongate and largely transversely striate first metasomal tergite. *Hymenochaoniakimberleysheldonae* differs because it has vein cu-a of forewing longer and slightly narrower (shorter and slightly wider in *H.maculicoxa*), hind femur and coxa yellowish (mainly blackish) and first tergite slenderer, especially in front of spiracles (less slender).

###### Holotype ♀.

Guanacaste, Sector Pitilla, Sendero Naciente, 10.98705, -85.42816, 700 meters, caterpillar collection date: 25/i/2014, wasp eclosion date: 24/ii/2014. Depository: CNC.

***Host data*.***Desmia* BioLep06 (Crambidae) feeding on *Hoffmannialiesneriana* (Rubiaceae).

***Caterpillar and holotype voucher codes*.** 14-SRNP-30295, DHJPAR0055155.

###### Paratypes.

Hosts = *Syngamiaflorella*, *Desmiaploralis*, *Desmia* Janzen09, *Desmia* BioLep06, *Desmia* Janzen14, *Desmia* Solis19 (all Crambidae). DHJPAR0036730, DHJPAR0036724, DHJPAR0028982, DHJPAR0037872, DHJPAR0037874, DHJPAR0039388, DHJPAR0040233, DHJPAR0050353, DHJPAR0050914, DHJPAR0055154, DHJPAR0056290. Depository: CNC.

###### Etymology.

*Hymenochaoniakimberlysheldonae* is named to honor Dr. Kimberly Sheldon of the University of Tennessee for her intense and effective efforts to translate why “Mountain Passes are higher in the Tropics” to today’s concern for climate change impacts on biodiversity.

**Figure 294.**
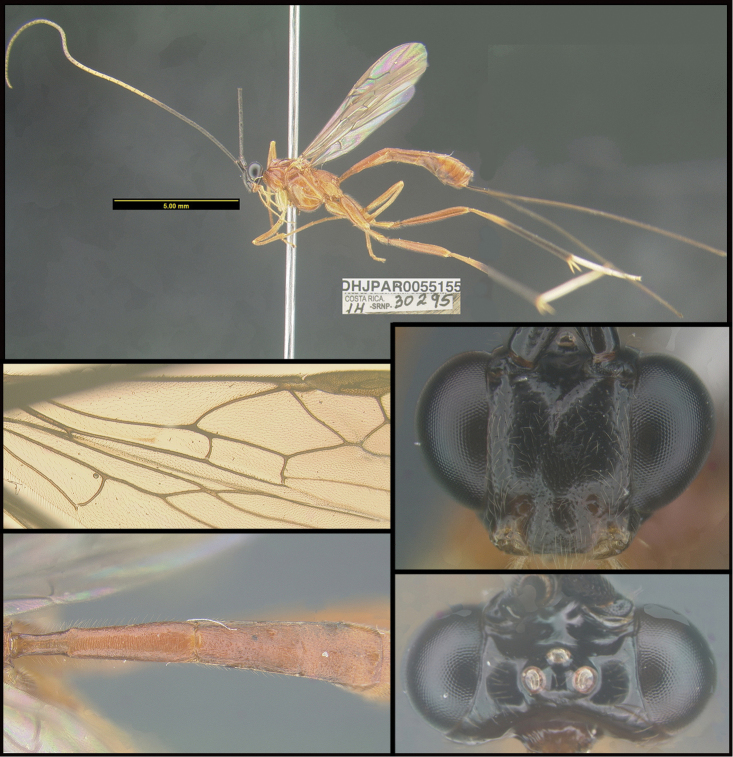
*Hymenochaoniakimberleysheldonae*, holotype.

##### Hymenochaonia krisnorvigae

Taxon classificationAnimaliaHymenopteraBraconidae

Sharkey & van Achterberg sp. nov.

http://zoobank.org/7A0B4044-DB83-4475-AC55-D90D948CBC83

Figure 295

###### Diagnostics.

BOLD:ACR5126. Consensus barcode. ATATTATATTTTTTATTCGGTATATGATCTGGTGTAATTGGTTTATCAATAAGATTAATTATTCGAATAGAATTAGGTCAATCTGGGTCTTTGATTGGTAATGATCAAATTTATAATAGTTTTGTTACTGCTCATGCTTTTATTATAATTTTTTTTATAGTTATACCAATTATAATTGGTGGGTTTGGTAATTGATTAATTCCTTTAATATTAGGAAGAGTTGATATAGCTTTCCCTCGGATAAATAATATAAGATTTTGATTATTAATTCCTTCAATTATATTATTAATTATAAGAGGATTTATAAATATTGGTGTAGGAACTGGGTGAACAGTTTATCCTCCTTTATCTTTAAATATTAGACATATAGGGATTTCAGTTGATATATCAATTTTTTCATTACATTTAGCAGGGGTATCCTCAATTATAGGTGCTATTAATTTTATTATTACAATTTTAAATATGCGAAATTATGGTGTATTAATAGATAAGATTAGATTATTATGTTGATCTGTATTAATTACAGCTATTTTATTATTATTATCATTACCAGTATTAGCAGGTGCTATTACTATATTATTAACAGAT------------------------------------------. Very similar to the Nearctic and Neotropical *H.delicata* (Cresson, 1872) because of its size, narrow temples, robust first metasomal tergite and pale yellow stigma, but differs by having simple tarsal claws (with small ventral lamella in *H.delicata*), vein cu-a of forewing slightly inclivous and straight (subvertical and slightly curved in *H.delicata*), vein 1Cua of forewing wider than vein 2-Cu1 (similar in *H.delicata*), T1–2 largely smooth and shiny (aciculate and less shiny in *H.delicata*) and malar space ca. as long as basal width of mandible (shorter in *H.delicata*).

###### Holotype ♀.

Guanacaste, Sector Santa Rosa, Bosque San Emilio, 10.8438, -85.6138, 300 meters, 16/iv/2012, Malaise trap. Depository: CNC.

***Host data*.** None.

***Holotype voucher code*.**BIOUG17470-D11.

###### Paratypes.

None.

###### Etymology.

*Hymenochaoniakrisnorvigae* is named to honor Mrs. Kris Norvig for her major contribution to the survival and land development of ACG.

**Figure 295.**
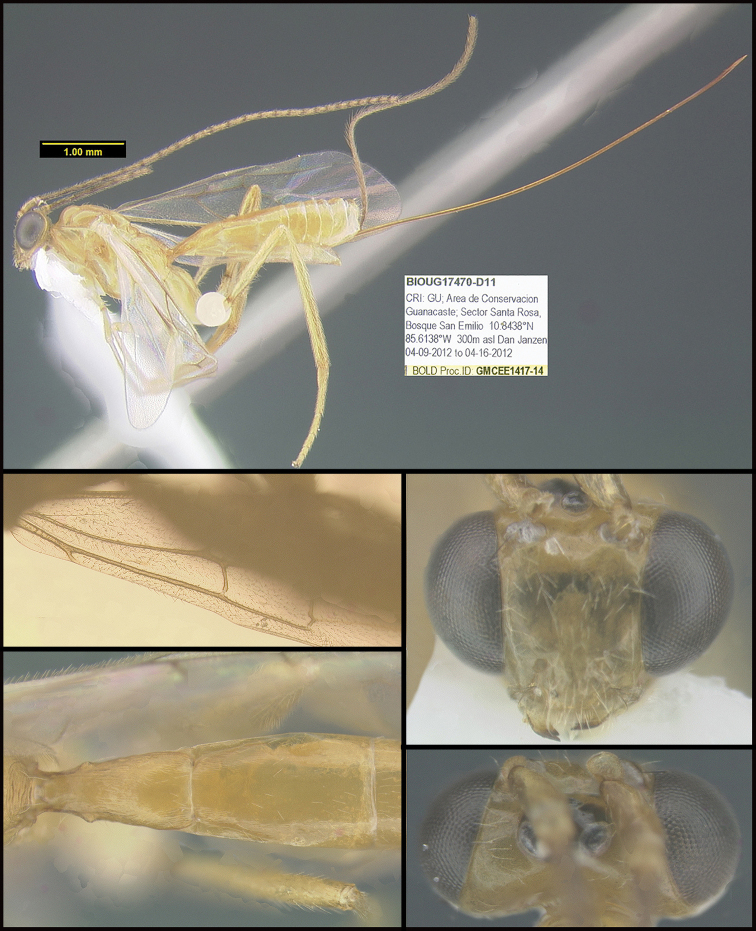
*Hymenochaoniakrisnorvigae*, holotype.

##### Hymenochaonia lilianamadrigalae

Taxon classificationAnimaliaHymenopteraBraconidae

Sharkey & van Achterberg sp. nov.

http://zoobank.org/E88AE694-95D9-43E1-9FA1-A7CAC0762F56

Figure 296

###### Diagnostics.

BOLD:AAA7058. Consensus barcode. GATATTGTATTTTTTATTTGGAATATGATCAGGAATAGTAGGTTTATCAAWAAGAATAATTATTCGAATGGAATTAGGTCAAGTGGGAGATTTAATTGGAAATGATCAAATTTATAATAGATTTGTTACTGCTCATGCATTTGTAATAATTTTTTTTATAGTTATACCAATTATAATTGGGGGATTTGGTAATTGRTTAATTCCTTTAATGTTAGGAAGAGTCGATATAGCTTTTCCTCGTATAAATAATATAAGATTTTGGTTATTAATTCCMTCAATTATATTATTAATTTTTAGTGGATTTATGAATATTGGGGCGGGGACTGGTTGAACAGTTTATCCACCTTTATCATTAAATATTAGACATATAGGAGTTTCRGTTGATATGGCAATTTTTTCTTTACATTTAGCTGGAATTTCYTCAATTATGGGTTCAGTTAATTTTATTATTACTATTATAAATATACGAAATTAYGGGGTATTAATAGAAAAAATTAGATTATTATGTTGATCAATTTTAATTACTGTAATTTTATTATTATTATCATTACCYGTATTAGCAGGTGCTATTACTATAYTATTAACTGATCGAAATTTAAATACTTCTTTCTTTGATCCTGCAGGTGGAGGTGATCCAATTTTATATCAGCATTTATTT. Strong sexual dimorphism, subbasal cell of forewing without brownish patch and sparsely setose, tarsal claws with ventral lamella, vein 1Cua of the forewing short and hardly widened, and vertex darkened this species is closely related to *H.nupera* (Cresson, 1872). *Hymenochaonialilianamadrigalae* differs by the yellowish brown mesoscutal lobes (more or less darkened) and POL approx. equal to diameter of posterior ocellus (approx. 1.6 × diameter of posterior ocellus).

###### Holotype ♀.

Guanacaste, Sector Santa Rosa, Sendero Natural, 10.83575, -85.61253, 290 meters, caterpillar collection date: 8/vi/2001, wasp eclosion date: 27/v/2002. Depository: CNC.

***Host data*.***Sylleptebelialis* (Crambidae) feeding on *Caseariacorymbosa* (Salicaceae).

***Caterpillar and holotype voucher codes*.** 01-SRNP-13023, DHJPAR0029366.

###### Paratypes.

Hosts = *Sylleptebelialis*. DHJPAR0029363, DHJPAR0029364, DHJPAR0029361, DHJPAR0029362, DHJPAR0035255, DHJPAR0035290, DHJPAR0055768, DHJPAR0055920.

###### Etymology.

*Hymenochaonialilianamadrigalae* is named to honor Mrs. Liliana Madrigal of Costa Rica for her seminal and founding role in all that is ACG since November 2005. Depository: CNC.

**Figure 296.**
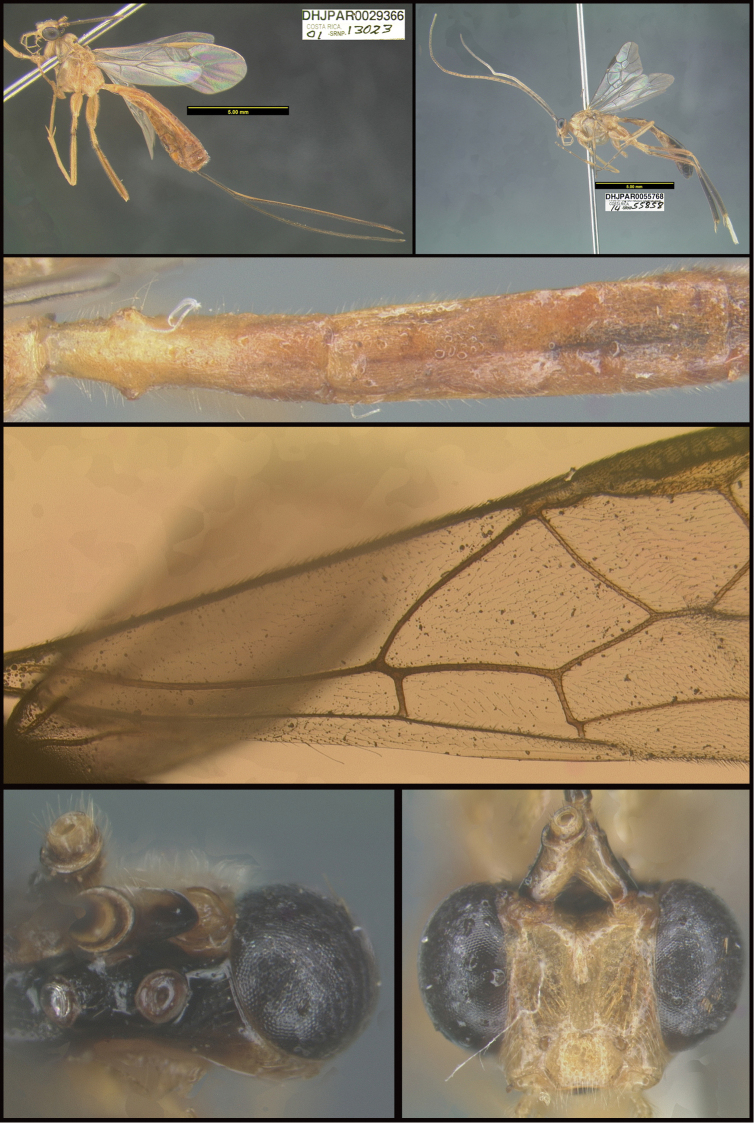
*Hymenochaonialilianamadrigalae* holotype female; except male paratype (top right) to show sexual dimorphism in color.

##### Hymenochaonia lizlangleyae

Taxon classificationAnimaliaHymenopteraBraconidae

Sharkey & van Achterberg sp. nov.

http://zoobank.org/4FC2E68A-3FB7-4E00-B401-B1BE6FE2650D

Figure 297

###### Diagnostics.

BOLD:ACK7939. Consensus barcode. AATATTATATTTTWTWTTTGGAATATGATCAGGAATAGTAGGTYTATCAATGAGAATAATTATTCGAATGGAATTAGGTCAAGTAGGGGATTTAATTGGAAATGATCAAATTTATAATAGATTTGTTACTGCTCATGCATTTGTAATAATTTTTTTTATGGTTATACCAATTATAATTGGGGGATTTGGTAATTGATTAATTCCATTAATATTAGGAAGAGTTGATATAGCTTTYCCTCGTATAAATAATATAAGGTTTTGATTATTAATTCCATCAATTATATTATTAATTTTTAGAGGATTYATAAATATTGGTGTAGGKACTGGATGAACAGTTTATCCACCTTTATCATTAAATATTAGACATATAGGAGTTTCAGTTGATATAGCAATTTTTTCTTTACATTTAGCTGGGRTTTCTTCAATTATAGGTTCAGTTAATTTTATTATTACTATTATAAATATACGAAATTAYGGAGTATTAATAGAAAAAATTAGRTTATTATGTTGATCAATTTTAATTACTGTGATTTTATTATTATTATCATTACCTGTATTAGCAGGTGCTATTACTATATTATTAACTGATCGAAATTTAAATACATCTTTTTTTGATCCTGCAGGTGGRGGGGATCCAATTTTATATCAACATTTATTT. Similar to *H.lilianamadrigalae* sp. nov. and *H.nupera* (Cresson, 1872) because of sharing the lack of a brownish patch in the subbasal cell of forewing, tarsal claws with ventral lamella, vein 1Cua of the forewing short and hardly widened, and darkened vertex. *Hymenochaonializlangleyae* differs by having the subbasal cell of forewing largely glabrous (sparsely setose in *H.nupera*), mesoscutal lobes yellowish brown (more or less darkened in *H.nupera*) and POL approx. 0.8 × diameter of posterior ocellus (approx. 1.6 × diameter of posterior ocellus in *H.nupera*).

###### Holotype ♀.

Guanacaste, Sector Santa Rosa, Bosque San Emilio, 10.84389, -85.61384, 300 meters, caterpillar collection date: 13/vii/2005, wasp eclosion date: 2/viii/2005. Depository: CNC.

***Host data*.***Eulepte* Solis15 (Crambidae) feeding on *Handroanthusochraceus* (Bignoniaceae).

***Caterpillar and holotype voucher codes*.** 05-SRNP-18593, DHJPAR0009306.

###### Paratypes.

Hosts = *Eulepte* Solis15, *Syllepteaechmisalis* (Crambidae). DHJPAR0009305, DHJPAR0009309, DHJPAR0009324, DHJPAR0009325, DHJPAR0009326, DHJPAR0009307, DHJPAR0009308, DHJPAR0029409, DHJPAR0028991, DHJPAR0036306, DHJPAR0041541, BIOUG06141-D03. Depository: CNC.

###### Etymology.

*Hymenochaonializlangleyae* is named to honor science writer Ms. Elizabeth Langley for her accurately popularizing the phenomenon of snake mimicry by ACG caterpillars.

**Figure 297.**
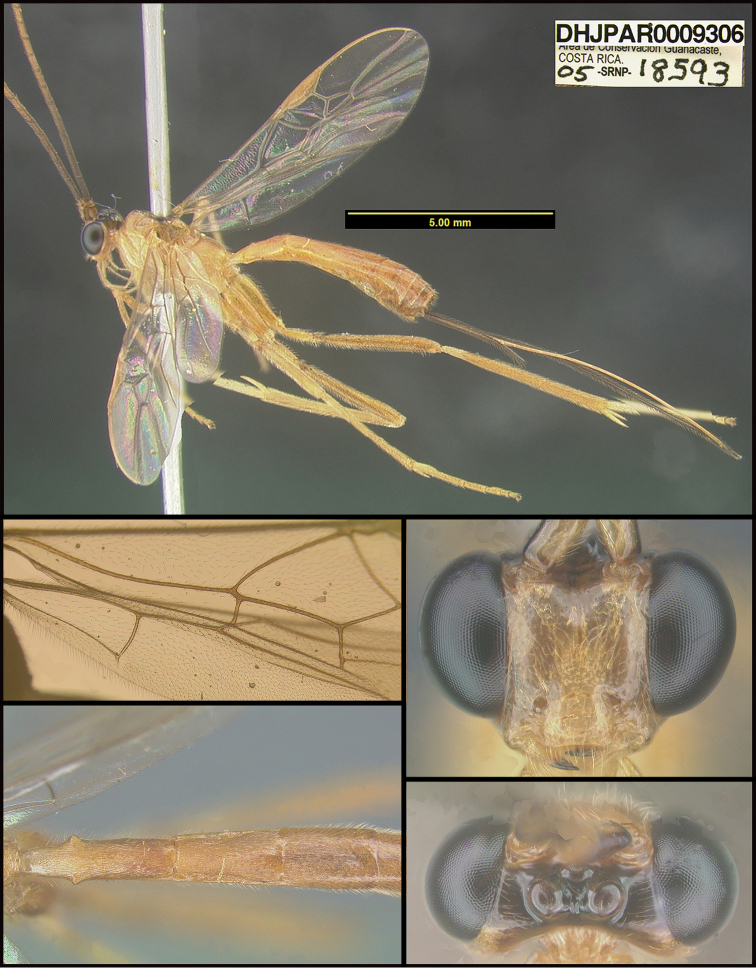
*Hymenochaonializlangleyae*, holotype.

#### *Macrocentrus* Curtis, 1833

*Macrocentrus* is worldwide in distribution with 190 described species, a few of which have been widely used in biological control. There are three species from Mexico described by Enderlein (1920) and one by Wharton (1984). It is possible that some of the Mexican species may be conspecific with some of those proposed here. Species of *Macrocentrus* have been recorded from a wide range of lepidopteran families. COI barcodes have worked remarkably well on Costa Rican braconids. However, in an exceptional case, seven species of *Macrocentrus* fall into a single BIN (BOLD:ACK7466), and two species fall into another BIN (BOLD:ACK7467). The species in these two BINs differ morphologically and in host choice, and can be separated unambiguously into independent clades within their respective BINs. Since the seven species in BINBOLD:ACK7466 cannot be separated by BIN placement, a morphological key is provided in addition to their barcode and their morphological diagnoses. The two species in BINBOLD:ACK7467 are diagnosed morphologically in their respective treatments.

### Key to the species of *Macrocentrus* in BIN BOLD: ACK7466.

**Table:** 

1	A. Hind femur partly to entirely black	**2**
–	B. Hind femur entirely yellow to reddish brown	**4**
2(1)	A. Mesopleuron black below precoxal groove	*** M. gustavogutierrezi ***
–	B. Mesopleuron pale yellow to orange-brown below precoxal groove	**3**
3(2)	A. Face and gena predominantly black	*** M. geoffbarnardi ***
–	B. Face and gena predominantly yellow.	*** M. gregburtoni ***
4(1)	A. Mesopleuron black below precoxal groove	*** M. gretchendailyae ***
–	B. Mesopleuron pale yellow to light brown below precoxal groove.	**5**
5(4)	A. Propodeum and first metasomal tergum black	*** M. lucianocapelli ***
–	B. Propodeum and first metasomal tergum yellowish brown	**6**
6(5)	A. Hind femur reddish brown.	*** M. grettelvegae ***
–	B. Hind femur yellow	*** M. fredsingeri ***

#### Macrocentrus fredsingeri

Taxon classificationAnimaliaHymenopteraBraconidae

Sharkey & van Achterberg sp. nov.

http://zoobank.org/265D1EB3-32EB-467F-BFA1-BFCB7D6966C1

Figure 298

##### Diagnostics.

BOLD:ACK7466. Consensus barcode. YATATTRTATTTTTTRTTTGGTATRTGRTCTGGGGWRWTAGGTTTATCAYTAAGTTTAATTATTCGTATAGAATTRGGTCAAATTGGTTYATTTATTGGAAATGATCAAATTTAYAATAGTATTGTTACTTCTCATGCTTTTATTATAATTTTTTTTATAGTTATRCCTATTATAATTGGGGGKTTTGGHAATTGRTTRATTCCYTTAATATTAGGRAGTGTTGATATAGCTTTYCCTCGAATAAATAATATAAGATTTTGATTATTARTTCCTTCTTTAATATTATTAATTTTAAGWGGRTTTATAAATATTGGTGTAGGTACAGGATGAACAGTWTAYCCYCCTTTATCAYTAAATGTTAGTCATATAGGRATTTCTGTMGATATAGYTATTTTTTCAYTACATTTGGCTGGWATTTCTTCAATTATAGGKGCTATTAATTTTATTGTTACTATTATAAATATACGAAATTATGGGGTATTAATAGATAAAATTAGATTATTATYATGATCAATTTTAATTACRGCTATTTTATTATTRTTATCTTTACCTGTGTTAGCTGGTGCWATTACAATATTRTTAACTGAYCGTAATTTAAATACATCYTTTTTTGAYCCTGCTGGAGGAGGRGRYCCTATTTTATAYCAACATTTATTT. Recognizable by the combinations of largely setose subbasal cell of forewing, vein 1Cua of forewing ca. as long as vein cu-a and moderately widened, POL 0.7 × diameter of posterior ocellus, dorsal carinae of first tergite surpassing middle of tergite, first tergite with oblique striae latero-posteriorly, pterostigma mainly dark brown and contrasting with pale R1b vein and antenna largely pale yellowish.

##### Holotype ♀.

Alajuela, Sector Rincon Rain Forest, Conguera, 10.91589, -85.26631, 420 meters, caterpillar collection date: 17/i/2011, wasp eclosion date: 10/ii/2011. Depository: CNC.

***Host data*.***Neurophyseta* clymenalisDHJ03 (Crambidae) feeding on *Cyatheatrichiata* (Cyatheaceae).

***Caterpillar and holotype voucher codes*.** 11-SRNP-40293, DHJPAR0042876.

##### Paratypes.

Hosts = *Neurophyseta* clymenalisDHJ03. DHJPAR0042874, DHJPAR0042869, DHJPAR0042865, DHJPAR0041508, DHJPAR0042878, DHJPAR0043228, DHJPAR0041474, DHJPAR0041458, DHJPAR0041522, DHJPAR0041528, DHJPAR0042866, DHJPAR0042868, DHJPAR0042871, DHJPAR0041476, DHJPAR0041477, DHJPAR0041479, DHJPAR0041480, DHJPAR0041481, DHJPAR0041483, DHJPAR0041484, DHJPAR0041486, DHJPAR0041489, DHJPAR0041501, DHJPAR0041505, DHJPAR0051407. Depository: CNC.

##### Etymology.

*Macrocentrusfredsingeri* is named to honor Dr. Fred Singer for his valiant effort to document and describe the academic forces by DHJ and WH leading up to and founding of ACG.

**Figure 298.**
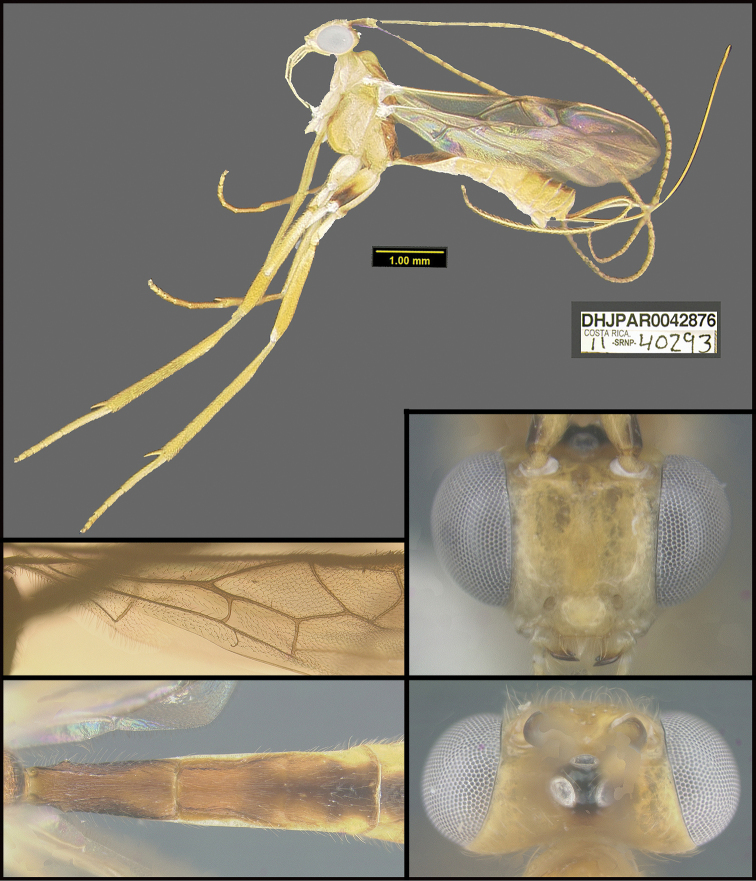
*Macrocentrusfredsingeri*, holotype.

#### Macrocentrus geoffbarnardi

Taxon classificationAnimaliaHymenopteraBraconidae

Sharkey & van Achterberg sp. nov.

http://zoobank.org/BDB6FB4F-5C49-4C3F-9690-08FC0700B3CD

Figure 299

##### Diagnostics.

BOLD:ACK7466. Consensus barcode. YATATTRTATTTTTTRTTTGGTATRTGRTCTGGGGWRWTAGGTTTATCAYTAAGTTTAATTATTCGTATAGAATTRGGTCAAATTGGTTYATTTATTGGAAATGATCAAATTTAYAATAGTATTGTTACTTCTCATGCTTTTATTATAATTTTTTTTATAGTTATRCCTATTATAATTGGGGGKTTTGGHAATTGRTTRATTCCYTTAATATTAGGRAGTGTTGATATAGCTTTYCCTCGAATAAATAATATAAGATTTTGATTATTARTTCCTTCTTTAATATTATTAATTTTAAGWGGRTTTATAAATATTGGTGTAGGTACAGGATGAACAGTWTAYCCYCCTTTATCAYTAAATGTTAGTCATATAGGRATTTCTGTMGATATAGYTATTTTTTCAYTACATTTGGCTGGWATTTCTTCAATTATAGGKGCTATTAATTTTATTGTTACTATTATAAATATACGAAATTATGGGGTATTAATAGATAAAATTAGATTATTATYATGATCAATTTTAATTACRGCTATTTTATTATTRTTATCTTTACCTGTGTTAGCTGGTGCWATTACAATATTRTTAACTGAYCGTAATTTAAATACATCYTTTTTTGAYCCTGCTGGAGGAGGRGRYCCTATTTTATAYCAACATTTATTT. Belongs to the group with vein M+Cu of forewing distinctly widened apically. Ventral half of mesopleuron and metapleuron brownish yellow; vein 1A of forewing narrow subapically. It differs from *M.gregburtoni* and *M.grettelvegae* by the strongly widened vein 1Cua of forewing (vs. approx. 5 × wider than vein 1Cub; approx. 3 × wider in both other species), and largely dark brown hind tibia, head, and first metasomal tergite (vs. mainly yellowish or brownish in both other species).

##### Holotype ♀.

Guanacaste, Sector Pitilla, Bullas, 10.98670, -85.38503, 440 meters, caterpillar collection date: 24/i/2013, wasp eclosion data: 19/ii/2013. Depository: CNC.

***Host data*.***Syllepismarialis* (Crambidae) feeding on *Allophyluspsilospermus* (Sapindaceae).

***Caterpillar and holotype voucher codes*.** 13-SRNP-70155, DHJPAR0050916.

##### Paratypes.

Host = *Syllepismarialis*: DHJPAR0035268, DHJPAR0038828, DHJPAR0028992, DHJPAR0028995, DHJPAR0039162, DHJPAR0048712, DHJPAR0028994, DHJPAR0038891, DHJPAR0040081, DHJPAR0049246. Depository: CNC.

##### Etymology.

*Macrocentrusgeoffbarnardi* is named to honor Mr. Geoff Barnard, formerly of The Nature Conservancy for his founding role in the planning and execution of the initiation of ACG.

**Figure 299.**
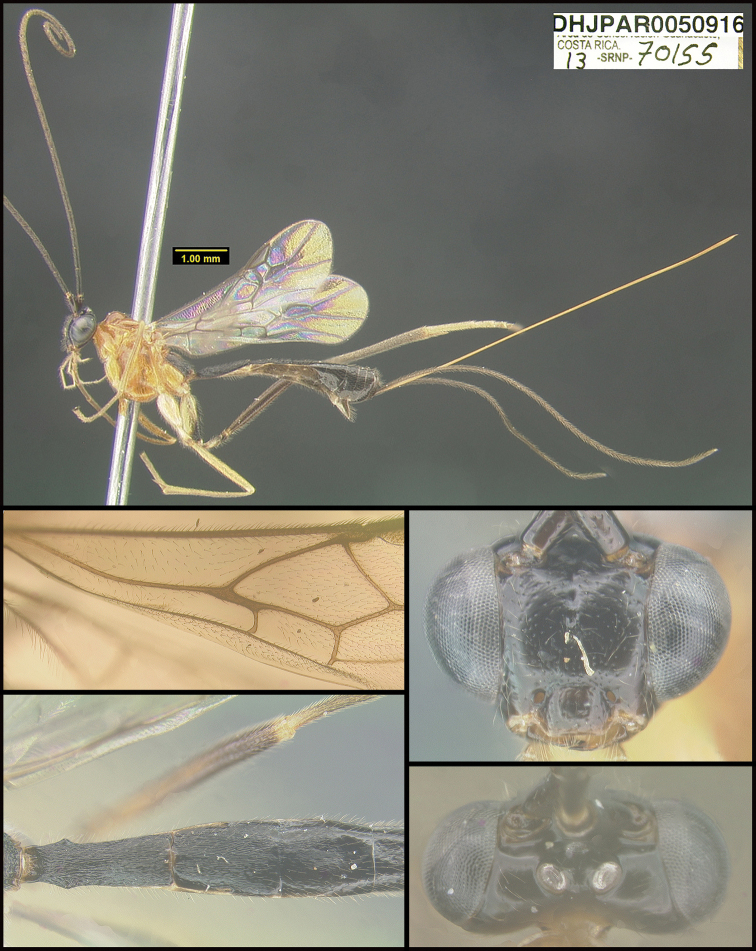
*Macrocentrusgeoffbarnardi*, holotype.

#### Macrocentrus gregburtoni

Taxon classificationAnimaliaHymenopteraBraconidae

Sharkey & van Achterberg sp. nov.

http://zoobank.org/AB1E2C3E-BD96-4A38-882F-4E748D166AE5

Figure 300

##### Diagnostics.

BOLD:ACK7466. Consensus barcode. YATATTRTATTTTTTRTTTGGTATRTGRTCTGGGGWRWTAGGTTTATCAYTAAGTTTAATTATTCGTATAGAATTRGGTCAAATTGGTTYATTTATTGGAAATGATCAAATTTAYAATAGTATTGTTACTTCTCATGCTTTTATTATAATTTTTTTTATAGTTATRCCTATTATAATTGGGGGKTTTGGHAATTGRTTRATTCCYTTAATATTAGGRAGTGTTGATATAGCTTTYCCTCGAATAAATAATATAAGATTTTGATTATTARTTCCTTCTTTAATATTATTAATTTTAAGWGGRTTTATAAATATTGGTGTAGGTACAGGATGAACAGTWTAYCCYCCTTTATCAYTAAATGTTAGTCATATAGGRATTTCTGTMGATATAGYTATTTTTTCAYTACATTTGGCTGGWATTTCTTCAATTATAGGKGCTATTAATTTTATTGTTACTATTATAAATATACGAAATTATGGGGTATTAATAGATAAAATTAGATTATTATYATGATCAATTTTAATTACRGCTATTTTATTATTRTTATCTTTACCTGTGTTAGCTGGTGCWATTACAATATTRTTAACTGAYCGTAATTTAAATACATCYTTTTTTGAYCCTGCTGGAGGAGGRGRYCCTATTTTATAYCAACATTTATTT. Belongs to the group with vein M+Cu of forewing distinctly widened apically. Vein 1Cua of forewing approx. 3 × wider than vein 1Cub, vein 1A of forewing narrow subapically and of metapleuron brownish yellow. Shares with *M.grettelvegae* the pale (yellowish brown or brownish yellow) hind tibia and pale yellow head (except for black stemmaticum); it differs by the largely dark brown pterostigma (yellowish brown in *M.grettelvegae*), rather robust second metasomal tergite (slenderer in *M.grettelvegae*), largely dark brown first tergite (pale brown in *M.grettelvegae*), and largely blackish dorsal face of mesosoma (mainly yellowish brown in *M.grettelvegae*).

##### Holotype ♀.

Guanacaste, Sector Pitilla, Estación Pitilla, 10.98931, -85.42581, 675 meters, caterpillar collection date: 5/iv/2010, wasp eclosion date: 21/iv/2010. Depository: CNC.

***Host data*.***Herpetogrammaphaeopteralis* (Crambidae) feeding on *Paspalumnutans* (Poaceae).

***Caterpillar and holotype voucher codes*.** 10-SRNP-30965, DHJPAR0039541.

##### Paratypes.

None.

##### Etymology.

*Macrocentrusgregburtoni* is named to honor Mr. Greg Burton of California for his support of ACG’s development of its inventory and biodiversity development.

**Figure 300.**
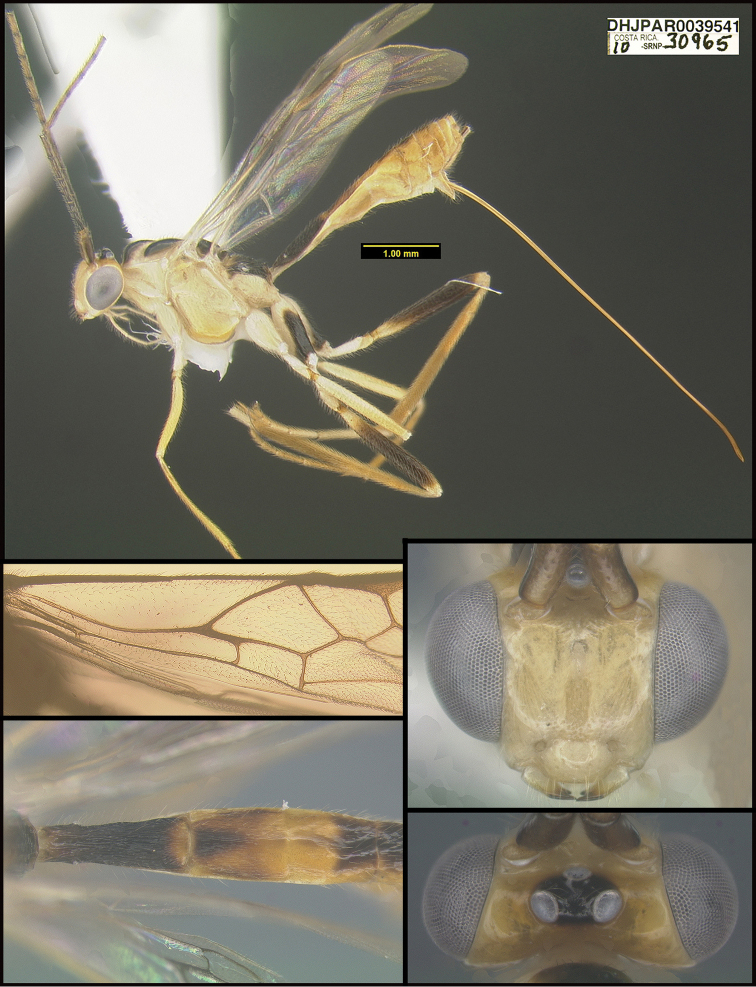
*Macrocentrusgregburtoni*, holotype.

#### Macrocentrus gretchendailyae

Taxon classificationAnimaliaHymenopteraBraconidae

Sharkey & van Achterberg sp. nov.

http://zoobank.org/7EBE7B1F-5215-49DA-91C2-427EECAD99B0

Figure 301

##### Diagnostics.

BOLD:ACK7466. Consensus barcode. YATATTRTATTTTTTRTTTGGTATRTGRTCTGGGGWRWTAGGTTTATCAYTAAGTTTAATTATTCGTATAGAATTRGGTCAAATTGGTTYATTTATTGGAAATGATCAAATTTAYAATAGTATTGTTACTTCTCATGCTTTTATTATAATTTTTTTTATAGTTATRCCTATTATAATTGGGGGKTTTGGHAATTGRTTRATTCCYTTAATATTAGGRAGTGTTGATATAGCTTTYCCTCGAATAAATAATATAAGATTTTGATTATTARTTCCTTCTTTAATATTATTAATTTTAAGWGGRTTTATAAATATTGGTGTAGGTACAGGATGAACAGTWTAYCCYCCTTTATCAYTAAATGTTAGTCATATAGGRATTTCTGTMGATATAGYTATTTTTTCAYTACATTTGGCTGGWATTTCTTCAATTATAGGKGCTATTAATTTTATTGTTACTATTATAAATATACGAAATTATGGGGTATTAATAGATAAAATTAGATTATTATYATGATCAATTTTAATTACRGCTATTTTATTATTRTTATCTTTACCTGTGTTAGCTGGTGCWATTACAATATTRTTAACTGAYCGTAATTTAAATACATCYTTTTTTGAYCCTGCTGGAGGAGGRGRYCCTATTTTATAYCAACATTTATTT. Belongs to the group with vein M+Cu of forewing distinctly widened apically. Ventral half of mesopleuron and part of metapleuron largely black and first metasomal tergite subparallel-sided. Differs from *M.jesseausubeli*, *M.gustavogutierrezi*, and *M.jennyphillipsae* by having the hind femur pale brown (black in other species) and the first metasomal tergite yellowish brown.

##### Holotype ♀.

Alajuela, Sector Rincon Rain Forest, San Lucas, 10.91846, -85.30338, 320 meters, caterpillar collection date: 8/i/2014, wasp eclosion date: 3/ii/2014. Depository: CNC.

***Host data*.***Piletosomathialis* (Crambidae) feeding on *Doliocarpusmultiflorus* (Dilleniaceae).

***Caterpillar and holotype voucher codes*.** 14-SRNP-40250, DHJPAR0054411.

##### Paratypes.

Host = *Piletosomathialis*: DHJPAR0017227, DHJPAR0035152, DHJPAR0035306, DHJPAR0035305, DHJPAR0040082, DHJPAR0035262, DHJPAR0035264, DHJPAR0035269, DHJPAR0035151, DHJPAR0038018, DHJPAR0063008, DHJPAR0063009, DHJPAR0063035. Depository: CNC.

##### Etymology.

*Macrocentrusgretchendailyae* is named to honor Dr. Gretchen Daily of Stanford University for her decades of intense interest in the bioeconomics of integrating society with wild nature.

**Figure 301.**
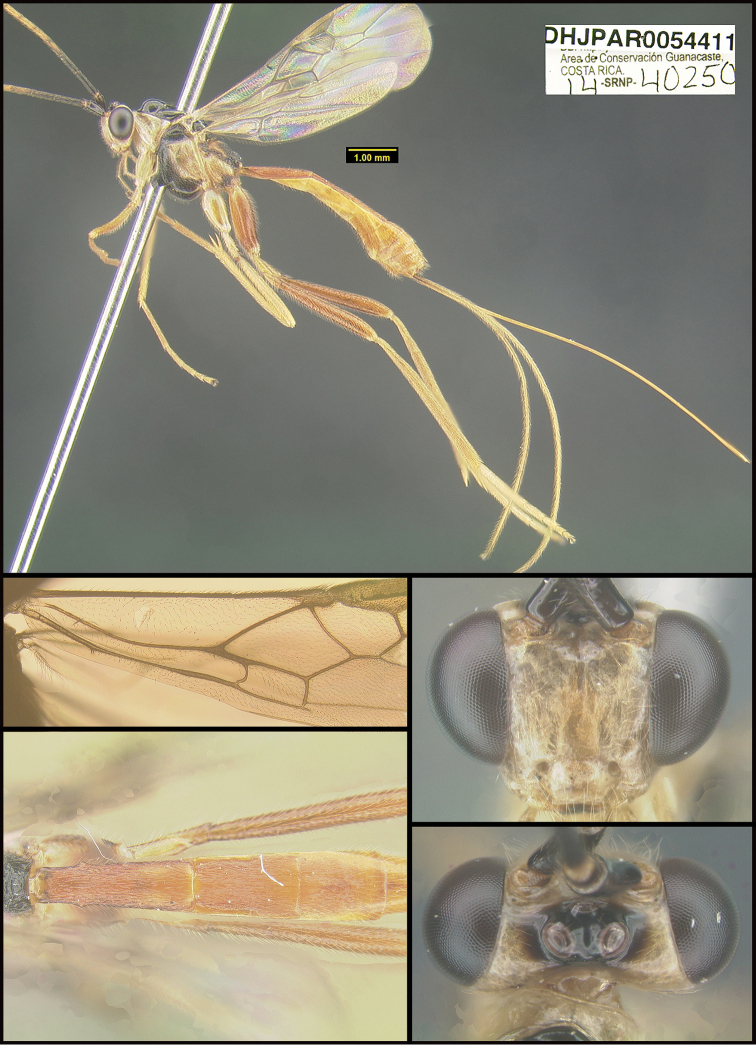
*Macrocentrusgretchendailyae*, holotype.

#### Macrocentrus grettelvegae

Taxon classificationAnimaliaHymenopteraBraconidae

Sharkey & van Achterberg sp. nov.

http://zoobank.org/B3C7DF5C-F7A7-4098-9099-71B1B37BA51D

Figure 302

##### Diagnostics.

BOLD:ACK7466. Consensus barcode. YATATTRTATTTTTTRTTTGGTATRTGRTCTGGGGWRWTAGGTTTATCAYTAAGTTTAATTATTCGTATAGAATTRGGTCAAATTGGTTYATTTATTGGAAATGATCAAATTTAYAATAGTATTGTTACTTCTCATGCTTTTATTATAATTTTTTTTATAGTTATRCCTATTATAATTGGGGGKTTTGGHAATTGRTTRATTCCYTTAATATTAGGRAGTGTTGATATAGCTTTYCCTCGAATAAATAATATAAGATTTTGATTATTARTTCCTTCTTTAATATTATTAATTTTAAGWGGRTTTATAAATATTGGTGTAGGTACAGGATGAACAGTWTAYCCYCCTTTATCAYTAAATGTTAGTCATATAGGRATTTCTGTMGATATAGYTATTTTTTCAYTACATTTGGCTGGWATTTCTTCAATTATAGGKGCTATTAATTTTATTGTTACTATTATAAATATACGAAATTATGGGGTATTAATAGATAAAATTAGATTATTATYATGATCAATTTTAATTACRGCTATTTTATTATTRTTATCTTTACCTGTGTTAGCTGGTGCWATTACAATATTRTTAACTGAYCGTAATTTAAATACATCYTTTTTTGAYCCTGCTGGAGGAGGRGRYCCTATTTTATAYCAACATTTATTT. Belongs to the group with vein M+Cu of forewing distinctly widened apically. Vein 1Cua of forewing approx. 3 × wider than vein 1Cub. Ventral half of mesopleuron and metapleuron brownish yellow. Vein 1A of forewing narrow subapically and of metapleuron brownish yellow. Shares with *M.gregburtoni* the pale hind tibia and pale yellow head (except for blackish stemmaticum and its surroundings); it differs by the yellowish brown pterostigma (largely dark brown in *M.gregburtoni*), comparatively slender second metasomal tergite (rather robust in *M.gregburtoni*), pale brown first tergite (largely dark brown in *M.gregburtoni*), and mainly yellowish brown dorsal face of mesosoma (largely blackish in *M.gregburtoni*).

##### Holotype ♀.

Guanacaste, Sector San Cristobal, Tajo Angeles, 10.86472, -85.41531, 540 meters, caterpillar collection date: 11/vi/2011, wasp eclosion date: 3/vii/2011. Depository: CNC.

***Host data*.***Desmiaploralis* (Crambidae) feeding on *Hameliapatens* (Rubiaceae).

***Caterpillar and holotype voucher codes*.** 11-SRNP-2291, DHJPAR0045379.

##### Paratypes.

Host = *Desmia* ploralisDHJ10 and *Desmia* benealisDHJ03: DHJPAR0040350, DHJPAR0045377. Depository: CNC.

##### Etymology.

*Macrocentrusgrettelvegae* is named to honor Sra. Grettel Vega of SINAC for her support of BioAlfa as Director of SINAC, Costa Rica’s National System of Conservation Areas.

**Figure 302.**
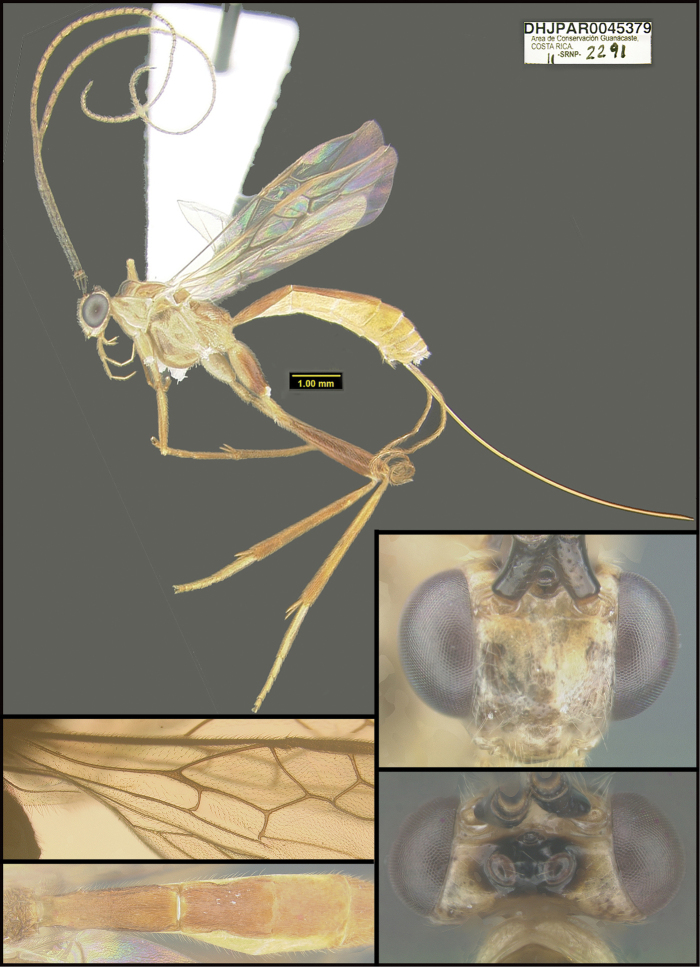
*Macrocentrusgrettelvegae*, holotype.

#### Macrocentrus gustavogutierrezi

Taxon classificationAnimaliaHymenopteraBraconidae

Sharkey & van Achterberg sp. nov.

http://zoobank.org/C9D185E7-12BE-4E8D-AA93-E3F4C36676F8

Figure 303

##### Diagnostics.

BOLD:ACK7466. Consensus barcode. YATATTRTATTTTTTRTTTGGTATRTGRTCTGGGGWRWTAGGTTTATCAYTAAGTTTAATTATTCGTATAGAATTRGGTCAAATTGGTTYATTTATTGGAAATGATCAAATTTAYAATAGTATTGTTACTTCTCATGCTTTTATTATAATTTTTTTTATAGTTATRCCTATTATAATTGGGGGKTTTGGHAATTGRTTRATTCCYTTAATATTAGGRAGTGTTGATATAGCTTTYCCTCGAATAAATAATATAAGATTTTGATTATTARTTCCTTCTTTAATATTATTAATTTTAAGWGGRTTTATAAATATTGGTGTAGGTACAGGATGAACAGTWTAYCCYCCTTTATCAYTAAATGTTAGTCATATAGGRATTTCTGTMGATATAGYTATTTTTTCAYTACATTTGGCTGGWATTTCTTCAATTATAGGKGCTATTAATTTTATTGTTACTATTATAAATATACGAAATTATGGGGTATTAATAGATAAAATTAGATTATTATYATGATCAATTTTAATTACRGCTATTTTATTATTRTTATCTTTACCTGTGTTAGCTGGTGCWATTACAATATTRTTAACTGAYCGTAATTTAAATACATCYTTTTTTGAYCCTGCTGGAGGAGGRGRYCCTATTTTATAYCAACATTTATTT. Belongs to the group with vein M+Cu of forewing distinctly widened apically. Ventral halves of mesopleuron and metapleuron largely black; first metasomal tergite subparallel-sided. Most similar to *M.jennyphillipsae* because of the largely black hind femur and first metasomal tergite but differs by having large ocelli (POL approx. 0.8 × diameter of posterior ocellus; approx. 1.5 × in *M.jennyphillipsae*) and face pale yellow medio-dorsally (partly dark brown in *M.jennyphillipsae*).

##### Holotype ♀.

Guanacaste, Sector Del Oro, Bosque Aguirre, 11.00060, -85.43800, 620 meters, caterpillar collection date: 3/ii/2010, wasp eclosion date: 17/ii/2010. Depository: CNC.

***Host data*.***Asturodes* fimbriauralisDHJ02 (Crambidae) feeding on *Colubrinaspinosa* (Rhamnaceae).

***Caterpillar and holotype voucher codes*.** 10-SRNP-20281, DHJPAR0038832.

##### Paratypes.

Hosts = *Asturodes* fimbriauralisDHJ02 and *Antaeotrichaspurca* (Depressariidae). DHJPAR0029383, DHJPAR0050856, DHJPAR0055165, DHJPAR0028986, DHJPAR0029341, DHJPAR0036261, DHJPAR0029386, DHJPAR0029389, DHJPAR0029360, DHJPAR0053557, DHJPAR0050803, DHJPAR0038831, DHJPAR0037707, DHJPAR0028998, DHJPAR0028997, DHJPAR0029393, DHJPAR0029392, DHJPAR0029391, DHJPAR0029390, DHJPAR0029388, DHJPAR0028981, DHJPAR0029076, DHJPAR0029349, DHJPAR0029379, DHJPAR0029384, DHJPAR0029339, DHJPAR0029340, DHJPAR0029338, DHJPAR0036731, DHJPAR0029380. Depository: CNC.

##### Etymology.

*Macrocentrusgustavogutierrezi* is named to honor Sr. Gustavo Gutierrez for his thousands of hectares donated to ACG and financial support of the ACG Biological Education Program.

**Figure 303.**
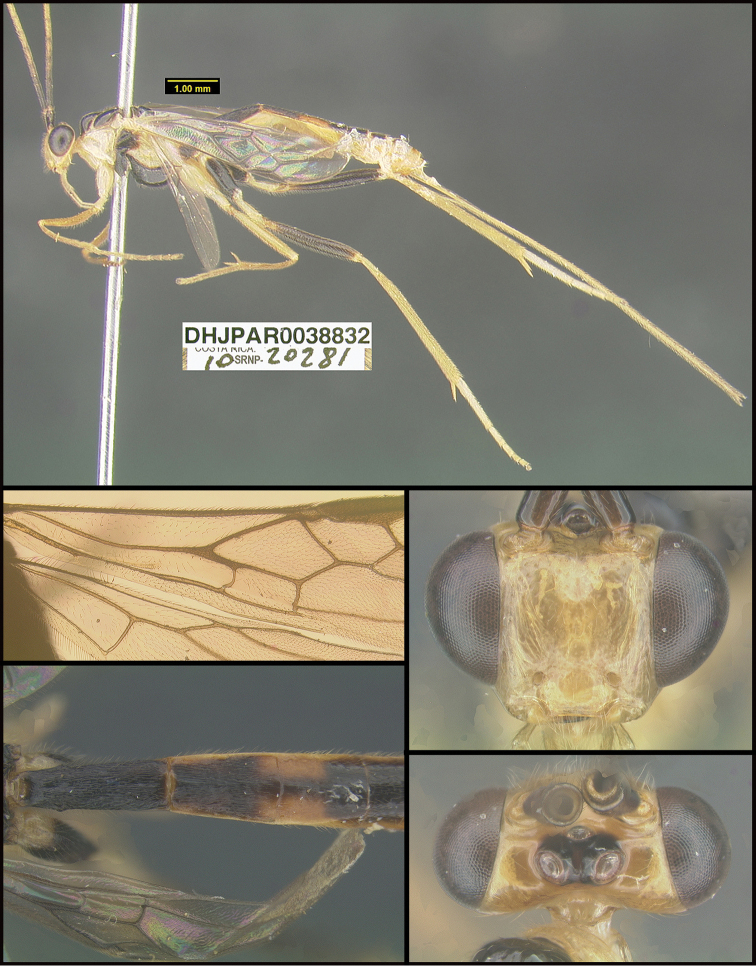
*Macrocentrusgustavogutierrezi*, holotype.

#### Macrocentrus hannahjamesae

Taxon classificationAnimaliaHymenopteraBraconidae

Sharkey & van Achterberg sp. nov.

http://zoobank.org/A31C451D-98C3-4A0B-917F-FEDDC9893B69

Figure 304

##### Diagnostics.

BOLD:ABA9321. Consensus barcode. TGTATTGTATTTTTTATTTGGTATATGATCCGGGGTATTGGGCTTGTCATTAAGTTTAATTATTCGTATAGAATTAGGTCAAATTGGTTCATTTATTGGAAATGATCAAATTTATAATAGTATTGTTACTTCACATGCTTTTATTATAATTTTTTTTATAGTTATACCTATTATAATCGGGGGATTTGGAAATTGATTAATTCCTTTAATATTAGGGAGTGTTGATATAGCTTTCCCTCGAATAAATAACATAAGATTTTGATTATTAATTCCTTCTTTAATATTATTAATTTTAAGAGGATTTATAAATATTGGTGTAGGTACAGGATGAACAGTTTACCCCCCTTTATCATTGAATATTAGTCATATAGGAATTTCTGTAGATATAGCTATTTTTTCATTACATTTGGCTGGGGTTTCTTCAATTATAGGTGCTATTAATTTTATCATTACTATTATAAATATACGAAATTATGGGGTATTAATAGATAAAATTAGATTATTATCATGATCAATTTTAATTACAGCTATTTTATTATTATTATCTTTACCTGTGTTAGCTGGTGCTATTACAATATTGTTAACTGATCGTAATTTAAATACATCTTTTTTTGATCCTGCTGGAGGGGGGGATCCTATTTTATATCAACATTTATTT. Belongs to the group with vein M+Cu of forewing distinctly widened apically, and mesosternum, ventral half of mesopleuron and metapleuron brownish yellow. It shares with *M.lucianocapelli* the somewhat widened subapical part of vein 1A of forewing, but differs by the posteriorly parallel-sided first tergite (distinctly widened in *M.lucianocapelli*), face with dark brown heart-shaped patch (entirely yellowish brown in *M.lucianocapelli*), and hind tibia largely dark brown (brownish yellow in *M.lucianocapelli*).

##### Holotype ♀.

Alajuela, Sector San Cristobal, Finca San Gabriel, 10.87766, -85.39343, 645 meters, caterpillar collection date: 14/vii/2011, wasp eclosion date: 7/viii/2011. Depository: CNC.

***Host data*.***Syllepismarialis* (Crambidae) feeding on *Allophyluspsilospermus* (Sapindaceae).

***Caterpillar and holotype voucher codes*.** 11-SRNP-2806, DHJPAR0045376.

##### Paratypes.

Host = *Syllepismarialis*: DHJPAR0028984, DHJPAR0036307, DHJPAR0036308, DHJPAR0045386. Depository: CNC.

##### Etymology.

*Macrocentrushannahjamesae* is named to honor Miss Hannah James for her intense effort to mold the publicity outreach for the Centre for Biodiversity Genomics at the University of Guelph, Canada.

**Figure 304.**
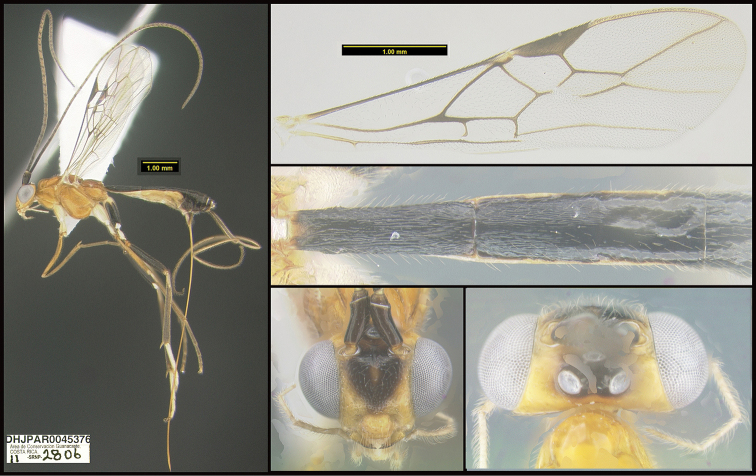
*Macrocentrushannahjamesae*, holotype.

#### Macrocentrus harisridhari

Taxon classificationAnimaliaHymenopteraBraconidae

Sharkey & van Achterberg sp. nov.

http://zoobank.org/27944FF6-D485-4D92-8A04-0813E4B866D2

Figure 305

##### Diagnostics.

BOLD:ACK7467. Consensus barcode. TATATTGTATTTTTTWWTTGGYATRTGRTCRGGAGTATTAGGTTTATCATTAAGTTTAATTATTCGTATAGAATTAGGTCAAATTGGKTCATTTATTGGAAATGATCAGATTTATAATAGTATYGTTACTTCTCATGCTTTYATTATAATTTTTTTTATAGTTATACCTATTATAATTGGKGGATTTGGMAATTGATTAATTCCTTTRATRTTRGGAAGTGTWGATATRGCYTTYCCTCGAATAAATAATATAAGATTTTGATTATTAATTCCTTCTTTAATATTATTAATTTTAAGAGGWTTTATAAATATTGGTGTAGGTACAGGATGAACAGTTTATCCYCCYTTATCATTAAATATTAGTCATATAGGAATTTCTGTAGATATAGCTATTTTTTCATTACATTTGGCGGGTATTTCTTCAATTATAGGTGCTATTAATTTTATTGTTACTATTATAAATATACGAAATTATGGGGTATTAATAGATAAAATTAGATTATTATCATGATCAATTTTAATCACAGCTATTTTATTATTATTATCTTTACCTGTRTTAGCTGGTGCWATTACAATRTTGTTAACTGATCGTAATTTAAATACATCTTTYTTTGACCCYGCYGGAGGGGGTGACCCTRTTTTATAYCAACATTTATTT. There are two species in this BIN (*M.harisridhari* and *M.hiroshikidonoi*) that differ by almost 2%, and both share having the metasoma dark brown medio-dorsally, vein 1Cua of forewing distinctly longer than vein cu-a, R1b paler than pterostigma, and vein cu-a curved and posteriorly widened. Their barcodes form two groups with no intermediates. The two species also differ in color: *M.harisridhari* is darker than *M.hiroshikidonoi* i.e., *M.harisridhari* is dark brown where *M.hiroshikidonoi* is light reddish brown, and brownish yellow where *M.hiroshikidonoi* is light yellow. Morphologically, both are very similar, but *M.harisridhari* differs by having the first metasomal tergite slightly widened posteriorly and slightly shorter POL (0.7 × diameter of posterior ocellus vs. 0.9 × in *M.hiroshikidonoi*.

##### Holotype ♀.

Alajuela, Sector Rincon Rain Forest, Flecha, 10.94741, -85.31501, 491 meters, caterpillar collection date: 21/i/2013, wasp eclosion date: 6/ii/2013. Depository: CNC.

***Host data*.***Undulambia* Solis02 (Crambidae) feeding on *Cyatheamultiflora* (Cyatheaceae).

***Caterpillar and holotype voucher codes*.** 13-SRNP-69137, DHJPAR0051314.

##### Paratypes.

Hosts = *Undulambia* Solis02, *Neurophyseta* camptogrammalisDHJ01 (Pyralidae). DHJPAR0045384, DHJPAR0043243, DHJPAR0041469, DHJPAR0050021, DHJPAR0051386. Depository: CNC.

##### Etymology.

*Macrocentrusharisridhari* is named to honor Dr. Hari Sridhari of the Indian Institute of Science, Bangalore, India for his efforts to popularize and explain, as a science writer, the processes behind some of DHJ’s papers.

**Figure 305.**
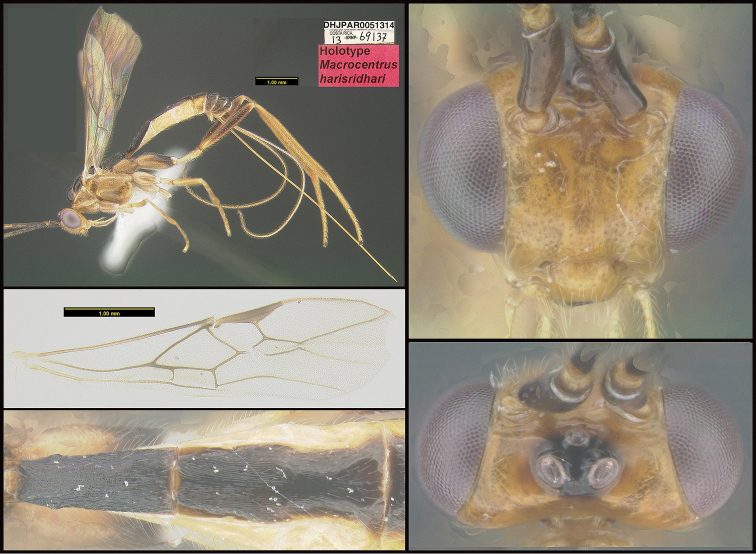
*Macrocentrusharisridhari*, holotype.

#### Macrocentrus hillaryrosnerae

Taxon classificationAnimaliaHymenopteraBraconidae

Sharkey & van Achterberg sp. nov.

http://zoobank.org/52AEF694-054E-4865-AEE9-6D5E8C1403B2

Figure 306

##### Diagnostics.

BOLD:ABA7284. Consensus barcode. TATATTGTATTTTTTATTTGGTATATGATCGGGAGTATTAGGTTTATCATTAAGTTTAATTATTCGTATAGAATTAGGCCAAATTGGTTCATTTATTGGAAATGATCAGATTTATAATAGTATTGTTACTTCTCATGCTTTCATTATAATTTTTTTTATAGTTATGCCTATTATAATTGGAGGATTTGGAAATTGATTGATTCCTTTAATGTTAGGAAGTGTTGATATAGCTTTCCCTCGAATAAATAATATAAGATTTTGATTATTAATTCCTTCTTTAATATTATTAATTTTAAGAGGGTTTGTAAATATTGGTGTAGGTACAGGATGAACGGTCTATCCTCCTTTATCATTAAATATTAGTCATATAGGAATTTCTGTAGATATCGCTATTTTTTCATTACATTTGGCGGGTATTTCTTCAATTATAGGTGCTATTAACTTTATTGTTACTATTATAAATATACGAAATTATGGGGTATTAATAGATAAAATTAGATTATTATCATGATCAATTTTAATTACAGCTATTTTATTATTATTATCTTTACCTGTGTTAGCTGGTGCTATTACAATATTGTTAACTGATCGTAATTTAAACACATCTTTTTTTGATCCTGCCGGAGGGGGTGATCCTATTTTATATCAGCATTTATTT. Recognizable by the wide elliptical bicolored pterostigma and elongate (approx. twice as long as vein cu-a) moderately widened vein 1Cua.

##### Holotype ♂.

Alajuela, Sector Rincon Rain Forest, Camino Porvenir, 10.90383, -85.25964, 383 meters, caterpillar collection date: 12/iii/2011, wasp eclosion date: 30/iii/2011. Depository: CNC.

***Host data*.***Undulambia* Solis02 (Crambidae) feeding on *Alsophilafirma* (Cyatheaceae).

***Caterpillar and holotype voucher codes*.** 11-SRNP-41204, DHJPAR0043202.

##### Paratypes.

None.

##### Etymology.

*Macrocentrushillaryrosnerae* is named to honor Ms. Hillary Rosner a science journalist who did a magnificent job of explaining why mountain passes are higher in the tropics and relating that to climate change.

**Figure 306.**
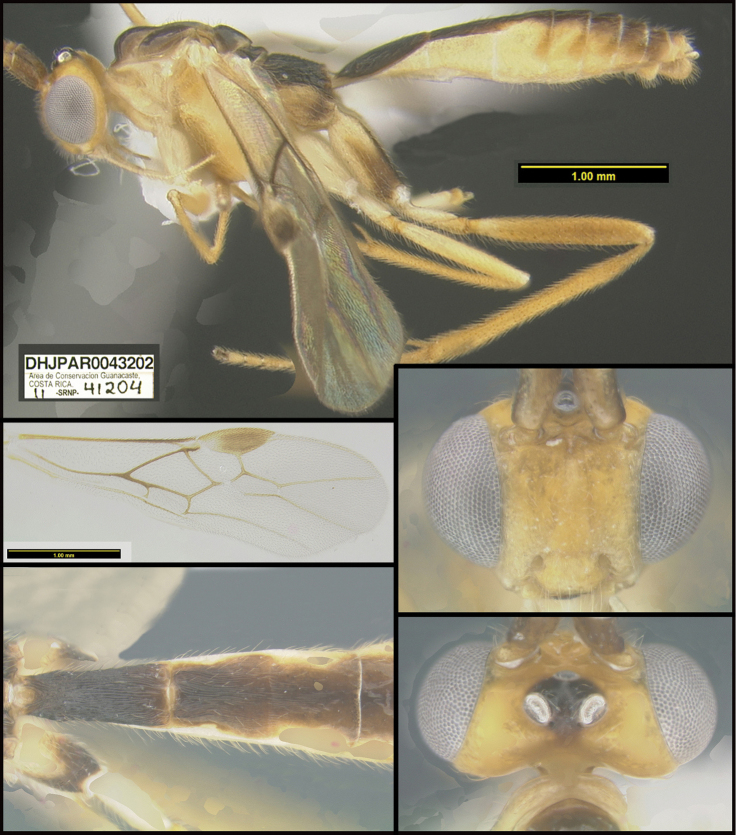
*Macrocentrushillaryrosnerae*, holotype.

#### Macrocentrus hiroshikidonoi

Taxon classificationAnimaliaHymenopteraBraconidae

Sharkey & van Achterberg sp. nov.

http://zoobank.org/F46934AC-8E57-4ED2-BD5C-4A4205932C5C

Figure 307

##### Diagnostics.

BOLD:ACK7467. Consensus barcode. TATATTGTATTTTTTWWTTGGYATRTGRTCRGGAGTATTAGGTTTATCATTAAGTTTAATTATTCGTATAGAATTAGGTCAAATTGGKTCATTTATTGGAAATGATCAGATTTATAATAGTATYGTTACTTCTCATGCTTTYATTATAATTTTTTTTATAGTTATACCTATTATAATTGGKGGATTTGGMAATTGATTAATTCCTTTRATRTTRGGAAGTGTWGATATRGCYTTYCCTCGAATAAATAATATAAGATTTTGATTATTAATTCCTTCTTTAATATTATTAATTTTAAGAGGWTTTATAAATATTGGTGTAGGTACAGGATGAACAGTTTATCCYCCYTTATCATTAAATATTAGTCATATAGGAATTTCTGTAGATATAGCTATTTTTTCATTACATTTGGCGGGTATTTCTTCAATTATAGGTGCTATTAATTTTATTGTTACTATTATAAATATACGAAATTATGGGGTATTAATAGATAAAATTAGATTATTATCATGATCAATTTTAATCACAGCTATTTTATTATTATTATCTTTACCTGTRTTAGCTGGTGCWATTACAATRTTGTTAACTGATCGTAATTTAAATACATCTTTYTTTGACCCYGCYGGAGGGGGTGACCCTRTTTTATAYCAACATTTATTT. There are two species in this BIN (*M.harisridhari* and *M.hiroshikidonoi*) that differ by almost 2%; both share having the metasomal dark brown medio-dorsally, vein 1Cua of forewing distinctly longer than vein cu-a, and vein cu-a curved and posteriorly widened. Their barcodes form two groups with no intermediates (Suppl. material 7), as in many other orders in the ACG inventory. The two species differ in color: *M.harisridhari* is dark brown, whereas *M.hiroshikidonoi* is light reddish brown; and the brownish yellow areas of *M.harisridhari* are light yellow in *M.hiroshikidonoi*. Morphologically, *M.hiroshikidonoi* differs by having the first metasomal tergite subparallel-sided and slightly longer POL (0.9 × diameter of posterior ocellus vs. 0.7 × in *M.harisridhari*.

##### Holotype ♀.

Alajuela, Sector Rincon Rain Forest, Quebrada Guarumo, 10.90445, -85.28412, 400 meters, caterpillar collection date: 22/xi/2012, wasp eclosion date: 14/xii/2012. Depository: CNC.

***Host data*.***Undulambia* Solis02 (Crambidae) feeding on *Alsophilafirma* (Cyatheaceae).

***Caterpillar and holotype voucher codes*.** 12-SRNP-86847, DHJPAR0051422.

##### Paratypes.

Hosts = *Neurophyseta* clymenalisDHJ03, *Neurophyseta* Janzen229, *Diacme* BioLep02, *Neurophyseta* Janzen222, *Undulambia* Solis02, *Neurophysetacompletalis*, *Neurophyseta* clymenalisDHJ03, *Neurophyseta* camptogrammalisDHJ01 (all Crambidae), *Paramorbia* Brown001 (Tortricidae). DHJPAR0050022, DHJPAR0051418, DHJPAR0051318, DHJPAR0051320, DHJPAR0051322, DHJPAR0052193, DHJPAR0052327, DHJPAR0052380, DHJPAR0053148, DHJPAR0053667, DHJPAR0053668, DHJPAR0053669, DHJPAR0054926, DHJPAR0055590, DHJPAR0028990, DHJPAR0023710, DHJPAR0038403, DHJPAR0038466, DHJPAR0038021, DHJPAR0041399, DHJPAR0041406, DHJPAR0041469, DHJPAR0041490, DHJPAR0041495, DHJPAR0041498, DHJPAR0041499, DHJPAR0041504, DHJPAR0041509, DHJPAR0041512, DHJPAR0041519, DHJPAR0041544, DHJPAR0042870, DHJPAR0043239, DHJPAR0043243, DHJPAR0043250, DHJPAR0045381, DHJPAR0045382, DHJPAR0045384, DHJPAR0045503, DHJPAR0048239, DHJPAR0050011, DHJPAR0050012, DHJPAR0050013, DHJPAR0050014, DHJPAR0050017, DHJPAR0050016, DHJPAR0050017, DHJPAR0050018, DHJPAR0050020, DHJPAR0028989, DHJPAR0041459, DHJPAR0041462, DHJPAR0041465, DHJPAR0041397. Depository: CNC.

##### Etymology.

*Macrocentrushiroshikidonoi* is named to honor Dr. Hiroshi Kidono of Japan, formerly of JICA, for his very direct and enthusiastic support for ACG’s Parataxonomist Program and INBio, and personally to DHJ and WH in the germination and growth of ACG and INBio.

**Figure 307.**
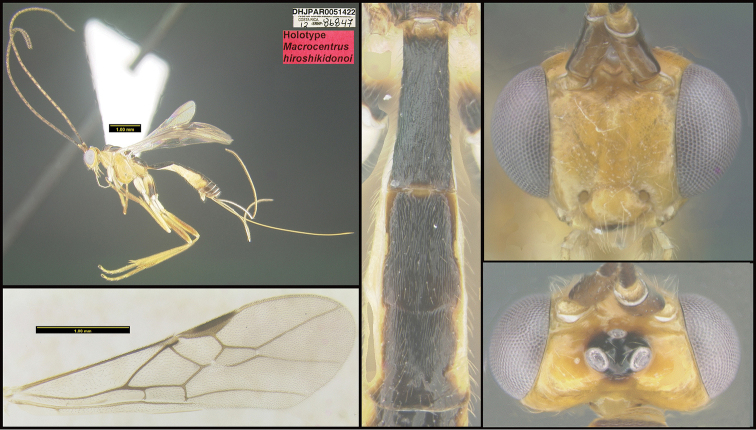
*Macrocentrushiroshikidonoi*, holotype.

#### Macrocentrus iangauldi

Taxon classificationAnimaliaHymenopteraBraconidae

Sharkey & van Achterberg sp. nov.

http://zoobank.org/41707FA4-3765-461B-B8B5-E8914AB0037D

Figure 308

##### Diagnostics.

BOLD:ABY7812. Consensus barcode. TATATTGTATTTTTTATTTGGTGTATGATCTGGGGTATTAGGTTTGTCACTAAGTTTAATTATTCGTATAGAATTAGGTCAAATTGGTTCATTTATTGGAAATGATCAAATTTATAATAGTATTGTTACTTYTCATGCTTTTATTATAATTTTTTTTATAGTTATACCTATTATAATTGGGGGGTTTGGAAATTGATTAATTCCTTTAATATTAGGAAGTGTTGATATAGCTTTTCCTCGAATAAATAATATAAGATTTTGATTATTAATTCCTTCTTTAATATTATTAATTTTAAGAGGATTTATAAATATTGGTGTAGGTACAGGATGAACAGTTTATCCTCCTTTATCATTAAATATTAGTCATATAGGAATTTCTGTAGATATATCTATTTTTTCATTACACTTGGCTGGTATTTCTTCAATTATAGGTGCTATTAATTTTATTGTTACTATTATAAATATACGAAATTATGGGGTATTAATAGATAAAATTAGATTATTATCATGATCKATTTTAATTACAGCTATTTTATTATTATTATCTTTACCTGTTTTAGCTGGTGCTATTACAATATTGTTAACTGATCGTAATTTAAATACATCTTTTTTTGATCCTGCCGGAGGGGGGGATCCTATTTTATACCAACATTTATTT. Easily recognizable by the pale yellowish R1 vein contrasting with mainly dark brown veins of the infuscated middle third of the forewing. In addition, is the first metasomal tergite robust, approx. 2.3 × longer than its apical width and vein 1Cua slightly widened and slightly longer than vein cu-a.

##### Holotype ♀.

Alajuela, Brasilia, Piedrona, 11.01618, -85.35902, 340 meters, caterpillar collection date: 26/x/2011, wasp eclosion date: 10/xi/2011. Depository: CNC.

***Host data*.***Antaeotricha* Janzen290 (Depressariidae) feeding on *Ingaoerstediana* (Fabaceae).

***Caterpillar and holotype voucher codes*.** 11-SRNP-66121, DHJPAR0046828.

##### Paratypes.

Hosts = Janzen433, *Antaeotrichamarmorea*, *Antaeotricha* Janzen31, *Antaeotricha* Janzen77 (Depressariidae). DHJPAR0045869, DHJPAR0045871, DHJPAR0045874, DHJPAR0046846, DHJPAR0048262, DHJPAR0048274. Depository: CNC.

##### Etymology.

*Macrocentrusiangauldi* is named to honor Dr. Ian Gauld (RIP) for his decades of highly enthusiastic support for all things taxonomic and bioinventory of Costa Rica in general and ACG specifically, with special emphasis on wasps in the family Ichneumonidae.

**Figure 308.**
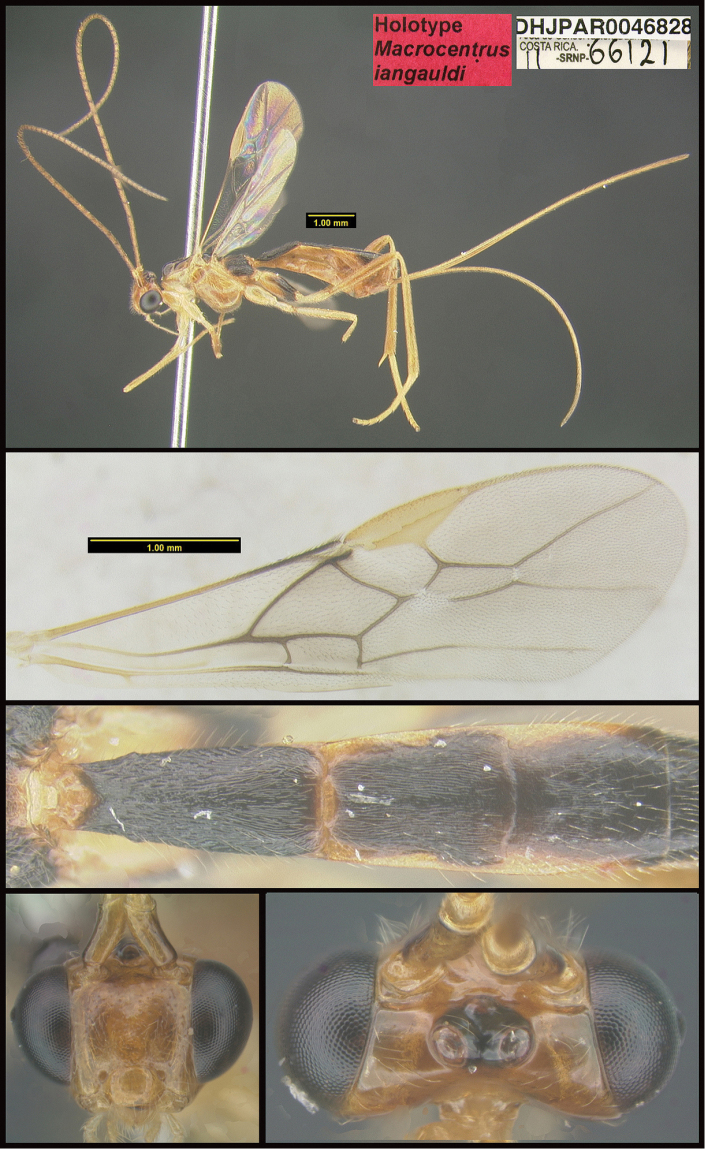
*Macrocentrusiangauldi*, holotype.

#### Macrocentrus jennyphillipsae

Taxon classificationAnimaliaHymenopteraBraconidae

Sharkey & van Achterberg sp. nov.

http://zoobank.org/8ACB9EA9-BD06-4797-8CC6-4E4A7A9C800C

Figure 309

##### Diagnostics.

BOLD:ADB4234. Consensus barcode. ATATTGTATTTTTTATTTGGTATATGATCTGGTATATTAGGCTTATCATTAAGTTTAATTATTCGAATAGAATTAGGTCAAATTGGTTCATTTATTGGAAATGATCAAATTTATAATAGTATCGTTACTTCTCATGCTTTTATTATAATTTTTTTTATAGTTATACCTATTATAATTGGGGGGTTTGGGAATTGATTAATCCCCCTAATATTAGGAAGTGTTGATATGGCTTTTCCTCGGATAAATAATATAAGATTTTGATTATTAATTCCTTCTTTAATATTATTAATTTTAAGAAGATTTATAAATATTGGTGTAGGGACAGGATGGACAGTTTATCCACCTTTATCAATAAATATTAGTCATATAGGTATTTCTGTAGATATGGCTATTTTTTCTTTACATTTAGCTGGTATTTCTTCAATTATGGGGGCTATTAATTTTATTGTTACTATTATAAATATACGAAATTATGGTGTATTAATAGATAAAATTAGATTATTATCATGATCAATTTTAATTACAGCTATTTTATTGTTATTATCTTTACCTGTATTAGCTGGTGCTATTACAATATTATTGACTGAT------------------------------------------------. Belongs to the group with vein M+Cu of forewing distinctly widened apically, mesosternum, ventral half of mesopleuron and of metapleuron largely black and first metasomal tergite subparallel-sided. Most similar to *M.gustavogutierrezi* because of the largely black hind femur and first metasomal tergite but differs by having comparatively small ocelli (POL approx. 1.5 × diameter of posterior ocellus; approx. 0.8 × in *M.gustavogutierrezi*) and face partly dark brown medio-dorsally (pale yellow in *M.gustavogutierrezi*).

##### Holotype ♀.

Guanacaste, Pailas Dos, PL12-3, 10.7631, -85.3344, 820 meters, 19/x/2013, Malaise trap. Depository: CNC.

***Host data*.** None.

***Holotype voucher code*.**BIOUG29288-B09.

##### Paratypes.

None.

##### Etymology.

*Macrocentrusjennyphillipsae* is named to honor Dr. Jenny Phillips of BioAlfa for her decades of enthusiastic taxonomic and administrative support of INBio, parataxonomists, and all matters biodiversity of Costa Rica coupled with intensely developing BioAlfa of Costa Rica.

**Figure 309.**
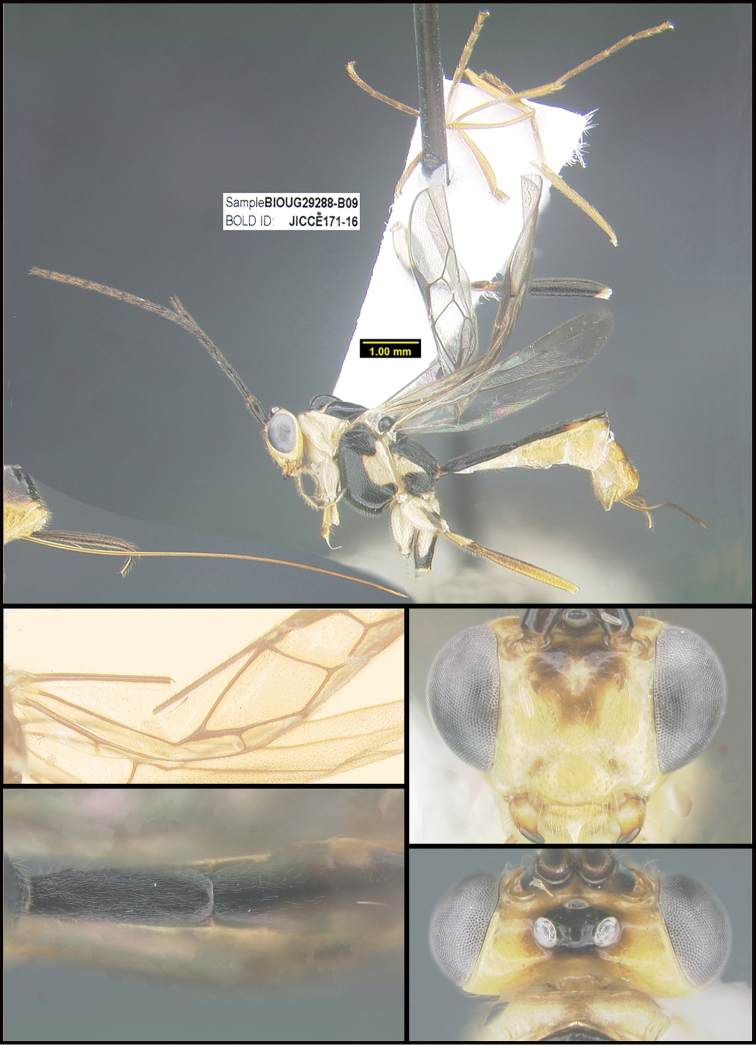
*Macrocentrusjennyphillipsae*, holotype.

#### Macrocentrus jesseausubeli

Taxon classificationAnimaliaHymenopteraBraconidae

Sharkey & van Achterberg sp. nov.

http://zoobank.org/4EBADC76-58DF-4DD2-9F5D-C839894147EF

Figure 310

##### Diagnostics.

BOLD:ACK7403. Consensus barcode. TATCTTATATTTTATATTTGGGCTATGGTCTGGGGTATTAGGTTTATCAATAAGGTTAATTATTCGTATAGAGTTAGGTCAAATTGGTTCATTTATTGGAAATGATCAAATTTATAATAGTATTGTTACTTCTCATGCTTTTATTATAATTTTTTTTATAGTTATACCTATTATAATTGGAGGATTTGGTAATTGGTTAATTCCTTTAATATTAGGTAGAGTTGATATAGCTTTTCCTCGAATAAATAATATAAGATTTTGATTATTGATTCCTTCTTTAACTTTATTAATTTTGAGAGGATTTATAAATATTGGGGTAGGYACAGGATGAACAGTTTATCCTCCTTTATCATTAAATATTAGACATATAGGARTTTCTGTAGATATGGCTATTTTTTCTTTACATTTGGCGGGTGTTTCTTCAATTATAGGTGCTATTAATTTTATTGTTACTATTATAAATATRCGAAATTATGGGGTATTAATAGATAAAATTAGATTATTATTATGATCAGTTTTAATTACAGCTATTTTATTGTTATTATCTTTACCTGTATTAGCTGGTGCCATTACTATATTATTAACTGATCGTAATTTAAATACATCTTTTTTTGATCCTGCTGGTGGGGGGGACCCTATTTTGTACCAACATTTATTT. Belongs to the group with vein M+Cu of forewing distinctly widened apically, mesosternum, ventral half of mesopleuron and metapleuron largely black and first metasomal tergite subparallel-sided. Differs from *M.gretchendailyae*, *M.gustavogutierrezi*, and *M.jennyphillipsae* by having vein 1A of forewing slender subapically and vein cu-a of forewing widened posteriorly.

##### Holotype ♀.

Alajuela, Sector San Cristobal, Río Blanco Abajo, 10.90037, -85.37254, 500 meters, caterpillar collection date: 15/x/2005, wasp eclosion date: 4/xi/2005. Depository: CNC.

***Host data*.** spiloBioLep01 BioLep382 (Crambidae) feeding on *Aphelandraaurantiaca* (Acanthaceae).

***Caterpillar and holotype voucher codes*.** 05-SRNP-6429, DHJPAR0028985.

##### Paratypes.

Host = *Herpetogramma* Solis10 (Crambidae): DHJPAR0028983, DHJPAR0028988, DHJPAR0041515, DHJPAR0041543, DHJPAR0048254. Depository: CNC.

##### Etymology.

*Macrocentrusjesseausubeli* is named to honor Dr. Jesse Ausubel of Rockefeller University for his early and strong support for the development of DNA barcoding for biodiversity identification.

**Figure 310.**
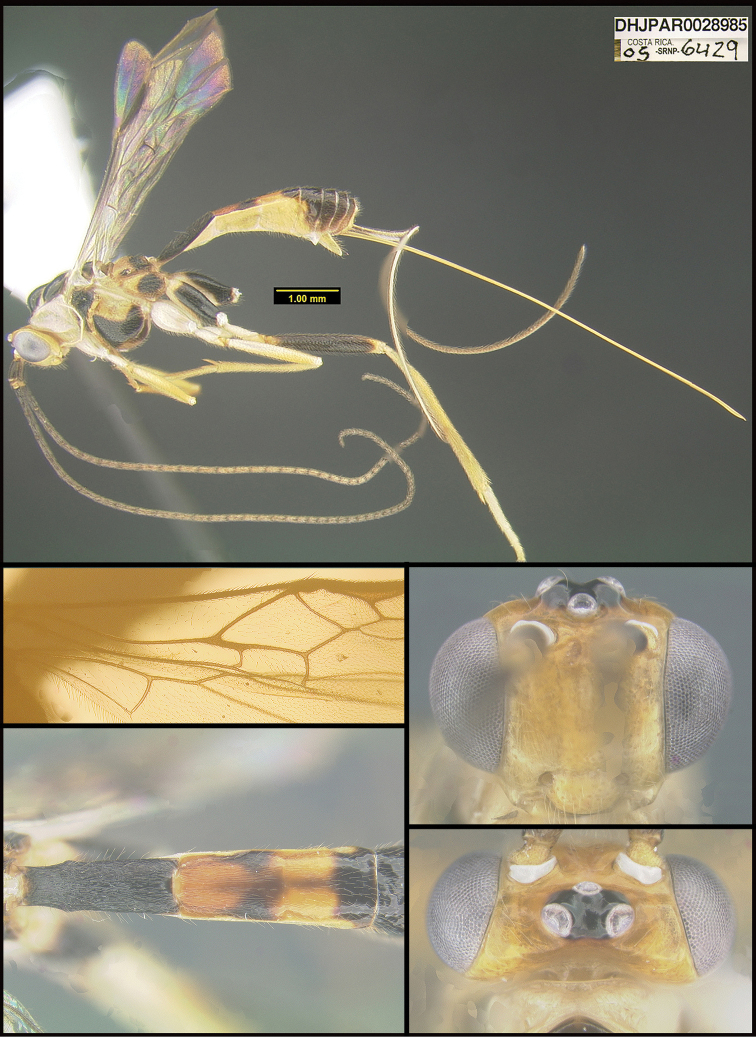
*Macrocentrusjesseausubeli*, holotype.

#### Macrocentrus jessemaysharkae

Taxon classificationAnimaliaHymenopteraBraconidae

Sharkey & van Achterberg sp. nov.

http://zoobank.org/393D1E26-3BC8-43F1-BFC8-3CF59B9E9837

Figure 311

##### Diagnostics.

BOLD:AAW4582. Consensus barcode. TATATTATATTTTTTATTTGGAATATGATCTGGTATATTGGGGTTATCAATAAGTTTAATTATTCGAATAGAATTAGGTCAAATTGGTTCATTTATTGGTAATGATCAAATTTATAATAGAATTGTCACATCTCATGCTTTTATTATAATTTTTTTTATAGTTATACCTATTATAATTGGGGGATTTGGAAATTGATTAATTCCTTTAATATTGGGTAGTGTTGATATAGCTTTTCCTCGAATAAATAATATAAGATTTTGGTTATTAATTCCATCTTTAATATTACTAATTTTAAGGGGATTTTTAAATATTGGTGTAGGTACAGGTTGAACAGTTTATCCTCCTTTATCTATAAATGTTAGTCATATAGGAATTTCTGTTGATATAGCTATTTTTTCTTTGCATTTGGCTGGTATTTCTTCAATTATAGGTGCTATTAATTTTATTGTTACTATTTTAAATATACGAAATTATGGAGTATTAATAGATAAAATTAGATTATTATCATGATCAATTTTAATTACAGCTATTTTATTATTATTATCATTACCAGTATTAGCTGGTGCTATTACTATATTATTAACTGATCGTAATTTGAATACATCATTTTTTGATCCTGCAGGAGGGGGGGATCCTATTTTATATCAACATTTATTT. Recognizable by the combination of the robust (subparallel-sided and approx. 2.3 × as long as wide posteriorly) and dark brown first tergite, with dorsal carinae only basally distinct, vein cu-a of forewing curved but not widened posteriorly, and the antenna largely pale yellowish.

##### Holotype ♀.

Guanacaste, Sector Cacao, Sendero a Maritza, 1 km NW Estación Cacao, 10.92691, -85.46822, 1150 meters, caterpillar collection date: 1/ix/2010, wasp eclosion date: 20/ix/2010. Depository: CNC.

***Host data*.** cram 10-SRNP-36093 (Crambidae) feeding on *Ficuspertusa* (Moraceae)

***Caterpillar and holotype voucher codes*.** 10-SRNP-36093, DHJPAR0042077.

##### Paratypes.

None.

##### Etymology.

*Macrocentrusjessemaysharkae* is named in honor of science writer Ms. Jesse Mayshark in recognition of her 2011 stellar reporting on the All Taxa Biodiversity Inventory of the Great Smoky Mountains National Park, an intellectual inspiration from the same project once anticipated as the ATBI of ACG.

**Figure 311.**
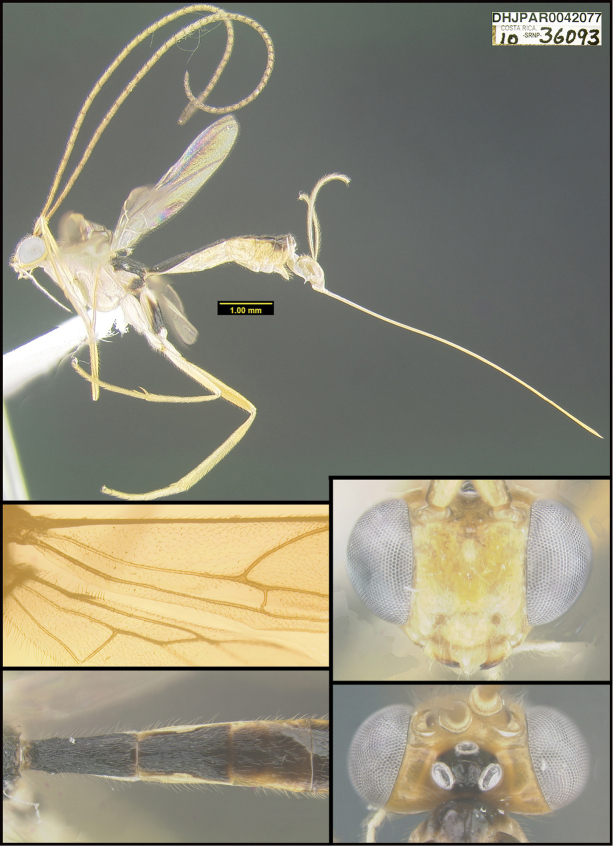
*Macrocentrusjessemaysharkae*, holotype.

#### Macrocentrus jimwhitfieldi

Taxon classificationAnimaliaHymenopteraBraconidae

Sharkey & van Achterberg sp. nov.

http://zoobank.org/4F41A3E2-9D32-43A7-87E8-A875018BAE04

Figure 312

##### Diagnostics.

BOLD:ABX5241. Consensus barcode. TATATTATATTTTTTATTTGGTTTATGATCTGGTGTGTTAGGATTATCTATAAGGCTAATTATTCGTATTGAATTAGGYCAAATTGGTTCTTTTATCGGTAATGATCAGATTTATAATAGAATTGTTACATCACATGCTTTTATTATAATTTTTTTTATAGTTATACCAATTATAATTGGGGGATTTGGAAATTGATTAATTCCTTTAATATTAGGAAGAGTTGATATAGCTTTYCCTCGTATAAATAATATAAGATTTTGGTTATTAGTTCCTTCATTAATATTACTAATTTTAAGGGGATTTATAAATATTGGRGTAGGTACTGGTTGAACAGTCTATCCCCCTTTATCTTTAAATATTAGGCATATAGGAGTTTCTGTTGATATAGCTATTTTTTCATTACATTTAGCAGGAATTTCATCAATTATAGGTGCTATTAATTTTATTATTACTATTATGAATATACGAAATTATGGAGTATTGATAGATAAAATTAGATTATTATCTTGATCTATTTTAATTACAGCAATTTTRTTATTATTATCTTTACCTGTTTTAGCAGGGGCTATTACAATATTATTAACTGATCGAAATTTAAATACATCTTTTTTTGAYCCTGCTGGAGGAGGGGATCCTATTTTGTATCAACATTTATTT. Easy to recognize because of the mostly black hind femur tibia (except base and apex) combined with the posteriorly widened vein cu-a of forewing; metasoma dorsally black; vein 1A of forewing slender.

##### Holotype ♀.

Guanacaste, Sector Pitilla, Leonel, 10.99637, -85.40195, 510 meters, caterpillar collection date: 27/i/2010, wasp eclosion date: 27/ii/2010. Depository: CNC.

***Host data*.***Chlamydastis* Janzen04 (Depressariidae) feeding on *Pouteriacampechiana* (Sapotaceae).

***Caterpillar and holotype voucher codes*.** 10-SRNP-70517, DHJPAR0039160.

##### Paratypes.

Hosts = *Stenoma* Janzen199, *Chlamydastis* Janzen04, and *Antaeotrichafascicularis* (all Depressariidae). DHJPAR0035285, DHJPAR0039156, DHJPAR0039161, DHJPAR0041521, DHJPAR0041523, DHJPAR0041525, DHJPAR0045356, DHJPAR0045361, DHJPAR0049997, DHJPAR0050004, DHJPAR0052194. Depository: CNC.

##### Etymology.

*Macrocentrusjimwhitfieldi* is named to honor Dr. Jim Whitfield of the University of Illinois for his decades of mentorship to DHJ about Braconidae taxonomy and stimulus for their collection and rearing from ACG, while carrying out many braconid taxonomic projects for ACG specimens.

**Figure 313.**
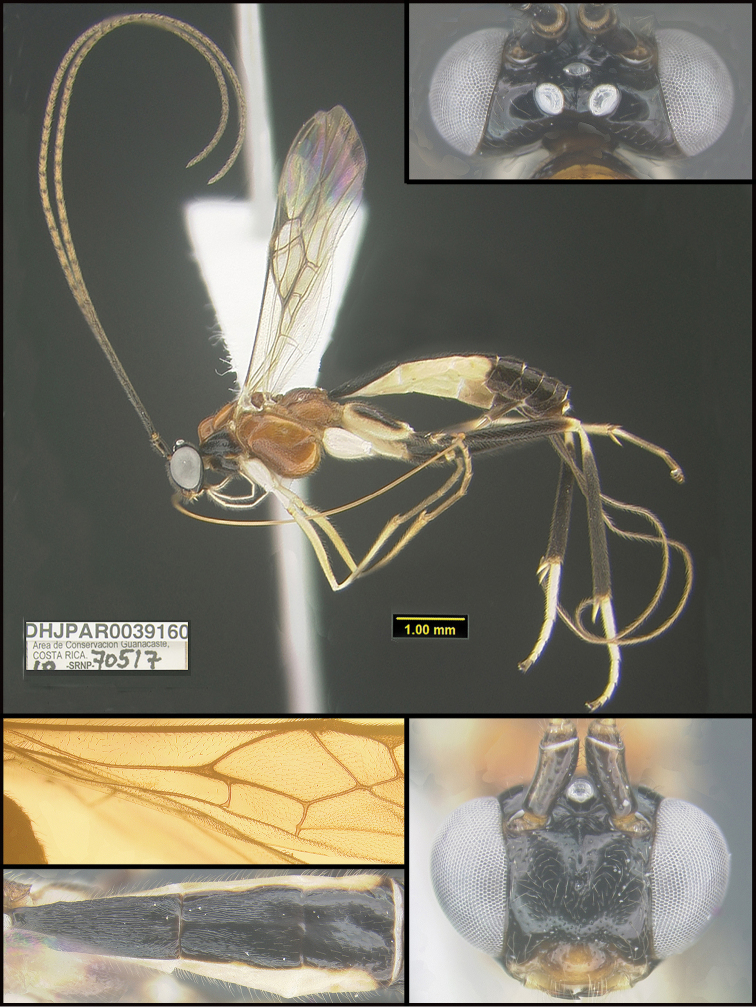
*Macrocentrusjohnbrowni*, holotype.

#### Macrocentrus johnbrowni

Taxon classificationAnimaliaHymenopteraBraconidae

Sharkey & van Achterberg sp. nov.

http://zoobank.org/C3298AB8-AF2A-495B-BB71-9A6B50E446B8

Figure 313

##### Diagnostics.

BOLD:ADJ5301. Consensus barcode. TATATTATATTTTTTATTTGGTATATGATCTGGGATATTAGGGTTATCATTAAGTTTAATTATTCGAATAGAATTAGGTCAAATTGGGGCATTTATTGGGAATGATCAAATTTATAATAGTATTGTTACATCTCATGCTTTTATTATAATTTTTTTTATAGTTATGCCGATTATAATTGGGGGATTTGGTAATTGATTAATTCCTTTAATATTGGGAAGAGTTGATATAGCTTTCCCTCGTATAAATAATATAAGATTTTGATTATTAATTCCGTCTTTAATATTATTAATTTTAAGGGGATTTGTAAATATTGGGGCAGGTACAGGGTGAACAGTTTACCCCCCTTTATCTTTAAATATTAGACATATAGGAGTTTCTGTTGATATAGTTATTTTTTCATTACATTTAGCTGGAATTTCATCAATTATAGGGGCTATTAATTTTATTGTTACTATTTTAAATATACGAAATTATGGGGTATTAATAGATAAAATTAGGTTATTATCATGATCTATTTTAATTACAGCTATTTTATTGTTATTATCTTTACCTGTATTAGCTGGTGCTATTACAATATTATTAACTGATCGTAATTTAAATACATCTTTTTTTGATCCTTCAGGAGGGGGGGATCCTATTTTATATCAACATTTATTT. Shares with *M.iangauldi* the pale yellowish stigma contrasting with mainly dark brown veins of middle third of forewing and the robust first tergite (approx. 2.3 × longer than its apical width). Differs because of the slightly wider vein 1Cua of forewing, which is approx. as long as vein cu-a (slightly narrower and ca. 1.2 × in *M.iangauldi*), basal half of vein C+SC+R of forewing dark brown (yellow in *M.iangauldi*) and head pale yellow (pale brown in *M.iangauldi*).

##### Holotype ♀.

Guanacaste, Sector Santa Maria, Crater Bosque Sendero Adentro, 10.80348, -85.32729, 1594 meters, light trapped, 7/iv/2016. Depository: CNC.

***Host data*.** None.

***Caterpillar and holotype voucher codes*.** 17-SRNP-11371, DHJPAR0061182.

##### Paratypes.

None.

##### Etymology.

*Macrocentrusjohnbrowni* is named to honor Dr. John Brown of the Smithsonian Institution and USDA for his decades of journal editing and Tortricidae species identification for the ACG biodiversity inventory.

**Figure 312.**
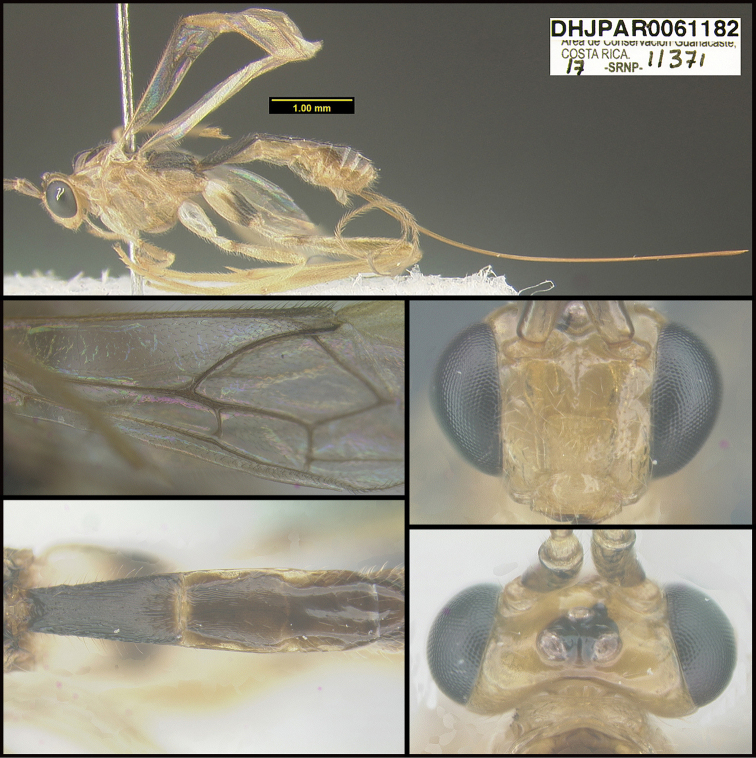
*Macrocentrusjimwhitfieldi*, holotype.

#### Macrocentrus johnburnsi

Taxon classificationAnimaliaHymenopteraBraconidae

Sharkey & van Achterberg sp. nov.

http://zoobank.org/A0356D7D-84DE-4F2E-9355-8D31B9B26D45

Figure 314

##### Diagnostics.

BOLD:ADB2238. Consensus barcode. ATATTATATTTTTTATTTGGAATATGATCAGGTATTTTAGGTTTATCTTTAAGGTTAATTATTCGGATAGAATTAGGTCAAGTTGGTTCATTTATTGGGAATGATCAAATTTATAATAGGATTGTTACATCTCATGCTTTTATTATAATTTTTTTTATAGTTATGCCTATTATAATTGGGGGGTTTGGTAATTGATTTATTCCTTTGATATTAGGTAGGGTTGATATAGCATTCCCTCGAATAAATAATATAAGGTTTTGATTATTGATTCCATCATTGTTTTTATTAATTTTAAGGGGGTTCTTAAATATTGGAGTAGGTACTGGTTGGACTGTTTATCCTCCATTATCATTAAATATTAGTCATATAGGGATTTCTGTTGATATAGCTATTTTTTCTTTACATTTAGCTGGGGTTTCTTCTATTATAGGGTCTATTAATTTTATTGTTACTATTCTTAATATACGAAATTATGGTGTTTTAATAGATAAAATTAGTTTGTTATCTTGATCAATTTTGATTACAGTTATTTTATTGTTATTATCATTACCTGTTTTAGCTGGGGCTATTACAATATTGTTAACTGATCGTAATTTAAATACT---------------------------. Similar to *M.jonathanfranzeni* sp. nov. because of the largely black hind femur and tibia and largely blackish head, but differs by the longer vein 1Cua of forewing (approx. as long as vein cu-a; much shorter than vein cu-a in *M.jonathanfranzeni*), widened vein 1A of forewing, first tergite more widened posteriorly (ca. 2.3 × longer than wide apically; ca. 2.8 × in *M.jonathanfranzeni*) and partly yellowish brown.

##### Holotype ♀.

Guanacaste, Pailas Dos, PL12-6, 10.7637, -85.333, 853 meters, 5/x/2013, Malaise trap. Depository: CNC.

***Host data*.** None.

***Holotype voucher code*.**BIOUG28712-G07.

##### Paratypes.

None.

##### Etymology.

*Macrocentrusjohnburnsi* is named to honor Dr. John Burns of the Smithsonian Institution for his decades of taxonomic projects and mentorship of DHJ and WH about the Hesperiidae of ACG, as well as happily accepting their DNA barcoding as part of their taxonomic discovery.

**Figure 314.**
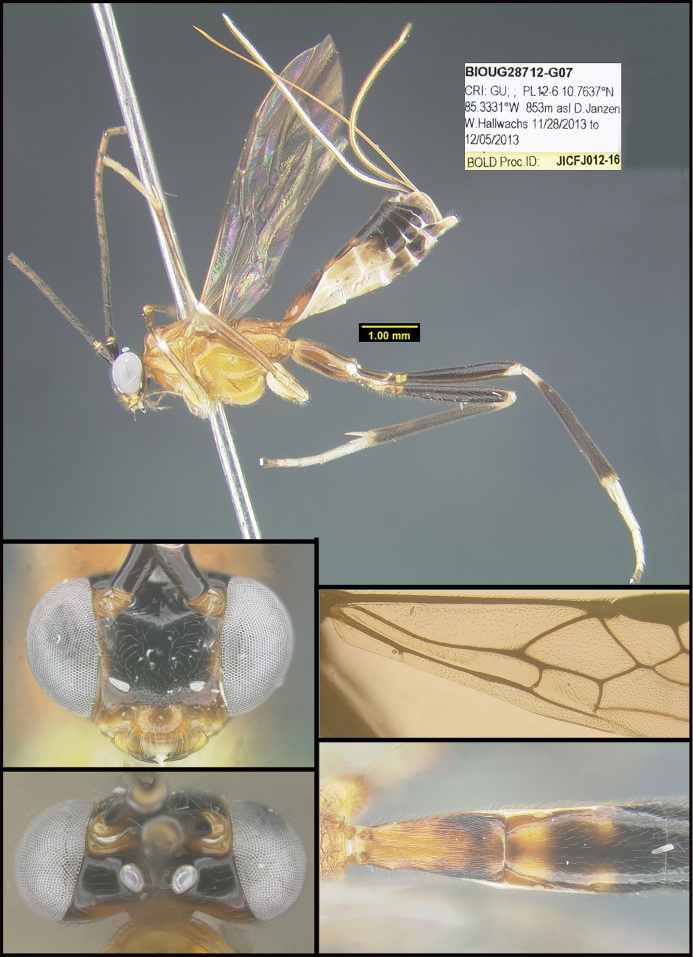
*Macrocentrusjohnburnsi*, holotype.

#### Macrocentrus jonathanfranzeni

Taxon classificationAnimaliaHymenopteraBraconidae

Sharkey & van Achterberg sp. nov.

http://zoobank.org/4AED9F6A-B980-4843-B7BD-2B20A3FB4A07

Figure 315

##### Diagnostics.

BOLD:AAX3465. Consensus barcode. ATATTATATTTTTTATTTGGAATATGATCAGGTATATTAGGTTTATCTATAAGTTTAATTATTCGAATAGAATTAGGTCAAGTAGGGTCATTAATTGGAAATGATCAAATTTATAATAGATTTGTAACTGCTCATGCATTTATTATAATTTTTTTTATAGTTATACCAATTATGATTGGGGGGTTTGGTAATTGATTAATTCCATTAATATTAGGTAGAGTTGATATAGCTTTCCCCCGAATAAATAATATAAGATTTTGATTGTTAATTCCTTCATTAATATTATTAATTTTAAGGGGATTTATAAATATTGGTGTAGGGACAGGATGAACAGTTTATCCTCCTTTATCTTTAAATATTAGACATATAGGTATTTCTGTAGATATAGCAATTTTTTCTTTACATTTAGCTGGAATTTCTTCAATTATAGGGGCTATAAATTTTATTATTACTATTATTAATATACGTAATAATGGAGTATTAATAGATAAAATTAGATTATTATGTTGATCTATTTTAATTACAGCTTTTTTATTATTATTATCTTTACCTGTATTAGCAGGAGCAATTACTATATTATTAACTGATCGTAATTTAAATACTTCTTTTTTTGATCCGGCCGGTGGGGGAGATCCTATTTTATATCAACATTTATTT. Characterized by the combination of short vein 1Cua of forewing (distinctly shorter than vein cu-a), metasoma dark brown dorsally, white face, and elongate maxillary palp.

##### Holotype ♂.

Guanacaste, Sector Cacao, Sendero Derrumbe, 10.92918, -85.46426, 1220 meters, caterpillar collection date: 21/iii/2007, wasp eclosion date: 15/iv/2007. Depository: CNC.

***Host data*.***Pantographasuffusalis* (Crambidae) feeding on *Hampeaappendiculata* (Malvaceae).

***Caterpillar and holotype voucher codes*.** 07-SRNP-35658, DHJPAR0028189.

##### Paratypes.

None.

##### Etymology.

*Macrocentrusjonathanfranzeni* is named to honor Mr. Jonathan Franzen for his perceptive portrayal to the world of ACG and its philosophy towards BioAlfa.

**Figure 315.**
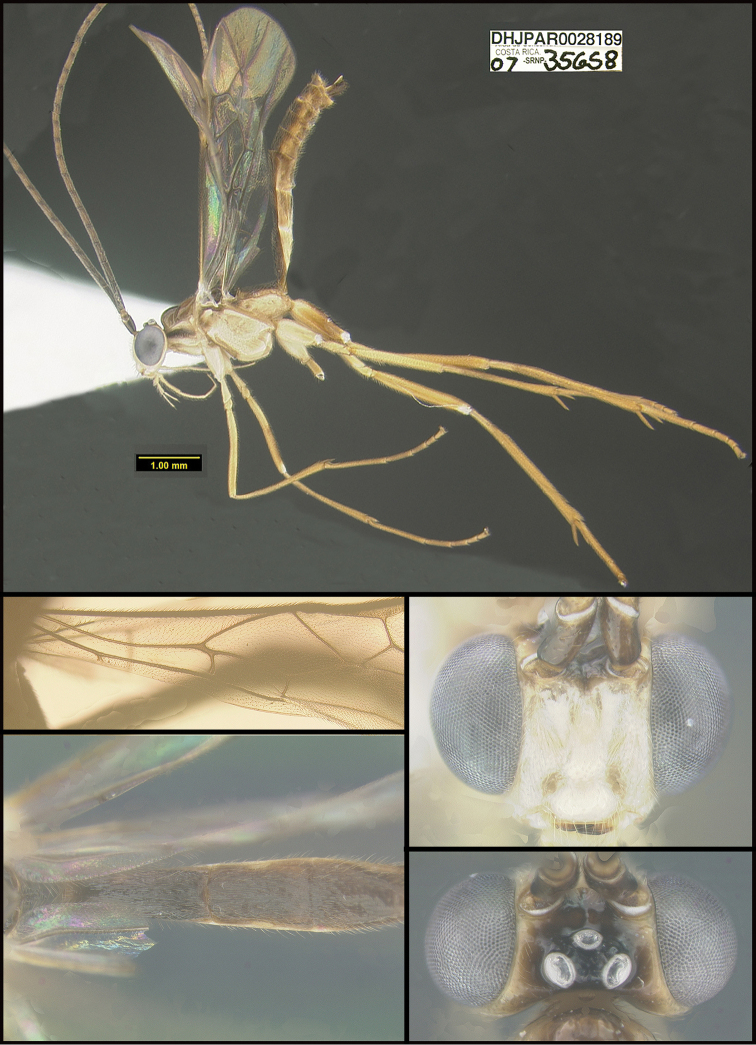
*Macrocentrusjonathanfranzeni*, holotype.

#### Macrocentrus jonathanrosenbergi

Taxon classificationAnimaliaHymenopteraBraconidae

Sharkey & van Achterberg sp. nov.

http://zoobank.org/5DD43C14-87A3-4E85-8DFF-49C394C11889

Figure 316

##### Diagnostics.

BOLD:AAT9449. Consensus barcode. TATATTATATTTTTTATTTGGAATATGGTCTGGTATAGTGGGTTTATCAATAAGTTTAATTATTCGGATGGAATTAGGTTGTGTAGGATCATTAATTGGAAATGATCAAATTTATAATAGATTTGTTACAGCTCATGCTTTTGTTATAATTTTTTTTATAGTTATACCTATTATAATTGGAGGATTTGGTAATTGATTAATTCCTTTAATATTAGGTAGTGTGGATATAGCTTTCCCTCGAATAAATAATATAAGATTTTGATTATTAATTCCTTCAATTATATTATTAATAATAAGGGGATTTATAAATATTGGAGTAGGAACTGGATGAACTGTTTATCCTCCTTTATCATTAAATATTAGTCATATAGGTATTTCTGTTGATATAGCTATTTTTTCATTACATTTAGCTGGTATTTCTTCAATTATAGGGGCTATCAATTTTATTGTTACTATTTTAAATATACGAAATTATGGTGTTTTAATGGATAAAATTAGTTTATTATCTTGATCTATTTTGATTACAGCTATTTTATTATTATTATCTTTACCTGTATTAGCTGGTGCAATTACTATATTATTAACGGATCGTAATTTAAATACTTCTTTTTTTGATCCAGCTGGAGGAGGAGATCCTATTTTATATCAACATTTATTT. Easily recognizable species because of the large ventral lamella (slender basal lobe) of the tarsal claws, anterior ocellus close to level of antennal sockets, metapleuron distinctly punctate, outer side of middle femur with numerous pegs, and elongate maxillary palp.

##### Holotype ♀.

Guanacaste, Sector Mundo Nuevo, Estación La Perla, 10.76737, -85.43313, 325 meters, caterpillar collection date: 31/v/2010, wasp eclosion date: 30/vii/2010. Depository: CNC.

***Host data*.***Hahncappsia* BioLep471 (Crambidae) feeding on *Iresinecalea* (Amaranthaceae).

***Caterpillar and holotype voucher codes*.** 10-SRNP-55504, DHJPAR0040239.

##### Paratypes.

None.

##### Etymology.

*Macrocentrusjonathanrosenbergi* is named to honor Mr. Jonathan Rosenberg of Google for his generous perception of the potential relevance of ACG to biodiversity understanding of Google employees.

**Figure 316.**
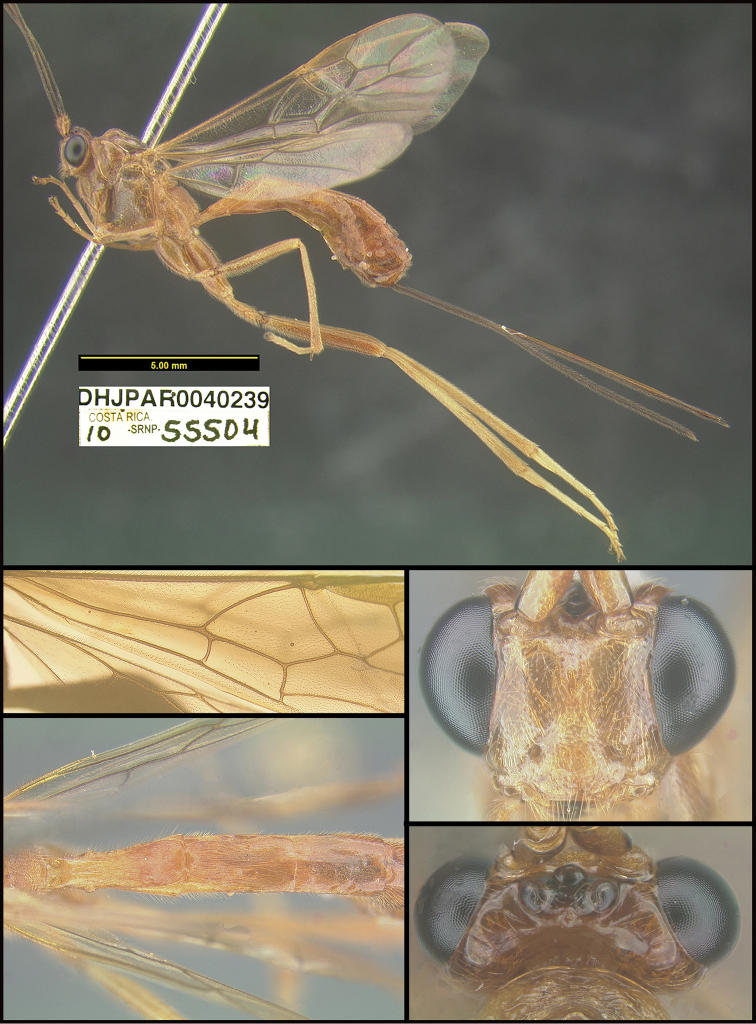
*Macrocentrusjonathanrosenbergi*, holotype.

#### Macrocentrus jorgebaltodanoi

Taxon classificationAnimaliaHymenopteraBraconidae

Sharkey & van Achterberg sp. nov.

http://zoobank.org/4F880DBD-2123-4EF1-8A3D-D5E81283C63E

Figure 317

##### Diagnostics.

BOLD:ADB3971. Consensus barcode. ATATTATATTTTTTATTTGGTATATGATCAGGTATATTGGGTTTATCAATAAGTTTAATTATTCGAATAGAATTAGGTCAAGTAGGTTCATTAATTGGTAATGATCAAATTTATAATAGAATTGTTACTGCTCATGCTTTTATTATAATTTTTTTTATAGTTATACCAATTATAATYGGGGGATTTGGAAATTGATTAATTCCTTTAATATTAGGAAGTGTGGATATAGCTTTCCCTCGAATAAATAATATAAGATTTTGGTTATTAATTCCTTCATTAATATTATTAATTTTAAGGGGATTTTTAAATATTGGGGTCGGAACAGGTTGAACAGTTTATCCTCCTTTATCATTAAATATTAGTCATATAGGTATTTCTGTGGATATAGCTATTTTTTCATTACATTTAGCTGGTGTCTCATCTATTATAGGAGCTATTAATTTTATTGTTACAATTTTAAATATACGAAATTATGGGGTTTTAATAGATAAAATTAGATTATTATCTTGATCAATTTTAATTACAGCTATTTTATTATTATTATCTTTACCTGTTTTAGCAGGAGCTATTACTATATTATTAACAGAT. Easily recognizable because of the slender and long vein 1Cua of forewing (much longer than vein cu-a) and largely setose subbasal cell of the forewing.

##### Holotype ♀.

Guanacaste, Pailas Dos, PL12-3, 10.7631, -85.3344, 820 meters, 6/iii/2014, Malaise trap. Depository: CNC.

***Host data*.** None.

***Holotype voucher code*.**BIOUG29687-D11.

##### Paratypes.

All Malaise-trapped: BIOUG29620-B02, BIOUG30041-H09, BIOUG30934-A02. Depository: CNC.

##### Etymology.

*Macrocentrusjorgebaltodanoi* is named to honor Sr. Jorge Baltodano (RIP) for his many years as a neighboring collaborator with ACG and for accepting the challenge of being the first President of the Comité Local for ACG; part of his ranch is now a permanent part of ACG thanks to his foresight and spirit for conservation.

**Figure 317.**
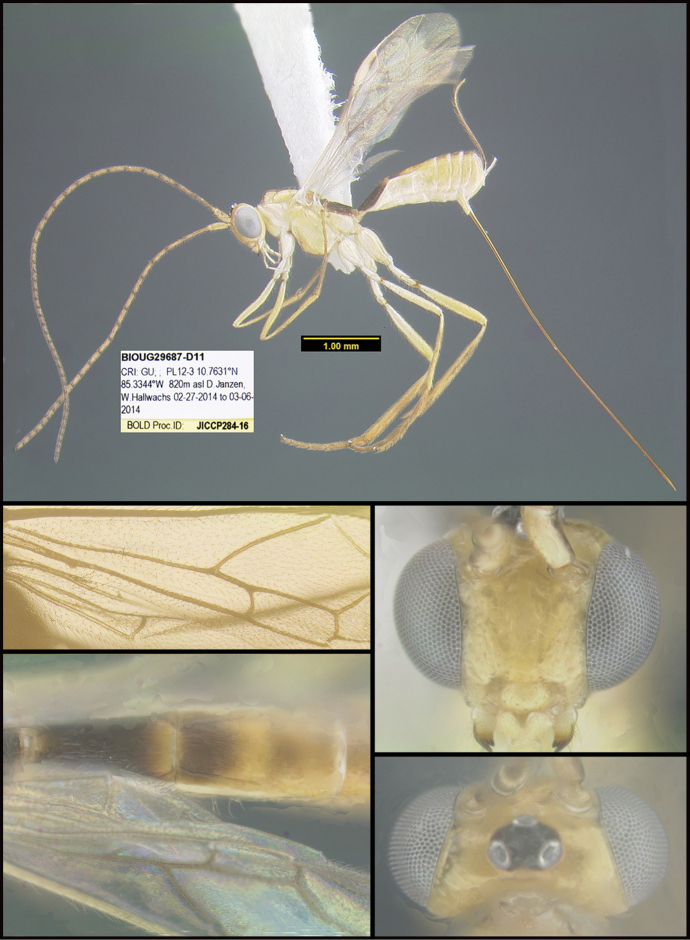
*Macrocentrusjorgebaltodanoi*, holotype.

#### Macrocentrus lucianocapelli

Taxon classificationAnimaliaHymenopteraBraconidae

Sharkey & van Achterberg sp. nov.

http://zoobank.org/5CA7A8CD-EA8D-40E6-9374-3465761C5D11

Figure 318

##### Diagnostics.

BOLD:ACK7466. Consensus barcode. YATATTRTATTTTTTRTTTGGTATRTGRTCTGGGGWRWTAGGTTTATCAYTAAGTTTAATTATTCGTATAGAATTRGGTCAAATTGGTTYATTTATTGGAAATGATCAAATTTAYAATAGTATTGTTACTTCTCATGCTTTTATTATAATTTTTTTTATAGTTATRCCTATTATAATTGGGGGKTTTGGHAATTGRTTRATTCCYTTAATATTAGGRAGTGTTGATATAGCTTTYCCTCGAATAAATAATATAAGATTTTGATTATTARTTCCTTCTTTAATATTATTAATTTTAAGWGGRTTTATAAATATTGGTGTAGGTACAGGATGAACAGTWTAYCCYCCTTTATCAYTAAATGTTAGTCATATAGGRATTTCTGTMGATATAGYTATTTTTTCAYTACATTTGGCTGGWATTTCTTCAATTATAGGKGCTATTAATTTTATTGTTACTATTATAAATATACGAAATTATGGGGTATTAATAGATAAAATTAGATTATTATYATGATCAATTTTAATTACRGCTATTTTATTATTRTTATCTTTACCTGTGTTAGCTGGTGCWATTACAATATTRTTAACTGAYCGTAATTTAAATACATCYTTTTTTGAYCCTGCTGGAGGAGGRGRYCCTATTTTATAYCAACATTTATTT. Belongs to the group with vein M+Cu of forewing distinctly widened apically. Ventral halves of mesopleuron and metapleuron brownish yellow. It shares with *M.hannahjamesae* the somewhat widened subapical part of vein 1A of forewing, but differs by the comparatively robust and posteriorly distinctly widened first tergite (parallel-sided in *M.hannahjamesae*), face entirely yellowish brown (with dark brown heart-shaped patch in *M.hannahjamesae*), and hind tibia brownish yellow (largely dark brown in *M.hannahjamesae*).

##### Holotype ♀.

Alajuela, Sector Rincon Rain Forest, Palomo, 10.962, -85.28, 96 meters, caterpillar collection date: 15/x/2013, wasp eclosion date: 2/xi/2013. Depository: CNC.

***Host data*.***Apogeshna* stenialisDHJ03 (Crambidae) feeding on *Bravaisiaintegerrima* (Acanthaceae).

***Caterpillar and holotype voucher codes*.** 13-SRNP-79776, DHJPAR0054348.

##### Paratypes.

None.

##### Etymology.

*Macrocentruslucianocapelli* is named to honor Sr. Luciano Capelli (RIP) for his generous and enormously illustrative photography of ACG and its biodiversity for ACG and public use.

**Figure 318.**
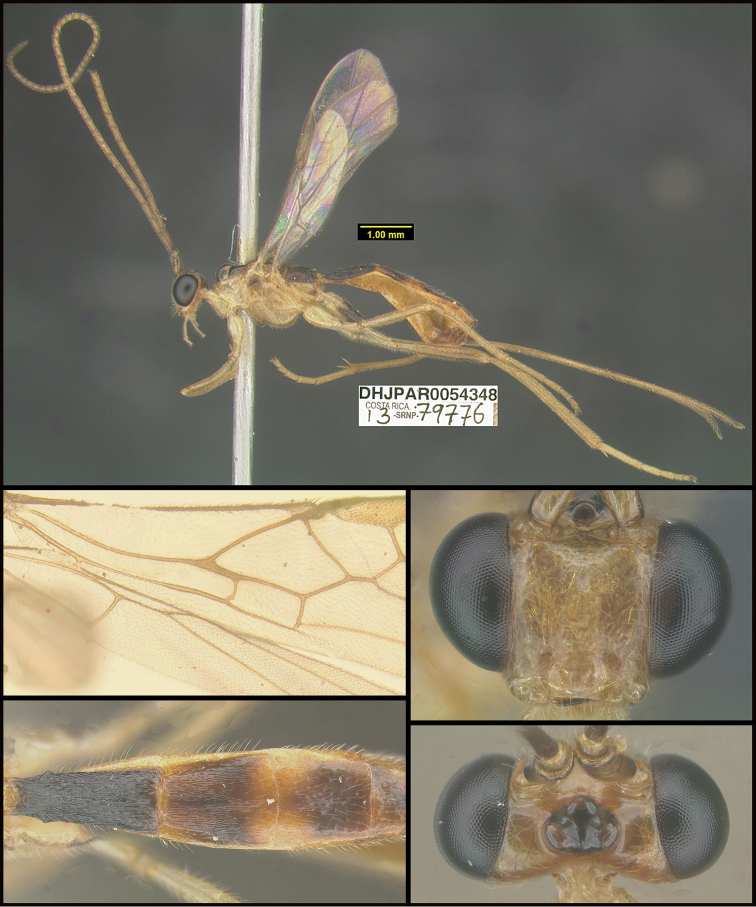
*Macrocentruslucianocapelli*, holotype.

## Chapter 9: Orgilinae

Members of all genera of Orgilinae are koinobiont endoparasitoids of caterpillars from a wide range of families. All species with previously known life-histories are solitary, but here we describe a gregarious species, *Orgilusebbenielsoni* Sharkey, sp. nov. (BOLD:ABA8537). Eleven species were recorded from Costa Rica and ca. 20 from the entire neotropics prior to this publication. The genera of New World Orgilinae may be identified with the following key. For the Orgilinae NJ tree, see Suppl. material 8.

### Key to the New World genera of the subfamily Orgilinae

**Table:** 

1	A. Forewing vein 1RS present; second submarginal cell quadrate; Chile and Argentina.	*** Antestrix ***
–	B. Forewing vein 1RS absent; second submarginal cell triangular or absent; widespread	**2**
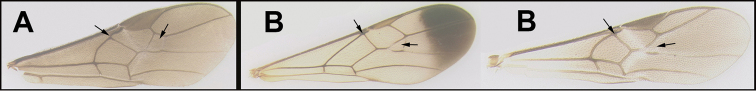		
2(1)	A. Subbasal cell of hind wing small; vein r-m of forewing usually (95%) present	*** Stantonia ***
–	B. Subbasal cell of hind wing large; vein r-m of forewing usually (99%) absent	**3**
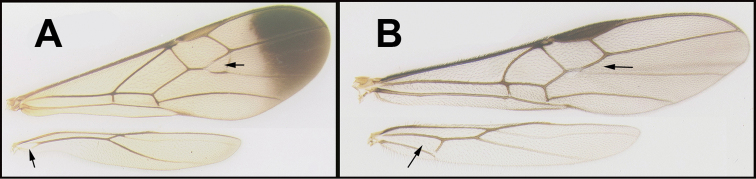		
3(2)	A. Basitarsomere of hind leg swollen; Neotropical, rare	*** Podorgilus ***
–	B. Basitarsomere of hind leg normal, not swollen; widespread, common	**4**
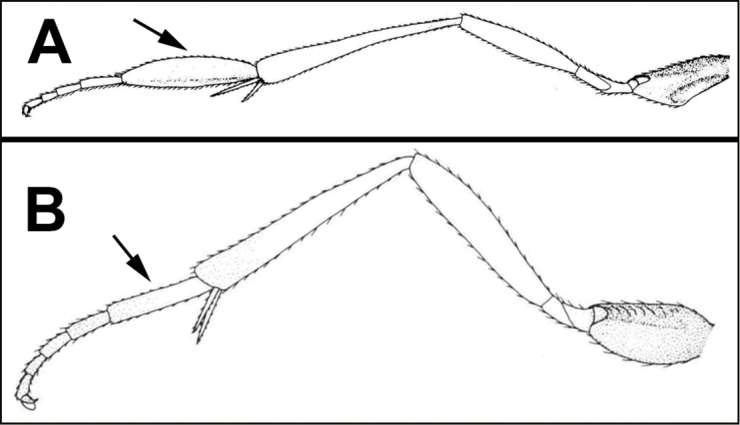		
4(3)	A. Mesosoma flat dorsally; hind trochantellus ventrally distinctly longer than hind trochanter; Neotropical, rare	*** Doryctorgilus ***
–	B. Mesosoma convex dorsally; hind trochantellus ventrally at most approx. as long as hind trochanter; widespread, common	*** Orgilus ***
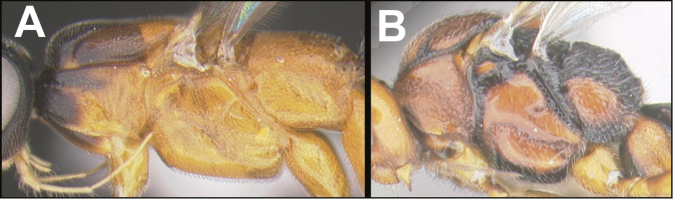		

#### *Orgilus* Haliday, 1833

*Orgilus* have been recorded from a wide range of Lepidoptera larvae (Yu et al. 2016). Only one species, *O.jennieae* Marsh, 1979, a parasite of the potato tuber-worm, *Phthorimaeaoperculella* (Gelechiidae), is recorded from Costa Rica. Of the species described here it is closest to *Orgilusmikesmithi* Sharkey, sp. nov. Fig. 339, BOLD:ADF7540, but differs in many color and structural ways, e.g., the ovipositor is 1.5 × the length of the metasoma in *O.jennieae* but shorter than the metasoma in *Orgilusmikesmithi*. A handful of described species are from Panama (van Achterberg 1987), but these are all in the subgenera *Aporgilus* and *Anakorgilus*, no members of which are treated below. The remaining described Neotropical species are from countries distant from Costa Rica with little probability of being conspecific with the species proposed below.

##### Orgilus amyrossmanae

Taxon classificationAnimaliaHymenopteraBraconidae

Sharkey sp. nov.

http://zoobank.org/37C47907-F949-47DE-9E21-A2A474AF7F5D

Figure 319

###### Diagnostics.

BOLD:ADY7227. Consensus barcode. AATTTTATATTTTTTATTTGGAATTTGATCAGGTATTTTAGGTATATCAATAAGTTTAATTATTCGTTTGGAATTAGGTATACCTGGTAGATTGATTGGTAATGATCAAATTTATAATAGGATTGTGACAGCACATGCTTTTGTAATAATTTTTTTTATAGTTATACCTATTATAATTGGTGGATTTGGTAATTGATTAATTCCTATAATATTAGGATGTCCAGATATGGCATTTCCTCGTATAAATAATATGAGATTTTGATTATTAATTCCTTCAATAATTTTTTTGATTTTTTCAAGAATTTTAAATATTGGTGTAGGAACTGGGTGAACAGTGTATCCTCCTTTATCATTAAGATTAGGACATGGTGGAATTTCAGTGGATATAGCTATTTTTTCTTTACATTTAGCTGGGGCTTCTTCAATTATAGGAGCTATTAATTTTATTACAACAATTTTTAATATACGATCAAGTATGATTACTATGGATAAAATTTCTTTATTAAGATGGTCAGTGTTAATTACTGCTATTTTATTATTATTATCATTACCTGTTTTAGCTGGAGCTATTACAATATTATTAAGTGATCGAAATTTAAATACTTCTTTTTTTGATCCTGCAGGGGGGGGCGATCCAATTTTATATCAACATTTATTT.

###### Holotype ♀.

Alajuela, Sector Rincon Rain Forest, Rio Francia Arriba, 10.89666, -85.29003, 400 meters, caterpillar collection date: 9/ix/2010, wasp eclosion date: 1/x/2010. Depository: CNC.

***Host data*.***Rectiostomaeowilsoni* (Depressariidae) feeding on *Turpiniaoccidentalis* (Staphyleaceae).

***Caterpillar and holotype voucher codes*.** 10-SRNP-43220, DHJPAR0041408.

###### Paratype.

DHJPAR0064113. Depository: CNC.

###### Etymology.

*Orgilusamyrossmanae* is named in honor of Amy Rossman for her participation in the international NSF-funded planning meeting for the All Taxa Biodiversity Inventory (ATBI) of Terrestrial Systems, and contributing her wisdom to the planning that was the founding of Costa Rica’s national BioAlfa today.

**Figure 319.**
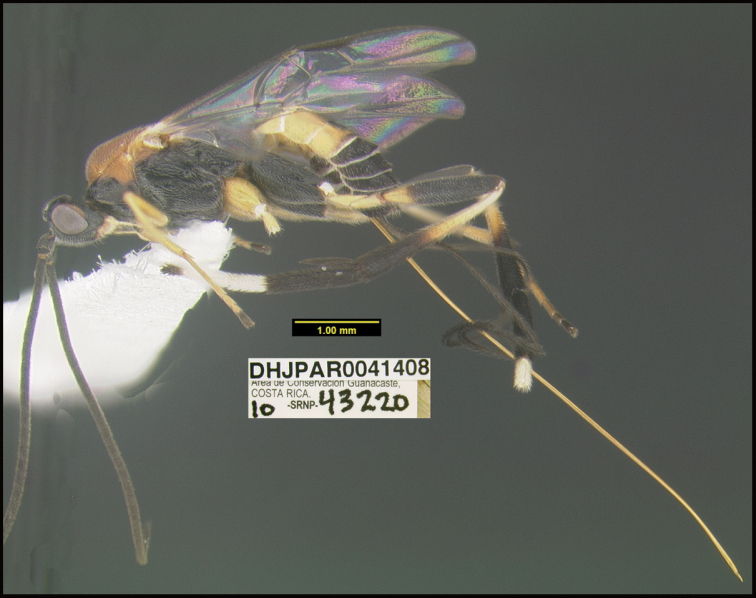
*Orgilusamyrossmanae*, holotype.

##### Orgilus carrolyoonae

Taxon classificationAnimaliaHymenopteraBraconidae

Sharkey sp. nov.

http://zoobank.org/5A5A7A46-03C1-4FB9-A1DE-DF9D2DCAE5DA

Figure 320

###### Diagnostics.

BOLD:ADB0533. Consensus barcode. ATTTTATATTTTATATTTGGTATTTGATCAGGTATTTTAGGAATATCTATAAGATTAATTATTCGAATGGAATTAGGTATACCTGGTAGATTGATTGGAAATGATCAAATTTATAATAGAATTGTAACAGCTCATGCATTTATTATAATTTTTTTTATAGTTATGCCAATTATAATTGGTGGTTTTGGAAATTGATTAATTCCTATAATACTAGGATGTCCAGATATAGCATTTCCTCGAATAAATAATATAAGGTTTTGGTTATTAATTCCTTCAATAATTTTTTTAATTTTTAGAAGAATTTTAAATGTTGGGGTTGGTACTGGTTGAACAGTGTATCCACCTTTATCTTTAATAATTGGTCATGGGGGTTTATCTGTTGATATAGCTATTTTTTCTTTACATTTAGCTGGAATTTCTTCAATTATGGGTGCAATTAATTTTATTACTACTATTTTAAATATACGATCTGATAAAGTTTTAATGGATAAAATTTCATTATTAAGATGATCTGTTTTAATTACAGCTATTTTATTATTATTATCATTACCTGTATTGGCAGGG------------------------------------------------------------------------.

###### Holotype ♂.

Guanacaste, Sector Pailas Dos, PL12-1, 10.7642, -85.335, 828 meters, 12/vi/2014, Malaise trap. Depository: CNC.

***Host data*.** None.

***Holotype voucher code*.** BIOUG29134-B08.

###### Paratypes.

None.

###### Etymology.

*Orgiluscarrolyoonae* is named in honor of Carrol Yoon’s long-appreciated contributions to publicity for ACG, GDFCF, and now, BioAlfa.

**Figure 320.**
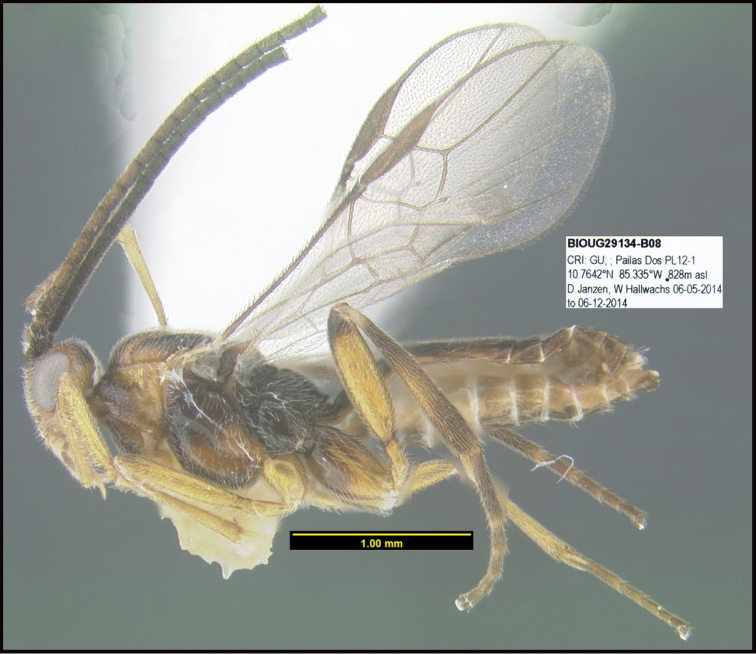
*Orgiluscarrolyoonae*, holotype.

##### Orgilus christhompsoni

Taxon classificationAnimaliaHymenopteraBraconidae

Sharkey sp. nov.

http://zoobank.org/2FA287BF-EB8C-4A3C-9E45-EF2004CC0499

Figure 321

###### Diagnostics.

BOLD:ADW2886. Consensus barcode. TTTATATTTTTTATTTGGAATTTGAGCTGGTATTTTAGGTATATCAATAAGATTAATTATTCGAATAGAATTAGGTATACCAGGAAGATTAATTGGAAATGATCAGATTTATAATAGAATTGTTACAGCTCATGCATTTGTAATAATTTTTTTTATAGTTATACCAATTATAATTGGRGGATTTGGTAATTGATTAATTCCTATAATATTAGGATGTCCTGATATAGCTTTCCCACGTATAAATAATATAAGTTTTTGATTATTAATTCCTTCACTTGTATTTTTAATTTTTAGAAGGGTATTAAATGTAGGGGTAGGTACTGGTTGAACTGTTTATCCTCCTTTATCTTTATTAATTGGTCATGGAGGTTTGTCAGTTGATATAGCTATTTTTTCTTTACATTTGGCAGGTATTTCTTCAATTATAGGTGCAGTTAATTTTATTACAACAATTTTAAATATGCGATCTGATTATKTTTATATAGATAAAATTTCTTTATTATGTTGATCAGTTTTAATTACAGCTATTTTATTATTATTATCTTTACCAGTATTAGCTGGAGCAATTACTATATTATTAACAGATCGGAATTTAAATACTTCATTTTTTGACCCTTCTGGAGGTGGAGATCCAATTTTATATCAACATTTATT.

###### Holotype ♀.

Guanacaste, Sector Pailas Dos, PL12-3, 10.7631, -85.3344, 820 meters, 2/iv/2015, Malaise trap. Depository: CNC.

***Host data*.** None.

***Holotype voucher code*.**BIOUG44707-C12.

###### Paratype.

BIOUG44846-A07. Depository: CNC.

###### Etymology.

*Orgiluschristhompsoni* is named in honor of Chris Thompson attending the international NSF-funded planning meeting for the All Taxa Biodiversity Inventory (ATBI) of Terrestrial Systems, and contributing his wisdom to the planning that was the founding of Costa Rica’s national BioAlfa today.

**Figure 321.**
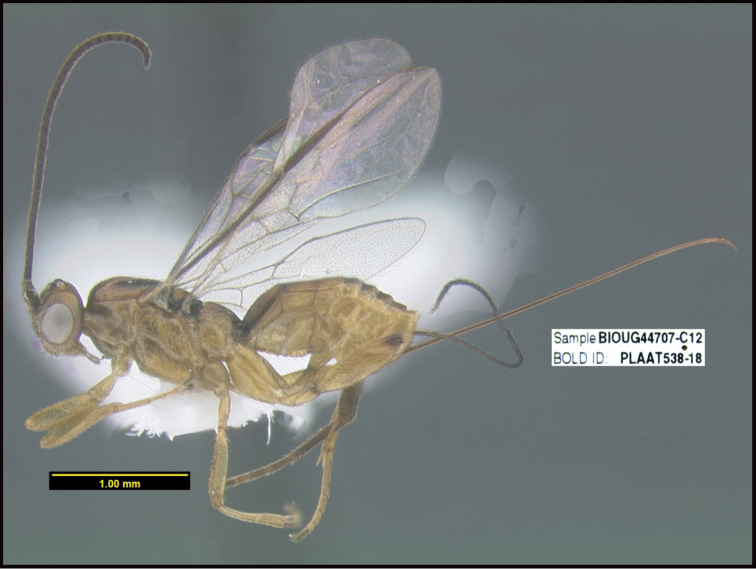
*Orgiluschristhompsoni*, holotype.

##### Orgilus christinemcmahonae

Taxon classificationAnimaliaHymenopteraBraconidae

Sharkey sp. nov.

http://zoobank.org/5057F001-B6A3-4B9B-87A8-B61375FE5162

Figure 322

###### Diagnostics.

BOLD:AAU1670. Consensus barcode. AATTTTATATTTTTTATTTGGTATTTGATCTGGAATTTTAGGAATATCTATAAGTTTATTAATTCGTATAGAATTAGGTATACCTGGGAGATTAATTGGTAATGATCAAATTTATAATAGTATTGTAACAGCACATGCTTTTATTATAATTTTTTTTATAGTTATACCTATTATAATTGGTGGATTTGGTAATTGATTAATTCCTATAATATTAGGATGTCCAGATATAGCTTTTCCTCGTATAAATAATATAAGGTTTTGATTATTGATTCCTTCTTTAATTTTTTTAATTTTTAGTAGTGTTTTAAATGTTGGAGTTGGTACAGGATGAACAGTTTATCCTCCTTTATCTTTAACTATTGGTCATGGGGGATTATCAGTTGATATGGCTATTTTTTCTTTACATTTAGCAGGTATTTCTTCAATTATAGGGGCTATTAATTTTATTACAACTATTTTGAATATACGTTCTGATAAGGTTTTAATAGATAAAATTTCTTTATTAAGATGATCTGTTTTAATTACTGCTATTTTATTATTATTATCTTTACCAGTTTTAGCTGGAGCAATTACAATATTATTAACAGATCGTAATTTAAATACATCTTTTTTTGATCCTTCTGGTGGGGGAGATCCTATTTTATATCAGCATTTATTT.

###### Holotype ♀.

Alajuela, Sector San Cristobal, Finca San Gabriel, 10.87766, -85.39343, 645 meters, caterpillar collection date: 26/vi/2013, wasp eclosion date: 20/vii/2013. Depository: CNC.

***Host data*.***Antaeotricha* Janzen24 (Depressariidae) feeding on *Coccolobapeltata* (Polygonaceae).

***Caterpillar and holotype voucher codes*.** 13-SRNP-3328, DHJPAR0053050.

###### Paratypes.

DHJPAR0040025, DHJPAR0040027, DHJPAR0052982, DHJPAR0053050. Depository: CNC.

###### Etymology.

*Orgiluschristinemcmahonae* is named in honor of Christine McMahon attending the international NSF-funded planning meeting for the All Taxa Biodiversity Inventory (ATBI) of Terrestrial Systems, and contributing her wisdom to the planning that was the founding of Costa Rica’s national BioAlfa today.

**Figure 322.**
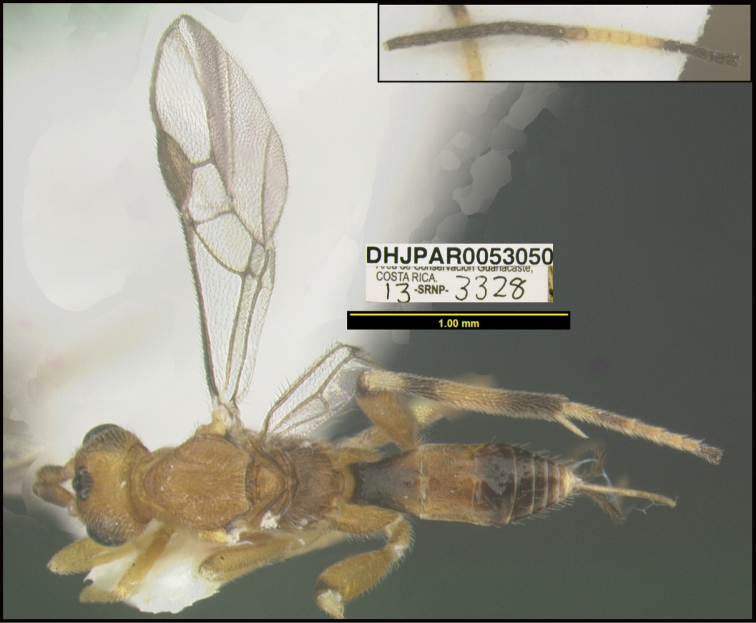
*Orgiluschristinemcmahonae*, holotype.

##### Orgilus dianalipscombae

Taxon classificationAnimaliaHymenopteraBraconidae

Sharkey sp. nov.

http://zoobank.org/4302B074-A74E-4094-94D5-43851E3027CA

Figure 323

###### Diagnostics.

BOLD:AAM1105. Consensus barcode. TATTTTATATTTTATATTTGGTATTTGATCAGGTATTTTAGGAATATCAATAAGTTTACTTATTCGAATAGAACTTGGAATACCTGGTAGTTTAATTGGTAATGATCAAATTTATAATAGAATTGTAACAGCTCATGCATTTATTATAATTTTTTTTATGGTTATACCAATTATAATTGGTGGATTTGGAAATTGATTGATTCCTATAATATTAGGATGTCCAGATATAGCATTTCCACGAATAAATAATATAAGATTTTGATTATTAATTCCTTCAATAATTTTTTTAATTTTTAGCAGAGTTTTAAATGTAGGGGTTGGTACTGGATGAACAGTTTATCCACCTTTATCTTTAACAATTGGGCATGGGGGATTATCTGTTGATATAGCTATTTTTTCTTTACATTTGGCGGGTATTTCTTCTATTATAGGAGCAATTAATTTTATTACTACAATTTTAAATATACGATCTGATAAAGTTTTAATAGATAAAATTTCATTGTTAAGATGATCTGTTTTAATTACAGCAATTTTATTATTATTATCATTACCTGTATTGGCAGGTGCTATTACAATATTATTAACTGATCGAAATTTAAATACTTCTTTTTTTGATCCTTCAGGTGGTGGAGATCCAATTTTATATCAACATTTATTT.

###### Holotype ♀.

Guanacaste, Sector Mundo Nuevo, Vado Miramonte, 10.77175, -85.43400, 305 meters, caterpillar collection date: 17/ii/2010, wasp eclosion date: 4/iii/2010. Depository: CNC.

***Host data*.** elachJanzen01 Janzen786 (Depressariidae) feeding on *Ingavera* (Fabaceae).

***Caterpillar and holotype voucher codes*.** 10-SRNP-55161, DHJPAR0039036.

###### Paratypes.

None.

###### Etymology.

*Orgilusdianalipscombae* is named in honor of Diana Lipscomb attending the international NSF-funded planning meeting for the All Taxa Biodiversity Inventory (ATBI) of Terrestrial Systems, and contributing her wisdom to the planning that was the founding of Costa Rica’s national BioAlfa today.

**Figure 323.**
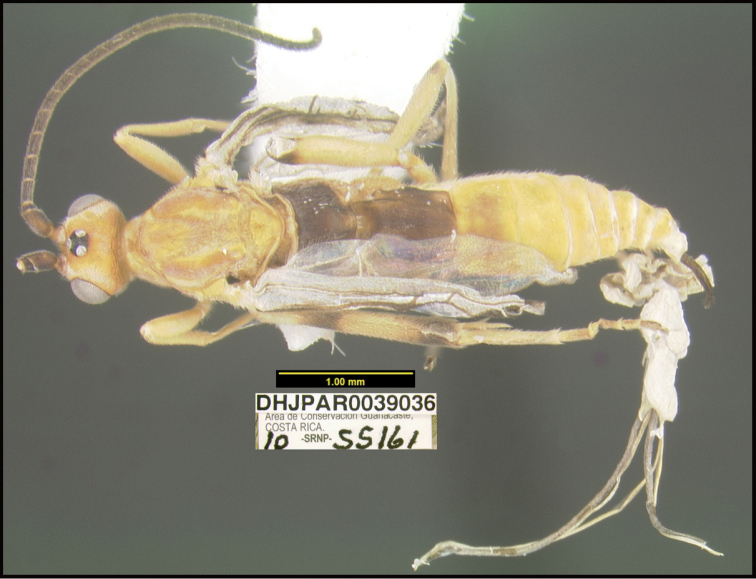
*Orgilusdianalipscombae*, holotype.

##### Orgilus ebbenielsoni

Taxon classificationAnimaliaHymenopteraBraconidae

Sharkey sp. nov.

http://zoobank.org/F186FF66-6CA6-4FE2-BA9D-9866A38E8333

Figure 324

###### Diagnostics.

BOLD:ABA8537. Consensus barcode. AATTTTATATTTTTTATTTGGAATTTGATCAGGAATTTTAGGAATATCAATAAGTTTAATTATTCGTATAGAATTGGGGATACCAGGGAGATTAATTGGTAATGATCAAATTTATAATAGTGTTGTAACAGCTCATGCATTTATTATAATTTTTTTTATAGTTATACCTATTATAATTGGTGGGTTTGGAAATTGATTGATTCCTATAATATTAGGATGTCCTGATATAGCTTTCCCTCGAATAAATAATATAAGGTTTTGATTGTTGATTCCATCTATAATTTTTTTAATTTTTAGAAGAATTTTAAATGTTGGTGTTGGYACAGGGTGAACTGTATATCCACCTTTATCTTTAACAATTGGTCATGGGGGGGTATCTGTTGATATAGCTATTTTTTCTTTACATTTAGCTGGTATTTCTTCTATTATAGGAGCAATTAATTTTATTACTACAATTTTAAATATACGTTCTGATAAAGTTTTAATAGATAAAATTTCTTTATTAAGTTGATCTGTATTAATTACAGCAATTTTATTATTATTATCATTACCTGTATTAGCAGGGGCAATTACAATATTATTAACAGATCGTAATTTAAATACATCTTTTTTTGATCCTTCAGGAGGGGGGGATCCTATTTTATATCAACATTTATTT.

###### Holotype ♀.

Guanacaste, Sector Pitilla, Medrano, 11.01602, -85.38053, 380 meters, caterpillar collection date: 17/iii/2012, wasp eclosion date: 3/iv/2012. Depository: CNC.

***Host data*.** Gregarious parasitoid on *Megalotacrassana* (Tortricidae) feeding on *Crotonbillbergianus* (Euphorbiaceae). Seven specimens emerged from the host caterpillar.

***Caterpillar and holotype voucher codes*.** 12-SRNP-70637, DHJPAR0049088.

###### Paratypes.

Hosts = *Megalotacrassana*, *Megalota* Janzen03, *Megalotaochreoapex*. DHJPAR0038979, DHJPAR0043266, DHJPAR0049086. Depository: CNC.

###### Etymology.

*Orgilusebbenielsoni* is named in honor of Ebbe Nielson (RIP) attending the international NSF-funded planning meeting for the All Taxa Biodiversity Inventory (ATBI) of Terrestrial Systems, and contributing his wisdom to the planning that was the founding of Costa Rica’s national BioAlfa today.

###### Note.

This is the first and only record of a gregarious species of *Orgilus*.

**Figure 324.**
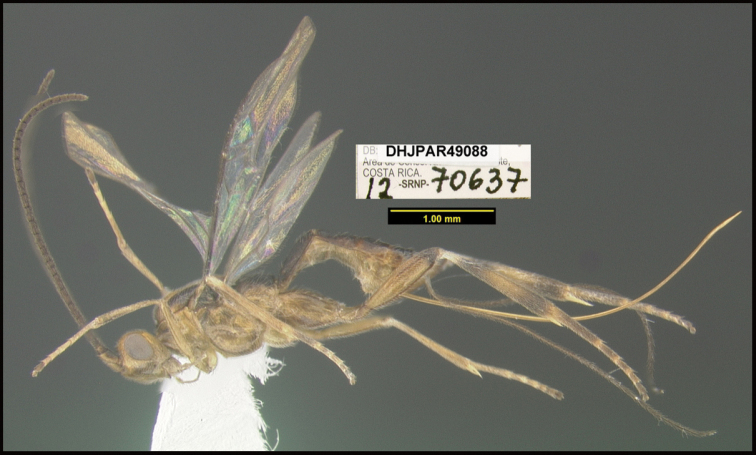
*Orgilusebbenielsoni*, holotype.

##### Orgilus elizabethpennisiae

Taxon classificationAnimaliaHymenopteraBraconidae

Sharkey sp. nov.

http://zoobank.org/F7FE9433-5B4B-42E3-BFD3-C8393EF762AB

Figure 325

###### Diagnostics.

BOLD:AAW8981. Consensus barcode. TATTTTGTATTTTGTATTTGGTATTTGATCAGGTATTTTGGGAATATCAATAAGGTTAATTATTCGAATAGAATTAGGGATACCAGGTAGAATAATYGGAAATGATCAGATTTATAATAGAATTGTTACAGCACATGCATTTATTATAATTTTTTTTATAGTTATGCCAATTATAATTGGTGGGTTTGGAAATTGACTTATTCCAATAATATTAGGATGTCCAGATATAGCGTTTCCACGAATAAATAATATAAGATTTTGATTGTTAATTCCTTCAATAATTTTATTGATTTTTAGAAGAATTTTAAATGTGGGTGTTGGGACAGGTTGGACAGTTTATCCACCTTTATCTTTAATAATTGGTCATGGGGGGGTATCTATTGATATAGCTATTTTTTCTTTACATTTAGCAGGAATTTCTTCTATTATGGGGTCAATTAATTTTATTACTACAATTTTAAATATGCGTTCTGATAAAATTTTAATAGATAAAATTTCATTATTAAGATGATCTATTTTAATTACAGTAATCTTATTGTTGTTATCGTTACCTGTATTAGCAGGTGCTATTACAATGTTATTGACTGATCGTAATTTAAATACATCTTTTTTTGATCCTTCAGGTGGGGGGGAYCCTATTTTATATCAACATTTATTT.

###### Holotype ♂.

Alajuela, Sector San Cristobal, Sendero Huerta, 10.93, -85.372, 527 meters, 18/iv/2012, Malaise trap. Depository: CNC.

***Host data*.** None.

***Holotype voucher code*.**DHJPAR0049293.

###### Paratypes.

Both Malaise – trapped. DHJPAR0051816, DHJPAR0049469. Depository: CNC.

###### Etymology.

*Orgiluselizabethpennisiae* is named in honor of Elizabeth Pennisi’s long-appreciated contributions to publicity for ACG, GDFCF, and now, BioAlfa.

**Figure 325.**
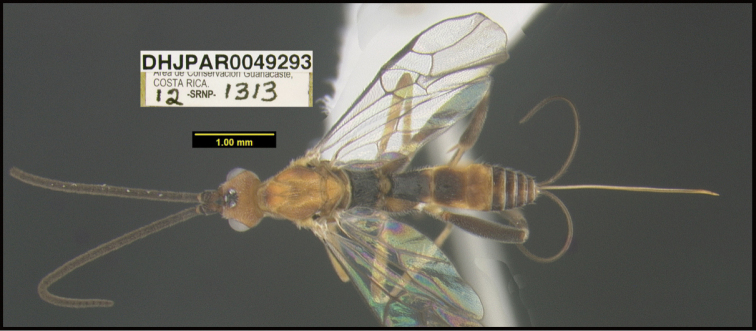
*Orgiluselizabethpennisiae*, holotype.

##### Orgilus evertlindquisti

Taxon classificationAnimaliaHymenopteraBraconidae

Sharkey sp. nov.

http://zoobank.org/3772DDFF-ADFE-4DAB-8EB9-32E225CD3BC5

Figure 326

###### Diagnostics.

BOLD:AAI2109. Consensus barcode. TTTTTATTTGGAATTTGATCGGGAATTTTGGGTATATCTATAAGATTAATTATTCGTATAGAGTTAGGTATACCTGGAAGATTGATTGGGAATGATCAAATTTATAATAGAATTGTTACAGCTCATGCATTTATTATAATTTTTTTTATAGTTATACCTATTATAATTGGTGGTTTTGGAAATTGATTAATTCCAATAATATTAGGATGTCCGGATATAGCTTTTCCACGTATAAATAATATAAGATTTTGATTATTAATTCCTTCAATAACTTTTTTAATTTTTAGRGGGGTTTTAAATGTTGGAGTGGGAACAGGTTGAACAGTTTATCCTCCATTATCATTAATTATTGGTCATGGTGGGTTATCTGTTGATATAGCAATTTTTTCTTTACATTTAGCTGGAATTTCTTCAATTATAGGGGCAATTAATTTTATTACTACAGTTTTAAATATACGATCTGATAAAGTTTATATAGATAAAATTTCTTTATTGAGTTGATCTATTTTGATTACAGCTATTTTGTTATTATTATCTTTACCGGTTTTAGCTGGGGCTATTACTATATTATTGACTGATCGTAATTTGAATACATCATTTTTTGATCCTTCAGGAGGGGGAGATCCTATTTTATATCAACATTTATTT.

###### Holotype ♀.

Alajuela, Sector San Cristobal, Potrero Argentina, 10.89021, -85.38803, 520 meters, 24/i/2008, Malaise trap. Depository: CNC.

***Host data*.** None.

***Holotype voucher code*.**DHJPAR0027553.

###### Paratypes.

Both Malaise-trapped. DHJPAR0027581, BIOUG28185-A09. Depository: CNC.

###### Etymology.

*Orgilusevertlindquisti* is named in honor of Evert Lindquist attending the international NSF-funded planning meeting for the All Taxa Biodiversity Inventory (ATBI) of Terrestrial Systems, and contributing his wisdom to the planning that was the founding of Costa Rica’s national BioAlfa today.

**Figure 326.**
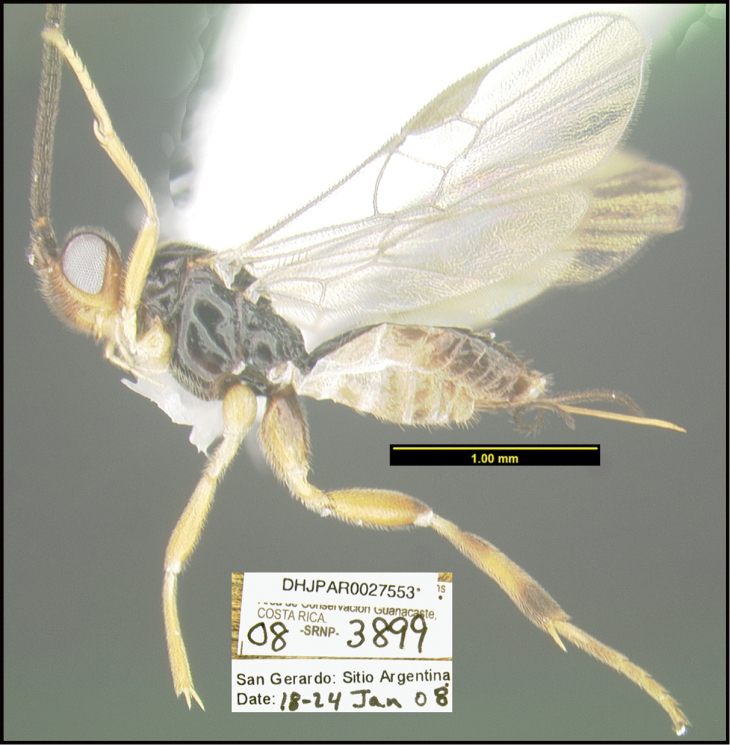
*Orgilusevertlindquisti*, holotype.

##### Orgilus genestoermeri

Taxon classificationAnimaliaHymenopteraBraconidae

Sharkey sp. nov.

http://zoobank.org/3209AE3E-F72F-4224-9712-CCB34FF4D87F

Figure 327

###### Diagnostics.

BOLD:ADH3907. Consensus barcode. TATTTTTGGAATTTGGTCAGGGATTTTAGGAATATCAATAAGATTACTTATTCGATTAGAATTAGGTATGCCAGGTAGATTAATTGGTAATGATCAAATTTATAATAGAGTCGTAACAGCTCATGCATTTTTAATAATTTTTTTTATAGTTATACCAATTATAATTGGAGGATTTGGAAATTGATTAATTCCTATAATATTAGGGTGCCCAGATATAGCATTTCCACGAATAAATAATATAAGATTTTGATTATTAATTCCTTCATTAATTTTGTTAATTTTTAGAGGAATTTTAAATATTGGAGTAGGGACTGGGTGAACAGTTTATCCTCCTTTATCATTAAATATTGGTCATGGGGGTATATCAGTAGATATAGCAATTTTTTCTTTACATTTGGCAGGAATTTCATCAATTATAGGAGCAATTAATTTTATTACAACGATTTTAAATATACGATCTAAAGGAGTAAGAATAGATAAAATTTCATTATTTAGATGGTCAGTGTTAATTACTGCTGTATTATTATTATTATCTTTACCAGTT.

###### Holotype ♀.

Guanacaste, Sector Cacao, Derrumbe, 10.9292, -85.4643, 1220 meters, 2/iv/2015, Malaise trap. Depository: CNC.

***Host data*.** None.

***Holotype voucher code*.**BIOUG33118-F04.

###### Paratypes.

None.

###### Etymology.

*Orgilusgenestoermeri* is named in honor of Gene Stoermer attending the international NSF-funded planning meeting for the All Taxa Biodiversity Inventory (ATBI) of Terrestrial Systems, and contributing his wisdom to the planning that was the founding of Costa Rica’s national BioAlfa today.

**Figure 327.**
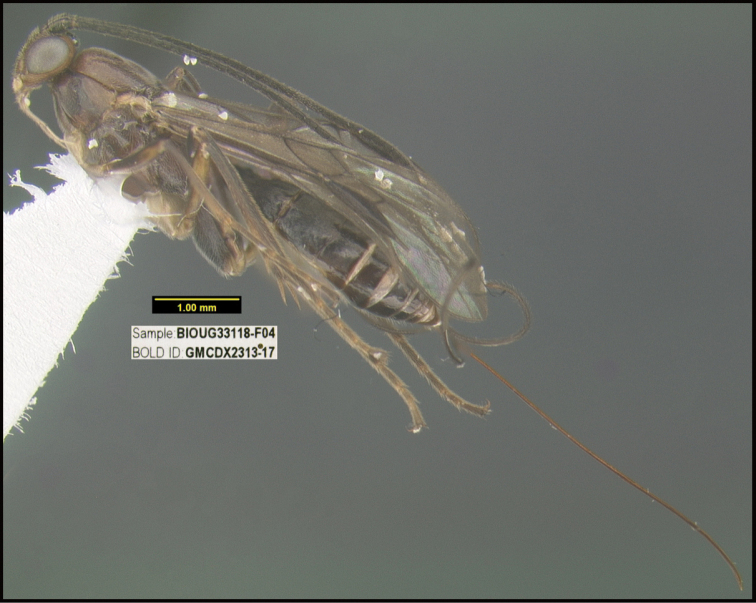
*Orgilusgenestoermeri*, holotype.

##### Orgilus jamesriegeri

Taxon classificationAnimaliaHymenopteraBraconidae

Sharkey sp. nov.

http://zoobank.org/B2E010C0-6561-46DE-9259-91B92E924EA7

Figure 328

###### Diagnostics.

BOLD:ACM9544. Consensus barcode. AATTTTATATTTTTTATTTGGTATTTGATCTGGAATTTTAGGAATATCTATAAGTTTATTAATTCGTGTAGAATTAGGTATACCTGGAAGATTAATTGGTAATGATCAAATTTATAATAGTATTGTAACAGCACATGCTTTTATTATAATTTTTTTTATAGTTATACCTATTATAATTGGTGGATTTGGTAATTGATTAATTCCTATAATATTGGGGTGTCCAGATATAGCTTTCCCTCGTATAAATAATATAAGTTTTTGATTATTGATTCCTTCTTTAATTTTTTTAATTTTTAGTAGTGTTTTAAATGTTGGAGTAGGAACTGGATGAACAGTTTATCCTCCTTTATCTTTAACTATTGGTCATGGGGGATTATCAGTTGATATGGCTATTTTTTCTTTACATTTGGCAGGTATTTCTTCAATTATAGGGGCTATTAATTTTATTACAACTATTTTGAATATACGTTCTGATAAAGTTTTAATAGATAAAATTTCTTTATTAAGATGATCTATTTTAATTACTGCTATTTTATTATTATTATCTTTACCAGTTTTAGCTGGAGCAATTACAATATTATTAACAGATCGTAATTTAAATACATCTTTTTTTGATCCTTCTGGTGGAGGGGATCCAATTTTATATCAGCATTTATTT.

###### Holotype ♀.

Alajuela, Sector Rincon Rain Forest, Comejen, 10.95669, -85.28933, 213 meters, caterpillar collection date: 7/ii/2014, wasp eclosion date: 19/ii/2014. Depository: CNC.

***Host data*.***Brenthia* Janzen13 (Choreutidae) feeding on *Heliconiairrasa* (Heliconiaceae).

***Caterpillar and holotype voucher codes*.** 14-SRNP-45281, DHJPAR0055301.

###### Paratypes.

None.

###### Etymology.

*Orgilusjamesriegeri* is named in honor of James Rieger attending the international NSF-funded planning meeting for the All Taxa Biodiversity Inventory (ATBI) of Terrestrial Systems, and contributing his wisdom to the planning that was the founding of Costa Rica’s national BioAlfa today.

**Figure 328.**
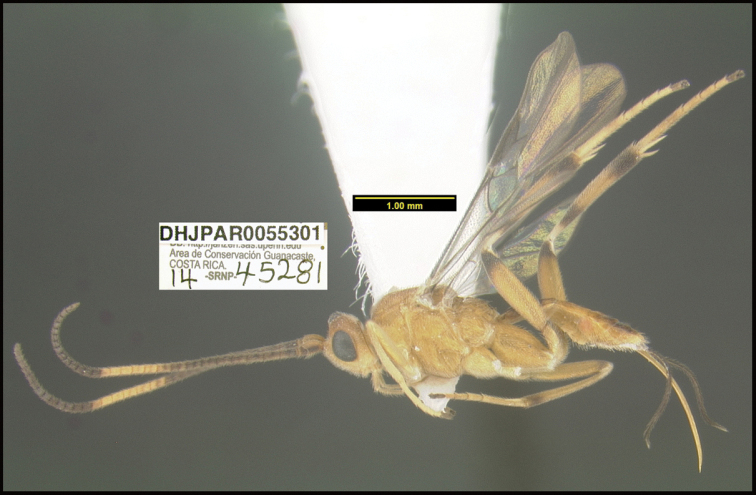
*Orgilusjamesriegeri*, holotype.

##### Orgilus jeanmillerae

Taxon classificationAnimaliaHymenopteraBraconidae

Sharkey sp. nov.

http://zoobank.org/40D2BA84-029F-4D1D-945A-9CC1227E6BAC

Figure 329

###### Diagnostics.

BOLD:ADA6087. Consensus barcode. GTTTTATATTTTATATTTGGAATTTGATCAGGAATTTTAGGTATATCTATAAGTTTAATTATTCGTTTAGAATTAGGTATATTAGGTAGATTGATTGGTAATGATCAAATTTATAATAGAGTAGTTACAGCTCATGCGTTTATTATAATTTTTTTTATAGTTATACCAATTATAATTGGAGGTTTTGGAAATTGATTAATTCCAATGATATTAGGATGTCCAGATATAGCTTTTCCTCGTATAAATAATATAAGATTTTGATTATTAGTTCCTTCTTTATTTTTTTTAATTTTTAGTAGAATTTTAAATATTGGTGTTGGGACTGGATGAACAGTTTATCCACCTTTATCTTTAAATATTGGTCATGGAGGATTATCAGTTGATATATCAATTTTTTCTTTACATTTAGCTGGTATTTCATCTATTATAGGAGCTATTAATTTTATTACTACAATTATTAATATACGATCAAGAATAATTTATATAGATAAAATTTCTTTATTAAGATGATCTATTTTGATTACAGCAATTTTATTATTATTATCTTTGCCTGTTTTGGCTGGTGCAATTACTATATTATTGACTGATCGT.

###### Holotype ♀.

Guanacaste, Sector Pailas Dos, PL12-3, 10.7631, -85.3344, 820 meters, 26/vi/2014, Malaise trap. Depository: CNC.

***Host data*.** None.

***Holotype voucher code*.**BIOUG29912-B11.

###### Paratypes.

BIOUG29685-A09, BIOUG28777-A07, BIOUG28807-F04, BIOUG44866-F09. Depository: CNC.

###### Etymology.

*Orgilusjeanmillerae* is named in honor of Jean Miller’s long-appreciated contributions to publicity for ACG, GDFCF, and now, BioAlfa.

**Figure 329.**
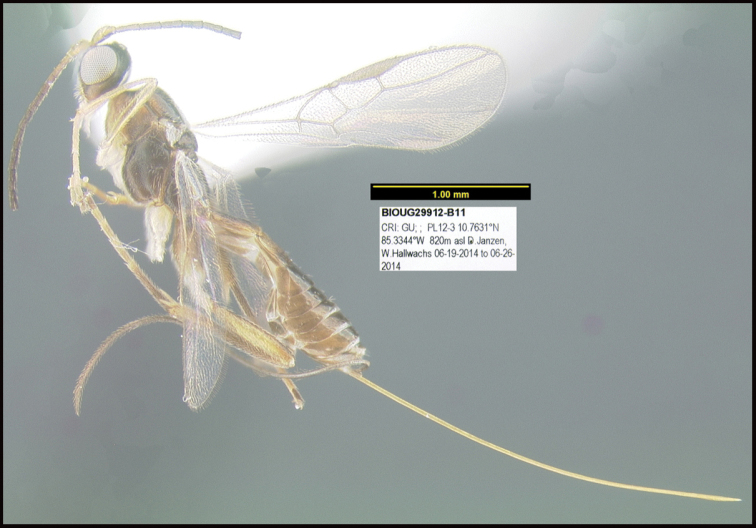
*Orgilusjeanmillerae*, holotype.

##### Orgilus jeffmilleri

Taxon classificationAnimaliaHymenopteraBraconidae

Sharkey sp. nov.

http://zoobank.org/8A690C3A-9CD1-452E-B14E-51C6C45BF6A2

Figure 330

###### Diagnostics.

BOLD:ACT7418. Consensus barcode. TTTATTTTTGGTATTTGATCAGGAATTTTAGGTATGTCAATAAGATTGATTATTCGATTAGAATTAGGTATATCTGGAAGATTAATTGGTAATGATCAAATTTATAATAGAATTGTTACTGCACATGCATTTTTAATAATTTTTTTTATAGTAATGCCTATTATAATTGGAGGGTTTGGTAATTGATTAATTCCTATAATATTAGGATGTCCAGATATAGCATTCCCACGTATAAATAATATAAGATTTTGATTATTAATTCCTTCATTGATTTTTTTAATATTTAGTGGTATTTTAAATATTGGAGTAGGAACAGGTTGAACAGTTTATCCTCCTTTATCTTTAAGAATTGGTCATGGTGGGATATCTGTAGATATAGCAATTTTTTCTTTACATTTAGCTGGGATTTCATCAATTATAGGAGCAGTTAATTTTATTACAACAATTTTAAATATACGATCTGAAGGAGTTACTATAGATAAAATTTCATTATTAAGATGATCAGTATTAATTACAGCTGTATTATTATTATTATCTTTACCAGTTTTAGCTGGAGCTATTACAATATTATTAACTGAT------------------------------------------------------------.

###### Holotype ♀.

Guanacaste, Sector San Cristobal, Estación San Gerardo, 10.88, -85.389, 575 meters, 26/xiii/2013, Malaise trap. Depository: CNC.

***Host data*.** None.

***Holotype voucher code*.**BIOUG19395-H01.

###### Paratypes.

None.

###### Etymology.

*Orgilusjeffmilleri* is named in honor of Jeff Miller’s long-appreciated contributions to publicity for ACG, GDFCF, and now, BioAlfa.

**Figure 330.**
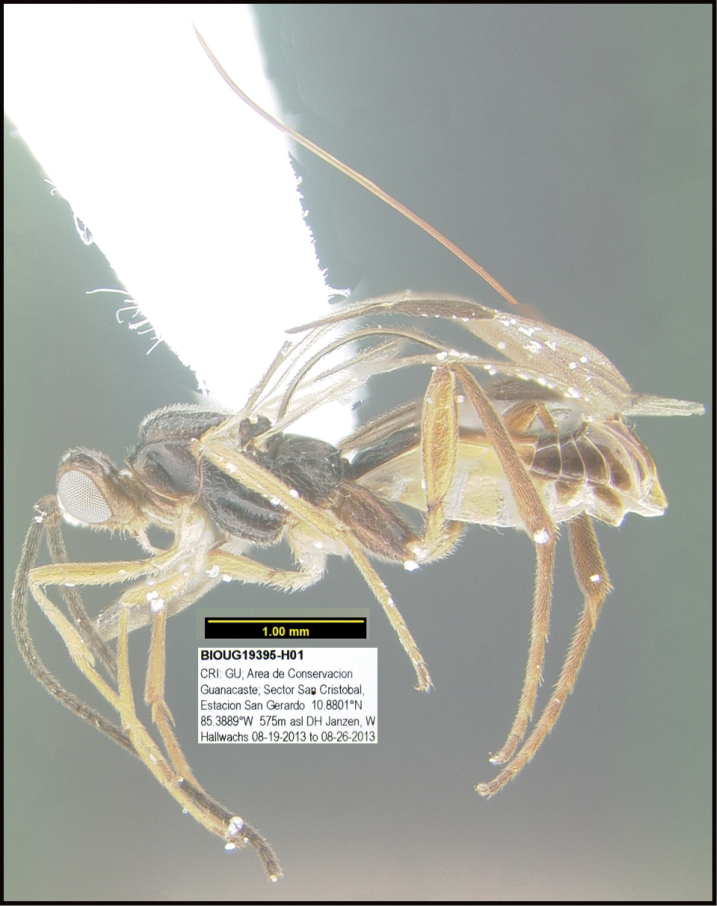
*Orgilusjeffmilleri*, holotype.

##### Orgilus jerrypowelli

Taxon classificationAnimaliaHymenopteraBraconidae

Sharkey sp. nov.

http://zoobank.org/55A34522-0246-4DC5-A8E4-F44F6D8ADCCF

Figure 331

###### Diagnostics.

BOLD:ABZ9478. Consensus barcode. AATTTTATATTTTTTGTTTGGAATTTGATCAGGAATTTTAGGTATATCAATAAGATTAATTATTCGAATGGARTTAGGGATACCGGGYAGRTTAATYGGTAATGATCAAATTTATAATAGAATTGTTACAGCTCATGCTTTTATTATAATTTTTTTTATAGTTATACCTATTATAATTGGTGGATTTGGTAATTGATTAATTCCTATAATATTAGGRTGTCCTGATATAGCTTTYCCTCGAATAAATAATATAAGATTTTGATTATTAATTCCTTCRATAATTTTTTTAATTTTTAGGAGAGTGTTAAATGTTGGTGTTGGAACGGGATGAACTGTTTATCCTCCTYTATCTTTAAYTATTGGTCATGGAGGTTTATCTGTTGATATAGCTATTTTTTCTTTACATTTRGCTGGAATTTCATCTATTATAGGAGCAATTAATTTTATTACAACAATTTTAAATATACGTTCTGAYAAAGTTTTTATAGATAAAATTTCTTTATTAAGTTGATCTGTTTTWATTACAGCTATTTTATTATTATTATCTTTACCAGTATTAGCTGGGGCAATTACAATATTATTAACTGATCGAAATTTAAATACTTCMTTTTTTGATCCGTCAGGTGGAGGTGATCCTATTTTATATCAACATTTATTT.

###### Holotype ♀.

Guanacaste, Sector San Cristobal, Tajo Angeles, 10.86472, -85.41531, 540 meters, caterpillar collection date: 2/ix/2011, wasp eclosion date: 21/ix/2011. Depository: CNC.

***Host data*.***Antaeotricha* Janzen412 (Depressariidae) feeding on *Semialariummexicanum* (Celastraceae).

***Caterpillar and holotype voucher codes*.** 11-SRNP-3427, DHJPAR0050158.

###### Paratypes.

Hosts = *Antaeotrichamarmorea*, *Antaeotricha* Janzen88, *Antaeotricha* Janzen412. DHJPAR0048258, DHJPAR0048266, DHJPAR0048271, DHJPAR0048277, DHJPAR0051415, DHJPAR0048251, DHJPAR0048267, DHJPAR0048268, DHJPAR0055593, DHJPAR0053161. Depository: CNC.

###### Etymology.

*Orgilusjerrypowelli* is named in honor of Jerry Powell participating in the international NSF-funded planning meeting for the All Taxa Biodiversity Inventory (ATBI) of Terrestrial Systems, and contributing his wisdom to the planning that was the founding of Costa Rica’s national BioAlfa today.

**Figure 331.**
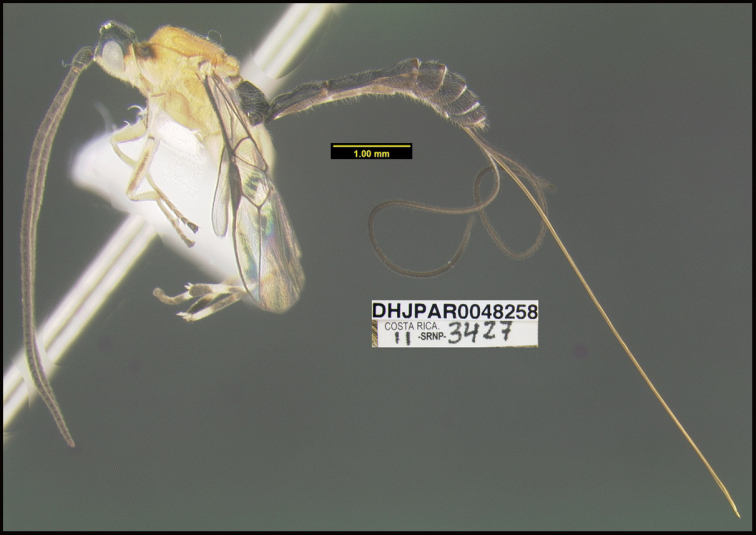
*Orgilusjerrypowelli*, holotype.

##### Orgilus jimtiedjei

Taxon classificationAnimaliaHymenopteraBraconidae

Sharkey sp. nov.

http://zoobank.org/4F589054-3990-4242-A7D8-FE8778592D4C

Figure 332

###### Diagnostics.

BOLD:ADX8196. Consensus barcode. TTTATATTTTATGTTTGGTATTTGATCAGGTATTTTAGGGATATCAATAAGTTTAATTATTCGAATAGAATTAGGTATACCAGGAAGTTTGATTGGTAATGATCAAATTTATAACAGAATTGTAACAGCTCATGCTTTTATTATAATTTTTTTTATAGTAATGCCAATTATAATTGGAGGATTTGGAAATTGATTAATTCCTATAATATTAGGATGTCCGGATATGGCATTTCCACGTATAAATAATATAAGATTTTGATTATTAATTCCTTCTATAATTTTTTTAATTTTTAGAAGAATTTTAAATGTTGGAGTTGGGACTGGATGAACAGTTTATCCACCTTTATCTTTAACTATTGGTCATGGAGGTTTATCTGTTGATATAGTTATTTTTTCTTTACATTTAGCAGGAATTTCTTCTATTATAGGGGCAATTAATTTTATTACAACAATTTTAAATATACGATCTGATAAAGTTTTAATAGATAAAATTTCATTATTAAGGTGATCTGTTTTAATTACAGCAATTTTGTTATTGTTATCATTACCTGTATTAGCAGGTGCTATTACAATATTATTGACTGATCGTAATTTAAATACATCTTTTTTTGATCCTTCAGGTGGAGGTGACCCAATTTTATACCAACATTTATT------------------------------------------.

###### Holotype ♀.

Guanacaste, Sector Pailas Dos, PL12-3, 10.7631, -85.3344, 820 meters, 23/iv/2015, Malaise trap. Depository: CNC.

***Host data*.** None.

***Holotype voucher code*.**BIOUG44165-G01.

###### Paratypes.

None.

###### Etymology.

*Orgilusjimtiedjei* is named in honor of Jim Tiedje attending the international NSF-funded planning meeting for the All Taxa Biodiversity Inventory (ATBI) of Terrestrial Systems, and contributing his wisdom to the planning that was the founding of Costa Rica’s national BioAlfa today.

**Figure 332.**
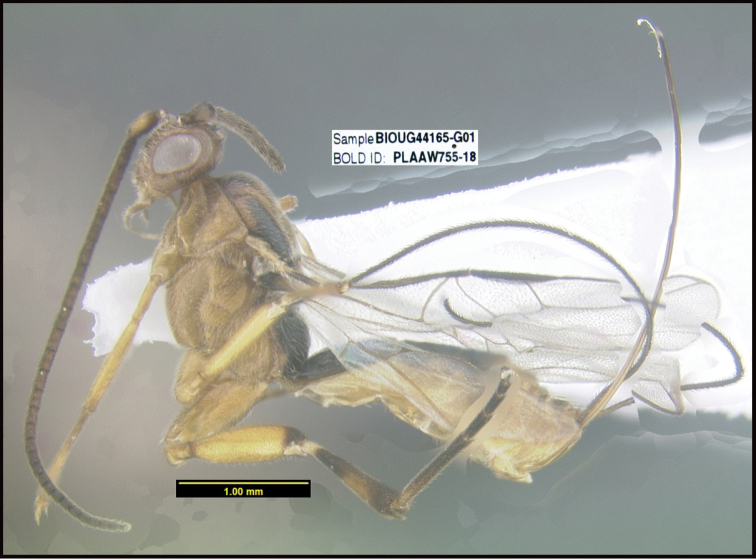
*Orgilusjimtiedjei*, holotype.

##### Orgilus johnlundbergi

Taxon classificationAnimaliaHymenopteraBraconidae

Sharkey sp. nov.

http://zoobank.org/135C404A-B83B-43ED-876C-CB952F12BDF8

Figure 333

###### Diagnostics.

BOLD:AAT9015. Consensus barcode. TATTTTATATTTTTTATTTGGAATTTGATCTGGRATTTTAGGAATATCTATAAGATTAATTATTCGATTAGAATTAGGTATACCTGGGAGTTTAATTGGTAATGATCAAATTTATAATAGAATTGTTACTGCTCATGCTTTTATTATAATTTTTTTTATAGTTATACCAATTATAATTGGGGGATTTGGAAATTGATTAATTCCAATAATATTAGGATGTCCTGATATGGCTTTCCCTCGGATAAATAATATAAGTTTTTGATTGTTAATTCCTTCAATAATTTTTTTAATTTTTAGAAGGGTTTTAAATGTTGGTGTAGGAACTGGTTGAACTGTRTATCCTCCTTTATCATTAATTATTGGTCATGGGGGGGTATCTGTTGATATARCTATTTTTTCTTTACATTTAGCAGGAATTTCTTCAATTATAGGAGCAATTAATTTTATTACAACAATTTTAAATATACGATCTGATAAAGTTTTAATGGATAAAATTTCTTTATTAAGATGATCAGTTTTAATTACAGCAATTTTATTATTATTATCTTTACCTGTTTTAGCAGGAGCTATTACTATATTATTAACAGATCGTAATTTAAATACATCTTTTTTTGAYCCTTCAGGAGGAGGTGATCCTATTTTATATCAGCATTTATTT.

###### Holotype ♀.

Alajuela, Sector Rincon Rain Forest, Rio Francia Arriba, 10.89666, -85.29003, 400 meters, caterpillar collection date: 14/v/2011, wasp eclosion date: 29/v/2011. Depository: CNC.

***Host data*.** elachBioLep01 BioLep81 (Depressariidae) feeding on *Conceveibapleiostemona* (Euphorbiaceae).

***Caterpillar and holotype voucher codes*.** 11-SRNP-42377, DHJPAR0042873.

###### Paratypes.

Hosts = elachBioLep01 BioLep81, elachBioLep01 Janzen48, elachJanzen01 Janzen698 (Depressaridae). DHJPAR0061511, DHJPAR0040026, DHJPAR0040028, DHJPAR0040029, DHJPAR0049918, DHJPAR0050141, DHJPAR0050150, DHJPAR0049671, DHJPAR0049679, DHJPAR0054529, DHJPAR0040553. Depository: CNC.

###### Etymology.

*Orgilusjohnlundbergi* is named in honor of John Lundberg attending the international NSF-funded planning meeting for the All Taxa Biodiversity Inventory (ATBI) of Terrestrial Systems, and contributing his wisdom to the planning that was the founding of Costa Rica’s national BioAlfa today.

**Figure 333.**
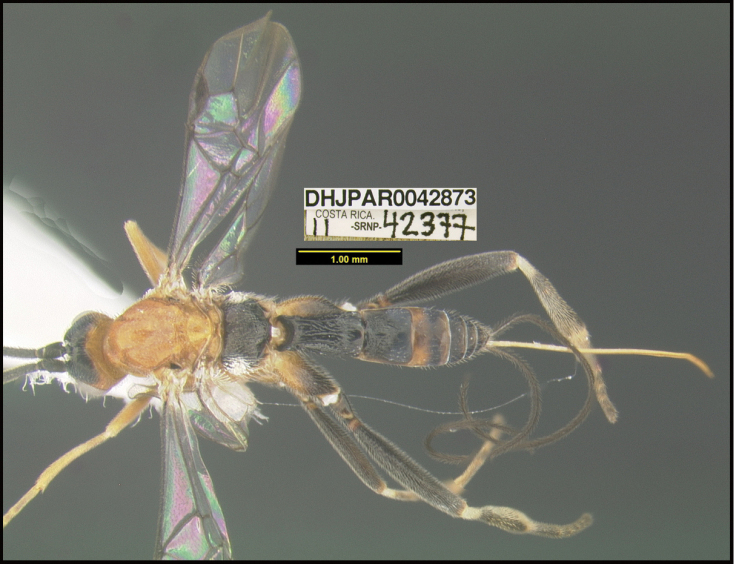
*Orgilusjohnlundbergi*, holotype.

##### Orgilus johnpipolyi

Taxon classificationAnimaliaHymenopteraBraconidae

Sharkey sp. nov.

http://zoobank.org/CCB11238-183D-4467-8011-B46B4A90B3AF

Figure 334

###### Diagnostics.

BOLD:ABU7457. Consensus barcode. TATTTTGTATTTTATATTTGGTATTTGATCTGGGGTATTAGGTATATCAATGAGATTTGTAGTTCGGATAGAATTAGGTATACCTGGGAGTTTAATTGGTAACGATCAAATTTATAATAGAATTGTTACTGCTCATGCTTTTTTAATAATTTTTTTTATAGTAATACCTATTATAATTGGAGGATTTGGGAATTGATTAATTCCTGTAATATTAGGATGTCCTGATATAGCTTTCCCTCGAATAAATAATATAAGTTTTTGGTTATTAATTCCTTCAATTTTATTTTTAATTTTTAGGGGAATTTTAAATATTGGTGTAGGGACTGGGTGAACCGTTTATCCTCCTTTATCTTTGAATATTGGTCATGGAGGGTTATCAGTTGATATAGCTATTTTTTCTTTACATTTAGCGGGGGCGTCATCTATTATGGGAGCTATTAATTTTATTACGACAATTATAAATATACGATCTAGAATGGTTTTTATAGATAAAATTTCCTTGTTATGTTGATCAGTATTAATTACTGCTGTTTTATTATTATTATCTTTACCAGTATTAGCTGGGGCTATTACTATATTATTAACTGATCGTAATATAAATACTTCTTTTTTTGATCCTTCCGGGGGTGGTGATCCAATTTTATATCAACATTTATTT.

###### Holotype ♂.

Alajuela, Sector Rincon Rain Forest, Sendero Anonas, 10.90528, -85.27882, 405 meters, caterpillar collection date: 9/iii/2012, wasp eclosion date: 10/iv/2012. Depository: CNC.

***Host data*.***Anadasmus* Janzen25 (Depressariidae) feeding on *Ocoteaatirrensis* (Lauraceae).

***Caterpillar and holotype voucher codes*.** 12-SRNP-41056, DHJPAR0048772.

###### Paratypes.

None.

###### Etymology.

*Orgilusjohnpipolyi* is named in honor of John Pipoly attending the international NSF-funded planning meeting for the All Taxa Biodiversity Inventory (ATBI) of Terrestrial Systems, and contributing his wisdom to the planning that was the founding of Costa Rica’s national BioAlfa today.

**Figure 334.**
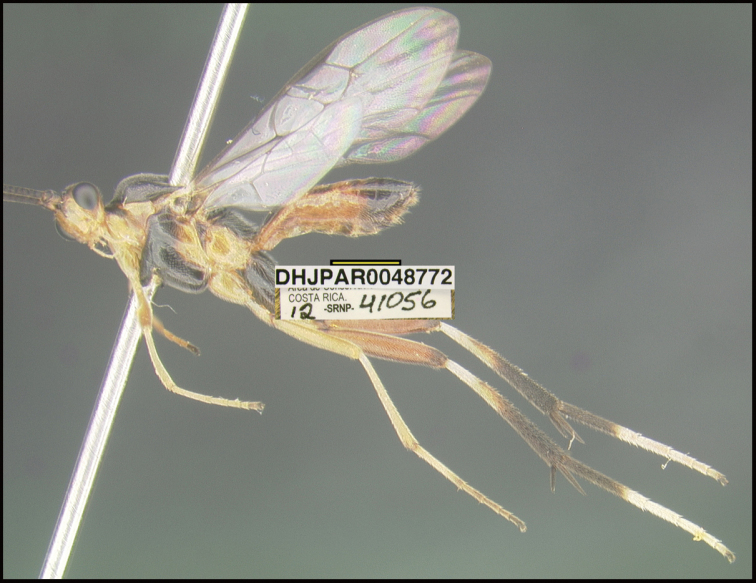
*Orgilusjohnpipolyi*, holotype.

##### Orgilus jorgellorentei

Taxon classificationAnimaliaHymenopteraBraconidae

Sharkey sp. nov.

http://zoobank.org/EC5ADD45-F133-4A3C-97C7-C654BE6DA98D

Figure 335

###### Diagnostics.

BOLD:AAN2501. Consensus barcode. AATTTTATATTTTTTATTTGGAATTTGATCAGGTATTTTAGGAATATCGATAAGATTAATTATTCGAATAGAATTAGGTATACCAGGAAGATTAATTGGTAATGATCAAATTTATAATAGAATTGTTACAGCTCATGCTTTTATTATAATTTTTTTTATAGTTATACCTATTATAATTGGGGGATTTGGAAATTGGTTAATTCCTATAATATTAGGATGTCCTGATATAGCTTTCCCTCGTATAAATAATATAAGTTTTTGGTTATTAATTCCTTCAATAATTTTTTTAATTTTTAGAAGAGTATTAAATGTTGGAGTAGGAACAGGATGAACTGTTTATCCTCCTTTATCATTAGGTATTGGTCATGGAGGATTATCTGTTGATATAGCAATTTTTTCTTTACATTTAGCAGGAATTTCTTCAATTATAGGAGCAATTAATTTTATTACAACAATTTTAAATATACGATCTGATAAAGTTTTAATAGATAAAATTTCTTTATTAAGGTGATCGGTTTTAATTACAGCAATTTTATTATTATTATCTTTACCTGTTTTAGCTGGGGCAATTACTATATTATTAACTGATCGTAATTTAAATACATCATTTTTTGATCCCTCAGGAGGTGGAGATCCTATTTTATATCAACATTTATTT.

###### Holotype ♀.

Alajuela, Sector San Cristobal, Puente Palma, 10.9163, -85.37869, 460 meters, caterpillar collection date: 21/v/2014, wasp eclosion date: 13/vi/2014. Depository: CNC.

***Host data*.***Antaeotricharadicalis* (Depressariidae) feeding on *Vochysiaguatemalensis* (Vochysiaceae).

***Caterpillar and holotype voucher codes*.** 14-SRNP-2556, DHJPAR0055612.

###### Paratype.

Host = *Stenoma* Janzen148 (Depressariidae): DHJPAR0039489. Depository: CNC.

###### Etymology.

*Orgilusjorgellorentei* is named in honor of Jorge Llorente attending the international NSF-funded planning meeting for the All Taxa Biodiversity Inventory (ATBI) of Terrestrial Systems, and contributing his wisdom to the planning that was the founding of Costa Rica’s national BioAlfa today.

**Figure 335.**
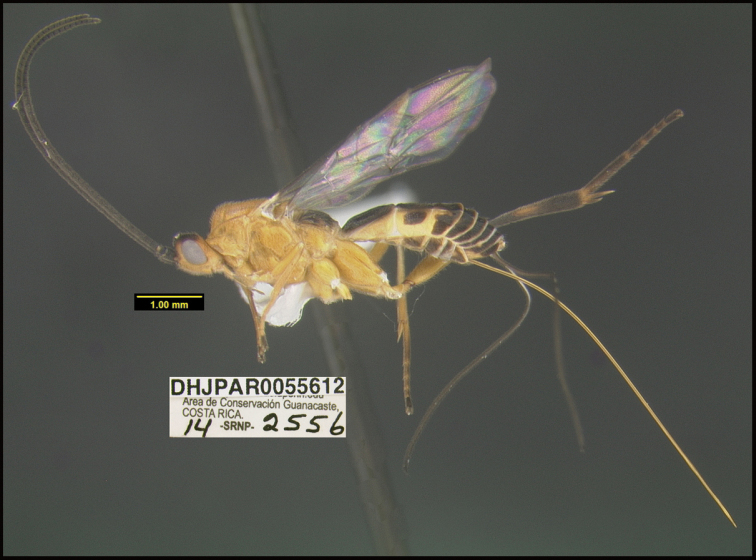
*Orgilusjorgellorentei*, holotype.

##### Orgilus larryspearsi

Taxon classificationAnimaliaHymenopteraBraconidae

Sharkey sp. nov.

http://zoobank.org/C8BE4315-1A22-42F5-80CC-E2150C168B98

Figure 336

###### Diagnostics.

BOLD:ACL4839. Consensus barcode. TATTTTATATTTTATGTTTGGTATTTGATCAGGTATTTTAGGAATATCAATAAGTTTAATTATTCGAATAGAATTAGGTATACCAGGAAGTTTAATTGGTAATGATCAAATTTATAACAGAATTGTAACAGCTCATGCATTTATTATAATTTTTTTTATAGTAATGCCAATTATAATTGGAGGATTTGGAAATTGATTAATTCCTATAATATTAGGATGTCCGGATATGGCATTCCCACGTATAAATAATATAAGATTTTGATTATTAATTCCTTCTATAATTTTTTTAATTTTTAGAAGAATTTTAAATGTTGGGGTTGGAACTGGGTGAACAGTTTATCCACCTTTATCTTTAACTATTGGTCAYGGAGGGTTATCTGTTGATATAGCTATTTTTTCTTTACATCTAGCGGGAATTTCTTCTATTATAGGAGCAATTAATTTTATTACAACAATTTTAAATATACGATCTGATAAAGTTTTAATAGATAAAATTTCATTATTAAGGTGATCTGTTTTAATTACAGCAATTTTATTATTATTATCATTACCTGTATTAGCAGGTGCTATTACAATATTATTGACTGATCGTAATTTAAATACATCTTTTTTTGATCCTTCAGGTGGAGGTGATCCAATTTTATATCAACATTTATTT.

###### Holotype ♀.

Guanacaste, Pailas Dos, PL12-3, 10.7631, -85.3344, 820 meters, 6/ii/2014, Malaise trap. Depository: CNC.

***Host data*.** None.

***Holotype voucher code*.**BIOUG29625-H11.

###### Paratypes.

All Malaise-trapped. BIOUG09739-H07, BIOUG17496-G11, BIOUG18248-D12, BIOUG29423-E02, BIOUG29479-G11, BIOUG29660-B09, BIOUG29724-F11. Depository: CNC.

###### Etymology.

*Orgiluslarryspearsi* is named in honor of Larry Spears attending the international NSF-funded planning meeting for the All Taxa Biodiversity Inventory (ATBI) of Terrestrial Systems, and contributing his wisdom to the planning that was the founding of Costa Rica’s national BioAlfa today.

**Figure 336.**
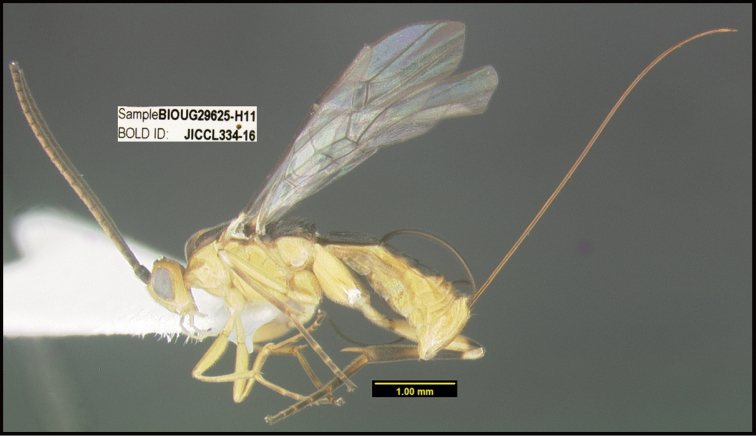
*Orgiluslarryspearsi*, holotype.

##### Orgilus marlinricei

Taxon classificationAnimaliaHymenopteraBraconidae

Sharkey sp. nov.

http://zoobank.org/71DD8DB0-40EA-434A-8367-C3A70AFE8E8C

Figure 337

###### Diagnostics.

BOLD:ACR9910. Consensus barcode. ATTTTATATTTTATATTTGGTATTTGATCAGGTATTTTAGGAATATCAATAAGTTTAATTATTCGAATAGAATTAGGTATACCTGGAAGTTTAATTGGAAATGATCAAATTTATAATAGAATTGTTACAGCTCATGCTTTTATTATAATTTTTTTTATAGTAATACCAATTATAATTGGTGGATTTGGAAATTGATTAATTCCTATAATATTAGGATGCCCAGATATAGCATTTCCACGAATAAATAATATAAGATTTTGATTATTAATTCCTTCAATAACTTTTTTAATTTTTAGAAGAATTTTGAATGTTGGTGTTGGAACTGGATGAACAGTATATCCACCTTTATCTTTAACAATTGGTCACGGAGGTTTATCTGTTGATATAGCTATTTTTTCTTTACATTTAGCTGGAATTTCTTCAATTATAGGGGCAATTAATTTTATTACTACAATTTTAAATATACGATCTGATAAAGTTTTAATAGATAAAATCTCATTATTAAGATGATCTGTATTAATTACAGCAATTTTATTATTATTATCATTACCAGTATTAGCAGGTGCTATTACAATATTATTAACTGAT---------------------.

###### Holotype ♀.

Guanacaste, Sector Santa Rosa, Bosque San Emilio, 10.8438, -85.6138, 300 meters, 28/v/2012, Malaise trap. Depository: CNC.

***Host data*.** None.

***Holotype voucher code*.**BIOUG18748-D07.

###### Paratypes.

None.

###### Etymology.

*Orgilusmarlinricei* is named in honor of Marlin Rice’s long-appreciated contributions to publicity for ACG, GDFCF, and now, BioAlfa.

**Figure 337.**
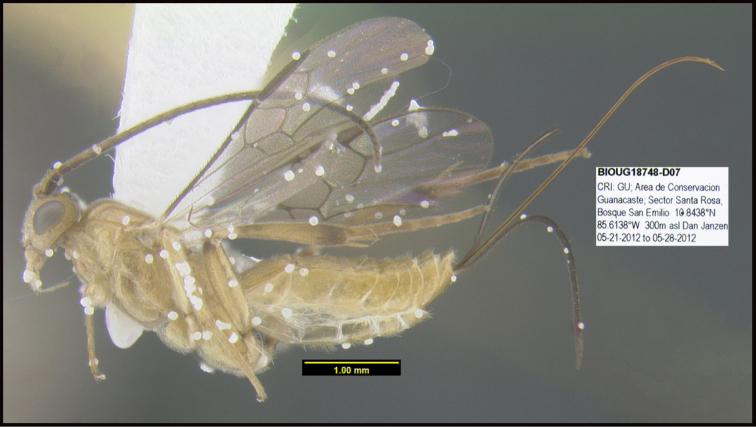
*Orgilusmarlinricei*, holotype.

##### Orgilus mellissaespinozae

Taxon classificationAnimaliaHymenopteraBraconidae

Sharkey sp. nov.

http://zoobank.org/064BF99A-9BCA-4818-8F9E-5CE1AE4698C7

Figure 338

###### Diagnostics.

BOLD:ADG8044. Consensus barcode. TTTTTATTTGGTATTTGATCAGGAATTTTAGGAATATCAATAAGTTTAATTATTCGATTAGAATTAGGTATGCCAGGCAGATTAATTGGTAATGATCAAATTTATAATAGAATTGTTACTGCTCATGCATTTATTATAATTTTTTTTATAGTTATACCTATTATAATTGGGGGATTTGGGAATTGATTAGTACCTATAATATTAGGATGTCCTGATATAGCTTTCCCACGAATAAATAATATAAGATTTTGATTATTAGTACCATCATTAATTTTTTTAATTTTTAGAGGAATTTTAAATATTGGAGTTGGGACAGGATGAACAGTTTATCCCCCTTTATCTTTAAGAATTGGACATGGGGGGGTATCAGTTGATTTAGCAATTTTTTCTTTACATTTAGCTGGTATCTCTTCAATTATAGGAGCTATTAATTTTATTACTACTATTTTAAATATACGTTCAAGAATAGTTTATATAGATAAAATTCCTTTATTTGTTTGATCAGTATTAATTACTGCAATTTTATTATTATTATCTTTACCAGTATTAGCTGGTGCTATTACTATA-------------------------------------------------------.

###### Holotype ♂.

Guanacaste, Sector Cacao, Derrumbe, 10.9292, -85.4643, 1220 meters, 26/ii/2015, Malaise trap. Depository: CNC.

***Host data*.** None.

***Holotype voucher code*.**BIOUG32860-D06.

###### Paratypes.

None.

###### Etymology.

*Orgilusmellissaespinozae* is named in honor of Mellissa Espinoza’s long-appreciated contributions to publicity for ACG, GDFCF, and now, BioAlfa.

**Figure 338.**
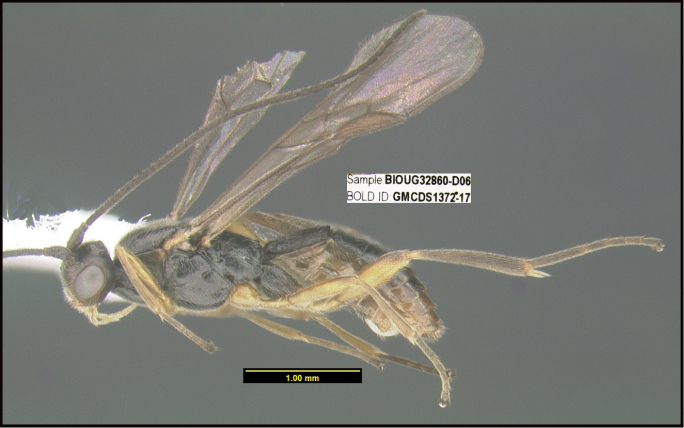
*Orgilusmellissaespinozae*, holotype.

##### Orgilus mikesmithi

Taxon classificationAnimaliaHymenopteraBraconidae

Sharkey sp. nov.

http://zoobank.org/5DE53B0A-D751-4D72-9418-C0127972ED21

Figure 339

###### Diagnostics.

BOLD:ADF7540. Consensus barcode. ATATTATATTTTTTATTTGGGATTTGGGCAGGCATTTTAGGGATATCAATAAGGTTAATTATTCGACTAGAATTGGGAATACCTGGGGGATTAATTGGTAACGATCAAATTTATAATAGAGTTGTTACTGCTCATGCTTTTATTATGATTTTTTTTATGGTTATACCAATTATAATTGGAGGGTTTGGTAATTGGTTGATTCCTTTAATATTAAGATGTCCTGATATAGCTTTCCCTCGAATGAATAATATAAGATTTTGATTATTAATTCCTTCTTTAATATTTTTAATTTTTAGTAGAATCTTAAATATTGGGGTAGGCACAGGGTGAACTGTTTATCCCCCTTTATCTTTATCTATTGGTCATGGAGGGCTTTCTGTTGATATGGCTATTTTTTCTTTACATTTAGCGGGTATTTCTTCAATTATGGGGGCTATTAATTTTATTACTACTATTATAAATATACGATTAAATATAATTTATATAGATAAAGTTTCTTTATTTATTTGGTCAGTTTTAATTACAGCAATTTTATTATTGTTATCTTTACCTGTATTAGCGGGG-------------------------------------------------------------------.

###### Holotype ♂.

Guanacaste, Sector Cacao, Derrumbe, 10.9292, -85.4643, 1220 meters, 18/xii/2014, Malaise trap. Depository: CNC.

***Host data*.** None.

***Holotype voucher code*.**BIOUG31641-F04.

###### Paratypes.

None.

###### Etymology.

*Orgilusmikesmithi* is named in honor of Mike Smith attending the international NSF-funded planning meeting for the All Taxa Biodiversity Inventory (ATBI) of Terrestrial Systems, and contributing his wisdom to the planning that was the founding of Costa Rica’s national BioAlfa today.

**Figure 339.**
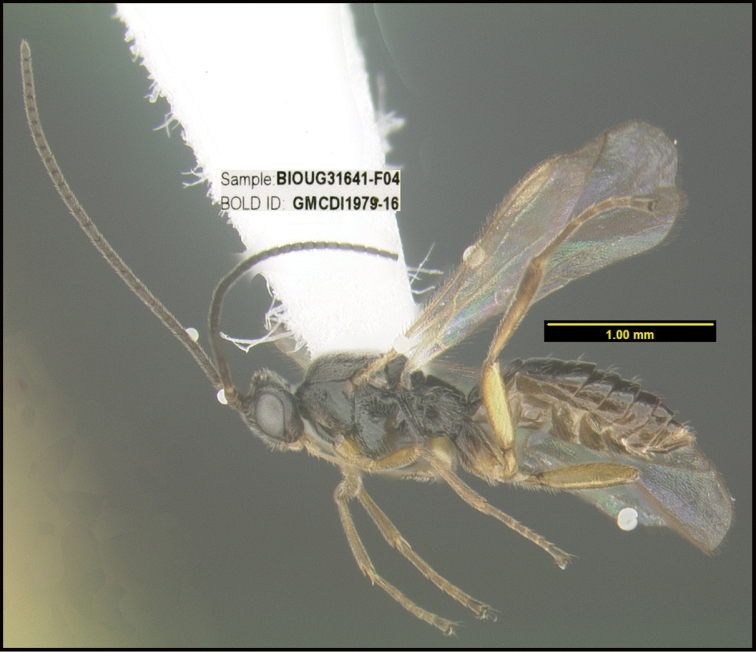
*Orgilusmikesmithi*, holotype.

##### Orgilus normplatnicki

Taxon classificationAnimaliaHymenopteraBraconidae

Sharkey sp. nov.

http://zoobank.org/48CB7122-5D24-49DC-88FE-848090543A39

Figure 340

###### Diagnostics.

BOLD:ABX5811. Consensus barcode. TGTTTTATATTTTATATTTGGTATTTGATCAGGTATTTTAGGAATATCAATAAGGTTAATTATTCGTATAGAATTGGGCATACCAGGTAGGTTAATTGGTAATGATCAAATTTATAATAGAATTGTTACAGCTCATGCATTTATTATAATTTTTTTTATAGTTATACCAATCATAATTGGTGGGTTTGGAAATTGATTAATTCCTATAATATTAGGATGTCCTGATATAGCATTCCCACGAATAAATAATATAAGATTTTGATTATTAATTCCTTCAATAATTTTTTTAATTTTTAGAAGAATTTTAAATGTTGGAGTGGGAACTGGTTGGACAGTATATCCTCCATTATCTTTAACAATTGGTCATGGAGGATTATCTGTTGATATAGCTATTTTTTCTTTACATTTAGCTGGAATTTCTTCTATTATAGGAGCAATTAATTTTATTACTACAATTTTAAATATACGGTCTGAAAAAGTTTTTATAGATAAAATTTCATTATTAAGATGATCAATTTTAATTACAGCAATTTTATTGTTATTATCATTACCTGTTTTAGCAGGTGCTATTACAATATTATTAACTGATCGTAATTTAAATACATCCTTTTTTGATCCTTCAGGTGGCGGCGATCCAATTTTATATCAACATTTATTT.

###### Holotype ♀.

Guanacaste, Sector San Cristobal, Tajo Angeles, 10.86472, -85.41531, 540 meters, caterpillar collection date: 2/ix/2011, wasp eclosion date: 23/ix/2011. Depository: CNC.

***Host data*.** pyrBioLep01 BioLep761 (Pyralidae) feeding on *Semialariummexicanum* (Celastraceae).

***Caterpillar and holotype voucher codes*.** 11-SRNP-3414, DHJPAR0048166.

###### Paratypes.

Host = phyBioLep01 BioLep761: DHJPAR0045298, DHJPAR0048187. Depository: CNC.

###### Etymology.

*Orgilusnormplatnicki* is named in honor of Norm Platnick (RIP) attending the international NSF-funded planning meeting for the All Taxa Biodiversity Inventory (ATBI) of Terrestrial Systems, and contributing his wisdom to the planning that was the founding of Costa Rica’s national BioAlfa today.

**Figure 340.**
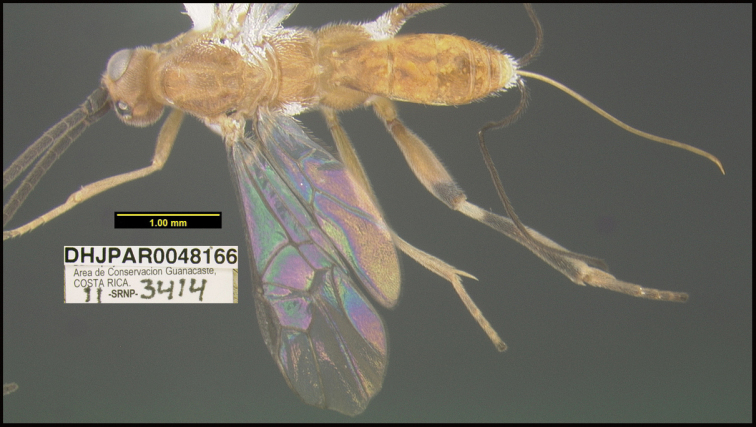
*Orgilusnormplatnicki*, holotype.

##### Orgilus peterrauchi

Taxon classificationAnimaliaHymenopteraBraconidae

Sharkey sp. nov.

http://zoobank.org/E915CC68-B465-4370-A9D2-3481B20092EE

Figure 341

###### Diagnostics.

BOLD:ACJ4626. Consensus barcode. AATTTTATATTTTTTGTTTGGAATTTGATCAGGAATTTTAGGTATGTCAATAAGATTGATTATTCGAATGGAATTAGGTATACCAGGTAGATTAATTGGTAATGATCAAATTTATAATAGGATTGTTACAGCTCATGCTTTTATTATAATTTTTTTTATAGTTATACCTATTATAATTGGTGGATTTGGTAATTGATTAATTCCTATAATATTAGGATGTCCTGATATAGCTTTTCCTCGAATAAATAATATAAGRTTTTGATTATTAATTCCTTCAATAATTTTTTTAATTTTTAGGAGAGTATTAAATGTTGGTGTTGGAACAGGATGAACTGTTTATCCTCCCYTATCTTTAACTATTGGTCATGGGGGTTTATCTGTTGATATGGCTATTTTTTCTTTACATTTAGCTGGAATTTCATCAATTATAGGGGCAATTAATTTTATTACAACAATTTTAAATATACGTTCTGATAAAGTTTTTATAGATAAAATTTCTTTATTAAGTTGATCTGTTTTAATTACAGCTATTTTATTATTATTATCTTTACCTGTATTAGCTGGGGCAATTACAATATTATTAACTGATCGAAATTTAAATACTTCATTTTTTGATCCGTCAGGTGGGGGTGATCCTATTTTATATCAACATTTATTT.

###### Holotype ♀.

Alajuela, Sector San Cristobal, Sendero Perdido, 10.87940, -85.38607, 620 meters, caterpillar collection date: 7/i/2013, wasp eclosion date: 31/i/2013. Depository: CNC.

***Host data*.***Antaeotricha* Janzen146 (Depressariidae) feeding on *Lonchocarpusoliganthus* (Fabaceae).

***Caterpillar and holotype voucher codes*.** 13-SRNP-68, DHJPAR0051319.

###### Paratype.

Host = *Antaeotricha* Janzen401: DHJPAR0055603. Depository: CNC.

###### Etymology.

*Orgiluspeterrauchi* is named in honor of Peter Rauch attending the international NSF-funded planning meeting for the All Taxa Biodiversity Inventory (ATBI) of Terrestrial Systems, and contributing his wisdom to the planning that was the founding of Costa Rica’s national BioAlfa today.

**Figure 341.**
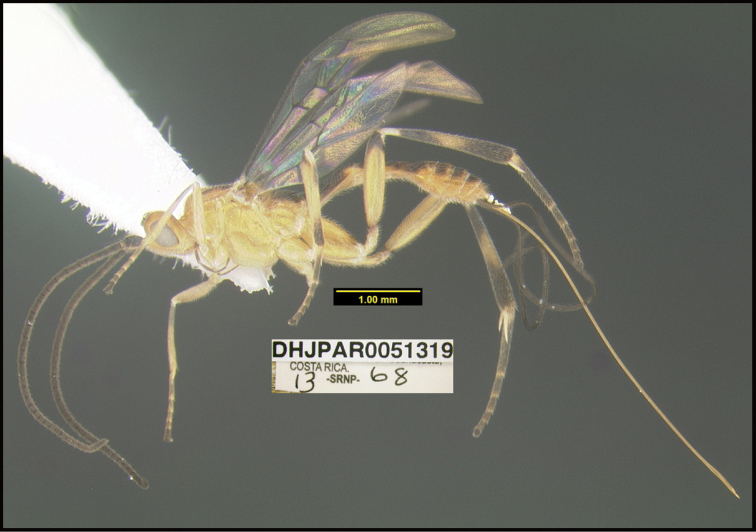
*Orgiluspeterrauchi*, holotype.

##### Orgilus richardprimacki

Taxon classificationAnimaliaHymenopteraBraconidae

Sharkey sp. nov.

http://zoobank.org/CE93F9AF-B12B-4A28-803C-A48F395CDE27

Figure 342

###### Diagnostics.

BOLD:ADB9574. Consensus barcode. ATTTTATATTTTTTATTTGGAGTTTGATCTGGTATTTTAGGTATATCAATAAGTTTTATTATTCGTTTAGAATTAGGTATACCTGGCAGACTTATTGGGAATGATCAAATTTATAATAGAATTGTTACGGCTCACGCTTTTATTATAATTTTTTTTATGGTTATACCAATTATAATTGGGGGGTTTGGTAATTGATTAGTTCCTATAATATTGGGGTGTCCTGATATAGCTTTCCCTCGTATAAATAATATGAGATTTTGATTATTAGTTCCTTCAATAATTTTTTTAATTTTTAGAGGGGTATTAAATATTGGGGTAGGTACTGGGTGAACTGTTTATCCTCCTTTATCTTTAATAATTGGACATGGTGGAGTTTCAGTTGATTTAGCTATTTTTTCTTTACATTTAGCTGGTATTTCCTCTATTATAGGTGCAATTAATTTTATTACTACTATTTTAAATATACGTTCTAATATAATTTATATAGATAAAATTTCTTTATTTATTTGGTCAGTATTGCTTACTGCTATTTTATTATTATTATCTTTACCTGTATTAGCAGGTGCTATTACTATATTATTAAGTGATCGA------------------------------------------.

###### Holotype ♀.

Guanacaste, Sector Pailas Dos, PL12-3, 10.7631, -85.3344, 820 meters, 18/ix/2014, Malaise trap. Depository: CNC.

***Host data*.** None.

***Holotype voucher code*.**BIOUG30111-A11.

###### Paratypes.

None.

###### Etymology.

*Orgilusrichardprimacki* is named in honor of Richard Primack’s long-appreciated contributions to publicity for ACG, GDFCF, and now, BioAlfa.

**Figure 342.**
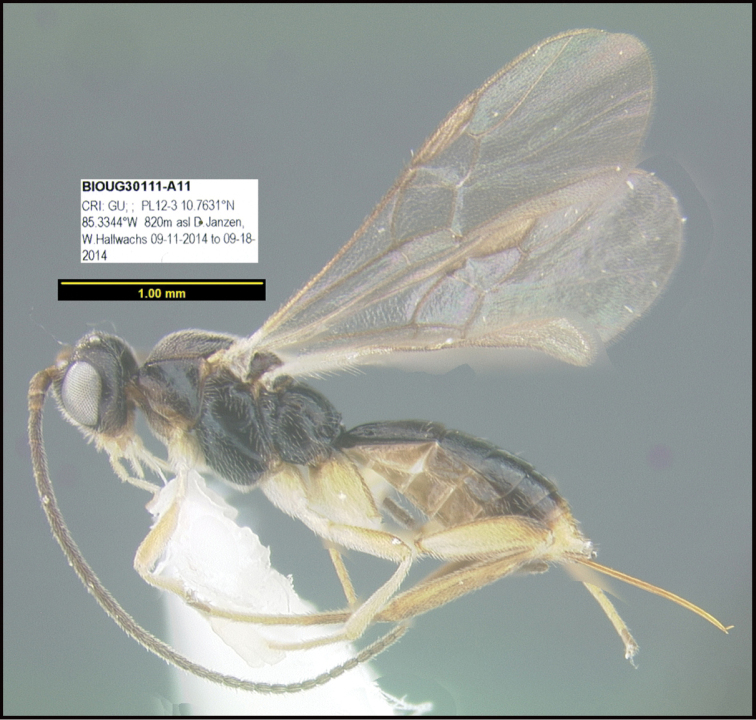
*Orgilusrichardprimacki*, holotype.

##### Orgilus sandraberriosae

Taxon classificationAnimaliaHymenopteraBraconidae

Sharkey sp. nov.

http://zoobank.org/FB6BFEB9-ABF4-4229-A742-743B2B0097B3

Figure 343

###### Diagnostics.

BOLD:ADB0231. Consensus barcode. GTTTTATATTTTTTATTTGGTATTTGAGCTGGAATTTTAGGTATGTCAATAAGATTAATTATTCGAATGGAATTAGGAATACCAGGAAGATTAATTGGTAATGATCAAATTTATAATAGGATTGTTACAGCTCATGCTTTTGTAATAATTTTTTTTATAGTTATACCAATTATAATTGGTGGTTTTGGGAATTGATTAATTCCTATAATATTAGGTTGTCCTGATATAGCTTTTCCACGTATAAATAATATAAGTTTTTGATTATTAATTCCTTCTCTTGTGTTTTTAATTTTTAGGAGAGTTTTAAATGTTGGGGTAGGAACTGGTTGAACTGTTTATCCTCCTTTATCTTTATTAATTGGACATGGTGGATTATCAGTTGATATAGCTATTTTTTCGTTACATTTAGCTGGTATTTCTTCAATTATAGGTGCAATTAATTTTATTACAACAATTTTGAATATACGGTCTGATAATGTTTATATAGATAAAATTTCTTTATTATGTTGATCAGTTTTAATTACAGCAATTTTATTATTATTATCTTTACCAGTTTTAGCTGGGGCAATTACTATATTATTAACAGATCGAAATTTAAATACT------------------------------------.

###### Holotype ♀.

Guanacaste, Sector Pailas Dos, PL12-9, 10.76, -85.3341, 809 meters, 27/ii/2014, Malaise trap. Depository: CNC.

***Host data*.** None.

***Holotype voucher code*.** BIOUG28825-B03.

###### Paratypes.

None.

###### Etymology.

*Orgilussandraberriosae* is named in honor of Sandra Berrios’ long-appreciated contributions to publicity for ACG, GDFCF, and now, BioAlfa.

**Figure 343.**
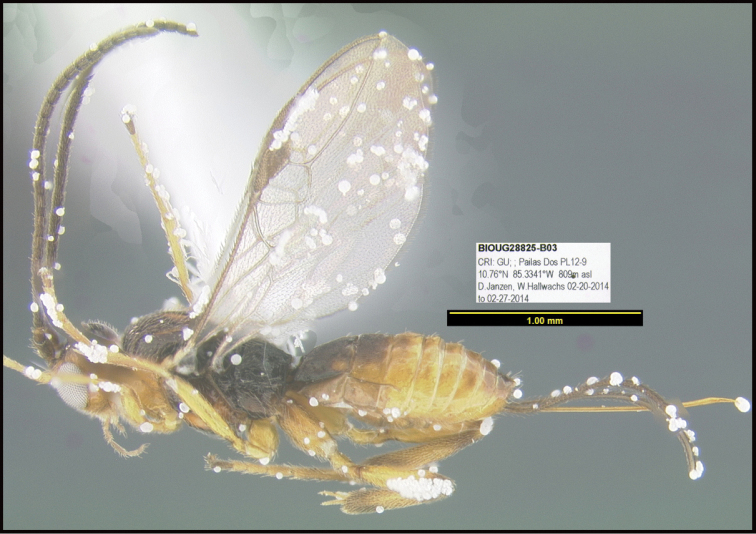
*Orgilussandraberriosae*, holotype.

##### Orgilus sarahmirandae

Taxon classificationAnimaliaHymenopteraBraconidae

Sharkey sp. nov.

http://zoobank.org/6F4388A8-0CEB-4539-B559-4E3A2159F768

Figure 344

###### Diagnostics.

BOLD:ADB0825. Consensus barcode. ATTTTGTATTTTTTATTTGGAATTTGATCAGGAATTTTAGGAATATCTATGAGTTTAATTATTCGAATGGAATTAGGGATACCTGGGAGATTAATTGGTAATGATCAAATTTATAATAGAATTGTAACAGCTCATGCTTTTATTATAATTTTTTTTATAGTTATACCAATTATAATTGGTGGGTTTGGGAATTGATTAATTCCTATAATATTAGGATGTCCTGATATAGCTTTTCCTCGTATAAATAATATAAGTTTTTGATTATTAATTCCTTCAATAATTTTTTTAATTTTTAGAAGAATTTTAAATGTTGGGGTGGGAACTGGTTGAACTGTTTATCCTCCTTTATCTTTAATAATTGGTCATGGTGGATTATCAGTTGATATAGCTATTTTTTCTTTACATTTAGCAGGAATTTCATCAATTATAGGGGCAATTAATTTTATTACTACTATTTTAAATATACGTTCTGATAAAGTTTTAATAGATAAAATTTCTTTATTAAGTTGATCAGTATTAATTACTGCTATTTTATTATTATTATCATTGCCTGTATTAGCAGGAGCAATTACAATATTATTAACGGATCGT.

###### Holotype ♀.

Guanacaste, Sector Pailas Dos, PL12-6, 10.7637, -85.333, 853 meters, 23/i/2014, Malaise trap. Depository: CNC.

***Host data*.** None.

***Holotype voucher code*.**BIOUG28804-H03.

###### Paratype.

Malaise-trapped. BIOUG28680-C02. Depository: CNC.

###### Etymology.

*Orgilussarahmirandae* is named in honor of Sarah Miranda’s long-appreciated contributions to publicity for ACG, GDFCF, and now, BioAlfa.

**Figure 344.**
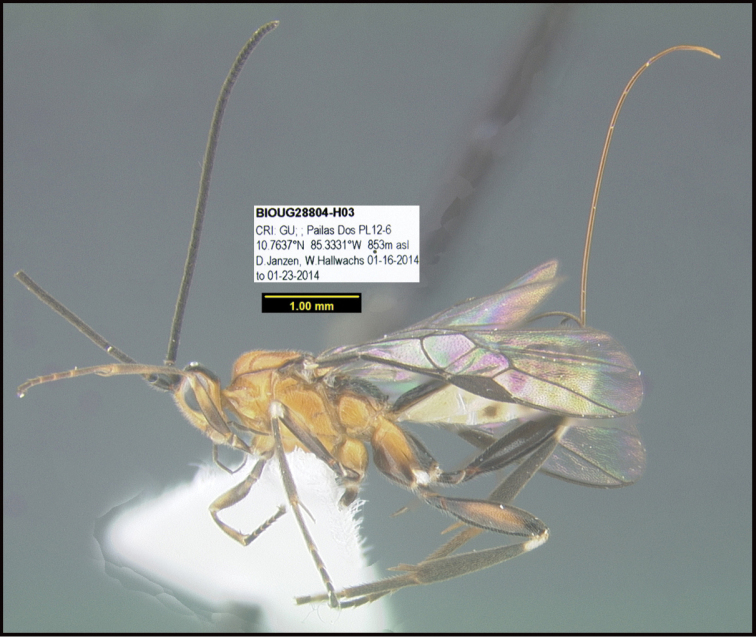
*Orgilussarahmirandae*, holotype.

##### Orgilus scottmilleri

Taxon classificationAnimaliaHymenopteraBraconidae

Sharkey sp. nov.

http://zoobank.org/30AD9929-8F88-4758-9710-2959BB1700B9

Figure 345

###### Diagnostics.

BOLD:AAW8985. Consensus barcode. TATTTTATATTTTTTATTTGGTATTTGATCTGGTATTTTAGGTATATCAATAAGTTTAATTATTCGTATAGAATTAGGTATACCTGGGACATTAATTGGAAATGATCAAATTTATAATAGAGTTGTAACAGCTCATGCATTTATTATAATTTTTTTTATAGTTATGCCAATTATAATTGGGGGGTTTGGTAATTGATTAATTCCAATAATATTAGGATGTCCTGATATGGCTTTTCCTCGTATAAATAATATAAGTTTTTGATTATTAATTCCCTCATTAATTTTTTTAATTTTTAGTGGGGTTTTAAATGTTGGAGTTGGTACAGGGTGAACTGTTTATCCTCCCTTATCTTTAGGTATTGGTCATGGTGGATTTTCAGTGGATATAGCTATTTTTTCTTTACATTTAGCTGGAATTTCTTCAATTATGGGGGCAATTAATTTTATTACTACTATTTTAAATATACGTTCTGATAAAGTTTTAATAGATAAAATTTCTTTATTAAGATGATCGGTATTAATTACAGCTATTTTGTTGTTATTATCTTTACCGGTATTAGCAGGGGCAATTACAATATTATTAACAGATCGTAATTTAAATACATCTTTTTTTGATCCTTCTGGAGGTGGGGATCCTATTTTATATCAACATTTATTT.

###### Holotype ♀.

Alajuela, Sector San Cristobal, Quebrada Cementerio, 10.87124, -85.38749, 700 meters, caterpillar collection date: 6/vii/2009, wasp eclosion date: 21/vii/2009. Depository: CNC.

***Host data*.***Anadasmus* Janzen11 (Depressariidae) feeding on *Nectandrahihua* (Lauraceae).

***Caterpillar and holotype voucher codes*.** 09-SRNP-3511, DHJPAR0040031.

###### Paratypes.

None.

###### Etymology.

*Orgilusscottmilleri* is named in honor of Scott Miller attending the international NSF-funded planning meeting for the All Taxa Biodiversity Inventory (ATBI) of Terrestrial Systems, and contributing his wisdom to the planning that was the founding of Costa Rica’s national BioAlfa today.

**Figure 345.**
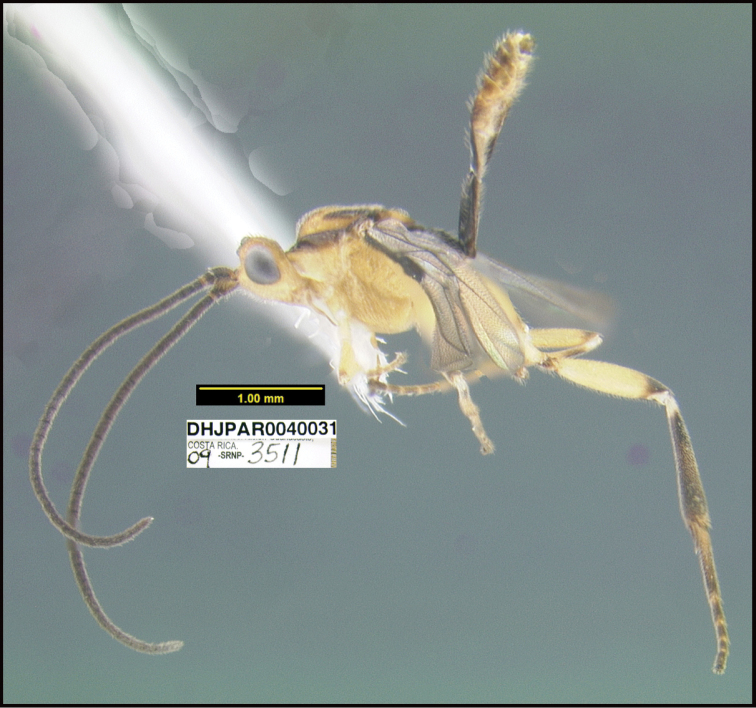
*Orgilusscottmilleri*, holotype.

##### Orgilus scottmorii

Taxon classificationAnimaliaHymenopteraBraconidae

Sharkey sp. nov.

http://zoobank.org/0D29BCFE-BDDB-4C83-B9CC-5923A2926C88

Figure 346

###### Diagnostics.

BOLD:ABA8481. Consensus barcode. TATTTTATATTTTTTATTTGGTATTTGATCTGGTATTTTAGGTATATCAATAAGTTTAATTATTCGTATAGAATTAGGTATACCTGGGACATTAATTGGAAATGATCAAATTTATAATAGAGTTGTAACAGCTCATGCATTTATTATAATTTTTTTTATAGTTATGCCAATTATAATTGGGGGGTTTGGTAATTGATTAATTCCAATAATATTAGGATGTCCTGATATGGCTTTTCCTCGTATAAATAATATAAGTTTTTGATTATTAATTCCCTCATTAATTTTTTTAATTTTTAGTGGGGTTTTAAATGTTGGAGTTGGTACAGGGTGAACTGTTTATCCTCCCTTATCTTTAGGTATTGGTCATGGTGGATTTTCAGTGGATATAGCTATTTTTTCTTTACATTTAGCTGGAATTTCTTCAATTATGGGGGCAATTAATTTTATTACTACTATTTTAAATATACGTTCTGATAAAGTTTTAATAGATAAAATTTCTTTATTAAGATGATCGGTATTAATTACAGCTATTTTGTTGTTATTATCTTTACCGGTATTAGCAGGGGCAATTACAATATTATTAACAGATCGTAATTTAAATACATCTTTTTTTGATCCTTCTGGAGGTGGGGATCCTATTTTATATCAACATTTATTT.

###### Holotype ♀.

Guanacaste, Sector San Cristobal, Tajo Angeles, 10.86472, -85.41531, 540 meters, caterpillar collection date: 13/v/2011, wasp eclosion date: 27/v/2011. Depository: CNC.

***Host data*.***Antaeotricha* Janzen233 (Depressariidae) feeding on *Tapirirabrenesii* (Anacardiaceae).

***Caterpillar and holotype voucher codes*.** 11-SRNP-1951, DHJPAR0043217.

###### Paratypes.

Hosts = elachBioLep01 BioLep754 (Depressariidae), *Antaeotricha* Janzen233. DHJPAR0049310, DHJPAR0043221, DHJPAR0043234, DHJPAR0043237, DHJPAR0048269, DHJPAR0048244, DHJPAR0043209, DHJPAR0043210, DHJPAR0043242, DHJPAR0043249. Depository: CNC.

###### Etymology.

*Orgilusscottmorii* is named in honor of Scott Mori attending the international NSF-funded planning meeting for the All Taxa Biodiversity Inventory (ATBI) of Terrestrial Systems, and contributing his wisdom to the planning that was the founding of Costa Rica’s national BioAlfa today.

**Figure 346.**
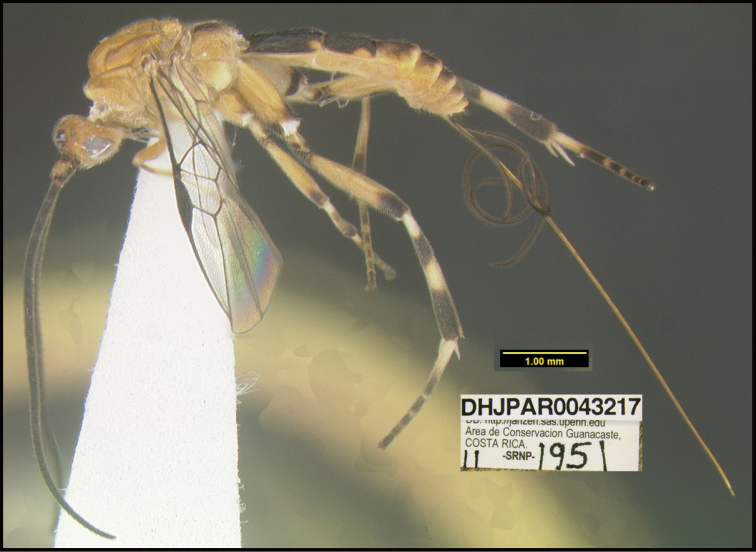
*Orgilusscottmorii*, holotype.

#### *Stantonia* Ashmead, 1904

There are eight species of *Stantonia* recorded from Costa Rica (Braet and Quicke 2004), but only three with the type specimen from Costa Rica, i.e., *S.osa*, *S rossa*, and *S.sirena* (all Braet & Quicke, 2004.) The holotypes of the others are from Brazil or the Caribbean. Based on the descriptions, none of the eight species is conspecific with those treated below. The following families have been recorded as hosts: Pyralidae, Crambidae, Noctuidae, and Tortricidae. Our data concur with those of Braet and Quick (2004) in that most hosts are in the family Crambidae. Here we report two new host families for *Stantonia*, Oecophoridae and Immidae.

##### Stantonia billalleni

Taxon classificationAnimaliaHymenopteraBraconidae

Sharkey sp. nov.

http://zoobank.org/57626613-7CCA-4798-A9A2-34C654239921

Figure 347

###### Diagnostics.

BOLD:AAM1059. Consensus barcode. TATTTTATATTTAAAATTTGGAATTTGAGCTGGTATTTTAGGTATGTCTATAAGATTAATTGTACGATTAGAATTAGGTATACCTGGTAGTATAATTGGTAATGATCAAATTTATAATAGAATTGTTACTGCTCATGCTTTTGTAATAATTTTTTTTATGGTTATACCAATTATAATTGGGGGGTTTGGTAATTGATTAATTCCAATAATATTGGGATGTCCAGATATAGCTTTCCCACGTATAAATAATATAAGATTTTGATTATTAATTCCTTCTTTAATATTATTAATTTTTAGAAGAGTTTTAAATATTGGGGTAGGTACTGGATGAACAGTATATCCACCATTATCYTTATTGATTGGCCATGGTGGATTATCAGTTGATATAGCAATTTTTTCTTTGCATTTGGCTGGTATTTCTTCTATTATAGGAGCAATTAATTTTATTACTACAATTTTAAATATGCGAGTTGATAAAGTTTATATAGATAAAATTTCTTTATTATCTTGATCAGTTTTTATTACTGCAATTTTATTATTATTATCTTTACCTGTATTAGCTGGTGCTATTACTATATTATTAACTGATCGTAATTTAAATACTTCTTTTTTTGATCCTTCAGGTGGRGGTGATCCAATTTTATATCAACATTTGTTT.

###### Holotype ♀.

Guanacaste, Sector Mundo Nuevo, Estación La Perla, 10.76737, -85.43313, 325 meters, caterpillar collection date: 16/x/2012, wasp eclosion date: 2/xi/2012. Depository: CNC.

***Host data*.***Hyaloristaexuvialis* (Crambidae) feeding on *Hyptisverticillata* (Lamiaceae).

***Caterpillar and holotype voucher codes*.** 12-SRNP-56280, DHJPAR0051309.

###### Paratypes.

Hosts = *Hyaloristaexuvialis* and *Hyalorista* exuvialisDHJ02. DHJPAR0038188, DHJPAR0051311. Depository: CNC.

###### Etymology.

*Stantoniabillalleni* is named in honor of Bill Allen’s long-appreciated contributions to publicity for ACG, GDFCF, and now, BioAlfa.

**Figure 347.**
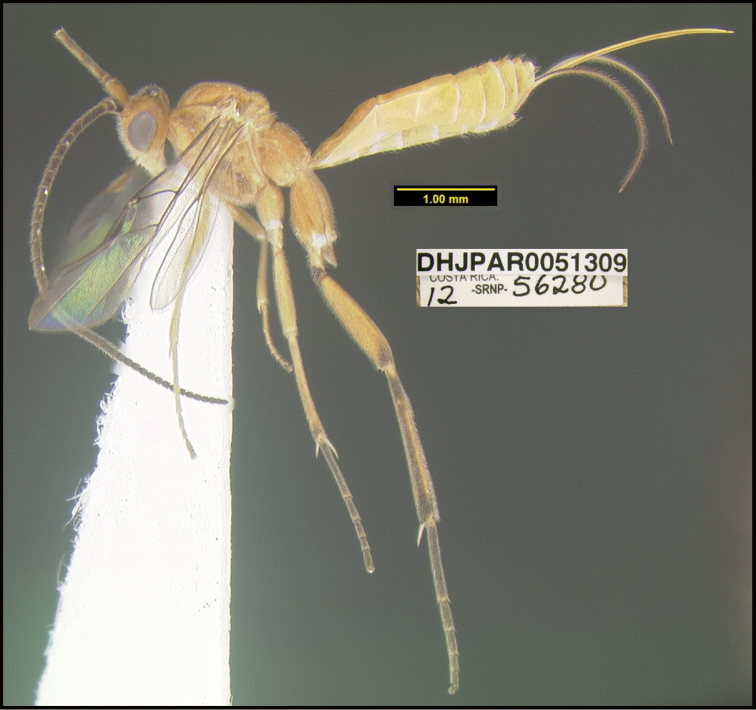
*Stantoniabillalleni*, holotype.

##### Stantonia brookejarvisae

Taxon classificationAnimaliaHymenopteraBraconidae

Sharkey sp. nov.

http://zoobank.org/8B3871C7-5C71-4EBF-8540-4E1DA069A8E4

Figure 348

###### Diagnostics.

BOLD:AAK5505. Consensus barcode. AATTTTGTATTTAAAATTTGGRATTTGGGCTGGGATTTTAGGTATATCATTAAGATTAATTATTCGATTAGAGTTAGGAACTCCTGGGAGATTAATTGGTAATGATCAAATTTATAATAGTGTGGTAACTTCTCATGCTTTTATTATAATTTTTTTTATAGTTATACCAATTATAATTGGGGGTTTTGGAAATTGGCTAATTCCTATAATATTGGGGTGTCCTGATATAGCATTTCCTCGTATAAATAATATAAGATTTTGGTTGTTAATTCCTTCTTTAATAATATTAATTTTTAGAGGTATTTTAAATATTGGAGTTGGTACAGGTTGAACTGTTTATCCTCCTTTATCTTTAAATATTGGTCATGGTGGAGTTTCAGTTGATATATCTATTTTTTCTTTACATTTAGCTGGTATTTCTTCCATYATAGGYGCCGTAAATTTTATTACTACAGTTTTAAATATACGAATTAAATTAATTTTAATGGATAAAATTTCTTTATTAATTTGATCAGTTTTTATYACAGCTATTTTATTGTTATTATCTTTACCTGTTTTAGCTGGGGCTATTACTATATTGTTAACAGATCGTAATTTAAATACTTCTTTTTTTGATCCWTCTGGGGGGGGTGATCCAGTTTTGTATCAACATTTATTT.

###### Holotype ♀.

Alajuela, Sector Rincon Rain Forest, Rio Francia Arriba, 10.89666, -85.29003, 400 meters, caterpillar collection date: 28/vi/2010, wasp eclosion date: 12/vii/2010. Depository: CNC.

***Host data*.***Pilocrocispurpurascens* (Crambidae) feeding on *Pentagoniadonnell-smithii* (Rubiaceae).

***Caterpillar and holotype voucher codes*.** 10-SRNP-42400, DHJPAR0040550.

###### Paratypes.

Hosts = *Desmia* benealisDHJ02, *Phostrialatiapicalis*, *Pilocrocispurpurascens* (all Crambidae). DHJPAR0050354, DHJPAR0050911, DHJPAR0050920, DHJPAR0057266, DHJPAR0036321, DHJPAR0052099, DHJPAR0053534, DHJPAR0053538. Depository: CNC.

###### Etymology.

*Stantoniabrookejarvisae* is named in honor of Brooke Jarvis’ long-appreciated contributions to publicity for ACG, GDFCF, and now, BioAlfa.

**Figure 348.**
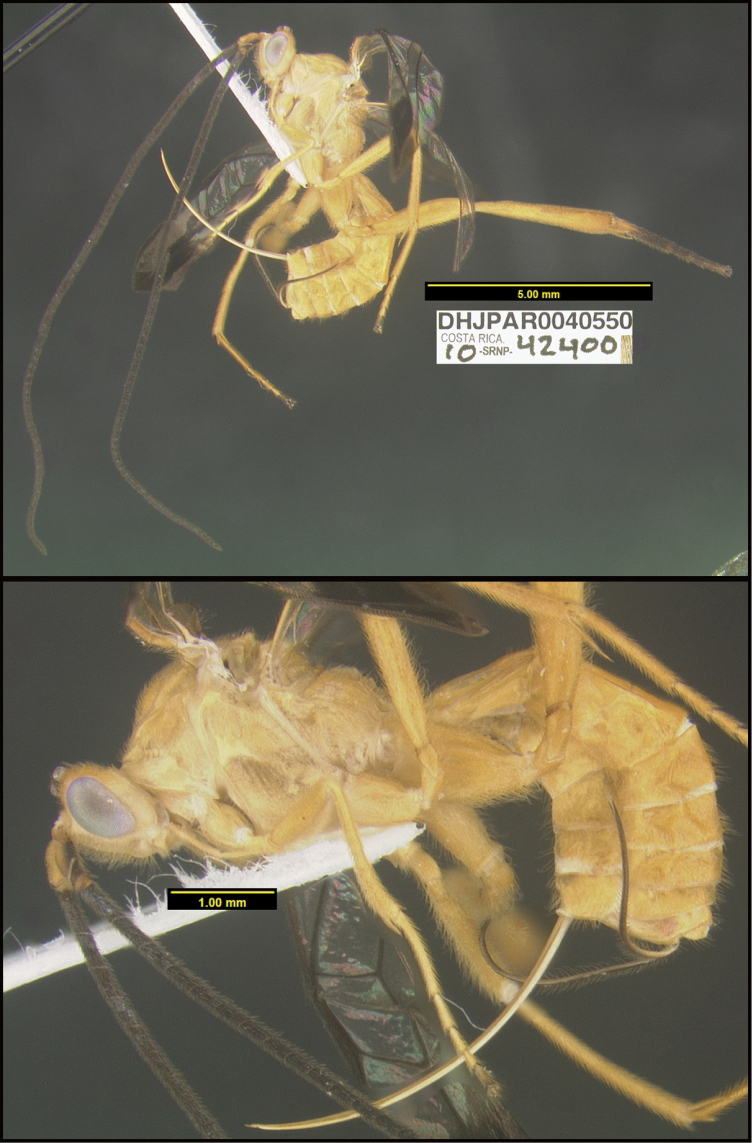
*Stantoniabrookejarvisae*, holotype.

##### Stantonia donwilsoni

Taxon classificationAnimaliaHymenopteraBraconidae

Sharkey sp. nov.

http://zoobank.org/8575FFEE-540A-409D-92F6-FAC63514D912

Figure 349

###### Diagnostics.

BOLD:AAG3930. Consensus barcode. TATTTTATATTTTAAATTTGGTATTTGATCTGGTATTATRGGAATATCTTTAAGATTAATTGTACGAATAGAGTTAGGTATACCTGGTAGTTTAATTGGTAATGATCAAATTTATAATAGGGTAGTAACTGCTCATGCATTTGTAATAATTTTTTTTATAGTTATACCTATTATAATTGRGGGATTTGGCAATTGATTAATTCCTTTAATATTAGGTTGTCCAGATATAGCTTTCCCTCGTATAAATAATATAAGGTTTTGATTATTAATYCCTTCTTTAATATTATTGATTTTTAGAAATGTTCTGAATATTGGGGTTGGTACAGGTTGAACTGTTTATCCTCCTTTATCTTTATTAATTGGTCATGGTGGTTTATCAGTTGATATAGCAATTTTTTCTTTACATTTAGCAGGTATTTCTTCTATTATAGGGGCTGTTAATTTTATTACTACAATTTTAAATATGCGTGTTGAAAAAATTTATATGGATAAAATTTCTTTATTATCTTGATCTATTTTTATTACTGCTATTTTATTATTATTGTCTTTACCTGTTTTAGCCGGTGCAATTACTATATTATTAACTGATCGTAATATAAATACTTCTTTTTTTGATCCATCTGGTGGTGGAGATCCTATTTTATATCAACATTTATTT.

###### Holotype ♀.

Guanacaste, Santa Rosa, Area Administrativa, 10.83764, -85.61871, 295 meters, caterpillar collection date: 27/vii/2013, wasp eclosion date: 6/viii/2013. Depository: CNC.

***Host data*.***Portentomorphaxanthialis* (Crambidae) feeding on *Margaritarianobilis* (Phyllanthaceae).

***Caterpillar and holotype voucher codes*.** 13-SRNP-19529, DHJPAR0052706.

###### Paratypes.

Hosts = *Omiodeshumeralis*, *Psara* obscuralisDHJ02, *Eulepteconcordalis*, *Portentomorphaxanthialis*, *Phostrialatiapicalis*, *Hahncappsia* BioLep471 (all Crambidae). DHJPAR0029187, DHJPAR0029429, DHJPAR0041487, DHJPAR0052872, DHJPAR0052875, DHJPAR0063891, DHJPAR0063896. Depository: CNC.

###### Etymology.

*Stantoniadonwilsoni* is named in honor of Don Wilson attending the international NSF-funded planning meeting for the All Taxa Biodiversity Inventory (ATBI) of Terrestrial Systems, and contributing his wisdom to the planning that was the founding of Costa Rica’s national BioAlfa today.

**Figure 349.**
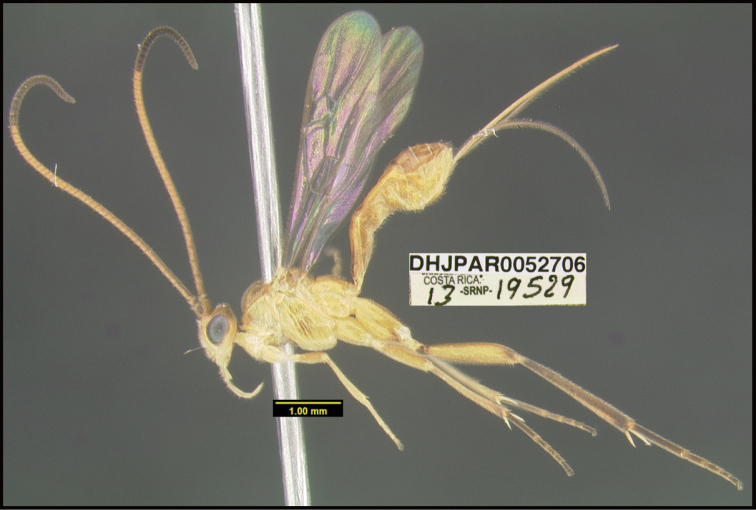
*Stantoniadonwilsoni*, holotype.

##### Stantonia erikabjorstromae

Taxon classificationAnimaliaHymenopteraBraconidae

Sharkey sp. nov.

http://zoobank.org/57B415F7-F634-4451-8691-4F06168E095B

Figure 350

###### Diagnostics.

BOLD:ABX0808. Consensus barcode. AATTTTATATTTAAAATTTGGAATTTGGTCAGGTATTTTAGGCATATCTTTAAGTTTAATTGTTCGAATAGAATTAGGGATACCYGGTAATTTAATTGGTAATGATCAAATTTATAATAGAATTGTTACATTTCATGCTTTTGTAATAATTTTTTTTATAGTTATACCAATTATAATTGGTGGGTTTGGAAATTGATTAATTCCTTTAATATTAGGATGTCCTGATATAGCTTTTCCTCGAATGAATAATATAAGATTTTGATTATTAATTCCTTCTTTATTGTTATTAATTTTTAGAAGAATTTTAAATATTGGTGTGGGGACTGGTTGAACAGTTTATCCYCCTTTATCTTTATCTATAGGTCATGGAGGAATTTCTGTAGATATGGCTATTTTTTCTTTACATTTAGCCGGAGCTTCTTCYATTATAGGYGCYGTAAATTTTATTACTACTATTTTAAATATACGAATTAATAAAATTTATTTAGATAAAATTTCTTTATTATCATGATCTGTTTTTATTACTGCTATTTTATTATTATTATCTTTACCTGTTTTAGCYGGAGCAATTACTATATTATTAACTGATCGTAATTTAAATACTTCTTTTTTTGATCCTTCTGGGGGTGGTGATCCTATTTTATACCAGCATTTATTT.

###### Holotype ♀.

Alajuela, Sector Rincon Rain Forest, Malaguenya, 10.9555, -85.28381, 221 meters, caterpillar collection date: 16/v/2012, wasp eclosion data: 4/vi/2012. Depository: CNC.

***Host data*.***Omiodes* Janzen05 (Crambidae) feeding on *Entadagigas* (Fabaceae).

***Caterpillar and holotype voucher codes*.** 12-SRNP-67833, DHJPAR0048713.

###### Paratypes.

DHJPAR0046874, DHJPAR0046876, DHJPAR0046879, DHJPAR0046880, DHJPAR0049677. Depository: CNC.

###### Etymology.

*Stantoniaerikabjorstromae* is named in honor of Erika Bjorstrom’s long-appreciated contributions to publicity for ACG, GDFCF, and now, BioAlfa.

**Figure 350.**
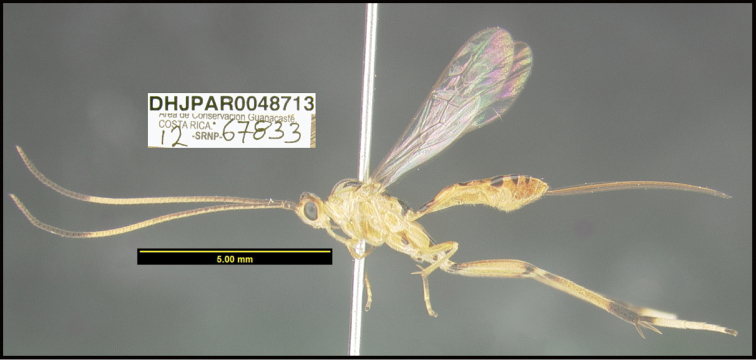
*Stantoniaerikabjorstromae*, holotype.

##### Stantonia garywolfi

Taxon classificationAnimaliaHymenopteraBraconidae

Sharkey sp. nov.

http://zoobank.org/A3E3165C-5F88-493C-965C-34C35A48FABE

Figure 351

###### Diagnostics.

BOLD:AAL9316. Consensus barcode. TTTTAGGTATATCTTTAAGTTTATTAATTCGTATAGAATTAGGTACTCCAGGAAGATTAATTGGTAATGATCAAATTTATAATAGAGTTGTTACATCTCATGCTTTTATTATAATTTTCTTTATAGTTATACCTATTATAATTGGTGGTTTTGGAAATTGATTAATTCCAATAATATTAGGGTGTCCTGATATAGCTTTTCCTCGAATAAATAATATAAGATTTTGGTTATTAATTCCTTCATTGTTAATATTACTTTTTAGTGGAGTTTTAAATATTGGTGTTGGTACTGGATGAACAGTTTATCCTCCTTTATCTTTAAATATTGGTCATGGTGGAATTTCTGTTGATATTGCTATTTTTTCTTTACATTTAGCTGGTATTTCTTCAATTATAGGTGCAATTAATTTTATTACTACAGTACTAAATATACGAACTGGAAAAATTATAATAGATAAGGTTACTTTATTAATTTGATCAGTTTTTATTACAGCTATCTTATTACTTTTGTCTTTACCAGTTTTAGCTGGTGCTATTACTATATTATTAACTGATCGTAATTTAAATACTTCATTTTTTGATCCTTCAGGTGGTGGTGATCCTGTTTTATATCAACATTTATTT.

###### Holotype ♀.

Guanacaste, Sector Pitilla, Pasmompa, 11.01926, -85.40997, 440 meters, caterpillar collection date: 29/xi/2005, wasp eclosion date: 2/i/2006. Depository: CNC.

***Host data*.** immidJanzen01 Janzen02 (Immidae) feeding on *Securidacasylvestris* (Polygalaceae).

***Caterpillar and holotype voucher codes*.** 05-SRNP-70049, DHJPAR0029189.

###### Paratype.

Host = same as holotype: DHJPAR0029188. Depository: CNC.

###### Etymology.

*Stantoniagarywolfi* is named in honor of Gary Wolf’s long-appreciated contributions to publicity for ACG, GDFCF, and now, BioAlfa.

**Figure 351.**
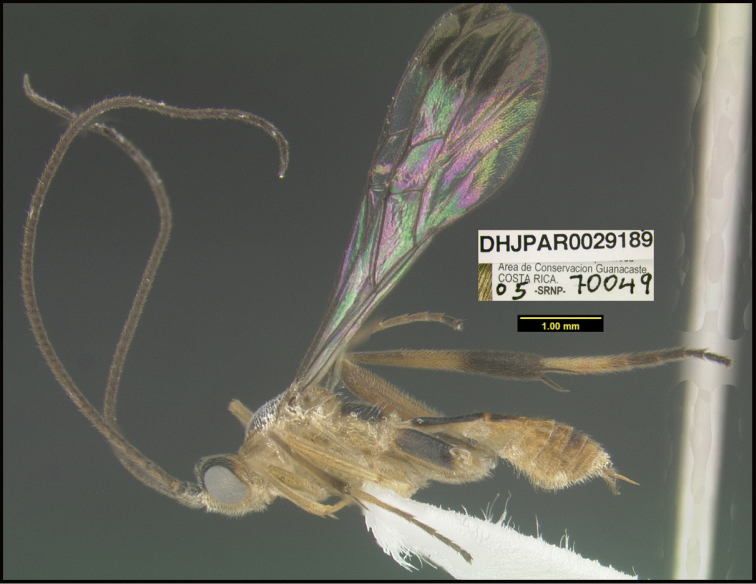
*Stantoniagarywolfi*, holotype.

##### Stantonia henrikekmani

Taxon classificationAnimaliaHymenopteraBraconidae

Sharkey sp. nov.

http://zoobank.org/5D20DAEF-DA5D-4021-B2F2-CCAC67B9572D

Figure 352

###### Diagnostics.

BOLD:AAI2111. Consensus barcode. AATTTTATATTTAAAATTTGGAATTTGGGCTGGGATTTTAGGTATATCATTAAGTTTAATTATTCGGTTAGAATTAGGTACTCCTGGAAGATTAATTGGGAATGATCAGATTTATAATAGTGTAGTTACTTCTCATGCTTTTATTATAATTTTTTTTATAGTTATACCAATTATAATTGGTGGYTTYGGRAATTGATTAATTCCTATAATATTAGGATGTCCTGATATAGCATTTCCTCGAATGAATAATATAAGATTTTGGTTATTAATTCCTTCTTTAATAATATTAATTTTTAGTGGTATTTTAAATATTGGTGTTGGGACAGGTTGAACTGTTTATCCACCTTTATCTTTAAATATTGGTCATGGGGGAATTTCAGTTGATATATCTATTTTTTCTTTACATTTAGCTGGTATTTCTTCYATCATAGGYGCYGTAAATTTTATTACTACGGTTTTAAATATACGAATTAAATTAATTTTAATAGATAAAATTTCTTTATTAATTTGATCAGTTTTTATTACAGCTATTTTATTATTATTATCTTTRCCTGTTTTAGCTGGAGCAATTACTATACTATTAACGGATCGTAATTTAAATACTTCTTTTTTTGATCCATCTGGAGGAGGAGATCCAGTATTATATCAACATTTATTT.

###### Holotype ♀.

Alajuela, Sector Rincon Rain Forest, Quebrada Bambu, 10.93009, -85.25204, 109 meters, caterpillar collection date: 5/viii/2014, wasp eclosion date: 28/viii/2014. Depository: CNC.

***Host data*.***Ceratociliasixolalis* (Crambidae) feeding on *Neeapsychotrioides* (Nyctaginaceae).

***Caterpillar and holotype voucher codes*.** 14-SRNP-76550, DHJPAR0056233.

###### Paratypes.

Hosts = *Pilocrocis* Solis20, *Desmia* ploralisDHJ03, *Eulepte* Janzen03, *Ceratociliasixolalis*, *Desmia* benealisDHJ02 (all Crambidae). DHJPAR0035220, DHJPAR0035221, DHJPAR0050358, DHJPAR0052825, DHJPAR0054462, DHJPAR0054465, DHJPAR0054466, DHJPAR0056229, DHJPAR0056230, DHJPAR0056231, DHJPAR0056232, DHJPAR0056234. Depository: CNC.

###### Etymology.

*Stantoniahenrikekmani* is named in honor of Henrick Ekman’s long-appreciated contributions to publicity for ACG, GDFCF, and now, BioAlfa.

**Figure 352.**
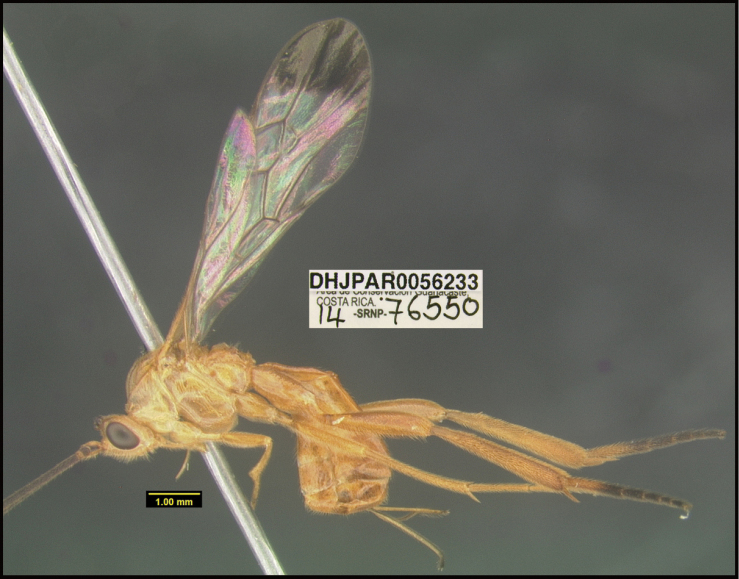
*Stantoniahenrikekmani*, holotype.

##### Stantonia luismirandai

Taxon classificationAnimaliaHymenopteraBraconidae

Sharkey sp. nov.

http://zoobank.org/6F5953DF-D797-4E98-8F04-806DCF06FF7A

Figure 353

###### Diagnostics.

BOLD:AAH9964. Consensus barcode. TATTTTATATTTAAAATTTGGTATTTGATCTGGTATTTTGGGTATATCATTAAGGTTAATTGTRCGATTAGAGTTRGGRATACCAGGTGGTTTAATTGGTAATGATCAAATTTATAATAGAGTTGTTACTGCTCATGCKTTTGTAATAATTTTTTTTATAGTAATGCCTATTATAATTGGGGGGTTTGGAAATTGGYTAATTCCAATAATGTTAGGGTGTCCAGATATGGCTTTYCCTCGAATAAATAATATAAGATTTTGGTTGTTAATTCCTTCATTAATTTTATTAATTTTTAGAAGAATTTTAAATATTGGTGTRGGGACTGGGTGAACTGTTTAYCCTCCATTATCTTTATTAATTGGTCATGGGGGTGTATCGGTTGATATGGCTATTTTTTCTTTACATTTGGCTGGTATTTCTTCAATTATGGGGGCCATTAATTTTATTACAACAATTTTAAATATACGTGTTAATAWAATTTATATAGATAAGATTTCTTTATTATGTTGGTCAGTTTTTATTACTGCTATTTTATTATTGTTATCTTTACCTGTTTTAGCYGGGGCTATTACTATATTGTTAACTGATCGAAATTTAAATACTTCTTTTTTTGATCCTTCTGGKGGAGGRGAYCCAATTTTATATCAACATTTATTT.

###### Holotype ♂.

Guanacaste, Sector Pailas Dos, PL12-3, 10.7631, -85.3344, 820 meters, 6/ii/2014, Malaise trap. Depository: CNC.

***Host data*.** None for the holotype but see below.

***Holotype voucher code*.**BIOUG29625-H09.

###### Paratypes.

Hosts = *Oryctometopiafossulatella* and *Spoladearecurvalis* (both Crambidae). DHJPAR0029190, DHJPAR0029191. Depository: CNC.

###### Etymology.

*Stantonialuismirandai* is named in honor of Luis Miranda’s long-appreciated contributions to publicity for ACG, GDFCF, and now, BioAlfa.

**Figure 353.**
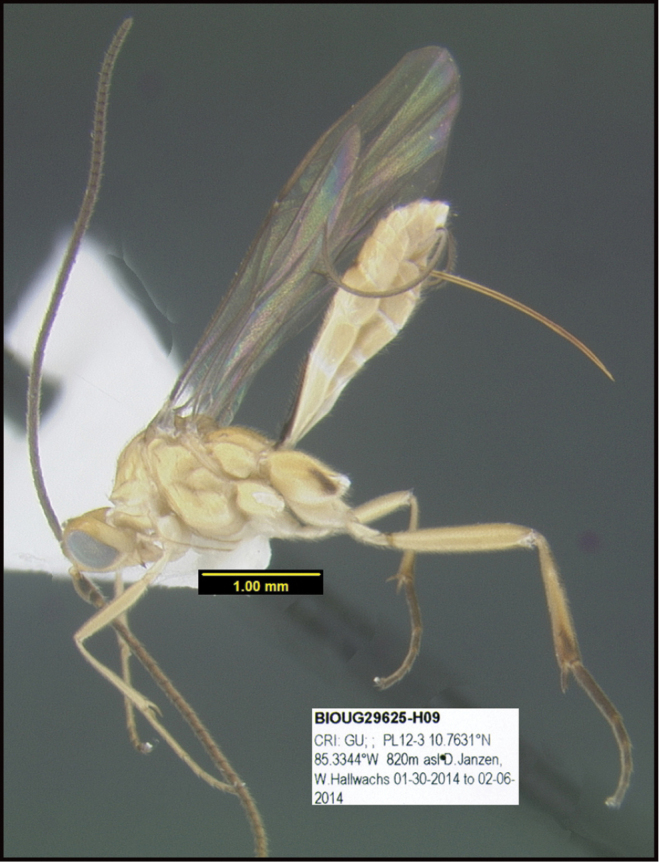
*Stantonialuismirandai*, holotype.

##### Stantonia miriamzunzae

Taxon classificationAnimaliaHymenopteraBraconidae

Sharkey sp. nov.

http://zoobank.org/5E2ABD85-58E3-49FE-962A-0B27D6DF16FF

Figure 354

###### Diagnostics.

BOLD:ACB1896. Consensus barcode. AATTTTATATTTAAAATTTGGTATTTGAGCAGGAATTTTAGGTATGTCTATAAGGTTAATTATTCGGTTAGAATTAGGAATGCCCGGAAGTTTATTAGGTAATGATCAAATTTATAATAGAATTGTAACATCTCATGCTTTTATTATAATTTTTTTTATAGTTATACCAATTATAATTGGTGGATTTGGAAATTGATTAATTCCAATAATATTAGGATGTCCTGATATAGCTTTCCCACGAATAAATAATATAAGATTTTGATTATTAATTCCTTCTTTAATAATATTAATTTTTAGAAGAATTTTAAATATTGGTGTTGGTACAGGTTGAACAGTTTATCCTCCTTTATCATTAAATATTAATCATGGTGGAATTTCTGTTGATATAGCAATTTTTTCTTTACATTTAGCTGGTATTTCTTCAATTATAGGAGCAATTAATTTTATTACTACAATTTTTAATATACGAATAAAATTAATTTTAATAGATAAAATTTCTTTATTAATTTGATCAGTATTTATTACAGCTATTTTATTATTATTATCTTTACCAGTTTTAGCTGGAGCTATTACAATATTATTAACTGATCGAAATTTAAATACTTCATTTTTTGATCCTTCAGGTGGTGGTGATCCAATTTTATATCAACATTTATTT.

###### Holotype ♂.

Alajuela, Sector Rincon Rain Forest, Quebrada Bambu, 10.9301, -85.25205, 109 meters, caterpillar collection date: 18/v/2012, wasp eclosion date: 2/vi/2012. Depository: CNC.

***Host data*.***Ategumialotanalis* (Crambidae) feeding on *Conostegiaxalapensis* (Melastomataceae).

***Caterpillar and holotype voucher codes*.** 12-SRNP-75835, DHJPAR0048704.

###### Paratypes.

None.

###### Etymology.

*Stantoniamiriamzunzae* is named in honor of Miriam Zunz’s (RIP) long-appreciated contributions to publicity for ACG, GDFCF, and now, BioAlfa.

**Figure 354.**
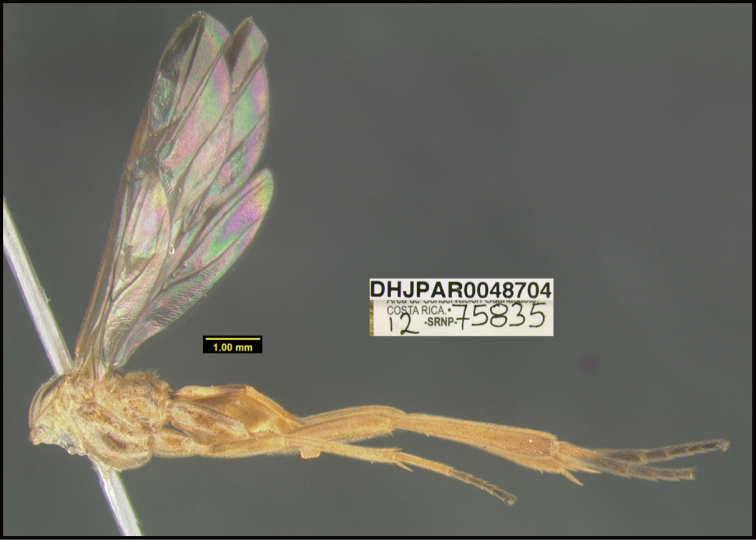
*Stantoniamiriamzunzae*, holotype.

##### Stantonia quentinwheeleri

Taxon classificationAnimaliaHymenopteraBraconidae

Sharkey sp. nov.

http://zoobank.org/E61D531A-ACB8-4396-9AC0-8EA71BA7F012

Figure 355

###### Diagnostics.

BOLD:AAD5731. Consensus barcode. AATCTTATATTTAAAATTTGGAATTTGGGCTGGGATTTTAGGTATGTCATTAAGTTTAATTATTCGATTAGAATTAGGAACTCCAGGGAGATTAATTGGTAATGATCAAATTTATAATAGTGTAGTAACTTCTCATGCTTTTATTATAATTTTTTTTATAGTTATACCAATTATAATTGGAGGGTTTGGAAATTGGTTAATTCCTATAATATTGGGGTGTCCTGATATAGCATTCCCTCGAATAAATAATATAAGATTTTGGTTATTAATTCCTTCTTTAATAATATTAATTTTTAGAGGTATTTTAAATATTGGTGTTGGGACAGGGTGAACTGTTTATCCTCCTTTATCTTTAAATATTGGTCATGGTGGAATTTCAGTTGATATATCTATTTTTTCTTTACATTTGGCTGGTATTTCTTCCATTATAGGAGCCGTAAATTTTATTACAACAGTTTTAAATATACGAATTAAAATAATTTTAATGGATAAAATTTCTTTATTAATTTGATCAGTTTTTATTACAGCTATTTTATTATTATTATCTTTACCTGTTTTAGCTGGGGCAATTACTATATTATTAACTGATCGTAATTTAAATACTACTTTTTTTGATCCGTCTGGAGGTGGGGATCCAGTTTTATATCAACATTTATTT.

###### Holotype ♂.

Guanacaste, Sector San Cristobal, Tajo Angeles, 10.86472, -85.41531, 540 meters, caterpillar collection date: 2/viii/2011, wasp eclosion date: 23/viii/2011. Depository: CNC.

***Host data*.***Syllepismarialis* (Crambidae) feeding on *Allophyluspsilospermus* (Sapindaceae).

***Caterpillar and holotype voucher codes*.** 11-SRNP-3032, DHJPAR0045054.

###### Paratypes.

Hosts = *Syllepishortalis* and *Syllepismarialis*. DHJPAR0029430, DHJPAR0029431, DHJPAR0029432, DHJPAR0029433, DHJPAR0036719, DHJPAR0029434. Depository: CNC.

###### Etymology.

*Stantoniaquentinwheeleri* is named in honor of Quentin Wheeler attending the international NSF-funded planning meeting for the All Taxa Biodiversity Inventory (ATBI) of Terrestrial Systems, and contributing his wisdom to the planning that was the founding of Costa Rica’s national BioAlfa today.

**Figure 355.**
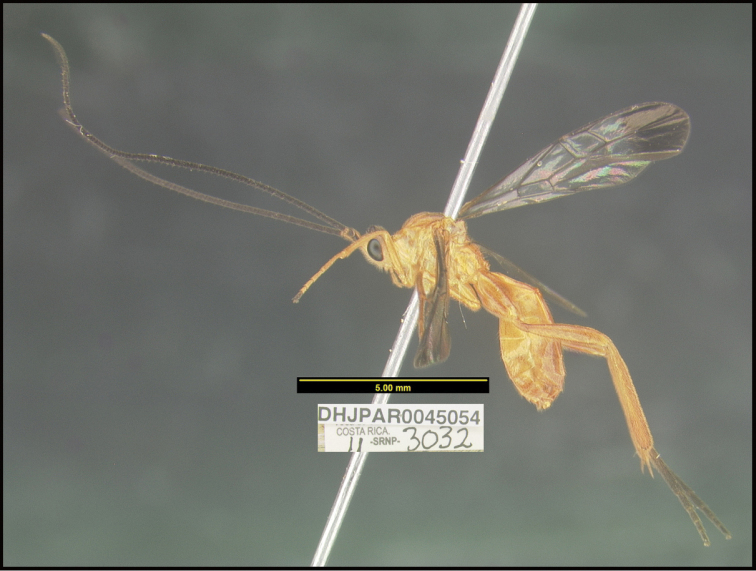
*Stantoniaquentinwheeleri*, holotype.

##### Stantonia robinkazmierae

Taxon classificationAnimaliaHymenopteraBraconidae

Sharkey sp. nov.

http://zoobank.org/6DB5B4E0-8151-48C3-A2D5-D215B0A9D055

Figure 356

###### Diagnostics.

BOLD:ACB2340. Consensus barcode. AATTTTATATTTAAAATTTGGTATTTGAGCTGGTATTTTGGGTATATCTTTAAGATTAATTGTTCGGTTAGAATTGGGTATACCTGGAAGATTAATTGGTAATGATCAAATTTATAATAGAATTGTTACTGCTCATGCTTTTGTTATAATTTTTTTTATGGTTATACCTATTATAATTGGTGGATTTGGTAATTGATTAATTCCTATAATATTAGGATGCCCTGATATGGCTTTTCCTCGAATAAATAATATGAGATTTTGGTTATTAATTCCTTCTTTGAGATTATTAATTTTTAGAAGAATTTTAAATATTGGTGTCGGTACAGGTTGAACTGTTTATCCTCCTTTATCTTTATTAATTGGCCATGGAGGAGTTTCAGTTGATATAGCAATTTTTTCTTTACATTTAGCTGGGATTTCTTCTATTATAGGGGCTATTAATTTTATTACAACAATTTTAAATATACGAATTAATAAAATTTATATAAATAATATTTCTTTATTGTCTTGATCTGGATTTATTACTGCTATTTTATTATTATTATCTTTGCCTGTTTTAGCTGGTGCTATTACTATATTATTGACTGATCGAAATTTAAATACATCTTTTTTTGATCCTTCTGGTGGAGGGGATCCTGTTTTGTATCAACATTTATTT.

###### Holotype ♀.

Alajuela, Sector San Cristobal, Sendero Huerta, 10.93050, -85.37223, 527 meters, caterpillar collection date: 28/iv/2012, wasp eclosion date: 14/v/2012. Depository: CNC.

***Host data*.***Inga* Janzen61 (Oecophoridae) feeding on *Neeapsychotrioides* (Nyctaginaceae).

***Caterpillar and holotype voucher codes*.** 12-SRNP-1693, DHJPAR0049288.

###### Paratypes.

Host = Inga Janzen61: DHJPAR0049427. Depository: CNC.

###### Etymology.

*Stantoniarobinkazmierae* is named in honor of Robin Kazmier’s long-appreciated contributions to publicity for ACG, GDFCF, and now, BioAlfa.

**Figure 356.**
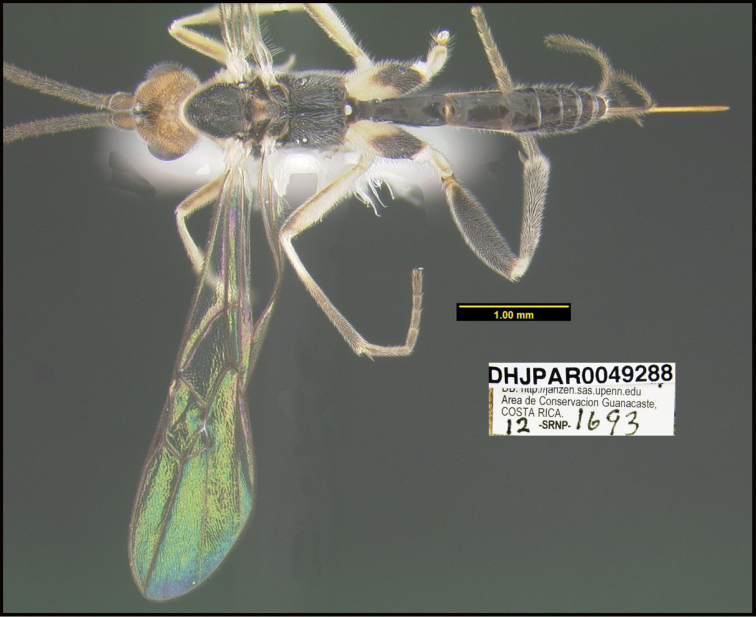
*Stantoniarobinkazmierae*, holotype.

##### Stantonia ruthtifferae

Taxon classificationAnimaliaHymenopteraBraconidae

Sharkey sp. nov.

http://zoobank.org/3E9092A9-27AD-4CFA-B3EF-CE3A9CDA9C89

Figure 357

###### Diagnostics.

BOLD:AAB3996. Consensus barcode. TATTTTATATTTTAAATTTGGTATTTGATCTGGTATTATAGGAATATCTTTAAGATTAATTGTACGAATAGAGTTAGGTATACCTGGTAGTTTAATTGGTAATGATCAAATTTATAATAGGGTAGTAACTGCTCATGCATTTGTAATGATTTTTTTTATAGTTATACCTATTATAATTGGAGGTTTTGGAAATTGATTAATTCCCTTAATATTAGGGTGTCCAGATATAGCTTTTCCTCGTATAAATAATATAAGGTTTTGATTATTAATTCCTTCTTTAATATTATTGATTTTTAGAAATGTTTTAAATATTGGGGTTGGGACAGGTTGAACTGTTTATCCTCCTTTATCTTTATTAATTGGTCATGGTGGTTTATCAGTTGATATAGCAATTTTTTCTTTACATTTAGCAGGTATTTCTTCTATTATAGGAGCTGTTAATTTTATTACTACTATTTTAAATATACGTGTTGATAWAATTTATATAGATAAAATTTCTTTGTTATCTTGATCTATTTTTATTACTGCTATTTTGTTACTATTGTCTTTACCTGTATTAGCTGGTGCAATTACTATATTATTAACTGATCGTAATATAAATACTTCTTTTTTTGATCCATCTGGTGGTGGAGATCCTA.

###### Holotype ♀.

Alajuela, Sector Rincon Rain Forest, Estación Llanura, 10.93332, -85.25331, 135 meters, caterpillar collection date: 09/x/2009, wasp eclosion date: 23/x/2009. Depository: CNC.

***Host data*.***Microthyris* prolongalisDHJ03 (Crambidae) feeding on *Ipomoeaphilomega* (Convolvulaceae).

***Caterpillar and holotype voucher codes*.** 09-SRNP-76086, DHJPAR0037144.

###### Paratype.

Host = *Stenoma* luctificaDHJ02 (Depressariidae): DHJPAR0049372. Depository: CNC.

###### Etymology.

*Stantoniaruthtifferae* is named in honor of Ruth Tiffer attending the international NSF-funded planning meeting for the All Taxa Biodiversity Inventory (ATBI) of Terrestrial Systems, and contributing her wisdom to the planning that was the founding of Costa Rica’s national BioAlfa today.

**Figure 357.**
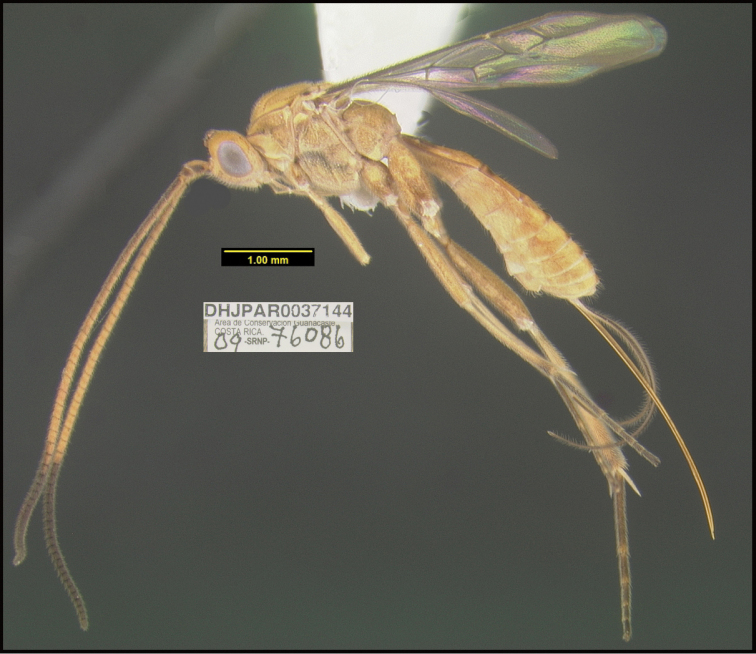
*Stantoniaruthtifferae*, holotype.

## Chapter 10: Proteropinae

Proteropinae are treated here as a subfamily following Chen and Achterberg (2019). This elevation is based on numerous phylogenetic analyses that do not resolve the Ichneutinae plus Proteropinae (i.e., the former definition of Ichneutinae s. l.) as monophyletic (Sharanowski et al. 2011). Proteropinae are difficult to diagnose due to the morphological divergence among the genera; however, the following may be somewhat helpful: mouthparts normal, not exodont nor cyclostome; ovipositor short, much shorter than metasoma; fore- and hind wings fully veined; setae of ovipositor sheath restricted to apex; and vein M of forewing not sharply curved where it meets the parastigma, as is the case with Ichneutinae. There are five genera, all of which are found in the New World. A key to genera follows. Hosts are published only for *Proterops*, and records include tenthredinid and argid sawfly larvae (Yu et al. 2016). Here we report the first host record for *Hebichneutes*. For the Proteropinae NJ tree, see Suppl. material 6.

### Key to the New World genera of Proteropinae

**Table:** 

1	A. Occipital carina present; Neotropical	*** Helconichia ***
–	B. Occipital carina absent; widespread	**2**
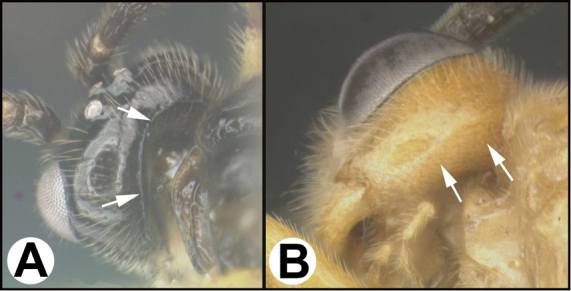		
2(1)	A. Epicnemial carina present; Neotropical	*** Muesonia ***
–	B. Epicnemial carina absent; widespread	**3**
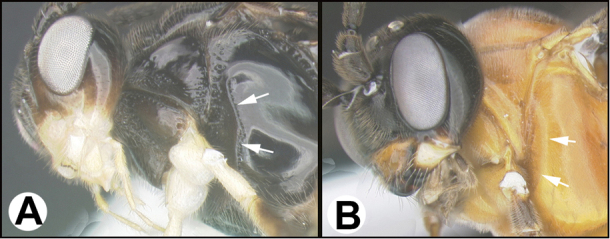		
3(2)	A. Notaulus smooth; widespread	**4**
–	B. Notaulus sculptured, at least some crenulae anteriorly; Neotropical	**5**
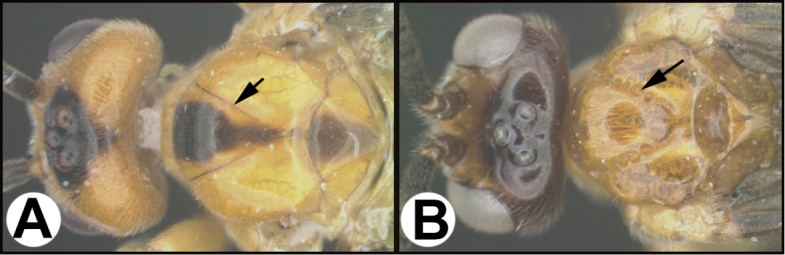		
4(3)	A. First median tergite smooth, lacking lateral carinae; widespread, common	*** Proterops ***
–	B. First median tergite with lateral carinae; Neotropical, rare	***Michener* gen. nov.**
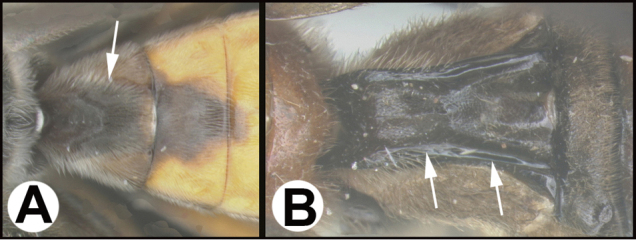		
5(3)	A. Longitudinal carina on propleuron present. AA. Petiole with a pair of weak, short, parallel, longitudinal carinae. AAA. Female without patch of setae at apex of hypopygium; Neotropical	*** Masonbeckia ***
–	B. Longitudinal carina of propleuron absent. BB. Petiole with pair of strong, converging, longitudinal carinae that form a spoon-shaped depression at the base of the tergum. BBB. Female with patch of setae at apex of hypopygium; Neotropical	*** Hebichneutes ***
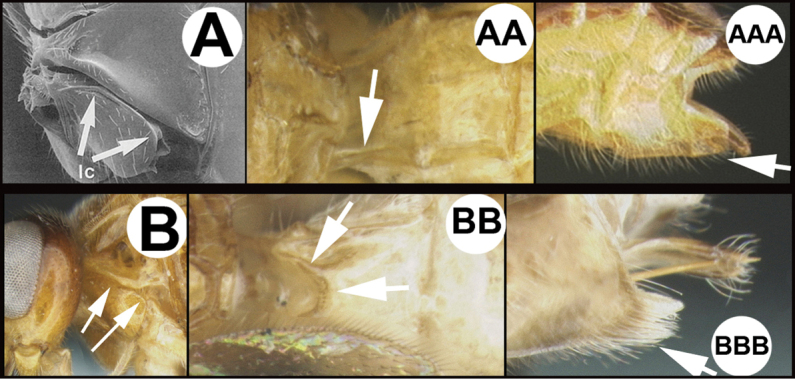		

#### *Hebichneutes* Sharkey & Wharton, 1994

Members of the genus are restricted to the Neotropics. The genus was erected and revised by Sharkey and Wharton (1994). There are five described species. Here we report the first host record for the genus.

##### Hebichneutes tricolor

Taxon classificationAnimaliaHymenopteraBraconidae

Sharkey & Wharton, 1994

Figure 358

###### Diagnostics.

BOLD:ACJ8785. Consensus barcode. TATTTTGTATTTTATATTTGGAATATGAAGAGGTATATTAGGTTTATCTATAAGTTTAATTATTCGGTTAGAATTAGGAATACCAGGAAGATTATTATTTAATGATCAAATTTATAATAGGATTGTAACTTCACATGCTTTTGTAATAATYTTTTTTATAGTTATACCAATTATAATTGGAGGATTTGGAAATTGATTAATTCCTTTAATATTAGGGGCTCCTGATATAGCTTTTCCTCGTATAAATAATATAAGATTTTGATTATTAATTCCTTCATTAATATTATTATTATTAAGAAGTTTAGTTAATGTAGGAGCTGGAACTGGTTGAACAGTTTACCCYCCTTTATCTTTAATTATAGGACATGGAGGAATTTCTGTTGATTTATGTATTTTTTCTTTACATTTAGCTGGTATATCATCTATTATAGGAGCAATTAATTTTATTAGAACTATTTTAAATATACGTGTTTTTGGAATAAAAATAGAAAATATTTCATTATTGAGTTGATCAATTTTTATTACTGCTTTTTTATTATTATTATCATTACCTGTTTTAGCAGGAGCCATTACTATATTATTAACTGATCGTAATTTAAATACAAGATTTTTTGATCCATCTGGAGGAGGTGATCCAATTTTATATCAACATTTATTT.

###### Illustrated specimen.

BIOUG17956-G03. Guanacaste, Sector Santa Rosa, Bosque San Emilio, 10.8438, -85.6138, 300 meters, Malaise trap, 11/vi/2012. Depository: CNC.

###### Other specimens.

BIOUG18749-D04, BIOUG18822-C03, BIOUG18748-A03, BIOUG08345-F02, BIOUG08345-F03, BIOUG08583-E09, DHJPAR0015420, DHJPAR0015418, DHJPAR0015417, DHJPAR0015419. Depository: CNC.

***Host data*.** The DHJPAR specimens were reared from sawfly larvae (family unknown because none reached adulthood) feeding on *Xylophragmaseemannianum* (Bignoniacae).

###### Note.

The holotype is from Guanacaste Province in Costa Rica.

**Figure 358.**
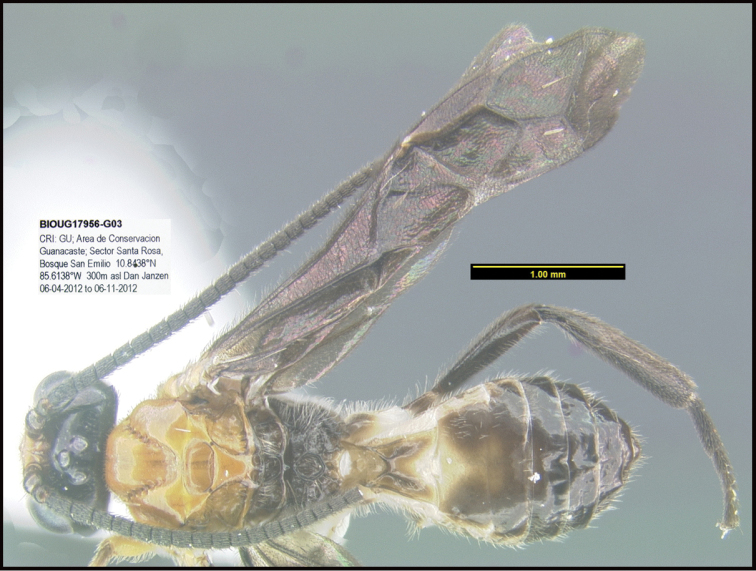
*Hebichneutestricolor*.

#### *Proterops* Wesmael, 1835

Members of this genus are widespread in the Holarctic and Oriental regions, but not recorded from Africa or Australia. There are 12 described species. Here we describe the first Neotropical species, although there are a dozen or more undescribed. Members are parasitoids of tenthredinid and argid sawfly larvae (Yu et al. 2016).

##### Proterops iangauldi

Taxon classificationAnimaliaHymenopteraBraconidae

Sharkey sp. nov.

http://zoobank.org/5AE9B32F-F1DF-447A-9C10-7BDD8A532BFE

Figure 359

###### Diagnostics.

BOLD:ADD0222. Consensus barcode. ATTTTATATTTTATATTTGGTATAGGAGAGGTATAATTGGATTTCTTTAAGAATAATTATTCGATTAGAGTTAGGTTTACCAGGAAAATTTTTAGGTAATGATCAAATTTATAACAGAATTGTTACTTTACATGCTTTTATTATAATTTTTTTTATAGTTATACCAATTATAATTGGAGGATTTGGTAATTGATTAATTCCTTTAATATTAGGAGCTCCTGATATAGCATTTCCTCGAATAAATAATATAAGATTTTGATTATTAATTCCTTCTTTATTTTTATTATTATTAAGAAGATTTATTAATAATGGGGTAGGAACTGGATGAACAGTTTATCCTCCTTTGTCATTATTAATAGGACATAGAGGTATTTCTGTTGATTTGAGAATTTTTTCTTTACATTTAGCTGGTATATCTTCTATTATAGGAGCTATTAATTTTATTACTACTATTTTTAATATACGATTAATTGGAATGAGAATAGATAAAATTTCTTTATTGGTTTGATCAGTTTTAATTACAGCATTTTTATTATTATTATCTTTACCTGTTTTAGCGGGAGCTATTACTATATTATTAACTGATCGTAATTTT------------------------------------------------------------.

###### Holotype ♀.

Guanacaste, Sector Horizontes, Quebrada San Pancho, 10.74769, -85.58577, 90 meters, sawfly larva collection date: 26/v/2000, wasp eclosion date: 12/vi/2000. Depository: CNC.

***Host data*.***Sphacophilusedus* (Argidae) feeding on *Heteropteryslaurifolia* (Malpighiaceae).

***Sawfly larva and holotype voucher codes*.** 00-SRNP-7495, DHJPAR0015459.

###### Paratypes.

DHJPAR0015457, DHJPAR0015458, DHJPAR0015460, DHJPAR0015461, DHJPAR0015462, DHJPAR0015463, DHJPAR0015464. Depository: CNC.

###### Etymology.

*Proteropsiangauldi* is named in honor of Ian Gauld (RIP) attending the international NSF-funded planning meeting for the All Taxa Biodiversity Inventory (ATBI) of Terrestrial Systems, and contributing his wisdom to the planning that was the founding of Costa Rica’s national BioAlfa today.

**Figure 359.**
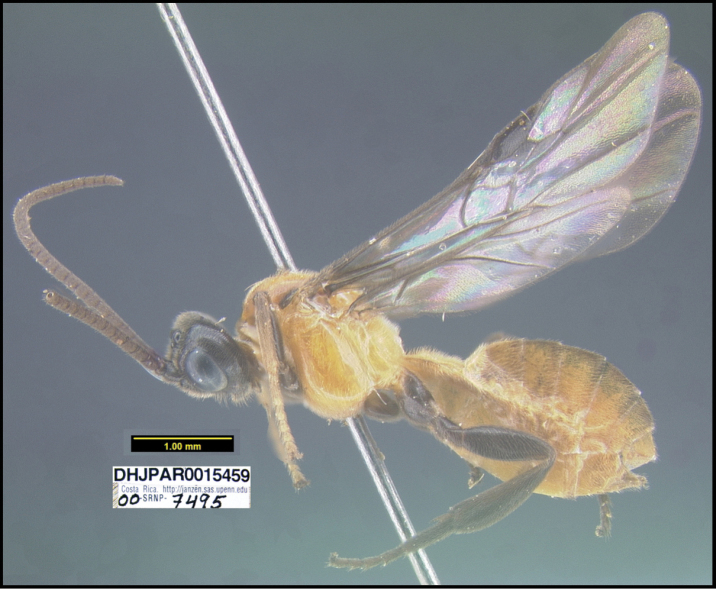
*Proteropsiangauldi*, holotype.

##### Proterops vickifunkae

Taxon classificationAnimaliaHymenopteraBraconidae

Sharkey sp. nov.

http://zoobank.org/7D56B99D-95B2-4077-8583-B3EEDB2B1553

Figure 360

###### Diagnostics.

BOLD:AAM1715. Consensus barcode. AATATTATATTTTTTATTTGGGATTTGATCGGGAATAGTAGGTCTTTCATTAAGATTAATTATTCGGTTGGAATTAGGAATCCCTGGAAAATTTTTAGGTAATGATCAAATTTATAATAGTATGGTTACTATACATGCTTTTATTATAATTTTTTTTATAGTTATACCTATTATAATTGGTGGATTTGGAAATTGGTTAATTCCTTTAATATTAGGGGCTCCTGATATAGTATTCCCTCGAATAAATAATATAAGATTTTGATTATTAATTCCTTCATTAATTTTATTATTATTAAGTGGATTTATAGGTAATGGTGTTGGGACTGGATGAACAGTTTATCCACCATTGTCTTTAATTATAGGTCATTCTAGTATATCTGTAGATATAAGAATTTTTTCTTTACATTTAGCAGGTATATCTTCTATTATAGGAGCTATAAATTTTATTACTACAATTTTTAATATGCGATTATTAGGGATAAGTATGGATAAAATTTCTTTATTAGTATGATCAATTTTTATTACTGCTTTTTTATTATTAATATCTTTACCAGTTTTAGCTGGTGCAATTACTATATTATTAACTGATCGTAATTTAAATACATGTTTTTTTGATCCTTCTGGTGGAGGGGATCCTATTTTGTATCAACATTTATTT.

###### Holotype ♀.

Guanacaste, Sector Pitilla, Bullas, 10.98670, -85.38503, 440 meters, sawfly larva collection date: 8/x/2009, wasp eclosion date: 3/xi/2009. Depository: CNC.

***Host data*.***Ptenosleucopoda* (Argidae) feeding on *Ingaoerstediana* (Fabaceae).

***Sawfly larva and holotype voucher codes*.** 09-SRNP-73070, DHJPAR0037943.

###### Paratype.

*Ptenosleucopoda* (Argidae): DHJPAR0052901. Depository: CNC.

###### Etymology.

*Proteropsvickifunkae* is named in honor of Vicki Funk (RIP) attending the international NSF-funded planning meeting for the All Taxa Biodiversity Inventory (ATBI) of Terrestrial Systems, and contributing her wisdom to the planning that was the founding of Costa Rica’s national BioAlfa today.

**Figure 360.**
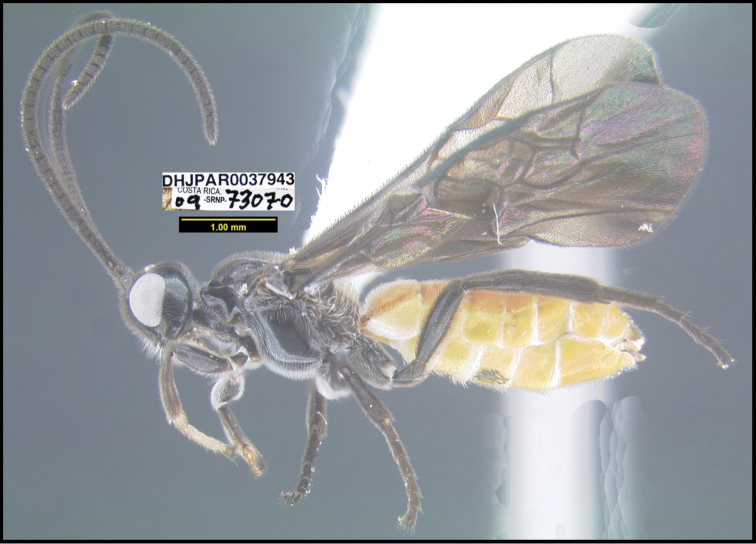
*Proteropsvickifunkae*, holotype.

##### Michener

Taxon classificationAnimaliaHymenopteraBraconidae

Sharkey gen. nov.

http://zoobank.org/83EAC616-ABC8-411B-936C-E79167088965

Figure 361

###### Type species.

*Michenercharlesi* Sharkey, sp. nov.

###### Diagnosis.

Similar to *Proterops* but differing in the following aspects: 1. *Michener* with tergum 1 with a pair of prominent, precurrent, longitudinal carinae. *Proterops* with tergum 1 lacking carinae. 2. *Michener* with tergum 1 slightly narrowed at midlength and almost 3 × longer than wide at narrowest point. *Proterops* with tergum 1 not narrowed at midlength, rather narrowest at base or apex; tergum 1 shorter, usually ca. as long as wide but always less than 2 × longer than wide. 3. *Michener* with tergum 2 short, half the length of tergum 3, measured along midline. *Proterops* with tergum 2 ca. the same length as tergum 3, measured along midline. 4. *Michener* with laterotergites of tergum 1 densely setose. *Proterops* with laterotergites sparsely setose.

###### Description.

***Head***. Occipital carina absent; antenna with 34 flagellomeres; first flagellomere without patch of basiconic sensillae; maxillary palpus with four segments; labial palpus with three segments; mandible not twisted for scissor-like function; base of mandible not depressed and without microsculptured area set off by acute transverse ridge. ***Wings***. Vein 1M of forewing evenly curved; vein 1RS of forewing present; venation complete; R of hind wing not sharply curved posteriad near apex; three hamuli in one cluster. ***Mesosoma***. Propleuron without longitudinal carina; pronotum with weak dorsolateral pit (subpronope); epicnemial carina absent; precoxal depression absent; notaulus smooth, weakly indicated as a smooth line, posterior scutellar depression absent; propodeum smooth; tarsal claws simple, without basal tooth or lobe. ***Metasoma***. Tergum 1 with a pair of prominent, precurrent, longitudinal carinae; tergum 1 narrowest at midlength and 2.8 × longer than wide at narrowest point; tergum 2 short less than half the length of tergum 3, measured along the midline. Female hypopygium without dense patch of setae apically

###### Distribution.

Known only from the type locality of the type species in Costa Rica.

###### Etymology.

Named with profound admiration for the lifetime achievements of Professor Charles Michener (RIP) of the University of Kansas, Lawrence, and the many professional and personal forms of support offered to Janzen’s entire professional academic career, as well as to many others.

**Figure 361.**
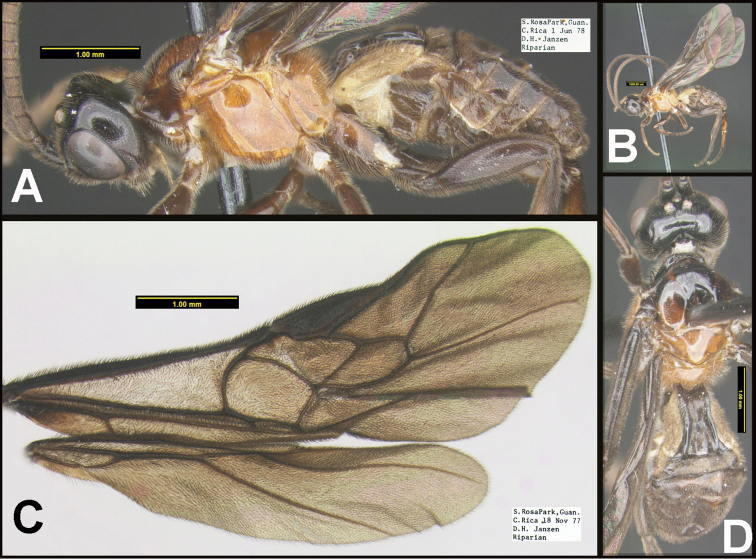
*Michenercharlesi***A, B** holotype **C, D** paratype.

##### Michener charlesi

Taxon classificationAnimaliaHymenopteraBraconidae

Sharkey sp. nov.

http://zoobank.org/3496A23B-C598-4F2C-BC27-58E23D5BECB9

Figure 361

###### Diagnosis.

Length 5.8 mm. Other details are illustrated in Fig. 361. Since there are no congeneric species, the generic diagnosis above functions as a species diagnosis as well.

###### Holotype ♀.

Guanacaste, Área de Conservación Guanacaste, Sector Santa Rosa, Bosque San Emilio, 10.84389, -85.61384, 300 meters, 01/vi/1978, D. H. Janzen, Depository: CNC.

***Host data*.** None.

###### Paratype.

♀, same data as holotype, except 18/xi/1977.

###### Etymology.

See generic etymology above.

## Chapter 11: Rhysipolinae

Rhysipolinae can be differentiated from all other New World braconid subfamilies by having the following suite of characters: oral cavity circular dorsally and labrum concave (cyclostome); occipital carina not meeting hypostomal carina; rather, meeting, or almost meeting, base of mandible; epicnemial carina present; propodeum lacking pair of lateral spines; second submarginal cell of forewing present and 4-sided, i.e., 2RS vein meeting (RS+M)b or junction of (RS+M)a and (RS+M)b. *Rhysipolis* may be differentiated from the other New World genera of Rhysipolinae using the key that follows.

Little is known about their life histories. They are solitary, koinobiont, ectoparasitoids of late instar leaf-miners and leaf-tiers, and that they move from the host leaves and/or otherwise feed and/or pupate elsewhere (Shaw 1983; Shaw and Sims 2015). Here we offer the first New World host record of a non-leaf-mining caterpillar, a member of Gelechiidae. For the Rhysipolinae NJ tree, see Suppl. material 9.

### Key to New World genera of Rhysipolinae

**Table:** 

1	A. Wide crenulate groove at base of propodeum present	*** Cantharoctonus ***
–	B. Wide crenulate groove at base of propodeum absent	**2**
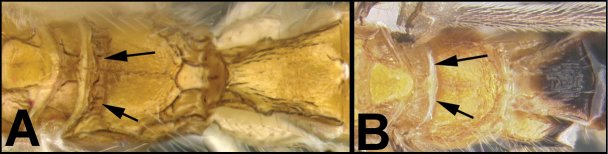		
2(1)	A. Setal comb present on medial (inner) side of apex of hind tibia	*** Pseudorhysipolis ***
–	B. Setal comb lacking on medial (inner) side of apex of hind tibia	*** Rhysipolis ***
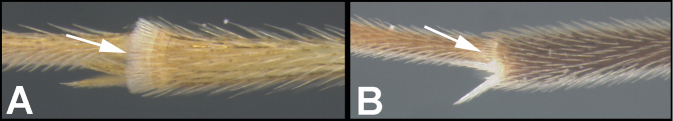		

#### *Pseudorhysipolis* Scatolini, Penteado-Dias & van Achterberg, 2002

There are ten previously described species, all from the neotropics (Scatolini et al. 2002; Papp 2012); here we add two more. Little is known about their life histories. One species has been reared from a species of Oecophoridae (Lepidoptera).

##### Pseudorhysipolis luisfonsecai

Taxon classificationAnimaliaHymenopteraBraconidae

Sharkey sp. nov.

http://zoobank.org/E5AD6085-E56E-4663-AD58-1A02349F692D

Figure 362

###### Diagnostics.

BOLD:ACW5686. Consensus barcode. GTATTATATTTTTTATTTGGAATTTGATCTGGTATAGTAGGTTTATCTATGAGTTTAATTATTCGTTTAGAGTTAGGTATACCTGGTAGTTTATTATTTAATGATCAGATTTATAATACTATAGTTACAGCTCATGCTTTTATTATAATTTTTTTTATAGTTATACCTGTAATGATTGGGGGGTTTGGTAATTGATTAGTTCCATTAATATTGGGGGCTCCTGATATAGCTTTCCCTCGTATGAATAATATAAGATTTTGATTATTAATTCCTTCTTTAATTTTATTATTTTTAAGGGGTTTAGTAAATGTTGGGGTAGGTACTGGGTGAACTGTTTATCCTCCTTTATCTTCTTCTATAGGTCATAGAGGTATTTCTGTTGATTTGGCTATTTTTTCTTTACATTTAGCTGGTATTTCTTCAATTATAGGAGCTATTAATTTTATTTCAACAATTTTTAATATATGTTTATATTCAATTAATATAGATCAAATTAGTTTATTTGTTTGATCTATTTTGATTACTGCTTTTTTATTATTATTATCTTTACCTGTCTTGGCAGGG------------------------------------------------------------------------. Keys to *P.areolaris* in Scatolini et al. (2002), but the antennal scape and pedicel are two different colors in *P.areolaris*, whereas they are the same color in *P.luisfonsecai*.

###### Holotype ♀.

Guanacaste, Sector San Cristobal, Estación San Gerardo, 10.88, -85.389, 575 meters, 23/xi/2013, Malaise trap. Depository: CNC.

***Host data*.** None.

***Holotype voucher code*.**BIOUG22620-D04.

###### Paratypes.

None.

###### Etymology.

*Pseudorhysipolisluisfonsecai* is named to honor Sr. Luis Fonseca for opening all of our eyes to the predator-prey relationship between ACG jaguars and nesting sea turtles.

**Figure 362.**
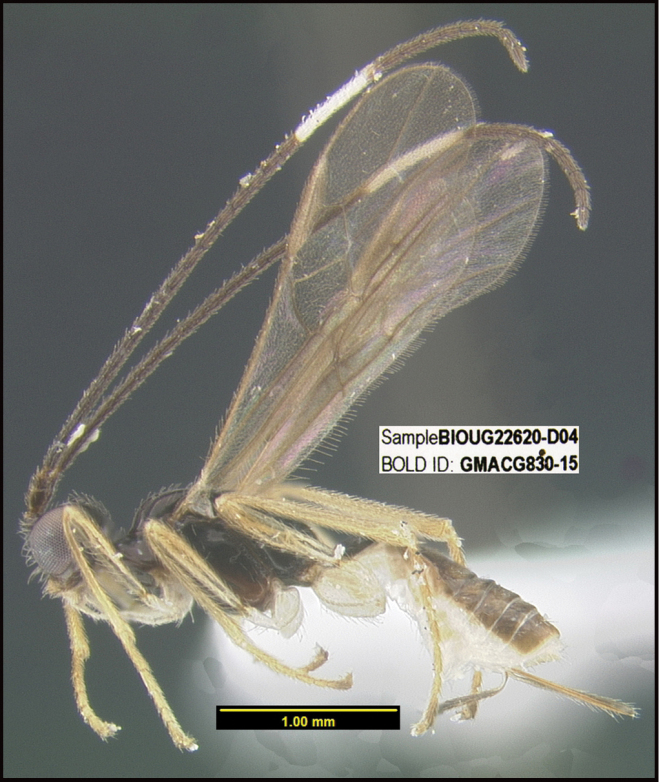
*Pseudorhysipolisluisfonsecai*, holotype.

##### Pseudorhysipolis mailyngonzalezae

Taxon classificationAnimaliaHymenopteraBraconidae

Sharkey sp. nov.

http://zoobank.org/0B153975-1F50-44CD-B266-233128E90A59

Figure 363

###### Diagnostics.

BOLD:ACL1561. Consensus barcode. TGTTTTGTATTTTATTTTTGGTATATGAGCTGGAATAGTTGGTTTATCTATAAGATTAAT-TATTCGATTAGAATTAGGGGTATCTGGAAGATTATTAGGGAATGATCAAAT------TTATAATACTATTGTTACATCTCATGCTTTTGTAATAATTTTTTTTATAGTTATACCTATTATATTAGGAGGATTTGGTAATTGATTAATTCCTTTAATATTAGGGGCTCCTGATATAGCATTTCCTCGAATAAATAATATAAGATTTTGATTAT-----------------TGATTCCATCATTAATTTTATTATTTTTAAGTAGA------------TCAATAAATTTAGGAGC-----------------------------------TGGAACGGGGTGAACTATATATCCTCCTTTATCT-------------------TCAAGAATTGGT--------CATAGAGGAATATCTGTTGATTTAACAATTTTT--TCTTTACATTTAGCTGG---TTGTTCTTCTATTATAGGATCAATTAATTTTATTTGTACAATTTTTAATATA--AAAATTAATTTT---TTAAAAATAGAACAATTAAGTTTATTTGTTTGGTCAGTTTTAATTACAACAATTTTATTATTATTATCTTTACCAGTTT--TAGCTGGTGCTATTACTATATTATTAACAGATCGTAATTTAAATACATC------TTTTTTTGATTTTTCAGGTGGTGGTGATCCAATTTTATTTCAACATTTATTT. Keys to *P.fuscaoicalis* in Scatolini et al. (2002) but lacks the swollen maxillary palpomeres of that species.

###### Holotype ♀.

Guanacaste, Sector Santa Rosa Bosque San Emilio, 10.8438, -85.6138, 300 meters, 10/xii/2012, Malaise trap. Depository: CNC.

***Host data*.** None.

***Holotype voucher code*.**BIOUG09442-D01.

###### Paratypes.

Both Malaise-trapped. BIOUG09441-H03, BIOUG09740-B12. Depository: CNC.

###### Etymology.

*Pseudorhysipolismailyngonzalezae* is named to honor Dr. Mailyn Gonzalez of the Humboldt Institute, Bogota, Colombia, for instigating the growing mutualism between Costa Rica’s BioAlfa and Colombia’s interest in biodevelopment of its massive biodiversity.

**Figure 363.**
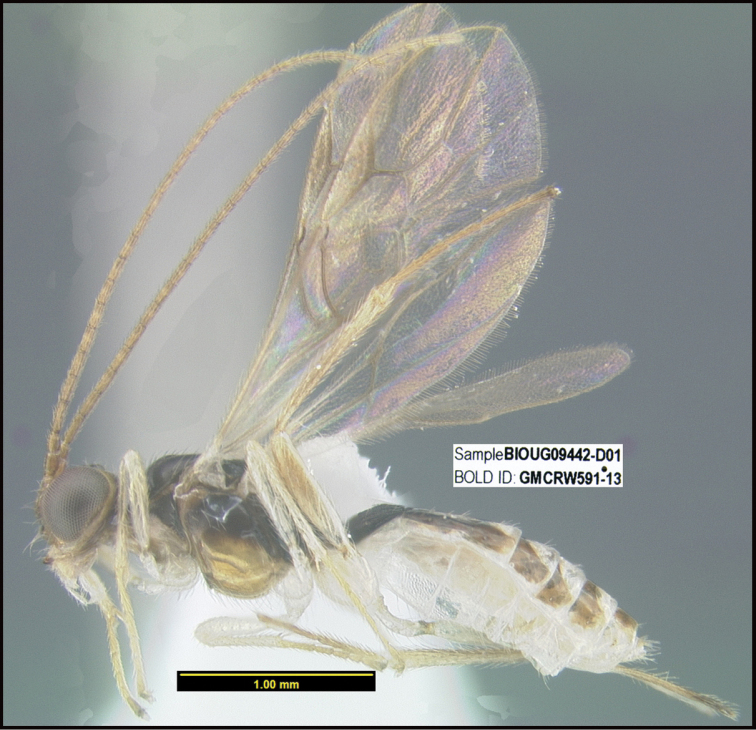
*Pseudorhysipolismailyngonzalezae*, holotype.

#### *Rhysipolis* Foester, 1862

Little is known about their life histories. Shaw (1983) stated that they are solitary, koinobiont, ectoparasitoids of late instar leaf-miners and leaf-tiers and that they change leaves and/or otherwise feed and/or pupate elsewhere. Here we have the first host record on a non-leaf-mining caterpillar, a member of Gelechiidae. There are two previously described Neotropical species, one from Mexico and one from Brazil.

##### Rhysipolis julioquirosi

Taxon classificationAnimaliaHymenopteraBraconidae

Sharkey sp. nov.

http://zoobank.org/149661B5-A9BE-4465-9A94-92BE055A7B47

Figures 364 , 365

###### Diagnostics.

BOLD:AAT9029. Consensus barcode. TGTTTTGTATTTTTTATTTGGAATATGATCAGGTATAGTTGGATTATCAATAAGTTTGATTATTCGATTAGAATTAGGGATACCTGGAAGTTTAATTGGTAATGATCAAATTTATAATACAATGGTTACAGCTCATGCTTTTATTATAATTTTTTTTATAGTTATACCAATTATAATTGGGGGATTTGGAAATTGATTAGTTCCATTAATATTAGGGGCTCCTGATATAGCTTTCCCTCGTATAAATAATATAAGGTTTTGATTATTAATTCCTTCTTTAATTTTATTATTTTTAAGAAGAATTGTAAATGTTGGGGTTGGTACTGGTTGAACAGTTTATCCGCCCCTATCTTCAATATTAGGTCATAGGGGTATTTCTGTAGATTTGGCTATTTTTTCTTTACACTTGGCTGGTGTTTCTTCTATTATAGGAGCTATTAATTTTATTTCTACAATTTTTAATATATGTATATAYTCAATTAAATTAGATCAACTTAGTTTGTTTGTTTGATCTATTTTAATTACTGCAATTTTATTATTATTATCTTTRCCTGTTTTGGCAGGGGCTATTACTATATTATTGACAGATCGTAATTTGAATACTACTTTTTTTGATTTTTCTGGTGGGGGGGATCCAATTTTATTTCAACATTTATTT. Neither of the two described Neotropical species, *R.annulator* Achterberg & Pentiado-Dias, 2002, and *R.chiapas* Spencer, 1999, have deeply infuscated wings.

###### Holotype ♀.

Alajuela, Sector San Cristobal, Sendero Huerta, elevation: 10.93050, -85.37223, 527 meters, caterpillar collection date: 13/vi/2013, wasp eclosion date: 02/vii/2013. Depository: CNC.

***Host data*.***Dichomeris* Janzen273 (Gelechiidae) feeding on *Mikaniacordifolia* (Asteraceae).

***Host caterpillar and holotype wasp voucher codes*.** 13-SRNP-3016, DHJPAR0053002. The host caterpillar rolls the food plant leaf to make a “nest,” and the wasp larva spins a dull white, thin-walled cocoon in the host’s pupal chamber (Fig. 363); we have no observations as to where the wasp larva was during its development.

###### Paratypes.

Hosts = seven rearing records from *Dichomeris* Janzen273, *Anacampsis* Janzen301 (both Gelechiidae), elachJanzen01 Janzen397 (Depressariidae). DHJPAR0041996 DHJPAR0041520. DHJPAR0043148, DHJPAR0049862, DHJPAR0054828, DHJPAR0054834. Depository: CNC.

###### Etymology.

*Rhysipolisjulioquirosi* is named in honor of Julio Quiros of Guanacaste Province, Costa Rica for his brave and diligent forest warden work in support of the foundling ACG in the 1980–1990’s.

**Figure 364.**
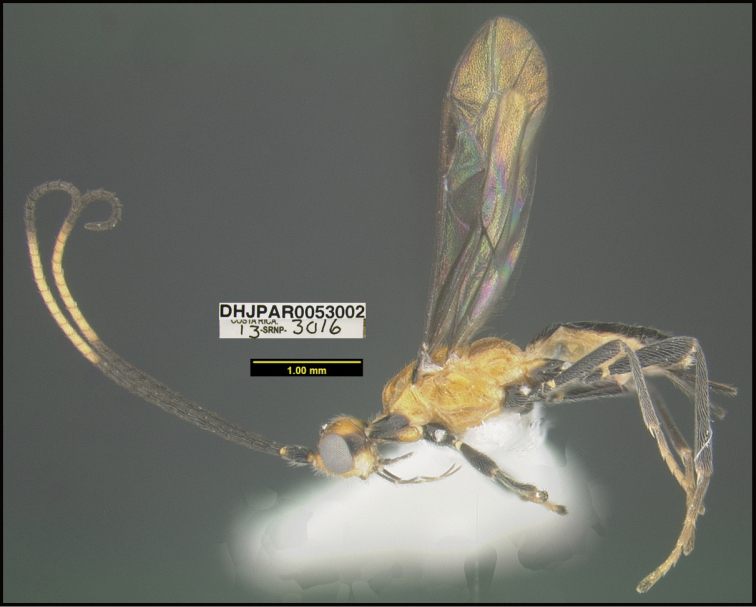
*Rhysipolisjulioquirosi*, holotype.

**Figure 365.**
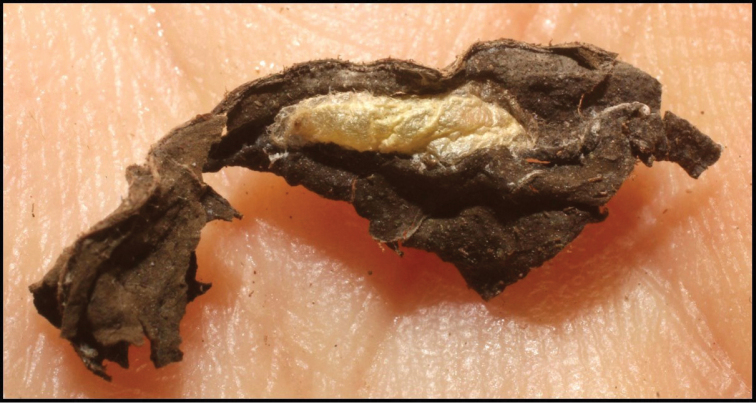
Cocoon of holotype of *Rhysipolisjulioquirosi*.

## Chapter 12: Rogadinae

Rogadinae is a large, cosmopolitan subfamily. The New World genera of Rogadinae may be distinguished using the key that follows. A total of 231 species is recorded from the Neotropical realm and 38 from Costa Rica, not including those described below. Members of all genera are koinobiont endoparasitoids of caterpillars from a wide range of families. Some are gregarious but this applies to only two described here, one species of *Aleiodes* and one of *Triraphis*. For the Rogadinae NJ tree, see Suppl. materia 10.

### Key to the New World genera of Rogadinae

Note: *Macrostomion* is not included in this key as it was in Shaw 1997 and is not known from the New World. *Bioalfa* and *Hermosomastax* gen. nov. are similar and belong to the same genus group.

**Table:** 

1	A. Body length (not including appendages) less than 3 mm	**2**
–	B. Body length more than 3 mm	**7**
2(1)	A. Metasoma with sclerotized carapace formed by terga 1–3; Chile	*** Gondwanocentrus ***
–	B. Metasoma with sclerotized carapace formed by terga 1–4. Widespread	**3**
–	C. Metasoma lacking sclerotized carapace; five or more sclerotized terga visible in dorsal view; CC. rarely 4^th^ and 5^th^ terga desclerotized but fully exposed; Neotropical	**5**
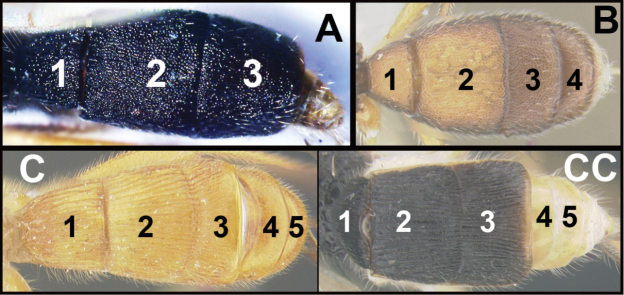		
3(2)	A. r-m crossvein of forewing present (though often weak), closing second submarginal cell	*** Stiropius ***
–	B. r-m crossvein of forewing absent, second submarginal cell of forewing not closed apically	**4**
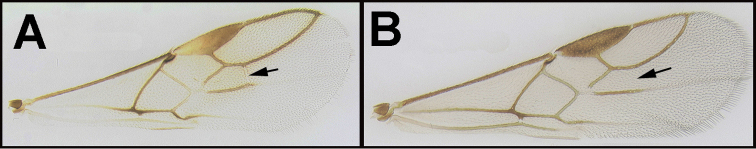		
4(3)	A. Malar space (distance between base of mandible and eye) relatively short and malar suture present between eye and mandible; Neotropical	*** Choreborogas ***
–	B. Malar space relatively long and malar suture absent; Nearctic	*** Polystenidea ***
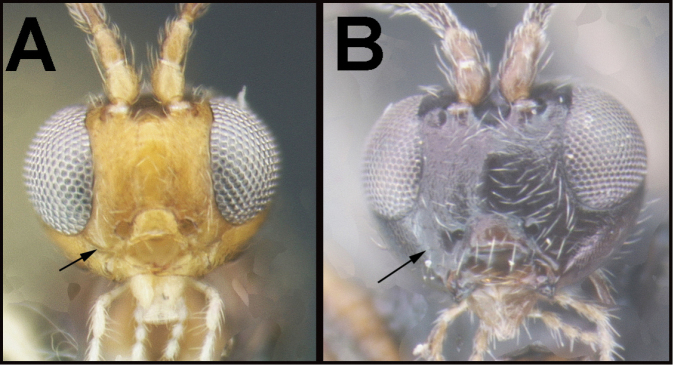		
5(2)	A. Second submarginal cell of forewing present; apical abscissa of forewing vein M relatively straight	**6**
–	B. Second submarginal cell of forewing absent; apical abscissa of forewing vein M sinuate; Neotropical and recently discovered in Papua New Guinea (Braet 2016)	*** Jannya ***
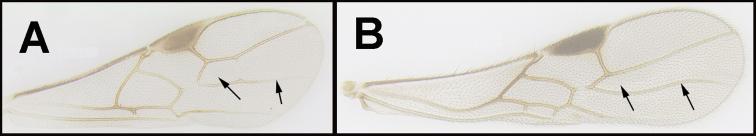		
6(5)	A. Antenna not emanating from a protruding shelf; eyes emarginate; head wider than long	*** Bulborogas ***
–	B. Antenna emanating from a protruding shelf; eyes not emarginate; head at least as long as wide	*** Conobregma ***
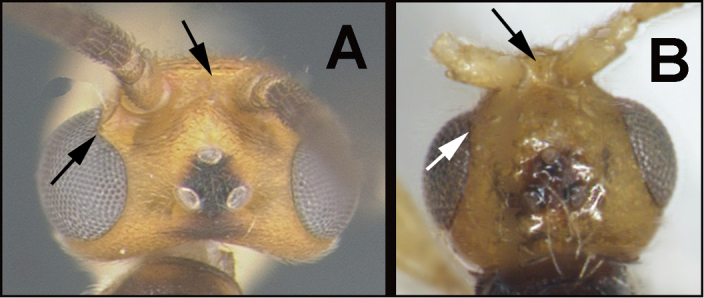		
7(1)	A. Tarsomeres 2‒4 of front and middle legs reduced and compact; tarsomeres 2‒4 each as short as or shorter than wide, 5^th^ tarsomere as long as or longer than tarsomeres 2‒4 combined	**8**
–	B. Tarsomeres 2‒4 of front and middle legs not reduced and compact; tarsomeres 2‒4 each longer than wide, 5^th^ tarsomere shorter than tarsomeres 2‒4 combined	**9**
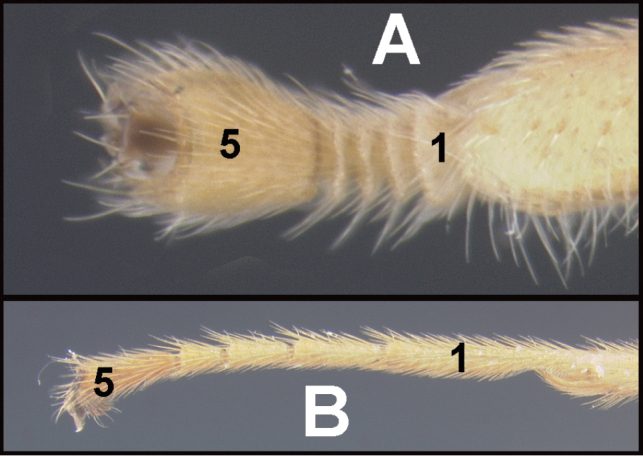		
8(7)	A. Hind wing vein R much longer than high and RS weakly curved or absent; widespread.	*** Yelicones ***
–	B. Hind wing vein R much higher than long and RS strongly curved; Neotropical	*** Pseudoyelicones ***
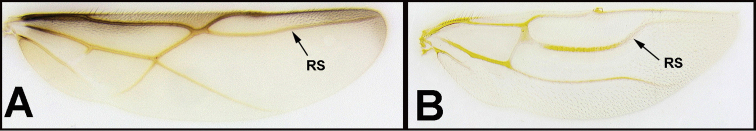		
9(7)	A. Membrane of forewing first submarginal evenly setose; widespread	**10**
–	B. Membrane of forewing first submarginal with a distinct windowlike patch devoid of setae; Neotropical	*** Cystomastax ***
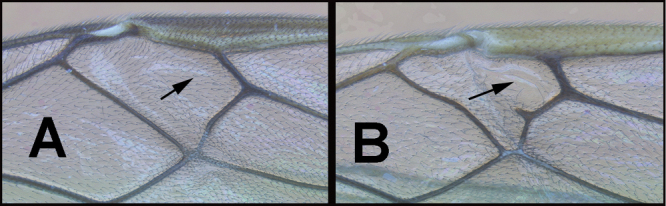		
10(9)	A. m-cu crossvein of hind wing long and conspicuous and directed towards base of wing; ovipositor as long as middle tibia or longer	*** Clinocentrus ***
–	B. m-cu crossvein of hind wing absent or if present then shorter and not directed towards the base of the wing; ovipositor usually shorter than middle tibia (variable in *Triraphis*)	**11**
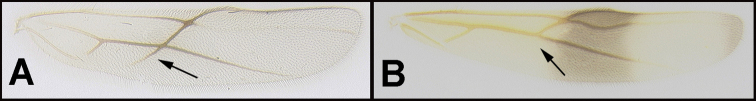		
11(10)	A. 2RS of forewing nearly parallel with r-m, thereby forming a roughly rectangular or subquadrate second submarginal cell	**12**
–	B. 2RS of forewing angled towards base of wing posteriorly, thereby forming a roughly trapezoidal second submarginal cell	**13**
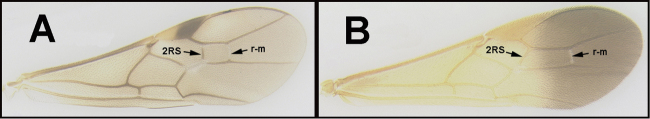		
12(11)	A. Second submarginal cell of forewing ca. as long as high. AA. Hind trochantellus elongate, 2 or more × longer than trochanter in ventral view; tarsal claws without pectination; Neotropical, rare	*** Heterogamus ***
–	B. Lacking one or more of the above characters; most specimens are as follows: second submarginal cell longer than high. BB. Hind trochantellus not elongate, less than 2 × longer than trochanter in ventral view; tarsal claws usually with pectination; widespread, common	***Aleiodes* (most species, ~90%)**
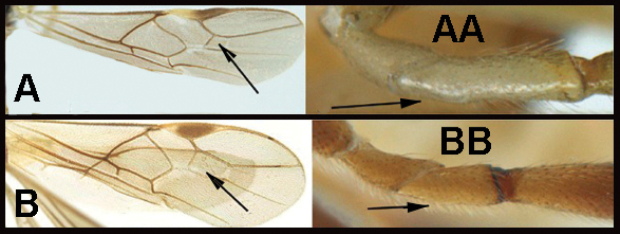		
13(11)	A. Hind tibial spurs mostly lacking setae and curved. AA. Hypopygium large; Neotropical, rare	**14**
–	B. Hind tibial spurs setose and relatively straight. BB. Hypopygium normal; widespread, common	**15**
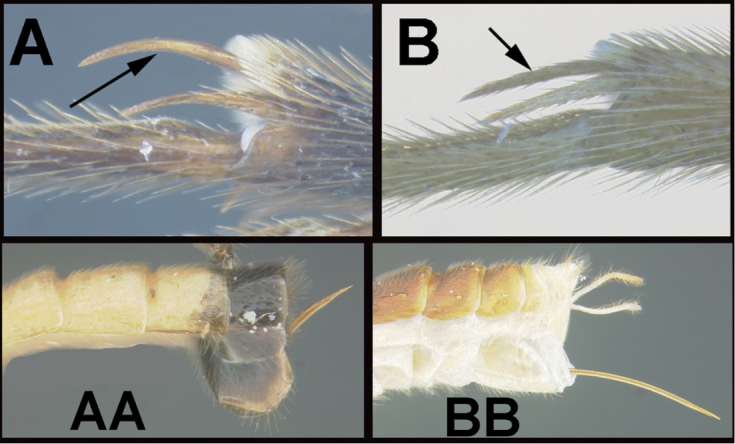		
14(13)	A. Tarsal claws simple, swollen basally but lacking a distinct lobe. AA. Hind femur swollen apically	***Hermosomastax* gen. nov.**
–	B. Tarsal claws with a squared or slightly acute basal lobe. BB. Hind femur not swollen apically	***Bioalfa* gen. nov.**
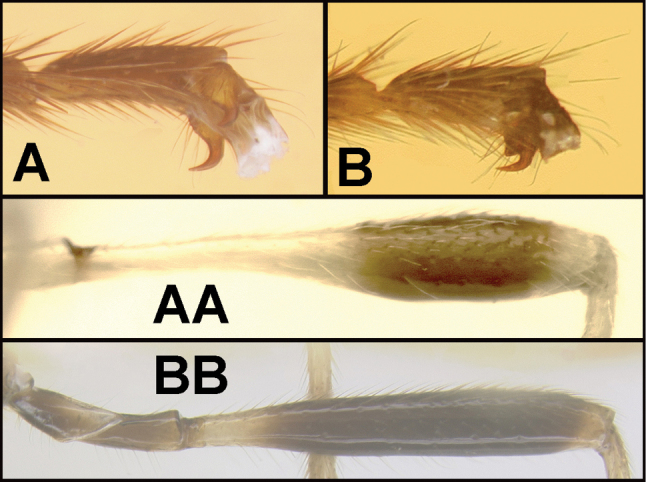		
15(13)	A. Tarsal claws lacking basal lobe, but often with basal pectin-like row of short spines	***Aleiodes* (~10% of species)**
–	B. Tarsal claws with well-defined sharp tooth or teeth	*** Triraphis ***
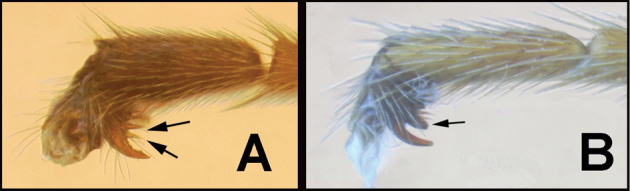		

#### Bioalfa

Taxon classificationAnimaliaHymenopteraBraconidae

Sharkey gen. nov.

http://zoobank.org/57F76CD5-303D-45E8-AEF4-FA7D41E48B29

**Figure 366.**
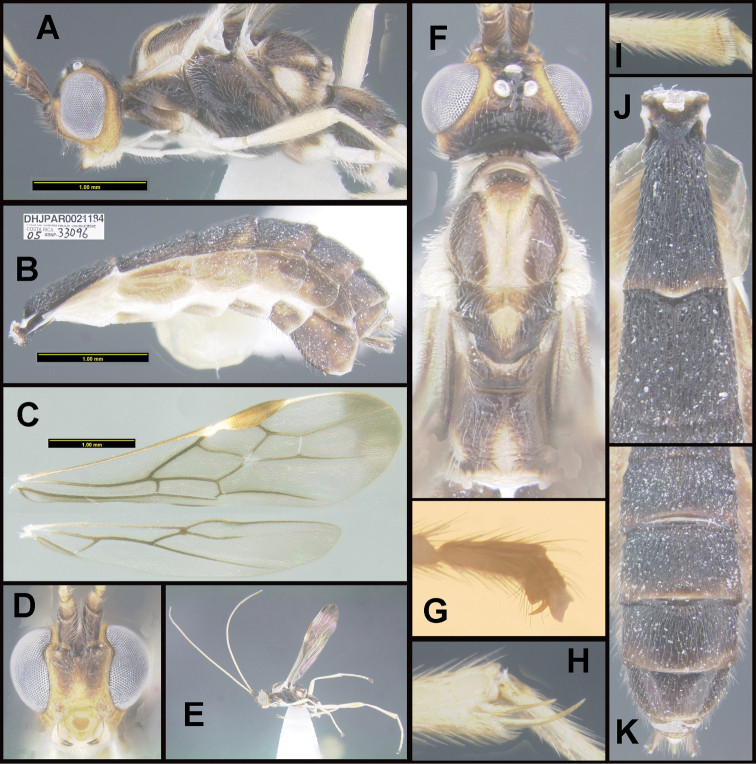
*Bioalfapedroleoni***A, B, D–H** holotype **C** paratype **A** lateral habitus **B** lateral habitus **C** wings **D** anterior head **E** dorsal head, mesosoma and terga 1–2 **F** tarsal claw **G** dorsal metasoma **H** hind tibial spurs.

**Figure 367.**
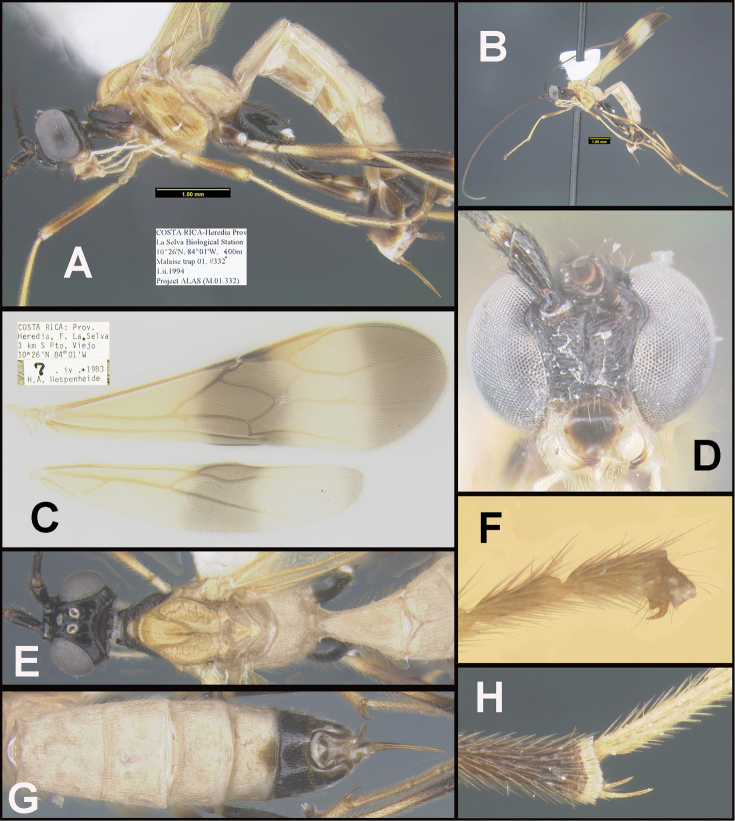
*Bioalfaalvarougaldei* holotype **A** lateral head and mesosoma **B** lateral metasoma **C** wings **D** anterior head **E** lateral habitus **F** dorsal head and mesosoma **G** tarsal claw **H** hind tibial spurs **I** apex of hind tibia **J** metasomal terga 1 and 2 **K** apex of metasoma, dorsal view.

**Figure 368.**
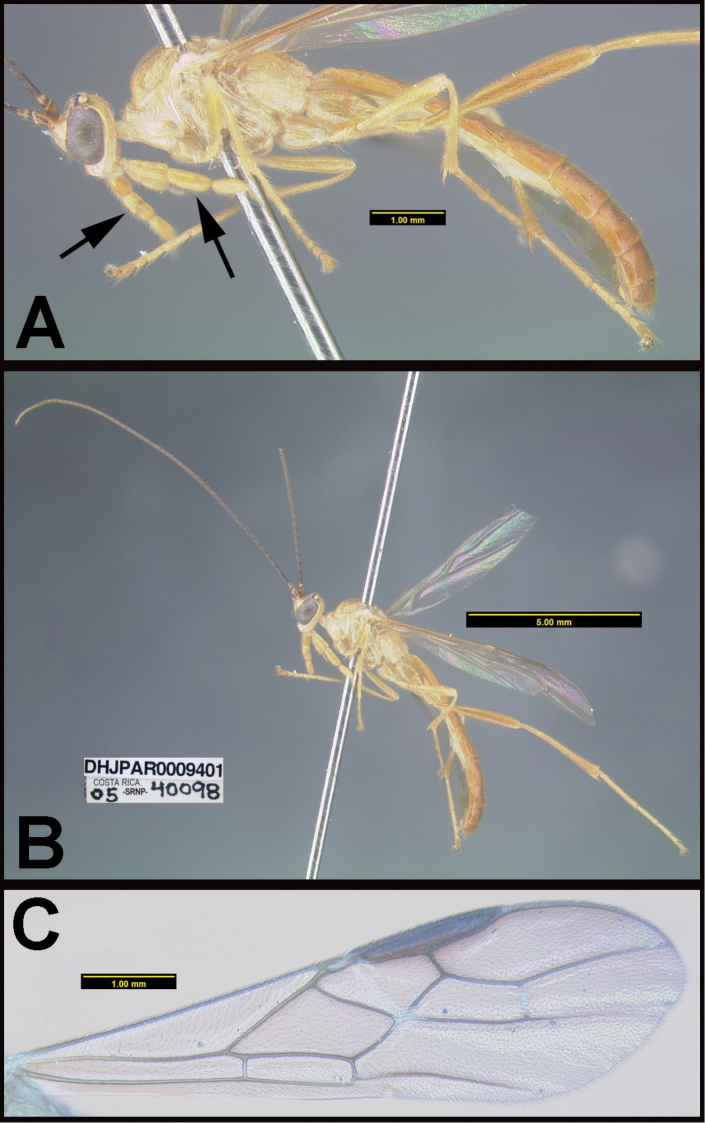
*Bioalfarodrigogamezi*: **A** lateral habitus, arrows point to maxillary and labial palpi **B** lateral habitus **C** forewing.

Figures 366 , 367 , 368

##### Type species.

*Bioalfapedroleoni* Sharkey, sp. nov.

##### Description.

**Female: *Head***. Antenna length approximately 1.5 × forewing length, with ca. 48 flagellomeres. Terminal flagellomere acuminate apically. Median flagellomeres transverse, ca. 2 × as long as wide. Face with oblique and transverse striation. Clypeus shallow, not protruding. Malar suture absent. Eye glabrous, large, and deeply emarginate, occupying entire height of head resulting in very small malar space. Eye strongly emarginated opposite antennal socket. Anterior tentorial pit large, very close to eye. Maxillary palp with six segments, all slender in female. Occipital carina complete dorsally, without dorsolateral posterior bulge, connecting to hypostomal carina ventrally. ***Mesosoma***. Smooth and shiny. Notauli deeply impressed, meeting at the midline and 2/3 length of mesonotum; a single weak medial depression continues to the scuto-scutellar suture. Mesoscutal mid-pit absent. Scutellar sulcus wide with single strong mid-longitudinal carina. Epicnemial carina complete. Mesopleuron smooth with mostly widely spaced minute setiferous punctures; precoxal sulcus wide, almost entirely smooth with a few weak transverse carinae at midlength. Median area of metanotum without mid-longitudinal carina. Propodeum with one precurrent median carina or two weak diverging carinae defining a narrow, elongate medial area, otherwise, finely rugose with or without weak posterolateral depressions set off by weak carinae. ***Forewing*.** Crossvein m-cu weakly-curved, forming a slightly obtuse angle with 1Cub. First submarginal cell evenly setose. Second submarginal cell approximately twice as long as wide. 2RS and r-m not parallel. Crossvein 1cu-a postfurcal. First subdiscal cell rectangular. Subbasal cell evenly setose. ***Hind wing*.** Vein R short, as long as wide to ca. 2 × as long as wide. Veins M+Cu and 1-M approximately equal length. Vein RS strongly curved. Vein m-cu absent. Base of hind wing evenly setose. Legs shining, with sparse long setae. ***Legs*.** Apex of hind tibia with comb of specialized setae medially. Mid and hind tibial spurs strongly curved and glabrous. Claws with squared or slightly acute basal lobe. ***Metasoma*.** Tergites 1–3 finely longitudinally striate. First metasomal tergite elongate, strongly and angularly widened in front of sub-basal constriction; with dorsal carinae uniting almost immediately and forming a weak carina along midline almost indistinguishable from longitudinal striae. Second metasomal tergite with midlongitudinal carina arising from small, transverse basal triangular area. Tergites 3–5 with sharp lateral crease. Hypopygium large, ventrally moderately to strongly convex. Ovipositor straight or curved ventrally. All four known species have patterned wings and colorful (for Rogadinae) bodies, suggesting diurnal activity. **Male**: As in the female but with swollen labial and maxillary palpi.

##### Biology.

Caterpillar hosts are known for two species, which are both external leaf-feeders in the family Uraniidae.

##### Distribution.

Neotropical rainforest, Costa Rica, Amazonian Colombia, and Peru.

##### Remarks.

Morphologically, the new genus displays a mixture of character states associated with the *Colastomion*-*Cystomastax* group of genera, i.e., the curved, glabrous tibial spurs, and the enlarged hypopygium. *Bioalfa* differs from all other genera in this group by the presence of squared to slightly acute basal lobes on the tarsal claws. DNA data (Quicke et al. unpublished) place it deeply within the *Colastomion*-*Cystomastax* complex and separate from other New World members.

##### Etymology.

The new genus *Bioalfa*, from “bioalfabetizacion,” is dedicated to Costa Rica’s brave and optimistic new concept of creating itself to be bioliterate about all of its wild Eukaryota biodiversity, through the combination of national sweat equity, international collaborations, political willpower, and DNA barcoding (c.f. https://news.mongabay.com/2020/04/bold-project-hopes-to-dna-barcode-every-species-in-costa-rica/ and https://ibol.org/barcodebulletin/features/how-a-tropical-country-can-dna-barcode-itself/).

#### Bioalfa pedroleoni

Taxon classificationAnimaliaHymenopteraBraconidae

Sharkey sp. nov.

http://zoobank.org/BD86B780-2FA9-459C-8B14-E15EB5989B28

Figure 366

##### Diagnostics.

BOLD:AAX3381. Consensus barcode. TGTTTTATATTTTATTTTTGGAATTTGATCTGGTATATTAGGTTTATCTATAAGAATTATTATTCGAATAGAATTAAGTAATCCTGGTGGATTAATTAAAAATGATTATATTTATAATTCAATAGTTACTGCTCATGCTTTTATTATGATTTTTTTTATAGTTATACCAATTATATTAGGGGGATTTGGAAATTGATTAATTCCTATAATATTAGGATCTCCTGATATAGCTTTTCCACGAATAAATAATATAAGATTTTGATTATTAATTCCTTCATTATTTTTATTATTAATAAGTTCATTTGTTAATATTGGTGTTGGTACTGGTTGAACTATATATCCTCCATTATCTTCTTTATTGGGTCATAGTGGATTTTCTGTTGATATAGCTATTTTTTCTTTACATTTAGCTGGGATATCTTCTATTATGGGGTCTATTAATTTTATTTCTACAATTTTTAATATAAAATTATATTCTTTAAATATAGATCAATTAAATTTATTTATTTGGTCAATTTTAATTACTGTAATTTTATTACTTTTATCATTACCTGTTTTAGCAGGTGCTATTACTATATTATTTACTGATCGTAATTTAAATACTACTTTTTTTGATTTTTCTGGTGGGGGGGATCCTATTTTATTTC. Differing from the five other known species (two undescribed and not included here) in many characters including coloration, e.g., it is the only species with one melanic band on the forewing, a terminal band. The others have two melanic bands, one at midlength and one terminal. Body length 5.5 mm. Other details can be gleaned from the images in Fig. 366.

##### Holotype ♀.

Alajuela, Área de Conservación Guanacaste, Sector Rincon Rain Forest, Laureles, 10.933, -85.253, 95 meters, caterpillar collection date: 5/viii/2005, wasp eclosion date: 18/viii/2005. Depository: CNC.

***Host data*.** The host caterpillar is *Syngriadruidaria* (Uraniidae) feeding externally on new full-sized leaves of *Amphilophiumcrucigerum*, a woody vine in the Bignoniaceae in the dry-rain forest interface.

***Host caterpillar and holotype wasp voucher codes*.** 07-SRNP-42145, DHJPAR0021184.

##### Paratypes.

None.

##### Etymology.

*Bioalfapedroleoni* is named in honor of Professor Pedro Leon of the Universidad de Costa Rica for his founding role in the initiation of Área de Conservación Guanacaste in 1986 and his founding role in the initiation of BioAlfa by alerting Costa Rica’s Ambassador to the United States, Dr. Román Macaya, to the potential of DNA barcoding Costa Rica’s biodiversity in 2017.

#### Bioalfa alvarougaldei

Taxon classificationAnimaliaHymenopteraBraconidae

Sharkey sp. nov.

http://zoobank.org/A3997C26-E20B-4215-8E92-EEFE94BA79DF

Figure 367

##### Diagnosis.

Differing from the five other known species (two undescribed and not included here) in many characters including a hypopygium that is much more convex than the others. Body length 7.1 mm. Other details can be gleaned from the images in Fig. 367.

##### Holotype ♀.

Costa Rica, Heredia Prov., La Selva Biological Station, 10.44, -84.01, 100 meters, 1/ii/1994, Malaise trap. Depository: CNC.

***Host data*.** None.

***Holotype voucher code*.** None.

##### Paratypes.

4♀♀, all from La Selva Biological Station on the following dates: 1/ix/1993, 1/iv/1985, iv-v/1993, iv/1993. Three specimens deposited in CNC, one in Paul Hanson’s collection at the University of Costa Rica.

##### Etymology.

*Bioalfaalvarougaldei* is named in honor of Sr. Alvaro Ugalde (RIP) in recognition of his major and critical support and role in the founding of Parque Nacional Santa Rosa and its subsequent development as Área de Conservación Guanacaste, and eventual stimulus for BioAlfa, the concept of facilitating Costa Rica to become bioliterate.

#### Bioalfa rodrigogamezi

Taxon classificationAnimaliaHymenopteraBraconidae

Sharkey sp. nov.

http://zoobank.org/2D1EFBAA-F204-44BF-8FCF-E532C06CA36D

Figure 368

##### Diagnosis.

Differing from all other species of *Bioalfa* in its lack of color and lack of banding on the forewing (Fig. 368).

##### Holotype ♂.

Costa Rica, Alajuela, Área de Conservación Guanacaste, Sector Rincon Rain Forest, Sendero Parcelas, 10.9077, -85.29137, 375 meters, caterpillar collection date: 10/i/2005, wasp eclosion date: 13/i/2005. Depository: CNC.

***Host data*.** The host caterpillar is *Hypena* Poole17 (Erebidae) feeding on *Ureracaracasana* (Urticaceae).

***Host caterpillar and holotype wasp voucher codes*.** 05-SRNP-40098, DHJPAR0009401.

##### Paratypes.

None.

##### Note.

This is the only known male specimen of *Bioalfa*. As in related rogadine genera, it has swollen labial and maxillary palpi.

##### Etymology.

*Bioalfarodrigogamezi* is named in honor of Professor Rodrigo Gámez, co-founder and executor of the Instituto Nacional de Costa Rica (INBio) in 1989, and co-founder of Área de Conservación Guanacaste in 1985.

#### Hermosomastax

Taxon classificationAnimaliaHymenopteraBraconidae

Quicke gen. nov.

http://zoobank.org/D6DF1F96-A697-48CB-B9DF-378D051AB3EB

##### Type species.

*Hermosomastaxclavifemorus* Quicke, sp. nov.

##### Description.

**Female: *Head***. Antenna approximately as long as forewing, with ca. 40 flagellomeres. Terminal flagellomere acuminate. Median flagellomeres very elongate, > 3 × longer than wide. Face protruding. Clypeus shallow, not protruding. Malar suture absent. Eyes glabrous, distinctly emarginated opposite antennal sockets. Anterior tentorial pits large, very close to eye. Maxillary palp with six segments. Occipital carina complete dorsally and ventrally, with dorsolateral posterior angulation and bulge, connecting to hypostomal carina ventrally. ***Mesosoma***. smooth and shiny. Pronotum wide and truncate anteriorly in dorsal view. Notauli deep, crenulate anteriorly. Middle lobe of mesoscutum anteriorly steep, nearly vertical. Mesoscutum longitudinally striate posteriorly. Scutellar sulcus strongly reniform with single strong midlongitudinal carina. Scutellum narrow. Epicnemial carina complete. Mesopleuron shiny; precoxal sulcus deep and strongly crenulate. Median area of metanotum with midlongitudinal carina and protruding posterodorsally into a point. Propodeum and metapleuron foveate rugose, with weak but distinct angulate apophyses. ***Forewing***. Second submarginal cell approximately twice as long as wide. Second submarginal cell very long (ca. 3 × longer than wide). Vein m-cu nearly straight; vein m-cu interstitial with 2RS. Vein 1cu-a postfurcal. First subdiscal cell rectangular. Subbasal cell evenly setose. ***Hind wing***. Vein R short longitudinal. Vein RS straight. Veins M+Cu and 1-M approximately equal length. Vein RS strongly curved. Vein m-cu absent. Base of hind wing evenly setose. ***Legs***. smooth, with sparse long setae. Hind femur clavate. Apex of hind tibia with comb of specialized setae medially. Mid and hind tibial spurs strongly curved and glabrous. Claws simple without basal lobe. ***Metasoma***. All tergites foveolate-punctate. First metasomal tergite not strongly narrowing subbasally; dorsope deep; with dorsal carinae uniting to form a weak, thin longitudinal carina. Second metasomal tergite with thin mid-longitudinal carina arising from small medio-basal triangular area. Tergites 3–5 with sharp lateral crease. Hypopygium large, ventrally strongly convex. Ovipositor curved ventrally. **Male**: Unknown.

##### Biology.

Unknown.

##### Distribution.

Only known from type specimen of the type species from Ecuador.

##### Remarks.

Morphologically, the new genus displays a mixture of character states associated with different genus groups. The curved glabrous tibial spurs suggest an affinity with the *Colastomion*-*Cystomastax* group of genera; however, it lacks the curved forewing vein m-cu and the elongate and basally narrowed first metasomal tergite that is characteristic of other members of that group. Nevertheless, DNA data (barcode and 28S; Quicke et al. unpublished) place it deeply derived within the *Colastomion*-*Cystomastax* group, and therefore the stouter first tergite and straight vein m-cu are presumed to be character state reversals.

##### Etymology.

From Spanish *hermoso* meaning beautiful, and the suffix *mastax* from the name of the closely related genus *Cystomastax*. The genus is masculine.

#### Hermosomastax clavifemorus

Taxon classificationAnimaliaHymenopteraBraconidae

Quicke sp. nov.

http://zoobank.org/3870D98D-368D-4B4B-BD3B-3CF4129CEF4B

Figure 369

##### Diagnostics.

No BIN is assigned, presumably because the barcode is only 411 bp long. Consensus barcode. ATTTGATCAGGTATANTAGGTTTATCTATAAGTATTATTATTCGAATAGAACTTAGTAATCCTGGAGGTTTAATTAAAAATGATTATATTTATAATTCAATAGTTACAGCTCATGCTTTTGTAATGATTTTTTTTATAGTTATACCTATTATAATTGGTGGATTTGGTAATTGATTAATTCCTTTGATATTAGGGGCTCCTGATATAGCTTTTCCTCGTATAAATAATATAAGATTTTGATTGTTAATTCCATCTTTATCAATATTATTAATTAGTTCATTTACTAATATTGGGGTAGGTACTGGATGAACAATATATCCTCCTTTATCTTCTTTAATTGGTCATAGTGGTGTTTCTGTAGATTTAGCAATTTTTTCTTTACATTTAGCAGGAATTTCTTCTATTATGGGG. Unique within Rogadinae, this species possesses a strongly petiolate (clavate) hind femur. The patchily patterned wings are also diagnostic within the *Colastomion*-*Cystomastax* group.

##### Description.

Length of body 5.4 mm, of forewing 4.0 mm and of antenna 6.8 mm. First flagellomere 1.25 and 1.35 × longer than the 2^nd^ and third respectively, the latter 4 × longer than wide. Width of head: width of face: height of eye = 2.6: 1.0: 1.5. Inter-tentorial distance 3.5 × shortest distance between tentorial pit and eye. Shortest distance between posterior ocelli: transverse diameter of posterior ocellus: shortest distance between posterior ocellus and eye = 1.0: 1.7: 1.7. Occiput smooth and shiny. Length of head behind eye 0.35 × length of eye in dorsal view. Lengths of forewing veins r-rs: 3RSa: 3RSb = 1.0: 5.0: 6.5: Forewing vein 1CUb 7.5 × 1CUa. Hind wing vein 1-M 1.15 × M+CU. Hind coxa deeply punctured basodorsally. Lengths of fore femur (excluding trochantellus): tibia: tarsus = 1.0: 1.2: 1.25. Hind tibia 1.3 × hind femur (excluding trochantellus). First tergite 1.6 × longer than maximally wide. Second tergite as long as wide. Third tergite 1.3 × wider than long.

***Coloration*.** Body, scape and pedicel, and hind coxa largely orange brown becoming darker ventrally. Flagellomeres white each with narrow darker apex. Lower part of face, labial and maxillary palps, fore and mid legs white. Hind leg white except swollen part of femur which is dark brown. Forewing grey with clear subapical transverse band and darker apical margin.

##### Holotype ♀.

Ecuador, Sucumbios, Sacha Lodge, 0.8, -76.1, 270 meters, 24/vi/1994, coll. Peter Hibbs. Depository: CNC.

***Host data*.** None.

***Holotype voucher code*.**BOLD process Id: ASQSQ128-09.

##### Paratypes.

None

##### Etymology.

The species name refers to the club-shaped hind femur.

**Figure 369.**
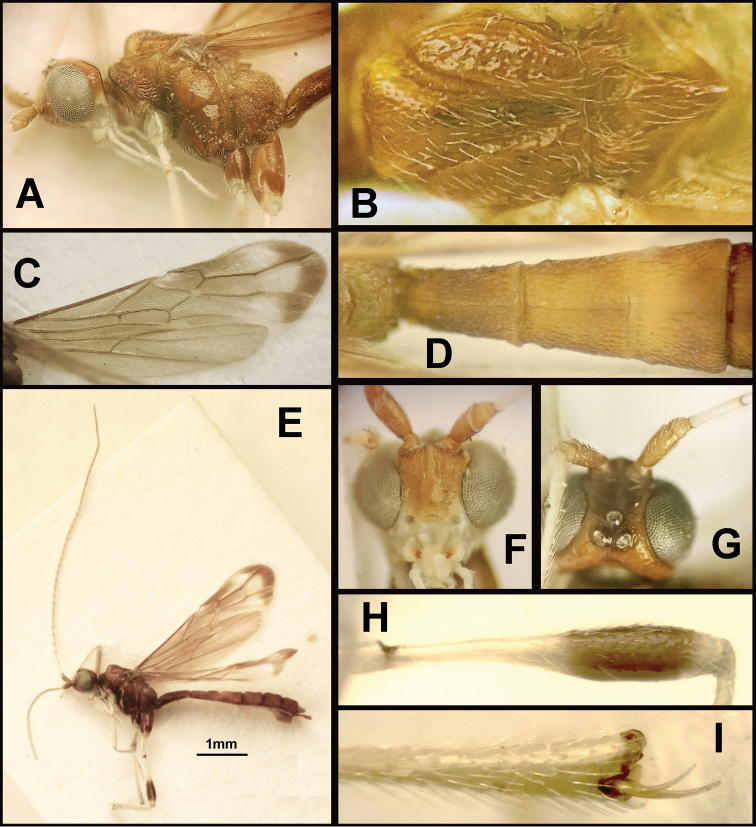
*Hermosomastaxclavifemorus*: **A** lateral head and mesosoma **B** dorsal thorax **C** wings **D** metasomal terga 1–3 **E** lateral habitus **F** anterior head **G** dorsal head **H** hind femur **I** apex of hind tibia.

### *Aleiodes* Wesmael, 1838

*Aleiodes* are found worldwide. There are records of approximately ten Costa Rican species scattered in publications. There is not a comprehensive generic treatment, and it is remotely possible that a few of the species below are junior synonyms of previously described species. When the holotypes are barcoded, this will become clear. Members are parasitoids of various macrolepidopterans, especially Noctuoidea, Geometroidea, and Bombycoidea (Shaw 1993).

#### Aleiodes adrianaradulovae

Taxon classificationAnimaliaHymenopteraBraconidae

Sharkey sp. nov.

http://zoobank.org/C960B605-DFC3-468A-8938-C9424FF46DBC

Figure 370

##### Diagnostics.

BOLD:AAG1413. Consensus barcode. AGTTTTATATTTTTTATTTGGAATATGATCAGGAATAGTAGGTTTATCAATAAGAATAATTATTCGATTAGAATTAAGAATTAGCGGAAGAATTTTGAAAAATGATCAAATTTATAATGGAATAGTTACTTTACATGCTTTTATTATAATTTTTTTTATAGTTATACCTATTATAATTGGTGGATTTGGTAATTGATTAATTCCTTTAATATTAAGAGTYCCTGATATAGCTTTCCCTCGAATAAATAATATAAGATTTTGATTATTAATTCCTTCTATTTTTTTTTTATTATTTAGAGGATTAATTAATTCAGGAGTAGGTACAGGATGAACTATATACCCTCCTTTATCTTCATTAATTGGTCATAGAGGGATATCTGTAGATATATCAATTTTTTCTTTACATTTAGCAGGTATATCTTCTATTATAGGTTCAGTTAATTTTATTACAACAATTTTTAATATTAATTTAAAAAGTATAAAAATAGATCAAATTTCATTATTTACTTGATCAGTAATAATTACTACAATTTTATTACTTTTATCATTACCTGTTTTAGCAGGAGCAATTACAATATTATTGACTGATCGAAATTTAAATTCAAGATTTTTTGATTTT---------------------------------------.

##### Holotype ♀.

Guanacaste, Pailas Dos, PL12-6, 10.7637, -85.333, 853 meters, 16/i/2014, forest, Malaise trap. Depository: CNC.

***Holotype voucher code*.**BIOUG28804-F01.

##### Paratypes.

Both Malaise-trapped. BIOUG28817-F07, BIOUG10014-B04. Depository: CNC.

##### Other material.

Specimens in the same BIN are from Belize (BMNHE897719), Chiapas Mexico (07TAPACH-01734), and Kentucky, USA (BCLDQ01649). The specimen from Chiapas has an image on BOLD that appears to be similar to the Costa Rican specimens and therefore it is likely the same species. The Belizean specimens do not have images but are presumably conspecific with the Costa Rican specimens. The Kentucky specimen does not have an image, and its identity is uncertain.

##### Etymology.

*Aleiodesadrianaradulovae* is named in honor of Adriana Radulov’s long-appreciated contributions to publicity for ACG, GDFCF, and now, BioAlfa.

**Figure 370.**
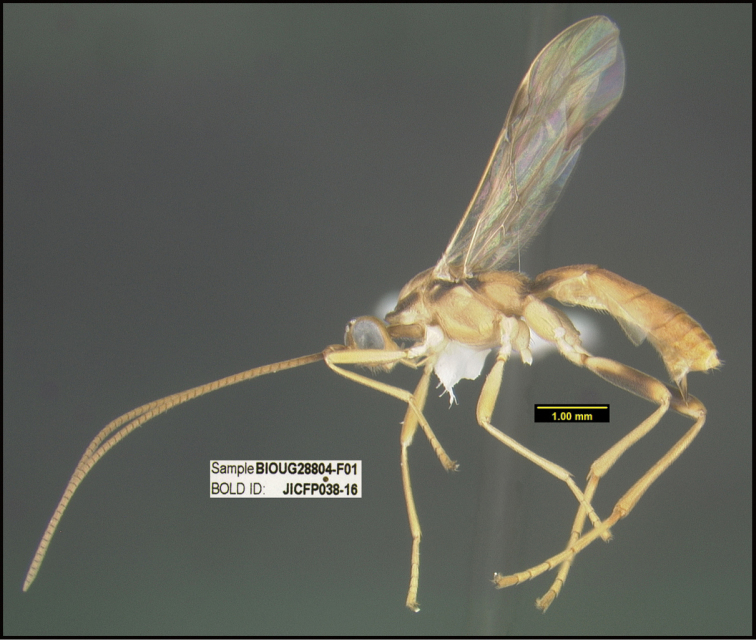
*Aleiodesadrianaradulovae*, holotype.

#### Aleiodes adrianforsythi

Taxon classificationAnimaliaHymenopteraBraconidae

Sharkey sp. nov.

http://zoobank.org/7A7EA260-2940-4536-8F8A-6BA13EBA3B0F

Figure 371

##### Diagnostics.

BOLD:ADJ1086. Consensus barcode. AATTTTATATTTTTTATTTGGTATATGAGCAGGAATAATTGGTATATCAATAAGATTAATTATTCGTCTAGAATTAAGAACGGGAGGAAGAATTTTAAAAAATGATCAAATCTACAATGGAATAGTAACATTACATGCTTTTATTATAATTTTTTTTATAGTTATACCAATTATAATTGGAGGGTTTGGTAATTGATTAATTCCTTTAATACTAGGAGCTCCTGATATAGCTTTCCCACGAATAAATAATATGAGATTTTGATTATTAATTCCTTCTCTAATACTTTTATTAACTAGAGGTATTATTAATACAGGTGTAGGAACAGGATGAACAATATATCCCCCCTTATCATCATTAATTGGTCATAATGGTATTTCAGTAGATATATCTATTTTTTCATTACATCTTGCTGGAACGTCTTCAATTATAGGAGCAATTAATTTTATTTCTACTATTTTTAATATAAATCTTTTAAGAATTAAGATAGACCAAATTATATTATTAATTTGATCTATCTTAATTACCACAATTTTATTATTATTATCTCTTCCAGTTCTTGCAGGAGCAATTACAATATTATTAACAGATCGAAATTTAAATACTAGATTTTTTGATTTTTCTGGAGGAGGGGACCCAATTTTATTCCAACATCTTTTT.

##### Holotype ♂.

Guanacaste, Sector Pitilla, Bullas, 10.98670, -85.38503, 440 meters, caterpillar collection date: 06/v/2017, wasp eclosion date: 21/v/2017. Depository: CNC.

***Host data*.***Eupithecia* Janzen774 (Geometridae) feeding on *Entadagigas* (Fabaceae)

***Host caterpillar and holotype wasp voucher codes*.** 17-SRNP-71403, DHJPAR0061499.

##### Paratypes.

None.

##### Etymology.

*Aleiodesadrianforsythi* is named in honor of Adrian Forsyth’s long-appreciated contributions to publicity for ACG, GDFCF, and now, BioAlfa.

**Figure 371.**
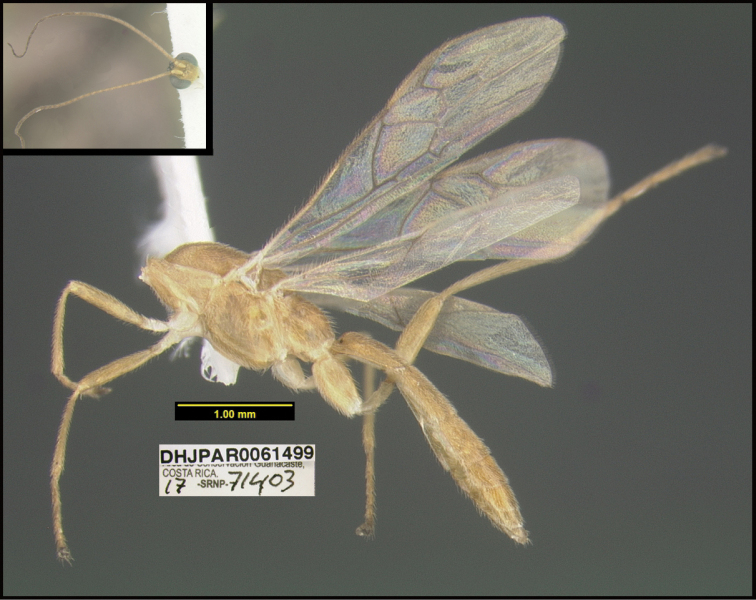
*Aleiodesadrianforsythi*, holotype.

#### Aleiodes agnespeelleae

Taxon classificationAnimaliaHymenopteraBraconidae

Sharkey sp. nov.

http://zoobank.org/6920F441-7E48-4526-87D8-AC6592C7FDBA

Figure 372

##### Diagnostics.

BOLD:AAH8705. Consensus barcode. GTTTTATATTTTATATTTGGGATATGGGCTGGTATAATTGGTTTATCGATAAGATTAATTATTCGATTGGAGTTAAGAGTTAGAGGAAGAATTTTAAAAAATGATCAAATTTATAATGGTATGGTTACATTACATGCTTTTGTAATAATTTTTTTTATAGTTATACCTATTATAATTGGGGGATTTGGAAATTGGTTGGTACCATTAATATTAAGGGCTCCTGATATAGCTTTCCCACGAATAAATAATATAAGATTTTGATTGTTAATTCCATCTTTTTTTTTATTATTAATTAGAGGTATTATTAATGCAGGAGTTGGAACTGGATGAACAATATACCCTCCCTTATCTTTATTAATTGGTCATAATGGAATTTCAGTTGATATATCTATTTTTTCTTTACATTTAGCTGGTGCTTCTTCAATTATAGGGTCAATTAATTTTATTACTACAATTTTTAATATAAAATTAAAAGAAATTAAGTTAGATCAAATTTCTTTATTAGTTTGATCTATTTTAATTACTACAATTTTATTATTACTTTCATTACCTGTTTTAGCAGGTGCTATTACTATATTATTAACT------------------------------------------------------------------------.

##### Holotype ♂.

Guanacaste, Pailas Dos, PL12-3, 10.7631, -85.3344, 820 meters, 02/i/2014, Malaise trap. Depository: CNC.

***Host data*.** None.

***Holotype voucher code*.**BIOUG29423-E01.

##### Paratypes.

None.

##### Other material.

Specimens from, Bolivia, Belize, and French Guiana are in the same BIN. There are no images of the specimens from Bolivia and Belize, though the specimens from Belize are likely conspecific with the Costa Rican specimens. The specimens from French Guiana are darker and may not be conspecific with the Costa Rican specimen.

##### Etymology.

*Aleiodesagnespeelleae* is named in honor of Agnes Peelle’s long-appreciated contributions to publicity for ACG, GDFCF, and now, BioAlfa.

**Figure 372.**
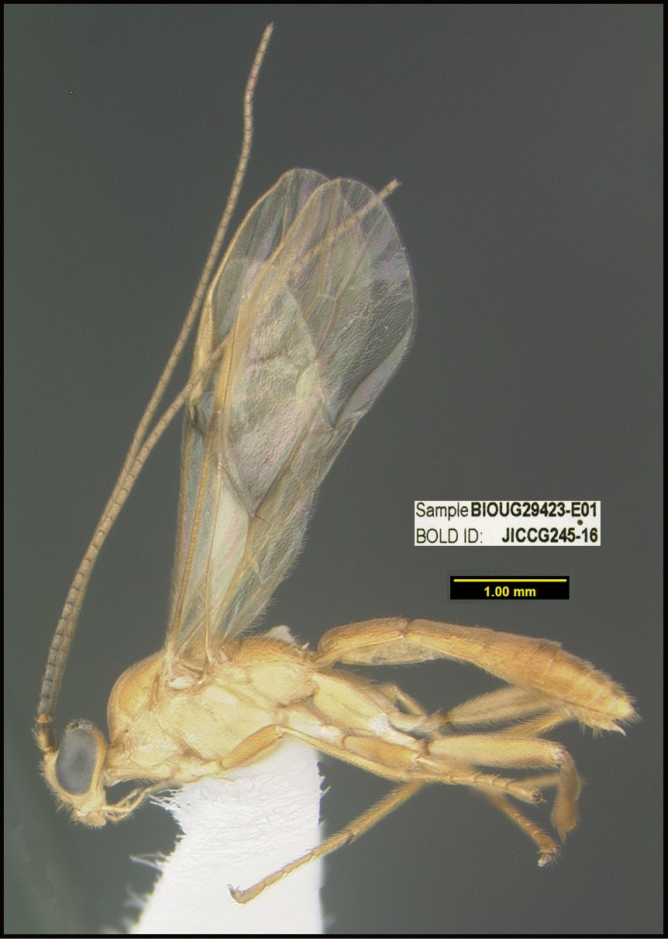
*Aleiodesagnespeelleae*, holotype.

#### Aleiodes alaneaglei

Taxon classificationAnimaliaHymenopteraBraconidae

Sharkey sp. nov.

http://zoobank.org/A1B5C4EE-4D1E-464E-A3B0-8D7AB9EB7DA1

Figure 373

##### Diagnostics.

BOLD:AAM5650. Consensus barcode. ATTCTTTACTTTTTATTTGGTATATGAGCAGGTATAATTGGTCTATCAATAAGATTAATTATTCGATTAGAATTAAGGACAAGAGGAAGAATCTTAAAAAATGATCAAATTTACAACGGAATAGTAACTTTACACGCTTTTATTATAATTTTTTTTATAGTTATACCAATTATAATTGGTGGATTTGGAAATTGACTCATTCCTTTAATATTAGGTGCTCCAGATATAGCTTTCCCACGAATAAATAATATAAGATTTTGATTATTAATTCCTTCTTTAATACTTTTATTAATTAGAGGAATAATTAATACAGGAGTAGGTACAGGATGAACAATATACCCTCCATTATCTTCATTAATTGGTCATAATGGATTTTCAGTAGATATATCTATTTTTTCTTTACATCTTGCTGGTGCTTCATCAATTATAGGAGCCATTAATTTTATTTCAACTATTTTTAATATAAATCTATTAAAAATTAAAATAGATCAAATTATATTATTAATTTGATCTATTTTAATTACTACAATTTTATTATTATTATCTTTACCAGTTCTTGCTGGTGCTATTACTATATTATTAACAGAT.

##### Holotype ♀.

Guanacaste, Sector Santa Rosa, Bosque San Emilio, 10.8438, -85.6138, 300 meters, dry forest, 17/xii/2012, forest, Malaise trap. Depository: CNC.

***Host data*.** None.

***Holotype voucher code*.**BIOUG17566-G02.

##### Paratypes.

None.

##### Other material.

Two specimens from Belize are in the same BIN (BMNHE897725, BMNHE897745) but were not examined.

##### Etymology.

*Aleiodesalaneaglei* is named in honor of Alan Eagle’s long-appreciated contributions to publicity for ACG, GDFCF, and now, BioAlfa.

**Figure 373.**
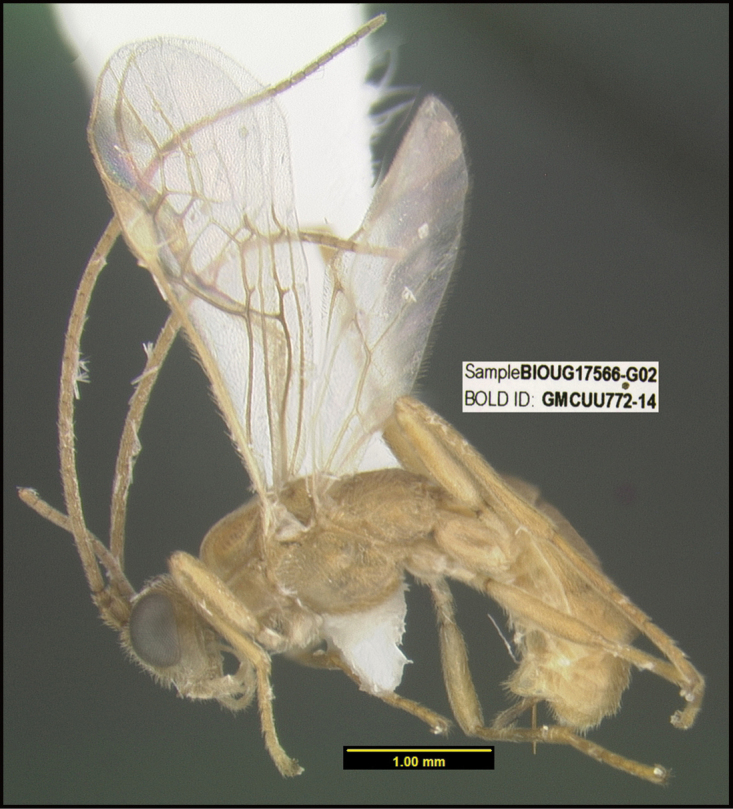
*Aleiodesalaneaglei*, holotype.

#### Aleiodes alanflemingi

Taxon classificationAnimaliaHymenopteraBraconidae

Sharkey sp. nov.

http://zoobank.org/7F786844-858D-42CE-AFF9-5ACE310FD925

Figure 374

##### Diagnostics.

BOLD:AAM5670. Consensus barcode. ATTCTATATTTTTTATTTGGGRTATGAGCAGGTATGATTGGTTTATCTATAAGGTTAATTATTCGTATAGAATTAAGAATAAGAGGGAGTATTTTAAAAAATGATCAGATTTATAATGGGATAGTGACATTACATGCTTTTATTATAATTTTTTTTATAGTTATACCTATTATAATTGGAGGGTTTGGTAATTGATTAATTCCTTTRATATTAGGAGCCCCTGATATGGCTTTYCCTCGAATAAATAATATAAGGTTTTGATYGTTGATCCCTTCATTATTTTTACTTTTAAATAGTGGAATTATTAATTTAGGAGTTGGTACAGGGTGAACTATATATCCTCCTTTATCTTCTTTAATTGGGCATAATGGTATATCTGTAGATATATCAATTTTTTCTTTACAYTTAGCTGGGGCTTCTTCAATTATAGGAGCTATTAATTTTATTTCTACAATTTTTAATATAAAAATAATAAAAATTAAAATAGATCAAATTAATTTATTTATTTGATCTRTTTTAATTACAGTAATTTTATTATTACTTTCATTGCCTGTTTTAGCGGGAGCTATTACTATATTATTAACTGATCGAAATTTAAATACAAGATTTTTTGATTTTTCTGGAGGAGGAGACCCTATTTTATTTCAACATCTT.

##### Holotype ♀.

Guanacaste, Sector San Cristobal, Estación San Gerardo, 10.8801, -85.389, 575 meters, rain forest/laguna, 12/v/2014, Malaise trap. Depository: CNC.

***Host data*.** None.

***Holotype voucher code*.**BIOUG27868-C03.

##### Paratypes.

None.

##### Other material.

One specimen from Belize (BMNHE897785) is in the same BIN but was not examined.

##### Etymology.

*Aleiodesalanflemingi* is named in honor of Alan Fleming’s long-appreciated contributions to publicity for ACG, GDFCF, and now, BioAlfa.

**Figure 374.**
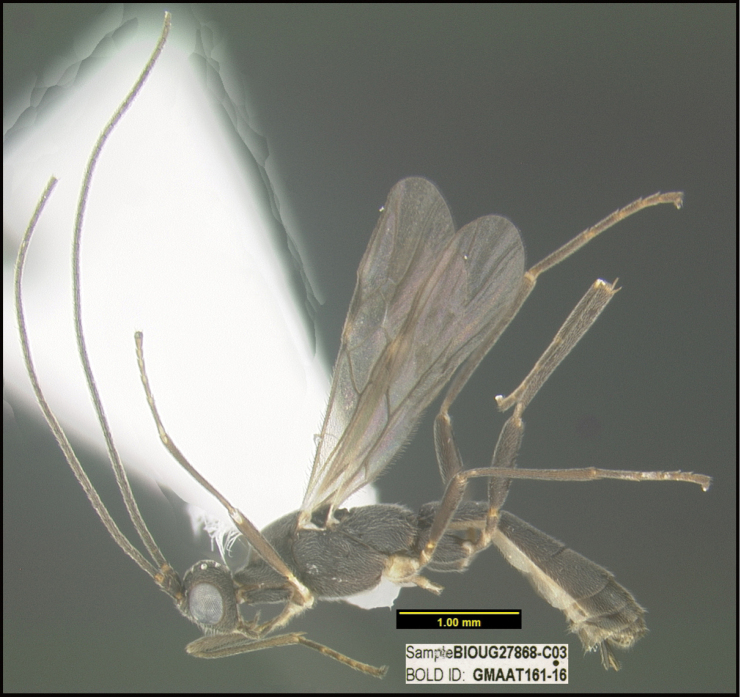
*Aleiodesalanflemingi*, holotype.

#### Aleiodes alanhalevii

Taxon classificationAnimaliaHymenopteraBraconidae

Sharkey sp. nov.

http://zoobank.org/C01B8513-6271-476F-9DA8-1BB4AFAAE676

Figure 375

##### Diagnostics.

BOLD:AAM5683. Consensus barcode. GTGTTATATTTTTTATTTGGTATATGAGCGGGTATRTTAGGTTTATCTATAAGTTTAATTATTCGATTGGAATTGAGAGTTGCTGGAAGATTGTTAAAGAATGATCAAATTTATAATGGTATAGTGACATTACATGCTTTTGTAATAATTTTTTTTATAGTTATACCTATTATGATTGGGGGGTTTGGTAATTGGTTAATTCCTTTGATATTAGGAGCTCCTGATATAGCTTTCCCTCGAATAAATAATATRAGATTTTGATTATTAATTCCTTCTTTTTTGTTATTGTTAATTAGAGGGGTAATTAAYTCAGGAGTAGGGACTGGTTGAACTATGTATCCTCCTCTTTCTTTATTAATTGGTCATAGTGGTTTTTCTGTGGATATATCTATTTTTTCATTACATTTAGCTGGGGCTTCTTCAATTATAGGATCTATTAATTTTATTTCAACTATTTTTAATATAAATTTATTWAAAATTAAATTAGATCARATTTCTTTATTTGTTTGATCAGTATTAATTACTACTGTATTGTTATTATTATCTTTACCTGTATTGGCAGGGGCTATTACTATATTGTTGACTGATCGTAATTTAAATACTAGATTTTTTGATTTTTCTGGGGGGGGTGATCCAATTTTATTTCAGCACTTA.

##### Holotype ♀.

Guanacaste, Pailas Dos, PL12-7, 10.7612, -85.3353, 791 meters, 09/i/2014, Malaise trap. Depository: CNC.

***Host data*.** None.

***Holotype voucher code*.**BIOUG28817-F08.

##### Paratype.

BIOUG29646-B02.

##### Other material.

One specimen from Belize (BMNHE897841) is in the same BIN but was not examined.

##### Etymology.

*Aleiodesalanhalevii* is named in honor of Alan Halevi’s long-appreciated contributions to publicity for ACG, GDFCF, and now, BioAlfa.

**Figure 375.**
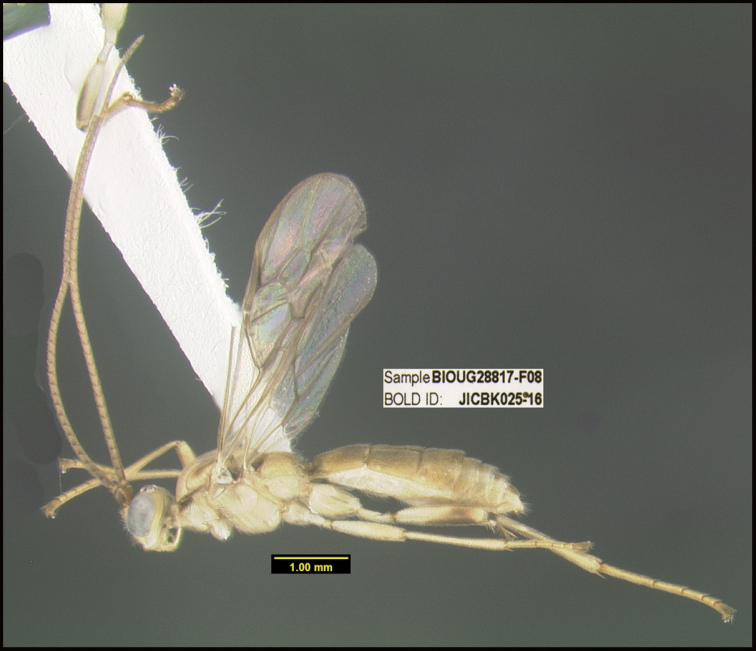
*Aleiodesalanhalevii*, holotype.

#### Aleiodes alejandromasisi

Taxon classificationAnimaliaHymenopteraBraconidae

Sharkey sp. nov.

http://zoobank.org/C10218BE-D767-4E83-9B37-F44F2F0C1A15

Figure 376

##### Diagnostics.

BOLD:ACG6844. Consensus barcode. AATTTTATATTTTTTATTTGGATTATGATCAGGAATAATTGGTATATCAATAAGCTTAATTATTCGATTAGAACTAAGGACAGGCGGAAGAATTTTAAAAAATGATCAAATTTATAATGGAATAGTAACTTTACATGCATTTATTATAATTTTTTTTATAGTTATACCTATTATAATTGGAGGTTTTGGAAATTGATTAATTCCTATCATACTAGGAGCTCCTGATATAGCTTTCCCTCGAATAAATAACATAAGATTTTGATTATTAATCCCATCTTTAATATTTTTATTAGTAAGAGGAATTATTAATATAGGGGTAGGGACAGGGTGAACTATATATCCCCCATTATCCTCATTAATTGGTCATAATAGAATATCAGTTGATATATCAATTTTCTCTTTACATATAGCAGGAGCATCTTCAATTATAGGATCAATTAACTTCATTTCAACAATCTTCAATATAAATTTAATAAAAATTAAACTAGACCAAATTATATTATTAGTTTGATCAGTATTAATTACAACTATTTTATTATTATTATCATTACCTGTATTAGCAGGAGCTATTACAATATTATTAACTGATCGTAATTTAAATACAAGTTTTTTTGATTTTTCAGGAGGAGGGGATCCAATTTTATTCCAACATTTATTT.

##### Holotype ♀.

Guanacaste, Sector Santa Rosa, Bosque San Emilio, 10.8438, -85.6138, 300 meters, 30/vii/2012, Malaise trap. Depository: CNC.

***Host data*.** None.

***Holotype voucher code*.**BIOUG05466-G08.

##### Paratypes.

None.

##### Etymology.

*Aleiodesalejandromasisi* is named in honor of Alejandro Masis’ long-appreciated contributions to publicity for ACG, GDFCF, and now, BioAlfa.

**Figure 376.**
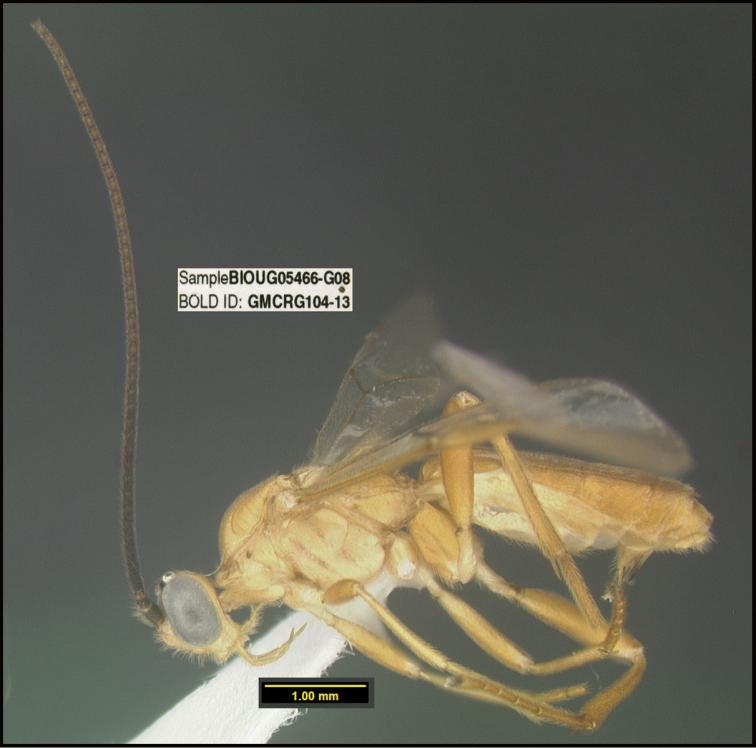
*Aleiodesalejandromasisi*, holotype.

#### Aleiodes alessandracallejae

Taxon classificationAnimaliaHymenopteraBraconidae

Sharkey sp. nov.

http://zoobank.org/861E0604-CA45-4883-8A66-137965A1957D

Figure 377

##### Diagnostics.

BOLD:ACJ2417. Consensus barcode. AGTTTTATATTTCCTTTTTGGCATATGAGCAGGAATAATTGGAATATCTATAAGCTTAATTATTCGATTAGAATTAAGAATGAGTGGAAGAATTTTAAAAAATGATCAAATTTATAACGGTATAGTAACTTTACATGCTTTCATTATAATTTTTTTTATAGTTATACCAATTATAATTGGAGGATTTGGAAATTGRTTAGTTCCTCTAATATTAGGAGCTCCTGATATAGCTTTCCCACGAATAAATAATATAAGATTTTGATTATTAATTCCTTCTTTAATACTTTTATTAATTAGTGGGGTAATTAATACAGGAGTAGGAACTGGTTGAACAATATACCCTCCATTATCATCATTAATTGGTCATAATGGAATTTCTGTAGATATATCTATTTTTTCATTACATCTAGCTGGAGCTTCATCAATTATAGGAGCCATTAACTTTATCTCAACTATTTTTAATATAAATTTAATAACAATTAAAATAGACCAAATCATACTATTAATTTGATCTATTCTAATTACCACAATTTTATTACTCTTATCTTTACCTGTTCTAGCTGGAGCTATTACTATATTATTAACAGATCGAAATTTAAACACAAGATTTTTTGACTTCACAGGAGGAGGGGATCCAATTTTATTCCAACATCTTTTT.

##### Holotype ♀.

Guanacaste, Pailas Dos, PL12-3, 10.7631, -85.3344, 820 meters, 29/v/2014, Malaise trap. Depository: CNC.

***Host data*.** None.

***Holotype voucher code*.**BIOUG29863-G04.

##### Paratypes.

BIOUG07616-F05, BIOUG09740-D04, BIOUG18397-D04, BIOUG29728-B07, BIOUG29725-E09, BIOUG29836-H03, BIOUG29862-A04, BIOUG29883-H11, BIOUG29879-F03, BIOUG30963-C06. Depository: CNC.

##### Etymology.

*Aleiodesalessandracallejae* is named in honor of Alessandra Calleja’s long-appreciated contributions to publicity for ACG, GDFCF, and now, BioAlfa.

**Figure 377.**
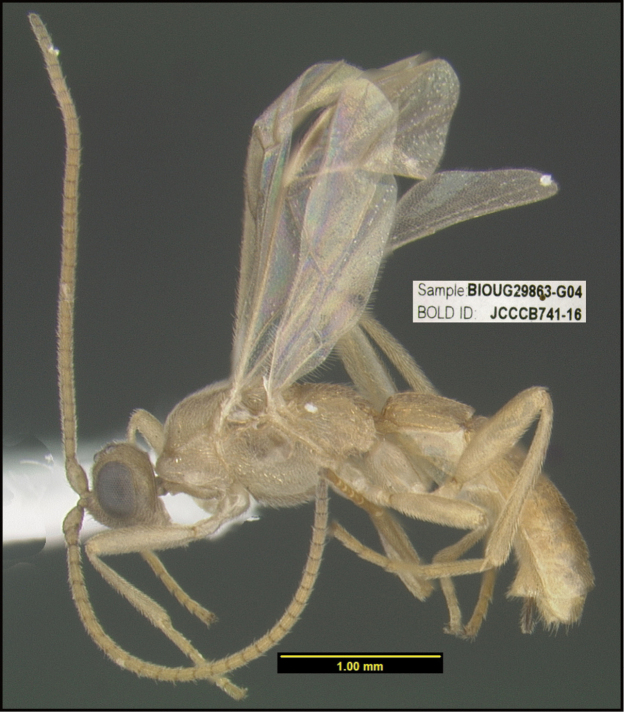
*Aleiodesalessandracallejae*, holotype.

#### Aleiodes alexsmithi

Taxon classificationAnimaliaHymenopteraBraconidae

Sharkey sp. nov.

http://zoobank.org/E7D6C858-54F0-4797-BBCB-939F707C3184

Figure 378

##### Diagnostics.

BOLD:AAV7512. Consensus barcode. AATTTTATACTTTTTATTTGGYATTTGAGCTGGAATAATTGGAATATCTATAAGTCTAATTATTCGAATAGAATTAAGAACAAATAAAAGAATTCTAAATAATGAACAAATCTATAATGGAATAGTAACTTTACATGCTTTTATTATAATTTTTTTTATAGTTATACCCATTATAATTGGAGGATTTGGTAATTGATTAATTCCTTTAATGCTAGGAGCCCCTGATATAGCTTTCCCACGAATAAATAAYATAAGATTTTGRTTATTAATTCCATCTTTATTATTTTTATTAACAAGAGGAATTATTAATTCAGGGGTAGGAACAGGATGAACAATATAYCCACCTTTATCATCATTAATTGGTCATAATGGAATATCAGTAGATATATCAATTTTTTCTTTACATTTAGCAGGAGCTTCTTCAATTATAGGAGCTATTAATTTCATCTCCACTATTTTCAATATAAATTTAAAAATAATTAAAATAGATCAATTACCTCTTCTTATTTGATCTATTTTAATTACAACAATTTTATTACTTTTATCTTTACCAGTYTTAGCAGGAGCTATTACCATATTATTAACAGATCGAAATCTAAATACTAGATTTTTTGATTTTTCAGGAGGAGGAGACCCCATTCTTTTCCAACATCTTTTT.

##### Holotype ♀.

Guanacaste, Sector Santa Rosa, Cuesta Canyon Tigre, 10.81703, -85.64366, 270 meters, caterpillar collection date: 20/v/2006, wasp eclosion date: 21/v/2007. Depository: CNC.

***Host data*.***Holochroaochra* (Geometridae) feeding on *Combretumfarinosum* (Combretaceae).

***Host caterpillar and holotype wasp voucher codes*.** 06-SRNP-14583, DHJPAR0062173.

##### Paratypes.

None.

##### Other material.

A specimen (BCLDQ01401) collected in Chamela, Jalisco, Mexico is in the same BIN. It was not examined.

##### Etymology.

*Aleiodesalexsmithi* is named in honor of Alex Smith’s long-appreciated contributions to publicity for ACG, GDFCF, and now, BioAlfa.

**Figure 378.**
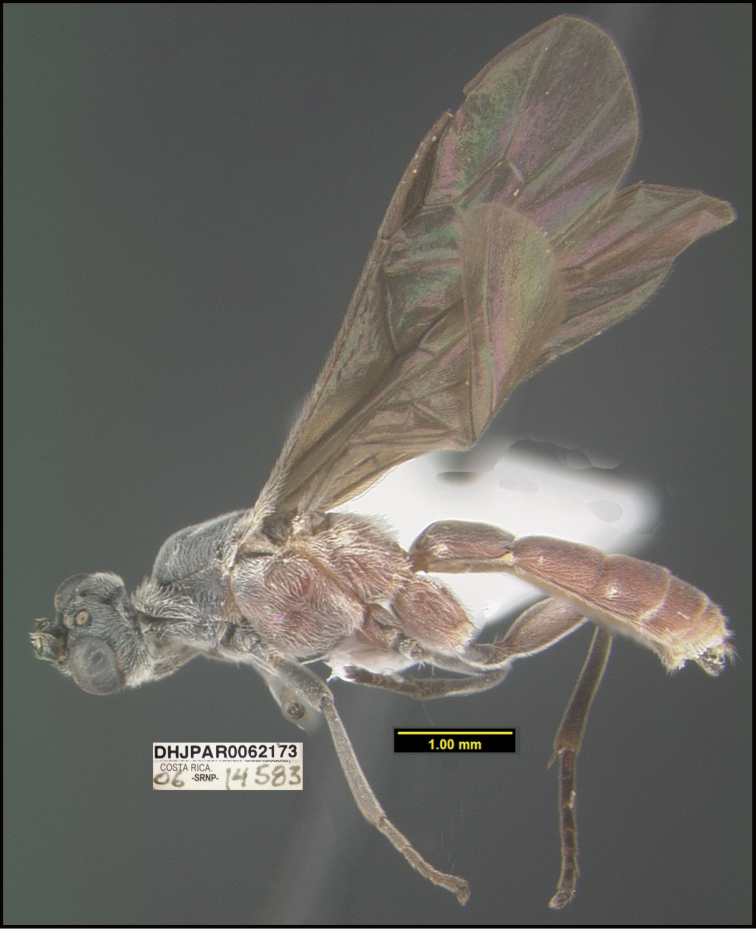
*Aleiodesalexsmithi*, holotype.

#### Aleiodes alfonsopescadori

Taxon classificationAnimaliaHymenopteraBraconidae

Sharkey sp. nov.

http://zoobank.org/6E696156-DD13-4D5B-8522-4EAB36827CE5

Figure 379

##### Diagnostics.

BOLD:ACR4858. Consensus barcode. AATTTTATATTTTTTATTTGGCATATGAGCAGGAATGGTTGGAGTATCAATAAGATTAATTATTCGTATAGAATTAAGAATAAGAGGAAGTATTTTAAAAAATGATCAAATTTATAATGGYATAGTTACATTACATGCTTTTATTATAATTTTTTTTATAGTTATRCCTATTATAATTGGGGGATTTGGRAATTGATTAGTTCCTTTAATATTGGGGGCTCCTGATATAGCTTTCCCYCGAATAAATAATATAAGATTTTGATTGTTAATYCCTTCATTATTTTTACTTTTAAATAGTGGAATTATTAATTTAGGTGTTGGYACAGGATGAACTATATAYCCTCCTTTATCTTCTTTAATTGGRCATAATGGTATATCTGTAGATATATCAATTTTYTCTTTACATTTAGCCGGAGCTTCTTCAATTATAGGAGCTGTTAATTTTATTTCTACAATTTTTAATATAAAAATAATAAAAATTAAAATAGATCAGATTAATTTATTTATTTGATCTATTTTAATTACAGTAATTTTATTATTACTTTCATTACCTGTTTTAGCGGGGGCTATTACTATATTATTAACTGATCGAAATTTAAATACAAGATTTTTTGATTTTTCTGGAGGGGGGG.

##### Holotype ♀.

Guanacaste, Sector Santa Rosa, Bosque San Emilio, 10.8438, -85.6138, 300 meters, 11/ii/2013, Malaise trap. Depository: CNC.

***Host data*.** None.

***Holotype voucher code*.**BIOUG18398-F08.

##### Paratypes.

BIOUG17569-F04, BIOUG18290-A11, BIOUG18499-F03. Depository: CNC.

##### Etymology.

*Aleiodesalfonsopescadori* is named in honor of Alfonso Pescador’s long-appreciated contributions to publicity for ACG, GDFCF, and now, BioAlfa.

**Figure 379.**
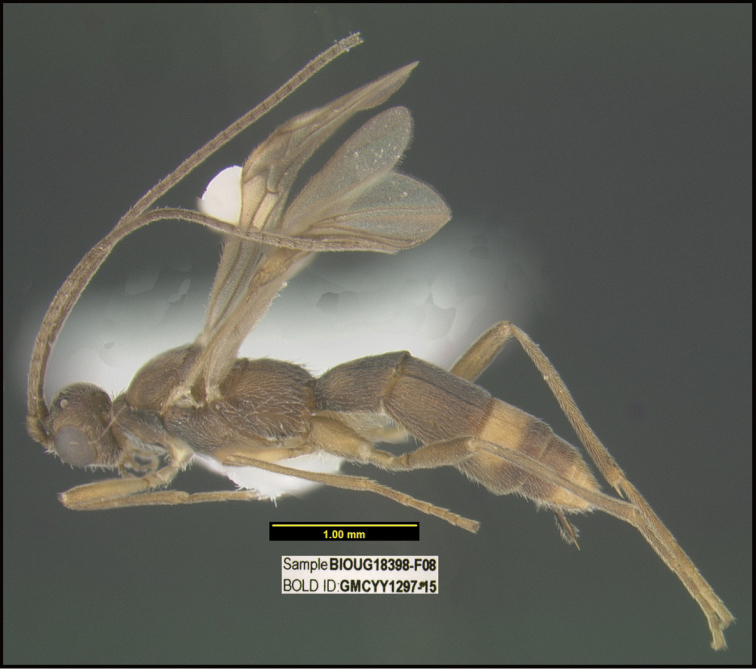
*Aleiodesalfonsopescadori*, holotype.

#### Aleiodes alisundermieri

Taxon classificationAnimaliaHymenopteraBraconidae

Sharkey sp. nov.

http://zoobank.org/7FED80D6-E336-4DA1-A37D-CD78D70C5253

Figure 380

##### Diagnostics.

BOLD:ADB3278. Consensus barcode. ATTTTATATTTTTTATTTGGTATATGAGCAGGAATAATTGGRATATCTATAAGTCTAATTATTCGTATAGAATTAAGAATAAATGGTAGAATTTTAAAAAATGATCAAATTTATAATGGTATAGTAACTTTACATGCTTTTATTATAATTTTTTTTATAGTTATACCAATTATAGTAGGAGGGTTTGGAAATTGACTAATTCCTTTAATATTAGGRAGGCCTGAYATAGCTTTCCCACGAATAAATAATATAAGATTTTGATTATTAATTCCTTCTTTAATACTTTTATTAATTAGAGGAATAATCAATACAGGAGTAGGRACRGGTTGAACAATATACCCCCCATTATCATCATTAATTGGTCACAATGGAATTTCAGTAGATATATCTATTTTCTCACTTCATTTAGCTGGAGCTTCATCAATYATAGGATCTATTAATTTTATTTCTACTATTTTTAATATAAATCTTATAGGAATTAAAATAGATCAAATTATACTATTTATTTGATCTATTTTAATTACTACAATTTTACTATTATTATCKTTACCAGTTCTTGCAGGAGCAATTACTATATTATTAACAGATCGAAATTTAAATACA.

##### Holotype ♀.

Guanacaste Pailas Dos PL12-3, 10.7631, -85.3344, 820 meters, 23/i/2014, Malaise trap. Depository: CNC.

***Host data*.** None.

***Holotype voucher code*.**BIOUG29481-G05.

##### Paratypes.

BIOUG29288-B08, BIOUG29288-A10.

##### Etymology.

*Aleiodesalisundermieri* is named in honor of Ali Sundermier’s long-appreciated contributions to publicity for ACG, GDFCF, and now, BioAlfa.

**Figure 380.**
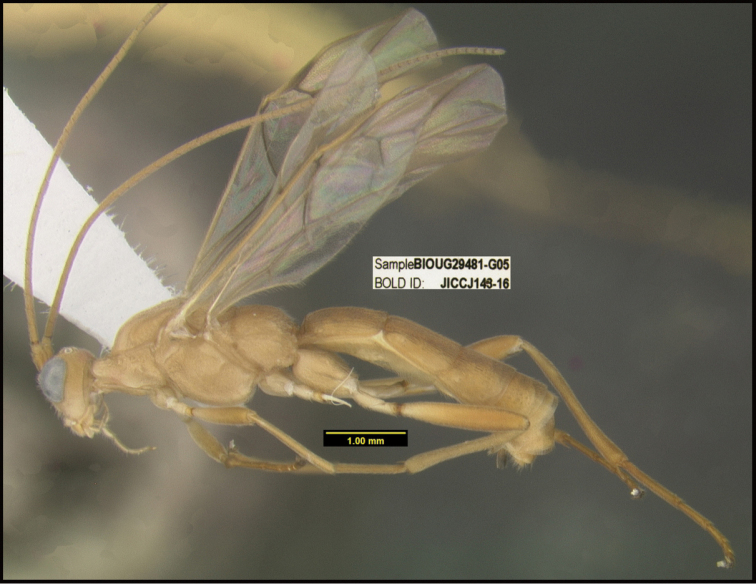
*Aleiodesalisundermieri*, holotype.

#### Aleiodes almasolisae

Taxon classificationAnimaliaHymenopteraBraconidae

Sharkey sp. nov.

http://zoobank.org/58247C42-6549-4361-8B13-8B5A3B8819D6

Figure 381

##### Diagnostics.

BOLD:ADF6889. Consensus barcode. ATCTTATATTTTTTATTTGGAATATGATCAGGTATAATCGGGTTAGCAATAAGGTTAATTATTCGAATAGAATTAGGAATTAGAGGGAGAGTTTTAAAAAATGATCAAATTTATAATGGTGTAGTAACATTGCATGCTTTTATTATAATTTTTTTTATGGTTATACCTATTATAATTGGAGGGTTTGGAAATTGATTAATTCCTTTAATATTAGGGGCCCCAGATATAGCATTCCCTCGTATAAATAATATAAGGTTTTGGTTATTAATTCCTTCATTATTTTTACTTTTAATAAGAGGAATTATTAACTTAGGAGTAGGAACGGGGTGAACAATATATCCTCCTTTATCTTCTTTAATTGGCCATAATGGGATATCTGTTGATATGTCTATTTTTTCTTTACATTTAGCAGGAATTTCTTCAATTATAGGAGCCATTAATTTTATTTCTACAATTTTTAATATAAATTTAATAAAAATTAAAATAGATCAAATTATATTATTAGTATGATCAATTTTAATTACAGTAATTTTATTATTACTTTCTTTGCCAGTTTTAGCGGGAGCAATTACTATACTATTAACTGACCGCAAT-------------------------.

##### Holotype ♀.

Guanacaste, Sector Cacao, Derrumbe, 10.9292, -85.4643, 1220 meters, 04/xii/2014, Malaise trap. Depository: CNC.

***Host data*.** None.

***Holotype voucher code*.**BIOUG31583-C08.

##### Paratypes.

None.

##### Etymology.

*Aleiodesalmasolisae* is named in honor of Alma Solis’ long-appreciated contributions to publicity for ACG, GDFCF, and now, BioAlfa.

**Figure 381.**
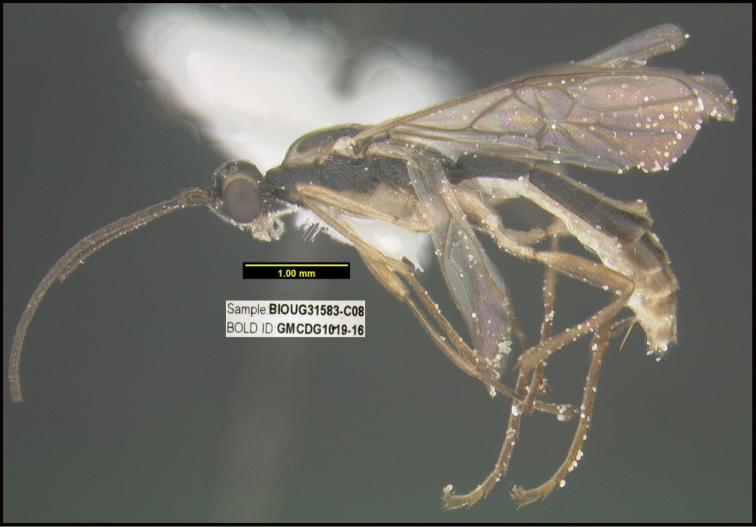
*Aleiodesalmasolisae*, holotype.

#### Aleiodes alvarougaldei

Taxon classificationAnimaliaHymenopteraBraconidae

Sharkey sp. nov.

http://zoobank.org/703815AF-2BBE-4BE9-AC05-61AB6E7470A9

Figure 382

##### Diagnostics.

BOLD:ABW3270. Consensus barcode. AGTTTTATATTTCCTTTTTGGTATATGAGCAGGAATAATTGGGATATCTATAAGTTTAATTATTCGGTTAGAATTAAGAATGAGTGGAAGAATTTTAAAAAATGATCAAATTTATAACGGTATAGTAACTTTACATGCTTTCATTATAATTTTTTTTATAGTTATACCAATTATAATTGGAGGCTTTGGRAATTGATTARTTCCTCTAATRTTAGGAGCTCCTGAYATAGCTTTCCCACGAATAAACAATATAAGATTTTGATTATTAATCCCTTCTTTAATACTTTTATTAATTAGYGGAATAATTAATACAGGTGTAGGAACTGGMTGAACAATATACCCYCCATTATCATCATTAATTGGTCATAATGGAATTTCTGTAGATATATCTATTTTTTCATTACAYCTAGCTGGAGCCTCATCAATTATAGGAGCCATTAATTTTATTTCAACTATTTTTAATATAAATTTAATAACAATTAAAATAGACCAAATCATATTATTAATTTGGKCTATTTTAATTACCACAATTTTATTACTCTTATCTTTACCTGTYCTAGCTGGAGCCATTACTATATTACTAACAGATCGAAATTTAAACACAAGATTTTTTGATTTCTCAGGAGGAGGTGAYCCAATTTTATTCCAACATCTTTTT.

##### Specimen ♀.

Guanacaste, Sector Pailas Dos, PL12-3, 10.7631, -85.3344, 820 meters, 05/ii/2015, Malaise trap. Depository: CNC.

***Host data*.** None.

***Specimen voucher code*.**BIOUG44435-B10.

##### Paratypes.

None.

##### Etymology.

*Aleiodesalvarougaldei* is named in honor of Alvaro Ugalde’s (RIP) long-appreciated contributions to publicity for ACG and GDFCF.

##### Note.

A second specimen in the same BIN on BOLD (BIOUG01036-F12) was collected in Ontario, Canada and identified as *A.wyomingensis* Shaw & Marsh, 2006, however the holotype of *A.wyomingensis* has a bicolored stigma and differs morphologically in other ways. Although placed in the same BIN the two specimens are not likely to be conspecific. The Canadian specimen has a similar color but is far more robust in the body and appendages than *A.alvarougaldei*.

**Figure 382.**
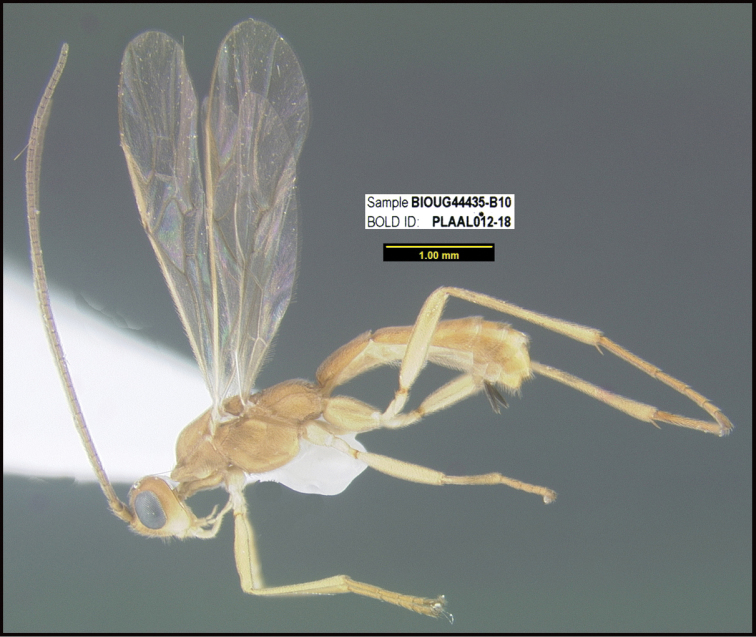
*Aleiodesalvarougaldei*, holotype.

#### Aleiodes alvaroumanai

Taxon classificationAnimaliaHymenopteraBraconidae

Sharkey sp. nov.

http://zoobank.org/440B0CA6-F6F0-4DE0-ABF0-A2E47B7C9D97

Figure 383

##### Diagnostics.

BOLD:AAM1443. Consensus barcode. AATTTTATATTTTTTATTTGGAATATGAGCTGGTATAATTGGGATATCAATAAGACTTATTATTCGATTAGAATTAAGAACTAACGGAAGAATTTTAAAAAATGATCAAATTTATAATAGAATAGTAACTTTACACGCTTTTATTATAATTTTTTTTATAGTTATACCAATTATAATTGGAGGATTTGGAAACTGATTAATTCCTTTAATATTAGGAGCCCCTGATATAGCTTTCCCACGTATAAATAATATAAGATTTTGATTATTAGTCCCTTCTTTAATACTATTATTAATTAGAGGTATTATCAACACAGGGGTAGGTACTGGATGAACAATATATCCACCATTATCATCCTTAATTGGCCACACCGGTATATCTGTAGATATATCTATTTTCTCACTACATTTAGCAGGAGCTTCATCAATTATAGGAGCAATTAATTTTATTTCCACTATCTTTAATATAAACTTAATAAAAATTAAAATAGATCAAATCATACTTTTAATTTGATCTATTTTAATTACTACTATTTTATTACTATTATCTTTACCTGTATTAGCAGGTGCAATTACTATATTATTAACTGATCGAAACTTAAATACAAGATTTTTTGATTTTTCAGGAGGAGGAGACCCAATTTTATTCCAGCATCTTTTT.

##### Holotype ♀.

Guanacaste, Pailas Dos, PL12-6, 10.7637, -85.333, 853 meters, 17/iv/2014, Malaise trap. Depository: CNC.

***Host data*.** None.

***Holotype voucher code*.**BIOUG29092-H10.

##### Paratypes.

Host = *Syngamiaflorella* (Crambidae): DHJPAR0038017, DHJPAR0038019.

##### Etymology.

*Aleiodesalvaroumanai* is named in honor of Alvaro Umaña’s long-appreciated contributions to publicity for ACG, GDFCF, and now, BioAlfa.

**Figure 383.**
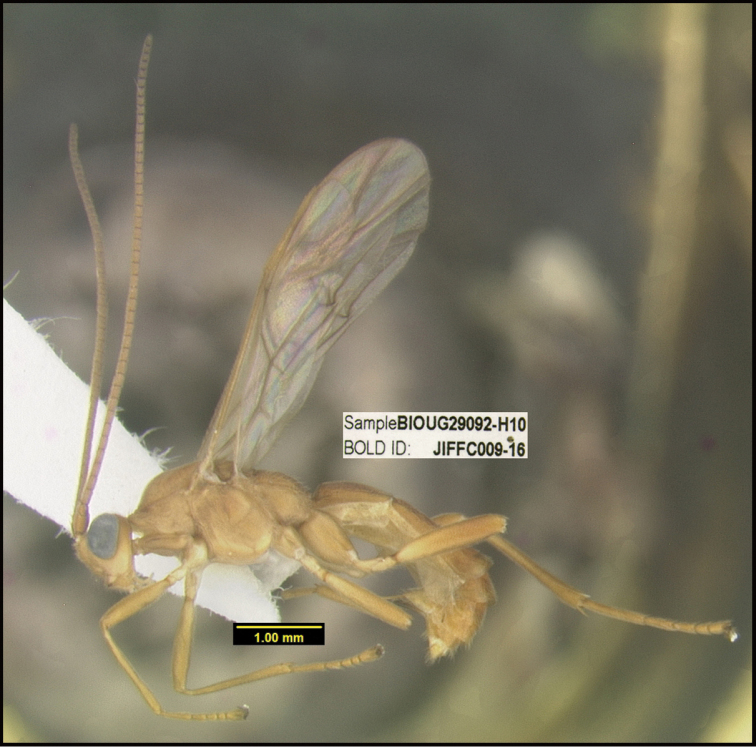
*Aleiodesalvaroumanai*, holotype.

#### Aleiodes angelsolisi

Taxon classificationAnimaliaHymenopteraBraconidae

Sharkey sp. nov.

http://zoobank.org/D101A7D2-F014-4FFE-AED9-F1C5A1BB529A

Figure 384

##### Diagnostics.

BOLD:AAM1722. Consensus barcode. GGTTTTATATTTTTTATTTGGAATTTGGGCAGGAATAGTAGGTTTATCAATAAGGTTAATTATTCGGTTAGAATTAAGGGTTTGTGGAAGAATTTTGAAAAATGATCAAATTTATAATGGTATAGTTACTTTACATGCTTTTGTGATAATTTTTTTTATAGTTATACCTATTATAATTGGTGGGTTTGGAAATTGGTTAATTCCTTTAATATTAGGGGCCCCAGATATAGCTTTCCCTCGTATAAATAATATAAGATTTTGATTATTGGTGCCATCTTTTTTTTTATTATTAATAAGAGGGTTAATTAATTCAGGAGTGGGGACGGGTTGAACCATATACCCCCCTTTATCTTTATTAATTGGTCATAGTGGTATTTCAGTTGATATATCTATTTTTTCTTTACATTTAGCGGGGGCTTCTTCAATTATAGGGGCTATTAATTTTATTTCAACTATTTTTAATATAAATTTAATAAAAATTAAATTAGATCAAATTTCTTTATTTATTTGGTCAGTTTTAATTACTACTATTTTATTACTTTTATCTTTACCGGTATTAGCGGGGGCTATTACTATGTTATTGACTGATCGTAATTTAAATACAAGATTTTTTGATTTTTCAGGAGGGGGGGATCCAATTTTGTTTCAGCATTTATTT.

##### Holotype ♀.

Guanacaste, Sector Pitilla, Charia, 10.99339, -85.40271, 530 meters, caterpillar collection date: 20/xi/2009, wasp eclosion date: 10/xii/2009. Depository: CNC.

***Host data*.***Nicetas* Poole11 (Erebidae) feeding on algae.

***Host caterpillar and holotype wasp voucher codes*.** 09-SRNP-73747, DHJPAR0038456.

##### Paratypes.

None.

##### Etymology.

*Aleiodesangelsolisi* is named in honor of Angel Solis’ long-appreciated contributions to publicity for ACG, GDFCF, and now, BioAlfa.

**Figure 384.**
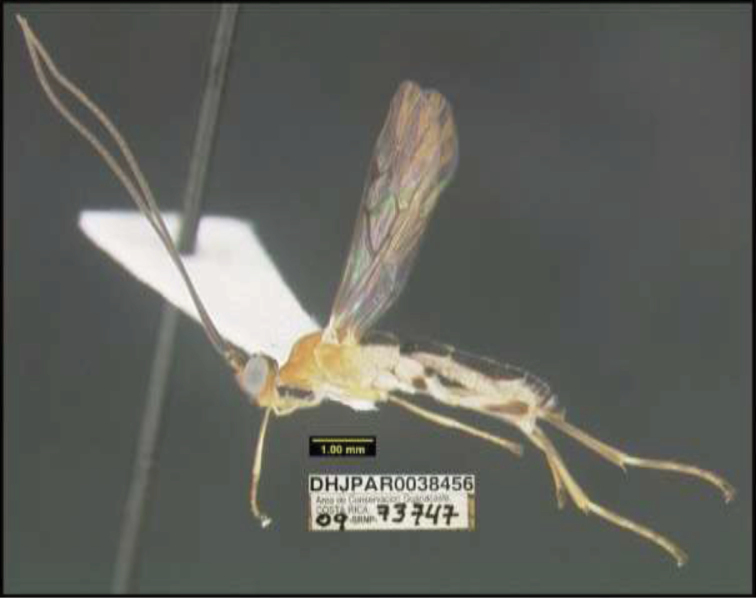
*Aleiodesangelsolisi*, holotype.

#### Aleiodes annhowdenae

Taxon classificationAnimaliaHymenopteraBraconidae

Sharkey sp. nov.

http://zoobank.org/23EB1816-4364-40A4-AB01-B7A5CA7FF989

Figure 385

##### Diagnostics.

BOLD:AAM5664. Consensus barcode. AATTTTATATTTTTTATTTGGAATATGAKCAGGRATAATCGGTATATCGATAAGATTAATTATTCGCATGGAATTAAGAACTAGAGGAAGAATTTTAAAAAATGATCAAATTTATAATAGAGTAGTAACTTTACATGCTTTTATTATAATTTTTTTTATAGTAATACCTATTATAATTGGAGGATTTGGTAATTGATTAATTCCACTAATATTAGGGGCTCCAGATATAGCTTTCCCRCGAATAAATAATATAAGATTTTGACTTTTAATCCCATCTTTATTATTACTTCTAAACAGGGGTATTATTAACTCAGGGGTGGGAACAGGGTGAACAATATACCCTCCCCTTTCTTCATTAATTGGCCATAATGGAATTTCAGTAGATATATCRATTTTTTCTCTTCATTTAGCGGGGGCTTCTTCAATTATAGGGGCTATTAATTTTATTTCTACTATTTTTAATATAAATTTATTAATAATTAAAATAAATCAATTRACTTTACTTACTTGATCAATTTTAATTACRACAATTTTACTACTTTTATCTTTACCAGTTTTAGCAGGGGCAATTACAATATTACTAACAGATCGAAATTTAAACACWRRWTTTTTTGATTTTTCCGGAGGGGGAGACCCAATTTTATTTCAACACTTA.

##### Holotype ♀.

Alajuela, Sector San Cristobal, Finca San Gabriel, 10.87766, -85.39343, 645 meters, caterpillar collection date: 23/ix/2013, wasp eclosion date: 12/x/2013. Depository: CNC.

***Host data*.***Eutomopeplaartena* (Geometridae) feeding on *Pleuranthodendronlindenii* (Salicaceae).

***Host caterpillar and holotype wasp voucher codes*.** 13-SRNP-4963, DHJPAR0053665.

##### Paratypes.

None.

##### Other material.

Four specimens in the same BIN (BMNHE897761, BMNHE897815, BMNHE897843, BMNHE897846) were collected in Belize. They were not examined.

##### Etymology.

*Aleiodesannhowdenae* is named in honor of Ann Howden’s long-appreciated contributions to publicity for ACG, GDFCF, and now, BioAlfa.

**Figure 385.**
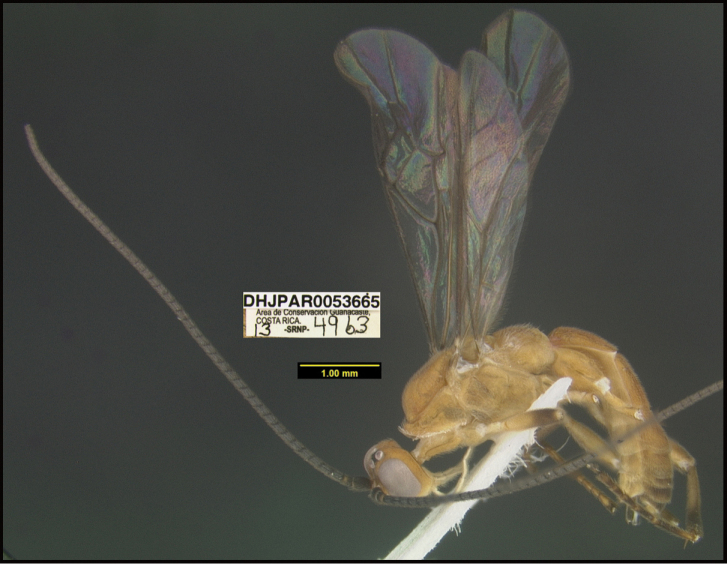
*Aleiodesannhowdenae*, holotype.

#### Aleiodes bobandersoni

Taxon classificationAnimaliaHymenopteraBraconidae

Sharkey sp. nov.

http://zoobank.org/00EF9FDA-6B61-40FE-A5D7-739887E1AD8E

Figure 386

##### Diagnostics.

BOLD:AAA5372. Consensus barcode. AGTTTTATATTTCTTATTTGGTATATGAGCAGGAATAATTGGTATATCAATAAGRTTAATTATTCGATTAGAATTAAGAACCAGAGGTAGTATCCTAAAAAATGATCAAATTTATAATGGAATAGTAACTTTACATGCTTTTATTATAATTTTTTTTATAGTTATACCAATTATAATTGGAGGATTTGGAAACTGAYTAATTCCATTAATATTAGGAGCCCCTGATATAGCTTTCCCACGAATAAATAATATAAGATTTTGATTATTAATTCCTTCCCTAATACTTTTRTTAATTAGAGGAATAATCAATACAGGAGTAGGGACAGGATGAACTATATACCCTCCCTTATCTTCATTAATTGGTCATAATGGAATTTCAGTAGATATATCAATTTTTTCATTACATTTAGCTGGAGCCTCATCAATTATAGGRGCAGTTAATTTTATTTCTACTATTTTTAATATAAATTTAATAATAATTAAAATAGACCAAATTACTTTACTAATTTGATCTATTTTAATTACTACAATCTTATTATTATTATCTTTACCAGTTTTAGCAGGAGCAATTACTATATTATTAACAGATCGAAATTTAAATACAAGATTTTTTGATTTTTCAGGWGGAGGAGATCCAATTTTATTTCAACATCTTTTT.

##### Holotype ♀.

Alajuela, Sector San Cristobal, Rio Blanco Abajo, 10.90037, -85.37254, 500 meters, caterpillar collection date: 14/vii/2000, wasp eclosion date: 28/vii/2000. Depository: CNC.

***Host data*.***Malocampapiratica* (Notodontidae) feeding on *Pouroumabicolor* (Urticaceae).

***Host caterpillar and holotype wasp voucher codes*.** 00-SRNP-11970, DHJPAR0062156.

##### Paratypes.

DHJPAR0051160, DHJPAR0029043, DHJPAR0052873, DHJPAR0062172. Depository: CNC.

##### Etymology.

*Aleiodesbobandersoni* is named in honor of Bob Anderson’s long-appreciated contributions to publicity for ACG, GDFCF, and now, BioAlfa.

**Figure 386.**
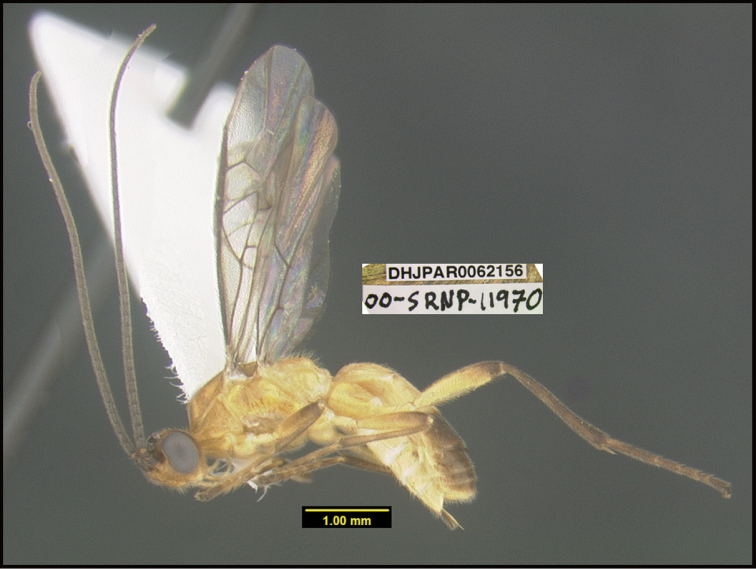
*Aleiodesbobandersoni*, holotype.

#### Aleiodes carolinagodoyae

Taxon classificationAnimaliaHymenopteraBraconidae

Sharkey sp. nov.

http://zoobank.org/3781C54F-CA30-4BF3-A20C-6962A8F91536

Figure 387

##### Diagnostics.

BOLD:AAW1567. Consensus barcode. ATTCTTTATTTTTTATTTGGAATATGAGCAGGAATAATTGGAATATCAATAAGTTTAATTATTCGATTAGAATTAAGATTAAG---AGGTAGAATGTTAAAAAATGATCAAATTTATAATGGTATAGTAACTTTACATGCTTTTATTATAATTTTTTTTATAGTTATACCAATTATAATTGGAGGATTCGGAAATTGATTAATTCCTTTAATATTAGGAGCTCCTGATATAGCTTTCCCACGAATAAATAATATAAGATTTTGAATTATTGTGCCTTCATTAATACTTTTATTAATAAGAGGAATTATTAATATAGGGGTAGGAACAGGATGAACTATATATCCTCCATTATCATCAACAATTGGACATAGGGGATTTTCTGTAGATATATCAATTTTTTCTTTACATTTAGCAGGAGCATCTTCAATTATAGGAGCTATCAATTTTATTTCAACTATTTTTAATATAAAAATTTTAAATATAAAAATAGATCAAATTATATTATTAATTTGATCTATTTTAATTACTACAATTTTATTATTATTATCATTACCAGTTCTTGCAGGAGCAATCACTATACTATTAACAGATCGAAATTTAAATACTAGATTCTTTGATTTTTCAGGAGGAGGAGACCCAA--------------------.

##### Holotype ♂.

Guanacaste, Sector Pitilla, Pasmompa, 11.01926, -85.40997, 440 meters, caterpillar collection date: 26/xii/2007, wasp eclosion date: 08/i/2008. Depository: CNC.

***Host data*.***Pleuroprucharudimentaria* (Geometridae) feeding on *Ingaoerstediana* (Fabaceae).

***Host caterpillar and holotype wasp voucher codes*.** 07-SRNP-34331, DHJPAR0023697.

##### Paratypes.

None.

##### Etymology.

*Aleiodescarolinagodoyae* is named in honor of Carolina Godoy’s long-appreciated contributions to publicity for ACG, GDFCF, and now, BioAlfa.

**Figure 387.**
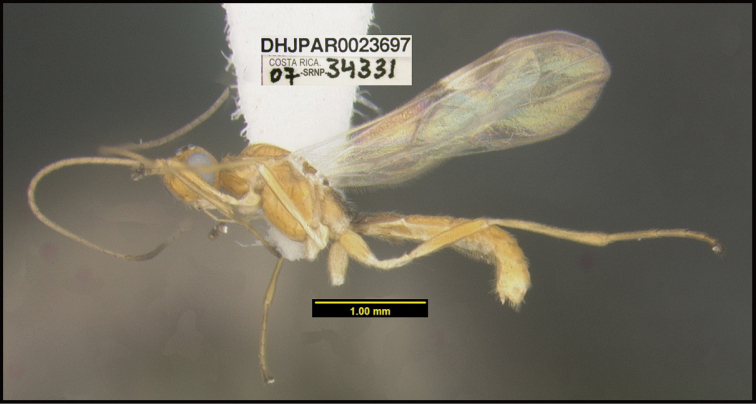
*Aleiodescarolinagodoyae*, holotype.

#### Aleiodes charlieobrieni

Taxon classificationAnimaliaHymenopteraBraconidae

Sharkey sp. nov.

http://zoobank.org/C874E238-E02B-4228-A8DB-1AEBFBEFCF0F

Figure 388

##### Diagnostics.

BOLD:ACJ4200. Consensus barcode. AATTTTATATTTTTTATTTGGTATATGATCAGGAATAATTGGATTAGGTATAAGATTAATTATTCGAATAGAATTAAGTGTTACAGGTAGAATTTTAAAAAATGATCAAATTTATAATGGTATGGTAACTATACATGCTTTTATTATAATTTTTTTTATGGTTATGCCTATTATAATAGGGGGATTTGGTAATTGATTAGTACCTTTAATATTAGGAGCTCCTGATATAGCATTTCCTCGAATAAATAATATAAGTTTTTGATTATTAATTCCATCTTTAATTTTATTATTAAATAGAAGATTAATTAATACAGGAATAGGGACAGGTTGAACAATATATCCTCCTTTATCTTCACTAATTGGACATATAGGTATATCAGTTGATATATCAATTTTTTCTTTACATTTAGCAGGAGCATCTTCAATTATAGGGGCAATTAATTTTATTTCTACAATTTTTAATATAAATTTAATAATAATTAAAATAGAACAATTAAGTTTATTAGTTTGATCTATTTTAATTACAGCAATTTTATTATTACTTTCTTTGCCTGTTTTAGCTGGGGCAATTACTATACTTTTAACTGATCGAAATTTAAATACAAGATTTTTTGATTTTTCTGGAGGAGGAGACCCAATTTTATTTCAGCATCTTTTT.

##### Holotype ♂.

Alajuela, Sector Rincon Rain Forest, Jacobo, 10.94076, -85.3177, 461 meters, caterpillar collection date: 08/i/2013, wasp eclosion date: 26/i/2013. Depository: CNC.

***Host data*.***Acrotomiamucia* (Geometridae) feeding on *Sabiceavillosa* (Rubiaceae)

***Host caterpillar and holotype wasp voucher codes*.** 13-SRNP-69051, DHJPAR0051316.

##### Paratypes.

None.

##### Etymology.

*Aleiodescharlieobrieni* is named in honor of Charlie O’Brien’s (RIP) long-appreciated contributions to publicity for ACG and GDFCF.

**Figure 388.**
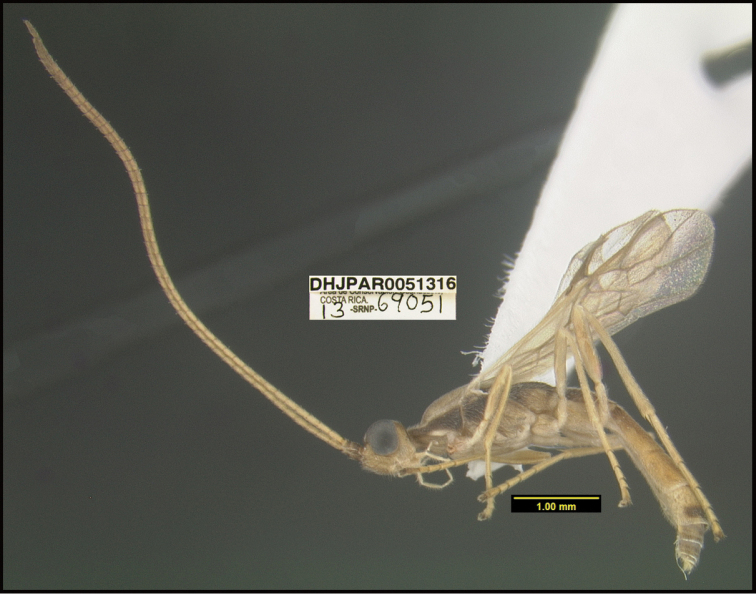
*Aleiodescharlieobrieni*, holotype.

#### Aleiodes davefurthi

Taxon classificationAnimaliaHymenopteraBraconidae

Sharkey sp. nov.

http://zoobank.org/7131CBBA-7BB7-40F0-920F-6B2EEA9A9EBE

Figure 389

##### Diagnostics.

BOLD:ACB2701. Consensus barcode. AATTTTATATTTTTTATTTGGAATATGAGCAGGAATAATTGGAATATCAATAAGACTAATTATTCGATTAGAATTAAGAATTAGAGGTAGAATTTTAATAAATGATCAAATTTATAATGGTATAGTAACTTTACATGCCTTTATTATAATTTTCTTTATAGTTATACCAATTATAATTGGAGGTTTTGGAAATTGATTAATTCCTTTAATACTAGGAGCCCCTGATATAGCTTTCCCTCGAATAAATAATATAAGATTTTGATTATTAATCCCTTCTTTATTATTCCTATTAATTAGAGGAATTATTAATACTGGAGTAGGAACAGGATGAACAATATATCCTCCTTTATCATCTTTAATTGGACATAATAGAATTTCAGTAGATATRTCAATTTTTGCTTTACATTTAGCAGGAGCTTCATCAATTATAGGAGCAATTAATTTTATTTCAACAATTTTTAATATAAATTTAATAAAAATTAAATTAGATCAAATTATATTATTAGTATGATCAGTATTAATTACTGCTATTCTATTATTACTTTCTTTACCTGTTTTAGCAGGTGCTATTACTATATTATTAACAGATCGTAATTTAAATACAAGATTTTTTGATTTCTCAGGAGGAGGAGACCCAATTTTATTTCAACATTTATTT.

##### Holotype ♂.

Guanacaste, Sector El Hacha, Estación Los Almendros, 11.03226, -85.52776, 290 meters, caterpillar collection date: 02/i/2012, wasp eclosion date: 16/i/2012. Depository: CNC.

***Host data*.***Herbita* medonaDHJ02 (Geometridae) feeding on *Hirtellaracemosa* (Chrysobalanaceae).

***Host caterpillar and holotype wasp voucher codes*.** 12-SRNP-20033, DHJPAR0049349.

##### Paratypes.

None.

##### Etymology.

*Aleiodesdavefurthi* is named in honor of Dave Furth’s long-appreciated contributions to publicity for ACG, GDFCF, and now, BioAlfa.

**Figure 389.**
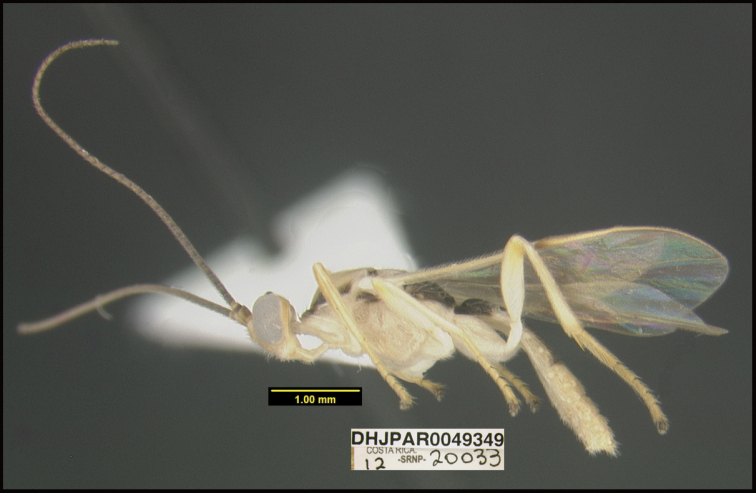
*Aleiodesdavefurthi*, holotype.

#### Aleiodes donwhiteheadi

Taxon classificationAnimaliaHymenopteraBraconidae

Sharkey sp. nov.

http://zoobank.org/C16B91F7-89CD-46C5-AF71-59EE8A632053

Figure 390

##### Diagnostics.

BOLD:AAA5378. Consensus barcode. TATTTTATATTTTTTATTTGGAATATGATCAGGGATAATTGGTTTATCAATAAGATTAATTATTCGTTTAGAATTAAGAACTTCTGGCAGAGTCTTAAAAAATGATCAAATTTATAATGGTATAGTTACATTACATGCATTTATTATAATTTTTTTTATAGTTATACCTGTAATATTGGGAGGATTTGGAAATTGGTTAGTTCCTTTAATATTAGGAGCTCCTGATATAGCTTTCCCACGAATAAATAATATAAGATTTTGGTTATTAATTCCTTCTTTAATTTTACTTTTAATAAGAGGAGTAATAAATTCAGGTGTAGGAACAGGTTGAACTATGTATCCACCTTTATCATCATTAATTGGTCATAGAGGATTTTCTGTTGATATATCTATTTTTTCTTTACATTTAGCTGGTGCATCTTCAATTATAGGATCAATTAATTTTATTTCAACAATTTTTAATATAAATTTAATAAAAATTAAGATAGATCAGATTTCATTATTTATTTGATCAATTTTAATTACAACAATTTTATTACTTCTTTCTTTACCAGTTTTAGCTGGAGCAATTACAATACTTTTAACAGATCGGAATTTAAATACAAGATTTTTTGATTTTTCAGGAGGAGGGGACCCAATTTTATTCCAGCATCTTTTT.

##### Holotype ♀.

Guanacaste, Sector Pitilla, Estación Quica, 10.99697, -85.39666, 470 meters, caterpillar collection date: 03/xi/2010, wasp eclosion date: 08/xi/2010. Depository: CNC.

***Host data*.***Calodesmamaculifrons* (Erebidae) feeding on unidentified plant.

***Host caterpillar and holotype wasp voucher codes*.** 10-SRNP-73227, DHJPAR0042076.

##### Paratype.

DHJPAR0029061. Depository: CNC.

##### Etymology.

*Aleiodesdonwhiteheadi* is named in honor of Don Whitehead’s (RIP) long-appreciated contributions to publicity for ACG and GDFCF.

**Figure 390.**
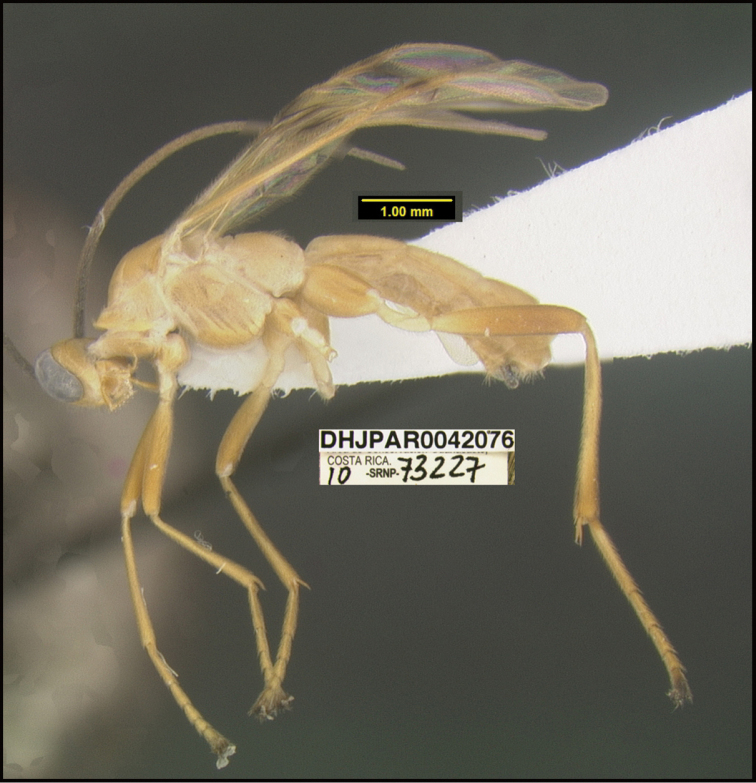
*Aleiodesdonwhiteheadi*, holotype.

#### Aleiodes doylemckeyi

Taxon classificationAnimaliaHymenopteraBraconidae

Sharkey sp. nov.

http://zoobank.org/2D8A3691-BA30-4A5D-9111-68F009EDFBC9

Figure 391

##### Diagnostics.

BOLD:AAH8920. Consensus barcode. ATTTTGTATTTTATTTTTGGGATGTGATCGGGGATAATTGGGATATCAATAAGGTTAATTATTCGATTAGAGTTAGGAGTTTGTGGGAGGGTTTTGGGGAATGATCAAATTTATAATGGGGTGGTAACTTTACATGCTTTTGTAATAATTTTTTTTATAGTTATACCTATTATAATTGGGGGGTTTGGAAATTGATTAATTCCTTTAATGTTAGGGTCCCCTGATATAGCTTTCCCTCGTATAAATAATATGAGGTTTTGGTTATTAGGACCTTCTTTATTATTTTTATTGATTAGGGGGTTAGTTAATAGAGGGGTAGGTACAGGGTGAACTATATATCCTCCCTTGTCTTTGGTATTAGGACATAGGGGGATATCTGTTGATATTTCTATTTTTTCATTACATTTAGCAGGGGTATCTTCTATTATGGGGGCTGTAAATTTTATTACTACTGTATTGAATATGAGTTTGTTTATAATTAAAATGGATCAAATTATATTATTTGTTTGGTCTGTAGTTATTACTGCTATATTATTATTGTTATCTTTACCAGTTTTAGCGGGGGCTATTACTATATTATTGACTGACCGA------------------------------.

##### Holotype ♀.

Guanacaste, Pailas Dos, PL12-9, 10.76, -85.3341, 809 meters, 16/i/2014, forest, Malaise trap. Depository: CNC.

***Host data*.** None.

***Holotype voucher code*.**BIOUG28676-D10.

##### Paratypes.

None.

##### Etymology.

*Aleiodesdoylemckeyi* is named in honor of Doyle McKey’s long-appreciated contributions to publicity for ACG, GDFCF, and now, BioAlfa.

**Figure 391.**
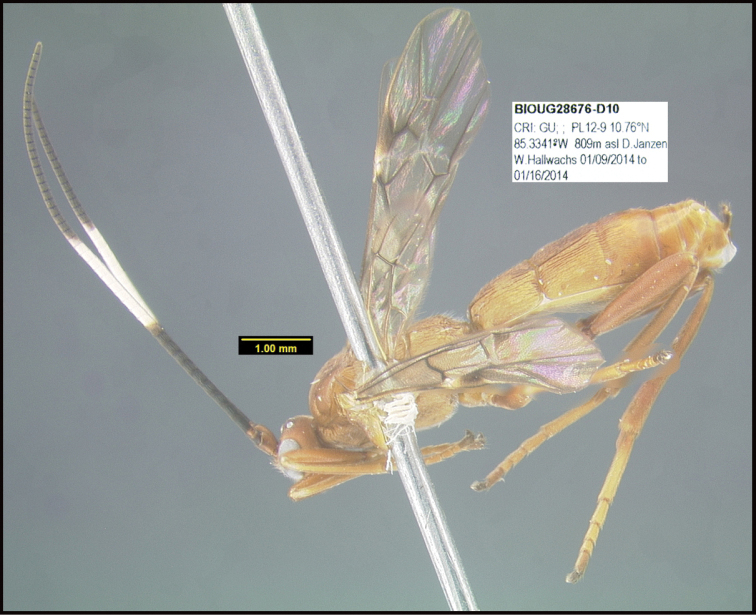
*Aleiodesdoylemckeyi*, holotype.

#### Aleiodes frankhovorei

Taxon classificationAnimaliaHymenopteraBraconidae

Sharkey sp. nov.

http://zoobank.org/66412467-FF24-434C-AA1C-314110977837

Figure 392

##### Diagnostics.

BOLD:AAM5640. Consensus barcode. AATTTTATATTTTTTATTTGGAATATGAGCAGGAATAATTGGTATATCAATAAGATTAATTATTCGCCTAGAATTAAGAACAGGAGGGAGAATTTTAAAAAATGATCAAATCTACAATGGAATAGTAACATTACATGCTTTTATTATAATTTTTTTTATAGTTATACCAATTATAATTGGAGGATTTGGTAATTGATTAATTCCTTTAATATTAGGAGCCCCTGATATAGCTTTCCCACGAATAAATAATATAAGATTTTGACTATTAATTCCTTCTCTAATACTTTTATTAATTAGAGGAATTATTAATACAGGGGTAGGTACAGGATGAACAATATATCCTCCTTTATCATCATTAATTGGTCATAATGGTATTTCAGTAGATATGTCTATTTTTTCGTTACATCTTGCTGGAGCCTCTTCAATTATAGGGGCAATTAATTTTATTTCCACTATTTTTAATATAAATCTTTTAAGAATTAAAATAGATCAAATTATATTATTAATTTGATCTATTTTAATTACCACAATTTTATTATTATTATCCCTTCCAGTTCTTGCAGGGGCAATTACAATATTATTAACAGATCGAAATTTAAATACTAGATTTTTTGAT------------------------------------------.

##### Holotype ♀.

Guanacaste, Sector Cacao, Estación Cacao, 10.92691, -85.46822, 1150 meters, caterpillar collection date: 13/vi/2009, wasp eclosion date: 26/vi/2009. Depository: CNC.

***Host data*.***Sabulodesarge* (Geometridae) feeding on *Nectandrasalicifolia* (flowers) (Lauraceae).

***Host caterpillar and holotype wasp voucher codes*.** 09-SRNP-36313, DHJPAR0040077.

##### Paratype.

DHJPAR0040076 Depository: CNC.

##### Etymology.

*Aleiodesfrankhovori* is named in honor of Frank Hovore’s (RIP) long-appreciated contributions to publicity for ACG and GDFCF.

**Figure 392.**
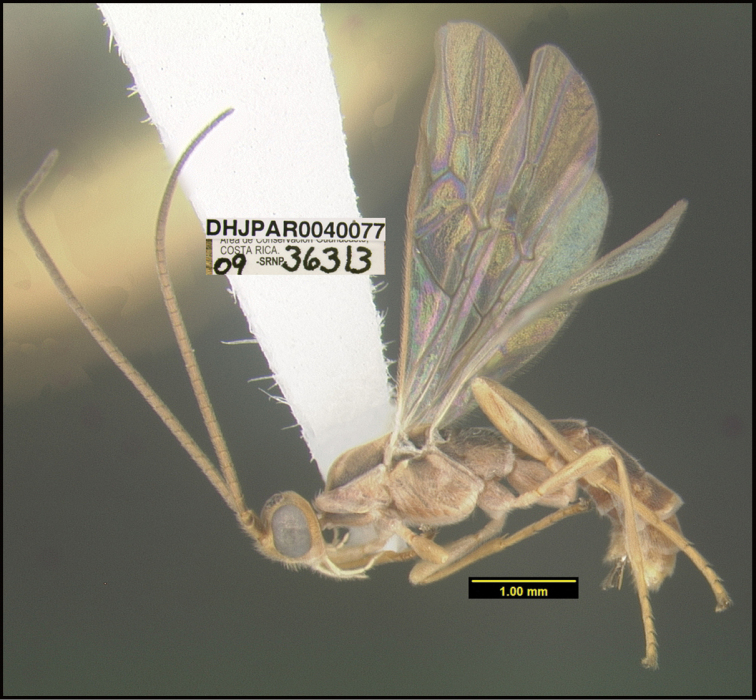
*Aleiodesfrankhovorei*, holotype.

#### Aleiodes henryhowdeni

Taxon classificationAnimaliaHymenopteraBraconidae

Sharkey sp. nov.

http://zoobank.org/0E27348B-52F6-40C6-8992-6447066B7B8E

Figure 393

##### Diagnostics.

BOLD:ABX5209. Consensus barcode. AATTTTATATTTTTTATTTGGAATATGAGCAGGAATAATTGGTATATCAATAAGATTAATTATTCGCCTAGAATTAAGAACAGGAGGGAGAATTTTAAAAAATGATCAAATCTACAATGGAATAGTAACATTACATGCTTTTATTATAATTTTTTTTATAGTTATACCAATTATAATTGGAGGATTTGGTAATTGATTAATTCCTTTAATATTAGGAGCCCCTGATATAGCTTTCCCACGAATAAATAATATAAGATTTTGACTATTAATTCCTTCTCTAATACTTTTATTAATTAGAGGAATTATTAATACAGGGGTAGGTACAGGATGAACAATATATCCTCCTTTATCATCATTAATTGGTCATAATGGTATTTCAGTAGATATGTCTATTTTTTCGTTACATCTTGCTGGAGCCTCTTCAATTATAGGGGCAATTAATTTTATTTCCACTATTTTTAATATAAATCTTTTAAGAATTAAAATAGATCAAATTATATTATTAATTTGATCTATTTTAATTACCACAATTTTATTATTATTATCCCTTCCAGTTCTTGCAGGGGCAATTACAATATTATTAACAGATCGAAATTTAAATACTAGATTTTTTGAT------------------------------------------.

##### Holotype ♀.

Guanacaste, Sector Mundo Nuevo, Estación La Perla, 10.76737, -85.43313, 325 meters, caterpillar collection date: 27/v/2011, wasp eclosion date: 09/vi/2011. Depository: CNC.

***Host data*.***Anticarsiagemmatalis* (Erebidae) feeding on *Centrosemasagittatum* (Fabaceae).

***Host caterpillar and holotype wasp voucher codes*.** 11-SRNP-55519, DHJPAR0045467.

##### Paratypes.

DHJPAR0052871, *Benisodes* Poole02. DHJPAR0063146, BIOUG30149-A08. Depository: CNC.

##### Other material.

A specimen from Belize (NHM749233) and another from Cuba (BCLDQ0693) are in the same BIN. They were not examined.

##### Etymology.

*Aleiodeshenryhowdeni* is named in honor of Henry Howden’s (RIP) long-appreciated contributions to publicity for ACG and GDFCF.

**Figure 393.**
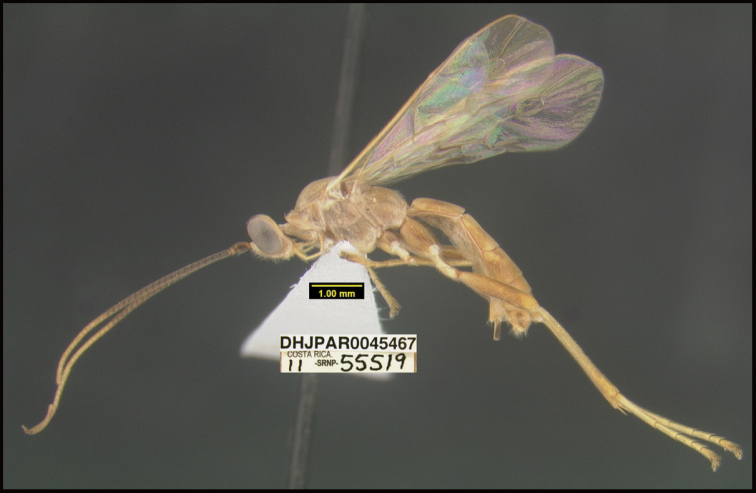
*Aleiodeshenryhowdeni*, holotype.

#### Aleiodes johnchemsaki

Taxon classificationAnimaliaHymenopteraBraconidae

Sharkey sp. nov.

http://zoobank.org/BF91388F-370D-4C89-B9B4-D4E37F4B333A

Figure 394

##### Diagnostics.

BOLD:ABX5740. Consensus barcode. AGTACTTTACTTTATATTTGGAATATGATCAGGAATAATTGGTTTATCAATAAGATTAATTATTCGATTAGAATTAAGATCAACATTAAGAATTTTAAAAAATGATCAAATTTATAATGGTATAGTAACTTTACATGCTTTTATTATAATTTTTTTTATAGTTATACCAATTATAATTGGAGGGTTTGGAAATTGATTAATTCCCCTAATATTAGGAGCACCTGATATAGCTTTCCCACGAATAAATAATATAAGATTTTGACTTCTAATTCCATCTTTAATACTTTTATTAACTAGAGGAATTATTAATACAGGAGTGGGGACAGGGTGAACAATATATCCTCCATTATCATCATTAATTGGTCATAATGGAATATCAGTAGATATATCTATTTTTTCATTACATTTAGCAGGGGCCTCATCAATCATAGGAGCTATTAATTTTATTTCCACTATCTTTAATATAAATTTATTAATAATTAAATTAGATCAAATTACATTATTAATTTGATCTATTTTAATTACTACAATTTTACTATTACTATCCCTACCAGTACTTGCAGGAGCTATTACTATATTATTAACAGACCGAAATTTAAATACTAGATTTTTTGATTTT---------------------------------------.

##### Holotype ♀.

Guanacaste, Sector Pitilla, Ingas, 11.00311, -85.42041, 580 meters, caterpillar collection date: 20/vi/2011, wasp eclosion date: 04/vii/2011. Depository: CNC.

***Host data*.***Nemoriaanae* (Geometridae) feeding on *Sabiceapanamensis* (Rubiaceae).

***Host caterpillar and holotype wasp voucher codes*.** 11-SRNP-31717, DHJPAR0045345.

##### Paratypes.

None.

##### Etymology.

*Aleiodesjohnchemsaki* is named in honor of John Chemsak’s (RIP) long-appreciated contributions to publicity for ACG and GDFCF.

**Figure 394.**
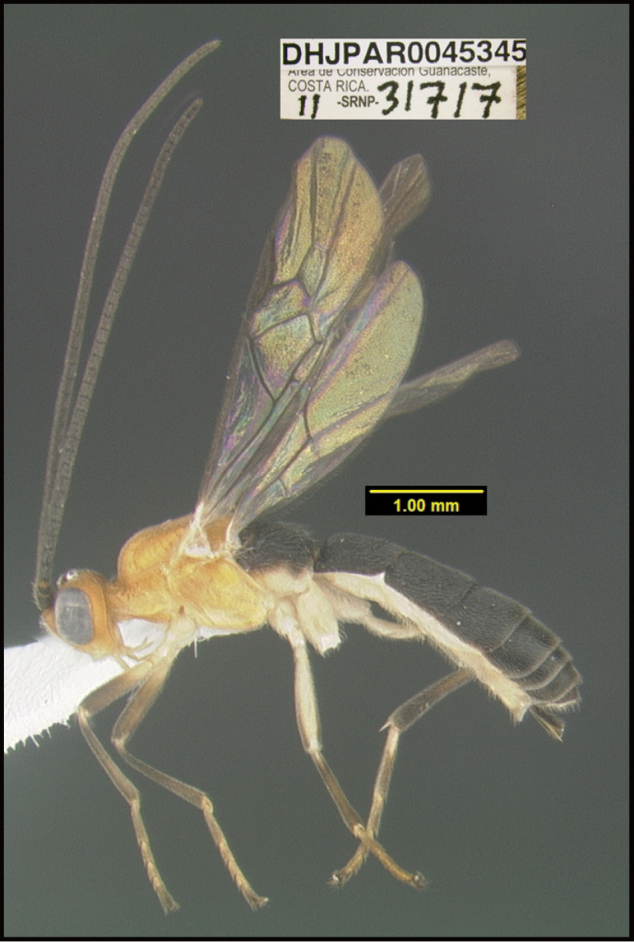
*Aleiodesjohnchemsaki*, holotype.

#### Aleiodes johnkingsolveri

Taxon classificationAnimaliaHymenopteraBraconidae

Sharkey sp. nov.

http://zoobank.org/0A6DE3A1-470F-4C54-B3C2-31A127959D4B

Figure 395

##### Diagnostics.

BOLD:AAT8850. Consensus barcode. TGTACTTTATTTCATATTTGGAATATGATCAGGAATAATTGGAATATCAATAAGTTTAATTATTCGATTAGAATTAAGATCAACTATAAGAATTTTAAAAAATGATCAAATTTATAATGGTATAGTAACAATACATGCTTTTATTATAATTTTTTTTATAGTAATACCAATTATAATTGGAGGATTTGGAAATTGATTAATTCCTTTAATGCTAGGAGCTCCTGATATAGCTTTCCCACGAATAAATAATATAAGATTTTGACTTCTAATCCCCTCTTTAATACTTTTATTAACTAGAGGTATTATTAATTCAGGAGTAGGAACAGGATGAACAATATACCCCCCATTATCCTCCTTAATTGGTCATAATGGTATATCAGTAGATATATCTATTTTTTCATTACATTTAGCAGGTGCTTCATCAATTATAGGGTCTATCAATTTTATTTCAACTATTTTTAATATAAATTTATTAATAATTAAAATAGATCAAATTACATTACTAATTTGATCTATTTTAATTACCACAATTTTATTATTACTATCTTTACCAGTTCTCGCAGGGGCCATTACTATATTATTAACAGATCGAAATTTAAATACTAGTTTCTTTGACTTTTCCGGAGGAGGAGACCCAATTTTATTTCAACATCTTTTT.

##### Holotype ♀.

Guanacaste, Sector Cacao, Sendero a Maritza, 1 km NW Estación Cacao, 10.92691, -85.46822, 1150 meters, caterpillar collection date: 17/v/2010, wasp eclosion date: 10/vi/2010. Depository: CNC.

***Host data*.***Chloropteryx* BioLep266 (Geometridae) feeding on *Gouanialupuloides* (Rhamnaceae).

***Host caterpillar and holotype wasp voucher codes*.** 10-SRNP-35211, DHJPAR0040450.

##### Paratypes.

None.

##### Etymology.

*Aleiodesjohnkingsolveri* is named in honor of John Kingsolver’s (RIP) long-appreciated contributions to publicity for ACG and GDFCF.

**Figure 395.**
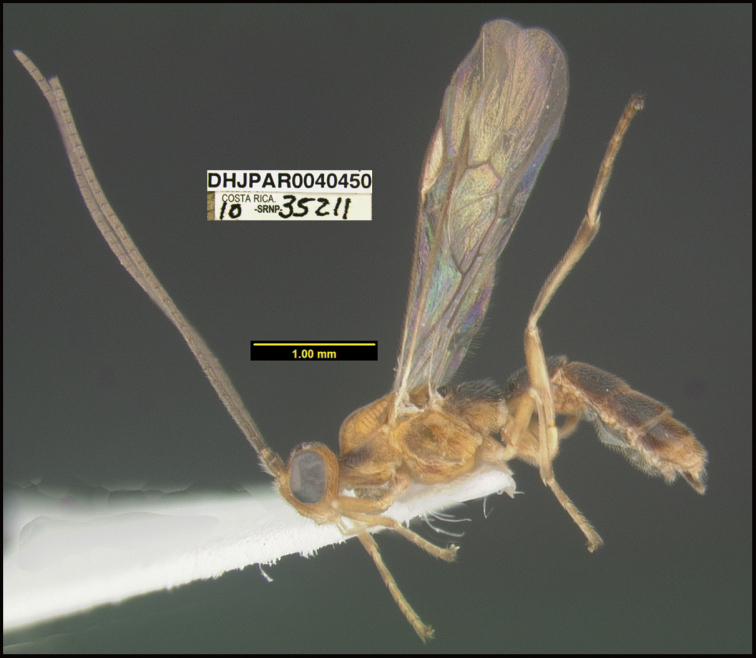
*Aleiodesjohnkingsolveri*, holotype.

#### Aleiodes gonodontovorus

Taxon classificationAnimaliaHymenopteraBraconidae

Shaw & Shimbori, 2020

Figure 396

##### Diagnostics.

BOLD:ACK7827. Consensus barcode. AATTTTATATTTTTTATTTGGTATATGATCTGGAATGRTTGGRTTATCTATAAGTTTAATTATTCGRTTAGAATTAAGAACTAYTGGAAGAATCTTAAAAAATGATCAAATTTATAATGGTATAGTTACTCTTCATGCTTTTATTATAATTTTTTTTATAGTTATACCTATTGTTTTAGGAGGATTYGGTAATTGATTAATTCCTTTAATATTAGGMGCCCCAGATATAGCTTTCCCTCGTATAAATAATATAAGGTTTTGRTTATTAATTCCATCATTATTATTCTTATTRATAAGAGGWRTTGTTAATACAGGYGTGGGRACAGGRTGRACAATTTAYCCVCCTTTRTCTTCATTAATTGGTCATAGAGGAATTTCTATAGATATATCAATTTTTTCTTTACATTTRGCAGGRGCTTCTTCAATTATAGGAGCTATTAATTTTATTTCAACAATTTTAAATATAAATTTAAATCAAATTAAATTRGATCAAATATCTTTATTTATTTGATCTGTATTAATTACAGCATTTTTATTACTTTTAGCTTTACCAGTTTTGGCAGGRGCTATTACYATACTTTTAACTGATCGAAATTTAAATACTAGATTTTTTGATTTTYYKGRRGGGGGGGATCCAATTTTATTYCAGCATCTTTTT.

##### Imaged specimen ♀.

Guanacaste, Sector Mundo Nuevo, Vado Miramonte, 10.77175, -85.43400, 305 meters, caterpillar collection date: 12/vii/2012, wasp eclosion date: 23/vii/2012. Depository: CNC.

***Host data*.***Gonodontaincurva* (Erebidae) feeding on *Piperumbellatum* (Piperaceae).

***Host caterpillar and imaged wasp voucher codes*.** 12-SRNP-55790, DHJPAR0049948.

##### Paratypes.

Hosts = *Gonodontaincurva*, *Gonodontafulvangula*, *Gonodontacorrecta*, *Gonodontaimmacula*, *Gonodontauxor*, *Gonodontabidens*, *Gonodontapyrgo*, *Gonodontanitidimacula*. DHJPAR0016434, DHJPAR0021153, DHJPAR0016919, DHJPAR0016925, DHJPAR0009351, DHJPAR0029041, DHJPAR0028026, DHJPAR0028027, DHJPAR0042799, DHJPAR0045387, DHJPAR0048706, DHJPAR0048707, DHJPAR0048708, DHJPAR0049663, DHJPAR0049664, DHJPAR0052874, DHJPAR0021154, DHJPAR0021156, DHJPAR0029068, DHJPAR0021131, DHJPAR0029065, DHJPAR0028025, DHJPAR0028034, DHJPAR0028035, DHJPAR0028029, DHJPAR0028028, DHJPAR0030593, DHJPAR0030599, DHJPAR0049662. DHJPAR0021155, DHJPAR0009352, DHJPAR0021181, DHJPAR0029042, DHJPAR0029063, DHJPAR0029062, DHJPAR0045056, DHJPAR0048702, DHJPAR0048709, DHJPAR0048710, DHJPAR0051310, DHJPAR0029049. Depository: CNC.

##### Note.

Two specimens (BCLDQ0956, BCLDQ0714) from French Guiana are in the same BIN but were not examined.

**Figure 396.**
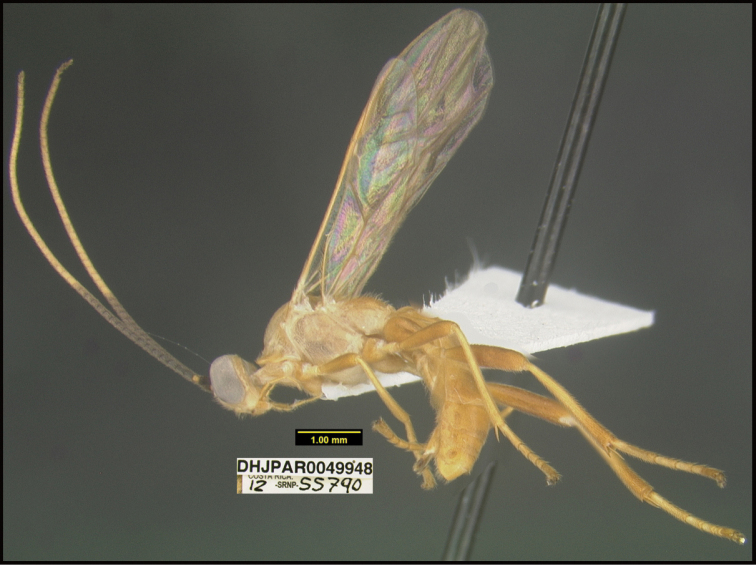
*Aleiodesgonodontovorus* Shaw & Shimbori.

#### Aleiodes manuelzumbadoi

Taxon classificationAnimaliaHymenopteraBraconidae

Sharkey sp. nov.

http://zoobank.org/B5C0E0F1-CE88-4198-966F-E5F48FEB5E14

Figure 397

##### Diagnostics.

BOLD:ACL0293. Consensus barcode. AGTTTTATATTTCCTATTTGGAATATGAGCAGGAATAATTGGGATATCTATAAGATTAATTATTCGATTAGAGTTAAGAGTAAGTGGAAGAATTTTAAAAAATGATCAAATTTATAATGGTATAGTAACTTTACATGCTTTCATTATAATTTTTTTCATAGTTATACCAATTATAATTGGGGGGTTTGGAAATTGATTAATTCCTTTAATATTAGGAGCCCCTGATATAGCTTTCCCCCGAATAAATAATATAAGATTCTGATTATTAATTCCCTCTTTAATACTTTTATTAATTAGAGGAGTAATTAATACAGGGGTAGGTACTGGCTGGACAATATACCCCCCATTATCTTCACTAATTGGTCATAATGGAATTTCTGTAGATATATCTATTTTTTCATTACATCTAGCTGGAGCCTCATCAATTATAGGAGCAATTAACTTTATTTCAACTATTTTTAATATAAACCTAATAACAATTAAAATAGACCAAATTATACTACTAATTTGGTCTATTTTAATTACCACAATTTTACTACTTTTATCCCTGCCCGTTCTAGCTGGAGCTATTACTATATTATTAACAGATCGAAATTTAAATACAAGATTTTTTGATTTTTCAGGAGGAGGAGACCCAATTTTATTCCAACATCTTTTT.

##### Holotype ♀.

Guanacaste, Sector Santa Rosa, Bosque San Emilio, 10.8438, -85.6138, 300 meters, 07/i/2013, Malaise trap. Depository: CNC.

***Host data*.** None.

***Holotype voucher code*.**BIOUG09827-B10.

##### Paratypes.

BIOUG09442-A12, BIOUG09739-H01, BIOUG17569-D12, BIOUG17575-B03, BIOUG17575-B06, BIOUG17616-A02, BIOUG28821-H03. Depository: CNC.

##### Etymology.

*Aleiodesmanuelzumbadoi* is named in honor of Manuel Zumbado’s long-appreciated contributions to publicity for ACG, GDFCF, and now, BioAlfa.

**Figure 397.**
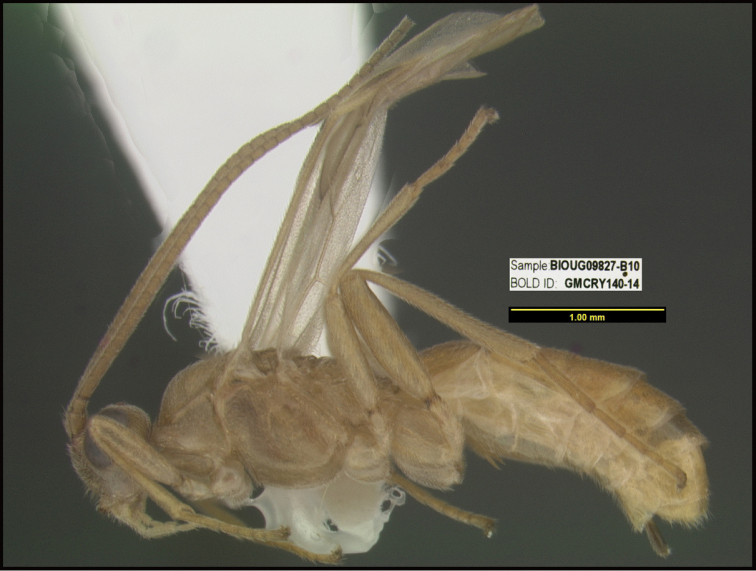
*Aleiodesmanuelzumbadoi*, holotype.

#### Aleiodes mayrabonillae

Taxon classificationAnimaliaHymenopteraBraconidae

Sharkey sp. nov.

http://zoobank.org/98EDD166-B03F-4A7F-AA9C-F1E56B472BA6

Figure 398

##### Diagnostics.

BOLD:AAD7873. Consensus barcode. TGTATTRTATTTTTTATTTGGTATATGAGCAGGTATAATTGGKTTATCAATAAGGTTAATTGTTCGGTTAGAGYTAGGTGTTTGTGGGAGAGTATTAGGCAATGATCAAATTTATAATGGTATAGTTACTTTGCATGCTTTTATTATAATTTTTTTTATGGTTATGCCTATTATAATTGGTGGGTTTGGGAATTGATTAATTCCTTTAATATTAGGGGCTCCTGATATAGCTTTCCCTCGAATAAATAATATAAGTTTTTGGCTATTATTACCTTCAATTTTATTACTATTATTAAGGGGTATTATTAATGTAGGGGTAGGTACTGGTTGAACAGTTTAYCCTCCTTTATCTTCATTAATTGGRCATAGTGGTATATCTGTTGATTTATCTATTTTTTCTTTACATTTAGCTGGTGTTTCTTCTATTATAGGGGCTATTAATTTTATTACAACTATTTTTAATATAAATTTATTTATAATTAAAATAGATCAAATTATATTATTTGTTTGATCTGTATTAATTACCGCTTTTTTACTTTTATTATCTTTACCTGTTTTAGCTGGTGCTATTACTATATTATTAACTGATCGTAATTTAAATACTACATTTTTTGATTTTTCGGGGGGGGGAGACCCTATTTTATTTCAACATTTATTT.

##### Holotype ♂.

Guanacaste, Sector Cacao, Sendero Circular, 10.92714, -85.46683, 1185 meters, caterpillar collection date: 12/ix/2012, wasp eclosion date: 18/x/2012. Depository: CNC.

***Host data*.***Acrotomiamucia* (Geometridae) feeding on *Coussareaaustin-smithii* (Rubiaceae).

***Host caterpillar and holotype wasp voucher codes*.** 12-SRNP-35331, DHJPAR0051376.

##### Paratypes.

Host: same caterpillar species as holotype and same host as holotype but on *Psychotriaelata* (Rubiaceae): DHJPAR0055814, DHJPAR0057810, DHJPAR0051676, DHJPAR0055814, DHJPAR0062993. Depository: CNC.

##### Etymology.

*Aleiodesmayrabonillae* is named in honor of Mayra Bonilla’s long-appreciated contributions to publicity for ACG, GDFCF, and now, BioAlfa.

**Figure 398.**
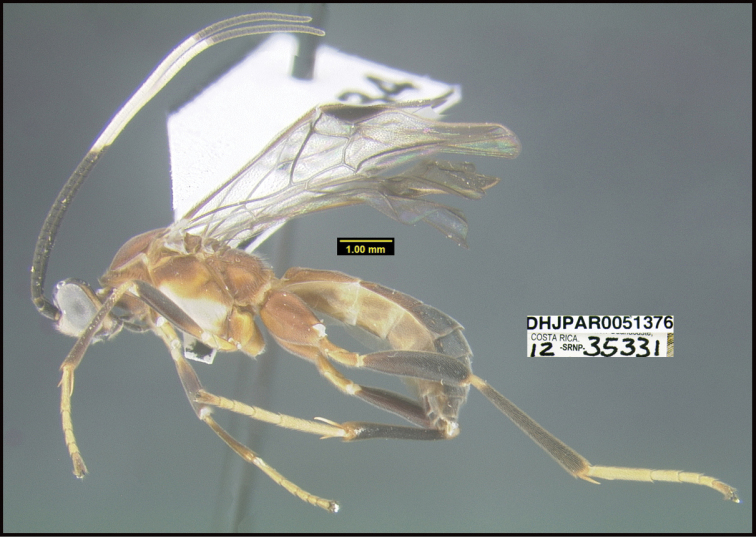
*Aleiodesmayrabonillae*, holotype.

#### Aleiodes michelledsouzae

Taxon classificationAnimaliaHymenopteraBraconidae

Sharkey sp. nov.

http://zoobank.org/902948EF-A59F-4159-9A86-A5DA58D53C1B

Figure 399

##### Diagnostics.

BOLD:ABA7287. Consensus barcode. GAGCAGGTATAATTGGGATATCAATAAGATTAATTATTCGAATAGAATTAAGGACAAGAGGGAGAATCTTAAAAAATGACCAAATTTATAATGGTATAGTAACTTTACATGCTTTCATTATAATTTTTTTTATAGTTATACCAATTATAATTGGAGGGTTCGGAAATTGATTAATTCCCTTAATATTAGGGGCTCCTGATATAGCATTCCCTCGAATAAATAATATAAGATTTTGATTATTAATTCCATCTTTATTATTTTTATTAATAAGAGGTGTTATTAATACAGGTGTTGGAACAGGATGAACCATATACCCCCCTTTATCATCCTTAATTGGGCATAATAGAATCTCAGTTGATATATCAATTTTTTCTTTACATTTAGCAGGAGCTTCTTCTATTATAGGGGCAATTAATTTTATTTCAACAATTTTCAATATAAATTTAATAAAAATTAAATTAGACCAAATTTCATTATTAGTCTGATCAATTTTAATTACTACTATTTTATTATTGTTATCTTTACCTGTACTAGCAGGGGCAATCACTATATTATTAACTGACCGTAACTTAAATACAAGATTTTTTGATTTTTCGGGGGGGGGGGACCCAATTTTA---------------.

##### Holotype ♀.

Guanacaste, Pitilla, Sendero Orosilito, 10.98332, -85.43623, 900 meters, caterpillar collection date: 25/v/2011, wasp eclosion date: 28/v/2011. Depository: CNC.

***Host data*.***Yidalpta* auragalisDHJ01 (Erebidae) feeding on *Securidacasylvestris* (Polygalaceae).

***Host caterpillar and holotype wasp voucher codes*.** 11-SRNP-31492, DHJPAR0042782.

##### Paratypes.

None.

##### Etymology.

*Aleiodesmichelledsouzae* is named in honor of Michelle D’souza’s long-appreciated contributions to publicity for ACG, GDFCF, and now, BioAlfa.

**Figure 399.**
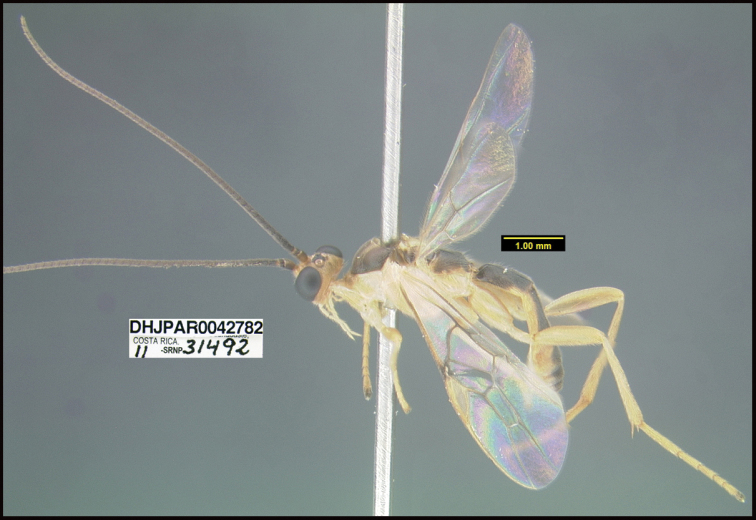
*Aleiodesmichelledsouzae*, holotype.

#### Aleiodes mikeiviei

Taxon classificationAnimaliaHymenopteraBraconidae

Sharkey sp. nov.

http://zoobank.org/8781E88B-CC9F-46AB-A12D-757917CF1418

Figure 400

##### Diagnostics.

BOLD:ACS9594. Consensus barcode. AGTTTTATATTTTATGTTTGGGATATGAGCTGGTATGATTGGGCTATCAATAAGATTAATTATTCGATTAGAATTAAGAATTAG---AGGGAGAATTTTAAAGAATGATCAAATTTATAATGGTATAGTTACATTACATGCTTTTGTAATAATTTTTTTTATAGTTATACCTATTATAATTGGAGGGTTTGGAAATTGATTGGTACCATTAATATTAGGGGCCCCTGATATAGCTTTTCCACGAATGAATAATATAAGGTTTTGATTATTAATTCCATCTTTTTTTTTATTATTAATTAGGGGGGTTATTAATGCAGGAGTTGGGACTGGATGGACAATGTATCCTCCTTTATCTTTATTAATTGGTCATAATGGTATTTCAGTTGATATATCTATTTTTTCTTTACATTTAGCTGGGGCTTCTTCAATTATAGGGGCTATTAATTTTATCACTACAATTTTTAATATAAAGTTAAAAGGGATTAAGTTAGATCAAATTTCTTTATTAATTTGATCTATTTTAATTACTACAATTTTGTTATTACTTTCATTACCTGTTTTAGCTGGTGCTATTACTATATTATTGACTGATCGTAATTTAAATACTAGATTTTTTGATTTTTCAGGGGGAGGAGACCCAG--------------------.

##### Holotype ♀.

Guanacaste, Sector Pitilla, Bullas, 10.98670, -85.38503, 440 meters, caterpillar collection date: 31/vii/2014, wasp eclosion date: 24/viii/2014. Depository: CNC.

***Host data*.** arctJanzen01 Janzen71434 (Erebidae) feeding on algae.

***Host caterpillar and holotype wasp voucher codes*.** 14-SRNP-71445, DHJPAR0056334.

##### Paratypes.

None.

##### Etymology.

*Aleiodesmikeiviei* is named in honor of Mike Ivie’s long-appreciated contributions to publicity for ACG, GDFCF, and now, BioAlfa.

**Figure 400.**
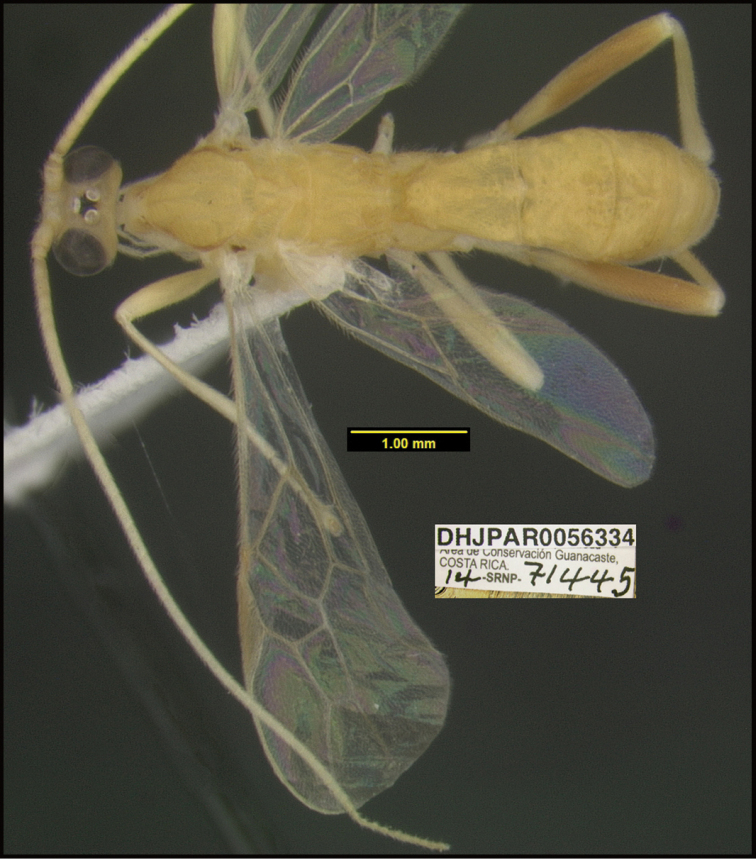
*Aleiodesmikeiviei*, holotype.

#### Aleiodes normwoodleyi

Taxon classificationAnimaliaHymenopteraBraconidae

Sharkey sp. nov.

http://zoobank.org/693E56DD-896D-4024-A9CA-15FE13027ABD

Figure 401

##### Diagnostics.

BOLD:AAJ4076. Consensus barcode. AGTTTTATACTTTTTATTTGGAATATGAGCAGGAATAATTGGCTTATCAATAAGCCTAATTATTCGATTAGAATTAAGAATAAGAGGAAGTATTCTTAAAAATGATCAAATTTATAATGGTATAGTAACTTTACATGCTTTTGTTATAATTTTTTTTATAGTTATACCAATTATAATTGGGGGGTTTGGAAATTGATTAATTCCATTAATGTTAGGAGCCCCTGATATAGCTTTCCCCCGCATAAATAATATAAGATTTTGATTATTAATTCCCTCTTTAATACTTCTATTAATTAGAGGATTAATTAATACAGGAGTAGGGACAGGATGAACTATATACCCCCCATTATCATCATTAATTGGCCATAATGGAATTTGTGTAGATATATCAATTTTTTCATTACATTTAGCTGGAGCCTCATCAATTATAGGAGCAATTAATTTTATTTCAACTATTTTTAATATAAACTTAATAACTATTAAAATAGATCAAATTACATTATTAATTTGCTATTTTAATTACTACAATTTTATTATTATTATCTTTACCAGTTTAGCAGGGGCAATTACTATACTATTAACAGATCGAAATTTAAATACAAGATTTTTG---------------------------------------------. The similar *Aleiodesleptocarina* Fortier, 2000 was reared in Costa Rica and is also a gregarious species. Its host is unknown and described simply as a “large caterpillar.” The two species differ in numerous ways: one of the most obvious is that the antenna of *A.leptocarina* are “honey yellow” differing from the melanic antenna of *A.normwoodleyi*.

##### Holotype ♀.

Alajuela, Sector San Cristobal, Finca San Gabriel, 10.87766, -85.39343, 645 meters, caterpillar collection date: 3/xii/2008, wasp eclosion date: 19/xii/2008. Depository: CNC.

***Host data*.***Dysschemaviuda* (Erebidae) feeding on *Zanthoxylumriedelianum* (Rutaceae). Gregarious parasitoid with 80 specimens emerging.

***Host caterpillar and holotype wasp voucher codes*.** 08-SRNP-6195, DHJPAR0030719.

##### Paratypes.

None.

##### Etymology.

*Aleiodesnormwoodleyi* is named in honor of Norm Woodley’s long-appreciated contributions to publicity for ACG, GDFCF, and now, BioAlfa.

##### Note.

This is the fourth species of *Aleiodes* known to be gregarious (Fortier 2000).

**Figure 401:**
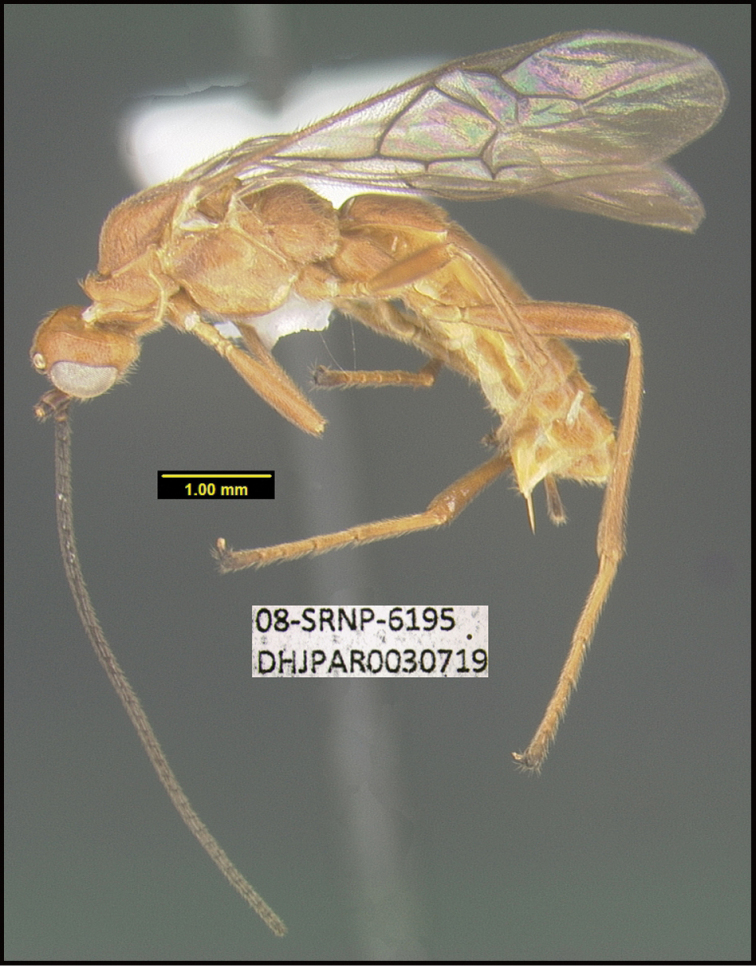
*Aleiodesnormwoodleyi*, holotype.

#### Aleiodes inga

Taxon classificationAnimaliaHymenopteraBraconidae

Shimbori & Shaw, 2020

Figure 402

##### Diagnostics.

BOLD:AAA5377. Consensus barcode. ATTTTATATTTTTTATTTGGGATATGATCTGGAATAATCGGTTTATCAATAAGATTAATTATTCGTTTAGAATTAAGATCAACTGGGAGAGTTTTAAAGAATGATCAGATTTATAATGGAATAGTTACATTACATGCATTTATTATAATTTTTTTTATAGTTATACCTGTAATACTAGGAGGATTTGGAAATTGATTAATTCCATTAATATTAGGAGCCCCTGATATAGCTTTCCCACGAATAAATAATATAAGATTTTGATTATTAATTCCATCTTTATTTTTACTTTTAATAAGAGGTGTAATAAATTCAGGTGTAGGAACTGGATGAACTATATACCCTCCTTTATCTTCATTAATTGGTCATAGAGGATTTTCTATTGATATATCTATTTTTTCTTTACATTTGGCTGGAATTTCTTCAATTATGGGGTCAATTAATTTTATTTCTACTATTTTTAATATAAATTTAATGAAAATTAAGATAGATCAAATCTCTTTATTTATTTGATCAGTTTTAATTACTACTATTTTGTTACTTCTTTCTTTACCAGTTTTAGCTGGGGCAATTACTATATTATTAACTGATCGAAATTTAAATACAAGATTTTTTGATTTTTCTGGAGGAGGAGATCCAATTTTATTTCAACATCTTTTT.

##### Imaged specimen ♀.

Alajuela, Sector Brasilia, Piedrona, 11.016, -85.359, 340 meters, caterpillar collection date: 21/ix/2010, wasp eclosion date: 30/ix/2010. Depository: CNC.

***Host data*.***Letismycerina* (Erebidae) feeding on *Ingaoerstediana* (Fabaceae).

***Host caterpillar and imaged wasp voucher codes*.** 10-SRNP-65094, DHJPAR0041207.

##### Paratypes.

None.

**Figure 402.**
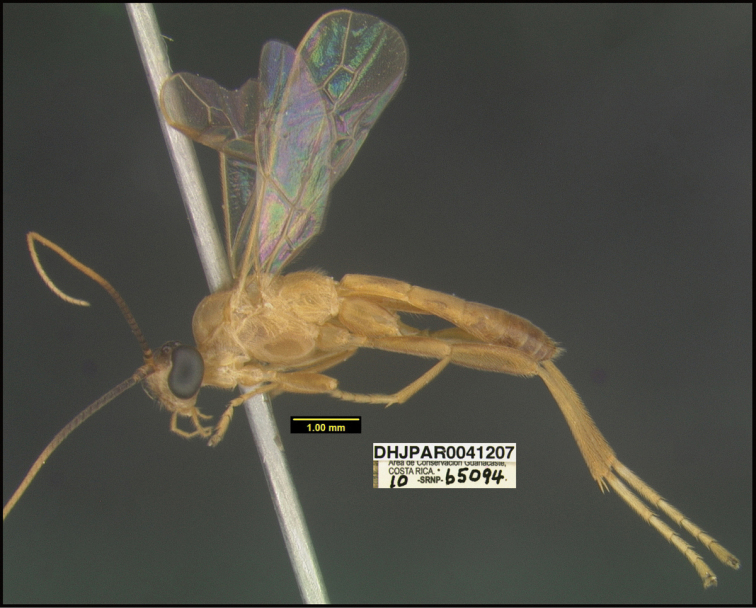
*Aleiodesinga* Shimbori & Shaw.

#### Aleiodes pammitchellae

Taxon classificationAnimaliaHymenopteraBraconidae

Sharkey sp. nov.

http://zoobank.org/D1E24ECC-5D86-4846-8DB4-494373589BEA

Figure 403

##### Diagnostics.

BOLD:AAD7872. Consensus barcode. GGTATTRTATTTTTTRTTTGGTATGTGATCAGGGATGGTTGGGATRTCAATAAGGTTAATTATTCGGTTAGAATTGGGAGTTTGTGGGAGAGTTTTAGGTAATGATCAAATTTATAATGGTATGGTTACTTTACATGCTTTTATTATAATTTTTTTTATAGTAATACCTATTATAATTGGAGGTTTTGGAAATTGATTAATTCCTTTAATATTAGGATCYCCGGATATAGCTTTCCCTCGTATGAATAATATGAGATTTTGAYTATTAATTCCTTCAATTTTATTATTRTTAATGAGAGGTGTTGTTAATGTTGGTGTAGGTACTGGGTGAACTATTTAYCCACCTTTATCTTCATTATTAGGTCATGGGGGGATATCTGTAGATTTGTCAATTTTTTCTATTCATTTAGCTGGGGCTTCTTCGATTATAGGAGCTATTAATTTTATTACTACAATTTTTAATATAAATTTATTTATAATTAAAATAGATCAAATTATATTATTTGTTTGATCTGTATTAATTACAGCTTTTTTATTATTATTATCTTTACCAGTTTTAGCTGGGGYAATTACAATATTATTAACAGATCGTAATATAAATACTACTTTTTTTGATTTTYCTGGAGGGGGYGATCCTATTTTATTTCAACACTTATTT.

##### Holotype ♀.

Guanacaste, Sector Santa Rosa, Vado Poza Salada, 10.79796, -85.64984, 8 meters, caterpillar collection date: 25/v/2006, wasp eclosion date: 24/v/2007. Depository: CNC.

***Host data*.***Toxonpruchaexcavata* (Erebidae) feeding on *Vachelliariparia* (Fabaceae).

***Host caterpillar and holotype wasp voucher codes*.** 06-SRNP-15047, DHJPAR0062177.

##### Paratypes.

DHJPAR0062411, DHJPAR0062410 light trapped. Depository: CNC.

##### Etymology.

*Aleiodespammitchellae* is named in honor of Pam Mitchell’s (RIP) long-appreciated contributions to publicity for ACG and GDFCF.

**Figure 403.**
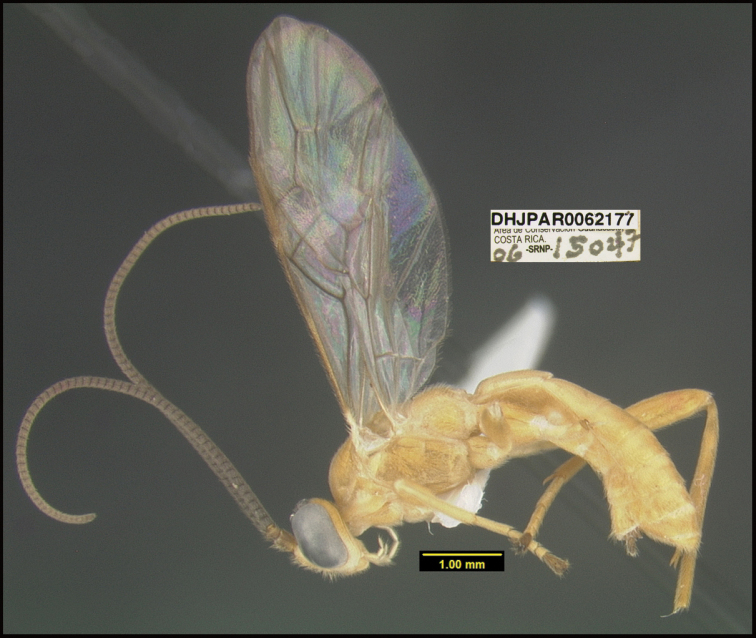
*Aleiodespammitchellae*, holotype.

#### Aleiodes pauljohnsoni

Taxon classificationAnimaliaHymenopteraBraconidae

Sharkey sp. nov.

http://zoobank.org/0B5E52A6-0306-4DC2-809B-32EA82873EBA

Figure 404

##### Diagnostics.

BOLD:ACM2562. Consensus barcode. AATATTATATTTTTTATTTGGAATATGAGCAGGAATAGTGGGTTCATCAATAAGATTAATTATTCGATTAGAATTAAGAACAAATGGGAGAATTTTAAAAAATGATCAAATTTATAATGGAATAGTAACATTACACGCTTTCATTATAATTTTTTTTATAGTTATACCAATTATAATTGGAGGATTCGGAAATTGATTAATTCCATTAATATTAGGGGCTCCTGACATAGCATTCCCACGAATAAATAATATAAGATTTTGATTATTAATCCCTTCTTTAATACTTTTATTAATTAGAGGGATAATTAATACAGGTGTTGGAACTGGATGAACAATATACCCCCCATTATCATCATTAATCGGACACAATGGTCTTTCAGTAGATATATCTATTTTTTCTTTACATTTAGCAGGGGCATCATCTATTATAGGAGCAATTAATTTTATTTCAACTATTTTTAATATAAACTTAATAAAAATTAAAATAGATCAAATTATATTATTAATTTGATCTATTTTAATTACTACAATTTTATTATTATTATCATTACCAGTTTTAGCAGGAGCTATTACCATATTATTAACAGACCGAAATTTAAATACAAGATTTTTTGATTTCTCAGGAGGGGGAGACCCAATTTTATTCCAACACCTTTTT.

##### Holotype ♀.

Guanacaste, Sector Cacao, Sendero Arenale, 10.92471, -85.46738, 1080 meters, caterpillar collection date: 31/x/2013, wasp eclosion date: 20/xi/2013. Depository: CNC.

***Host data*.***Erosiaveninotata* (Uraniidae) feeding on *Randiagrandifolia* (Rubiaceae)

***Host caterpillar and holotype wasp voucher codes*.** 13-SRNP-36425, DHJPAR0054919.

##### Paratype.

DHJPAR0054923. Depository: CNC.

##### Etymology.

*Aleiodespauljohnsoni* is named in honor of Paul Johnson’s long-appreciated contributions to publicity for ACG, GDFCF, and now, BioAlfa.

**Figure 404.**
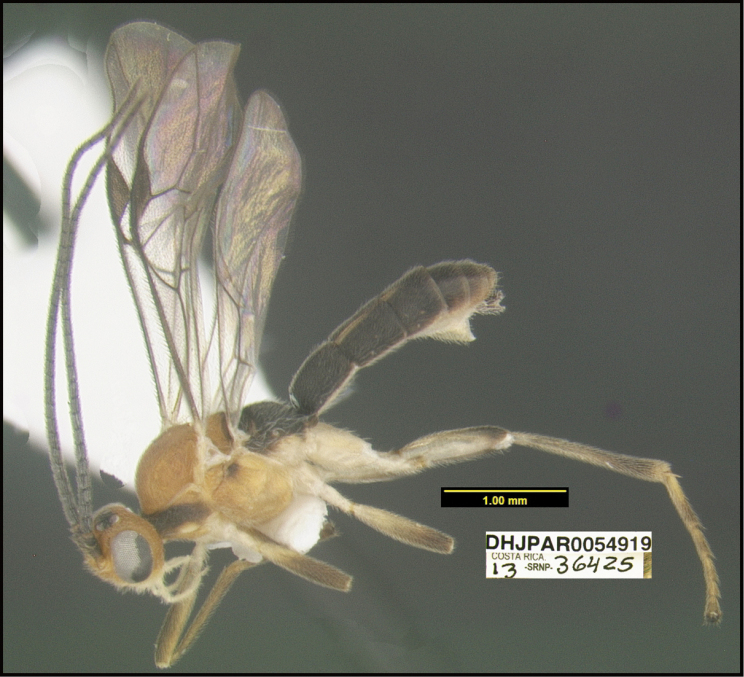
*Aleiodespauljohnsoni*, holotype.

#### Aleiodes rosewarnerae

Taxon classificationAnimaliaHymenopteraBraconidae

Sharkey sp. nov.

http://zoobank.org/A7F3F46D-7E43-4A24-BF2A-C86E57743F95

Figure 405

##### Diagnostics.

BOLD:AAW1573. Consensus barcode. ATTTTATATTTCTTATTTGGAATATGAGCAGGTATAATTGGAATATCAATAAGATTAATTATTCGGTTAGAATTAAGATCTACTGGTAGAATTTTAAAAAATGATCAAATTTATAACAGTATAGTAACTTTACATGCATTTATTATAATTTTTTTTATAGTTATACCTATTATAATTGGAGGGTTTGGTAATTGATTAATCCCTTTAATACTAGGAGCCCCAGATATAGCCTTCCCACGAATAAATAATATAAGATTCTGATTATTAATTCCTTCATTATTATTATTATTAATAAGAGGAATTATTAATTCAGGTGTAGGCACAGGATGAACAATATACCCTCCATTATCATCACTAATTGGTCATAGAGGTATCTCCGTAGATTTATCTATTTTTTCTTTACATTTAGCAGGAGCTTCATCTATTATAGGAGCTATTAATTTCATTTCAACTATTTTCAATATAAATTTAATAATAATTAAAATAGACCAAATTATATTATTAGTATGAGCAATTTTAATTACTACAATTTTATTATTATTATCTTTACCAGTTTTAGCAGGAGCAATTACRATATTATTAACAGACCGAAATTTAAATACAAGATTTTTTGACTTTTCTGGAGGTGGGGACCCCATTTTATTTCAACATCTCTTT.

##### Holotype ♂.

Guanacaste, Sector Pitilla, Pasmompa, 11.01926, -85.40997, 440 meters, caterpillar collection date: 26/xii/2007, wasp eclosion date: 11/i/2008. Depository: CNC.

***Host data*.***Pleuroprucharudimentaria* (Geometridae) feeding on *Ingaoerstediana* (Fabaceae).

***Host caterpillar and holotype wasp voucher codes*.** 07-SRNP-34419, DHJPAR0023522.

##### Paratype.

Host = *Pleuroprucharudimentaria*: DHJPAR0023524. Depository: CNC.

##### Etymology.

*Aleiodesrosewarnerae* is named in honor of Rose Warner’s (RIP) long-appreciated contributions to publicity for ACG and GDFCF.

**Figure 405.**
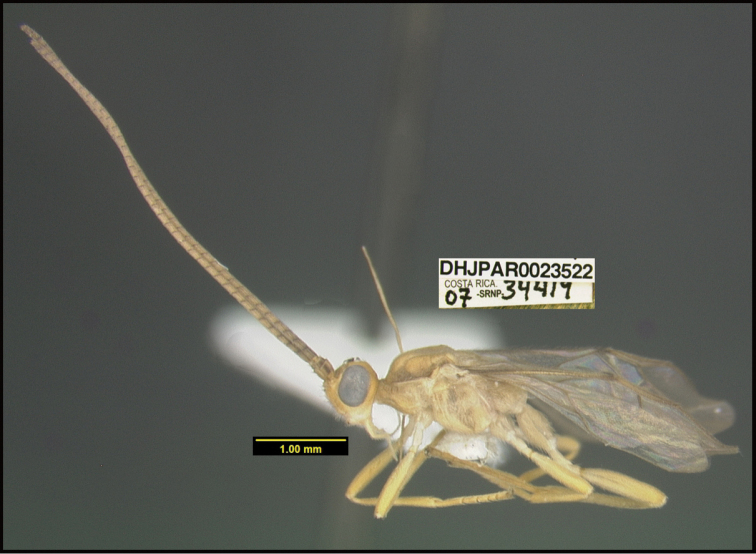
*Aleiodesrosewarnerae*, holotype.

#### Aleiodes steveashei

Taxon classificationAnimaliaHymenopteraBraconidae

Sharkey sp. nov.

http://zoobank.org/34638CD7-C157-4389-8F4D-36F0445F1EA1

Figure 406

##### Diagnostics.

BOLD:AAJ4092. Consensus barcode. AGTTTTATATTTCTTATTTGGTATATGAGCAGGAATAATTGGTATATCAATAAGACTAATTATTCGATTAGAATTAAGAACCAGAGGTAGAATCCTTAAAAATGATCAAATTTATAATGGAATAGTAACTTTACATGCTTTTATTATAATTTTTTTTATAGTTATACCAATTATAATTGGAGGATTTGGAAACTGATTAATCCCATTAATATTAGGAGCTCCTGATATAGCCTTCCCACGAATAAATAATATAAGGTTTTGATTATTAATTCCTTCCTTAATACTTTTATTAATTAGAGGGATAATCAATACAGGAGTAGGGACAGGATGAACTATATACCCTCCCTTATCTTCATTAATTGGTCATAATGGAATTTCAGTAGATATATCAATTTTTTCACTACATTTAGCTGGGGCCTCATCAATTATAGGAGCAGTTAATTTTATTTCTACTATTTTTAATATAAATTTAATAATAATTAAAATAGATCAAATTACCTTATTAATTTGATCTATTTTAATTACTACAATCTTATTATTATTATCTTTACCAGTTTTAGCAGGTGCAATTACTATRTTATTAACAGATCGAAATTTAAATACAAGATTTTTTGATTTTTCAGGGGGGGGAGACCCAATTTTATTTCAACATCTTTTT.

##### Holotype ♀.

Guanacaste, Sector Cacao, Estación Cacao, 10.92691, -85.46822, 1150 meters, caterpillar collection date: 29/vii/2010, wasp eclosion date: 20/vii/2010. Depository: CNC.

***Host data*.***Tachudadiscreta* (Notodontidae) feeding on *Serjaniaschiedeana* (Sapindaceae).

***Host caterpillar and holotype wasp voucher codes*.** 10-SRNP-35614, DHJPAR0040461.

##### Paratypes.

Hosts = *Tachudadiscrete*, *Lysana* Janzen02, and *Elymiotisplechelm* (all Notodontidae). DHJPAR0047201. DHJPAR0021180, DHJPAR0062073, DHJPAR0062081, DHJPAR0062112. Depository: CNC.

##### Etymology.

*Aleiodessteveashei* is named in honor of Steve Ashe’s (RIP) long-appreciated contributions to publicity for ACG and GDFCF.

**Figure 406.**
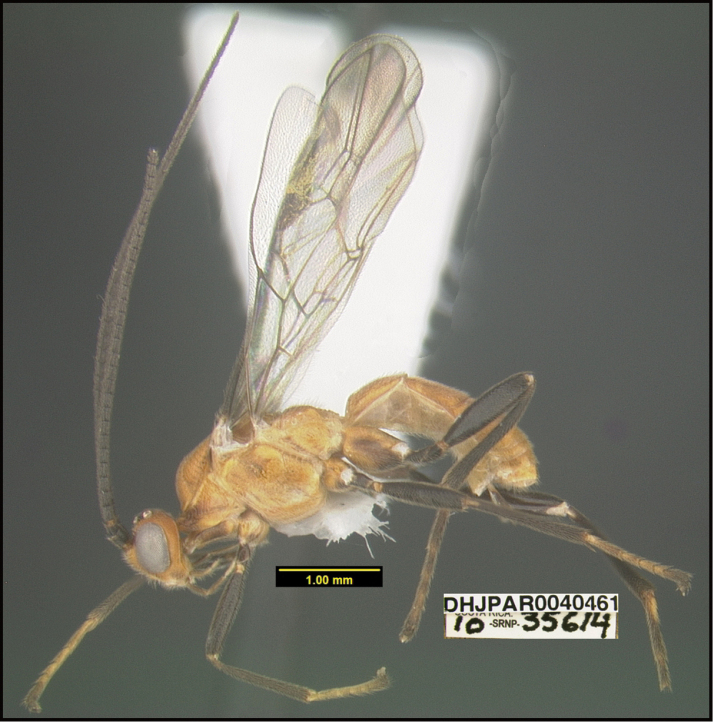
*Aleiodessteveashei*, holotype.

#### Aleiodes terryerwini

Taxon classificationAnimaliaHymenopteraBraconidae

Sharkey sp. nov.

http://zoobank.org/79FB78BF-50C2-4670-ADD7-EF8132E78AF8

Figure 407

##### Diagnostics.

BOLD:ADJ0647. Consensus barcode. TATTTTATATTTTTTATTTGGTATATGAGCAGGTATAATTGGAATATCAATAAGATTAATTATTCGATTAGAATTAAGAATTAATGGAAGAATTTTAAAAAATGATCAAATTTATAATGGAATAGTAACTTTACATGCTTTTATTATAATTTTTTTTATAGTAATACCAATTATAATTGGAGGGTTTGGAAATTGATTAATTCCATTAATATTAGGAAGACCTGATATAGCTTTCCCACGTATAAATAATATAAGATTTTGATTATTAATTCCTTCTTTAATACTTTTATTAATTAGAGGAATTATTAATACGGGAGTAGGAACAGGATGAACTATATACCCTCCTTTATCTTCACTTATTGGACATAATGGAATTTCCGTAGATATATCAATTTTTTCTTTACACTTAGCAGGGGCTTCATCTATTATAGGAGCAATTAATTTTATTTCAACTATTTTTAATATAAATTTAATAATAATTAAAATAGACCAAATTACATTATTAATTTGATCTATTTTAATTACTGCAATTTTATTACTATTATCTTTACCAGTCTTAGCAGGAGCTATTACTATATTATTAACAGATCGAAATCTAAACACAAGTTTTTTTGATTTTTCAGGAGGAGGAGACCCAATTTTATTCCAACATCTTTTT.

##### Holotype ♀.

Guanacaste, Sector Pitilla, Sendero Manguera, 10.99590, -85.39842, 470 meters, caterpillar collection date: 10/v/2017, wasp eclosion date: 21/v/2017. Depository: CNC.

***Host data*.***Paectes* Poole10 (Euteliidae) feeding on *Mosquitoxylumjamaicense* (Anacardiaceae).

***Host caterpillar and holotype wasp voucher codes*.** 17-SRNP-71490, DHJPAR0061494.

##### Paratypes.

None.

##### Etymology.

*Aleiodesterryerwini* is named in honor of Terry Erwin’s (RIP) long-appreciated contributions to publicity for ACG, GDFCF, and BioAlfa existence.

**Figure 407.**
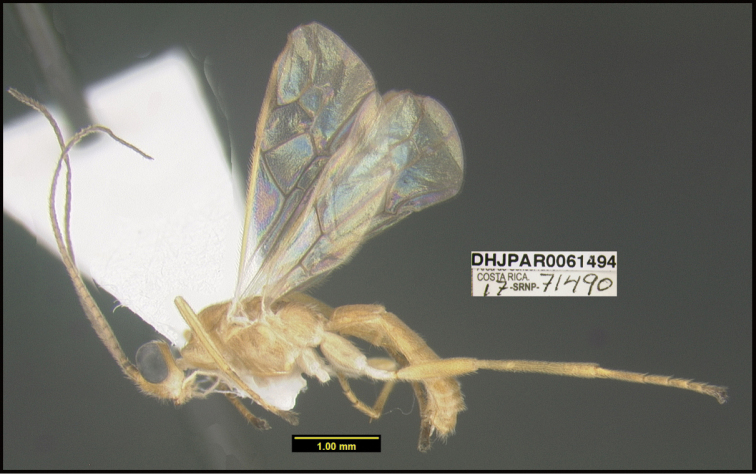
*Aleiodesterryerwini*, holotype.

#### Aleiodes willsflowersi

Taxon classificationAnimaliaHymenopteraBraconidae

Sharkey sp. nov.

http://zoobank.org/F299D1A5-E8EA-42B0-A451-3E73A5E5CEDF

Figure 408

##### Diagnostics.

BOLD:AAM1704. Consensus barcode. TGTTTTATATTTTTTATTCGGATTATGAGCAGGAATAATTGGAATATCAATAAGATTAATTATTCGACTAGAGTTAAGAACTAGAGGGAGAATATTAAAAAATGATCAAATTTATAATGGAATAGTAACCTTACATGCTTTTATTATAATTTTTTTTATAGTTATACCAATTATAATTGGGGGGTTTGGGAATTGATTAATTCCTTTAATATTAGGTGCCCCTGATATAGCCTTCCCACGTATAAATAATATAAGATTTTGATTATTAATTCCTTCTTTAATACTTTTATTAATTAGAGGAATAATTAATTCAGGGGTAGGGACAGGATGAACTATATATCCCCCCCTATCATCATTAATTGGTCATAACGGGGCTTCAGTAGATATATCAATTTTTTCTTTACATTTAGCGGGGGCATCCTCAATTATAGGAGCAATTAATTTTATTTCAACTATTTTTAATATAAATTTAATAAATATTAAAATAGATCAAATTATATTACTAATTTGATCTATTTTAATTACTACAATTCTATTATTACTATCATTACCAGTATTAGCGGGGGCAATCACAATACTATTAACAGACCGAAATTTAAATACAAGATTTTTTGATTTTTCAGGAGGGGGGGATCCAATTTTATTCCAACATCTTTTT.

##### Holotype ♀.

Guanacaste, Sector Del Oro, Quebrada Oro, 11.03376, -85.47715, 290 meters, caterpillar collection date: 27/viii/2009, wasp eclosion date: 10/ix/2009. Depository: CNC.

***Host data*.***Antaeotricha* Janzen730 (Depressariidae) feeding on *Cupaniacinerea* (Sapindaceae).

***Host caterpillar and holotype wasp voucher codes*.** 09-SRNP-22602, DHJPAR0037966.

##### Paratypes.

None.

##### Etymology.

*Aleiodeswillsflowersi* is named in honor of Wills Flowers’ long-appreciated contributions to publicity for ACG, GDFCF, and now, BioAlfa.

**Figure 408.**
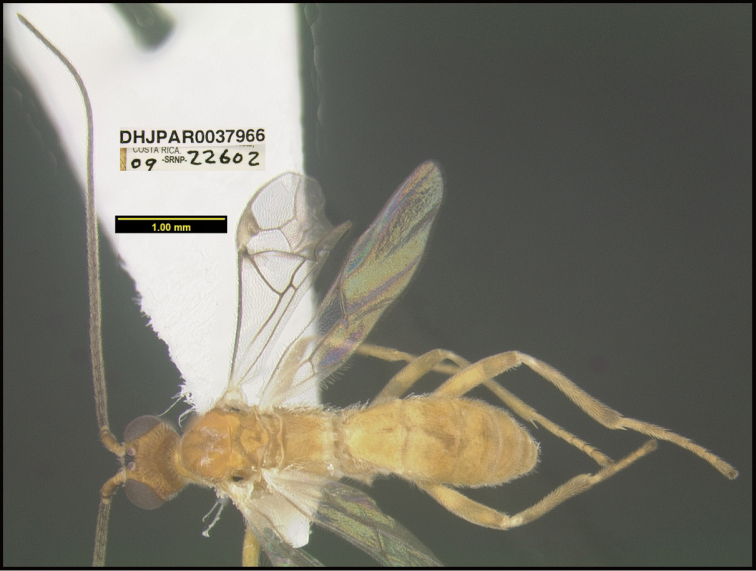
*Aleiodeswillsflowersi*, holotype.

### *Choreborogas* Whitfield, 1990

The genus is restricted to the New World with nine described species, none of which is recorded from Costa Rica. Members are parasitoids of leaf-mining Lyonetiidae and Gracillariidae (Whitfield 1990).

#### Choreborogas andydeansi

Taxon classificationAnimaliaHymenopteraBraconidae

Sharkey sp. nov.

http://zoobank.org/6272EAEB-4921-4804-A7A8-4C6967E2FE78

Figure 409

##### Diagnostics.

BOLD:ADV9625. Consensus barcode. TTATATTTTTTTTTTGGTATATGATCAGGTATGTTGGGGTTATCAATAAGATTAATTATTCGTTTTGAATTGGGGGTTCCTGGTTCATTTTTAGGTAATGATCAAATTTATAATAGAATTGTTACTGCTCATGCTTTAGTTATAATTTTTTTTATAGTTATACCTGTTATAATTGGTGGGTTTGGAAATTGATTAATTCCCTTAATATTAGGAGCTCCTGATATGGCTTTCCCTCGTATAAATAATATAAGATTTTGATTATTAATTCCTTCTATTTTAATATTATTAATTAGATCTTTGGTTAATGTTGGGGTAGGTACAGGTTGAACAATTTATCCTCCATTATCTTCTTTGATAGGTCATGGGGGTATTTCAGTTGATTTGGCTATTTTTTCTTTACATTTAGCAGGGGCATCTTCAATTATAGGGGCTATTAATTTTATTTCAACTATTTTCAATATAAATTTATTTTCAATGAAAATAGATCAAATTATATTATTAGTTTGATCTGTTTTAATTACTGCTTTCTTACTGTTGTTATCTTTACCTGTTTTGGCTGGGGCAATTACTATATTATTGTTTGATCGTAATATTAATAGAACATTTTTTGATTTTTCAGGGGGGGGGGATCCTATTTTGTTTCAACATTTATT-------------------------------------------.

##### Holotype ♀.

Guanacaste, Sector Pailas Dos, PL12-3, 10.7631, -85.3344, 8200 meters, 18/xii/2014, Malaise trap. Depository: CNC.

***Host data*.** None

***Holotype voucher code*.**BIOUG44129-A07.

##### Paratypes.

None.

##### Etymology.

*Choreborogasandydeansi* is named in honor of Andy Deans’ long-appreciated contributions to publicity for ACG, GDFCF, and now, BioAlfa.

**Figure 409.**
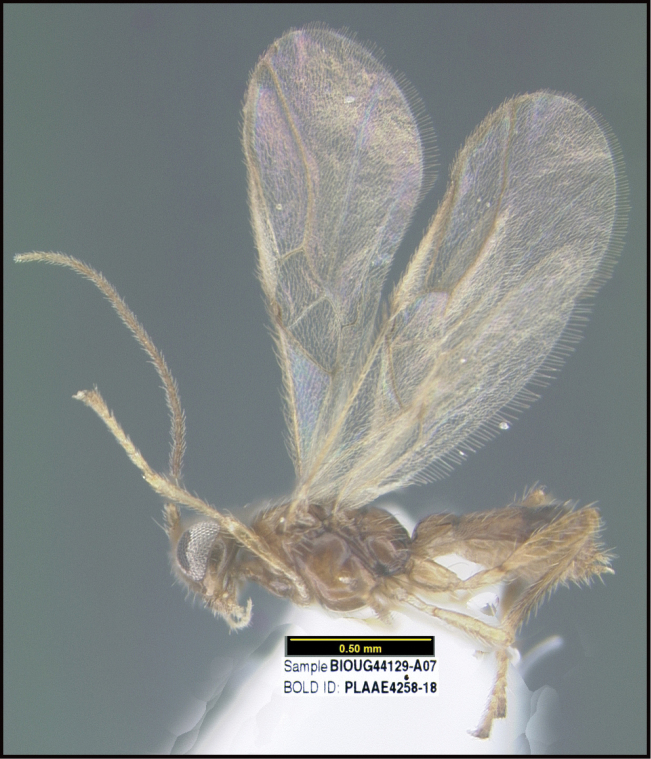
*Choreborogasandydeansi*, holotype.

#### Choreborogas eladiocastroi

Taxon classificationAnimaliaHymenopteraBraconidae

Sharkey sp. nov.

http://zoobank.org/0BB7F00F-6C40-4835-B94F-0EBB21556FB6

Figure 410

##### Diagnostics.

BOLD:ACS0876. Consensus barcode. TTATATTTTTTTTTTGGTATATGATCAGGTATGTTGGGGTTATCAATAAGATTAATTATTCGTTTTGAATTGGGGGTTCCTGGTTCATTTTTAGGTAATGATCAAATTTATAATAGAATTGTTACTGCTCATGCTTTAGTTATAATTTTTTTTATAGTTATACCTGTTATAATTGGTGGGTTTGGAAATTGATTAATTCCCTTAATATTAGGAGCTCCTGATATGGCTTTCCCTCGTATAAATAATATAAGATTTTGATTATTAATTCCTTCTATTTTAATATTATTAATTAGATCTTTGGTTAATGTTGGGGTAGGTACAGGTTGAACAATTTATCCTCCATTATCTTCTTTGATAGGTCATGGGGGTATTTCAGTTGATTTGGCTATTTTTTCTTTACATTTAGCAGGGGCATCTTCAATTATAGGGGCTATTAATTTTATTTCAACTATTTTCAATATAAATTTATTTTCAATGAAAATAGATCAAATTATATTATTAGTTTGATCTGTTTTAATTACTGCTTTCTTACTGTTGTTATCTTTACCTGTTTTGGCTGGGGCAATTACTATATTATTGTTTGATCGTAATATTAATAGAACATTTTTTGATTTTTCAGGGGGGGGGGATCCTATTTTGTTTCAACATTTATT-------------------------------------------.

##### Holotype ♀.

Guanacaste, Pailas Dos, PL12-9, 10.76, -85.3341, 809 meters, 17/vii/2014, forest, Malaise trap. Depository: CNC.

***Host data*.** None.

***Holotype voucher code*.**BIOUG29393-G11.

##### Paratypes.

BIOUG18822-B04, BIOUG29473-F10, BIOUG29531-D01, BIOUG30966-F05.

##### Etymology.

*Choreborogaseladiocastroi* is named in honor of Eladio Castro’s long-appreciated contributions to publicity for ACG, GDFCF, and now, BioAlfa.

**Figure 410.**
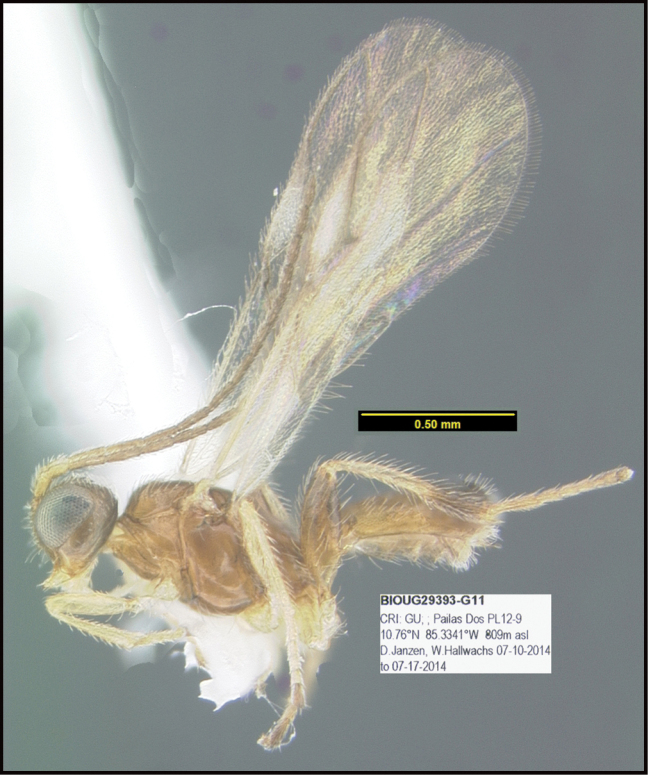
*Choreborogaseladiocastroi*, holotype.

#### Choreborogas felipechavarriai

Taxon classificationAnimaliaHymenopteraBraconidae

Sharkey sp. nov.

http://zoobank.org/E235480E-F070-42E8-8367-1FFCE8F27DAD

Figure 411

##### Diagnostics.

BOLD:ADA2251. Consensus barcode. ATATGATCTGGTATGTTAGGTCTTTCTATAAGATTGATTATTCGTTTTGAATTAAGATCTTGTGGTTCATTTTTAGGAAATGACCAAATTTATAATAGTATTGTTACAGCTCATGCCTTAGTTATAATTTTTTTTATGGTTATACCTGTTATAATTGGTGGTTTTGGTAATTGATTAATTCCTTTAATGTTAGGGGCCCCTGATATAGCTTTTCCTCGAATAAATAATATAAGTTTTTGATTATTAATTCCTTCTATTTTTTTGTTGATAATTAGTTCTATTGTTAATGTTGGAGTAGGTACTGGTTGAACTATTTATCCTCCATTGTCTTCTTTGATGGGTCATAGTGGTATTTCTATGGATTTAGCTATTTTTTCTTTACATTTGGCTGGGGCTTCTTCAATTATAGGGGCTATTAATTTTATTACTACTATTTTTAATATAAATTTATTTTATATGAAAATAGATCAAATTGTTTTGTTAGTTTGGTCTGTTTTAATTACTGCTTTTTTATTATTGTTATCTTTACCTGTTTTAGCTGGAGCTATTACTATATTATTATTTGATCGT------------------------------------------------------------.

##### Holotype ♀.

Guanacaste, Sector San Cristobal, Estación San Gerardo, 10.8801, -85.389, 575 meters, rain forest/laguna, 11/xi/2013, Malaise trap. Depository: CNC.

***Host data*.** None.

***Holotype voucher code*.**BIOUG28042-C10.

##### Paratypes.

None.

##### Etymology.

*Choreborogasfelipechavarriai* is named in honor of Felipe Chavarria’s long-appreciated contributions to publicity for ACG, GDFCF, and now, BioAlfa.

**Figure 411.**
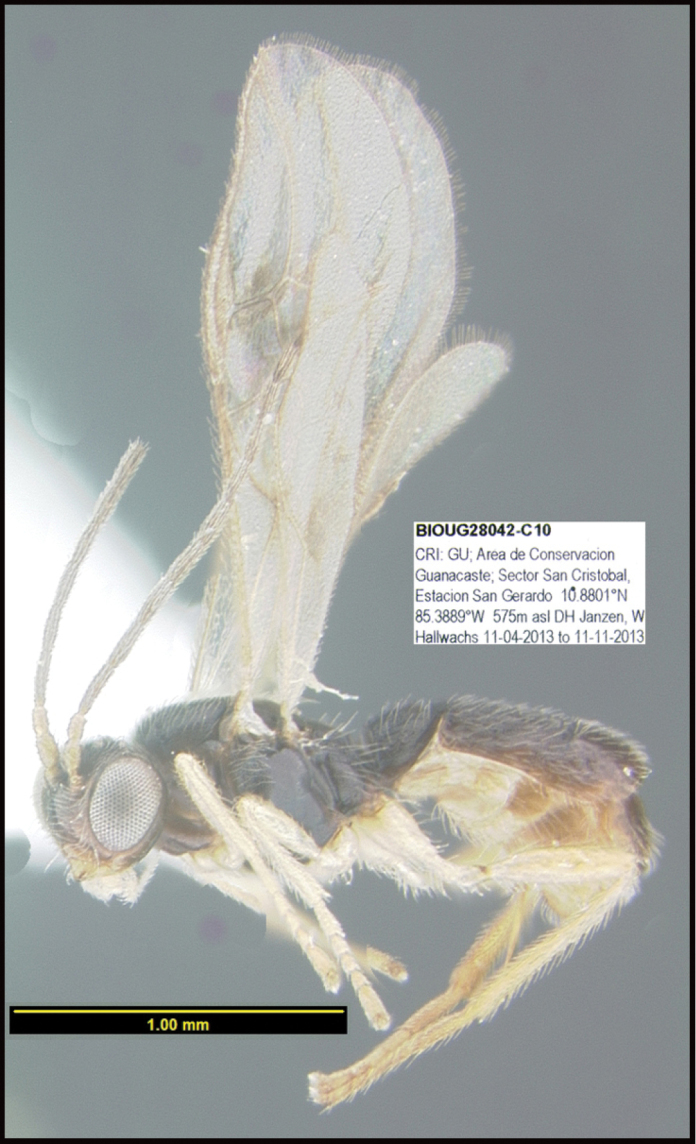
*Choreborogasfelipechavarriai*, holotype.

#### Choreborogas frankjoycei

Taxon classificationAnimaliaHymenopteraBraconidae

Sharkey sp. nov.

http://zoobank.org/361B14BB-F810-459E-921C-714DA18ED291

Figure 412

##### Diagnostics.

BOLD:ADB6582. Consensus barcode. ATATGATCTGGTATGTTAGGTTTATCTATAAGAATAATTATTCGTTTTGAATTAGGGGTTCCTGGTTCATTATTAGGTAATGATCAAATTTATAATAGGATTGTTACAGCTCATGCTTTAGTAATAATTTTTTTTATAGTTATGCCTGTAATGATTGGTGGGTTTGGTAATTGATTAATTCCTTTAATATTGGGGGCTCCTGATATAGCTTTCCCTCGTATAAATAATATAAGATTTTGATTATTAATTCCTTCTATTTTTTTATTATTGATTAGTTCTATTGTTAATGTTGGGGTTGGTACTGGGTGGACTATTTATCCACCACTTTCTTCTTTATTAGGTCATGGGGGTATATCTGTTGATTTAGCTATTTTTTCCTTACATTTAGCGGGGGTTTCTTCAATTATGGGAGCTATTAATTTTATTTCAACTATTTTTAATATAAATTTATTTCATATGAAAATAGATCAAATTATATTATTAATCTGATCTGTTTTAATTACTGCTTTTTTATTATTATTGTCATTACCAGTTTTAGCAGGGGCTATTACTATATTATTATTTGATCGT----------------------------------------------------------------------.

##### Holotype ♀.

Guanacaste, Pailas Dos, PL12-3, 10.7631, -85.3344, 820 meters, 13/ii/2014, Malaise trap. Depository: CNC.

***Host data*.** None.

***Holotype voucher code*.**BIOUG29660-C05.

##### Paratypes.

None.

##### Etymology.

*Choreborogasfrankjoycei* is named in honor of Frank Joyce’s long-appreciated contributions to publicity for ACG, GDFCF, and now, BioAlfa.

**Figure 412.**
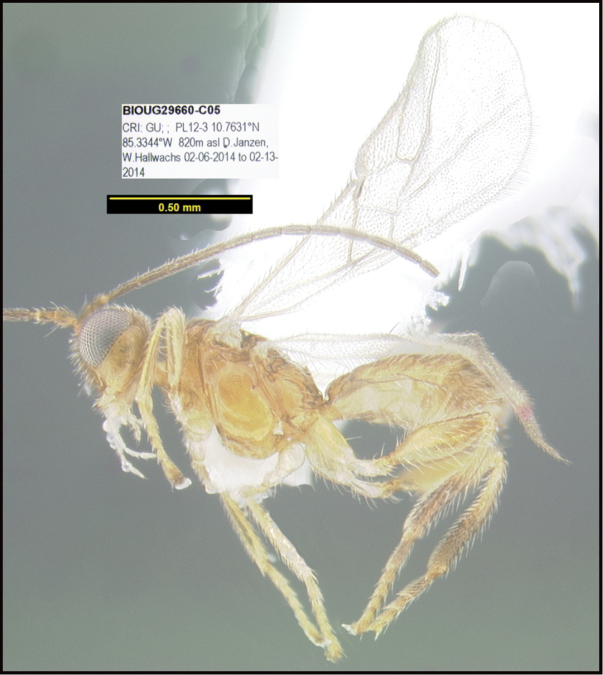
*Choreborogasfrankjoycei*, holotype.

#### *Clinocentrus* Haliday, 1833

The genus is worldwide in distribution with six described New World species, none of the holotypes of which are known from Costa Rica or nearby countries. Members are parasitoids of concealed caterpillars (Shaw 1983).

##### Clinocentrus andywarreni

Taxon classificationAnimaliaHymenopteraBraconidae

Sharkey sp. nov.

http://zoobank.org/04DB91AB-C206-4267-8023-B5364F26AF5B

Figure 413

###### Diagnostics.

BOLD:ACB2341. Consensus barcode. AGTTTTATATTTTATTTTTGGTATTTGATCTGGTTTAGTAGGTCTTTCTATGAGGTTAATTATTCGTTTAGAGTTGGGTATTCCTGGTAGATTATTAGGTAATGATCAACTTTATAATGTTATAGTTACTGCTCATGCTTTTGTTATAATTTTTTTTATAGTTATACCAATTATAATTGGGGGATTTGGAAATTGGTTAATTCCTTTAATATTAGGAGCTCCTGATATAGCTTTCCCTCGTATAAATAATATAAGATTTTGATTATTAATTCCATCATTATTTATATTAGTAATGAGAGGTTTATTAAATGTAGGTGTAGGAACAGGATGAACAGTTTACCCTCCTTTATCTTCATTAATTGGTCATAGAGGTATTTCAGTAGATTTAGCTATTTTTTCTTTACATTTAGCTGGAGCTTCATCAATTATGGGGGCAATTAATTTTATTTCTACTATTTTTAATATAAATTTATTAATAATTAAATTAGATCAAATAAGATTATTTATTTGATCAGTTTTAATTACAGCAGTTTTATTATTATTATCTTTACCAGTTTTAGCTGGCGCAATTACTATATTATTAACTGATCGTAATTTAAATACTACTTTTTTTGATTTTTCTGGTGGAGGGGATCCTATTTTATTTCAGCATTTATTT.

###### Holotype ♀.

Alajuela, Sector San Cristobal, Sendero Corredor, 10.87868, -85.38963, 620 meters, caterpillar collection date: 23/iii/2012, wasp eclosion date: 08/iv/2012. Depository: CNC.

***Host data*.***Antaeotricha* Janzen146 (Depressariidae) feeding on *Lonchocarpusoliganthus* (Fabaceae).

***Host caterpillar and holotype wasp voucher codes*.** 12-SRNP-1189, DHJPAR0049287.

###### Paratypes.

None.

###### Etymology.

*Clinocentrusandywarreni* is named in honor of Andy Warren’s long-appreciated contributions to publicity for ACG, GDFCF, and now, BioAlfa.

**Figure 413.**
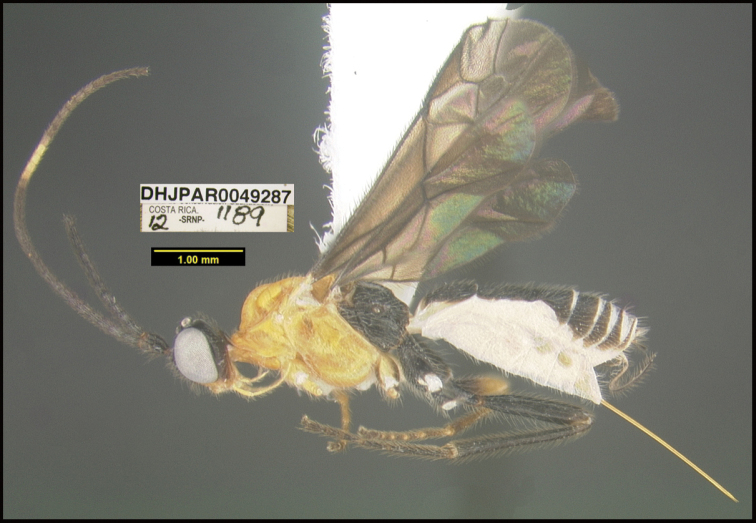
*Clinocentrusandywarreni*, holotype.

##### Clinocentrus angelsolisi

Taxon classificationAnimaliaHymenopteraBraconidae

Sharkey sp. nov.

http://zoobank.org/20460EFC-4BCE-4A18-9ED8-AE8495F76D5A

Figure 414

###### Diagnostics.

BOLD:ACL3899. Consensus barcode. AGTTTTATATTTTTTATTTGGAATTTGATCAGGATTACTAGGTTTATCAATAAGTATAATTATTCGATTAGAATTAGGTATATCAGGTAGATTATTAGGCAATGATCAAATTTATAATGTTATGGTTACAGCTCATGCTTTTGTTATAATTTTTTTTATGGTTATACCAATTATAATTGGAGGATTTGGTAATTGATTAATTCCTTTAATATTAGGTGCACCTGACATAGCTTTTCCCCGTATAAATAATATAAGTTTTTGGTTATTAATTCCTTCTTTAATATTATTATTATTAAGATCTATATTAAATGTTGGTGTAGGTACAGGATGGACTGTTTATCCTCCTTTATCTTCATTAATTGGTCATGGAGGGATATCTGTAGATTTAGCTATTTTTTCTTTACATTTAGCTGGTATTTCATCTATTATAGGTGCTATTAATTTTATTTCAACAATTTTTAATATAAGATTATTAACTTTAAAATTAGATCAAATAAGTTTATTTGTATGGTCAGTATTAATTACTGCTTTTTTATTATTATTATCTTTACCTGTATTAGCAGGTGCTATTACTATATTATTAACTGATCGTAATTTAAATACTACATTTTTTGATTTTTCTGGTGGAGGTGACCCTATTTTATTTCAACATTTATTT.

###### Holotype ♀.

Guanacaste, Sector Santa Rosa, Bosque San Emilio, 10.8438, -85.6138, 300 meters, 28/i/2013, Malaise trap. Depository: CNC.

***Holotype voucher code*.**BIOUG18290-G11.

###### Paratypes.

BIOUG10016-F03, BIOUG10016-F07, BIOUG10017-C03, BIOUG10017-H11, BIOUG17569-C11, BIOUG13946-A11, BIOUG18290-B06, BIOUG18290-D09, BIOUG18398-B01, BIOUG18398-C09, BIOUG18398-C11, BIOUG18398-G06. Depository: CNC.

###### Etymology.

*Clinocentrusangelsolisi* is named in honor of Angel Solis’ long-appreciated contributions to publicity for ACG, GDFCF, and now, BioAlfa.

**Figure 414.**
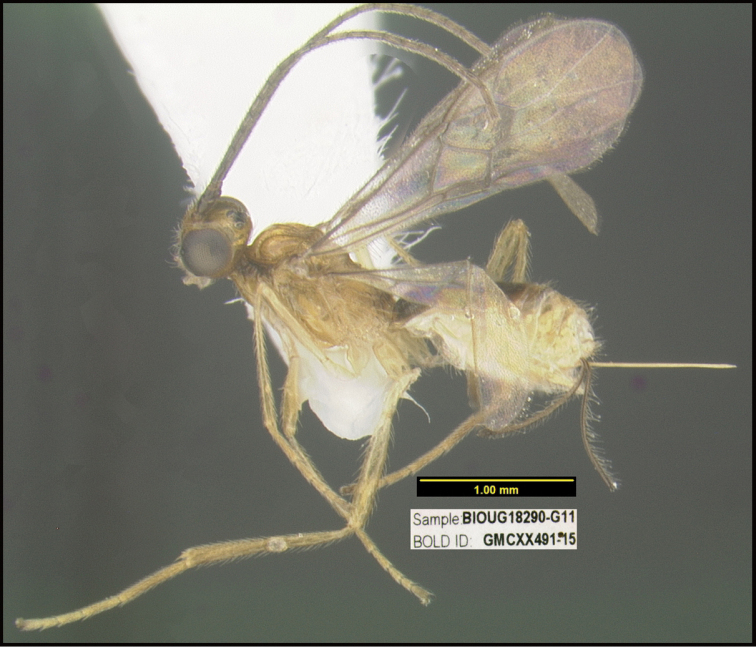
*Clinocentrusangelsolisi*, holotype.

#### *Cystomastax* Szépligeti, 1904

The genus is restricted to the neotropics with six described species, none of which is known from Costa Rica or Central America. Members are parasitoids of concealed caterpillars (Shaw 1983). Here we report the first three host records for the genus, all on three very different Erebidae, a vine, a shrub, and a tree.

##### Cystomastax alexhausmanni

Taxon classificationAnimaliaHymenopteraBraconidae

Sharkey sp. nov.

http://zoobank.org/6B249CCE-B4CB-41E4-8C93-A8E0C62F00EB

Figure 415

###### Diagnostics.

BOLD:AAJ5011. Consensus barcode. AATTTTATATTTTATTTTTGGTATTTGAGCTGGTATATTAGGTTTGTCTATAAGAATTATTATTCGTATAGAATTAATTAATCCTATGGGT---TTAATTAAAAATGATAATATTTATAATAGTATAGTAACGTCTCATGCATTTGTAATAATTTTTTTTATAGTTATACCAATTATAATTGGTGGATTTGGAAATTGATTAATTCCTTTAATATTAGGATCTCCTGATATAGCTTTTCCTCGTATAAATAATATAAGATTTTGGTTATTAATTCCTTCTTTATTTTTATTATTAATAAGGTCTTTTATTAATGTTGGTGTTGGTACAGGATGAACTATATATCCTCCTTTATCTTCTTTTTTAGGTCATAGTGGTATATCAGTAGATGTGGCTATTTTTTCTTTGCATTTAGCTGGTGCTTCCTCAATTATAGGTTCTATTAATTTTATTTCTACAATTTTTAATATAAAATTGTTAAATTTAAAATTAGATCAAATAAGATTGTTTATTTGGTCTGTTTTAATTACAGTGATTTTATTGTTGTTATCATTACCTGTTTTAGCTGGTGCTATTACAATGTTATTAACGGATCGTAATTTAAATACTACTTTTTTTGATTTTTCAGGGGGAGGGGATCCTATTTTATTTCAGCATTTATTT.

###### Holotype ♂.

Guanacaste, Sector Pitilla, Calma, 11.00986, -85.39213, 412 meters, caterpillar collection date: 03/19/2014, wasp eclosion date: 04/08/2014. Depository: CNC.

***Host data*.***Myrmecopsisstrigose* (Erebidae) feeding on *Vailiaanomala* (Asclepiadaceae).

***Host caterpillar and holotype wasp voucher codes*.** 14-SRNP-70673, DHJPAR0055780.

###### Paratypes.

None.

###### Other material.

A specimen (AL0391) in the same BIN was collected in Belize. It was not examined.

###### Etymology.

*Cystomastaxalexhausmanni* is named in honor of Alex Hausmann’s long-appreciated contributions to publicity for ACG, GDFCF, and now, BioAlfa.

**Figure 415.**
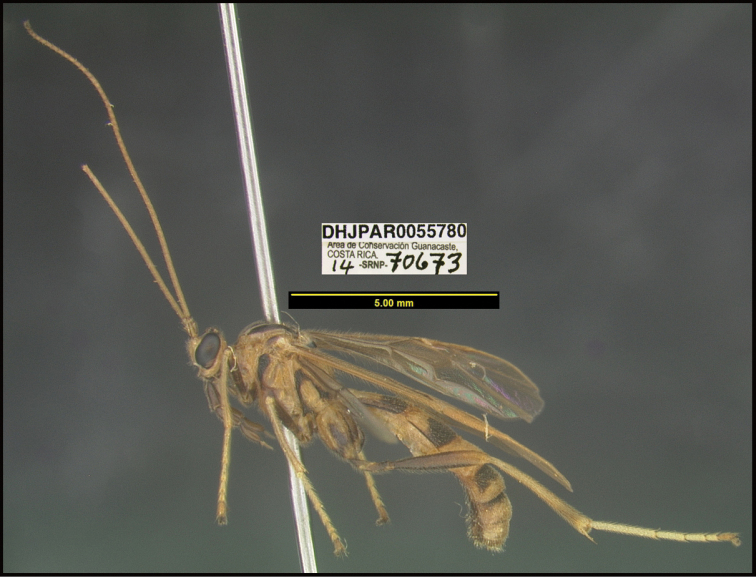
*Cystomastaxalexhausmanni*, holotype.

##### Cystomastax angelagonzalezae

Taxon classificationAnimaliaHymenopteraBraconidae

Sharkey sp. nov.

http://zoobank.org/01F95B51-87AE-48E9-989E-943FFFD5C28B

Figure 416

###### Diagnostics.

BOLD:AAA5370. Consensus barcode. TGTTTTATATTTTATTTTTGGTATTTGATCTGGRATATTAGGYTTATCTATAAGAATTATTATTCGAATAGAATTAATTAATCCTGGGGGTTTTATTAAAAATGATTATATTTATAATAGAATAGTTACTTCTCATGCTTTTATTATAATTTTTTTTATAGTTATGCCTGTAATAATTGGTGGATTTGGTAATTGATTAATTCCTTTAATGTTGGGGGCTCCTGATATGGCTTTCCCTCGAATAAATAATATAAGATTTTGATTATTAATTCCTTCTTTATTTATATTGTTAATAAGATCTTTTGTTAATGTTGGAGTTGGGACGGGKTGAACTATRTAYCCTCCTTTATCTTCTTTTTTAGGTCATGGAGGAATATCTGTAGATATATCTATTTTTTCTTTACATTTAGCAGGGTCTTCTTCTATTATAGGTGCTATTAATTTTATTTCTACAATTTTTAATATAAAATTATTTTCTTTAAAATTAGACCAATTAAATTTATTTATTTGGTCTGTTTTAATTACGGTAATTTTATTATTATTATCTTTACCAGTATTAGCTGGTGCTATTACTATGTTGTTAACTGATCGTAATTTAAATACTACTTTTTTTGATTTTYCTGGTGGGGGGGATCCTGTTTTATTTCAACATTTATTT.

###### Holotype ♂.

Guanacaste, Sector Pitilla, Medrano, 11.01602, -85.38053, 380 meters, collection date: 06/30/2012, wasp eclosion date: 07/16/2012. Depository: CNC.

***Host data*.***Hypena* zarabenaDHJ01 (Erebidae) feeding on *Ureralianoides* (Urticaceae).

***Host caterpillar and holotype wasp voucher codes*.** 12-SRNP-71556, DHJPAR0049684.

###### Paratype.

Host = *Hypena* Poole11: DHJPAR0029040. Depository: CNC.

###### Etymology.

*Cystomastaxangelagonzalezae* is named in honor of *Angela Gonzalez*’s long-appreciated contributions to publicity for ACG, GDFCF, and now, BioAlfa.

**Figure 416.**
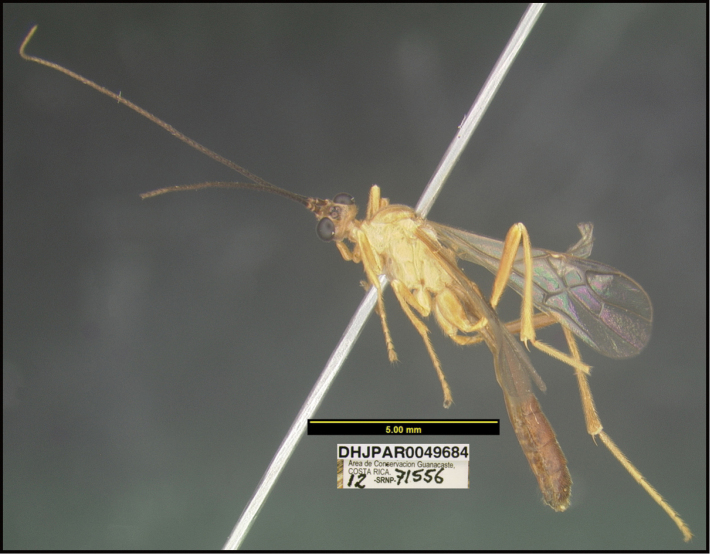
*Cystomastaxangelagonzalezae*, holotype.

##### Cystomastax ayaigarashiae

Taxon classificationAnimaliaHymenopteraBraconidae

Sharkey sp. nov.

http://zoobank.org/96BEDE1E-5A12-4127-AE86-659CFDA1CE04

Figure 417

###### Diagnostics.

BOLD:AAW1603. Consensus barcode. AATTTTATATTTTATTTTTGGTATTTGATCTGGAATATTAGGTTTATCTATAAGTATTATTATTCGAATAGAATTAATTAATCCTGGTGGATTAATTAAAAATGATAATATTTATAATAATATAGTTACTTCACATGCTTTTGTAATAATTTTTTTTATAGTTATACCTATTATAATTGGAGGATTTGGAAATTGATTAATTCCTTTAATATTAGGATCTCCAGATATAGCTTTTCCTCGTATAAATAATATAAGATTTTGATTATTAATTCCTTCTTTATTTTTATTATTAACTAGATCATTTATTAATGTTGGGGTTGGTACAGGTTGAACTATATATCCTCCTTTATCTTCTTTTTTAGGACATAGAGGAATATCTGTAGATATGGCTATTTTTTCTTTACATTTAGCTGGATCTTCTTCAATTATAGGTTCTATTAATTTTATTTCTACTATTTTTAATATAAAATTATTTTCTTTAAAATTAGATCAAATAAGTTTATTTATTTGATCTGTTTTGATTACAGTAATTTTATTATTATTATCTTTACCTGTATTAGCGGGAGCTATTACTATATTATTAACTGATCGTAATTTAAATACTACTTTTTTTGATTTTTCAGGGGGGGGAGATCCTATTTTATTTCAACATTTATTT.

###### Holotype ♀.

Guanacaste, Sector Pitilla, Sendero Laguna, 10.9888, -85.42336, 680 meters, caterpillar collection date: 06/19/2009, wasp eclosion date: 07/16/2009. Depository: CNC.

***Host data*.***Symphlebiaipsea* (Erebidae) feeding on *Clethralanata* (Clethraceae).

***Host caterpillar and holotype wasp voucher codes*.** 09-SRNP-32021, DHJPAR0035929.

###### Paratypes.

None.

###### Etymology.

*Cystomastaxayaigarashiae* is named in honor of Aya Igarashi’s long-appreciated contributions to publicity for ACG, GDFCF, and now, BioAlfa.

**Figure 417.**
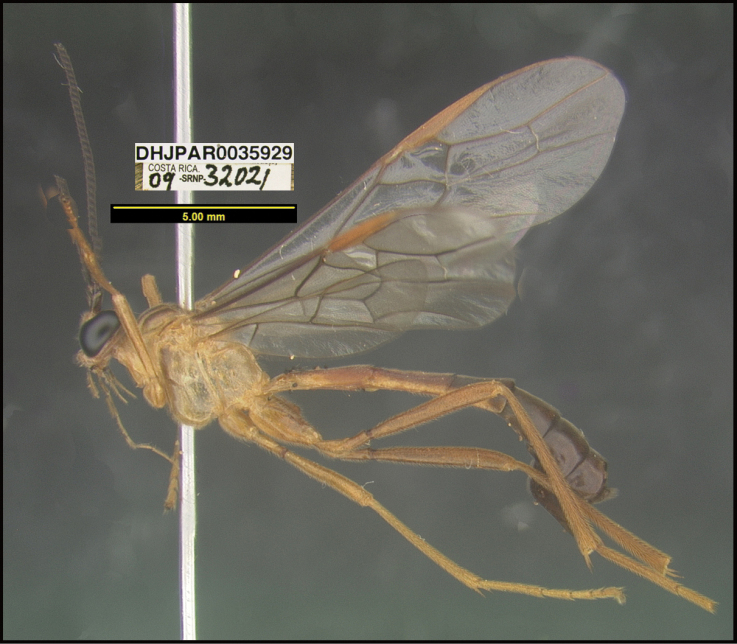
*Cystomastaxayaigarashiae*, holotype.

#### *Heterogamus* Wesmael, 1838

*Heterogamus* is nearly cosmopolitan, absent only from the Nearctic. There are 20 described species, only two of which are Neotropical, and neither is previously reported from Costa Rica. Hosts are unknown.

##### Heterogamus donstonei

Taxon classificationAnimaliaHymenopteraBraconidae

Sharkey sp. nov.

http://zoobank.org/7AFF801F-4AF0-4EDB-BE36-59DE5017197C

Figure 418

###### Diagnostics.

BOLD:AAH8697. Consensus barcode. AATTTTATATTTTATYTTTGGGATATGGGCTGGTATAATTGGTATRTCTATAAGATTAATTATTCGAATAGAGTTGGGKGTGTGTGGAAGRYTATTAATAAATGATCAAATTTATAATGGTATAGTRACTTTACAYGCTTTTATTATRATTTTTTTTATAGTTATACCTATTATRATTGGAGGRTTTGGTAATTGAYTAATTCCTTTAATATTAGGRTCTCCTGATATAGCTTTYCCWCGAATAAATAATATAAGTTTTTGATTATTAATTCCTTCTTTAATTTTATTAATTRYTAGGGGGGTGATAAATGTRGGTGTTGGYACGGTTGAACAGTTTAYCCYCCYTTATCTTCRTTAGTAGGGCATATAGGGRTRTCTGTAGATTTATCAATTTTTTCYTTACAYTTAGCTGGGGCTTCTTCAATTATAGGGGCTATTAATTTTATTACAACTATTTTTAATATGAATTTATTTAAAATTAAAATAGATCAGATGTCTTTATTTATTTGATCTGTTTTAATTACAGCATTTTTATTATTRTTATCTTTRCCTGTATTAGCAGGGGCTATTACTATATTATTAACGGATCGAAATTTAAAYACTACATTTTTTGATTTTTCAGGGGGGARGGGGATCCTATTTTTATTYCAACATTTATTTTGATTTTTTG.

###### Holotype ♀.

Guanacaste, Pailas Dos, PL12-9, 10.76, -85.3341, 809 meters, 17/vii/2014, forest, Malaise trap. Depository: CNC.

***Host data* .***None*.

***Holotype voucher code*.**BIOUG29390-H04.

###### Paratypes.

None.

###### Other material.

In BOLD there are specimens in the same BIN from Colombia (BCLDQ0397, BCLDQ0877) and Belize (BF002031, BMNHE897721). These were not examined.

###### Etymology.

*Heterogamusdonstonei* is named in honor of Don Stone’s (RIP) long-appreciated contributions to publicity for ACG and GDFCF.

**Figure 418.**
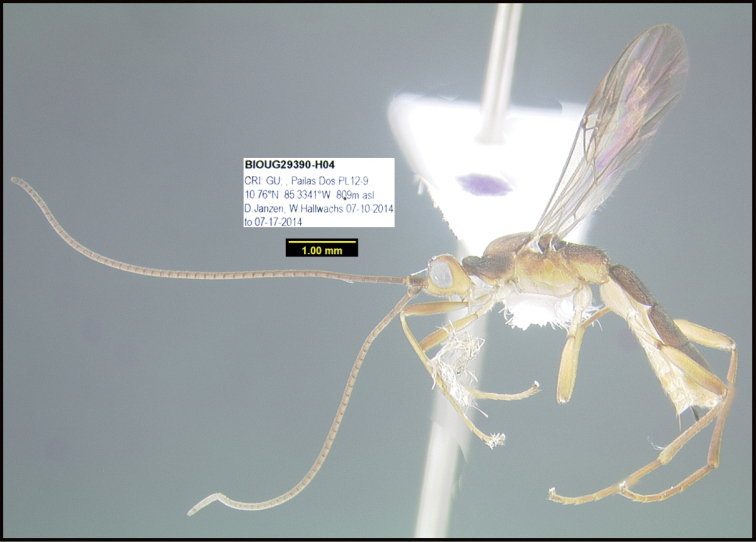
*Heterogamusdonstonei*, holotype.

#### *Pseudoyelicones* van Achterberg, Penteado-Dias & Quicke, 1997

*Pseudoyelicones* is restricted to the Neotropics with four of the five previously described species recorded from Costa Rica. Here we present the first host record for the genus, a pyralid caterpillar (Pyralidae).

##### Pseudoyelicones bernsweeneyi

Taxon classificationAnimaliaHymenopteraBraconidae

Sharkey sp. nov.

http://zoobank.org/5C79A772-A9F4-4B2D-9C87-889747A690B2

Figures 419 , 420

###### Diagnostics.

BOLD:AAL5801. Consensus barcode. AGTTTTATATTTTATTTTTGGTATGTGATCAGGAATTTTAGGAATATCATTAAGATTAATTATTCGAATAGAATTAGGTACTCCTGGGAAAATATTAGGAGATGATCAGTTATATAATAGTTTTGTTACTTTACATGCTTTTATTATAATTTTTTTTATAGTTATACCTATTATAATTGGTGGTTTCGGAAATTGATTAATTCCTTTAATATTAGGAGCTCCTGATATAGCTTTTCCTCGTATAAATAATATAAGATTTTGATTGTTAATTTCTTCTTTATTTATATTATTAATTAGAGGTATTATTAATGTAGGAACAGGAACTGGATGAACTATATATCCACCTTTATCTTCATTAATAGGTCATAGTGGAATATCTGTTGATTTATCAATTTTTTCTTTACATTTAGCTGGTGCTTCCTCTATTATAGGAGCTGTTAATTTTATTTCAACAATTTTTAATATAAAATTAACTAATATTGATATAGATAAAATTAGTTTGTTAATTTGATCAGTATTAATTACTGCTTTATTATTATTATTATCCTTACCTGTTTTGGCAGGTGCTATTACAATATTATTAACTGATCGTAACTTAAATACTACTTTTTTTGATTGTTCAGGTGGGGGCGATCCTGTTTTATTKCAACATTTRTTT. Similar to *P.limonensis* to which it keys in Buntika and Quicke (2004). It differs in many respects, but the easiest to observe is that the scape is yellow in *P.bernsweeneyi* and brown in *P.limonensis*.

###### Holotype ♀.

Alajuela, Sector Rincon Rain Forest, Finca Esmeralda, 10.93548, -85.25314, 123 meters, caterpillar collection date: 09/14/2011, wasp eclosion date: 10/18/2011. Depository: CNC.

***Host data*.***Chloropaschiamennusalis* (Pyralidae) feeding on *Vismiabaccifera* (Hypericaceae). This is the first host record for the genus. The image of the mummified pupa of the host, *Chloropaschiamennusalis* (Fig. 420), shows the exit hole at the caudal end.

***Host caterpillar and holotype wasp voucher codes*.** 11-SRNP-75949, DHJPAR0045816.

###### Paratypes.

DHJPAR0035971, DHJPAR0045814, DHJPAR0063815. Depository: CNC.

###### Etymology.

*Pseudoyeliconesbernsweeneyi* is named in honor of Bern Sweeney’s contributions to publicity for ACG, GDFCF, and BioAlfa.

**Figure 419.**
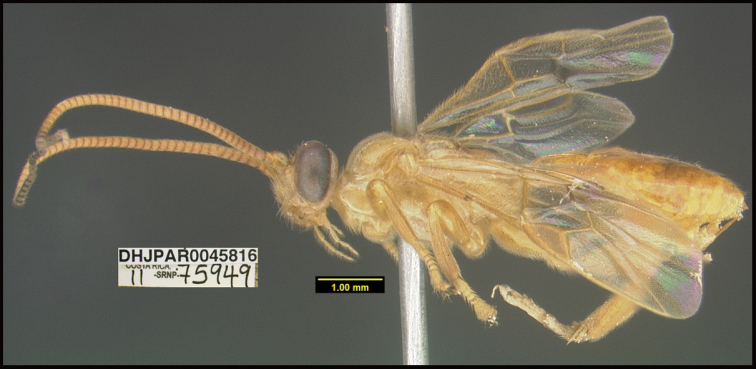
*Pseudoyeliconesbernsweeneyi*, holotype.

**Figure 420.**
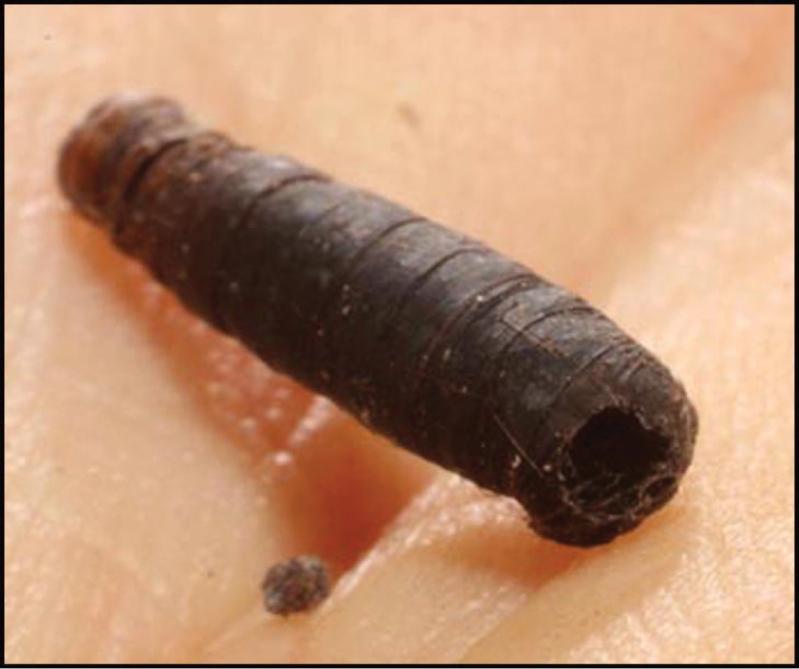
Mummified remains of the pupa of *Chloropaschiamennusalis* after emergence of the parasitoid.

#### *Stiropius* Cameron, 1911

*Stiropius* is restricted to the New World with most species occurring in the Neotropics. No species were previously recorded from Costa Rica. The genus has previously been recorded on larval Lyonetiidae and Gracillariidae, and here we present the first record from Crambidae. There are no revisions of the Neotropical species.

##### Stiropius bencrairi

Taxon classificationAnimaliaHymenopteraBraconidae

Sharkey sp. nov.

http://zoobank.org/8408A421-848F-4A29-852F-55F859B4F5CE

Figure 421

###### Diagnostics.

BOLD:ACB2702. Consensus barcode. TGTATTGTATTTTTTATTTGGTATATGATCAGGAGTTTTAGGTTTATCGATAAGAATAATTATTCGATTTGAATTAAGAATTCCGGGATCTTTTTTAGGTAATGATCAAATTTATAATAGAATTGTTACATCACATGCTTTAGTAATAATTTTTTTTATAGTTATACCTGTAATAATTGGAGGGTTTGGAAATTGGTTAATTCCATTAATATTAGGAGCTCCTGATATAGCTTTTCCTCGAATAAATAATATGAGATTTTGATTATTGTTTCCTGCTATTATATTATTATTATTAAGTTCTTTTGTTAGGGTTGGAAGAGGAACAGGATGAACAATATATCCTCCATTATCTTCTTTAGTTGGTCATGGGGGAGTTTCAGTGGATTTGTCAATTTTTTCTTTACATTTAGCAGGAGTATCTTCTATTATAGGTGCTATTAATTTTATTACAACAGTTTTTAATATAAATTTATTTTATATTAAAATAGATCAGGTTATATTATTAGTTTGATCAGTATTAATTACTGCTTTTTTATTATTATTATCTTTACCTGTATTAGCAGGTGCTATTACAATATTGTTGTTTGATCGAAATATTAATACAACATTTTTTGATTTTTCTGGGGGAGGGGATCCTATTTTATTTCAACATTTATTT.

###### Holotype ♂.

Alajuela, Sector Rincon Rain Forest, San Lucas, 10.91847, -85.30338, 320 meters, caterpillar collection date: 05/01/2012, wasp eclosion date: 05/19/2012. Depository: CNC.

***Host data*.** graciJanzen01 Janzen6864 (Gracillariidae) feeding on *Protiumcostaricense* (Burseraceae).

***Host caterpillar and holotype wasp voucher codes*.** 12-SRNP-41777, DHJPAR0049332.

###### Paratypes.

None.

###### Etymology.

The specific epithet acknowledges the contributions of Ben Crair to publicity for ACG, GDFCF, and BioAlfa.

**Figure 421.**
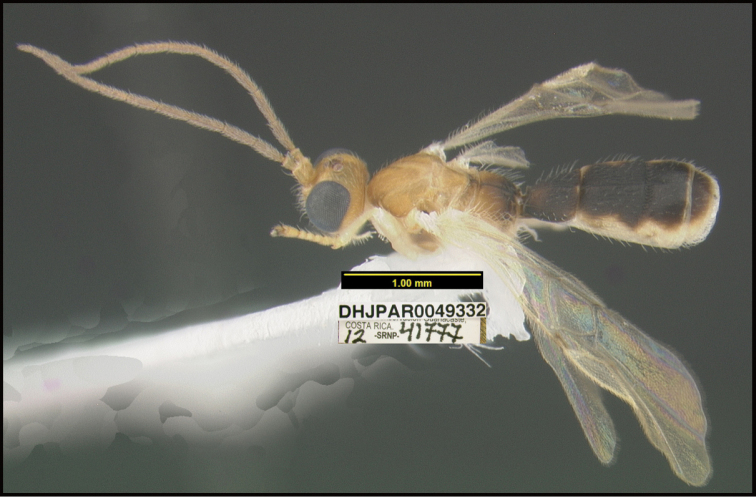
*Stiropiusbencrairi*, holotype.

##### Stiropius berndkerni

Taxon classificationAnimaliaHymenopteraBraconidae

Sharkey sp. nov.

http://zoobank.org/97CFD2F3-6CA8-4A42-93D9-E749D8646171

Figure 422

###### Diagnostics.

BOLD:ABV9879. Consensus barcode. TTTTTATATTTTTTTTTTGGTATATGATCAGGTATTTTGGGTATATCTATAAGTATAATTATTCGTTTTGAATTAAGTATTTCAGGTTCTTTTTTGGGTAATGAACAAATTTATAATAGAATTGTTACTTCTCATGCTTTAATTATAATTTTTTTTATAGTTATACCTGTAATGATTGGAGGATTTGGTAATTGGTTGATTCCTTTAATATTAGGGGCTCCTGATATAGCTTTCCCTCGTATAAATAATATAAGGTTTTGATTATTAATTCCTTCTATAATAATATTATTAATAACTTTTTTTGTTAATGTGGGGGTAGGCACTGGGTGAACTATTTATCCTCCTTTGTCTTCTTTATTAGGTCATAGAGGTATTTCTATAGATTTTGCTATTTTTTCTTTGCATTTAGCAGGAGTTTCTTCAATTATAGGTGCTATTAATTTTATTACAACAATTTTTAATATAAATTTATTTCATATAAAAATAGATCAGATTGTTTTATTTATTTGATCTGTTTTGATTACTGCTTTTTTATTATTATTATCTTTACCTGTATTAGCTGGGGCTATTACAATATTGTTATTTGATCGTAATATTAATACTACTTTTTTTGATTTTC.

###### Holotype ♀.

Guanacaste, Sector Cacao, Sendero Cima, 10.93328, -85.45729, 1460 meters, caterpillar collection date: 06/i/2009, Malaise trap. Depository: CNC.

***Host data*.** None.

***Host caterpillar and holotype wasp voucher codes*.**DHJPAR0045983.

###### Paratypes.

None.

###### Etymology.

*Stiropiusberndkerni* is a patronym for the late Bernd Kern in appreciation of his many years of contributions to publicity for ACG and GDFCF.

**Figure 422.**
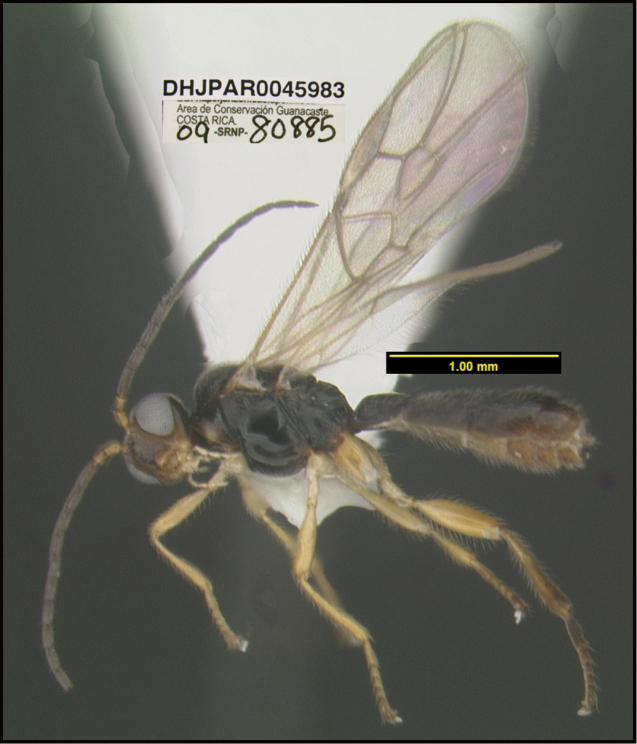
*Stiropiusberndkerni*, holotype.

##### Stiropius edgargutierrezi

Taxon classificationAnimaliaHymenopteraBraconidae

Sharkey sp. nov.

http://zoobank.org/198A21F9-666C-4089-964F-72194C2F3078

Figure 423

###### Diagnostics.

BOLD:ACJ1403. Consensus barcode. AATTTTATATTTTATTTTTGGTTTATGGTCTGGTATGTTAGGATTATCAATAAGTTTAATTATTCGGTTTGAATTGAGGTGTCCTGGGTCATTTTTGGGGAATGATCAAATTTATAATAGAATTGTTACTGCACATGCTTTAATTATAATTTTTTTTATAGTTATACCTGTTATAATTGGGGGATTTGGAAATTGATTAATTCCTTTAATGTTAGGGGCACCTGATATAGCTTTTCCACGTATAAATAATATAAGTTTTTGATTATTAATTCCTTCATTATTGTTGTTAATATTTAGATCTTTAGTTAATGTTGGGGTGGGTACTGGGTGAACAATTTATCCTCCATTATCTTCTTTATTAGGTCATGGAGGGATTTCGATAGATTTAGCTATTTTTTCTTTACATTTAGCGGGGACTTCTTCAATTATAGGGGCAATTAATTTTATTACTACTATTTTTAATATAAAATTATTTTATATAAAGTTAGATCAGTTAACTTTATTTATTTGATCAGTTTTAATTACAGCTTTTTTATTACTTTTATCTTTGCCAGTTTTAGCTGGGGCTATTACTATATTATTATTTGATCGTAATATTAATACTACATTTTTTGA.

###### Holotype ♀.

Guanacaste, Sector Santa Rosa, Bosque San Emilio, 10.8438, -85.6138, 300 meters, dry forest, 7/v/2012, forest, Malaise trap. Depository: CNC.

***Host data*.** None.

***Holotype voucher code*.**BIOUG07918-G09.

###### Paratypes.

BIOUG07457-F12, BIOUG10016-H10.

###### Etymology.

*Stiropiusedgargutierrezi* is named in honor of the contributions of Edgar Gutierrez toward increasing publicity for ACG, GDFCF, and BioAlfa.

**Figure 423.**
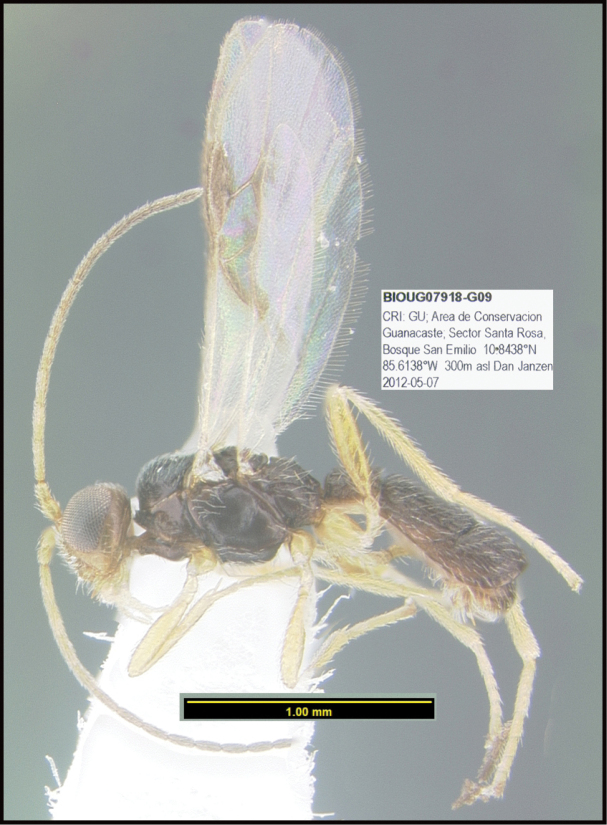
*Stiropiusedgargutierrezi*, holotype

##### Stiropius edwilsoni

Taxon classificationAnimaliaHymenopteraBraconidae

Sharkey sp. nov.

http://zoobank.org/4DB3EAD3-08D8-4F68-869D-79CC34837434

Figure 424

###### Diagnostics.

BOLD:ACG5440. Consensus barcode. TATTTTATATTTTTTTTTTGGTATTTGATCTGGTGTTTTAGGCATATCTATAAGGTTAATTATTCGTTTTGAGTTAGGGGTTCCTGGATCTTTTTTAGGTAATGATCAGATTTATAATAGAATTGTTACTGCTCATGCTTTGGTGATAATTTTTTTTATAGTTATACCAATTATAATTGGGGGGTTTGGAAATTGATTAATTCCTTTAATATTGGGGTGTCCTGATATAGCTTTCCCTCGTATAAATAATATGAGGTTTTGATTGTTATTRCCTTCAATTTTTTTGTTATTAATTAGTTCAATTGTTAATATTGGTGTTGGAACTGGGTGAACTATTTATCCTCCTTTATCTTCTTTAATAGGTCATAGAGGTGTTTCTTTAGATTTAGCAATTTTTTCTTTACACTTAGCTGGGGCTTCTTCAATTATAGGTGCTATTAATTTTATTACTACTATTTTTAATATGAATTTATTTTCAATAAAAATAGATCAAATTATATTAATAGTTTGATCTGTGTTAATTACTGCATTTTTATTATTATTATCTTTACCTGTTTTAGCGGGGGCAATTACTATATTATTATTTGATCGTAATATTAATACAACATTTTTTGATTTTTCTGGAGGAGGGGACCCAATTTTATTTCAACATTTGTTT.

###### Holotype ♀.

Guanacaste, Sector Santa Rosa, Bosque San Emilio, 10.8438, -85.6138, 300 meters, dry forest, 24/xii/2012, forest, Malaise trap. Depository: CNC.

***Host data* .***None*.

***Holotype voucher code*.**BIOUG09739-D10.

###### Paratypes.

BIOUG05883-A10, BIOUG09739-F06, BIOUG18290-E10.

###### Etymology.

The species name is a patronym for Ed Wilson in recognition of his contributions to publicity for ACG, GDFCF, and BioAlfa.

**Figure 424.**
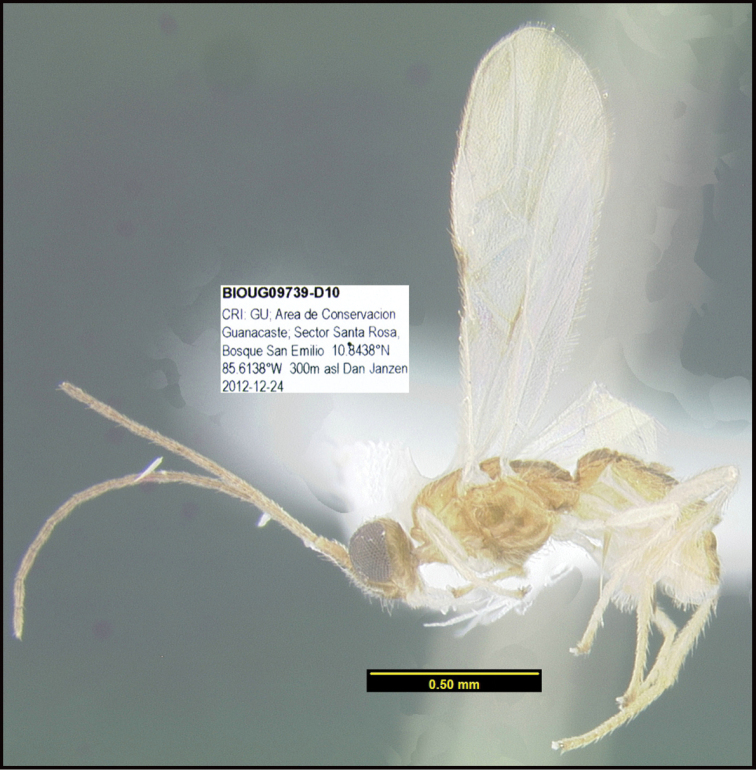
*Stiropiusedwilsoni*, holotype.

##### Stiropius ehakernae

Taxon classificationAnimaliaHymenopteraBraconidae

Sharkey sp. nov.

http://zoobank.org/41426EC7-02A4-4F22-A4F1-643FECA94E96

Figure 425

###### Diagnostics.

BOLD:ACL3545. Consensus barcode. TTTATATTTTTTTTTTGGAATATGATCTGGTATTTTGGGCTTATCTATAAGATTGATTATTCGTTTTGAAYTAGGTGTTCCAGGTTCTTTTTTGGGTAATGATCAAATTTATAATAGAATTGTTACTGCTCATGCTTTAATTATAATTTTTTTTATGGTTATACCTGTTATAATTGGGGGGTTTGGTAATTGGTTAATTCCTTTAATATTAGGGGCTCCAGATATAGCTTTTCCTCGGATAAATAATATAAGATTTTGATTATTGATTCCTTCWATTTTTTTATTATTGATTAGGTCTTTGATTAATATTGGGGTTGGTACTGGATGAACTATTTATCCTCCCTTATCATCATTAATAGGTCATAGGGGAATATCAATAGATATAGCTATTTTTTCTTTGCATTTGGCTGGTGCTTCTTCTATTATAGGTGCAATTAATTTTATTACTACTATTTTTAATATAAATTTATTTTATATAAAAATAGATCAAATTACATTATTAATTTGATCTATTTTAATTACTGCTTTTTTATTATTATTATCTTTACCTGTTTTAGCTGGGGCTATTACAATATTATTATTTGATCGTAATGTTAATACTACTTTTTTTGATTTTTCTGGGGGGGGGGA.

###### Holotype ♀.

Guanacaste, Pailas Dos, PL12-4, 10.7629, -85.3341, 825 meters, 9/x/2014, forest, Malaise trap. Depository: CNC.

***Host data*.** None.

***Holotype voucher code*.**BIOUG29249-F01.

###### Paratype.

BIOUG09826-B01. Depository: CNC.

###### Etymology.

*Stiropiusehakernae* is named in recognition of Eha Kern’s long-appreciated contributions to publicity for ACG, GDFCF, and BioAlfa.

**Figure 425.**
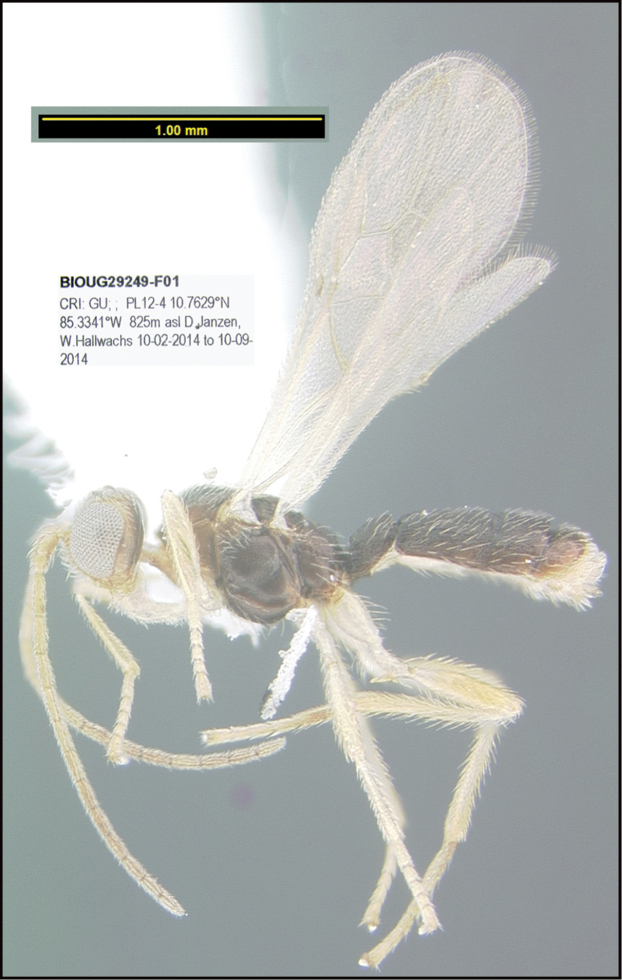
*Stiropiusehakernae*, holotype.

#### *Triraphis* Ruthe, 1855

*Triraphis* occurs in all realms except the Australian and Antarctic. In the New World, most species are Neotropical, where several thousand remain to be described. Previous host records are Lepidoptera, including Limacodidae, Zygaenidae, Megalopygidae, Lycaenidae, and Riodinidae (van Achterberg 1991; Valerio and Shaw 2015). These are all slug-shaped (actually copepod- or sowbug-shaped) caterpillars, as are the hosts recorded here. Valerio and Shaw (2015) described 13 species from Costa Rica, only one of which, *T.defectus*, was collected by us. There are no revisions of the Neotropical species.

##### Triraphis billfreelandi

Taxon classificationAnimaliaHymenopteraBraconidae

Sharkey sp. nov.

http://zoobank.org/CB9BC55D-9E67-471F-9222-B9149AEA03D5

Figure 426

###### Diagnostics.

BOLD:AAA5375. Consensus barcode. TGTTTTATATTTTTTATTTGGTATTTGAGCTGGTATAGTTGGTTTATCCATAAGTTTAATTATTCGTTTAGAATTAAGTATACCCGGTAGATTATTGGGTAATGACCAAATTTATAATGGTATAGTAACTGCCCATGCTTTTATTATAATTTTTTTTATAGTTATGCCTATTATAATTGGTGGTTTTGGAAATTGATTGATTCCCTTAATATTGGGGGCTCCTGATATAGCTTTCCCTCGTATAAATAATATGAGGTTTTGATTATTAATTCCCTCATTGACATTATTAATTTTAAGAGCTGTAGTTAATGTTGGGGTAGGCACTGGGTGAACTTTGTACCCTCCTTTATCTTCTTTAGTTGGTCATAGGGGTATATCTGTAGATATGGCTATTTTTTCTTTACATTTAGCTGGTGCTTCTTCTATTATAGGGGTTGTTAATTTTATTTCTACTATTTTTAATATAAAGTTAGTATCAATTAATTTAGATCAAATTAATTTATTTGTTTGATCAGTATTAATTACAGCTGTCTTATTATTATTATCTTTACCAGTGTTAGCTGGTGCAATTACTATATTATTGACAGATCGTAATTTAAATACAACATTTTTTGATTTTTCTGGTGGGGGTGACCCTATTTTATTCCAACATTTATTT.

###### Holotype ♀.

Guanacaste, Sector Pitilla, Sendero Mismo, 10.98758, -85.41967, 680 meters, caterpillar collection date: 18/x/2012, wasp eclosion date: 05/xi/2012. Depository: CNC.

***Host data*.***Parasasandrae* (Limacodidae) feeding on *Calatolacostaricensis* (Icacinaceae).

***Host caterpillar and holotype wasp voucher codes*.** 12-SRNP-31534, DHJPAR0050910.

###### Paratypes.

DHJPAR0029047, DHJPAR0040078. Depository: CNC.

###### Etymology.

The species name honors the contributions of Bill Freeland toward increasing publicity for ACG, GDFCF, and BioAlfa.

**Figure 426.**
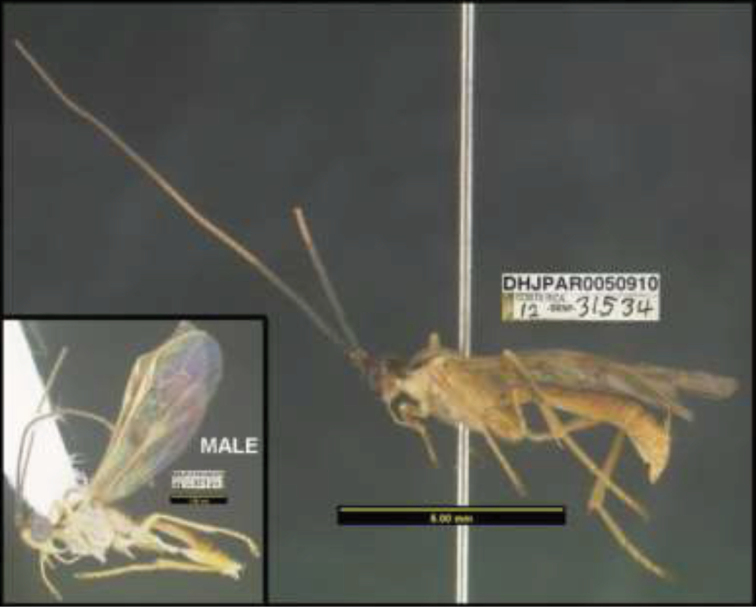
*Triraphisbillfreelandi*, holotype.

##### Triraphis billmclarneyi

Taxon classificationAnimaliaHymenopteraBraconidae

Sharkey sp. nov.

http://zoobank.org/2AAB7A2C-71E0-4EBB-98C0-56E0FBCDFED9

Figure 427

###### Diagnostics.

BOLD:AAA7065. Consensus barcode. TGTTTTATATTTTTTATTTGGAATTTGAGCRGGTATAGTTGGATTATCTATAAGATTAATTATTCGTTTAGAATTAAGTATACCGGGGAGATTATTAGGGAATGAYCAAATTTATAAYGGTATAGTGACTGCYCATGCTTTTATTATAATTTTTTTTATAGTTATACCTATTATAATTGGYGGTTTTGGAAATTGATTAATTCCTTTAATATTRGGGGCYCCTGATATGGCTTTCCCTCGTATAAATAATATGAGTTTTTGGTTATTAATTCCTTCATTGACTTTATTAATTTTAAGGGCTGTAGTTAATGTTGGAGTRGGTACTGGATGAACTTTATACCCACCTTTATCTTCTTTAGTTGGTCATGGGGGTATATCTGTAGATATAGCTATTTTTTCTTTACATTTAGCTGGAGCATCTTCTATTATAGGGGTTGTTAATTTTATTTCTACTATTTTTAATATAAAATTAGTATCAATTAATTTAGATCAAATTAATTTATTTGTTTGATCAGTATTAATTACAGCTATTTTATTATTATTGTCTTTACCTGTRTTAGCTGGCGCAATTACTATATTATTAACAGATCGTAATTTAAATACAACATTTTTTGATTTTTCTGGGGGGGGKGATCCTATTTTATTTCAACATTTATTT.

###### Holotype ♂.

Guanacaste, Sector Pitilla, Estación Pitilla, 10.98931, -85.42581, 675 meters, caterpillar collection date: 20/xi/2008, wasp eclosion date: 01/xii/2008. Depository: CNC.

***Host data*.** zygjanzen01 Janzen23 (Zygaenidae) feeding on *Doliocarpusmultiflorus* (Dilleniaceae).

***Host caterpillar and holotype wasp voucher codes*.** 08-SRNP-32864, DHJPAR0030596.

###### Paratypes.

DHJPAR0030594 DHJPAR0030595 DHJPAR0030597 DHJPAR0030598 DHJPAR0036310 DHJPAR0036311 DHJPAR0030592 DHJPAR0037873. Depository: CNC.

###### Other material.

A specimen (BIOUG24424-E06) in the same BIN (BIOUG24424-E06) was collected in Misiones, Argentina and is deposited in Museo Argentino de Ciencias Naturales. It was not examined, but the image in BOLD is consistent with the Costa Rican specimens.

###### Etymology.

*Triraphisbillmclarneyi* is named in honor of Bill McLarney’s long-appreciated contributions to publicity for ACG, GDFCF, and BioAlfa.

**Figure 427.**
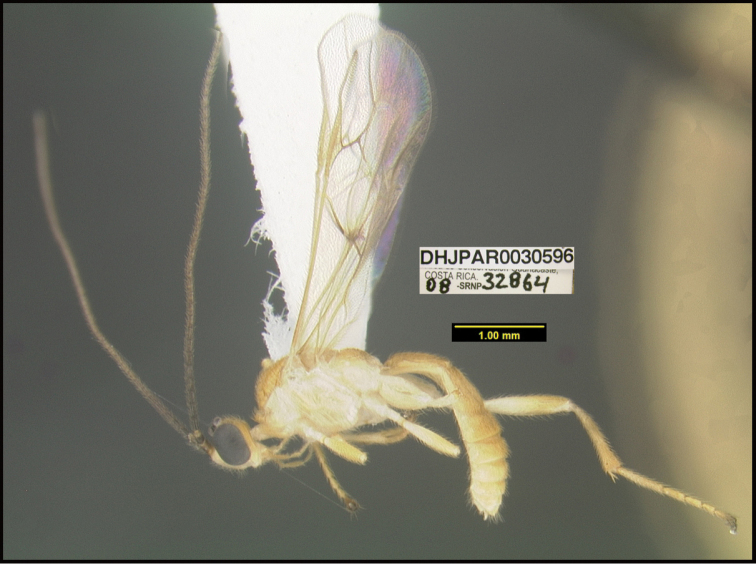
*Triraphisbillmclarneyi*, holotype.

##### Triraphis billripplei

Taxon classificationAnimaliaHymenopteraBraconidae

Sharkey sp. nov.

http://zoobank.org/9B28CCD8-18A4-4C53-9A30-F126EC51963C

Figure 428

###### Diagnostics.

BOLD:AAB1658. Consensus barcode. AGTATTATATTTTTTATTTGGAATTTGAGCAGGTATGGTTGGATTATCTATAAGTTTAATTATTCGTTTAGAATTAAGAATACCTGGAAGTTTATTAGGTAATGATCAAATTTATAATGGTATAGTTACTGCTCACGCTTTTATTATAATTTTTTTTATAGTTATACCTATTATAATTGGTGGGTTTGGTAATTGATTAATTCCATTAATATTGGGTGCTCCTGATATAGCTTTTCCTCGTATAAATAATATAAGATTTTGGTTATTAATTCCTTCATTAACATTATTAATTTTAAGGGCTGTTGTTAATGYTGGAGTAGGGACTGGGTGAACATTATATCCTCCCTTATCTTCTTTAGTTGGTCATGGGGGGATATCTGTAGATATGGCTATTTTTTCTTTACATTTAGCTGGTGCTTCTTCAATTATAGGTGTTGTTAATTTTCTTTCTACTATTTTTAATATAAAATTAGTATCTATTAATTTAGATCAAATTAATTTATTTGTCTGATCAGTATTAATTACTGCTGTTTTATTATTATTATCTTTACCAGTGTTAGCTGGTGCTATTACTATATTATTGACAGATCGTAATTTAAATACAACTTTTTTTGATTTTTCTGGTGGAGGGGACCCCATTTTATTTCAACATTTATTT.

###### Holotype ♀.

Guanacaste, Sector Horizontes, Vado Esperanza, 10.78938, -85.55098, 85 meters, caterpillar collection date: 15/vi/2009, wasp eclosion date: 16/vi/2009. Depository: CNC.

***Host data*.***Norape* Janzend03 (Megalopygidae) feeding on *Mimosapigra* (Fabaceae).

***Host caterpillar and holotype wasp voucher codes*.** 09-SRNP-13855, DHJPAR0036305.

###### Paratypes.

DHJPAR0021201, DHJPAR0028329, DHJPAR0028330. Depository: CNC.

###### Etymology.

*Triraphisbillripplei* is named in acknowledgment of Bill Ripple’s long-appreciated contributions to publicity for ACG, GDFCF, and BioAlfa.

**Figure 428.**
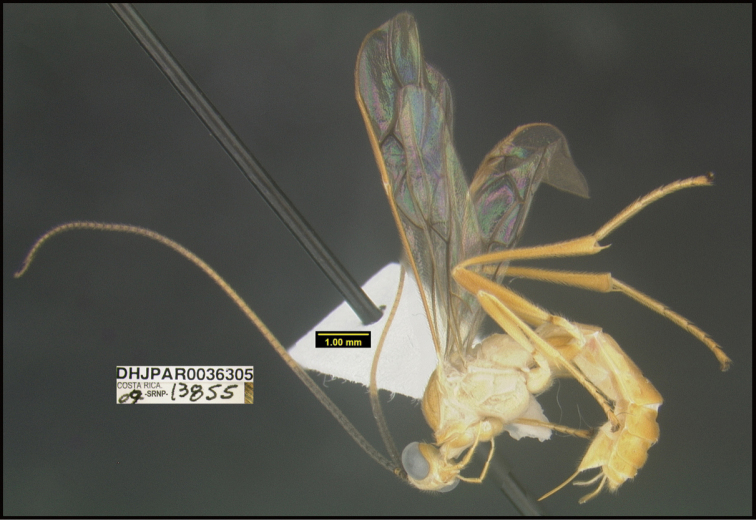
*Triraphisbillripplei*, holotype.

##### Triraphis bobandersoni

Taxon classificationAnimaliaHymenopteraBraconidae

Sharkey sp. nov.

http://zoobank.org/E50E8FC2-9480-498F-874D-77D3D3C5B703

Figure 429

###### Diagnostics.

BOLD:AAB8975. Consensus barcode. TGTGTTATATTTTTTATTTGGGATTTGATCAGGTATAGTTGGTTTATCTATAAGTTTAATTATTCGTTTAGAATTAAGAATACCCGGGAGTTTATTGGGTAATGATCAAATTTATAATGGTATAGTTACTGCTCATGCCTTTATTATAATTTTTTTTATGGTTATACCTATTATAATTGGTGGATTTGGAAATTGATTAATTCCTTTAATATTAGGGGCTCCTGATATAGCTTTCCCACGTATAAATAACATAAGATTTTGATTATTAATTCCTTCATTAACATTATTAATTTTAAGGGCTGTAGTTAATGTTGGGGTAGGTACTGGATGAACTTTATATCCTCCTTTATCTTCTTTAGTTGGGCACGGYGGGATATCCGTTGATATAGCTATTTTTTCTTTACATTTGGCTGGGGCTTCTTCAATTATAGGTGTTGTTAATTTTATTTCTACTATTTTTAATATAAAATTAGTGTCAATTAATTTAGATCAAATTAATTTATTTGTTTGATCAGTATTAATTACTGCTGTTTTATTATTATTATCTTTACCAGTATTGGCAGGGGCTATTACTATATTGTTAACAGATCGTAATTTAAATACAACATTTTTTGATTTTTCTGGTGGTGGTGATCCTATTTTATTTCAACATTTATTT.

###### Holotype ♀.

Guanacaste, Sector Pitilla, Sendero Memos, 10.98171 -85.42785, 740 meters, caterpillar collection date: 18/iii/2013, wasp eclosion date: 03/iv/2013. Depository: CNC.

***Host data*.***Epiperolavaferella* (Limacodidae) feeding on *Cyatheamultiflora* (Cyatheaceae).

***Host caterpillar and holotype wasp voucher codes*.** 13-SRNP-30462, DHJPAR0051666.

###### Paratypes.

DHJPAR0017286, DHJPAR0023518, DHJPAR0023519, DHJPAR0023527, DHJPAR0023532, DHJPAR0023533, DHJPAR0023689, DHJPAR0023690, DHJPAR0023694, DHJPAR0023698, DHJPAR0023702, DHJPAR0023704, DHJPAR0023716, DHJPAR0023722, DHJPAR0023701, DHJPAR0030619, DHJPAR0038384, DHJPAR0038385, DHJPAR0038386, DHJPAR0037964, DHJPAR0037969, DHJPAR0037970, DHJPAR0037972, DHJPAR0037973, DHJPAR0051315. Depository: CNC.

###### Etymology.

The specific epithet acknowledges the contributions of Bob Anderson toward increasing publicity for ACG, GDFCF, and BioAlfa.

**Figure 429.**
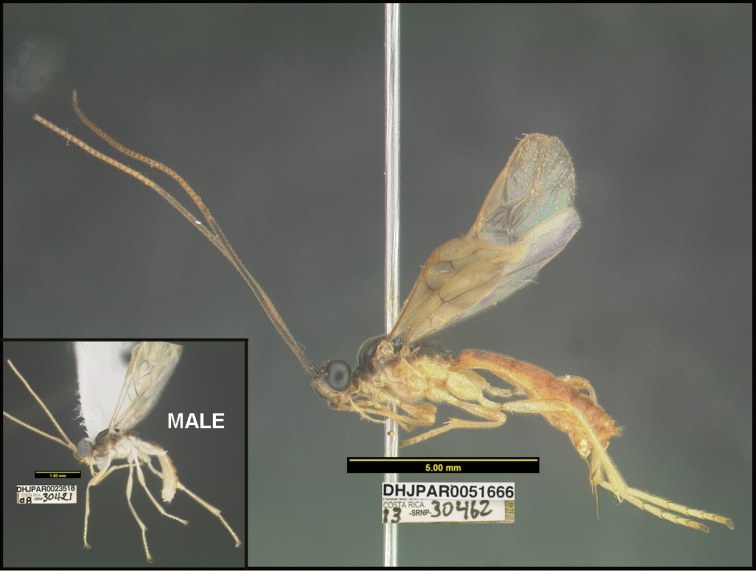
*Triraphisbobandersoni*, holotype female and paratype male.

##### Triraphis bobrobbinsi

Taxon classificationAnimaliaHymenopteraBraconidae

Sharkey sp. nov.

http://zoobank.org/40F75DD6-F184-45BD-9BCF-A3A4C7793127

Figure 430

###### Diagnostics.

BOLD:AAD2033. Consensus barcode. TGTTTTATATTTTTTATTTGGAATTTGAGCARGRATATTRGGTTTATCAATGAGATTAATTATTCGATTAGAGTTAAGTATACCAGGAAGTTTATTAGGTAATGATCAAATTTATAATGGTATAGTAACTGCTCATGCTTTTATTATAATTTTTTTTATAGTTATGCCTATTATAATTGGGGGATTTGGTAATTGGTTGATTCCTTTAATATTAGGGGCTCCTGATATAGCTTTTCCTCGGATAAATAATATAAGTTTTTGATTATTAATTCCTTCATTAACTTTGTTAATTTTAAGTGCTGTAGTTAATGTTGGGGTTGGTACTGGTTGAACTATATATCCTCCTTTATCTTCTTTAGTTGGTCATGGGGGTATATCTGTTGATATAGCTATTTTTTCTTTACATTTGGCGGGTGCATCATCTATTATAGGTGTAGTTAATTTTATTTCTACTATTTTTAATATAAAATTAATTTCTATTAGATTAGATCAAATTAATTTATTTGTTTGATCAGTTTTAATTACAGCTGTATTATTATTATTATCTTTACCTGTTTTAGCTGGTGCAATTACTATATTACTTACTGATCGTAATTTAAATACGACTTTTTTTGATTTTTCTGGTGGTGGGGATCCTATTTTATTTCAACATTTATTT.

###### Holotype ♀.

Alajuela, Sector Rincon Rain Forest, Estación Llanura, 10.93332, -85.25331, 135 meters, caterpillar collection date: 25/v/2013, wasp eclosion date: 07/vi/2013. Depository: CNC.

***Host data*.***Thisbeirenea* (Riodinidae) feeding on *Crotonmorifolius* (Euphorbiaceae).

***Host caterpillar and holotype wasp voucher codes*.** 13-SRNP-76430, DHJPAR0052094.

###### Paratypes.

DHJPAR0021182, DHJPAR0021183, DHJPAR0021185, DHJPAR0021187, DHJPAR0021190, DHJPAR0021192, DHJPAR0021193, DHJPAR0021195, DHJPAR0040352, DHJPAR0040353, DHJPAR0040354, DHJPAR0040355, DHJPAR0040356, DHJPAR0040357, DHJPAR0041144, DHJPAR0048705, DHJPAR0056005. Depository: CNC.

###### Etymology.

The species name is a patronym for Bob Robbins in recognition of his contribution to the ACG inventory throughout the identification of Lycaenidae.

**Figure 430.**
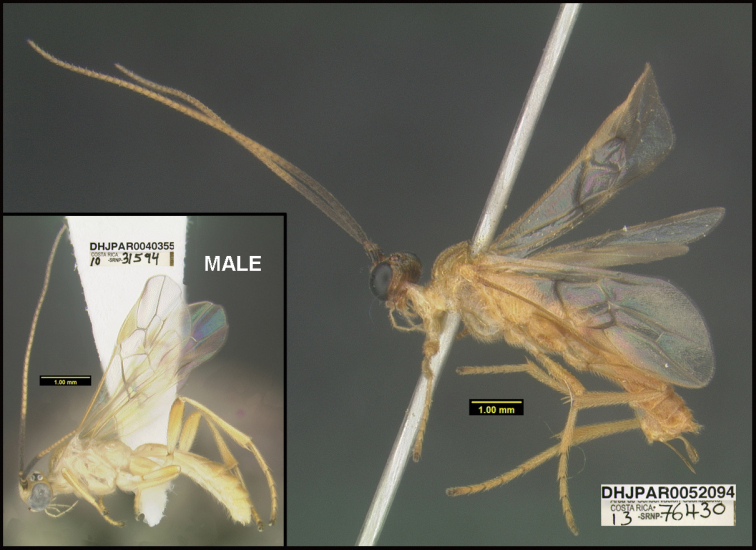
*Triraphisbobrobbinsi*, holotype female and paratype male.

##### Triraphis bradzlotnicki

Taxon classificationAnimaliaHymenopteraBraconidae

Sharkey sp. nov.

http://zoobank.org/39055987-2C2E-4ACD-9513-A286188A4E1C

Figure 431

###### Diagnostics.

BOLD:AAD7334. Consensus barcode. TGTATTATATTTTTTATTTGGTATTTGGGCTGGTATGGTTGGTTTATCTATAAGATTAATTATTCGATTAGAATTAAGAATGCCAGGTAGTTTATTAGGTAATGATCAAATTTATAATGGYATAGTTACTGCTCATGCTTTTATTATAATTTTTTTTATAGTTATACCTATTATAATTGGGGGGTTTGGAAATTGGTTAATTCCATTAATGTTAGGGGCYCCTGATATAGCTTTCCCTCGTATAAATAATATAAGATTTTGATTATTAATTCCTTCATTAACTTTATTAATTTTAAGTGCTGTAGTTAATGTRGGAGTTGGAACAGGATGAACTATRTATCCTCCATTATCTTCTTTAGTTGGTCATGGGGGAATATCGGTTGATATAGCTATTTTTTCTTTACATTTGGCTGGTGCYTCATCTATTATAGGTGTTGTTAATTTTATTTCTACTATTTTTAATATAAAATTGGTATCTATTAATTTGGATCAAATTAATTTATTTGTTTGGTCTGTTTTAATTACTGCTGTTTTATTATTATTGTCTTTACCGGTTTTGGCTGGTGCTATTACTATATTATTAACTGATCGTAATTTAAATACAACATTTTTTGATTTTTCAGGTGGGGGAGATCCTATTTTATTTCAACATTTATTT.

###### Holotype ♀.

Guanacaste, Sector Pitilla, Sendero Naciente, 10.98705, -85.42816, 700 meters, caterpillar collection date: 11/iv/2009, wasp eclosion date: 22/iv/2009. Depository: CNC.

***Host data*.***Podaliaorsilocha* (Megalopygidae) feeding on *Chimarrhisparviflora* (Rubiaceae).

***Host caterpillar and holotype wasp voucher codes*.** 09-SRNP-31273, DHJPAR0035258.

###### Paratypes.

Hosts = *Podaliaorsilocha* and *Venadicodiacaneti* (Limacodidae). DHJPAR0035536, DHJPAR0035545, DHJPAR0035546, DHJPAR0035251. DHJPAR0045688, DHJPAR0062195. Depository: CNC.

###### Other material.

Four specimens in the same BIN were collected in Jalisco, Mexico (BIOUG20632-B06, BIOUG20863-E05, BIOUG20444-H09, BIOUG20584-D04). They were not examined, but the one specimen that has an image on BOLD is somewhat lighter in coloration than the Costa Rican specimens.

###### Etymology.

*Triraphisbradzlotnicki* is named in honor of Brad Zlotnick’s contributions to publicity for ACG, GDFCF, and BioAlfa.

**Figure 431.**
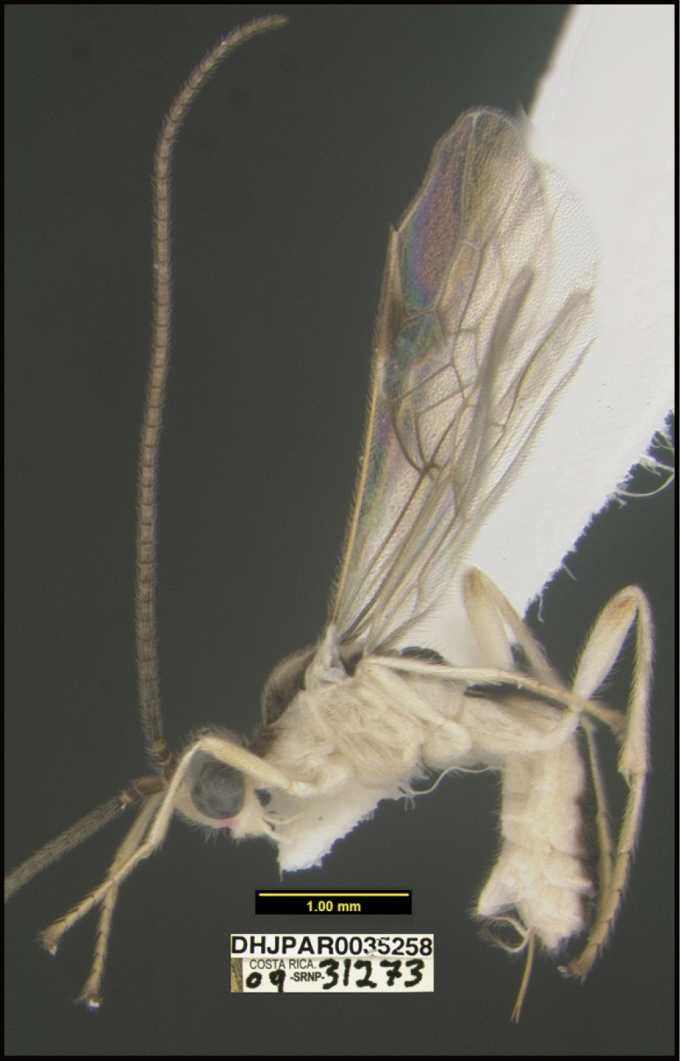
*Triraphisbradzlotnicki*, holotype.

##### Triraphis brianbrowni

Taxon classificationAnimaliaHymenopteraBraconidae

Sharkey sp. nov.

http://zoobank.org/2FA1F96E-4BD2-4C4F-BD2A-4AA0A56382B3

Figure 432

###### Diagnostics.

BOLD:AAE0329. Consensus barcode. TGTTTTATATTTTTTATTTGGAATTTGAGCAGGAATAGTRGGTTTGTCAATAAGATTAATTATTCGATTRGAATTAAGTATACCAGGTAGTTTATTAGGTAATGATCAAATTTATAATGGTATAGTAACAGCKCATGCTTTTATTATAATTTTTTTTATAGTTATACCTATYATAATTGGGGGGTTTGGTAATTGGTTRATTCCTTTAATATTAGGGGCTCCTGATATAGCTTTYCCTCGTATAAATAATATGAGTTTTTGATTATTAATTCCTTCATTAACTTTATTAATTTTAAGGGCTGTAGTWAATGTTGGGGTTGGTACTGGTTGAACTATRTATCCTCCTTTGTCTTCTTTAGTTGGTCATGGGGGTATGTCTGTTGATATAGCTATTTTTTCTTTACATTTAGCTGGTGCATCATCAATTATAGGTGTAGTTAATTTTATTTCTACTATTTTTAATATAAAATTAGTTTCTATTAGATTGGATCAAATTAATTTATTTGTTTGATCAGTTTTAATTACAGCTGTATTATTATTATTATCTTTACCTGTTTTAGCTGGTGCAATTACTATATTACTTACTGATCGTAATTTAAATACAACTTTTTTTGATTTTTCTGGTGGTGGAGATCCTATTTTATTTCAACATTTATTT.

###### Holotype ♀.

Guanacaste, Sector Mundo Nuevo, Quebrada Tibio Perla, 10.76261, -85.42979, 330 meters, caterpillar collection date: 16/xii/2013, wasp eclosion date: 03/i/2014. Depository: CNC.

***Host data*.***Panthiadesbitias* (Lycaenidae) feeding on *Ingavera* (Fabaceae).

***Host caterpillar and holotype wasp voucher codes*.** 13-SRNP-57666, DHJPAR0054548.

###### Paratypes.

DHJPAR0028327, DHJPAR0061496, BIOUG29630-A05, BIOUG09733-H06. Depository: CNC.

###### Other material.

Five specimens from the Chiquibul Rainforest in Belize are in the same BIN and are likely conspecific (BMNHE897790, BMNHE897797, BMNHE897810, BMNHE897828, BMNHE897833). They were not examined.

###### Etymology.

*Triraphisbrianbrowni* is named to acknowledge the contributions of Brian Brown to ACG, GDFCF, and BioAlfa through the identification of Costa Rican Diptera.

**Figure 432.**
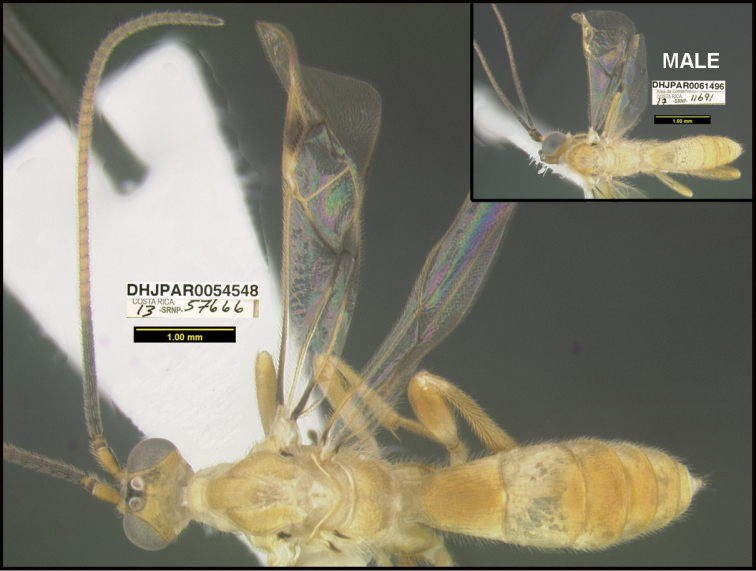
*Triraphisbrianbrowni*, holotype female and paratype male.

##### Triraphis brianlaueri

Taxon classificationAnimaliaHymenopteraBraconidae

Sharkey sp. nov.

http://zoobank.org/B2CCA099-B925-4827-91AD-4691EDF97591

Figure 433

###### Diagnostics.

BOLD:ACL0916. Consensus barcode. TGTATTATATTTTTTATTTGGTATTTGATCAGGTATGGTTGGTTTATCTATGAGGGTAATTATTCGGTTAGAATTAAGGATACCTGGTAGTTTATTAGGTAATGATCAAATTTATAATGGAATAGTTACTGCTCATGCACTTATTATAATTTTTTTTATAGTAATACCAATTATAATTGGAGGATTTGGTAATTGATTAATTCCTTTAATATTAGGAGCTCCTGATATGGCTTTCCCACGTATGAATAATATAAGATTTTGATTGTTAATTCCTTCATTAATTTTATTAATTTTGAGGGCGGTAGTAAATGTTGGAGTTGGGACAGGKTGAACAATTTATCCTCCTTTATCTTCTTTAATAGGTCATGGGGGGATATCTGTTGATATAGCTATTTTTTCTTTACATTTAGCAGGGGCTTCTTCTATTATGGGAGTAATTAATTTYATTTCTACTATTTTTAATATAAAATTAATTTCAATTAAGTTAGATCAAATTAATTTATTTGTTTGATCAGTTTTAATTACTGCGGTGYTATTATTATTATCTTTACCTGTTTTGGCAGGGGCTATYACTATATTATTAACTGATCGTAACTTAAATACTACTTTTTTTGATTTTTCTGGTGGAGGGGATCCTATTTTATTTCAACATTTATTT.

###### Holotype ♀.

Guanacaste, Sector Santa Rosa, Bosque San Emilio, 10.8438, -85.6138, 300 meters, dry forest, 31/xii/2012, Malaise trap. Depository: CNC.

***Host data*.** None.

***Holotype voucher code*.**BIOUG17956-H11.

###### Paratypes.

BIOUG09430-G03, BIOUG09740-C12, BIOUG09827-C08, BIOUG17566-G07, BIOUG18248-A06, BIOUG28779-A10, BIOUG29092-C07, BIOUG30950-C06. Depository: CNC.

###### Etymology.

*Triraphisbrianlaueri* is named in honor of Brian Lauer in appreciation of his years of IT support.

**Figure 433.**
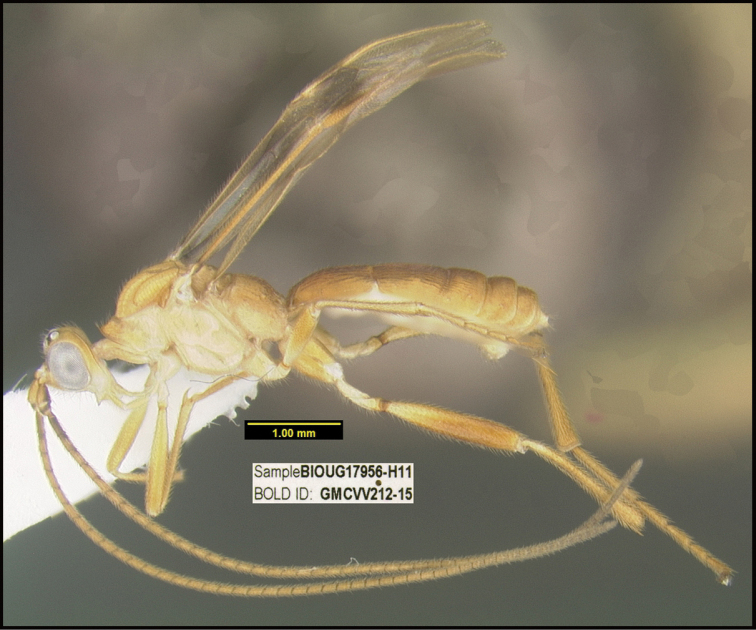
*Triraphisbrianlaueri*, holotype.

##### Triraphis briannestjacquesae

Taxon classificationAnimaliaHymenopteraBraconidae

Sharkey sp. nov.

http://zoobank.org/1F6C99C0-8262-4FD2-AB72-0C908E5B4F89

Figure 434

###### Diagnostics.

BOLD:AAF7900. Consensus barcode. AGTTTTATATTTTTTATTTGGTATTTGAGCGGGAATAGTTGGTTTATCTATAAGTTTGATTATTCGACTAGAATTAAGAATACCTGGTAGTTTATTAGGTAATGATCAAATTTATAATGGAATAGTTACTGCTCATGCTTTTATTATAATTTTTTTTATAGTAATACCTATTATAATTGGTGGATTTGGAAATTGGTTAATTCCTTTAATATTAGGAGCACCTGATATAGCTTTCCCTCGTATAAATAATATAAGATTTTGATTATTAATTCCTTCATTAACATTATTAATTTTAAGAGCTGTAGTTAATGTTGGGGTAGGAACTGGTTGAACATTGTATCCTCCTTTATCTTCTCTAGTGGGTCATGGGGGTATATCTGTAGATATAGCTATTTTTTCTTTACATTTAGCTGGGGTTTCTTCTATTATAGGTGTTGTAAATTTTATTTCTACTATTTTTAATATAAAATTAATTTCTATTAATTTAGATCAAATTAATTTATTTGTTTGATCAGTATTAATTACTGCTGTTTTATTATTATTATCTTTACCTGTATTGGCAGGGGCTATTACTATATTATTAACAGATCGTAATTTAAATACAACATTTTTTGATTTTTCTGGAGGAGGAGATCCAATTTTATTTCAACATTTATTT.

###### Holotype ♀.

Alajuela, Sector Rincon Rain Forest, Estación Caribe, 10.90187, -85.27495, 415 meters, caterpillar collection date: 07/vii/2014, wasp eclosion date: 18/vii/2014. Depository: CNC.

***Host data*.***Trosianigropunctigera* (Megalopygidae) feeding on *Miconiaxalapensis* (Melastomataceae).

***Host caterpillar and holotype wasp voucher codes*.** 14-SRNP-43092, DHJPAR0055967.

###### Paratypes.

DHJPAR0045388 DHJPAR0055965 DHJPAR0055966 DHJPAR0055968. Depository: CNC.

###### Etymology.

*Triraphisbriannestjacquesae* is named in appreciation of Brianne St. Jacques’ contributions to publicity for ACG, GDFCF, and BioAlfa.

**Figure 434.**
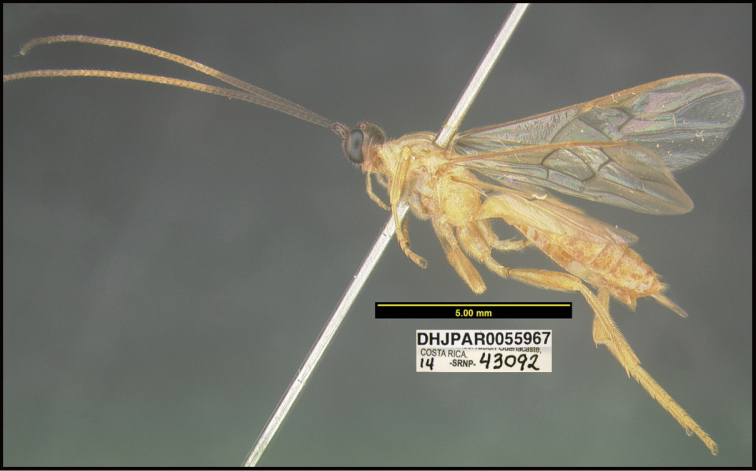
*Triraphisbriannestjacquesae*, holotype.

##### Triraphis camilocamargoi

Taxon classificationAnimaliaHymenopteraBraconidae

Sharkey sp. nov.

http://zoobank.org/0B1F10B3-D932-429A-816E-9DA392B38981

Figure 435

###### Diagnostics.

BOLD:AAJ3968. Consensus barcode. TGTTTTATATTTTTTATTTGGTATTTGAGCTGGTATAGTGGGATTATCAATAAGTTTAATTATTCGTTTAGAATTAAGTATACCAGGTAGTTTATTAGGTAATGATCAAATTTATAATGGAATAGTAACGGCTCATGCTTTTATTATAATTTTTTTTATAGTTATACCTATTATAATTGGGGGATTTGGTAATTGATTAATTCCTTTAATATTGGGGGCACCTGATATAGCTTTTCCACGTATAAATAATATAAGTTTTTGATTATTAATTCCTTCATTAACTTTATTAATTTTAAGTGCTGTAGTAAATGTTGGGGTAGGGACTGGGTGAACTATATATCCTCCTTTATCTTCTTTAGTTGGTCATGGGGGGATATCTGTTGATATGGCAATTTTTTCTTTACATTTAGCAGGGGCATCTTCTATTATAGGGGTAGTTAATTTTATTTCTACTATTTTTAATATAAAATTAATTTCTATTAGTTTAGATCAAATTAATTTATTTGTTTGATCTGTTTTAATTACAGCAGTATTATTATTATTATCTTTACCTGTTTTAGCTGGGGCTATTACTATATTACTTACTGATCGTAATTTGAATACAACTTTTTTTGACTTTTCTGGTGGGGGGGATCCTATTTTATTTCAACATTTATTT. *Triraphisdonbrighti* occupies the same BIN as *T.camilocamargoi* but can be separated by multiple morphological characters, the most obvious of which is the single terminal melanic band in the forewing of *T.donbrighti* which in *T.camilocamargoi* is accompanied by a second band near midlength of the forewing.

###### Holotype ♀.

Alajuela, Sector San Cristobal, Sendero Huerta, 10.93050, -85.37223, 527 meters, caterpillar collection date: 02/vii/2012, wasp eclosion date: 15/vii/2012. Depository: CNC.

***Host data*.***Anterosformosus* (Riodinidae) feeding on *Miconiaxalapensis* (Melastomataceae).

***Host caterpillar and holotype wasp voucher codes*.** 12-SRNP-2680, DHJPAR0049947.

###### Paratypes.

Hosts = same as holotype. DHJPAR0017285, DHJPAR0049949, DHJPAR0051362. Depository: CNC.

###### Etymology.

*Triraphiscamilocamargoi* is named in honor of Camilo Camargo’s long-appreciated contributions to publicity for ACG, GDFCF, and now, BioAlfa.

**Figure 435.**
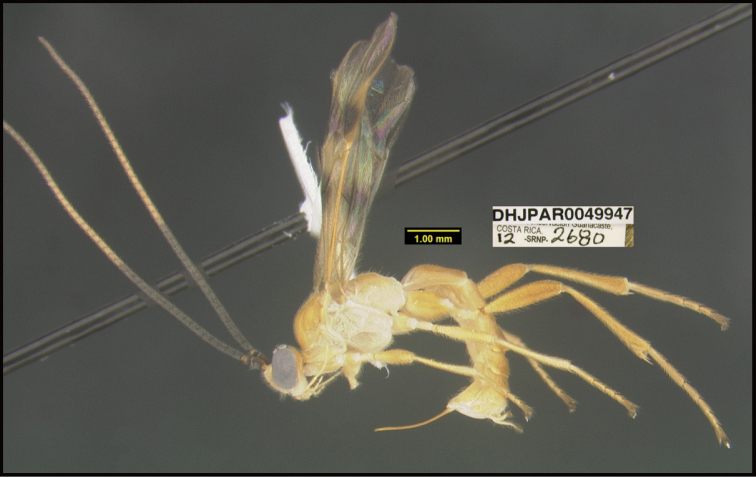
*Triraphiscamilocamargoi*, holotype.

##### Triraphis carlosherrerai

Taxon classificationAnimaliaHymenopteraBraconidae

Sharkey sp. nov.

http://zoobank.org/774EA64C-8E50-4BD7-A274-24CD17154DF4

Figure 436

###### Diagnostics.

BOLD:AAW1546. Consensus barcode. TGTTTTATATTTTTTATTTGGTATTTGAGCAGGAATAGTGGGATTATCAATAAGTTTAATTATTCGTTTAGAGTTAAGAATACCAGGTAGTTTATTAGGTAATGATCAAATTTATAATAGAATAGTAACAGCTCATGCTTTTATTATAATTTTTTTTATGGTTATACCTATTATAATTGGGGGGTTTGGTAATTGATTAATTCCATTAATATTGGGGGCYCCTGATATAGCTTTTCCACGTATAAATAATATAAGATTTTGATTATTAATTCCTTCATTGACTTTATTAATTTTAAGGGCTATAGTTAATGTTGGTGTAGGGACTGGTTGAACTATATATCCTCCTTTATCTTCTTTAGTTGGTCATGGTGGCATATCAGTTGATATAGCAATTTTTTCTTTACATTTAGCAGGTATTTCATCTATTATAGGTGTAGTTAATTTTATTTCTACAATTTTTAATATAAAATTAATTTCTATTGGTTTAGATCAAATTAATTTATTTGTTTGGTCTGTTTTAATTACAGCAGTATTATTATTATTATCTTTACCTGTTTTGGCAGGGGCTATTACTATATTACTTACTGATCGTAATTTAAATACAACTTTTTTTGATTTTTCTGGGGGTGGAGATCCTATTTTATTTCAACATTTATTT.

###### Holotype ♀.

Alajuela, Sector San Cristobal, Puente Palma, 10.9163, -85.37869, 460 meters, caterpillar collection date: 04/xi/2008, wasp eclosion date: 11/xi/2008. Depository: CNC.

***Host data*.***Emesismandana* (Riodinidae) feeding on *Hieronymaalchorneoides* (Phyllanthaceae). Gregarious parasitoid with 27 specimens emerging from the host.

***Host caterpillar and holotype wasp voucher codes*.** 08-SRNP-6211, DHJPAR0030715.

###### Paratypes.

Host = same as holotype. DHJPAR0050030. Depository: CNC.

###### Etymology.

*Triraphiscarlosherrerai* is named to acknowledge Carlos Herrera’s contributions to publicity for ACG, GDFCF, and now, BioAlfa.

###### Note.

This is a gregarious parasitoid with 27 wasps emerging from the host larval mummy.

**Figure 436.**
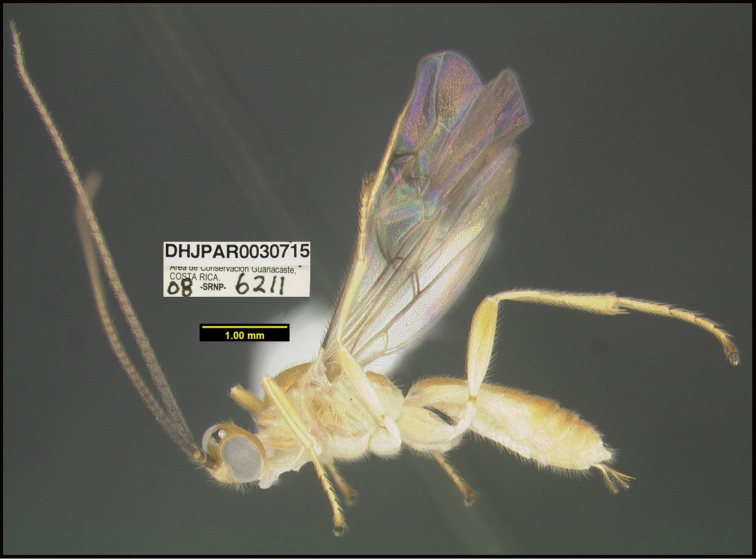
*Triraphiscarlosherrerai*, holotype.

##### Triraphis carolinepalmerae

Taxon classificationAnimaliaHymenopteraBraconidae

Sharkey sp. nov.

http://zoobank.org/52000592-EB89-4DD2-9E35-E2012A2AFF98

Figure 437

###### Diagnostics.

BOLD:ABA9319. Consensus barcode. TGTTTTATATTTTTTATTTGGTATTTGGGCTGGTATAGTTGGTTTATCTATAAGGTTAATTATTCGTTTAGAATTAAGTATACCTGGTAGATTACTGGGTAATGATCAAATTTATAATGGTATAGTAACTGCTCATGCTTTTATTATAATTTTTTTTATAGTTATACCTATTATAATTGGCGGGTTTGGGAATTGATTAATTCCTTTAATATTGGGGGCACCTGATATAGCTTTTCCTCGTATAAATAATATGAGTTTTTGATTATTAATTCCTTCATTAACATTATTAATTTTAAGGGCTGTAGTTAATGTTGGGGTAGGTACTGGGTGAACTTTATATCCACCTTTATCTTCTTTAGTTGGTCATGGGGGCATATCTGTAGATATAKCTATTTTTTCTTTACATTTAGCTGGTGCTTCTTCTATTATGGGGGTTGTTAATTTTATTTCTACTATTTTTAATATAAAATTAGTATCAATTAATTTAGATCAAATTAATTTATTTGTTTGATCAGTATTAATTACAGCTGTTTTATTATTATTGTCTTTACCAGTATTAGCTGGTGCAATTACTATATTATTGACAGATCGTAATTTAAATACAACATTTTTTGATTTTTCTGGTGGGGGGGA-CCTATTTTA-TTCAACACTTATTT.

###### Holotype ♂.

Guanacaste, Sector Pitilla, Sendero Laguna, 10.9888, -85.42336, 680 meters, caterpillar collection date: 06/vi/2011, wasp eclosion date: 20/vi/2011. Depository: CNC.

***Host data*.***Isochaetesdwagsi* (Limacodidae) feeding on Solanaceae 16453 (Solanaceae).

***Host caterpillar and holotype wasp voucher codes*.** 11-SRNP-31583, DHJPAR0045383.

###### Paratype.

BIOUG27999-A10. Depository: CNC.

###### Etymology.

*Triraphiscarolinepalmerae* is named in honor of Caroline Palmer’s long-appreciated contributions to publicity for ACG, GDFCF, and now, BioAlfa.

**Figure 437.**
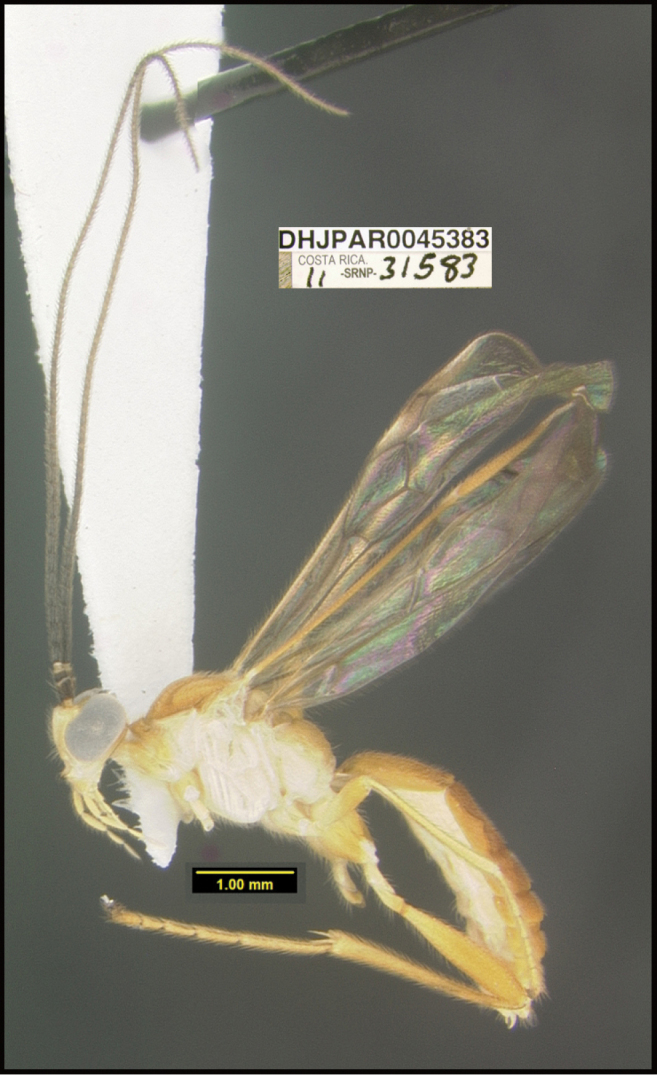
*Triraphiscarolinepalmerae*, holotype.

##### Triraphis charlesmorrisi

Taxon classificationAnimaliaHymenopteraBraconidae

Sharkey sp. nov.

http://zoobank.org/CB1DA8EA-1A73-44C8-9500-AEB335D13977

Figure 438

###### Diagnostics.

BOLD:ABU8062. Consensus barcode. TGTTTTATATTTTTTATTTGGAATTTGATCAGGTATAGTTGGTTTGTCAATAAGATTAATTATTCGTTTAGAATTAAGAATACCAGGTAGTTTATTAGGTAATGATCAAATTTATAATGGTATAGTTACAGCTCATGCATTTATTATAATTTTTTTTATAGTTATGCCTATTATAATTGGTGGGTTTGGTAATTGATTAATTCCTTTAATATTAGGTGCTCCTGATATAGCTTTTCCTCGTATAAATAATATAAGTTTTTGGTTATTAATTCCTTCTTTAACTTTATTAATTTTAAGAGCTGTAGTTAATGTTGGGGTTGGTACTGGATGAACTATATATCCTCCTTTATCTTCTTTAGTTGGTCATGGTGGAATATCTGTTGATATAGCAATTTTTTCTTTACATTTGGCTGGGGTATCTTCTATTATGGGGGTAATTAATTTTATTTCAACTATTTTTAATATAAAATTAATTTCTATTAAATTAGATCAAATTAATTTATTTGTTTGATCAGTTTTAATTACAGCTTTTTTATTATTATTATCTTTACCTGTTTTAGCAGGTGCTATTACTATATTATTTACTGATCGTAATTTAAATACAACTTTTTTTGATTTTTCGGGTGGAGGAGATCCAATTTTATTTCAACATTTATTT.

###### Holotype ♀.

Guanacaste, Sector Pitilla, Sendero Laguna, 10.9888, -85.42336, 680 meters, caterpillar collection date: 23/ix/2011, wasp eclosion date: 10/x/2011. Depository: CNC.

***Host data*.***Eurybialycisca* (Riodinidae) feeding on *Calathealasiostachya* (Marantaceae).

***Host caterpillar and holotype wasp voucher codes*.** 11-SRNP-32841, DHJPAR0045793.

###### Paratypes.

Host = same as holotype. DHJPAR0045790, DHJPAR0045791, DHJPAR0045792, DHJPAR0045794. Depository: CNC.

###### Etymology.

*Triraphischarlesmorrisi* is named in appreciation of the contributions of Charles Morris to increase publicity for ACG, GDFCF, and now, BioAlfa.

**Figure 438.**
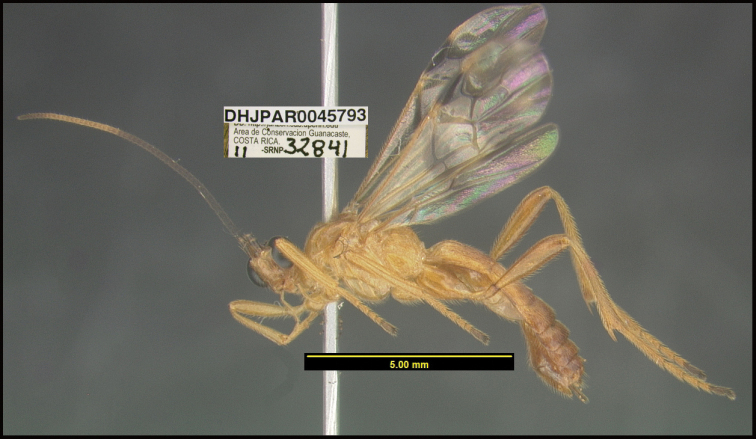
*Triraphischarlesmorrisi*, holotype.

##### Triraphis chigiybinellae

Taxon classificationAnimaliaHymenopteraBraconidae

Sharkey sp. nov.

http://zoobank.org/97D46995-0093-4CBB-97B5-4D2C848E1551

Figure 439

###### Diagnostics.

BOLD:ACK5778. Consensus barcode. TGTTTTATATTTTTTATTTGGAATTTGAGCAGGAATATTAGGTTTATCAATAAGGTTAATTATTCGATTAGAATTAAGTATACCT---GGTAGTTTATTAGGTAATGATCAAATTTATAATGGTATAGTTACAGCCCATGCTTTTATTATAATTTTTTTTATAGTTATACCTATTATAATTGGTGGTTTTGGAAATTGATTAATTCCTTTAATATTAGGGGCTCCTGATATAGCTTTTCCTCGTATAAATAATATAAGTTTTTGATTATTAATTCCTTCATTAACTTTATTGATTTTAAGTGCTGTAGTTAATGTTGGGGTTGGTACTGGTTGAACTATATATCCTCCTTTGTCTTCTTTAGTT---GGTCATGGGGGAATATCTGTTGATATGGCAATTTTTTCTTTACATTTAGCTGGTGCGTCATCTATTATAGGAGTAGTTAATTTTATTTCTACTATTTTTAATATAAAATTAATTTCTATTAGATTAGATCAAATTAATTTATTTGTTTGATCAGTTTTAATTACAGCTATATTATTATTATTATCTTTACCTGTATTAGCTGGTGCAATTACTATATTACTTACTGATCGTAATTTAAATACAACTTTTTTTGATTTTTCTGGTGGTGGGGATCCTATTTTATTTCAACATTTATTT.

###### Holotype ♀.

Alajuela, Sector San Cristobal, Finca San Gabriel, 10.87766, -85.39343, 645 meters, caterpillar collection date: 23/ix/2013, wasp eclosion date: 08/x/2013. Depository: CNC.

***Host data*.***Metacharisvictrix* (Riodinidae) feeding on *Heisteriacostaricensis* (Olacaceae).

***Host caterpillar and holotype wasp voucher codes*.** 13-SRNP-4931, DHJPAR0053602.

###### Paratypes.

None.

###### Etymology.

*Triraphischigiybinellae* is named in honor of Chigiy Binell’s long-appreciated contributions to publicity for ACG, GDFCF, and now, BioAlfa.

**Figure 439.**
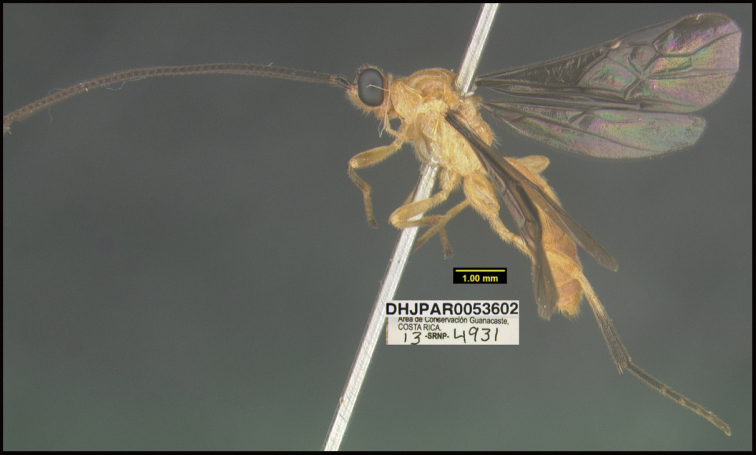
*Triraphischigiybinellae*, holotype.

##### Triraphis christerhanssoni

Taxon classificationAnimaliaHymenopteraBraconidae

Sharkey sp. nov.

http://zoobank.org/D38DC4D0-240C-43B2-A197-F02F308DD57C

Figure 440

###### Diagnostics.

BOLD:ADB1219. Consensus barcode. AGTATTATATTTTTTATTTGGAATTTGGGCAGGTATAGTTGGATTATCTATAAGTTTAATTATTCGTTTAGAATTAAGAATGCCTGGAAGTTTACTAGGTAATGATCAAATTTATAATGGGATAGTTACTGCTCATGCTTTTATTATAATTTTTTTTATAGTTATACCTATTATAATTGGTGGTTTTGGTAATTGACTAATTCCATTAATATTGGGGGCTCCTGATATAGCTTTTCCTCGTATAAATAATATAAGATTTTGGCTATTAATTCCTTCATTAACATTATTAATTTTAAGAGCTGTTGTTAATGTTGGAGTAGGGACTGGGTGAACATTATATCCTCCCTTATCTTCTTTAGTTGGTCATGGTGGGATATCTGTAGATATAGCTATTTTTTCTTTACATTTAGCTGGTGCTTCTTCAATTATAGGGGTTGTTAATTTTCTTTCTACTATTTTTAATATAAAATTAGTATCTATTAATTTAGATCAAATTAATTTATTTGTTTGATCAGTATTAATTACTGCTGTTTTATTATTATTATCTTTACCAGTATTAGCTGGGGCTATTACTATATTATTGACAGATCGTAATTTAAATACAACCTTTTTTGATTTTTCTGGTGGAGGAGATCCTATTTTATTTCAACATTTATTT.

###### Holotype ♀.

Guanacaste, Sector Cacao, Sendero Arenales, 10.92471, -85.46738, 1080 meters, caterpillar collection date: 04/xi/2015, wasp eclosion date: 21/xi/2015. Depository: CNC.

***Host data*.** megaJanzen01 98-SRNP-3934 (Megalopygidae) feeding on *Ingapunctata* (Fabaceae).

***Host caterpillar and holotype wasp voucher codes*.** 15-SRNP-35615, DHJPAR0058772.

###### Paratypes.

Hosts = megaJanzen01. DHJPAR0058770, DHJPAR0058771, DHJPAR0058773, DHJPAR0058774, DHJPAR0058775. Depository: CNC.

###### Etymology.

*Triraphischristerhanssoni* is named in honor of Christer Hansson’s long-appreciated contributions to publicity for ACG, GDFCF, and now, BioAlfa.

**Figure 440.**
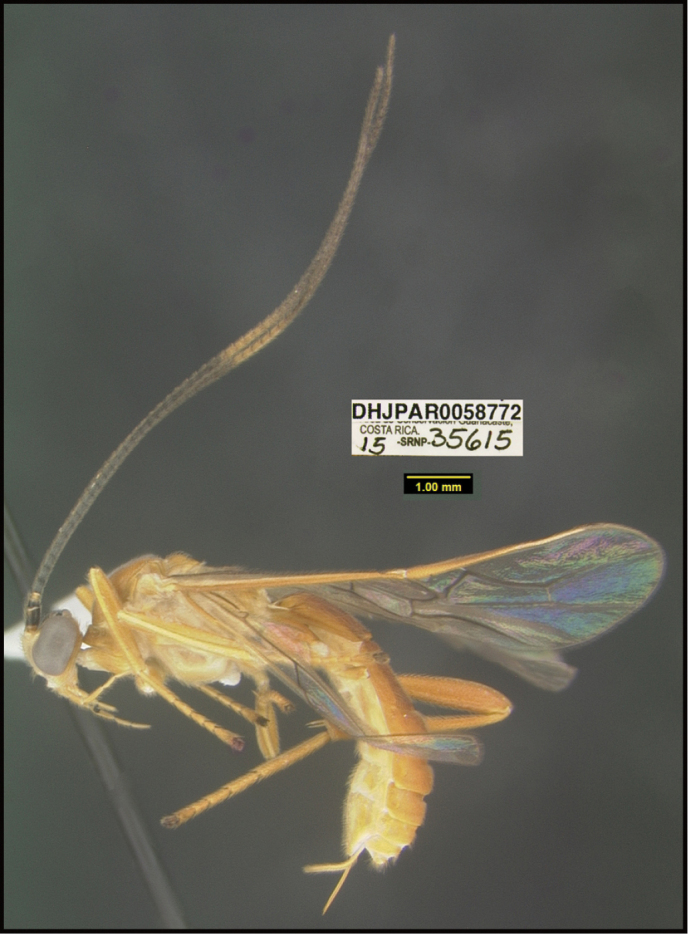
*Triraphischristerhanssoni*, holotype.

##### Triraphis christhompsoni

Taxon classificationAnimaliaHymenopteraBraconidae

Sharkey sp. nov.

http://zoobank.org/27FEFBEB-8911-4B3F-8617-DF15812728EA

Figure 441

###### Diagnostics.

BOLD:ACK7801. Consensus barcode. TGTATTATATTTTTTATTTGGAATTTGATCAGGTATAGTTGGGTTATCTATAAGTTTAATTATTCGTTTAGAATTAAGAATACCTGGAAGTTTATTGGGGAATGATCAAATTTATAATGGTATAGTGACTGCCCATGCTTTTATTATAATTTTTTTTATGGTTATACCTATTATAATTGGTGGGTTTGGAAATTGATTAATTCCCYTAATATTAGGGGCTCCTGATATAGCTTTTCCTCGTATAAATAATATAAGATTTTGATTATTAATTCCTTCATTAACTTTAYTAATTTTAAGGGCTGTAGTTAATGTTGGGGTAGGAACAGGATGAACTTTATATCCACCTTTATCTTCTTTAGTTGGACATGGTGGAATATCTGTTGATATAGCTATTTTTTCTTTACATTTGGCTGGGGCGTCTTCAATTATAGGGGTTGTTAATTTTATTTCTACTATTTTTAATATAAAATTAGTATCAATTAATTTAGATCAAATTAATTTATTTGTTTGATCAGTATTAATTACTGCTGTTTTATTATTATTATCTTTACCAGTATTGGCAGGTGCTATTACTATATTGTTAACAGATCGTAATTTAAATACAACATTTTTTGATTTTTCTGGTGGTGGGGATCCTATTTTATTTCAACATTTATTT.

###### Holotype ♀.

Guanacaste, Sector El Hacha, Estación Los Almendros, 11.03226, -85.52776, 290 meters, caterpillar collection date: 29/ix/2011, wasp eclosion date: 10/x/2011. Depository: CNC.

***Host data*.** zygJanzen01 Janzen04 (Zygaenidae) feeding on *Davillanitida* (Dilleniaceae).

***Host caterpillar and holotype wasp voucher codes*.** 11-SRNP-22833, DHJPAR0045818.

###### Paratypes.

Hosts = same as holotype. DHJPAR0029048, DHJPAR0029064, DHJPAR0029059, DHJPAR0048107, DHJPAR0050069, DHJPAR0059698. Depository: CNC.

###### Other material.

A specimen from Mexico (BIOUG20849-A08) and one from Belize (NHM749275) are in the same BIN and are likely conspecific with *T.christhompsoni*. They were not examined.

###### Etymology.

The species epithet is a patronym for Chris Thompson, a noted North American dipterist, and acknowledges his significant contributions to the inventory of ACG, and Costa and Rica in general, through the identification of Diptera and assisting with the curation of the collection formerly known as INBio.

**Figure 441.**
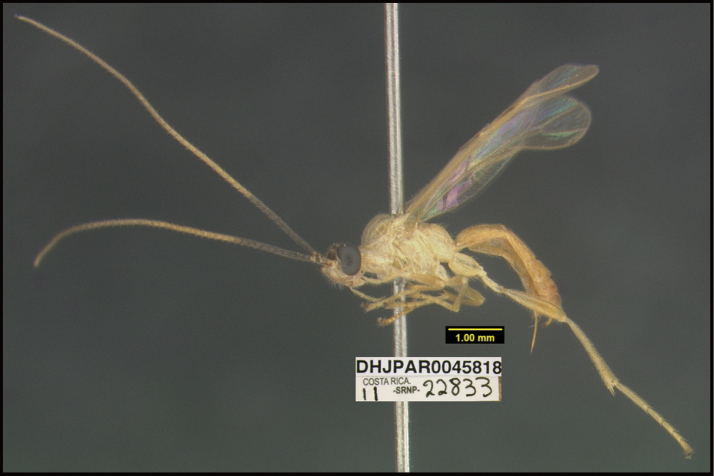
*Triraphischristhompsoni*, holotype.

##### Triraphis conniebarlowae

Taxon classificationAnimaliaHymenopteraBraconidae

Sharkey sp. nov.

http://zoobank.org/81277D3E-4CB2-420D-B6A1-503C3F320368

Figure 442

###### Diagnostics.

BOLD:ADJ3083. Consensus barcode. TGTTTTATATTTTTTATTTGGAATTTGAGCAGGAATAGTAGGTTTATCAATGAGATTAATTATTCGATTAGAATTAAGTATACCGGGAAGTTTATTAGGTAATGATCAAATTTATAATGGTATAGTAACTGCTCATGCTTTTATTATAATTTTTTTTATAGTTATACCTATTATAATTGGGGGATTTGGTAATTGGTTGATTCCTTTAATATTAGGGGCTCCTGATATAGCTTTTCCTCGTATAAATAATATAAGTTTTTGATTATTAATTCCTTCATTAACTTTATTAATTTTAAGGGCTGTAGTTAATGTTGGGGTTGGGACTGGTTGGACTATATATCCTCCTTTATCTTCTTTAGTTGGTCATGGGGGCATATCTGTTGATATGGCTATTTTTTCTTTACATTTAGCGGGTGCATCATCTATTATAGGTGTAGTTAATTTTATTTCTACTATTTTTAATATAAAATTAGTTTCTATTAGATTGGATCAAATTAATTTATTTGTTTGATCAGTTTTAATTACAGCTGTATTATTATTATTATCTTTACCTGTTTTAGCCGGTGCAATTACTATACTACTTACTGATCGTAATTTAAATACAACTTTTTTTGATTTTTCTGGTGGTGGGGATCCTATTTTATTTCAACATTTATTT.

###### Holotype ♀.

Guanacaste, Sector Pitilla, Medrano, 11.01601, -85.38052, 380 meters, caterpillar collection date: 22/vii/2017, wasp eclosion date: 07/viii/2017. Depository: CNC.

***Host data*.***Cariarhacotis* (Riodinidae) feeding on *Celtisiguanaea* (Ulmaceae).

***Host caterpillar and holotype wasp voucher codes*.** 17-SRNP-72605, DHJPAR0061495.

###### Paratype.

Host = same as holotype. DHJPAR0061498. Depository: CNC.

###### Etymology.

*Triraphisconniebarlowae* is named in honor of Connie Barlow’s long-appreciated contributions to publicity for ACG, GDFCF, and now, BioAlfa.

**Figure 442.**
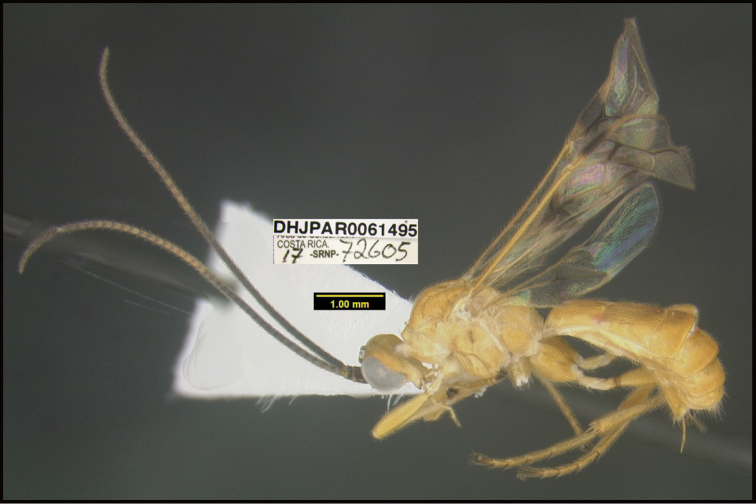
*Triraphisconniebarlowae*, holotype.

##### Triraphis craigsimonsi

Taxon classificationAnimaliaHymenopteraBraconidae

Sharkey sp. nov.

http://zoobank.org/FBB8BEC4-99C2-498D-A198-894B7EC70653

Figure 443

###### Diagnostics.

BOLD:ADM7982. Consensus barcode. TGTTTTATATTTTTTATTTGGAATTTGAGCAGGAATAGTAGGTTTATCAATGAGATTAATTATTCGATTAGAATTAAGTATACCAGGAAGTTTATTAGGTAATGATCAGATTTATAATGGTATAGTTACAGCTCATGCTTTTATTATAATTTTTTTTATAGTTATACCTATTATAATTGGGGGATTTGGTAATTGGTTAATTCCTTTAATATTAGGGGCTCCTGACATAGCTTTTCCTCGTATAAATAATATGAGGTTTTGATTATTAATTCCTTCATTAACTTTATTAATTTTAAGAGCTGTAGTTAATGTTGGAGTTGGTACTGGTTGAACTATATATCCTCCTTTATCTTCTTTGGTTGGTCATGGGGGTATATCTGTTGATATAGCTATTTTTTCTTTACATTTAGCGGGTGCATCATCTATTATAGGTGTAGTTAATTTTATTTCTACTATTTTTAATATAAAATTAATTTCTATTGGATTGGATCAAATTAATTTATTTGTTTGATCAGTTTTAATTACAGCTGTATTATTATTATTATCTTTACCTGTTTTGGCTGGTGCAATTACTATATTACTTACTGATCGTAATTTAAATACAACTTTTTTTGATTTTTCTGGTGGTGGGGACCCTATTTTATTTCAACATTTATTT.

###### Holotype ♀.

Guanacaste, Sector Pitilla, Loaiciga, 11.01983, -85.41342, 445 meters, caterpillar collection date: 19/iii/2007, wasp eclosion date: 09/iv/2007. Depository: CNC.

***Host data*.***Napaeaeucharila* (Riodinidae) feeding on *Vrieseasanguinolenta*.

***Host caterpillar and holotype wasp voucher codes*.** 07-SRNP-31839, DHJPAR0062174.

###### Paratypes.

None.

###### Etymology.

*Triraphiscraigsimonsi* is named in honor of Craig Simons’ long-appreciated contributions to publicity for ACG, GDFCF, and now, BioAlfa.

**Figure 443.**
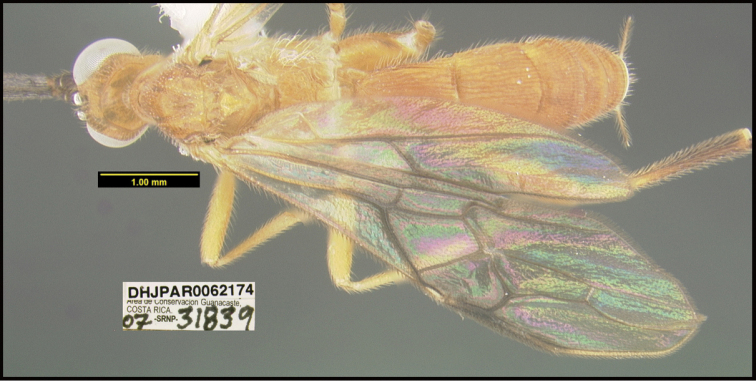
*Triraphiscraigsimonsi*, holotype.

##### Triraphis danielhubi

Taxon classificationAnimaliaHymenopteraBraconidae

Sharkey sp. nov.

http://zoobank.org/B138C6B0-F730-4034-8E6B-AE0097E483F2

Figure 444

###### Diagnostics.

BOLD:AAM9596. Consensus barcode. AGTATTATATTTTTTATTTGGTATTTGATCYGGTATGGTRGGATTATCTATGAGTTTAATTATTCGTTTAGAATTAAGAATACCYGGTAGTTTATTGGGTAATGATCAAATTTACAATGGRATAGTTACTGCTCATGCTTTAATTATAATTTTTTTTATAGTTATGCCTATTATAATTGGTGGGTTTGGAAATTGATTAGTACCYTTAATATTGGGGTCCCCCGATATAGCTTTTCCTCGAATAAATAATATAAGATTTTGATTATTAATTCCYTCTTTAATTTTATTAATATTAAGAGCTGTAGTAAATATTGGGGTTGGCACTGGTTGAACTATATAYCCTCCTTTATCTTCTTTAATTGGTCATGGGGGTATATCTGTTGATATAGCAATTTTTTCTTTACATTTAGCCGGTATTTCATCTATTATAGGGGTAATTAATTTTATTTCTACTATTTTTAATATGAAATTAGTTTCAATTAAGTTGGATCAAATTAATTTATTTATTTGATCTGTTTTAATTACTGCTATATTATTATTATTATCTTTACCTGTTTTAGCGGGAGCAATTACTATATTATTGACTGATCGTAATTTAAATACAACTTTTTTTGATTTTTCTGGGGGAGGAGATCCTATTTTATTTCAGCATTTATTT.

###### Holotype ♀.

Guanacaste, Sector Santa Rosa, Bosque San Emilio, 10.8438, -85.6138, 300 meters, dry forest, 01/x/2012, Malaise trap. Depository: CNC.

***Host data*.** None.

***Holotype voucher code*.**BIOUG17468-D07.

###### Paratypes.

All Malaise-trapped. BIOUG08905-F04, BIOUG08911-B10, BIOUG08911-B11, BIOUG17494-D05. Depository: CNC.

###### Other material.

A specimen from Belize (BMNHE897776) is in the same BIN and is likely conspecific.

###### Etymology.

*Triraphisdanielhubi* is named in honor of Daniel Hub’s long-appreciated contributions to publicity for ACG, GDFCF, and now, BioAlfa.

###### Note.

Males lack the pale band on the antenna.

**Figure 444.**
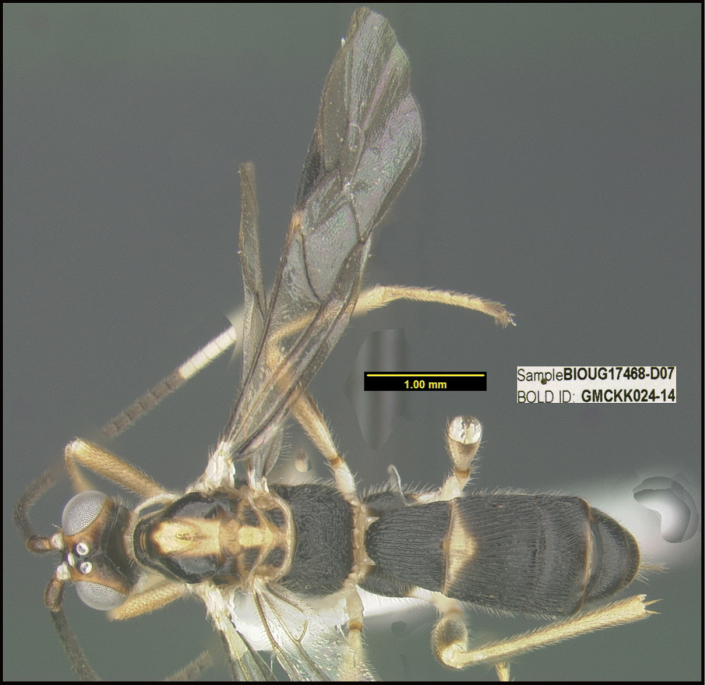
*Triraphisdanielhubi*, holotype.

##### Triraphis davidduthiei

Taxon classificationAnimaliaHymenopteraBraconidae

Sharkey sp. nov.

http://zoobank.org/2D4BF74F-A4DD-44BC-A3A5-E9934DAE7E37

Figure 445

###### Diagnostics.

BOLD:ACF6933. Consensus barcode. TGTTTTATATTTTTTATTTGGTATTTGAGCGGGTATAGTGGGTTTATCAATGAGGTTAATTATTCGTTTAGAATTAAGTATGCCAGGTAGTTTATTAGGTAATGATCAGATTTATAATGGTATAGTTACAGCTCATGCTTTTATTATAATTTTTTTTATAGTTATACCTATTATAATTGGTGGATTTGGTAATTGATTAATTCCTTTAATATTAGGAGCTCCTGATATAGCTTTCCCTCGTATAAATAATATAAGTTTTTGATTATTAATTCCTTCATTAACTTTATTAATTTTAAGAGCTGTAGTTAATGTTGGGGTTGGGACTGGTTGAACTATGTATCCTCCTTTGTCTTCTTTAATTGGTCATGGGGGGATATCTGTTGATATAGCAATTTTTTCTTTACATTTAGCGGGGGYATCATCTATTATAGGTGTAGTTAATTTTATTTCTACTATTTTTAATATAAAATTAATCTCTATTAGATTAGATCAAATTAATTTATTTGTTTGATCAGTTTTAATTACAGCTGTATTATTATTATTATCTTTACCTGTTTTAGCTGGTGCAATTACTATATTACTTACTGATCGTAATTTAAATACAACTTTTTTTGATTTTTCTGGTGGTGGAGATCCTATTTTATTTCAACATTTATTT.

###### Holotype ♀.

Guanacaste, Pailas Dos, PL12-6, 10.7637, -85.333, 853 meters, 23/i/2014, Malaise trap. Depository: CNC.

***Host data*.** None.

***Holotype voucher code*.**BIOUG28804-H04.

###### Paratypes.

All Malaise-trapped. BIOUG05181-G05, BIOUG07453-D06, BIOUG17330-A05, BIOUG18040-B11, BIOUG29481-H06, BIOUG29677-D09.

###### Etymology.

*Triraphisdavidduthiei* is named in honor of David Duthie’s long-appreciated contributions to publicity for ACG, GDFCF, and now, BioAlfa.

**Figure 445.**
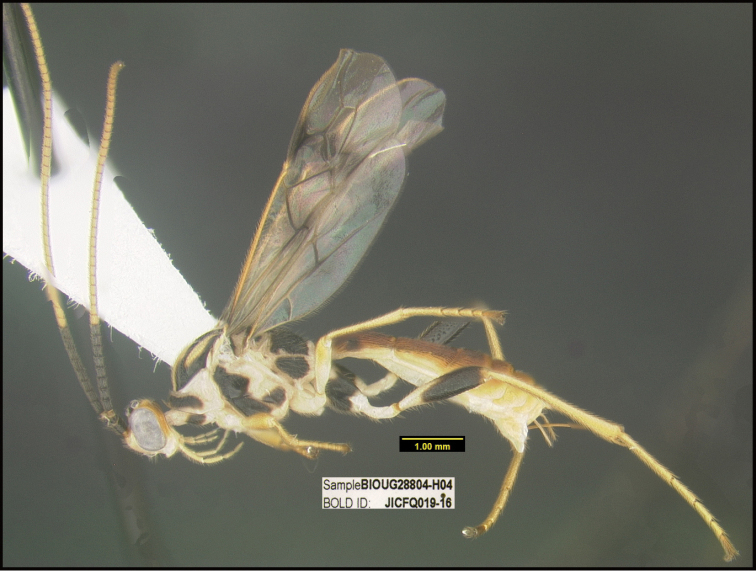
*Triraphisdavidduthiei*, holotype.

##### Triraphis davidwahli

Taxon classificationAnimaliaHymenopteraBraconidae

Sharkey sp. nov.

http://zoobank.org/49874F24-61F4-477B-B5EC-8385C89190D5

Figure 446

###### Diagnostics.

BOLD:AAM9598. Consensus barcode. TGTYTTATATTTTATATTTGGTATTTGAGCAGGRATAGTTGGYTTATCAATGAGATTAATTATTCGGTTAGAATTAAGCATACCAGGTAGTTTATTAGGTAATGATCAAATTTATAATGGYATAGTAACAGCTCATGCTTTTATTATAATTTTTTTTATAGTTATGCCTATTATAATTGGGGGGTTTGGTAATTGGTTGATTCCTTTGATATTAGGGGCTCCTGATATAGCTTTCYCTCGTATAAATAACATAAGTTTTTGAYTATTAATTCCTTCATTAACTTTATTAGTTTTAAGAGCTGTAGTTAATGTAGGGGTTGGAACTGGATGGACTATATATCCTCCTTTATCTTCTTTAATTGGTCATGGTGGAATGTCTGTTGATATAGCTATTTTTTCTTTACATTTAGCGGGYGYATCATCKATTATAGGTGTAGTTAATTTTATTTCTACTATTTTTAATATAAAATTAATTTCTATTAAATTGGATCAAATTAATTTATTTGTTTGATCAGTTTTAATTACAGCTGTATTATTATTATTATCTTTACCTGTTTTAGCTGGYGCAATTACTATATTACTTACTGATCGTAATTTAAATACAACTTTTTTTGATTTTTCTGGTGGTGGTGATCCTATTTTATTTCAACATTTATTT.

###### Holotype ♀.

Guanacaste, Sector Santa Rosa, Bosque San Emilio, 10.8438, -85.6138, 300 meters, dry forest, 25/ii/2013, Malaise trap. Depository: CNC.

***Host data*.** No data for holotype, but see below for paratype.

***Holotype voucher code*.**BIOUG18433-B04.

###### Paratypes.

BIOUG05082-G10, BIOUG08078-H08, BIOUG08539-D09, BIOUG09433-G08, BIOUG09826-F07, BIOUG09740-D11, BIOUG09740-D11, DHJPAR0055202 [Host = *Panthiadesbitias* (Lycaenidae)], BIOUG17616-C02, BIOUG17714-E06, BIOUG17759-D05. Depository: CNC.

###### Other material.

A specimen from Belize (BMNHE897830) is in the same BIN and is likely conspecific. A specimen from French Guiana (CCDB-07375 D01) is in the same BIN, and the image on BOLD is consistent with those from Costa Rica. Neither was examined.

###### Etymology.

*Triraphisdavidwahli* is named in honor of David Wahl’s long-appreciated contributions to publicity for ACG, GDFCF, and now, BioAlfa.

**Figure 446.**
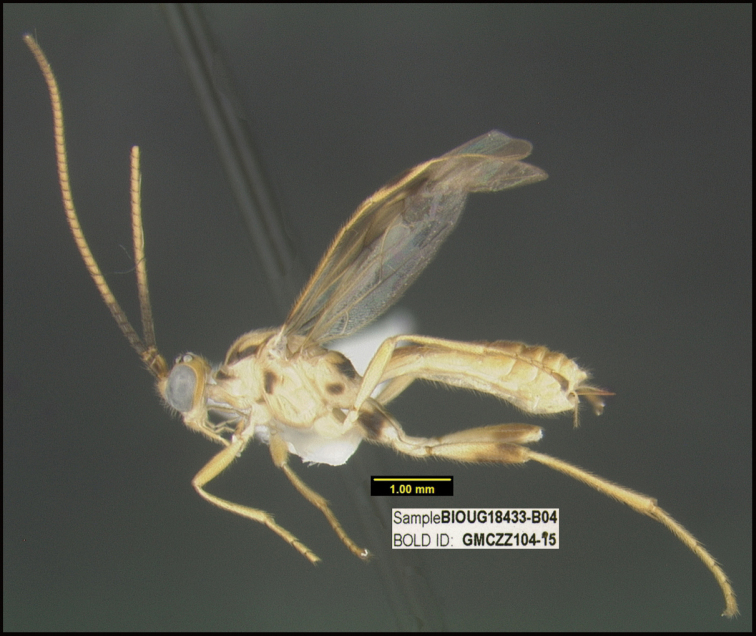
*Triraphisdavidwahli*, holotype.

##### Triraphis defectus

Taxon classificationAnimaliaHymenopteraBraconidae

Valerio, 2015

Figure 447

###### Diagnostics.

BOLD:AAA5374. Consensus barcode. TGTATTGTAT--------TTTTTATTTGGAATTTGATCAGGTATAGTTGGGYTATCTATAAGTTTAATTATTCGRTTAGAATTAAGAATACCTGGGAGTTTATTGGGTAATGATCAAATTTATAATGGTATAGTGACTGCTCATGCCTTTATTATAATTTTTTTTATRGTTATGCCTATTATAATTGGTGGATTTGGAAATTGATTAATTCCTTTAATG--TTAGGAGCTCCTG---ATATAGCTTTYCCTCGTATAAATAATATAAGATTTTGATTATTAATTCCTTCATTAACTTTATTAATTTTAAGGGCYGTAGTTAATGTTGGGGTAGGGACAGGATGAACTTTATATCCWCCTTTATCTTCTTTAGTTGGACATGGKGGTATATCTGTTGATATAGCTATTTTTTCTTTACATTTGGCAGGGGCYTCTTCAATTATAGGGGTTGTTAAYTTTATTTCTACTATTTTTAATATAAAATTAGTATCAATTAATTTAG------------ATCAAATTAATTTATTTGTTTGATCA-GTATTAATTACTGCTGTTTTATTATTATTATCT---TTACCAGTA---TTGGCRGGGGCTATTACTATATTGTTAACAGATCGTAATTTAAACACAACATTTTTTGATTTTTCTGGTGGTGGGGATCCTATTTTATTTCAACATTTATTT.

###### Specimens examined ♀.

Guanacaste, Sector Santa Rosa, Bosque San Emilio, 10.8438, -85.6138, 300 meters, dry forest, 16/vii/2012, Malaise trap. DHJPAR0021194, DHJPAR0029046, BIOUG05279-H01. Depository: CNC.

***Host data***. Valerio and Shaw (2015) recorded this species as a parasitoid of *Parasawellesca* and *Vipsaniarosabella*, the data for which they mined from the ACG inventory database. Here we record the same two hosts.

***Imaged specimen voucher code***. BIOUG05386-B06.

###### Other material.

A specimen from Jalisco, Mexico (BIOUG19130-H10) is in the same BIN and is likely conspecific with the Costa Rican specimens.

**Figure 447.**
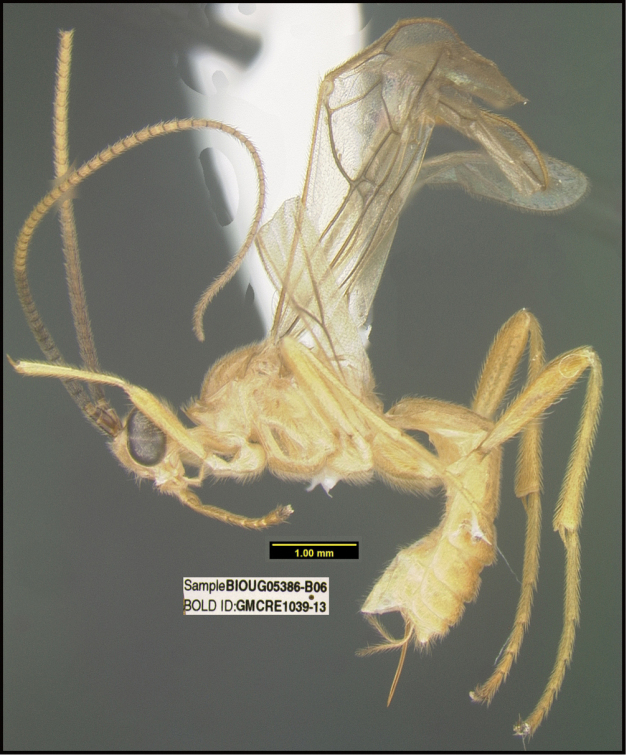
*Triraphisdefectus*.

##### Triraphis federicomatarritai

Taxon classificationAnimaliaHymenopteraBraconidae

Sharkey sp. nov.

http://zoobank.org/52F6EF0F-8B12-47F5-8DB4-8AF9391D997A

Figure 448

###### Diagnostics.

BOLD:ACX5348. Consensus barcode. GTATTATATTTTTTATTTGGAATTTGGGCGGGTATAGTTGGATTATCAATAAGTTTAATTATTCGTTTAGAATTAAGTATGCCGGGTAGTTTATTGGGGAATGATCAAATTTATAATGGCATGGTAACTGCTCATGCTTTTATTATAATTTTTTTTATGGTTATACCCATTATAATTGGGGGATTTGGGAATTGATTGATTCCCTTAATGTTGGGGGCCCCCGACATAGCTTTCCCTCGTATAAATAATATGAGATTTTGGTTATTAATTCCCTCATTAACGTTATTAATTTTAAGGGCTGTGGTTAATGTCGGGGTAGGAACAGGGTGAACTTTATATCCCCCCCTATCTTCCCTAGTTGGTCATGGGGGTATATCTGTGGATATGGCTATTTTTTCTTTACATTTAGCTGGGGCTTCTTCAATTATAGGAGTTGTAAATTTTATTTCTACTATTTTTAATATAAAATTAGTATCAATTAATTTAGATCAAATTAATTTATTTGTTTGATCAGTATTAATTACTGCTGTTTTATTATTATTATCTTTGCCTGTATTAGCTGGGGCAATTACCATATTATTAACAGATCGT---------------------------------------------.

###### Holotype ♀.

Guanacaste, Sector San Cristobal, Estación San Gerardo, 10.8801, -85.389, 575 meters, rain forest/laguna, 23/ix/2013, Malaise trap. Depository: CNC.

***Host data*.** None.

***Holotype voucher code*.**BIOUG19932-A06.

###### Paratypes.

None.

###### Etymology.

*Triraphisfedericomatarritai* is named in honor of Federico Matarrita’s long-appreciated contributions to publicity for ACG, GDFCF, and now, BioAlfa.

**Figure 448.**
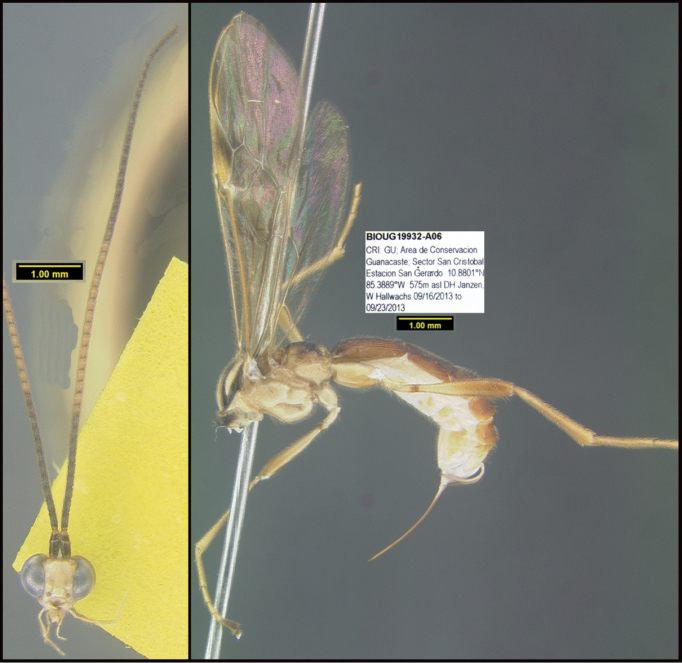
*Triraphisfedericomatarritai*, holotype.

##### Triraphis ferrisjabri

Taxon classificationAnimaliaHymenopteraBraconidae

Sharkey sp. nov.

http://zoobank.org/24920925-DA3D-4C8A-A0A6-E560F70984B3

Figure 449

###### Diagnostics.

BOLD:ADA2501. Consensus barcode. GTTTTATATTTTTTATTCGGGATTTGAGCGGGTATGATTGGGCTATCTATAAGTATAATTATTCGGTTAGAATTAAGTATACCGGGCAGATTTTTGGGGAATGATCAAATTTATAATGGCATAGTGACTGCACATGCTTTTATTATAATCTTTTTTATAGTTATGCCCATTATAATTGGCGGTTTTGGAAATTGATTAATTCCCCTAATGTTAGGGGCTCCCGATATAGCTTTCCCTCGTATAAATAATATGAGATTTTGATTATTAATTCCCTCTTTGACGTTGCTTATTTTAAGGGCAGTAGTTAATGTTGGGGTAGGTACTGGCTGAACTTTATACCCCCCTTTATCTTCATTAGTTGGTCATGGGGGCATATCTGTAGATATAGCAATTTTTTCTTTACATCTAGCTGGGGCCTCTTCTATTATAGGGGTTGTTAATTTTATTTCTACTATTTTTAATATAAAATTAGTATCAATTAATTTAGATCAAATTAATTTATTTGTTTGATCTGTATTAATTACAGCTGTTTTATTATTATTATCTTTGCCGGTATTAGCTGGTGCCATCACTATATTATTGACA------------------------------------------------.

###### Holotype ♀.

Guanacaste, Sector San Cristobal, Estación San Gerardo, 10.8801, -85.389, 575 meters, rain forest/laguna, 8/ix/2014, Malaise trap. Depository: CNC.

***Host data*.** None.

***Holotype voucher code*.**BIOUG27980-B02.

###### Paratypes.

None.

###### Etymology.

*Triraphisferrisjabri* is named in honor of Ferris Jabr’s long-appreciated contributions to publicity for ACG, GDFCF, and now, BioAlfa.

**Figure 449.**
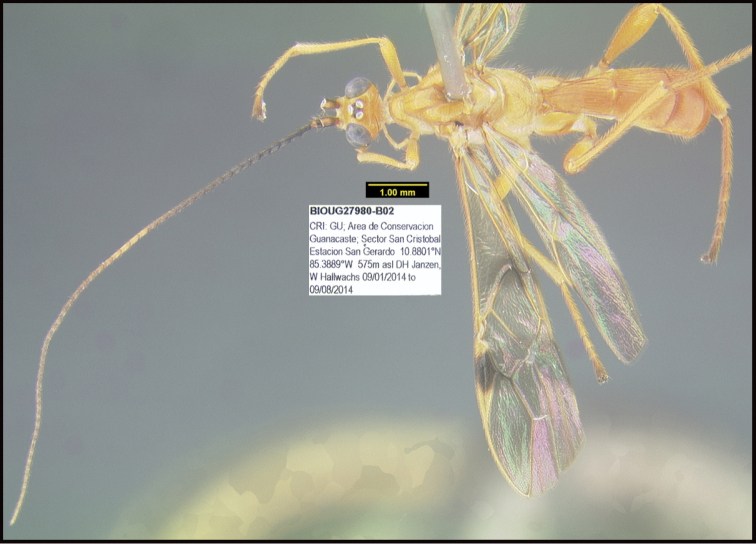
*Triraphisferrisjabri*, holotype.

##### Triraphis mariobozai

Taxon classificationAnimaliaHymenopteraBraconidae

Sharkey sp. nov.

http://zoobank.org/D42FE599-1E2F-4237-9064-5C6451945EF0

Figure 450

###### Diagnostics.

BOLD:AAB1652. Consensus barcode. GTTTTATATTTTTTATTTGGAATTTGAGCTGGTATAGTTGGGTTATCTATAARATTAATTATTCGATTAGAATTAAGTATACCTGGTAGATTATTGGGGAATGATCAAATTTATAATGGTATGGTTACTGCTCATGCTTTTATTATAATTTTTTTTATAGTTATACCTATTATAATTGGTGGTTTTGGAAATTGATTAATTCCTTTAATATTGGGGGCTCCCGATATAGCTTTTCCTCGTATAAATAATATGAGGTTTTGATTATTAATTCCTTCATTGACATTATTAATTCTAAGGGCTGTAGTTAATGTTGGGGTAGGGACTGGATGAACTTTATATCCACCTTTATCTTCTTTAGTTGGTCATGGTGGGATATCTGTGGATATAGCTATTTTTTCTTTACATTTAGCTGGGGCTTCTTCTATTATGGGGGTTGTTAATTTTATTTCTACTATTTTTAATATAAAATTAGTATCAATTAATTTAGATCAAATTAATTTATTTGTTTGATCAGTATTAATTACAGCTGTTTTATTATTATTATCTTTACCAGTATTAGCTGGTGCAATTACTATATTATTGACAGATCGTAATTTAAATACAACATTTTTTGATTTTTCTGGGGGGGGTGACCCTATTTTATTTCAACATTTATTT.

###### Holotype ♀.

Guanacaste, Sector Santa Elena, Casa Potrero Grande, 10.84918, -85.77315, 17 meters, caterpillar collection date: 29/xii/2005, wasp eclosion date: 23/i/2005. Depository: CNC.

***Host data*.***Megalopyge* Janzen06 (Megalopygidae) feeding on *Ingavera* (Fabaceae).

***Host caterpillar and holotype wasp voucher codes*.** 05-SRNP-64341, DHJPAR0021178.

###### Paratypes.

All with host data the same as holotype: DHJPAR0021189, DHJPAR0021172, DHJPAR0021173, DHJPAR0021174, DHJPAR0021175, DHJPAR0021176, DHJPAR0021177, DHJPAR0021179, DHJPAR0021186, DHJPAR0028756. Depository: CNC.

###### Etymology.

*Triraphismariobozai* is named in honor of Mario Boza’s long-appreciated contributions to publicity for ACG, GDFCF, and now, BioAlfa.

**Figure 450.**
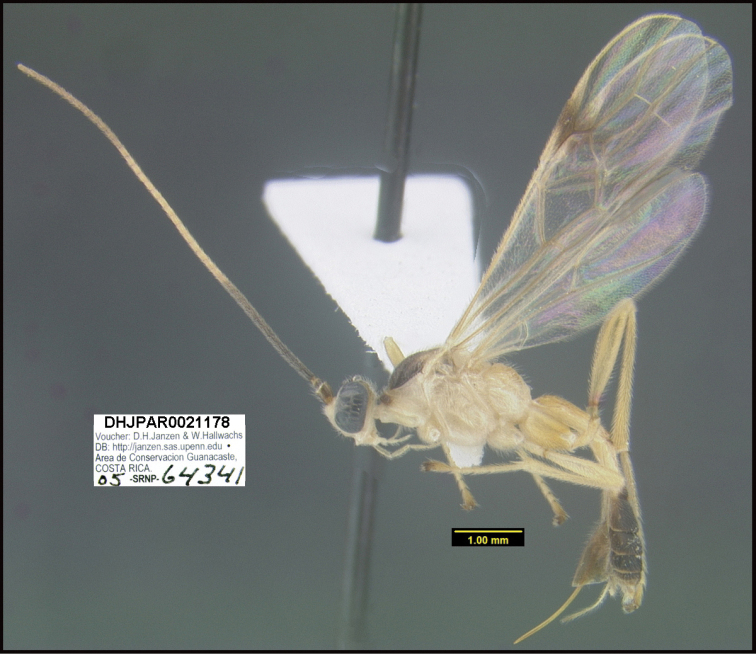
*Triraphismariobozai*, holotype.

##### Triraphis martindohrni

Taxon classificationAnimaliaHymenopteraBraconidae

Sharkey sp. nov.

http://zoobank.org/FECB9477-D08E-4F10-AE08-00F82871831E

Figure 451

###### Diagnostics.

BOLD:ABZ7672. Consensus barcode. TGTTTTATATTTTTTATTTGGAATTTGAGCAGGAATAGTAGGTTTATCAATGAGRTTAATTATTCGATTAGARTTAAGTATACCAGGAAGTTTATTAGGTAATGATCAAATTTATAATGGTATAGTAACAGCTCATGCTTTTATTATAATTTTTTTTATAGTTATACCTATTATAATTGGGGGATTTGGTAATTGGTTRATTCCTTTAATATTAGGGGCTCCTGATATAGCTTTYCCTCGTATAAATAATATAAGTTTTTGATTATTAATTCCTTCATTAACTTTGTTAATTTTAAGAGCTGTAGTTAATGTTGGGGTTGGTACTGGTTGAACTATATATCCTCCYTTATCTTCTTTAGTTGGTCATGGGGGGATATCTGTTGATATAGCTATTTTTTCTTTACATTTGGCGGGTGCATCATCTATTATAGGTGTAGTTAATTTTATTTCTACTATTTTTAATATAAAATTAATTTCTATTARATTGGAYCAAATTAATTTATTTGTTTGATCAGTTTTAATTACAGCTGTATTATTATTATTATCTTTACCTGTTTTAGCTGGTGCAATTACTATATTACTTACTGATCGTAATTTAAATACAACTTTTTTTGATTTTTCTGGTGGTGGGGATCCTATTTTATTTCAACATTTATTT.

###### Holotype ♀.

Guanacaste, Sector Del Oro, Bosque Aguirre, 11.004, -85.441, 571 meters, caterpillar collection date: 20/i/2011, wasp eclosion date: 5/ii/2011. Depository: CNC.

***Host data*.***Mesosemiagrandis* (Riodinidae) feeding on *Psychotrialamarinensis* (Rubiaceae).

***Host caterpillar and holotype wasp voucher codes*.** 05-SRNP-64341, DHJPAR0042388.

###### Paratypes.

None.

###### Other material.

A specimen from French Guiana (CCDB-07376 H09) is in the same BIN. The image on BOLD shows an almost entirely straw-colored specimen, suggesting that it is probably not conspecific with *T.martindohrni*.

###### Etymology.

*Triraphismartindohrni* is named in honor of Martin Dohrn’s long-appreciated contributions to publicity for ACG, GDFCF, and now, BioAlfa.

**Figure 451.**
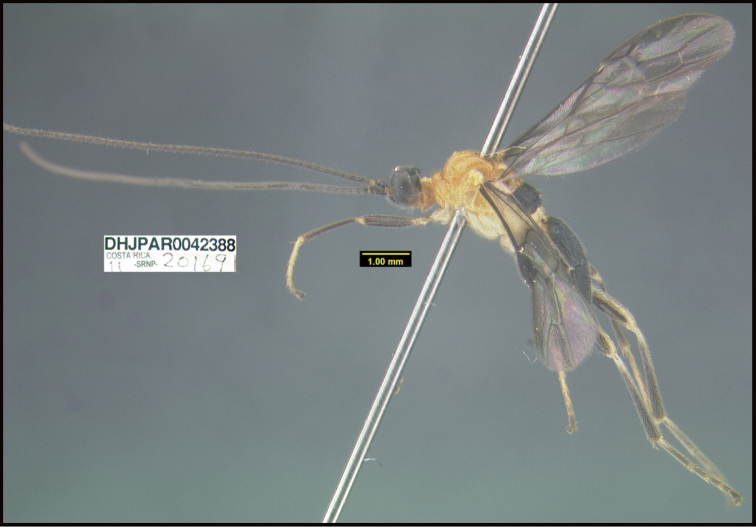
*Triraphismartindohrni*, holotype.

##### Triraphis matssegnestami

Taxon classificationAnimaliaHymenopteraBraconidae

Sharkey sp. nov.

http://zoobank.org/EBC7CB0B-A6BC-4652-9EC0-E68E627D6428

Figure 452

###### Diagnostics.

BOLD:ABU7454. Consensus barcode. TGTATTGTATTTTTTATTTGGTATTTGGGCGGGTATGGTTGGTTTATCTATAAGATTAATTATTCGATTAGAATTAAGAATGCCGGGTAGTTTATTGGGTAATGATCAAATTTATAATGGTATAGTTACTGCTCATGCTTTTATTATAATTTTTTTTATAGTTATACCTATTATAATTGGGGGGTTTGGAAATTGATTAATTCCATTAATGTTAGGGGCTCCTGATATGGCTTTTCCACGTATAAATAATATAAGATTTTGGTTATTAATTCCTTCATTAACTTTATTAATTTTAAGTGCTGTAGTTAATGTAGGAGTTGGAACAGGATGAACTATATACCCTCCATTATCTTCTTTAGTTGGTCATGGGGGTATATCAGTTGATATGGCTATTTTTTCTTTACATTTGGCGGGGGCTTCATCTATTATAGGTGTTGTTAATTTTATTTCTACTATTTTTAATATAAAATTGGTATCTATTAATTTAGATCAAATTAATTTATTTGTTTGATCTGTTTTAATTACTGCTGTTTTACTATTATTGTCTTTACCGGTTTTGGCTGGTGCTATTACTATATTATTAACTGATCGTAATTTAAATACAACATTTTTTGATTTTTCAGGTGGGGGGGATCCTATTTTATTTCAACATTTATTT.

###### Holotype ♀.

Guanacaste, Sector Orosi, Quebrada Las Yeguitas, 10.96160, -85.49585, 560 meters, caterpillar collection date: 28/ix/2011, wasp eclosion date: 9/x/2011. Depository: CNC.

***Host data*.***Venadicodiacaneti* (Limacodidae) feeding on *Mespilodaphneveraguensis* (Lauraceae).

***Host caterpillar and holotype wasp voucher codes*.** 05-SRNP-64341, DHJPAR0045708.

###### Paratypes.

None.

###### Etymology.

*Triraphismatssegnestami* is named in honor of Mats Segnestam’s long-appreciated contributions to publicity for ACG, GDFCF, and now, BioAlfa.

**Figure 452.**
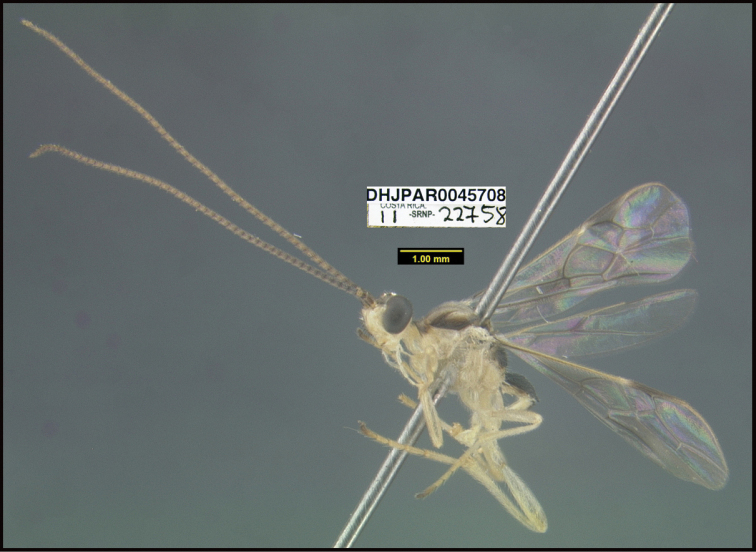
*Triraphismatssegnestami*, holotype.

##### Triraphis mehrdadhajibabaei

Taxon classificationAnimaliaHymenopteraBraconidae

Sharkey sp. nov.

http://zoobank.org/4EEF24DF-0E20-451A-A946-0E9559414E4E

Figure 453

###### Diagnostics.

BOLD:AAJ2715. Consensus barcode. TGTTTTATATTTTTTATTTGGTATTTGAGCTGGTATAGTAGGATTGTCAATAAGATTAATTATTCGGTTAGAATTAAGAATGCCGGGAAGTTTATTAGGTAATGATCAAATTTATAATGGTATAGTAACAGCCCATGCATTTATTATAATTTTTTTTATAGTTATACCTATTATAATTGGGGGGTTTGGTAATTGATTAATTCCTTTAATATTAGGGGCACCTGATATAGCTTTCCCACGTATAAATAATATAAGATTTTGATTATTAATTCCTTCATTAACTTTATTAATTTTAAGTGCGGTAGTAAATGTTGGGGTAGGAACTGGTTGGACTATGTATCCTCCTTTATCTTCTTTAGTGGGTCATGGRGGGATATCTGTTGATATAGCAATTTTTTCTTTACATTTAGCAGGYGCATCTTCTATTATAGGGGTAGTTAATTTTATTTCTACTATTTATAATATAAAATTAGTTTCTATTAGGTTAGATCAAATTAATTTATTTGTTTGATCTGTTTTAATTACTGCAGTATTATTATTATTATCTTTACCAGTTTTRGCTGGRGCTATTACTATATTACTTACTGATCGTAATTTAAATACAACTTTTTTTGATTTTTCWGGCGGGGGRRRMCCTATTTTATTCCAACATTTATTT.

###### Holotype ♀.

Guanacaste, Sector Pitilla, Bullas, 10.98670, -85.38503, 440 meters, caterpillar collection date: 15/iv/2011, wasp eclosion date: 30/iv/2011. Depository: CNC.

***Host data*.***Ancylurisinca* (Riodinidae) feeding on *Miconiaargentea* (Melastomataceae).

***Host caterpillar and holotype wasp voucher codes*.** 11-SRNP-65193, DHJPAR0042798.

###### Paratypes.

None.

###### Etymology.

*Triraphismehrdadhajibabaei* is named in honor of Mehrdad Hajibabaei’s long-appreciated contributions to publicity for ACG, GDFCF, and now, BioAlfa.

**Figure 453.**
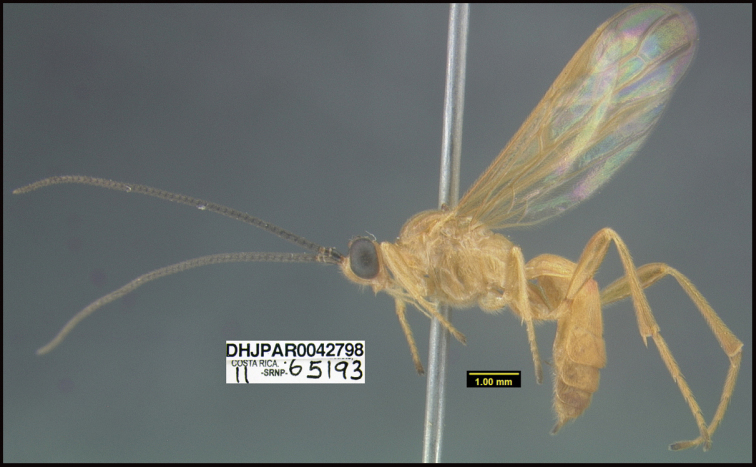
*Triraphismehrdadhajibabaei* holotype.

##### Triraphis ollieflinti

Taxon classificationAnimaliaHymenopteraBraconidae

Sharkey sp. nov.

http://zoobank.org/DA1CFCB1-358A-4BCF-B1B5-0409FBB68186

Figure 454

###### Diagnostics.

BOLD:ABU5849. Consensus barcode. TGTTTTATATTTTTTATTTGGTATTTGAGCTGGTATAGTGGGTTTATCAATAAGGTTGATTATTCGTTTAGAATTAAGAATACCAGGTAGTTTATTAGGTAATGATCAAATTTATAATGGGATAGTAACGGCTCATGCTTTTATTATAATTTTCTTTATAGTTATACCTATTATAATTGGGGGATTTGGTAATTGATTAATTCCCTTAATATTGGGGGCACCTGATATAGCTTTTCCACGTATAAATAATATAAGTTTTTGATTATTAATTCCTTCATTAACTTTATTAATTTTAAGTGCTGTAGTAAATGTTGGGGTAGGGACTGGGTGAACTATTTATCCTCCTTTATCTTCTTTAGTTGGTCATGGAGGTATATCTGTTGATATAGCAATTTTTTCTTTACATTTAGCAGGGGCATCTTCTATTATAGGAGTAGTTAATTTTATTTCTACTATTTTTAATATAAAATTAATTTCTATTAGTTTAGATCAAATTAATTTATTCGTTTGATCTGTTTTAATTACAGCAGTATTATTATTATTATCTTTACCTGTTTTAGCTGGGGCTATTACTATATTACTTACTGATCGTAATTTGAATACAACTTTTTTTGATTTTTCTGGTGGGGGGGATCCTATTTTATTTCAACATTTATTT.

###### Holotype ♀.

Guanacaste, Sector Pitilla, Pasmompa, 11.01926, -85.40997, 440 meters, caterpillar collection date: 21/x/2010, wasp eclosion date: 9/xi/2010. Depository: CNC.

***Host data*.** ereJanzen 10-SRNP-32223 (Erebidae) feeding on algae scraped off the surface of a palm leaf.

***Host caterpillar and holotype wasp voucher codes*.** 10-SRNP-32223, DHJPAR0041604.

###### Paratypes.

None.

###### Etymology.

*Triraphisollieflinti* is named in honor of Oliver Flint’s (RIP) long-appreciated contributions to publicity for ACG and GDFCF.

**Figure 454.**
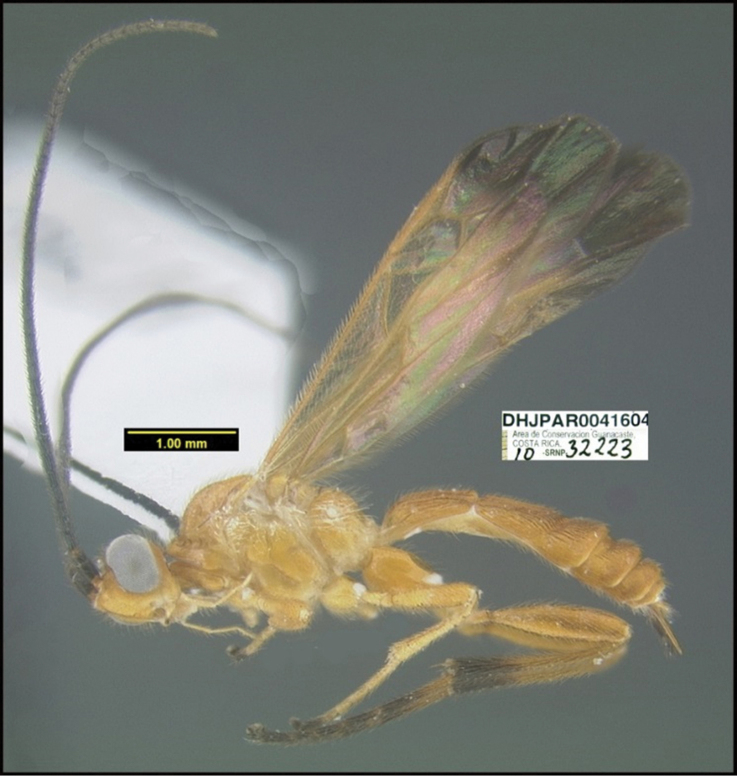
*Triraphisollieflinti*, holotype.

##### Triraphis tildalauerae

Taxon classificationAnimaliaHymenopteraBraconidae

Sharkey sp. nov.

http://zoobank.org/D624C0BE-8E92-44BE-894A-FC5487D4959F

Figure 455

###### Diagnostics.

BOLD:ADB6156. Consensus barcode. GTGTTATATTTTTTGTTTGGTATTTGAGCGGGTATGTTGGGGTTATCAATAAGTTTAATTATTCGATTAGAGTTGAGAATGCCTGGGAGATTATTAGGTAATGATCAAATTTATAATGGTATAGTTACAGGGCATGCTTTTATTATAATTTTTTTTATAGTTATACCTATTATAATTGGTGGTTTTGGAAATTGATTAATTCCTTTGATGTTAGGGGCCCCTGATATAGCTTTTCCTCGTATAAATAATATGAGATTTTGATTATTAATTCCTTCGTTAATTTTATTAATTTTAAGGGCTGTAGTTAATGTTGGGGTTGGGACTGGGTGGACTATATATCCTCCTTTATCTTCTTTAGTTGGTCATGGAGGGATATCTGTTGATATGGCGATTTTTTCTTTACATTTAGCTGGGGCATCTTCTATTATAGGGGTAATTAATTTTATTTCTACTATTTTTAATATAAAATTAATTTCTATTAAATTGGATCAAGTTAATTTATTTGTGTGATCAGTTTTAATTACGGCTATTTTATTATTATTATCATTGCCTGTATTAGCTGGGGCTATTACTATATTACTTACT------------------------------------------------.

###### Holotype ♀.

Guanacaste, Pailas Dos, PL12-3, 10.7631, -85.3344, 820 meters, 09/i/2014, Malaise trap. Depository: CNC.

***Host data*.** None.

***Holotype voucher code*.**BIOUG29480-H10.

###### Paratypes.

None.

###### Etymology.

*Triraphistildalauerae* is named in honor of Tilda Lauer, young insect-lover and daughter of Brian Lauer.

**Figure 455.**
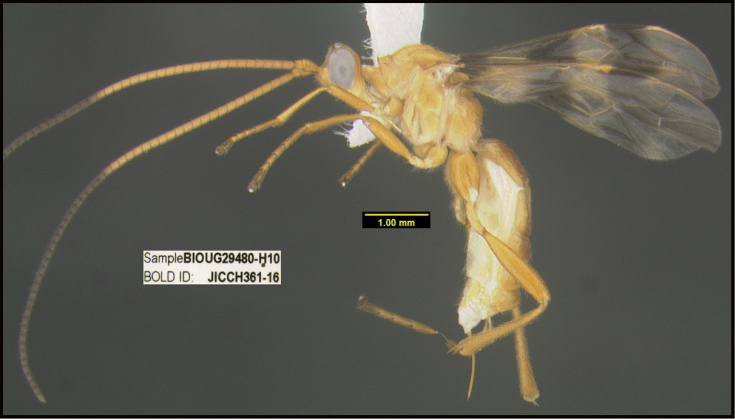
*Triraphistildalauerae*, holotype.

#### *Yelicones* Cameron, 1887

Members of *Yelicones* are found worldwide. There are 85 described species in the New World. Members are recorded as parasitoids of Pyralidae and Crambidae. Areekul and Quicke (2006) revised many New World species.

##### Yelicones dirksteinkei

Taxon classificationAnimaliaHymenopteraBraconidae

Sharkey sp. nov.

http://zoobank.org/114E667F-554F-4C45-A7E7-2C4F404A091A

Figure 456

###### Diagnostics.

BOLD:AAT9023. Consensus barcode. TTTATTATATTTTTTTTTTGGTATTTGATCAGGTTTATTAGGTTTATCGTTAAGGTTAGTTATTCGTATAGAATTGAGGAATCCTGGAAGTTTGTTAGGGAGTGATCAATTATATAATTTAATAGTAACAATTCATGCATTTATTATAATTTTTTTTATAGTTATACCTATTATAATTGGTGGATTTGGTAATTGATTAATTCCATTAATATTAGGTTCACCTGATATGGCTTTCCCTCGTATAAATAATATAAGATTTTGATTATTAATTCCTTCTTTAATATTATTATTATTAAGAGGATTTACTAATATGGGTGTAGGCACAGGTTGAACTATATATCCTCCTTTAAGATCTTTATCTGGACATCCTGGGATTTCTGTTGATATAGCTATTTTTTCTTTACATTTAGCAGGGGTATCATCTATTATAGGGGCTGTTAATTTTATTACTACTATTTTTAATATAAAATTGTATAAGTTAAAATTAGATCAATTAAGATTATTTGTATGATCTGTTTTAATTACTGCATTTTTATTATTGTTATCTTTACCTGTTTTGGCAGGTGGGATTACTATATTATTAACTGATCGTAATTTAAATACTAGGTTTTTTGATTTTGCTGGGGGAGGAGATCCAATTTTATTTCAACATTTATTT. It is distinguished from all other described New World *Yelicones* by the uniquely colored mesoscutum and scutellum, which are piceous-black laterally and pale yellow-cream medially. In the key by Areekul and Quicke (2006) it keys out to couplet 22, but neither couplet matches it.

###### Holotype ♂.

Alajuela, Sector Rincon Rain Forest, Rio Francia Arriba, 10.89666, -85.29003, 400 meters, caterpillar collection date: 16/v/2012, wasp eclosion date: 18/vi/2012. Depository: CNC.

***Host data*.** pyrabiolep01 biolep29 (Pyralidae) feeding on *Crotonbillbergianus* (Euphorbiaceae).

***Host caterpillar and holotype wasp voucher codes*.** 12-SRNP-42090, DHJPAR0049352.

###### Paratypes.

Host = phyBioLep01 BioLep757 (Pyralidae): DHJPAR0049307, DHJPAR0049352. Depository: CNC.

###### Etymology.

*Yeliconesdirksteinkei* is named in honor of Dirk Steinke’s long-appreciated contributions to publicity for ACG, GDFCF, and now, BioAlfa.

**Figure 456.**
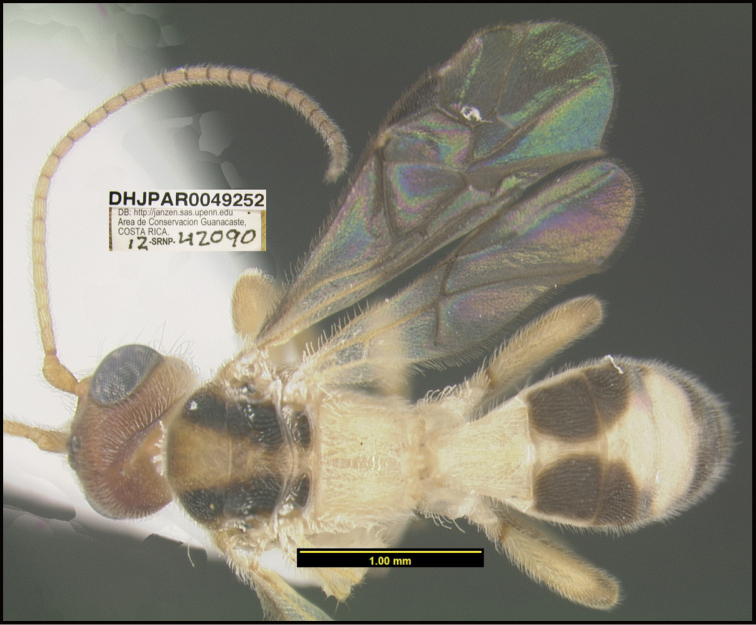
*Yeliconesdirksteinkei*, holotype.

##### Yelicones markmetzi

Taxon classificationAnimaliaHymenopteraBraconidae

Sharkey sp. nov.

http://zoobank.org/53BDA602-C3EC-4E5C-8DA5-99D3949EA09C

Figure 457

###### Diagnostics.

BOLD:ADD0488. Consensus barcode. GGATTAGTAGGATTATCTTTAAGAGTGATTATTCGATTAGAATTAAGTAATTTGGGGAGGTTACTAGGAAGAGACCAGATTTATAATGTAGTAGTAACTATACATGCCTTTATTATAATTTTTTTTATAGTGATACCAATTATAATAGGAGGGTTTGGGAATTGGTTAGTTCCATTAATGATAGGGGGGCCAGATATAGCTTTTCCACGGATGAATAATATGAGGTTTTGGTTATTATTACCCTCATTTTTTTTGATAATATTAAGAGGATTTATAAATGTAGGGGGAGGAACGGGGTGGACTATTTACCCTCCTTTAAGGTCTTTAATAGGGCATATAGGAGTTTCTGTTGATATAATAATTTTTTCTTTGCATTTAGCAGGGGTTCTTCTATTATAGGGGCTGTAAATTTTATTACTACTATTTTTAATATAAATTTATTTTTACAGATGGATCAAATTGTATTATTTTCATGGTCGGTATTAATTACTGCTTTTTTATTATTAATATCTTTGCCAGTTTTAGCAGGAGGGATTACGATGTTATTAACAGATCGAAATTTAAATACTAGGTTTTTTGATTATTCGGGGGGGGGG. Keys to couplet 40 in Areekul and Quicke (2006) where it fails because the forewing is yellow basally and dark apically, without a transverse band below the stigma, which is uniformly brown rather than bicolored or dark yellow.

###### Holotype ♂.

Guanacaste, Sector Mundo Nuevo, Vado Agria, 10.75876, -85.37543, 560 meters, caterpillar collection date: 7/vii/2008, wasp eclosion date: 25/viii/2008. Depository: CNC.

***Host data*.***Accinctapubesalbifasciata* (Pyralidae) feeding on *Damburneyasalicifolia* (Lauraceae).

***Host caterpillar and holotype wasp voucher codes*.** 08-SRNP-56572, DHJPAR0028135.

###### Paratypes.

None.

###### Etymology.

*Yeliconesmarkmetzi* is named in recognition of Mark Metz’s contribution to the inventory of ACG through the identification of Gelechioidea.

**Figure 457.**
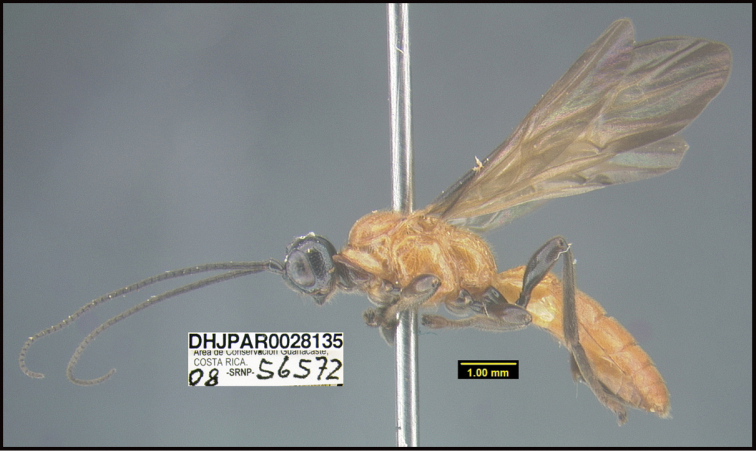
*Yeliconesmarkmetzi*, holotype.

##### Yelicones monserrathvargasae

Taxon classificationAnimaliaHymenopteraBraconidae

Sharkey sp. nov.

http://zoobank.org/47373731-6421-4481-A813-E5C6ED042DDE

Figure 458

###### Diagnostics.

BOLD:ADQ1035. Consensus barcode. TTTATTATACTTTATTTTTGGTATTTGATCAGGTTTATTAGGATTATCTTTTAGTATAATTATTCGGTTAGAATTAAATAACCCTGGGAGTTTATTAGGTAATGATCAAATTTATAATTTAATGGTTACTATTCATGCTTTTATTATGATTTTTTTTATAGTTATACCAATTATAATTGGAGGATTTGGGAATTGATTGATCCCTTTAATATTAGGGGCTCCTGATATAGCATTTCCTCGAATAAATAATATAAGATTTTGGTTATTGATTCCTTCTTTATTTTTGATAATATTGAGTGGTTTTATAAATGTAGGGGTAGGAACAGGTTGAACAATATATCCTCCTTTAAGATCTTTAATAGGTCATAGAGGATTTTCTGTAGATTTTATAATTTTTTCTTTACATTTAGCGGGGTTTTCTTCAATCATGGGTGCTATTAATTTTATTACTACTATTTTTAATATAAAATTAATTTTTATAAAATTAGATCAAATAAGTTTATTTATTTGATCTGTTCTAATTACAGCTTTTTTATTATTATTATCATTACCTGTATTAGCAGGTGGAATTACTATATTATTAACTGATCGAAATTTAAATACTAGTTTTTTTGATTTTTCTGGGGGGGGGGATCCTATTTTGTTTCAACATTTATTT. This species keys to couplet 69 in Areekul and Quicke (2006) where it fails because the head and face (including clypeus) are entirely black.

###### Holotype ♂.

Guanacaste, Sector Pitilla, Sendero Memos, 10.9817, -85.4278, 740 meters, caterpillar collection date: 10/i/2018, wasp eclosion date: 13/iii/2018. Depository: CNC.

***Host data*.***Monoloxis* flavicintalisDHJ02 (Pyralidae) feeding on *Lacistemaaggregatum* (Lacistemataceae).

***Host caterpillar and holotype wasp voucher codes*.** 18-SRNP-30118, DHJPAR0062960.

###### Paratypes.

None.

###### Etymology.

*Yeliconesmonserrathvargasae* is named in honor of Monserrath Vargas’ long-appreciated contributions to publicity for ACG, GDFCF, and now, BioAlfa.

**Figure 458.**
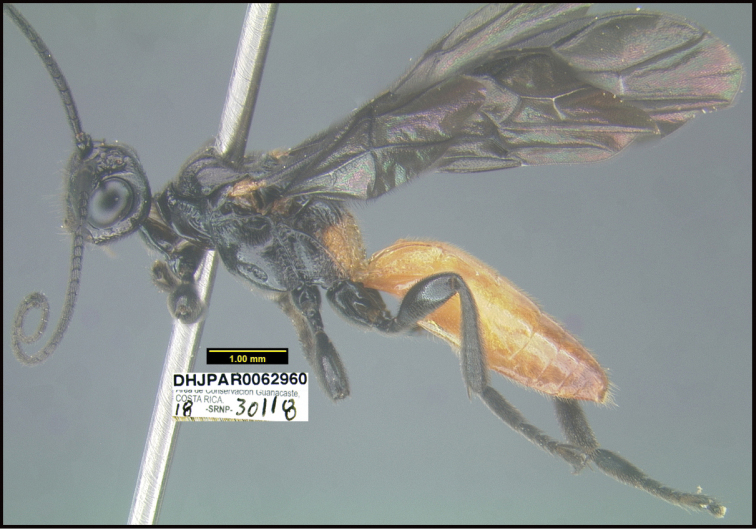
*Yeliconesmonserrathvargasae*, holotype.

##### Yelicones tricolor

Taxon classificationAnimaliaHymenopteraBraconidae

Quicke, Chishti & Basibuyuk, 1996

Figure 459

###### Diagnostics.

BOLD:ACB2775. Consensus barcode. ATTATTATATTTTATTTTTGGTATTTGATCTGGATTATTAGGTTTATCTTTTAGGATAATTATTCGTATAGAATTAAATAATCCTGGTAGGTTATTAGGTAATGATCAAATTTATAATTTAATAGTGACAATTCATGCTTTTATTATAATTTTTTTTATGGTAATACCTATTATAATTGGTGGATTTGGAAATTGATTAATTCCTTTAATATTAGGAGCTCCTGATATAGCTTTYCCTCGTATAAATAATATGAGGTTTTGATTATTAATTCCTTCTTTATTTTTAATAATATTAAGAGGATTTAATAATGTAGGGGTAGGTACTGGTTGGACTATATATCCACCTCTTAGATCATTGGTAGGTCATAGAGGATTTTCTGTTGATTTTATAATTTTTTCATTACATTTAGCAGGAGTTTCTTCTATTATAGGGGCTATTAATTTTATTACTACAATTTTTAATATAAAATTAATATTTATTAAATTGGATCAAATAAGATTATTTATTTGGTCTGTTTTAATCACAGCTATTTTATTATTATTATCTTTACCTGTATTAGCAGGTGGTATCACAATATTATTAACGGATCGAAATTTAAATACTAGATTTTTTGATTTTTCAGGAGGGGGAGATCCAGTTTTATTTCAACATTTATTT.

###### Material examined.

♀: Alajuela, Sector Rincon Rain Forest, Sendero Anonas, 10.90528, -85.27882, 405 meters, caterpillar collection date: 01/iii/2012, wasp eclosion date: 06/iv/2012. Depository: CNC.

***Host data*.** chryJanzen01 Janzen165 (Pyralidae) feeding on Piper21150 (Piperaceae).

***Host caterpillar and holotype wasp voucher codes*.** 12-SRNP-40868, DHJPAR0049366.

###### Other material.

DHJPAR0049360.

**Figure 459.**
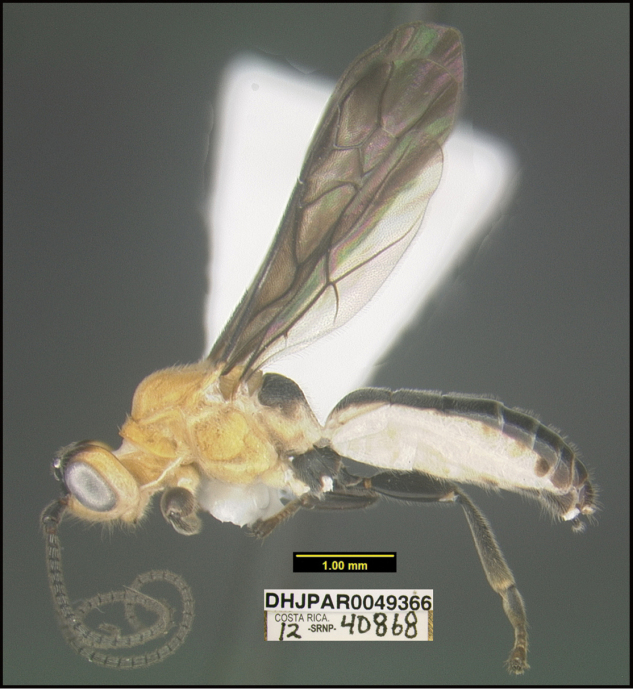
*Yeliconestricolor*.

##### Yelicones woldai

Taxon classificationAnimaliaHymenopteraBraconidae

Quicke, Chishti & Basibuyuk, 1996

Figure 460

###### Diagnostics.

BOLD:ACR8199. Consensus barcode. TGTTTTATATTTTTTGTTTGGTATTTGATGTGGGTTATTAGGATTATCTTTAAGAATTATTATTCGATTAGAGTTAAGGAATTTAGGGAGATTATTGGGAAATGATCAAATTTATAATGTAATTGTTACGATACATGCTTTTGTAATAATTTTTTTTATAGTAATACCTATTATAATTGGGGGGTTTGGAAATTGATTGATTCCTTTAATATTAGGGTGTCCTGATATAGCCTTCCCTCGTATAAATAATATAAGATTTTGGTTATTGATTCCTTCTTTAATTTTAATGATTATAAGAGGGTTTATTATAGTAGGGAGAGGAACGGGTTGGACAATATATCCTCCTTTAAGTTCTTTAATTGGTCATGGAGGGTTTTCAGTTGATATAGTTATTTTTTCTTTACATTTAGCAGGGGTATCTTCCATTATAGGAGCTATTAATTTTATTACAACAATTTTTAATATAAAATTAATGTTAAAATTGGATCAAGTTATATTGTTTGTATGATCTGTTTTGATTACTGCTTTTTTATTATTATTATCTTTGCCTGTGTTAGCAGGAGGAATTACTATATTATTAACAGATCGTAATTTAAATACTTCTTTTTTTGATTTTTCAGGGGGAGGAGATCCTATTTTATTTCAGCATTTATTT.

###### Illustrated specimen ♀.

Alajuela, Sector San Cristobal, Puente Palma, 10.9163, -85.37869, 460 meters, caterpillar collection date: 11/ix/2011, wasp eclosion date: 21/x/2011. Depository: CNC.

***Host data*.***Chloropaschiamennusalis* (Pyralidae) feeding on *Clusiacylindrica* (Clusiaceae).

***Host caterpillar and holotype wasp voucher codes*.** 11-SRNP-3557, DHJPAR0045811.

###### Other material.

2♀♀: Hosts = Same as above and *Chloropaschiapegalis*. DHJPAR0055816, DHJPAR0039359. Depository: CNC.

**Figure 460.**
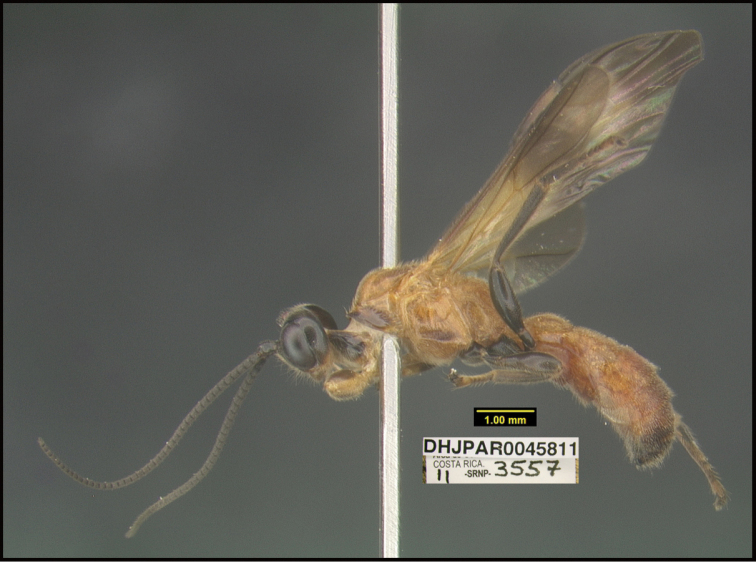
*Yeliconeswoldai*.

## Supplementary materials

Supplementary material 1

Agathidinae

Data type molecular data https://binary.pensoft.net/file/504024This dataset is made available under the Open Database License (http://opendatacommons.org/licenses/odbl/1.0/). The Open Database License (ODbL) is a license agreement intended to allow users to freely share, modify, and use this Dataset while maintaining this same freedom for others, provided that the original source and author(s) are credited.Michael J. Sharkey, Daniel H. Janzen, Winnie Hallwachs, Eric G. Chapman, M. Alex Smith, Tanya Dapkey, Allison Brown, Sujeevan Ratnasingham, Suresh Naik, Ramya Manjunath, Kate Perez, Megan Milton, Paul Hebert, Scott R. Shaw, Rebecca N. Kittel, M. Alma Solis, Mark A. Metz, Paul Z. Goldstein, John W. Brown, Donald L.J. Quicke, C. van Achterberg, Brian V. Brown, John M. Burns

Supplementary material 2

Braconinae

Data type molecular data https://binary.pensoft.net/file/504025This dataset is made available under the Open Database License (http://opendatacommons.org/licenses/odbl/1.0/). The Open Database License (ODbL) is a license agreement intended to allow users to freely share, modify, and use this Dataset while maintaining this same freedom for others, provided that the original source and author(s) are credited.Michael J. Sharkey, Daniel H. Janzen, Winnie Hallwachs, Eric G. Chapman, M. Alex Smith, Tanya Dapkey, Allison Brown, Sujeevan Ratnasingham, Suresh Naik, Ramya Manjunath, Kate Perez, Megan Milton, Paul Hebert, Scott R. Shaw, Rebecca N. Kittel, M. Alma Solis, Mark A. Metz, Paul Z. Goldstein, John W. Brown, Donald L.J. Quicke, C. van Achterberg, Brian V. Brown, John M. Burns

Supplementary material 3

Cheloninae

Data type molecular data https://binary.pensoft.net/file/504026This dataset is made available under the Open Database License (http://opendatacommons.org/licenses/odbl/1.0/). The Open Database License (ODbL) is a license agreement intended to allow users to freely share, modify, and use this Dataset while maintaining this same freedom for others, provided that the original source and author(s) are credited.Michael J. Sharkey, Daniel H. Janzen, Winnie Hallwachs, Eric G. Chapman, M. Alex Smith, Tanya Dapkey, Allison Brown, Sujeevan Ratnasingham, Suresh Naik, Ramya Manjunath, Kate Perez, Megan Milton, Paul Hebert, Scott R. Shaw, Rebecca N. Kittel, M. Alma Solis, Mark A. Metz, Paul Z. Goldstein, John W. Brown, Donald L.J. Quicke, C. van Achterberg, Brian V. Brown, John M. Burns

Supplementary material 4

Homolobinae

Data type molecular data https://binary.pensoft.net/file/504027This dataset is made available under the Open Database License (http://opendatacommons.org/licenses/odbl/1.0/). The Open Database License (ODbL) is a license agreement intended to allow users to freely share, modify, and use this Dataset while maintaining this same freedom for others, provided that the original source and author(s) are credited.Michael J. Sharkey, Daniel H. Janzen, Winnie Hallwachs, Eric G. Chapman, M. Alex Smith, Tanya Dapkey, Allison Brown, Sujeevan Ratnasingham, Suresh Naik, Ramya Manjunath, Kate Perez, Megan Milton, Paul Hebert, Scott R. Shaw, Rebecca N. Kittel, M. Alma Solis, Mark A. Metz, Paul Z. Goldstein, John W. Brown, Donald L.J. Quicke, C. van Achterberg, Brian V. Brown, John M. Burns

Supplementary material 5

Hormiinae

Data type molecular data https://binary.pensoft.net/file/504028This dataset is made available under the Open Database License (http://opendatacommons.org/licenses/odbl/1.0/). The Open Database License (ODbL) is a license agreement intended to allow users to freely share, modify, and use this Dataset while maintaining this same freedom for others, provided that the original source and author(s) are credited.Michael J. Sharkey, Daniel H. Janzen, Winnie Hallwachs, Eric G. Chapman, M. Alex Smith, Tanya Dapkey, Allison Brown, Sujeevan Ratnasingham, Suresh Naik, Ramya Manjunath, Kate Perez, Megan Milton, Paul Hebert, Scott R. Shaw, Rebecca N. Kittel, M. Alma Solis, Mark A. Metz, Paul Z. Goldstein, John W. Brown, Donald L.J. Quicke, C. van Achterberg, Brian V. Brown, John M. Burns

Supplementary material 6 Ichneutinae and Proteropinae Data type molecular data https://binary.pensoft.net/file/504029This dataset is made available under the Open Database License (http://opendatacommons.org/licenses/odbl/1.0/). The Open Database License (ODbL) is a license agreement intended to allow users to freely share, modify, and use this Dataset while maintaining this same freedom for others, provided that the original source and author(s) are credited.Michael J. Sharkey, Daniel H. Janzen, Winnie Hallwachs, Eric G. Chapman, M. Alex Smith, Tanya Dapkey, Allison Brown, Sujeevan Ratnasingham, Suresh Naik, Ramya Manjunath, Kate Perez, Megan Milton, Paul Hebert, Scott R. Shaw, Rebecca N. Kittel, M. Alma Solis, Mark A. Metz, Paul Z. Goldstein, John W. Brown, Donald L.J. Quicke, C. van Achterberg, Brian V. Brown, John M. Burns

Supplementary material 7

Macrocentrinae

Data type molecular data https://binary.pensoft.net/file/504030This dataset is made available under the Open Database License (http://opendatacommons.org/licenses/odbl/1.0/). The Open Database License (ODbL) is a license agreement intended to allow users to freely share, modify, and use this Dataset while maintaining this same freedom for others, provided that the original source and author(s) are credited.Michael J. Sharkey, Daniel H. Janzen, Winnie Hallwachs, Eric G. Chapman, M. Alex Smith, Tanya Dapkey, Allison Brown, Sujeevan Ratnasingham, Suresh Naik, Ramya Manjunath, Kate Perez, Megan Milton, Paul Hebert, Scott R. Shaw, Rebecca N. Kittel, M. Alma Solis, Mark A. Metz, Paul Z. Goldstein, John W. Brown, Donald L.J. Quicke, C. van Achterberg, Brian V. Brown, John M. Burns

Supplementary material 8

Orgilinae

Data type molecular data https://binary.pensoft.net/file/504031This dataset is made available under the Open Database License (http://opendatacommons.org/licenses/odbl/1.0/). The Open Database License (ODbL) is a license agreement intended to allow users to freely share, modify, and use this Dataset while maintaining this same freedom for others, provided that the original source and author(s) are credited.Michael J. Sharkey, Daniel H. Janzen, Winnie Hallwachs, Eric G. Chapman, M. Alex Smith, Tanya Dapkey, Allison Brown, Sujeevan Ratnasingham, Suresh Naik, Ramya Manjunath, Kate Perez, Megan Milton, Paul Hebert, Scott R. Shaw, Rebecca N. Kittel, M. Alma Solis, Mark A. Metz, Paul Z. Goldstein, John W. Brown, Donald L.J. Quicke, C. van Achterberg, Brian V. Brown, John M. Burns

Supplementary material 9

Rhysipolinae

Data type molecular data https://binary.pensoft.net/file/504032This dataset is made available under the Open Database License (http://opendatacommons.org/licenses/odbl/1.0/). The Open Database License (ODbL) is a license agreement intended to allow users to freely share, modify, and use this Dataset while maintaining this same freedom for others, provided that the original source and author(s) are credited.Michael J. Sharkey, Daniel H. Janzen, Winnie Hallwachs, Eric G. Chapman, M. Alex Smith, Tanya Dapkey, Allison Brown, Sujeevan Ratnasingham, Suresh Naik, Ramya Manjunath, Kate Perez, Megan Milton, Paul Hebert, Scott R. Shaw, Rebecca N. Kittel, M. Alma Solis, Mark A. Metz, Paul Z. Goldstein, John W. Brown, Donald L.J. Quicke, C. van Achterberg, Brian V. Brown, John M. Burns

Supplementary material 10

Rogadinae

Data type molecular data https://binary.pensoft.net/file/504033This dataset is made available under the Open Database License (http://opendatacommons.org/licenses/odbl/1.0/). The Open Database License (ODbL) is a license agreement intended to allow users to freely share, modify, and use this Dataset while maintaining this same freedom for others, provided that the original source and author(s) are credited.Michael J. Sharkey, Daniel H. Janzen, Winnie Hallwachs, Eric G. Chapman, M. Alex Smith, Tanya Dapkey, Allison Brown, Sujeevan Ratnasingham, Suresh Naik, Ramya Manjunath, Kate Perez, Megan Milton, Paul Hebert, Scott R. Shaw, Rebecca N. Kittel, M. Alma Solis, Mark A. Metz, Paul Z. Goldstein, John W. Brown, Donald L.J. Quicke, C. van Achterberg, Brian V. Brown, John M. Burns
