# Supplementary material for: Minimalist revision and description of 403 new species in 11 subfamilies of Costa Rican braconid parasitoid wasps, including host records for 219 species
Source: Zookeys. 2021 Feb 2;1013:1–665. doi: 10.3897/zookeys.1013.55600 (PMC8390796; doi:10.3897/zookeys.1013.55600)
Supplement: Supplementary material 1 — Agathidinae [file zookeys-1013-001-s001.pdf]

1. Agathidinae BOLD TaxonID Tree

Title : Tree Result - Search: Sample IDs (2130 records returned) (2130 records selected)
Date : 23-Nov-2020
Data Type : Nucleotide
Distance Model : Kimura 2 Parameter
Marker : COI-5P
Colourization : [blue]=Stop Codons [red]=Contamination or misidentification

Label : Sample ID
Label : Taxon
Label : Extra Info
Label : Sequence Length
Label : Barcode Cluster (BIN)

Filter : exclude records with stop codons

Sequence Count : 1693
Species count : 142
Genus count : 14
Family count : 1
Unidentified : 0

BIN Count : 139

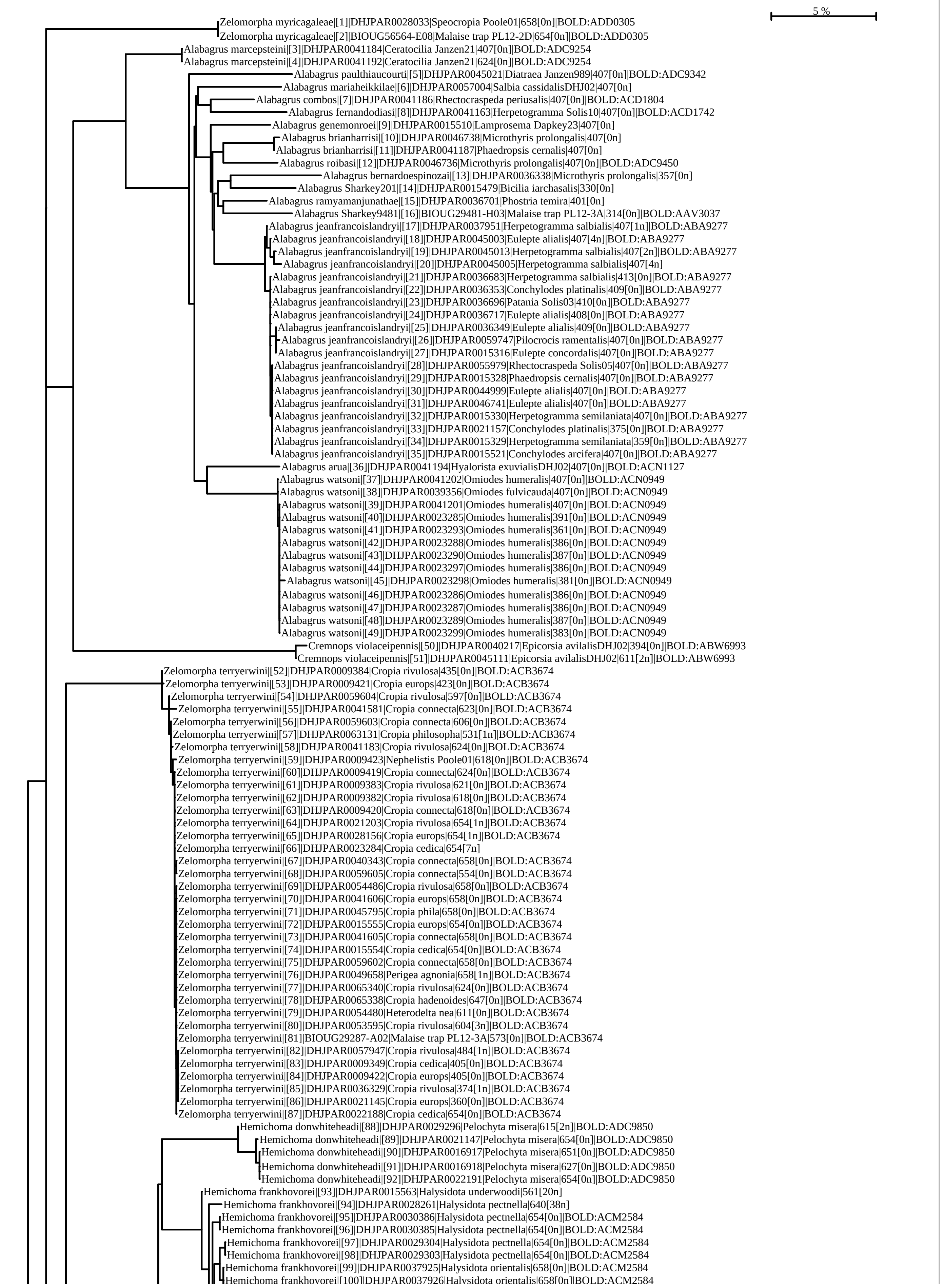
5 %
Zelomorpha myricagaleae|[1]|DHJPAR0028033|Speocropia Poole01|658[0n]|BOLD:ADD0305
Zelomorpha myricagaleae|[2]|BIOUG56564-E08|Malaise trap PL12-2D|654[0n]|BOLD:ADD0305
Alabagrus marcepsteini|[3]|DHJPAR0041184|Ceratocilia Janzen21|407[0n]|BOLD:ADC9254
Alabagrus marcepsteini|[4]|DHJPAR0041192|Ceratocilia Janzen21|624[0n]|BOLD:ADC9254
Alabagrus paulthiaucourti|[5]|DHJPAR0045021|Diatraea Janzen989|407[0n]|BOLD:ADC9342
Alabagrus mariaheikkilae|[6]|DHJPAR0057004|Salbia cassidalisDHJ02|407[0n]
Alabagrus combos|[7]|DHJPAR0041186|Rhectocraspeda periusalis|407[0n]|BOLD:ACD1804
Alabagrus fernandodiasi|[8]|DHJPAR0041163|Herpetogramma Solis10|407[0n]|BOLD:ACD1742
Alabagrus genemonroei|[9]|DHJPAR0015510|Lamprosema Dapkey23|407[0n]
Alabagrus brianharrisi|[10]|DHJPAR0046738|Microthyris prolongalis|407[0n]
Alabagrus brianharrisi|[11]|DHJPAR0041187|Phaedropsis cernalis|407[0n]
Alabagrus roibasi|[12]|DHJPAR0046736|Microthyris prolongalis|407[0n]|BOLD:ADC9450
Alabagrus bernardoespinozai|[13]|DHJPAR0036338|Microthyris prolongalis|357[0n]
Alabagrus Sharkey201|[14]|DHJPAR0015479|Bicilia iarchasalis|330[0n]
Alabagrus ramyamanjunathae|[15]|DHJPAR0036701|Phostria temira|401[0n]
Alabagrus Sharkey9481|[16]|BIOUG29481-H03|Malaise trap PL12-3A|314[0n]|BOLD:AAV3037
Alabagrus jeanfrancoislandryi|[17]|DHJPAR0037951|Herpetogramma salbialis|407[1n]|BOLD:ABA9277
Alabagrus jeanfrancoislandryi|[18]|DHJPAR0045003|Eulepte alialis|407[4n]|BOLD:ABA9277
Alabagrus jeanfrancoislandryi|[19]|DHJPAR0045013|Herpetogramma salbialis|407[2n]|BOLD:ABA9277
Alabagrus jeanfrancoislandryi|[20]|DHJPAR0045005|Herpetogramma salbialis|407[4n]
Alabagrus jeanfrancoislandryi|[21]|DHJPAR0036683|Herpetogramma salbialis|413[0n]|BOLD:ABA9277
Alabagrus jeanfrancoislandryi|[22]|DHJPAR0036353|Conchylodes platinalis|409[0n]|BOLD:ABA9277
Alabagrus jeanfrancoislandryi|[23]|DHJPAR0036696|Patania Solis03|410[0n]|BOLD:ABA9277
Alabagrus jeanfrancoislandryi|[24]|DHJPAR0036717|Eulepte alialis|408[0n]|BOLD:ABA9277
Alabagrus jeanfrancoislandryi|[25]|DHJPAR0036349|Eulepte alialis|409[0n]|BOLD:ABA9277
Alabagrus jeanfrancoislandryi|[26]|DHJPAR0059747|Pilocrocis ramentalis|407[0n]|BOLD:ABA9277
Alabagrus jeanfrancoislandryi|[27]|DHJPAR0015316|Eulepte concordalis|407[0n]|BOLD:ABA9277
Alabagrus jeanfrancoislandryi|[28]|DHJPAR0055979|Rhectocraspeda Solis05|407[0n]|BOLD:ABA9277
Alabagrus jeanfrancoislandryi|[29]|DHJPAR0015328|Phaedropsis cernalis|407[0n]|BOLD:ABA9277
Alabagrus jeanfrancoislandryi|[30]|DHJPAR0044999|Eulepte alialis|407[0n]|BOLD:ABA9277
Alabagrus jeanfrancoislandryi|[31]|DHJPAR0046741|Eulepte alialis|407[0n]|BOLD:ABA9277
Alabagrus jeanfrancoislandryi|[32]|DHJPAR0015330|Herpetogramma semilaniata|407[0n]|BOLD:ABA9277
Alabagrus jeanfrancoislandryi|[33]|DHJPAR0021157|Conchylodes platinalis|375[0n]|BOLD:ABA9277
Alabagrus jeanfrancoislandryi|[34]|DHJPAR0015329|Herpetogramma semilaniata|359[0n]|BOLD:ABA9277
Alabagrus jeanfrancoislandryi|[35]|DHJPAR0015521|Conchylodes arcifera|407[0n]|BOLD:ABA9277
Alabagrus arua|[36]|DHJPAR0041194|Hyalorista exuvialisDHJ02|407[0n]|BOLD:ACN1127
Alabagrus watsoni|[37]|DHJPAR0041202|Omiodes humeralis|407[0n]|BOLD:ACN0949
Alabagrus watsoni|[38]|DHJPAR0039356|Omiodes fulvicauda|407[0n]|BOLD:ACN0949
Alabagrus watsoni|[39]|DHJPAR0041201|Omiodes humeralis|407[0n]|BOLD:ACN0949
Alabagrus watsoni|[40]|DHJPAR0023285|Omiodes humeralis|391[0n]|BOLD:ACN0949
Alabagrus watsoni|[41]|DHJPAR0023293|Omiodes humeralis|361[0n]|BOLD:ACN0949
Alabagrus watsoni|[42]|DHJPAR0023288|Omiodes humeralis|386[0n]|BOLD:ACN0949
Alabagrus watsoni|[43]|DHJPAR0023290|Omiodes humeralis|387[0n]|BOLD:ACN0949
Alabagrus watsoni|[44]|DHJPAR0023297|Omiodes humeralis|386[0n]|BOLD:ACN0949
Alabagrus watsoni|[45]|DHJPAR0023298|Omiodes humeralis|381[0n]|BOLD:ACN0949
Alabagrus watsoni|[46]|DHJPAR0023286|Omiodes humeralis|386[0n]|BOLD:ACN0949
Alabagrus watsoni|[47]|DHJPAR0023287|Omiodes humeralis|386[0n]|BOLD:ACN0949
Alabagrus watsoni|[48]|DHJPAR0023289|Omiodes humeralis|387[0n]|BOLD:ACN0949
Alabagrus watsoni|[49]|DHJPAR0023299|Omiodes humeralis|383[0n]|BOLD:ACN0949
Cremnops violaceipennis|[50]|DHJPAR0040217|Epicorsia avilalisDHJ02|394[0n]|BOLD:ABW6993
Cremnops violaceipennis|[51]|DHJPAR0045111|Epicorsia avilalisDHJ02|611[2n]|BOLD:ABW6993
Zelomorpha terryerwini|[52]|DHJPAR0009384|Cropia rivulosa|435[0n]|BOLD:ACB3674
Zelomorpha terryerwini|[53]|DHJPAR0009421|Cropia europs|423[0n]|BOLD:ACB3674
Zelomorpha terryerwini|[54]|DHJPAR0059604|Cropia rivulosa|597[0n]|BOLD:ACB3674
Zelomorpha terryerwini|[55]|DHJPAR0041581|Cropia connecta|623[0n]|BOLD:ACB3674
Zelomorpha terryerwini|[56]|DHJPAR0059603|Cropia connecta|606[0n]|BOLD:ACB3674
Zelomorpha terryerwini|[57]|DHJPAR0063131|Cropia philosopha|531[1n]|BOLD:ACB3674
Zelomorpha terryerwini|[58]|DHJPAR0041183|Cropia rivulosa|624[0n]|BOLD:ACB3674
Zelomorpha terryerwini|[59]|DHJPAR0009423|Nephelistis Poole01|618[0n]|BOLD:ACB3674
Zelomorpha terryerwini|[60]|DHJPAR0009419|Cropia connecta|624[0n]|BOLD:ACB3674
Zelomorpha terryerwini|[61]|DHJPAR0009383|Cropia rivulosa|621[0n]|BOLD:ACB3674
Zelomorpha terryerwini|[62]|DHJPAR0009382|Cropia rivulosa|618[0n]|BOLD:ACB3674
Zelomorpha terryerwini|[63]|DHJPAR0009420|Cropia connecta|618[0n]|BOLD:ACB3674
Zelomorpha terryerwini|[64]|DHJPAR0021203|Cropia rivulosa|654[1n]|BOLD:ACB3674
Zelomorpha terryerwini|[65]|DHJPAR0028156|Cropia europs|654[1n]|BOLD:ACB3674
Zelomorpha terryerwini|[66]|DHJPAR0023284|Cropia cedica|654[7n]
Zelomorpha terryerwini|[67]|DHJPAR0040343|Cropia connecta|658[0n]|BOLD:ACB3674
Zelomorpha terryerwini|[68]|DHJPAR0059605|Cropia connecta|554[0n]|BOLD:ACB3674
Zelomorpha terryerwini|[69]|DHJPAR0054486|Cropia rivulosa|658[0n]|BOLD:ACB3674
Zelomorpha terryerwini|[70]|DHJPAR0041606|Cropia europs|658[0n]|BOLD:ACB3674
Zelomorpha terryerwini|[71]|DHJPAR0045795|Cropia phila|658[0n]|BOLD:ACB3674
Zelomorpha terryerwini|[72]|DHJPAR0015555|Cropia europs|654[0n]|BOLD:ACB3674
Zelomorpha terryerwini|[73]|DHJPAR0041605|Cropia connecta|658[0n]|BOLD:ACB3674
Zelomorpha terryerwini|[74]|DHJPAR0015554|Cropia cedica|654[0n]|BOLD:ACB3674
Zelomorpha terryerwini|[75]|DHJPAR0059602|Cropia connecta|658[0n]|BOLD:ACB3674
Zelomorpha terryerwini|[76]|DHJPAR0049658|Perigea agnonia|658[1n]|BOLD:ACB3674
Zelomorpha terryerwini|[77]|DHJPAR0065340|Cropia rivulosa|624[0n]|BOLD:ACB3674
Zelomorpha terryerwini|[78]|DHJPAR0065338|Cropia hadenoides|647[0n]|BOLD:ACB3674
Zelomorpha terryerwini|[79]|DHJPAR0054480|Heterodelta nea|611[0n]|BOLD:ACB3674
Zelomorpha terryerwini|[80]|DHJPAR0053595|Cropia rivulosa|604[3n]|BOLD:ACB3674
Zelomorpha terryerwini|[81]|BIOUG29287-A02|Malaise trap PL12-3A|573[0n]|BOLD:ACB3674
Zelomorpha terryerwini|[82]|DHJPAR0057947|Cropia rivulosa|484[1n]|BOLD:ACB3674
Zelomorpha terryerwini|[83]|DHJPAR0009349|Cropia cedica|405[0n]|BOLD:ACB3674
Zelomorpha terryerwini|[84]|DHJPAR0009422|Cropia europs|405[0n]|BOLD:ACB3674
Zelomorpha terryerwini|[85]|DHJPAR0036329|Cropia rivulosa|374[1n]|BOLD:ACB3674
Zelomorpha terryerwini|[86]|DHJPAR0021145|Cropia europs|360[0n]|BOLD:ACB3674
Zelomorpha terryerwini|[87]|DHJPAR0022188|Cropia cedica|654[0n]|BOLD:ACB3674
Hemichoma donwhiteheadi|[88]|DHJPAR0029296|Pelochyta misera|615[2n]|BOLD:ADC9850
Hemichoma donwhiteheadi|[89]|DHJPAR0021147|Pelochyta misera|654[0n]|BOLD:ADC9850
Hemichoma donwhiteheadi|[90]|DHJPAR0016917|Pelochyta misera|651[0n]|BOLD:ADC9850
Hemichoma donwhiteheadi|[91]|DHJPAR0016918|Pelochyta misera|627[0n]|BOLD:ADC9850
Hemichoma donwhiteheadi|[92]|DHJPAR0022191|Pelochyta misera|654[0n]|BOLD:ADC9850
Hemichoma frankhovorei|[93]|DHJPAR0015563|Halysidota underwoodi|561[20n]
Hemichoma frankhovorei|[94]|DHJPAR0028261|Halysidota pectnella|640[38n]
Hemichoma frankhovorei|[95]|DHJPAR0030386|Halysidota pectnella|654[0n]|BOLD:ACM2584
Hemichoma frankhovorei|[96]|DHJPAR0030385|Halysidota pectnella|654[0n]|BOLD:ACM2584
Hemichoma frankhovorei|[97]|DHJPAR0029304|Halysidota pectnella|654[0n]|BOLD:ACM2584
Hemichoma frankhovorei|[98]|DHJPAR0029303|Halysidota pectnella|654[0n]|BOLD:ACM2584
Hemichoma frankhovorei|[99]|DHJPAR0037925|Halysidota orientalis|658[0n]|BOLD:ACM2584
Hemichoma frankhovorei|[100]|DHJPAR0037926|Halysidota orientalis|658[0n]|BOLD:ACM2584

[illegible]
Hemichoma frankhovorei|[98]|DHJPAR0029303|Halysidota pectnella|654[0n]|BOLD:ACM2584
Hemichoma frankhovorei|[99]|DHJPAR0037925|Halysidota orientalis|658[0n]|BOLD:ACM2584
Hemichoma frankhovorei|[100]|DHJPAR0037926|Halysidota orientalis|658[0n]|BOLD:ACM2584
Hemichoma frankhovorei|[101]|DHJPAR0054503|Halysidota schausi|658[0n]|BOLD:ACM2584
Hemichoma frankhovorei|[102]|DHJPAR0036708|Halysidota pectnella|642[0n]|BOLD:ACM2584
Hemichoma frankhovorei|[103]|DHJPAR0036689|Halysidota pectnella|627[0n]|BOLD:ACM2584
Hemichoma frankhovorei|[104]|DHJPAR0036713|Halysidota pectnella|627[0n]|BOLD:ACM2584
Hemichoma frankhovorei|[105]|DHJPAR0041155|Halysidota underwoodi|291[0n]
Hemichoma frankhovorei|[106]|DHJPAR0041160|Halysidota underwoodi|291[0n]
Hemichoma frankhovorei|[107]|DHJPAR0041161|Halysidota underwoodi|291[0n]
Hemichoma frankhovorei|[108]|DHJPAR0041158|Halysidota underwoodi|291[0n]
Hemichoma frankhovorei|[109]|DHJPAR0041154|Halysidota underwoodi|288[0n]
Hemichoma frankhovorei|[110]|DHJPAR0028250|Halysidota pectnella|321[0n]|BOLD:ACM2584
Hemichoma frankhovorei|[111]|DHJPAR0028249|Halysidota pectnella|255[0n]
Hemichoma frankhovorei|[112]|DHJPAR0028252|Halysidota pectnella|258[0n]
Hemichoma frankhovorei|[113]|DHJPAR0028263|Halysidota pectnella|258[0n]
Hemichoma frankhovorei|[114]|DHJPAR0028247|Halysidota pectnella|261[0n]
Hemichoma frankhovorei|[115]|DHJPAR0028248|Halysidota pectnella|261[0n]
Hemichoma frankhovorei|[116]|DHJPAR0028242|Halysidota pectnella|261[0n]
Hemichoma frankhovorei|[117]|DHJPAR0028258|Halysidota pectnella|261[0n]
Hemichoma frankhovorei|[118]|DHJPAR0028260|Halysidota pectnella|261[0n]
Hemichoma frankhovorei|[119]|DHJPAR0028246|Halysidota pectnella|261[0n]
Hemichoma frankhovorei|[120]|DHJPAR0028254|Halysidota pectnella|261[0n]
Hemichoma frankhovorei|[121]|DHJPAR0028243|Halysidota pectnella|261[0n]
Hemichoma frankhovorei|[122]|DHJPAR0028262|Halysidota pectnella|261[0n]
Hemichoma frankhovorei|[123]|DHJPAR0028259|Halysidota pectnella|261[0n]
Hemichoma frankhovorei|[124]|DHJPAR0028244|Halysidota pectnella|261[0n]
Hemichoma frankhovorei|[125]|DHJPAR0006774|Halysidota orientalis|234[0n]
Hemichoma frankhovorei|[126]|DHJPAR0028264|Halysidota pectnella|267[0n]
Hemichoma frankhovorei|[127]|DHJPAR0054502|Halysidota schausi|658[0n]|BOLD:ACM2584
Hemichoma frankhovorei|[128]|DHJPAR0054501|Halysidota schausi|658[0n]|BOLD:ACM2584
Hemichoma johnkingsolveri|[129]|DHJPAR0060429|Pachydota saduca|558[0n]|BOLD:ABU8130
Hemichoma johnkingsolveri|[130]|DHJPAR0038613|Carathis septentrionalis|606[0n]|BOLD:ABU8130
Hemichoma johnkingsolveri|[131]|DHJPAR0041168|Pachydota saduca|622[0n]|BOLD:ABU8130
Hemichoma johnkingsolveri|[132]|DHJPAR0015560|Pachydota saduca|555[0n]|BOLD:ABU8130
Hemichoma johnkingsolveri|[133]|DHJPAR0022195|Pachydota saduca|591[0n]|BOLD:ABU8130
Hemichoma johnkingsolveri|[134]|DHJPAR0062414|Pachydota saduca|658[0n]|BOLD:ABU8130
Hemichoma johnkingsolveri|[135]|DHJPAR0046730|Pachydota saduca|658[0n]|BOLD:ABU8130
Hemichoma johnkingsolveri|[136]|DHJPAR0036333|Pachydota saduca|654[0n]|BOLD:ABU8130
Hemichoma johnkingsolveri|[137]|DHJPAR0058547|Pachydota saduca|658[0n]|BOLD:ABU8130
Hemichoma johnkingsolveri|[138]|DHJPAR0042359|Pachydota saduca|658[0n]|BOLD:ABU8130
Hemichoma johnkingsolveri|[139]|DHJPAR0061015|Pachydota saduca|658[0n]|BOLD:ABU8130
Hemichoma johnkingsolveri|[140]|DHJPAR0061442|Pachydota saduca|658[0n]|BOLD:ABU8130
Hemichoma johnkingsolveri|[141]|DHJPAR0062416|Pachydota saduca|658[0n]|BOLD:ABU8130
Hemichoma johnkingsolveri|[142]|DHJPAR0042358|Pachydota saduca|658[0n]|BOLD:ABU8130
Hemichoma johnkingsolveri|[143]|DHJPAR0057646|Pachydota saduca|658[0n]|BOLD:ABU8130
Hemichoma johnkingsolveri|[144]|DHJPAR0057457|Pachydota saduca|658[0n]|BOLD:ABU8130
Hemichoma johnkingsolveri|[145]|DHJPAR0060428|Pachydota saduca|658[0n]|BOLD:ABU8130
Hemichoma johnkingsolveri|[146]|DHJPAR0063374|Pachydota saduca|658[0n]|BOLD:ABU8130
Hemichoma johnkingsolveri|[147]|DHJPAR0046732|Pachydota saduca|658[0n]|BOLD:ABU8130
Hemichoma johnkingsolveri|[148]|DHJPAR0062011|Pachydota saduca|658[0n]|BOLD:ABU8130
Hemichoma johnkingsolveri|[149]|DHJPAR0015558|Pachydota saduca|654[0n]|BOLD:ABU8130
Hemichoma johnkingsolveri|[150]|DHJPAR0062013|Pachydota drucei|658[0n]|BOLD:ABU8130
Hemichoma johnkingsolveri|[151]|DHJPAR0058548|Pachydota saduca|658[0n]|BOLD:ABU8130
Hemichoma johnkingsolveri|[152]|DHJPAR0061016|Pachydota saduca|658[1n]|BOLD:ABU8130
Hemichoma johnkingsolveri|[153]|DHJPAR0060427|Carathis septentrionalis|633[0n]|BOLD:ABU8130
Hemichoma johnkingsolveri|[154]|DHJPAR0015559|Pachydota saduca|556[15n]
Hemichoma johnkingsolveri|[155]|DHJPAR0057945|Pachydota saduca|614[0n]|BOLD:ABU8130
Hemichoma johnkingsolveri|[156]|DHJPAR0062010|Pachydota saduca|658[0n]|BOLD:ABU8130
Hemichoma johnkingsolveri|[157]|DHJPAR0046731|Pachydota saduca|658[0n]|BOLD:ABU8130
Zelomorpha Janzen4431|[158]|DHJPAR0064388|Oraesia serpens|653[0n]|BOLD:ADJ4431
Zelomorpha Janzen4431|[159]|DHJPAR0065127|Gonodonta sinaldus|621[0n]|BOLD:ADJ4431
Zelomorpha Janzen4431|[160]|DHJPAR0061441|Graphigona Poole01|658[0n]|BOLD:ADJ4431
Zelomorpha willsflowersi|[161]|DHJPAR0009412|Coenipeta bibitrix|624[0n]|BOLD:ADD0170
Zelomorpha willsflowersi|[162]|DHJPAR0009413|Coenipeta bibitrix|624[0n]|BOLD:ADD0170
Zelomorpha willsflowersi|[163]|DHJPAR0009414|Coenipeta bibitrix|624[0n]|BOLD:ADD0170
Zelomorpha willsflowersi|[164]|DHJPAR0009415|Coenipeta bibitrix|624[0n]|BOLD:ADD0170
Zelomorpha willsflowersi|[165]|DHJPAR0009418|Coenipeta bibitrix|597[0n]|BOLD:ADD0170
Zelomorpha willsflowersi|[166]|DHJPAR0021205|Goniohelia Poole02|654[0n]|BOLD:ADD0170
Zelomorpha willsflowersi|[167]|DHJPAR0010194|Coenipeta bibitrix|658[0n]|BOLD:ADD0170
Zelomorpha willsflowersi|[168]|DHJPAR0057944|Cyclopteryx niveinotata|387[2n]|BOLD:ADD0170
Zelomorpha willsflowersi|[169]|DHJPAR0021146|Tyrissa acygonia|645[0n]|BOLD:ADD0170
Zelomorpha paulgoldsteini|[170]|DHJPAR0041159|Nicetas Poole22|255[1n]
Zelomorpha paulgoldsteini|[171]|DHJPAR0040222|Callopistria mexicana|540[0n]|BOLD:AAZ6457
Zelomorpha paulgoldsteini|[172]|DHJPAR0009404|Rejectaria splendida|624[0n]|BOLD:AAZ6457
Zelomorpha paulgoldsteini|[173]|DHJPAR0041152|Rejectaria splendida|622[0n]|BOLD:AAZ6457
Zelomorpha paulgoldsteini|[174]|DHJPAR0041153|Rejectaria splendida|623[0n]|BOLD:AAZ6457
Zelomorpha paulgoldsteini|[175]|BIOUG27746-F03|ESG Malaise Trap|621[0n]|BOLD:AAZ6457
Zelomorpha paulgoldsteini|[176]|DHJPAR0054485|Rejectaria splendida|620[0n]|BOLD:AAZ6457
Zelomorpha paulgoldsteini|[177]|BIOUG22611-D10|ESG Malaise Trap|627[0n]|BOLD:AAZ6457
Zelomorpha paulgoldsteini|[178]|DHJPAR0036684|Nicetas Janzen02|618[0n]|BOLD:AAZ6457
Zelomorpha paulgoldsteini|[179]|DHJPAR0016426|Rejectaria Janzen02|658[1n]|BOLD:AAZ6457
Zelomorpha paulgoldsteini|[180]|DHJPAR0030382|Rejectaria Janzen06|654[0n]|BOLD:AAZ6457
Zelomorpha paulgoldsteini|[181]|DHJPAR0064387|Salia Poole03|658[0n]|BOLD:AAZ6457
Zelomorpha paulgoldsteini|[182]|DHJPAR0042810|Nicetas Poole20|658[0n]|BOLD:AAZ6457
Zelomorpha paulgoldsteini|[183]|DHJPAR0057458|Rejectaria Janzen02|658[0n]|BOLD:AAZ6457
Zelomorpha paulgoldsteini|[184]|DHJPAR0042808|Nicetas Poole20|658[0n]|BOLD:AAZ6457
Zelomorpha paulgoldsteini|[185]|DHJPAR0042357|Callopistria floridensis|658[0n]|BOLD:AAZ6457
Zelomorpha paulgoldsteini|[186]|DHJPAR0065134|Callopistria floridensis|658[0n]|BOLD:AAZ6457
Zelomorpha paulgoldsteini|[187]|DHJPAR0052697|Callopistria floridensis|658[0n]|BOLD:AAZ6457
Zelomorpha paulgoldsteini|[188]|DHJPAR0057443|Nicetas antonalisDHJ02|658[0n]|BOLD:AAZ6457
Zelomorpha paulgoldsteini|[189]|DHJPAR0028032|Rejectaria Janzen06|658[0n]|BOLD:AAZ6457
Zelomorpha paulgoldsteini|[190]|DHJPAR0044986|Nicetas Poole21|661[0n]|BOLD:AAZ6457
Zelomorpha paulgoldsteini|[191]|DHJPAR0054469|Rejectaria splendida|658[0n]|BOLD:AAZ6457
Zelomorpha paulgoldsteini|[192]|DHJPAR0054470|Rejectaria splendida|658[0n]|BOLD:AAZ6457
Zelomorpha paulgoldsteini|[193]|DHJPAR0016425|Rejectaria Janzen02|658[0n]|BOLD:AAZ6457
Zelomorpha paulgoldsteini|[194]|DHJPAR0057447|Rejectaria Janzen06|658[0n]|BOLD:AAZ6457
Zelomorpha paulgoldsteini|[195]|DHJPAR0057649|Rejectaria splendidaDHJ01|658[0n]|BOLD:AAZ6457
Zelomorpha paulgoldsteini|[196]|BIOUG27999-D09|ESG Malaise Trap|600[0n]|BOLD:AAZ6457
Zelomorpha paulgoldsteini|[197]|DHJPAR0064391|Dusponera fannia|653[0n]|BOLD:AAZ6457
Zelomorpha paulgoldsteini|[198]|DHJPAR0015539|Rejectaria Poole11|645[0n]|BOLD:AAZ6457
Zelomorpha paulgoldsteini|[199]|BIOUG27762-C12|ESG Malaise Trap Year 2|564[0n]|BOLD:AAZ6457
Zelomorpha paulgoldsteini|[200]|DHJPAR0057460|Nicetas Poole20|632[0n]|BOLD:AAZ6457

[illegible]
Zelomorpha paulgoldsteini|[198]|DHJPAR0015539|Rejectaria Poole11|645[0n]|BOLD:AAZ6457
Zelomorpha paulgoldsteini|[199]|BIOUG27762-C12|ESG Malaise Trap Year 2|564[0n]|BOLD:AAZ6457
Zelomorpha paulgoldsteini|[200]|DHJPAR0057460|Nicetas Poole20|632[0n]|BOLD:AAZ6457
Zelomorpha paulgoldsteini|[201]|BIOUG22611-D08|ESG Malaise Trap|628[0n]|BOLD:AAZ6457
Zelomorpha paulgoldsteini|[202]|BIOUG23959-F01|ESG Malaise Trap|588[0n]|BOLD:AAZ6457
Zelomorpha danjohnsoni|[203]|DHJPAR0009409|Diastema morata|624[0n]|BOLD:ADC9984
Zelomorpha larrykirkendalli|[204]|DHJPAR0052087|Opisthoxia uncinata|574[0n]|BOLD:ACC2880
Zelomorpha larrykirkendalli|[205]|DHJPAR0063795|Opisthoxia molpadia|609[0n]|BOLD:ACC2880
Zelomorpha larrykirkendalli|[206]|DHJPAR0064520|Opisthoxia amabilis|658[0n]|BOLD:ACC2880
Zelomorpha larrykirkendalli|[207]|DHJPAR0063996|Argyrotome alba|658[0n]|BOLD:ACC2880
Zelomorpha larrykirkendalli|[208]|DHJPAR0055084|Opisthoxia molpadia|658[0n]|BOLD:ACC2880
Zelomorpha larrykirkendalli|[209]|DHJPAR0064515|Argyrotome alba|658[0n]|BOLD:ACC2880
Zelomorpha larrykirkendalli|[210]|DHJPAR0064509|Opisthoxia amabilis|658[0n]|BOLD:ACC2880
Zelomorpha larrykirkendalli|[211]|DHJPAR0064514|Opisthoxia amabilis|658[0n]|BOLD:ACC2880
Zelomorpha larrykirkendalli|[212]|DHJPAR0064519|Opisthoxia asopis|658[0n]|BOLD:ACC2880
Zelomorpha larrykirkendalli|[213]|DHJPAR0015540|Opisthoxia bella|654[0n]|BOLD:ACC2880
Zelomorpha larrykirkendalli|[214]|DHJPAR0055988|Opisthoxia molpadia|407[0n]|BOLD:ACC2880
Zelomorpha larrykirkendalli|[215]|DHJPAR0055981|Opisthoxia uncinata|670[0n]|BOLD:ACC2880
Zelomorpha arizonensis|[216]|DHJPAR0062199|Bulia mexicana|586[1n]|BOLD:AAG7943
Zelomorpha arizonensis|[217]|DHJPAR0062200|Bulia mexicana|512[3n]|BOLD:AAG7943
Zelomorpha arizonensis|[218]|DHJPAR0062206|Bulia mexicana|586[1n]|BOLD:AAG7943
Zelomorpha arizonensis|[219]|DHJPAR0062202|Bulia mexicana|586[0n]|BOLD:AAG7943
Zelomorpha arizonensis|[220]|DHJPAR0062207|Bulia mexicana|511[2n]|BOLD:AAG7943
Zelomorpha arizonensis|[221]|DHJPAR0062201|Bulia mexicana|586[0n]|BOLD:AAG7943
Zelomorpha arizonensis|[222]|DHJPAR0062208|Bulia mexicana|526[3n]|BOLD:AAG7943
Zelomorpha arizonensis|[223]|DHJPAR0052702|Bulia mexicana|605[0n]|BOLD:AAG7943
Zelomorpha arizonensis|[224]|DHJPAR0062204|Bulia mexicana|615[0n]|BOLD:AAG7943
Zelomorpha arizonensis|[225]|DHJPAR0062205|Bulia mexicana|622[0n]|BOLD:AAG7943
Zelomorpha arizonensis|[226]|DHJPAR0052705|Bulia mexicana|658[1n]|BOLD:AAG7943
Zelomorpha arizonensis|[227]|DHJPAR0052707|Bulia mexicana|658[0n]|BOLD:AAG7943
Zelomorpha arizonensis|[228]|DHJPAR0052704|Bulia mexicana|658[0n]|BOLD:AAG7943
Zelomorpha arizonensis|[229]|DHJPAR0052708|Bulia mexicana|658[1n]|BOLD:AAG7943
Zelomorpha arizonensis|[230]|DHJPAR0052703|Bulia mexicana|658[1n]|BOLD:AAG7943
Zelomorpha arizonensis|[231]|DHJPAR0052709|Bulia mexicana|658[0n]|BOLD:AAG7943
Zelomorpha arizonensis|[232]|DHJPAR0062203|Bulia mexicana|616[2n]|BOLD:AAG7943
Zelomorpha donwindsori|[233]|DHJPAR0052679|Paectes lunodes|573[11n]
Zelomorpha donwindsori|[234]|DHJPAR0048721|Paectes fuscescens|658[0n]|BOLD:ACB1906
Zelomorpha mariyavladmirovnae|[235]|DHJPAR0023528|Ormetica sicilia|654[0n]|BOLD:ADD0088
Zelomorpha kellyanneae|[236]|DHJPAR0029301|Nephodia Janzen18|618[0n]|BOLD:ADD0384
Zelomorpha kellyanneae|[237]|DHJPAR0042809|Nephodia Janzen18|658[0n]|BOLD:ADD0384
Zelomorpha kellyanneae|[238]|DHJPAR0015543|Nephodia Janzen18|654[0n]|BOLD:ADD0384
Zelomorpha kellyanneae|[239]|DHJPAR0015536|Nephodia Janzen18|654[0n]|BOLD:ADD0384
Zelomorpha kellyanneae|[240]|DHJPAR0009395|Nephodia Janzen18|609[2n]|BOLD:ADD0384
Zelomorpha kellyanneae|[241]|DHJPAR0015542|Nephodia Janzen18|654[0n]|BOLD:ADD0384
Zelomorpha kellyanneae|[242]|DHJPAR0042806|Nephodia Janzen18|658[0n]|BOLD:ADD0384
Zelomorpha kellyanneae|[243]|DHJPAR0009394|Nephodia Janzen18|654[0n]|BOLD:ADD0384
Zelomorpha noahjaneae|[244]|DHJPAR0048723|Paectes fuscescens|658[0n]|BOLD:AAV7758
Zelomorpha noahjaneae|[245]|DHJPAR0028023|Paectes Poole10|657[0n]|BOLD:AAV7758
Zelomorpha noahjaneae|[246]|DHJPAR0028024|Paectes Poole10|627[0n]|BOLD:AAV7758
Zelomorpha noahjaneae|[247]|DHJPAR0052678|Eutelia chrysotermina|658[1n]|BOLD:AAV7758
Zelomorpha noahjaneae|[248]|DHJPAR0048719|Paectes fuscescens|636[0n]|BOLD:AAV7758
Zelomorpha noahjaneae|[249]|DHJPAR0048720|Paectes fuscescens|658[0n]|BOLD:AAV7758
Zelomorpha effugia|[250]|DHJPAR0009330|Cosmosoma hercyna|606[8n]
Zelomorpha effugia|[251]|DHJPAR0064000|Cosmosoma hercyna|619[0n]|BOLD:ACM2491
Zelomorpha effugia|[252]|DHJPAR0009380|Cosmosoma hercyna|615[5n]|BOLD:ACM2491
Zelomorpha effugia|[253]|DHJPAR0009337|Cosmosoma hercyna|654[2n]|BOLD:ACM2491
Zelomorpha effugia|[254]|DHJPAR0009341|Cosmosoma hercyna|654[2n]|BOLD:ACM2491
Zelomorpha effugia|[255]|DHJPAR0009336|Cosmosoma hercyna|651[4n]|BOLD:ACM2491
Zelomorpha effugia|[256]|DHJPAR0009332|Cosmosoma hercyna|654[4n]|BOLD:ACM2491
Zelomorpha effugia|[257]|DHJPAR0009333|Cosmosoma hercyna|654[3n]|BOLD:ACM2491
Zelomorpha effugia|[258]|DHJPAR0009328|Cosmosoma hercyna|654[2n]|BOLD:ACM2491
Zelomorpha effugia|[259]|DHJPAR0015545|Cosmosoma hercyna|645[0n]|BOLD:ACM2491
Zelomorpha effugia|[260]|DHJPAR0009342|Cosmosoma hercyna|651[6n]|BOLD:ACM2491
Zelomorpha effugia|[261]|DHJPAR0009334|Cosmosoma hercyna|654[1n]|BOLD:ACM2491
Zelomorpha effugia|[262]|DHJPAR0009331|Cosmosoma hercyna|654[1n]|BOLD:ACM2491
Zelomorpha effugia|[263]|DHJPAR0009379|Cosmosoma hercyna|654[1n]|BOLD:ACM2491
Zelomorpha effugia|[264]|DHJPAR0015546|Cosmosoma hercyna|654[0n]|BOLD:ACM2491
Zelomorpha effugia|[265]|DHJPAR0057452|Cosmosoma hercyna|658[0n]|BOLD:ACM2491
Zelomorpha effugia|[266]|DHJPAR0057455|Cosmosoma hercyna|658[0n]|BOLD:ACM2491
Zelomorpha effugia|[267]|DHJPAR0015535|Cosmosoma hercyna|654[0n]|BOLD:ACM2491
Zelomorpha effugia|[268]|DHJPAR0056359|Cosmosoma hercyna|658[0n]|BOLD:ACM2491
Zelomorpha effugia|[269]|DHJPAR0057453|Cosmosoma hercyna|658[0n]|BOLD:ACM2491
Zelomorpha effugia|[270]|DHJPAR0009343|Cosmosoma hercyna|654[0n]|BOLD:ACM2491
Zelomorpha effugia|[271]|DHJPAR0054472|Cosmosoma hercyna|658[0n]|BOLD:ACM2491
Zelomorpha effugia|[272]|DHJPAR0017278|Cosmosoma hercyna|654[0n]|BOLD:ACM2491
Zelomorpha effugia|[273]|DHJPAR0017281|Cosmosoma hercyna|654[0n]|BOLD:ACM2491
Zelomorpha effugia|[274]|DHJPAR0015551|Cosmosoma hercyna|654[0n]|BOLD:ACM2491
Zelomorpha effugia|[275]|DHJPAR0009432|Cosmosoma hercyna|654[0n]|BOLD:ACM2491
Zelomorpha effugia|[276]|DHJPAR0015544|Cosmosoma hercyna|654[0n]|BOLD:ACM2491
Zelomorpha effugia|[277]|DHJPAR0015541|Cosmosoma hercyna|654[0n]|BOLD:ACM2491
Zelomorpha effugia|[278]|DHJPAR0017283|Cosmosoma hercyna|654[0n]|BOLD:ACM2491
Zelomorpha effugia|[279]|DHJPAR0054516|Cosmosoma hercyna|658[0n]|BOLD:ACM2491
Zelomorpha effugia|[280]|DHJPAR0017282|Cosmosoma hercyna|654[0n]|BOLD:ACM2491
Zelomorpha effugia|[281]|DHJPAR0015547|Cosmosoma hercyna|654[0n]|BOLD:ACM2491
Zelomorpha effugia|[282]|DHJPAR0017279|Cosmosoma hercyna|654[0n]|BOLD:ACM2491
Zelomorpha effugia|[283]|DHJPAR0054479|Cosmosoma hercyna|658[0n]|BOLD:ACM2491
Zelomorpha effugia|[284]|DHJPAR0057456|Cosmosoma hercyna|658[0n]|BOLD:ACM2491
Zelomorpha effugia|[285]|DHJPAR0054474|Cosmosoma hercyna|658[0n]|BOLD:ACM2491
Zelomorpha effugia|[286]|DHJPAR0054483|Cosmosoma hercyna|658[0n]|BOLD:ACM2491
Zelomorpha effugia|[287]|DHJPAR0054475|Cosmosoma hercyna|658[0n]|BOLD:ACM2491
Zelomorpha effugia|[288]|DHJPAR0017280|Cosmosoma hercyna|654[0n]|BOLD:ACM2491
Zelomorpha effugia|[289]|DHJPAR0015550|Cosmosoma hercyna|654[0n]|BOLD:ACM2491
Zelomorpha effugia|[290]|DHJPAR0057454|Cosmosoma hercyna|658[0n]|BOLD:ACM2491
Zelomorpha effugia|[291]|DHJPAR0054489|Cosmosoma hercyna|658[0n]|BOLD:ACM2491
Zelomorpha effugia|[292]|DHJPAR0015552|Cosmosoma hercyna|654[0n]|BOLD:ACM2491
Zelomorpha effugia|[293]|DHJPAR0015549|Cosmosoma hercyna|654[0n]|BOLD:ACM2491
Zelomorpha effugia|[294]|DHJPAR0015538|Cosmosoma hercyna|654[0n]|BOLD:ACM2491
Zelomorpha effugia|[295]|DHJPAR0009338|Cosmosoma hercyna|651[3n]|BOLD:ACM2491
Zelomorpha effugia|[296]|DHJPAR0009329|Cosmosoma hercyna|654[2n]|BOLD:ACM2491
Zelomorpha effugia|[297]|DHJPAR0009431|Cosmosoma hercyna|654[3n]|BOLD:ACM2491
Zelomorpha effugia|[298]|DHJPAR0054473|Cosmosoma hercyna|615[0n]|BOLD:ACM2491
Zelomorpha effugia|[299]|DHJPAR0054482|Cosmosoma hercyna|647[0n]|BOLD:ACM2491
[illegible]

Zelomorpha effugia|[297]|DHJPAR0009431|Cosmosoma hercyna|654[3n]|BOLD:ACM2491
Zelomorpha effugia|[298]|DHJPAR0054473|Cosmosoma hercyna|615[0n]|BOLD:ACM2491
Zelomorpha effugia|[299]|DHJPAR0054482|Cosmosoma hercyna|647[0n]|BOLD:ACM2491
Zelomorpha effugia|[300]|DHJPAR0009381|Cosmosoma hercyna|606[1n]|BOLD:ACM2491
Zelomorpha effugia|[301]|BIOUG19932-H06|ESG Malaise Trap|588[0n]|BOLD:ACM2491
Zelomorpha effugia|[302]|DHJPAR0009340|Cosmosoma hercyna|576[0n]|BOLD:ACM2491
Zelomorpha effugia|[303]|DHJPAR0009339|Cosmosoma hercyna|594[0n]|BOLD:ACM2491
Zelomorpha effugia|[304]|DHJPAR0009335|Cosmosoma hercyna|651[3n]|BOLD:ACM2491
Zelomorpha effugia|[305]|DHJPAR0054484|Cosmosoma hercyna|658[0n]|BOLD:ACM2491
Zelomorpha effugia|[306]|DHJPAR0054476|Cosmosoma hercyna|658[0n]|BOLD:ACM2491
Zelomorpha effugia|[307]|DHJPAR0054481|Cosmosoma hercyna|658[0n]|BOLD:ACM2491
Zelomorpha effugia|[308]|DHJPAR0017275|Cosmosoma hercyna|654[0n]|BOLD:ACM2491
Zelomorpha effugia|[309]|DHJPAR0015548|Cosmosoma hercyna|654[0n]|BOLD:ACM2491
Zelomorpha effugia|[310]|DHJPAR0056979|Cosmosoma hercyna|658[0n]|BOLD:ACM2491
Zelomorpha effugia|[311]|DHJPAR0054478|Cosmosoma hercyna|658[0n]|BOLD:ACM2491
Zelomorpha effugia|[312]|DHJPAR0015553|Cosmosoma hercyna|654[0n]|BOLD:ACM2491
Zelomorpha effugia|[313]|DHJPAR0054477|Cosmosoma hercyna|658[0n]|BOLD:ACM2491
Zelomorpha effugia|[314]|DHJPAR0062008|Rhynchopyga cryptoleuca|658[0n]|BOLD:ACM2491
Zelomorpha sp.|[315]|BIOUG27761-E02|ESG Malaise Trap Year 2|594[0n]|BOLD:AEF3784
Zelomorpha sp.|[316]|BIOUG55300-F02|Malaise trap PL12-3D|644[0n]|BOLD:AAV3008
Zelomorpha sp.|[317]|BIOUG53753-A11|Malaise trap PL12-7D|652[0n]|BOLD:AAV3008
Zelomorpha sarahmeierottoae|[318]|BIOUG05883-E01|BSE Malaise Trap|658[1n]|BOLD:ACG5511
Zelomorpha mikeiviei|[319]|DHJPAR0029297|geoJanzen01 geo9455|621[0n]|BOLD:ADC9455
Zelomorpha mikeiviei|[320]|DHJPAR0040325|geoJanzen01 geo9455|658[0n]|BOLD:ADC9455
Zelomorpha mikeiviei|[321]|DHJPAR0058546|geoJanzen01 geo9455|229[2n]
Zelomorpha bobandersoni|[322]|DHJPAR0064390|Hemiceras plusiata|605[1n]|BOLD:ACN0769
Zelomorpha bobandersoni|[323]|DHJPAR0036332|Hemiceras plusiata|521[0n]|BOLD:ACN0769
Zelomorpha bobandersoni|[324]|DHJPAR0036330|Hemiceras plusiata|622[0n]|BOLD:ACN0769
Zelomorpha bobandersoni|[325]|DHJPAR0052686|Hemiceras plusiata|658[1n]|BOLD:ACN0769
Zelomorpha bobandersoni|[326]|DHJPAR0057648|Hemiceras plusiata|658[1n]|BOLD:ACN0769
Zelomorpha bobandersoni|[327]|DHJPAR0059599|Hemiceras plusiata|657[0n]|BOLD:ACN0769
Zelomorpha bobandersoni|[328]|DHJPAR0059338|Hemiceras plusiata|657[0n]|BOLD:ACN0769
Zelomorpha bobandersoni|[329]|DHJPAR0061033|Hemiceras plusiata|658[0n]|BOLD:ACN0769
Zelomorpha bobandersoni|[330]|DHJPAR0059335|Hemiceras plusiata|657[1n]|BOLD:ACN0769
Zelomorpha bobandersoni|[331]|DHJPAR0063377|Hemiceras plusiata|628[0n]|BOLD:ACN0769
Zelomorpha bobandersoni|[332]|DHJPAR0063376|Hemiceras plusiata|609[2n]|BOLD:ACN0769
Zelomorpha bobandersoni|[333]|DHJPAR0059600|Hemiceras plusiata|612[0n]|BOLD:ACN0769
Zelomorpha bobandersoni|[334]|DHJPAR0059334|Hemiceras plusiata|611[1n]|BOLD:ACN0769
Zelomorpha bobandersoni|[335]|DHJPAR0063375|Hemiceras plusiata|631[0n]|BOLD:ACN0769
Zelomorpha bobandersoni|[336]|DHJPAR0062012|Hemiceras plusiata|633[1n]|BOLD:ACN0769
Zelomorpha bobandersoni|[337]|DHJPAR0009345|Hemiceras plusiata|573[2n]|BOLD:ACN0769
Zelomorpha bobandersoni|[338]|DHJPAR0028037|Hemiceras plusiata|627[0n]|BOLD:ACN0769
Zelomorpha bobandersoni|[339]|DHJPAR0057650|Hemiceras plusiata|658[1n]|BOLD:ACN0769
Zelomorpha bobandersoni|[340]|DHJPAR0009346|Hemiceras plusiata|658[1n]|BOLD:ACN0769
Zelomorpha johnchemsaki|[341]|DHJPAR0036324|Hemiceras pallidula|507[0n]|BOLD:ADD0338
Zelomorpha johnchemsaki|[342]|DHJPAR0036327|Hemiceras pallidula|622[5n]|BOLD:ADD0338
Zelomorpha johnchemsaki|[343]|DHJPAR0036326|Hemiceras pallidula|622[1n]|BOLD:ADD0338
Zelomorpha johnchemsaki|[344]|DHJPAR0036325|Hemiceras pallidula|622[1n]|BOLD:ADD0338
Zelomorpha johnchemsaki|[345]|DHJPAR0036328|Hemiceras pallidula|622[1n]|BOLD:ADD0338
Zelomorpha johnchemsaki|[346]|DHJPAR0023296|Hemiceras pallidula|658[2n]|BOLD:ADD0338
Zelomorpha johnchemsaki|[347]|DHJPAR0040546|Hemiceras pallidula|658[1n]|BOLD:ADD0338
Zelomorpha johnchemsaki|[348]|DHJPAR0036368|Hemiceras pallidula|658[1n]|BOLD:ADD0338
Zelomorpha johnchemsaki|[349]|DHJPAR0040541|Hemiceras pallidula|658[1n]|BOLD:ADD0338
Zelomorpha johnchemsaki|[350]|DHJPAR0040540|Hemiceras pallidula|658[1n]|BOLD:ADD0338
Zelomorpha johnchemsaki|[351]|DHJPAR0040543|Hemiceras pallidula|658[1n]|BOLD:ADD0338
Zelomorpha johnchemsaki|[352]|DHJPAR0040538|Hemiceras pallidula|658[1n]|BOLD:ADD0338
Zelomorpha johnchemsaki|[353]|DHJPAR0040536|Hemiceras pallidula|658[1n]|BOLD:ADD0338
Zelomorpha johnchemsaki|[354]|DHJPAR0040547|Hemiceras pallidula|640[1n]|BOLD:ADD0338
Zelomorpha johnchemsaki|[355]|DHJPAR0040535|Hemiceras pallidula|658[1n]|BOLD:ADD0338
Zelomorpha johnchemsaki|[356]|DHJPAR0040537|Hemiceras pallidula|658[1n]|BOLD:ADD0338
Zelomorpha johnchemsaki|[357]|DHJPAR0036369|Hemiceras pallidula|658[1n]|BOLD:ADD0338
Zelomorpha johnchemsaki|[358]|DHJPAR0040545|Hemiceras pallidula|658[1n]|BOLD:ADD0338
Zelomorpha johnchemsaki|[359]|DHJPAR0040542|Hemiceras pallidula|658[1n]|BOLD:ADD0338
Zelomorpha johnchemsaki|[360]|DHJPAR0040544|Hemiceras pallidula|658[1n]|BOLD:ADD0338
Zelomorpha johnchemsaki|[361]|DHJPAR0040539|Hemiceras pallidula|658[1n]|BOLD:ADD0338
Zelomorpha angelsolisi|[362]|DHJPAR0015578|Chabora repugnalisDHJ01|499[25n]
Zelomorpha angelsolisi|[363]|DHJPAR0015577|Chabora repugnalisDHJ01|551[16n]
Zelomorpha angelsolisi|[364]|DHJPAR0015592|Chabora repugnalisDHJ01|390[5n]
Zelomorpha angelsolisi|[365]|DHJPAR0015584|Chabora repugnalisDHJ01|600[0n]|BOLD:AAT9451
Zelomorpha angelsolisi|[366]|DHJPAR0009318|Azeta ceramina|606[3n]|BOLD:AAT9451
Zelomorpha angelsolisi|[367]|DHJPAR0009317|Azeta ceramina|621[0n]|BOLD:AAT9451
Zelomorpha angelsolisi|[368]|DHJPAR0015593|Chabora repugnalisDHJ01|618[0n]|BOLD:AAT9451
Zelomorpha angelsolisi|[369]|DHJPAR0009321|Azeta ceramina|618[0n]|BOLD:AAT9451
Zelomorpha angelsolisi|[370]|DHJPAR0029184|Coenipeta bibitrix|621[0n]|BOLD:AAT9451
Zelomorpha angelsolisi|[371]|DHJPAR0028275|Azeta ceramina|618[0n]|BOLD:AAT9451
Zelomorpha angelsolisi|[372]|DHJPAR0009310|Azeta ceramina|621[0n]|BOLD:AAT9451
Zelomorpha angelsolisi|[373]|DHJPAR0009313|Azeta ceramina|621[0n]|BOLD:AAT9451
Zelomorpha angelsolisi|[374]|DHJPAR0009319|Azeta ceramina|621[0n]|BOLD:AAT9451
Zelomorpha angelsolisi|[375]|DHJPAR0028276|Azeta ceramina|624[0n]|BOLD:AAT9451
Zelomorpha angelsolisi|[376]|BIOUG55293-G04|Malaise trap PL12-3D|654[0n]|BOLD:AAT9451
Zelomorpha angelsolisi|[377]|DHJPAR0021152|Azeta ceramina|594[0n]|BOLD:AAT9451
Zelomorpha angelsolisi|[378]|DHJPAR0015590|Coenipeta bibitrix|642[4n]|BOLD:AAT9451
Zelomorpha angelsolisi|[379]|BIOUG55221-C10|Malaise trap PL12-3D|654[0n]|BOLD:AAT9451
Zelomorpha angelsolisi|[380]|BIOUG55356-A08|Malaise trap PL12-9D|654[0n]|BOLD:AAT9451
Zelomorpha angelsolisi|[381]|BIOUG53713-E04|Malaise trap PL12-5C|654[0n]|BOLD:AAT9451
Zelomorpha angelsolisi|[382]|BIOUG55357-G01|Malaise trap PL12-9D|652[0n]|BOLD:AAT9451
Zelomorpha angelsolisi|[383]|BIOUG55356-F11|Malaise trap PL12-9D|654[0n]|BOLD:AAT9451
Zelomorpha angelsolisi|[384]|BIOUG52716-E05|Malaise trap PL12-9C|655[0n]|BOLD:AAT9451
Zelomorpha angelsolisi|[385]|BIOUG52715-B08|Malaise trap PL12-9C|654[0n]|BOLD:AAT9451
Zelomorpha angelsolisi|[386]|BIOUG52715-B10|Malaise trap PL12-9C|654[0n]|BOLD:AAT9451
Zelomorpha angelsolisi|[387]|BIOUG55358-D07|Malaise trap PL12-9D|655[0n]|BOLD:AAT9451
Zelomorpha angelsolisi|[388]|BIOUG51774-B03|Malaise trap PL12-3C|652[0n]|BOLD:AAT9451
Zelomorpha angelsolisi|[389]|BIOUG53714-E11|Malaise trap PL12-5C|653[0n]|BOLD:AAT9451
Zelomorpha angelsolisi|[390]|BIOUG55358-D11|Malaise trap PL12-9D|655[0n]|BOLD:AAT9451
Zelomorpha angelsolisi|[391]|BIOUG52716-E09|Malaise trap PL12-9C|654[0n]|BOLD:AAT9451
Zelomorpha angelsolisi|[392]|BIOUG55242-G07|Malaise trap PL12-5C|653[0n]|BOLD:AAT9451
Zelomorpha angelsolisi|[393]|BIOUG53713-E02|Malaise trap PL12-5C|654[0n]|BOLD:AAT9451
Zelomorpha angelsolisi|[394]|BIOUG53713-E06|Malaise trap PL12-5C|654[0n]|BOLD:AAT9451
Zelomorpha angelsolisi|[395]|BIOUG52715-B06|Malaise trap PL12-9C|654[0n]|BOLD:AAT9451
Zelomorpha angelsolisi|[396]|BIOUG52716-E08|Malaise trap PL12-9C|653[0n]|BOLD:AAT9451
Zelomorpha angelsolisi|[397]|BIOUG51774-A05|Malaise trap PL12-3C|651[0n]|BOLD:AAT9451
Zelomorpha angelsolisi|[398]|BIOUG55357-F12|Malaise trap PL12-9D|655[0n]|BOLD:AAT9451
Zelomorpha angelsolisi|[399]|BIOUG52720-B07|Malaise trap PL12-9C|651[0n]|BOLD:AAT9451

Zelomorpha angelsolisi|[397]|BIOUG51774-A05|Malaise trap PL12-3C|651[0n]|BOLD:AAT9451
Zelomorpha angelsolisi|[398]|BIOUG55357-F12|Malaise trap PL12-9D|655[0n]|BOLD:AAT9451
Zelomorpha angelsolisi|[399]|BIOUG52720-B07|Malaise trap PL12-9C|651[0n]|BOLD:AAT9451
Zelomorpha angelsolisi|[400]|BIOUG55331-E03|Malaise trap PL12-6D|651[0n]|BOLD:AAT9451
Zelomorpha angelsolisi|[401]|DHJPAR0040234|Macaria abydataDHJ01|651[0n]|BOLD:AAT9451
Zelomorpha angelsolisi|[402]|BIOUG53729-G01|Malaise trap PL12-3D|654[0n]|BOLD:AAT9451
Zelomorpha angelsolisi|[403]|DHJPAR0009315|Azeta ceramina|654[1n]|BOLD:AAT9451
Zelomorpha angelsolisi|[404]|DHJPAR0009312|Azeta ceramina|654[0n]|BOLD:AAT9451
Zelomorpha angelsolisi|[405]|BIOUG08539-A09|BSE Malaise Trap|658[0n]|BOLD:AAT9451
Zelomorpha angelsolisi|[406]|DHJPAR0015556|Coenipeta bibitrix|654[0n]|BOLD:AAT9451
Zelomorpha angelsolisi|[407]|DHJPAR0009316|Azeta ceramina|654[0n]|BOLD:AAT9451
Zelomorpha angelsolisi|[408]|DHJPAR0015588|Coenipeta bibitrix|654[1n]|BOLD:AAT9451
Zelomorpha angelsolisi|[409]|DHJPAR0009311|Azeta ceramina|654[3n]|BOLD:AAT9451
Zelomorpha angelsolisi|[410]|DHJPAR0009323|Azeta ceramina|654[3n]|BOLD:AAT9451
Zelomorpha angelsolisi|[411]|DHJPAR0009320|Azeta ceramina|579[2n]|BOLD:AAT9451
Zelomorpha angelsolisi|[412]|DHJPAR0029181|Azeta ceramina|282[0n]
Zelomorpha angelsolisi|[413]|DHJPAR0009314|Azeta ceramina|519[0n]|BOLD:AAT9451
Zelomorpha angelsolisi|[414]|DHJPAR0009322|Azeta ceramina|558[1n]|BOLD:AAT9451
Alabagrus jeanmariecadioui|[415]|DHJPAR0015507|Desmia ploralisDHJ02|654[0n]|BOLD:AAT9514
Alabagrus jeanmariecadioui|[416]|DHJPAR0052691|Desmia ploralisDHJ03|658[0n]|BOLD:AAT9514
Alabagrus jeanmariecadioui|[417]|DHJPAR0028030|Desmia ploralisDHJ03|658[0n]|BOLD:AAT9514
Alabagrus jeanmariecadioui|[418]|DHJPAR0048717|Desmia ploralisDHJ03|658[0n]|BOLD:AAT9514
Alabagrus jeanmariecadioui|[419]|DHJPAR0045797|Desmia ploralisDHJ03|658[0n]|BOLD:AAT9514
Alabagrus jeanmariecadioui|[420]|DHJPAR0037934|Desmia ploralisDHJ03|658[0n]|BOLD:AAT9514
Alabagrus jeanmariecadioui|[421]|DHJPAR0052695|Desmia ploralisDHJ03|626[1n]|BOLD:AAT9514
Alabagrus jeanmariecadioui|[422]|DHJPAR0037954|Desmia ploralisDHJ04|658[0n]|BOLD:AAT9514
Alabagrus jeanmariecadioui|[423]|DHJPAR0037905|Desmia Solis100|658[0n]|BOLD:AAT9514
Alabagrus jeanmariecadioui|[424]|DHJPAR0044987|Desmia ploralisDHJ03|658[0n]|BOLD:AAT9514
Alabagrus jeanmariecadioui|[425]|DHJPAR0044985|Desmia ploralisDHJ03|658[0n]|BOLD:AAT9514
Alabagrus jeanmariecadioui|[426]|DHJPAR0052690|Desmia ploralisDHJ03|658[0n]|BOLD:AAT9514
Alabagrus jeanmariecadioui|[427]|DHJPAR0044989|Desmia ploralisDHJ03|658[0n]|BOLD:AAT9514
Alabagrus jeanmariecadioui|[428]|DHJPAR0051346|Desmia ploralisDHJ01|658[0n]|BOLD:AAT9514
Alabagrus jeanmariecadioui|[429]|DHJPAR0023534|Desmia ploralisDHJ06|654[0n]|BOLD:AAT9514
Alabagrus jeanmariecadioui|[430]|DHJPAR0037893|Desmia Solis19|658[0n]|BOLD:AAT9514
Alabagrus jeanmariecadioui|[431]|DHJPAR0045796|Desmia ploralisDHJ01|658[0n]|BOLD:AAT9514
Alabagrus jeanmariecadioui|[432]|DHJPAR0038804|Desmia ploralisDHJ04|658[0n]|BOLD:AAT9514
Alabagrus jeanmariecadioui|[433]|DHJPAR0052084|Desmia ploralisDHJ03|658[0n]|BOLD:AAT9514
Alabagrus jeanmariecadioui|[434]|DHJPAR0053592|Desmia octomaculalis|658[0n]|BOLD:AAT9514
Alabagrus jeanmariecadioui|[435]|DHJPAR0048716|Desmia ploralisDHJ03|658[0n]|BOLD:AAT9514
Alabagrus jeanmariecadioui|[436]|DHJPAR0037901|Desmia Solis100|658[0n]|BOLD:AAT9514
Alabagrus jeanmariecadioui|[437]|DHJPAR0015508|Desmia Janzen09|654[0n]|BOLD:AAT9514
Alabagrus jeanmariecadioui|[438]|DHJPAR0036710|Desmia ploralisDHJ03|632[0n]|BOLD:AAT9514
Alabagrus jeanmariecadioui|[439]|DHJPAR0041190|Desmia Solis100|623[3n]|BOLD:AAT9514
Alabagrus jeanmariecadioui|[440]|DHJPAR0041193|Desmia ploralisDHJ03|624[0n]|BOLD:AAT9514
Alabagrus jeanmariecadioui|[441]|DHJPAR0009425|Desmia ploralisDHJ03|651[0n]|BOLD:AAT9514
Alabagrus jeanmariecadioui|[442]|DHJPAR0036687|Desmia ploralisDHJ03|627[0n]|BOLD:AAT9514
Alabagrus jeanmariecadioui|[443]|DHJPAR0041204|Desmia Solis100|624[1n]|BOLD:AAT9514
Alabagrus jeanmariecadioui|[444]|DHJPAR0041206|Desmia ploralisDHJ03|624[1n]|BOLD:AAT9514
Alabagrus jeanmariecadioui|[445]|DHJPAR0037903|Desmia Solis100|658[0n]|BOLD:AAT9514
Alabagrus jeanmariecadioui|[446]|DHJPAR0037904|Desmia Solis100|658[0n]|BOLD:AAT9514
Alabagrus jeanmariecadioui|[447]|DHJPAR0010513|Desmia ploralisDHJ03|658[0n]|BOLD:AAT9514
Alabagrus jeanmariecadioui|[448]|DHJPAR0015509|Desmia ploralisDHJ03|654[0n]|BOLD:AAT9514
Alabagrus jeanmariecadioui|[449]|DHJPAR0055969|Desmia ploralisDHJ03|670[0n]|BOLD:AAT9514
Alabagrus jeanmariecadioui|[450]|DHJPAR0045807|Desmia ploralisDHJ01|658[0n]|BOLD:AAT9514
Alabagrus jeanmariecadioui|[451]|DHJPAR0049656|Desmia ploralisDHJ03|658[0n]|BOLD:AAT9514
Alabagrus jeanmariecadioui|[452]|DHJPAR0046751|Desmia ploralisDHJ01|658[0n]|BOLD:AAT9514
Alabagrus jeanmariecadioui|[453]|DHJPAR0046752|Desmia ploralisDHJ01|658[0n]|BOLD:AAT9514
Alabagrus jeanmariecadioui|[454]|DHJPAR0048730|Desmia ploralisDHJ03|658[0n]|BOLD:AAT9514
Alabagrus jeanmariecadioui|[455]|DHJPAR0009430|Desmia ploralisDHJ03|624[0n]|BOLD:AAT9514
Alabagrus jeanmariecadioui|[456]|DHJPAR0009427|Desmia ploralisDHJ03|624[0n]|BOLD:AAT9514
Alabagrus jeanmariecadioui|[457]|DHJPAR0009424|Desmia Janzen09|651[0n]|BOLD:AAT9514
Alabagrus jeanmariecadioui|[458]|DHJPAR0009416|Desmia ploralisDHJ03|651[0n]|BOLD:AAT9514
Alabagrus jeanmariecadioui|[459]|DHJPAR0009429|Desmia ploralisDHJ03|651[0n]|BOLD:AAT9514
Alabagrus jeanmariecadioui|[460]|DHJPAR0009428|Desmia ploralisDHJ03|651[0n]|BOLD:AAT9514
Alabagrus jeanmariecadioui|[461]|DHJPAR0036704|Desmia ploralisDHJ03|625[0n]|BOLD:AAT9514
Alabagrus jeanmariecadioui|[462]|DHJPAR0037921|Desmia Solis100|658[0n]|BOLD:AAT9514
Alabagrus jeanmariecadioui|[463]|DHJPAR0036702|Desmia Solis100|658[0n]|BOLD:AAT9514
Alabagrus jeanmariecadioui|[464]|DHJPAR0045806|Desmia ploralisDHJ01|658[0n]|BOLD:AAT9514
Alabagrus jeanmariecadioui|[465]|DHJPAR0037918|Desmia ploralisDHJ04|658[0n]|BOLD:AAT9514
Alabagrus jackiemillerae|[466]|DHJPAR0057450|Rhectocraspeda Solis05|658[4n]
Alabagrus jackiemillerae|[467]|DHJPAR0037886|Herpetogramma Solis11|658[2n]|BOLD:ACD1741
Alabagrus jackiemillerae|[468]|DHJPAR0048729|Desmia Solis19|658[0n]|BOLD:ACD1741
Alabagrus jackiemillerae|[469]|DHJPAR0057448|Desmia Solis19|658[0n]|BOLD:ACD1741
Alabagrus jackiemillerae|[470]|DHJPAR0052682|Patania Solis03|658[0n]|BOLD:ACD1741
Alabagrus jackiemillerae|[471]|DHJPAR0053593|Desmia Janzen07|658[0n]|BOLD:ACD1741
Alabagrus jackiemillerae|[472]|DHJPAR0045805|spiloBioLep01 BioLep403|658[0n]|BOLD:ACD1741
Alabagrus jackiemillerae|[473]|DHJPAR0041165|Desmia Solis19|583[0n]|BOLD:ACD1741
Alabagrus jackiemillerae|[474]|DHJPAR0051668|Phostria Janzen05|613[2n]|BOLD:ACD1741
Alabagrus jackiemillerae|[475]|DHJPAR0053596|Herpetogramma Solis11|658[0n]|BOLD:ACD1741
Alabagrus jackiemillerae|[476]|DHJPAR0045803|spiloBioLep01 BioLep403|658[0n]|BOLD:ACD1741
Alabagrus jackiemillerae|[477]|DHJPAR0045802|spiloBioLep01 BioLep403|658[0n]|BOLD:ACD1741
Alabagrus jackiemillerae|[478]|DHJPAR0057446|Phostria Janzen05|658[1n]|BOLD:ACD1741
Alabagrus jackiemillerae|[479]|DHJPAR0046753|Pilocrocis purpurascens|658[1n]|BOLD:ACD1741
Alabagrus jackiemillerae|[480]|DHJPAR0038805|Herpetogramma Solis11|658[0n]|BOLD:ACD1741
Alabagrus jackiemillerae|[481]|DHJPAR0057439|Desmia Solis19|658[0n]|BOLD:ACD1741
Alabagrus jackiemillerae|[482]|DHJPAR0057441|Desmia Solis19|658[0n]|BOLD:ACD1741
Alabagrus jackiemillerae|[483]|DHJPAR0057445|Rhectocraspeda Solis05|658[0n]|BOLD:ACD1741
Alabagrus jackiemillerae|[484]|DHJPAR0057440|Desmia Solis19|658[0n]|BOLD:ACD1741
Alabagrus jackiemillerae|[485]|DHJPAR0054491|Rhectocraspeda Janzen347|658[0n]|BOLD:ACD1741
Alabagrus jackiemillerae|[486]|DHJPAR0052078|Herpetogramma Solis11|658[0n]|BOLD:ACD1741
Alabagrus jackiemillerae|[487]|DHJPAR0054492|Rhectocraspeda Janzen347|658[0n]|BOLD:ACD1741
Alabagrus jackiemillerae|[488]|DHJPAR0045798|Herpetogramma salbialis|658[0n]|BOLD:ACD1741
Alabagrus jackiemillerae|[489]|DHJPAR0037895|Desmia Solis19|658[1n]|BOLD:ACD1741
Alabagrus fernandodiasi|[490]|DHJPAR0050360|Desmia benealisDHJ02|658[0n]|BOLD:ACD1742
Alabagrus fernandodiasi|[491]|DHJPAR0048732|Herpetogramma Solis11|658[0n]|BOLD:ACD1742
Alabagrus fernandodiasi|[492]|DHJPAR0046750|Rhectocraspeda periusalis|658[0n]|BOLD:ACD1742
Alabagrus fernandodiasi|[493]|DHJPAR0054488|Herpetogramma Solis11|658[0n]|BOLD:ACD1742
Alabagrus fernandodiasi|[494]|DHJPAR0055818|Herpetogramma Solis11|670[0n]|BOLD:ACD1742
Alabagrus fernandodiasi|[495]|DHJPAR0052085|Herpetogramma Solis11|658[1n]|BOLD:ACD1742
Alabagrus fernandodiasi|[496]|DHJPAR0045800|spiloBioLep01 BioLep402|658[0n]|BOLD:ACD1742
Alabagrus fernandodiasi|[497]|DHJPAR0009377|Herpetogramma Solis10|651[0n]|BOLD:ACD1742
Alabagrus fernandodiasi|[498]|DHJPAR0040215|Herpetogramma Solis11|651[0n]|BOLD:ACD1742
Alabagrus fernandodiasi|[499]|DHJPAR0048731|Herpetogramma Solis11|658[0n]|BOLD:ACD1742

Alabagrus fernandodiasi|[496]|DHJPAR0045800|spiloBioLep01 BioLep402|658[0n]|BOLD:ACD1742
Alabagrus fernandodiasi|[497]|DHJPAR0009377|Herpetogramma Solis10|651[0n]|BOLD:ACD1742
Alabagrus fernandodiasi|[498]|DHJPAR0040215|Herpetogramma Solis11|651[0n]|BOLD:ACD1742
Alabagrus fernandodiasi|[499]|DHJPAR0048731|Herpetogramma Solis11|658[0n]|BOLD:ACD1742
Alabagrus fernandodiasi|[500]|DHJPAR0057002|spiloBioLep01 BioLep415|658[0n]|BOLD:ACD1742
Alabagrus fernandodiasi|[501]|DHJPAR0055236|Herpetogramma Solis11|658[0n]|BOLD:ACD1742
Alabagrus fernandodiasi|[502]|DHJPAR0037883|Rhectocraspeda Janzen347|658[0n]|BOLD:ACD1742
Alabagrus fernandodiasi|[503]|DHJPAR0042804|Rhectocraspeda periusalis|658[0n]|BOLD:ACD1742
Alabagrus fernandodiasi|[504]|DHJPAR0044991|spiloBioLep01 BioLep403|658[0n]|BOLD:ACD1742
Alabagrus fernandodiasi|[505]|DHJPAR0045801|Herpetogramma Solis11|658[0n]|BOLD:ACD1742
Alabagrus fernandodiasi|[506]|DHJPAR0052080|spiloBioLep01 BioLep414|658[0n]|BOLD:ACD1742
Alabagrus fernandodiasi|[507]|DHJPAR0055088|Herpetogramma Solis11|658[0n]|BOLD:ACD1742
Alabagrus fernandodiasi|[508]|DHJPAR0017273|Herpetogramma Solis11|654[0n]|BOLD:ACD1742
Alabagrus fernandodiasi|[509]|DHJPAR0052090|Desmia Janzen19|658[1n]|BOLD:ACD1742
Alabagrus fernandodiasi|[510]|DHJPAR0054490|spiloBioLep01 BioLep243|658[1n]|BOLD:ACD1742
Alabagrus fernandodiasi|[511]|DHJPAR0056299|Rhectocraspeda Solis05|658[0n]|BOLD:ACD1742
Alabagrus fernandodiasi|[512]|DHJPAR0045804|spiloBioLep01 BioLep403|625[1n]|BOLD:ACD1742
Alabagrus fernandodiasi|[513]|DHJPAR0045799|spiloBioLep01 BioLep402|633[0n]|BOLD:ACD1742
Alabagrus fernandodiasi|[514]|DHJPAR0037890|Rhectocraspeda Janzen347|604[4n]
Alabagrus fernandodiasi|[515]|DHJPAR0021198|Herpetogramma Solis11|654[0n]|BOLD:ACD1742
Alabagrus fernandodiasi|[516]|DHJPAR0055098|Rhectocraspeda periusalis|658[2n]|BOLD:ACD1742
Alabagrus fernandodiasi|[517]|DHJPAR0052081|spiloBioLep01 BioLep414|658[3n]|BOLD:ACD1742
Alabagrus fernandodiasi|[518]|DHJPAR0052089|spiloBioLep01 BioLep311|658[0n]|BOLD:ACD1742
Alabagrus fernandodiasi|[519]|DHJPAR0055099|Rhectocraspeda periusalis|658[0n]|BOLD:ACD1742
Alabagrus combos|[520]|DHJPAR0046739|Herpetogramma Solis10|658[0n]|BOLD:ACD1804
Alabagrus combos|[521]|DHJPAR0046734|Herpetogramma Solis10|658[0n]|BOLD:ACD1804
Alabagrus combos|[522]|DHJPAR0030609|Patania Solis03|654[0n]|BOLD:ACD1804
Alabagrus combos|[523]|DHJPAR0057437|Herpetogramma salbialis|658[0n]|BOLD:ACD1804
Alabagrus combos|[524]|DHJPAR0036376|Ategumia lotanalisDHJ09|658[2n]|BOLD:ACD1804
Alabagrus combos|[525]|DHJPAR0015530|Patania Solis03|654[0n]|BOLD:ACD1804
Alabagrus combos|[526]|DHJPAR0054498|Ategumia lotanalis|658[4n]
Alabagrus combos|[527]|DHJPAR0050368|Herpetogramma salbialis|658[1n]|BOLD:ACD1804
Alabagrus combos|[528]|DHJPAR0050372|Ategumia lotanalisDHJ09|658[0n]|BOLD:ACD1804
Alabagrus combos|[529]|DHJPAR0036341|Ategumia lotanalisDHJ09|658[13n]|BOLD:ACD1804
Alabagrus combos|[530]|DHJPAR0057438|Herpetogramma salbialis|658[0n]|BOLD:ACD1804
Alabagrus combos|[531]|DHJPAR0057427|Ategumia lotanalis|658[0n]|BOLD:ACD1804
Alabagrus combos|[532]|DHJPAR0017276|Conchylodes platinalis|654[0n]|BOLD:ACD1804
Alabagrus combos|[533]|DHJPAR0038812|Rhectocraspeda periusalis|658[0n]|BOLD:ACD1804
Alabagrus combos|[534]|DHJPAR0037914|Ategumia matutinalisDHJ03|658[0n]|BOLD:ACD1804
Alabagrus combos|[535]|DHJPAR0037891|Herpetogramma salbialis|658[0n]|BOLD:ACD1804
Alabagrus combos|[536]|DHJPAR0041185|Herpetogramma salbialis|624[4n]
Alabagrus combos|[537]|DHJPAR0037958|Herpetogramma salbialis|658[0n]|BOLD:ACD1804
Alabagrus combos|[538]|DHJPAR0046735|Ategumia lotanalisDHJ09|658[1n]|BOLD:ACD1804
Alabagrus combos|[539]|DHJPAR0051383|Patania Solis03|658[0n]|BOLD:ACD1804
Alabagrus combos|[540]|DHJPAR0058538|Ategumia lotanalis|658[0n]|BOLD:ACD1804
Alabagrus combos|[541]|DHJPAR0037884|Herpetogramma salbialis|658[0n]|BOLD:ACD1804
Alabagrus combos|[542]|DHJPAR0037897|Ategumia lotanalis|658[0n]|BOLD:ACD1804
Alabagrus combos|[543]|DHJPAR0037878|Herpetogramma salbialis|658[0n]|BOLD:ACD1804
Alabagrus combos|[544]|DHJPAR0038807|Rhectocraspeda periusalis|658[0n]|BOLD:ACD1804
Alabagrus combos|[545]|DHJPAR0051384|Patania Solis03|658[0n]|BOLD:ACD1804
Alabagrus combos|[546]|DHJPAR0037898|Ategumia lotanalis|658[0n]|BOLD:ACD1804
Alabagrus combos|[547]|DHJPAR0055974|Ategumia matutinalis|670[0n]|BOLD:ACD1804
Alabagrus combos|[548]|DHJPAR0037929|Herpetogramma salbialis|658[0n]|BOLD:ACD1804
Alabagrus combos|[549]|DHJPAR0042826|spiloJanzen01 Janzen14DHJ02|658[0n]|BOLD:ACD1804
Alabagrus combos|[550]|DHJPAR0049475|Patania Solis03|658[0n]|BOLD:ACD1804
Alabagrus combos|[551]|DHJPAR0037879|Herpetogramma salbialis|658[0n]|BOLD:ACD1804
Alabagrus combos|[552]|DHJPAR0037955|Ategumia matutinalisDHJ03|658[0n]|BOLD:ACD1804
Alabagrus combos|[553]|DHJPAR0036377|Ategumia lotanalisDHJ09|658[1n]|BOLD:ACD1804
Alabagrus combos|[554]|DHJPAR0041598|Herpetogramma salbialis|658[0n]|BOLD:ACD1804
Alabagrus combos|[555]|DHJPAR0041596|Herpetogramma salbialis|658[0n]|BOLD:ACD1804
Alabagrus combos|[556]|DHJPAR0053607|Herpetogramma salbialis|658[1n]|BOLD:ACD1804
Alabagrus combos|[557]|DHJPAR0041562|Herpetogramma salbialis|658[0n]|BOLD:ACD1804
Alabagrus combos|[558]|DHJPAR0053635|Herpetogramma salbialis|658[0n]|BOLD:ACD1804
Alabagrus combos|[559]|DHJPAR0035295|Ategumia lotanalisDHJ09|654[0n]|BOLD:ACD1804
Alabagrus combos|[560]|DHJPAR0056358|Ategumia lotanalis|658[0n]|BOLD:ACD1804
Alabagrus combos|[561]|DHJPAR0055975|Ategumia matutinalis|670[0n]|BOLD:ACD1804
Alabagrus combos|[562]|DHJPAR0051350|Patania Solis03|626[0n]|BOLD:ACD1804
Alabagrus combos|[563]|DHJPAR0051354|Patania Solis03|658[1n]|BOLD:ACD1804
Alabagrus combos|[564]|DHJPAR0035529|Pycnarmon BioLep66|658[0n]|BOLD:ACD1804
Alabagrus combos|[565]|DHJPAR0054506|Rhectocraspeda periusalis|658[0n]|BOLD:ACD1804
Alabagrus combos|[566]|DHJPAR0042819|Ategumia lotanalisDHJ08|630[0n]|BOLD:ACD1804
Alabagrus combos|[567]|DHJPAR0041583|Herpetogramma salbialis|658[0n]|BOLD:ACD1804
Alabagrus combos|[568]|DHJPAR0009361|Patania Solis03|651[0n]|BOLD:ACD1804
Alabagrus combos|[569]|DHJPAR0037896|Ategumia matutinalisDHJ03|658[0n]|BOLD:ACD1804
Alabagrus combos|[570]|DHJPAR0037877|Herpetogramma salbialis|658[0n]|BOLD:ACD1804
Alabagrus keithwillmotti|[571]|DHJPAR0037912|Desmia Janzen07|658[0n]|BOLD:ACJ2565
Alabagrus keithwillmotti|[572]|DHJPAR0009389|Pilocrocis Solis20|654[4n]|BOLD:ACJ2565
Alabagrus keithwillmotti|[573]|DHJPAR0009390|Pilocrocis Solis20|651[3n]|BOLD:ACJ2565
Alabagrus keithwillmotti|[574]|DHJPAR0009386|Pilocrocis Solis20|648[5n]|BOLD:ACJ2565
Alabagrus keithwillmotti|[575]|DHJPAR0037907|Desmia Janzen07|658[0n]|BOLD:ACJ2565
Alabagrus keithwillmotti|[576]|DHJPAR0037909|Desmia Janzen07|658[0n]|BOLD:ACJ2565
Alabagrus keithwillmotti|[577]|DHJPAR0037911|Desmia Janzen07|658[0n]|BOLD:ACJ2565
Alabagrus keithwillmotti|[578]|DHJPAR0037913|Desmia Janzen07|658[0n]|BOLD:ACJ2565
Alabagrus keithwillmotti|[579]|DHJPAR0037910|Desmia Janzen07|658[0n]|BOLD:ACJ2565
Alabagrus keithwillmotti|[580]|DHJPAR0009387|Pilocrocis Solis20|654[1n]|BOLD:ACJ2565
Alabagrus keithwillmotti|[581]|DHJPAR0009388|Pilocrocis Solis20|654[1n]|BOLD:ACJ2565
Alabagrus keithwillmotti|[582]|DHJPAR0009385|Pilocrocis Solis20|654[6n]|BOLD:ACJ2565
Alabagrus keithwillmotti|[583]|DHJPAR0052730|Desmia Janzen07|658[1n]|BOLD:ACJ2565
Alabagrus keithwillmotti|[584]|DHJPAR0038808|Phostria Janzen03|658[0n]|BOLD:ACJ2565
Alabagrus keithwillmotti|[585]|DHJPAR0035227|spilobiolep01 biolep498|658[0n]|BOLD:ACJ2565
Alabagrus keithwillmotti|[586]|DHJPAR0050868|Desmia Janzen14|661[0n]|BOLD:ACJ2565
Alabagrus keithwillmotti|[587]|DHJPAR0038809|Asturodes fimbriauralisDHJ02|658[0n]|BOLD:ACJ2565
Alabagrus keithwillmotti|[588]|DHJPAR0045809|Herpetogramma Solis10|658[0n]|BOLD:ACJ2565
Alabagrus keithwillmotti|[589]|DHJPAR0045808|Herpetogramma Solis10|658[0n]|BOLD:ACJ2565
Alabagrus keithwillmotti|[590]|DHJPAR0036339|Asturodes fimbriauralisDHJ02|658[0n]|BOLD:ACJ2565
Alabagrus keithwillmotti|[591]|DHJPAR0045789|Herpetogramma Solis10|658[0n]|BOLD:ACJ2565
Alabagrus keithwillmotti|[592]|DHJPAR0039357|Desmia octomaculalis|658[0n]|BOLD:ACJ2565
Alabagrus keithwillmotti|[593]|DHJPAR0007207|Pilocrocis Solis20|658[0n]|BOLD:ACJ2565
Alabagrus paulheberti|[594]|DHJPAR0046953|Aponia minnithalis|658[0n]|BOLD:ACD1800
Alabagrus paulheberti|[595]|DHJPAR0046746|Aponia minnithalis|658[1n]|BOLD:ACD1800
Alabagrus paulheberti|[596]|DHJPAR0040223|Aponia itzalis|658[1n]|BOLD:ACD1800
Alabagrus donharveyi|[597]|DHJPAR0021163|Coelorhyncidia pandaralis|624[0n]|BOLD:ACD1739
Alabagrus donharveyi|[598]|DHJPAR0036711|Coenostolopsis apicalis|625[0n]|BOLD:ACD1739
[illegible]

Alabagrus paulheberti|[596]|DHJPAR0040223|Aponia itzalis|658[1n]|BOLD:ACD1800
Alabagrus donharveyi|[597]|DHJPAR0021163|Coelorhyncidia pandaralis|624[0n]|BOLD:ACD1739
Alabagrus donharveyi|[598]|DHJPAR0036711|Coenostolopsis apicalis|625[0n]|BOLD:ACD1739
Alabagrus donharveyi|[599]|DHJPAR0021165|Coelorhyncidia pandaralis|654[0n]|BOLD:ACD1739
Alabagrus donharveyi|[600]|DHJPAR0054513|Coenostolopsis apicalis|658[0n]|BOLD:ACD1739
Alabagrus donharveyi|[601]|DHJPAR0048734|Coenostolopsis apicalis|658[0n]|BOLD:ACD1739
Alabagrus donharveyi|[602]|DHJPAR0037930|Coenostolopsis apicalis|658[0n]|BOLD:ACD1739
Alabagrus donharveyi|[603]|DHJPAR0045810|Coenostolopsis apicalis|658[0n]|BOLD:ACD1739
Alabagrus donharveyi|[604]|DHJPAR0036688|Coenostolopsis apicalis|658[0n]|BOLD:ACD1739
Alabagrus donharveyi|[605]|DHJPAR0054512|Coenostolopsis apicalis|658[0n]|BOLD:ACD1739
Alabagrus bobpoolei|[606]|DHJPAR0042815|Eulepte Solis15|489[5n]|BOLD:ACD2038
Alabagrus bobpoolei|[607]|DHJPAR0038924|Eulepte Solis15|658[0n]|BOLD:ACD2038
Alabagrus bobpoolei|[608]|DHJPAR0048725|Eulepte Solis15|658[1n]|BOLD:ACD2038
Alabagrus bobpoolei|[609]|DHJPAR0045785|Eulepte Solis15|658[0n]|BOLD:ACD2038
Alabagrus bobpoolei|[610]|DHJPAR0054495|Eulepte Solis15|658[0n]|BOLD:ACD2038
Alabagrus bobpoolei|[611]|DHJPAR0042813|Eulepte Solis15|658[2n]|BOLD:ACD2038
Alabagrus bobpoolei|[612]|DHJPAR0042805|Eulepte Solis15|658[1n]|BOLD:ACD2038
Alabagrus bobpoolei|[613]|DHJPAR0054514|Eulepte Solis15|658[0n]|BOLD:ACD2038
Alabagrus bobpoolei|[614]|DHJPAR0051382|Eulepte Solis15|658[1n]|BOLD:ACD2038
Alabagrus bobpoolei|[615]|DHJPAR0042828|Eulepte Solis15|658[0n]|BOLD:ACD2038
Alabagrus bobpoolei|[616]|DHJPAR0042814|Eulepte Solis15|658[0n]|BOLD:ACD2038
Alabagrus bobpoolei|[617]|DHJPAR0048727|Eulepte Solis15|658[0n]|BOLD:ACD2038
Alabagrus bobpoolei|[618]|DHJPAR0037887|Eulepte Solis15|632[1n]|BOLD:ACD2038
Alabagrus bobpoolei|[619]|DHJPAR0050364|Eulepte Solis15|658[0n]|BOLD:ACD2038
Alabagrus bobpoolei|[620]|DHJPAR0041169|Eulepte Janzen12|624[2n]|BOLD:ACD2038
Alabagrus bobpoolei|[621]|DHJPAR0036694|Eulepte Janzen06|633[0n]|BOLD:ACD2038
Alabagrus bobpoolei|[622]|DHJPAR0051380|Eulepte Solis15|658[0n]|BOLD:ACD2038
Alabagrus bobpoolei|[623]|DHJPAR0042811|Eulepte Solis15|658[2n]|BOLD:ACD2038
Alabagrus jennyphillipsae|[624]|DHJPAR0039523|Leucochromodes melusinalisDHJ01|658[0n]|BOLD:ADC...
Alabagrus jennyphillipsae|[625]|DHJPAR0035225|Leucochromodes melusinalisDHJ01|658[0n]|BOLD:ADC...
Alabagrus jennyphillipsae|[626]|DHJPAR0037923|Leucochromodes melusinalisDHJ01|658[1n]|BOLD:ADC...
Alabagrus isidrochaconi|[627]|DHJPAR0053608|Glyphodes sibillalisDHJ01|658[0n]|BOLD:ACJ5020
Alabagrus isidrochaconi|[628]|DHJPAR0053626|Glyphodes sibillalisDHJ01|658[0n]|BOLD:ACJ5020
Alabagrus isidrochaconi|[629]|DHJPAR0015520|Glyphodes sibillalisDHJ01|654[0n]|BOLD:ACJ5020
Alabagrus isidrochaconi|[630]|DHJPAR0052674|Glyphodes sibillalisDHJ01|658[0n]|BOLD:ACJ5020
Alabagrus isidrochaconi|[631]|DHJPAR0053609|Glyphodes sibillalisDHJ01|658[0n]|BOLD:ACJ5020
Alabagrus isidrochaconi|[632]|DHJPAR0053633|Glyphodes sibillalisDHJ01|658[0n]|BOLD:ACJ5020
Alabagrus isidrochaconi|[633]|DHJPAR0053630|Glyphodes sibillalisDHJ01|658[0n]|BOLD:ACJ5020
Alabagrus isidrochaconi|[634]|DHJPAR0015523|Glyphodes sibillalisDHJ01|654[0n]|BOLD:ACJ5020
Alabagrus isidrochaconi|[635]|DHJPAR0052683|Glyphodes sibillalisDHJ01|658[0n]|BOLD:ACJ5020
Alabagrus isidrochaconi|[636]|DHJPAR0053627|Glyphodes sibillalisDHJ01|626[0n]|BOLD:ACJ5020
Alabagrus isidrochaconi|[637]|DHJPAR0009405|Glyphodes sibillalisDHJ01|654[2n]|BOLD:ACJ5020
Alabagrus isidrochaconi|[638]|DHJPAR0015525|Eulepte Janzen03|654[0n]|BOLD:ACJ5020
Alabagrus isidrochaconi|[639]|DHJPAR0053624|Glyphodes sibillalisDHJ01|658[0n]|BOLD:ACJ5020
Alabagrus isidrochaconi|[640]|DHJPAR0053631|Glyphodes sibillalisDHJ01|658[0n]|BOLD:ACJ5020
Alabagrus isidrochaconi|[641]|DHJPAR0052684|Glyphodes sibillalisDHJ01|658[0n]|BOLD:ACJ5020
Alabagrus isidrochaconi|[642]|DHJPAR0052676|Glyphodes sibillalisDHJ01|658[0n]|BOLD:ACJ5020
Alabagrus isidrochaconi|[643]|DHJPAR0053629|Glyphodes sibillalisDHJ01|658[0n]|BOLD:ACJ5020
Alabagrus isidrochaconi|[644]|DHJPAR0052675|Glyphodes sibillalisDHJ01|658[0n]|BOLD:ACJ5020
Alabagrus isidrochaconi|[645]|DHJPAR0053634|Glyphodes sibillalisDHJ01|658[0n]|BOLD:ACJ5020
Alabagrus tanyadapkeyae|[646]|DHJPAR0061440|Phostria cyrisalis|658[19n]
Alabagrus tanyadapkeyae|[647]|DHJPAR0040225|Phostria cyrisalis|647[0n]|BOLD:ACR3678
Alabagrus tanyadapkeyae|[648]|DHJPAR0055970|Phostria cyrisalis|670[0n]|BOLD:ACR3678
Alabagrus tanyadapkeyae|[649]|DHJPAR0040218|Phostria cyrisalis|658[0n]|BOLD:ACR3678
Alabagrus tanyadapkeyae|[650]|DHJPAR0057429|Phostria cyrisalis|658[0n]|BOLD:ACR3678
Alabagrus jeanfrancoislandryi|[651]|DHJPAR0050932|Eulepte alialis|421[0n]|BOLD:ABA9277
Alabagrus jeanfrancoislandryi|[652]|DHJPAR0037900|Hyalorista exuvialisDHJ02|624[2n]|BOLD:ABA9277
Alabagrus jeanfrancoislandryi|[653]|DHJPAR0015522|Microthyris anormalis|657[1n]|BOLD:ABA9277
Alabagrus jeanfrancoislandryi|[654]|DHJPAR0041196|Eulepte alialis|622[3n]|BOLD:ABA9277
Alabagrus jeanfrancoislandryi|[655]|DHJPAR0045786|Eulepte alialis|622[0n]|BOLD:ABA9277
Alabagrus jeanfrancoislandryi|[656]|DHJPAR0009357|Eulepte alialis|630[4n]|BOLD:ABA9277
Alabagrus jeanfrancoislandryi|[657]|DHJPAR0057431|Orphanostigma haemorrhoidalis|658[1n]|BOLD:A...
Alabagrus jeanfrancoislandryi|[658]|DHJPAR0045783|Herpetogramma Solis11|658[0n]|BOLD:ABA9277
Alabagrus jeanfrancoislandryi|[659]|DHJPAR0057745|Eulepte alialis|647[0n]|BOLD:ABA9277
Alabagrus jeanfrancoislandryi|[660]|DHJPAR0009406|Conchylodes arcifera|624[0n]|BOLD:ABA9277
Alabagrus jeanfrancoislandryi|[661]|DHJPAR0041603|Herpetogramma Solis11|628[1n]|BOLD:ABA9277
Alabagrus jeanfrancoislandryi|[662]|DHJPAR0036703|Eulepte alialis|637[0n]|BOLD:ABA9277
Alabagrus jeanfrancoislandryi|[663]|DHJPAR0057430|Herpetogramma salbialis|622[0n]|BOLD:ABA9277
Alabagrus jeanfrancoislandryi|[664]|DHJPAR0009353|Eulepte alialis|627[0n]|BOLD:ABA9277
Alabagrus jeanfrancoislandryi|[665]|DHJPAR0037187|Eulepte alialis|654[0n]|BOLD:ABA9277
Alabagrus jeanfrancoislandryi|[666]|DHJPAR0056298|Eulepte alialis|658[0n]|BOLD:ABA9277
Alabagrus jeanfrancoislandryi|[667]|DHJPAR0037950|Eulepte alialis|658[0n]|BOLD:ABA9277
Alabagrus jeanfrancoislandryi|[668]|DHJPAR0045011|Herpetogramma salbialis|658[0n]|BOLD:ABA9277
Alabagrus jeanfrancoislandryi|[669]|DHJPAR0050370|Patania Solis03|658[0n]|BOLD:ABA9277
Alabagrus jeanfrancoislandryi|[670]|DHJPAR0045014|Herpetogramma salbialis|658[0n]|BOLD:ABA9277
Alabagrus jeanfrancoislandryi|[671]|DHJPAR0036356|Ategumia lotanalisDHJ09|658[1n]|BOLD:ABA9277
Alabagrus jeanfrancoislandryi|[672]|DHJPAR0015387|Ategumia lotanalisDHJ07|642[3n]|BOLD:ABA9277
Alabagrus jeanfrancoislandryi|[673]|DHJPAR0056296|Eulepte alialis|657[0n]|BOLD:ABA9277
Alabagrus jeanfrancoislandryi|[674]|DHJPAR0037953|Orphanostigma haemorrhoidalis|658[0n]|BOLD:A...
Alabagrus jeanfrancoislandryi|[675]|DHJPAR0037956|Eulepte alialis|658[0n]|BOLD:ABA9277
Alabagrus jeanfrancoislandryi|[676]|DHJPAR0036685|Eulepte alialis|626[0n]|BOLD:ABA9277
Alabagrus jeanfrancoislandryi|[677]|DHJPAR0009373|Eulepte alialis|654[1n]|BOLD:ABA9277
Alabagrus jeanfrancoislandryi|[678]|DHJPAR0057738|Eulepte alialis|658[4n]|BOLD:ABA9277
Alabagrus jeanfrancoislandryi|[679]|DHJPAR0037917|Eulepte alialis|658[0n]|BOLD:ABA9277
Alabagrus jeanfrancoislandryi|[680]|DHJPAR0037948|Eulepte alialis|658[0n]|BOLD:ABA9277
Alabagrus jeanfrancoislandryi|[681]|DHJPAR0053613|Orphanostigma haemorrhoidalis|658[0n]|BOLD:A...
Alabagrus jeanfrancoislandryi|[682]|DHJPAR0036700|Phostria samealis|606[0n]|BOLD:ABA9277
Alabagrus jeanfrancoislandryi|[683]|DHJPAR0056994|Herpetogramma salbialis|658[0n]|BOLD:ABA9277
Alabagrus jeanfrancoislandryi|[684]|DHJPAR0056999|Herpetogramma salbialis|658[0n]|BOLD:ABA9277
Alabagrus jeanfrancoislandryi|[685]|DHJPAR0030388|Ategumia lotanalis|621[3n]|BOLD:ABA9277
Alabagrus jeanfrancoislandryi|[686]|DHJPAR0042824|Herpetogramma salbialis|418[0n]|BOLD:ABA9277
Alabagrus jeanfrancoislandryi|[687]|DHJPAR0045015|Herpetogramma salbialis|621[3n]|BOLD:ABA9277
Alabagrus jeanfrancoislandryi|[688]|DHJPAR0049939|Eulepte alialis|658[10n]
Alabagrus jeanfrancoislandryi|[689]|DHJPAR0050065|Eulepte alialis|658[3n]|BOLD:ABA9277
Alabagrus jeanfrancoislandryi|[690]|DHJPAR0036374|Eulepte alialis|558[0n]|BOLD:ABA9277
Alabagrus jeanfrancoislandryi|[691]|DHJPAR0045004|Herpetogramma salbialis|656[4n]|BOLD:ABA9277
Alabagrus jeanfrancoislandryi|[692]|DHJPAR0052689|Eulepte alialis|658[0n]|BOLD:ABA9277
Alabagrus jeanfrancoislandryi|[693]|DHJPAR0045010|Herpetogramma salbialis|658[3n]|BOLD:ABA9277
Alabagrus jeanfrancoislandryi|[694]|DHJPAR0045012|Herpetogramma salbialis|597[1n]|BOLD:ABA9277
Alabagrus jeanfrancoislandryi|[695]|DHJPAR0045000|Eulepte alialis|658[1n]|BOLD:ABA9277
Alabagrus jeanfrancoislandryi|[696]|DHJPAR0052693|Eulepte alialis|359[4n]
Alabagrus jeanfrancoislandryi|[697]|DHJPAR0045006|Herpetogramma phaeopteralis|658[0n]|BOLD:ABA...
Alabagrus lindapitkinae|[698]|DHJPAR0052082|Omiodes Janzen05|659[0n]|BOLD:ACJ2111

Alabagrus jeanfrancoislandryi|[696]|DHJPAR0052693|Eulepte alialis|359[4n]
Alabagrus jeanfrancoislandryi|[697]|DHJPAR0045006|Herpetogramma phaeopteralis|658[0n]|BOLD:ABA...
Alabagrus lindapitkinae|[698]|DHJPAR0052082|Omiodes Janzen05|659[0n]|BOLD:ACJ2111
Alabagrus lindapitkinae|[699]|DHJPAR0054497|Omiodes Janzen05|658[0n]|BOLD:ACJ2111
Alabagrus watsoni|[700]|DHJPAR0028283|Omiodes humeralis|417[0n]|BOLD:ACN0949
Alabagrus watsoni|[701]|DHJPAR0023531|Omiodes humeralis|421[0n]|BOLD:ACN0949
Alabagrus watsoni|[702]|DHJPAR0028284|Omiodes humeralis|417[0n]|BOLD:ACN0949
Alabagrus watsoni|[703]|DHJPAR0028285|Omiodes humeralis|417[0n]|BOLD:ACN0949
Alabagrus watsoni|[704]|DHJPAR0042827|Omiodes insolutalis|421[0n]|BOLD:ACN0949
Alabagrus watsoni|[705]|DHJPAR0028282|Omiodes humeralis|417[0n]|BOLD:ACN0949
Alabagrus watsoni|[706]|DHJPAR0028286|Omiodes humeralis|417[0n]|BOLD:ACN0949
Alabagrus watsoni|[707]|DHJPAR0054496|Omiodes humeralis|658[0n]|BOLD:ACN0949
Alabagrus watsoni|[708]|DHJPAR0023530|Omiodes humeralisDHJ07|651[0n]|BOLD:ACN0949
Alabagrus watsoni|[709]|DHJPAR0023535|Omiodes humeralis|651[0n]|BOLD:ACN0949
Alabagrus watsoni|[710]|DHJPAR0028291|Omiodes humeralis|618[0n]|BOLD:ACN0949
Alabagrus watsoni|[711]|DHJPAR0021196|Omiodes humeralis|654[0n]|BOLD:ACN0949
Alabagrus watsoni|[712]|DHJPAR0037936|Omiodes humeralisDHJ02|658[0n]|BOLD:ACN0949
Alabagrus watsoni|[713]|DHJPAR0037915|Omiodes humeralis|658[0n]|BOLD:ACN0949
Alabagrus watsoni|[714]|DHJPAR0023300|Omiodes humeralis|654[1n]|BOLD:ACN0949
Alabagrus watsoni|[715]|DHJPAR0042825|Omiodes insolutalis|658[1n]|BOLD:ACN0949
Alabagrus watsoni|[716]|DHJPAR0015526|Omiodes humeralis|642[1n]|BOLD:ACN0949
Alabagrus watsoni|[717]|DHJPAR0022189|Omiodes humeralis|609[1n]|BOLD:ACN0949
Alabagrus watsoni|[718]|DHJPAR0015519|Omiodes humeralis|522[7n]
Alabagrus watsoni|[719]|DHJPAR0037937|Omiodes humeralis|658[0n]|BOLD:ACN0949
Alabagrus watsoni|[720]|DHJPAR0023292|Omiodes humeralis|377[0n]|BOLD:ACN0949
Alabagrus watsoni|[721]|DHJPAR0028308|Omiodes humeralis|387[0n]|BOLD:ACN0949
Alabagrus jimmilleri|[722]|DHJPAR0036373|Lygropia tripunctataDHJ04|626[0n]|BOLD:ACD1847
Alabagrus jimmilleri|[723]|DHJPAR0049046|Microthyris prolongalis|658[0n]|BOLD:ACD1847
Alabagrus jimmilleri|[724]|DHJPAR0049938|Microthyris prolongalisDHJ02|658[0n]|BOLD:ACD1847
Alabagrus jimmilleri|[725]|DHJPAR0009372|Palpusia Janzen02|651[0n]|BOLD:ACD1847
Alabagrus jimmilleri|[726]|DHJPAR0049946|Microthyris anormalisDHJ02|658[1n]|BOLD:ACD1847
Alabagrus jimmilleri|[727]|DHJPAR0040337|Microthyris prolongalis|621[0n]|BOLD:ACD1847
Alabagrus kaydodgeae|[728]|DHJPAR0053007|Ategumia Solis01|658[0n]|BOLD:AAW1640
Alabagrus cara|[729]|DHJPAR0058550|Desmia BioLep22DHJ02|530[1n]|BOLD:AAW1643
Alabagrus cara|[730]|BIOUG24991-C07|ESG Malaise Trap|501[0n]|BOLD:AAW1643
Alabagrus cara|[731]|DHJPAR0035232|Desmia ufeus|658[1n]|BOLD:AAW1643
Alabagrus cara|[732]|DHJPAR0052681|Desmia benealisDHJ03|658[1n]|BOLD:AAW1643
Alabagrus cara|[733]|DHJPAR0041579|Diacme BioLep02|658[0n]|BOLD:AAW1643
Alabagrus cara|[734]|BIOUG25233-E04|ESG Malaise Trap|652[1n]|BOLD:AAW1643
Alabagrus arua|[735]|DHJPAR0049652|Hyalorista exuvialis|612[9n]
Alabagrus arua|[736]|DHJPAR0035297|Hyalorista exuvialisDHJ02|658[0n]|BOLD:ACN1127
Alabagrus arua|[737]|DHJPAR0035224|Hyalorista exuvialisDHJ02|658[0n]|BOLD:ACN1127
Alabagrus arua|[738]|DHJPAR0049054|Hyalorista exuvialisDHJ02|658[0n]|BOLD:ACN1127
Alabagrus arua|[739]|DHJPAR0064508|Hyalorista exuvialis|658[2n]|BOLD:ACN1127
Alabagrus arua|[740]|DHJPAR0051379|Hyalorista exuvialisDHJ02|658[0n]|BOLD:ACN1127
Alabagrus arua|[741]|DHJPAR0021202|Hyalorista exuvialisDHJ02|654[0n]|BOLD:ACN1127
Alabagrus arua|[742]|DHJPAR0015518|Hyalorista exuvialisDHJ02|654[0n]|BOLD:ACN1127
Alabagrus arua|[743]|DHJPAR0030487|Hyalorista exuvialisDHJ02|658[0n]|BOLD:ACN1127
Alabagrus arua|[744]|DHJPAR0035296|Hyalorista exuvialisDHJ02|658[1n]|BOLD:ACN1127
Alabagrus johnburnsi|[745]|BIOUG27980-H02|ESG Malaise Trap Year 2|491[0n]
Alabagrus johnburnsi|[746]|BIOUG27999-A11|ESG Malaise Trap|516[0n]|BOLD:ACY1996
Alabagrus johnburnsi|[747]|BIOUG28160-D10|ESG Malaise Trap Year 2|498[0n]|BOLD:ACY1996
Alabagrus johnburnsi|[748]|BIOUG27868-B02|ESG Malaise Trap|498[0n]|BOLD:ACY1996
Alabagrus johnburnsi|[749]|BIOUG28111-D02|ESG Malaise Trap Year 2|516[0n]|BOLD:ACY1996
Alabagrus johnburnsi|[750]|BIOUG28111-D03|ESG Malaise Trap Year 2|495[0n]|BOLD:ACY1996
Alabagrus johnburnsi|[751]|BIOUG25233-D08|ESG Malaise Trap|504[0n]|BOLD:ACY1996
Alabagrus johnburnsi|[752]|BIOUG24991-C10|ESG Malaise Trap|504[0n]|BOLD:ACY1996
Alabagrus malcolmscoblei|[753]|DHJPAR0048718|Phostria latiapicalis|658[0n]|BOLD:ADC9541
Alabagrus iankitchingi|[754]|DHJPAR0054545|Phaedropsis Solis350DHJ02|658[0n]|BOLD:ADD0095
Alabagrus iankitchingi|[755]|DHJPAR0045001|Phaedropsis Solis350DHJ02|658[0n]|BOLD:ADD0095
Alabagrus nickgrishini|[756]|DHJPAR0037892|Microthyris alvinalis|658[2n]|BOLD:ACD1740
Alabagrus nickgrishini|[757]|DHJPAR0057425|Erilusa leucoplagialis|658[0n]|BOLD:ACD1740
Alabagrus nickgrishini|[758]|DHJPAR0050367|Patania Solis02|658[1n]|BOLD:ACD1740
Alabagrus nickgrishini|[759]|DHJPAR0045022|Microthyris alvinalis|658[0n]|BOLD:ACD1740
Alabagrus nickgrishini|[760]|DHJPAR0036347|Microthyris alvinalis|658[0n]|BOLD:ACD1740
Alabagrus nickgrishini|[761]|DHJPAR0050366|Patania Solis02|658[0n]|BOLD:ACD1740
Alabagrus markmetzi|[762]|DHJPAR0063798|Syllepte aechmisalis|627[1n]|BOLD:ACD1803
Alabagrus markmetzi|[763]|DHJPAR0009363|Dichocrocis sabatalis|651[0n]|BOLD:ACD1803
Alabagrus markmetzi|[764]|DHJPAR0064005|Syllepte aechmisalis|637[2n]|BOLD:ACD1803
Alabagrus markmetzi|[765]|DHJPAR0063999|Syllepte aechmisalis|637[0n]|BOLD:ACD1803
Alabagrus markmetzi|[766]|DHJPAR0015388|Dichocrocis sabatalis|654[0n]|BOLD:ACD1803
Alabagrus markmetzi|[767]|DHJPAR0009359|Dichocrocis sabatalis|651[0n]|BOLD:ACD1803
Alabagrus markmetzi|[768]|DHJPAR0046737|Dichocrocis sabatalis|658[5n]|BOLD:ACD1803
Alabagrus markmetzi|[769]|DHJPAR0009417|Dichocrocis sabatalis|648[1n]|BOLD:ACD1803
Alabagrus paulgoldsteini|[770]|DHJPAR0037908|Syllepte amandoDHJ02|658[0n]|BOLD:ADC9636
Alabagrus paulgoldsteini|[771]|DHJPAR0064392|Syllepte amando|653[1n]|BOLD:ADC9636
Alabagrus ekchuah|[772]|DHJPAR0015403|Syllepte amandoDHJ02|654[0n]|BOLD:ADC9606
Alabagrus ekchuah|[773]|DHJPAR0021168|Syllepte amandoDHJ02|658[0n]|BOLD:ADC9606
Alabagrus ekchuah|[774]|DHJPAR0015405|Pantographa suffusalis|453[1n]|BOLD:ADC9606
Alabagrus ekchuah|[775]|DHJPAR0015404|Pantographa suffusalis|603[5n]|BOLD:ADC9606
Alabagrus ekchuah|[776]|DHJPAR0064003|Pantographa suffusalis|530[0n]|BOLD:ADC9606
Alabagrus yuanmaofangi|[777]|DHJPAR0037899|Desmia Janzen09|621[0n]|BOLD:ADD0265
Alabagrus Sharkey9671|[778]|DHJPAR0042834|Desmia ploralisDHJ03|540[1n]|BOLD:ADC9671
Alabagrus Sharkey0055|[779]|DHJPAR0037894|Desmia Janzen09|658[1n]|BOLD:ACN0055
Alabagrus Sharkey0055|[780]|DHJPAR0053600|Desmia ploralisDHJ03|658[0n]|BOLD:ACN0055
Alabagrus Sharkey0055|[781]|DHJPAR0037941|Desmia BioLep06|658[0n]|BOLD:ACN0055
Alabagrus Sharkey0055|[782]|DHJPAR0036334|Desmia biolep02|658[0n]|BOLD:ACN0055
Alabagrus johnbrowni|[783]|DHJPAR0041591|Desmia tages|598[5n]
Alabagrus johnbrowni|[784]|DHJPAR0056297|Desmia BioLep19|658[2n]|BOLD:ACD1801
Alabagrus johnbrowni|[785]|DHJPAR0056357|Desmia BioLep19|658[0n]|BOLD:ACD1801
Alabagrus johnbrowni|[786]|DHJPAR0009378|Desmia tages|651[5n]
Alabagrus johnbrowni|[787]|DHJPAR0051669|Lamprosema Dapkey23|613[3n]
Alabagrus johnbrowni|[788]|DHJPAR0045782|Desmia tages|658[2n]|BOLD:ACD1801
Alabagrus johnbrowni|[789]|DHJPAR0052086|Piletosoma thialis|586[12n]
Alabagrus johnbrowni|[790]|DHJPAR0045058|Desmia BioLep09|658[0n]|BOLD:ACD1801
Alabagrus johnbrowni|[791]|DHJPAR0045059|Desmia BioLep09|644[0n]|BOLD:ACD1801
Alabagrus johnbrowni|[792]|DHJPAR0054531|Piletosoma thialis|658[0n]|BOLD:ACD1801
Alabagrus johnbrowni|[793]|DHJPAR0009364|Piletosoma thialisDHJ03|651[1n]|BOLD:ACD1801
Alabagrus johnbrowni|[794]|DHJPAR0035954|Piletosoma thialis|658[0n]|BOLD:ACD1801
Alabagrus johnbrowni|[795]|DHJPAR0015517|Piletosoma thialisDHJ03|642[4n]|BOLD:ACD1801
Alabagrus johnbrowni|[796]|DHJPAR0036336|Piletosoma thialis|658[0n]|BOLD:ACD1801
Alabagrus johnbrowni|[797]|DHJPAR0036707|Phostria Janzen05|658[0n]|BOLD:ACD1801
Alabagrus johnbrowni|[798]|DHJPAR0045029|Piletosoma thialis|631[1n]|BOLD:ACD1801

[illegible]
Alabagrus johnbrowni|[796]|DHJPAR0036336|Piletosoma thialis|658[0n]|BOLD:ACD1801
Alabagrus johnbrowni|[797]|DHJPAR0036707|Phostria Janzen05|658[0n]|BOLD:ACD1801
Alabagrus johnbrowni|[798]|DHJPAR0045029|Piletosoma thialis|631[1n]|BOLD:ACD1801
Alabagrus johnbrowni|[799]|DHJPAR0036335|Piletosoma thialis|636[0n]|BOLD:ACD1801
Alabagrus johnbrowni|[800]|DHJPAR0046755|Piletosoma thialis|658[4n]
Alabagrus johnbrowni|[801]|DHJPAR0017277|Piletosoma thialisDHJ03|654[1n]|BOLD:ACD1801
Alabagrus johnbrowni|[802]|DHJPAR0040069|Piletosoma thialis|658[2n]|BOLD:ACD1801
Alabagrus johnbrowni|[803]|DHJPAR0035226|Piletosoma thialis|643[2n]|BOLD:ACD1801
Alabagrus johnbrowni|[804]|DHJPAR0036375|Piletosoma thialis|608[0n]|BOLD:ACD1801
Alabagrus johnbrowni|[805]|DHJPAR0009407|Piletosoma thialisDHJ03|651[0n]|BOLD:ACD1801
Alabagrus johnbrowni|[806]|DHJPAR0015516|Piletosoma thialisDHJ03|654[0n]|BOLD:ACD1801
Alabagrus johnbrowni|[807]|DHJPAR0055980|Desmia BioLep42|670[0n]|BOLD:ACD1801
Alabagrus johnbrowni|[808]|DHJPAR0046748|Piletosoma thialis|658[1n]|BOLD:ACD1801
Alabagrus johnbrowni|[809]|DHJPAR0046749|Piletosoma thialis|412[0n]|BOLD:ACD1801
Alabagrus johnbrowni|[810]|DHJPAR0046754|Piletosoma thialis|658[0n]|BOLD:ACD1801
Alabagrus johnbrowni|[811]|DHJPAR0057743|Desmia BioLep42|658[0n]|BOLD:ACD1801
Alabagrus cocto|[812]|DHJPAR0053599|Phostria vitrifera|604[23n]
Alabagrus cocto|[813]|DHJPAR0045784|Orphanostigma haemorrhoidalis|629[3n]
Alabagrus cocto|[814]|DHJPAR0062014|Phostria vitrifera|658[0n]|BOLD:ACD1799
Alabagrus cocto|[815]|DHJPAR0063799|Syllepte Solis22|628[0n]|BOLD:ACD1799
Alabagrus cocto|[816]|DHJPAR0021151|Conchylodes arcifera|654[0n]|BOLD:ACD1799
Alabagrus cocto|[817]|DHJPAR0053601|Orphanostigma haemorrhoidalis|658[1n]|BOLD:ACD1799
Alabagrus cocto|[818]|DHJPAR0053603|Desmia ploralisDHJ01|621[2n]|BOLD:ACD1799
Alabagrus cocto|[819]|DHJPAR0030383|Herpetogramma salbialis|658[0n]|BOLD:ACD1799
Alabagrus cocto|[820]|DHJPAR0063364|Syllepte Solis22|631[0n]|BOLD:ACD1799
Alabagrus cocto|[821]|DHJPAR0040345|Syllepte Solis22|658[0n]|BOLD:ACD1799
Alabagrus cocto|[822]|DHJPAR0041594|Syllepte Solis22|658[0n]|BOLD:ACD1799
Alabagrus cocto|[823]|DHJPAR0050361|Syllepte Solis22|658[1n]|BOLD:ACD1799
Alabagrus cocto|[824]|DHJPAR0053598|Orphanostigma haemorrhoidalis|658[1n]|BOLD:ACD1799
Alabagrus cocto|[825]|DHJPAR0042356|Syllepte Solis22|658[2n]|BOLD:ACD1799
Alabagrus cocto|[826]|DHJPAR0048715|Desmia ploralisDHJ03|658[0n]|BOLD:ACD1799
Alabagrus cocto|[827]|DHJPAR0016927|Conchylodes grammaphora|654[0n]|BOLD:ACD1799
Alabagrus andresfreitasi|[828]|DHJPAR0050365|Phostria truncatalis|658[0n]|BOLD:ACD1743
Alabagrus andresfreitasi|[829]|DHJPAR0037959|Phostria truncatalis|658[0n]|BOLD:ACD1743
Alabagrus andresfreitasi|[830]|DHJPAR0036714|Phostria truncatalis|658[0n]|BOLD:ACD1743
Alabagrus andresfreitasi|[831]|DHJPAR0037880|Phostria truncatalis|598[1n]|BOLD:ACD1743
Alabagrus andywarreni|[832]|DHJPAR0041199|Phostria metalobalis|632[0n]|BOLD:ADC9708
Alabagrus andywarreni|[833]|DHJPAR0015471|Phostria truncatalis|651[1n]|BOLD:ADC9708
Alabagrus andywarreni|[834]|DHJPAR0037906|Phostria metalobalis|658[0n]|BOLD:ADC9708
Alabagrus andywarreni|[835]|DHJPAR0044988|Phostria metalobalis|632[0n]|BOLD:ADC9708
Alabagrus andywarreni|[836]|DHJPAR0015472|Phostria truncatalis|654[1n]|BOLD:ADC9708
Alabagrus axelhausmanni|[837]|DHJPAR0042832|musoBioLep01 BioLep448|658[0n]|BOLD:ADC9637
Alabagrus donlafontainei|[838]|DHJPAR0038928|musoBioLep01 BioLep448|658[0n]|BOLD:ACD1997
Alabagrus donlafontainei|[839]|DHJPAR0038927|musoBioLep01 BioLep448|658[0n]|BOLD:ACD1997
Alabagrus donlafontainei|[840]|DHJPAR0038929|musoBioLep01 BioLep448|658[0n]|BOLD:ACD1997
Alabagrus donlafontainei|[841]|DHJPAR0038923|musoBioLep01 BioLep448|658[0n]|BOLD:ACD1997
Alabagrus donlafontainei|[842]|DHJPAR0050137|musoBioLep01 BioLep448|613[0n]|BOLD:ACD1997
Alabagrus scottmilleri|[843]|DHJPAR0052088|Neurophyseta camptogrammalisDHJ01|658[3n]|BOLD:ACJ2110
Alabagrus scottmilleri|[844]|DHJPAR0030469|Neurophyseta Janzen222|658[0n]|BOLD:ACJ2110
Alabagrus scottmilleri|[845]|DHJPAR0037942|Neurophyseta BioLep219|658[0n]|BOLD:ACJ2110
Alabagrus cuna|[846]|DHJPAR0042807|Neurophyseta BioLep226|628[0n]|BOLD:ACN9714
Alabagrus cuna|[847]|DHJPAR0054521|Neurophyseta BioLep226|658[0n]|BOLD:ACN9714
Alabagrus cuna|[848]|DHJPAR0054525|Neurophyseta BioLep226|658[0n]|BOLD:ACN9714
Alabagrus cuna|[849]|DHJPAR0049047|Neurophyseta BioLep226|658[0n]|BOLD:ACN9714
Alabagrus cuna|[850]|DHJPAR0054535|Neurophyseta BioLep226|658[0n]|BOLD:ACN9714
Alabagrus cuna|[851]|DHJPAR0043025|Neurophyseta BioLep226|658[1n]|BOLD:ACN9714
Alabagrus cuna|[852]|DHJPAR0049045|Neurophyseta BioLep226|658[0n]|BOLD:ACN9714
Alabagrus cuna|[853]|DHJPAR0043033|Neurophyseta BioLep226|657[0n]|BOLD:ACN9714
Alabagrus bobrobbinsi|[854]|DHJPAR0049657|Microthyris prolongalisDHJ02|658[8n]
Alabagrus bobrobbinsi|[855]|DHJPAR0045007|Microthyris prolongalisDHJ02|658[1n]|BOLD:ADC9607
Alabagrus bobrobbinsi|[856]|DHJPAR0045008|Microthyris prolongalisDHJ02|658[2n]|BOLD:ADC9607
Alabagrus bobrobbinsi|[857]|DHJPAR0037881|Microthyris anormalisDHJ02|287[1n]
Alabagrus karensharkeyae|[858]|DHJPAR0010196|Microthyris prolongalis|658[0n]|BOLD:ADC9430
Alabagrus karensharkeyae|[859]|DHJPAR0041200|Microthyris prolongalis|624[0n]|BOLD:ADC9430
Alabagrus karensharkeyae|[860]|DHJPAR0036706|Microthyris prolongalis|626[0n]|BOLD:ADC9430
Alabagrus karensharkeyae|[861]|DHJPAR0036715|Microthyris prolongalis|635[0n]|BOLD:ACJ5330
Alabagrus karensharkeyae|[862]|DHJPAR0036348|Microthyris prolongalis|616[0n]|BOLD:ACJ5330
Alabagrus karensharkeyae|[863]|DHJPAR0052904|Microthyris prolongalis|658[0n]|BOLD:ACJ5330
Alabagrus karensharkeyae|[864]|DHJPAR0055978|Palpusia Janzen02|670[0n]|BOLD:ADC9429
Alabagrus nicoya|[865]|DHJPAR0015399|Dichogama colotha|654[3n]|BOLD:ACD2045
Alabagrus nicoya|[866]|DHJPAR0015400|Dichogama colotha|627[3n]|BOLD:ACD2045
Alabagrus nicoya|[867]|DHJPAR0049650|Dichogama colotha|658[1n]|BOLD:ACD2045
Alabagrus nicoya|[868]|DHJPAR0022193|Dichogama colotha|654[0n]|BOLD:ACD2045
Alabagrus nicoya|[869]|DHJPAR0016931|Dichogama colotha|651[0n]|BOLD:ACD2045
Alabagrus nicoya|[870]|DHJPAR0055976|Dichogama redtenbacheri|670[0n]|BOLD:ACD2045
Alabagrus nicoya|[871]|DHJPAR0063378|Dichogama colotha|629[0n]|BOLD:ACD2045
Alabagrus nicoya|[872]|DHJPAR0055977|Dichogama redtenbacheri|670[0n]|BOLD:ACD2045
Alabagrus nicoya|[873]|DHJPAR0055972|Dichogama redtenbacheri|670[0n]|BOLD:ACD2045
Alabagrus paulthiaucourti|[874]|DHJPAR0045016|Diatraea Janzen989|632[0n]|BOLD:ADC9342
Alabagrus almasolisae|[875]|DHJPAR0063805|Ceratocilia sixolalis|251[3n]
Alabagrus almasolisae|[876]|DHJPAR0063371|Ceratocilia sixolalis|239[1n]
Alabagrus almasolisae|[877]|DHJPAR0061021|Ceratocilia sixolalis|445[11n]
Alabagrus almasolisae|[878]|DHJPAR0054493|Ceratocilia sixolalis|602[13n]
Alabagrus almasolisae|[879]|DHJPAR0063800|Ceratocilia sixolalis|422[4n]|BOLD:ACN0056
Alabagrus almasolisae|[880]|DHJPAR0058544|Ceratocilia sixolalis|531[0n]|BOLD:ACN0056
Alabagrus almasolisae|[881]|DHJPAR0058543|Ceratocilia sixolalis|531[0n]|BOLD:ACN0056
Alabagrus almasolisae|[882]|DHJPAR0041205|Ceratocilia sixolalis|624[0n]|BOLD:ACN0056
Alabagrus almasolisae|[883]|DHJPAR0061018|Ceratocilia sixolalis|657[1n]|BOLD:ACN0056
Alabagrus almasolisae|[884]|DHJPAR0028272|Ceratocilia sixolalis|621[0n]|BOLD:ACN0056
Alabagrus almasolisae|[885]|DHJPAR0028271|Ceratocilia sixolalis|618[0n]|BOLD:ACN0056
Alabagrus almasolisae|[886]|DHJPAR0028273|Ceratocilia sixolalis|618[0n]|BOLD:ACN0056
Alabagrus almasolisae|[887]|DHJPAR0054507|Ceratocilia sixolalis|658[1n]|BOLD:ACN0056
Alabagrus almasolisae|[888]|DHJPAR0054508|Ceratocilia sixolalis|658[3n]|BOLD:ACN0056
Alabagrus almasolisae|[889]|DHJPAR0030384|Ceratocilia sixolalis|658[0n]|BOLD:ACN0056
Alabagrus almasolisae|[890]|DHJPAR0028269|Ceratocilia sixolalis|627[0n]|BOLD:ACN0056
Alabagrus almasolisae|[891]|DHJPAR0028270|Ceratocilia sixolalis|627[0n]|BOLD:ACN0056
Alabagrus almasolisae|[892]|DHJPAR0063369|Ceratocilia sixolalis|632[1n]|BOLD:ACN0056
Alabagrus almasolisae|[893]|DHJPAR0063803|Ceratocilia sixolalis|631[1n]|BOLD:ACN0056
Alabagrus almasolisae|[894]|DHJPAR0063804|Ceratocilia sixolalis|630[0n]|BOLD:ACN0056
Alabagrus almasolisae|[895]|DHJPAR0061030|Ceratocilia sixolalis|658[1n]|BOLD:ACN0056
Alabagrus almasolisae|[896]|DHJPAR0037932|Ceratocilia sixolalis|658[0n]|BOLD:ACN0056
Alabagrus almasolisae|[897]|DHJPAR0061029|Ceratocilia sixolalis|658[0n]|BOLD:ACN0056
[illegible]

Alabagrus almasolisae|[895]|DHJPAR0061030|Ceratocilia sixolalis|658[1n]|BOLD:ACN0056
Alabagrus almasolisae|[896]|DHJPAR0037932|Ceratocilia sixolalis|658[0n]|BOLD:ACN0056
Alabagrus almasolisae|[897]|DHJPAR0061029|Ceratocilia sixolalis|658[0n]|BOLD:ACN0056
Alabagrus almasolisae|[898]|DHJPAR0061486|Ceratocilia sixolalis|658[0n]|BOLD:ACN0056
Alabagrus almasolisae|[899]|DHJPAR0037935|Ceratocilia sixolalis|658[0n]|BOLD:ACN0056
Alabagrus almasolisae|[900]|DHJPAR0061023|Ceratocilia sixolalis|658[0n]|BOLD:ACN0056
Alabagrus almasolisae|[901]|DHJPAR0055101|Ceratocilia sixolalis|658[0n]|BOLD:ACN0056
Alabagrus almasolisae|[902]|DHJPAR0061024|Ceratocilia sixolalis|658[0n]|BOLD:ACN0056
Alabagrus almasolisae|[903]|DHJPAR0061027|Ceratocilia sixolalis|658[0n]|BOLD:ACN0056
Alabagrus almasolisae|[904]|DHJPAR0037924|Ceratocilia sixolalis|658[0n]|BOLD:ACN0056
Alabagrus almasolisae|[905]|DHJPAR0061020|Ceratocilia sixolalis|658[0n]|BOLD:ACN0056
Alabagrus almasolisae|[906]|DHJPAR0061019|Ceratocilia sixolalis|658[0n]|BOLD:ACN0056
Alabagrus almasolisae|[907]|DHJPAR0037931|Ceratocilia sixolalis|658[0n]|BOLD:ACN0056
Alabagrus almasolisae|[908]|DHJPAR0061026|Ceratocilia sixolalis|658[0n]|BOLD:ACN0056
Alabagrus almasolisae|[909]|DHJPAR0037933|Ceratocilia sixolalis|658[0n]|BOLD:ACN0056
Alabagrus almasolisae|[910]|DHJPAR0061028|Ceratocilia sixolalis|657[1n]|BOLD:ACN0056
Alabagrus almasolisae|[911]|DHJPAR0042816|Ceratocilia sixolalis|658[2n]|BOLD:ACN0056
Alabagrus almasolisae|[912]|DHJPAR0063368|Ceratocilia sixolalis|590[1n]|BOLD:ACN0056
Alabagrus almasolisae|[913]|DHJPAR0063367|Ceratocilia sixolalis|597[3n]|BOLD:ACN0056
Alabagrus almasolisae|[914]|DHJPAR0042818|Ceratocilia sixolalis|637[1n]|BOLD:ACN0056
Alabagrus almasolisae|[915]|DHJPAR0063373|Ceratocilia sixolalis|604[0n]|BOLD:ACN0056
Alabagrus almasolisae|[916]|DHJPAR0063370|Ceratocilia sixolalis|597[1n]|BOLD:ACN0056
Alabagrus almasolisae|[917]|DHJPAR0063372|Ceratocilia sixolalis|627[0n]|BOLD:ACN0056
Alabagrus almasolisae|[918]|DHJPAR0042817|Ceratocilia sixolalis|658[0n]|BOLD:ACN0056
Alabagrus almasolisae|[919]|DHJPAR0058545|Ceratocilia sixolalis|510[0n]|BOLD:ACN0056
Alabagrus almasolisae|[920]|DHJPAR0063806|Ceratocilia sixolalis|590[1n]|BOLD:ACN0056
Alabagrus almasolisae|[921]|DHJPAR0061025|Ceratocilia sixolalis|632[1n]|BOLD:ACN0056
Alabagrus almasolisae|[922]|DHJPAR0059337|Ceratocilia sixolalis|614[0n]|BOLD:ACN0056
Alabagrus almasolisae|[923]|DHJPAR0059336|Ceratocilia sixolalis|613[0n]|BOLD:ACN0056
Alabagrus almasolisae|[924]|DHJPAR0053625|Ceratocilia sixolalis|614[0n]|BOLD:ACN0056
Alabagrus almasolisae|[925]|DHJPAR0063801|Ceratocilia sixolalis|574[1n]|BOLD:ACN0056
Alabagrus almasolisae|[926]|DHJPAR0063802|Ceratocilia sixolalis|397[3n]|BOLD:ACN0056
Alabagrus almasolisae|[927]|DHJPAR0041578|Ceratocilia sixolalis|584[0n]|BOLD:ACN0056
Alabagrus almasolisae|[928]|DHJPAR0064516|Ceratocilia sixolalis|615[1n]|BOLD:ACN0056
Alabagrus maculipes|[929]|DHJPAR0040212|Palpusia Janzen02|615[0n]|BOLD:AAE6454
Alabagrus maculipes|[930]|DHJPAR0040213|Palpusia Janzen02|658[1n]|BOLD:AAE6454
Alabagrus maculipes|[931]|DHJPAR0037188|Orphanostigma haemorrhoidalis|612[1n]|BOLD:AAE6454
Alabagrus maculipes|[932]|DHJPAR0048736|Pilocrocis ramentalis|658[1n]|BOLD:AAE6454
Alabagrus maculipes|[933]|DHJPAR0041151|Herpetogramma Janzen04|624[2n]|BOLD:AAE6454
Alabagrus maculipes|[934]|DHJPAR0041150|Herpetogramma Janzen04|624[3n]|BOLD:AAE6454
Alabagrus maculipes|[935]|DHJPAR0040329|Herpetogramma semilaniata|658[0n]|BOLD:AAE6454
Alabagrus maculipes|[936]|DHJPAR0036337|Desmia ploralisDHJ02|658[1n]|BOLD:AAE6454
Alabagrus maculipes|[937]|DHJPAR0036725|Syngamia florella|641[0n]|BOLD:AAE6454
Alabagrus maculipes|[938]|DHJPAR0056292|Herpetogramma salbialis|670[0n]|BOLD:AAE6454
Alabagrus maculipes|[939]|DHJPAR0048735|Pilocrocis ramentalis|658[0n]|BOLD:AAE6454
Alabagrus yuchinkengae|[940]|DHJPAR0045112|Microthyris prolongalis|658[0n]|BOLD:ADC9470
Alabagrus roibasi|[941]|DHJPAR0009358|Phostria oajacalis|654[0n]|BOLD:ADC9450
Alabagrus roibasi|[942]|DHJPAR0037882|Phostria oajacalis|658[3n]|BOLD:ADC9450
Alabagrus roibasi|[943]|DHJPAR0036693|Phostria oajacalis|630[0n]|BOLD:ADC9450
Alabagrus roibasi|[944]|DHJPAR0036709|Phostria oajacalis|644[0n]|BOLD:ADC9450
Alabagrus roibasi|[945]|DHJPAR0036690|Phostria oajacalis|629[0n]|BOLD:ADC9450
Alabagrus roibasi|[946]|DHJPAR0036691|Phostria oajacalis|640[0n]|BOLD:ADC9450
Alabagrus janzeni|[947]|DHJPAR0044996|Eulepte Janzen06|616[3n]|BOLD:ACD1738
Alabagrus janzeni|[948]|DHJPAR0044992|Eulepte Janzen06|619[2n]|BOLD:ACD1738
Alabagrus janzeni|[949]|DHJPAR0055089|Eulepte concordalis|658[2n]|BOLD:ACD1738
Alabagrus janzeni|[950]|DHJPAR0054487|Eulepte Janzen06|658[7n]
Alabagrus janzeni|[951]|DHJPAR0055094|Eulepte concordalis|604[14n]
Alabagrus janzeni|[952]|DHJPAR0044994|Eulepte Janzen06|619[1n]|BOLD:ACD1738
Alabagrus janzeni|[953]|DHJPAR0053623|Eulepte concordalis|658[1n]|BOLD:ACD1738
Alabagrus janzeni|[954]|DHJPAR0055093|Eulepte concordalis|658[3n]|BOLD:ACD1738
Alabagrus janzeni|[955]|DHJPAR0053588|Eulepte Janzen06|658[2n]|BOLD:ACD1738
Alabagrus janzeni|[956]|DHJPAR0015384|Eulepte concordalis|636[10n]
Alabagrus janzeni|[957]|DHJPAR0016416|Eulepte concordalis|655[3n]|BOLD:ACD1738
Alabagrus janzeni|[958]|DHJPAR0009374|Eulepte concordalis|654[6n]|BOLD:ACD1738
Alabagrus janzeni|[959]|DHJPAR0048724|Eulepte Solis15|658[0n]|BOLD:ACD1738
Alabagrus janzeni|[960]|DHJPAR0009362|Eulepte concordalis|654[2n]|BOLD:ACD1738
Alabagrus janzeni|[961]|DHJPAR0009403|Eulepte concordalis|654[3n]|BOLD:ACD1738
Alabagrus janzeni|[962]|DHJPAR0009348|Eulepte concordalis|654[3n]|BOLD:ACD1738
Alabagrus janzeni|[963]|DHJPAR0009356|Eulepte concordalis|654[3n]|BOLD:ACD1738
Alabagrus janzeni|[964]|DHJPAR0015493|Eulepte Solis15|651[1n]|BOLD:ACD1738
Alabagrus janzeni|[965]|DHJPAR0010509|Eulepte concordalis|615[0n]|BOLD:ACD1738
Alabagrus janzeni|[966]|DHJPAR0049940|Eulepte concordalis|658[1n]|BOLD:ACD1738
Alabagrus janzeni|[967]|DHJPAR0016431|Eulepte concordalis|614[1n]|BOLD:ACD1738
Alabagrus janzeni|[968]|DHJPAR0009375|Eulepte concordalis|648[2n]|BOLD:ACD1738
Alabagrus janzeni|[969]|DHJPAR0015512|Eulepte concordalis|648[2n]|BOLD:ACD1738
Alabagrus janzeni|[970]|DHJPAR0010083|Eulepte concordalis|658[0n]|BOLD:ACD1738
Alabagrus janzeni|[971]|DHJPAR0044993|Eulepte Janzen06|658[0n]|BOLD:ACD1738
Alabagrus janzeni|[972]|DHJPAR0015511|Eulepte concordalis|654[0n]|BOLD:ACD1738
Alabagrus janzeni|[973]|DHJPAR0016926|Eulepte concordalis|654[0n]|BOLD:ACD1738
Alabagrus janzeni|[974]|DHJPAR0016928|Eulepte concordalis|654[0n]|BOLD:ACD1738
Alabagrus janzeni|[975]|DHJPAR0041195|Eulepte Janzen06|658[0n]|BOLD:ACD1738
Alabagrus janzeni|[976]|DHJPAR0015498|Eulepte concordalis|654[0n]|BOLD:ACD1738
Alabagrus janzeni|[977]|DHJPAR0016428|Eulepte concordalis|658[0n]|BOLD:ACD1738
Alabagrus janzeni|[978]|DHJPAR0009355|Eulepte concordalis|654[0n]|BOLD:ACD1738
Alabagrus janzeni|[979]|DHJPAR0050363|Eulepte concordalis|658[0n]|BOLD:ACD1738
Alabagrus janzeni|[980]|DHJPAR0044998|Eulepte concordalis|658[0n]|BOLD:ACD1738
Alabagrus janzeni|[981]|DHJPAR0036363|Eulepte concordalis|658[0n]|BOLD:ACD1738
Alabagrus janzeni|[982]|DHJPAR0036366|Eulepte concordalis|658[0n]|BOLD:ACD1738
Alabagrus janzeni|[983]|DHJPAR0016432|Eulepte concordalis|658[0n]|BOLD:ACD1738
Alabagrus janzeni|[984]|DHJPAR0009433|Eulepte concordalis|654[0n]|BOLD:ACD1738
Alabagrus janzeni|[985]|DHJPAR0016417|Eulepte concordalis|658[0n]|BOLD:ACD1738
Alabagrus janzeni|[986]|DHJPAR0041197|Eulepte Janzen06|658[0n]|BOLD:ACD1738
Alabagrus janzeni|[987]|DHJPAR0015497|Eulepte concordalis|654[0n]|BOLD:ACD1738
Alabagrus janzeni|[988]|DHJPAR0050371|Eulepte concordalis|658[0n]|BOLD:ACD1738
Alabagrus janzeni|[989]|DHJPAR0009400|Eulepte concordalis|654[0n]|BOLD:ACD1738
Alabagrus janzeni|[990]|DHJPAR0053589|Eulepte Janzen06|658[0n]|BOLD:ACD1738
Alabagrus janzeni|[991]|DHJPAR0016930|Eulepte concordalis|654[0n]|BOLD:ACD1738
Alabagrus janzeni|[992]|DHJPAR0016429|Eulepte concordalis|658[0n]|BOLD:ACD1738
Alabagrus janzeni|[993]|DHJPAR0015515|Eulepte concordalis|658[0n]|BOLD:ACD1738
Alabagrus janzeni|[994]|DHJPAR0015513|Eulepte concordalis|654[0n]|BOLD:ACD1738
Alabagrus janzeni|[995]|DHJPAR0021211|Eulepte concordalis|654[0n]|BOLD:ACD1738
Alabagrus janzeni|[996]|DHJPAR0041576|Eulepte Janzen06|658[0n]|BOLD:ACD1738
Alabagrus janzeni|[997]|DHJPAR0037190|Eulepte concordalis|654[0n]|BOLD:ACD1738

Alabagrus janzeni|[995]|DHJPAR0021211|Eulepte concordalis|654[0n]|BOLD:ACD1738
Alabagrus janzeni|[996]|DHJPAR0041576|Eulepte Janzen06|658[0n]|BOLD:ACD1738
Alabagrus janzeni|[997]|DHJPAR0037190|Eulepte concordalis|654[0n]|BOLD:ACD1738
Alabagrus janzeni|[998]|DHJPAR0036699|Eulepte concordalis|635[0n]|BOLD:ACD1738
Alabagrus janzeni|[999]|DHJPAR0015495|Eulepte Solis15|639[5n]|BOLD:ACD1738
Alabagrus janzeni|[1000]|DHJPAR0009434|Eulepte concordalis|654[1n]|BOLD:ACD1738
Alabagrus janzeni|[1001]|DHJPAR0036365|Eulepte concordalis|611[0n]|BOLD:ACD1738
Alabagrus janzeni|[1002]|DHJPAR0009354|Eulepte concordalis|654[5n]|BOLD:ACD1738
Alabagrus janzeni|[1003]|DHJPAR0015496|Eulepte concordalis|645[0n]|BOLD:ACD1738
Alabagrus janzeni|[1004]|DHJPAR0036697|Eulepte concordalis|641[0n]|BOLD:ACD1738
Alabagrus janzeni|[1005]|DHJPAR0058539|Eulepte concordalis|530[0n]|BOLD:ACD1738
Alabagrus janzeni|[1006]|DHJPAR0058542|Eulepte concordalis|530[0n]|BOLD:ACD1738
Alabagrus janzeni|[1007]|DHJPAR0010510|Eulepte concordalis|658[0n]|BOLD:ACD1738
Alabagrus janzeni|[1008]|DHJPAR0058537|Eulepte concordalis|530[0n]|BOLD:ACD1738
Alabagrus janzeni|[1009]|DHJPAR0036712|Eulepte concordalis|635[0n]|BOLD:ACD1738
Alabagrus janzeni|[1010]|DHJPAR0016430|Eulepte concordalis|623[0n]|BOLD:ACD1738
Alabagrus janzeni|[1011]|DHJPAR0041198|Eulepte Janzen06|585[0n]|BOLD:ACD1738
Alabagrus janzeni|[1012]|DHJPAR0063366|Eulepte concordalis|570[0n]|BOLD:ACD1738
Alabagrus janzeni|[1013]|DHJPAR0041167|Eulepte Janzen02|539[0n]|BOLD:ACD1738
Alabagrus janzeni|[1014]|DHJPAR0015494|Eulepte Solis15|534[3n]|BOLD:ACD1738
Alabagrus janzeni|[1015]|DHJPAR0036698|Eulepte concordalis|374[0n]|BOLD:ACD1738
Alabagrus janzeni|[1016]|DHJPAR0041166|Eulepte Janzen06|284[1n]
Alabagrus janzeni|[1017]|DHJPAR0036718|Eulepte concordalis|446[1n]|BOLD:ACD1738
Alabagrus janzeni|[1018]|DHJPAR0029179|Eulepte concordalis|362[1n]|BOLD:ACD1738
Alabagrus janzeni|[1019]|DHJPAR0036716|Eulepte concordalis|383[1n]|BOLD:ACD1738
Alabagrus janzeni|[1020]|DHJPAR0015499|Eulepte concordalis|615[0n]|BOLD:ACD1738
Alabagrus janzeni|[1021]|DHJPAR0009376|Eulepte concordalis|609[1n]|BOLD:ACD1738
Alabagrus janzeni|[1022]|DHJPAR0044995|Eulepte Janzen06|658[0n]|BOLD:ACD1738
Alabagrus janzeni|[1023]|DHJPAR0055092|Eulepte concordalis|658[1n]|BOLD:ACD1738
Alabagrus janzeni|[1024]|DHJPAR0053622|Eulepte Janzen06|604[7n]
Alabagrus janzeni|[1025]|DHJPAR0055095|Eulepte concordalis|629[2n]|BOLD:ACD1738
Alabagrus janzeni|[1026]|DHJPAR0037922|Eulepte concordalis|658[0n]|BOLD:ACD1738
Alabagrus janzeni|[1027]|DHJPAR0037902|Eulepte concordalis|658[2n]|BOLD:ACD1738
Alabagrus janzeni|[1028]|DHJPAR0055097|Eulepte concordalis|658[2n]|BOLD:ACD1738
Alabagrus janzeni|[1029]|DHJPAR0044997|Eulepte Janzen06|658[1n]|BOLD:ACD1738
Alabagrus janzeni|[1030]|DHJPAR0055091|Eulepte concordalis|658[0n]|BOLD:ACD1738
Alabagrus janzeni|[1031]|DHJPAR0053587|Eulepte concordalis|658[3n]|BOLD:ACD1738
Alabagrus janzeni|[1032]|DHJPAR0021208|Eulepte concordalis|639[0n]|BOLD:ACD1738
Alabagrus janzeni|[1033]|DHJPAR0021149|Eulepte concordalis|654[0n]|BOLD:ACD1738
Alabagrus janzeni|[1034]|DHJPAR0052083|Eulepte concordalis|611[7n]
Alabagrus janzeni|[1035]|DHJPAR0055090|Eulepte concordalis|604[8n]
Alabagrus janzeni|[1036]|DHJPAR0053590|Eulepte Janzen06|658[5n]|BOLD:ACD1738
Alabagrus janzeni|[1037]|DHJPAR0055096|Eulepte concordalis|658[3n]|BOLD:ACD1738
Hemichoma frankhovorei|[1038]|DHJPAR0028266|Halysidota pectenella|618[0n]|BOLD:AEF1239
Plesiocoelus vanachterbergi|[1039]|DHJPAR0051104|Brenthia Janzen05|527[3n]|BOLD:ABX6701
Plesiocoelus vanachterbergi|[1040]|DHJPAR0045360|Brenthia Janzen14|658[6n]
Plesiocoelus vanachterbergi|[1041]|DHJPAR0048215|Brenthia Janzen15|658[3n]|BOLD:ABX6701
Plesiocoelus vanachterbergi|[1042]|DHJPAR0053040|Brenthia Janzen02|658[0n]|BOLD:ABX6701
Plesiocoelus vanachterbergi|[1043]|DHJPAR0053045|Brenthia Janzen02|658[0n]|BOLD:ABX6701
Plesiocoelus vanachterbergi|[1044]|DHJPAR0053044|Brenthia Janzen02|658[0n]|BOLD:ABX6701
Plesiocoelus vanachterbergi|[1045]|DHJPAR0053033|Brenthia Janzen02|658[5n]
Plesiocoelus vanachterbergi|[1046]|DHJPAR0050114|Brenthia Janzen14|658[5n]
Plesiocoelus vanachterbergi|[1047]|DHJPAR0053046|Brenthia Janzen02|647[1n]|BOLD:ABX6701
Plesiocoelus vanachterbergi|[1048]|DHJPAR0049867|Brenthia Janzen15|658[4n]|BOLD:ABX6701
Aerophilus sandraberriosae|[1049]|DHJPAR0052688|Deuterollyta oediperalis|658[0n]|BOLD:ACJ1459
Aerophilus sandraberriosae|[1050]|DHJPAR0049659|Deuterollyta basalata|613[6n]
Aerophilus sandraberriosae|[1051]|DHJPAR0021148|Deuterollyta oediperalisDHJ01|390[0n]
Aerophilus sandraberriosae|[1052]|DHJPAR0040219|Deuterollyta oediperalis|275[1n]
Aerophilus sandraberriosae|[1053]|DHJPAR0040220|Deuterollyta oediperalis|622[3n]|BOLD:ACJ1459
Lytopylus okchunae|[1054]|DHJPAR0056977|Antaeotricha Janzen78|658[0n]|BOLD:ACC1822
Lytopylus okchunae|[1055]|DHJPAR0051910|Antaeotricha Janzen78|658[0n]|BOLD:ACC1822
Lytopylus okchunae|[1056]|DHJPAR0051363|Antaeotricha Janzen727|658[0n]|BOLD:ACC1822
Lytopylus okchunae|[1057]|DHJPAR0037191|Antaeotricha Janzen78|654[0n]|BOLD:ACC1822
Lytopylus okchunae|[1058]|BIOUG25661-C04|ESG Malaise Trap|600[0n]|BOLD:ACC1822
Lytopylus okchunae|[1059]|BIOUG25661-A07|ESG Malaise Trap|594[0n]|BOLD:ACC1822
Lytopylus okchunae|[1060]|BIOUG28100-G03|ESG Malaise Trap Year 2|576[0n]|BOLD:ACC1822
Lytopylus okchunae|[1061]|DHJPAR0056981|Antaeotricha Janzen78|658[0n]|BOLD:ACC1822
Lytopylus okchunae|[1062]|DHJPAR0050369|Antaeotricha Janzen224|658[0n]|BOLD:ACC1822
Lytopylus okchunae|[1063]|DHJPAR0056980|Antaeotricha Janzen78|658[0n]|BOLD:ACC1822
Lytopylus okchunae|[1064]|DHJPAR0041164|Antaeotricha Janzen78|624[0n]|BOLD:ACC1822
Lytopylus okchunae|[1065]|DHJPAR0055819|Antaeotricha Janzen727|658[0n]|BOLD:ACC1822
Lytopylus okchunae|[1066]|DHJPAR0050939|Antaeotricha Janzen78|658[0n]|BOLD:ACC1822
Lytopylus okchunae|[1067]|DHJPAR0056982|Antaeotricha Janzen78|658[0n]|BOLD:ACC1822
Lytopylus okchunae|[1068]|DHJPAR0055345|Antaeotricha Janzen727|658[0n]|BOLD:ACC1822
Lytopylus okchunae|[1069]|DHJPAR0055984|Antaeotricha Janzen727|670[0n]|BOLD:ACC1822
Lytopylus okchunae|[1070]|BIOUG28198-C07|ESG Malaise Trap Year 2|658[1n]|BOLD:ACC1822
Lytopylus okchunae|[1071]|DHJPAR0055355|Antaeotricha Janzen727|658[0n]|BOLD:ACC1822
Lytopylus okchunae|[1072]|DHJPAR0057424|Antaeotricha Janzen78|658[0n]|BOLD:ACC1822
Lytopylus okchunae|[1073]|DHJPAR0015529|Antaeotricha Janzen78|654[0n]|BOLD:ACC1822
Lytopylus gustavoindunii|[1074]|DHJPAR0053646|Stenoma Janzen284|486[3n]|BOLD:ACJ2804
Lytopylus gustavoindunii|[1075]|DHJPAR0051090|Stenoma Janzen142|658[4n]|BOLD:ACJ2804
Lytopylus gustavoindunii|[1076]|DHJPAR0053857|Stenoma Janzen284|661[0n]|BOLD:ACJ2804
Lytopylus gustavoindunii|[1077]|DHJPAR0051359|Stenoma Janzen142|658[1n]|BOLD:ACJ2804
Lytopylus gustavoindunii|[1078]|DHJPAR0056821|Stenoma Janzen142|658[0n]|BOLD:ACJ2804
Lytopylus gustavoindunii|[1079]|DHJPAR0041560|Stenoma Janzen142|658[0n]|BOLD:ACJ2804
Lytopylus gustavoindunii|[1080]|BIOUG27761-A10|ESG Malaise Trap Year 2|543[0n]|BOLD:ACJ2804
Lytopylus gustavoindunii|[1081]|DHJPAR0051093|Stenoma Janzen142|658[0n]|BOLD:ACJ2804
Lytopylus gustavoindunii|[1082]|DHJPAR0056431|Stenoma Janzen142|658[0n]|BOLD:ACJ2804
Lytopylus gustavoindunii|[1083]|DHJPAR0053653|Stenoma Janzen284|658[0n]|BOLD:ACJ2804
Lytopylus gustavoindunii|[1084]|DHJPAR0051372|Stenoma Janzen142|658[0n]|BOLD:ACJ2804
Lytopylus gustavoindunii|[1085]|DHJPAR0051369|Stenoma Janzen142|658[0n]|BOLD:ACJ2804
Lytopylus alfredomainieri|[1086]|DHJPAR0038251|Olethreutes Brown22|658[0n]|BOLD:ADD0100
Lytopylus alfredomainieri|[1087]|DHJPAR0035519|Olethreutes Janzen188|658[0n]|BOLD:ADD0100
Lytopylus alfredomainieri|[1088]|DHJPAR0035513|Olethreutes Janzen188|658[0n]|BOLD:ADD0100
Lytopylus alfredomainieri|[1089]|DHJPAR0037861|Olethreutes Brown22|658[0n]|BOLD:ADD0100
Lytopylus alfredomainieri|[1090]|DHJPAR0035525|Olethreutes Janzen188|658[0n]|BOLD:ADD0100
Lytopylus chrysockeras|[1091]|DHJPAR0055986|gelJanzen01 Janzen179|670[0n]|BOLD:AAJ9259
Lytopylus chrysockeras|[1092]|DHJPAR0049276|gelJanzen01 Janzen356|658[0n]|BOLD:AAJ9259
Lytopylus chrysockeras|[1093]|DHJPAR0052673|gelJanzen01 Janzen19|658[0n]|BOLD:AAJ9259
Lytopylus chrysockeras|[1094]|DHJPAR0040503|gelJanzen01 Janzen485|658[0n]|BOLD:AAJ9259
Lytopylus chrysockeras|[1095]|DHJPAR0040067|Dichomeris Janzen703|658[0n]|BOLD:AAJ9259
Lytopylus chrysockeras|[1096]|DHJPAR0040487|gelJanzen01 Janzen485|658[0n]|BOLD:AAJ9259
Lytopylus chrysockeras|[1097]|DHJPAR0055507|gelJanzen01 Janzen485|658[0n]|BOLD:AAJ9259

[illegible]
Lytopylus chrysockeras|[1095]|DHJPAR0040067|Dichomeris Janzen703|658[0n]|BOLD:AAJ9259
Lytopylus chrysockeras|[1096]|DHJPAR0040487|gelJanzen01 Janzen485|658[0n]|BOLD:AAJ9259
Lytopylus chrysockeras|[1097]|DHJPAR0055507|gelJanzen01 Janzen485|658[0n]|BOLD:AAJ9259
Lytopylus chrysockeras|[1098]|DHJPAR0055353|gelJanzen01 Janzen485|658[0n]|BOLD:AAJ9259
Lytopylus chrysockeras|[1099]|DHJPAR0051364|gelJanzen01 Janzen356|658[0n]|BOLD:AAJ9259
Lytopylus chrysockeras|[1100]|DHJPAR0051817|gelJanzen01 Janzen485|661[0n]|BOLD:AAJ9259
Lytopylus chrysockeras|[1101]|DHJPAR0055513|gelJanzen01 Janzen485|658[0n]|BOLD:AAJ9259
Lytopylus chrysockeras|[1102]|DHJPAR0055528|gelJanzen01 Janzen485|658[0n]|BOLD:AAJ9259
Lytopylus chrysockeras|[1103]|DHJPAR0040473|gelJanzen01 Janzen485|658[0n]|BOLD:AAJ9259
Lytopylus chrysockeras|[1104]|DHJPAR0040492|gelJanzen01 Janzen485|658[0n]|BOLD:AAJ9259
Lytopylus chrysockeras|[1105]|DHJPAR0038356|gelJanzen01 Janzen1485|658[0n]|BOLD:AAJ9259
Lytopylus chrysockeras|[1106]|DHJPAR0040476|gelJanzen01 Janzen485|658[0n]|BOLD:AAJ9259
Lytopylus chrysockeras|[1107]|DHJPAR0015527|gelJanzen01 Janzen781|654[0n]|BOLD:AAJ9259
Lytopylus chrysockeras|[1108]|DHJPAR0055490|gelJanzen01 Janzen485|658[0n]|BOLD:AAJ9259
Lytopylus chrysockeras|[1109]|DHJPAR0055543|gelJanzen01 Janzen485|661[0n]|BOLD:AAJ9259
Lytopylus chrysockeras|[1110]|DHJPAR0055504|gelJanzen01 Janzen485|658[0n]|BOLD:AAJ9259
Lytopylus chrysockeras|[1111]|DHJPAR0040070|gelJanzen01 Janzen356|658[0n]|BOLD:AAJ9259
Lytopylus chrysockeras|[1112]|DHJPAR0040331|gelJanzen01 Janzen485|658[0n]|BOLD:AAJ9259
Lytopylus chrysockeras|[1113]|DHJPAR0040485|gelJanzen01 Janzen485|658[0n]|BOLD:AAJ9259
Lytopylus chrysockeras|[1114]|DHJPAR0064107|gelJanzen01 Janzen485|658[0n]|BOLD:AAJ9259
Lytopylus chrysockeras|[1115]|DHJPAR0049359|gelJanzen01 Janzen485|658[0n]|BOLD:AAJ9259
Lytopylus chrysockeras|[1116]|DHJPAR0040065|gelJanzen01 Janzen485|658[0n]|BOLD:AAJ9259
Lytopylus chrysockeras|[1117]|DHJPAR0057739|gelJanzen01 Janzen356|657[0n]|BOLD:AAJ9259
Lytopylus chrysockeras|[1118]|DHJPAR0049388|gelJanzen01 Janzen485|639[0n]|BOLD:AAJ9259
Lytopylus chrysockeras|[1119]|DHJPAR0051162|gelJanzen01 Janzen356|603[0n]|BOLD:AAJ9259
Lytopylus chrysockeras|[1120]|DHJPAR0040344|gelJanzen01 Janzen485|658[0n]|BOLD:AAJ9259
Lytopylus chrysockeras|[1121]|DHJPAR0055527|gelJanzen01 Janzen485|658[0n]|BOLD:AAJ9259
Lytopylus chrysockeras|[1122]|DHJPAR0055505|gelJanzen01 Janzen485|658[0n]|BOLD:AAJ9259
Lytopylus chrysockeras|[1123]|DHJPAR0055522|gelJanzen01 Janzen485|658[0n]|BOLD:AAJ9259
Lytopylus chrysockeras|[1124]|DHJPAR0040335|gelJanzen01 Janzen485|658[0n]|BOLD:AAJ9259
Lytopylus chrysockeras|[1125]|DHJPAR0042837|Dichomeris Janzen512|658[0n]|BOLD:AAJ9259
Lytopylus chrysockeras|[1126]|DHJPAR0043002|Dichomeris Janzen512|658[0n]|BOLD:AAJ9259
Lytopylus chrysockeras|[1127]|DHJPAR0040505|gelJanzen01 Janzen356|658[0n]|BOLD:AAJ9259
Lytopylus chrysockeras|[1128]|DHJPAR0039071|gelJanzen01 Janzen485|634[0n]|BOLD:AAJ9259
Lytopylus chrysockeras|[1129]|DHJPAR0042835|Dichomeris Janzen512|658[0n]|BOLD:AAJ9259
Lytopylus chrysockeras|[1130]|DHJPAR0055352|gelJanzen01 Janzen179|658[0n]|BOLD:AAJ9259
Lytopylus chrysockeras|[1131]|DHJPAR0040460|gelJanzen01 Janzen356|658[0n]|BOLD:AAJ9259
Lytopylus chrysockeras|[1132]|DHJPAR0051373|gelJanzen01 Janzen356|658[0n]|BOLD:AAJ9259
Lytopylus sarahmeierottoae|[1133]|DHJPAR0056992|Cerconota Janzen82|658[1n]|BOLD:ACZ0985
Lytopylus miguelviquezi|[1134]|DHJPAR0041574|gelJanzen01 Janzen485|658[0n]|BOLD:ABX5568
Lytopylus miguelviquezi|[1135]|DHJPAR0041567|gelJanzen01 Janzen485|658[0n]|BOLD:ABX5568
Lytopylus miguelviquezi|[1136]|DHJPAR0040326|Dichomeris designatellaDHJ02|658[0n]|BOLD:ABX5568
Lytopylus miguelviquezi|[1137]|DHJPAR0041565|gelJanzen01 Janzen485|658[2n]|BOLD:ABX5568
Lytopylus miguelviquezi|[1138]|DHJPAR0041561|Dichomeris designatellaDHJ02|658[0n]|BOLD:ABX5568
Lytopylus miguelviquezi|[1139]|DHJPAR0041566|gelJanzen01 Janzen485|658[0n]|BOLD:ABX5568
Lytopylus miguelviquezi|[1140]|DHJPAR0041570|gelJanzen01 Janzen485|633[0n]|BOLD:ABX5568
Lytopylus miguelviquezi|[1141]|DHJPAR0055562|gelJanzen01 Janzen179|661[0n]|BOLD:ABX5568
Lytopylus miguelviquezi|[1142]|DHJPAR0038918|Dichomeris designatellaDHJ02|658[0n]|BOLD:ABX5568
Lytopylus miguelviquezi|[1143]|DHJPAR0038917|Dichomeris designatellaDHJ01|658[0n]|BOLD:ABX5568
Lytopylus miguelviquezi|[1144]|DHJPAR0055506|gelJanzen01 Janzen179|658[0n]|BOLD:ABX5568
Lytopylus miguelviquezi|[1145]|DHJPAR0041553|gelJanzen01 Janzen485|658[0n]|BOLD:ABX5568
Lytopylus miguelviquezi|[1146]|DHJPAR0041575|Dichomeris designatellaDHJ02|658[0n]|BOLD:ABX5568
Lytopylus miguelviquezi|[1147]|DHJPAR0041555|gelJanzen01 Janzen485|658[0n]|BOLD:ABX5568
Lytopylus miguelviquezi|[1148]|DHJPAR0041589|Dichomeris designatellaDHJ02|658[0n]|BOLD:ABX5568
Lytopylus miguelviquezi|[1149]|DHJPAR0040502|gelJanzen01 Janzen485|658[0n]|BOLD:ABX5568
Lytopylus miguelviquezi|[1150]|DHJPAR0040340|Dichomeris designatellaDHJ02|658[0n]|BOLD:ABX5568
Lytopylus miguelviquezi|[1151]|DHJPAR0040327|Dichomeris designatellaDHJ02|627[0n]|BOLD:ABX5568
Lytopylus miguelviquezi|[1152]|DHJPAR0040336|gelJanzen01 Janzen485|658[0n]|BOLD:ABX5568
Lytopylus miguelviquezi|[1153]|DHJPAR0041563|gelJanzen01 Janzen485|658[0n]|BOLD:ABX5568
Lytopylus miguelviquezi|[1154]|DHJPAR0056065|gelJanzen01 Janzen179|610[0n]|BOLD:ABX5568
Lytopylus miguelviquezi|[1155]|DHJPAR0038912|Dichomeris designatellaDHJ02|658[0n]|BOLD:ABX5568
Lytopylus miguelviquezi|[1156]|DHJPAR0040342|Dichomeris designatellaDHJ03|658[0n]|BOLD:ABX5568
Lytopylus miguelviquezi|[1157]|DHJPAR0040348|Dichomeris designatellaDHJ03|658[0n]|BOLD:ABX5568
Lytopylus miguelviquezi|[1158]|DHJPAR0040483|Dichomeris designatellaDHJ03|658[0n]|BOLD:ABX5568
Lytopylus miguelviquezi|[1159]|DHJPAR0041588|Dichomeris designatellaDHJ03|658[0n]|BOLD:ABX5568
Lytopylus miguelviquezi|[1160]|DHJPAR0038906|Dichomeris designatellaDHJ02|658[0n]|BOLD:ABX5568
Lytopylus miguelviquezi|[1161]|DHJPAR0041949|Dichomeris designatellaDHJ02|658[0n]|BOLD:ABX5568
Lytopylus miguelviquezi|[1162]|DHJPAR0040330|Dichomeris designatellaDHJ03|658[0n]|BOLD:ABX5568
Lytopylus miguelviquezi|[1163]|DHJPAR0045369|gelJanzen01 Janzen179|658[0n]|BOLD:ABX5568
Lytopylus miguelviquezi|[1164]|DHJPAR0045276|gelJanzen01 Janzen179|658[1n]|BOLD:ABX5568
Lytopylus miguelviquezi|[1165]|DHJPAR0045371|gelJanzen01 Janzen179|658[1n]|BOLD:ABX5568
Lytopylus miguelviquezi|[1166]|DHJPAR0055484|gelJanzen01 Janzen485|658[0n]|BOLD:ABX5568
Lytopylus miguelviquezi|[1167]|DHJPAR0062682|gelJanzen01 Janzen179|607[0n]|BOLD:ABX5568
Lytopylus miguelviquezi|[1168]|DHJPAR0045373|gelJanzen01 Janzen179|658[1n]|BOLD:ABX5568
Lytopylus miguelviquezi|[1169]|DHJPAR0045296|gelJanzen01 Janzen179|658[2n]|BOLD:ABX5568
Lytopylus miguelviquezi|[1170]|DHJPAR0045368|gelJanzen01 Janzen179|658[2n]|BOLD:ABX5568
Lytopylus miguelviquezi|[1171]|DHJPAR0045305|gelJanzen01 Janzen179|658[3n]|BOLD:ABX5568
Lytopylus miguelviquezi|[1172]|DHJPAR0040347|Dichomeris designatellaDHJ02|658[0n]|BOLD:ABX5568
Lytopylus miguelviquezi|[1173]|DHJPAR0015528|gelJanzen01 Janzen485|654[0n]|BOLD:ABX5568
Lytopylus miguelviquezi|[1174]|DHJPAR0038916|Dichomeris designatellaDHJ02|658[0n]|BOLD:ABX5568
Lytopylus miguelviquezi|[1175]|DHJPAR0045374|gelJanzen01 Janzen179|658[0n]|BOLD:ABX5568
Lytopylus miguelviquezi|[1176]|DHJPAR0039509|gelJanzen01 Janzen485|658[0n]|BOLD:ABX5568
Lytopylus miguelviquezi|[1177]|DHJPAR0040341|Dichomeris designatellaDHJ02|658[0n]|BOLD:ABX5568
Lytopylus miguelviquezi|[1178]|DHJPAR0039516|gelJanzen01 Janzen485|658[0n]|BOLD:ABX5568
Lytopylus miguelviquezi|[1179]|DHJPAR0038909|gelJanzen01 Janzen485|658[0n]|BOLD:ABX5568
Lytopylus miguelviquezi|[1180]|DHJPAR0038911|Dichomeris designatellaDHJ02|658[0n]|BOLD:ABX5568
Lytopylus miguelviquezi|[1181]|DHJPAR0042843|Dichomeris designatellaDHJ02|658[0n]|BOLD:ABX5568
Lytopylus miguelviquezi|[1182]|DHJPAR0041592|Dichomeris designatellaDHJ02|658[0n]|BOLD:ABX5568
Lytopylus miguelviquezi|[1183]|DHJPAR0041597|Dichomeris designatellaDHJ02|658[0n]|BOLD:ABX5568
Lytopylus miguelviquezi|[1184]|DHJPAR0040459|Dichomeris designatellaDHJ03|658[0n]|BOLD:ABX5568
Lytopylus miguelviquezi|[1185]|DHJPAR0040474|Dichomeris designatellaDHJ03|645[0n]|BOLD:ABX5568
Lytopylus miguelviquezi|[1186]|DHJPAR0039508|gelJanzen01 Janzen485|658[1n]|BOLD:ABX5568
Lytopylus miguelviquezi|[1187]|DHJPAR0043147|Dichomeris designatellaDHJ03|643[0n]|BOLD:ABX5568
Lytopylus miguelviquezi|[1188]|DHJPAR0038920|Dichomeris designatellaDHJ02|658[0n]|BOLD:ABX5568
Lytopylus miguelviquezi|[1189]|DHJPAR0037860|Dichomeris designatellaDHJ02|630[0n]|BOLD:ABX5568
Lytopylus miguelviquezi|[1190]|DHJPAR0040332|Dichomeris designatellaDHJ02|658[0n]|BOLD:ABX5568
Lytopylus miguelviquezi|[1191]|DHJPAR0042842|gelJanzen01 Janzen179|658[0n]|BOLD:ABX5568
Lytopylus miguelviquezi|[1192]|DHJPAR0045372|gelJanzen01 Janzen179|658[0n]|BOLD:ABX5568
Lytopylus youngcheae|[1193]|DHJPAR0054499|Cerconota Janzen140|658[0n]|BOLD:ACI8518
Lytopylus youngcheae|[1194]|DHJPAR0021132|Cerconota Janzen140|654[0n]|BOLD:ACI8518
Lytopylus youngcheae|[1195]|DHJPAR0052197|Antaeotricha Janzen134|658[0n]|BOLD:ACI8518
Lytopylus youngcheae|[1196]|DHJPAR0041586|Antaeotricha Janzen134|658[0n]|BOLD:ACI8518
[illegible]

Lytopylus youngcheae|[1194]|DHJPAR0021132|Cerconota Janzen140|654[0n]|BOLD:ACI8518
Lytopylus youngcheae|[1195]|DHJPAR0052197|Antaeotricha Janzen134|658[0n]|BOLD:ACI8518
Lytopylus youngcheae|[1196]|DHJPAR0041586|Antaeotricha Janzen134|658[0n]|BOLD:ACI8518
Lytopylus youngcheae|[1197]|DHJPAR0041585|Antaeotricha Janzen134|658[0n]|BOLD:ACI8518
Lytopylus motohasegawai|[1198]|DHJPAR0041963|gelJanzen01 Janzen28|622[0n]|BOLD:ABX5613
Lytopylus motohasegawai|[1199]|DHJPAR0041602|gelJanzen01 Janzen28|629[0n]|BOLD:ABX5613
Lytopylus motohasegawai|[1200]|DHJPAR0041203|gelJanzen01 Janzen28|635[0n]|BOLD:ABX5613
Lytopylus motohasegawai|[1201]|DHJPAR0041958|gelJanzen01 Janzen28|658[1n]|BOLD:ABX5613
Lytopylus motohasegawai|[1202]|DHJPAR0045330|gelJanzen01 Janzen28|658[1n]|BOLD:ABX5613
Lytopylus motohasegawai|[1203]|DHJPAR0045364|gelJanzen01 Janzen28|658[1n]|BOLD:ABX5613
Lytopylus motohasegawai|[1204]|DHJPAR0045365|gelJanzen01 Janzen28|658[1n]|BOLD:ABX5613
Lytopylus motohasegawai|[1205]|DHJPAR0045318|gelJanzen01 Janzen28|658[1n]|BOLD:ABX5613
Lytopylus motohasegawai|[1206]|DHJPAR0054745|gelJanzen01 Janzen28|658[0n]|BOLD:ABX5613
Lytopylus motohasegawai|[1207]|DHJPAR0041584|gelJanzen01 Janzen28|658[0n]|BOLD:ABX5613
Lytopylus motohasegawai|[1208]|DHJPAR0041573|gelJanzen01 Janzen28|658[0n]|BOLD:ABX5613
Lytopylus motohasegawai|[1209]|DHJPAR0048067|gelJanzen01 Janzen28|658[0n]|BOLD:ABX5613
Lytopylus motohasegawai|[1210]|DHJPAR0048064|gelJanzen01 Janzen28|658[0n]|BOLD:ABX5613
Lytopylus motohasegawai|[1211]|DHJPAR0041960|gelJanzen01 Janzen28|658[0n]|BOLD:ABX5613
Lytopylus motohasegawai|[1212]|DHJPAR0048061|gelJanzen01 Janzen28|658[0n]|BOLD:ABX5613
Lytopylus motohasegawai|[1213]|DHJPAR0041954|gelJanzen01 Janzen28|658[0n]|BOLD:ABX5613
Lytopylus motohasegawai|[1214]|DHJPAR0048071|gelJanzen01 Janzen28|658[0n]|BOLD:ABX5613
Lytopylus motohasegawai|[1215]|DHJPAR0048059|gelJanzen01 Janzen28|658[0n]|BOLD:ABX5613
Lytopylus motohasegawai|[1216]|DHJPAR0048063|gelJanzen01 Janzen28|658[0n]|BOLD:ABX5613
Lytopylus motohasegawai|[1217]|DHJPAR0048068|gelJanzen01 Janzen28|658[0n]|BOLD:ABX5613
Lytopylus motohasegawai|[1218]|DHJPAR0041568|gelJanzen01 Janzen28|658[0n]|BOLD:ABX5613
Lytopylus motohasegawai|[1219]|DHJPAR0048062|gelJanzen01 Janzen28|658[0n]|BOLD:ABX5613
Lytopylus motohasegawai|[1220]|DHJPAR0041962|gelJanzen01 Janzen28|658[0n]|BOLD:ABX5613
Lytopylus motohasegawai|[1221]|DHJPAR0041601|gelJanzen01 Janzen28|658[0n]|BOLD:ABX5613
Lytopylus motohasegawai|[1222]|DHJPAR0048066|gelJanzen01 Janzen28|658[0n]|BOLD:ABX5613
Lytopylus motohasegawai|[1223]|DHJPAR0048060|gelJanzen01 Janzen28|658[0n]|BOLD:ABX5613
Lytopylus motohasegawai|[1224]|DHJPAR0048072|gelJanzen01 Janzen28|658[0n]|BOLD:ABX5613
Lytopylus motohasegawai|[1225]|DHJPAR0048065|gelJanzen01 Janzen28|658[0n]|BOLD:ABX5613
Lytopylus motohasegawai|[1226]|DHJPAR0048070|gelJanzen01 Janzen28|658[0n]|BOLD:ABX5613
Lytopylus motohasegawai|[1227]|DHJPAR0040071|gelJanzen01 Janzen28|658[0n]|BOLD:ABX5613
Lytopylus motohasegawai|[1228]|DHJPAR0042463|gelJanzen01 Janzen28|658[0n]|BOLD:ABX5613
Lytopylus motohasegawai|[1229]|DHJPAR0041564|gelJanzen01 Janzen28|658[0n]|BOLD:ABX5613
Lytopylus motohasegawai|[1230]|DHJPAR0041577|gelJanzen01 Janzen28|658[0n]|BOLD:ABX5613
Lytopylus motohasegawai|[1231]|DHJPAR0048073|gelJanzen01 Janzen28|658[0n]|BOLD:ABX5613
Lytopylus cesarmorai|[1232]|DHJPAR0038304|Stenoma BioLep82|658[2n]|BOLD:ADD0212
Lytopylus cesarmorai|[1233]|DHJPAR0037960|Stenoma BioLep82|658[0n]|BOLD:ADD0212
Lytopylus gisukae|[1234]|DHJPAR0048076|Antaeotricha Janzen405|658[0n]|BOLD:ABY3703
Lytopylus Malaise2253|[1235]|BIOUG25227-B02|ESG Malaise Trap|579[0n]|BOLD:ACY2253
Lytopylus eddysanchezi|[1236]|DHJPAR0049278|elachJanzen01 Janzen900|658[4n]|BOLD:ACB2941
Lytopylus eddysanchezi|[1237]|DHJPAR0038231|elachJanzen01 Janzen900|658[0n]|BOLD:ACB2941
Lytopylus eddysanchezi|[1238]|DHJPAR0038180|elachJanzen01 Janzen900|533[0n]|BOLD:ACB2941
Lytopylus eddysanchezi|[1239]|DHJPAR0055232|elachJanzen01 Janzen900|658[0n]|BOLD:ACB2941
Lytopylus eddysanchezi|[1240]|DHJPAR0038225|elachJanzen01 Janzen900|658[0n]|BOLD:ACB2941
Lytopylus eddysanchezi|[1241]|DHJPAR0041600|elachJanzen01 Janzen900|658[0n]|BOLD:ACB2941
Lytopylus eddysanchezi|[1242]|DHJPAR0052903|elachJanzen01 Janzen900|658[0n]|BOLD:ACB2941
Lytopylus eddysanchezi|[1243]|DHJPAR0051357|elachJanzen01 Janzen900|658[0n]|BOLD:ACB2941
Lytopylus eddysanchezi|[1244]|DHJPAR0038240|elachJanzen01 Janzen900|658[0n]|BOLD:ACB2941
Lytopylus eddysanchezi|[1245]|DHJPAR0056978|elachJanzen01 Janzen900|658[0n]|BOLD:ACB2941
Lytopylus eddysanchezi|[1246]|DHJPAR0038208|elachJanzen01 Janzen900|658[0n]|BOLD:ACB2941
Lytopylus pablocobbi|[1247]|DHJPAR0037189|elachJanzen01 Janzen640|630[0n]|BOLD:ADD0043
Lytopylus pablocobbi|[1248]|BIOUG33274-H10|Derrumbe Malaise Trap|589[0n]|BOLD:ADD0043
Lytopylus sangyeoni|[1249]|DHJPAR0046955|elachJanzen01 Janzen847|627[4n]|BOLD:ABX0816
Lytopylus sigifredomarini|[1250]|DHJPAR0055234|elachJanzen01 Janzen726|658[0n]|BOLD:ACJ5331
Lytopylus sigifredomarini|[1251]|DHJPAR0042545|Antaeotricha Janzen224|658[0n]|BOLD:ACJ5331
Lytopylus sigifredomarini|[1252]|DHJPAR0061031|Antaeotricha BioLep51|658[0n]|BOLD:ACJ5331
Lytopylus sigifredomarini|[1253]|DHJPAR0040328|Antaeotricha Janzen224|658[0n]|BOLD:ACJ5331
Lytopylus sigifredomarini|[1254]|DHJPAR0064518|Antaeotricha Janzen224|658[0n]|BOLD:ACJ5331
Lytopylus sigifredomarini|[1255]|DHJPAR0063387|Antaeotricha Janzen224|658[0n]|BOLD:ACJ5331
Lytopylus sigifredomarini|[1256]|DHJPAR0063383|Antaeotricha Janzen224|658[0n]|BOLD:ACJ5331
Lytopylus sigifredomarini|[1257]|DHJPAR0052899|Antaeotricha Janzen224|658[0n]|BOLD:ACJ5331
Lytopylus hernanbravoi|[1258]|DHJPAR0028303|Anadasmus Janzen08|654[0n]|BOLD:ADD0090
Lytopylus salvadorlopezi|[1259]|DHJPAR0036345|Stenoma Janzen06|654[0n]|BOLD:ADC9936
Lytopylus salvadorlopezi|[1260]|DHJPAR0036355|Stenoma Janzen06|654[0n]|BOLD:ADC9936
Lytopylus salvadorlopezi|[1261]|DHJPAR0037949|Stenoma Janzen12|628[0n]|BOLD:ADC9936
Lytopylus robertofernandezi|[1262]|DHJPAR0040221|Stenoma Janzen687|623[4n]|BOLD:ADD0042
Lytopylus ericchapmani|[1263]|DHJPAR0054522|Stenoma adytodes|605[0n]|BOLD:ACM2585
Lytopylus ericchapmani|[1264]|DHJPAR0054526|Stenoma adytodes|658[0n]|BOLD:ACM2585
Lytopylus ericchapmani|[1265]|DHJPAR0054527|Stenoma adytodes|658[0n]|BOLD:ACM2585
Lytopylus ericchapmani|[1266]|DHJPAR0054533|Stenoma adytodes|658[0n]|BOLD:ACM2585
Lytopylus ericchapmani|[1267]|DHJPAR0055238|Stenoma adytodes|658[0n]|BOLD:ACM2585
Neothlipsis bobkulai|[1268]|BIOUG29646-F07|Malaise trap PL12-3A|507[0n]|BOLD:ADB1901
Neothlipsis bobkulai|[1269]|BIOUG29620-B06|Malaise trap PL12-3A|510[0n]|BOLD:ADB1901
Neothlipsis bobkulai|[1270]|BIOUG29646-E01|Malaise trap PL12-3A|510[0n]|BOLD:ADB1901
Neothlipsis bobkulai|[1271]|BIOUG29112-G02|Malaise trap PL12-3A|510[0n]|BOLD:ADB1901
Lytopylus eliethcantillanoae|[1272]|DHJPAR0035345|elachJanzen01 Janzen873|658[0n]|BOLD:ADD0287
Lytopylus angelagonzalezae|[1273]|BIOUG52682-E01|Malaise trap PL12-6C|656[0n]|BOLD:ACP6342
Lytopylus angelagonzalezae|[1274]|BIOUG29868-F08|Malaise trap PL12-3A|594[0n]|BOLD:ACP6342
Lytopylus angelagonzalezae|[1275]|DHJPAR0055351|Anacampsis Janzen353|658[0n]|BOLD:ACP6342
Lytopylus rogerblancoi|[1276]|DHJPAR0051361|gelJanzen01 Janzen356|658[0n]|BOLD:ACJ3683
Lytopylus rogerblancoi|[1277]|DHJPAR0036354|gelJanzen01 Janzen356|658[0n]|BOLD:ACJ3683
Lytopylus rogerblancoi|[1278]|DHJPAR0054536|gelJanzen01 Janzen356|648[0n]|BOLD:ACJ3683
Lytopylus rogerblancoi|[1279]|DHJPAR0051914|gelJanzen01 Janzen356|658[1n]|BOLD:ACJ3683
Lytopylus rogerblancoi|[1280]|DHJPAR0028281|gelJanzen01 Janzen356|621[0n]|BOLD:ACJ3683
Lytopylus rogerblancoi|[1281]|DHJPAR0021199|gelJanzen01 Janzen356|654[3n]|BOLD:ACJ3683
Zacremnops brianbrowni|[1282]|DHJPAR0041189|Microthyris prolongalis|293[0n]
Zacremnops coatlicue|[1283]|DHJPAR0030603|Syllepte belialis|654[0n]|BOLD:AAV3186
Zacremnops coatlicue|[1284]|DHJPAR0015533|Syllepte belialis|654[0n]|BOLD:AAV3186
Zacremnops coatlicue|[1285]|DHJPAR0030604|Syllepte belialis|654[0n]|BOLD:AAV3186
Zacremnops coatlicue|[1286]|DHJPAR0035301|Syllepte belialis|654[0n]|BOLD:AAV3186
Zacremnops coatlicue|[1287]|DHJPAR0030606|Syllepte belialis|654[0n]|BOLD:AAV3186
Zacremnops coatlicue|[1288]|DHJPAR0042822|Syllepte belialis|624[0n]|BOLD:AAV3186
Zacremnops coatlicue|[1289]|DHJPAR0015429|Syllepte belialis|596[4n]|BOLD:AAV3186
Zacremnops coatlicue|[1290]|DHJPAR0030602|Syllepte belialis|402[0n]|BOLD:AAV3186
Zacremnops coatlicue|[1291]|DHJPAR0006773|Syllepte belialis|426[0n]|BOLD:AAV3186
Zacremnops coatlicue|[1292]|DHJPAR0042821|Syllepte belialis|624[0n]|BOLD:AAV3186
Zacremnops coatlicue|[1293]|DHJPAR0042823|Syllepte belialis|626[0n]|BOLD:AAV3186
Zacremnops coatlicue|[1294]|DHJPAR0030607|Syllepte belialis|654[0n]|BOLD:AAV3186
Zacremnops coatlicue|[1295]|DHJPAR0035304|Syllepte belialis|654[0n]|BOLD:AAV3186
Zacremnops coatlicue|[1296]|DHJPAR0035302|Syllepte belialis|654[0n]|BOLD:AAV3186

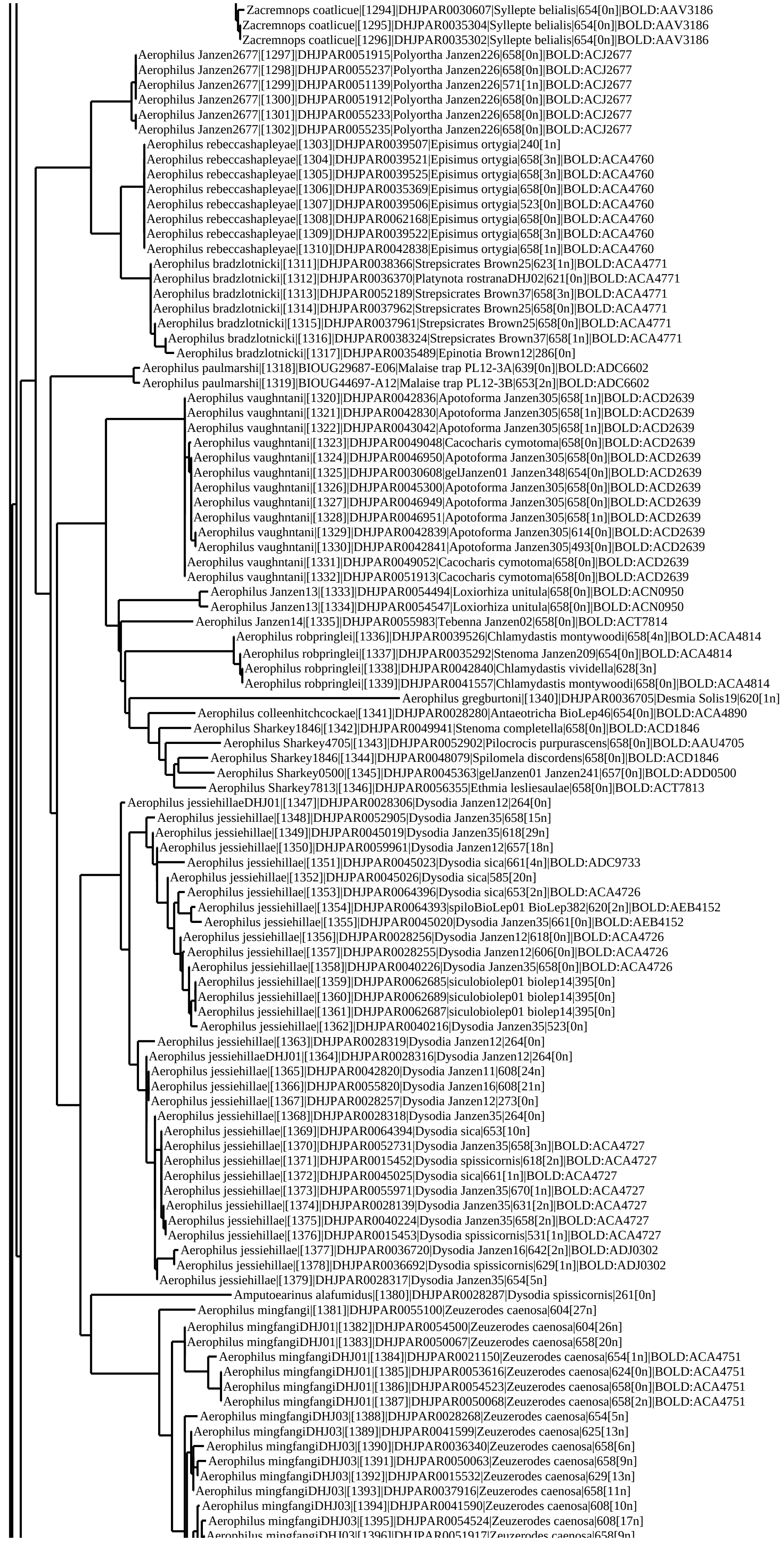
Zacremnops coatlicue|[1294]|DHJPAR0030607|Syllepte belialis|654[0n]|BOLD:AAV3186
Zacremnops coatlicue|[1295]|DHJPAR0035304|Syllepte belialis|654[0n]|BOLD:AAV3186
Zacremnops coatlicue|[1296]|DHJPAR0035302|Syllepte belialis|654[0n]|BOLD:AAV3186
Aerophilus Janzen2677|[1297]|DHJPAR0051915|Polyortha Janzen226|658[0n]|BOLD:ACJ2677
Aerophilus Janzen2677|[1298]|DHJPAR0055237|Polyortha Janzen226|658[0n]|BOLD:ACJ2677
Aerophilus Janzen2677|[1299]|DHJPAR0051139|Polyortha Janzen226|571[1n]|BOLD:ACJ2677
Aerophilus Janzen2677|[1300]|DHJPAR0051912|Polyortha Janzen226|658[0n]|BOLD:ACJ2677
Aerophilus Janzen2677|[1301]|DHJPAR0055233|Polyortha Janzen226|658[0n]|BOLD:ACJ2677
Aerophilus Janzen2677|[1302]|DHJPAR0055235|Polyortha Janzen226|658[0n]|BOLD:ACJ2677
Aerophilus rebeccashapleyae|[1303]|DHJPAR0039507|Episimus ortygia|240[1n]
Aerophilus rebeccashapleyae|[1304]|DHJPAR0039521|Episimus ortygia|658[3n]|BOLD:ACA4760
Aerophilus rebeccashapleyae|[1305]|DHJPAR0039525|Episimus ortygia|658[3n]|BOLD:ACA4760
Aerophilus rebeccashapleyae|[1306]|DHJPAR0035369|Episimus ortygia|658[0n]|BOLD:ACA4760
Aerophilus rebeccashapleyae|[1307]|DHJPAR0039506|Episimus ortygia|523[0n]|BOLD:ACA4760
Aerophilus rebeccashapleyae|[1308]|DHJPAR0062168|Episimus ortygia|658[0n]|BOLD:ACA4760
Aerophilus rebeccashapleyae|[1309]|DHJPAR0039522|Episimus ortygia|658[3n]|BOLD:ACA4760
Aerophilus rebeccashapleyae|[1310]|DHJPAR0042838|Episimus ortygia|658[1n]|BOLD:ACA4760
Aerophilus bradzlotnicki|[1311]|DHJPAR0038366|Strepsicrates Brown25|623[1n]|BOLD:ACA4771
Aerophilus bradzlotnicki|[1312]|DHJPAR0036370|Platynota rostranaDHJ02|621[0n]|BOLD:ACA4771
Aerophilus bradzlotnicki|[1313]|DHJPAR0052189|Strepsicrates Brown37|658[3n]|BOLD:ACA4771
Aerophilus bradzlotnicki|[1314]|DHJPAR0037962|Strepsicrates Brown25|658[0n]|BOLD:ACA4771
Aerophilus bradzlotnicki|[1315]|DHJPAR0037961|Strepsicrates Brown25|658[0n]|BOLD:ACA4771
Aerophilus bradzlotnicki|[1316]|DHJPAR0038324|Strepsicrates Brown37|658[1n]|BOLD:ACA4771
Aerophilus bradzlotnicki|[1317]|DHJPAR0035489|Epinotia Brown12|286[0n]
Aerophilus paulmarshi|[1318]|BIOUG29687-E06|Malaise trap PL12-3A|639[0n]|BOLD:ADC6602
Aerophilus paulmarshi|[1319]|BIOUG44697-A12|Malaise trap PL12-3B|653[2n]|BOLD:ADC6602
Aerophilus vaughntani|[1320]|DHJPAR0042836|Apotoforma Janzen305|658[1n]|BOLD:ACD2639
Aerophilus vaughntani|[1321]|DHJPAR0042830|Apotoforma Janzen305|658[1n]|BOLD:ACD2639
Aerophilus vaughntani|[1322]|DHJPAR0043042|Apotoforma Janzen305|658[1n]|BOLD:ACD2639
Aerophilus vaughntani|[1323]|DHJPAR0049048|Cacocharis cymotoma|658[0n]|BOLD:ACD2639
Aerophilus vaughntani|[1324]|DHJPAR0046950|Apotoforma Janzen305|658[0n]|BOLD:ACD2639
Aerophilus vaughntani|[1325]|DHJPAR0030608|gelJanzen01 Janzen348|654[0n]|BOLD:ACD2639
Aerophilus vaughntani|[1326]|DHJPAR0045300|Apotoforma Janzen305|658[0n]|BOLD:ACD2639
Aerophilus vaughntani|[1327]|DHJPAR0046949|Apotoforma Janzen305|658[0n]|BOLD:ACD2639
Aerophilus vaughntani|[1328]|DHJPAR0046951|Apotoforma Janzen305|658[1n]|BOLD:ACD2639
Aerophilus vaughntani|[1329]|DHJPAR0042839|Apotoforma Janzen305|614[0n]|BOLD:ACD2639
Aerophilus vaughntani|[1330]|DHJPAR0042841|Apotoforma Janzen305|493[0n]|BOLD:ACD2639
Aerophilus vaughntani|[1331]|DHJPAR0049052|Cacocharis cymotoma|658[0n]|BOLD:ACD2639
Aerophilus vaughntani|[1332]|DHJPAR0051913|Cacocharis cymotoma|658[0n]|BOLD:ACD2639
Aerophilus Janzen13|[1333]|DHJPAR0054494|Loxiorhiza unitula|658[0n]|BOLD:ACN0950
Aerophilus Janzen13|[1334]|DHJPAR0054547|Loxiorhiza unitula|658[0n]|BOLD:ACN0950
Aerophilus Janzen14|[1335]|DHJPAR0055983|Tebenna Janzen02|658[0n]|BOLD:ACT7814
Aerophilus robpringlei|[1336]|DHJPAR0039526|Chlamydastis montywoodi|658[4n]|BOLD:ACA4814
Aerophilus robpringlei|[1337]|DHJPAR0035292|Stenoma Janzen209|654[0n]|BOLD:ACA4814
Aerophilus robpringlei|[1338]|DHJPAR0042840|Chlamydastis vividella|628[3n]
Aerophilus robpringlei|[1339]|DHJPAR0041557|Chlamydastis montywoodi|658[0n]|BOLD:ACA4814
Aerophilus gregburtoni|[1340]|DHJPAR0036705|Desmia Solis19|620[1n]
Aerophilus colleenhitchcockae|[1341]|DHJPAR0028280|Antaeotricha BioLep46|654[0n]|BOLD:ACA4890
Aerophilus Sharkey1846|[1342]|DHJPAR0049941|Stenoma completella|658[0n]|BOLD:ACD1846
Aerophilus Sharkey4705|[1343]|DHJPAR0052902|Pilocrocis purpurascens|658[0n]|BOLD:AAU4705
Aerophilus Sharkey1846|[1344]|DHJPAR0048079|Spilomela discordens|658[0n]|BOLD:ACD1846
Aerophilus Sharkey0500|[1345]|DHJPAR0045363|gelJanzen01 Janzen241|657[0n]|BOLD:ADD0500
Aerophilus Sharkey7813|[1346]|DHJPAR0056355|Ethmia lesliesaulae|658[0n]|BOLD:ACT7813
Aerophilus jessiehillaeDHJ01|[1347]|DHJPAR0028306|Dysodia Janzen12|264[0n]
Aerophilus jessiehillae|[1348]|DHJPAR0052905|Dysodia Janzen35|658[15n]
Aerophilus jessiehillae|[1349]|DHJPAR0045019|Dysodia Janzen35|618[29n]
Aerophilus jessiehillae|[1350]|DHJPAR0059961|Dysodia Janzen12|657[18n]
Aerophilus jessiehillae|[1351]|DHJPAR0045023|Dysodia sica|661[4n]|BOLD:ADC9733
Aerophilus jessiehillae|[1352]|DHJPAR0045026|Dysodia sica|585[20n]
Aerophilus jessiehillae|[1353]|DHJPAR0064396|Dysodia sica|653[2n]|BOLD:ACA4726
Aerophilus jessiehillae|[1354]|DHJPAR0064393|spiloBioLep01 BioLep382|620[2n]|BOLD:AEB4152
Aerophilus jessiehillae|[1355]|DHJPAR0045020|Dysodia Janzen35|661[0n]|BOLD:AEB4152
Aerophilus jessiehillae|[1356]|DHJPAR0028256|Dysodia Janzen12|618[0n]|BOLD:ACA4726
Aerophilus jessiehillae|[1357]|DHJPAR0028255|Dysodia Janzen12|606[0n]|BOLD:ACA4726
Aerophilus jessiehillae|[1358]|DHJPAR0040226|Dysodia Janzen35|658[0n]|BOLD:ACA4726
Aerophilus jessiehillae|[1359]|DHJPAR0062685|siculobiolep01 biolep14|395[0n]
Aerophilus jessiehillae|[1360]|DHJPAR0062689|siculobiolep01 biolep14|395[0n]
Aerophilus jessiehillae|[1361]|DHJPAR0062687|siculobiolep01 biolep14|395[0n]
Aerophilus jessiehillae|[1362]|DHJPAR0040216|Dysodia Janzen35|523[0n]
Aerophilus jessiehillae|[1363]|DHJPAR0028319|Dysodia Janzen12|264[0n]
Aerophilus jessiehillaeDHJ01|[1364]|DHJPAR0028316|Dysodia Janzen12|264[0n]
Aerophilus jessiehillae|[1365]|DHJPAR0042820|Dysodia Janzen11|608[24n]
Aerophilus jessiehillae|[1366]|DHJPAR0055820|Dysodia Janzen16|608[21n]
Aerophilus jessiehillae|[1367]|DHJPAR0028257|Dysodia Janzen12|273[0n]
Aerophilus jessiehillae|[1368]|DHJPAR0028318|Dysodia Janzen35|264[0n]
Aerophilus jessiehillae|[1369]|DHJPAR0064394|Dysodia sica|653[10n]
Aerophilus jessiehillae|[1370]|DHJPAR0052731|Dysodia Janzen35|658[3n]|BOLD:ACA4727
Aerophilus jessiehillae|[1371]|DHJPAR0015452|Dysodia spissicornis|618[2n]|BOLD:ACA4727
Aerophilus jessiehillae|[1372]|DHJPAR0045025|Dysodia sica|661[1n]|BOLD:ACA4727
Aerophilus jessiehillae|[1373]|DHJPAR0055971|Dysodia Janzen35|670[1n]|BOLD:ACA4727
Aerophilus jessiehillae|[1374]|DHJPAR0028139|Dysodia Janzen35|631[2n]|BOLD:ACA4727
Aerophilus jessiehillae|[1375]|DHJPAR0040224|Dysodia Janzen35|658[2n]|BOLD:ACA4727
Aerophilus jessiehillae|[1376]|DHJPAR0015453|Dysodia spissicornis|531[1n]|BOLD:ACA4727
Aerophilus jessiehillae|[1377]|DHJPAR0036720|Dysodia Janzen16|642[2n]|BOLD:ADJ0302
Aerophilus jessiehillae|[1378]|DHJPAR0036692|Dysodia spissicornis|629[1n]|BOLD:ADJ0302
Aerophilus jessiehillae|[1379]|DHJPAR0028317|Dysodia Janzen35|654[5n]
Amputoearinus alafumidus|[1380]|DHJPAR0028287|Dysodia spissicornis|261[0n]
Aerophilus mingfangi|[1381]|DHJPAR0055100|Zeuzerodes caenosa|604[27n]
Aerophilus mingfangiDHJ01|[1382]|DHJPAR0054500|Zeuzerodes caenosa|604[26n]
Aerophilus mingfangiDHJ01|[1383]|DHJPAR0050067|Zeuzerodes caenosa|658[20n]
Aerophilus mingfangiDHJ01|[1384]|DHJPAR0021150|Zeuzerodes caenosa|654[1n]|BOLD:ACA4751
Aerophilus mingfangiDHJ01|[1385]|DHJPAR0053616|Zeuzerodes caenosa|624[0n]|BOLD:ACA4751
Aerophilus mingfangiDHJ01|[1386]|DHJPAR0054523|Zeuzerodes caenosa|658[0n]|BOLD:ACA4751
Aerophilus mingfangiDHJ01|[1387]|DHJPAR0050068|Zeuzerodes caenosa|658[2n]|BOLD:ACA4751
Aerophilus mingfangiDHJ03|[1388]|DHJPAR0028268|Zeuzerodes caenosa|654[5n]
Aerophilus mingfangiDHJ03|[1389]|DHJPAR0041599|Zeuzerodes caenosa|625[13n]
Aerophilus mingfangiDHJ03|[1390]|DHJPAR0036340|Zeuzerodes caenosa|658[6n]
Aerophilus mingfangiDHJ03|[1391]|DHJPAR0050063|Zeuzerodes caenosa|658[9n]
Aerophilus mingfangiDHJ03|[1392]|DHJPAR0015532|Zeuzerodes caenosa|629[13n]
Aerophilus mingfangiDHJ03|[1393]|DHJPAR0037916|Zeuzerodes caenosa|658[11n]
Aerophilus mingfangiDHJ03|[1394]|DHJPAR0041590|Zeuzerodes caenosa|608[10n]
Aerophilus mingfangiDHJ03|[1395]|DHJPAR0054524|Zeuzerodes caenosa|608[17n]
Aerophilus mingfangiDHJ03|[1396]|DHJPAR0051917|Zeuzerodes caenosa|658[9n]

[illegible]
Aerophilus mingfangiDHJ03|[1394]|DHJPAR0041590|Zeuzerodes caenosa|608[10n]
Aerophilus mingfangiDHJ03|[1395]|DHJPAR0054524|Zeuzerodes caenosa|608[17n]
Aerophilus mingfangiDHJ03|[1396]|DHJPAR0051917|Zeuzerodes caenosa|658[9n]
Aerophilus mingfangiDHJ03|[1397]|DHJPAR0045027|Zeuzerodes caenosa|626[0n]|BOLD:ACN1059
Aerophilus mingfangiDHJ03|[1398]|DHJPAR0045024|Zeuzerodes caenosa|661[0n]|BOLD:ACN1059
Aerophilus mingfangiDHJ03|[1399]|DHJPAR0054505|Zeuzerodes caenosa|658[0n]|BOLD:ACN1059
Aerophilus mingfangiDHJ03|[1400]|DHJPAR0052692|Zeuzerodes caenosa|658[0n]|BOLD:ACN1059
Aerophilus mingfangiDHJ03|[1401]|DHJPAR0038806|Zeuzerodes caenosa|658[5n]|BOLD:ACN1059
Aerophilus mingfangiDHJ03|[1402]|DHJPAR0037928|Zeuzerodes caenosa|658[0n]|BOLD:ACN1059
Aerophilus mingfangiDHJ03|[1403]|DHJPAR0021167|Zeuzerodes caenosa|654[2n]|BOLD:ACN1059
Aerophilus mingfangiDHJ03|[1404]|DHJPAR0051356|Zeuzerodes caenosa|658[3n]|BOLD:ACN1059
Aerophilus mingfangiDHJ03|[1405]|DHJPAR0041593|Zeuzerodes caenosa|658[0n]|BOLD:ACN1059
Aerophilus mingfangiDHJ03|[1406]|DHJPAR0051367|Zeuzerodes caenosa|658[1n]|BOLD:ACN1059
Aerophilus mingfangiDHJ03|[1407]|DHJPAR0051375|Zeuzerodes caenosa|658[0n]|BOLD:ACN1059
Aerophilus mingfangiDHJ04|[1408]|DHJPAR0045787|Zeuzerodes caenosa|421[0n]
Aerophilus mingfangiDHJ04|[1409]|DHJPAR0041170|Zeuzerodes caenosa|624[6n]|BOLD:ADD0377
Aerophilus mingfangiDHJ04|[1410]|DHJPAR0041191|Zeuzerodes caenosa|624[0n]|BOLD:ADD0377
Aerophilus mingfangiDHJ04|[1411]|DHJPAR0045009|Zeuzerodes caenosa|411[0n]
Aerophilus mingfangiDHJ04|[1412]|DHJPAR0036343|Zeuzerodes caenosa|409[2n]
Aerophilus mingfangiDHJ04|[1413]|DHJPAR0036342|Zeuzerodes caenosa|408[0n]
Aerophilus mingfangiDHJ04|[1414]|DHJPAR0041162|Zeuzerodes caenosa|624[1n]|BOLD:ADD0377
Aerophilus mingfangiDHJ03|[1415]|DHJPAR0028031|Zeuzerodes caenosa|658[1n]|BOLD:ACN1059
Aerophilus mingfangiDHJ03|[1416]|DHJPAR0051672|Zeuzerodes caenosa|626[2n]|BOLD:ACN1059
Aerophilus mingfangiDHJ03|[1417]|DHJPAR0036723|Zeuzerodes caenosa|658[0n]|BOLD:ACN1059
Aerophilus mingfangiDHJ03|[1418]|DHJPAR0052196|Zeuzerodes caenosa|657[3n]|BOLD:ACN1059
Aerophilus mingfangiDHJ03|[1419]|DHJPAR0055973|Zeuzerodes caenosa|642[1n]|BOLD:ACN1059
Aerophilus mingfangiDHJ03|[1420]|DHJPAR0053617|Zeuzerodes caenosa|658[0n]|BOLD:ACN1059
Aerophilus mingfangi|[1421]|DHJPAR0051347|Zeuzerodes caenosa|613[21n]
Aerophilus mingfangi|[1422]|DHJPAR0023291|Zeuzerodes caenosa|654[18n]
Aerophilus mingfangiDHJ02|[1423]|DHJPAR0053620|Zeuzerodes caenosa|658[1n]|BOLD:ACA4752
Aerophilus mingfangiDHJ02|[1424]|DHJPAR0045028|Zeuzerodes caenosa|657[3n]|BOLD:ACA4752
Aerophilus mingfangi|[1425]|DHJPAR0054541|Zeuzerodes caenosa|658[5n]
Aerophilus mingfangiDHJ02|[1426]|DHJPAR0040346|Zeuzerodes caenosa|658[1n]|BOLD:ACA4752
Aerophilus mingfangi|[1427]|DHJPAR0052694|Zeuzerodes caenosa|603[20n]
Aerophilus mingfangiDHJ03|[1428]|DHJPAR0051352|Zeuzerodes caenosa|613[8n]
Aerophilus mingfangiDHJ03|[1429]|DHJPAR0053614|Zeuzerodes caenosa|658[1n]|BOLD:ACA4752
Aerophilus mingfangiDHJ03|[1430]|DHJPAR0037927|Zeuzerodes caenosa|658[2n]|BOLD:ACA4752
Aerophilus mingfangiDHJ03|[1431]|DHJPAR0051353|Zeuzerodes caenosa|658[11n]
Zamicrodus josefernandezi|[1432]|DHJPAR0048728|Quasimellana sethos|658[0n]|BOLD:ACC5208
Zamicrodus josefernandezi|[1433]|DHJPAR0046954|Quasimellana sethos|658[0n]|BOLD:ACC5208
Zamicrodus josefernandezi|[1434]|DHJPAR0048733|Quasimellana sethos|658[1n]|BOLD:ACC5208
Pneumagathis erythrogastra|[1435]|DHJPAR0016422|Carrhenes canescens|517[17n]
Pneumagathis erythrogastra|[1436]|DHJPAR0029299|Astraptes anaphus annetta|641[0n]|BOLD:AAV3035
Aphelagathis rociofernandezae|[1437]|DHJPAR0049654|Morys lydeDHJ01|658[12n]
Aphelagathis rociofernandezae|[1438]|DHJPAR0009368|Morys valerius valda|654[2n]|BOLD:AAN8643
Aphelagathis rociofernandezae|[1439]|DHJPAR0064004|Mnasicles hicetaon|637[0n]|BOLD:AAN8643
Aphelagathis rociofernandezae|[1440]|DHJPAR0058694|Anthoptus epictetus|658[0n]|BOLD:AAN8643
Aphelagathis rociofernandezae|[1441]|DHJPAR0057741|Lerema liris|658[1n]|BOLD:AAN8643
Pneumagathis erythrogastra|[1442]|DHJPAR0006845|Epargyreus Burns02|273[3n]
Pneumagathis erythrogastra|[1443]|DHJPAR0006784|Carrhenes fuscescens|273[7n]
Pneumagathis erythrogastra|[1444]|DHJPAR0006821|Cabares potrillo|273[12n]
Pneumagathis erythrogastra|[1445]|DHJPAR0006850|Antigonus erosus|276[3n]
Pneumagathis erythrogastra|[1446]|DHJPAR0006786|Cabares potrillo|276[6n]
Pneumagathis erythrogastra|[1447]|DHJPAR0006852|Antigonus erosus|276[9n]
Pneumagathis erythrogastra|[1448]|DHJPAR0006832|Epargyreus Burns02|279[3n]
Pneumagathis erythrogastra|[1449]|DHJPAR0022190|Epargyreus Burns11|651[0n]|BOLD:AAV3035
Pneumagathis erythrogastra|[1450]|DHJPAR0006823|Cabares potrillo|276[10n]
Pneumagathis erythrogastra|[1451]|DHJPAR0006792|Astraptes anaphus annetta|282[11n]
Pneumagathis erythrogastra|[1452]|DHJPAR0006840|Astraptes anaphus annetta|282[10n]
Pneumagathis erythrogastra|[1453]|DHJPAR0006816|Epargyreus Burns02|276[4n]
Pneumagathis erythrogastra|[1454]|DHJPAR0006834|Epargyreus Burns02|273[0n]
Pneumagathis erythrogastra|[1455]|DHJPAR0006798|Epargyreus Burns02|282[4n]
Pneumagathis erythrogastra|[1456]|DHJPAR0006829|Epargyreus Burns02|291[0n]
Pneumagathis erythrogastra|[1457]|DHJPAR0006785|Epargyreus Burns02|276[0n]
Pneumagathis erythrogastra|[1458]|DHJPAR0006796|Epargyreus Burns02|276[0n]
Pneumagathis erythrogastra|[1459]|DHJPAR0021207|Antigonus erosus|627[0n]|BOLD:AAV3035
Pneumagathis erythrogastra|[1460]|DHJPAR0006830|Epargyreus Burns02|276[0n]
Pneumagathis erythrogastra|[1461]|DHJPAR0006795|Urbanus esmeraldus|276[0n]
Pneumagathis erythrogastra|[1462]|DHJPAR0006827|Epargyreus Burns02|279[7n]
Pneumagathis erythrogastra|[1463]|DHJPAR0006802|Urbanus esmeraldus|249[8n]
Pneumagathis erythrogastra|[1464]|DHJPAR0006811|Gesta invisius|279[7n]
Pneumagathis erythrogastra|[1465]|DHJPAR0028288|Antigonus erosus|283[14n]
Pneumagathis erythrogastra|[1466]|DHJPAR0006771|Epargyreus Burns02|273[1n]
Pneumagathis erythrogastra|[1467]|DHJPAR0006817|Urbanus dorantes|282[9n]
Pneumagathis erythrogastra|[1468]|DHJPAR0006782|Epargyreus Burns02|276[11n]
Pneumagathis erythrogastra|[1469]|DHJPAR0006841|Antigonus erosus|276[11n]
Pneumagathis erythrogastra|[1470]|DHJPAR0006815|Gesta invisius|276[10n]
Pneumagathis erythrogastra|[1471]|DHJPAR0006826|Epargyreus Burns02|276[12n]
Pneumagathis erythrogastra|[1472]|DHJPAR0006844|Astraptes anaphus annetta|282[11n]
Pneumagathis erythrogastra|[1473]|DHJPAR0006789|Cabares potrillo|282[12n]
Pneumagathis erythrogastra|[1474]|DHJPAR0006824|Cabares potrillo|276[10n]
Pneumagathis erythrogastra|[1475]|DHJPAR0007215|Urbanus esmeraldus|282[2n]
Pneumagathis erythrogastra|[1476]|DHJPAR0007206|Antigonus erosus|282[4n]
Pneumagathis erythrogastra|[1477]|DHJPAR0006847|Urbanus proteus|282[6n]
Pneumagathis erythrogastra|[1478]|DHJPAR0007213|Gesta invisius|282[2n]
Pneumagathis erythrogastra|[1479]|DHJPAR0007205|Epargyreus Burns02|246[5n]
Pneumagathis erythrogastra|[1480]|DHJPAR0006820|Epargyreus Burns02|282[10n]
Pneumagathis erythrogastra|[1481]|DHJPAR0006777|Epargyreus Burns11|276[4n]
Pneumagathis erythrogastra|[1482]|DHJPAR0006778|Epargyreus Burns02|276[6n]
Pneumagathis erythrogastra|[1483]|DHJPAR0006776|Astraptes anaphus annetta|273[4n]
Pneumagathis erythrogastra|[1484]|DHJPAR0006764|Antigonus erosus|273[1n]
Pneumagathis erythrogastra|[1485]|DHJPAR0007224|Antigonus erosus|291[9n]
Pneumagathis erythrogastra|[1486]|DHJPAR0006818|Epargyreus Burns02|276[7n]
Pneumagathis erythrogastra|[1487]|DHJPAR0021169|Chioides catillus|277[33n]
Pneumagathis erythrogastra|[1488]|DHJPAR0006828|Epargyreus Burns02|282[7n]
Pneumagathis erythrogastra|[1489]|DHJPAR0006833|Urbanus esmeraldus|282[8n]
Pneumagathis erythrogastra|[1490]|DHJPAR0006772|Antigonus erosus|273[3n]
Pneumagathis erythrogastra|[1491]|DHJPAR0007217|Urbanus evona|243[3n]
Pneumagathis erythrogastra|[1492]|DHJPAR0006800|Urbanus esta|282[7n]
Pneumagathis erythrogastra|[1493]|DHJPAR0006803|Urbanus esta|267[4n]
Pneumagathis erythrogastra|[1494]|DHJPAR0007219|Antigonus erosus|246[0n]
Pneumagathis erythrogastra|[1495]|DHJPAR0006808|Urbanus proteus|282[7n]
[illegible]

Pneumagathis erythrogastra|[1493]|DHJPAR0006803|Urbanus esta|267[4n]
Pneumagathis erythrogastra|[1494]|DHJPAR0007219|Antigonus erosus|246[0n]
Pneumagathis erythrogastra|[1495]|DHJPAR0006808|Urbanus proteus|282[7n]
Pneumagathis erythrogastra|[1496]|DHJPAR0021209|Antigonus erosus|280[17n]
Pneumagathis erythrogastra|[1497]|DHJPAR0006768|Epargyreus Burns02|225[2n]
Pneumagathis erythrogastra|[1498]|DHJPAR0021171|Chioides catillus|261[30n]
Pneumagathis erythrogastra|[1499]|DHJPAR0006763|Antigonus erosus|282[6n]
Pneumagathis erythrogastra|[1500]|DHJPAR0006780|Epargyreus Burns02|279[4n]
Pneumagathis erythrogastra|[1501]|DHJPAR0006761|Antigonus erosus|276[5n]
Pneumagathis erythrogastra|[1502]|DHJPAR0006779|Epargyreus Burns02|279[1n]
Pneumagathis erythrogastra|[1503]|DHJPAR0007222|Urbanus proteus|282[0n]
Pneumagathis erythrogastra|[1504]|DHJPAR0006766|Antigonus erosus|279[3n]
Pneumagathis erythrogastra|[1505]|DHJPAR0006767|Antigonus erosus|273[1n]
Pneumagathis erythrogastra|[1506]|DHJPAR0006765|Antigonus erosus|279[1n]
Pneumagathis erythrogastra|[1507]|DHJPAR0006843|Astraptes anaphus annetta|282[9n]
Pneumagathis erythrogastra|[1508]|DHJPAR0006809|Epargyreus Burns02|273[3n]
Pneumagathis erythrogastra|[1509]|DHJPAR0006825|Epargyreus Burns02|276[3n]
Pneumagathis erythrogastra|[1510]|DHJPAR0006848|Antigonus erosus|282[0n]
Pneumagathis erythrogastra|[1511]|DHJPAR0006787|Epargyreus Burns02|252[0n]
Pneumagathis erythrogastra|[1512]|DHJPAR0006807|Urbanus esta|264[0n]
Pneumagathis erythrogastra|[1513]|DHJPAR0021166|Paches loxus|654[0n]|BOLD:AAV3035
Pneumagathis erythrogastra|[1514]|DHJPAR0021161|Chioides catillus|654[0n]|BOLD:AAV3035
Pneumagathis erythrogastra|[1515]|DHJPAR0021164|Antigonus erosus|654[0n]|BOLD:AAV3035
Pneumagathis erythrogastra|[1516]|DHJPAR0006793|Epargyreus Burns02|273[0n]
Pneumagathis erythrogastra|[1517]|DHJPAR0021160|Urbanus proteus|654[0n]|BOLD:AAV3035
Pneumagathis erythrogastra|[1518]|DHJPAR0016421|Antigonus erosus|654[0n]|BOLD:AAV3035
Pneumagathis erythrogastra|[1519]|DHJPAR0021206|Astraptes anaphus annetta|654[0n]|BOLD:AAV3035
Pneumagathis erythrogastra|[1520]|DHJPAR0021162|Chioides catillus|654[1n]|BOLD:AAV3035
Pneumagathis erythrogastra|[1521]|DHJPAR0006806|Gesta invisius|273[0n]
Pneumagathis erythrogastra|[1522]|DHJPAR0006846|Cabares potrillo|273[0n]
Pneumagathis erythrogastra|[1523]|DHJPAR0006783|Epargyreus Burns02|282[4n]
Pneumagathis erythrogastra|[1524]|DHJPAR0006810|Gesta invisius|276[0n]
Pneumagathis erythrogastra|[1525]|DHJPAR0006791|Urbanus ‘proteus group‘|282[7n]
Pneumagathis erythrogastra|[1526]|DHJPAR0006839|Epargyreus Burns02|282[4n]
Pneumagathis erythrogastra|[1527]|DHJPAR0006760|Antigonus erosus|273[0n]
Pneumagathis erythrogastra|[1528]|DHJPAR0006762|Antigonus erosus|273[0n]
Pneumagathis erythrogastra|[1529]|DHJPAR0006799|Epargyreus Burns02|282[5n]
Pneumagathis erythrogastra|[1530]|DHJPAR0006770|Astraptes anaphus annetta|279[0n]
Pneumagathis erythrogastra|[1531]|DHJPAR0006769|Antigonus erosus|276[0n]
Pneumagathis erythrogastra|[1532]|DHJPAR0006812|Epargyreus Burns02|276[0n]
Pneumagathis erythrogastra|[1533]|DHJPAR0006788|Epargyreus Burns02|276[0n]
Pneumagathis erythrogastra|[1534]|DHJPAR0006805|Gesta invisius|276[0n]
Pneumagathis erythrogastra|[1535]|DHJPAR0006797|Epargyreus Burns02|276[0n]
Pneumagathis erythrogastra|[1536]|DHJPAR0006813|Epargyreus Burns02|276[0n]
Pneumagathis erythrogastra|[1537]|DHJPAR0006849|Urbanus esta|276[0n]
Pneumagathis erythrogastra|[1538]|DHJPAR0006837|Epargyreus Burns02|276[0n]
Pneumagathis erythrogastra|[1539]|DHJPAR0006836|Epargyreus Burns02|291[0n]
Pneumagathis erythrogastra|[1540]|DHJPAR0021159|Paches loxus|624[0n]|BOLD:AAV3035
Pneumagathis erythrogastra|[1541]|DHJPAR0006804|Cabares potrillo|288[0n]
Pneumagathis erythrogastra|[1542]|DHJPAR0007216|Gesta invisius|282[0n]
Pneumagathis erythrogastra|[1543]|DHJPAR0007221|Cabares potrillo|282[0n]
Pneumagathis erythrogastra|[1544]|DHJPAR0007225|Epargyreus Burns02|282[0n]
Pneumagathis erythrogastra|[1545]|DHJPAR0006781|Epargyreus Burns02|282[0n]
Pneumagathis erythrogastra|[1546]|DHJPAR0006838|Epargyreus Burns02|282[0n]
Pneumagathis erythrogastra|[1547]|DHJPAR0006835|Epargyreus Burns11|282[0n]
Pneumagathis erythrogastra|[1548]|DHJPAR0006814|Gesta invisius|285[0n]
Pneumagathis erythrogastra|[1549]|DHJPAR0006801|Antigonus erosus|282[0n]
Pneumagathis erythrogastra|[1550]|DHJPAR0006775|Urbanus proteus|279[0n]
Pneumagathis erythrogastra|[1551]|DHJPAR0006851|Antigonus erosus|285[0n]
Pneumagathis erythrogastra|[1552]|DHJPAR0007208|Antigonus erosus|282[1n]
Pneumagathis erythrogastra|[1553]|DHJPAR0007212|Gesta invisius|249[0n]
Pneumagathis erythrogastra|[1554]|DHJPAR0007218|Antigonus erosus|252[2n]
Pneumagathis erythrogastra|[1555]|DHJPAR0007209|Antigonus erosus|243[2n]
Pneumagathis erythrogastra|[1556]|DHJPAR0059606|Urbanus esta|628[23n]
Pneumagathis erythrogastra|[1557]|DHJPAR0007226|Epargyreus Burns11|249[0n]
Pneumagathis erythrogastra|[1558]|DHJPAR0007211|Gesta invisius|249[0n]
Pneumagathis erythrogastra|[1559]|DHJPAR0038612|Carrhenes canescens|285[1n]
Pneumagathis erythrogastra|[1560]|DHJPAR0036346|Cabares potrillo|290[0n]
Pneumagathis erythrogastra|[1561]|DHJPAR0029300|Antigonus erosus|308[0n]
Pneumagathis erythrogastra|[1562]|DHJPAR0007210|Antigonus erosus|246[0n]
Lytopylus Sharkey02|[1563]|DHJPAR0028296|elachJanzen01 Janzen900|630[0n]|BOLD:ACB1289
Lytopylus Sharkey02|[1564]|DHJPAR0049050|elachJanzen01 Janzen185|658[0n]|BOLD:ACB1289
Lytopylus Sharkey01|[1565]|DHJPAR0065343|elachJanzen01 Janzen527|658[0n]|BOLD:ACJ2185
Lytopylus Sharkey01|[1566]|DHJPAR0050946|elachJanzen01 Janzen527|658[0n]|BOLD:ACJ2185
Lytopylus Sharkey01|[1567]|DHJPAR0057411|elachJanzen01 Janzen527|658[0n]|BOLD:ACJ2185
Lytopylus Sharkey01|[1568]|DHJPAR0050948|elachJanzen01 Janzen527|658[0n]|BOLD:ACJ2185
Lytopylus Janzen1291|[1569]|DHJPAR0055239|Oecophora Janzen52|658[0n]|BOLD:ACB1291
Lytopylus Janzen1291|[1570]|DHJPAR0049053|Oecophora Janzen52|658[0n]|BOLD:ACB1291
Lytopylus hartmanguidoi|[1571]|DHJPAR0039513|Psilocorsis Janzen369|658[0n]|BOLD:ADA3837
Lytopylus hartmanguidoi|[1572]|DHJPAR0042844|Antaeotricha Janzen126|658[0n]|BOLD:ADA3837
Lytopylus hartmanguidoi|[1573]|DHJPAR0042831|Antaeotricha Janzen126|658[0n]|BOLD:ADA3837
Lytopylus hartmanguidoi|[1574]|DHJPAR0039524|elachJanzen01 Janzen392|658[0n]|BOLD:ADA3837
Lytopylus hartmanguidoi|[1575]|DHJPAR0039514|elachJanzen01 Janzen392|282[0n]
Lytopylus hartmanguidoi|[1576]|BIOUG28246-D08|ESG Malaise Trap|603[0n]|BOLD:ADA3837
Therophilus penteadodiasae|[1577]|DHJPAR0055292|Antaeotricha Janzen146|658[0n]|BOLD:ABZ8830
Therophilus penteadodiasae|[1578]|DHJPAR0055209|Antaeotricha Janzen146|658[0n]|BOLD:ABZ8830
Therophilus penteadodiasae|[1579]|DHJPAR0048238|Antaeotricha Janzen366|658[0n]|BOLD:ABZ8830
Therophilus penteadodiasae|[1580]|DHJPAR0048221|Antaeotricha Janzen366|658[0n]|BOLD:ABZ8830
Therophilus penteadodiasae|[1581]|DHJPAR0055544|Antaeotricha Janzen146|661[0n]|BOLD:ABZ8830
Therophilus bobwhartoni|[1582]|DHJPAR0041864|elachJanzen01 Janzen161|658[0n]|BOLD:AAY4688
Therophilus bobwhartoni|[1583]|DHJPAR0062169|elachJanzen01 Janzen161|624[0n]|BOLD:AAY4688
Therophilus gracewoodae|[1584]|DHJPAR0039519|Rhodoneura terminalis|614[0n]|BOLD:ADD0457
Therophilus gracewoodae|[1585]|DHJPAR0064507|Rhodoneura terminalis|633[0n]|BOLD:ADD0457
Therophilus montywoodi|[1586]|DHJPAR0040068|Strepsicrates Brown25|658[0n]|BOLD:ADD0519
Therophilus montywoodi|[1587]|DHJPAR0038338|Strepsicrates Brown25|658[0n]|BOLD:ADD0519
Lytopylus Sharkey23|[1588]|DHJPAR0041571|Antaeotricha Janzen31|658[0n]|BOLD:ADD0188
Lytopylus andreamezae|[1589]|DHJPAR0051349|Antaeotricha Janzen09|658[2n]|BOLD:ABU8129
Lytopylus andreamezae|[1590]|DHJPAR0038910|Antaeotricha Janzen07|632[3n]|BOLD:ABU8129
Lytopylus andreamezae|[1591]|DHJPAR0053652|Antaeotricha Janzen09|658[0n]|BOLD:ABU8129
Lytopylus andreamezae|[1592]|DHJPAR0049649|Stenoma Janzen144|658[0n]|BOLD:ABU8129
Lytopylus andreamezae|[1593]|DHJPAR0036722|Antaeotricha Janzen09|645[0n]|BOLD:ABU8129
Lytopylus andreamezae|[1594]|DHJPAR0036721|Antaeotricha Janzen09|624[0n]|BOLD:ABU8129
Lytopylus andreamezae|[1595]|DHJPAR0036686|Antaeotricha Janzen09|624[0n]|BOLD:ABU8129

Lytopylus andreamezae|[1593]|DHJPAR0036722|Antaeotricha Janzen09|645[0n]|BOLD:ABU8129
Lytopylus andreamezae|[1594]|DHJPAR0036721|Antaeotricha Janzen09|624[0n]|BOLD:ABU8129
Lytopylus andreamezae|[1595]|DHJPAR0036686|Antaeotricha Janzen09|624[0n]|BOLD:ABU8129
Lytopylus andreamezae|[1596]|DHJPAR0054520|Antaeotricha ribbei|658[1n]|BOLD:ABU8129
Lytopylus andreamezae|[1597]|DHJPAR0054518|Antaeotricha Janzen09|658[1n]|BOLD:ABU8129
Lytopylus andreamezae|[1598]|DHJPAR0035229|Antaeotricha Janzen09|621[0n]|BOLD:ABU8129
Lytopylus andreamezae|[1599]|DHJPAR0035231|Antaeotricha Janzen09|654[0n]|BOLD:ABU8129
Lytopylus andreamezae|[1600]|DHJPAR0053618|Antaeotricha Janzen09|664[0n]|BOLD:ABU8129
Lytopylus andreamezae|[1601]|DHJPAR0053658|Antaeotricha Janzen09|658[0n]|BOLD:ABU8129
Lytopylus andreamezae|[1602]|DHJPAR0051360|Antaeotricha Janzen09|658[0n]|BOLD:ABU8129
Lytopylus andreamezae|[1603]|DHJPAR0051365|Antaeotricha Janzen12|658[0n]|BOLD:ABU8129
Lytopylus andreamezae|[1604]|DHJPAR0035294|Antaeotricha Janzen09|654[0n]|BOLD:ABU8129
Lytopylus andreamezae|[1605]|DHJPAR0053649|Antaeotricha Janzen09|658[0n]|BOLD:ABU8129
Lytopylus andreamezae|[1606]|DHJPAR0051370|Antaeotricha Janzen09|658[0n]|BOLD:ABU8129
Lytopylus andreamezae|[1607]|DHJPAR0054515|Antaeotricha Janzen09|658[0n]|BOLD:ABU8129
Lytopylus andreamezae|[1608]|DHJPAR0046956|Antaeotricha Janzen09|658[0n]|BOLD:ABU8129
Lytopylus andreamezae|[1609]|DHJPAR0046745|Antaeotricha Janzen07|658[0n]|BOLD:ABU8129
Lytopylus andreamezae|[1610]|DHJPAR0035298|Antaeotricha Janzen09|654[0n]|BOLD:ABU8129
Lytopylus andreamezae|[1611]|DHJPAR0036371|Antaeotricha Janzen09|621[2n]|BOLD:ABU8129
Lytopylus andreamezae|[1612]|DHJPAR0053645|Antaeotricha Janzen07|658[0n]|BOLD:ABU8129
Lytopylus andreamezae|[1613]|DHJPAR0054519|Antaeotricha Janzen07|658[0n]|BOLD:ABU8129
Lytopylus andreamezae|[1614]|DHJPAR0054534|Antaeotricha ribbei|658[0n]|BOLD:ABU8129
Lytopylus andreamezae|[1615]|DHJPAR0049943|Antaeotricha Janzen09|658[0n]|BOLD:ABU8129
Lytopylus andreamezae|[1616]|DHJPAR0054517|Antaeotricha Janzen09|658[0n]|BOLD:ABU8129
Lytopylus andreamezae|[1617]|DHJPAR0054539|Antaeotricha Janzen07|658[0n]|BOLD:ABU8129
Lytopylus andreamezae|[1618]|DHJPAR0054538|Antaeotricha Janzen07|658[0n]|BOLD:ABU8129
Lytopylus andreamezae|[1619]|DHJPAR0053621|Antaeotricha Janzen09|664[0n]|BOLD:ABU8129
Lytopylus andreamezae|[1620]|DHJPAR0045788|Antaeotricha Janzen07|658[0n]|BOLD:ABU8129
Lytopylus andreamezae|[1621]|DHJPAR0054511|Antaeotricha Janzen09|658[0n]|BOLD:ABU8129
Lytopylus andreamezae|[1622]|DHJPAR0051371|Antaeotricha Janzen09|658[0n]|BOLD:ABU8129
Lytopylus andreamezae|[1623]|DHJPAR0053619|Antaeotricha Janzen09|664[0n]|BOLD:ABU8129
Lytopylus andreamezae|[1624]|DHJPAR0051374|Antaeotricha Janzen09|658[0n]|BOLD:ABU8129
Lytopylus andreamezae|[1625]|DHJPAR0053655|Antaeotricha Janzen09|658[0n]|BOLD:ABU8129
Lytopylus andreamezae|[1626]|DHJPAR0046744|Antaeotricha Janzen09|658[0n]|BOLD:ABU8129
Lytopylus andreamezae|[1627]|DHJPAR0054532|Antaeotricha Janzen07|658[0n]|BOLD:ABU8129
Lytopylus andreamezae|[1628]|DHJPAR0053654|Antaeotricha Janzen09|658[0n]|BOLD:ABU8129
Lytopylus hokwoni|[1629]|DHJPAR0029302|Cerconota recurvella|261[1n]
Lytopylus hokwoni|[1630]|DHJPAR0015414|Cerconota recurvella|654[0n]|BOLD:ACB1292
Lytopylus hokwoni|[1631]|DHJPAR0037940|elachJanzen01 Janzen7940|658[0n]|BOLD:ACB1292
Lytopylus hokwoni|[1632]|DHJPAR0030605|Stenoma Janzen08|654[0n]|BOLD:ACB1292
Lytopylus hokwoni|[1633]|DHJPAR0028279|Antaeotricha Phillips01|627[0n]|BOLD:ACB1292
Lytopylus hokwoni|[1634]|DHJPAR0049051|elachJanzen01 Janzen211|634[0n]|BOLD:ACB1292
Lytopylus hokwoni|[1635]|DHJPAR0048714|Antaeotricha Janzen127|658[0n]|BOLD:ACB1292
Lytopylus hokwoni|[1636]|DHJPAR0017274|Cerconota recurvella|654[0n]|BOLD:ACB1292
Lytopylus Sharkey06DHJ01|[1637]|DHJPAR0048726|Stenoma Janzen08|658[0n]|BOLD:ACY8111
Lytopylus sergiobermudezi|[1638]|BIOUG55346-H08|Malaise trap PL12-9D|654[0n]|BOLD:ADA9104
Lytopylus sergiobermudezi|[1639]|BIOUG52724-H10|Malaise trap PL12-9C|653[0n]|BOLD:ADA9104
Lytopylus sergiobermudezi|[1640]|BIOUG55212-D08|Malaise trap PL12-5C|656[0n]|BOLD:ADA9104
Lytopylus sergiobermudezi|[1641]|DHJPAR0030601|Dichomeris santarosensis|658[0n]|BOLD:ADA9104
Lytopylus sergiobermudezi|[1642]|BIOUG51910-E08|Malaise trap PL12-7B|652[0n]|BOLD:ADA9104
Lytopylus sergiobermudezi|[1643]|BIOUG51763-F08|Malaise trap PL12-3C|654[0n]|BOLD:ADA9104
Lytopylus sergiobermudezi|[1644]|BIOUG55212-D07|Malaise trap PL12-5C|654[0n]|BOLD:ADA9104
Lytopylus sergiobermudezi|[1645]|BIOUG55212-D10|Malaise trap PL12-5C|654[0n]|BOLD:ADA9104
Lytopylus sergiobermudezi|[1646]|BIOUG54474-F10|Malaise trap PL12-5C|640[0n]|BOLD:ADA9104
Lytopylus sergiobermudezi|[1647]|BIOUG53107-F03|Malaise trap PL12-6C|652[0n]|BOLD:ADA9104
Lytopylus sergiobermudezi|[1648]|BIOUG53340-E05|Malaise trap PL12-4C|652[0n]|BOLD:ADA9104
Lytopylus sergiobermudezi|[1649]|BIOUG53962-A09|Malaise trap PL12-9C|651[0n]|BOLD:ADA9104
Lytopylus sergiobermudezi|[1650]|BIOUG53378-D04|Malaise trap PL12-7C|652[0n]|BOLD:ADA9104
Lytopylus sergiobermudezi|[1651]|BIOUG54168-F12|Malaise trap PL12-5C|653[0n]|BOLD:ADA9104
Lytopylus sergiobermudezi|[1652]|BIOUG53616-G05|Malaise trap PL12-3C|654[0n]|BOLD:ADA9104
Lytopylus sergiobermudezi|[1653]|BIOUG53929-B04|Malaise trap PL12-9C|651[0n]|BOLD:ADA9104
Lytopylus sergiobermudezi|[1654]|BIOUG54846-D05|Malaise trap PL12-5C|652[0n]|BOLD:ADA9104
Lytopylus sergiobermudezi|[1655]|BIOUG52716-H09|Malaise trap PL12-9C|654[0n]|BOLD:ADA9104
Lytopylus sergiobermudezi|[1656]|BIOUG53968-A11|Malaise trap PL12-9C|654[0n]|BOLD:ADA9104
Lytopylus sergiobermudezi|[1657]|BIOUG54430-H07|Malaise trap PL12-6D|653[0n]|BOLD:ADA9104
Lytopylus sergiobermudezi|[1658]|BIOUG52824-D07|Malaise trap PL12-3C|653[0n]|BOLD:ADA9104
Lytopylus sergiobermudezi|[1659]|BIOUG52711-H05|Malaise trap PL12-9C|653[0n]|BOLD:ADA9104
Lytopylus sergiobermudezi|[1660]|BIOUG55212-C11|Malaise trap PL12-5C|654[0n]|BOLD:ADA9104
Lytopylus sergiobermudezi|[1661]|BIOUG53118-D12|Malaise trap PL12-9C|653[0n]|BOLD:ADA9104
Lytopylus sergiobermudezi|[1662]|BIOUG55212-D05|Malaise trap PL12-5C|656[0n]|BOLD:ADA9104
Lytopylus sergiobermudezi|[1663]|BIOUG55246-G06|Malaise trap PL12-8C|654[0n]|BOLD:ADA9104
Lytopylus sergiobermudezi|[1664]|BIOUG52720-A12|Malaise trap PL12-9C|657[0n]|BOLD:ADA9104
Lytopylus sergiobermudezi|[1665]|BIOUG55329-D06|Malaise trap PL12-6D|654[0n]|BOLD:ADA9104
Lytopylus sergiobermudezi|[1666]|BIOUG52684-H06|Malaise trap PL12-6C|654[0n]|BOLD:ADA9104
Lytopylus sergiobermudezi|[1667]|BIOUG53929-C10|Malaise trap PL12-9C|640[0n]|BOLD:ADA9104
Lytopylus sergiobermudezi|[1668]|BIOUG53155-C07|Malaise trap PL12-9C|652[0n]|BOLD:ADA9104
Lytopylus sergiobermudezi|[1669]|BIOUG28680-C05|Malaise trap PL12-9A|591[0n]|BOLD:ADA9104
Lytopylus sergiobermudezi|[1670]|BIOUG56081-B06|Malaise trap PL12-9D|640[0n]|BOLD:ADA9104
Lytopylus sergiobermudezi|[1671]|BIOUG28804-B07|Malaise trap PL12-6A|588[0n]|BOLD:ADA9104
Lytopylus sergiobermudezi|[1672]|BIOUG28680-A06|Malaise trap PL12-9A|585[0n]|BOLD:ADA9104
Lytopylus sergiobermudezi|[1673]|BIOUG29390-C03|Malaise trap PL12-9A|594[0n]|BOLD:ADA9104
Lytopylus sergiobermudezi|[1674]|BIOUG28680-A05|Malaise trap PL12-9A|594[0n]|BOLD:ADA9104
Lytopylus sergiobermudezi|[1675]|BIOUG29142-G01|Malaise trap PL12-1A|588[0n]|BOLD:ADA9104
Lytopylus sergiobermudezi|[1676]|DHJPAR0034286|Dichomeris santarosensis|653[0n]|BOLD:ADA9104
Lytopylus sergiobermudezi|[1677]|BIOUG30927-E08|Malaise trap PL12-2A|561[0n]|BOLD:ADA9104
Lytopylus sergiobermudezi|[1678]|BIOUG29142-G02|Malaise trap PL12-1A|564[0n]|BOLD:ADA9104
Lytopylus sergiobermudezi|[1679]|BIOUG55356-A12|Malaise trap PL12-9D|655[0n]|BOLD:ADA9104
Lytopylus sergiobermudezi|[1680]|BIOUG54116-H07|Malaise trap PL12-2C|653[0n]|BOLD:ADA9104
Lytopylus sergiobermudezi|[1681]|BIOUG28809-E07|Malaise trap PL12-6A|594[0n]|BOLD:ADA9104
Lytopylus sergiobermudezi|[1682]|BIOUG28599-C11|Malaise trap PL12-1X|594[0n]|BOLD:ADA9104
Lytopylus sergiobermudezi|[1683]|BIOUG53614-A06|Malaise trap PL12-3C|653[0n]|BOLD:ADA9104
Lytopylus sergiobermudezi|[1684]|BIOUG56235-E11|Malaise trap PL12-6D|655[0n]|BOLD:ADA9104
Lytopylus sergiobermudezi|[1685]|BIOUG56081-E09|Malaise trap PL12-9D|650[0n]|BOLD:ADA9104
Lytopylus sergiobermudezi|[1686]|BIOUG52720-B05|Malaise trap PL12-9C|652[0n]|BOLD:ADA9104
Lytopylus sergiobermudezi|[1687]|BIOUG46359-E03|Malaise trap PL12-6B|654[0n]|BOLD:ADA9104
Lytopylus sergiobermudezi|[1688]|BIOUG55056-A01|Malaise trap PL12-8C|652[0n]|BOLD:ADA9104
Lytopylus johnvalerioi|[1689]|DHJPAR0049935|Cerconota Janzen82|658[2n]|BOLD:ACC1326
Lytopylus johnvalerioi|[1690]|DHJPAR0040066|Cerconota Janzen216|658[0n]|BOLD:ACC1326
Lytopylus johnvalerioi|[1691]|DHJPAR0035498|Cerconota Janzen216|658[0n]|BOLD:ACC1326
Lytopylus johnvalerioi|[1692]|DHJPAR0055354|Cerconota Janzen82|658[0n]|BOLD:ACC1326
Lytopylus johnvalerioi|[1693]|DHJPAR0056634|Cerconota Janzen82|658[2n]|BOLD:ACC1326
