# Supplementary material for: Minimalist revision and description of 403 new species in 11 subfamilies of Costa Rican braconid parasitoid wasps, including host records for 219 species
Source: Zookeys. 2021 Feb 2;1013:1–665. doi: 10.3897/zookeys.1013.55600 (PMC8390796; doi:10.3897/zookeys.1013.55600)
Supplement: Supplementary material 2 — Braconinae [file zookeys-1013-001-s002.pdf]

# 2. Braconinae BOLD TaxonID Tree

Title : Tree Result - Search: Sample IDs (203 records returned) (203 records selected)

Date : 17-Nov-2020

Data Type : Nucleotide

Distance Model : Kimura 2 Parameter

Marker : COI-5P

Colourization : [blue]=Stop Codons [red]=Contamination or misidentification

Label : Sample ID

Label : Taxon

Label : Extra Info

Label : Sequence Length

Label : Barcode Cluster (BIN)

Filter : exclude records with stop codons

Sequence Count : 201

Species count : 77

Genus count : 7

Family count : 1

Unidentified : 6

BIN Count : 77

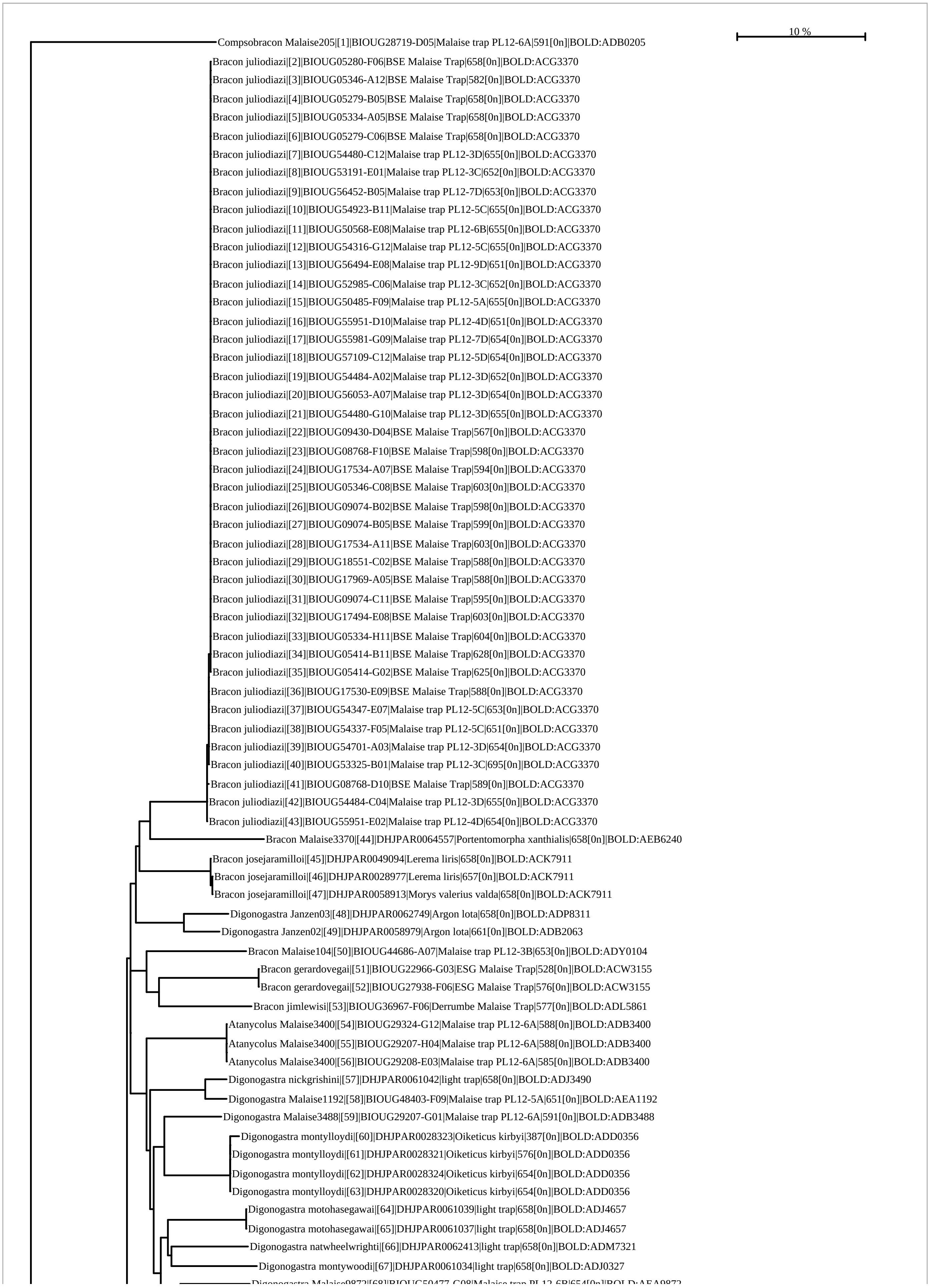
10 %
Compsobracon Malaise205|[1]|BIOUG28719-D05|Malaise trap PL12-6A|591[0n]|BOLD:ADB0205
Bracon juliodiazi|[2]|BIOUG05280-F06|BSE Malaise Trap|658[0n]|BOLD:ACG3370
Bracon juliodiazi|[3]|BIOUG05346-A12|BSE Malaise Trap|582[0n]|BOLD:ACG3370
Bracon juliodiazi|[4]|BIOUG05279-B05|BSE Malaise Trap|658[0n]|BOLD:ACG3370
Bracon juliodiazi|[5]|BIOUG05334-A05|BSE Malaise Trap|658[0n]|BOLD:ACG3370
Bracon juliodiazi|[6]|BIOUG05279-C06|BSE Malaise Trap|658[0n]|BOLD:ACG3370
Bracon juliodiazi|[7]|BIOUG54480-C12|Malaise trap PL12-3D|655[0n]|BOLD:ACG3370
Bracon juliodiazi|[8]|BIOUG53191-E01|Malaise trap PL12-3C|652[0n]|BOLD:ACG3370
Bracon juliodiazi|[9]|BIOUG56452-B05|Malaise trap PL12-7D|653[0n]|BOLD:ACG3370
Bracon juliodiazi|[10]|BIOUG54923-B11|Malaise trap PL12-5C|655[0n]|BOLD:ACG3370
Bracon juliodiazi|[11]|BIOUG50568-E08|Malaise trap PL12-6B|655[0n]|BOLD:ACG3370
Bracon juliodiazi|[12]|BIOUG54316-G12|Malaise trap PL12-5C|655[0n]|BOLD:ACG3370
Bracon juliodiazi|[13]|BIOUG56494-E08|Malaise trap PL12-9D|651[0n]|BOLD:ACG3370
Bracon juliodiazi|[14]|BIOUG52985-C06|Malaise trap PL12-3C|652[0n]|BOLD:ACG3370
Bracon juliodiazi|[15]|BIOUG50485-F09|Malaise trap PL12-5A|655[0n]|BOLD:ACG3370
Bracon juliodiazi|[16]|BIOUG55951-D10|Malaise trap PL12-4D|651[0n]|BOLD:ACG3370
Bracon juliodiazi|[17]|BIOUG55981-G09|Malaise trap PL12-7D|654[0n]|BOLD:ACG3370
Bracon juliodiazi|[18]|BIOUG57109-C12|Malaise trap PL12-5D|654[0n]|BOLD:ACG3370
Bracon juliodiazi|[19]|BIOUG54484-A02|Malaise trap PL12-3D|652[0n]|BOLD:ACG3370
Bracon juliodiazi|[20]|BIOUG56053-A07|Malaise trap PL12-3D|654[0n]|BOLD:ACG3370
Bracon juliodiazi|[21]|BIOUG54480-G10|Malaise trap PL12-3D|655[0n]|BOLD:ACG3370
Bracon juliodiazi|[22]|BIOUG09430-D04|BSE Malaise Trap|567[0n]|BOLD:ACG3370
Bracon juliodiazi|[23]|BIOUG08768-F10|BSE Malaise Trap|598[0n]|BOLD:ACG3370
Bracon juliodiazi|[24]|BIOUG17534-A07|BSE Malaise Trap|594[0n]|BOLD:ACG3370
Bracon juliodiazi|[25]|BIOUG05346-C08|BSE Malaise Trap|603[0n]|BOLD:ACG3370
Bracon juliodiazi|[26]|BIOUG09074-B02|BSE Malaise Trap|598[0n]|BOLD:ACG3370
Bracon juliodiazi|[27]|BIOUG09074-B05|BSE Malaise Trap|599[0n]|BOLD:ACG3370
Bracon juliodiazi|[28]|BIOUG17534-A11|BSE Malaise Trap|603[0n]|BOLD:ACG3370
Bracon juliodiazi|[29]|BIOUG18551-C02|BSE Malaise Trap|588[0n]|BOLD:ACG3370
Bracon juliodiazi|[30]|BIOUG17969-A05|BSE Malaise Trap|588[0n]|BOLD:ACG3370
Bracon juliodiazi|[31]|BIOUG09074-C11|BSE Malaise Trap|595[0n]|BOLD:ACG3370
Bracon juliodiazi|[32]|BIOUG17494-E08|BSE Malaise Trap|603[0n]|BOLD:ACG3370
Bracon juliodiazi|[33]|BIOUG05334-H11|BSE Malaise Trap|604[0n]|BOLD:ACG3370
Bracon juliodiazi|[34]|BIOUG05414-B11|BSE Malaise Trap|628[0n]|BOLD:ACG3370
Bracon juliodiazi|[35]|BIOUG05414-G02|BSE Malaise Trap|625[0n]|BOLD:ACG3370
Bracon juliodiazi|[36]|BIOUG17530-E09|BSE Malaise Trap|588[0n]|BOLD:ACG3370
Bracon juliodiazi|[37]|BIOUG54347-E07|Malaise trap PL12-5C|653[0n]|BOLD:ACG3370
Bracon juliodiazi|[38]|BIOUG54337-F05|Malaise trap PL12-5C|651[0n]|BOLD:ACG3370
Bracon juliodiazi|[39]|BIOUG54701-A03|Malaise trap PL12-3D|654[0n]|BOLD:ACG3370
Bracon juliodiazi|[40]|BIOUG53325-B01|Malaise trap PL12-3C|695[0n]|BOLD:ACG3370
Bracon juliodiazi|[41]|BIOUG08768-D10|BSE Malaise Trap|589[0n]|BOLD:ACG3370
Bracon juliodiazi|[42]|BIOUG54484-C04|Malaise trap PL12-3D|655[0n]|BOLD:ACG3370
Bracon juliodiazi|[43]|BIOUG55951-E02|Malaise trap PL12-4D|654[0n]|BOLD:ACG3370
Bracon Malaise3370|[44]|DHJPAR0064557|Portentomorpha xanthialis|658[0n]|BOLD:AEB6240
Bracon josejaramilloi|[45]|DHJPAR0049094|Lerema liris|658[0n]|BOLD:ACK7911
Bracon josejaramilloi|[46]|DHJPAR0028977|Lerema liris|657[0n]|BOLD:ACK7911
Bracon josejaramilloi|[47]|DHJPAR0058913|Morys valerius valda|658[0n]|BOLD:ACK7911
Digonogastra Janzen03|[48]|DHJPAR0062749|Argon lota|658[0n]|BOLD:ADP8311
Digonogastra Janzen02|[49]|DHJPAR0058979|Argon lota|661[0n]|BOLD:ADB2063
Bracon Malaise104|[50]|BIOUG44686-A07|Malaise trap PL12-3B|653[0n]|BOLD:ADY0104
Bracon gerardovegai|[51]|BIOUG22966-G03|ESG Malaise Trap|528[0n]|BOLD:ACW3155
Bracon gerardovegai|[52]|BIOUG27938-F06|ESG Malaise Trap|576[0n]|BOLD:ACW3155
Bracon jimlewisi|[53]|BIOUG36967-F06|Derrumbe Malaise Trap|577[0n]|BOLD:ADL5861
Atanycolus Malaise3400|[54]|BIOUG29324-G12|Malaise trap PL12-6A|588[0n]|BOLD:ADB3400
Atanycolus Malaise3400|[55]|BIOUG29207-H04|Malaise trap PL12-6A|588[0n]|BOLD:ADB3400
Atanycolus Malaise3400|[56]|BIOUG29208-E03|Malaise trap PL12-6A|585[0n]|BOLD:ADB3400
Digonogastra nickgrishini|[57]|DHJPAR0061042|light trap|658[0n]|BOLD:ADJ3490
Digonogastra Malaise1192|[58]|BIOUG48403-F09|Malaise trap PL12-5A|651[0n]|BOLD:AEA1192
Digonogastra Malaise3488|[59]|BIOUG29207-G01|Malaise trap PL12-6A|591[0n]|BOLD:ADB3488
Digonogastra montylloydi|[60]|DHJPAR0028323|Oiketicus kirbyi|387[0n]|BOLD:ADD0356
Digonogastra montylloydi|[61]|DHJPAR0028321|Oiketicus kirbyi|576[0n]|BOLD:ADD0356
Digonogastra montylloydi|[62]|DHJPAR0028324|Oiketicus kirbyi|654[0n]|BOLD:ADD0356
Digonogastra montylloydi|[63]|DHJPAR0028320|Oiketicus kirbyi|654[0n]|BOLD:ADD0356
Digonogastra motohasegawai|[64]|DHJPAR0061039|light trap|658[0n]|BOLD:ADJ4657
Digonogastra motohasegawai|[65]|DHJPAR0061037|light trap|658[0n]|BOLD:ADJ4657
Digonogastra natwheelwrighti|[66]|DHJPAR0062413|light trap|658[0n]|BOLD:ADM7321
Digonogastra montywoodi|[67]|DHJPAR0061034|light trap|658[0n]|BOLD:ADJ0327

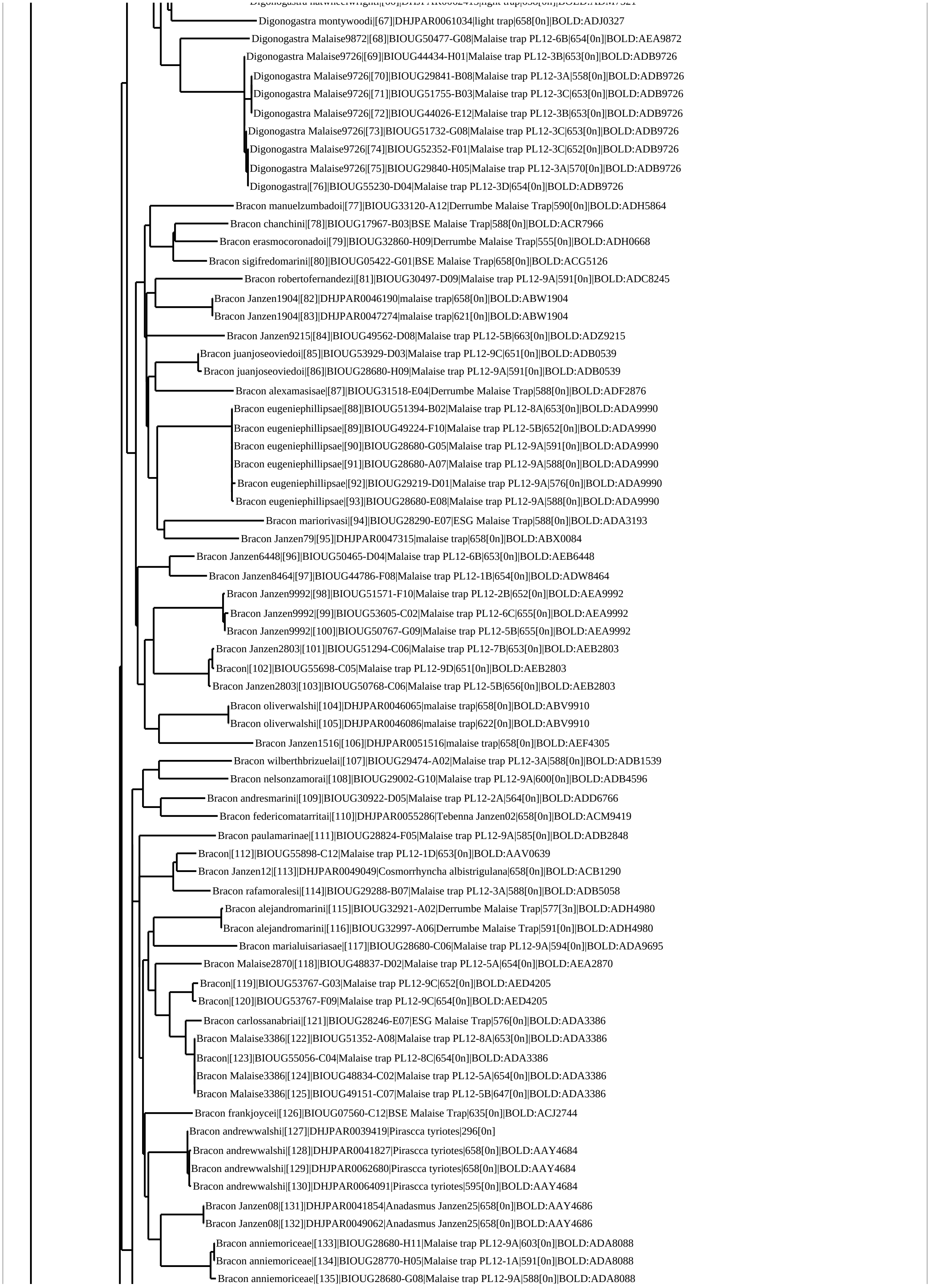
Digonogastra montywoodi|[67]|DHJPAR0061034|light trap|658[0n]|BOLD:ADJ0327
Digonogastra Malaise9872|[68]|BIOUG50477-G08|Malaise trap PL12-6B|654[0n]|BOLD:AEA9872
Digonogastra Malaise9726|[69]|BIOUG44434-H01|Malaise trap PL12-3B|653[0n]|BOLD:ADB9726
Digonogastra Malaise9726|[70]|BIOUG29841-B08|Malaise trap PL12-3A|558[0n]|BOLD:ADB9726
Digonogastra Malaise9726|[71]|BIOUG51755-B03|Malaise trap PL12-3C|653[0n]|BOLD:ADB9726
Digonogastra Malaise9726|[72]|BIOUG44026-E12|Malaise trap PL12-3B|653[0n]|BOLD:ADB9726
Digonogastra Malaise9726|[73]|BIOUG51732-G08|Malaise trap PL12-3C|653[0n]|BOLD:ADB9726
Digonogastra Malaise9726|[74]|BIOUG52352-F01|Malaise trap PL12-3C|652[0n]|BOLD:ADB9726
Digonogastra Malaise9726|[75]|BIOUG29840-H05|Malaise trap PL12-3A|570[0n]|BOLD:ADB9726
Digonogastra|[76]|BIOUG55230-D04|Malaise trap PL12-3D|654[0n]|BOLD:ADB9726
Bracon manuelzumbadoi|[77]|BIOUG33120-A12|Derrumbe Malaise Trap|590[0n]|BOLD:ADH5864
Bracon chanchini|[78]|BIOUG17967-B03|BSE Malaise Trap|588[0n]|BOLD:ACR7966
Bracon erasmocoronadoi|[79]|BIOUG32860-H09|Derrumbe Malaise Trap|555[0n]|BOLD:ADH0668
Bracon sigifredomarini|[80]|BIOUG05422-G01|BSE Malaise Trap|658[0n]|BOLD:ACG5126
Bracon robertofernandezi|[81]|BIOUG30497-D09|Malaise trap PL12-9A|591[0n]|BOLD:ADC8245
Bracon Janzen1904|[82]|DHJPAR0046190|malaise trap|658[0n]|BOLD:ABW1904
Bracon Janzen1904|[83]|DHJPAR0047274|malaise trap|621[0n]|BOLD:ABW1904
Bracon Janzen9215|[84]|BIOUG49562-D08|Malaise trap PL12-5B|663[0n]|BOLD:ADZ9215
Bracon juanjoseoviedoi|[85]|BIOUG53929-D03|Malaise trap PL12-9C|651[0n]|BOLD:ADB0539
Bracon juanjoseoviedoi|[86]|BIOUG28680-H09|Malaise trap PL12-9A|591[0n]|BOLD:ADB0539
Bracon alexamasisae|[87]|BIOUG31518-E04|Derrumbe Malaise Trap|588[0n]|BOLD:ADF2876
Bracon eugeniephillipsae|[88]|BIOUG51394-B02|Malaise trap PL12-8A|653[0n]|BOLD:ADA9990
Bracon eugeniephillipsae|[89]|BIOUG49224-F10|Malaise trap PL12-5B|652[0n]|BOLD:ADA9990
Bracon eugeniephillipsae|[90]|BIOUG28680-G05|Malaise trap PL12-9A|591[0n]|BOLD:ADA9990
Bracon eugeniephillipsae|[91]|BIOUG28680-A07|Malaise trap PL12-9A|588[0n]|BOLD:ADA9990
Bracon eugeniephillipsae|[92]|BIOUG29219-D01|Malaise trap PL12-9A|576[0n]|BOLD:ADA9990
Bracon eugeniephillipsae|[93]|BIOUG28680-E08|Malaise trap PL12-9A|588[0n]|BOLD:ADA9990
Bracon mariorivasi|[94]|BIOUG28290-E07|ESG Malaise Trap|588[0n]|BOLD:ADA3193
Bracon Janzen79|[95]|DHJPAR0047315|malaise trap|658[0n]|BOLD:ABX0084
Bracon Janzen6448|[96]|BIOUG50465-D04|Malaise trap PL12-6B|653[0n]|BOLD:AEB6448
Bracon Janzen8464|[97]|BIOUG44786-F08|Malaise trap PL12-1B|654[0n]|BOLD:ADW8464
Bracon Janzen9992|[98]|BIOUG51571-F10|Malaise trap PL12-2B|652[0n]|BOLD:AEA9992
Bracon Janzen9992|[99]|BIOUG53605-C02|Malaise trap PL12-6C|655[0n]|BOLD:AEA9992
Bracon Janzen9992|[100]|BIOUG50767-G09|Malaise trap PL12-5B|655[0n]|BOLD:AEA9992
Bracon Janzen2803|[101]|BIOUG51294-C06|Malaise trap PL12-7B|653[0n]|BOLD:AEB2803
Bracon|[102]|BIOUG55698-C05|Malaise trap PL12-9D|651[0n]|BOLD:AEB2803
Bracon Janzen2803|[103]|BIOUG50768-C06|Malaise trap PL12-5B|656[0n]|BOLD:AEB2803
Bracon oliverwalshi|[104]|DHJPAR0046065|malaise trap|658[0n]|BOLD:ABV9910
Bracon oliverwalshi|[105]|DHJPAR0046086|malaise trap|622[0n]|BOLD:ABV9910
Bracon Janzen1516|[106]|DHJPAR0051516|malaise trap|658[0n]|BOLD:AEF4305
Bracon wilberthbrizuelai|[107]|BIOUG29474-A02|Malaise trap PL12-3A|588[0n]|BOLD:ADB1539
Bracon nelsonzamorai|[108]|BIOUG29002-G10|Malaise trap PL12-9A|600[0n]|BOLD:ADB4596
Bracon andresmarini|[109]|BIOUG30922-D05|Malaise trap PL12-2A|564[0n]|BOLD:ADD6766
Bracon federicomatarritai|[110]|DHJPAR0055286|Tebenna Janzen02|658[0n]|BOLD:ACM9419
Bracon paulamarinae|[111]|BIOUG28824-F05|Malaise trap PL12-9A|585[0n]|BOLD:ADB2848
Bracon|[112]|BIOUG55898-C12|Malaise trap PL12-1D|653[0n]|BOLD:AAV0639
Bracon Janzen12|[113]|DHJPAR0049049|Cosmorrhyncha albistrigulana|658[0n]|BOLD:ACB1290
Bracon rafamoralesi|[114]|BIOUG29288-B07|Malaise trap PL12-3A|588[0n]|BOLD:ADB5058
Bracon alejandromarini|[115]|BIOUG32921-A02|Derrumbe Malaise Trap|577[3n]|BOLD:ADH4980
Bracon alejandromarini|[116]|BIOUG32997-A06|Derrumbe Malaise Trap|591[0n]|BOLD:ADH4980
Bracon marialuisariasae|[117]|BIOUG28680-C06|Malaise trap PL12-9A|594[0n]|BOLD:ADA9695
Bracon Malaise2870|[118]|BIOUG48837-D02|Malaise trap PL12-5A|654[0n]|BOLD:AEA2870
Bracon|[119]|BIOUG53767-G03|Malaise trap PL12-9C|652[0n]|BOLD:AED4205
Bracon|[120]|BIOUG53767-F09|Malaise trap PL12-9C|654[0n]|BOLD:AED4205
Bracon carlossanabriai|[121]|BIOUG28246-E07|ESG Malaise Trap|576[0n]|BOLD:ADA3386
Bracon Malaise3386|[122]|BIOUG51352-A08|Malaise trap PL12-8A|653[0n]|BOLD:ADA3386
Bracon|[123]|BIOUG55056-C04|Malaise trap PL12-8C|654[0n]|BOLD:ADA3386
Bracon Malaise3386|[124]|BIOUG48834-C02|Malaise trap PL12-5A|654[0n]|BOLD:ADA3386
Bracon Malaise3386|[125]|BIOUG49151-C07|Malaise trap PL12-5B|647[0n]|BOLD:ADA3386
Bracon frankjoycei|[126]|BIOUG07560-C12|BSE Malaise Trap|635[0n]|BOLD:ACJ2744
Bracon andrewwalshi|[127]|DHJPAR0039419|Pirascca tyriotes|296[0n]
Bracon andrewwalshi|[128]|DHJPAR0041827|Pirascca tyriotes|658[0n]|BOLD:AAY4684
Bracon andrewwalshi|[129]|DHJPAR0062680|Pirascca tyriotes|658[0n]|BOLD:AAY4684
Bracon andrewwalshi|[130]|DHJPAR0064091|Pirascca tyriotes|595[0n]|BOLD:AAY4684
Bracon Janzen08|[131]|DHJPAR0041854|Anadasmus Janzen25|658[0n]|BOLD:AAY4686
Bracon Janzen08|[132]|DHJPAR0049062|Anadasmus Janzen25|658[0n]|BOLD:AAY4686
Bracon anniemoriceae|[133]|BIOUG28680-H11|Malaise trap PL12-9A|603[0n]|BOLD:ADA8088
Bracon anniemoriceae|[134]|BIOUG28770-H05|Malaise trap PL12-1A|591[0n]|BOLD:ADA8088
Bracon anniemoriceae|[135]|BIOUG28680-G08|Malaise trap PL12-9A|588[0n]|BOLD:ADA8088

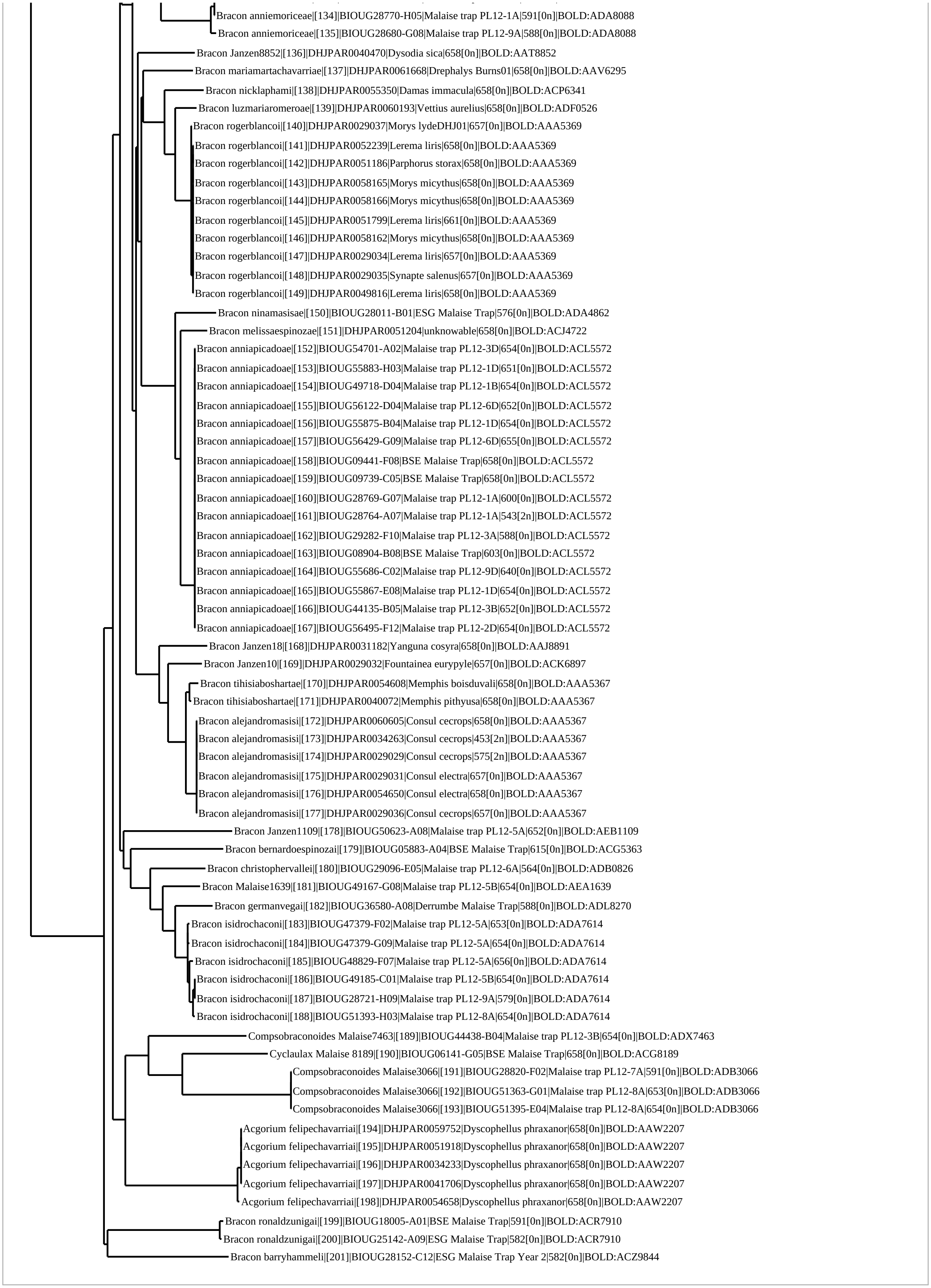
Bracon anniemoriceae|[134]|BIOUG28770-H05|Malaise trap PL12-1A|591[0n]|BOLD:ADA8088
Bracon anniemoriceae|[135]|BIOUG28680-G08|Malaise trap PL12-9A|588[0n]|BOLD:ADA8088
Bracon Janzen8852|[136]|DHJPAR0040470|Dysodia sica|658[0n]|BOLD:AAT8852
Bracon mariamartachavarriae|[137]|DHJPAR0061668|Drephalys Burns01|658[0n]|BOLD:AAV6295
Bracon nicklaphami|[138]|DHJPAR0055350|Damas immacula|658[0n]|BOLD:ACP6341
Bracon luzmariaromeroae|[139]|DHJPAR0060193|Vettius aurelius|658[0n]|BOLD:ADF0526
Bracon rogerblancoi|[140]|DHJPAR0029037|Morys lydeDHJ01|657[0n]|BOLD:AAA5369
Bracon rogerblancoi|[141]|DHJPAR0052239|Lerema liris|658[0n]|BOLD:AAA5369
Bracon rogerblancoi|[142]|DHJPAR0051186|Parphorus storax|658[0n]|BOLD:AAA5369
Bracon rogerblancoi|[143]|DHJPAR0058165|Morys micythus|658[0n]|BOLD:AAA5369
Bracon rogerblancoi|[144]|DHJPAR0058166|Morys micythus|658[0n]|BOLD:AAA5369
Bracon rogerblancoi|[145]|DHJPAR0051799|Lerema liris|661[0n]|BOLD:AAA5369
Bracon rogerblancoi|[146]|DHJPAR0058162|Morys micythus|658[0n]|BOLD:AAA5369
Bracon rogerblancoi|[147]|DHJPAR0029034|Lerema liris|657[0n]|BOLD:AAA5369
Bracon rogerblancoi|[148]|DHJPAR0029035|Synapte salenus|657[0n]|BOLD:AAA5369
Bracon rogerblancoi|[149]|DHJPAR0049816|Lerema liris|658[0n]|BOLD:AAA5369
Bracon ninamasisae|[150]|BIOUG28011-B01|ESG Malaise Trap|576[0n]|BOLD:ADA4862
Bracon melissaespinozae|[151]|DHJPAR0051204|unknowable|658[0n]|BOLD:ACJ4722
Bracon anniapicadoae|[152]|BIOUG54701-A02|Malaise trap PL12-3D|654[0n]|BOLD:ACL5572
Bracon anniapicadoae|[153]|BIOUG55883-H03|Malaise trap PL12-1D|651[0n]|BOLD:ACL5572
Bracon anniapicadoae|[154]|BIOUG49718-D04|Malaise trap PL12-1B|654[0n]|BOLD:ACL5572
Bracon anniapicadoae|[155]|BIOUG56122-D04|Malaise trap PL12-6D|652[0n]|BOLD:ACL5572
Bracon anniapicadoae|[156]|BIOUG55875-B04|Malaise trap PL12-1D|654[0n]|BOLD:ACL5572
Bracon anniapicadoae|[157]|BIOUG56429-G09|Malaise trap PL12-6D|655[0n]|BOLD:ACL5572
Bracon anniapicadoae|[158]|BIOUG09441-F08|BSE Malaise Trap|658[0n]|BOLD:ACL5572
Bracon anniapicadoae|[159]|BIOUG09739-C05|BSE Malaise Trap|658[0n]|BOLD:ACL5572
Bracon anniapicadoae|[160]|BIOUG28769-G07|Malaise trap PL12-1A|600[0n]|BOLD:ACL5572
Bracon anniapicadoae|[161]|BIOUG28764-A07|Malaise trap PL12-1A|543[2n]|BOLD:ACL5572
Bracon anniapicadoae|[162]|BIOUG29282-F10|Malaise trap PL12-3A|588[0n]|BOLD:ACL5572
Bracon anniapicadoae|[163]|BIOUG08904-B08|BSE Malaise Trap|603[0n]|BOLD:ACL5572
Bracon anniapicadoae|[164]|BIOUG55686-C02|Malaise trap PL12-9D|640[0n]|BOLD:ACL5572
Bracon anniapicadoae|[165]|BIOUG55867-E08|Malaise trap PL12-1D|654[0n]|BOLD:ACL5572
Bracon anniapicadoae|[166]|BIOUG44135-B05|Malaise trap PL12-3B|652[0n]|BOLD:ACL5572
Bracon anniapicadoae|[167]|BIOUG56495-F12|Malaise trap PL12-2D|654[0n]|BOLD:ACL5572
Bracon Janzen18|[168]|DHJPAR0031182|Yanguna cosyra|658[0n]|BOLD:AAJ8891
Bracon Janzen10|[169]|DHJPAR0029032|Fountainea eurypyle|657[0n]|BOLD:ACK6897
Bracon tihisiaboshartae|[170]|DHJPAR0054608|Memphis boisduvali|658[0n]|BOLD:AAA5367
Bracon tihisiaboshartae|[171]|DHJPAR0040072|Memphis pithyusa|658[0n]|BOLD:AAA5367
Bracon alejandromasisi|[172]|DHJPAR0060605|Consul cecrops|658[0n]|BOLD:AAA5367
Bracon alejandromasisi|[173]|DHJPAR0034263|Consul cecrops|453[2n]|BOLD:AAA5367
Bracon alejandromasisi|[174]|DHJPAR0029029|Consul cecrops|575[2n]|BOLD:AAA5367
Bracon alejandromasisi|[175]|DHJPAR0029031|Consul electra|657[0n]|BOLD:AAA5367
Bracon alejandromasisi|[176]|DHJPAR0054650|Consul electra|658[0n]|BOLD:AAA5367
Bracon alejandromasisi|[177]|DHJPAR0029036|Consul cecrops|657[0n]|BOLD:AAA5367
Bracon Janzen1109|[178]|BIOUG50623-A08|Malaise trap PL12-5A|652[0n]|BOLD:AEB1109
Bracon bernardoespinozai|[179]|BIOUG05883-A04|BSE Malaise Trap|615[0n]|BOLD:ACG5363
Bracon christophervallei|[180]|BIOUG29096-E05|Malaise trap PL12-6A|564[0n]|BOLD:ADB0826
Bracon Malaise1639|[181]|BIOUG49167-G08|Malaise trap PL12-5B|654[0n]|BOLD:AEA1639
Bracon germanvegai|[182]|BIOUG36580-A08|Derrumbe Malaise Trap|588[0n]|BOLD:ADL8270
Bracon isidrochaconi|[183]|BIOUG47379-F02|Malaise trap PL12-5A|653[0n]|BOLD:ADA7614
Bracon isidrochaconi|[184]|BIOUG47379-G09|Malaise trap PL12-5A|654[0n]|BOLD:ADA7614
Bracon isidrochaconi|[185]|BIOUG48829-F07|Malaise trap PL12-5A|656[0n]|BOLD:ADA7614
Bracon isidrochaconi|[186]|BIOUG49185-C01|Malaise trap PL12-5B|654[0n]|BOLD:ADA7614
Bracon isidrochaconi|[187]|BIOUG28721-H09|Malaise trap PL12-9A|579[0n]|BOLD:ADA7614
Bracon isidrochaconi|[188]|BIOUG51393-H03|Malaise trap PL12-8A|654[0n]|BOLD:ADA7614
Compsobraconoides Malaise7463|[189]|BIOUG44438-B04|Malaise trap PL12-3B|654[0n]|BOLD:ADX7463
Cyclaulax Malaise 8189|[190]|BIOUG06141-G05|BSE Malaise Trap|658[0n]|BOLD:ACG8189
Compsobraconoides Malaise3066|[191]|BIOUG28820-F02|Malaise trap PL12-7A|591[0n]|BOLD:ADB3066
Compsobraconoides Malaise3066|[192]|BIOUG51363-G01|Malaise trap PL12-8A|653[0n]|BOLD:ADB3066
Compsobraconoides Malaise3066|[193]|BIOUG51395-E04|Malaise trap PL12-8A|654[0n]|BOLD:ADB3066
Acgorium felipechavarriai|[194]|DHJPAR0059752|Dyscophellus phraxanor|658[0n]|BOLD:AAW2207
Acgorium felipechavarriai|[195]|DHJPAR0051918|Dyscophellus phraxanor|658[0n]|BOLD:AAW2207
Acgorium felipechavarriai|[196]|DHJPAR0034233|Dyscophellus phraxanor|658[0n]|BOLD:AAW2207
Acgorium felipechavarriai|[197]|DHJPAR0041706|Dyscophellus phraxanor|658[0n]|BOLD:AAW2207
Acgorium felipechavarriai|[198]|DHJPAR0054658|Dyscophellus phraxanor|658[0n]|BOLD:AAW2207
Bracon ronaldzunigai|[199]|BIOUG18005-A01|BSE Malaise Trap|591[0n]|BOLD:ACR7910
Bracon ronaldzunigai|[200]|BIOUG25142-A09|ESG Malaise Trap|582[0n]|BOLD:ACR7910
Bracon barryhammeli|[201]|BIOUG28152-C12|ESG Malaise Trap Year 2|582[0n]|BOLD:ACZ9844
