# Supplementary material for: Minimalist revision and description of 403 new species in 11 subfamilies of Costa Rican braconid parasitoid wasps, including host records for 219 species
Source: Zookeys. 2021 Feb 2;1013:1–665. doi: 10.3897/zookeys.1013.55600 (PMC8390796; doi:10.3897/zookeys.1013.55600)
Supplement: Supplementary material 3 — Cheloninae [file zookeys-1013-001-s003.pdf]

3. Cheloninae BOLD TaxonID Tree

Title : Tree Result - Search: Sample IDs (1544 records returned) (1544 records selected)
Date : 17-Nov-2020
Data Type : Nucleotide
Distance Model : Kimura 2 Parameter
Marker : COI-5P
Colourization : [blue]=Stop Codons [red]=Contamination or misidentification

Label : Sample ID
Label : Taxon
Label : Extra Info
Label : Sequence Length
Label : Barcode Cluster (BIN)

Filter : exclude records with stop codons

Sequence Count : 1471
Species count : 150
Genus count : 6
Family count : 1
Unidentified : 41

BIN Count : 146

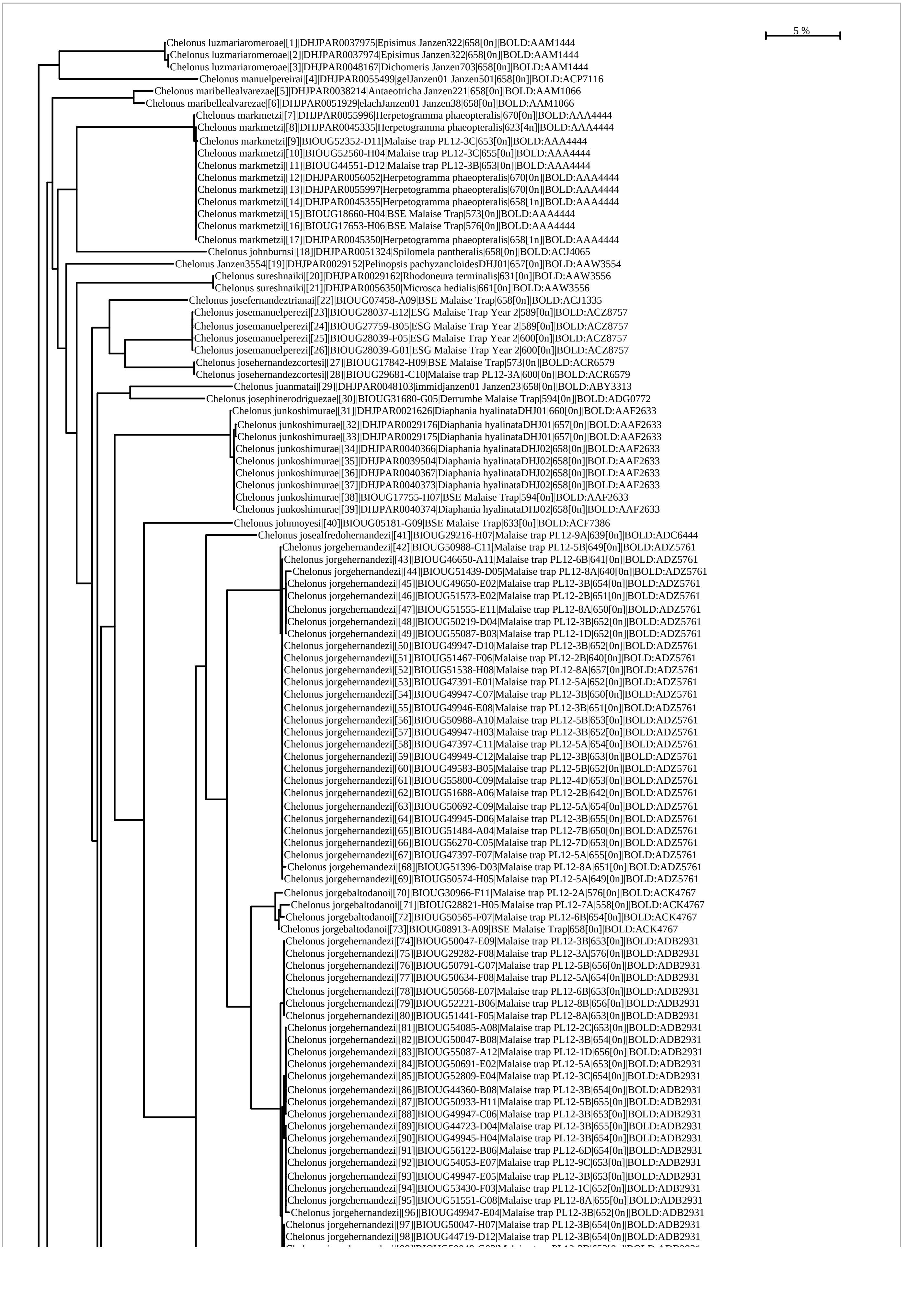
5 %
Chelonus luzmariaromeroae|[1]|DHJPAR0037975|Episimus Janzen322|658[0n]|BOLD:AAM1444
Chelonus luzmariaromeroae|[2]|DHJPAR0037974|Episimus Janzen322|658[0n]|BOLD:AAM1444
Chelonus luzmariaromeroae|[3]|DHJPAR0048167|Dichomeris Janzen703|658[0n]|BOLD:AAM1444
Chelonus manuelpereirai|[4]|DHJPAR0055499|gelJanzen01 Janzen501|658[0n]|BOLD:ACP7116
Chelonus maribellealvarezae|[5]|DHJPAR0038214|Antaeotricha Janzen221|658[0n]|BOLD:AAM1066
Chelonus maribellealvarezae|[6]|DHJPAR0051929|elachJanzen01 Janzen38|658[0n]|BOLD:AAM1066
Chelonus markmetzi|[7]|DHJPAR0055996|Herpetogramma phaeopteralis|670[0n]|BOLD:AAA4444
Chelonus markmetzi|[8]|DHJPAR0045335|Herpetogramma phaeopteralis|623[4n]|BOLD:AAA4444
Chelonus markmetzi|[9]|BIOUG52352-D11|Malaise trap PL12-3C|653[0n]|BOLD:AAA4444
Chelonus markmetzi|[10]|BIOUG52560-H04|Malaise trap PL12-3C|655[0n]|BOLD:AAA4444
Chelonus markmetzi|[11]|BIOUG44551-D12|Malaise trap PL12-3B|653[0n]|BOLD:AAA4444
Chelonus markmetzi|[12]|DHJPAR0056052|Herpetogramma phaeopteralis|670[0n]|BOLD:AAA4444
Chelonus markmetzi|[13]|DHJPAR0055997|Herpetogramma phaeopteralis|670[0n]|BOLD:AAA4444
Chelonus markmetzi|[14]|DHJPAR0045355|Herpetogramma phaeopteralis|658[1n]|BOLD:AAA4444
Chelonus markmetzi|[15]|BIOUG18660-H04|BSE Malaise Trap|573[0n]|BOLD:AAA4444
Chelonus markmetzi|[16]|BIOUG17653-H06|BSE Malaise Trap|576[0n]|BOLD:AAA4444
Chelonus markmetzi|[17]|DHJPAR0045350|Herpetogramma phaeopteralis|658[1n]|BOLD:AAA4444
Chelonus johnburnsi|[18]|DHJPAR0051324|Spilomela pantheralis|658[0n]|BOLD:ACJ4065
Chelonus Janzen3554|[19]|DHJPAR0029152|Pelinopsis pachyzancloidesDHJ01|657[0n]|BOLD:AAW3554
Chelonus sureshnaiki|[20]|DHJPAR0029162|Rhodoneura terminalis|631[0n]|BOLD:AAW3556
Chelonus sureshnaiki|[21]|DHJPAR0056350|Microsca hedialis|661[0n]|BOLD:AAW3556
Chelonus josefernandeztrianai|[22]|BIOUG07458-A09|BSE Malaise Trap|658[0n]|BOLD:ACJ1335
Chelonus josemanuelperezi|[23]|BIOUG28037-E12|ESG Malaise Trap Year 2|589[0n]|BOLD:ACZ8757
Chelonus josemanuelperezi|[24]|BIOUG27759-B05|ESG Malaise Trap Year 2|589[0n]|BOLD:ACZ8757
Chelonus josemanuelperezi|[25]|BIOUG28039-F05|ESG Malaise Trap Year 2|600[0n]|BOLD:ACZ8757
Chelonus josemanuelperezi|[26]|BIOUG28039-G01|ESG Malaise Trap Year 2|600[0n]|BOLD:ACZ8757
Chelonus josehernandezcortesi|[27]|BIOUG17842-H09|BSE Malaise Trap|573[0n]|BOLD:ACR6579
Chelonus josehernandezcortesi|[28]|BIOUG29681-C10|Malaise trap PL12-3A|600[0n]|BOLD:ACR6579
Chelonus juanmatai|[29]|DHJPAR0048103|immidjanzen01 Janzen23|658[0n]|BOLD:ABY3313
Chelonus josephinerodriguezae|[30]|BIOUG31680-G05|Derrumbe Malaise Trap|594[0n]|BOLD:ADG0772
Chelonus junkoshimurae|[31]|DHJPAR0021626|Diaphania hyalinataDHJ01|660[0n]|BOLD:AAF2633
Chelonus junkoshimurae|[32]|DHJPAR0029176|Diaphania hyalinataDHJ01|657[0n]|BOLD:AAF2633
Chelonus junkoshimurae|[33]|DHJPAR0029175|Diaphania hyalinataDHJ01|657[0n]|BOLD:AAF2633
Chelonus junkoshimurae|[34]|DHJPAR0040366|Diaphania hyalinataDHJ02|658[0n]|BOLD:AAF2633
Chelonus junkoshimurae|[35]|DHJPAR0039504|Diaphania hyalinataDHJ02|658[0n]|BOLD:AAF2633
Chelonus junkoshimurae|[36]|DHJPAR0040367|Diaphania hyalinataDHJ02|658[0n]|BOLD:AAF2633
Chelonus junkoshimurae|[37]|DHJPAR0040373|Diaphania hyalinataDHJ02|658[0n]|BOLD:AAF2633
Chelonus junkoshimurae|[38]|BIOUG17755-H07|BSE Malaise Trap|594[0n]|BOLD:AAF2633
Chelonus junkoshimurae|[39]|DHJPAR0040374|Diaphania hyalinataDHJ02|658[0n]|BOLD:AAF2633
Chelonus johnnoyesi|[40]|BIOUG05181-G09|BSE Malaise Trap|633[0n]|BOLD:ACF7386
Chelonus josealfredohernandezi|[41]|BIOUG29216-H07|Malaise trap PL12-9A|639[0n]|BOLD:ADC6444
Chelonus jorgehernandezi|[42]|BIOUG50988-C11|Malaise trap PL12-5B|649[0n]|BOLD:ADZ5761
Chelonus jorgehernandezi|[43]|BIOUG46650-A11|Malaise trap PL12-6B|641[0n]|BOLD:ADZ5761
Chelonus jorgehernandezi|[44]|BIOUG51439-D05|Malaise trap PL12-8A|640[0n]|BOLD:ADZ5761
Chelonus jorgehernandezi|[45]|BIOUG49650-E02|Malaise trap PL12-3B|654[0n]|BOLD:ADZ5761
Chelonus jorgehernandezi|[46]|BIOUG51573-E02|Malaise trap PL12-2B|651[0n]|BOLD:ADZ5761
Chelonus jorgehernandezi|[47]|BIOUG51555-E11|Malaise trap PL12-8A|650[0n]|BOLD:ADZ5761
Chelonus jorgehernandezi|[48]|BIOUG50219-D04|Malaise trap PL12-3B|652[0n]|BOLD:ADZ5761
Chelonus jorgehernandezi|[49]|BIOUG55087-B03|Malaise trap PL12-1D|652[0n]|BOLD:ADZ5761
Chelonus jorgehernandezi|[50]|BIOUG49947-D10|Malaise trap PL12-3B|652[0n]|BOLD:ADZ5761
Chelonus jorgehernandezi|[51]|BIOUG51467-F06|Malaise trap PL12-2B|640[0n]|BOLD:ADZ5761
Chelonus jorgehernandezi|[52]|BIOUG51538-H08|Malaise trap PL12-8A|657[0n]|BOLD:ADZ5761
Chelonus jorgehernandezi|[53]|BIOUG47391-E01|Malaise trap PL12-5A|652[0n]|BOLD:ADZ5761
Chelonus jorgehernandezi|[54]|BIOUG49947-C07|Malaise trap PL12-3B|650[0n]|BOLD:ADZ5761
Chelonus jorgehernandezi|[55]|BIOUG49946-E08|Malaise trap PL12-3B|651[0n]|BOLD:ADZ5761
Chelonus jorgehernandezi|[56]|BIOUG50988-A10|Malaise trap PL12-5B|653[0n]|BOLD:ADZ5761
Chelonus jorgehernandezi|[57]|BIOUG49947-H03|Malaise trap PL12-3B|652[0n]|BOLD:ADZ5761
Chelonus jorgehernandezi|[58]|BIOUG47397-C11|Malaise trap PL12-5A|654[0n]|BOLD:ADZ5761
Chelonus jorgehernandezi|[59]|BIOUG49949-C12|Malaise trap PL12-3B|653[0n]|BOLD:ADZ5761
Chelonus jorgehernandezi|[60]|BIOUG49583-B05|Malaise trap PL12-5B|652[0n]|BOLD:ADZ5761
Chelonus jorgehernandezi|[61]|BIOUG55800-C09|Malaise trap PL12-4D|653[0n]|BOLD:ADZ5761
Chelonus jorgehernandezi|[62]|BIOUG51688-A06|Malaise trap PL12-2B|642[0n]|BOLD:ADZ5761
Chelonus jorgehernandezi|[63]|BIOUG50692-C09|Malaise trap PL12-5A|654[0n]|BOLD:ADZ5761
Chelonus jorgehernandezi|[64]|BIOUG49945-D06|Malaise trap PL12-3B|655[0n]|BOLD:ADZ5761
Chelonus jorgehernandezi|[65]|BIOUG51484-A04|Malaise trap PL12-7B|650[0n]|BOLD:ADZ5761
Chelonus jorgehernandezi|[66]|BIOUG56270-C05|Malaise trap PL12-7D|653[0n]|BOLD:ADZ5761
Chelonus jorgehernandezi|[67]|BIOUG47397-F07|Malaise trap PL12-5A|655[0n]|BOLD:ADZ5761
Chelonus jorgehernandezi|[68]|BIOUG51396-D03|Malaise trap PL12-8A|651[0n]|BOLD:ADZ5761
Chelonus jorgehernandezi|[69]|BIOUG50574-H05|Malaise trap PL12-5A|649[0n]|BOLD:ADZ5761
Chelonus jorgebaltodanoi|[70]|BIOUG30966-F11|Malaise trap PL12-2A|576[0n]|BOLD:ACK4767
Chelonus jorgebaltodanoi|[71]|BIOUG28821-H05|Malaise trap PL12-7A|558[0n]|BOLD:ACK4767
Chelonus jorgebaltodanoi|[72]|BIOUG50565-F07|Malaise trap PL12-6B|654[0n]|BOLD:ACK4767
Chelonus jorgebaltodanoi|[73]|BIOUG08913-A09|BSE Malaise Trap|658[0n]|BOLD:ACK4767
Chelonus jorgehernandezi|[74]|BIOUG50047-E09|Malaise trap PL12-3B|653[0n]|BOLD:ADB2931
Chelonus jorgehernandezi|[75]|BIOUG29282-F08|Malaise trap PL12-3A|576[0n]|BOLD:ADB2931
Chelonus jorgehernandezi|[76]|BIOUG50791-G07|Malaise trap PL12-5B|656[0n]|BOLD:ADB2931
Chelonus jorgehernandezi|[77]|BIOUG50634-F08|Malaise trap PL12-5A|654[0n]|BOLD:ADB2931
Chelonus jorgehernandezi|[78]|BIOUG50568-E07|Malaise trap PL12-6B|653[0n]|BOLD:ADB2931
Chelonus jorgehernandezi|[79]|BIOUG52221-B06|Malaise trap PL12-8B|656[0n]|BOLD:ADB2931
Chelonus jorgehernandezi|[80]|BIOUG51441-F05|Malaise trap PL12-8A|653[0n]|BOLD:ADB2931
Chelonus jorgehernandezi|[81]|BIOUG54085-A08|Malaise trap PL12-2C|653[0n]|BOLD:ADB2931
Chelonus jorgehernandezi|[82]|BIOUG50047-B08|Malaise trap PL12-3B|654[0n]|BOLD:ADB2931
Chelonus jorgehernandezi|[83]|BIOUG55087-A12|Malaise trap PL12-1D|656[0n]|BOLD:ADB2931
Chelonus jorgehernandezi|[84]|BIOUG50691-E02|Malaise trap PL12-5A|653[0n]|BOLD:ADB2931
Chelonus jorgehernandezi|[85]|BIOUG52809-E04|Malaise trap PL12-3C|654[0n]|BOLD:ADB2931
Chelonus jorgehernandezi|[86]|BIOUG44360-B08|Malaise trap PL12-3B|654[0n]|BOLD:ADB2931
Chelonus jorgehernandezi|[87]|BIOUG50933-H11|Malaise trap PL12-5B|655[0n]|BOLD:ADB2931
Chelonus jorgehernandezi|[88]|BIOUG49947-C06|Malaise trap PL12-3B|653[0n]|BOLD:ADB2931
Chelonus jorgehernandezi|[89]|BIOUG44723-D04|Malaise trap PL12-3B|655[0n]|BOLD:ADB2931
Chelonus jorgehernandezi|[90]|BIOUG49945-H04|Malaise trap PL12-3B|654[0n]|BOLD:ADB2931
Chelonus jorgehernandezi|[91]|BIOUG56122-B06|Malaise trap PL12-6D|654[0n]|BOLD:ADB2931
Chelonus jorgehernandezi|[92]|BIOUG54053-E07|Malaise trap PL12-9C|653[0n]|BOLD:ADB2931
Chelonus jorgehernandezi|[93]|BIOUG49947-E05|Malaise trap PL12-3B|653[0n]|BOLD:ADB2931
Chelonus jorgehernandezi|[94]|BIOUG53430-F03|Malaise trap PL12-1C|652[0n]|BOLD:ADB2931
Chelonus jorgehernandezi|[95]|BIOUG51551-G08|Malaise trap PL12-8A|655[0n]|BOLD:ADB2931
Chelonus jorgehernandezi|[96]|BIOUG49947-E04|Malaise trap PL12-3B|652[0n]|BOLD:ADB2931
Chelonus jorgehernandezi|[97]|BIOUG50047-H07|Malaise trap PL12-3B|654[0n]|BOLD:ADB2931
Chelonus jorgehernandezi|[98]|BIOUG44719-D12|Malaise trap PL12-3B|654[0n]|BOLD:ADB2931

Chelonus jorgehernandezi|[96]|BIOUG49947-E04|Malaise trap PL12-3B|652[0n]|BOLD:ADB2931
Chelonus jorgehernandezi|[97]|BIOUG50047-H07|Malaise trap PL12-3B|654[0n]|BOLD:ADB2931
Chelonus jorgehernandezi|[98]|BIOUG44719-D12|Malaise trap PL12-3B|654[0n]|BOLD:ADB2931
Chelonus jorgehernandezi|[99]|BIOUG50048-G03|Malaise trap PL12-3B|653[0n]|BOLD:ADB2931
Chelonus jorgehernandezi|[100]|BIOUG49949-B04|Malaise trap PL12-3B|654[0n]|BOLD:ADB2931
Chelonus jorgehernandezi|[101]|BIOUG55656-C07|Malaise trap PL12-1D|654[0n]|BOLD:ADB2931
Chelonus jorgehernandezi|[102]|BIOUG54760-B12|Malaise trap PL12-5C|655[0n]|BOLD:ADB2931
Chelonus jorgehernandezi|[103]|BIOUG49982-A05|Malaise trap PL12-3B|653[0n]|BOLD:ADB2931
Chelonus jorgehernandezi|[104]|BIOUG56256-G07|Malaise trap PL12-6D|654[0n]|BOLD:ADB2931
Chelonus jorgehernandezi|[105]|BIOUG49947-H08|Malaise trap PL12-3B|654[0n]|BOLD:ADB2931
Chelonus jorgehernandezi|[106]|BIOUG50058-A05|Malaise trap PL12-3B|655[0n]|BOLD:ADB2931
Chelonus jorgehernandezi|[107]|BIOUG50219-B03|Malaise trap PL12-3B|654[0n]|BOLD:ADB2931
Chelonus jorgehernandezi|[108]|BIOUG49945-B04|Malaise trap PL12-3B|654[0n]|BOLD:ADB2931
Chelonus jorgehernandezi|[109]|BIOUG49583-B06|Malaise trap PL12-5B|654[0n]|BOLD:ADB2931
Chelonus jorgehernandezi|[110]|BIOUG50092-C11|Malaise trap PL12-3B|653[0n]|BOLD:ADB2931
Chelonus jorgehernandezi|[111]|BIOUG52254-E04|Malaise trap PL12-8B|653[0n]|BOLD:ADB2931
Chelonus jorgehernandezi|[112]|BIOUG50988-C03|Malaise trap PL12-5B|654[0n]|BOLD:ADB2931
Chelonus jorgehernandezi|[113]|BIOUG49558-H09|Malaise trap PL12-5B|654[0n]|BOLD:ADB2931
Chelonus jorgehernandezi|[114]|BIOUG49367-B04|Malaise trap PL12-5A|654[0n]|BOLD:ADB2931
Chelonus jorgehernandezi|[115]|BIOUG47397-E06|Malaise trap PL12-5A|654[0n]|BOLD:ADB2931
Chelonus jorgehernandezi|[116]|BIOUG51658-E10|Malaise trap PL12-2B|652[0n]|BOLD:ADB2931
Chelonus jorgehernandezi|[117]|BIOUG49945-D07|Malaise trap PL12-3B|654[0n]|BOLD:ADB2931
Chelonus jorgehernandezi|[118]|BIOUG29803-C11|Malaise trap PL12-3A|612[0n]|BOLD:ADB2931
Chelonus jorgehernandezi|[119]|BIOUG49714-G03|Malaise trap PL12-1B|656[0n]|BOLD:ADB2931
Chelonus jorgehernandezi|[120]|BIOUG56702-A05|Malaise trap PL12-9D|654[0n]|BOLD:ADB2931
Chelonus jorgehernandezi|[121]|BIOUG50048-F04|Malaise trap PL12-3B|652[0n]|BOLD:ADB2931
Chelonus|[122]|BIOUG54657-C06|Malaise trap PL12-5C|654[0n]|BOLD:AEA4215
Chelonus Janzen4215|[123]|BIOUG49892-B07|Malaise trap PL12-3B|654[0n]|BOLD:AEA4215
Chelonus Janzen4215|[124]|BIOUG52044-A01|Malaise trap PL12-9B|655[0n]|BOLD:AEA4215
Chelonus Janzen4215|[125]|BIOUG49982-G10|Malaise trap PL12-3B|655[0n]|BOLD:AEA4215
Chelonus Janzen4215|[126]|BIOUG50794-C08|Malaise trap PL12-5B|654[0n]|BOLD:AEA4215
Chelonus Janzen4215|[127]|BIOUG49945-A11|Malaise trap PL12-3B|653[0n]|BOLD:AEA4215
Chelonus johanvalerioi|[128]|DHJPAR0055397|Phostria latiapicalis|658[0n]|BOLD:AAF2611
Chelonus johanvalerioi|[129]|DHJPAR0048106|Pilocrocis purpurascens|658[0n]|BOLD:AAF2611
Chelonus johanvalerioi|[130]|DHJPAR0055951|Pilocrocis purpurascens|670[0n]|BOLD:AAF2611
Chelonus johanvalerioi|[131]|DHJPAR0056967|Pilocrocis purpurascens|616[0n]|BOLD:AAF2611
Chelonus johanvalerioi|[132]|DHJPAR0055381|Phostria latiapicalis|658[0n]|BOLD:AAF2611
Chelonus johanvalerioi|[133]|DHJPAR0052096|Pilocrocis purpurascens|658[0n]|BOLD:AAF2611
Chelonus johanvalerioi|[134]|DHJPAR0042092|Pilocrocis purpurascens|658[0n]|BOLD:AAF2611
Chelonus johanvalerioi|[135]|DHJPAR0055398|Phostria latiapicalis|658[0n]|BOLD:AAF2611
Chelonus johanvalerioi|[136]|DHJPAR0029142|Pilocrocis purpurascens|657[0n]|BOLD:AAF2611
Chelonus johanvalerioi|[137]|DHJPAR0057409|Pilocrocis purpurascens|658[0n]|BOLD:AAF2611
Chelonus johanvalerioi|[138]|DHJPAR0042090|Ceratocilia Janzen02|658[0n]|BOLD:AAF2611
Chelonus johanvalerioi|[139]|DHJPAR0036388|Pilocrocis purpurascens|569[0n]|BOLD:AAF2611
Chelonus johanvalerioi|[140]|DHJPAR0039119|Ceratocilia Janzen02|630[0n]|BOLD:AAF2611
Chelonus johanvalerioi|[141]|DHJPAR0036728|Pilocrocis purpurascens|509[0n]|BOLD:AAF2611
Chelonus johanvalerioi|[142]|DHJPAR0042087|Pilocrocis purpurascens|658[0n]|BOLD:AAF2611
Chelonus johanvalerioi|[143]|DHJPAR0057408|Pilocrocis purpurascens|658[0n]|BOLD:AAF2611
Chelonus Janzen201|[144]|DHJPAR0029173|Herpetogramma Janzen04|229[0n]
Chelonus Janzen201|[145]|DHJPAR0029150|Spoladea recurvalis|271[0n]
Chelonus jimwhitfieldi|[146]|DHJPAR0050073|Prenesta scyllalisDHJ01|658[0n]|BOLD:ACC1273
Chelonus jimwhitfieldi|[147]|DHJPAR0050070|Prenesta scyllalis|658[0n]|BOLD:ACC1273
Chelonus jesusugaldei|[148]|DHJPAR0048100|Herpetogramma phaeopteralis|658[0n]|BOLD:ABY3312
Chelonus jessiehillae|[149]|DHJPAR0042088|gelJanzen01 Janzen349|658[0n]|BOLD:AAW4588
Chelonus jimmilleri|[150]|DHJPAR0062578|Antaeotricha Janzen78|658[0n]|BOLD:ADM8275
Chelonus jimmilleri|[151]|DHJPAR0062577|Antaeotricha Janzen78|658[0n]|BOLD:ADM8275
Chelonus jimmilleri|[152]|DHJPAR0062576|Antaeotricha Janzen78|658[0n]|BOLD:ADM8275
Chelonus jimlewisi|[153]|DHJPAR0029137|Neoleucinodes Janzen02|657[0n]|BOLD:AAF2610
Chelonus jimlewisi|[154]|DHJPAR0029148|Neoleucinodes Janzen02|657[0n]|BOLD:AAF2610
Chelonus jimlewisi|[155]|DHJPAR0030973|Neoleucinodes Janzen02|657[1n]|BOLD:AAF2610
Chelonus jimlewisi|[156]|DHJPAR0051336|Neoleucinodes Janzen02|658[0n]|BOLD:AAF2610
Chelonus jimlewisi|[157]|DHJPAR0054553|Neoleucinodes alegralis|658[0n]|BOLD:AAF2610
Chelonus jimlewisi|[158]|DHJPAR0054556|Neoleucinodes alegralis|658[0n]|BOLD:AAF2610
Chelonus jimlewisi|[159]|DHJPAR0054551|Neoleucinodes alegralis|658[0n]|BOLD:AAF2610
Chelonus Janzen645|[160]|DHJPAR0064502|Desmia benealis|658[0n]|BOLD:AEB3509
Chelonus Janzen645|[161]|DHJPAR0064501|Desmia benealis|658[0n]|BOLD:AEB3509
Chelonus Janzen645|[162]|DHJPAR0064500|Desmia benealis|622[0n]|BOLD:AEB3509
Chelonus janecheverriae|[163]|DHJPAR0039179|crambidJanzen01 Janzen27|599[0n]|BOLD:AAL5555
Chelonus jennyphillipsae|[164]|DHJPAR0053682|Eulepte Janzen12|658[0n]|BOLD:ACL6996
Chelonus jeffmilleri|[165]|DHJPAR0040375|Phostria oajacalis|614[0n]|BOLD:ACF0845
Chelonus jeffmilleri|[166]|DHJPAR0042862|Eulepte Solis15|658[0n]|BOLD:ACF0845
Chelonus jeffmilleri|[167]|DHJPAR0042859|Eulepte Solis15|658[0n]|BOLD:ACF0845
Chelonus jeremydewaardi|[168]|DHJPAR0037989|Patania Solis04|658[0n]|BOLD:AAB1653
Chelonus jeremydewaardi|[169]|DHJPAR0051337|Asturodes fimbriauralis|658[0n]|BOLD:AAB1653
Chelonus jeremydewaardi|[170]|DHJPAR0042084|Herpetogramma salbialis|658[0n]|BOLD:AAB1653
Chelonus jeremydewaardi|[171]|DHJPAR0037982|Patania Solis04|658[0n]|BOLD:AAB1653
Chelonus jeremydewaardi|[172]|DHJPAR0037981|Patania Solis04|658[0n]|BOLD:AAB1653
Chelonus jeremydewaardi|[173]|DHJPAR0053674|Herpetogramma salbialis|658[0n]|BOLD:AAB1653
Chelonus jeremydewaardi|[174]|DHJPAR0046798|Omiodes fulvicauda|658[0n]|BOLD:AAB1653
Chelonus jeremydewaardi|[175]|DHJPAR0046935|Omiodes fulvicauda|658[0n]|BOLD:AAB1653
Chelonus jeremydewaardi|[176]|DHJPAR0045403|Microthyris prolongalisDHJ02|658[0n]|BOLD:AAB1653
Chelonus jeremydewaardi|[177]|DHJPAR0048104|Leucochromodes melusinalisDHJ02|658[0n]|BOLD:AAB1653
Chelonus jeremydewaardi|[178]|DHJPAR0062182|Desmia Janzen03|658[0n]|BOLD:AAB1653
Chelonus jeremydewaardi|[179]|DHJPAR0062188|Desmia Janzen03|658[0n]|BOLD:AAB1653
Chelonus jeremydewaardi|[180]|DHJPAR0062190|Desmia Janzen03|658[0n]|BOLD:AAB1653
Chelonus jeremydewaardi|[181]|DHJPAR0040364|Microthyris prolongalis|658[3n]|BOLD:AAB1653
Chelonus jeremydewaardi|[182]|DHJPAR0029070|Desmia benealisDHJ02|657[0n]|BOLD:AAB1653
Chelonus jeremydewaardi|[183]|DHJPAR0050072|spiloBioLep01 BioLep311|658[0n]|BOLD:AAB1653
Chelonus jeremydewaardi|[184]|DHJPAR0062184|Desmia Janzen03|658[0n]|BOLD:AAB1653
Chelonus jeremydewaardi|[185]|DHJPAR0042860|Eulepte Solis15|658[0n]|BOLD:AAB1653
Chelonus jeremydewaardi|[186]|DHJPAR0048097|Psara obscuralisDHJ01|658[0n]|BOLD:AAB1653
Chelonus jeremydewaardi|[187]|DHJPAR0062189|Desmia Janzen03|658[0n]|BOLD:AAB1653
Chelonus jeremydewaardi|[188]|DHJPAR0048973|Patania Solis03|658[0n]|BOLD:AAB1653
Chelonus jeremydewaardi|[189]|DHJPAR0062187|Desmia Janzen03|658[0n]|BOLD:AAB1653
Chelonus jeremydewaardi|[190]|DHJPAR0062181|Desmia Janzen03|658[0n]|BOLD:AAB1653
Chelonus jeremydewaardi|[191]|DHJPAR0035315|Herpetogramma Solis10|658[0n]|BOLD:AAB1653
Chelonus jeremydewaardi|[192]|DHJPAR0052891|Microthyris prolongalis|658[0n]|BOLD:AAB1653
Chelonus jeremydewaardi|[193]|DHJPAR0062185|Desmia Janzen03|658[0n]|BOLD:AAB1653
Chelonus jeremydewaardi|[194]|DHJPAR0042093|Phostria cyrisalis|658[0n]|BOLD:AAB1653
Chelonus jeremydewaardi|[195]|DHJPAR0053681|Herpetogramma salbialis|658[0n]|BOLD:AAB1653
Chelonus jeremydewaardi|[196]|DHJPAR0035311|Herpetogramma Solis10|658[0n]|BOLD:AAB1653
Chelonus jeremydewaardi|[197]|DHJPAR0062186|Desmia Janzen03|658[0n]|BOLD:AAB1653

Chelonus jeremydewaardi|[194]|[illegible]
Chelonus jeremydewaardi|[195]|DHJPAR0053681|Herpetogramma salbialis|658[0n]|BOLD:AAB1653
Chelonus jeremydewaardi|[196]|DHJPAR0035311|Herpetogramma Solis10|658[0n]|BOLD:AAB1653
Chelonus jeremydewaardi|[197]|DHJPAR0062186|Desmia Janzen03|658[0n]|BOLD:AAB1653
Chelonus jeremydewaardi|[198]|DHJPAR0035309|Eulepte Solis15|658[0n]|BOLD:AAB1653
Chelonus jeremydewaardi|[199]|DHJPAR0062191|Desmia Janzen03|658[0n]|BOLD:AAB1653
Chelonus jeremydewaardi|[200]|DHJPAR0029117|Asturodes fimbriauralisDHJ01|657[0n]|BOLD:AAB1653
Chelonus jeremydewaardi|[201]|DHJPAR0055370|Syllepis marialis|658[0n]|BOLD:AAB1653
Chelonus jeremydewaardi|[202]|DHJPAR0062183|Desmia Janzen03|658[0n]|BOLD:AAB1653
Chelonus jeremydewaardi|[203]|DHJPAR0036400|Asturodes fimbriauralis|658[0n]|BOLD:AAB1653
Chelonus jeremydewaardi|[204]|DHJPAR0056949|Herpetogramma salbialis|661[0n]|BOLD:AAB1653
Chelonus jeremydewaardi|[205]|DHJPAR0048094|Psara obscuralisDHJ01|658[0n]|BOLD:AAB1653
Chelonus jeremydewaardi|[206]|DHJPAR0045402|Microthyris prolongalisDHJ02|657[0n]|BOLD:AAB1653
Chelonus jeremydewaardi|[207]|DHJPAR0052887|Desmia Solis100|658[0n]|BOLD:AAB1653
Chelonus jeremydewaardi|[208]|DHJPAR0055376|Syllepis marialis|658[0n]|BOLD:AAB1653
Chelonus jeremydewaardi|[209]|DHJPAR0039121|Asturodes fimbriauralis|658[0n]|BOLD:AAB1653
Chelonus jeremydewaardi|[210]|DHJPAR0062180|Desmia Janzen03|658[0n]|BOLD:AAB1653
Chelonus jeremydewaardi|[211]|DHJPAR0037182|Patania Solis04|658[0n]|BOLD:AAB1653
Chelonus jeremydewaardi|[212]|DHJPAR0048093|Psara obscuralisDHJ01|658[0n]|BOLD:AAB1653
Chelonus jeremydewaardi|[213]|DHJPAR0042096|Patania Solis04|658[0n]|BOLD:AAB1653
Chelonus jeremydewaardi|[214]|DHJPAR0048092|Psara obscuralisDHJ01|658[0n]|BOLD:AAB1653
Chelonus jeremydewaardi|[215]|DHJPAR0037979|Rhectocraspeda periusalis|658[0n]|BOLD:AAB1653
Chelonus jeremydewaardi|[216]|DHJPAR0040368|Phostria oajacalis|658[0n]|BOLD:AAB1653
Chelonus jeremydewaardi|[217]|DHJPAR0048091|Psara obscuralisDHJ01|658[0n]|BOLD:AAB1653
Chelonus jeremydewaardi|[218]|DHJPAR0045408|Asturodes fimbriauralis|656[3n]|BOLD:AAB1653
Chelonus jeremydewaardi|[219]|DHJPAR0042857|Eulepte Solis15|633[0n]|BOLD:AAB1653
Chelonus jeremydewaardi|[220]|DHJPAR0037862|Ceratocilia sixolalis|658[0n]|BOLD:AAB1653
Chelonus jeremydewaardi|[221]|DHJPAR0048169|Desmia ploralisDHJ10|658[0n]|BOLD:AAB1653
Chelonus jeremydewaardi|[222]|DHJPAR0058149|Asturodes fimbriauralis|658[0n]|BOLD:AAB1653
Chelonus jeremydewaardi|[223]|DHJPAR0029069|Salbia cassidalis|657[0n]|BOLD:AAB1653
Chelonus jeremydewaardi|[224]|DHJPAR0039502|Asturodes fimbriauralis|658[0n]|BOLD:AAB1653
Chelonus torbjornekremi|[225]|DHJPAR0045404|spiloJanzen01 Janzen57|541[19n]
Chelonus torbjornekremi|[226]|DHJPAR0056964|pyrausJanzen01 Janzen26|658[0n]|BOLD:ACZ0402
Chelonus torbjornekremi|[227]|DHJPAR0060594|pyrausJanzen01 Janzen26|658[0n]|BOLD:ACZ0402
Chelonus torbjornekremi|[228]|DHJPAR0061493|Aponia Janzen469|658[0n]|BOLD:ACZ0402
Chelonus hazelcambroneroae|[229]|DHJPAR0022199|Prenesta scyllalisDHJ02|657[0n]|BOLD:AAE9128
Chelonus hazelcambroneroae|[230]|DHJPAR0030988|Prenesta scyllalisDHJ02|657[0n]|BOLD:AAE9128
Chelonus hazelcambroneroae|[231]|DHJPAR0030990|Prenesta scyllalisDHJ02|657[0n]|BOLD:AAE9128
Chelonus hazelcambroneroae|[232]|DHJPAR0030989|Prenesta scyllalisDHJ02|657[0n]|BOLD:AAE9128
Chelonus hazelcambroneroae|[233]|DHJPAR0049930|Prenesta scyllalisDHJ02|658[0n]|BOLD:AAE9128
Chelonus isidrochaconi|[234]|DHJPAR0037173|Parastenia retractalis|655[0n]|BOLD:AAC8260
Chelonus isidrochaconi|[235]|DHJPAR0037181|Parastenia retractalis|633[0n]|BOLD:AAC8260
Chelonus isidrochaconi|[236]|DHJPAR0037162|Parastenia retractalis|658[0n]|BOLD:AAC8260
Chelonus isidrochaconi|[237]|DHJPAR0037176|Parastenia retractalis|658[0n]|BOLD:AAC8260
Chelonus isidrochaconi|[238]|DHJPAR0037174|Parastenia retractalis|626[0n]|BOLD:AAC8260
Chelonus isidrochaconi|[239]|DHJPAR0037159|Parastenia retractalis|606[0n]|BOLD:AAC8260
Chelonus isidrochaconi|[240]|DHJPAR0037166|Parastenia retractalis|646[0n]|BOLD:AAC8260
Chelonus isidrochaconi|[241]|DHJPAR0037172|Parastenia retractalis|654[0n]|BOLD:AAC8260
Chelonus isidrochaconi|[242]|DHJPAR0037179|Parastenia retractalis|658[0n]|BOLD:AAC8260
Chelonus isidrochaconi|[243]|DHJPAR0049019|Parastenia retractalis|658[0n]|BOLD:AAC8260
Chelonus isidrochaconi|[244]|DHJPAR0049008|Parastenia retractalis|658[0n]|BOLD:AAC8260
Chelonus isidrochaconi|[245]|DHJPAR0049038|Parastenia retractalis|658[0n]|BOLD:AAC8260
Chelonus isidrochaconi|[246]|DHJPAR0049041|Parastenia retractalis|658[0n]|BOLD:AAC8260
Chelonus isidrochaconi|[247]|DHJPAR0049028|Parastenia retractalis|658[0n]|BOLD:AAC8260
Chelonus isidrochaconi|[248]|DHJPAR0049015|Parastenia retractalis|658[0n]|BOLD:AAC8260
Chelonus isidrochaconi|[249]|DHJPAR0049030|Parastenia retractalis|658[0n]|BOLD:AAC8260
Chelonus isidrochaconi|[250]|DHJPAR0048990|Parastenia retractalis|658[0n]|BOLD:AAC8260
Chelonus isidrochaconi|[251]|DHJPAR0049035|Parastenia retractalis|658[0n]|BOLD:AAC8260
Chelonus isidrochaconi|[252]|DHJPAR0048997|Parastenia retractalis|658[0n]|BOLD:AAC8260
Chelonus isidrochaconi|[253]|DHJPAR0049016|Parastenia retractalis|658[0n]|BOLD:AAC8260
Chelonus isidrochaconi|[254]|DHJPAR0048999|Parastenia retractalis|658[0n]|BOLD:AAC8260
Chelonus isidrochaconi|[255]|DHJPAR0049012|Parastenia retractalis|658[0n]|BOLD:AAC8260
Chelonus isidrochaconi|[256]|DHJPAR0049004|Parastenia retractalis|658[0n]|BOLD:AAC8260
Chelonus isidrochaconi|[257]|DHJPAR0049005|Parastenia retractalis|658[0n]|BOLD:AAC8260
Chelonus isidrochaconi|[258]|DHJPAR0048998|Parastenia retractalis|658[0n]|BOLD:AAC8260
Chelonus isidrochaconi|[259]|DHJPAR0049002|Parastenia retractalis|658[0n]|BOLD:AAC8260
Chelonus isidrochaconi|[260]|DHJPAR0048991|Parastenia retractalis|658[0n]|BOLD:AAC8260
Chelonus isidrochaconi|[261]|DHJPAR0048983|Parastenia retractalis|658[0n]|BOLD:AAC8260
Chelonus isidrochaconi|[262]|DHJPAR0049040|Parastenia retractalis|658[0n]|BOLD:AAC8260
Chelonus isidrochaconi|[263]|DHJPAR0049025|Parastenia retractalis|658[0n]|BOLD:AAC8260
Chelonus isidrochaconi|[264]|DHJPAR0049020|Parastenia retractalis|658[0n]|BOLD:AAC8260
Chelonus isidrochaconi|[265]|DHJPAR0049034|Parastenia retractalis|658[0n]|BOLD:AAC8260
Chelonus isidrochaconi|[266]|DHJPAR0049024|Parastenia retractalis|658[0n]|BOLD:AAC8260
Chelonus isidrochaconi|[267]|DHJPAR0049026|Parastenia retractalis|658[0n]|BOLD:AAC8260
Chelonus isidrochaconi|[268]|DHJPAR0049000|Parastenia retractalis|658[0n]|BOLD:AAC8260
Chelonus isidrochaconi|[269]|DHJPAR0049039|Parastenia retractalis|658[0n]|BOLD:AAC8260
Chelonus isidrochaconi|[270]|DHJPAR0049011|Parastenia retractalis|658[0n]|BOLD:AAC8260
Chelonus isidrochaconi|[271]|DHJPAR0049021|Parastenia retractalis|658[0n]|BOLD:AAC8260
Chelonus isidrochaconi|[272]|DHJPAR0048986|Parastenia retractalis|658[0n]|BOLD:AAC8260
Chelonus isidrochaconi|[273]|DHJPAR0049037|Parastenia retractalis|658[0n]|BOLD:AAC8260
Chelonus isidrochaconi|[274]|DHJPAR0049023|Parastenia retractalis|658[0n]|BOLD:AAC8260
Chelonus isidrochaconi|[275]|DHJPAR0048988|Parastenia retractalis|658[0n]|BOLD:AAC8260
Chelonus isidrochaconi|[276]|DHJPAR0048987|Parastenia retractalis|658[0n]|BOLD:AAC8260
Chelonus isidrochaconi|[277]|DHJPAR0049009|Parastenia retractalis|658[0n]|BOLD:AAC8260
Chelonus isidrochaconi|[278]|DHJPAR0048993|Parastenia retractalis|658[0n]|BOLD:AAC8260
Chelonus isidrochaconi|[279]|DHJPAR0048995|Parastenia retractalis|658[0n]|BOLD:AAC8260
Chelonus isidrochaconi|[280]|DHJPAR0048989|Parastenia retractalis|658[0n]|BOLD:AAC8260
Chelonus isidrochaconi|[281]|DHJPAR0049022|Parastenia retractalis|658[0n]|BOLD:AAC8260
Chelonus isidrochaconi|[282]|DHJPAR0049031|Parastenia retractalis|658[0n]|BOLD:AAC8260
Chelonus isidrochaconi|[283]|DHJPAR0049029|Parastenia retractalis|658[0n]|BOLD:AAC8260
Chelonus isidrochaconi|[284]|DHJPAR0048984|Parastenia retractalis|658[0n]|BOLD:AAC8260
Chelonus Janzen7002|[285]|DHJPAR0029071|Hileithia Solis01DHJ01|609[0n]|BOLD:ACK7002
Chelonus harryramirezi|[286]|DHJPAR0020804|Omiodes humeralis|660[0n]|BOLD:AAE2698
Chelonus harryramirezi|[287]|DHJPAR0038012|Omiodes humeralis|658[0n]|BOLD:AAE2698
Chelonus harryramirezi|[288]|DHJPAR0054938|Omiodes humeralis|658[1n]|BOLD:AAE2698
Chelonus harryramirezi|[289]|DHJPAR0051330|Omiodes humeralis|658[0n]|BOLD:AAE2698
Chelonus harryramirezi|[290]|DHJPAR0020624|Omiodes humeralis|657[0n]|BOLD:AAE2698
Chelonus harryramirezi|[291]|DHJPAR0051326|Omiodes humeralis|658[0n]|BOLD:AAE2698
Chelonus harryramirezi|[292]|DHJPAR0051328|Omiodes humeralis|658[0n]|BOLD:AAE2698
Chelonus harryramirezi|[293]|DHJPAR0038013|Omiodes humeralis|658[0n]|BOLD:AAE2698
Chelonus harryramirezi|[294]|DHJPAR0046938|Omiodes humeralis|658[0n]|BOLD:AAE2698
Chelonus harryramirezi|[295]|DHJPAR0038014|Omiodes humeralis|658[0n]|BOLD:AAE2698

[illegible]
Chelonus harryramirezi|[293]|DHJPAR0038013|Omiodes humeralis|658[0n]|BOLD:AAE2698
Chelonus harryramirezi|[294]|DHJPAR0046938|Omiodes humeralis|658[0n]|BOLD:AAE2698
Chelonus harryramirezi|[295]|DHJPAR0038014|Omiodes humeralis|658[0n]|BOLD:AAE2698
Chelonus harryramirezi|[296]|DHJPAR0020802|Omiodes humeralis|657[0n]|BOLD:AAE2698
Chelonus harryramirezi|[297]|DHJPAR0054564|Omiodes humeralis|658[0n]|BOLD:AAE2698
Chelonus harryramirezi|[298]|DHJPAR0020623|Omiodes humeralis|653[0n]|BOLD:AAE2698
Chelonus harryramirezi|[299]|DHJPAR0023700|Omiodes humeralis|646[0n]|BOLD:AAE2698
Chelonus harryramirezi|[300]|DHJPAR0051927|Omiodes humeralis|652[2n]|BOLD:AAE2698
Chelonus harryramirezi|[301]|DHJPAR0055229|Omiodes humeralis|658[0n]|BOLD:AAE2698
Chelonus hartmanguidoi|[302]|DHJPAR0054439|Omiodes Janzen05|614[0n]|BOLD:AAD1994
Chelonus hartmanguidoi|[303]|DHJPAR0054571|Omiodes Janzen05|658[0n]|BOLD:AAD1994
Chelonus hartmanguidoi|[304]|DHJPAR0054570|Omiodes Janzen05|658[0n]|BOLD:AAD1994
Chelonus hartmanguidoi|[305]|DHJPAR0056963|Omiodes Janzen05|614[0n]|BOLD:AAD1994
Chelonus hartmanguidoi|[306]|DHJPAR0046801|Omiodes Janzen05|658[0n]|BOLD:AAD1994
Chelonus hartmanguidoi|[307]|DHJPAR0036398|Phostria cyrisalis|658[0n]|BOLD:AAD1994
Chelonus hartmanguidoi|[308]|DHJPAR0035222|spiloBioLep01 BioLep577|658[0n]|BOLD:AAD1994
Chelonus hartmanguidoi|[309]|DHJPAR0042543|Omiodes Janzen05|658[0n]|BOLD:AAD1994
Chelonus hartmanguidoi|[310]|DHJPAR0046947|Omiodes Janzen05|658[0n]|BOLD:AAD1994
Chelonus hartmanguidoi|[311]|DHJPAR0029166|Omiodes Janzen03|657[0n]|BOLD:AAD1994
Chelonus hartmanguidoi|[312]|DHJPAR0029154|Omiodes Janzen03|657[0n]|BOLD:AAD1994
Chelonus hartmanguidoi|[313]|DHJPAR0053677|Omiodes Janzen05|658[0n]|BOLD:AAD1994
Chelonus hartmanguidoi|[314]|DHJPAR0056958|Omiodes Janzen03|658[0n]|BOLD:AAD1994
Chelonus hartmanguidoi|[315]|DHJPAR0053686|Omiodes Janzen05|658[0n]|BOLD:AAD1994
Chelonus hartmanguidoi|[316]|DHJPAR0056968|Omiodes Janzen05|658[0n]|BOLD:AAD1994
Chelonus hartmanguidoi|[317]|DHJPAR0052896|Omiodes Janzen05|658[0n]|BOLD:AAD1994
Chelonus hartmanguidoi|[318]|DHJPAR0035308|Piletosoma thialis|658[0n]|BOLD:AAD1994
Chelonus hartmanguidoi|[319]|DHJPAR0035313|Piletosoma thialis|658[0n]|BOLD:AAD1994
Chelonus hartmanguidoi|[320]|DHJPAR0056969|Omiodes Janzen05|658[0n]|BOLD:AAD1994
Chelonus hartmanguidoi|[321]|DHJPAR0056957|Omiodes Janzen03|658[0n]|BOLD:AAD1994
Chelonus hartmanguidoi|[322]|DHJPAR0035312|Omiodes Janzen05|658[0n]|BOLD:AAD1994
Chelonus hartmanguidoi|[323]|DHJPAR0035307|Piletosoma thialis|658[0n]|BOLD:AAD1994
Chelonus iangauldi|[324]|DHJPAR0037168|Prenesta scyllalisDHJ01|658[0n]|BOLD:AAD4709
Chelonus iangauldi|[325]|DHJPAR0036726|Prenesta scyllalisDHJ01|658[0n]|BOLD:AAD4709
Chelonus iangauldi|[326]|DHJPAR0037160|Prenesta scyllalisDHJ01|633[0n]|BOLD:AAD4709
Chelonus iangauldi|[327]|DHJPAR0037175|Prenesta scyllalisDHJ01|658[0n]|BOLD:AAD4709
Chelonus iangauldi|[328]|DHJPAR0037183|Prenesta scyllalisDHJ01|658[0n]|BOLD:AAD4709
Chelonus iangauldi|[329]|DHJPAR0037169|Prenesta scyllalisDHJ01|658[0n]|BOLD:AAD4709
Chelonus iangauldi|[330]|DHJPAR0037165|Prenesta scyllalisDHJ01|658[0n]|BOLD:AAD4709
Chelonus iangauldi|[331]|DHJPAR0040017|Prenesta scyllalisDHJ01|658[0n]|BOLD:AAD4709
Chelonus gustavogutierrezi|[332]|DHJPAR0029122|Omiodes cuniculalis|274[0n]
Chelonus gustavogutierrezi|[333]|DHJPAR0029121|Omiodes cuniculalis|272[0n]
Chelonus gustavogutierrezi|[334]|DHJPAR0029112|Omiodes cuniculalis|507[2n]|BOLD:AAA6603
Chelonus gustavogutierrezi|[335]|DHJPAR0029098|Omiodes cuniculalis|266[0n]
Chelonus gustavogutierrezi|[336]|DHJPAR0029099|Omiodes cuniculalis|621[0n]|BOLD:AAA6603
Chelonus gustavogutierrezi|[337]|DHJPAR0055601|Omiodes cuniculalis|661[0n]|BOLD:AAA6603
Chelonus gustavogutierrezi|[338]|DHJPAR0055822|Omiodes cuniculalis|658[0n]|BOLD:AAA6603
Chelonus gustavogutierrezi|[339]|DHJPAR0064013|Omiodes cuniculalis|626[0n]|BOLD:AAA6603
Chelonus gustavogutierrezi|[340]|DHJPAR0063961|Omiodes cuniculalis|622[0n]|BOLD:AAA6603
Chelonus gustavogutierrezi|[341]|DHJPAR0062691|Omiodes cuniculalis|614[0n]|BOLD:AAA6603
Chelonus gustavogutierrezi|[342]|DHJPAR0062712|Omiodes cuniculalis|614[0n]|BOLD:AAA6603
Chelonus gustavogutierrezi|[343]|DHJPAR0049036|Omiodes Janzen07|658[0n]|BOLD:AAA6603
Chelonus gustavogutierrezi|[344]|DHJPAR0029151|Omiodes cuniculalis|657[0n]|BOLD:AAA6603
Chelonus gustavogutierrezi|[345]|DHJPAR0049017|Omiodes Janzen07|658[0n]|BOLD:AAA6603
Chelonus gustavogutierrezi|[346]|DHJPAR0038011|Omiodes cuniculalis|658[0n]|BOLD:AAA6603
Chelonus gustavogutierrezi|[347]|DHJPAR0037863|Omiodes cuniculalis|658[0n]|BOLD:AAA6603
Chelonus gustavogutierrezi|[348]|DHJPAR0029165|Omiodes cuniculalis|657[0n]|BOLD:AAA6603
Chelonus gustavogutierrezi|[349]|DHJPAR0038008|Omiodes cuniculalis|658[0n]|BOLD:AAA6603
Chelonus gustavogutierrezi|[350]|DHJPAR0055823|Omiodes cuniculalis|658[0n]|BOLD:AAA6603
Chelonus gustavogutierrezi|[351]|DHJPAR0062711|Omiodes cuniculalis|658[0n]|BOLD:AAA6603
Chelonus gustavogutierrezi|[352]|DHJPAR0037985|Pyralidae 09-SRNP-71481|658[0n]|BOLD:AAA6603
Chelonus gustavogutierrezi|[353]|DHJPAR0038005|Omiodes cuniculalis|658[0n]|BOLD:AAA6603
Chelonus gustavogutierrezi|[354]|DHJPAR0029144|Omiodes cuniculalis|657[0n]|BOLD:AAA6603
Chelonus gustavogutierrezi|[355]|DHJPAR0038001|Omiodes cuniculalis|658[0n]|BOLD:AAA6603
Chelonus gustavogutierrezi|[356]|DHJPAR0037983|Omiodes cuniculalis|658[0n]|BOLD:AAA6603
Chelonus gustavogutierrezi|[357]|DHJPAR0038000|Omiodes cuniculalis|658[0n]|BOLD:AAA6603
Chelonus gustavogutierrezi|[358]|DHJPAR0037976|Omiodes cuniculalis|658[0n]|BOLD:AAA6603
Chelonus gustavogutierrezi|[359]|DHJPAR0029138|Omiodes cuniculalis|657[0n]|BOLD:AAA6603
Chelonus gustavogutierrezi|[360]|DHJPAR0062703|Omiodes cuniculalis|658[0n]|BOLD:AAA6603
Chelonus gustavogutierrezi|[361]|DHJPAR0029139|Omiodes cuniculalis|657[0n]|BOLD:AAA6603
Chelonus gustavogutierrezi|[362]|DHJPAR0029149|Omiodes cuniculalis|657[0n]|BOLD:AAA6603
Chelonus gustavogutierrezi|[363]|DHJPAR0029164|Omiodes cuniculalis|657[0n]|BOLD:AAA6603
Chelonus gustavogutierrezi|[364]|DHJPAR0062706|Omiodes cuniculalis|658[0n]|BOLD:AAA6603
Chelonus gustavogutierrezi|[365]|DHJPAR0038009|Omiodes cuniculalis|658[0n]|BOLD:AAA6603
Chelonus gustavogutierrezi|[366]|DHJPAR0055821|Omiodes cuniculalis|658[0n]|BOLD:AAA6603
Chelonus gustavogutierrezi|[367]|DHJPAR0062700|Omiodes cuniculalis|658[0n]|BOLD:AAA6603
Chelonus gustavogutierrezi|[368]|DHJPAR0029159|Omiodes cuniculalis|657[0n]|BOLD:AAA6603
Chelonus gustavogutierrezi|[369]|DHJPAR0029156|Omiodes cuniculalis|657[0n]|BOLD:AAA6603
Chelonus gustavogutierrezi|[370]|DHJPAR0054568|Omiodes cuniculalis|658[0n]|BOLD:AAA6603
Chelonus gustavogutierrezi|[371]|DHJPAR0055083|Omiodes cuniculalis|658[0n]|BOLD:AAA6603
Chelonus gustavogutierrezi|[372]|DHJPAR0037980|Omiodes cuniculalis|658[0n]|BOLD:AAA6603
Chelonus gustavogutierrezi|[373]|DHJPAR0029143|Omiodes cuniculalis|657[0n]|BOLD:AAA6603
Chelonus gustavogutierrezi|[374]|DHJPAR0062705|Omiodes cuniculalis|658[0n]|BOLD:AAA6603
Chelonus gustavogutierrezi|[375]|DHJPAR0048985|Omiodes Janzen07|658[0n]|BOLD:AAA6603
Chelonus gustavogutierrezi|[376]|DHJPAR0054569|Omiodes cuniculalis|658[0n]|BOLD:AAA6603
Chelonus gustavogutierrezi|[377]|DHJPAR0062696|Omiodes cuniculalis|658[0n]|BOLD:AAA6603
Chelonus gustavogutierrezi|[378]|DHJPAR0049010|Omiodes Janzen07|658[0n]|BOLD:AAA6603
Chelonus gustavogutierrezi|[379]|DHJPAR0037991|Omiodes cuniculalis|658[0n]|BOLD:AAA6603
Chelonus gustavogutierrezi|[380]|DHJPAR0029171|Omiodes cuniculalis|657[0n]|BOLD:AAA6603
Chelonus gustavogutierrezi|[381]|DHJPAR0062699|Omiodes cuniculalis|658[0n]|BOLD:AAA6603
Chelonus gustavogutierrezi|[382]|DHJPAR0029155|Omiodes cuniculalis|657[0n]|BOLD:AAA6603
Chelonus gustavogutierrezi|[383]|DHJPAR0055368|Omiodes cuniculalis|658[0n]|BOLD:AAA6603
Chelonus gustavogutierrezi|[384]|DHJPAR0062692|Omiodes cuniculalis|658[0n]|BOLD:AAA6603
Chelonus gustavogutierrezi|[385]|DHJPAR0037999|Omiodes cuniculalis|658[0n]|BOLD:AAA6603
Chelonus gustavogutierrezi|[386]|DHJPAR0029153|Omiodes cuniculalis|657[0n]|BOLD:AAA6603
Chelonus gustavogutierrezi|[387]|DHJPAR0037997|Omiodes cuniculalis|658[0n]|BOLD:AAA6603
Chelonus gustavogutierrezi|[388]|DHJPAR0049013|Omiodes Janzen07|658[0n]|BOLD:AAA6603
Chelonus gustavogutierrezi|[389]|DHJPAR0038002|Omiodes cuniculalis|658[0n]|BOLD:AAA6603
Chelonus gustavogutierrezi|[390]|DHJPAR0055367|Omiodes cuniculalis|658[0n]|BOLD:AAA6603
Chelonus gustavogutierrezi|[391]|DHJPAR0029169|Omiodes cuniculalis|657[0n]|BOLD:AAA6603
Chelonus gustavogutierrezi|[392]|DHJPAR0049014|Omiodes Janzen07|658[0n]|BOLD:AAA6603
Chelonus gustavogutierrezi|[393]|DHJPAR0029172|Omiodes cuniculalis|657[0n]|BOLD:AAA6603

Chelonus gustavogutierrezi|[391]|DHJPAR0029169|Omiodes cuniculalis|657[0n]|BOLD:AAA6603
Chelonus gustavogutierrezi|[392]|DHJPAR0049014|Omiodes Janzen07|658[0n]|BOLD:AAA6603
Chelonus gustavogutierrezi|[393]|DHJPAR0029172|Omiodes cuniculalis|657[0n]|BOLD:AAA6603
Chelonus gustavogutierrezi|[394]|DHJPAR0062707|Omiodes cuniculalis|658[0n]|BOLD:AAA6603
Chelonus gustavogutierrezi|[395]|DHJPAR0037987|Omiodes cuniculalis|658[0n]|BOLD:AAA6603
Chelonus gustavogutierrezi|[396]|DHJPAR0037170|Omiodes cuniculalis|658[0n]|BOLD:AAA6603
Chelonus gustavogutierrezi|[397]|DHJPAR0038006|Omiodes cuniculalis|658[0n]|BOLD:AAA6603
Chelonus gustavogutierrezi|[398]|DHJPAR0037994|Omiodes cuniculalis|658[0n]|BOLD:AAA6603
Chelonus gustavogutierrezi|[399]|DHJPAR0037986|Omiodes cuniculalis|658[0n]|BOLD:AAA6603
Chelonus gustavogutierrezi|[400]|DHJPAR0038010|Omiodes cuniculalis|658[0n]|BOLD:AAA6603
Chelonus gustavogutierrezi|[401]|DHJPAR0029167|Omiodes cuniculalis|657[0n]|BOLD:AAA6603
Chelonus gustavogutierrezi|[402]|DHJPAR0021578|Omiodes cuniculalis|657[0n]|BOLD:AAA6603
Chelonus gustavogutierrezi|[403]|DHJPAR0037163|Omiodes cuniculalis|658[0n]|BOLD:AAA6603
Chelonus gustavogutierrezi|[404]|DHJPAR0055357|Omiodes cuniculalis|658[0n]|BOLD:AAA6603
Chelonus gustavogutierrezi|[405]|DHJPAR0037984|Omiodes cuniculalis|658[0n]|BOLD:AAA6603
Chelonus gustavogutierrezi|[406]|DHJPAR0055356|Omiodes cuniculalis|658[0n]|BOLD:AAA6603
Chelonus gustavogutierrezi|[407]|DHJPAR0062710|Omiodes cuniculalis|658[0n]|BOLD:AAA6603
Chelonus gustavogutierrezi|[408]|DHJPAR0062701|Omiodes cuniculalis|658[0n]|BOLD:AAA6603
Chelonus gustavogutierrezi|[409]|DHJPAR0062694|Omiodes cuniculalis|614[0n]|BOLD:AAA6603
Chelonus gustavogutierrezi|[410]|DHJPAR0062698|Omiodes cuniculalis|608[0n]|BOLD:AAA6603
Chelonus gustavogutierrezi|[411]|DHJPAR0029092|Omiodes cuniculalis|657[2n]|BOLD:AAA6603
Chelonus gustavogutierrezi|[412]|DHJPAR0029074|Omiodes cuniculalis|657[0n]|BOLD:AAA6603
Chelonus gustavogutierrezi|[413]|DHJPAR0029090|Omiodes cuniculalis|657[0n]|BOLD:AAA6603
Chelonus gustavogutierrezi|[414]|DHJPAR0029130|Omiodes cuniculalis|657[0n]|BOLD:AAA6603
Chelonus gustavogutierrezi|[415]|DHJPAR0039498|Omiodes cuniculalis|658[0n]|BOLD:AAA6603
Chelonus gustavogutierrezi|[416]|DHJPAR0029080|Omiodes cuniculalis|657[0n]|BOLD:AAA6603
Chelonus gustavogutierrezi|[417]|DHJPAR0029072|Omiodes cuniculalis|657[0n]|BOLD:AAA6603
Chelonus gustavogutierrezi|[418]|DHJPAR0029129|Omiodes cuniculalis|657[0n]|BOLD:AAA6603
Chelonus gustavogutierrezi|[419]|DHJPAR0029091|Omiodes cuniculalis|657[0n]|BOLD:AAA6603
Chelonus gustavogutierrezi|[420]|DHJPAR0064010|Omiodes cuniculalis|658[0n]|BOLD:AAA6603
Chelonus gustavogutierrezi|[421]|DHJPAR0029089|Omiodes cuniculalis|657[0n]|BOLD:AAA6603
Chelonus gustavogutierrezi|[422]|DHJPAR0029125|Omiodes cuniculalis|657[5n]|BOLD:AAA6603
Chelonus gustavogutierrezi|[423]|DHJPAR0029126|Omiodes cuniculalis|583[0n]|BOLD:AAA6603
Chelonus gustavogutierrezi|[424]|DHJPAR0062702|Omiodes cuniculalis|607[0n]|BOLD:AAA6603
Chelonus gustavogutierrezi|[425]|DHJPAR0062704|Omiodes cuniculalis|607[0n]|BOLD:AAA6603
Chelonus gustavogutierrezi|[426]|DHJPAR0029107|Omiodes cuniculalis|657[2n]|BOLD:AAA6603
Chelonus gustavogutierrezi|[427]|DHJPAR0062709|Omiodes cuniculalis|640[0n]|BOLD:AAA6603
Chelonus gustavogutierrezi|[428]|DHJPAR0064014|Omiodes cuniculalis|631[0n]|BOLD:AAA6603
Chelonus gustavogutierrezi|[429]|DHJPAR0062695|Omiodes cuniculalis|648[0n]|BOLD:AAA6603
Chelonus gustavogutierrezi|[430]|DHJPAR0029110|Omiodes cuniculalis|629[0n]|BOLD:AAA6603
Chelonus gustavogutierrezi|[431]|DHJPAR0029158|Omiodes cuniculalis|629[0n]|BOLD:AAA6603
Chelonus gustavogutierrezi|[432]|DHJPAR0037185|Omiodes cuniculalis|630[0n]|BOLD:AAA6603
Chelonus gustavogutierrezi|[433]|DHJPAR0037164|Omiodes cuniculalis|649[0n]|BOLD:AAA6603
Chelonus gustavogutierrezi|[434]|DHJPAR0049006|Omiodes Janzen07|633[0n]|BOLD:AAA6603
Chelonus gustavogutierrezi|[435]|DHJPAR0029170|Omiodes cuniculalis|630[0n]|BOLD:AAA6603
Chelonus gustavogutierrezi|[436]|DHJPAR0062708|Omiodes cuniculalis|633[0n]|BOLD:AAA6603
Chelonus gustavogutierrezi|[437]|DHJPAR0055602|Omiodes cuniculalis|597[0n]|BOLD:AAA6603
Chelonus gustavogutierrezi|[438]|DHJPAR0036401|Omiodes cuniculalis|640[0n]|BOLD:AAA6603
Chelonus gustavogutierrezi|[439]|DHJPAR0029097|Omiodes cuniculalis|657[0n]|BOLD:AAA6603
Chelonus gustavogutierrezi|[440]|DHJPAR0029102|Omiodes cuniculalis|657[0n]|BOLD:AAA6603
Chelonus gustavogutierrezi|[441]|DHJPAR0029111|Omiodes cuniculalis|657[0n]|BOLD:AAA6603
Chelonus gustavogutierrezi|[442]|DHJPAR0042094|Omiodes cuniculalis|658[0n]|BOLD:AAA6603
Chelonus gustavogutierrezi|[443]|DHJPAR0042085|Omiodes cuniculalis|658[0n]|BOLD:AAA6603
Chelonus gustavogutierrezi|[444]|DHJPAR0055382|Omiodes cuniculalis|658[0n]|BOLD:AAA6603
Chelonus gustavogutierrezi|[445]|DHJPAR0029119|Omiodes cuniculalis|657[0n]|BOLD:AAA6603
Chelonus gustavogutierrezi|[446]|DHJPAR0029085|Omiodes cuniculalis|657[0n]|BOLD:AAA6603
Chelonus gustavogutierrezi|[447]|DHJPAR0039501|Omiodes cuniculalis|658[0n]|BOLD:AAA6603
Chelonus gustavogutierrezi|[448]|DHJPAR0029075|Omiodes cuniculalis|657[0n]|BOLD:AAA6603
Chelonus gustavogutierrezi|[449]|DHJPAR0029113|Omiodes cuniculalis|657[0n]|BOLD:AAA6603
Chelonus gustavogutierrezi|[450]|DHJPAR0037996|Omiodes cuniculalis|658[0n]|BOLD:AAA6603
Chelonus gustavogutierrezi|[451]|DHJPAR0029105|Omiodes cuniculalis|657[0n]|BOLD:AAA6603
Chelonus gustavogutierrezi|[452]|DHJPAR0029106|Omiodes cuniculalis|657[0n]|BOLD:AAA6603
Chelonus gustavogutierrezi|[453]|DHJPAR0039500|Omiodes cuniculalis|658[0n]|BOLD:AAA6603
Chelonus gustavogutierrezi|[454]|DHJPAR0039497|Omiodes cuniculalis|658[0n]|BOLD:AAA6603
Chelonus gustavogutierrezi|[455]|DHJPAR0029083|Omiodes cuniculalis|657[0n]|BOLD:AAA6603
Chelonus gustavogutierrezi|[456]|DHJPAR0037998|Omiodes cuniculalis|658[0n]|BOLD:AAA6603
Chelonus gustavogutierrezi|[457]|DHJPAR0064016|Omiodes cuniculalis|658[0n]|BOLD:AAA6603
Chelonus gustavogutierrezi|[458]|DHJPAR0064011|Omiodes cuniculalis|658[0n]|BOLD:AAA6603
Chelonus gustavogutierrezi|[459]|DHJPAR0037990|Omiodes cuniculalis|658[0n]|BOLD:AAA6603
Chelonus gustavogutierrezi|[460]|DHJPAR0039499|Omiodes cuniculalis|658[0n]|BOLD:AAA6603
Chelonus gustavogutierrezi|[461]|DHJPAR0064015|Omiodes cuniculalis|658[0n]|BOLD:AAA6603
Chelonus gustavogutierrezi|[462]|DHJPAR0029114|Omiodes cuniculalis|657[0n]|BOLD:AAA6603
Chelonus gustavogutierrezi|[463]|DHJPAR0029084|Omiodes cuniculalis|657[0n]|BOLD:AAA6603
Chelonus gustavogutierrezi|[464]|DHJPAR0029101|Omiodes cuniculalis|657[0n]|BOLD:AAA6603
Chelonus gustavogutierrezi|[465]|DHJPAR0029081|Omiodes cuniculalis|657[0n]|BOLD:AAA6603
Chelonus gustavogutierrezi|[466]|DHJPAR0038007|Omiodes cuniculalis|658[0n]|BOLD:AAA6603
Chelonus gustavogutierrezi|[467]|DHJPAR0029118|Omiodes cuniculalis|657[0n]|BOLD:AAA6603
Chelonus gustavogutierrezi|[468]|DHJPAR0029116|Omiodes cuniculalis|657[0n]|BOLD:AAA6603
Chelonus gustavogutierrezi|[469]|DHJPAR0064017|Omiodes cuniculalis|658[0n]|BOLD:AAA6603
Chelonus gustavogutierrezi|[470]|DHJPAR0029127|Omiodes cuniculalis|657[0n]|BOLD:AAA6603
Chelonus gustavogutierrezi|[471]|DHJPAR0029086|Omiodes cuniculalis|657[0n]|BOLD:AAA6603
Chelonus gustavogutierrezi|[472]|DHJPAR0029096|Omiodes cuniculalis|657[0n]|BOLD:AAA6603
Chelonus gustavogutierrezi|[473]|DHJPAR0038003|Omiodes cuniculalis|658[0n]|BOLD:AAA6603
Chelonus gustavogutierrezi|[474]|DHJPAR0029132|Omiodes cuniculalis|657[0n]|BOLD:AAA6603
Chelonus gustavogutierrezi|[475]|DHJPAR0029100|Omiodes cuniculalis|657[0n]|BOLD:AAA6603
Chelonus gustavogutierrezi|[476]|DHJPAR0029109|Omiodes cuniculalis|657[0n]|BOLD:AAA6603
Chelonus gustavogutierrezi|[477]|DHJPAR0048882|Omiodes cuniculalis|658[0n]|BOLD:AAA6603
Chelonus gustavogutierrezi|[478]|DHJPAR0029104|Omiodes cuniculalis|657[0n]|BOLD:AAA6603
Chelonus gustavogutierrezi|[479]|DHJPAR0029087|Omiodes cuniculalis|657[0n]|BOLD:AAA6603
Chelonus gustavogutierrezi|[480]|DHJPAR0029128|Omiodes cuniculalis|657[0n]|BOLD:AAA6603
Chelonus gustavogutierrezi|[481]|DHJPAR0029108|Omiodes cuniculalis|657[0n]|BOLD:AAA6603
Chelonus gustavogutierrezi|[482]|DHJPAR0029095|Omiodes cuniculalis|657[0n]|BOLD:AAA6603
Chelonus gustavogutierrezi|[483]|DHJPAR0029123|Omiodes cuniculalis|657[0n]|BOLD:AAA6603
Chelonus gustavogutierrezi|[484]|DHJPAR0029078|Omiodes cuniculalis|657[0n]|BOLD:AAA6603
Chelonus gustavogutierrezi|[485]|DHJPAR0029103|Omiodes cuniculalis|657[0n]|BOLD:AAA6603
Chelonus gustavogutierrezi|[486]|DHJPAR0029115|Omiodes cuniculalis|657[0n]|BOLD:AAA6603
Chelonus gustavogutierrezi|[487]|DHJPAR0029131|Omiodes cuniculalis|657[0n]|BOLD:AAA6603
Chelonus michellevanderbankae|[488]|DHJPAR0029134|Anacrusis nephrodes|422[3n]|BOLD:AAT8838
Chelonus michellevanderbankae|[489]|DHJPAR0055415|Anacrusis nephrodes|583[5n]|BOLD:AAT8838
Chelonus michellevanderbankae|[490]|DHJPAR0040021|Anacrusis nephrodes|421[0n]|BOLD:AAT8838
Chelonus michellevanderbankae|[491]|DHJPAR0040018|Anacrusis nephrodes|421[0n]|BOLD:AAT8838

Chelonus michellevanderbankae|[489]|DHJPAR0055415|Anacrusis nephrodes|583[5n]|BOLD:AAT8838
Chelonus michellevanderbankae|[490]|DHJPAR0040021|Anacrusis nephrodes|421[0n]|BOLD:AAT8838
Chelonus michellevanderbankae|[491]|DHJPAR0040018|Anacrusis nephrodes|421[0n]|BOLD:AAT8838
Chelonus michellevanderbankae|[492]|DHJPAR0040406|Anacrusis nephrodes|645[4n]|BOLD:AAT8838
Chelonus michellevanderbankae|[493]|DHJPAR0029135|Anacrusis nephrodes|426[0n]|BOLD:AAT8838
Chelonus michellevanderbankae|[494]|DHJPAR0047069|Anacrusis nephrodes|612[2n]|BOLD:AAT8838
Chelonus michellevanderbankae|[495]|DHJPAR0038977|Anacrusis nephrodes|613[0n]|BOLD:AAT8838
Chelonus michellevanderbankae|[496]|DHJPAR0050964|Anacrusis nephrodes|629[0n]|BOLD:AAT8838
Chelonus michellevanderbankae|[497]|DHJPAR0049118|Anacrusis nephrodes|658[0n]|BOLD:AAT8838
Chelonus michellevanderbankae|[498]|DHJPAR0039447|Anacrusis nephrodes|620[1n]|BOLD:AAT8838
Chelonus nataliaivanovae|[499]|DHJPAR0045406|Anacrusis nephrodes|658[2n]|BOLD:AAF2632
Chelonus nataliaivanovae|[500]|DHJPAR0035559|Anacrusis nephrodes|658[0n]|BOLD:AAF2632
Chelonus nataliaivanovae|[501]|DHJPAR0035415|Omiodes humeralis|558[0n]|BOLD:AAF2632
Chelonus nataliaivanovae|[502]|DHJPAR0049124|Amorbia productana|658[0n]|BOLD:AAF2632
Chelonus nataliaivanovae|[503]|DHJPAR0040020|Antaeotricha ianthinaEPR01|658[0n]|BOLD:AAF2632
Chelonus nataliaivanovae|[504]|DHJPAR0053679|Megalota vulgaris|658[0n]|BOLD:AAF2632
Chelonus nataliaivanovae|[505]|DHJPAR0056713|Antaeotricha spurca|658[0n]|BOLD:AAF2632
Chelonus nataliaivanovae|[506]|DHJPAR0029133|Anacrusis nephrodes|657[0n]|BOLD:AAF2632
Chelonus nataliaivanovae|[507]|DHJPAR0042960|Antaeotricha Janzen140DHJ03|658[0n]|BOLD:AAF2632
Chelonus nataliaivanovae|[508]|DHJPAR0034268|Spilomela pantheralis|633[0n]|BOLD:AAF2632
Chelonus nataliaivanovae|[509]|DHJPAR0039111|Amorbia revolutana|658[0n]|BOLD:AAF2632
Chelonus nataliaivanovae|[510]|DHJPAR0052897|Megalota crassana|658[0n]|BOLD:AAF2632
Chelonus nataliaivanovae|[511]|DHJPAR0049786|Antaeotricha BioLep38|658[0n]|BOLD:AAF2632
Chelonus nataliaivanovae|[512]|DHJPAR0040019|Anacrusis nephrodes|658[0n]|BOLD:AAF2632
Chelonus nataliaivanovae|[513]|DHJPAR0055461|Strepsicrates Brown37|658[0n]|BOLD:AAF2632
Chelonus nataliaivanovae|[514]|DHJPAR0035316|Antaeotricha Janzen401|658[0n]|BOLD:AAF2632
Chelonus paulgoldsteini|[515]|DHJPAR0042855|elachBioLep01 BioLep754|598[0n]|BOLD:AAE9140
Chelonus paulgoldsteini|[516]|DHJPAR0048102|elachBioLep01 BioLep754|658[0n]|BOLD:AAE9140
Chelonus paulgoldsteini|[517]|DHJPAR0051928|elachBioLep01 BioLep754|658[0n]|BOLD:AAE9140
Chelonus paulgoldsteini|[518]|DHJPAR0051921|elachBioLep01 BioLep754|658[0n]|BOLD:AAE9140
Chelonus paulgoldsteini|[519]|DHJPAR0035527|Stenoma BioLep86|633[0n]|BOLD:AAE9140
Chelonus paulgoldsteini|[520]|DHJPAR0042851|elachBioLep01 BioLep754|633[0n]|BOLD:AAE9140
Chelonus paulgoldsteini|[521]|DHJPAR0039120|elachBioLep01 BioLep754|629[0n]|BOLD:AAE9140
Chelonus paulgoldsteini|[522]|DHJPAR0046940|elachBioLep01 BioLep754|606[0n]|BOLD:AAE9140
Chelonus paulgoldsteini|[523]|DHJPAR0056954|elachBioLep01 BioLep754|608[0n]|BOLD:AAE9140
Chelonus paulgoldsteini|[524]|DHJPAR0048980|elachBioLep01 BioLep754|608[0n]|BOLD:AAE9140
Chelonus paulgoldsteini|[525]|DHJPAR0035526|Stenoma BioLep86|622[0n]|BOLD:AAE9140
Chelonus paulgoldsteini|[526]|DHJPAR0051923|elachBioLep01 BioLep754|633[0n]|BOLD:AAE9140
Chelonus paulgoldsteini|[527]|DHJPAR0056976|elachBioLep01 BioLep754|632[0n]|BOLD:AAE9140
Chelonus paulgoldsteini|[528]|DHJPAR0035528|Stenoma BioLep86|632[0n]|BOLD:AAE9140
Chelonus pamelacastilloae|[529]|DHJPAR0045401|Antaeotricha BioLep91|658[0n]|BOLD:AAK1568
Chelonus paulhansoni|[530]|DHJPAR0061491|Antaeotricha Janzen204|658[0n]|BOLD:ACB1254
Chelonus paulhansoni|[531]|DHJPAR0051343|Antaeotricha Janzen204|658[0n]|BOLD:ACB1254
Chelonus paulhansoni|[532]|DHJPAR0048978|Antaeotricha Janzen204|658[0n]|BOLD:ACB1254
Chelonus paulhansoni|[533]|DHJPAR0049326|Antaeotricha Janzen204|658[0n]|BOLD:ACB1254
Chelonus petronariosae|[534]|DHJPAR0048976|Antaeotricha Janzen146|658[0n]|BOLD:ACB1255
Chelonus randallgarciai|[535]|DHJPAR0064504|Antaeotricha Janzen239|658[0n]|BOLD:ADR1783
Chelonus randallgarciai|[536]|DHJPAR0062693|Antaeotricha Janzen146|618[0n]|BOLD:ADR1783
Chelonus ramyamanjunathae|[537]|DHJPAR0045410|Antaeotricha Janzen321|658[2n]|BOLD:ABA9309
Chelonus ramyamanjunathae|[538]|DHJPAR0045412|Antaeotricha Janzen321|658[0n]|BOLD:ABA9309
Chelonus ramyamanjunathae|[539]|DHJPAR0055369|Antaeotricha Janzen146|658[0n]|BOLD:ABA9309
Chelonus ramyamanjunathae|[540]|DHJPAR0048975|Antaeotricha Janzen321|658[0n]|BOLD:ABA9309
Chelonus ramyamanjunathae|[541]|DHJPAR0048971|Antaeotricha Janzen321|658[0n]|BOLD:ABA9309
Chelonus ramyamanjunathae|[542]|DHJPAR0045411|Antaeotricha Janzen321|658[0n]|BOLD:ABA9309
Chelonus ramyamanjunathae|[543]|DHJPAR0045413|Antaeotricha Janzen321|658[1n]|BOLD:ABA9309
Chelonus ramyamanjunathae|[544]|DHJPAR0055366|Antaeotricha Janzen146|658[0n]|BOLD:ABA9309
Chelonus ramyamanjunathae|[545]|DHJPAR0064503|Antaeotricha Janzen239|658[0n]|BOLD:ABA9309
Chelonus ramyamanjunathae|[546]|DHJPAR0040016|Antaeotricha Janzen126|658[0n]|BOLD:ABA9309
Chelonus paulheberti|[547]|DHJPAR0048105|Stenoma BioLep86|658[0n]|BOLD:AAN2554
Chelonus paulheberti|[548]|DHJPAR0042863|elachBioLep01 BioLep754|658[0n]|BOLD:AAN2554
Chelonus paulheberti|[549]|DHJPAR0039503|elachBioLep01 BioLep55|658[0n]|BOLD:AAN2554
Chelonus paulheberti|[550]|DHJPAR0042854|Antaeotricha Janzen233|658[0n]|BOLD:AAN2554
Chelonus paulheberti|[551]|DHJPAR0042850|elachBioLep01 BioLep754|658[0n]|BOLD:AAN2554
Chelonus paulheberti|[552]|DHJPAR0048972|Antaeotricha Janzen233|627[0n]|BOLD:AAN2554
Chelonus paulheberti|[553]|DHJPAR0042861|Antaeotricha Janzen233|658[0n]|BOLD:AAN2554
Chelonus rebeccakittelae|[554]|DHJPAR0048982|Antaeotricha Janzen224|604[0n]|BOLD:ACB1121
Chelonus robertofernandezi|[555]|DHJPAR0052883|Antaeotricha Janzen110|658[0n]|BOLD:ACJ5332
Chelonus robertoespinozai|[556]|DHJPAR0029177|Ategumia lotanalis|657[0n]|BOLD:AAD4678
Chelonus robertoespinozai|[557]|DHJPAR0029174|Ategumia lotanalis|657[0n]|BOLD:AAD4678
Chelonus robertoespinozai|[558]|DHJPAR0064008|elachJanzen01 Janzen211|628[0n]|BOLD:AAD4678
Chelonus robertoespinozai|[559]|DHJPAR0046939|elachJanzen01 Janzen211|658[0n]|BOLD:AAD4678
Chelonus robertoespinozai|[560]|DHJPAR0064506|elachJanzen01 Janzen211|658[0n]|BOLD:AAD4678
Chelonus rocioecheverriae|[561]|DHJPAR0048970|Antaeotricha Janzen146|658[0n]|BOLD:ACB1122
Chelonus rodrigogamezi|[562]|DHJPAR0042097|Antaeotricha Janzen88|421[0n]|BOLD:AAJ0362
Chelonus rodrigogamezi|[563]|DHJPAR0056001|Antaeotricha Janzen88|670[0n]|BOLD:AAJ0362
Chelonus rodrigogamezi|[564]|DHJPAR0040360|Antaeotricha Janzen88|627[0n]|BOLD:AAJ0362
Chelonus rodrigogamezi|[565]|DHJPAR0055383|Antaeotricha Janzen88|658[0n]|BOLD:AAJ0362
Chelonus rodrigogamezi|[566]|DHJPAR0055375|Antaeotricha Janzen88|658[0n]|BOLD:AAJ0362
Chelonus rodrigogamezi|[567]|DHJPAR0055385|Antaeotricha Janzen88|658[0n]|BOLD:AAJ0362
Chelonus rodrigogamezi|[568]|DHJPAR0055359|Antaeotricha Janzen88|658[0n]|BOLD:AAJ0362
Chelonus rodrigogamezi|[569]|DHJPAR0064029|Antaeotricha Janzen88|636[0n]|BOLD:AAJ0362
Chelonus rodrigogamezi|[570]|DHJPAR0055374|Antaeotricha Janzen88|658[0n]|BOLD:AAJ0362
Chelonus rodrigogamezi|[571]|DHJPAR0050074|Antaeotricha Janzen88|658[0n]|BOLD:AAJ0362
Chelonus rodrigogamezi|[572]|DHJPAR0055378|Antaeotricha Janzen88|624[0n]|BOLD:AAJ0362
Chelonus rodrigogamezi|[573]|DHJPAR0056951|Antaeotricha Janzen88|661[0n]|BOLD:AAJ0362
Chelonus rodrigogamezi|[574]|DHJPAR0056953|Antaeotricha Janzen88|661[0n]|BOLD:AAJ0362
Chelonus rodrigogamezi|[575]|DHJPAR0054565|Antaeotricha Janzen88|658[0n]|BOLD:AAJ0362
Chelonus rodrigogamezi|[576]|DHJPAR0052884|Antaeotricha Janzen88|658[0n]|BOLD:AAJ0362
Chelonus rodrigogamezi|[577]|DHJPAR0056971|Antaeotricha Janzen88|658[0n]|BOLD:AAJ0362
Chelonus rodrigogamezi|[578]|DHJPAR0056956|Antaeotricha Janzen88|661[0n]|BOLD:AAJ0362
Chelonus rodrigogamezi|[579]|DHJPAR0048099|Antaeotricha Janzen88|658[0n]|BOLD:AAJ0362
Chelonus rodrigogamezi|[580]|DHJPAR0040363|Antaeotricha Janzen88|658[0n]|BOLD:AAJ0362
Chelonus rodrigogamezi|[581]|DHJPAR0040365|Antaeotricha Janzen88|658[0n]|BOLD:AAJ0362
Chelonus rodrigogamezi|[582]|DHJPAR0055361|Antaeotricha Janzen88|658[0n]|BOLD:AAJ0362
Chelonus rodrigogamezi|[583]|DHJPAR0055358|Antaeotricha Janzen88|658[0n]|BOLD:AAJ0362
Chelonus rodrigogamezi|[584]|DHJPAR0055394|Antaeotricha Janzen88|658[0n]|BOLD:AAJ0362
Chelonus rodrigogamezi|[585]|DHJPAR0040362|Antaeotricha Janzen88|618[0n]|BOLD:AAJ0362
Chelonus rodrigogamezi|[586]|DHJPAR0046937|Antaeotricha Janzen88|658[0n]|BOLD:AAJ0362
Chelonus rodrigogamezi|[587]|DHJPAR0056349|Antaeotricha Janzen88|661[0n]|BOLD:AAJ0362
Chelonus rodrigogamezi|[588]|DHJPAR0056352|Antaeotricha Janzen88|624[0n]|BOLD:AAJ0362
Chelonus rodrigogamezi|[589]|DHJPAR0029168|Antaeotricha Janzen88|657[0n]|BOLD:AAJ0362
[illegible]

Chelonus rodrigogamezi|[587]|DHJPAR0056349|Antaeotricha Janzen88|661[0n]|BOLD:AAJ0362
Chelonus rodrigogamezi|[588]|DHJPAR0056352|Antaeotricha Janzen88|624[0n]|BOLD:AAJ0362
Chelonus rodrigogamezi|[589]|DHJPAR0029168|Antaeotricha Janzen88|657[0n]|BOLD:AAJ0362
Chelonus rodrigogamezi|[590]|DHJPAR0055230|Antaeotricha Janzen88|658[0n]|BOLD:AAJ0362
Chelonus rodrigogamezi|[591]|DHJPAR0040361|Antaeotricha Janzen88|658[0n]|BOLD:AAJ0362
Chelonus rodrigogamezi|[592]|DHJPAR0062575|Antaeotricha Janzen88|658[0n]|BOLD:AAJ0362
Chelonus rodrigogamezi|[593]|DHJPAR0052885|Antaeotricha Janzen88|658[0n]|BOLD:AAJ0362
Chelonus rodrigogamezi|[594]|DHJPAR0063428|Antaeotricha Janzen88|658[0n]|BOLD:AAJ0362
Chelonus rodrigogamezi|[595]|DHJPAR0052888|Antaeotricha Janzen88|658[0n]|BOLD:AAJ0362
Chelonus rodrigogamezi|[596]|DHJPAR0056959|Antaeotricha Janzen88|658[0n]|BOLD:AAJ0362
Chelonus rodrigogamezi|[597]|DHJPAR0052893|Antaeotricha Janzen88|658[0n]|BOLD:AAJ0362
Chelonus rodrigogamezi|[598]|DHJPAR0055993|Antaeotricha Janzen88|670[0n]|BOLD:AAJ0362
Chelonus rodrigogamezi|[599]|DHJPAR0055362|Antaeotricha Janzen88|658[0n]|BOLD:AAJ0362
Chelonus rodrigogamezi|[600]|DHJPAR0051329|Antaeotricha Janzen88|658[0n]|BOLD:AAJ0362
Chelonus rodrigogamezi|[601]|DHJPAR0052892|Antaeotricha Janzen88|658[0n]|BOLD:AAJ0362
Chelonus rodrigogamezi|[602]|DHJPAR0055994|Antaeotricha Janzen88|670[0n]|BOLD:AAJ0362
Chelonus rodrigogamezi|[603]|DHJPAR0055999|Antaeotricha Janzen88|670[0n]|BOLD:AAJ0362
Chelonus rodrigogamezi|[604]|DHJPAR0040370|Antaeotricha Janzen88|658[0n]|BOLD:AAJ0362
Chelonus rodrigogamezi|[605]|DHJPAR0052895|Antaeotricha Janzen88|658[0n]|BOLD:AAJ0362
Chelonus rodrigogamezi|[606]|DHJPAR0040359|Antaeotricha Janzen88|658[0n]|BOLD:AAJ0362
Chelonus rodrigogamezi|[607]|DHJPAR0052880|Antaeotricha Janzen88|658[0n]|BOLD:AAJ0362
Chelonus rodrigogamezi|[608]|DHJPAR0048098|Antaeotricha Janzen88|658[0n]|BOLD:AAJ0362
Chelonus rodrigogamezi|[609]|DHJPAR0050121|Antaeotricha Janzen88|658[0n]|BOLD:AAJ0362
Chelonus rodrigogamezi|[610]|DHJPAR0051341|Antaeotricha Janzen88|658[0n]|BOLD:AAJ0362
Chelonus rodrigogamezi|[611]|DHJPAR0051335|Antaeotricha Janzen88|658[0n]|BOLD:AAJ0362
Chelonus rodrigogamezi|[612]|DHJPAR0053675|Antaeotricha Janzen88|658[0n]|BOLD:AAJ0362
Chelonus rodrigogamezi|[613]|DHJPAR0052894|Antaeotricha Janzen88|613[0n]|BOLD:AAJ0362
Chelonus rodrigogamezi|[614]|DHJPAR0053680|Antaeotricha Janzen88|605[0n]|BOLD:AAJ0362
Chelonus rodrigogamezi|[615]|DHJPAR0054558|Antaeotricha Janzen88|658[0n]|BOLD:AAJ0362
Chelonus rodrigogamezi|[616]|DHJPAR0054562|Antaeotricha Janzen88|658[0n]|BOLD:AAJ0362
Chelonus rodrigogamezi|[617]|DHJPAR0054554|Antaeotricha Janzen88|658[0n]|BOLD:AAJ0362
Chelonus rodrigogamezi|[618]|DHJPAR0055401|Antaeotricha Janzen88|658[0n]|BOLD:AAJ0362
Chelonus rodrigogamezi|[619]|DHJPAR0062574|Antaeotricha Janzen88|658[0n]|BOLD:AAJ0362
Chelonus ronaldzunigai|[620]|DHJPAR0037167|Antaeotricha Janzen134|624[0n]|BOLD:AAK1016
Chelonus rosibelelizondoae|[621]|DHJPAR0051334|Antaeotricha renselariana|658[1n]|BOLD:AAM1446
Chelonus rosibelelizondoae|[622]|DHJPAR0038004|Antaeotricha Janzen146|658[0n]|BOLD:AAM1446
Chelonus rostermoragai|[623]|DHJPAR0056000|Antaeotricha stigmatiasDHJ01|670[0n]|BOLD:ACJ3551
Chelonus rostermoragai|[624]|DHJPAR0053673|Antaeotricha stigmatiasDHJ01|657[0n]|BOLD:ACJ3551
Chelonus rostermoragai|[625]|DHJPAR0053685|Antaeotricha stigmatiasDHJ01|657[0n]|BOLD:ACJ3551
Chelonus rostermoragai|[626]|DHJPAR0051926|Antaeotricha stigmatiasDHJ01|658[0n]|BOLD:ACJ3551
Chelonus rostermoragai|[627]|DHJPAR0051922|Antaeotricha stigmatiasDHJ01|658[0n]|BOLD:ACJ3551
Chelonus rostermoragai|[628]|DHJPAR0051925|Antaeotricha stigmatiasDHJ01|658[0n]|BOLD:ACJ3551
Chelonus ruthfrancoae|[629]|DHJPAR0056354|Antaeotricha BioLep46|661[0n]|BOLD:AAW4589
Chelonus ruthfrancoae|[630]|DHJPAR0051344|Antaeotricha similisEPR03|658[0n]|BOLD:AAW4589
Chelonus ruthfrancoae|[631]|DHJPAR0051094|Antaeotricha similisEPR02|658[0n]|BOLD:AAW4589
Chelonus ruthfrancoae|[632]|DHJPAR0050136|Cerconota Janzen82|658[0n]|BOLD:AAW4589
Chelonus ruthfrancoae|[633]|DHJPAR0055363|Antaeotricha Janzen13|658[0n]|BOLD:AAW4589
Chelonus ruthfrancoae|[634]|DHJPAR0056353|Antaeotricha BioLep46|634[0n]|BOLD:AAW4589
Chelonus ruthfrancoae|[635]|DHJPAR0056965|Antaeotricha BioLep46|632[1n]|BOLD:AAW4589
Chelonus ruthfrancoae|[636]|DHJPAR0056962|Antaeotricha Janzen31|658[0n]|BOLD:AAW4589
Chelonus ruthfrancoae|[637]|DHJPAR0042089|Stenoma Janzen09|658[0n]|BOLD:AAW4589
Chelonus ruthfrancoae|[638]|DHJPAR0042091|Stenoma Janzen09|658[0n]|BOLD:AAW4589
Chelonus ruthfrancoae|[639]|DHJPAR0056950|Antaeotricha BioLep46|661[0n]|BOLD:AAW4589
Chelonus ruthfrancoae|[640]|DHJPAR0055399|Antaeotricha Janzen290|658[0n]|BOLD:AAW4589
Chelonus scottmilleri|[641]|DHJPAR0061492|Antaeotricha radicalisEPR03|658[0n]|BOLD:ABY5286
Chelonus scottmilleri|[642]|DHJPAR0051345|Antaeotricha radicalisEPR03|658[0n]|BOLD:ABY5286
Chelonus scottmilleri|[643]|DHJPAR0055384|Antaeotricha Janzen204|658[0n]|BOLD:ABY5286
Chelonus scottmilleri|[644]|DHJPAR0051325|Antaeotricha Janzen04|658[0n]|BOLD:ABY5286
Chelonus scottmilleri|[645]|DHJPAR0040369|Antaeotricha radicalisEPR03|658[0n]|BOLD:ABY5286
Chelonus scottmilleri|[646]|DHJPAR0055388|Antaeotricha radicalisEPR03|658[0n]|BOLD:ABY5286
Chelonus scottmilleri|[647]|DHJPAR0051339|Antaeotricha radicalisEPR03|658[0n]|BOLD:ABY5286
Chelonus scottmilleri|[648]|DHJPAR0048096|Antaeotricha radicalisEPR02|658[0n]|BOLD:ABY5286
Chelonus scottmilleri|[649]|DHJPAR0048095|Antaeotricha radicalisEPR02|658[0n]|BOLD:ABY5286
Chelonus scottmilleri|[650]|DHJPAR0064505|elachJanzen01 Janzen211|658[0n]|BOLD:ABY5286
Chelonus scottmilleri|[651]|DHJPAR0055389|Antaeotricha radicalisEPR03|658[0n]|BOLD:ABY5286
Chelonus scottmilleri|[652]|DHJPAR0035310|Antaeotricha Janzen04|658[0n]|BOLD:ABY5286
Chelonus scottmilleri|[653]|DHJPAR0053670|Antaeotricha marmorea|657[0n]|BOLD:ABY5286
Chelonus scottmilleri|[654]|DHJPAR0049934|elachJanzen01 Janzen131|326[0n]
Chelonus scottmilleri|[655]|DHJPAR0039122|Antaeotricha marmorea|632[0n]|BOLD:ABY5286
Chelonus scottmilleri|[656]|DHJPAR0045285|Antaeotricha radicalisEPR03|658[2n]|BOLD:ABY5286
Chelonus scottshawi|[657]|DHJPAR0062573|Antaeotricha Janzen146|658[0n]|BOLD:ABX5499
Chelonus scottshawi|[658]|DHJPAR0055380|Antaeotricha Janzen146|658[0n]|BOLD:ABX5499
Chelonus scottshawi|[659]|DHJPAR0037992|Antaeotricha renselariana|658[0n]|BOLD:ABX5499
Chelonus scottshawi|[660]|DHJPAR0037988|Antaeotricha Janzen146|658[0n]|BOLD:ABX5499
Chelonus scottshawi|[661]|DHJPAR0055395|Antaeotricha Janzen146|658[0n]|BOLD:ABX5499
Chelonus scottshawi|[662]|DHJPAR0051924|Antaeotricha Janzen146|658[2n]|BOLD:ABX5499
Chelonus scottshawi|[663]|DHJPAR0055392|Antaeotricha Janzen146|658[0n]|BOLD:ABX5499
Chelonus scottshawi|[664]|DHJPAR0037995|Antaeotricha renselariana|658[0n]|BOLD:ABX5499
Chelonus scottshawi|[665]|DHJPAR0052182|Antaeotricha Janzen49|658[0n]|BOLD:ABX5499
Chelonus scottshawi|[666]|DHJPAR0052185|Antaeotricha Janzen49|658[0n]|BOLD:ABX5499
Chelonus scottshawi|[667]|DHJPAR0042853|Antaeotricha Janzen146|658[0n]|BOLD:ABX5499
Chelonus scottshawi|[668]|DHJPAR0052187|Antaeotricha Janzen49|658[0n]|BOLD:ABX5499
Chelonus scottshawi|[669]|DHJPAR0062697|Antaeotricha Janzen40|621[0n]|BOLD:ABX5499
Chelonus scottshawi|[670]|DHJPAR0052280|Antaeotricha Janzen24|658[0n]|BOLD:ABX5499
Chelonus scottshawi|[671]|DHJPAR0052183|Antaeotricha Janzen24|658[0n]|BOLD:ABX5499
Chelonus scottshawi|[672]|DHJPAR0052186|Antaeotricha Janzen24|658[0n]|BOLD:ABX5499
Chelonus scottshawi|[673]|DHJPAR0049018|Antaeotricha Janzen24|658[0n]|BOLD:ABX5499
Chelonus scottshawi|[674]|DHJPAR0055372|Antaeotricha Janzen24|658[0n]|BOLD:ABX5499
Chelonus scottshawi|[675]|DHJPAR0055379|Antaeotricha Janzen24|658[0n]|BOLD:ABX5499
Chelonus scottshawi|[676]|DHJPAR0055393|Antaeotricha Janzen24|658[0n]|BOLD:ABX5499
Chelonus scottshawi|[677]|DHJPAR0056955|Antaeotricha Janzen24|661[0n]|BOLD:ABX5499
Chelonus scottshawi|[678]|DHJPAR0036384|Antaeotricha Janzen24|658[0n]|BOLD:ABX5499
Chelonus scottshawi|[679]|DHJPAR0055365|Antaeotricha Janzen24|658[0n]|BOLD:ABX5499
Chelonus scottshawi|[680]|DHJPAR0055386|Antaeotricha Janzen24|658[0n]|BOLD:ABX5499
Chelonus scottshawi|[681]|DHJPAR0049007|Antaeotricha Janzen24|658[0n]|BOLD:ABX5499
Chelonus scottshawi|[682]|DHJPAR0055991|Antaeotricha Janzen24|670[0n]|BOLD:ABX5499
Chelonus scottshawi|[683]|DHJPAR0048981|Antaeotricha Janzen245|658[0n]|BOLD:ABX5499
Chelonus scottshawi|[684]|DHJPAR0055391|Antaeotricha Janzen24|658[0n]|BOLD:ABX5499
Chelonus scottshawi|[685]|DHJPAR0055396|Antaeotricha Janzen24|658[0n]|BOLD:ABX5499
Chelonus scottshawi|[686]|DHJPAR0036386|Antaeotricha Janzen24|658[0n]|BOLD:ABX5499
Chelonus scottshawi|[687]|DHJPAR0055387|Antaeotricha Janzen24|658[0n]|BOLD:ABX5499
Chelonus scottshawi|[688]|[illegible]

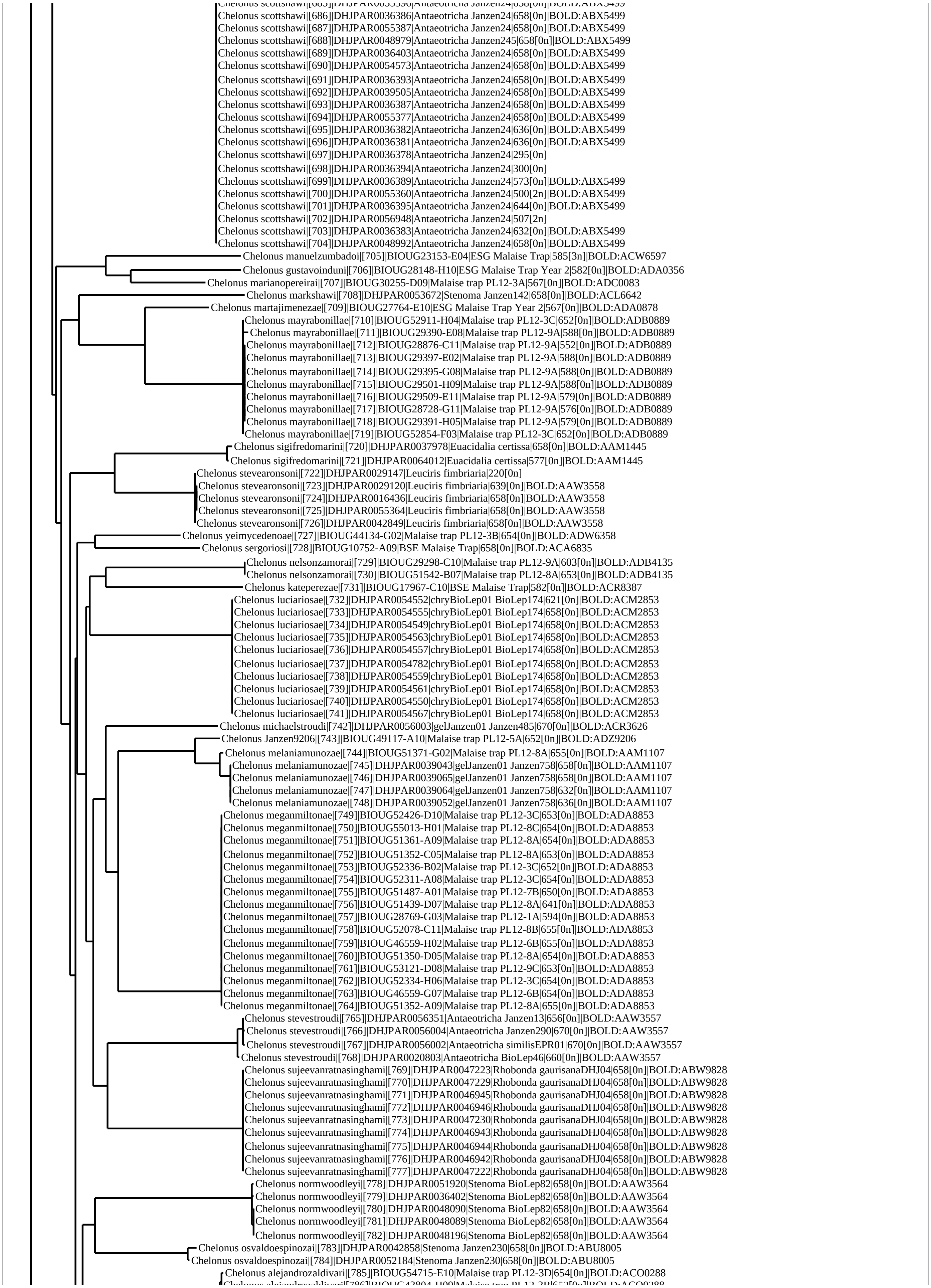
Chelonus scottshawi|[685]|DHJPAR0055396|Antaeotricha Janzen24|658[0n]|BOLD:ABX5499
Chelonus scottshawi|[686]|DHJPAR0036386|Antaeotricha Janzen24|658[0n]|BOLD:ABX5499
Chelonus scottshawi|[687]|DHJPAR0055387|Antaeotricha Janzen24|658[0n]|BOLD:ABX5499
Chelonus scottshawi|[688]|DHJPAR0048979|Antaeotricha Janzen245|658[0n]|BOLD:ABX5499
Chelonus scottshawi|[689]|DHJPAR0036403|Antaeotricha Janzen24|658[0n]|BOLD:ABX5499
Chelonus scottshawi|[690]|DHJPAR0054573|Antaeotricha Janzen24|658[0n]|BOLD:ABX5499
Chelonus scottshawi|[691]|DHJPAR0036393|Antaeotricha Janzen24|658[0n]|BOLD:ABX5499
Chelonus scottshawi|[692]|DHJPAR0039505|Antaeotricha Janzen24|658[0n]|BOLD:ABX5499
Chelonus scottshawi|[693]|DHJPAR0036387|Antaeotricha Janzen24|658[0n]|BOLD:ABX5499
Chelonus scottshawi|[694]|DHJPAR0055377|Antaeotricha Janzen24|658[0n]|BOLD:ABX5499
Chelonus scottshawi|[695]|DHJPAR0036382|Antaeotricha Janzen24|636[0n]|BOLD:ABX5499
Chelonus scottshawi|[696]|DHJPAR0036381|Antaeotricha Janzen24|636[0n]|BOLD:ABX5499
Chelonus scottshawi|[697]|DHJPAR0036378|Antaeotricha Janzen24|295[0n]
Chelonus scottshawi|[698]|DHJPAR0036394|Antaeotricha Janzen24|300[0n]
Chelonus scottshawi|[699]|DHJPAR0036389|Antaeotricha Janzen24|573[0n]|BOLD:ABX5499
Chelonus scottshawi|[700]|DHJPAR0055360|Antaeotricha Janzen24|500[2n]|BOLD:ABX5499
Chelonus scottshawi|[701]|DHJPAR0036395|Antaeotricha Janzen24|644[0n]|BOLD:ABX5499
Chelonus scottshawi|[702]|DHJPAR0056948|Antaeotricha Janzen24|507[2n]
Chelonus scottshawi|[703]|DHJPAR0036383|Antaeotricha Janzen24|632[0n]|BOLD:ABX5499
Chelonus scottshawi|[704]|DHJPAR0048992|Antaeotricha Janzen24|658[0n]|BOLD:ABX5499
Chelonus manuelzumbadoi|[705]|BIOUG23153-E04|ESG Malaise Trap|585[3n]|BOLD:ACW6597
Chelonus gustavoinduni|[706]|BIOUG28148-H10|ESG Malaise Trap Year 2|582[0n]|BOLD:ADA0356
Chelonus marianopereirai|[707]|BIOUG30255-D09|Malaise trap PL12-3A|567[0n]|BOLD:ADC0083
Chelonus markshawi|[708]|DHJPAR0053672|Stenoma Janzen142|658[0n]|BOLD:ACL6642
Chelonus martajimenezae|[709]|BIOUG27764-E10|ESG Malaise Trap Year 2|567[0n]|BOLD:ADA0878
Chelonus mayrabonillae|[710]|BIOUG52911-H04|Malaise trap PL12-3C|652[0n]|BOLD:ADB0889
Chelonus mayrabonillae|[711]|BIOUG29390-E08|Malaise trap PL12-9A|588[0n]|BOLD:ADB0889
Chelonus mayrabonillae|[712]|BIOUG28876-C11|Malaise trap PL12-9A|552[0n]|BOLD:ADB0889
Chelonus mayrabonillae|[713]|BIOUG29397-E02|Malaise trap PL12-9A|588[0n]|BOLD:ADB0889
Chelonus mayrabonillae|[714]|BIOUG29395-G08|Malaise trap PL12-9A|588[0n]|BOLD:ADB0889
Chelonus mayrabonillae|[715]|BIOUG29501-H09|Malaise trap PL12-9A|588[0n]|BOLD:ADB0889
Chelonus mayrabonillae|[716]|BIOUG29509-E11|Malaise trap PL12-9A|579[0n]|BOLD:ADB0889
Chelonus mayrabonillae|[717]|BIOUG28728-G11|Malaise trap PL12-9A|576[0n]|BOLD:ADB0889
Chelonus mayrabonillae|[718]|BIOUG29391-H05|Malaise trap PL12-9A|579[0n]|BOLD:ADB0889
Chelonus mayrabonillae|[719]|BIOUG52854-F03|Malaise trap PL12-3C|652[0n]|BOLD:ADB0889
Chelonus sigifredomarini|[720]|DHJPAR0037978|Euacidalia certissa|658[0n]|BOLD:AAM1445
Chelonus sigifredomarini|[721]|DHJPAR0064012|Euacidalia certissa|577[0n]|BOLD:AAM1445
Chelonus stevearonsoni|[722]|DHJPAR0029147|Leuciris fimbriaria|220[0n]
Chelonus stevearonsoni|[723]|DHJPAR0029120|Leuciris fimbriaria|639[0n]|BOLD:AAW3558
Chelonus stevearonsoni|[724]|DHJPAR0016436|Leuciris fimbriaria|658[0n]|BOLD:AAW3558
Chelonus stevearonsoni|[725]|DHJPAR0055364|Leuciris fimbriaria|658[0n]|BOLD:AAW3558
Chelonus stevearonsoni|[726]|DHJPAR0042849|Leuciris fimbriaria|658[0n]|BOLD:AAW3558
Chelonus yeimycedenoae|[727]|BIOUG44134-G02|Malaise trap PL12-3B|654[0n]|BOLD:ADW6358
Chelonus sergoriosi|[728]|BIOUG10752-A09|BSE Malaise Trap|658[0n]|BOLD:ACA6835
Chelonus nelsonzamorai|[729]|BIOUG29298-C10|Malaise trap PL12-9A|603[0n]|BOLD:ADB4135
Chelonus nelsonzamorai|[730]|BIOUG51542-B07|Malaise trap PL12-8A|653[0n]|BOLD:ADB4135
Chelonus kateperezae|[731]|BIOUG17967-C10|BSE Malaise Trap|582[0n]|BOLD:ACR8387
Chelonus luciariosae|[732]|DHJPAR0054552|chryBioLep01 BioLep174|621[0n]|BOLD:ACM2853
Chelonus luciariosae|[733]|DHJPAR0054555|chryBioLep01 BioLep174|658[0n]|BOLD:ACM2853
Chelonus luciariosae|[734]|DHJPAR0054549|chryBioLep01 BioLep174|658[0n]|BOLD:ACM2853
Chelonus luciariosae|[735]|DHJPAR0054563|chryBioLep01 BioLep174|658[0n]|BOLD:ACM2853
Chelonus luciariosae|[736]|DHJPAR0054557|chryBioLep01 BioLep174|658[0n]|BOLD:ACM2853
Chelonus luciariosae|[737]|DHJPAR0054782|chryBioLep01 BioLep174|658[0n]|BOLD:ACM2853
Chelonus luciariosae|[738]|DHJPAR0054559|chryBioLep01 BioLep174|658[0n]|BOLD:ACM2853
Chelonus luciariosae|[739]|DHJPAR0054561|chryBioLep01 BioLep174|658[0n]|BOLD:ACM2853
Chelonus luciariosae|[740]|DHJPAR0054550|chryBioLep01 BioLep174|658[0n]|BOLD:ACM2853
Chelonus luciariosae|[741]|DHJPAR0054567|chryBioLep01 BioLep174|658[0n]|BOLD:ACM2853
Chelonus michaelstroudi|[742]|DHJPAR0056003|gelJanzen01 Janzen485|670[0n]|BOLD:ACR3626
Chelonus Janzen9206|[743]|BIOUG49117-A10|Malaise trap PL12-5A|652[0n]|BOLD:ADZ9206
Chelonus melaniamunozae|[744]|BIOUG51371-G02|Malaise trap PL12-8A|655[0n]|BOLD:AAM1107
Chelonus melaniamunozae|[745]|DHJPAR0039043|gelJanzen01 Janzen758|658[0n]|BOLD:AAM1107
Chelonus melaniamunozae|[746]|DHJPAR0039065|gelJanzen01 Janzen758|658[0n]|BOLD:AAM1107
Chelonus melaniamunozae|[747]|DHJPAR0039064|gelJanzen01 Janzen758|632[0n]|BOLD:AAM1107
Chelonus melaniamunozae|[748]|DHJPAR0039052|gelJanzen01 Janzen758|636[0n]|BOLD:AAM1107
Chelonus meganmiltonae|[749]|BIOUG52426-D10|Malaise trap PL12-3C|653[0n]|BOLD:ADA8853
Chelonus meganmiltonae|[750]|BIOUG55013-H01|Malaise trap PL12-8C|654[0n]|BOLD:ADA8853
Chelonus meganmiltonae|[751]|BIOUG51361-A09|Malaise trap PL12-8A|654[0n]|BOLD:ADA8853
Chelonus meganmiltonae|[752]|BIOUG51352-C05|Malaise trap PL12-8A|653[0n]|BOLD:ADA8853
Chelonus meganmiltonae|[753]|BIOUG52336-B02|Malaise trap PL12-3C|652[0n]|BOLD:ADA8853
Chelonus meganmiltonae|[754]|BIOUG52311-A08|Malaise trap PL12-3C|654[0n]|BOLD:ADA8853
Chelonus meganmiltonae|[755]|BIOUG51487-A01|Malaise trap PL12-7B|650[0n]|BOLD:ADA8853
Chelonus meganmiltonae|[756]|BIOUG51439-D07|Malaise trap PL12-8A|641[0n]|BOLD:ADA8853
Chelonus meganmiltonae|[757]|BIOUG28769-G03|Malaise trap PL12-1A|594[0n]|BOLD:ADA8853
Chelonus meganmiltonae|[758]|BIOUG52078-C11|Malaise trap PL12-8B|655[0n]|BOLD:ADA8853
Chelonus meganmiltonae|[759]|BIOUG46559-H02|Malaise trap PL12-6B|655[0n]|BOLD:ADA8853
Chelonus meganmiltonae|[760]|BIOUG51350-D05|Malaise trap PL12-8A|654[0n]|BOLD:ADA8853
Chelonus meganmiltonae|[761]|BIOUG53121-D08|Malaise trap PL12-9C|653[0n]|BOLD:ADA8853
Chelonus meganmiltonae|[762]|BIOUG52334-H06|Malaise trap PL12-3C|654[0n]|BOLD:ADA8853
Chelonus meganmiltonae|[763]|BIOUG46559-G07|Malaise trap PL12-6B|654[0n]|BOLD:ADA8853
Chelonus meganmiltonae|[764]|BIOUG51352-A09|Malaise trap PL12-8A|655[0n]|BOLD:ADA8853
Chelonus stevestroudi|[765]|DHJPAR0056351|Antaeotricha Janzen13|656[0n]|BOLD:AAW3557
Chelonus stevestroudi|[766]|DHJPAR0056004|Antaeotricha Janzen290|670[0n]|BOLD:AAW3557
Chelonus stevestroudi|[767]|DHJPAR0056002|Antaeotricha similisEPR01|670[0n]|BOLD:AAW3557
Chelonus stevestroudi|[768]|DHJPAR0020803|Antaeotricha BioLep46|660[0n]|BOLD:AAW3557
Chelonus sujeevanratnasinghami|[769]|DHJPAR0047223|Rhobonda gaurisanaDHJ04|658[0n]|BOLD:ABW9828
Chelonus sujeevanratnasinghami|[770]|DHJPAR0047229|Rhobonda gaurisanaDHJ04|658[0n]|BOLD:ABW9828
Chelonus sujeevanratnasinghami|[771]|DHJPAR0046945|Rhobonda gaurisanaDHJ04|658[0n]|BOLD:ABW9828
Chelonus sujeevanratnasinghami|[772]|DHJPAR0046946|Rhobonda gaurisanaDHJ04|658[0n]|BOLD:ABW9828
Chelonus sujeevanratnasinghami|[773]|DHJPAR0047230|Rhobonda gaurisanaDHJ04|658[0n]|BOLD:ABW9828
Chelonus sujeevanratnasinghami|[774]|DHJPAR0046943|Rhobonda gaurisanaDHJ04|658[0n]|BOLD:ABW9828
Chelonus sujeevanratnasinghami|[775]|DHJPAR0046944|Rhobonda gaurisanaDHJ04|658[0n]|BOLD:ABW9828
Chelonus sujeevanratnasinghami|[776]|DHJPAR0046942|Rhobonda gaurisanaDHJ04|658[0n]|BOLD:ABW9828
Chelonus sujeevanratnasinghami|[777]|DHJPAR0047222|Rhobonda gaurisanaDHJ04|658[0n]|BOLD:ABW9828
Chelonus normwoodleyi|[778]|DHJPAR0051920|Stenoma BioLep82|658[0n]|BOLD:AAW3564
Chelonus normwoodleyi|[779]|DHJPAR0036402|Stenoma BioLep82|658[0n]|BOLD:AAW3564
Chelonus normwoodleyi|[780]|DHJPAR0048090|Stenoma BioLep82|658[0n]|BOLD:AAW3564
Chelonus normwoodleyi|[781]|DHJPAR0048089|Stenoma BioLep82|658[0n]|BOLD:AAW3564
Chelonus normwoodleyi|[782]|DHJPAR0048196|Stenoma BioLep82|658[0n]|BOLD:AAW3564
Chelonus osvaldoespinozai|[783]|DHJPAR0042858|Stenoma Janzen230|658[0n]|BOLD:ABU8005
Chelonus osvaldoespinozai|[784]|DHJPAR0052184|Stenoma Janzen230|658[0n]|BOLD:ABU8005
Chelonus alejandrozaldivari|[785]|BIOUG54715-E10|Malaise trap PL12-3D|654[0n]|BOLD:ACO0288
Chelonus alejandrozaldivari|[786]|BIOUG43804-H09|Malaise trap PL12-3B|652[0n]|BOLD:ACO0288

Chelonus osvaldoespinozai|[783]|[illegible]|Stenoma Janzen230|658[0n]|BOLD:ABU8005
Chelonus osvaldoespinozai|[784]|DHJPAR0052184|Stenoma Janzen230|658[0n]|BOLD:ABU8005
Chelonus alejandrozaldivari|[785]|BIOUG54715-E10|Malaise trap PL12-3D|654[0n]|BOLD:ACO0288
Chelonus alejandrozaldivari|[786]|BIOUG43804-H09|Malaise trap PL12-3B|652[0n]|BOLD:ACO0288
Chelonus alejandrozaldivari|[787]|BIOUG44551-E11|Malaise trap PL12-3B|654[0n]|BOLD:ACO0288
Chelonus alejandrozaldivari|[788]|BIOUG44692-B02|Malaise trap PL12-3B|654[0n]|BOLD:ACO0288
Chelonus motohasegawai|[789]|DHJPAR0030992|Ardeutica Brown002|657[0n]|BOLD:AAW3555
Chelonus motohasegawai|[790]|DHJPAR0042920|Anacrusis ellensatterleeae|658[0n]|BOLD:AAW3555
Chelonus mingfangi|[791]|BIOUG28344-E03|ESG Malaise Trap|594[0n]|BOLD:ADA5459
Chelonus minorcarmonai|[792]|BIOUG29282-H09|Malaise trap PL12-3A|480[4n]|BOLD:ADB0320
Chelonus minorcarmonai|[793]|BIOUG55555-E12|Malaise trap PL12-7D|655[0n]|BOLD:ADB0320
Chelonus minorcarmonai|[794]|BIOUG44207-G08|Malaise trap PL12-3B|654[0n]|BOLD:ADB0320
Chelonus minorcarmonai|[795]|BIOUG29112-C06|Malaise trap PL12-3A|612[0n]|BOLD:ADB0320
Chelonus minorcarmonai|[796]|BIOUG29415-F01|Malaise trap PL12-3A|594[0n]|BOLD:ADB0320
Chelonus minorcarmonai|[797]|BIOUG29112-F08|Malaise trap PL12-3A|564[0n]|BOLD:ADB0320
Chelonus minorcarmonai|[798]|BIOUG28697-B07|Malaise trap PL12-3A|594[0n]|BOLD:ADB0320
Chelonus monikaspringerae|[799]|BIOUG19587-A04|ESG Malaise Trap|594[0n]|BOLD:ACU3782
Chelonus moniquegilbertae|[800]|DHJPAR0020801|Omiodes humeralis|648[0n]|BOLD:AAJ0370
Chelonus moniquegilbertae|[801]|DHJPAR0042517|elachJanzen01 Janzen38|621[0n]|BOLD:AAJ0370
Chelonus moniquegilbertae|[802]|DHJPAR0040022|Antaeotricha spurca|658[0n]|BOLD:AAJ0370
Chelonus moniquegilbertae|[803]|DHJPAR0051323|elachJanzen01 Janzen235|658[0n]|BOLD:AAJ0370
Chelonus moniquegilbertae|[804]|DHJPAR0020805|Omiodes humeralis|617[3n]|BOLD:AAJ0370
Adelius Malaise2086|[805]|BIOUG50557-F04|Malaise trap PL12-6B|654[0n]|BOLD:AEB2086
Adelius gauldi|[806]|BIOUG07619-D09|BSE Malaise Trap|661[0n]|BOLD:ACJ2315
Adelius gauldi|[807]|BIOUG18398-G03|BSE Malaise Trap|624[0n]|BOLD:ACJ2315
Adelius adrianguadamuzi|[808]|BIOUG31661-B07|Derrumbe Malaise Trap|588[0n]|BOLD:ADF7277
Adelius janzeni|[809]|BIOUG07614-F11|BSE Malaise Trap|566[0n]|BOLD:ACJ2270
Ascogaster Malaise9605|[810]|DHJPAR0048332|malaise trap|658[0n]|BOLD:ABZ9605
Leptodrepana erasmocoronadoi|[811]|BIOUG28344-E11|ESG Malaise Trap|594[0n]|BOLD:ADA3192
Leptodrepana gilbertfuentesi|[812]|BIOUG05181-B07|BSE Malaise Trap|664[0n]|BOLD:ACF7385
Leptodrepana felipechavarriai|[813]|BIOUG44780-D08|Malaise trap PL12-1B|653[0n]|BOLD:ADB5898
Leptodrepana felipechavarriai|[814]|BIOUG54514-B07|Malaise trap PL12-5C|652[0n]|BOLD:ADB5898
Leptodrepana felipechavarriai|[815]|BIOUG54514-F06|Malaise trap PL12-5C|657[0n]|BOLD:ADB5898
Leptodrepana felipechavarriai|[816]|BIOUG49590-H06|Malaise trap PL12-1B|640[0n]|BOLD:ADB5898
Leptodrepana felipechavarriai|[817]|BIOUG29764-B11|Malaise trap PL12-3A|585[0n]|BOLD:ADB5898
Leptodrepana felipechavarriai|[818]|BIOUG49337-D07|Malaise trap PL12-5A|644[0n]|BOLD:ADB5898
Leptodrepana manuelriosi|[819]|BIOUG18005-B12|BSE Malaise Trap|561[0n]|BOLD:ACR7934
Leptodrepana alexisae|[820]|DHJPAR0047288|malaise trap|617[0n]|BOLD:ABV9918
Leptodrepana alexisae|[821]|DHJPAR0047295|malaise trap|658[0n]|BOLD:ABV9918
Leptodrepana alexisae|[822]|DHJPAR0047309|malaise trap|658[0n]|BOLD:ABV9918
Leptodrepana alexisae|[823]|DHJPAR0045916|malaise trap|658[0n]|BOLD:ABV9918
Leptodrepana alexisae|[824]|DHJPAR0046029|malaise trap|658[0n]|BOLD:ABV9918
Leptodrepana alexisae|[825]|DHJPAR0047332|malaise trap|658[0n]|BOLD:ABV9918
Leptodrepana alexisae|[826]|DHJPAR0047325|malaise trap|658[0n]|BOLD:ABV9918
Leptodrepana alexisae|[827]|DHJPAR0047301|malaise trap|658[0n]|BOLD:ABV9918
Leptodrepana alexisae|[828]|DHJPAR0047319|malaise trap|658[0n]|BOLD:ABV9918
Leptodrepana alexisae|[829]|DHJPAR0047294|malaise trap|658[0n]|BOLD:ABV9918
Leptodrepana alexisae|[830]|DHJPAR0046006|malaise trap|658[0n]|BOLD:ABV9918
Leptodrepana alexisae|[831]|DHJPAR0046015|malaise trap|658[0n]|BOLD:ABV9918
Leptodrepana alexisae|[832]|DHJPAR0045947|malaise trap|658[0n]|BOLD:ABV9918
Leptodrepana alexisae|[833]|DHJPAR0046011|malaise trap|658[0n]|BOLD:ABV9918
Leptodrepana alexisae|[834]|BIOUG33274-F09|Derrumbe Malaise Trap|601[0n]|BOLD:ABV9918
Leptodrepana alexisae|[835]|BIOUG32997-E10|Derrumbe Malaise Trap|604[0n]|BOLD:ABV9918
Leptodrepana alexisae|[836]|DHJPAR0047323|malaise trap|642[0n]|BOLD:ABV9918
Leptodrepana alexisae|[837]|DHJPAR0047327|malaise trap|642[0n]|BOLD:ABV9918
Leptodrepana alexisae|[838]|DHJPAR0046014|malaise trap|637[0n]|BOLD:ABV9918
Leptodrepana alexisae|[839]|BIOUG33411-B08|Derrumbe Malaise Trap|594[0n]|BOLD:ABV9918
Leptodrepana alexisae|[840]|BIOUG36495-H04|Derrumbe Malaise Trap|595[0n]|BOLD:ABV9918
Leptodrepana alexisae|[841]|BIOUG36488-A02|Derrumbe Malaise Trap|595[0n]|BOLD:ABV9918
Leptodrepana alexisae|[842]|DHJPAR0047318|malaise trap|658[0n]|BOLD:ABV9918
Leptodrepana freddyquesadai|[843]|BIOUG46868-C12|Malaise trap PL12-4B|654[0n]|BOLD:ADD4610
Leptodrepana freddyquesadai|[844]|BIOUG44209-D04|Malaise trap PL12-3B|653[0n]|BOLD:ADD4610
Leptodrepana freddyquesadai|[845]|BIOUG52059-H04|Malaise trap PL12-8B|661[0n]|BOLD:ADD4610
Leptodrepana freddyquesadai|[846]|BIOUG44814-C12|Malaise trap PL12-1B|654[0n]|BOLD:ADD4610
Leptodrepana freddyquesadai|[847]|BIOUG44786-F12|Malaise trap PL12-1B|654[0n]|BOLD:ADD4610
Leptodrepana freddyquesadai|[848]|BIOUG44761-B06|Malaise trap PL12-3B|654[0n]|BOLD:ADD4610
Leptodrepana freddyquesadai|[849]|BIOUG44165-A06|Malaise trap PL12-3B|653[0n]|BOLD:ADD4610
Leptodrepana freddyquesadai|[850]|BIOUG30966-F04|Malaise trap PL12-2A|585[0n]|BOLD:ADD4610
Leptodrepana freddyquesadai|[851]|BIOUG44761-G09|Malaise trap PL12-3B|655[0n]|BOLD:ADD4610
Leptodrepana freddyquesadai|[852]|BIOUG44722-B09|Malaise trap PL12-3B|654[0n]|BOLD:ADD4610
Leptodrepana freddyquesadai|[853]|BIOUG44761-C02|Malaise trap PL12-3B|655[0n]|BOLD:ADD4610
Leptodrepana freddyquesadai|[854]|BIOUG44230-C08|Malaise trap PL12-3B|654[0n]|BOLD:ADD4610
Leptodrepana freddyquesadai|[855]|BIOUG44228-G09|Malaise trap PL12-3B|654[0n]|BOLD:ADD4610
Leptodrepana freddyquesadai|[856]|BIOUG44761-G02|Malaise trap PL12-3B|655[0n]|BOLD:ADD4610
Leptodrepana freddyquesadai|[857]|BIOUG44228-G11|Malaise trap PL12-3B|653[0n]|BOLD:ADD4610
Leptodrepana freddyquesadai|[858]|BIOUG52817-G07|Malaise trap PL12-3C|653[0n]|BOLD:ADD4610
Leptodrepana freddyquesadai|[859]|BIOUG43805-A02|Malaise trap PL12-3B|654[0n]|BOLD:ADD4610
Leptodrepana freddyquesadai|[860]|BIOUG51656-D01|Malaise trap PL12-2B|652[0n]|BOLD:ADD4610
Leptodrepana freddyquesadai|[861]|BIOUG51481-F09|Malaise trap PL12-7B|654[0n]|BOLD:ADD4610
Leptodrepana freddyquesadai|[862]|BIOUG44777-D02|Malaise trap PL12-1B|652[0n]|BOLD:ADD4610
Leptodrepana freddyquesadai|[863]|BIOUG44737-H05|Malaise trap PL12-3B|654[0n]|BOLD:ADD4610
Leptodrepana freddyquesadai|[864]|BIOUG44164-G06|Malaise trap PL12-3B|654[0n]|BOLD:ADD4610
Leptodrepana freddyquesadai|[865]|BIOUG53382-F12|Malaise trap PL12-1C|654[0n]|BOLD:ADD4610
Leptodrepana freddyquesadai|[866]|BIOUG52070-E09|Malaise trap PL12-8B|653[0n]|BOLD:ADD4610
Leptodrepana freddyquesadai|[867]|BIOUG44226-H08|Malaise trap PL12-3B|654[0n]|BOLD:ADD4610
Leptodrepana freddyquesadai|[868]|BIOUG44762-H03|Malaise trap PL12-3B|653[0n]|BOLD:ADD4610
Leptodrepana freddyquesadai|[869]|BIOUG44229-E01|Malaise trap PL12-3B|655[0n]|BOLD:ADD4610
Ascogaster gloriasihezarae|[870]|BIOUG28068-C02|ESG Malaise Trap|600[0n]|BOLD:ADA4144
Ascogaster Janzen5720|[871]|DHJPAR0045405|chryBioLep01 BioLep813|658[3n]|BOLD:AAF5720
Ascogaster Janzen6762|[872]|BIOUG50091-F04|Malaise trap PL12-3B|654[0n]|BOLD:AEA6762
Ascogaster luisdiegogomezi|[873]|DHJPAR0049931|Stenoma Janzen414|658[0n]|BOLD:ACC1221
Ascogaster Malaise2547|[874]|BIOUG49600-C06|Malaise trap PL12-1B|652[0n]|BOLD:AEA2547
Ascogaster Janzen1177|[875]|DHJPAR0048996|Tancoa crinitaDHJ03|658[0n]|BOLD:ACB1177
Ascogaster Janzen1028|[876]|DHJPAR0036399|Grapholita Janzen30|658[0n]|BOLD:AAK1028
Ascogaster gustavoecheverri|[877]|DHJPAR0045414|Megalota delphinosema|654[0n]|BOLD:AAL0574
Ascogaster gustavoecheverri|[878]|DHJPAR0054566|Megalota crassana|654[0n]|BOLD:AAL0574
Ascogaster gustavoecheverri|[879]|DHJPAR0049003|Megalota simpliciana|654[0n]|BOLD:AAL0574
Ascogaster gustavoecheverri|[880]|DHJPAR0062572|Megalota crassana|592[0n]|BOLD:AAL0574
Ascogaster gustavoecheverri|[881]|DHJPAR0042856|Megalota spinulosa|654[0n]|BOLD:AAL0574
Ascogaster gustavoecheverri|[882]|DHJPAR0053678|Megalota crassana|654[0n]|BOLD:AAL0574
Ascogaster gustavoecheverri|[883]|DHJPAR0042852|Megalota ochreoapex|654[0n]|BOLD:AAL0574
Ascogaster gustavoecheverri|[884]|DHJPAR0046941|Megalota ochreoapex|654[1n]|BOLD:AAL0574

Ascogaster gustavoecheverri|[882]|DHJPAR0053678|Megalota crassana|654[0n]|BOLD:AAL0574
Ascogaster gustavoecheverri|[883]|DHJPAR0042852|Megalota ochreoapex|654[0n]|BOLD:AAL0574
Ascogaster gustavoecheverri|[884]|DHJPAR0046941|Megalota ochreoapex|654[1n]|BOLD:AAL0574
Ascogaster gustavoecheverri|[885]|DHJPAR0054560|Megalota crassana|654[1n]|BOLD:AAL0574
Ascogaster Janzen31|[886]|DHJPAR0037177|Megalota spinulosa|407[0n]
Ascogaster guillermopereirai|[887]|DHJPAR0051333|Mictopsichia Janzen330|658[0n]|BOLD:ACJ4048
Ascogaster guillermopereirai|[888]|DHJPAR0051327|Mictopsichia Janzen330|658[0n]|BOLD:ACJ4048
Ascogaster guillermopereirai|[889]|DHJPAR0051332|Mictopsichia Janzen330|624[0n]|BOLD:ACJ4048
Ascogaster grettelvegae|[890]|DHJPAR0043010|Episimus ortygia|623[0n]|BOLD:ABX8525
Ascogaster Janzen372|[891]|DHJPAR0040372|Strepsicrates Janzen342|239[0n]
Ascogaster katyvandusenae|[892]|DHJPAR0055824|Megalota spinulosa|658[0n]|BOLD:AAK1026
Ascogaster katyvandusenae|[893]|DHJPAR0035314|Amorbia decerptana|658[0n]|BOLD:AAK1026
Phanerotoma Janzen5955|[894]|BIOUG46544-F10|Malaise trap PL12-6B|653[0n]|BOLD:ADX5955
Phanerotoma Janzen5955|[895]|BIOUG46567-G10|Malaise trap PL12-6B|657[0n]|BOLD:ADX5955
Phanerotoma|[896]|BIOUG54550-E02|Malaise trap PL12-5C|662[0n]|BOLD:ADX5955
Phanerotoma Janzen5955|[897]|BIOUG50487-H09|Malaise trap PL12-5A|659[0n]|BOLD:ADX5955
Phanerotoma|[898]|BIOUG54860-B03|Malaise trap PL12-5C|656[0n]|BOLD:ADX5955
Phanerotoma|[899]|BIOUG53968-E11|Malaise trap PL12-9C|658[0n]|BOLD:ADX5955
Phanerotoma Janzen5955|[900]|BIOUG49774-G09|Malaise trap PL12-3B|660[0n]|BOLD:ADX5955
Phanerotoma Janzen5955|[901]|BIOUG49281-A10|Malaise trap PL12-5B|658[0n]|BOLD:ADX5955
Phanerotoma Janzen5955|[902]|BIOUG44872-E04|Malaise trap PL12-1B|643[0n]|BOLD:ADX5955
Phanerotoma Janzen5955|[903]|BIOUG49367-D01|Malaise trap PL12-5A|659[0n]|BOLD:ADX5955
Phanerotoma Janzen5955|[904]|BIOUG52352-F07|Malaise trap PL12-3C|653[0n]|BOLD:ADX5955
Phanerotoma|[905]|BIOUG56227-C06|Malaise trap PL12-4D|659[0n]|BOLD:ADX5955
Phanerotoma|[906]|BIOUG55997-C05|Malaise trap PL12-7D|641[0n]|BOLD:ADX5955
Phanerotoma|[907]|BIOUG56227-A07|Malaise trap PL12-4D|660[0n]|BOLD:ADX5955
Phanerotoma Janzen5955|[908]|BIOUG49776-C06|Malaise trap PL12-3B|666[0n]|BOLD:ADX5955
Phanerotoma Janzen5955|[909]|BIOUG44207-C06|Malaise trap PL12-3B|656[0n]|BOLD:ADX5955
Phanerotoma|[910]|BIOUG54768-H08|Malaise trap PL12-8C|659[0n]|BOLD:ADX5955
Phanerotoma Janzen5955|[911]|BIOUG52352-H05|Malaise trap PL12-3C|657[0n]|BOLD:ADX5955
Phanerotoma|[912]|BIOUG56451-C01|Malaise trap PL12-6D|656[0n]|BOLD:ADX5955
Phanerotoma Janzen5955|[913]|BIOUG46559-G12|Malaise trap PL12-6B|660[0n]|BOLD:ADX5955
Phanerotoma Janzen5955|[914]|BIOUG46571-A02|Malaise trap PL12-6B|658[0n]|BOLD:ADX5955
Phanerotoma Janzen5955|[915]|BIOUG51393-H06|Malaise trap PL12-8A|658[0n]|BOLD:ADX5955
Phanerotoma|[916]|BIOUG54626-B07|Malaise trap PL12-4D|657[0n]|BOLD:ADX5955
Phanerotoma Janzen5955|[917]|BIOUG51826-D12|Malaise trap PL12-7B|645[0n]|BOLD:ADX5955
Phanerotoma Janzen5955|[918]|BIOUG52824-D03|Malaise trap PL12-3C|642[0n]|BOLD:ADX5955
Phanerotoma Janzen5955|[919]|BIOUG44686-F02|Malaise trap PL12-3B|655[0n]|BOLD:ADX5955
Phanerotoma Janzen5955|[920]|BIOUG49375-D06|Malaise trap PL12-5B|657[0n]|BOLD:ADX5955
Phanerotoma|[921]|BIOUG53877-F06|Malaise trap PL12-9C|658[0n]|BOLD:ADX5955
Phanerotoma Janzen5955|[922]|BIOUG51827-F11|Malaise trap PL12-7B|654[0n]|BOLD:ADX5955
Phanerotoma Janzen5955|[923]|BIOUG49375-C12|Malaise trap PL12-5B|661[0n]|BOLD:ADX5955
Phanerotoma Janzen5955|[924]|BIOUG51854-C11|Malaise trap PL12-7B|643[0n]|BOLD:ADX5955
Phanerotoma Janzen5955|[925]|BIOUG52367-C12|Malaise trap PL12-3C|658[0n]|BOLD:ADX5955
Phanerotoma Janzen5955|[926]|BIOUG49638-F09|Malaise trap PL12-1B|658[0n]|BOLD:ADX5955
Phanerotoma Janzen5955|[927]|BIOUG50600-D11|Malaise trap PL12-5A|658[0n]|BOLD:ADX5955
Phanerotoma Janzen5955|[928]|BIOUG50485-G07|Malaise trap PL12-5A|658[0n]|BOLD:ADX5955
Phanerotoma|[929]|BIOUG56256-H01|Malaise trap PL12-6D|657[0n]|BOLD:ADX5955
Phanerotoma Janzen5955|[930]|BIOUG50485-F06|Malaise trap PL12-5A|659[0n]|BOLD:ADX5955
Phanerotoma Janzen5955|[931]|BIOUG44386-C02|Malaise trap PL12-3B|659[0n]|BOLD:ADX5955
Phanerotoma|[932]|BIOUG56230-G09|Malaise trap PL12-4D|644[0n]|BOLD:ADX5955
Phanerotoma|[933]|BIOUG55830-C03|Malaise trap PL12-4D|640[0n]|BOLD:ADX5955
Phanerotoma|[934]|BIOUG56342-H04|Malaise trap PL12-9D|656[0n]|BOLD:ADX5955
Phanerotoma Janzen5955|[935]|BIOUG53160-B04|Malaise trap PL12-9C|660[0n]|BOLD:ADX5955
Phanerotoma Janzen5955|[936]|BIOUG52432-D11|Malaise trap PL12-3C|657[0n]|BOLD:ADX5955
Phanerotoma Janzen5955|[937]|BIOUG46756-F03|Malaise trap PL12-4B|642[0n]|BOLD:ADX5955
Phanerotoma Janzen5955|[938]|BIOUG51902-B07|Malaise trap PL12-7B|660[0n]|BOLD:ADX5955
Phanerotoma Janzen5955|[939]|BIOUG51350-B05|Malaise trap PL12-8A|640[0n]|BOLD:ADX5955
Phanerotoma Janzen5955|[940]|BIOUG46760-C10|Malaise trap PL12-4B|658[0n]|BOLD:ADX5955
Phanerotoma Janzen5955|[941]|BIOUG48971-E02|Malaise trap PL12-5A|645[0n]|BOLD:ADX5955
Phanerotoma Janzen5955|[942]|BIOUG44206-G04|Malaise trap PL12-3B|658[0n]|BOLD:ADX5955
Phanerotoma Janzen5955|[943]|BIOUG44172-F04|Malaise trap PL12-3B|639[0n]|BOLD:ADX5955
Phanerotoma|[944]|BIOUG55502-F01|Malaise trap PL12-1D|641[0n]|BOLD:ADX5955
Phanerotoma|[945]|BIOUG53861-B10|Malaise trap PL12-9C|641[0n]|BOLD:ADX5955
Phanerotoma Janzen5955|[946]|BIOUG51498-D11|Malaise trap PL12-8B|640[0n]|BOLD:ADX5955
Phanerotoma Janzen5955|[947]|BIOUG44780-D12|Malaise trap PL12-1B|657[0n]|BOLD:ADX5955
Phanerotoma Janzen5955|[948]|BIOUG52935-F06|Malaise trap PL12-3C|640[0n]|BOLD:ADX5955
Phanerotoma|[949]|BIOUG56256-G08|Malaise trap PL12-6D|657[0n]|BOLD:ADX5955
Phanerotoma Janzen5955|[950]|BIOUG52039-C05|Malaise trap PL12-8B|656[0n]|BOLD:ADX5955
Phanerotoma|[951]|BIOUG55979-E08|Malaise trap PL12-4D|656[0n]|BOLD:ADX5955
Phanerotoma Janzen5955|[952]|BIOUG51829-G08|Malaise trap PL12-7B|640[0n]|BOLD:ADX5955
Phanerotoma Janzen5955|[953]|BIOUG49303-E08|Malaise trap PL12-5B|645[0n]|BOLD:ADX5955
Phanerotoma Janzen5955|[954]|BIOUG46567-G07|Malaise trap PL12-6B|657[0n]|BOLD:ADX5955
Phanerotoma Janzen5955|[955]|BIOUG51350-B11|Malaise trap PL12-8A|657[0n]|BOLD:ADX5955
Phanerotoma|[956]|BIOUG56227-B12|Malaise trap PL12-4D|657[0n]|BOLD:ADX5955
Phanerotoma Janzen5955|[957]|BIOUG48840-C01|Malaise trap PL12-5A|657[0n]|BOLD:ADX5955
Phanerotoma Janzen5955|[958]|BIOUG52294-G11|Malaise trap PL12-3C|640[0n]|BOLD:ADX5955
Phanerotoma Janzen5955|[959]|BIOUG52138-E04|Malaise trap PL12-8B|658[0n]|BOLD:ADX5955
Phanerotoma Janzen5955|[960]|BIOUG46559-G05|Malaise trap PL12-6B|656[0n]|BOLD:ADX5955
Phanerotoma Janzen5955|[961]|BIOUG49348-D08|Malaise trap PL12-3B|642[0n]|BOLD:ADX5955
Phanerotoma Janzen5955|[962]|BIOUG44846-A11|Malaise trap PL12-1B|643[0n]|BOLD:ADX5955
Phanerotoma Janzen5955|[963]|BIOUG51543-H01|Malaise trap PL12-8A|640[0n]|BOLD:ADX5955
Phanerotoma|[964]|BIOUG55975-H06|Malaise trap PL12-4D|664[0n]|BOLD:ADX5955
Phanerotoma Janzen5955|[965]|BIOUG49810-G04|Malaise trap PL12-3B|657[0n]|BOLD:ADX5955
Phanerotoma|[966]|BIOUG56233-A08|Malaise trap PL12-4D|658[0n]|BOLD:ADX5955
Phanerotoma|[967]|BIOUG54622-D04|Malaise trap PL12-1D|658[0n]|BOLD:ADX5955
Phanerotoma Janzen5955|[968]|BIOUG53054-F06|Malaise trap PL12-3C|658[0n]|BOLD:ADX5955
Phanerotoma|[969]|BIOUG56077-E06|Malaise trap PL12-9D|656[0n]|BOLD:ADX5955
Phanerotoma|[970]|BIOUG55656-B11|Malaise trap PL12-1D|643[0n]|BOLD:ADX5955
Phanerotoma Janzen5955|[971]|BIOUG44019-H03|Malaise trap PL12-1B|657[0n]|BOLD:ADX5955
Phanerotoma Janzen5955|[972]|BIOUG52122-F03|Malaise trap PL12-8B|658[0n]|BOLD:ADX5955
Phanerotoma Janzen5955|[973]|BIOUG51885-H11|Malaise trap PL12-7B|640[0n]|BOLD:ADX5955
Phanerotoma Janzen5955|[974]|BIOUG48966-H04|Malaise trap PL12-5A|663[0n]|BOLD:ADX5955
Phanerotoma Janzen5955|[975]|BIOUG51375-A10|Malaise trap PL12-8A|658[0n]|BOLD:ADX5955
Phanerotoma Janzen5955|[976]|BIOUG47379-F06|Malaise trap PL12-5A|660[0n]|BOLD:ADX5955
Phanerotoma|[977]|BIOUG56227-A12|Malaise trap PL12-4D|640[0n]|BOLD:ADX5955
Phanerotoma Janzen5955|[978]|BIOUG52432-A08|Malaise trap PL12-3C|640[0n]|BOLD:ADX5955
Phanerotoma Janzen5955|[979]|BIOUG52368-E02|Malaise trap PL12-3C|642[0n]|BOLD:ADX5955
Phanerotoma Janzen5955|[980]|BIOUG52034-G09|Malaise trap PL12-8B|642[0n]|BOLD:ADX5955
Phanerotoma Janzen5955|[981]|BIOUG47383-G02|Malaise trap PL12-5A|656[0n]|BOLD:ADX5955
Phanerotoma|[982]|BIOUG54628-D10|Malaise trap PL12-4D|640[0n]|BOLD:ADX5955

Phanerotoma Janzen5955|[980]|BIOUG52034-G09|Malaise trap PL12-8B|642[0n]|BOLD:ADX5955
Phanerotoma Janzen5955|[981]|BIOUG47383-G02|Malaise trap PL12-5A|656[0n]|BOLD:ADX5955
Phanerotoma|[982]|BIOUG54628-D10|Malaise trap PL12-4D|640[0n]|BOLD:ADX5955
Phanerotoma Janzen5955|[983]|BIOUG51431-C05|Malaise trap PL12-8A|642[0n]|BOLD:ADX5955
Phanerotoma Janzen5955|[984]|BIOUG46756-F04|Malaise trap PL12-4B|658[0n]|BOLD:ADX5955
Phanerotoma|[985]|BIOUG55809-H10|Malaise trap PL12-1D|649[0n]|BOLD:ADX5955
Phanerotoma Janzen5955|[986]|BIOUG47383-F08|Malaise trap PL12-5A|652[0n]|BOLD:ADX5955
Phanerotoma Janzen5955|[987]|BIOUG51826-E04|Malaise trap PL12-7B|656[0n]|BOLD:ADX5955
Phanerotoma Janzen5955|[988]|BIOUG48966-H02|Malaise trap PL12-5A|641[0n]|BOLD:ADX5955
Phanerotoma|[989]|BIOUG54782-B02|Malaise trap PL12-8C|659[0n]|BOLD:ADX5955
Phanerotoma Janzen5955|[990]|BIOUG49593-A09|Malaise trap PL12-1B|659[0n]|BOLD:ADX5955
Phanerotoma Janzen5955|[991]|BIOUG49124-F07|Malaise trap PL12-5A|640[0n]|BOLD:ADX5955
Phanerotoma Janzen5955|[992]|BIOUG52352-G03|Malaise trap PL12-3C|657[0n]|BOLD:ADX5955
Phanerotoma Janzen5955|[993]|BIOUG51825-A05|Malaise trap PL12-7B|643[0n]|BOLD:ADX5955
Phanerotoma Janzen5955|[994]|BIOUG46548-D11|Malaise trap PL12-6B|640[0n]|BOLD:ADX5955
Phanerotoma Janzen5955|[995]|BIOUG49590-H04|Malaise trap PL12-1B|657[0n]|BOLD:ADX5955
Phanerotoma Janzen5955|[996]|BIOUG53514-F10|Malaise trap PL12-6C|660[0n]|BOLD:ADX5955
Phanerotoma|[997]|BIOUG56233-A02|Malaise trap PL12-4D|660[0n]|BOLD:ADX5955
Phanerotoma Janzen5955|[998]|BIOUG44177-H08|Malaise trap PL12-3B|658[0n]|BOLD:ADX5955
Phanerotoma|[999]|BIOUG54554-B11|Malaise trap PL12-5C|661[0n]|BOLD:ADX5955
Phanerotoma|[1000]|BIOUG55056-C08|Malaise trap PL12-8C|654[0n]|BOLD:ADX5955
Phanerotoma Janzen5955|[1001]|BIOUG52535-A11|Malaise trap PL12-3C|659[0n]|BOLD:ADX5955
Phanerotoma|[1002]|BIOUG56227-B07|Malaise trap PL12-4D|658[0n]|BOLD:ADX5955
Phanerotoma Janzen5955|[1003]|BIOUG49525-D01|Malaise trap PL12-5B|660[0n]|BOLD:ADX5955
Phanerotoma|[1004]|BIOUG55860-D09|Malaise trap PL12-4D|663[0n]|BOLD:ADX5955
Phanerotoma|[1005]|BIOUG56077-E05|Malaise trap PL12-9D|657[0n]|BOLD:ADX5955
Phanerotoma|[1006]|BIOUG55009-A11|Malaise trap PL12-8C|657[0n]|BOLD:ADX5955
Phanerotoma Janzen5955|[1007]|BIOUG51387-H02|Malaise trap PL12-8A|657[0n]|BOLD:ADX5955
Phanerotoma Janzen5955|[1008]|BIOUG50585-G08|Malaise trap PL12-5A|664[0n]|BOLD:ADX5955
Phanerotoma Janzen5955|[1009]|BIOUG51829-G11|Malaise trap PL12-7B|658[0n]|BOLD:ADX5955
Phanerotoma Janzen5955|[1010]|BIOUG52138-G02|Malaise trap PL12-8B|658[0n]|BOLD:ADX5955
Phanerotoma Janzen5955|[1011]|BIOUG53206-F04|Malaise trap PL12-3C|657[0n]|BOLD:ADX5955
Phanerotoma Janzen5955|[1012]|BIOUG52059-F02|Malaise trap PL12-8B|660[0n]|BOLD:ADX5955
Phanerotoma Janzen5955|[1013]|BIOUG52352-H10|Malaise trap PL12-3C|648[0n]|BOLD:ADX5955
Phanerotoma Janzen5955|[1014]|BIOUG51826-E01|Malaise trap PL12-7B|655[0n]|BOLD:ADX5955
Phanerotoma Janzen5955|[1015]|BIOUG49121-B09|Malaise trap PL12-5A|657[0n]|BOLD:ADX5955
Phanerotoma Janzen5955|[1016]|BIOUG51854-D03|Malaise trap PL12-7B|661[0n]|BOLD:ADX5955
Phanerotoma Janzen5955|[1017]|BIOUG51886-C08|Malaise trap PL12-7B|656[0n]|BOLD:ADX5955
Phanerotoma Janzen5955|[1018]|BIOUG52968-C10|Malaise trap PL12-3C|657[0n]|BOLD:ADX5955
Phanerotoma Janzen5955|[1019]|BIOUG51350-B09|Malaise trap PL12-8A|664[0n]|BOLD:ADX5955
Phanerotoma Janzen5955|[1020]|BIOUG52094-H06|Malaise trap PL12-3C|659[0n]|BOLD:ADX5955
Phanerotoma Janzen5955|[1021]|BIOUG52120-E12|Malaise trap PL12-8B|655[0n]|BOLD:ADX5955
Phanerotoma Janzen5955|[1022]|BIOUG49111-G03|Malaise trap PL12-5A|659[0n]|BOLD:ADX5955
Phanerotoma Janzen5955|[1023]|BIOUG52352-H01|Malaise trap PL12-3C|659[0n]|BOLD:ADX5955
Phanerotoma Janzen5955|[1024]|BIOUG49280-H08|Malaise trap PL12-5B|642[0n]|BOLD:ADX5955
Phanerotoma Janzen5955|[1025]|BIOUG51828-E03|Malaise trap PL12-7B|641[0n]|BOLD:ADX5955
Phanerotoma Janzen5955|[1026]|BIOUG48840-D07|Malaise trap PL12-5A|658[0n]|BOLD:ADX5955
Phanerotoma Janzen5955|[1027]|BIOUG51829-G10|Malaise trap PL12-7B|641[0n]|BOLD:ADX5955
Phanerotoma Janzen5955|[1028]|BIOUG44762-D10|Malaise trap PL12-3B|658[0n]|BOLD:ADX5955
Phanerotoma Janzen5955|[1029]|BIOUG49610-G12|Malaise trap PL12-1B|660[0n]|BOLD:ADX5955
Phanerotoma|[1030]|BIOUG53973-B09|Malaise trap PL12-9C|657[0n]|BOLD:ADX5955
Phanerotoma Janzen5955|[1031]|BIOUG49616-D04|Malaise trap PL12-1B|642[0n]|BOLD:ADX5955
Phanerotoma|[1032]|BIOUG56227-B05|Malaise trap PL12-4D|660[0n]|BOLD:ADX5955
Phanerotoma|[1033]|BIOUG55965-D08|Malaise trap PL12-4D|641[0n]|BOLD:ADX5955
Phanerotoma|[1034]|BIOUG54782-B06|Malaise trap PL12-8C|658[0n]|BOLD:ADX5955
Phanerotoma Janzen5955|[1035]|BIOUG50487-H10|Malaise trap PL12-5A|658[0n]|BOLD:ADX5955
Phanerotoma Janzen5955|[1036]|BIOUG48966-H09|Malaise trap PL12-5A|658[0n]|BOLD:ADX5955
Phanerotoma|[1037]|BIOUG56233-A03|Malaise trap PL12-4D|656[0n]|BOLD:ADX5955
Phanerotoma Janzen5955|[1038]|BIOUG51882-H01|Malaise trap PL12-7B|641[0n]|BOLD:ADX5955
Phanerotoma anamariamongeae|[1039]|BIOUG30934-H03|Malaise trap PL12-2A|504[0n]|BOLD:ADA6322
Phanerotoma anamariamongeae|[1040]|BIOUG29135-F05|Malaise trap PL12-1A|585[0n]|BOLD:ADA6322
Phanerotoma anamariamongeae|[1041]|BIOUG28770-H10|Malaise trap PL12-1A|585[0n]|BOLD:ADA6322
Phanerotoma anamariamongeae|[1042]|BIOUG29000-F05|Malaise trap PL12-9A|588[0n]|BOLD:ADA6322
Phanerotoma anamariamongeae|[1043]|BIOUG30004-F07|Malaise trap PL12-3A|600[0n]|BOLD:ADA6322
Phanerotoma anamariamongeae|[1044]|BIOUG29783-D03|Malaise trap PL12-3A|600[0n]|BOLD:ADA6322
Phanerotoma anamariamongeae|[1045]|BIOUG29300-G05|Malaise trap PL12-9A|594[0n]|BOLD:ADA6322
Phanerotoma anamariamongeae|[1046]|BIOUG29646-G09|Malaise trap PL12-3A|594[0n]|BOLD:ADA6322
Phanerotoma anamariamongeae|[1047]|BIOUG28846-B09|Malaise trap PL12-7A|564[0n]|BOLD:ADA6322
Phanerotoma anamariamongeae|[1048]|BIOUG28729-D07|Malaise trap PL12-9A|564[0n]|BOLD:ADA6322
Phanerotoma anamariamongeae|[1049]|BIOUG28674-H11|Malaise trap PL12-9A|564[0n]|BOLD:ADA6322
Phanerotoma anamariamongeae|[1050]|BIOUG29689-G09|Malaise trap PL12-3A|555[0n]|BOLD:ADA6322
Phanerotoma anamariamongeae|[1051]|BIOUG28728-G10|Malaise trap PL12-9A|582[0n]|BOLD:ADA6322
Phanerotoma anamariamongeae|[1052]|BIOUG28724-C04|Malaise trap PL12-9A|600[0n]|BOLD:ADA6322
Phanerotoma anamariamongeae|[1053]|BIOUG29223-H09|Malaise trap PL12-9A|555[0n]|BOLD:ADA6322
Phanerotoma anamariamongeae|[1054]|BIOUG28825-B01|Malaise trap PL12-9A|564[0n]|BOLD:ADA6322
Phanerotoma anamariamongeae|[1055]|BIOUG29058-B09|Malaise trap PL12-1A|564[0n]|BOLD:ADA6322
Phanerotoma anamariamongeae|[1056]|BIOUG28721-A12|Malaise trap PL12-9A|564[1n]|BOLD:ADA6322
Phanerotoma anamariamongeae|[1057]|BIOUG29064-E02|Malaise trap PL12-1A|501[2n]|BOLD:ADA6322
Phanerotoma anamariamongeae|[1058]|BIOUG56110-A09|Malaise trap PL12-4D|652[0n]|BOLD:ADA6322
Phanerotoma anamariamongeae|[1059]|BIOUG49781-C11|Malaise trap PL12-3B|652[0n]|BOLD:ADA6322
Phanerotoma anamariamongeae|[1060]|BIOUG29783-D02|Malaise trap PL12-3A|594[0n]|BOLD:ADA6322
Phanerotoma anamariamongeae|[1061]|BIOUG28728-A09|Malaise trap PL12-9A|588[0n]|BOLD:ADA6322
Phanerotoma anamariamongeae|[1062]|BIOUG28779-B01|Malaise trap PL12-1A|594[0n]|BOLD:ADA6322
Phanerotoma anamariamongeae|[1063]|BIOUG28743-A02|Malaise trap PL12-4A|594[0n]|BOLD:ADA6322
Phanerotoma anamariamongeae|[1064]|BIOUG29603-F01|Malaise trap PL12-9A|594[0n]|BOLD:ADA6322
Phanerotoma anamariamongeae|[1065]|BIOUG29322-E07|Malaise trap PL12-6A|594[0n]|BOLD:ADA6322
Phanerotoma anamariamongeae|[1066]|BIOUG28728-G08|Malaise trap PL12-9A|594[0n]|BOLD:ADA6322
Phanerotoma anamariamongeae|[1067]|BIOUG30232-B11|Malaise trap PL12-3A|565[0n]|BOLD:ADA6322
Phanerotoma anamariamongeae|[1068]|BIOUG28728-G09|Malaise trap PL12-9A|564[0n]|BOLD:ADA6322
Phanerotoma anamariamongeae|[1069]|BIOUG30916-E08|Malaise trap PL12-2A|585[0n]|BOLD:ADA6322
Phanerotoma anamariamongeae|[1070]|BIOUG51684-D10|Malaise trap PL12-2B|653[0n]|BOLD:ADA6322
Phanerotoma anamariamongeae|[1071]|BIOUG28667-D07|Malaise trap PL12-9A|576[0n]|BOLD:ADA6322
Phanerotoma anamariamongeae|[1072]|BIOUG29229-F07|Malaise trap PL12-9A|555[0n]|BOLD:ADA6322
Phanerotoma anamariamongeae|[1073]|BIOUG28770-H07|Malaise trap PL12-1A|591[0n]|BOLD:ADA6322
Phanerotoma anamariamongeae|[1074]|BIOUG29794-F10|Malaise trap PL12-3A|555[0n]|BOLD:ADA6322
Phanerotoma anamariamongeae|[1075]|BIOUG28830-H04|Malaise trap PL12-9A|600[0n]|BOLD:ADA6322
Phanerotoma anamariamongeae|[1076]|BIOUG28678-G12|Malaise trap PL12-9A|606[0n]|BOLD:ADA6322
Phanerotoma anamariamongeae|[1077]|BIOUG29831-A06|Malaise trap PL12-3A|603[0n]|BOLD:ADA6322
Phanerotoma anamariamongeae|[1078]|BIOUG54922-G01|Malaise trap PL12-5C|649[0n]|BOLD:ADA6322
Phanerotoma anamariamongeae|[1079]|BIOUG29717-E03|Malaise trap PL12-3A|564[0n]|BOLD:ADA6322
Phanerotoma anamariamongeae|[1080]|BIOUG28876-C09|Malaise trap PL12-9A|564[0n]|BOLD:ADA6322

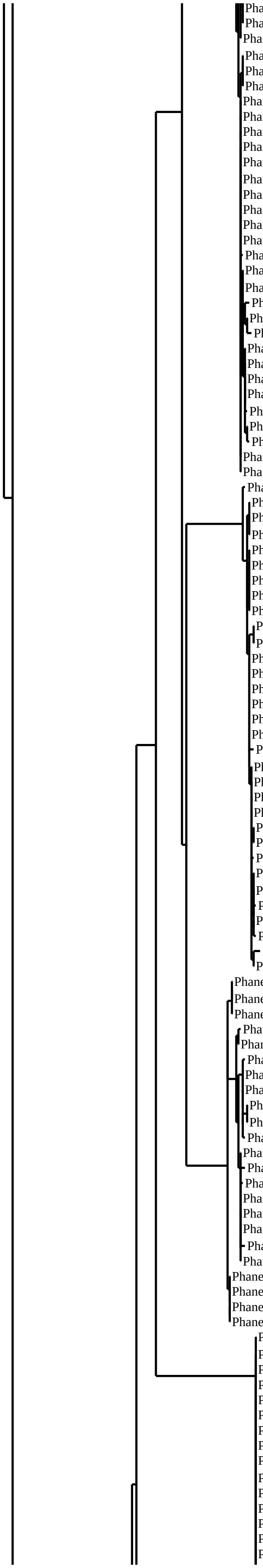

Phanerotoma anamariamongeae|[1078]|BIOUG54922-G01|Malaise trap PL12-5C|649[0n]|BOLD:ADA6322
Phanerotoma anamariamongeae|[1079]|BIOUG29717-E03|Malaise trap PL12-3A|564[0n]|BOLD:ADA6322
Phanerotoma anamariamongeae|[1080]|BIOUG28876-C09|Malaise trap PL12-9A|564[0n]|BOLD:ADA6322
Phanerotoma anamariamongeae|[1081]|BIOUG28831-F02|Malaise trap PL12-9A|594[0n]|BOLD:ADA6322
Phanerotoma anamariamongeae|[1082]|BIOUG29646-A01|Malaise trap PL12-3A|564[0n]|BOLD:ADA6322
Phanerotoma anamariamongeae|[1083]|BIOUG28823-E08|Malaise trap PL12-9A|594[0n]|BOLD:ADA6322
Phanerotoma anamariamongeae|[1084]|BIOUG28770-H11|Malaise trap PL12-1A|600[0n]|BOLD:ADA6322
Phanerotoma anamariamongeae|[1085]|BIOUG28830-H03|Malaise trap PL12-9A|576[0n]|BOLD:ADA6322
Phanerotoma anamariamongeae|[1086]|BIOUG30914-C02|Malaise trap PL12-2A|594[0n]|BOLD:ADA6322
Phanerotoma anamariamongeae|[1087]|BIOUG29689-D06|Malaise trap PL12-3A|594[0n]|BOLD:ADA6322
Phanerotoma anamariamongeae|[1088]|BIOUG30959-G10|Malaise trap PL12-2A|585[0n]|BOLD:ADA6322
Phanerotoma anamariamongeae|[1089]|BIOUG28777-A04|Malaise trap PL12-1A|555[0n]|BOLD:ADA6322
Phanerotoma anamariamongeae|[1090]|BIOUG28651-A02|Malaise trap PL12-1A|558[0n]|BOLD:ADA6322
Phanerotoma anamariamongeae|[1091]|BIOUG29473-H08|Malaise trap PL12-3A|552[0n]|BOLD:ADA6322
Phanerotoma anamariamongeae|[1092]|BIOUG29073-F06|Malaise trap PL12-7A|552[0n]|BOLD:ADA6322
Phanerotoma anamariamongeae|[1093]|BIOUG28388-H02|Malaise trap PL12-9X|606[3n]|BOLD:ADA6322
Phanerotoma anamariamongeae|[1094]|BIOUG54531-A08|Malaise trap PL12-5C|651[0n]|BOLD:ADA6322
Phanerotoma anamariamongeae|[1095]|BIOUG48838-A01|Malaise trap PL12-5A|656[0n]|BOLD:ADA6322
Phanerotoma anamariamongeae|[1096]|BIOUG51825-A10|Malaise trap PL12-7B|640[0n]|BOLD:ADA6322
Phanerotoma anamariamongeae|[1097]|BIOUG46553-B05|Malaise trap PL12-6B|640[0n]|BOLD:ADA6322
Phanerotoma anamariamongeae|[1098]|BIOUG49284-D12|Malaise trap PL12-5B|653[0n]|BOLD:ADA6322
Phanerotoma anamariamongeae|[1099]|BIOUG29165-D02|Malaise trap PL12-6A|459[0n]|BOLD:ADA6322
Phanerotoma anamariamongeae|[1100]|BIOUG48838-B04|Malaise trap PL12-5A|656[0n]|BOLD:ADA6322
Phanerotoma anamariamongeae|[1101]|BIOUG48966-H08|Malaise trap PL12-5A|651[0n]|BOLD:ADA6322
Phanerotoma anamariamongeae|[1102]|BIOUG55996-A10|Malaise trap PL12-7D|651[0n]|BOLD:ADA6322
Phanerotoma anamariamongeae|[1103]|BIOUG54531-A04|Malaise trap PL12-5C|651[0n]|BOLD:ADA6322
Phanerotoma anamariamongeae|[1104]|BIOUG54774-A09|Malaise trap PL12-8C|650[0n]|BOLD:ADA6322
Phanerotoma anamariamongeae|[1105]|BIOUG51829-H01|Malaise trap PL12-7B|646[0n]|BOLD:ADA6322
Phanerotoma anamariamongeae|[1106]|BIOUG54626-A09|Malaise trap PL12-4D|640[0n]|BOLD:ADA6322
Phanerotoma anamariamongeae|[1107]|BIOUG30934-H04|Malaise trap PL12-2A|519[0n]|BOLD:ADA6322
Phanerotoma anamariamongeae|[1108]|BIOUG29000-F06|Malaise trap PL12-9A|555[0n]|BOLD:ADA6322
Phanerotoma anacordobae|[1109]|BIOUG18290-D04|BSE Malaise Trap|564[0n]|BOLD:ACJ2167
Phanerotoma anacordobae|[1110]|BIOUG10753-B11|BSE Malaise Trap|553[0n]|BOLD:ACJ2167
Phanerotoma anacordobae|[1111]|BIOUG10016-G07|BSE Malaise Trap|568[0n]|BOLD:ACJ2167
Phanerotoma anacordobae|[1112]|BIOUG18290-H09|BSE Malaise Trap|555[0n]|BOLD:ACJ2167
Phanerotoma anacordobae|[1113]|BIOUG07616-F09|BSE Malaise Trap|535[0n]|BOLD:ACJ2167
Phanerotoma anacordobae|[1114]|BIOUG18498-H02|BSE Malaise Trap|564[0n]|BOLD:ACJ2167
Phanerotoma anacordobae|[1115]|BIOUG07781-H06|BSE Malaise Trap|576[0n]|BOLD:ACJ2167
Phanerotoma anacordobae|[1116]|BIOUG17335-E04|BSE Malaise Trap|567[0n]|BOLD:ACJ2167
Phanerotoma anacordobae|[1117]|BIOUG10016-H06|BSE Malaise Trap|565[0n]|BOLD:ACJ2167
Phanerotoma anacordobae|[1118]|BIOUG18661-G06|BSE Malaise Trap|594[1n]|BOLD:ACJ2167
Phanerotoma anacordobae|[1119]|BIOUG17684-D11|BSE Malaise Trap|555[0n]|BOLD:ACJ2167
Phanerotoma anacordobae|[1120]|BIOUG10247-H02|BSE Malaise Trap|544[0n]|BOLD:ACJ2167
Phanerotoma anacordobae|[1121]|BIOUG07616-D05|BSE Malaise Trap|542[0n]|BOLD:ACJ2167
Phanerotoma anacordobae|[1122]|BIOUG18499-H09|BSE Malaise Trap|564[0n]|BOLD:ACJ2167
Phanerotoma anacordobae|[1123]|BIOUG17346-E10|BSE Malaise Trap|576[0n]|BOLD:ACJ2167
Phanerotoma anacordobae|[1124]|BIOUG17684-B01|BSE Malaise Trap|564[0n]|BOLD:ACJ2167
Phanerotoma anacordobae|[1125]|BIOUG18290-E12|BSE Malaise Trap|564[0n]|BOLD:ACJ2167
Phanerotoma anacordobae|[1126]|BIOUG17684-B12|BSE Malaise Trap|555[0n]|BOLD:ACJ2167
Phanerotoma anacordobae|[1127]|BIOUG18290-C10|BSE Malaise Trap|582[0n]|BOLD:ACJ2167
Phanerotoma anacordobae|[1128]|BIOUG18499-A12|BSE Malaise Trap|594[0n]|BOLD:ACJ2167
Phanerotoma anacordobae|[1129]|BIOUG10017-H03|BSE Malaise Trap|597[0n]|BOLD:ACJ2167
Phanerotoma anacordobae|[1130]|BIOUG08266-F05|BSE Malaise Trap|597[1n]|BOLD:ACJ2167
Phanerotoma anacordobae|[1131]|BIOUG18397-C12|BSE Malaise Trap|632[0n]|BOLD:ACJ2167
Phanerotoma anacordobae|[1132]|BIOUG18398-D11|BSE Malaise Trap|632[0n]|BOLD:ACJ2167
Phanerotoma anacordobae|[1133]|BIOUG17335-H08|BSE Malaise Trap|600[0n]|BOLD:ACJ2167
Phanerotoma anacordobae|[1134]|BIOUG18664-E01|BSE Malaise Trap|582[0n]|BOLD:ACJ2167
Phanerotoma anacordobae|[1135]|BIOUG07918-H09|BSE Malaise Trap|658[0n]|BOLD:ACJ2167
Phanerotoma anacordobae|[1136]|BIOUG18398-F09|BSE Malaise Trap|632[0n]|BOLD:ACJ2167
Phanerotoma anacordobae|[1137]|BIOUG18398-B04|BSE Malaise Trap|632[0n]|BOLD:ACJ2167
Phanerotoma anacordobae|[1138]|BIOUG18398-G07|BSE Malaise Trap|632[0n]|BOLD:ACJ2167
Phanerotoma anacordobae|[1139]|BIOUG17335-B07|BSE Malaise Trap|609[0n]|BOLD:ACJ2167
Phanerotoma anacordobae|[1140]|BIOUG18290-G07|BSE Malaise Trap|585[0n]|BOLD:ACJ2167
Phanerotoma alvaroherrerai|[1141]|BIOUG09739-D11|BSE Malaise Trap|591[0n]|BOLD:AAL8247
Phanerotoma alvaroherrerai|[1142]|BIOUG10247-E11|BSE Malaise Trap|557[0n]|BOLD:AAL8247
Phanerotoma alvaroherrerai|[1143]|BIOUG18290-D02|BSE Malaise Trap|594[0n]|BOLD:AAL8247
Phanerotoma alvaroherrerai|[1144]|BIOUG18499-F06|BSE Malaise Trap|549[0n]|BOLD:AAL8247
Phanerotoma alvaroherrerai|[1145]|BIOUG08348-E03|BSE Malaise Trap|609[0n]|BOLD:AAL8247
Phanerotoma alvaroherrerai|[1146]|BIOUG09739-C01|BSE Malaise Trap|567[0n]|BOLD:AAL8247
Phanerotoma alvaroherrerai|[1147]|BIOUG10112-B02|BSE Malaise Trap|591[0n]|BOLD:AAL8247
Phanerotoma alvaroherrerai|[1148]|BIOUG09739-H08|BSE Malaise Trap|597[0n]|BOLD:AAL8247
Phanerotoma alvaroherrerai|[1149]|BIOUG18499-D10|BSE Malaise Trap|600[0n]|BOLD:AAL8247
Phanerotoma alvaroherrerai|[1150]|BIOUG10112-B03|BSE Malaise Trap|557[0n]|BOLD:AAL8247
Phanerotoma alvaroherrerai|[1151]|BIOUG18290-F11|BSE Malaise Trap|570[0n]|BOLD:AAL8247
Phanerotoma alvaroherrerai|[1152]|BIOUG10112-E11|BSE Malaise Trap|598[0n]|BOLD:AAL8247
Phanerotoma alvaroherrerai|[1153]|BIOUG18398-B05|BSE Malaise Trap|628[0n]|BOLD:AAL8247
Phanerotoma alvaroherrerai|[1154]|BIOUG10016-F02|BSE Malaise Trap|575[0n]|BOLD:AAL8247
Phanerotoma alvaroherrerai|[1155]|BIOUG18289-H10|BSE Malaise Trap|585[0n]|BOLD:AAL8247
Phanerotoma alvaroherrerai|[1156]|BIOUG17575-A10|BSE Malaise Trap|555[0n]|BOLD:AAL8247
Phanerotoma alvaroherrerai|[1157]|BIOUG18397-C11|BSE Malaise Trap|632[0n]|BOLD:AAL8247
Phanerotoma alvaroherrerai|[1158]|BIOUG07918-F10|BSE Malaise Trap|658[0n]|BOLD:AAL8247
Phanerotoma alvaroherrerai|[1159]|BIOUG18430-E07|BSE Malaise Trap|632[0n]|BOLD:AAL8247
Phanerotoma alvaroherrerai|[1160]|BIOUG18290-F04|BSE Malaise Trap|600[0n]|BOLD:AAL8247
Phanerotoma alvaroherrerai|[1161]|BIOUG18290-A04|BSE Malaise Trap|594[0n]|BOLD:AAL8247
Phanerotoma alvaroherrerai|[1162]|BIOUG10017-G06|BSE Malaise Trap|557[0n]|BOLD:AAL8247
Phanerotoma alvaroherrerai|[1163]|BIOUG18499-F05|BSE Malaise Trap|555[0n]|BOLD:AAL8247
Phanerotoma andydeansi|[1164]|BIOUG28830-H02|Malaise trap PL12-9A|582[0n]|BOLD:ADA7613
Phanerotoma andydeansi|[1165]|BIOUG28728-H01|Malaise trap PL12-9A|582[0n]|BOLD:ADA7613
Phanerotoma andydeansi|[1166]|BIOUG29059-D04|Malaise trap PL12-1A|582[0n]|BOLD:ADA7613
Phanerotoma andydeansi|[1167]|BIOUG29511-G04|Malaise trap PL12-9A|582[0n]|BOLD:ADA7613
Phanerotoma andydeansi|[1168]|BIOUG29322-E10|Malaise trap PL12-6A|573[0n]|BOLD:ADA7613
Phanerotoma andydeansi|[1169]|BIOUG29060-G09|Malaise trap PL12-1A|573[0n]|BOLD:ADA7613
Phanerotoma andydeansi|[1170]|BIOUG29146-E10|Malaise trap PL12-1A|588[0n]|BOLD:ADA7613
Phanerotoma andydeansi|[1171]|BIOUG29388-H08|Malaise trap PL12-9A|588[0n]|BOLD:ADA7613
Phanerotoma andydeansi|[1172]|BIOUG29064-E01|Malaise trap PL12-1A|555[2n]|BOLD:ADA7613
Phanerotoma andydeansi|[1173]|BIOUG28394-F08|Malaise trap PL12-9A|576[0n]|BOLD:ADA7613
Phanerotoma andydeansi|[1174]|BIOUG29725-C09|Malaise trap PL12-3A|594[0n]|BOLD:ADA7613
Phanerotoma andydeansi|[1175]|BIOUG28722-F01|Malaise trap PL12-9A|594[0n]|BOLD:ADA7613
Phanerotoma andydeansi|[1176]|BIOUG28764-F10|Malaise trap PL12-1A|594[0n]|BOLD:ADA7613
Phanerotoma andydeansi|[1177]|BIOUG28766-E08|Malaise trap PL12-1A|594[0n]|BOLD:ADA7613
Phanerotoma andydeansi|[1178]|BIOUG28673-C05|Malaise trap PL12-9A|594[0n]|BOLD:ADA7613
[illegible]

Phanerotoma andydeansi|[1176]|BIOUG28764-F10|Malaise trap PL12-1A|594[0n]|BOLD:ADA7613
Phanerotoma andydeansi|[1177]|BIOUG28766-E08|Malaise trap PL12-1A|594[0n]|BOLD:ADA7613
Phanerotoma andydeansi|[1178]|BIOUG28673-C05|Malaise trap PL12-9A|594[0n]|BOLD:ADA7613
Phanerotoma andydeansi|[1179]|BIOUG29406-G10|Malaise trap PL12-6A|594[0n]|BOLD:ADA7613
Phanerotoma andydeansi|[1180]|BIOUG30959-G11|Malaise trap PL12-2A|579[0n]|BOLD:ADA7613
Phanerotoma andydeansi|[1181]|BIOUG29140-A12|Malaise trap PL12-1A|579[0n]|BOLD:ADA7613
Phanerotoma andydeansi|[1182]|BIOUG29377-F12|Malaise trap PL12-4A|567[0n]|BOLD:ADA7613
Phanerotoma andydeansi|[1183]|BIOUG29322-E08|Malaise trap PL12-6A|585[0n]|BOLD:ADA7613
Phanerotoma andydeansi|[1184]|BIOUG29991-F01|Malaise trap PL12-3A|585[0n]|BOLD:ADA7613
Phanerotoma andydeansi|[1185]|BIOUG29398-F04|Malaise trap PL12-9A|600[0n]|BOLD:ADA7613
Phanerotoma andydeansi|[1186]|BIOUG28823-E07|Malaise trap PL12-9A|600[0n]|BOLD:ADA7613
Phanerotoma andydeansi|[1187]|BIOUG28667-D08|Malaise trap PL12-9A|473[0n]|BOLD:ADA7613
Phanerotoma andydeansi|[1188]|BIOUG29530-G09|Malaise trap PL12-3A|567[0n]|BOLD:ADA7613
Phanerotoma andydeansi|[1189]|BIOUG50557-G01|Malaise trap PL12-6B|654[0n]|BOLD:ADA7613
Phanerotoma andydeansi|[1190]|BIOUG50236-G01|Malaise trap PL12-3B|657[0n]|BOLD:ADA7613
Phanerotoma andydeansi|[1191]|BIOUG49281-A07|Malaise trap PL12-5B|656[0n]|BOLD:ADA7613
Phanerotoma andydeansi|[1192]|BIOUG53877-G10|Malaise trap PL12-9C|652[0n]|BOLD:ADA7613
Phanerotoma andydeansi|[1193]|BIOUG49284-B08|Malaise trap PL12-5B|653[0n]|BOLD:ADA7613
Phanerotoma andydeansi|[1194]|BIOUG50326-F08|Malaise trap PL12-6B|656[0n]|BOLD:ADA7613
Phanerotoma andydeansi|[1195]|BIOUG56110-B01|Malaise trap PL12-4D|654[0n]|BOLD:ADA7613
Phanerotoma andydeansi|[1196]|BIOUG55890-D10|Malaise trap PL12-1D|653[0n]|BOLD:ADA7613
Phanerotoma andydeansi|[1197]|BIOUG54624-H10|Malaise trap PL12-4D|653[0n]|BOLD:ADA7613
Phanerotoma andydeansi|[1198]|BIOUG53929-A03|Malaise trap PL12-9C|654[0n]|BOLD:ADA7613
Phanerotoma andydeansi|[1199]|BIOUG51377-G08|Malaise trap PL12-8A|653[0n]|BOLD:ADA7613
Phanerotoma andydeansi|[1200]|BIOUG50565-F01|Malaise trap PL12-6B|654[0n]|BOLD:ADA7613
Phanerotoma andydeansi|[1201]|BIOUG44862-C02|Malaise trap PL12-1B|653[0n]|BOLD:ADA7613
Phanerotoma andydeansi|[1202]|BIOUG49371-E08|Malaise trap PL12-5B|650[0n]|BOLD:ADA7613
Phanerotoma andydeansi|[1203]|BIOUG50316-E12|Malaise trap PL12-3B|649[0n]|BOLD:ADA7613
Phanerotoma andydeansi|[1204]|BIOUG50623-C03|Malaise trap PL12-5A|646[0n]|BOLD:ADA7613
Phanerotoma andydeansi|[1205]|BIOUG44226-A04|Malaise trap PL12-3B|653[0n]|BOLD:ADA7613
Phanerotoma andydeansi|[1206]|BIOUG56230-E09|Malaise trap PL12-4D|653[0n]|BOLD:ADA7613
Phanerotoma andydeansi|[1207]|BIOUG50553-C06|Malaise trap PL12-6B|656[0n]|BOLD:ADA7613
Phanerotoma andydeansi|[1208]|BIOUG55814-G07|Malaise trap PL12-1D|652[0n]|BOLD:ADA7613
Phanerotoma andydeansi|[1209]|BIOUG46569-F04|Malaise trap PL12-6B|654[0n]|BOLD:ADA7613
Phanerotoma andydeansi|[1210]|BIOUG55877-E09|Malaise trap PL12-1D|652[0n]|BOLD:ADA7613
Phanerotoma andydeansi|[1211]|BIOUG50322-F04|Malaise trap PL12-6B|655[0n]|BOLD:ADA7613
Phanerotoma andydeansi|[1212]|BIOUG44868-G01|Malaise trap PL12-1B|653[0n]|BOLD:ADA7613
Phanerotoma andydeansi|[1213]|BIOUG51421-G05|Malaise trap PL12-6B|653[0n]|BOLD:ADA7613
Phanerotoma andydeansi|[1214]|BIOUG44185-G07|Malaise trap PL12-3B|653[0n]|BOLD:ADA7613
Phanerotoma andydeansi|[1215]|BIOUG50566-F01|Malaise trap PL12-6B|654[0n]|BOLD:ADA7613
Phanerotoma andydeansi|[1216]|BIOUG56122-A08|Malaise trap PL12-6D|654[0n]|BOLD:ADA7613
Phanerotoma andydeansi|[1217]|BIOUG29403-F11|Malaise trap PL12-6A|573[0n]|BOLD:ADA7613
Phanerotoma angelsolisi|[1218]|BIOUG46717-A02|Malaise trap PL12-4B|653[0n]|BOLD:AAG8211
Phanerotoma angelsolisi|[1219]|BIOUG53206-G04|Malaise trap PL12-3C|652[0n]|BOLD:AAG8211
Phanerotoma angelsolisi|[1220]|BIOUG52293-E11|Malaise trap PL12-3C|653[0n]|BOLD:AAG8211
Phanerotoma angelsolisi|[1221]|BIOUG51524-G02|Malaise trap PL12-3C|640[0n]|BOLD:AAG8211
Phanerotoma angelsolisi|[1222]|BIOUG29415-C09|Malaise trap PL12-3A|600[0n]|BOLD:AAG8211
Phanerotoma Janzen6054|[1223]|BIOUG50089-B02|Malaise trap PL12-6B|654[0n]|BOLD:AEA6054
Phanerotoma barryhammeli|[1224]|BIOUG32861-A06|Derrumbe Malaise Trap|561[0n]|BOLD:ADG7956
Phanerotoma angelagonzalezae|[1225]|BIOUG30984-H06|Malaise trap PL12-2A|582[0n]|BOLD:ADB3156
Phanerotoma angelagonzalezae|[1226]|BIOUG29845-C10|Malaise trap PL12-3A|600[0n]|BOLD:ADB3156
Phanerotoma angelagonzalezae|[1227]|BIOUG29320-A09|Malaise trap PL12-6A|588[0n]|BOLD:ADB3156
Phanerotoma angelagonzalezae|[1228]|BIOUG29862-C07|Malaise trap PL12-3A|585[0n]|BOLD:ADB3156
Phanerotoma angelagonzalezae|[1229]|BIOUG50617-B11|Malaise trap PL12-5A|654[0n]|BOLD:ADB3156
Phanerotoma angelagonzalezae|[1230]|BIOUG49778-F05|Malaise trap PL12-3B|654[0n]|BOLD:ADB3156
Phanerotoma angelagonzalezae|[1231]|BIOUG56616-C12|Malaise trap PL12-7D|657[0n]|BOLD:ADB3156
Phanerotoma angelagonzalezae|[1232]|BIOUG50485-G01|Malaise trap PL12-5A|653[0n]|BOLD:ADB3156
Phanerotoma angelagonzalezae|[1233]|BIOUG44761-E07|Malaise trap PL12-3B|654[0n]|BOLD:ADB3156
Phanerotoma angelagonzalezae|[1234]|BIOUG56230-H05|Malaise trap PL12-4D|654[0n]|BOLD:ADB3156
Phanerotoma angelagonzalezae|[1235]|BIOUG51350-D04|Malaise trap PL12-8A|651[0n]|BOLD:ADB3156
Phanerotoma Kittel4221|[1236]|DHJPAR0052290|gelBioLep01 BioLep292|658[0n]|BOLD:ACH4221
Phanerotoma Kittel4221|[1237]|DHJPAR0052288|gelJanzen01 Janzen350|658[0n]|BOLD:ACH4221
Phanerotoma Kittel02|[1238]|DHJPAR0035477|gelBioLep01 BioLep292|283[0n]
Phanerotoma Janzen1106|[1239]|DHJPAR0039037|Argyrotaenia Brown13|658[0n]|BOLD:AAM1106
Phanerotoma christerhanssoni|[1240]|BIOUG17462-A10|BSE Malaise Trap|528[0n]|BOLD:ACL3879
Phanerotoma christerhanssoni|[1241]|BIOUG17575-C11|BSE Malaise Trap|555[0n]|BOLD:ACL3879
Phanerotoma christerhanssoni|[1242]|BIOUG18397-B07|BSE Malaise Trap|627[0n]|BOLD:ACL3879
Phanerotoma christerhanssoni|[1243]|BIOUG09826-F06|BSE Malaise Trap|656[0n]|BOLD:ACL3879
Phanerotoma christerhanssoni|[1244]|BIOUG10016-H08|BSE Malaise Trap|658[0n]|BOLD:ACL3879
Phanerotoma christerhanssoni|[1245]|BIOUG17616-B12|BSE Malaise Trap|537[0n]|BOLD:ACL3879
Phanerotoma christerhanssoni|[1246]|BIOUG18290-F03|BSE Malaise Trap|537[1n]|BOLD:ACL3879
Phanerotoma davesmithi|[1247]|BIOUG49616-E07|Malaise trap PL12-1B|654[0n]|BOLD:ADB4617
Phanerotoma davesmithi|[1248]|BIOUG30497-D05|Malaise trap PL12-9A|594[0n]|BOLD:ADB4617
Phanerotoma davesmithi|[1249]|BIOUG29999-D12|Malaise trap PL12-3A|564[0n]|BOLD:ADB4617
Phanerotoma davesmithi|[1250]|BIOUG44148-F04|Malaise trap PL12-3B|655[0n]|BOLD:ADB4617
Phanerotoma davesmithi|[1251]|BIOUG29964-B02|Malaise trap PL12-3A|600[0n]|BOLD:ADB4617
Phanerotoma davesmithi|[1252]|BIOUG29436-A09|Malaise trap PL12-6A|600[0n]|BOLD:ADB4617
Phanerotoma davesmithi|[1253]|BIOUG50653-H06|Malaise trap PL12-5A|654[0n]|BOLD:ADB4617
Phanerotoma davesmithi|[1254]|BIOUG46162-C09|Malaise trap PL12-6B|654[0n]|BOLD:ADB4617
Phanerotoma davesmithi|[1255]|BIOUG50617-B06|Malaise trap PL12-5A|654[0n]|BOLD:ADB4617
Phanerotoma davesmithi|[1256]|BIOUG50465-F04|Malaise trap PL12-6B|653[0n]|BOLD:ADB4617
Phanerotoma davesmithi|[1257]|BIOUG53929-A02|Malaise trap PL12-9C|654[0n]|BOLD:ADB4617
Phanerotoma davesmithi|[1258]|BIOUG51602-D02|Malaise trap PL12-8A|653[0n]|BOLD:ADB4617
Phanerotoma davesmithi|[1259]|BIOUG51602-C12|Malaise trap PL12-8A|651[0n]|BOLD:ADB4617
Phanerotoma davesmithi|[1260]|BIOUG51817-D12|Malaise trap PL12-7B|640[0n]|BOLD:ADB4617
Phanerotoma davesmithi|[1261]|BIOUG30507-F05|Malaise trap PL12-6A|564[0n]|BOLD:ADB4617
Phanerotoma davesmithi|[1262]|BIOUG29992-B03|Malaise trap PL12-3A|564[0n]|BOLD:ADB4617
Phanerotoma davesmithi|[1263]|BIOUG29437-F01|Malaise trap PL12-6A|564[0n]|BOLD:ADB4617
Phanerotoma davesmithi|[1264]|BIOUG30041-B07|Malaise trap PL12-3A|579[0n]|BOLD:ADB4617
Phanerotoma davesmithi|[1265]|BIOUG29584-C07|Malaise trap PL12-6A|564[0n]|BOLD:ADB4617
Phanerotoma christhompsoni|[1266]|BIOUG08905-F11|BSE Malaise Trap|658[0n]|BOLD:ACL5703
Phanerotoma christhompsoni|[1267]|BIOUG17575-E05|BSE Malaise Trap|600[0n]|BOLD:ACL5703
Phanerotoma christhompsoni|[1268]|BIOUG09827-A02|BSE Malaise Trap|595[0n]|BOLD:ACL5703
Phanerotoma christhompsoni|[1269]|BIOUG18290-E11|BSE Malaise Trap|591[0n]|BOLD:ACL5703
Phanerotoma christhompsoni|[1270]|BIOUG17494-C11|BSE Malaise Trap|606[0n]|BOLD:ACL5703
Phanerotoma christhompsoni|[1271]|BIOUG08904-A11|BSE Malaise Trap|567[0n]|BOLD:ACL5703
Phanerotoma christhompsoni|[1272]|BIOUG09739-F12|BSE Malaise Trap|594[0n]|BOLD:ACL5703
Phanerotoma christhompsoni|[1273]|BIOUG18290-F08|BSE Malaise Trap|588[0n]|BOLD:ACL5703
Phanerotoma christhompsoni|[1274]|BIOUG08356-G03|BSE Malaise Trap|664[0n]|BOLD:ACL5703
Phanerotoma christhompsoni|[1275]|BIOUG08905-F02|BSE Malaise Trap|658[0n]|BOLD:ACL5703
Phanerotoma christhompsoni|[1276]|BIOUG09739-B06|BSE Malaise Trap|658[0n]|BOLD:ACL5703
[illegible]

Phanerotoma christhompsoni|[1274]|BIOUG08356-G03|BSE Malaise Trap|664[0n]|BOLD:ACL5703
Phanerotoma christhompsoni|[1275]|BIOUG08905-F02|BSE Malaise Trap|658[0n]|BOLD:ACL5703
Phanerotoma christhompsoni|[1276]|BIOUG09739-B06|BSE Malaise Trap|658[0n]|BOLD:ACL5703
Phanerotoma christhompsoni|[1277]|BIOUG09435-D12|BSE Malaise Trap|658[0n]|BOLD:ACL5703
Phanerotoma christhompsoni|[1278]|BIOUG09435-D11|BSE Malaise Trap|658[0n]|BOLD:ACL5703
Phanerotoma christhompsoni|[1279]|BIOUG17575-E09|BSE Malaise Trap|582[0n]|BOLD:ACL5703
Phanerotoma christhompsoni|[1280]|BIOUG08911-A03|BSE Malaise Trap|632[0n]|BOLD:ACL5703
Phanerotoma christhompsoni|[1281]|BIOUG18397-B01|BSE Malaise Trap|632[0n]|BOLD:ACL5703
Phanerotoma christhompsoni|[1282]|BIOUG05346-A11|BSE Malaise Trap|546[3n]|BOLD:ACL5703
Phanerotoma christhompsoni|[1283]|BIOUG17569-F01|BSE Malaise Trap|567[0n]|BOLD:ACL5703
Phanerotoma christhompsoni|[1284]|BIOUG18551-A08|BSE Malaise Trap|555[0n]|BOLD:ACL5703
Phanerotoma christhompsoni|[1285]|BIOUG17569-F07|BSE Malaise Trap|567[1n]|BOLD:ACL5703
Phanerotoma christhompsoni|[1286]|BIOUG10017-D06|BSE Malaise Trap|574[0n]|BOLD:ACL5703
Phanerotoma christhompsoni|[1287]|BIOUG17494-A10|BSE Malaise Trap|567[0n]|BOLD:ACL5703
Phanerotoma christhompsoni|[1288]|BIOUG05422-D07|BSE Malaise Trap|658[0n]|BOLD:ACL5703
Phanerotoma jenopappi|[1289]|BIOUG46564-D07|Malaise trap PL12-6B|654[0n]|BOLD:ADY2296
Phanerotoma almasolisae|[1290]|BIOUG29157-B02|Malaise trap PL12-6A|579[0n]|BOLD:ADB0692
Phanerotoma calixtomoragai|[1291]|DHJPAR0045952|malaise trap|658[0n]|BOLD:ABV9880
Pseudophanerotoma bobrobbinsi|[1292]|BIOUG29681-H06|Malaise trap PL12-3A|531[0n]|BOLD:ADB6487
Pseudophanerotoma bobrobbinsi|[1293]|BIOUG33410-G12|Derrumbe Malaise Trap|591[0n]|BOLD:ADB6487
Pseudophanerotoma bobrobbinsi|[1294]|BIOUG33411-C08|Derrumbe Malaise Trap|582[0n]|BOLD:ADB6487
Pseudophanerotoma bobrobbinsi|[1295]|BIOUG32982-B12|Derrumbe Malaise Trap|588[0n]|BOLD:ADB6487
Pseudophanerotoma bobrobbinsi|[1296]|BIOUG33411-D01|Derrumbe Malaise Trap|585[0n]|BOLD:ADB6487
Pseudophanerotoma bobrobbinsi|[1297]|BIOUG33275-C02|Derrumbe Malaise Trap|585[0n]|BOLD:ADB6487
Pseudophanerotoma bobrobbinsi|[1298]|BIOUG33275-D10|Derrumbe Malaise Trap|582[0n]|BOLD:ADB6487
Pseudophanerotoma bobrobbinsi|[1299]|BIOUG36496-E05|Derrumbe Malaise Trap|576[0n]|BOLD:ADB6487
Phanerotoma bernardoespinozai|[1300]|BIOUG08356-H07|BSE Malaise Trap|664[0n]|BOLD:ACJ8069
Phanerotoma bernardoespinozai|[1301]|BIOUG49562-D05|Malaise trap PL12-5B|653[0n]|BOLD:ACJ8069
Phanerotoma Janzen598|[1302]|BIOUG44707-G01|Malaise trap PL12-3B|653[0n]|BOLD:ADX0598
Phanerotoma Janzen598|[1303]|BIOUG44707-D06|Malaise trap PL12-3B|654[0n]|BOLD:ADX0598
Phanerotoma Janzen598|[1304]|BIOUG51524-H04|Malaise trap PL12-3C|640[0n]|BOLD:ADX0598
Phanerotoma Janzen598|[1305]|BIOUG44178-B03|Malaise trap PL12-3B|640[0n]|BOLD:ADX0598
Phanerotoma Janzen598|[1306]|BIOUG52293-H01|Malaise trap PL12-3C|651[0n]|BOLD:ADX0598
Phanerotoma carolinacanoae|[1307]|BIOUG50945-B01|Malaise trap PL12-5B|655[0n]|BOLD:ADB0776
Phanerotoma carolinacanoae|[1308]|BIOUG49774-F10|Malaise trap PL12-3B|654[0n]|BOLD:ADB0776
Phanerotoma carolinacanoae|[1309]|BIOUG28722-E11|Malaise trap PL12-9A|594[0n]|BOLD:ADB0776
Phanerotoma carolinacanoae|[1310]|BIOUG56238-G12|Malaise trap PL12-6D|641[0n]|BOLD:ADB0776
Phanerotoma duniagarciae|[1311]|BIOUG29456-B07|Malaise trap PL12-3A|588[0n]|BOLD:ADB2375
Phanerotoma duvalierbricenoi|[1312]|BIOUG30941-E12|Malaise trap PL12-2A|582[0n]|BOLD:ADD5784
Phanerotoma duvalierbricenoi|[1313]|BIOUG56492-E01|Malaise trap PL12-9D|640[0n]|BOLD:ADD5784
Phanerotoma eddysanchezi|[1314]|BIOUG18005-A02|BSE Malaise Trap|600[0n]|BOLD:AAW3563
Phanerotoma eddysanchezi|[1315]|DHJPAR0034287|Oryctometopia fossulatella|637[0n]|BOLD:AAW3563
Phanerotoma eddysanchezi|[1316]|DHJPAR0045461|Oryctometopia fossulatella|658[2n]|BOLD:AAW3563
Phanerotoma donquickei|[1317]|BIOUG18510-C09|BSE Malaise Trap|615[0n]|BOLD:ACS0581
Phanerotoma davidduthiei|[1318]|BIOUG10247-H11|BSE Malaise Trap|658[0n]|BOLD:ACL9696
Phanerotoma eldarayae|[1319]|BIOUG07919-D03|BSE Malaise Trap|661[0n]|BOLD:ACJ4087
Phanerotoma eliethcantillanoae|[1320]|DHJPAR0059458|phyBioLep01 BioLep17|631[0n]|BOLD:ADE1674
Phanerotoma Janzen30|[1321]|DHJPAR0029157|Chloropaschia Solis12|266[0n]
Phanerotoma Janzen2711|[1322]|DHJPAR0048177|Chloropaschia Janzen05|658[0n]|BOLD:AAE2711
Phanerotoma Janzen2711|[1323]|DHJPAR0029146|Chloropaschia Solis12|610[0n]|BOLD:AAE2711
Phanerotoma Janzen2711|[1324]|DHJPAR0029141|Chloropaschia Solis12|657[0n]|BOLD:AAE2711
Phanerotoma Janzen2711|[1325]|DHJPAR0048220|Chloropaschia Janzen05|658[0n]|BOLD:AAE2711
Phanerotoma Janzen2711|[1326]|DHJPAR0029140|Chloropaschia Solis12|657[0n]|BOLD:AAE2711
Phanerotoma Janzen2711|[1327]|DHJPAR0029145|Chloropaschia Solis12|657[0n]|BOLD:AAE2711
Phanerotoma Janzen2711|[1328]|DHJPAR0048069|Chloropaschia Janzen05|658[0n]|BOLD:AAE2711
Phanerotoma Janzen2711|[1329]|DHJPAR0048222|Chloropaschia Janzen05|658[0n]|BOLD:AAE2711
Phanerotoma Janzen2711|[1330]|DHJPAR0029136|Chloropaschia Solis12|657[0n]|BOLD:AAE2711
Phanerotoma Janzen2711|[1331]|DHJPAR0048212|Chloropaschia Janzen05|658[0n]|BOLD:AAE2711
Phanerotoma Janzen5678|[1332]|BIOUG46548-D08|Malaise trap PL12-6B|640[0n]|BOLD:ADY5678
Phanerotoma Janzen4152|[1333]|DHJPAR0052295|Antaeotricha spurca|632[0n]|BOLD:ACH4152
Phanerotoma dirksteinkei|[1334]|BIOUG13945-D01|BSE Malaise Trap|612[0n]|BOLD:ACA7259
Phanerotoma dirksteinkei|[1335]|BIOUG09826-A02|BSE Malaise Trap|605[0n]|BOLD:ACA7259
Phanerotoma dirksteinkei|[1336]|BIOUG09827-A01|BSE Malaise Trap|602[0n]|BOLD:ACA7259
Phanerotoma dirksteinkei|[1337]|BIOUG29209-A05|Malaise trap PL12-6A|600[0n]|BOLD:ACA7259
Phanerotoma dirksteinkei|[1338]|BIOUG17607-G02|BSE Malaise Trap|600[0n]|BOLD:ACA7259
Phanerotoma dirksteinkei|[1339]|BIOUG09826-A08|BSE Malaise Trap|603[0n]|BOLD:ACA7259
Phanerotoma dirksteinkei|[1340]|BIOUG18499-E11|BSE Malaise Trap|600[0n]|BOLD:ACA7259
Phanerotoma dirksteinkei|[1341]|BIOUG29327-C10|Malaise trap PL12-6A|582[0n]|BOLD:ACA7259
Phanerotoma dirksteinkei|[1342]|BIOUG50634-G02|Malaise trap PL12-5A|656[0n]|BOLD:ACA7259
Phanerotoma dirksteinkei|[1343]|BIOUG50894-F03|Malaise trap PL12-5B|652[0n]|BOLD:ACA7259
Phanerotoma dirksteinkei|[1344]|BIOUG50588-B12|Malaise trap PL12-6B|656[0n]|BOLD:ACA7259
Phanerotoma dirksteinkei|[1345]|BIOUG50945-C05|Malaise trap PL12-5B|655[0n]|BOLD:ACA7259
Phanerotoma dirksteinkei|[1346]|BIOUG50579-C05|Malaise trap PL12-6B|654[0n]|BOLD:ACA7259
Phanerotoma dirksteinkei|[1347]|BIOUG50716-H05|Malaise trap PL12-5A|653[0n]|BOLD:ACA7259
Phanerotoma dirksteinkei|[1348]|BIOUG50894-F04|Malaise trap PL12-5B|655[0n]|BOLD:ACA7259
Phanerotoma dirksteinkei|[1349]|BIOUG50718-A04|Malaise trap PL12-5A|654[0n]|BOLD:ACA7259
Phanerotoma dirksteinkei|[1350]|BIOUG50553-B12|Malaise trap PL12-6B|655[0n]|BOLD:ACA7259
Phanerotoma dirksteinkei|[1351]|BIOUG50894-D11|Malaise trap PL12-5B|653[0n]|BOLD:ACA7259
Phanerotoma dirksteinkei|[1352]|BIOUG51004-E01|Malaise trap PL12-5B|654[0n]|BOLD:ACA7259
Phanerotoma dirksteinkei|[1353]|BIOUG51004-E02|Malaise trap PL12-5B|653[0n]|BOLD:ACA7259
Phanerotoma dirksteinkei|[1354]|BIOUG50322-D06|Malaise trap PL12-6B|654[0n]|BOLD:ACA7259
Phanerotoma dirksteinkei|[1355]|BIOUG50894-G03|Malaise trap PL12-5B|652[0n]|BOLD:ACA7259
Phanerotoma dirksteinkei|[1356]|BIOUG53787-E06|Malaise trap PL12-9C|654[0n]|BOLD:ACA7259
Phanerotoma dirksteinkei|[1357]|BIOUG51828-E04|Malaise trap PL12-7B|655[0n]|BOLD:ACA7259
Phanerotoma dirksteinkei|[1358]|BIOUG50684-G11|Malaise trap PL12-5A|656[0n]|BOLD:ACA7259
Phanerotoma dirksteinkei|[1359]|BIOUG44748-E02|Malaise trap PL12-1B|653[0n]|BOLD:ACA7259
Phanerotoma dirksteinkei|[1360]|BIOUG50934-H08|Malaise trap PL12-5B|654[0n]|BOLD:ACA7259
Phanerotoma dirksteinkei|[1361]|BIOUG50716-H01|Malaise trap PL12-5A|654[0n]|BOLD:ACA7259
Phanerotoma dirksteinkei|[1362]|BIOUG50692-H10|Malaise trap PL12-5A|654[0n]|BOLD:ACA7259
Phanerotoma dirksteinkei|[1363]|BIOUG50945-B02|Malaise trap PL12-5B|654[0n]|BOLD:ACA7259
Phanerotoma dirksteinkei|[1364]|BIOUG50549-E06|Malaise trap PL12-5B|654[0n]|BOLD:ACA7259
Phanerotoma dirksteinkei|[1365]|BIOUG50480-A04|Malaise trap PL12-6B|654[0n]|BOLD:ACA7259
Phanerotoma dirksteinkei|[1366]|BIOUG50662-G09|Malaise trap PL12-5B|653[0n]|BOLD:ACA7259
Phanerotoma dirksteinkei|[1367]|BIOUG50894-B04|Malaise trap PL12-5B|654[0n]|BOLD:ACA7259
Phanerotoma dirksteinkei|[1368]|BIOUG50684-G02|Malaise trap PL12-5A|655[0n]|BOLD:ACA7259
Phanerotoma dirksteinkei|[1369]|BIOUG50934-G05|Malaise trap PL12-5B|654[0n]|BOLD:ACA7259
Phanerotoma dirksteinkei|[1370]|BIOUG50718-B04|Malaise trap PL12-5A|654[0n]|BOLD:ACA7259
Phanerotoma dirksteinkei|[1371]|BIOUG50718-B03|Malaise trap PL12-5A|654[0n]|BOLD:ACA7259
Phanerotoma dirksteinkei|[1372]|BIOUG51004-D12|Malaise trap PL12-5B|654[0n]|BOLD:ACA7259
Phanerotoma dirksteinkei|[1373]|BIOUG50718-A02|Malaise trap PL12-5A|653[0n]|BOLD:ACA7259
Phanerotoma dirksteinkei|[1374]|BIOUG50662-G07|Malaise trap PL12-5B|652[0n]|BOLD:ACA7259
Phanerotoma dirksteinkei|[1375]|BIOUG50557-F06|Malaise trap PL12-6B|654[0n]|BOLD:ACA7259

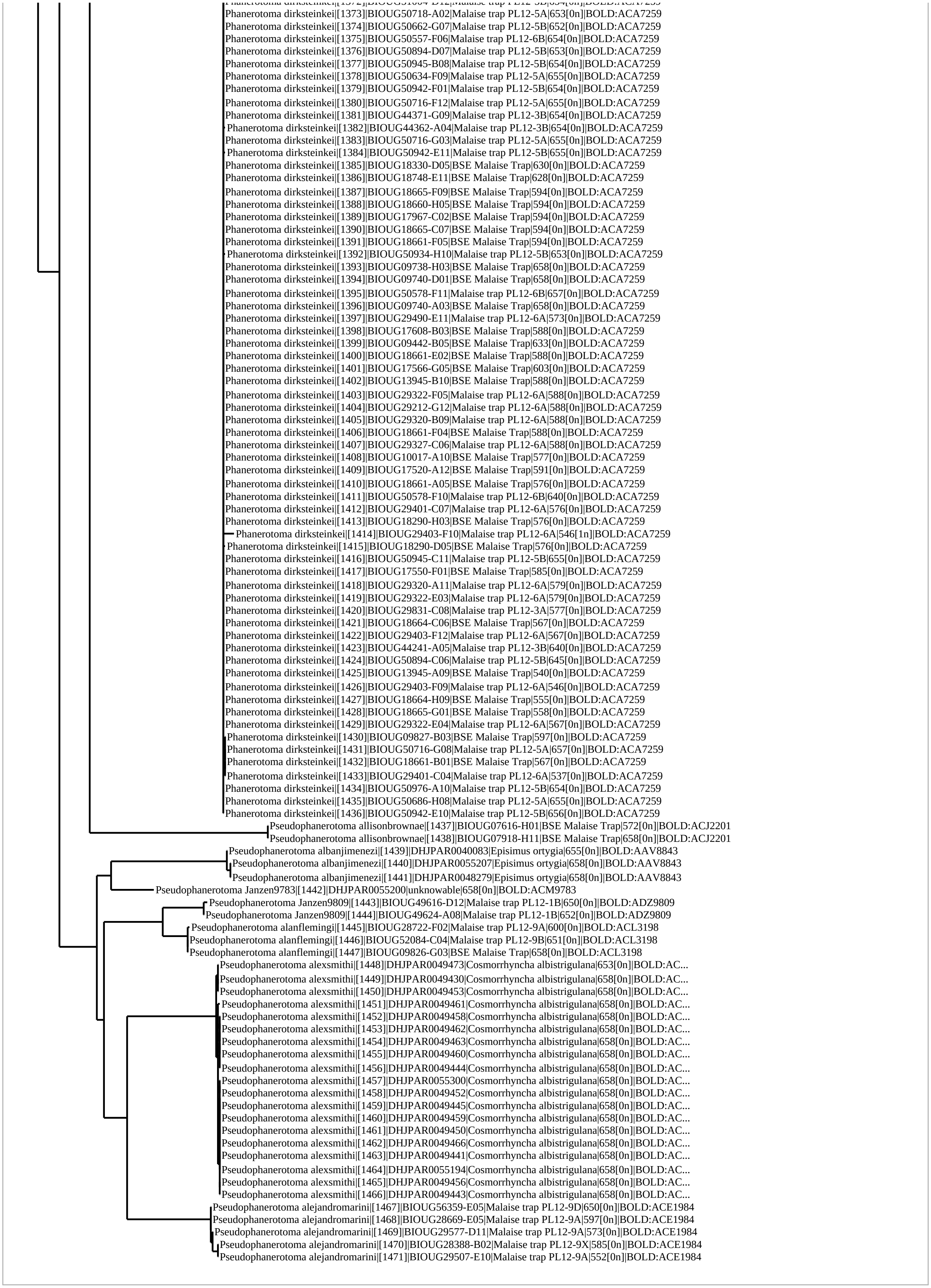
Phanerotoma dirksteinkei|[1372]|BIOUG51004-D12|Malaise trap PL12-5B|654[0n]|BOLD:ACA7259
Phanerotoma dirksteinkei|[1373]|BIOUG50718-A02|Malaise trap PL12-5A|653[0n]|BOLD:ACA7259
Phanerotoma dirksteinkei|[1374]|BIOUG50662-G07|Malaise trap PL12-5B|652[0n]|BOLD:ACA7259
Phanerotoma dirksteinkei|[1375]|BIOUG50557-F06|Malaise trap PL12-6B|654[0n]|BOLD:ACA7259
Phanerotoma dirksteinkei|[1376]|BIOUG50894-D07|Malaise trap PL12-5B|653[0n]|BOLD:ACA7259
Phanerotoma dirksteinkei|[1377]|BIOUG50945-B08|Malaise trap PL12-5B|654[0n]|BOLD:ACA7259
Phanerotoma dirksteinkei|[1378]|BIOUG50634-F09|Malaise trap PL12-5A|655[0n]|BOLD:ACA7259
Phanerotoma dirksteinkei|[1379]|BIOUG50942-F01|Malaise trap PL12-5B|654[0n]|BOLD:ACA7259
Phanerotoma dirksteinkei|[1380]|BIOUG50716-F12|Malaise trap PL12-5A|655[0n]|BOLD:ACA7259
Phanerotoma dirksteinkei|[1381]|BIOUG44371-G09|Malaise trap PL12-3B|654[0n]|BOLD:ACA7259
Phanerotoma dirksteinkei|[1382]|BIOUG44362-A04|Malaise trap PL12-3B|654[0n]|BOLD:ACA7259
Phanerotoma dirksteinkei|[1383]|BIOUG50716-G03|Malaise trap PL12-5A|655[0n]|BOLD:ACA7259
Phanerotoma dirksteinkei|[1384]|BIOUG50942-E11|Malaise trap PL12-5B|655[0n]|BOLD:ACA7259
Phanerotoma dirksteinkei|[1385]|BIOUG18330-D05|BSE Malaise Trap|630[0n]|BOLD:ACA7259
Phanerotoma dirksteinkei|[1386]|BIOUG18748-E11|BSE Malaise Trap|628[0n]|BOLD:ACA7259
Phanerotoma dirksteinkei|[1387]|BIOUG18665-F09|BSE Malaise Trap|594[0n]|BOLD:ACA7259
Phanerotoma dirksteinkei|[1388]|BIOUG18660-H05|BSE Malaise Trap|594[0n]|BOLD:ACA7259
Phanerotoma dirksteinkei|[1389]|BIOUG17967-C02|BSE Malaise Trap|594[0n]|BOLD:ACA7259
Phanerotoma dirksteinkei|[1390]|BIOUG18665-C07|BSE Malaise Trap|594[0n]|BOLD:ACA7259
Phanerotoma dirksteinkei|[1391]|BIOUG18661-F05|BSE Malaise Trap|594[0n]|BOLD:ACA7259
Phanerotoma dirksteinkei|[1392]|BIOUG50934-H10|Malaise trap PL12-5B|653[0n]|BOLD:ACA7259
Phanerotoma dirksteinkei|[1393]|BIOUG09738-H03|BSE Malaise Trap|658[0n]|BOLD:ACA7259
Phanerotoma dirksteinkei|[1394]|BIOUG09740-D01|BSE Malaise Trap|658[0n]|BOLD:ACA7259
Phanerotoma dirksteinkei|[1395]|BIOUG50578-F11|Malaise trap PL12-6B|657[0n]|BOLD:ACA7259
Phanerotoma dirksteinkei|[1396]|BIOUG09740-A03|BSE Malaise Trap|658[0n]|BOLD:ACA7259
Phanerotoma dirksteinkei|[1397]|BIOUG29490-E11|Malaise trap PL12-6A|573[0n]|BOLD:ACA7259
Phanerotoma dirksteinkei|[1398]|BIOUG17608-B03|BSE Malaise Trap|588[0n]|BOLD:ACA7259
Phanerotoma dirksteinkei|[1399]|BIOUG09442-B05|BSE Malaise Trap|633[0n]|BOLD:ACA7259
Phanerotoma dirksteinkei|[1400]|BIOUG18661-E02|BSE Malaise Trap|588[0n]|BOLD:ACA7259
Phanerotoma dirksteinkei|[1401]|BIOUG17566-G05|BSE Malaise Trap|603[0n]|BOLD:ACA7259
Phanerotoma dirksteinkei|[1402]|BIOUG13945-B10|BSE Malaise Trap|588[0n]|BOLD:ACA7259
Phanerotoma dirksteinkei|[1403]|BIOUG29322-F05|Malaise trap PL12-6A|588[0n]|BOLD:ACA7259
Phanerotoma dirksteinkei|[1404]|BIOUG29212-G12|Malaise trap PL12-6A|588[0n]|BOLD:ACA7259
Phanerotoma dirksteinkei|[1405]|BIOUG29320-B09|Malaise trap PL12-6A|588[0n]|BOLD:ACA7259
Phanerotoma dirksteinkei|[1406]|BIOUG18661-F04|BSE Malaise Trap|588[0n]|BOLD:ACA7259
Phanerotoma dirksteinkei|[1407]|BIOUG29327-C06|Malaise trap PL12-6A|588[0n]|BOLD:ACA7259
Phanerotoma dirksteinkei|[1408]|BIOUG10017-A10|BSE Malaise Trap|577[0n]|BOLD:ACA7259
Phanerotoma dirksteinkei|[1409]|BIOUG17520-A12|BSE Malaise Trap|591[0n]|BOLD:ACA7259
Phanerotoma dirksteinkei|[1410]|BIOUG18661-A05|BSE Malaise Trap|576[0n]|BOLD:ACA7259
Phanerotoma dirksteinkei|[1411]|BIOUG50578-F10|Malaise trap PL12-6B|640[0n]|BOLD:ACA7259
Phanerotoma dirksteinkei|[1412]|BIOUG29401-C07|Malaise trap PL12-6A|576[0n]|BOLD:ACA7259
Phanerotoma dirksteinkei|[1413]|BIOUG18290-H03|BSE Malaise Trap|576[0n]|BOLD:ACA7259
Phanerotoma dirksteinkei|[1414]|BIOUG29403-F10|Malaise trap PL12-6A|546[1n]|BOLD:ACA7259
Phanerotoma dirksteinkei|[1415]|BIOUG18290-D05|BSE Malaise Trap|576[0n]|BOLD:ACA7259
Phanerotoma dirksteinkei|[1416]|BIOUG50945-C11|Malaise trap PL12-5B|655[0n]|BOLD:ACA7259
Phanerotoma dirksteinkei|[1417]|BIOUG17550-F01|BSE Malaise Trap|585[0n]|BOLD:ACA7259
Phanerotoma dirksteinkei|[1418]|BIOUG29320-A11|Malaise trap PL12-6A|579[0n]|BOLD:ACA7259
Phanerotoma dirksteinkei|[1419]|BIOUG29322-E03|Malaise trap PL12-6A|579[0n]|BOLD:ACA7259
Phanerotoma dirksteinkei|[1420]|BIOUG29831-C08|Malaise trap PL12-3A|577[0n]|BOLD:ACA7259
Phanerotoma dirksteinkei|[1421]|BIOUG18664-C06|BSE Malaise Trap|567[0n]|BOLD:ACA7259
Phanerotoma dirksteinkei|[1422]|BIOUG29403-F12|Malaise trap PL12-6A|567[0n]|BOLD:ACA7259
Phanerotoma dirksteinkei|[1423]|BIOUG44241-A05|Malaise trap PL12-3B|640[0n]|BOLD:ACA7259
Phanerotoma dirksteinkei|[1424]|BIOUG50894-C06|Malaise trap PL12-5B|645[0n]|BOLD:ACA7259
Phanerotoma dirksteinkei|[1425]|BIOUG13945-A09|BSE Malaise Trap|540[0n]|BOLD:ACA7259
Phanerotoma dirksteinkei|[1426]|BIOUG29403-F09|Malaise trap PL12-6A|546[0n]|BOLD:ACA7259
Phanerotoma dirksteinkei|[1427]|BIOUG18664-H09|BSE Malaise Trap|555[0n]|BOLD:ACA7259
Phanerotoma dirksteinkei|[1428]|BIOUG18665-G01|BSE Malaise Trap|558[0n]|BOLD:ACA7259
Phanerotoma dirksteinkei|[1429]|BIOUG29322-E04|Malaise trap PL12-6A|567[0n]|BOLD:ACA7259
Phanerotoma dirksteinkei|[1430]|BIOUG09827-B03|BSE Malaise Trap|597[0n]|BOLD:ACA7259
Phanerotoma dirksteinkei|[1431]|BIOUG50716-G08|Malaise trap PL12-5A|657[0n]|BOLD:ACA7259
Phanerotoma dirksteinkei|[1432]|BIOUG18661-B01|BSE Malaise Trap|567[0n]|BOLD:ACA7259
Phanerotoma dirksteinkei|[1433]|BIOUG29401-C04|Malaise trap PL12-6A|537[0n]|BOLD:ACA7259
Phanerotoma dirksteinkei|[1434]|BIOUG50976-A10|Malaise trap PL12-5B|654[0n]|BOLD:ACA7259
Phanerotoma dirksteinkei|[1435]|BIOUG50686-H08|Malaise trap PL12-5A|655[0n]|BOLD:ACA7259
Phanerotoma dirksteinkei|[1436]|BIOUG50942-E10|Malaise trap PL12-5B|656[0n]|BOLD:ACA7259
Pseudophanerotoma allisonbrownae|[1437]|BIOUG07616-H01|BSE Malaise Trap|572[0n]|BOLD:ACJ2201
Pseudophanerotoma allisonbrownae|[1438]|BIOUG07918-H11|BSE Malaise Trap|658[0n]|BOLD:ACJ2201
Pseudophanerotoma albanjimenezi|[1439]|DHJPAR0040083|Episimus ortygia|655[0n]|BOLD:AAV8843
Pseudophanerotoma albanjimenezi|[1440]|DHJPAR0055207|Episimus ortygia|658[0n]|BOLD:AAV8843
Pseudophanerotoma albanjimenezi|[1441]|DHJPAR0048279|Episimus ortygia|658[0n]|BOLD:AAV8843
Pseudophanerotoma Janzen9783|[1442]|DHJPAR0055200|unknowable|658[0n]|BOLD:ACM9783
Pseudophanerotoma Janzen9809|[1443]|BIOUG49616-D12|Malaise trap PL12-1B|650[0n]|BOLD:ADZ9809
Pseudophanerotoma Janzen9809|[1444]|BIOUG49624-A08|Malaise trap PL12-1B|652[0n]|BOLD:ADZ9809
Pseudophanerotoma alanflemingi|[1445]|BIOUG28722-F02|Malaise trap PL12-9A|600[0n]|BOLD:ACL3198
Pseudophanerotoma alanflemingi|[1446]|BIOUG52084-C04|Malaise trap PL12-9B|651[0n]|BOLD:ACL3198
Pseudophanerotoma alanflemingi|[1447]|BIOUG09826-G03|BSE Malaise Trap|658[0n]|BOLD:ACL3198
Pseudophanerotoma alexsmithi|[1448]|DHJPAR0049473|Cosmorrhyncha albistrigulana|653[0n]|BOLD:AC...
Pseudophanerotoma alexsmithi|[1449]|DHJPAR0049430|Cosmorrhyncha albistrigulana|658[0n]|BOLD:AC...
Pseudophanerotoma alexsmithi|[1450]|DHJPAR0049453|Cosmorrhyncha albistrigulana|658[0n]|BOLD:AC...
Pseudophanerotoma alexsmithi|[1451]|DHJPAR0049461|Cosmorrhyncha albistrigulana|658[0n]|BOLD:AC...
Pseudophanerotoma alexsmithi|[1452]|DHJPAR0049458|Cosmorrhyncha albistrigulana|658[0n]|BOLD:AC...
Pseudophanerotoma alexsmithi|[1453]|DHJPAR0049462|Cosmorrhyncha albistrigulana|658[0n]|BOLD:AC...
Pseudophanerotoma alexsmithi|[1454]|DHJPAR0049463|Cosmorrhyncha albistrigulana|658[0n]|BOLD:AC...
Pseudophanerotoma alexsmithi|[1455]|DHJPAR0049460|Cosmorrhyncha albistrigulana|658[0n]|BOLD:AC...
Pseudophanerotoma alexsmithi|[1456]|DHJPAR0049444|Cosmorrhyncha albistrigulana|658[0n]|BOLD:AC...
Pseudophanerotoma alexsmithi|[1457]|DHJPAR0055300|Cosmorrhyncha albistrigulana|658[0n]|BOLD:AC...
Pseudophanerotoma alexsmithi|[1458]|DHJPAR0049452|Cosmorrhyncha albistrigulana|658[0n]|BOLD:AC...
Pseudophanerotoma alexsmithi|[1459]|DHJPAR0049445|Cosmorrhyncha albistrigulana|658[0n]|BOLD:AC...
Pseudophanerotoma alexsmithi|[1460]|DHJPAR0049459|Cosmorrhyncha albistrigulana|658[0n]|BOLD:AC...
Pseudophanerotoma alexsmithi|[1461]|DHJPAR0049450|Cosmorrhyncha albistrigulana|658[0n]|BOLD:AC...
Pseudophanerotoma alexsmithi|[1462]|DHJPAR0049466|Cosmorrhyncha albistrigulana|658[0n]|BOLD:AC...
Pseudophanerotoma alexsmithi|[1463]|DHJPAR0049441|Cosmorrhyncha albistrigulana|658[0n]|BOLD:AC...
Pseudophanerotoma alexsmithi|[1464]|DHJPAR0055194|Cosmorrhyncha albistrigulana|658[0n]|BOLD:AC...
Pseudophanerotoma alexsmithi|[1465]|DHJPAR0049456|Cosmorrhyncha albistrigulana|658[0n]|BOLD:AC...
Pseudophanerotoma alexsmithi|[1466]|DHJPAR0049443|Cosmorrhyncha albistrigulana|658[0n]|BOLD:AC...
Pseudophanerotoma alejandromarini|[1467]|BIOUG56359-E05|Malaise trap PL12-9D|650[0n]|BOLD:ACE1984
Pseudophanerotoma alejandromarini|[1468]|BIOUG28669-E05|Malaise trap PL12-9A|597[0n]|BOLD:ACE1984
Pseudophanerotoma alejandromarini|[1469]|BIOUG29577-D11|Malaise trap PL12-9A|573[0n]|BOLD:ACE1984
Pseudophanerotoma alejandromarini|[1470]|BIOUG28388-B02|Malaise trap PL12-9X|585[0n]|BOLD:ACE1984
Pseudophanerotoma alejandromarini|[1471]|BIOUG29507-E10|Malaise trap PL12-9A|552[0n]|BOLD:ACE1984
