# Supplementary material for: Minimalist revision and description of 403 new species in 11 subfamilies of Costa Rican braconid parasitoid wasps, including host records for 219 species
Source: Zookeys. 2021 Feb 2;1013:1–665. doi: 10.3897/zookeys.1013.55600 (PMC8390796; doi:10.3897/zookeys.1013.55600)
Supplement: Supplementary material 4 — Homolobinae [file zookeys-1013-001-s004.pdf]

4. Homolobinae BOLD TaxonID Tree

Title : Tree Result - Search: Sample IDs (99 records returned) (99 records selected)

Date : 17-Nov-2020

Data Type : Nucleotide

Distance Model : Kimura 2 Parameter

Marker : COI-5P

Colourization : [blue]=Stop Codons [red]=Contamination or misidentification

Label : Sample ID

Label : Taxon

Label : Extra Info

Label : Sequence Length

Label : Barcode Cluster (BIN)

Filter : exclude records with stop codons

Sequence Count : 90

Species count : 6

Genus count : 2

Family count : 1

Unidentified : 0

BIN Count : 6

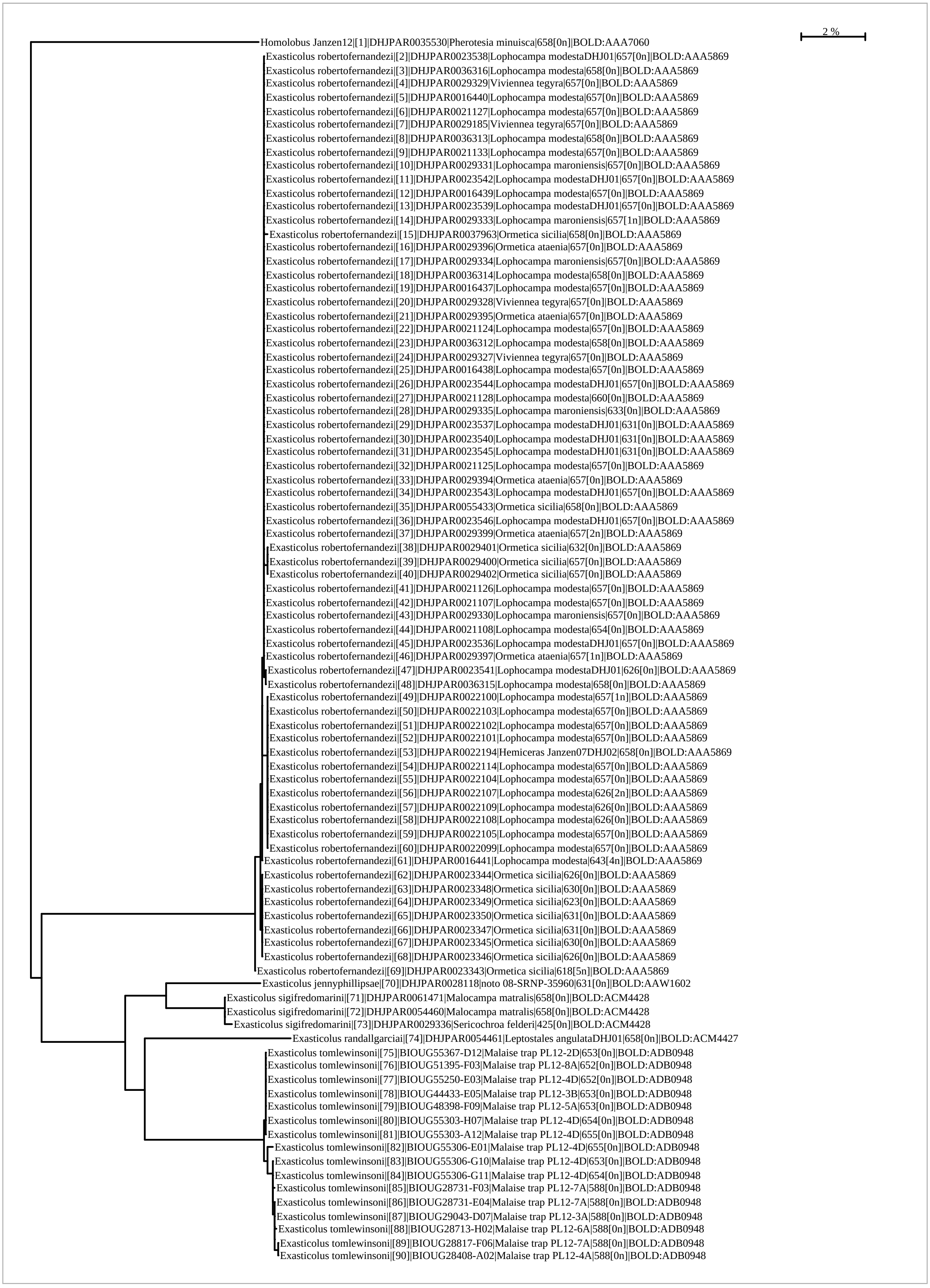
2 %
Homolobus Janzen12|[1]|DHJPAR0035530|Pherotesia minuisca|658[0n]|BOLD:AAA7060
Exasticolus robertofernandezi|[2]|DHJPAR0023538|Lophocampa modestaDHJ01|657[0n]|BOLD:AAA5869
Exasticolus robertofernandezi|[3]|DHJPAR0036316|Lophocampa modesta|658[0n]|BOLD:AAA5869
Exasticolus robertofernandezi|[4]|DHJPAR0029329|Viviennea tegyra|657[0n]|BOLD:AAA5869
Exasticolus robertofernandezi|[5]|DHJPAR0016440|Lophocampa modesta|657[0n]|BOLD:AAA5869
Exasticolus robertofernandezi|[6]|DHJPAR0021127|Lophocampa modesta|657[0n]|BOLD:AAA5869
Exasticolus robertofernandezi|[7]|DHJPAR0029185|Viviennea tegyra|657[0n]|BOLD:AAA5869
Exasticolus robertofernandezi|[8]|DHJPAR0036313|Lophocampa modesta|658[0n]|BOLD:AAA5869
Exasticolus robertofernandezi|[9]|DHJPAR0021133|Lophocampa modesta|657[0n]|BOLD:AAA5869
Exasticolus robertofernandezi|[10]|DHJPAR0029331|Lophocampa maroniensis|657[0n]|BOLD:AAA5869
Exasticolus robertofernandezi|[11]|DHJPAR0023542|Lophocampa modestaDHJ01|657[0n]|BOLD:AAA5869
Exasticolus robertofernandezi|[12]|DHJPAR0016439|Lophocampa modesta|657[0n]|BOLD:AAA5869
Exasticolus robertofernandezi|[13]|DHJPAR0023539|Lophocampa modestaDHJ01|657[0n]|BOLD:AAA5869
Exasticolus robertofernandezi|[14]|DHJPAR0029333|Lophocampa maroniensis|657[1n]|BOLD:AAA5869
Exasticolus robertofernandezi|[15]|DHJPAR0037963|Ormetica sicilia|658[0n]|BOLD:AAA5869
Exasticolus robertofernandezi|[16]|DHJPAR0029396|Ormetica ataenia|657[0n]|BOLD:AAA5869
Exasticolus robertofernandezi|[17]|DHJPAR0029334|Lophocampa maroniensis|657[0n]|BOLD:AAA5869
Exasticolus robertofernandezi|[18]|DHJPAR0036314|Lophocampa modesta|658[0n]|BOLD:AAA5869
Exasticolus robertofernandezi|[19]|DHJPAR0016437|Lophocampa modesta|657[0n]|BOLD:AAA5869
Exasticolus robertofernandezi|[20]|DHJPAR0029328|Viviennea tegyra|657[0n]|BOLD:AAA5869
Exasticolus robertofernandezi|[21]|DHJPAR0029395|Ormetica ataenia|657[0n]|BOLD:AAA5869
Exasticolus robertofernandezi|[22]|DHJPAR0021124|Lophocampa modesta|657[0n]|BOLD:AAA5869
Exasticolus robertofernandezi|[23]|DHJPAR0036312|Lophocampa modesta|658[0n]|BOLD:AAA5869
Exasticolus robertofernandezi|[24]|DHJPAR0029327|Viviennea tegyra|657[0n]|BOLD:AAA5869
Exasticolus robertofernandezi|[25]|DHJPAR0016438|Lophocampa modesta|657[0n]|BOLD:AAA5869
Exasticolus robertofernandezi|[26]|DHJPAR0023544|Lophocampa modestaDHJ01|657[0n]|BOLD:AAA5869
Exasticolus robertofernandezi|[27]|DHJPAR0021128|Lophocampa modesta|660[0n]|BOLD:AAA5869
Exasticolus robertofernandezi|[28]|DHJPAR0029335|Lophocampa maroniensis|633[0n]|BOLD:AAA5869
Exasticolus robertofernandezi|[29]|DHJPAR0023537|Lophocampa modestaDHJ01|631[0n]|BOLD:AAA5869
Exasticolus robertofernandezi|[30]|DHJPAR0023540|Lophocampa modestaDHJ01|631[0n]|BOLD:AAA5869
Exasticolus robertofernandezi|[31]|DHJPAR0023545|Lophocampa modestaDHJ01|631[0n]|BOLD:AAA5869
Exasticolus robertofernandezi|[32]|DHJPAR0021125|Lophocampa modesta|657[0n]|BOLD:AAA5869
Exasticolus robertofernandezi|[33]|DHJPAR0029394|Ormetica ataenia|657[0n]|BOLD:AAA5869
Exasticolus robertofernandezi|[34]|DHJPAR0023543|Lophocampa modestaDHJ01|657[0n]|BOLD:AAA5869
Exasticolus robertofernandezi|[35]|DHJPAR0055433|Ormetica sicilia|658[0n]|BOLD:AAA5869
Exasticolus robertofernandezi|[36]|DHJPAR0023546|Lophocampa modestaDHJ01|657[0n]|BOLD:AAA5869
Exasticolus robertofernandezi|[37]|DHJPAR0029399|Ormetica ataenia|657[2n]|BOLD:AAA5869
Exasticolus robertofernandezi|[38]|DHJPAR0029401|Ormetica sicilia|632[0n]|BOLD:AAA5869
Exasticolus robertofernandezi|[39]|DHJPAR0029400|Ormetica sicilia|657[0n]|BOLD:AAA5869
Exasticolus robertofernandezi|[40]|DHJPAR0029402|Ormetica sicilia|657[0n]|BOLD:AAA5869
Exasticolus robertofernandezi|[41]|DHJPAR0021126|Lophocampa modesta|657[0n]|BOLD:AAA5869
Exasticolus robertofernandezi|[42]|DHJPAR0021107|Lophocampa modesta|657[0n]|BOLD:AAA5869
Exasticolus robertofernandezi|[43]|DHJPAR0029330|Lophocampa maroniensis|657[0n]|BOLD:AAA5869
Exasticolus robertofernandezi|[44]|DHJPAR0021108|Lophocampa modesta|654[0n]|BOLD:AAA5869
Exasticolus robertofernandezi|[45]|DHJPAR0023536|Lophocampa modestaDHJ01|657[0n]|BOLD:AAA5869
Exasticolus robertofernandezi|[46]|DHJPAR0029397|Ormetica ataenia|657[1n]|BOLD:AAA5869
Exasticolus robertofernandezi|[47]|DHJPAR0023541|Lophocampa modestaDHJ01|626[0n]|BOLD:AAA5869
Exasticolus robertofernandezi|[48]|DHJPAR0036315|Lophocampa modesta|658[0n]|BOLD:AAA5869
Exasticolus robertofernandezi|[49]|DHJPAR0022100|Lophocampa modesta|657[1n]|BOLD:AAA5869
Exasticolus robertofernandezi|[50]|DHJPAR0022103|Lophocampa modesta|657[0n]|BOLD:AAA5869
Exasticolus robertofernandezi|[51]|DHJPAR0022102|Lophocampa modesta|657[0n]|BOLD:AAA5869
Exasticolus robertofernandezi|[52]|DHJPAR0022101|Lophocampa modesta|657[0n]|BOLD:AAA5869
Exasticolus robertofernandezi|[53]|DHJPAR0022194|Hemiceras Janzen07DHJ02|658[0n]|BOLD:AAA5869
Exasticolus robertofernandezi|[54]|DHJPAR0022114|Lophocampa modesta|657[0n]|BOLD:AAA5869
Exasticolus robertofernandezi|[55]|DHJPAR0022104|Lophocampa modesta|657[0n]|BOLD:AAA5869
Exasticolus robertofernandezi|[56]|DHJPAR0022107|Lophocampa modesta|626[2n]|BOLD:AAA5869
Exasticolus robertofernandezi|[57]|DHJPAR0022109|Lophocampa modesta|626[0n]|BOLD:AAA5869
Exasticolus robertofernandezi|[58]|DHJPAR0022108|Lophocampa modesta|626[0n]|BOLD:AAA5869
Exasticolus robertofernandezi|[59]|DHJPAR0022105|Lophocampa modesta|657[0n]|BOLD:AAA5869
Exasticolus robertofernandezi|[60]|DHJPAR0022099|Lophocampa modesta|657[0n]|BOLD:AAA5869
Exasticolus robertofernandezi|[61]|DHJPAR0016441|Lophocampa modesta|643[4n]|BOLD:AAA5869
Exasticolus robertofernandezi|[62]|DHJPAR0023344|Ormetica sicilia|626[0n]|BOLD:AAA5869
Exasticolus robertofernandezi|[63]|DHJPAR0023348|Ormetica sicilia|630[0n]|BOLD:AAA5869
Exasticolus robertofernandezi|[64]|DHJPAR0023349|Ormetica sicilia|623[0n]|BOLD:AAA5869
Exasticolus robertofernandezi|[65]|DHJPAR0023350|Ormetica sicilia|631[0n]|BOLD:AAA5869
Exasticolus robertofernandezi|[66]|DHJPAR0023347|Ormetica sicilia|631[0n]|BOLD:AAA5869
Exasticolus robertofernandezi|[67]|DHJPAR0023345|Ormetica sicilia|630[0n]|BOLD:AAA5869
Exasticolus robertofernandezi|[68]|DHJPAR0023346|Ormetica sicilia|626[0n]|BOLD:AAA5869
Exasticolus robertofernandezi|[69]|DHJPAR0023343|Ormetica sicilia|618[5n]|BOLD:AAA5869
Exasticolus jennyphillipsae|[70]|DHJPAR0028118|noto 08-SRNP-35960|631[0n]|BOLD:AAW1602
Exasticolus sigifredomarini|[71]|DHJPAR0061471|Malocampa matralis|658[0n]|BOLD:ACM4428
Exasticolus sigifredomarini|[72]|DHJPAR0054460|Malocampa matralis|658[0n]|BOLD:ACM4428
Exasticolus sigifredomarini|[73]|DHJPAR0029336|Sericochroa felderi|425[0n]|BOLD:ACM4428
Exasticolus randallgarciai|[74]|DHJPAR0054461|Leptostales angulataDHJ01|658[0n]|BOLD:ACM4427
Exasticolus tomlewinsoni|[75]|BIOUG55367-D12|Malaise trap PL12-2D|653[0n]|BOLD:ADB0948
Exasticolus tomlewinsoni|[76]|BIOUG51395-F03|Malaise trap PL12-8A|652[0n]|BOLD:ADB0948
Exasticolus tomlewinsoni|[77]|BIOUG55250-E03|Malaise trap PL12-4D|652[0n]|BOLD:ADB0948
Exasticolus tomlewinsoni|[78]|BIOUG44433-E05|Malaise trap PL12-3B|653[0n]|BOLD:ADB0948
Exasticolus tomlewinsoni|[79]|BIOUG48398-F09|Malaise trap PL12-5A|653[0n]|BOLD:ADB0948
Exasticolus tomlewinsoni|[80]|BIOUG55303-H07|Malaise trap PL12-4D|654[0n]|BOLD:ADB0948
Exasticolus tomlewinsoni|[81]|BIOUG55303-A12|Malaise trap PL12-4D|655[0n]|BOLD:ADB0948
Exasticolus tomlewinsoni|[82]|BIOUG55306-E01|Malaise trap PL12-4D|655[0n]|BOLD:ADB0948
Exasticolus tomlewinsoni|[83]|BIOUG55306-G10|Malaise trap PL12-4D|653[0n]|BOLD:ADB0948
Exasticolus tomlewinsoni|[84]|BIOUG55306-G11|Malaise trap PL12-4D|654[0n]|BOLD:ADB0948
Exasticolus tomlewinsoni|[85]|BIOUG28731-F03|Malaise trap PL12-7A|588[0n]|BOLD:ADB0948
Exasticolus tomlewinsoni|[86]|BIOUG28731-E04|Malaise trap PL12-7A|588[0n]|BOLD:ADB0948
Exasticolus tomlewinsoni|[87]|BIOUG29043-D07|Malaise trap PL12-3A|588[0n]|BOLD:ADB0948
Exasticolus tomlewinsoni|[88]|BIOUG28713-H02|Malaise trap PL12-6A|588[0n]|BOLD:ADB0948
Exasticolus tomlewinsoni|[89]|BIOUG28817-F06|Malaise trap PL12-7A|588[0n]|BOLD:ADB0948
Exasticolus tomlewinsoni|[90]|BIOUG28408-A02|Malaise trap PL12-4A|588[0n]|BOLD:ADB0948
