# Supplementary material for: Minimalist revision and description of 403 new species in 11 subfamilies of Costa Rican braconid parasitoid wasps, including host records for 219 species
Source: Zookeys. 2021 Feb 2;1013:1–665. doi: 10.3897/zookeys.1013.55600 (PMC8390796; doi:10.3897/zookeys.1013.55600)
Supplement: Supplementary material 5 — Hormiinae [file zookeys-1013-001-s005.pdf]

5. Hormiinae BOLD TaxonID Tree

Title : Tree Result - Search: Sample IDs (488 records returned) (488 records selected)

Date : 17-Nov-2020

Data Type : Nucleotide

Distance Model : Kimura 2 Parameter

Marker : COI-5P

Colourization : [blue]=Stop Codons [red]=Contamination or misidentification

Label : Sample ID

Label : Taxon

Label : Extra Info

Label : Sequence Length

Label : Barcode Cluster (BIN)

Sequence Count : 455

Species count : 53

Genus count : 2

Family count : 1

Unidentified : 12

BIN Count : 53

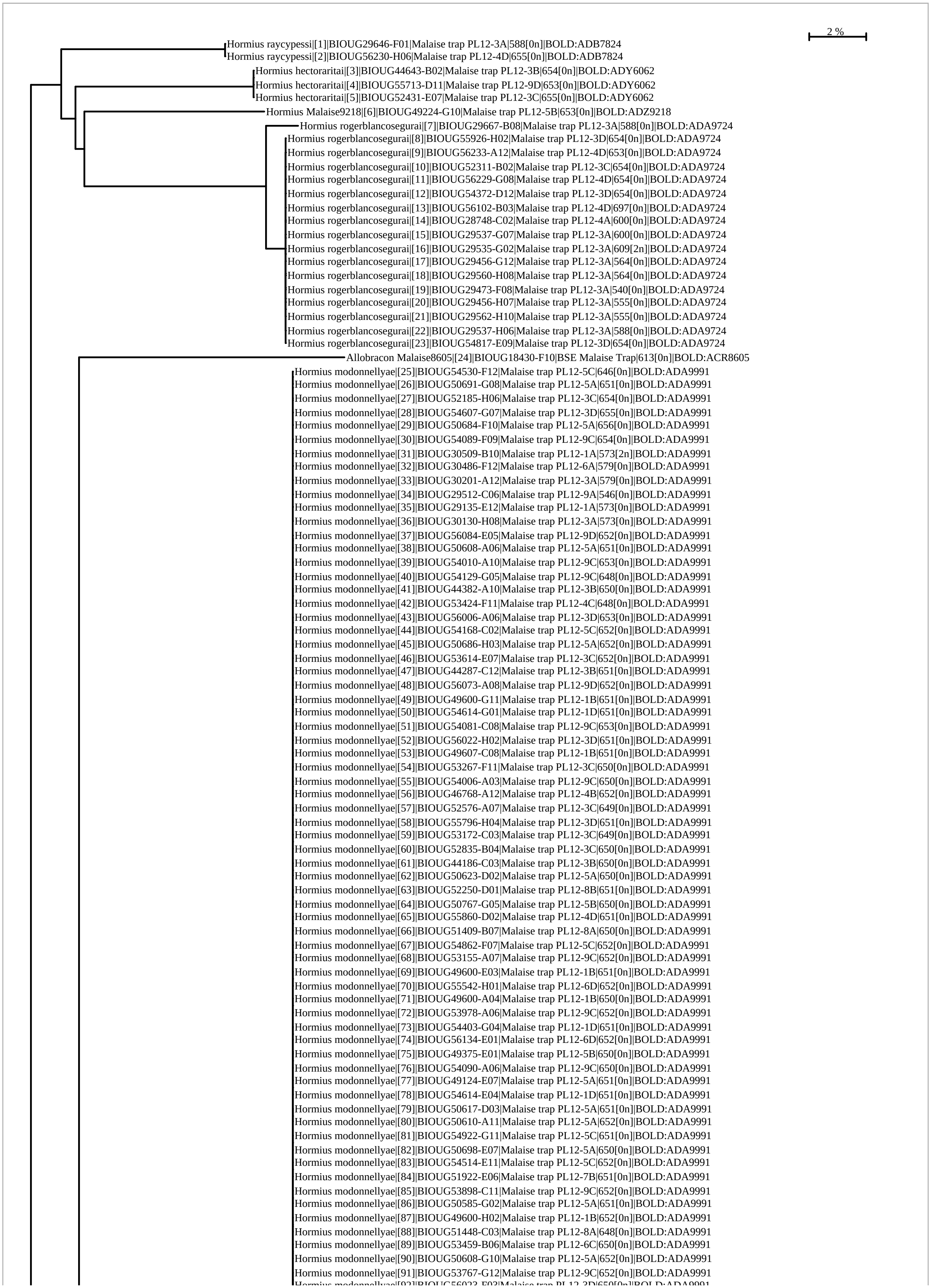
2 %
Hormius raycypessi|[1]|BIOUG29646-F01|Malaise trap PL12-3A|588[0n]|BOLD:ADB7824
Hormius raycypessi|[2]|BIOUG56230-H06|Malaise trap PL12-4D|655[0n]|BOLD:ADB7824
Hormius hectoraritai|[3]|BIOUG44643-B02|Malaise trap PL12-3B|654[0n]|BOLD:ADY6062
Hormius hectoraritai|[4]|BIOUG55713-D11|Malaise trap PL12-9D|653[0n]|BOLD:ADY6062
Hormius hectoraritai|[5]|BIOUG52431-E07|Malaise trap PL12-3C|655[0n]|BOLD:ADY6062
Hormius Malaise9218|[6]|BIOUG49224-G10|Malaise trap PL12-5B|653[0n]|BOLD:ADZ9218
Hormius rogerblancosegurai|[7]|BIOUG29667-B08|Malaise trap PL12-3A|588[0n]|BOLD:ADA9724
Hormius rogerblancosegurai|[8]|BIOUG55926-H02|Malaise trap PL12-3D|654[0n]|BOLD:ADA9724
Hormius rogerblancosegurai|[9]|BIOUG56233-A12|Malaise trap PL12-4D|653[0n]|BOLD:ADA9724
Hormius rogerblancosegurai|[10]|BIOUG52311-B02|Malaise trap PL12-3C|654[0n]|BOLD:ADA9724
Hormius rogerblancosegurai|[11]|BIOUG56229-G08|Malaise trap PL12-4D|654[0n]|BOLD:ADA9724
Hormius rogerblancosegurai|[12]|BIOUG54372-D12|Malaise trap PL12-3D|654[0n]|BOLD:ADA9724
Hormius rogerblancosegurai|[13]|BIOUG56102-B03|Malaise trap PL12-4D|697[0n]|BOLD:ADA9724
Hormius rogerblancosegurai|[14]|BIOUG28748-C02|Malaise trap PL12-4A|600[0n]|BOLD:ADA9724
Hormius rogerblancosegurai|[15]|BIOUG29537-G07|Malaise trap PL12-3A|600[0n]|BOLD:ADA9724
Hormius rogerblancosegurai|[16]|BIOUG29535-G02|Malaise trap PL12-3A|609[2n]|BOLD:ADA9724
Hormius rogerblancosegurai|[17]|BIOUG29456-G12|Malaise trap PL12-3A|564[0n]|BOLD:ADA9724
Hormius rogerblancosegurai|[18]|BIOUG29560-H08|Malaise trap PL12-3A|564[0n]|BOLD:ADA9724
Hormius rogerblancosegurai|[19]|BIOUG29473-F08|Malaise trap PL12-3A|540[0n]|BOLD:ADA9724
Hormius rogerblancosegurai|[20]|BIOUG29456-H07|Malaise trap PL12-3A|555[0n]|BOLD:ADA9724
Hormius rogerblancosegurai|[21]|BIOUG29562-H10|Malaise trap PL12-3A|555[0n]|BOLD:ADA9724
Hormius rogerblancosegurai|[22]|BIOUG29537-H06|Malaise trap PL12-3A|588[0n]|BOLD:ADA9724
Hormius rogerblancosegurai|[23]|BIOUG54817-E09|Malaise trap PL12-3D|654[0n]|BOLD:ADA9724
Allobracon Malaise8605|[24]|BIOUG18430-F10|BSE Malaise Trap|613[0n]|BOLD:ACR8605
Hormius modonnellyae|[25]|BIOUG54530-F12|Malaise trap PL12-5C|646[0n]|BOLD:ADA9991
Hormius modonnellyae|[26]|BIOUG50691-G08|Malaise trap PL12-5A|651[0n]|BOLD:ADA9991
Hormius modonnellyae|[27]|BIOUG52185-H06|Malaise trap PL12-3C|654[0n]|BOLD:ADA9991
Hormius modonnellyae|[28]|BIOUG54607-G07|Malaise trap PL12-3D|655[0n]|BOLD:ADA9991
Hormius modonnellyae|[29]|BIOUG50684-F10|Malaise trap PL12-5A|656[0n]|BOLD:ADA9991
Hormius modonnellyae|[30]|BIOUG54089-F09|Malaise trap PL12-9C|654[0n]|BOLD:ADA9991
Hormius modonnellyae|[31]|BIOUG30509-B10|Malaise trap PL12-1A|573[2n]|BOLD:ADA9991
Hormius modonnellyae|[32]|BIOUG30486-F12|Malaise trap PL12-6A|579[0n]|BOLD:ADA9991
Hormius modonnellyae|[33]|BIOUG30201-A12|Malaise trap PL12-3A|579[0n]|BOLD:ADA9991
Hormius modonnellyae|[34]|BIOUG29512-C06|Malaise trap PL12-9A|546[0n]|BOLD:ADA9991
Hormius modonnellyae|[35]|BIOUG29135-E12|Malaise trap PL12-1A|573[0n]|BOLD:ADA9991
Hormius modonnellyae|[36]|BIOUG30130-H08|Malaise trap PL12-3A|573[0n]|BOLD:ADA9991
Hormius modonnellyae|[37]|BIOUG56084-E05|Malaise trap PL12-9D|652[0n]|BOLD:ADA9991
Hormius modonnellyae|[38]|BIOUG50608-A06|Malaise trap PL12-5A|651[0n]|BOLD:ADA9991
Hormius modonnellyae|[39]|BIOUG54010-A10|Malaise trap PL12-9C|653[0n]|BOLD:ADA9991
Hormius modonnellyae|[40]|BIOUG54129-G05|Malaise trap PL12-9C|648[0n]|BOLD:ADA9991
Hormius modonnellyae|[41]|BIOUG44382-A10|Malaise trap PL12-3B|650[0n]|BOLD:ADA9991
Hormius modonnellyae|[42]|BIOUG53424-F11|Malaise trap PL12-4C|648[0n]|BOLD:ADA9991
Hormius modonnellyae|[43]|BIOUG56006-A06|Malaise trap PL12-3D|653[0n]|BOLD:ADA9991
Hormius modonnellyae|[44]|BIOUG54168-C02|Malaise trap PL12-5C|652[0n]|BOLD:ADA9991
Hormius modonnellyae|[45]|BIOUG50686-H03|Malaise trap PL12-5A|652[0n]|BOLD:ADA9991
Hormius modonnellyae|[46]|BIOUG53614-E07|Malaise trap PL12-3C|652[0n]|BOLD:ADA9991
Hormius modonnellyae|[47]|BIOUG44287-C12|Malaise trap PL12-3B|651[0n]|BOLD:ADA9991
Hormius modonnellyae|[48]|BIOUG56073-A08|Malaise trap PL12-9D|652[0n]|BOLD:ADA9991
Hormius modonnellyae|[49]|BIOUG49600-G11|Malaise trap PL12-1B|651[0n]|BOLD:ADA9991
Hormius modonnellyae|[50]|BIOUG54614-G01|Malaise trap PL12-1D|651[0n]|BOLD:ADA9991
Hormius modonnellyae|[51]|BIOUG54081-C08|Malaise trap PL12-9C|653[0n]|BOLD:ADA9991
Hormius modonnellyae|[52]|BIOUG56022-H02|Malaise trap PL12-3D|651[0n]|BOLD:ADA9991
Hormius modonnellyae|[53]|BIOUG49607-C08|Malaise trap PL12-1B|651[0n]|BOLD:ADA9991
Hormius modonnellyae|[54]|BIOUG53267-F11|Malaise trap PL12-3C|650[0n]|BOLD:ADA9991
Hormius modonnellyae|[55]|BIOUG54006-A03|Malaise trap PL12-9C|650[0n]|BOLD:ADA9991
Hormius modonnellyae|[56]|BIOUG46768-A12|Malaise trap PL12-4B|652[0n]|BOLD:ADA9991
Hormius modonnellyae|[57]|BIOUG52576-A07|Malaise trap PL12-3C|649[0n]|BOLD:ADA9991
Hormius modonnellyae|[58]|BIOUG55796-H04|Malaise trap PL12-3D|651[0n]|BOLD:ADA9991
Hormius modonnellyae|[59]|BIOUG53172-C03|Malaise trap PL12-3C|649[0n]|BOLD:ADA9991
Hormius modonnellyae|[60]|BIOUG52835-B04|Malaise trap PL12-3C|650[0n]|BOLD:ADA9991
Hormius modonnellyae|[61]|BIOUG44186-C03|Malaise trap PL12-3B|650[0n]|BOLD:ADA9991
Hormius modonnellyae|[62]|BIOUG50623-D02|Malaise trap PL12-5A|650[0n]|BOLD:ADA9991
Hormius modonnellyae|[63]|BIOUG52250-D01|Malaise trap PL12-8B|651[0n]|BOLD:ADA9991
Hormius modonnellyae|[64]|BIOUG50767-G05|Malaise trap PL12-5B|650[0n]|BOLD:ADA9991
Hormius modonnellyae|[65]|BIOUG55860-D02|Malaise trap PL12-4D|651[0n]|BOLD:ADA9991
Hormius modonnellyae|[66]|BIOUG51409-B07|Malaise trap PL12-8A|650[0n]|BOLD:ADA9991
Hormius modonnellyae|[67]|BIOUG54862-F07|Malaise trap PL12-5C|652[0n]|BOLD:ADA9991
Hormius modonnellyae|[68]|BIOUG53155-A07|Malaise trap PL12-9C|652[0n]|BOLD:ADA9991
Hormius modonnellyae|[69]|BIOUG49600-E03|Malaise trap PL12-1B|651[0n]|BOLD:ADA9991
Hormius modonnellyae|[70]|BIOUG55542-H01|Malaise trap PL12-6D|652[0n]|BOLD:ADA9991
Hormius modonnellyae|[71]|BIOUG49600-A04|Malaise trap PL12-1B|650[0n]|BOLD:ADA9991
Hormius modonnellyae|[72]|BIOUG53978-A06|Malaise trap PL12-9C|652[0n]|BOLD:ADA9991
Hormius modonnellyae|[73]|BIOUG54403-G04|Malaise trap PL12-1D|651[0n]|BOLD:ADA9991
Hormius modonnellyae|[74]|BIOUG56134-E01|Malaise trap PL12-6D|652[0n]|BOLD:ADA9991
Hormius modonnellyae|[75]|BIOUG49375-E01|Malaise trap PL12-5B|650[0n]|BOLD:ADA9991
Hormius modonnellyae|[76]|BIOUG54090-A06|Malaise trap PL12-9C|650[0n]|BOLD:ADA9991
Hormius modonnellyae|[77]|BIOUG49124-E07|Malaise trap PL12-5A|651[0n]|BOLD:ADA9991
Hormius modonnellyae|[78]|BIOUG54614-E04|Malaise trap PL12-1D|651[0n]|BOLD:ADA9991
Hormius modonnellyae|[79]|BIOUG50617-D03|Malaise trap PL12-5A|651[0n]|BOLD:ADA9991
Hormius modonnellyae|[80]|BIOUG50610-A11|Malaise trap PL12-5A|652[0n]|BOLD:ADA9991
Hormius modonnellyae|[81]|BIOUG54922-G11|Malaise trap PL12-5C|651[0n]|BOLD:ADA9991
Hormius modonnellyae|[82]|BIOUG50698-E07|Malaise trap PL12-5A|650[0n]|BOLD:ADA9991
Hormius modonnellyae|[83]|BIOUG54514-E11|Malaise trap PL12-5C|652[0n]|BOLD:ADA9991
Hormius modonnellyae|[84]|BIOUG51922-E06|Malaise trap PL12-7B|651[0n]|BOLD:ADA9991
Hormius modonnellyae|[85]|BIOUG53898-C11|Malaise trap PL12-9C|652[0n]|BOLD:ADA9991
Hormius modonnellyae|[86]|BIOUG50585-G02|Malaise trap PL12-5A|651[0n]|BOLD:ADA9991
Hormius modonnellyae|[87]|BIOUG49600-H02|Malaise trap PL12-1B|652[0n]|BOLD:ADA9991
Hormius modonnellyae|[88]|BIOUG51448-C03|Malaise trap PL12-8A|648[0n]|BOLD:ADA9991
Hormius modonnellyae|[89]|BIOUG53459-B06|Malaise trap PL12-6C|650[0n]|BOLD:ADA9991
Hormius modonnellyae|[90]|BIOUG50608-G10|Malaise trap PL12-5A|652[0n]|BOLD:ADA9991
Hormius modonnellyae|[91]|BIOUG53767-G12|Malaise trap PL12-9C|652[0n]|BOLD:ADA9991

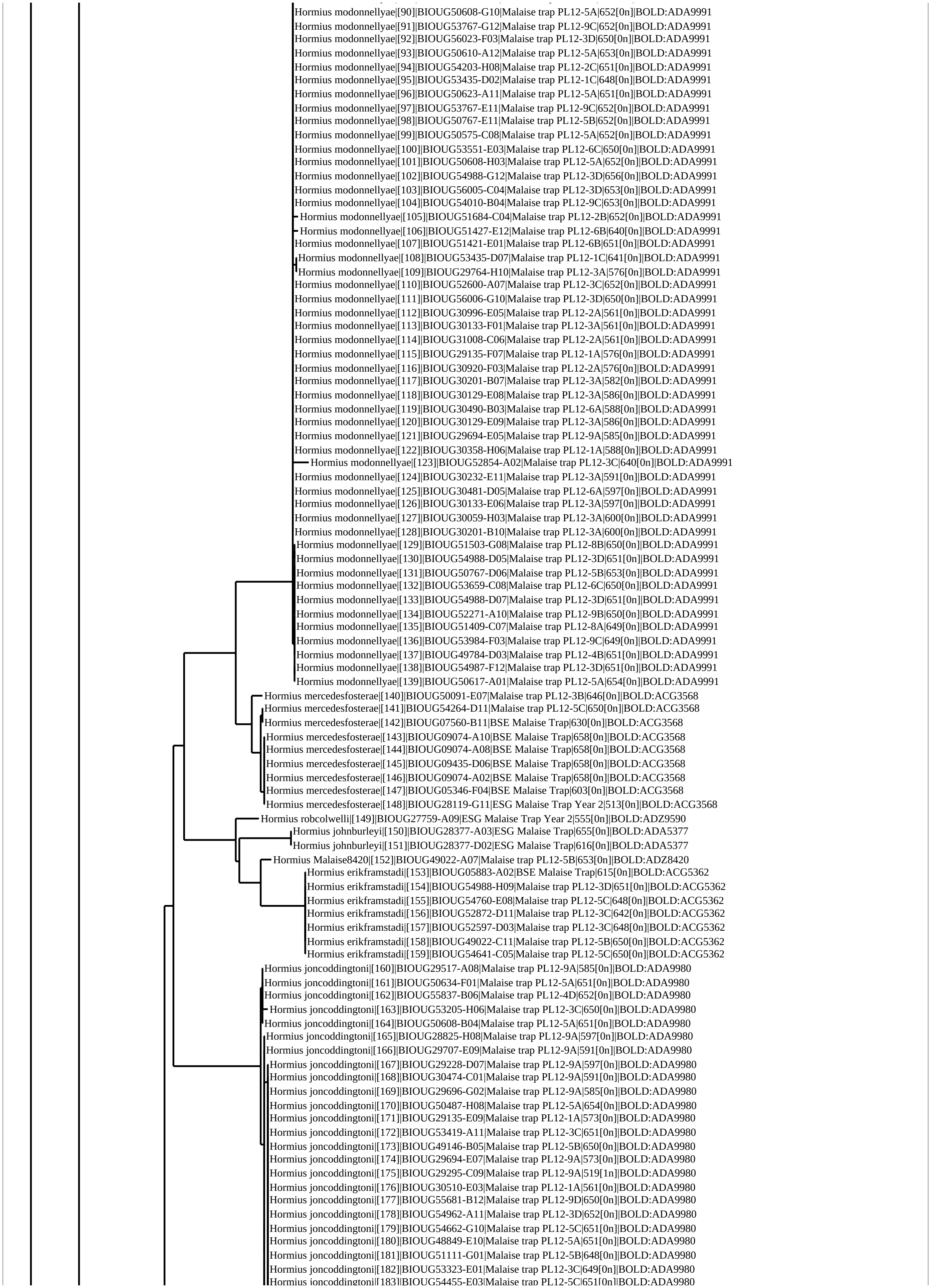

Hormius modonnellyae|[90]|BIOUG50608-G10|Malaise trap PL12-5A|652[0n]|BOLD:ADA9991
Hormius modonnellyae|[91]|BIOUG53767-G12|Malaise trap PL12-9C|652[0n]|BOLD:ADA9991
Hormius modonnellyae|[92]|BIOUG56023-F03|Malaise trap PL12-3D|650[0n]|BOLD:ADA9991
Hormius modonnellyae|[93]|BIOUG50610-A12|Malaise trap PL12-5A|653[0n]|BOLD:ADA9991
Hormius modonnellyae|[94]|BIOUG54203-H08|Malaise trap PL12-2C|651[0n]|BOLD:ADA9991
Hormius modonnellyae|[95]|BIOUG53435-D02|Malaise trap PL12-1C|648[0n]|BOLD:ADA9991
Hormius modonnellyae|[96]|BIOUG50623-A11|Malaise trap PL12-5A|651[0n]|BOLD:ADA9991
Hormius modonnellyae|[97]|BIOUG53767-E11|Malaise trap PL12-9C|652[0n]|BOLD:ADA9991
Hormius modonnellyae|[98]|BIOUG50767-E11|Malaise trap PL12-5B|652[0n]|BOLD:ADA9991
Hormius modonnellyae|[99]|BIOUG50575-C08|Malaise trap PL12-5A|652[0n]|BOLD:ADA9991
Hormius modonnellyae|[100]|BIOUG53551-E03|Malaise trap PL12-6C|650[0n]|BOLD:ADA9991
Hormius modonnellyae|[101]|BIOUG50608-H03|Malaise trap PL12-5A|652[0n]|BOLD:ADA9991
Hormius modonnellyae|[102]|BIOUG54988-G12|Malaise trap PL12-3D|656[0n]|BOLD:ADA9991
Hormius modonnellyae|[103]|BIOUG56005-C04|Malaise trap PL12-3D|653[0n]|BOLD:ADA9991
Hormius modonnellyae|[104]|BIOUG54010-B04|Malaise trap PL12-9C|653[0n]|BOLD:ADA9991
Hormius modonnellyae|[105]|BIOUG51684-C04|Malaise trap PL12-2B|652[0n]|BOLD:ADA9991
Hormius modonnellyae|[106]|BIOUG51427-E12|Malaise trap PL12-6B|640[0n]|BOLD:ADA9991
Hormius modonnellyae|[107]|BIOUG51421-E01|Malaise trap PL12-6B|651[0n]|BOLD:ADA9991
Hormius modonnellyae|[108]|BIOUG53435-D07|Malaise trap PL12-1C|641[0n]|BOLD:ADA9991
Hormius modonnellyae|[109]|BIOUG29764-H10|Malaise trap PL12-3A|576[0n]|BOLD:ADA9991
Hormius modonnellyae|[110]|BIOUG52600-A07|Malaise trap PL12-3C|652[0n]|BOLD:ADA9991
Hormius modonnellyae|[111]|BIOUG56006-G10|Malaise trap PL12-3D|650[0n]|BOLD:ADA9991
Hormius modonnellyae|[112]|BIOUG30996-E05|Malaise trap PL12-2A|561[0n]|BOLD:ADA9991
Hormius modonnellyae|[113]|BIOUG30133-F01|Malaise trap PL12-3A|561[0n]|BOLD:ADA9991
Hormius modonnellyae|[114]|BIOUG31008-C06|Malaise trap PL12-2A|561[0n]|BOLD:ADA9991
Hormius modonnellyae|[115]|BIOUG29135-F07|Malaise trap PL12-1A|576[0n]|BOLD:ADA9991
Hormius modonnellyae|[116]|BIOUG30920-F03|Malaise trap PL12-2A|576[0n]|BOLD:ADA9991
Hormius modonnellyae|[117]|BIOUG30201-B07|Malaise trap PL12-3A|582[0n]|BOLD:ADA9991
Hormius modonnellyae|[118]|BIOUG30129-E08|Malaise trap PL12-3A|586[0n]|BOLD:ADA9991
Hormius modonnellyae|[119]|BIOUG30490-B03|Malaise trap PL12-6A|588[0n]|BOLD:ADA9991
Hormius modonnellyae|[120]|BIOUG30129-E09|Malaise trap PL12-3A|586[0n]|BOLD:ADA9991
Hormius modonnellyae|[121]|BIOUG29694-E05|Malaise trap PL12-9A|585[0n]|BOLD:ADA9991
Hormius modonnellyae|[122]|BIOUG30358-H06|Malaise trap PL12-1A|588[0n]|BOLD:ADA9991
Hormius modonnellyae|[123]|BIOUG52854-A02|Malaise trap PL12-3C|640[0n]|BOLD:ADA9991
Hormius modonnellyae|[124]|BIOUG30232-E11|Malaise trap PL12-3A|591[0n]|BOLD:ADA9991
Hormius modonnellyae|[125]|BIOUG30481-D05|Malaise trap PL12-6A|597[0n]|BOLD:ADA9991
Hormius modonnellyae|[126]|BIOUG30133-E06|Malaise trap PL12-3A|597[0n]|BOLD:ADA9991
Hormius modonnellyae|[127]|BIOUG30059-H03|Malaise trap PL12-3A|600[0n]|BOLD:ADA9991
Hormius modonnellyae|[128]|BIOUG30201-B10|Malaise trap PL12-3A|600[0n]|BOLD:ADA9991
Hormius modonnellyae|[129]|BIOUG51503-G08|Malaise trap PL12-8B|650[0n]|BOLD:ADA9991
Hormius modonnellyae|[130]|BIOUG54988-D05|Malaise trap PL12-3D|651[0n]|BOLD:ADA9991
Hormius modonnellyae|[131]|BIOUG50767-D06|Malaise trap PL12-5B|653[0n]|BOLD:ADA9991
Hormius modonnellyae|[132]|BIOUG53659-C08|Malaise trap PL12-6C|650[0n]|BOLD:ADA9991
Hormius modonnellyae|[133]|BIOUG54988-D07|Malaise trap PL12-3D|651[0n]|BOLD:ADA9991
Hormius modonnellyae|[134]|BIOUG52271-A10|Malaise trap PL12-9B|650[0n]|BOLD:ADA9991
Hormius modonnellyae|[135]|BIOUG51409-C07|Malaise trap PL12-8A|649[0n]|BOLD:ADA9991
Hormius modonnellyae|[136]|BIOUG53984-F03|Malaise trap PL12-9C|649[0n]|BOLD:ADA9991
Hormius modonnellyae|[137]|BIOUG49784-D03|Malaise trap PL12-4B|651[0n]|BOLD:ADA9991
Hormius modonnellyae|[138]|BIOUG54987-F12|Malaise trap PL12-3D|651[0n]|BOLD:ADA9991
Hormius modonnellyae|[139]|BIOUG50617-A01|Malaise trap PL12-5A|654[0n]|BOLD:ADA9991
Hormius mercedesfosterae|[140]|BIOUG50091-E07|Malaise trap PL12-3B|646[0n]|BOLD:ACG3568
Hormius mercedesfosterae|[141]|BIOUG54264-D11|Malaise trap PL12-5C|650[0n]|BOLD:ACG3568
Hormius mercedesfosterae|[142]|BIOUG07560-B11|BSE Malaise Trap|630[0n]|BOLD:ACG3568
Hormius mercedesfosterae|[143]|BIOUG09074-A10|BSE Malaise Trap|658[0n]|BOLD:ACG3568
Hormius mercedesfosterae|[144]|BIOUG09074-A08|BSE Malaise Trap|658[0n]|BOLD:ACG3568
Hormius mercedesfosterae|[145]|BIOUG09435-D06|BSE Malaise Trap|658[0n]|BOLD:ACG3568
Hormius mercedesfosterae|[146]|BIOUG09074-A02|BSE Malaise Trap|658[0n]|BOLD:ACG3568
Hormius mercedesfosterae|[147]|BIOUG05346-F04|BSE Malaise Trap|603[0n]|BOLD:ACG3568
Hormius mercedesfosterae|[148]|BIOUG28119-G11|ESG Malaise Trap Year 2|513[0n]|BOLD:ACG3568
Hormius robcolwelli|[149]|BIOUG27759-A09|ESG Malaise Trap Year 2|555[0n]|BOLD:ADZ9590
Hormius johnburleyi|[150]|BIOUG28377-A03|ESG Malaise Trap|655[0n]|BOLD:ADA5377
Hormius johnburleyi|[151]|BIOUG28377-D02|ESG Malaise Trap|616[0n]|BOLD:ADA5377
Hormius Malaise8420|[152]|BIOUG49022-A07|Malaise trap PL12-5B|653[0n]|BOLD:ADZ8420
Hormius erikframstadi|[153]|BIOUG05883-A02|BSE Malaise Trap|615[0n]|BOLD:ACG5362
Hormius erikframstadi|[154]|BIOUG54988-H09|Malaise trap PL12-3D|651[0n]|BOLD:ACG5362
Hormius erikframstadi|[155]|BIOUG54760-E08|Malaise trap PL12-5C|648[0n]|BOLD:ACG5362
Hormius erikframstadi|[156]|BIOUG52872-D11|Malaise trap PL12-3C|642[0n]|BOLD:ACG5362
Hormius erikframstadi|[157]|BIOUG52597-D03|Malaise trap PL12-3C|648[0n]|BOLD:ACG5362
Hormius erikframstadi|[158]|BIOUG49022-C11|Malaise trap PL12-5B|650[0n]|BOLD:ACG5362
Hormius erikframstadi|[159]|BIOUG54641-C05|Malaise trap PL12-5C|650[0n]|BOLD:ACG5362
Hormius joncoddingtoni|[160]|BIOUG29517-A08|Malaise trap PL12-9A|585[0n]|BOLD:ADA9980
Hormius joncoddingtoni|[161]|BIOUG50634-F01|Malaise trap PL12-5A|651[0n]|BOLD:ADA9980
Hormius joncoddingtoni|[162]|BIOUG55837-B06|Malaise trap PL12-4D|652[0n]|BOLD:ADA9980
Hormius joncoddingtoni|[163]|BIOUG53205-H06|Malaise trap PL12-3C|650[0n]|BOLD:ADA9980
Hormius joncoddingtoni|[164]|BIOUG50608-B04|Malaise trap PL12-5A|651[0n]|BOLD:ADA9980
Hormius joncoddingtoni|[165]|BIOUG28825-H08|Malaise trap PL12-9A|597[0n]|BOLD:ADA9980
Hormius joncoddingtoni|[166]|BIOUG29707-E09|Malaise trap PL12-9A|591[0n]|BOLD:ADA9980
Hormius joncoddingtoni|[167]|BIOUG29228-D07|Malaise trap PL12-9A|597[0n]|BOLD:ADA9980
Hormius joncoddingtoni|[168]|BIOUG30474-C01|Malaise trap PL12-9A|591[0n]|BOLD:ADA9980
Hormius joncoddingtoni|[169]|BIOUG29696-G02|Malaise trap PL12-9A|585[0n]|BOLD:ADA9980
Hormius joncoddingtoni|[170]|BIOUG50487-H08|Malaise trap PL12-5A|654[0n]|BOLD:ADA9980
Hormius joncoddingtoni|[171]|BIOUG29135-E09|Malaise trap PL12-1A|573[0n]|BOLD:ADA9980
Hormius joncoddingtoni|[172]|BIOUG53419-A11|Malaise trap PL12-3C|651[0n]|BOLD:ADA9980
Hormius joncoddingtoni|[173]|BIOUG49146-B05|Malaise trap PL12-5B|650[0n]|BOLD:ADA9980
Hormius joncoddingtoni|[174]|BIOUG29694-E07|Malaise trap PL12-9A|573[0n]|BOLD:ADA9980
Hormius joncoddingtoni|[175]|BIOUG29295-C09|Malaise trap PL12-9A|519[1n]|BOLD:ADA9980
Hormius joncoddingtoni|[176]|BIOUG30510-E03|Malaise trap PL12-1A|561[0n]|BOLD:ADA9980
Hormius joncoddingtoni|[177]|BIOUG55681-B12|Malaise trap PL12-9D|650[0n]|BOLD:ADA9980
Hormius joncoddingtoni|[178]|BIOUG54962-A11|Malaise trap PL12-3D|652[0n]|BOLD:ADA9980
Hormius joncoddingtoni|[179]|BIOUG54662-G10|Malaise trap PL12-5C|651[0n]|BOLD:ADA9980
Hormius joncoddingtoni|[180]|BIOUG48849-E10|Malaise trap PL12-5A|651[0n]|BOLD:ADA9980
Hormius joncoddingtoni|[181]|BIOUG51111-G01|Malaise trap PL12-5B|648[0n]|BOLD:ADA9980
Hormius joncoddingtoni|[182]|BIOUG53323-E01|Malaise trap PL12-3C|649[0n]|BOLD:ADA9980
Hormius joncoddingtoni|[183]|BIOUG54455-E03|Malaise trap PL12-5C|651[0n]|BOLD:ADA9980

Hormius joncoddingtoni|[181]|BIOUG51111-G01|Malaise trap PL12-5B|648[0n]|BOLD:ADA9980
Hormius joncoddingtoni|[182]|BIOUG53323-E01|Malaise trap PL12-3C|649[0n]|BOLD:ADA9980
Hormius joncoddingtoni|[183]|BIOUG54455-E03|Malaise trap PL12-5C|651[0n]|BOLD:ADA9980
Hormius joncoddingtoni|[184]|BIOUG53025-B11|Malaise trap PL12-3C|650[0n]|BOLD:ADA9980
Hormius joncoddingtoni|[185]|BIOUG54711-A11|Malaise trap PL12-3D|651[0n]|BOLD:ADA9980
Hormius joncoddingtoni|[186]|BIOUG50700-E01|Malaise trap PL12-5A|652[0n]|BOLD:ADA9980
Hormius joncoddingtoni|[187]|BIOUG49584-A05|Malaise trap PL12-5B|650[0n]|BOLD:ADA9980
Hormius joncoddingtoni|[188]|BIOUG29973-F01|Malaise trap PL12-3A|585[0n]|BOLD:ADA9980
Hormius joncoddingtoni|[189]|BIOUG29599-D02|Malaise trap PL12-9A|582[0n]|BOLD:ADA9980
Hormius joncoddingtoni|[190]|BIOUG53280-E09|Malaise trap PL12-3C|647[0n]|BOLD:ADA9980
Hormius joncoddingtoni|[191]|BIOUG54337-H09|Malaise trap PL12-5C|641[0n]|BOLD:ADA9980
Hormius joncoddingtoni|[192]|BIOUG53271-H05|Malaise trap PL12-3C|640[0n]|BOLD:ADA9980
Hormius joncoddingtoni|[193]|BIOUG53391-C09|Malaise trap PL12-3C|640[0n]|BOLD:ADA9980
Hormius joncoddingtoni|[194]|BIOUG52833-B10|Malaise trap PL12-3C|652[0n]|BOLD:ADA9980
Hormius joncoddingtoni|[195]|BIOUG49784-B09|Malaise trap PL12-4B|652[0n]|BOLD:ADA9980
Hormius joncoddingtoni|[196]|BIOUG54081-D02|Malaise trap PL12-9C|651[0n]|BOLD:ADA9980
Hormius joncoddingtoni|[197]|BIOUG53873-H09|Malaise trap PL12-9C|649[0n]|BOLD:ADA9980
Hormius joncoddingtoni|[198]|BIOUG54454-F01|Malaise trap PL12-5C|651[0n]|BOLD:ADA9980
Hormius joncoddingtoni|[199]|BIOUG56351-E08|Malaise trap PL12-9D|648[0n]|BOLD:ADA9980
Hormius joncoddingtoni|[200]|BIOUG52999-H11|Malaise trap PL12-3C|650[0n]|BOLD:ADA9980
Hormius joncoddingtoni|[201]|BIOUG56057-H09|Malaise trap PL12-3D|652[0n]|BOLD:ADA9980
Hormius joncoddingtoni|[202]|BIOUG53444-F10|Malaise trap PL12-3C|651[0n]|BOLD:ADA9980
Hormius joncoddingtoni|[203]|BIOUG29696-F02|Malaise trap PL12-9A|564[0n]|BOLD:ADA9980
Hormius joncoddingtoni|[204]|BIOUG53269-F10|Malaise trap PL12-3C|647[0n]|BOLD:ADA9980
Hormius joncoddingtoni|[205]|BIOUG55077-H03|Malaise trap PL12-1D|651[0n]|BOLD:ADA9980
Hormius joncoddingtoni|[206]|BIOUG50629-G09|Malaise trap PL12-5A|652[0n]|BOLD:ADA9980
Hormius joncoddingtoni|[207]|BIOUG50610-B07|Malaise trap PL12-5A|650[0n]|BOLD:ADA9980
Hormius joncoddingtoni|[208]|BIOUG51698-C07|Malaise trap PL12-2B|652[0n]|BOLD:ADA9980
Hormius joncoddingtoni|[209]|BIOUG53391-B07|Malaise trap PL12-3C|649[0n]|BOLD:ADA9980
Hormius joncoddingtoni|[210]|BIOUG54076-F05|Malaise trap PL12-9C|649[0n]|BOLD:ADA9980
Hormius joncoddingtoni|[211]|BIOUG29667-G07|Malaise trap PL12-3A|597[0n]|BOLD:ADA9980
Hormius joncoddingtoni|[212]|BIOUG29707-E10|Malaise trap PL12-9A|585[0n]|BOLD:ADA9980
Hormius joncoddingtoni|[213]|BIOUG29689-B07|Malaise trap PL12-3A|585[0n]|BOLD:ADA9980
Hormius joncoddingtoni|[214]|BIOUG29330-G03|Malaise trap PL12-4A|585[0n]|BOLD:ADA9980
Hormius joncoddingtoni|[215]|BIOUG29696-G05|Malaise trap PL12-9A|585[0n]|BOLD:ADA9980
Hormius joncoddingtoni|[216]|BIOUG29696-E09|Malaise trap PL12-9A|585[0n]|BOLD:ADA9980
Hormius joncoddingtoni|[217]|BIOUG29903-F04|Malaise trap PL12-3A|540[1n]|BOLD:ADA9980
Hormius joncoddingtoni|[218]|BIOUG29576-B06|Malaise trap PL12-9A|582[0n]|BOLD:ADA9980
Hormius joncoddingtoni|[219]|BIOUG29603-E07|Malaise trap PL12-9A|583[0n]|BOLD:ADA9980
Hormius joncoddingtoni|[220]|BIOUG29884-G12|Malaise trap PL12-3A|579[0n]|BOLD:ADA9980
Hormius joncoddingtoni|[221]|BIOUG29332-H06|Malaise trap PL12-4A|576[0n]|BOLD:ADA9980
Hormius joncoddingtoni|[222]|BIOUG49263-H08|Malaise trap PL12-5B|651[0n]|BOLD:ADA9980
Hormius joncoddingtoni|[223]|BIOUG50652-B10|Malaise trap PL12-5A|654[0n]|BOLD:ADA9980
Hormius joncoddingtoni|[224]|BIOUG52835-D06|Malaise trap PL12-3C|654[0n]|BOLD:ADA9980
Hormius joncoddingtoni|[225]|BIOUG54053-D09|Malaise trap PL12-9C|652[0n]|BOLD:ADA9980
Hormius joncoddingtoni|[226]|BIOUG50610-A05|Malaise trap PL12-5A|651[0n]|BOLD:ADA9980
Hormius joncoddingtoni|[227]|BIOUG52758-F02|Malaise trap PL12-3C|652[0n]|BOLD:ADA9980
Hormius joncoddingtoni|[228]|BIOUG52835-E04|Malaise trap PL12-3C|650[0n]|BOLD:ADA9980
Hormius joncoddingtoni|[229]|BIOUG52835-E05|Malaise trap PL12-3C|649[0n]|BOLD:ADA9980
Hormius joncoddingtoni|[230]|BIOUG53191-H10|Malaise trap PL12-3C|650[0n]|BOLD:ADA9980
Hormius joncoddingtoni|[231]|BIOUG49185-A09|Malaise trap PL12-5B|651[0n]|BOLD:ADA9980
Hormius joncoddingtoni|[232]|BIOUG48852-D02|Malaise trap PL12-5A|650[0n]|BOLD:ADA9980
Hormius joncoddingtoni|[233]|BIOUG53242-E10|Malaise trap PL12-3C|650[0n]|BOLD:ADA9980
Hormius joncoddingtoni|[234]|BIOUG50726-D09|Malaise trap PL12-5A|651[0n]|BOLD:ADA9980
Hormius joncoddingtoni|[235]|BIOUG50652-G06|Malaise trap PL12-5A|652[0n]|BOLD:ADA9980
Hormius joncoddingtoni|[236]|BIOUG53391-A01|Malaise trap PL12-3C|650[0n]|BOLD:ADA9980
Hormius joncoddingtoni|[237]|BIOUG50608-F11|Malaise trap PL12-5A|652[0n]|BOLD:ADA9980
Hormius joncoddingtoni|[238]|BIOUG50585-B09|Malaise trap PL12-5A|652[0n]|BOLD:ADA9980
Hormius joncoddingtoni|[239]|BIOUG54497-E11|Malaise trap PL12-3D|650[0n]|BOLD:ADA9980
Hormius joncoddingtoni|[240]|BIOUG53205-C03|Malaise trap PL12-3C|648[0n]|BOLD:ADA9980
Hormius joncoddingtoni|[241]|BIOUG50988-B03|Malaise trap PL12-5B|653[0n]|BOLD:ADA9980
Hormius joncoddingtoni|[242]|BIOUG54809-H03|Malaise trap PL12-3D|650[0n]|BOLD:ADA9980
Hormius joncoddingtoni|[243]|BIOUG50600-D12|Malaise trap PL12-5A|649[0n]|BOLD:ADA9980
Hormius joncoddingtoni|[244]|BIOUG29696-G03|Malaise trap PL12-9A|573[0n]|BOLD:ADA9980
Hormius joncoddingtoni|[245]|BIOUG29880-G09|Malaise trap PL12-3A|573[0n]|BOLD:ADA9980
Hormius joncoddingtoni|[246]|BIOUG54793-G06|Malaise trap PL12-3D|651[0n]|BOLD:ADA9980
Hormius joncoddingtoni|[247]|BIOUG50698-C06|Malaise trap PL12-5A|651[0n]|BOLD:ADA9980
Hormius joncoddingtoni|[248]|BIOUG53419-B01|Malaise trap PL12-3C|647[0n]|BOLD:ADA9980
Hormius joncoddingtoni|[249]|BIOUG46577-G08|Malaise trap PL12-4B|643[0n]|BOLD:ADA9980
Hormius joncoddingtoni|[250]|BIOUG47397-E05|Malaise trap PL12-5A|651[0n]|BOLD:ADA9980
Hormius joncoddingtoni|[251]|BIOUG54711-B07|Malaise trap PL12-3D|650[0n]|BOLD:ADA9980
Hormius joncoddingtoni|[252]|BIOUG53648-D12|Malaise trap PL12-9C|651[0n]|BOLD:ADA9980
Hormius joncoddingtoni|[253]|BIOUG50726-F05|Malaise trap PL12-5A|653[0n]|BOLD:ADA9980
Hormius joncoddingtoni|[254]|BIOUG50652-B06|Malaise trap PL12-5A|651[0n]|BOLD:ADA9980
Hormius joncoddingtoni|[255]|BIOUG53026-D04|Malaise trap PL12-3C|651[0n]|BOLD:ADA9980
Hormius joncoddingtoni|[256]|BIOUG53191-C12|Malaise trap PL12-3C|648[0n]|BOLD:ADA9980
Hormius joncoddingtoni|[257]|BIOUG53417-C04|Malaise trap PL12-3C|648[0n]|BOLD:ADA9980
Hormius joncoddingtoni|[258]|BIOUG50652-D11|Malaise trap PL12-5A|650[0n]|BOLD:ADA9980
Hormius joncoddingtoni|[259]|BIOUG54727-E07|Malaise trap PL12-3D|650[0n]|BOLD:ADA9980
Hormius joncoddingtoni|[260]|BIOUG50485-G05|Malaise trap PL12-5A|652[0n]|BOLD:ADA9980
Hormius joncoddingtoni|[261]|BIOUG46724-E09|Malaise trap PL12-4B|650[0n]|BOLD:ADA9980
Hormius joncoddingtoni|[262]|BIOUG54090-A08|Malaise trap PL12-9C|652[0n]|BOLD:ADA9980
Hormius joncoddingtoni|[263]|BIOUG53324-H11|Malaise trap PL12-3C|648[0n]|BOLD:ADA9980
Hormius joncoddingtoni|[264]|BIOUG49969-G09|Malaise trap PL12-3B|652[0n]|BOLD:ADA9980
Hormius joncoddingtoni|[265]|BIOUG54001-H08|Malaise trap PL12-9C|648[0n]|BOLD:ADA9980
Hormius joncoddingtoni|[266]|BIOUG56055-E03|Malaise trap PL12-3D|650[0n]|BOLD:ADA9980
Hormius joncoddingtoni|[267]|BIOUG56052-G06|Malaise trap PL12-3D|648[0n]|BOLD:ADA9980
Hormius joncoddingtoni|[268]|BIOUG55087-C04|Malaise trap PL12-1D|654[0n]|BOLD:ADA9980
Hormius Malaise2497|[269]|BIOUG08355-H11|BSE Malaise Trap|658[0n]|BOLD:ACK2497
Hormius Malaise8560|[270]|BIOUG30025-F07|Malaise trap PL12-3A|597[0n]|BOLD:AAP8560
Hormius Malaise8560|[271]|BIOUG30060-B01|Malaise trap PL12-3A|600[0n]|BOLD:AAP8560
Hormius Malaise8560|[272]|BIOUG29696-E07|Malaise trap PL12-9A|576[0n]|BOLD:AAP8560
Hormius Malaise8560|[273]|BIOUG46725-F12|Malaise trap PL12-4B|651[0n]|BOLD:AAP8560
Hormius Malaise8560|[274]|BIOUG50684-H02|Malaise trap PL12-5A|652[0n]|BOLD:AAP8560

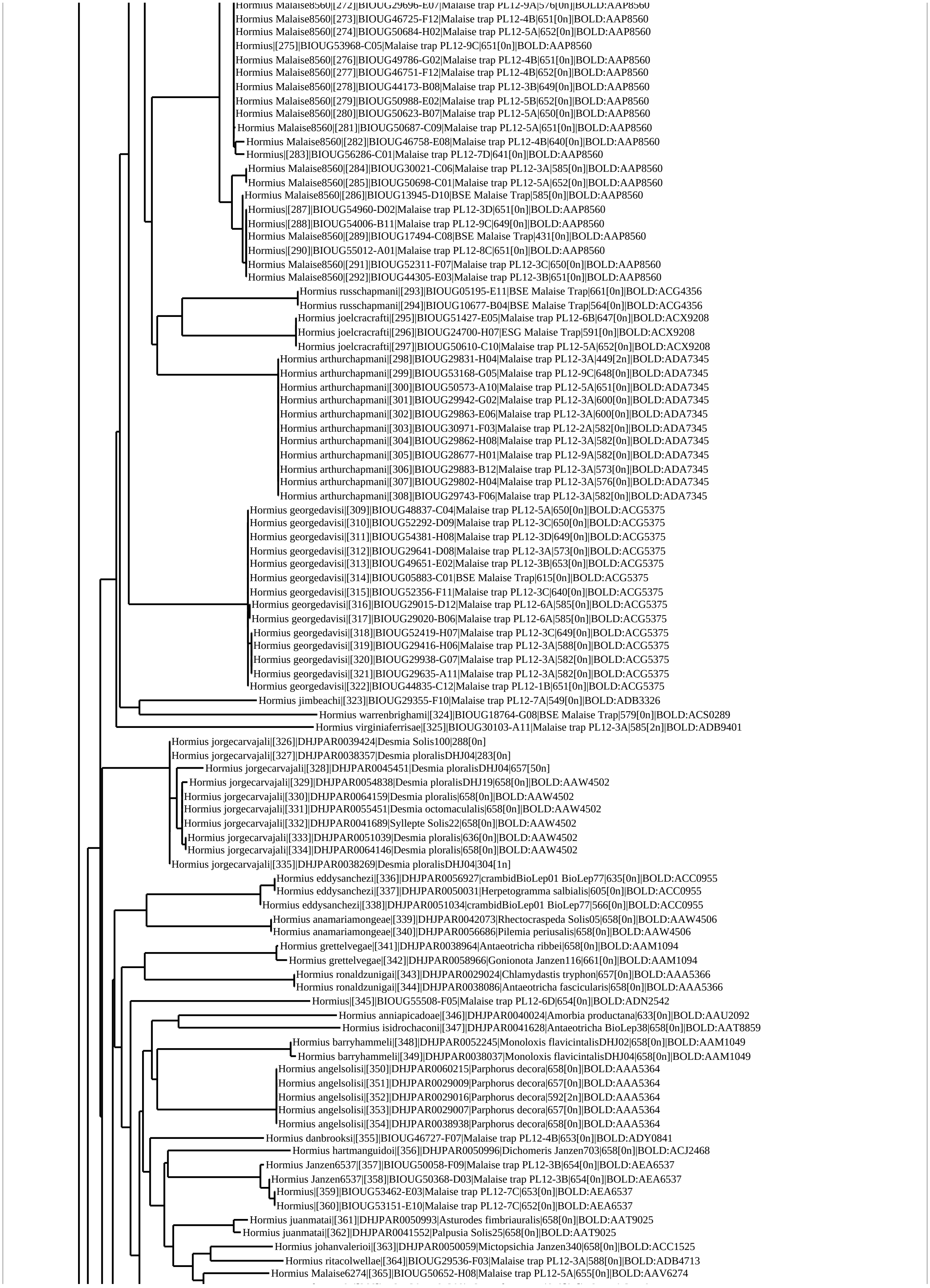
Hormius Malaise8560|[272]|BIOUG29696-E07|Malaise trap PL12-9A|576[0n]|BOLD:AAP8560
Hormius Malaise8560|[273]|BIOUG46725-F12|Malaise trap PL12-4B|651[0n]|BOLD:AAP8560
Hormius Malaise8560|[274]|BIOUG50684-H02|Malaise trap PL12-5A|652[0n]|BOLD:AAP8560
Hormius|[275]|BIOUG53968-C05|Malaise trap PL12-9C|651[0n]|BOLD:AAP8560
Hormius Malaise8560|[276]|BIOUG49786-G02|Malaise trap PL12-4B|651[0n]|BOLD:AAP8560
Hormius Malaise8560|[277]|BIOUG46751-F12|Malaise trap PL12-4B|652[0n]|BOLD:AAP8560
Hormius Malaise8560|[278]|BIOUG44173-B08|Malaise trap PL12-3B|649[0n]|BOLD:AAP8560
Hormius Malaise8560|[279]|BIOUG50988-E02|Malaise trap PL12-5B|652[0n]|BOLD:AAP8560
Hormius Malaise8560|[280]|BIOUG50623-B07|Malaise trap PL12-5A|650[0n]|BOLD:AAP8560
Hormius Malaise8560|[281]|BIOUG50687-C09|Malaise trap PL12-5A|651[0n]|BOLD:AAP8560
Hormius Malaise8560|[282]|BIOUG46758-E08|Malaise trap PL12-4B|640[0n]|BOLD:AAP8560
Hormius|[283]|BIOUG56286-C01|Malaise trap PL12-7D|641[0n]|BOLD:AAP8560
Hormius Malaise8560|[284]|BIOUG30021-C06|Malaise trap PL12-3A|585[0n]|BOLD:AAP8560
Hormius Malaise8560|[285]|BIOUG50698-C01|Malaise trap PL12-5A|652[0n]|BOLD:AAP8560
Hormius Malaise8560|[286]|BIOUG13945-D10|BSE Malaise Trap|585[0n]|BOLD:AAP8560
Hormius|[287]|BIOUG54960-D02|Malaise trap PL12-3D|651[0n]|BOLD:AAP8560
Hormius|[288]|BIOUG54006-B11|Malaise trap PL12-9C|649[0n]|BOLD:AAP8560
Hormius Malaise8560|[289]|BIOUG17494-C08|BSE Malaise Trap|431[0n]|BOLD:AAP8560
Hormius|[290]|BIOUG55012-A01|Malaise trap PL12-8C|651[0n]|BOLD:AAP8560
Hormius Malaise8560|[291]|BIOUG52311-F07|Malaise trap PL12-3C|650[0n]|BOLD:AAP8560
Hormius Malaise8560|[292]|BIOUG44305-E03|Malaise trap PL12-3B|651[0n]|BOLD:AAP8560
Hormius russchapmani|[293]|BIOUG05195-E11|BSE Malaise Trap|661[0n]|BOLD:ACG4356
Hormius russchapmani|[294]|BIOUG10677-B04|BSE Malaise Trap|564[0n]|BOLD:ACG4356
Hormius joelcracrafti|[295]|BIOUG51427-E05|Malaise trap PL12-6B|647[0n]|BOLD:ACX9208
Hormius joelcracrafti|[296]|BIOUG24700-H07|ESG Malaise Trap|591[0n]|BOLD:ACX9208
Hormius joelcracrafti|[297]|BIOUG50610-C10|Malaise trap PL12-5A|652[0n]|BOLD:ACX9208
Hormius arthurchapmani|[298]|BIOUG29831-H04|Malaise trap PL12-3A|449[2n]|BOLD:ADA7345
Hormius arthurchapmani|[299]|BIOUG53168-G05|Malaise trap PL12-9C|648[0n]|BOLD:ADA7345
Hormius arthurchapmani|[300]|BIOUG50573-A10|Malaise trap PL12-5A|651[0n]|BOLD:ADA7345
Hormius arthurchapmani|[301]|BIOUG29942-G02|Malaise trap PL12-3A|600[0n]|BOLD:ADA7345
Hormius arthurchapmani|[302]|BIOUG29863-E06|Malaise trap PL12-3A|600[0n]|BOLD:ADA7345
Hormius arthurchapmani|[303]|BIOUG30971-F03|Malaise trap PL12-2A|582[0n]|BOLD:ADA7345
Hormius arthurchapmani|[304]|BIOUG29862-H08|Malaise trap PL12-3A|582[0n]|BOLD:ADA7345
Hormius arthurchapmani|[305]|BIOUG28677-H01|Malaise trap PL12-9A|582[0n]|BOLD:ADA7345
Hormius arthurchapmani|[306]|BIOUG29883-B12|Malaise trap PL12-3A|573[0n]|BOLD:ADA7345
Hormius arthurchapmani|[307]|BIOUG29802-H04|Malaise trap PL12-3A|576[0n]|BOLD:ADA7345
Hormius arthurchapmani|[308]|BIOUG29743-F06|Malaise trap PL12-3A|582[0n]|BOLD:ADA7345
Hormius georgedavisi|[309]|BIOUG48837-C04|Malaise trap PL12-5A|650[0n]|BOLD:ACG5375
Hormius georgedavisi|[310]|BIOUG52292-D09|Malaise trap PL12-3C|650[0n]|BOLD:ACG5375
Hormius georgedavisi|[311]|BIOUG54381-H08|Malaise trap PL12-3D|649[0n]|BOLD:ACG5375
Hormius georgedavisi|[312]|BIOUG29641-D08|Malaise trap PL12-3A|573[0n]|BOLD:ACG5375
Hormius georgedavisi|[313]|BIOUG49651-E02|Malaise trap PL12-3B|653[0n]|BOLD:ACG5375
Hormius georgedavisi|[314]|BIOUG05883-C01|BSE Malaise Trap|615[0n]|BOLD:ACG5375
Hormius georgedavisi|[315]|BIOUG52356-F11|Malaise trap PL12-3C|640[0n]|BOLD:ACG5375
Hormius georgedavisi|[316]|BIOUG29015-D12|Malaise trap PL12-6A|585[0n]|BOLD:ACG5375
Hormius georgedavisi|[317]|BIOUG29020-B06|Malaise trap PL12-6A|585[0n]|BOLD:ACG5375
Hormius georgedavisi|[318]|BIOUG52419-H07|Malaise trap PL12-3C|649[0n]|BOLD:ACG5375
Hormius georgedavisi|[319]|BIOUG29416-H06|Malaise trap PL12-3A|588[0n]|BOLD:ACG5375
Hormius georgedavisi|[320]|BIOUG29938-G07|Malaise trap PL12-3A|582[0n]|BOLD:ACG5375
Hormius georgedavisi|[321]|BIOUG29635-A11|Malaise trap PL12-3A|582[0n]|BOLD:ACG5375
Hormius georgedavisi|[322]|BIOUG44835-C12|Malaise trap PL12-1B|651[0n]|BOLD:ACG5375
Hormius jimbeachi|[323]|BIOUG29355-F10|Malaise trap PL12-7A|549[0n]|BOLD:ADB3326
Hormius warrenbrighami|[324]|BIOUG18764-G08|BSE Malaise Trap|579[0n]|BOLD:ACS0289
Hormius virginiaferrisae|[325]|BIOUG30103-A11|Malaise trap PL12-3A|585[2n]|BOLD:ADB9401
Hormius jorgecarvajali|[326]|DHJPAR0039424|Desmia Solis100|288[0n]
Hormius jorgecarvajali|[327]|DHJPAR0038357|Desmia ploralisDHJ04|283[0n]
Hormius jorgecarvajali|[328]|DHJPAR0045451|Desmia ploralisDHJ04|657[50n]
Hormius jorgecarvajali|[329]|DHJPAR0054838|Desmia ploralisDHJ19|658[0n]|BOLD:AAW4502
Hormius jorgecarvajali|[330]|DHJPAR0064159|Desmia ploralis|658[0n]|BOLD:AAW4502
Hormius jorgecarvajali|[331]|DHJPAR0055451|Desmia octomaculalis|658[0n]|BOLD:AAW4502
Hormius jorgecarvajali|[332]|DHJPAR0041689|Syllepte Solis22|658[0n]|BOLD:AAW4502
Hormius jorgecarvajali|[333]|DHJPAR0051039|Desmia ploralis|636[0n]|BOLD:AAW4502
Hormius jorgecarvajali|[334]|DHJPAR0064146|Desmia ploralis|658[0n]|BOLD:AAW4502
Hormius jorgecarvajali|[335]|DHJPAR0038269|Desmia ploralisDHJ04|304[1n]
Hormius eddysanchezi|[336]|DHJPAR0056927|crambidBioLep01 BioLep77|635[0n]|BOLD:ACC0955
Hormius eddysanchezi|[337]|DHJPAR0050031|Herpetogramma salbialis|605[0n]|BOLD:ACC0955
Hormius eddysanchezi|[338]|DHJPAR0051034|crambidBioLep01 BioLep77|566[0n]|BOLD:ACC0955
Hormius anamariamongeae|[339]|DHJPAR0042073|Rhectocraspeda Solis05|658[0n]|BOLD:AAW4506
Hormius anamariamongeae|[340]|DHJPAR0056686|Pilemia periusalis|658[0n]|BOLD:AAW4506
Hormius grettelvegae|[341]|DHJPAR0038964|Antaeotricha ribbei|658[0n]|BOLD:AAM1094
Hormius grettelvegae|[342]|DHJPAR0058966|Gonionota Janzen116|661[0n]|BOLD:AAM1094
Hormius ronaldzunigai|[343]|DHJPAR0029024|Chlamydastis tryphon|657[0n]|BOLD:AAA5366
Hormius ronaldzunigai|[344]|DHJPAR0038086|Antaeotricha fascicularis|658[0n]|BOLD:AAA5366
Hormius|[345]|BIOUG55508-F05|Malaise trap PL12-6D|654[0n]|BOLD:ADN2542
Hormius anniapicadoae|[346]|DHJPAR0040024|Amorbia productana|633[0n]|BOLD:AAU2092
Hormius isidrochaconi|[347]|DHJPAR0041628|Antaeotricha BioLep38|658[0n]|BOLD:AAT8859
Hormius barryhammeli|[348]|DHJPAR0052245|Monoloxis flavicintalisDHJ02|658[0n]|BOLD:AAM1049
Hormius barryhammeli|[349]|DHJPAR0038037|Monoloxis flavicintalisDHJ04|658[0n]|BOLD:AAM1049
Hormius angelsolisi|[350]|DHJPAR0060215|Parphorus decora|658[0n]|BOLD:AAA5364
Hormius angelsolisi|[351]|DHJPAR0029009|Parphorus decora|657[0n]|BOLD:AAA5364
Hormius angelsolisi|[352]|DHJPAR0029016|Parphorus decora|592[2n]|BOLD:AAA5364
Hormius angelsolisi|[353]|DHJPAR0029007|Parphorus decora|657[0n]|BOLD:AAA5364
Hormius angelsolisi|[354]|DHJPAR0038938|Parphorus decora|658[0n]|BOLD:AAA5364
Hormius danbrooksi|[355]|BIOUG46727-F07|Malaise trap PL12-4B|653[0n]|BOLD:ADY0841
Hormius hartmanguidoi|[356]|DHJPAR0050996|Dichomeris Janzen703|658[0n]|BOLD:ACJ2468
Hormius Janzen6537|[357]|BIOUG50058-F09|Malaise trap PL12-3B|654[0n]|BOLD:AEA6537
Hormius Janzen6537|[358]|BIOUG50368-D03|Malaise trap PL12-3B|654[0n]|BOLD:AEA6537
Hormius|[359]|BIOUG53462-E03|Malaise trap PL12-7C|653[0n]|BOLD:AEA6537
Hormius|[360]|BIOUG53151-E10|Malaise trap PL12-7C|652[0n]|BOLD:AEA6537
Hormius juanmatai|[361]|DHJPAR0050993|Asturodes fimbriauralis|658[0n]|BOLD:AAT9025
Hormius juanmatai|[362]|DHJPAR0041552|Palpusia Solis25|658[0n]|BOLD:AAT9025
Hormius johanvalerioi|[363]|DHJPAR0050059|Mictopsichia Janzen340|658[0n]|BOLD:ACC1525
Hormius ritacolwellae|[364]|BIOUG29536-F03|Malaise trap PL12-3A|588[0n]|BOLD:ADB4713
Hormius Malaise6274|[365]|BIOUG50652-H08|Malaise trap PL12-5A|655[0n]|BOLD:AAV6274

[illegible]
Hormius ritacolwellae|[364]|BIOUG29536-F03|Malaise trap PL12-3A|588[0n]|BOLD:ADB4713
Hormius Malaise6274|[365]|BIOUG50652-H08|Malaise trap PL12-5A|655[0n]|BOLD:AAV6274
Hormius Malaise1284|[366]|BIOUG07116-G08|BSE Malaise Trap|658[0n]|BOLD:ACJ1284
Hormius cesarsuarezi|[367]|DHJPAR0029000|Herpetogramma salbialis|657[0n]|BOLD:ACK7796
Hormius cesarsuarezi|[368]|DHJPAR0029013|Herpetogramma salbialis|657[0n]|BOLD:ACK7796
Hormius cesarsuarezi|[369]|DHJPAR0041724|Herpetogramma salbialis|658[0n]|BOLD:ACK7796
Hormius cesarsuarezi|[370]|DHJPAR0031136|Herpetogramma salbialis|611[0n]|BOLD:ACK7796
Hormius cesarsuarezi|[371]|DHJPAR0039421|Herpetogramma salbialis|633[0n]|BOLD:ACK7796
Hormius cesarsuarezi|[372]|DHJPAR0029006|Herpetogramma salbialis|649[2n]|BOLD:ACK7796
Hormius cesarsuarezi|[373]|DHJPAR0041709|Herpetogramma salbialis|658[0n]|BOLD:ACK7796
Hormius cesarsuarezi|[374]|DHJPAR0038262|Herpetogramma salbialis|648[0n]|BOLD:ACK7796
Hormius cesarsuarezi|[375]|BIOUG28717-D09|Malaise trap PL12-6A|591[0n]|BOLD:ACK7796
Hormius cesarsuarezi|[376]|DHJPAR0049093|Herpetogramma salbialis|658[0n]|BOLD:ACK7796
Hormius cesarsuarezi|[377]|DHJPAR0035321|Herpetogramma salbialis|658[0n]|BOLD:ACK7796
Hormius cesarsuarezi|[378]|DHJPAR0053699|Herpetogramma salbialis|658[0n]|BOLD:ACK7796
Hormius cesarsuarezi|[379]|DHJPAR0035390|Herpetogramma salbialis|658[1n]|BOLD:ACK7796
Hormius cesarsuarezi|[380]|DHJPAR0038115|Herpetogramma salbialis|619[0n]|BOLD:ACK7796
Hormius cesarsuarezi|[381]|DHJPAR0039420|Herpetogramma salbialis|658[0n]|BOLD:ACK7796
Hormius cesarsuarezi|[382]|DHJPAR0041698|Herpetogramma salbialis|658[0n]|BOLD:ACK7796
Hormius cesarsuarezi|[383]|DHJPAR0047041|Herpetogramma salbialis|661[1n]|BOLD:ACK7796
Hormius cesarsuarezi|[384]|DHJPAR0047116|Herpetogramma salbialis|620[0n]|BOLD:ACK7796
Hormius pamelacastilloae|[385]|DHJPAR0038272|Omiodes humeralisDHJ07|647[0n]|BOLD:AAM1076
Hormius jaygallegosi|[386]|DHJPAR0029001|Cliniodes opalalis|657[0n]|BOLD:ACK6504
Hormius jaygallegosi|[387]|DHJPAR0029015|Cliniodes opalalis|657[0n]|BOLD:ACK6504
Hormius jaygallegosi|[388]|DHJPAR0062198|Cliniodes opalalis|658[0n]|BOLD:ACK6504
Hormius jaygallegosi|[389]|DHJPAR0029023|Cliniodes opalalis|657[0n]|BOLD:ACK6504
Hormius jaygallegosi|[390]|DHJPAR0029014|Cliniodes opalalis|657[0n]|BOLD:ACK6504
Hormius jaygallegosi|[391]|DHJPAR0029025|Cliniodes opalalis|631[0n]|BOLD:ACK6504
Hormius carmenretanae|[392]|DHJPAR0042983|phyBioLep01 BioLep757|658[0n]|BOLD:ABA7266
Hormius nelsonzamorai|[393]|DHJPAR0035445|Stenoma Janzen07|572[0n]|BOLD:AAM1047
Hormius irenecanasae|[394]|DHJPAR0049383|gelJanzen01 Janzen235|658[0n]|BOLD:ACB2774
Hormius irenecanasae|[395]|DHJPAR0052293|Asturodes fimbriauralisDHJ02|658[0n]|BOLD:ACB2774
Hormius|[396]|BIOUG55686-E06|Malaise trap PL12-9D|652[0n]|BOLD:AEE2066
Hormius|[397]|BIOUG57109-H07|Malaise trap PL12-5D|654[0n]|BOLD:AEE2066
Hormius|[398]|BIOUG56291-H03|Malaise trap PL12-2D|653[0n]|BOLD:AEE2066
Hormius Janzen06|[399]|DHJPAR0030610|Xenophanes tryxus|407[0n]
Hormius Janzen07|[400]|DHJPAR0030612|Lygropia tripunctataDHJ01|407[0n]
Hormius|[401]|BIOUG54787-E04|Malaise trap PL12-3D|640[0n]|BOLD:AEE0648
Hormius jimlewisi|[402]|DHJPAR0047155|Orphanostigma haemorrhoidalis|627[0n]|BOLD:AAK5352
Hormius jimlewisi|[403]|DHJPAR0042956|Orphanostigma haemorrhoidalis|658[0n]|BOLD:AAK5352
Hormius jimlewisi|[404]|DHJPAR0038122|Orphanostigma haemorrhoidalis|658[0n]|BOLD:AAK5352
Hormius jimlewisi|[405]|DHJPAR0051229|Orphanostigma haemorrhoidalis|658[0n]|BOLD:AAK5352
Hormius jimlewisi|[406]|DHJPAR0030615|Salbia cassidalis|407[0n]|BOLD:AAK5352
Hormius jimlewisi|[407]|DHJPAR0030617|Salbia cassidalis|658[1n]|BOLD:AAK5352
Hormius willsflowersi|[408]|BIOUG50600-G05|Malaise trap PL12-5A|655[0n]|BOLD:ADB9850
Hormius willsflowersi|[409]|BIOUG29991-F05|Malaise trap PL12-3A|555[0n]|BOLD:ADB9850
Hormius willsflowersi|[410]|BIOUG51421-C08|Malaise trap PL12-6B|654[0n]|BOLD:ADB9850
Hormius willsflowersi|[411]|BIOUG49022-C08|Malaise trap PL12-5B|656[0n]|BOLD:ADB9850
Hormius gustavoinduni|[412]|DHJPAR0043056|elachJanzen01 Janzen764|658[0n]|BOLD:ABA7247
Hormius gustavoinduni|[413]|DHJPAR0038344|elachJanzen01 Janzen764|658[0n]|BOLD:ABA7247
Hormius hesiquiobenitezi|[414]|BIOUG07616-G03|BSE Malaise Trap|543[0n]|BOLD:ACJ1489
Hormius hesiquiobenitezi|[415]|BIOUG51481-E11|Malaise trap PL12-7B|651[0n]|BOLD:ACJ1489
Hormius hesiquiobenitezi|[416]|BIOUG54767-E10|Malaise trap PL12-8C|655[0n]|BOLD:ACJ1489
Hormius hesiquiobenitezi|[417]|BIOUG49968-E10|Malaise trap PL12-3B|654[0n]|BOLD:ACJ1489
Hormius hesiquiobenitezi|[418]|BIOUG44185-A05|Malaise trap PL12-3B|652[0n]|BOLD:ACJ1489
Hormius hesiquiobenitezi|[419]|BIOUG44312-G10|Malaise trap PL12-3B|655[0n]|BOLD:ACJ1489
Hormius hesiquiobenitezi|[420]|BIOUG50092-E06|Malaise trap PL12-3B|654[0n]|BOLD:ACJ1489
Hormius hesiquiobenitezi|[421]|BIOUG55916-G08|Malaise trap PL12-3D|653[0n]|BOLD:ACJ1489
Hormius hesiquiobenitezi|[422]|BIOUG44312-F10|Malaise trap PL12-3B|654[0n]|BOLD:ACJ1489
Hormius hesiquiobenitezi|[423]|BIOUG44207-H08|Malaise trap PL12-3B|653[0n]|BOLD:ACJ1489
Hormius hesiquiobenitezi|[424]|BIOUG53830-H01|Malaise trap PL12-9C|655[0n]|BOLD:ACJ1489
Hormius hesiquiobenitezi|[425]|BIOUG44331-H02|Malaise trap PL12-3B|654[0n]|BOLD:ACJ1489
Hormius hesiquiobenitezi|[426]|BIOUG52361-F11|Malaise trap PL12-3C|651[0n]|BOLD:ACJ1489
Hormius hesiquiobenitezi|[427]|BIOUG51524-B11|Malaise trap PL12-3C|653[0n]|BOLD:ACJ1489
Hormius hesiquiobenitezi|[428]|BIOUG56243-F01|Malaise trap PL12-6D|654[0n]|BOLD:ACJ1489
Hormius hesiquiobenitezi|[429]|BIOUG54833-D08|Malaise trap PL12-5C|654[0n]|BOLD:ACJ1489
Hormius hesiquiobenitezi|[430]|BIOUG44738-E04|Malaise trap PL12-3B|653[0n]|BOLD:ACJ1489
Hormius hesiquiobenitezi|[431]|BIOUG53830-H04|Malaise trap PL12-9C|653[0n]|BOLD:ACJ1489
Hormius hesiquiobenitezi|[432]|BIOUG07458-G07|BSE Malaise Trap|658[0n]|BOLD:ACJ1489
Hormius hesiquiobenitezi|[433]|BIOUG52185-E06|Malaise trap PL12-3C|654[0n]|BOLD:ACJ1489
Hormius hesiquiobenitezi|[434]|BIOUG54749-G09|Malaise trap PL12-5C|640[0n]|BOLD:ACJ1489
Hormius hesiquiobenitezi|[435]|BIOUG07617-B11|BSE Malaise Trap|592[0n]|BOLD:ACJ1489
Hormius hesiquiobenitezi|[436]|BIOUG29535-E08|Malaise trap PL12-3A|576[0n]|BOLD:ACJ1489
Hormius hesiquiobenitezi|[437]|BIOUG18290-C08|BSE Malaise Trap|594[0n]|BOLD:ACJ1489
Hormius hesiquiobenitezi|[438]|BIOUG49691-H08|Malaise trap PL12-3B|645[0n]|BOLD:ACJ1489
Hormius carloswalkeri|[439]|DHJPAR0053764|Vettius aurelius|491[0n]|BOLD:ACK8132
Hormius carloswalkeri|[440]|DHJPAR0029027|Vettius aurelius|657[1n]|BOLD:ACK8132
Hormius carloswalkeri|[441]|DHJPAR0058823|Conga chydaea|656[0n]|BOLD:ACK8132
Hormius carloswalkeri|[442]|DHJPAR0047119|Vettius aurelius|658[0n]|BOLD:ACK8132
Hormius carloswalkeri|[443]|DHJPAR0029010|Joanna joanna|657[0n]|BOLD:ACK8132
Hormius carloswalkeri|[444]|DHJPAR0049084|Vettius aurelius|658[0n]|BOLD:ACK8132
Hormius carloswalkeri|[445]|DHJPAR0058136|Morys lyde|658[0n]|BOLD:ACK8132
Hormius carloswalkeri|[446]|DHJPAR0047127|Justinia norda|658[0n]|BOLD:ACK8132
Hormius carloswalkeri|[447]|DHJPAR0049061|Tigasis simplex|658[0n]|BOLD:ACK8132
Hormius carloswalkeri|[448]|DHJPAR0047098|Vehilius vetula|658[0n]|BOLD:ACK8132
Hormius carloswalkeri|[449]|DHJPAR0057466|Cynea irma|630[0n]|BOLD:ACK8132
Hormius Janzen21|[450]|DHJPAR0030620|Lerema liris|407[0n]
Hormius manuelzumbadoi|[451]|DHJPAR0035421|Stenoma Janzen20|270[0n]
Hormius manuelzumbadoi|[452]|DHJPAR0049109|Stenoma Janzen20|658[0n]|BOLD:AAD0121
Hormius manuelzumbadoi|[453]|DHJPAR0049476|Stenoma Janzen20|658[0n]|BOLD:AAD0121
Hormius manuelzumbadoi|[454]|DHJPAR0049128|Stenoma Janzen20|658[0n]|BOLD:AAD0121
Hormius manuelzumbadoi|[455]|DHJPAR0035338|Antaeotricha marmorea|658[0n]|BOLD:AAD0121
