# Supplementary material for: Minimalist revision and description of 403 new species in 11 subfamilies of Costa Rican braconid parasitoid wasps, including host records for 219 species
Source: Zookeys. 2021 Feb 2;1013:1–665. doi: 10.3897/zookeys.1013.55600 (PMC8390796; doi:10.3897/zookeys.1013.55600)
Supplement: Supplementary material 6 — Ichneutinae and Proteropinae [file zookeys-1013-001-s006.pdf]

6. Ichneutinae and Proteropinae BOLD TaxonID Tree

```
Title           : Tree Result - Search:  Sample IDs (51 records returned) (51 records
selected)
Date            : 17-Nov-2020
Data Type       : Nucleotide
Distance Model  : Kimura 2 Parameter
Marker          : COI-5P
Colourization   : [blue]=Stop Codons [red]=Contamination or misidentification

Label           : Sample ID
Label           : Taxon
Label           : Extra Info
Label           : Sequence Length
Label           : Barcode Cluster (BIN)

Filter          : exclude records with stop codons

Sequence Count  : 39
Species count   : 11
Genus count     : 4
Family count    : 1
Unidentified    : 0

BIN Count       : 11
```

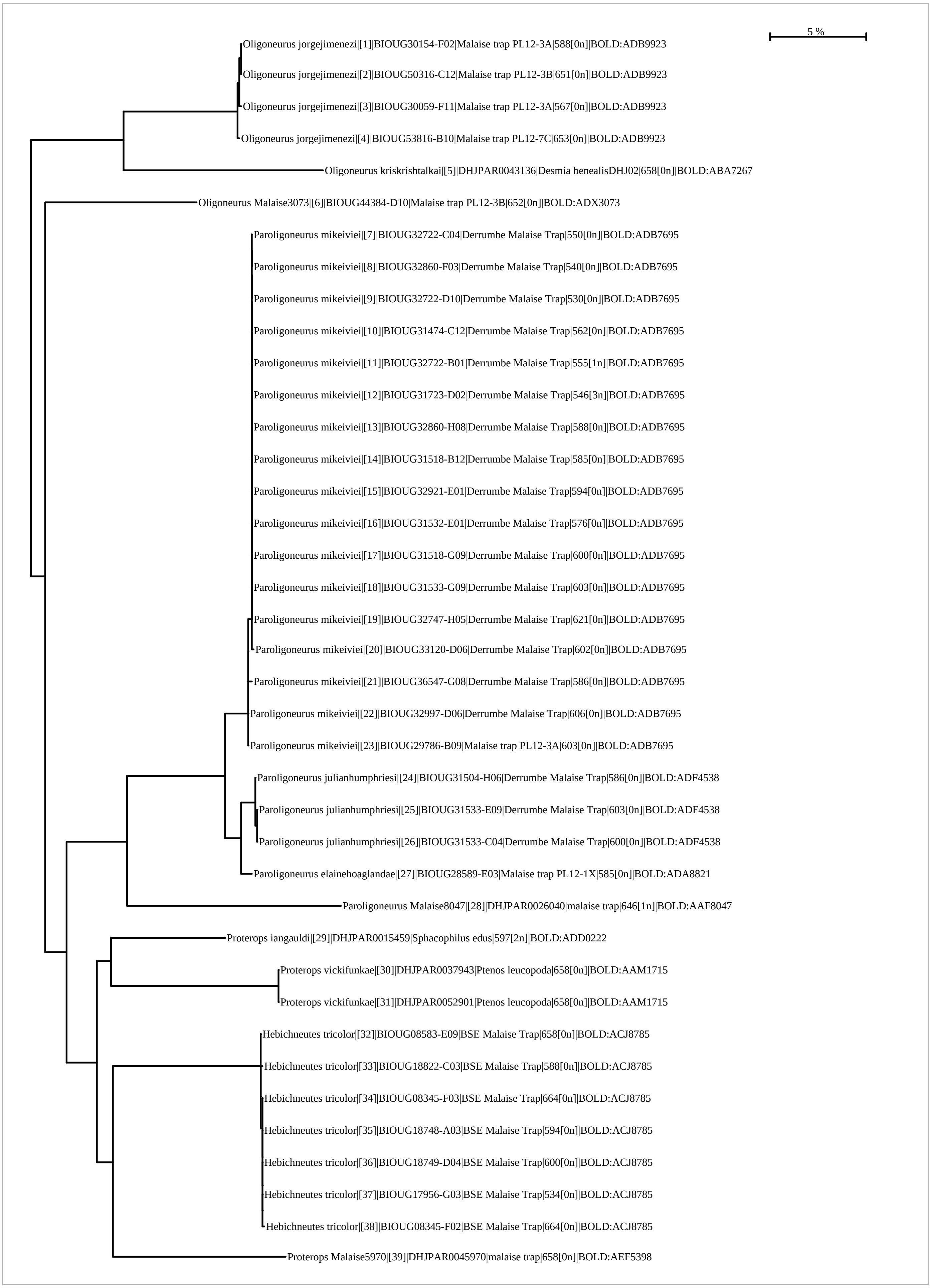
5 %
Oligoneurus jorgejimenezi|[1]|BIOUG30154-F02|Malaise trap PL12-3A|588[0n]|BOLD:ADB9923
Oligoneurus jorgejimenezi|[2]|BIOUG50316-C12|Malaise trap PL12-3B|651[0n]|BOLD:ADB9923
Oligoneurus jorgejimenezi|[3]|BIOUG30059-F11|Malaise trap PL12-3A|567[0n]|BOLD:ADB9923
Oligoneurus jorgejimenezi|[4]|BIOUG53816-B10|Malaise trap PL12-7C|653[0n]|BOLD:ADB9923
Oligoneurus kriskrishtalkai|[5]|DHJPAR0043136|Desmia benealisDHJ02|658[0n]|BOLD:ABA7267
Oligoneurus Malaise3073|[6]|BIOUG44384-D10|Malaise trap PL12-3B|652[0n]|BOLD:ADX3073
Paroligoneurus mikeiviei|[7]|BIOUG32722-C04|Derrumbe Malaise Trap|550[0n]|BOLD:ADB7695
Paroligoneurus mikeiviei|[8]|BIOUG32860-F03|Derrumbe Malaise Trap|540[0n]|BOLD:ADB7695
Paroligoneurus mikeiviei|[9]|BIOUG32722-D10|Derrumbe Malaise Trap|530[0n]|BOLD:ADB7695
Paroligoneurus mikeiviei|[10]|BIOUG31474-C12|Derrumbe Malaise Trap|562[0n]|BOLD:ADB7695
Paroligoneurus mikeiviei|[11]|BIOUG32722-B01|Derrumbe Malaise Trap|555[1n]|BOLD:ADB7695
Paroligoneurus mikeiviei|[12]|BIOUG31723-D02|Derrumbe Malaise Trap|546[3n]|BOLD:ADB7695
Paroligoneurus mikeiviei|[13]|BIOUG32860-H08|Derrumbe Malaise Trap|588[0n]|BOLD:ADB7695
Paroligoneurus mikeiviei|[14]|BIOUG31518-B12|Derrumbe Malaise Trap|585[0n]|BOLD:ADB7695
Paroligoneurus mikeiviei|[15]|BIOUG32921-E01|Derrumbe Malaise Trap|594[0n]|BOLD:ADB7695
Paroligoneurus mikeiviei|[16]|BIOUG31532-E01|Derrumbe Malaise Trap|576[0n]|BOLD:ADB7695
Paroligoneurus mikeiviei|[17]|BIOUG31518-G09|Derrumbe Malaise Trap|600[0n]|BOLD:ADB7695
Paroligoneurus mikeiviei|[18]|BIOUG31533-G09|Derrumbe Malaise Trap|603[0n]|BOLD:ADB7695
Paroligoneurus mikeiviei|[19]|BIOUG32747-H05|Derrumbe Malaise Trap|621[0n]|BOLD:ADB7695
Paroligoneurus mikeiviei|[20]|BIOUG33120-D06|Derrumbe Malaise Trap|602[0n]|BOLD:ADB7695
Paroligoneurus mikeiviei|[21]|BIOUG36547-G08|Derrumbe Malaise Trap|586[0n]|BOLD:ADB7695
Paroligoneurus mikeiviei|[22]|BIOUG32997-D06|Derrumbe Malaise Trap|606[0n]|BOLD:ADB7695
Paroligoneurus mikeiviei|[23]|BIOUG29786-B09|Malaise trap PL12-3A|603[0n]|BOLD:ADB7695
Paroligoneurus julianhumphriesi|[24]|BIOUG31504-H06|Derrumbe Malaise Trap|586[0n]|BOLD:ADF4538
Paroligoneurus julianhumphriesi|[25]|BIOUG31533-E09|Derrumbe Malaise Trap|603[0n]|BOLD:ADF4538
Paroligoneurus julianhumphriesi|[26]|BIOUG31533-C04|Derrumbe Malaise Trap|600[0n]|BOLD:ADF4538
Paroligoneurus elainehoaglandae|[27]|BIOUG28589-E03|Malaise trap PL12-1X|585[0n]|BOLD:ADA8821
Paroligoneurus Malaise8047|[28]|DHJPAR0026040|malaise trap|646[1n]|BOLD:AAF8047
Proterops iangauldi|[29]|DHJPAR0015459|Sphacophilus edus|597[2n]|BOLD:ADD0222
Proterops vickifunkae|[30]|DHJPAR0037943|Ptenos leucopoda|658[0n]|BOLD:AAM1715
Proterops vickifunkae|[31]|DHJPAR0052901|Ptenos leucopoda|658[0n]|BOLD:AAM1715
Hebichneutes tricolor|[32]|BIOUG08583-E09|BSE Malaise Trap|658[0n]|BOLD:ACJ8785
Hebichneutes tricolor|[33]|BIOUG18822-C03|BSE Malaise Trap|588[0n]|BOLD:ACJ8785
Hebichneutes tricolor|[34]|BIOUG08345-F03|BSE Malaise Trap|664[0n]|BOLD:ACJ8785
Hebichneutes tricolor|[35]|BIOUG18748-A03|BSE Malaise Trap|594[0n]|BOLD:ACJ8785
Hebichneutes tricolor|[36]|BIOUG18749-D04|BSE Malaise Trap|600[0n]|BOLD:ACJ8785
Hebichneutes tricolor|[37]|BIOUG17956-G03|BSE Malaise Trap|534[0n]|BOLD:ACJ8785
Hebichneutes tricolor|[38]|BIOUG08345-F02|BSE Malaise Trap|664[0n]|BOLD:ACJ8785
Proterops Malaise5970|[39]|DHJPAR0045970|malaise trap|658[0n]|BOLD:AEF5398
