# Supplementary material for: Minimalist revision and description of 403 new species in 11 subfamilies of Costa Rican braconid parasitoid wasps, including host records for 219 species
Source: Zookeys. 2021 Feb 2;1013:1–665. doi: 10.3897/zookeys.1013.55600 (PMC8390796; doi:10.3897/zookeys.1013.55600)
Supplement: Supplementary material 7 — Macrocentrinae [file zookeys-1013-001-s007.pdf]

7. Macrocentrinae BOLD TaxonID Tree

Title : Tree Result - Search: Sample IDs (559 records returned) (559 records selected)

Date : 17-Nov-2020

Data Type : Nucleotide

Distance Model : Kimura 2 Parameter

Marker : COI-5P

Colourization : [blue]=Stop Codons [red]=Contamination or misidentification

Label : Sample ID

Label : Taxon

Label : Extra Info

Label : Sequence Length

Label : Barcode Cluster (BIN)

Filter : exclude records with stop codons

Sequence Count : 518

Species count : 53

Genus count : 5

Family count : 1

Unidentified : 0

BIN Count : 36

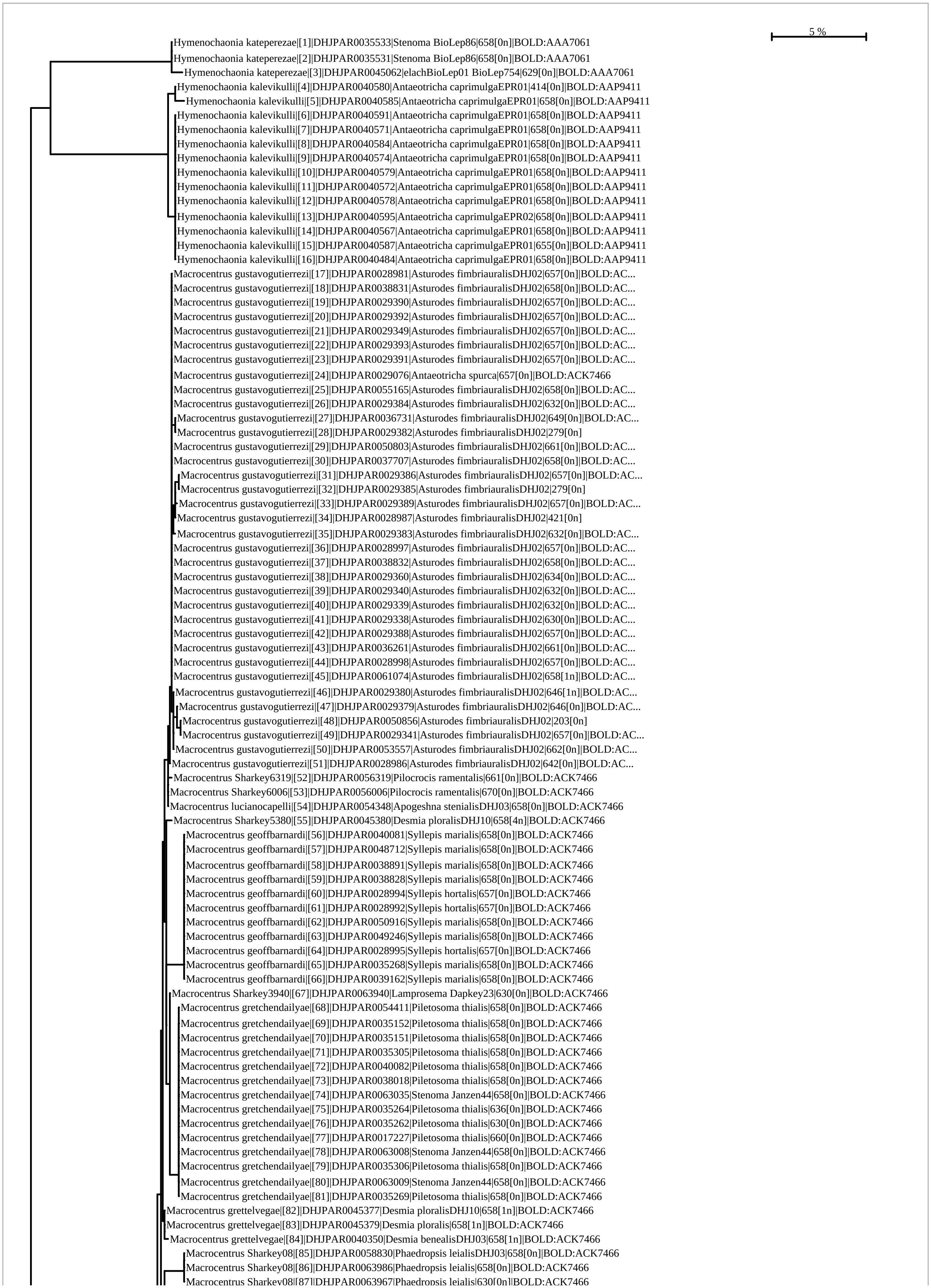
5 %
Hymenochaonia kateperezae|[1]|DHJPAR0035533|Stenoma BioLep86|658[0n]|BOLD:AAA7061
Hymenochaonia kateperezae|[2]|DHJPAR0035531|Stenoma BioLep86|658[0n]|BOLD:AAA7061
Hymenochaonia kateperezae|[3]|DHJPAR0045062|elachBioLep01 BioLep754|629[0n]|BOLD:AAA7061
Hymenochaonia kalevikulli|[4]|DHJPAR0040580|Antaeotricha caprimulgaEPR01|414[0n]|BOLD:AAP9411
Hymenochaonia kalevikulli|[5]|DHJPAR0040585|Antaeotricha caprimulgaEPR01|658[0n]|BOLD:AAP9411
Hymenochaonia kalevikulli|[6]|DHJPAR0040591|Antaeotricha caprimulgaEPR01|658[0n]|BOLD:AAP9411
Hymenochaonia kalevikulli|[7]|DHJPAR0040571|Antaeotricha caprimulgaEPR01|658[0n]|BOLD:AAP9411
Hymenochaonia kalevikulli|[8]|DHJPAR0040584|Antaeotricha caprimulgaEPR01|658[0n]|BOLD:AAP9411
Hymenochaonia kalevikulli|[9]|DHJPAR0040574|Antaeotricha caprimulgaEPR01|658[0n]|BOLD:AAP9411
Hymenochaonia kalevikulli|[10]|DHJPAR0040579|Antaeotricha caprimulgaEPR01|658[0n]|BOLD:AAP9411
Hymenochaonia kalevikulli|[11]|DHJPAR0040572|Antaeotricha caprimulgaEPR01|658[0n]|BOLD:AAP9411
Hymenochaonia kalevikulli|[12]|DHJPAR0040578|Antaeotricha caprimulgaEPR01|658[0n]|BOLD:AAP9411
Hymenochaonia kalevikulli|[13]|DHJPAR0040595|Antaeotricha caprimulgaEPR02|658[0n]|BOLD:AAP9411
Hymenochaonia kalevikulli|[14]|DHJPAR0040567|Antaeotricha caprimulgaEPR01|658[0n]|BOLD:AAP9411
Hymenochaonia kalevikulli|[15]|DHJPAR0040587|Antaeotricha caprimulgaEPR01|655[0n]|BOLD:AAP9411
Hymenochaonia kalevikulli|[16]|DHJPAR0040484|Antaeotricha caprimulgaEPR01|658[0n]|BOLD:AAP9411
Macrocentrus gustavogutierrezi|[17]|DHJPAR0028981|Asturodes fimbriauralisDHJ02|657[0n]|BOLD:AC...
Macrocentrus gustavogutierrezi|[18]|DHJPAR0038831|Asturodes fimbriauralisDHJ02|658[0n]|BOLD:AC...
Macrocentrus gustavogutierrezi|[19]|DHJPAR0029390|Asturodes fimbriauralisDHJ02|657[0n]|BOLD:AC...
Macrocentrus gustavogutierrezi|[20]|DHJPAR0029392|Asturodes fimbriauralisDHJ02|657[0n]|BOLD:AC...
Macrocentrus gustavogutierrezi|[21]|DHJPAR0029349|Asturodes fimbriauralisDHJ02|657[0n]|BOLD:AC...
Macrocentrus gustavogutierrezi|[22]|DHJPAR0029393|Asturodes fimbriauralisDHJ02|657[0n]|BOLD:AC...
Macrocentrus gustavogutierrezi|[23]|DHJPAR0029391|Asturodes fimbriauralisDHJ02|657[0n]|BOLD:AC...
Macrocentrus gustavogutierrezi|[24]|DHJPAR0029076|Antaeotricha spurca|657[0n]|BOLD:ACK7466
Macrocentrus gustavogutierrezi|[25]|DHJPAR0055165|Asturodes fimbriauralisDHJ02|658[0n]|BOLD:AC...
Macrocentrus gustavogutierrezi|[26]|DHJPAR0029384|Asturodes fimbriauralisDHJ02|632[0n]|BOLD:AC...
Macrocentrus gustavogutierrezi|[27]|DHJPAR0036731|Asturodes fimbriauralisDHJ02|649[0n]|BOLD:AC...
Macrocentrus gustavogutierrezi|[28]|DHJPAR0029382|Asturodes fimbriauralisDHJ02|279[0n]
Macrocentrus gustavogutierrezi|[29]|DHJPAR0050803|Asturodes fimbriauralisDHJ02|661[0n]|BOLD:AC...
Macrocentrus gustavogutierrezi|[30]|DHJPAR0037707|Asturodes fimbriauralisDHJ02|658[0n]|BOLD:AC...
Macrocentrus gustavogutierrezi|[31]|DHJPAR0029386|Asturodes fimbriauralisDHJ02|657[0n]|BOLD:AC...
Macrocentrus gustavogutierrezi|[32]|DHJPAR0029385|Asturodes fimbriauralisDHJ02|279[0n]
Macrocentrus gustavogutierrezi|[33]|DHJPAR0029389|Asturodes fimbriauralisDHJ02|657[0n]|BOLD:AC...
Macrocentrus gustavogutierrezi|[34]|DHJPAR0028987|Asturodes fimbriauralisDHJ02|421[0n]
Macrocentrus gustavogutierrezi|[35]|DHJPAR0029383|Asturodes fimbriauralisDHJ02|632[0n]|BOLD:AC...
Macrocentrus gustavogutierrezi|[36]|DHJPAR0028997|Asturodes fimbriauralisDHJ02|657[0n]|BOLD:AC...
Macrocentrus gustavogutierrezi|[37]|DHJPAR0038832|Asturodes fimbriauralisDHJ02|658[0n]|BOLD:AC...
Macrocentrus gustavogutierrezi|[38]|DHJPAR0029360|Asturodes fimbriauralisDHJ02|634[0n]|BOLD:AC...
Macrocentrus gustavogutierrezi|[39]|DHJPAR0029340|Asturodes fimbriauralisDHJ02|632[0n]|BOLD:AC...
Macrocentrus gustavogutierrezi|[40]|DHJPAR0029339|Asturodes fimbriauralisDHJ02|632[0n]|BOLD:AC...
Macrocentrus gustavogutierrezi|[41]|DHJPAR0029338|Asturodes fimbriauralisDHJ02|630[0n]|BOLD:AC...
Macrocentrus gustavogutierrezi|[42]|DHJPAR0029388|Asturodes fimbriauralisDHJ02|657[0n]|BOLD:AC...
Macrocentrus gustavogutierrezi|[43]|DHJPAR0036261|Asturodes fimbriauralisDHJ02|661[0n]|BOLD:AC...
Macrocentrus gustavogutierrezi|[44]|DHJPAR0028998|Asturodes fimbriauralisDHJ02|657[0n]|BOLD:AC...
Macrocentrus gustavogutierrezi|[45]|DHJPAR0061074|Asturodes fimbriauralisDHJ02|658[1n]|BOLD:AC...
Macrocentrus gustavogutierrezi|[46]|DHJPAR0029380|Asturodes fimbriauralisDHJ02|646[1n]|BOLD:AC...
Macrocentrus gustavogutierrezi|[47]|DHJPAR0029379|Asturodes fimbriauralisDHJ02|646[0n]|BOLD:AC...
Macrocentrus gustavogutierrezi|[48]|DHJPAR0050856|Asturodes fimbriauralisDHJ02|203[0n]
Macrocentrus gustavogutierrezi|[49]|DHJPAR0029341|Asturodes fimbriauralisDHJ02|657[0n]|BOLD:AC...
Macrocentrus gustavogutierrezi|[50]|DHJPAR0053557|Asturodes fimbriauralisDHJ02|662[0n]|BOLD:AC...
Macrocentrus gustavogutierrezi|[51]|DHJPAR0028986|Asturodes fimbriauralisDHJ02|642[0n]|BOLD:AC...
Macrocentrus Sharkey6319|[52]|DHJPAR0056319|Pilocrocis ramentalis|661[0n]|BOLD:ACK7466
Macrocentrus Sharkey6006|[53]|DHJPAR0056006|Pilocrocis ramentalis|670[0n]|BOLD:ACK7466
Macrocentrus lucianocapelli|[54]|DHJPAR0054348|Apogeshna stenialisDHJ03|658[0n]|BOLD:ACK7466
Macrocentrus Sharkey5380|[55]|DHJPAR0045380|Desmia ploralisDHJ10|658[4n]|BOLD:ACK7466
Macrocentrus geoffbarnardi|[56]|DHJPAR0040081|Syllepis marialis|658[0n]|BOLD:ACK7466
Macrocentrus geoffbarnardi|[57]|DHJPAR0048712|Syllepis marialis|658[0n]|BOLD:ACK7466
Macrocentrus geoffbarnardi|[58]|DHJPAR0038891|Syllepis marialis|658[0n]|BOLD:ACK7466
Macrocentrus geoffbarnardi|[59]|DHJPAR0038828|Syllepis marialis|658[0n]|BOLD:ACK7466
Macrocentrus geoffbarnardi|[60]|DHJPAR0028994|Syllepis hortalis|657[0n]|BOLD:ACK7466
Macrocentrus geoffbarnardi|[61]|DHJPAR0028992|Syllepis hortalis|657[0n]|BOLD:ACK7466
Macrocentrus geoffbarnardi|[62]|DHJPAR0050916|Syllepis marialis|658[0n]|BOLD:ACK7466
Macrocentrus geoffbarnardi|[63]|DHJPAR0049246|Syllepis marialis|658[0n]|BOLD:ACK7466
Macrocentrus geoffbarnardi|[64]|DHJPAR0028995|Syllepis hortalis|657[0n]|BOLD:ACK7466
Macrocentrus geoffbarnardi|[65]|DHJPAR0035268|Syllepis marialis|658[0n]|BOLD:ACK7466
Macrocentrus geoffbarnardi|[66]|DHJPAR0039162|Syllepis marialis|658[0n]|BOLD:ACK7466
Macrocentrus Sharkey3940|[67]|DHJPAR0063940|Lamprosema Dapkey23|630[0n]|BOLD:ACK7466
Macrocentrus gretchendailyae|[68]|DHJPAR0054411|Piletosoma thialis|658[0n]|BOLD:ACK7466
Macrocentrus gretchendailyae|[69]|DHJPAR0035152|Piletosoma thialis|658[0n]|BOLD:ACK7466
Macrocentrus gretchendailyae|[70]|DHJPAR0035151|Piletosoma thialis|658[0n]|BOLD:ACK7466
Macrocentrus gretchendailyae|[71]|DHJPAR0035305|Piletosoma thialis|658[0n]|BOLD:ACK7466
Macrocentrus gretchendailyae|[72]|DHJPAR0040082|Piletosoma thialis|658[0n]|BOLD:ACK7466
Macrocentrus gretchendailyae|[73]|DHJPAR0038018|Piletosoma thialis|658[0n]|BOLD:ACK7466
Macrocentrus gretchendailyae|[74]|DHJPAR0063035|Stenoma Janzen44|658[0n]|BOLD:ACK7466
Macrocentrus gretchendailyae|[75]|DHJPAR0035264|Piletosoma thialis|636[0n]|BOLD:ACK7466
Macrocentrus gretchendailyae|[76]|DHJPAR0035262|Piletosoma thialis|630[0n]|BOLD:ACK7466
Macrocentrus gretchendailyae|[77]|DHJPAR0017227|Piletosoma thialis|660[0n]|BOLD:ACK7466
Macrocentrus gretchendailyae|[78]|DHJPAR0063008|Stenoma Janzen44|658[0n]|BOLD:ACK7466
Macrocentrus gretchendailyae|[79]|DHJPAR0035306|Piletosoma thialis|658[0n]|BOLD:ACK7466
Macrocentrus gretchendailyae|[80]|DHJPAR0063009|Stenoma Janzen44|658[0n]|BOLD:ACK7466
Macrocentrus gretchendailyae|[81]|DHJPAR0035269|Piletosoma thialis|658[0n]|BOLD:ACK7466
Macrocentrus grettelvegae|[82]|DHJPAR0045377|Desmia ploralisDHJ10|658[1n]|BOLD:ACK7466
Macrocentrus grettelvegae|[83]|DHJPAR0045379|Desmia ploralis|658[1n]|BOLD:ACK7466
Macrocentrus grettelvegae|[84]|DHJPAR0040350|Desmia benealisDHJ03|658[1n]|BOLD:ACK7466
Macrocentrus Sharkey08|[85]|DHJPAR0058830|Phaedropsis leialisDHJ03|658[0n]|BOLD:ACK7466
Macrocentrus Sharkey08|[86]|DHJPAR0063986|Phaedropsis leialis|658[0n]|BOLD:ACK7466
Macrocentrus Sharkey08|[87]|DHJPAR0063967|Phaedropsis leialis|630[0n]|BOLD:ACK7466

Macrocentrus Sharkey08|[85]|DHJPAR0058830|Phaedropsis leialisDHJ03|658[0n]|BOLD:ACK7466
Macrocentrus Sharkey08|[86]|DHJPAR0063986|Phaedropsis leialis|658[0n]|BOLD:ACK7466
Macrocentrus Sharkey08|[87]|DHJPAR0063967|Phaedropsis leialis|630[0n]|BOLD:ACK7466
Macrocentrus gregburtoni|[88]|DHJPAR0039541|Herpetogramma phaeopteralis|658[0n]|BOLD:ACK7466
Macrocentrus fredsingeri|[89]|DHJPAR0041500|Neurophyseta clymenalisDHJ03|658[0n]|BOLD:ACK7466
Macrocentrus fredsingeri|[90]|DHJPAR0042878|Neurophyseta clymenalisDHJ03|658[0n]|BOLD:ACK7466
Macrocentrus fredsingeri|[91]|DHJPAR0042865|Neurophyseta clymenalisDHJ03|658[0n]|BOLD:ACK7466
Macrocentrus fredsingeri|[92]|DHJPAR0043228|Neurophyseta clymenalisDHJ03|658[0n]|BOLD:ACK7466
Macrocentrus fredsingeri|[93]|DHJPAR0042874|Neurophyseta clymenalisDHJ03|658[0n]|BOLD:ACK7466
Macrocentrus fredsingeri|[94]|DHJPAR0045385|Neurophyseta clymenalisDHJ03|658[0n]|BOLD:ACK7466
Macrocentrus fredsingeri|[95]|DHJPAR0041474|Neurophyseta clymenalisDHJ03|658[0n]|BOLD:ACK7466
Macrocentrus fredsingeri|[96]|DHJPAR0042876|Neurophyseta clymenalisDHJ03|658[0n]|BOLD:ACK7466
Macrocentrus fredsingeri|[97]|DHJPAR0042867|Neurophyseta clymenalisDHJ03|658[0n]|BOLD:ACK7466
Macrocentrus fredsingeri|[98]|DHJPAR0042869|Neurophyseta clymenalisDHJ03|658[0n]|BOLD:ACK7466
Macrocentrus fredsingeri|[99]|DHJPAR0041508|Neurophyseta clymenalisDHJ03|632[0n]|BOLD:ACK7466
Macrocentrus fredsingeri|[100]|DHJPAR0041528|Neurophyseta clymenalisDHJ03|658[0n]|BOLD:ACK7466
Macrocentrus fredsingeri|[101]|DHJPAR0042875|Neurophyseta clymenalisDHJ03|633[0n]|BOLD:ACK7466
Macrocentrus fredsingeri|[102]|DHJPAR0041484|Neurophyseta clymenalisDHJ03|658[0n]|BOLD:ACK7466
Macrocentrus fredsingeri|[103]|DHJPAR0041458|Neurophyseta clymenalisDHJ03|658[0n]|BOLD:ACK7466
Macrocentrus fredsingeri|[104]|DHJPAR0041505|Neurophyseta clymenalisDHJ03|658[0n]|BOLD:ACK7466
Macrocentrus fredsingeri|[105]|DHJPAR0041477|Neurophyseta clymenalisDHJ03|658[0n]|BOLD:ACK7466
Macrocentrus fredsingeri|[106]|DHJPAR0041486|Neurophyseta clymenalisDHJ03|658[0n]|BOLD:ACK7466
Macrocentrus fredsingeri|[107]|DHJPAR0042866|Neurophyseta clymenalisDHJ03|658[0n]|BOLD:ACK7466
Macrocentrus fredsingeri|[108]|DHJPAR0042868|Neurophyseta clymenalisDHJ03|658[0n]|BOLD:ACK7466
Macrocentrus fredsingeri|[109]|DHJPAR0041501|Neurophyseta clymenalisDHJ03|658[0n]|BOLD:ACK7466
Macrocentrus fredsingeri|[110]|DHJPAR0041479|Neurophyseta clymenalisDHJ03|658[0n]|BOLD:ACK7466
Macrocentrus fredsingeri|[111]|DHJPAR0037967|Neurophyseta clymenalisDHJ03|658[0n]|BOLD:ACK7466
Macrocentrus fredsingeri|[112]|DHJPAR0041476|Neurophyseta clymenalisDHJ03|658[0n]|BOLD:ACK7466
Macrocentrus fredsingeri|[113]|DHJPAR0041522|Neurophyseta clymenalisDHJ03|658[0n]|BOLD:ACK7466
Macrocentrus fredsingeri|[114]|DHJPAR0041481|Neurophyseta clymenalisDHJ03|658[0n]|BOLD:ACK7466
Macrocentrus fredsingeri|[115]|DHJPAR0051407|Neurophyseta clymenalisDHJ03|661[0n]|BOLD:ACK7466
Macrocentrus fredsingeri|[116]|DHJPAR0042877|Neurophyseta clymenalisDHJ03|658[0n]|BOLD:ACK7466
Macrocentrus fredsingeri|[117]|DHJPAR0041480|Neurophyseta clymenalisDHJ03|658[0n]|BOLD:ACK7466
Macrocentrus fredsingeri|[118]|DHJPAR0042871|Neurophyseta clymenalisDHJ03|658[0n]|BOLD:ACK7466
Macrocentrus fredsingeri|[119]|DHJPAR0041483|Neurophyseta clymenalisDHJ03|658[0n]|BOLD:ACK7466
Macrocentrus fredsingeri|[120]|DHJPAR0041489|Neurophyseta clymenalisDHJ03|658[0n]|BOLD:ACK7466
Macrocentrus Janzen3861|[121]|DHJPAR0063861|spiloJanzen01 Janzen88|658[0n]|BOLD:ACK7466
Macrocentrus Janzen3861|[122]|DHJPAR0063862|spiloJanzen01 Janzen88|538[0n]|BOLD:ACK7466
Macrocentrus hannahjamesae|[123]|DHJPAR0045376|Syllepis marialis|658[1n]|BOLD:ABA9321
Macrocentrus hannahjamesae|[124]|DHJPAR0045386|Syllepis marialis|658[0n]|BOLD:ABA9321
Macrocentrus hannahjamesae|[125]|DHJPAR0036308|Syllepis marialis|658[0n]|BOLD:ABA9321
Macrocentrus hannahjamesae|[126]|DHJPAR0036307|Syllepis marialis|658[0n]|BOLD:ABA9321
Macrocentrus hannahjamesae|[127]|DHJPAR0028984|Syllepis marialis|657[2n]|BOLD:ABA9321
Macrocentrus hillaryrosnerae|[128]|DHJPAR0043202|Undulambia Solis02|658[0n]|BOLD:ABA7284
Macrocentrus hiroshikidonoi|[129]|DHJPAR0054926|Undulambia Solis02|618[0n]|BOLD:ACK7467
Macrocentrus hiroshikidonoi|[130]|DHJPAR0052380|Neurophyseta camptogrammalisDHJ01|620[0n]|BOLD ...
Macrocentrus hiroshikidonoi|[131]|DHJPAR0053148|Undulambia Solis02|624[0n]|BOLD:ACK7467
Macrocentrus hiroshikidonoi|[132]|DHJPAR0038466|Diacme BioLep02|658[0n]|BOLD:ACK7467
Macrocentrus hiroshikidonoi|[133]|DHJPAR0050011|Neurophyseta camptogrammalisDHJ01|658[0n]|BOLD ...
Macrocentrus hiroshikidonoi|[134]|DHJPAR0041406|Diacme BioLep02|658[0n]|BOLD:ACK7467
Macrocentrus hiroshikidonoi|[135]|DHJPAR0045381|Undulambia Solis02|658[1n]|BOLD:ACK7467
Macrocentrus hiroshikidonoi|[136]|DHJPAR0045382|Undulambia Solis02|658[1n]|BOLD:ACK7467
Macrocentrus hiroshikidonoi|[137]|DHJPAR0041399|Undulambia Solis02|658[0n]|BOLD:ACK7467
Macrocentrus hiroshikidonoi|[138]|DHJPAR0041504|Undulambia Solis02|658[0n]|BOLD:ACK7467
Macrocentrus hiroshikidonoi|[139]|DHJPAR0041498|Neurophyseta completalis|658[0n]|BOLD:ACK7467
Macrocentrus hiroshikidonoi|[140]|DHJPAR0041462|Undulambia Solis02|658[0n]|BOLD:ACK7467
Macrocentrus hiroshikidonoi|[141]|DHJPAR0028990|Neurophyseta Janzen229|657[0n]|BOLD:ACK7467
Macrocentrus hiroshikidonoi|[142]|DHJPAR0053668|Undulambia Solis02|658[0n]|BOLD:ACK7467
Macrocentrus hiroshikidonoi|[143]|DHJPAR0053667|Undulambia Solis02|658[0n]|BOLD:ACK7467
Macrocentrus hiroshikidonoi|[144]|DHJPAR0051422|Undulambia Solis02|661[0n]|BOLD:ACK7467
Macrocentrus hiroshikidonoi|[145]|DHJPAR0041495|Undulambia Solis02|658[0n]|BOLD:ACK7467
Macrocentrus hiroshikidonoi|[146]|DHJPAR0050013|Neurophyseta camptogrammalisDHJ01|658[0n]|BOLD ...
Macrocentrus hiroshikidonoi|[147]|DHJPAR0051418|Undulambia Solis02|661[0n]|BOLD:ACK7467
Macrocentrus hiroshikidonoi|[148]|DHJPAR0043250|Undulambia Solis02|658[0n]|BOLD:ACK7467
Macrocentrus hiroshikidonoi|[149]|DHJPAR0041459|Undulambia Solis02|658[0n]|BOLD:ACK7467
Macrocentrus hiroshikidonoi|[150]|DHJPAR0052327|Undulambia Solis02|658[0n]|BOLD:ACK7467
Macrocentrus hiroshikidonoi|[151]|DHJPAR0050018|Neurophyseta camptogrammalisDHJ01|658[0n]|BOLD ...
Macrocentrus hiroshikidonoi|[152]|DHJPAR0052193|Undulambia Solis02|658[0n]|BOLD:ACK7467
Macrocentrus hiroshikidonoi|[153]|DHJPAR0023710|Diacme BioLep02|657[0n]|BOLD:ACK7467
Macrocentrus hiroshikidonoi|[154]|DHJPAR0051322|Neurophyseta camptogrammalisDHJ01|658[0n]|BOLD ...
Macrocentrus hiroshikidonoi|[155]|DHJPAR0050014|Undulambia Solis02|658[0n]|BOLD:ACK7467
Macrocentrus hiroshikidonoi|[156]|DHJPAR0041519|Undulambia Solis02|658[0n]|BOLD:ACK7467
Macrocentrus hiroshikidonoi|[157]|DHJPAR0050017|Neurophyseta camptogrammalisDHJ01|658[0n]|BOLD ...
Macrocentrus hiroshikidonoi|[158]|DHJPAR0053669|Undulambia Solis02|658[0n]|BOLD:ACK7467
Macrocentrus hiroshikidonoi|[159]|DHJPAR0041499|Neurophyseta completalis|658[0n]|BOLD:ACK7467
Macrocentrus hiroshikidonoi|[160]|DHJPAR0042870|Undulambia Solis02|658[0n]|BOLD:ACK7467
Macrocentrus hiroshikidonoi|[161]|DHJPAR0048239|Undulambia Solis02|658[0n]|BOLD:ACK7467
Macrocentrus hiroshikidonoi|[162]|DHJPAR0050020|Neurophyseta camptogrammalisDHJ01|658[0n]|BOLD ...
Macrocentrus hiroshikidonoi|[163]|DHJPAR0041509|Neurophyseta completalis|658[0n]|BOLD:ACK7467
Macrocentrus hiroshikidonoi|[164]|DHJPAR0043239|Undulambia Solis02|658[0n]|BOLD:ACK7467
Macrocentrus hiroshikidonoi|[165]|DHJPAR0045503|Neurophyseta camptogrammalisDHJ01|658[0n]|BOLD ...
Macrocentrus hiroshikidonoi|[166]|DHJPAR0050022|Neurophyseta camptogrammalisDHJ01|658[0n]|BOLD ...
Macrocentrus hiroshikidonoi|[167]|DHJPAR0041490|Neurophyseta completalis|658[0n]|BOLD:ACK7467
Macrocentrus hiroshikidonoi|[168]|DHJPAR0050012|Neurophyseta camptogrammalisDHJ01|658[0n]|BOLD ...
Macrocentrus hiroshikidonoi|[169]|DHJPAR0041544|Undulambia Solis02|658[0n]|BOLD:ACK7467
Macrocentrus hiroshikidonoi|[170]|DHJPAR0041512|Neurophyseta clymenalisDHJ03|658[0n]|BOLD:ACK7467
Macrocentrus hiroshikidonoi|[171]|DHJPAR0056340|Neurophyseta camptogrammalisDHJ01|661[0n]|BOLD ...
Macrocentrus hiroshikidonoi|[172]|DHJPAR0028989|Neurophyseta Janzen229|657[0n]|BOLD:ACK7467
Macrocentrus hiroshikidonoi|[173]|DHJPAR0051320|Undulambia Solis02|658[0n]|BOLD:ACK7467
[illegible]

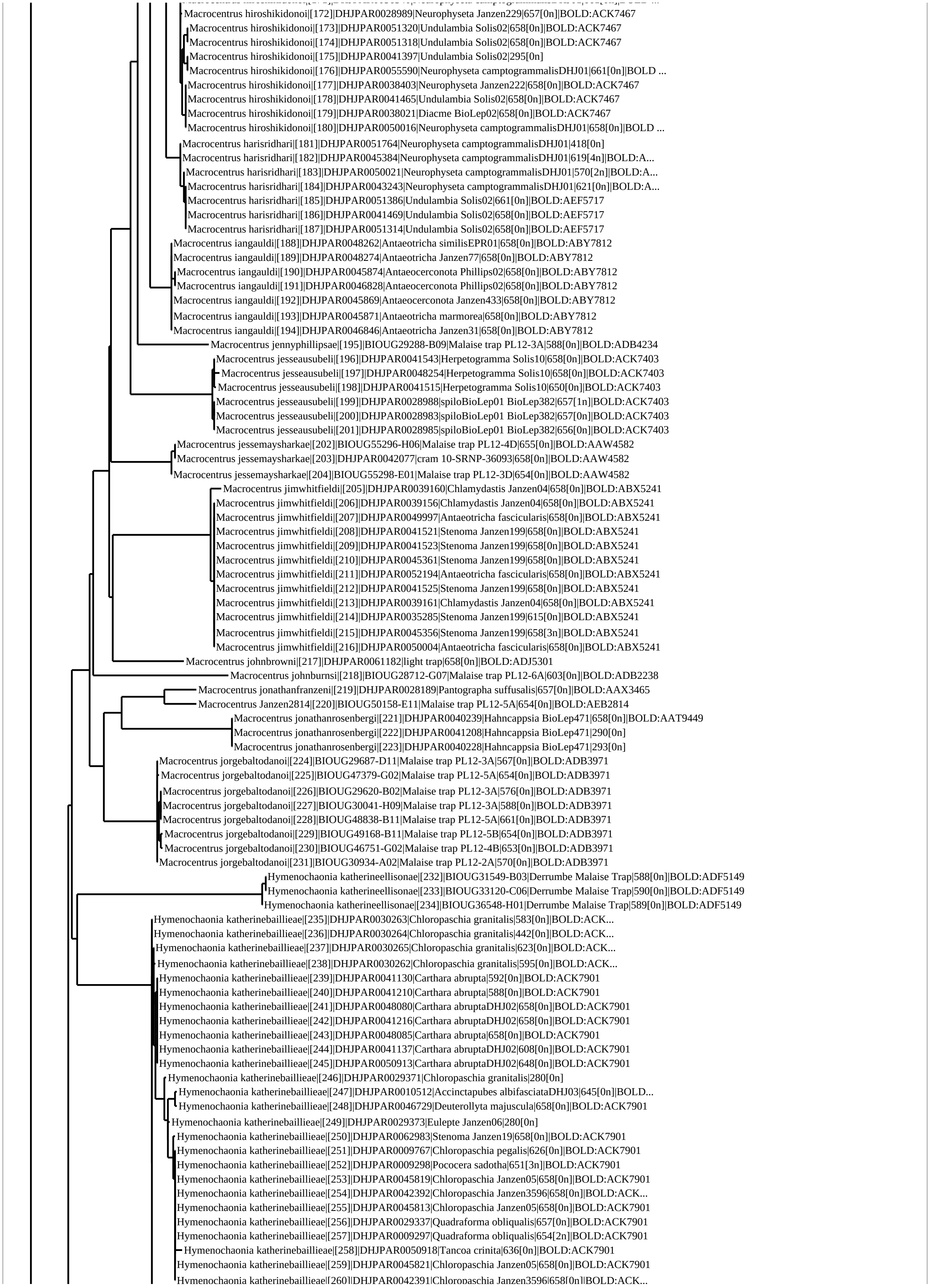
Macrocentrus hiroshikidonoi|[172]|DHJPAR0028989|Neurophyseta Janzen229|657[0n]|BOLD:ACK7467
Macrocentrus hiroshikidonoi|[173]|DHJPAR0051320|Undulambia Solis02|658[0n]|BOLD:ACK7467
Macrocentrus hiroshikidonoi|[174]|DHJPAR0051318|Undulambia Solis02|658[0n]|BOLD:ACK7467
Macrocentrus hiroshikidonoi|[175]|DHJPAR0041397|Undulambia Solis02|295[0n]
Macrocentrus hiroshikidonoi|[176]|DHJPAR0055590|Neurophyseta camptogrammalisDHJ01|661[0n]|BOLD ...
Macrocentrus hiroshikidonoi|[177]|DHJPAR0038403|Neurophyseta Janzen222|658[0n]|BOLD:ACK7467
Macrocentrus hiroshikidonoi|[178]|DHJPAR0041465|Undulambia Solis02|658[0n]|BOLD:ACK7467
Macrocentrus hiroshikidonoi|[179]|DHJPAR0038021|Diacme BioLep02|658[0n]|BOLD:ACK7467
Macrocentrus hiroshikidonoi|[180]|DHJPAR0050016|Neurophyseta camptogrammalisDHJ01|658[0n]|BOLD ...
Macrocentrus harisridhari|[181]|DHJPAR0051764|Neurophyseta camptogrammalisDHJ01|418[0n]
Macrocentrus harisridhari|[182]|DHJPAR0045384|Neurophyseta camptogrammalisDHJ01|619[4n]|BOLD:A...
Macrocentrus harisridhari|[183]|DHJPAR0050021|Neurophyseta camptogrammalisDHJ01|570[2n]|BOLD:A...
Macrocentrus harisridhari|[184]|DHJPAR0043243|Neurophyseta camptogrammalisDHJ01|621[0n]|BOLD:A...
Macrocentrus harisridhari|[185]|DHJPAR0051386|Undulambia Solis02|661[0n]|BOLD:AEF5717
Macrocentrus harisridhari|[186]|DHJPAR0041469|Undulambia Solis02|658[0n]|BOLD:AEF5717
Macrocentrus harisridhari|[187]|DHJPAR0051314|Undulambia Solis02|658[0n]|BOLD:AEF5717
Macrocentrus iangauldi|[188]|DHJPAR0048262|Antaeotricha similisEPR01|658[0n]|BOLD:ABY7812
Macrocentrus iangauldi|[189]|DHJPAR0048274|Antaeotricha Janzen77|658[0n]|BOLD:ABY7812
Macrocentrus iangauldi|[190]|DHJPAR0045874|Antaeocerconota Phillips02|658[0n]|BOLD:ABY7812
Macrocentrus iangauldi|[191]|DHJPAR0046828|Antaeocerconota Phillips02|658[0n]|BOLD:ABY7812
Macrocentrus iangauldi|[192]|DHJPAR0045869|Antaeocerconota Janzen433|658[0n]|BOLD:ABY7812
Macrocentrus iangauldi|[193]|DHJPAR0045871|Antaeotricha marmorea|658[0n]|BOLD:ABY7812
Macrocentrus iangauldi|[194]|DHJPAR0046846|Antaeotricha Janzen31|658[0n]|BOLD:ABY7812
Macrocentrus jennyphillipsae|[195]|BIOUG29288-B09|Malaise trap PL12-3A|588[0n]|BOLD:ADB4234
Macrocentrus jesseausubeli|[196]|DHJPAR0041543|Herpetogramma Solis10|658[0n]|BOLD:ACK7403
Macrocentrus jesseausubeli|[197]|DHJPAR0048254|Herpetogramma Solis10|658[0n]|BOLD:ACK7403
Macrocentrus jesseausubeli|[198]|DHJPAR0041515|Herpetogramma Solis10|650[0n]|BOLD:ACK7403
Macrocentrus jesseausubeli|[199]|DHJPAR0028988|spiloBioLep01 BioLep382|657[1n]|BOLD:ACK7403
Macrocentrus jesseausubeli|[200]|DHJPAR0028983|spiloBioLep01 BioLep382|657[0n]|BOLD:ACK7403
Macrocentrus jesseausubeli|[201]|DHJPAR0028985|spiloBioLep01 BioLep382|656[0n]|BOLD:ACK7403
Macrocentrus jessemaysharkae|[202]|BIOUG55296-H06|Malaise trap PL12-4D|655[0n]|BOLD:AAW4582
Macrocentrus jessemaysharkae|[203]|DHJPAR0042077|cram 10-SRNP-36093|658[0n]|BOLD:AAW4582
Macrocentrus jessemaysharkae|[204]|BIOUG55298-E01|Malaise trap PL12-3D|654[0n]|BOLD:AAW4582
Macrocentrus jimwhitfieldi|[205]|DHJPAR0039160|Chlamydastis Janzen04|658[0n]|BOLD:ABX5241
Macrocentrus jimwhitfieldi|[206]|DHJPAR0039156|Chlamydastis Janzen04|658[0n]|BOLD:ABX5241
Macrocentrus jimwhitfieldi|[207]|DHJPAR0049997|Antaeotricha fascicularis|658[0n]|BOLD:ABX5241
Macrocentrus jimwhitfieldi|[208]|DHJPAR0041521|Stenoma Janzen199|658[0n]|BOLD:ABX5241
Macrocentrus jimwhitfieldi|[209]|DHJPAR0041523|Stenoma Janzen199|658[0n]|BOLD:ABX5241
Macrocentrus jimwhitfieldi|[210]|DHJPAR0045361|Stenoma Janzen199|658[0n]|BOLD:ABX5241
Macrocentrus jimwhitfieldi|[211]|DHJPAR0052194|Antaeotricha fascicularis|658[0n]|BOLD:ABX5241
Macrocentrus jimwhitfieldi|[212]|DHJPAR0041525|Stenoma Janzen199|658[0n]|BOLD:ABX5241
Macrocentrus jimwhitfieldi|[213]|DHJPAR0039161|Chlamydastis Janzen04|658[0n]|BOLD:ABX5241
Macrocentrus jimwhitfieldi|[214]|DHJPAR0035285|Stenoma Janzen199|615[0n]|BOLD:ABX5241
Macrocentrus jimwhitfieldi|[215]|DHJPAR0045356|Stenoma Janzen199|658[3n]|BOLD:ABX5241
Macrocentrus jimwhitfieldi|[216]|DHJPAR0050004|Antaeotricha fascicularis|658[0n]|BOLD:ABX5241
Macrocentrus johnbrowni|[217]|DHJPAR0061182|light trap|658[0n]|BOLD:ADJ5301
Macrocentrus johnburnsi|[218]|BIOUG28712-G07|Malaise trap PL12-6A|603[0n]|BOLD:ADB2238
Macrocentrus jonathanfranzeni|[219]|DHJPAR0028189|Pantographa suffusalis|657[0n]|BOLD:AAX3465
Macrocentrus Janzen2814|[220]|BIOUG50158-E11|Malaise trap PL12-5A|654[0n]|BOLD:AEB2814
Macrocentrus jonathanrosenbergi|[221]|DHJPAR0040239|Hahncappsia BioLep471|658[0n]|BOLD:AAT9449
Macrocentrus jonathanrosenbergi|[222]|DHJPAR0041208|Hahncappsia BioLep471|290[0n]
Macrocentrus jonathanrosenbergi|[223]|DHJPAR0040228|Hahncappsia BioLep471|293[0n]
Macrocentrus jorgebaltodanoi|[224]|BIOUG29687-D11|Malaise trap PL12-3A|567[0n]|BOLD:ADB3971
Macrocentrus jorgebaltodanoi|[225]|BIOUG47379-G02|Malaise trap PL12-5A|654[0n]|BOLD:ADB3971
Macrocentrus jorgebaltodanoi|[226]|BIOUG29620-B02|Malaise trap PL12-3A|576[0n]|BOLD:ADB3971
Macrocentrus jorgebaltodanoi|[227]|BIOUG30041-H09|Malaise trap PL12-3A|588[0n]|BOLD:ADB3971
Macrocentrus jorgebaltodanoi|[228]|BIOUG48838-B11|Malaise trap PL12-5A|661[0n]|BOLD:ADB3971
Macrocentrus jorgebaltodanoi|[229]|BIOUG49168-B11|Malaise trap PL12-5B|654[0n]|BOLD:ADB3971
Macrocentrus jorgebaltodanoi|[230]|BIOUG46751-G02|Malaise trap PL12-4B|653[0n]|BOLD:ADB3971
Macrocentrus jorgebaltodanoi|[231]|BIOUG30934-A02|Malaise trap PL12-2A|570[0n]|BOLD:ADB3971
Hymenochaonia katherineellisonae|[232]|BIOUG31549-B03|Derrumbe Malaise Trap|588[0n]|BOLD:ADF5149
Hymenochaonia katherineellisonae|[233]|BIOUG33120-C06|Derrumbe Malaise Trap|590[0n]|BOLD:ADF5149
Hymenochaonia katherineellisonae|[234]|BIOUG36548-H01|Derrumbe Malaise Trap|589[0n]|BOLD:ADF5149
Hymenochaonia katherinebaillieae|[235]|DHJPAR0030263|Chloropaschia granitalis|583[0n]|BOLD:ACK...
Hymenochaonia katherinebaillieae|[236]|DHJPAR0030264|Chloropaschia granitalis|442[0n]|BOLD:ACK...
Hymenochaonia katherinebaillieae|[237]|DHJPAR0030265|Chloropaschia granitalis|623[0n]|BOLD:ACK...
Hymenochaonia katherinebaillieae|[238]|DHJPAR0030262|Chloropaschia granitalis|595[0n]|BOLD:ACK...
Hymenochaonia katherinebaillieae|[239]|DHJPAR0041130|Carthara abrupta|592[0n]|BOLD:ACK7901
Hymenochaonia katherinebaillieae|[240]|DHJPAR0041210|Carthara abrupta|588[0n]|BOLD:ACK7901
Hymenochaonia katherinebaillieae|[241]|DHJPAR0048080|Carthara abruptaDHJ02|658[0n]|BOLD:ACK7901
Hymenochaonia katherinebaillieae|[242]|DHJPAR0041216|Carthara abruptaDHJ02|658[0n]|BOLD:ACK7901
Hymenochaonia katherinebaillieae|[243]|DHJPAR0048085|Carthara abrupta|658[0n]|BOLD:ACK7901
Hymenochaonia katherinebaillieae|[244]|DHJPAR0041137|Carthara abruptaDHJ02|608[0n]|BOLD:ACK7901
Hymenochaonia katherinebaillieae|[245]|DHJPAR0050913|Carthara abruptaDHJ02|648[0n]|BOLD:ACK7901
Hymenochaonia katherinebaillieae|[246]|DHJPAR0029371|Chloropaschia granitalis|280[0n]
Hymenochaonia katherinebaillieae|[247]|DHJPAR0010512|Accinctapubes albifasciataDHJ03|645[0n]|BOLD...
Hymenochaonia katherinebaillieae|[248]|DHJPAR0046729|Deuterollyta majuscula|658[0n]|BOLD:ACK7901
Hymenochaonia katherinebaillieae|[249]|DHJPAR0029373|Eulepte Janzen06|280[0n]
Hymenochaonia katherinebaillieae|[250]|DHJPAR0062983|Stenoma Janzen19|658[0n]|BOLD:ACK7901
Hymenochaonia katherinebaillieae|[251]|DHJPAR0009767|Chloropaschia pegalis|626[0n]|BOLD:ACK7901
Hymenochaonia katherinebaillieae|[252]|DHJPAR0009298|Pococera sadotha|651[3n]|BOLD:ACK7901
Hymenochaonia katherinebaillieae|[253]|DHJPAR0045819|Chloropaschia Janzen05|658[0n]|BOLD:ACK7901
Hymenochaonia katherinebaillieae|[254]|DHJPAR0042392|Chloropaschia Janzen3596|658[0n]|BOLD:ACK...
Hymenochaonia katherinebaillieae|[255]|DHJPAR0045813|Chloropaschia Janzen05|658[0n]|BOLD:ACK7901
Hymenochaonia katherinebaillieae|[256]|DHJPAR0029337|Quadraforma obliqualis|657[0n]|BOLD:ACK7901
Hymenochaonia katherinebaillieae|[257]|DHJPAR0009297|Quadraforma obliqualis|654[2n]|BOLD:ACK7901
Hymenochaonia katherinebaillieae|[258]|DHJPAR0050918|Tancoa crinita|636[0n]|BOLD:ACK7901
Hymenochaonia katherinebaillieae|[259]|DHJPAR0045821|Chloropaschia Janzen05|658[0n]|BOLD:ACK7901
Hymenochaonia katherinebaillieae|[260]|DHJPAR0042391|Chloropaschia Janzen3596|658[0n]|BOLD:ACK...

Hymenochaonia katherinebaillieae|[258]|DHJPAR0050918|Tancoa crinita|636[0n]|BOLD:ACK7901
Hymenochaonia katherinebaillieae|[259]|DHJPAR0045821|Chloropaschia Janzen05|658[0n]|BOLD:ACK7901
Hymenochaonia katherinebaillieae|[260]|DHJPAR0042391|Chloropaschia Janzen3596|658[0n]|BOLD:ACK...
Hymenochaonia katherinebaillieae|[261]|DHJPAR0028996|Carthara abruptaDHJ02|620[2n]|BOLD:ACK7901
Hymenochaonia katherinebaillieae|[262]|DHJPAR0041220|Carthara abrupta|619[0n]|BOLD:ACK7901
Hymenochaonia katherinebaillieae|[263]|DHJPAR0041209|Carthara abrupta|636[0n]|BOLD:ACK7901
Hymenochaonia katherinebaillieae|[264]|DHJPAR0029369|Carthara abrupta|657[0n]|BOLD:ACK7901
Hymenochaonia katherinebaillieae|[265]|DHJPAR0041214|Chloropaschia mennusalis|658[0n]|BOLD:ACK...
Hymenochaonia katherinebaillieae|[266]|DHJPAR0041221|Carthara abrupta|658[0n]|BOLD:ACK7901
Hymenochaonia katherinebaillieae|[267]|DHJPAR0041211|Carthara abrupta|636[0n]|BOLD:ACK7901
Hymenochaonia katherinebaillieae|[268]|DHJPAR0041217|Carthara abrupta|658[0n]|BOLD:ACK7901
Hymenochaonia katherinebaillieae|[269]|DHJPAR0029368|Carthara abrupta|624[0n]|BOLD:ACK7901
Hymenochaonia katherinebaillieae|[270]|DHJPAR0009657|Carthara abruptaDHJ02|655[14n]
Hymenochaonia katherinebaillieae|[271]|DHJPAR0037792|Carthara abruptaDHJ02|661[0n]|BOLD:ACK7901
Hymenochaonia katherinebaillieae|[272]|DHJPAR0041219|Carthara abruptaDHJ02|658[0n]|BOLD:ACK7901
Hymenochaonia katherinebaillieae|[273]|DHJPAR0040235|Carthara abruptaDHJ02|658[0n]|BOLD:ACK7901
Hymenochaonia katherinebaillieae|[274]|DHJPAR0040236|Carthara abruptaDHJ02|658[0n]|BOLD:ACK7901
Hymenochaonia katherinebaillieae|[275]|DHJPAR0040231|Carthara abruptaDHJ02|658[0n]|BOLD:ACK7901
Hymenochaonia katherinebaillieae|[276]|DHJPAR0048084|Carthara abrupta|658[0n]|BOLD:ACK7901
Hymenochaonia katherinebaillieae|[277]|DHJPAR0040232|Carthara abruptaDHJ02|658[0n]|BOLD:ACK7901
Hymenochaonia katherinebaillieae|[278]|DHJPAR0041134|Carthara abruptaDHJ02|605[0n]|BOLD:ACK7901
Hymenochaonia katherinebaillieae|[279]|DHJPAR0041218|Carthara abruptaDHJ02|632[0n]|BOLD:ACK7901
Hymenochaonia katherinebaillieae|[280]|DHJPAR0045055|Carthara abrupta|650[0n]|BOLD:ACK7901
Hymenochaonia katherinebaillieae|[281]|DHJPAR0040609|Carthara abruptaDHJ02|626[0n]|BOLD:ACK7901
Hymenochaonia katherinebaillieae|[282]|DHJPAR0029180|Carthara abruptaDHJ01|657[0n]|BOLD:ACK7901
Hymenochaonia katherinebaillieae|[283]|DHJPAR0040237|Carthara abruptaDHJ02|658[0n]|BOLD:ACK7901
Hymenochaonia katherinebaillieae|[284]|DHJPAR0046728|Carthara abruptaDHJ02|658[0n]|BOLD:ACK7901
Hymenochaonia katherinebaillieae|[285]|DHJPAR0046725|Accinctapubes albifasciataDHJ01|658[0n]|BOLD...
Hymenochaonia katherinebaillieae|[286]|DHJPAR0041136|Carthara abruptaDHJ02|590[0n]|BOLD:ACK7901
Hymenochaonia katherinebaillieae|[287]|DHJPAR0041133|Carthara abruptaDHJ02|589[0n]|BOLD:ACK7901
Hymenochaonia katherinebaillieae|[288]|DHJPAR0041212|Carthara abruptaDHJ02|658[0n]|BOLD:ACK7901
Hymenochaonia katherinebaillieae|[289]|DHJPAR0045829|Chloropaschia granitalis|658[0n]|BOLD:ACK...
Hymenochaonia katherinebaillieae|[290]|DHJPAR0054451|Carthara abrupta|658[0n]|BOLD:ACK7901
Hymenochaonia katherinebaillieae|[291]|DHJPAR0048082|Carthara abruptaDHJ02|658[0n]|BOLD:ACK7901
Hymenochaonia katherinebaillieae|[292]|DHJPAR0037871|Carthara abruptaDHJ02|658[0n]|BOLD:ACK7901
Hymenochaonia katherinebaillieae|[293]|DHJPAR0037866|Carthara abruptaDHJ02|658[0n]|BOLD:ACK7901
Hymenochaonia katherinebaillieae|[294]|DHJPAR0037865|Carthara abruptaDHJ02|658[0n]|BOLD:ACK7901
Hymenochaonia katherinebaillieae|[295]|DHJPAR0054448|Carthara abrupta|658[0n]|BOLD:ACK7901
Hymenochaonia katherinebaillieae|[296]|DHJPAR0054449|Carthara abrupta|658[0n]|BOLD:ACK7901
Hymenochaonia katherinebaillieae|[297]|DHJPAR0054450|Carthara abrupta|658[0n]|BOLD:ACK7901
Hymenochaonia katherinebaillieae|[298]|DHJPAR0040238|Carthara abruptaDHJ02|658[0n]|BOLD:ACK7901
Hymenochaonia katherinebaillieae|[299]|DHJPAR0037868|Carthara abruptaDHJ02|658[0n]|BOLD:ACK7901
Hymenochaonia katherinebaillieae|[300]|DHJPAR0037869|Carthara abruptaDHJ02|658[0n]|BOLD:ACK7901
Hymenochaonia katherinebaillieae|[301]|DHJPAR0040229|Carthara abruptaDHJ02|658[0n]|BOLD:ACK7901
Hymenochaonia katherinebaillieae|[302]|DHJPAR0041213|Carthara abruptaDHJ02|658[0n]|BOLD:ACK7901
Hymenochaonia katherinebaillieae|[303]|DHJPAR0054452|Carthara abrupta|658[0n]|BOLD:ACK7901
Hymenochaonia katherinebaillieae|[304]|DHJPAR0040230|Carthara abruptaDHJ02|658[0n]|BOLD:ACK7901
Hymenochaonia katherinebaillieae|[305]|DHJPAR0048088|Carthara abruptaDHJ02|658[0n]|BOLD:ACK7901
Hymenochaonia katherinebaillieae|[306]|DHJPAR0037867|Carthara abruptaDHJ02|658[0n]|BOLD:ACK7901
Hymenochaonia katherinebaillieae|[307]|DHJPAR0037870|Carthara abruptaDHJ02|658[0n]|BOLD:ACK7901
Hymenochaonia katherinebaillieae|[308]|DHJPAR0055123|Carthara abruptaDHJ02|658[0n]|BOLD:ACK7901
Hymenochaonia katherinebaillieae|[309]|DHJPAR0028980|Carthara abruptaDHJ02|657[0n]|BOLD:ACK7901
Hymenochaonia katherinebaillieae|[310]|DHJPAR0009658|Carthara abruptaDHJ02|603[2n]|BOLD:ACK7901
Hymenochaonia katherinebaillieae|[311]|DHJPAR0045378|Carthara abruptaDHJ02|658[1n]|BOLD:ACK7901
Hymenochaonia katherinebaillieae|[312]|DHJPAR0045072|Chloropaschia granitalis|654[0n]|BOLD:ACK...
Hymenochaonia katherinebaillieae|[313]|DHJPAR0041131|Carthara abruptaDHJ02|605[1n]|BOLD:ACK7901
Hymenochaonia katherinebaillieae|[314]|DHJPAR0041135|Carthara abruptaDHJ02|608[0n]|BOLD:ACK7901
Hymenochaonia katherinebaillieae|[315]|DHJPAR0029376|Chloropaschia granitalis|280[0n]
Hymenochaonia katherinebaillieae|[316]|DHJPAR0029372|Chloropaschia granitalis|280[0n]
Hymenochaonia katherinebaillieae|[317]|DHJPAR0041132|Carthara abruptaDHJ02|293[0n]
Hymenochaonia katherinebaillieae|[318]|DHJPAR0046727|Carthara abruptaDHJ02|658[0n]|BOLD:ACK7901
Hymenochaonia katherinebaillieae|[319]|DHJPAR0046726|Carthara abrupta|637[0n]|BOLD:ACK7901
Hymenochaonia katherinebaillieae|[320]|DHJPAR0040240|Carthara abruptaDHJ02|658[0n]|BOLD:ACK7901
Hymenochaonia Whitfield03|[321]|DHJPAR0048703|Eulepte Solis15|658[0n]|BOLD:ACB2136
Hymenochaonia Whitfield03|[322]|DHJPAR0055781|Eulepte Solis15|661[0n]|BOLD:ACB2136
Hymenochaonia Whitfield03|[323]|DHJPAR0056257|Eulepte Janzen03|670[0n]|BOLD:ACB2136
Hymenochaonia Whitfield03|[324]|DHJPAR0062967|Eulepte Solis15|658[2n]|BOLD:ACB2136
Hymenochaonia Whitfield03|[325]|DHJPAR0062968|Eulepte Solis15|658[0n]|BOLD:ACB2136
Hymenochaonia Whitfield03|[326]|DHJPAR0062969|Eulepte Solis15|658[0n]|BOLD:ACB2136
Hymenochaonia Whitfield03|[327]|DHJPAR0065298|Eulepte Janzen07|631[0n]
Hymenochaonia Whitfield03|[328]|DHJPAR0054459|Eulepte Janzen07|658[0n]|BOLD:ACB2136
Hymenochaonia Whitfield03|[329]|DHJPAR0065325|Eulepte Janzen07|636[0n]|BOLD:ACB2136
Hymenochaonia Whitfield03|[330]|DHJPAR0062966|Eulepte Solis15|658[0n]|BOLD:ACB2136
Hymenochaonia Whitfield03|[331]|DHJPAR0062970|Eulepte Solis15|658[0n]|BOLD:ACB2136
Hymenochaonia Whitfield03|[332]|DHJPAR0062965|Eulepte Solis15|658[0n]|BOLD:ACB2136
Hymenochaonia katyvandusenae|[333]|DHJPAR0039536|Pilocrocis purpurascens|623[0n]|BOLD:AAL5547
Hymenochaonia katyvandusenae|[334]|DHJPAR0052093|Pilocrocis purpurascens|658[0n]|BOLD:AAL5547
Hymenochaonia katyvandusenae|[335]|DHJPAR0057264|Pilocrocis purpurascens|661[0n]|BOLD:AAL5547
Hymenochaonia katyvandusenae|[336]|DHJPAR0036780|Pilocrocis purpurascens|534[1n]|BOLD:AAL5547
Hymenochaonia katyvandusenae|[337]|DHJPAR0038863|Pilocrocis purpurascens|658[0n]|BOLD:AAL5547
Hymenochaonia katyvandusenae|[338]|DHJPAR0057303|Pilocrocis purpurascens|661[0n]|BOLD:AAL5547
Hymenochaonia katyvandusenae|[339]|DHJPAR0042732|Pilocrocis purpurascens|658[0n]|BOLD:AAL5547
Hymenochaonia katyvandusenae|[340]|DHJPAR0052092|Pilocrocis purpurascens|658[0n]|BOLD:AAL5547
Hymenochaonia katyvandusenae|[341]|DHJPAR0040227|Pilocrocis purpurascens|658[0n]|BOLD:AAL5547
Hymenochaonia katyvandusenae|[342]|DHJPAR0052091|Pilocrocis purpurascens|658[0n]|BOLD:AAL5547
Hymenochaonia katyvandusenae|[343]|DHJPAR0037835|Pilocrocis purpurascens|658[0n]|BOLD:AAL5547
Hymenochaonia katyvandusenae|[344]|DHJPAR0041215|Pilocrocis purpurascens|658[0n]|BOLD:AAL5547
Hymenochaonia kazumifukunagae|[345]|DHJPAR0021643|Amorbia 07-SRNP-22617|660[0n]|BOLD:AAX3464
Hymenochaonia keithlangdoni|[346]|DHJPAR0040597|Dichomeris Janzen169|658[0n]|BOLD:AAC3252
[illegible]

Hymenochaonia kazumifukunagae|[345]|DHJPAR0021643|Amorbia 07-SRNP-22617|660[0n]|BOLD:AAX3464
Hymenochaonia keithlangdoni|[346]|DHJPAR0040597|Dichomeris Janzen169|658[0n]|BOLD:AAC3252
Hymenochaonia keithlangdoni|[347]|DHJPAR0040592|Conchylodes salamisalis|658[0n]|BOLD:AAC3252
Hymenochaonia keithlangdoni|[348]|DHJPAR0021647|gelJanzen01 Janzen356|660[0n]|BOLD:AAC3252
Hymenochaonia keithwillmotti|[349]|DHJPAR0040603|gelJanzen01 Janzen461|538[0n]|BOLD:AAP9457
Hymenochaonia keithwillmotti|[350]|BIOUG52676-D05|Malaise trap PL12-6C|652[0n]|BOLD:AAP9457
Hymenochaonia keithwillmotti|[351]|BIOUG54281-C09|Malaise trap PL12-5C|653[0n]|BOLD:AAP9457
Hymenochaonia keithwillmotti|[352]|DHJPAR0048081|gelJanzen01 Janzen407|658[0n]|BOLD:AAP9457
Hymenochaonia keithwillmotti|[353]|BIOUG51353-H03|Malaise trap PL12-8A|654[0n]|BOLD:AAP9457
Hymenochaonia keithwillmotti|[354]|BIOUG52677-F09|Malaise trap PL12-6C|650[0n]|BOLD:AAP9457
Hymenochaonia keithwillmotti|[355]|DHJPAR0042758|Dichomeris Janzen703|566[0n]|BOLD:AAP9457
Hymenochaonia keithwillmotti|[356]|BIOUG51739-B06|Malaise trap PL12-3C|650[0n]|BOLD:AAP9457
Hymenochaonia Janzen10|[357]|DHJPAR0029418|Carthara abruptaDHJ02|277[0n]
Hymenochaonia keithwillmotti|[358]|BIOUG56564-A02|Malaise trap PL12-9D|656[0n]|BOLD:AAP9457
Hymenochaonia keithwillmotti|[359]|DHJPAR0040607|gelJanzen01 Janzen461|658[0n]|BOLD:AAP9457
Hymenochaonia keithwillmotti|[360]|BIOUG51317-E10|Malaise trap PL12-3C|655[0n]|BOLD:AAP9457
Hymenochaonia keithwillmotti|[361]|BIOUG52707-C12|Malaise trap PL12-9C|653[0n]|BOLD:AAP9457
Hymenochaonia keithwillmotti|[362]|BIOUG52708-B01|Malaise trap PL12-9C|653[0n]|BOLD:AAP9457
Hymenochaonia keithwillmotti|[363]|BIOUG55332-C08|Malaise trap PL12-6D|653[0n]|BOLD:AAP9457
Hymenochaonia keithwillmotti|[364]|BIOUG53737-G09|Malaise trap PL12-8C|655[0n]|BOLD:AAP9457
Hymenochaonia keithwillmotti|[365]|BIOUG56550-G03|Malaise trap PL12-7D|653[0n]|BOLD:AAP9457
Hymenochaonia keithwillmotti|[366]|BIOUG51071-E11|Malaise trap PL12-8A|653[0n]|BOLD:AAP9457
Hymenochaonia keithwillmotti|[367]|BIOUG51353-H02|Malaise trap PL12-8A|654[0n]|BOLD:AAP9457
Hymenochaonia keithwillmotti|[368]|BIOUG52675-E03|Malaise trap PL12-6C|653[0n]|BOLD:AAP9457
Hymenochaonia keithwillmotti|[369]|BIOUG52728-H02|Malaise trap PL12-5C|654[0n]|BOLD:AAP9457
Hymenochaonia keithwillmotti|[370]|BIOUG53716-E07|Malaise trap PL12-5C|654[0n]|BOLD:AAP9457
Hymenochaonia keithwillmotti|[371]|DHJPAR0043246|Dichomeris Janzen703|633[0n]|BOLD:AAP9457
Hymenochaonia keithwillmotti|[372]|BIOUG46564-D01|Malaise trap PL12-6B|650[0n]|BOLD:AAP9457
Hymenochaonia keithwillmotti|[373]|BIOUG28849-H10|Malaise trap PL12-7A|612[2n]|BOLD:AAP9457
Hymenochaonia keithwillmotti|[374]|BIOUG29481-G06|Malaise trap PL12-3A|564[5n]|BOLD:AAP9457
Hymenochaonia keithwillmotti|[375]|BIOUG52677-F12|Malaise trap PL12-6C|640[0n]|BOLD:AAP9457
Hymenochaonia keithwillmotti|[376]|BIOUG28824-F08|Malaise trap PL12-9A|631[2n]|BOLD:AAP9457
Hymenochaonia keithwillmotti|[377]|DHJPAR0040606|gelJanzen01 Janzen461|356[0n]|BOLD:AAP9457
Hymenochaonia keithwillmotti|[378]|DHJPAR0040601|gelJanzen01 Janzen461|356[0n]|BOLD:AAP9457
Hymenochaonia keithwillmotti|[379]|DHJPAR0040602|gelJanzen01 Janzen461|357[0n]
Hymenochaonia keithwillmotti|[380]|BIOUG29019-A01|Malaise trap PL12-6A|419[1n]|BOLD:AAP9457
Hymenochaonia keithwillmotti|[381]|DHJPAR0040613|gelJanzen01 Janzen461|658[0n]|BOLD:AAP9457
Hymenochaonia keithwillmotti|[382]|DHJPAR0040570|elachJanzen01 Janzen272|658[0n]|BOLD:AAP9457
Hymenochaonia keithwillmotti|[383]|DHJPAR0048083|gelJanzen01 Janzen407|658[0n]|BOLD:AAP9457
Hymenochaonia keithwillmotti|[384]|DHJPAR0048087|gelJanzen01 Janzen407|658[0n]|BOLD:AAP9457
Hymenochaonia keithwillmotti|[385]|BIOUG52702-E11|Malaise trap PL12-9C|658[0n]|BOLD:AAP9457
Hymenochaonia keithwillmotti|[386]|DHJPAR0048086|gelJanzen01 Janzen407|658[0n]|BOLD:AAP9457
Hymenochaonia keithwillmotti|[387]|BIOUG53726-B08|Malaise trap PL12-5C|657[0n]|BOLD:AAP9457
Hymenochaonia keithwillmotti|[388]|BIOUG53716-E08|Malaise trap PL12-5C|658[0n]|BOLD:AAP9457
Hymenochaonia keithwillmotti|[389]|BIOUG52678-E09|Malaise trap PL12-6C|652[0n]|BOLD:AAP9457
Hymenochaonia keithwillmotti|[390]|BIOUG52677-F11|Malaise trap PL12-6C|651[0n]|BOLD:AAP9457
Hymenochaonia keithwillmotti|[391]|BIOUG51300-F05|Malaise trap PL12-3C|642[0n]|BOLD:AAP9457
Hymenochaonia keithwillmotti|[392]|BIOUG52701-E11|Malaise trap PL12-9C|653[0n]|BOLD:AAP9457
Hymenochaonia keithwillmotti|[393]|BIOUG53696-D04|Malaise trap PL12-5C|655[0n]|BOLD:AAP9457
Hymenochaonia keithwillmotti|[394]|BIOUG52712-H03|Malaise trap PL12-9C|654[0n]|BOLD:AAP9457
Hymenochaonia keithwillmotti|[395]|BIOUG52728-F07|Malaise trap PL12-5C|654[0n]|BOLD:AAP9457
Hymenochaonia keithwillmotti|[396]|BIOUG48416-B06|Malaise trap PL12-5A|652[0n]|BOLD:AAP9457
Hymenochaonia keithwillmotti|[397]|BIOUG52728-F05|Malaise trap PL12-5C|654[0n]|BOLD:AAP9457
Hymenochaonia keithwillmotti|[398]|BIOUG51739-C06|Malaise trap PL12-3C|652[0n]|BOLD:AAP9457
Hymenochaonia keithwillmotti|[399]|BIOUG53696-H08|Malaise trap PL12-5C|659[0n]|BOLD:AAP9457
Hymenochaonia keithwillmotti|[400]|BIOUG51721-C08|Malaise trap PL12-3C|656[0n]|BOLD:AAP9457
Hymenochaonia keithwillmotti|[401]|BIOUG52728-F06|Malaise trap PL12-5C|653[0n]|BOLD:AAP9457
Hymenochaonia keithwillmotti|[402]|BIOUG53744-E05|Malaise trap PL12-8C|729[0n]|BOLD:AAP9457
Hymenochaonia keithwillmotti|[403]|DHJPAR0041403|elachJanzen01 Janzen272|607[0n]|BOLD:AAP9457
Hymenochaonia keithwillmotti|[404]|BIOUG46359-A01|Malaise trap PL12-6B|653[0n]|BOLD:AAP9457
Hymenochaonia keithwillmotti|[405]|BIOUG53696-H09|Malaise trap PL12-5C|655[0n]|BOLD:AAP9457
Hymenochaonia keithwillmotti|[406]|BIOUG44022-F02|Malaise trap PL12-3B|653[0n]|BOLD:AAP9457
Hymenochaonia keithwillmotti|[407]|BIOUG52702-E05|Malaise trap PL12-9C|652[0n]|BOLD:AAP9457
Hymenochaonia keithwillmotti|[408]|BIOUG51309-F07|Malaise trap PL12-8B|653[0n]|BOLD:AAP9457
Hymenochaonia kenjinishidai|[409]|BIOUG06141-G06|BSE Malaise Trap|658[0n]|BOLD:ACG8190
Hymenochaonia kimberleysheldonae|[410]|DHJPAR0037874|Desmia ploralis|658[0n]|BOLD:AAB4677
Hymenochaonia kimberleysheldonae|[411]|DHJPAR0028982|Desmia Janzen09|657[0n]|BOLD:AAB4677
Hymenochaonia kimberleysheldonae|[412]|DHJPAR0056290|Desmia Solis19|670[0n]|BOLD:AAB4677
Hymenochaonia kimberleysheldonae|[413]|DHJPAR0039388|Desmia BioLep06|658[0n]|BOLD:AAB4677
Hymenochaonia kimberleysheldonae|[414]|DHJPAR0055154|Desmia BioLep06|658[0n]|BOLD:AAB4677
Hymenochaonia kimberleysheldonae|[415]|DHJPAR0037872|Desmia ploralis|658[0n]|BOLD:AAB4677
Hymenochaonia kimberleysheldonae|[416]|DHJPAR0050353|Desmia Janzen14|658[0n]|BOLD:AAB4677
Hymenochaonia kimberleysheldonae|[417]|DHJPAR0040233|Desmia BioLep06|658[0n]|BOLD:AAB4677
Hymenochaonia kimberleysheldonae|[418]|DHJPAR0050914|Desmia Janzen14|658[0n]|BOLD:AAB4677
Hymenochaonia kimberleysheldonae|[419]|BIOUG51763-B12|Malaise trap PL12-3C|652[0n]|BOLD:AAB4677
Hymenochaonia kimberleysheldonae|[420]|DHJPAR0036730|Syngamia florella|614[0n]|BOLD:AAB4677
Hymenochaonia kimberleysheldonae|[421]|DHJPAR0036724|Desmia ploralis|631[0n]|BOLD:AAB4677
macroJanzen01 Janzen07|[422]|DHJPAR0029367|pyrJanzen01 Janzen9367|287[0n]
Hymenochaonia kimberleysheldonae|[423]|DHJPAR0055155|Desmia BioLep06|658[0n]|BOLD:AAB4677
Hymenochaonia krisnorvigae|[424]|BIOUG17470-D11|BSE Malaise Trap|588[0n]|BOLD:ACR5126
Hymenochaonia Malaise6869|[425]|DHJPAR0062481|light trap|658[0n]|BOLD:ADH6869
Hymenochaonia Malaise6869|[426]|BIOUG32664-D04|Derrumbe Malaise Trap|532[0n]|BOLD:ADH6869
Hymenochaonia lilianamadrigalae|[427]|DHJPAR0035290|Syllepte belialis|658[0n]|BOLD:AAA7058
Hymenochaonia lilianamadrigalae|[428]|DHJPAR0055920|Syllepte belialis|670[0n]|BOLD:AAA7058
Hymenochaonia lilianamadrigalae|[429]|DHJPAR0029366|Syllepte belialis|657[1n]|BOLD:AAA7058
Hymenochaonia lilianamadrigalae|[430]|DHJPAR0029362|Syllepte belialis|625[0n]|BOLD:AAA7058
Hymenochaonia lilianamadrigalae|[431]|DHJPAR0029363|Syllepte belialis|623[0n]|BOLD:AAA7058
Hymenochaonia lilianamadrigalae|[432]|DHJPAR0035255|Syllepte belialis|658[0n]|BOLD:AAA7058
Hymenochaonia lilianamadrigalae|[433]|DHJPAR0055768|Syllepte belialis|661[0n]|BOLD:AAA7058

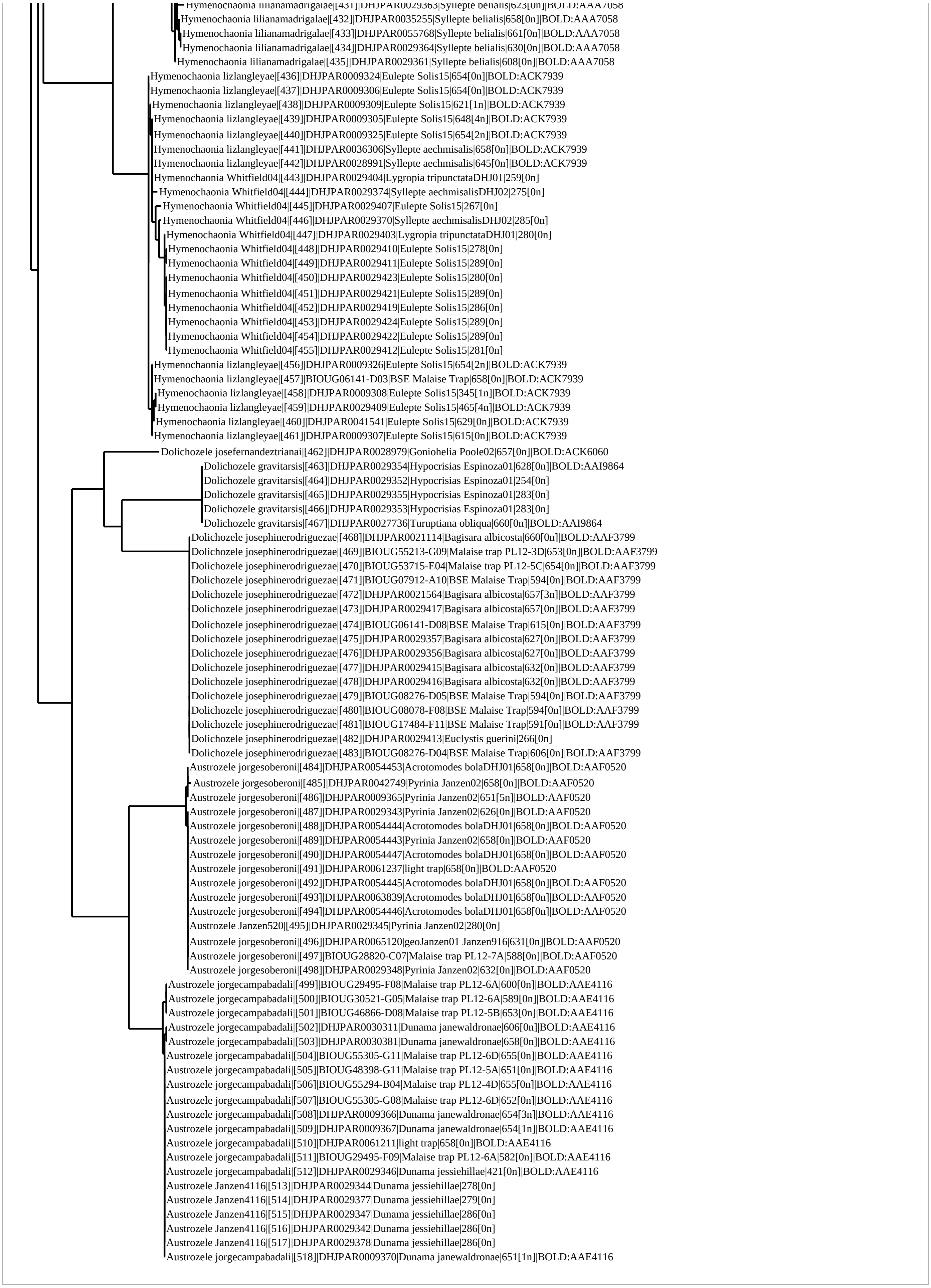
Hymenochaonia lilianamadrigalae|[431]|DHJPAR0029363|Syllepte belialis|623[0n]|BOLD:AAA7058
Hymenochaonia lilianamadrigalae|[432]|DHJPAR0035255|Syllepte belialis|658[0n]|BOLD:AAA7058
Hymenochaonia lilianamadrigalae|[433]|DHJPAR0055768|Syllepte belialis|661[0n]|BOLD:AAA7058
Hymenochaonia lilianamadrigalae|[434]|DHJPAR0029364|Syllepte belialis|630[0n]|BOLD:AAA7058
Hymenochaonia lilianamadrigalae|[435]|DHJPAR0029361|Syllepte belialis|608[0n]|BOLD:AAA7058
Hymenochaonia lizlangleyae|[436]|DHJPAR0009324|Eulepte Solis15|654[0n]|BOLD:ACK7939
Hymenochaonia lizlangleyae|[437]|DHJPAR0009306|Eulepte Solis15|654[0n]|BOLD:ACK7939
Hymenochaonia lizlangleyae|[438]|DHJPAR0009309|Eulepte Solis15|621[1n]|BOLD:ACK7939
Hymenochaonia lizlangleyae|[439]|DHJPAR0009305|Eulepte Solis15|648[4n]|BOLD:ACK7939
Hymenochaonia lizlangleyae|[440]|DHJPAR0009325|Eulepte Solis15|654[2n]|BOLD:ACK7939
Hymenochaonia lizlangleyae|[441]|DHJPAR0036306|Syllepte aechmisalis|658[0n]|BOLD:ACK7939
Hymenochaonia lizlangleyae|[442]|DHJPAR0028991|Syllepte aechmisalis|645[0n]|BOLD:ACK7939
Hymenochaonia Whitfield04|[443]|DHJPAR0029404|Lygropia tripunctataDHJ01|259[0n]
Hymenochaonia Whitfield04|[444]|DHJPAR0029374|Syllepte aechmisalisDHJ02|275[0n]
Hymenochaonia Whitfield04|[445]|DHJPAR0029407|Eulepte Solis15|267[0n]
Hymenochaonia Whitfield04|[446]|DHJPAR0029370|Syllepte aechmisalisDHJ02|285[0n]
Hymenochaonia Whitfield04|[447]|DHJPAR0029403|Lygropia tripunctataDHJ01|280[0n]
Hymenochaonia Whitfield04|[448]|DHJPAR0029410|Eulepte Solis15|278[0n]
Hymenochaonia Whitfield04|[449]|DHJPAR0029411|Eulepte Solis15|289[0n]
Hymenochaonia Whitfield04|[450]|DHJPAR0029423|Eulepte Solis15|280[0n]
Hymenochaonia Whitfield04|[451]|DHJPAR0029421|Eulepte Solis15|289[0n]
Hymenochaonia Whitfield04|[452]|DHJPAR0029419|Eulepte Solis15|286[0n]
Hymenochaonia Whitfield04|[453]|DHJPAR0029424|Eulepte Solis15|289[0n]
Hymenochaonia Whitfield04|[454]|DHJPAR0029422|Eulepte Solis15|289[0n]
Hymenochaonia Whitfield04|[455]|DHJPAR0029412|Eulepte Solis15|281[0n]
Hymenochaonia lizlangleyae|[456]|DHJPAR0009326|Eulepte Solis15|654[2n]|BOLD:ACK7939
Hymenochaonia lizlangleyae|[457]|BIOUG06141-D03|BSE Malaise Trap|658[0n]|BOLD:ACK7939
Hymenochaonia lizlangleyae|[458]|DHJPAR0009308|Eulepte Solis15|345[1n]|BOLD:ACK7939
Hymenochaonia lizlangleyae|[459]|DHJPAR0029409|Eulepte Solis15|465[4n]|BOLD:ACK7939
Hymenochaonia lizlangleyae|[460]|DHJPAR0041541|Eulepte Solis15|629[0n]|BOLD:ACK7939
Hymenochaonia lizlangleyae|[461]|DHJPAR0009307|Eulepte Solis15|615[0n]|BOLD:ACK7939
Dolichozele josefernandeztrianai|[462]|DHJPAR0028979|Goniohelia Poole02|657[0n]|BOLD:ACK6060
Dolichozele gravitarsis|[463]|DHJPAR0029354|Hypocrisias Espinoza01|628[0n]|BOLD:AAI9864
Dolichozele gravitarsis|[464]|DHJPAR0029352|Hypocrisias Espinoza01|254[0n]
Dolichozele gravitarsis|[465]|DHJPAR0029355|Hypocrisias Espinoza01|283[0n]
Dolichozele gravitarsis|[466]|DHJPAR0029353|Hypocrisias Espinoza01|283[0n]
Dolichozele gravitarsis|[467]|DHJPAR0027736|Turuptiana obliqua|660[0n]|BOLD:AAI9864
Dolichozele josephinerodriguezae|[468]|DHJPAR0021114|Bagisara albicosta|660[0n]|BOLD:AAF3799
Dolichozele josephinerodriguezae|[469]|BIOUG55213-G09|Malaise trap PL12-3D|653[0n]|BOLD:AAF3799
Dolichozele josephinerodriguezae|[470]|BIOUG53715-E04|Malaise trap PL12-5C|654[0n]|BOLD:AAF3799
Dolichozele josephinerodriguezae|[471]|BIOUG07912-A10|BSE Malaise Trap|594[0n]|BOLD:AAF3799
Dolichozele josephinerodriguezae|[472]|DHJPAR0021564|Bagisara albicosta|657[3n]|BOLD:AAF3799
Dolichozele josephinerodriguezae|[473]|DHJPAR0029417|Bagisara albicosta|657[0n]|BOLD:AAF3799
Dolichozele josephinerodriguezae|[474]|BIOUG06141-D08|BSE Malaise Trap|615[0n]|BOLD:AAF3799
Dolichozele josephinerodriguezae|[475]|DHJPAR0029357|Bagisara albicosta|627[0n]|BOLD:AAF3799
Dolichozele josephinerodriguezae|[476]|DHJPAR0029356|Bagisara albicosta|627[0n]|BOLD:AAF3799
Dolichozele josephinerodriguezae|[477]|DHJPAR0029415|Bagisara albicosta|632[0n]|BOLD:AAF3799
Dolichozele josephinerodriguezae|[478]|DHJPAR0029416|Bagisara albicosta|632[0n]|BOLD:AAF3799
Dolichozele josephinerodriguezae|[479]|BIOUG08276-D05|BSE Malaise Trap|594[0n]|BOLD:AAF3799
Dolichozele josephinerodriguezae|[480]|BIOUG08078-F08|BSE Malaise Trap|594[0n]|BOLD:AAF3799
Dolichozele josephinerodriguezae|[481]|BIOUG17484-F11|BSE Malaise Trap|591[0n]|BOLD:AAF3799
Dolichozele josephinerodriguezae|[482]|DHJPAR0029413|Euclystis guerini|266[0n]
Dolichozele josephinerodriguezae|[483]|BIOUG08276-D04|BSE Malaise Trap|606[0n]|BOLD:AAF3799
Austrozele jorgesoberoni|[484]|DHJPAR0054453|Acrotomodes bolaDHJ01|658[0n]|BOLD:AAF0520
Austrozele jorgesoberoni|[485]|DHJPAR0042749|Pyrinia Janzen02|658[0n]|BOLD:AAF0520
Austrozele jorgesoberoni|[486]|DHJPAR0009365|Pyrinia Janzen02|651[5n]|BOLD:AAF0520
Austrozele jorgesoberoni|[487]|DHJPAR0029343|Pyrinia Janzen02|626[0n]|BOLD:AAF0520
Austrozele jorgesoberoni|[488]|DHJPAR0054444|Acrotomodes bolaDHJ01|658[0n]|BOLD:AAF0520
Austrozele jorgesoberoni|[489]|DHJPAR0054443|Pyrinia Janzen02|658[0n]|BOLD:AAF0520
Austrozele jorgesoberoni|[490]|DHJPAR0054447|Acrotomodes bolaDHJ01|658[0n]|BOLD:AAF0520
Austrozele jorgesoberoni|[491]|DHJPAR0061237|light trap|658[0n]|BOLD:AAF0520
Austrozele jorgesoberoni|[492]|DHJPAR0054445|Acrotomodes bolaDHJ01|658[0n]|BOLD:AAF0520
Austrozele jorgesoberoni|[493]|DHJPAR0063839|Acrotomodes bolaDHJ01|658[0n]|BOLD:AAF0520
Austrozele jorgesoberoni|[494]|DHJPAR0054446|Acrotomodes bolaDHJ01|658[0n]|BOLD:AAF0520
Austrozele Janzen520|[495]|DHJPAR0029345|Pyrinia Janzen02|280[0n]
Austrozele jorgesoberoni|[496]|DHJPAR0065120|geoJanzen01 Janzen916|631[0n]|BOLD:AAF0520
Austrozele jorgesoberoni|[497]|BIOUG28820-C07|Malaise trap PL12-7A|588[0n]|BOLD:AAF0520
Austrozele jorgesoberoni|[498]|DHJPAR0029348|Pyrinia Janzen02|632[0n]|BOLD:AAF0520
Austrozele jorgecampabadali|[499]|BIOUG29495-F08|Malaise trap PL12-6A|600[0n]|BOLD:AAE4116
Austrozele jorgecampabadali|[500]|BIOUG30521-G05|Malaise trap PL12-6A|589[0n]|BOLD:AAE4116
Austrozele jorgecampabadali|[501]|BIOUG46866-D08|Malaise trap PL12-5B|653[0n]|BOLD:AAE4116
Austrozele jorgecampabadali|[502]|DHJPAR0030311|Dunama janewaldronae|606[0n]|BOLD:AAE4116
Austrozele jorgecampabadali|[503]|DHJPAR0030381|Dunama janewaldronae|658[0n]|BOLD:AAE4116
Austrozele jorgecampabadali|[504]|BIOUG55305-G11|Malaise trap PL12-6D|655[0n]|BOLD:AAE4116
Austrozele jorgecampabadali|[505]|BIOUG48398-G11|Malaise trap PL12-5A|651[0n]|BOLD:AAE4116
Austrozele jorgecampabadali|[506]|BIOUG55294-B04|Malaise trap PL12-4D|655[0n]|BOLD:AAE4116
Austrozele jorgecampabadali|[507]|BIOUG55305-G08|Malaise trap PL12-6D|652[0n]|BOLD:AAE4116
Austrozele jorgecampabadali|[508]|DHJPAR0009366|Dunama janewaldronae|654[3n]|BOLD:AAE4116
Austrozele jorgecampabadali|[509]|DHJPAR0009367|Dunama janewaldronae|654[1n]|BOLD:AAE4116
Austrozele jorgecampabadali|[510]|DHJPAR0061211|light trap|658[0n]|BOLD:AAE4116
Austrozele jorgecampabadali|[511]|BIOUG29495-F09|Malaise trap PL12-6A|582[0n]|BOLD:AAE4116
Austrozele jorgecampabadali|[512]|DHJPAR0029346|Dunama jessiehillae|421[0n]|BOLD:AAE4116
Austrozele Janzen4116|[513]|DHJPAR0029344|Dunama jessiehillae|278[0n]
Austrozele Janzen4116|[514]|DHJPAR0029377|Dunama jessiehillae|279[0n]
Austrozele Janzen4116|[515]|DHJPAR0029347|Dunama jessiehillae|286[0n]
Austrozele Janzen4116|[516]|DHJPAR0029342|Dunama jessiehillae|286[0n]
Austrozele Janzen4116|[517]|DHJPAR0029378|Dunama jessiehillae|286[0n]
Austrozele jorgecampabadali|[518]|DHJPAR0009370|Dunama janewaldronae|651[1n]|BOLD:AAE4116
