# Supplementary material for: Minimalist revision and description of 403 new species in 11 subfamilies of Costa Rican braconid parasitoid wasps, including host records for 219 species
Source: Zookeys. 2021 Feb 2;1013:1–665. doi: 10.3897/zookeys.1013.55600 (PMC8390796; doi:10.3897/zookeys.1013.55600)
Supplement: Supplementary material 8 — Orgilinae [file zookeys-1013-001-s008.pdf]

8. Orgilinae BOLD TaxonID Tree

Title : Tree Result - Search: Sample IDs (228 records returned) (228 records selected)
Date : 17-Nov-2020
Data Type : Nucleotide
Distance Model : Kimura 2 Parameter
Marker : COI-5P
Colourization : [blue]=Stop Codons [red]=Contamination or misidentification

Label : Sample ID
Label : Taxon
Label : Extra Info
Label : Sequence Length
Label : Barcode Cluster (BIN)

Filter : exclude records with stop codons

Sequence Count : 221
Species count : 47
Genus count : 2
Family count : 1
Unidentified : 1

BIN Count : 47

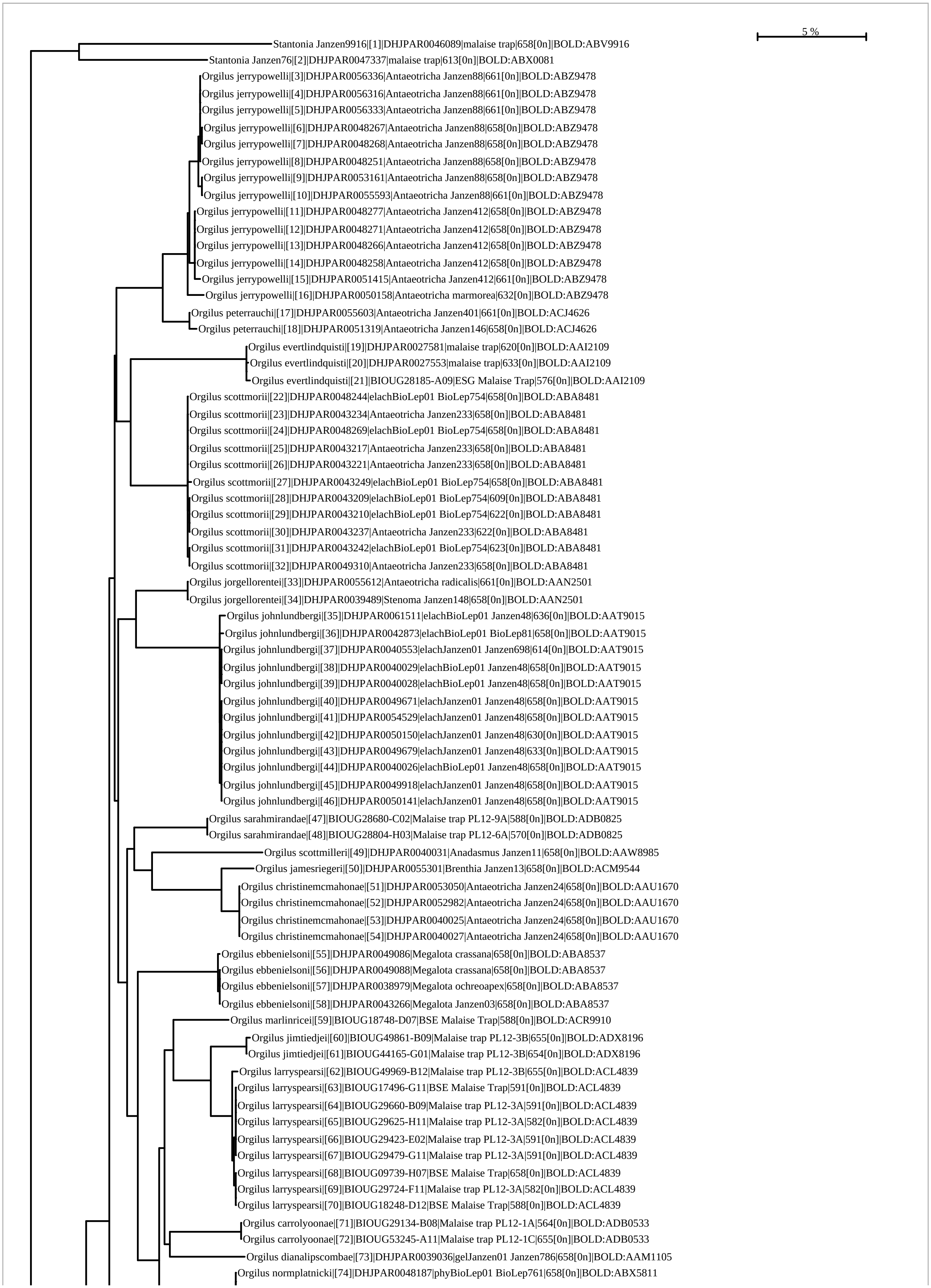
5 %
Stantonia Janzen9916|[1]|DHJPAR0046089|malaise trap|658[0n]|BOLD:ABV9916
Stantonia Janzen76|[2]|DHJPAR0047337|malaise trap|613[0n]|BOLD:ABX0081
Orgilus jerrypowelli|[3]|DHJPAR0056336|Antaeotricha Janzen88|661[0n]|BOLD:ABZ9478
Orgilus jerrypowelli|[4]|DHJPAR0056316|Antaeotricha Janzen88|661[0n]|BOLD:ABZ9478
Orgilus jerrypowelli|[5]|DHJPAR0056333|Antaeotricha Janzen88|661[0n]|BOLD:ABZ9478
Orgilus jerrypowelli|[6]|DHJPAR0048267|Antaeotricha Janzen88|658[0n]|BOLD:ABZ9478
Orgilus jerrypowelli|[7]|DHJPAR0048268|Antaeotricha Janzen88|658[0n]|BOLD:ABZ9478
Orgilus jerrypowelli|[8]|DHJPAR0048251|Antaeotricha Janzen88|658[0n]|BOLD:ABZ9478
Orgilus jerrypowelli|[9]|DHJPAR0053161|Antaeotricha Janzen88|658[0n]|BOLD:ABZ9478
Orgilus jerrypowelli|[10]|DHJPAR0055593|Antaeotricha Janzen88|661[0n]|BOLD:ABZ9478
Orgilus jerrypowelli|[11]|DHJPAR0048277|Antaeotricha Janzen412|658[0n]|BOLD:ABZ9478
Orgilus jerrypowelli|[12]|DHJPAR0048271|Antaeotricha Janzen412|658[0n]|BOLD:ABZ9478
Orgilus jerrypowelli|[13]|DHJPAR0048266|Antaeotricha Janzen412|658[0n]|BOLD:ABZ9478
Orgilus jerrypowelli|[14]|DHJPAR0048258|Antaeotricha Janzen412|658[0n]|BOLD:ABZ9478
Orgilus jerrypowelli|[15]|DHJPAR0051415|Antaeotricha Janzen412|661[0n]|BOLD:ABZ9478
Orgilus jerrypowelli|[16]|DHJPAR0050158|Antaeotricha marmorea|632[0n]|BOLD:ABZ9478
Orgilus peterrauchi|[17]|DHJPAR0055603|Antaeotricha Janzen401|661[0n]|BOLD:ACJ4626
Orgilus peterrauchi|[18]|DHJPAR0051319|Antaeotricha Janzen146|658[0n]|BOLD:ACJ4626
Orgilus evertlindquisti|[19]|DHJPAR0027581|malaise trap|620[0n]|BOLD:AAI2109
Orgilus evertlindquisti|[20]|DHJPAR0027553|malaise trap|633[0n]|BOLD:AAI2109
Orgilus evertlindquisti|[21]|BIOUG28185-A09|ESG Malaise Trap|576[0n]|BOLD:AAI2109
Orgilus scottmorii|[22]|DHJPAR0048244|elachBioLep01 BioLep754|658[0n]|BOLD:ABA8481
Orgilus scottmorii|[23]|DHJPAR0043234|Antaeotricha Janzen233|658[0n]|BOLD:ABA8481
Orgilus scottmorii|[24]|DHJPAR0048269|elachBioLep01 BioLep754|658[0n]|BOLD:ABA8481
Orgilus scottmorii|[25]|DHJPAR0043217|Antaeotricha Janzen233|658[0n]|BOLD:ABA8481
Orgilus scottmorii|[26]|DHJPAR0043221|Antaeotricha Janzen233|658[0n]|BOLD:ABA8481
Orgilus scottmorii|[27]|DHJPAR0043249|elachBioLep01 BioLep754|658[0n]|BOLD:ABA8481
Orgilus scottmorii|[28]|DHJPAR0043209|elachBioLep01 BioLep754|609[0n]|BOLD:ABA8481
Orgilus scottmorii|[29]|DHJPAR0043210|elachBioLep01 BioLep754|622[0n]|BOLD:ABA8481
Orgilus scottmorii|[30]|DHJPAR0043237|Antaeotricha Janzen233|622[0n]|BOLD:ABA8481
Orgilus scottmorii|[31]|DHJPAR0043242|elachBioLep01 BioLep754|623[0n]|BOLD:ABA8481
Orgilus scottmorii|[32]|DHJPAR0049310|Antaeotricha Janzen233|658[0n]|BOLD:ABA8481
Orgilus jorgellorentei|[33]|DHJPAR0055612|Antaeotricha radicalis|661[0n]|BOLD:AAN2501
Orgilus jorgellorentei|[34]|DHJPAR0039489|Stenoma Janzen148|658[0n]|BOLD:AAN2501
Orgilus johnlundbergi|[35]|DHJPAR0061511|elachBioLep01 Janzen48|636[0n]|BOLD:AAT9015
Orgilus johnlundbergi|[36]|DHJPAR0042873|elachBioLep01 BioLep81|658[0n]|BOLD:AAT9015
Orgilus johnlundbergi|[37]|DHJPAR0040553|elachJanzen01 Janzen698|614[0n]|BOLD:AAT9015
Orgilus johnlundbergi|[38]|DHJPAR0040029|elachBioLep01 Janzen48|658[0n]|BOLD:AAT9015
Orgilus johnlundbergi|[39]|DHJPAR0040028|elachBioLep01 Janzen48|658[0n]|BOLD:AAT9015
Orgilus johnlundbergi|[40]|DHJPAR0049671|elachJanzen01 Janzen48|658[0n]|BOLD:AAT9015
Orgilus johnlundbergi|[41]|DHJPAR0054529|elachJanzen01 Janzen48|658[0n]|BOLD:AAT9015
Orgilus johnlundbergi|[42]|DHJPAR0050150|elachJanzen01 Janzen48|630[0n]|BOLD:AAT9015
Orgilus johnlundbergi|[43]|DHJPAR0049679|elachJanzen01 Janzen48|633[0n]|BOLD:AAT9015
Orgilus johnlundbergi|[44]|DHJPAR0040026|elachBioLep01 Janzen48|658[0n]|BOLD:AAT9015
Orgilus johnlundbergi|[45]|DHJPAR0049918|elachJanzen01 Janzen48|658[0n]|BOLD:AAT9015
Orgilus johnlundbergi|[46]|DHJPAR0050141|elachJanzen01 Janzen48|658[0n]|BOLD:AAT9015
Orgilus sarahmirandae|[47]|BIOUG28680-C02|Malaise trap PL12-9A|588[0n]|BOLD:ADB0825
Orgilus sarahmirandae|[48]|BIOUG28804-H03|Malaise trap PL12-6A|570[0n]|BOLD:ADB0825
Orgilus scottmilleri|[49]|DHJPAR0040031|Anadasmus Janzen11|658[0n]|BOLD:AAW8985
Orgilus jamesriegeri|[50]|DHJPAR0055301|Brenthia Janzen13|658[0n]|BOLD:ACM9544
Orgilus christinemcmahonae|[51]|DHJPAR0053050|Antaeotricha Janzen24|658[0n]|BOLD:AAU1670
Orgilus christinemcmahonae|[52]|DHJPAR0052982|Antaeotricha Janzen24|658[0n]|BOLD:AAU1670
Orgilus christinemcmahonae|[53]|DHJPAR0040025|Antaeotricha Janzen24|658[0n]|BOLD:AAU1670
Orgilus christinemcmahonae|[54]|DHJPAR0040027|Antaeotricha Janzen24|658[0n]|BOLD:AAU1670
Orgilus ebbenielsoni|[55]|DHJPAR0049086|Megalota crassana|658[0n]|BOLD:ABA8537
Orgilus ebbenielsoni|[56]|DHJPAR0049088|Megalota crassana|658[0n]|BOLD:ABA8537
Orgilus ebbenielsoni|[57]|DHJPAR0038979|Megalota ochreoapex|658[0n]|BOLD:ABA8537
Orgilus ebbenielsoni|[58]|DHJPAR0043266|Megalota Janzen03|658[0n]|BOLD:ABA8537
Orgilus marlinricei|[59]|BIOUG18748-D07|BSE Malaise Trap|588[0n]|BOLD:ACR9910
Orgilus jimtiedjei|[60]|BIOUG49861-B09|Malaise trap PL12-3B|655[0n]|BOLD:ADX8196
Orgilus jimtiedjei|[61]|BIOUG44165-G01|Malaise trap PL12-3B|654[0n]|BOLD:ADX8196
Orgilus larryspearsi|[62]|BIOUG49969-B12|Malaise trap PL12-3B|655[0n]|BOLD:ACL4839
Orgilus larryspearsi|[63]|BIOUG17496-G11|BSE Malaise Trap|591[0n]|BOLD:ACL4839
Orgilus larryspearsi|[64]|BIOUG29660-B09|Malaise trap PL12-3A|591[0n]|BOLD:ACL4839
Orgilus larryspearsi|[65]|BIOUG29625-H11|Malaise trap PL12-3A|582[0n]|BOLD:ACL4839
Orgilus larryspearsi|[66]|BIOUG29423-E02|Malaise trap PL12-3A|591[0n]|BOLD:ACL4839
Orgilus larryspearsi|[67]|BIOUG29479-G11|Malaise trap PL12-3A|591[0n]|BOLD:ACL4839
Orgilus larryspearsi|[68]|BIOUG09739-H07|BSE Malaise Trap|658[0n]|BOLD:ACL4839
Orgilus larryspearsi|[69]|BIOUG29724-F11|Malaise trap PL12-3A|582[0n]|BOLD:ACL4839
Orgilus larryspearsi|[70]|BIOUG18248-D12|BSE Malaise Trap|588[0n]|BOLD:ACL4839
Orgilus carrolyoonae|[71]|BIOUG29134-B08|Malaise trap PL12-1A|564[0n]|BOLD:ADB0533
Orgilus carrolyoonae|[72]|BIOUG53245-A11|Malaise trap PL12-1C|655[0n]|BOLD:ADB0533
Orgilus dianalipscombae|[73]|DHJPAR0039036|gelJanzen01 Janzen786|658[0n]|BOLD:AAM1105
Orgilus normplatnicki|[74]|DHJPAR0048187|phyBioLep01 BioLep761|658[0n]|BOLD:ABX5811

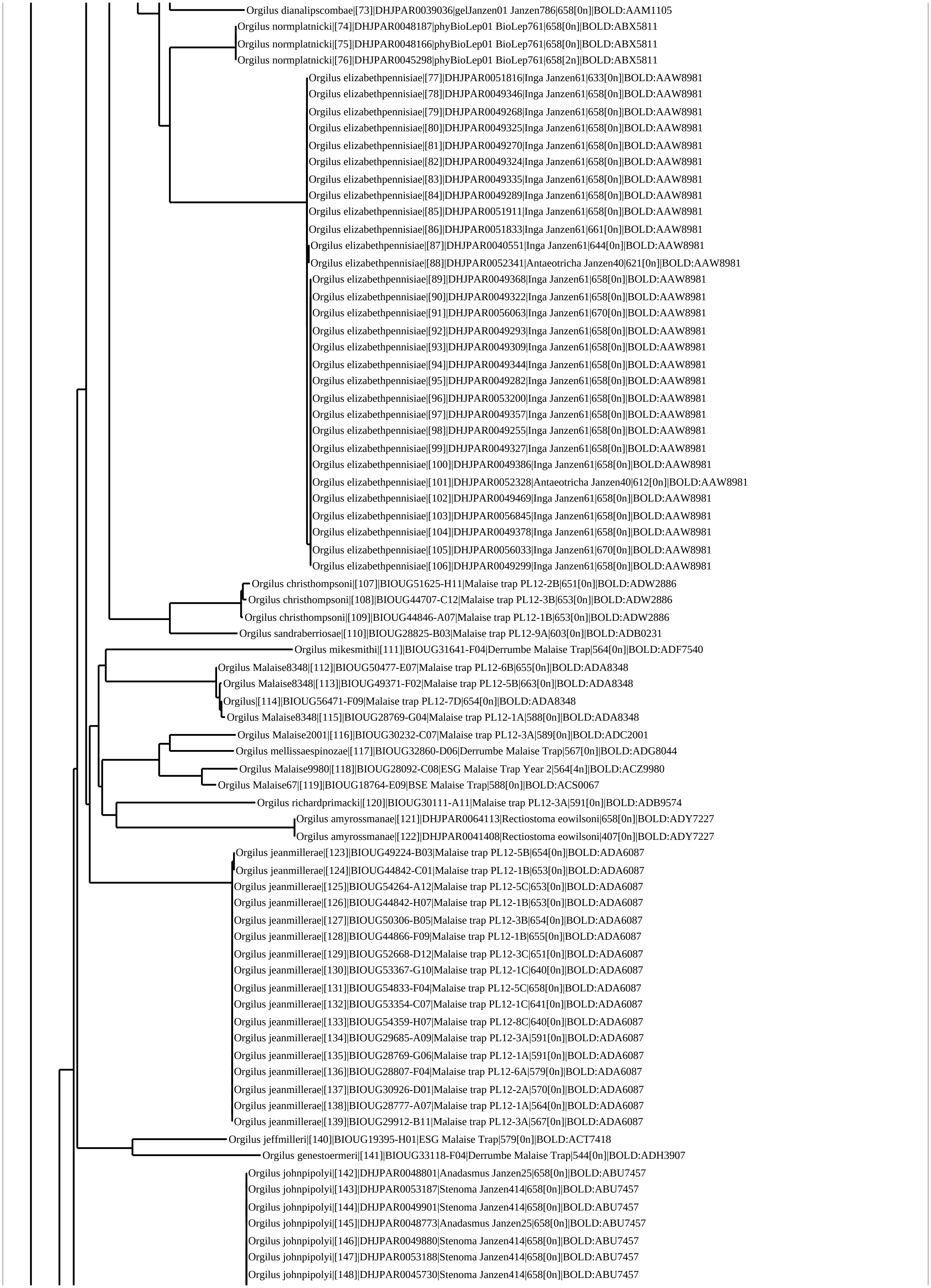
Orgilus dianalipscombae|[73]|DHJPAR0039036|gelJanzen01 Janzen786|658[0n]|BOLD:AAM1105
Orgilus normplatnicki|[74]|DHJPAR0048187|phyBioLep01 BioLep761|658[0n]|BOLD:ABX5811
Orgilus normplatnicki|[75]|DHJPAR0048166|phyBioLep01 BioLep761|658[0n]|BOLD:ABX5811
Orgilus normplatnicki|[76]|DHJPAR0045298|phyBioLep01 BioLep761|658[2n]|BOLD:ABX5811
Orgilus elizabethpennisiae|[77]|DHJPAR0051816|Inga Janzen61|633[0n]|BOLD:AAW8981
Orgilus elizabethpennisiae|[78]|DHJPAR0049346|Inga Janzen61|658[0n]|BOLD:AAW8981
Orgilus elizabethpennisiae|[79]|DHJPAR0049268|Inga Janzen61|658[0n]|BOLD:AAW8981
Orgilus elizabethpennisiae|[80]|DHJPAR0049325|Inga Janzen61|658[0n]|BOLD:AAW8981
Orgilus elizabethpennisiae|[81]|DHJPAR0049270|Inga Janzen61|658[0n]|BOLD:AAW8981
Orgilus elizabethpennisiae|[82]|DHJPAR0049324|Inga Janzen61|658[0n]|BOLD:AAW8981
Orgilus elizabethpennisiae|[83]|DHJPAR0049335|Inga Janzen61|658[0n]|BOLD:AAW8981
Orgilus elizabethpennisiae|[84]|DHJPAR0049289|Inga Janzen61|658[0n]|BOLD:AAW8981
Orgilus elizabethpennisiae|[85]|DHJPAR0051911|Inga Janzen61|658[0n]|BOLD:AAW8981
Orgilus elizabethpennisiae|[86]|DHJPAR0051833|Inga Janzen61|661[0n]|BOLD:AAW8981
Orgilus elizabethpennisiae|[87]|DHJPAR0040551|Inga Janzen61|644[0n]|BOLD:AAW8981
Orgilus elizabethpennisiae|[88]|DHJPAR0052341|Antaeotricha Janzen40|621[0n]|BOLD:AAW8981
Orgilus elizabethpennisiae|[89]|DHJPAR0049368|Inga Janzen61|658[0n]|BOLD:AAW8981
Orgilus elizabethpennisiae|[90]|DHJPAR0049322|Inga Janzen61|658[0n]|BOLD:AAW8981
Orgilus elizabethpennisiae|[91]|DHJPAR0056063|Inga Janzen61|670[0n]|BOLD:AAW8981
Orgilus elizabethpennisiae|[92]|DHJPAR0049293|Inga Janzen61|658[0n]|BOLD:AAW8981
Orgilus elizabethpennisiae|[93]|DHJPAR0049309|Inga Janzen61|658[0n]|BOLD:AAW8981
Orgilus elizabethpennisiae|[94]|DHJPAR0049344|Inga Janzen61|658[0n]|BOLD:AAW8981
Orgilus elizabethpennisiae|[95]|DHJPAR0049282|Inga Janzen61|658[0n]|BOLD:AAW8981
Orgilus elizabethpennisiae|[96]|DHJPAR0053200|Inga Janzen61|658[0n]|BOLD:AAW8981
Orgilus elizabethpennisiae|[97]|DHJPAR0049357|Inga Janzen61|658[0n]|BOLD:AAW8981
Orgilus elizabethpennisiae|[98]|DHJPAR0049255|Inga Janzen61|658[0n]|BOLD:AAW8981
Orgilus elizabethpennisiae|[99]|DHJPAR0049327|Inga Janzen61|658[0n]|BOLD:AAW8981
Orgilus elizabethpennisiae|[100]|DHJPAR0049386|Inga Janzen61|658[0n]|BOLD:AAW8981
Orgilus elizabethpennisiae|[101]|DHJPAR0052328|Antaeotricha Janzen40|612[0n]|BOLD:AAW8981
Orgilus elizabethpennisiae|[102]|DHJPAR0049469|Inga Janzen61|658[0n]|BOLD:AAW8981
Orgilus elizabethpennisiae|[103]|DHJPAR0056845|Inga Janzen61|658[0n]|BOLD:AAW8981
Orgilus elizabethpennisiae|[104]|DHJPAR0049378|Inga Janzen61|658[0n]|BOLD:AAW8981
Orgilus elizabethpennisiae|[105]|DHJPAR0056033|Inga Janzen61|670[0n]|BOLD:AAW8981
Orgilus elizabethpennisiae|[106]|DHJPAR0049299|Inga Janzen61|658[0n]|BOLD:AAW8981
Orgilus christhompsoni|[107]|BIOUG51625-H11|Malaise trap PL12-2B|651[0n]|BOLD:ADW2886
Orgilus christhompsoni|[108]|BIOUG44707-C12|Malaise trap PL12-3B|653[0n]|BOLD:ADW2886
Orgilus christhompsoni|[109]|BIOUG44846-A07|Malaise trap PL12-1B|653[0n]|BOLD:ADW2886
Orgilus sandraberriosae|[110]|BIOUG28825-B03|Malaise trap PL12-9A|603[0n]|BOLD:ADB0231
Orgilus mikesmithi|[111]|BIOUG31641-F04|Derrumbe Malaise Trap|564[0n]|BOLD:ADF7540
Orgilus Malaise8348|[112]|BIOUG50477-E07|Malaise trap PL12-6B|655[0n]|BOLD:ADA8348
Orgilus Malaise8348|[113]|BIOUG49371-F02|Malaise trap PL12-5B|663[0n]|BOLD:ADA8348
Orgilus|[114]|BIOUG56471-F09|Malaise trap PL12-7D|654[0n]|BOLD:ADA8348
Orgilus Malaise8348|[115]|BIOUG28769-G04|Malaise trap PL12-1A|588[0n]|BOLD:ADA8348
Orgilus Malaise2001|[116]|BIOUG30232-C07|Malaise trap PL12-3A|589[0n]|BOLD:ADC2001
Orgilus mellissaespinozae|[117]|BIOUG32860-D06|Derrumbe Malaise Trap|567[0n]|BOLD:ADG8044
Orgilus Malaise9980|[118]|BIOUG28092-C08|ESG Malaise Trap Year 2|564[4n]|BOLD:ACZ9980
Orgilus Malaise67|[119]|BIOUG18764-E09|BSE Malaise Trap|588[0n]|BOLD:ACS0067
Orgilus richardprimacki|[120]|BIOUG30111-A11|Malaise trap PL12-3A|591[0n]|BOLD:ADB9574
Orgilus amyrossmanae|[121]|DHJPAR0064113|Rectiostoma eowilsoni|658[0n]|BOLD:ADY7227
Orgilus amyrossmanae|[122]|DHJPAR0041408|Rectiostoma eowilsoni|407[0n]|BOLD:ADY7227
Orgilus jeanmillerae|[123]|BIOUG49224-B03|Malaise trap PL12-5B|654[0n]|BOLD:ADA6087
Orgilus jeanmillerae|[124]|BIOUG44842-C01|Malaise trap PL12-1B|653[0n]|BOLD:ADA6087
Orgilus jeanmillerae|[125]|BIOUG54264-A12|Malaise trap PL12-5C|653[0n]|BOLD:ADA6087
Orgilus jeanmillerae|[126]|BIOUG44842-H07|Malaise trap PL12-1B|653[0n]|BOLD:ADA6087
Orgilus jeanmillerae|[127]|BIOUG50306-B05|Malaise trap PL12-3B|654[0n]|BOLD:ADA6087
Orgilus jeanmillerae|[128]|BIOUG44866-F09|Malaise trap PL12-1B|655[0n]|BOLD:ADA6087
Orgilus jeanmillerae|[129]|BIOUG52668-D12|Malaise trap PL12-3C|651[0n]|BOLD:ADA6087
Orgilus jeanmillerae|[130]|BIOUG53367-G10|Malaise trap PL12-1C|640[0n]|BOLD:ADA6087
Orgilus jeanmillerae|[131]|BIOUG54833-F04|Malaise trap PL12-5C|658[0n]|BOLD:ADA6087
Orgilus jeanmillerae|[132]|BIOUG53354-C07|Malaise trap PL12-1C|641[0n]|BOLD:ADA6087
Orgilus jeanmillerae|[133]|BIOUG54359-H07|Malaise trap PL12-8C|640[0n]|BOLD:ADA6087
Orgilus jeanmillerae|[134]|BIOUG29685-A09|Malaise trap PL12-3A|591[0n]|BOLD:ADA6087
Orgilus jeanmillerae|[135]|BIOUG28769-G06|Malaise trap PL12-1A|591[0n]|BOLD:ADA6087
Orgilus jeanmillerae|[136]|BIOUG28807-F04|Malaise trap PL12-6A|579[0n]|BOLD:ADA6087
Orgilus jeanmillerae|[137]|BIOUG30926-D01|Malaise trap PL12-2A|570[0n]|BOLD:ADA6087
Orgilus jeanmillerae|[138]|BIOUG28777-A07|Malaise trap PL12-1A|564[0n]|BOLD:ADA6087
Orgilus jeanmillerae|[139]|BIOUG29912-B11|Malaise trap PL12-3A|567[0n]|BOLD:ADA6087
Orgilus jeffmilleri|[140]|BIOUG19395-H01|ESG Malaise Trap|579[0n]|BOLD:ACT7418
Orgilus genestoermeri|[141]|BIOUG33118-F04|Derrumbe Malaise Trap|544[0n]|BOLD:ADH3907
Orgilus johnpipolyi|[142]|DHJPAR0048801|Anadasmus Janzen25|658[0n]|BOLD:ABU7457
Orgilus johnpipolyi|[143]|DHJPAR0053187|Stenoma Janzen414|658[0n]|BOLD:ABU7457
Orgilus johnpipolyi|[144]|DHJPAR0049901|Stenoma Janzen414|658[0n]|BOLD:ABU7457
Orgilus johnpipolyi|[145]|DHJPAR0048773|Anadasmus Janzen25|658[0n]|BOLD:ABU7457
Orgilus johnpipolyi|[146]|DHJPAR0049880|Stenoma Janzen414|658[0n]|BOLD:ABU7457
Orgilus johnpipolyi|[147]|DHJPAR0053188|Stenoma Janzen414|658[0n]|BOLD:ABU7457
Orgilus johnpipolyi|[148]|DHJPAR0045730|Stenoma Janzen414|658[0n]|BOLD:ABU7457

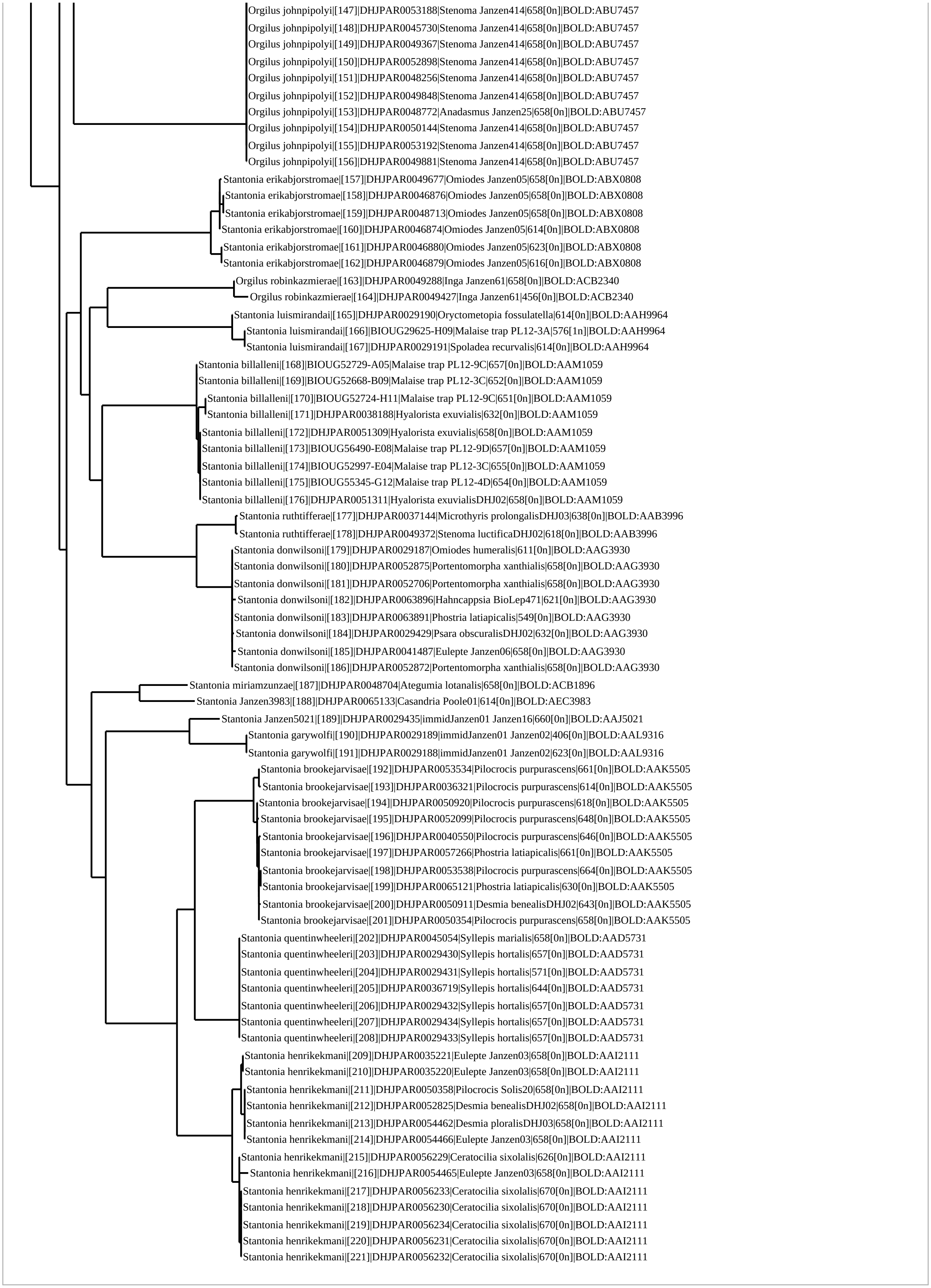
Orgilus johnpipolyi|[147]|DHJPAR0053188|Stenoma Janzen414|658[0n]|BOLD:ABU7457
Orgilus johnpipolyi|[148]|DHJPAR0045730|Stenoma Janzen414|658[0n]|BOLD:ABU7457
Orgilus johnpipolyi|[149]|DHJPAR0049367|Stenoma Janzen414|658[0n]|BOLD:ABU7457
Orgilus johnpipolyi|[150]|DHJPAR0052898|Stenoma Janzen414|658[0n]|BOLD:ABU7457
Orgilus johnpipolyi|[151]|DHJPAR0048256|Stenoma Janzen414|658[0n]|BOLD:ABU7457
Orgilus johnpipolyi|[152]|DHJPAR0049848|Stenoma Janzen414|658[0n]|BOLD:ABU7457
Orgilus johnpipolyi|[153]|DHJPAR0048772|Anadasmus Janzen25|658[0n]|BOLD:ABU7457
Orgilus johnpipolyi|[154]|DHJPAR0050144|Stenoma Janzen414|658[0n]|BOLD:ABU7457
Orgilus johnpipolyi|[155]|DHJPAR0053192|Stenoma Janzen414|658[0n]|BOLD:ABU7457
Orgilus johnpipolyi|[156]|DHJPAR0049881|Stenoma Janzen414|658[0n]|BOLD:ABU7457
Stantonia erikabjorstromae|[157]|DHJPAR0049677|Omiodes Janzen05|658[0n]|BOLD:ABX0808
Stantonia erikabjorstromae|[158]|DHJPAR0046876|Omiodes Janzen05|658[0n]|BOLD:ABX0808
Stantonia erikabjorstromae|[159]|DHJPAR0048713|Omiodes Janzen05|658[0n]|BOLD:ABX0808
Stantonia erikabjorstromae|[160]|DHJPAR0046874|Omiodes Janzen05|614[0n]|BOLD:ABX0808
Stantonia erikabjorstromae|[161]|DHJPAR0046880|Omiodes Janzen05|623[0n]|BOLD:ABX0808
Stantonia erikabjorstromae|[162]|DHJPAR0046879|Omiodes Janzen05|616[0n]|BOLD:ABX0808
Orgilus robinkazmierae|[163]|DHJPAR0049288|Inga Janzen61|658[0n]|BOLD:ACB2340
Orgilus robinkazmierae|[164]|DHJPAR0049427|Inga Janzen61|456[0n]|BOLD:ACB2340
Stantonia luismirandai|[165]|DHJPAR0029190|Oryctometopia fossulatella|614[0n]|BOLD:AAH9964
Stantonia luismirandai|[166]|BIOUG29625-H09|Malaise trap PL12-3A|576[1n]|BOLD:AAH9964
Stantonia luismirandai|[167]|DHJPAR0029191|Spoladea recurvalis|614[0n]|BOLD:AAH9964
Stantonia billalleni|[168]|BIOUG52729-A05|Malaise trap PL12-9C|657[0n]|BOLD:AAM1059
Stantonia billalleni|[169]|BIOUG52668-B09|Malaise trap PL12-3C|652[0n]|BOLD:AAM1059
Stantonia billalleni|[170]|BIOUG52724-H11|Malaise trap PL12-9C|651[0n]|BOLD:AAM1059
Stantonia billalleni|[171]|DHJPAR0038188|Hyalorista exuvialis|632[0n]|BOLD:AAM1059
Stantonia billalleni|[172]|DHJPAR0051309|Hyalorista exuvialis|658[0n]|BOLD:AAM1059
Stantonia billalleni|[173]|BIOUG56490-E08|Malaise trap PL12-9D|657[0n]|BOLD:AAM1059
Stantonia billalleni|[174]|BIOUG52997-E04|Malaise trap PL12-3C|655[0n]|BOLD:AAM1059
Stantonia billalleni|[175]|BIOUG55345-G12|Malaise trap PL12-4D|654[0n]|BOLD:AAM1059
Stantonia billalleni|[176]|DHJPAR0051311|Hyalorista exuvialisDHJ02|658[0n]|BOLD:AAM1059
Stantonia ruthtifferae|[177]|DHJPAR0037144|Microthyris prolongalisDHJ03|638[0n]|BOLD:AAB3996
Stantonia ruthtifferae|[178]|DHJPAR0049372|Stenoma luctificaDHJ02|618[0n]|BOLD:AAB3996
Stantonia donwilsoni|[179]|DHJPAR0029187|Omiodes humeralis|611[0n]|BOLD:AAG3930
Stantonia donwilsoni|[180]|DHJPAR0052875|Portentomorpha xanthialis|658[0n]|BOLD:AAG3930
Stantonia donwilsoni|[181]|DHJPAR0052706|Portentomorpha xanthialis|658[0n]|BOLD:AAG3930
Stantonia donwilsoni|[182]|DHJPAR0063896|Hahncappsia BioLep471|621[0n]|BOLD:AAG3930
Stantonia donwilsoni|[183]|DHJPAR0063891|Phostria latiapicalis|549[0n]|BOLD:AAG3930
Stantonia donwilsoni|[184]|DHJPAR0029429|Psara obscuralisDHJ02|632[0n]|BOLD:AAG3930
Stantonia donwilsoni|[185]|DHJPAR0041487|Eulepte Janzen06|658[0n]|BOLD:AAG3930
Stantonia donwilsoni|[186]|DHJPAR0052872|Portentomorpha xanthialis|658[0n]|BOLD:AAG3930
Stantonia miriamzunzae|[187]|DHJPAR0048704|Ategumia lotanalis|658[0n]|BOLD:ACB1896
Stantonia Janzen3983|[188]|DHJPAR0065133|Casandria Poole01|614[0n]|BOLD:AEC3983
Stantonia Janzen5021|[189]|DHJPAR0029435|immidJanzen01 Janzen16|660[0n]|BOLD:AAJ5021
Stantonia garywolfi|[190]|DHJPAR0029189|immidJanzen01 Janzen02|406[0n]|BOLD:AAL9316
Stantonia garywolfi|[191]|DHJPAR0029188|immidJanzen01 Janzen02|623[0n]|BOLD:AAL9316
Stantonia brookejarvisae|[192]|DHJPAR0053534|Pilocrocis purpurascens|661[0n]|BOLD:AAK5505
Stantonia brookejarvisae|[193]|DHJPAR0036321|Pilocrocis purpurascens|614[0n]|BOLD:AAK5505
Stantonia brookejarvisae|[194]|DHJPAR0050920|Pilocrocis purpurascens|618[0n]|BOLD:AAK5505
Stantonia brookejarvisae|[195]|DHJPAR0052099|Pilocrocis purpurascens|648[0n]|BOLD:AAK5505
Stantonia brookejarvisae|[196]|DHJPAR0040550|Pilocrocis purpurascens|646[0n]|BOLD:AAK5505
Stantonia brookejarvisae|[197]|DHJPAR0057266|Phostria latiapicalis|661[0n]|BOLD:AAK5505
Stantonia brookejarvisae|[198]|DHJPAR0053538|Pilocrocis purpurascens|664[0n]|BOLD:AAK5505
Stantonia brookejarvisae|[199]|DHJPAR0065121|Phostria latiapicalis|630[0n]|BOLD:AAK5505
Stantonia brookejarvisae|[200]|DHJPAR0050911|Desmia benealisDHJ02|643[0n]|BOLD:AAK5505
Stantonia brookejarvisae|[201]|DHJPAR0050354|Pilocrocis purpurascens|658[0n]|BOLD:AAK5505
Stantonia quentinwheeleri|[202]|DHJPAR0045054|Syllepis marialis|658[0n]|BOLD:AAD5731
Stantonia quentinwheeleri|[203]|DHJPAR0029430|Syllepis hortalis|657[0n]|BOLD:AAD5731
Stantonia quentinwheeleri|[204]|DHJPAR0029431|Syllepis hortalis|571[0n]|BOLD:AAD5731
Stantonia quentinwheeleri|[205]|DHJPAR0036719|Syllepis hortalis|644[0n]|BOLD:AAD5731
Stantonia quentinwheeleri|[206]|DHJPAR0029432|Syllepis hortalis|657[0n]|BOLD:AAD5731
Stantonia quentinwheeleri|[207]|DHJPAR0029434|Syllepis hortalis|657[0n]|BOLD:AAD5731
Stantonia quentinwheeleri|[208]|DHJPAR0029433|Syllepis hortalis|657[0n]|BOLD:AAD5731
Stantonia henrikekmani|[209]|DHJPAR0035221|Eulepte Janzen03|658[0n]|BOLD:AAI2111
Stantonia henrikekmani|[210]|DHJPAR0035220|Eulepte Janzen03|658[0n]|BOLD:AAI2111
Stantonia henrikekmani|[211]|DHJPAR0050358|Pilocrocis Solis20|658[0n]|BOLD:AAI2111
Stantonia henrikekmani|[212]|DHJPAR0052825|Desmia benealisDHJ02|658[0n]|BOLD:AAI2111
Stantonia henrikekmani|[213]|DHJPAR0054462|Desmia ploralisDHJ03|658[0n]|BOLD:AAI2111
Stantonia henrikekmani|[214]|DHJPAR0054466|Eulepte Janzen03|658[0n]|BOLD:AAI2111
Stantonia henrikekmani|[215]|DHJPAR0056229|Ceratocilia sixolalis|626[0n]|BOLD:AAI2111
Stantonia henrikekmani|[216]|DHJPAR0054465|Eulepte Janzen03|658[0n]|BOLD:AAI2111
Stantonia henrikekmani|[217]|DHJPAR0056233|Ceratocilia sixolalis|670[0n]|BOLD:AAI2111
Stantonia henrikekmani|[218]|DHJPAR0056230|Ceratocilia sixolalis|670[0n]|BOLD:AAI2111
Stantonia henrikekmani|[219]|DHJPAR0056234|Ceratocilia sixolalis|670[0n]|BOLD:AAI2111
Stantonia henrikekmani|[220]|DHJPAR0056231|Ceratocilia sixolalis|670[0n]|BOLD:AAI2111
Stantonia henrikekmani|[221]|DHJPAR0056232|Ceratocilia sixolalis|670[0n]|BOLD:AAI2111
