# Supplementary material for: Minimalist revision and description of 403 new species in 11 subfamilies of Costa Rican braconid parasitoid wasps, including host records for 219 species
Source: Zookeys. 2021 Feb 2;1013:1–665. doi: 10.3897/zookeys.1013.55600 (PMC8390796; doi:10.3897/zookeys.1013.55600)
Supplement: Supplementary material 9 — Rhysipolinae [file zookeys-1013-001-s009.pdf]

9. Rhysipolinae BOLD TaxonID Tree

```
Title           : Tree Result - Search:  Sample IDs (17 records returned) (17 records
selected)
Date            : 17-Nov-2020
Data Type       : Nucleotide
Distance Model  : Kimura 2 Parameter
Marker          : COI-5P
Colourization   : [blue]=Stop Codons [red]=Contamination or misidentification

Label           : Sample ID
Label           : Taxon
Label           : Extra Info
Label           : Sequence Length
Label           : Barcode Cluster (BIN)

Filter          : exclude records with stop codons

Sequence Count  : 17
Species count   : 5
Genus count     : 2
Family count    : 1
Unidentified    : 1

BIN Count       : 5
```

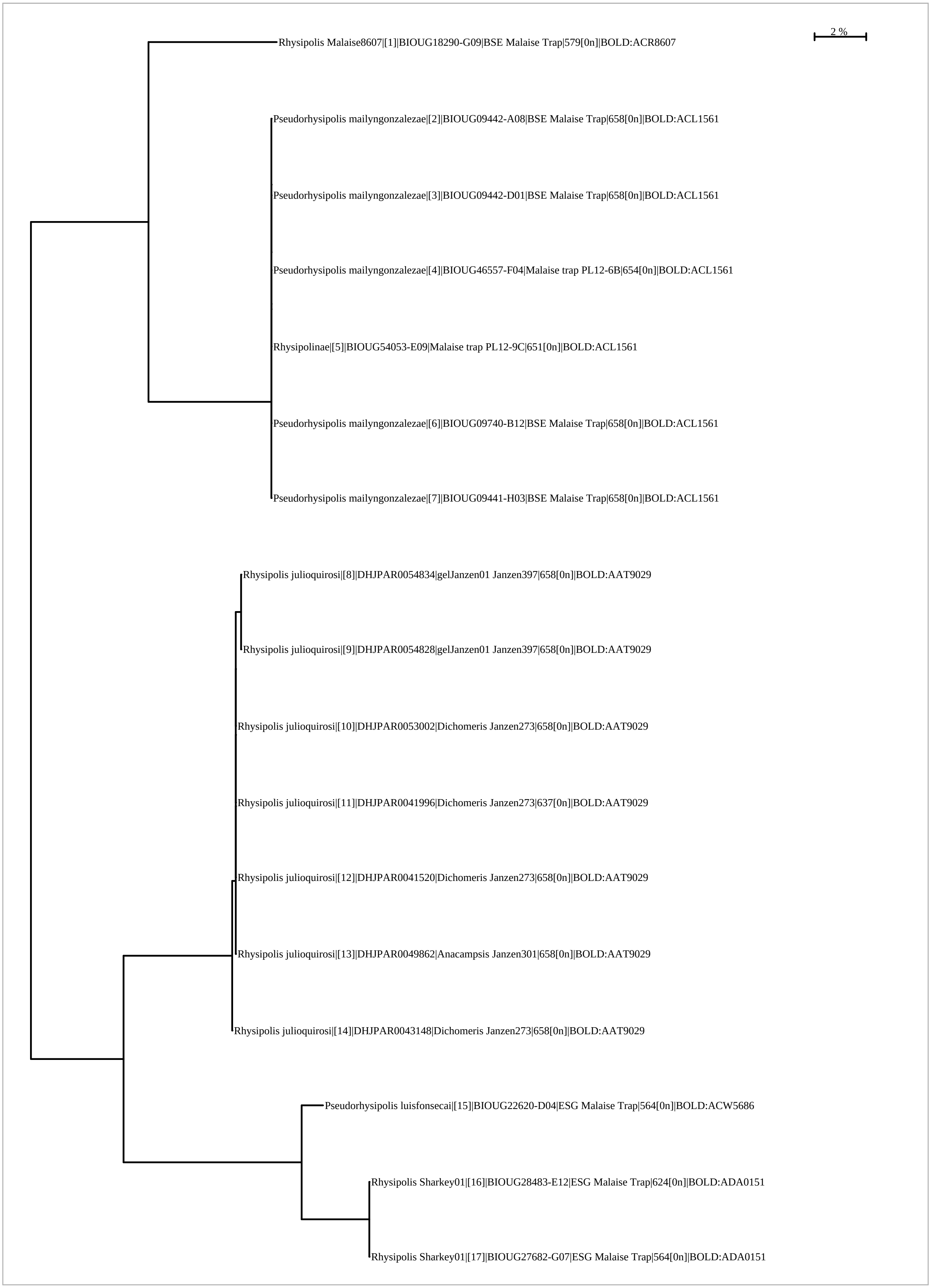
2 %
Rhysipolis Malaise8607|[1]|BIOUG18290-G09|BSE Malaise Trap|579[0n]|BOLD:ACR8607
Pseudorhysipolis mailyngonzalezae|[2]|BIOUG09442-A08|BSE Malaise Trap|658[0n]|BOLD:ACL1561
Pseudorhysipolis mailyngonzalezae|[3]|BIOUG09442-D01|BSE Malaise Trap|658[0n]|BOLD:ACL1561
Pseudorhysipolis mailyngonzalezae|[4]|BIOUG46557-F04|Malaise trap PL12-6B|654[0n]|BOLD:ACL1561
Rhysipolinae|[5]|BIOUG54053-E09|Malaise trap PL12-9C|651[0n]|BOLD:ACL1561
Pseudorhysipolis mailyngonzalezae|[6]|BIOUG09740-B12|BSE Malaise Trap|658[0n]|BOLD:ACL1561
Pseudorhysipolis mailyngonzalezae|[7]|BIOUG09441-H03|BSE Malaise Trap|658[0n]|BOLD:ACL1561
Rhysipolis julioquirosi|[8]|DHJPAR0054834|gelJanzen01 Janzen397|658[0n]|BOLD:AAT9029
Rhysipolis julioquirosi|[9]|DHJPAR0054828|gelJanzen01 Janzen397|658[0n]|BOLD:AAT9029
Rhysipolis julioquirosi|[10]|DHJPAR0053002|Dichomeris Janzen273|658[0n]|BOLD:AAT9029
Rhysipolis julioquirosi|[11]|DHJPAR0041996|Dichomeris Janzen273|637[0n]|BOLD:AAT9029
Rhysipolis julioquirosi|[12]|DHJPAR0041520|Dichomeris Janzen273|658[0n]|BOLD:AAT9029
Rhysipolis julioquirosi|[13]|DHJPAR0049862|Anacampsis Janzen301|658[0n]|BOLD:AAT9029
Rhysipolis julioquirosi|[14]|DHJPAR0043148|Dichomeris Janzen273|658[0n]|BOLD:AAT9029
Pseudorhysipolis luisfonsecai|[15]|BIOUG22620-D04|ESG Malaise Trap|564[0n]|BOLD:ACW5686
Rhysipolis Sharkey01|[16]|BIOUG28483-E12|ESG Malaise Trap|624[0n]|BOLD:ADA0151
Rhysipolis Sharkey01|[17]|BIOUG27682-G07|ESG Malaise Trap|564[0n]|BOLD:ADA0151
