# Supplementary material for: Minimalist revision and description of 403 new species in 11 subfamilies of Costa Rican braconid parasitoid wasps, including host records for 219 species
Source: Zookeys. 2021 Feb 2;1013:1–665. doi: 10.3897/zookeys.1013.55600 (PMC8390796; doi:10.3897/zookeys.1013.55600)
Supplement: Supplementary material 10 — Rogadinae [file zookeys-1013-001-s010.pdf]

10. Rogadinae BOLD TaxonID Tree

Title : Tree Result - Search: Sample IDs (699 records returned) (699 records selected)

Date : 17-Nov-2020

Data Type : Nucleotide

Distance Model : Kimura 2 Parameter

Marker : COI-5P

Colourization : [blue]=Stop Codons [red]=Contamination or misidentification

Label : Sample ID

Label : Taxon

Label : Extra Info

Label : Sequence Length

Label : Barcode Cluster (BIN)

Filter : exclude records with stop codons

Sequence Count : 680

Species count : 134

Genus count : 10

Family count : 1

Unidentified : 36

BIN Count : 129

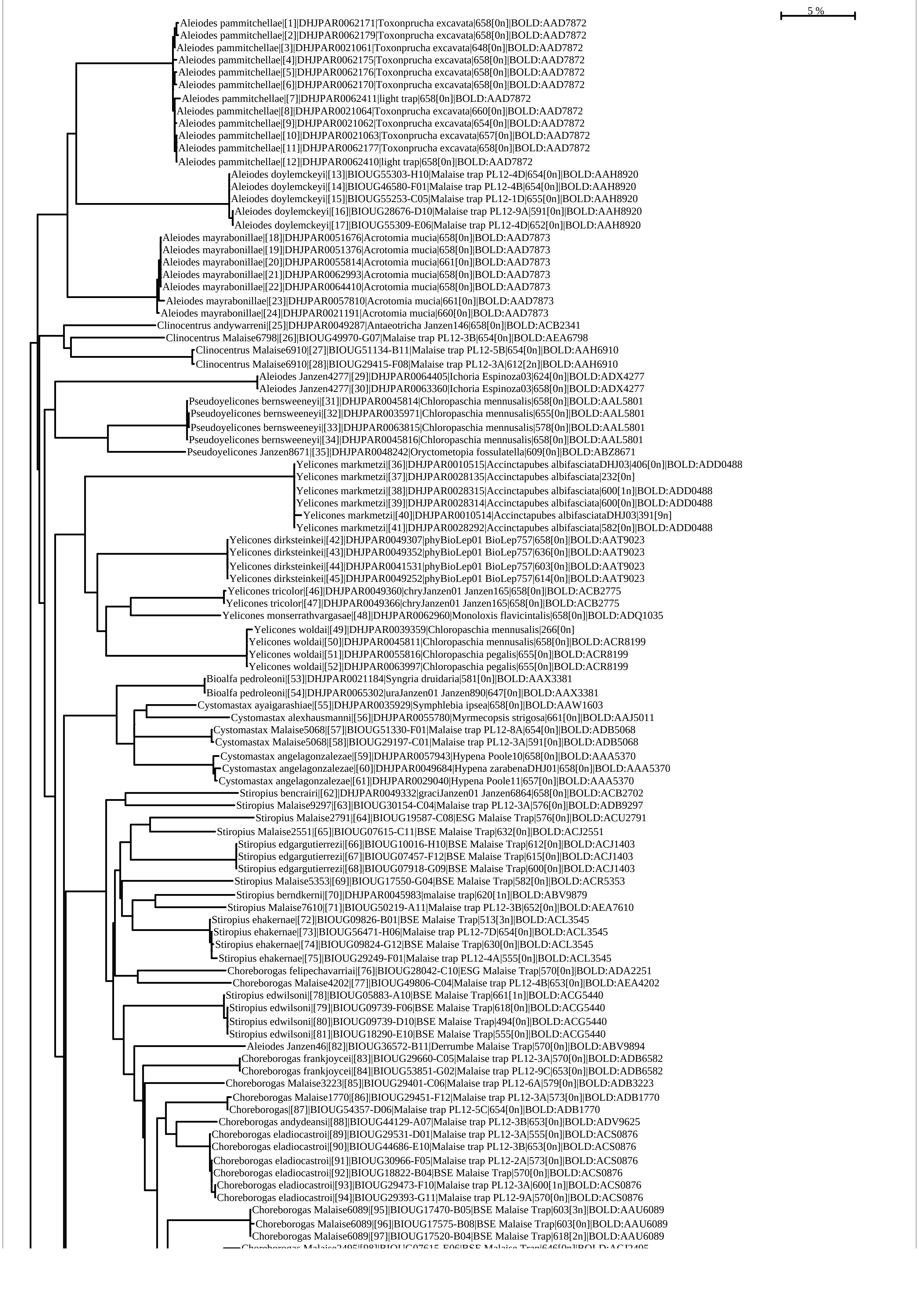
5 %
Aleiodes pammitchellae|[1]|DHJPAR0062171|Toxonprucha excavata|658[0n]|BOLD:AAD7872
Aleiodes pammitchellae|[2]|DHJPAR0062179|Toxonprucha excavata|658[0n]|BOLD:AAD7872
Aleiodes pammitchellae|[3]|DHJPAR0021061|Toxonprucha excavata|648[0n]|BOLD:AAD7872
Aleiodes pammitchellae|[4]|DHJPAR0062175|Toxonprucha excavata|658[0n]|BOLD:AAD7872
Aleiodes pammitchellae|[5]|DHJPAR0062176|Toxonprucha excavata|658[0n]|BOLD:AAD7872
Aleiodes pammitchellae|[6]|DHJPAR0062170|Toxonprucha excavata|658[0n]|BOLD:AAD7872
Aleiodes pammitchellae|[7]|DHJPAR0062411|light trap|658[0n]|BOLD:AAD7872
Aleiodes pammitchellae|[8]|DHJPAR0021064|Toxonprucha excavata|660[0n]|BOLD:AAD7872
Aleiodes pammitchellae|[9]|DHJPAR0021062|Toxonprucha excavata|654[0n]|BOLD:AAD7872
Aleiodes pammitchellae|[10]|DHJPAR0021063|Toxonprucha excavata|657[0n]|BOLD:AAD7872
Aleiodes pammitchellae|[11]|DHJPAR0062177|Toxonprucha excavata|658[0n]|BOLD:AAD7872
Aleiodes pammitchellae|[12]|DHJPAR0062410|light trap|658[0n]|BOLD:AAD7872
Aleiodes doylemckeyi|[13]|BIOUG55303-H10|Malaise trap PL12-4D|654[0n]|BOLD:AAH8920
Aleiodes doylemckeyi|[14]|BIOUG46580-F01|Malaise trap PL12-4B|654[0n]|BOLD:AAH8920
Aleiodes doylemckeyi|[15]|BIOUG55253-C05|Malaise trap PL12-1D|655[0n]|BOLD:AAH8920
Aleiodes doylemckeyi|[16]|BIOUG28676-D10|Malaise trap PL12-9A|591[0n]|BOLD:AAH8920
Aleiodes doylemckeyi|[17]|BIOUG55309-E06|Malaise trap PL12-4D|652[0n]|BOLD:AAH8920
Aleiodes mayrabonillae|[18]|DHJPAR0051676|Acrotomia mucia|658[0n]|BOLD:AAD7873
Aleiodes mayrabonillae|[19]|DHJPAR0051376|Acrotomia mucia|658[0n]|BOLD:AAD7873
Aleiodes mayrabonillae|[20]|DHJPAR0055814|Acrotomia mucia|661[0n]|BOLD:AAD7873
Aleiodes mayrabonillae|[21]|DHJPAR0062993|Acrotomia mucia|658[0n]|BOLD:AAD7873
Aleiodes mayrabonillae|[22]|DHJPAR0064410|Acrotomia mucia|658[0n]|BOLD:AAD7873
Aleiodes mayrabonillae|[23]|DHJPAR0057810|Acrotomia mucia|661[0n]|BOLD:AAD7873
Aleiodes mayrabonillae|[24]|DHJPAR0021191|Acrotomia mucia|660[0n]|BOLD:AAD7873
Clinocentrus andywarreni|[25]|DHJPAR0049287|Antaeotricha Janzen146|658[0n]|BOLD:ACB2341
Clinocentrus Malaise6798|[26]|BIOUG49970-G07|Malaise trap PL12-3B|654[0n]|BOLD:AEA6798
Clinocentrus Malaise6910|[27]|BIOUG51134-B11|Malaise trap PL12-5B|654[0n]|BOLD:AAH6910
Clinocentrus Malaise6910|[28]|BIOUG29415-F08|Malaise trap PL12-3A|612[2n]|BOLD:AAH6910
Aleiodes Janzen4277|[29]|DHJPAR0064405|Ichoria Espinoza03|624[0n]|BOLD:ADX4277
Aleiodes Janzen4277|[30]|DHJPAR0063360|Ichoria Espinoza03|658[0n]|BOLD:ADX4277
Pseudoyelicones bernsweeneyi|[31]|DHJPAR0045814|Chloropaschia mennusalis|658[0n]|BOLD:AAL5801
Pseudoyelicones bernsweeneyi|[32]|DHJPAR0035971|Chloropaschia mennusalis|655[0n]|BOLD:AAL5801
Pseudoyelicones bernsweeneyi|[33]|DHJPAR0063815|Chloropaschia mennusalis|578[0n]|BOLD:AAL5801
Pseudoyelicones bernsweeneyi|[34]|DHJPAR0045816|Chloropaschia mennusalis|658[0n]|BOLD:AAL5801
Pseudoyelicones Janzen8671|[35]|DHJPAR0048242|Oryctometopia fossulatella|609[0n]|BOLD:ABZ8671
Yelicones markmetzi|[36]|DHJPAR0010515|Accinctapubes albifasciataDHJ03|406[0n]|BOLD:ADD0488
Yelicones markmetzi|[37]|DHJPAR0028135|Accinctapubes albifasciata|232[0n]
Yelicones markmetzi|[38]|DHJPAR0028315|Accinctapubes albifasciata|600[1n]|BOLD:ADD0488
Yelicones markmetzi|[39]|DHJPAR0028314|Accinctapubes albifasciata|600[0n]|BOLD:ADD0488
Yelicones markmetzi|[40]|DHJPAR0010514|Accinctapubes albifasciataDHJ03|391[9n]
Yelicones markmetzi|[41]|DHJPAR0028292|Accinctapubes albifasciata|582[0n]|BOLD:ADD0488
Yelicones dirksteinkei|[42]|DHJPAR0049307|phyBioLep01 BioLep757|658[0n]|BOLD:AAT9023
Yelicones dirksteinkei|[43]|DHJPAR0049352|phyBioLep01 BioLep757|636[0n]|BOLD:AAT9023
Yelicones dirksteinkei|[44]|DHJPAR0041531|phyBioLep01 BioLep757|603[0n]|BOLD:AAT9023
Yelicones dirksteinkei|[45]|DHJPAR0049252|phyBioLep01 BioLep757|614[0n]|BOLD:AAT9023
Yelicones tricolor|[46]|DHJPAR0049360|chryJanzen01 Janzen165|658[0n]|BOLD:ACB2775
Yelicones tricolor|[47]|DHJPAR0049366|chryJanzen01 Janzen165|658[0n]|BOLD:ACB2775
Yelicones monserrathvargasae|[48]|DHJPAR0062960|Monoloxis flavicintalis|658[0n]|BOLD:ADQ1035
Yelicones woldai|[49]|DHJPAR0039359|Chloropaschia mennusalis|266[0n]
Yelicones woldai|[50]|DHJPAR0045811|Chloropaschia mennusalis|658[0n]|BOLD:ACR8199
Yelicones woldai|[51]|DHJPAR0055816|Chloropaschia pegalis|655[0n]|BOLD:ACR8199
Yelicones woldai|[52]|DHJPAR0063997|Chloropaschia pegalis|655[0n]|BOLD:ACR8199
Bioalfa pedroleoni|[53]|DHJPAR0021184|Syngria druidaria|581[0n]|BOLD:AAX3381
Bioalfa pedroleoni|[54]|DHJPAR0065302|uraJanzen01 Janzen890|647[0n]|BOLD:AAX3381
Cystomastax ayaigarashiae|[55]|DHJPAR0035929|Symphlebia ipsea|658[0n]|BOLD:AAW1603
Cystomastax alexhausmanni|[56]|DHJPAR0055780|Myrmecopsis strigosa|661[0n]|BOLD:AAJ5011
Cystomastax Malaise5068|[57]|BIOUG51330-F01|Malaise trap PL12-8A|654[0n]|BOLD:ADB5068
Cystomastax Malaise5068|[58]|BIOUG29197-C01|Malaise trap PL12-3A|591[0n]|BOLD:ADB5068
Cystomastax angelagonzalezae|[59]|DHJPAR0057943|Hypena Poole10|658[0n]|BOLD:AAA5370
Cystomastax angelagonzalezae|[60]|DHJPAR0049684|Hypena zarabenaDHJ01|658[0n]|BOLD:AAA5370
Cystomastax angelagonzalezae|[61]|DHJPAR0029040|Hypena Poole11|657[0n]|BOLD:AAA5370
Stiropius bencrairi|[62]|DHJPAR0049332|graciJanzen01 Janzen6864|658[0n]|BOLD:ACB2702
Stiropius Malaise9297|[63]|BIOUG30154-C04|Malaise trap PL12-3A|576[0n]|BOLD:ADB9297
Stiropius Malaise2791|[64]|BIOUG19587-C08|ESG Malaise Trap|576[0n]|BOLD:ACU2791
Stiropius Malaise2551|[65]|BIOUG07615-C11|BSE Malaise Trap|632[0n]|BOLD:ACJ2551
Stiropius edgargutierrezi|[66]|BIOUG10016-H10|BSE Malaise Trap|612[0n]|BOLD:ACJ1403
Stiropius edgargutierrezi|[67]|BIOUG07457-F12|BSE Malaise Trap|615[0n]|BOLD:ACJ1403
Stiropius edgargutierrezi|[68]|BIOUG07918-G09|BSE Malaise Trap|600[0n]|BOLD:ACJ1403
Stiropius Malaise5353|[69]|BIOUG17550-G04|BSE Malaise Trap|582[0n]|BOLD:ACR5353
Stiropius berndkerni|[70]|DHJPAR0045983|malaise trap|620[1n]|BOLD:ABV9879
Stiropius Malaise7610|[71]|BIOUG50219-A11|Malaise trap PL12-3B|652[0n]|BOLD:AEA7610
Stiropius ehakernae|[72]|BIOUG09826-B01|BSE Malaise Trap|513[3n]|BOLD:ACL3545
Stiropius ehakernae|[73]|BIOUG56471-H06|Malaise trap PL12-7D|654[0n]|BOLD:ACL3545
Stiropius ehakernae|[74]|BIOUG09824-G12|BSE Malaise Trap|630[0n]|BOLD:ACL3545
Stiropius ehakernae|[75]|BIOUG29249-F01|Malaise trap PL12-4A|555[0n]|BOLD:ACL3545
Choreborogas felipechavarriai|[76]|BIOUG28042-C10|ESG Malaise Trap|570[0n]|BOLD:ADA2251
Choreborogas Malaise4202|[77]|BIOUG49806-C04|Malaise trap PL12-4B|653[0n]|BOLD:AEA4202
Stiropius edwilsoni|[78]|BIOUG05883-A10|BSE Malaise Trap|661[1n]|BOLD:ACG5440
Stiropius edwilsoni|[79]|BIOUG09739-F06|BSE Malaise Trap|618[0n]|BOLD:ACG5440
Stiropius edwilsoni|[80]|BIOUG09739-D10|BSE Malaise Trap|494[0n]|BOLD:ACG5440
Stiropius edwilsoni|[81]|BIOUG18290-E10|BSE Malaise Trap|555[0n]|BOLD:ACG5440
Aleiodes Janzen46|[82]|BIOUG36572-B11|Derrumbe Malaise Trap|570[0n]|BOLD:ABV9894
Choreborogas frankjoycei|[83]|BIOUG29660-C05|Malaise trap PL12-3A|570[0n]|BOLD:ADB6582
Choreborogas frankjoycei|[84]|BIOUG53851-G02|Malaise trap PL12-9C|653[0n]|BOLD:ADB6582
Choreborogas Malaise3223|[85]|BIOUG29401-C06|Malaise trap PL12-6A|579[0n]|BOLD:ADB3223
Choreborogas Malaise1770|[86]|BIOUG29451-F12|Malaise trap PL12-3A|573[0n]|BOLD:ADB1770
Choreborogas|[87]|BIOUG54357-D06|Malaise trap PL12-5C|654[0n]|BOLD:ADB1770
Choreborogas andydeansi|[88]|BIOUG44129-A07|Malaise trap PL12-3B|653[0n]|BOLD:ADV9625
Choreborogas eladiocastroi|[89]|BIOUG29531-D01|Malaise trap PL12-3A|555[0n]|BOLD:ACS0876
Choreborogas eladiocastroi|[90]|BIOUG44686-E10|Malaise trap PL12-3B|653[0n]|BOLD:ACS0876
Choreborogas eladiocastroi|[91]|BIOUG30966-F05|Malaise trap PL12-2A|573[0n]|BOLD:ACS0876
Choreborogas eladiocastroi|[92]|BIOUG18822-B04|BSE Malaise Trap|570[0n]|BOLD:ACS0876
Choreborogas eladiocastroi|[93]|BIOUG29473-F10|Malaise trap PL12-3A|600[1n]|BOLD:ACS0876
Choreborogas eladiocastroi|[94]|BIOUG29393-G11|Malaise trap PL12-9A|570[0n]|BOLD:ACS0876
Choreborogas Malaise6089|[95]|BIOUG17470-B05|BSE Malaise Trap|603[3n]|BOLD:AAU6089
Choreborogas Malaise6089|[96]|BIOUG17575-B08|BSE Malaise Trap|603[0n]|BOLD:AAU6089
Choreborogas Malaise6089|[97]|BIOUG17520-B04|BSE Malaise Trap|618[2n]|BOLD:AAU6089

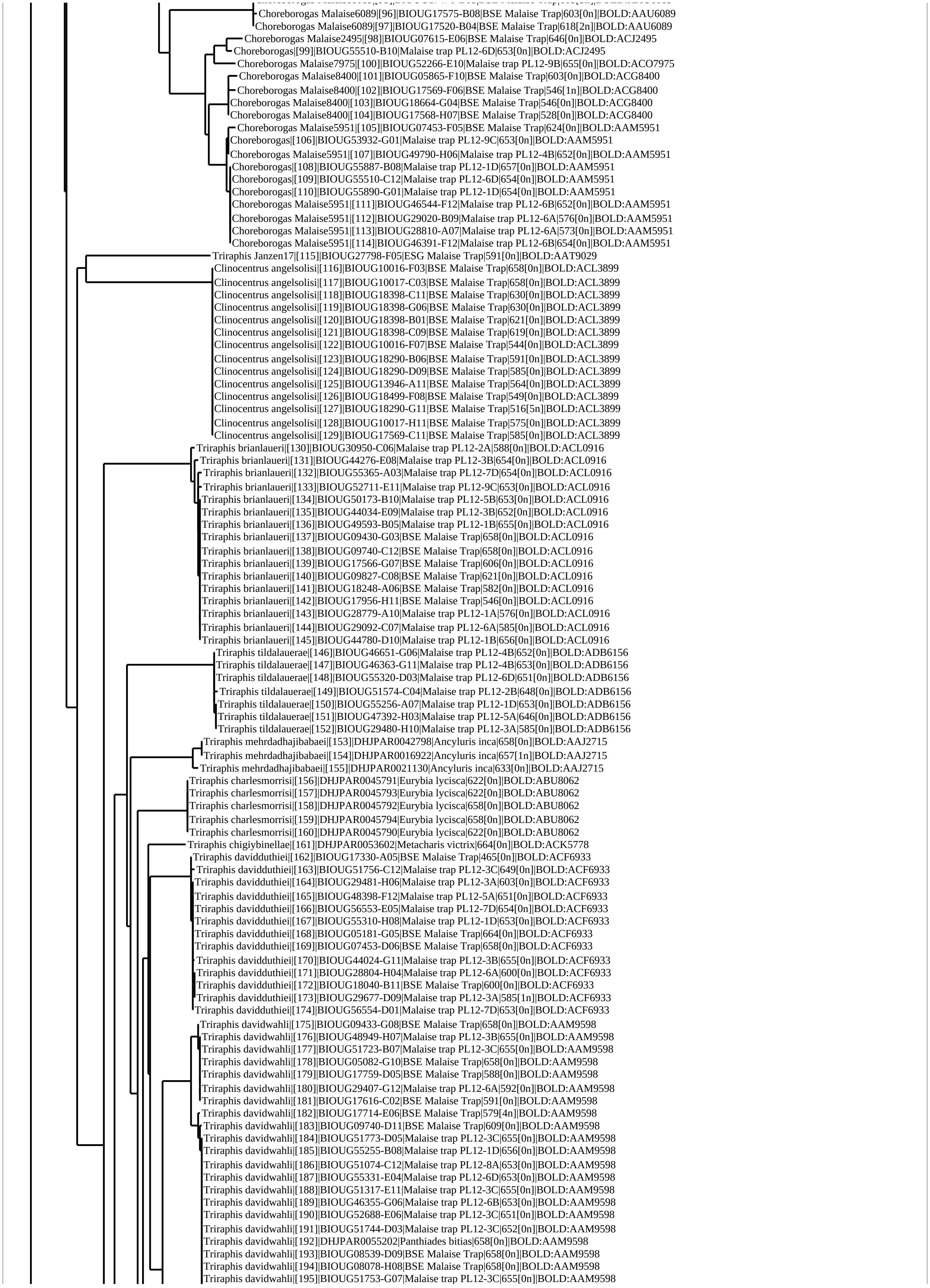
Choreborogas Malaise6089|[96]|BIOUG17575-B08|BSE Malaise Trap|603[0n]|BOLD:AAU6089
Choreborogas Malaise6089|[97]|BIOUG17520-B04|BSE Malaise Trap|618[2n]|BOLD:AAU6089
Choreborogas Malaise2495|[98]|BIOUG07615-E06|BSE Malaise Trap|646[0n]|BOLD:ACJ2495
Choreborogas|[99]|BIOUG55510-B10|Malaise trap PL12-6D|653[0n]|BOLD:ACJ2495
Choreborogas Malaise7975|[100]|BIOUG52266-E10|Malaise trap PL12-9B|655[0n]|BOLD:ACO7975
Choreborogas Malaise8400|[101]|BIOUG05865-F10|BSE Malaise Trap|603[0n]|BOLD:ACG8400
Choreborogas Malaise8400|[102]|BIOUG17569-F06|BSE Malaise Trap|546[1n]|BOLD:ACG8400
Choreborogas Malaise8400|[103]|BIOUG18664-G04|BSE Malaise Trap|546[0n]|BOLD:ACG8400
Choreborogas Malaise8400|[104]|BIOUG17568-H07|BSE Malaise Trap|528[0n]|BOLD:ACG8400
Choreborogas Malaise5951|[105]|BIOUG07453-F05|BSE Malaise Trap|624[0n]|BOLD:AAM5951
Choreborogas|[106]|BIOUG53932-G01|Malaise trap PL12-9C|653[0n]|BOLD:AAM5951
Choreborogas Malaise5951|[107]|BIOUG49790-H06|Malaise trap PL12-4B|652[0n]|BOLD:AAM5951
Choreborogas|[108]|BIOUG55887-B08|Malaise trap PL12-1D|657[0n]|BOLD:AAM5951
Choreborogas|[109]|BIOUG55510-C12|Malaise trap PL12-6D|654[0n]|BOLD:AAM5951
Choreborogas|[110]|BIOUG55890-G01|Malaise trap PL12-1D|654[0n]|BOLD:AAM5951
Choreborogas Malaise5951|[111]|BIOUG46544-F12|Malaise trap PL12-6B|652[0n]|BOLD:AAM5951
Choreborogas Malaise5951|[112]|BIOUG29020-B09|Malaise trap PL12-6A|576[0n]|BOLD:AAM5951
Choreborogas Malaise5951|[113]|BIOUG28810-A07|Malaise trap PL12-6A|573[0n]|BOLD:AAM5951
Choreborogas Malaise5951|[114]|BIOUG46391-F12|Malaise trap PL12-6B|654[0n]|BOLD:AAM5951
Triraphis Janzen17|[115]|BIOUG27798-F05|ESG Malaise Trap|591[0n]|BOLD:AAT9029
Clinocentrus angelsolisi|[116]|BIOUG10016-F03|BSE Malaise Trap|658[0n]|BOLD:ACL3899
Clinocentrus angelsolisi|[117]|BIOUG10017-C03|BSE Malaise Trap|658[0n]|BOLD:ACL3899
Clinocentrus angelsolisi|[118]|BIOUG18398-C11|BSE Malaise Trap|630[0n]|BOLD:ACL3899
Clinocentrus angelsolisi|[119]|BIOUG18398-G06|BSE Malaise Trap|630[0n]|BOLD:ACL3899
Clinocentrus angelsolisi|[120]|BIOUG18398-B01|BSE Malaise Trap|621[0n]|BOLD:ACL3899
Clinocentrus angelsolisi|[121]|BIOUG18398-C09|BSE Malaise Trap|619[0n]|BOLD:ACL3899
Clinocentrus angelsolisi|[122]|BIOUG10016-F07|BSE Malaise Trap|544[0n]|BOLD:ACL3899
Clinocentrus angelsolisi|[123]|BIOUG18290-B06|BSE Malaise Trap|591[0n]|BOLD:ACL3899
Clinocentrus angelsolisi|[124]|BIOUG18290-D09|BSE Malaise Trap|585[0n]|BOLD:ACL3899
Clinocentrus angelsolisi|[125]|BIOUG13946-A11|BSE Malaise Trap|564[0n]|BOLD:ACL3899
Clinocentrus angelsolisi|[126]|BIOUG18499-F08|BSE Malaise Trap|549[0n]|BOLD:ACL3899
Clinocentrus angelsolisi|[127]|BIOUG18290-G11|BSE Malaise Trap|516[5n]|BOLD:ACL3899
Clinocentrus angelsolisi|[128]|BIOUG10017-H11|BSE Malaise Trap|575[0n]|BOLD:ACL3899
Clinocentrus angelsolisi|[129]|BIOUG17569-C11|BSE Malaise Trap|585[0n]|BOLD:ACL3899
Triraphis brianlaueri|[130]|BIOUG30950-C06|Malaise trap PL12-2A|588[0n]|BOLD:ACL0916
Triraphis brianlaueri|[131]|BIOUG44276-E08|Malaise trap PL12-3B|654[0n]|BOLD:ACL0916
Triraphis brianlaueri|[132]|BIOUG55365-A03|Malaise trap PL12-7D|654[0n]|BOLD:ACL0916
Triraphis brianlaueri|[133]|BIOUG52711-E11|Malaise trap PL12-9C|653[0n]|BOLD:ACL0916
Triraphis brianlaueri|[134]|BIOUG50173-B10|Malaise trap PL12-5B|653[0n]|BOLD:ACL0916
Triraphis brianlaueri|[135]|BIOUG44034-E09|Malaise trap PL12-3B|652[0n]|BOLD:ACL0916
Triraphis brianlaueri|[136]|BIOUG49593-B05|Malaise trap PL12-1B|655[0n]|BOLD:ACL0916
Triraphis brianlaueri|[137]|BIOUG09430-G03|BSE Malaise Trap|658[0n]|BOLD:ACL0916
Triraphis brianlaueri|[138]|BIOUG09740-C12|BSE Malaise Trap|658[0n]|BOLD:ACL0916
Triraphis brianlaueri|[139]|BIOUG17566-G07|BSE Malaise Trap|606[0n]|BOLD:ACL0916
Triraphis brianlaueri|[140]|BIOUG09827-C08|BSE Malaise Trap|621[0n]|BOLD:ACL0916
Triraphis brianlaueri|[141]|BIOUG18248-A06|BSE Malaise Trap|582[0n]|BOLD:ACL0916
Triraphis brianlaueri|[142]|BIOUG17956-H11|BSE Malaise Trap|546[0n]|BOLD:ACL0916
Triraphis brianlaueri|[143]|BIOUG28779-A10|Malaise trap PL12-1A|576[0n]|BOLD:ACL0916
Triraphis brianlaueri|[144]|BIOUG29092-C07|Malaise trap PL12-6A|585[0n]|BOLD:ACL0916
Triraphis brianlaueri|[145]|BIOUG44780-D10|Malaise trap PL12-1B|656[0n]|BOLD:ACL0916
Triraphis tildalauerae|[146]|BIOUG46651-G06|Malaise trap PL12-4B|652[0n]|BOLD:ADB6156
Triraphis tildalauerae|[147]|BIOUG46363-G11|Malaise trap PL12-4B|653[0n]|BOLD:ADB6156
Triraphis tildalauerae|[148]|BIOUG55320-D03|Malaise trap PL12-6D|651[0n]|BOLD:ADB6156
Triraphis tildalauerae|[149]|BIOUG51574-C04|Malaise trap PL12-2B|648[0n]|BOLD:ADB6156
Triraphis tildalauerae|[150]|BIOUG55256-A07|Malaise trap PL12-1D|653[0n]|BOLD:ADB6156
Triraphis tildalauerae|[151]|BIOUG47392-H03|Malaise trap PL12-5A|646[0n]|BOLD:ADB6156
Triraphis tildalauerae|[152]|BIOUG29480-H10|Malaise trap PL12-3A|585[0n]|BOLD:ADB6156
Triraphis mehrdadhajibabaei|[153]|DHJPAR0042798|Ancyluris inca|658[0n]|BOLD:AAJ2715
Triraphis mehrdadhajibabaei|[154]|DHJPAR0016922|Ancyluris inca|657[1n]|BOLD:AAJ2715
Triraphis mehrdadhajibabaei|[155]|DHJPAR0021130|Ancyluris inca|633[0n]|BOLD:AAJ2715
Triraphis charlesmorrisi|[156]|DHJPAR0045791|Eurybia lycisca|622[0n]|BOLD:ABU8062
Triraphis charlesmorrisi|[157]|DHJPAR0045793|Eurybia lycisca|622[0n]|BOLD:ABU8062
Triraphis charlesmorrisi|[158]|DHJPAR0045792|Eurybia lycisca|658[0n]|BOLD:ABU8062
Triraphis charlesmorrisi|[159]|DHJPAR0045794|Eurybia lycisca|658[0n]|BOLD:ABU8062
Triraphis charlesmorrisi|[160]|DHJPAR0045790|Eurybia lycisca|622[0n]|BOLD:ABU8062
Triraphis chigiybinellae|[161]|DHJPAR0053602|Metacharis victrix|664[0n]|BOLD:ACK5778
Triraphis davidduthiei|[162]|BIOUG17330-A05|BSE Malaise Trap|465[0n]|BOLD:ACF6933
Triraphis davidduthiei|[163]|BIOUG51756-C12|Malaise trap PL12-3C|649[0n]|BOLD:ACF6933
Triraphis davidduthiei|[164]|BIOUG29481-H06|Malaise trap PL12-3A|603[0n]|BOLD:ACF6933
Triraphis davidduthiei|[165]|BIOUG48398-F12|Malaise trap PL12-5A|651[0n]|BOLD:ACF6933
Triraphis davidduthiei|[166]|BIOUG56553-E05|Malaise trap PL12-7D|654[0n]|BOLD:ACF6933
Triraphis davidduthiei|[167]|BIOUG55310-H08|Malaise trap PL12-1D|653[0n]|BOLD:ACF6933
Triraphis davidduthiei|[168]|BIOUG05181-G05|BSE Malaise Trap|664[0n]|BOLD:ACF6933
Triraphis davidduthiei|[169]|BIOUG07453-D06|BSE Malaise Trap|658[0n]|BOLD:ACF6933
Triraphis davidduthiei|[170]|BIOUG44024-G11|Malaise trap PL12-3B|655[0n]|BOLD:ACF6933
Triraphis davidduthiei|[171]|BIOUG28804-H04|Malaise trap PL12-6A|600[0n]|BOLD:ACF6933
Triraphis davidduthiei|[172]|BIOUG18040-B11|BSE Malaise Trap|600[0n]|BOLD:ACF6933
Triraphis davidduthiei|[173]|BIOUG29677-D09|Malaise trap PL12-3A|585[1n]|BOLD:ACF6933
Triraphis davidduthiei|[174]|BIOUG56554-D01|Malaise trap PL12-7D|653[0n]|BOLD:ACF6933
Triraphis davidwahli|[175]|BIOUG09433-G08|BSE Malaise Trap|658[0n]|BOLD:AAM9598
Triraphis davidwahli|[176]|BIOUG48949-H07|Malaise trap PL12-3B|655[0n]|BOLD:AAM9598
Triraphis davidwahli|[177]|BIOUG51723-B07|Malaise trap PL12-3C|655[0n]|BOLD:AAM9598
Triraphis davidwahli|[178]|BIOUG05082-G10|BSE Malaise Trap|658[0n]|BOLD:AAM9598
Triraphis davidwahli|[179]|BIOUG17759-D05|BSE Malaise Trap|588[0n]|BOLD:AAM9598
Triraphis davidwahli|[180]|BIOUG29407-G12|Malaise trap PL12-6A|592[0n]|BOLD:AAM9598
Triraphis davidwahli|[181]|BIOUG17616-C02|BSE Malaise Trap|591[0n]|BOLD:AAM9598
Triraphis davidwahli|[182]|BIOUG17714-E06|BSE Malaise Trap|579[4n]|BOLD:AAM9598
Triraphis davidwahli|[183]|BIOUG09740-D11|BSE Malaise Trap|609[0n]|BOLD:AAM9598
Triraphis davidwahli|[184]|BIOUG51773-D05|Malaise trap PL12-3C|655[0n]|BOLD:AAM9598
Triraphis davidwahli|[185]|BIOUG55255-B08|Malaise trap PL12-1D|656[0n]|BOLD:AAM9598
Triraphis davidwahli|[186]|BIOUG51074-C12|Malaise trap PL12-8A|653[0n]|BOLD:AAM9598
Triraphis davidwahli|[187]|BIOUG55331-E04|Malaise trap PL12-6D|653[0n]|BOLD:AAM9598
Triraphis davidwahli|[188]|BIOUG51317-E11|Malaise trap PL12-3C|655[0n]|BOLD:AAM9598
Triraphis davidwahli|[189]|BIOUG46355-G06|Malaise trap PL12-6B|653[0n]|BOLD:AAM9598
Triraphis davidwahli|[190]|BIOUG52688-E06|Malaise trap PL12-3C|651[0n]|BOLD:AAM9598
Triraphis davidwahli|[191]|BIOUG51744-D03|Malaise trap PL12-3C|652[0n]|BOLD:AAM9598
Triraphis davidwahli|[192]|DHJPAR0055202|Panthiades bitias|658[0n]|BOLD:AAM9598
Triraphis davidwahli|[193]|BIOUG08539-D09|BSE Malaise Trap|658[0n]|BOLD:AAM9598
Triraphis davidwahli|[194]|BIOUG08078-H08|BSE Malaise Trap|658[0n]|BOLD:AAM9598
Triraphis davidwahli|[195]|BIOUG51753-G07|Malaise trap PL12-3C|655[0n]|BOLD:AAM9598

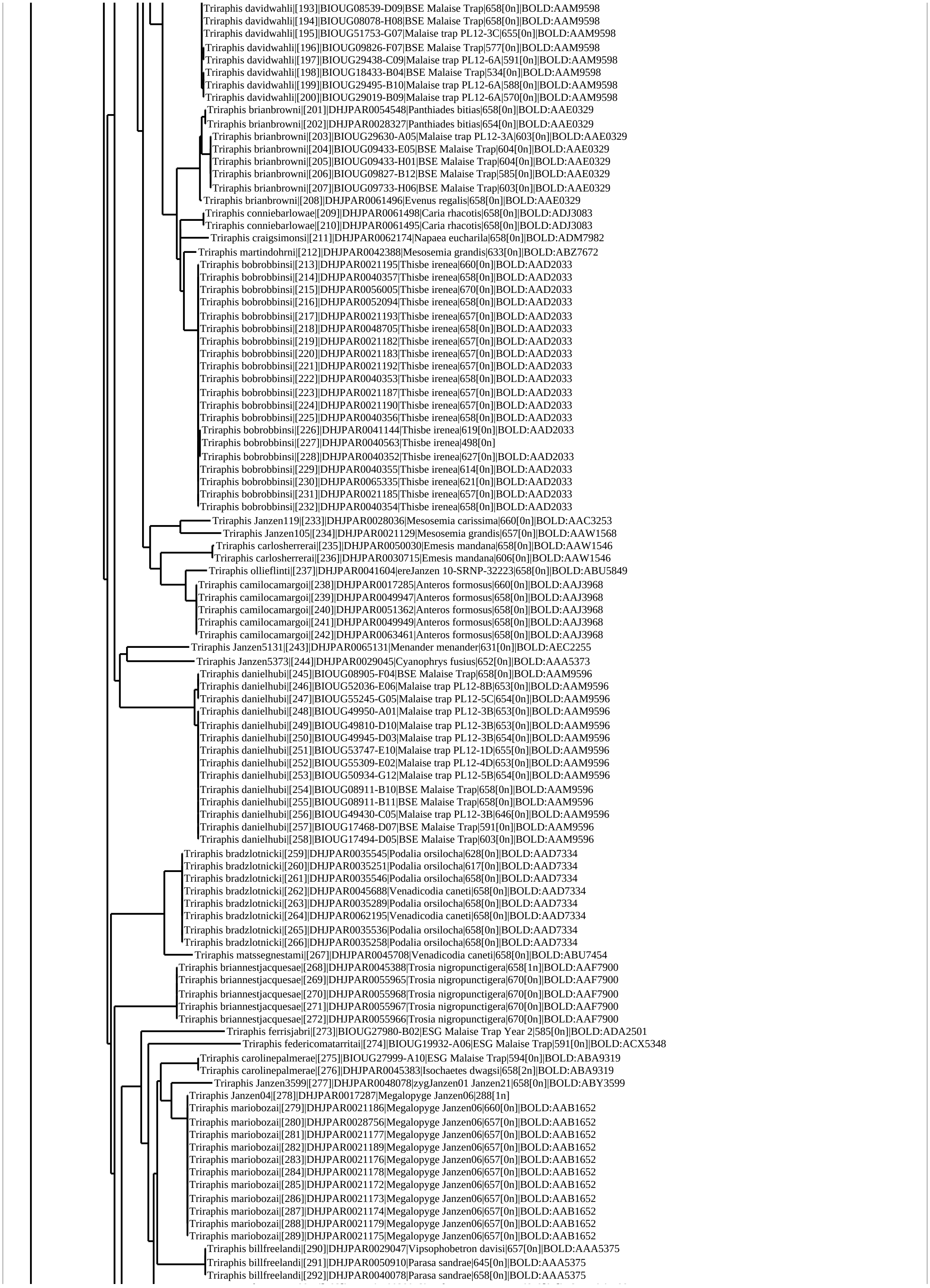
Triraphis davidwahli|[193]|BIOUG08539-D09|BSE Malaise Trap|658[0n]|BOLD:AAM9598
Triraphis davidwahli|[194]|BIOUG08078-H08|BSE Malaise Trap|658[0n]|BOLD:AAM9598
Triraphis davidwahli|[195]|BIOUG51753-G07|Malaise trap PL12-3C|655[0n]|BOLD:AAM9598
Triraphis davidwahli|[196]|BIOUG09826-F07|BSE Malaise Trap|577[0n]|BOLD:AAM9598
Triraphis davidwahli|[197]|BIOUG29438-C09|Malaise trap PL12-6A|591[0n]|BOLD:AAM9598
Triraphis davidwahli|[198]|BIOUG18433-B04|BSE Malaise Trap|534[0n]|BOLD:AAM9598
Triraphis davidwahli|[199]|BIOUG29495-B10|Malaise trap PL12-6A|588[0n]|BOLD:AAM9598
Triraphis davidwahli|[200]|BIOUG29019-B09|Malaise trap PL12-6A|570[0n]|BOLD:AAM9598
Triraphis brianbrowni|[201]|DHJPAR0054548|Panthiades bitias|658[0n]|BOLD:AAE0329
Triraphis brianbrowni|[202]|DHJPAR0028327|Panthiades bitias|654[0n]|BOLD:AAE0329
Triraphis brianbrowni|[203]|BIOUG29630-A05|Malaise trap PL12-3A|603[0n]|BOLD:AAE0329
Triraphis brianbrowni|[204]|BIOUG09433-E05|BSE Malaise Trap|604[0n]|BOLD:AAE0329
Triraphis brianbrowni|[205]|BIOUG09433-H01|BSE Malaise Trap|604[0n]|BOLD:AAE0329
Triraphis brianbrowni|[206]|BIOUG09827-B12|BSE Malaise Trap|585[0n]|BOLD:AAE0329
Triraphis brianbrowni|[207]|BIOUG09733-H06|BSE Malaise Trap|603[0n]|BOLD:AAE0329
Triraphis brianbrowni|[208]|DHJPAR0061496|Evenus regalis|658[0n]|BOLD:AAE0329
Triraphis conniebarlowae|[209]|DHJPAR0061498|Caria rhacotis|658[0n]|BOLD:ADJ3083
Triraphis conniebarlowae|[210]|DHJPAR0061495|Caria rhacotis|658[0n]|BOLD:ADJ3083
Triraphis craigsimonsi|[211]|DHJPAR0062174|Napaea eucharila|658[0n]|BOLD:ADM7982
Triraphis martindohrni|[212]|DHJPAR0042388|Mesosemia grandis|633[0n]|BOLD:ABZ7672
Triraphis bobrobbinsi|[213]|DHJPAR0021195|Thisbe irenea|660[0n]|BOLD:AAD2033
Triraphis bobrobbinsi|[214]|DHJPAR0040357|Thisbe irenea|658[0n]|BOLD:AAD2033
Triraphis bobrobbinsi|[215]|DHJPAR0056005|Thisbe irenea|670[0n]|BOLD:AAD2033
Triraphis bobrobbinsi|[216]|DHJPAR0052094|Thisbe irenea|658[0n]|BOLD:AAD2033
Triraphis bobrobbinsi|[217]|DHJPAR0021193|Thisbe irenea|657[0n]|BOLD:AAD2033
Triraphis bobrobbinsi|[218]|DHJPAR0048705|Thisbe irenea|658[0n]|BOLD:AAD2033
Triraphis bobrobbinsi|[219]|DHJPAR0021182|Thisbe irenea|657[0n]|BOLD:AAD2033
Triraphis bobrobbinsi|[220]|DHJPAR0021183|Thisbe irenea|657[0n]|BOLD:AAD2033
Triraphis bobrobbinsi|[221]|DHJPAR0021192|Thisbe irenea|657[0n]|BOLD:AAD2033
Triraphis bobrobbinsi|[222]|DHJPAR0040353|Thisbe irenea|658[0n]|BOLD:AAD2033
Triraphis bobrobbinsi|[223]|DHJPAR0021187|Thisbe irenea|657[0n]|BOLD:AAD2033
Triraphis bobrobbinsi|[224]|DHJPAR0021190|Thisbe irenea|657[0n]|BOLD:AAD2033
Triraphis bobrobbinsi|[225]|DHJPAR0040356|Thisbe irenea|658[0n]|BOLD:AAD2033
Triraphis bobrobbinsi|[226]|DHJPAR0041144|Thisbe irenea|619[0n]|BOLD:AAD2033
Triraphis bobrobbinsi|[227]|DHJPAR0040563|Thisbe irenea|498[0n]
Triraphis bobrobbinsi|[228]|DHJPAR0040352|Thisbe irenea|627[0n]|BOLD:AAD2033
Triraphis bobrobbinsi|[229]|DHJPAR0040355|Thisbe irenea|614[0n]|BOLD:AAD2033
Triraphis bobrobbinsi|[230]|DHJPAR0065335|Thisbe irenea|621[0n]|BOLD:AAD2033
Triraphis bobrobbinsi|[231]|DHJPAR0021185|Thisbe irenea|657[0n]|BOLD:AAD2033
Triraphis bobrobbinsi|[232]|DHJPAR0040354|Thisbe irenea|658[0n]|BOLD:AAD2033
Triraphis Janzen119|[233]|DHJPAR0028036|Mesosemia carissima|660[0n]|BOLD:AAC3253
Triraphis Janzen105|[234]|DHJPAR0021129|Mesosemia grandis|657[0n]|BOLD:AAW1568
Triraphis carlosherrerai|[235]|DHJPAR0050030|Emesis mandana|658[0n]|BOLD:AAW1546
Triraphis carlosherrerai|[236]|DHJPAR0030715|Emesis mandana|606[0n]|BOLD:AAW1546
Triraphis ollieflinti|[237]|DHJPAR0041604|ereJanzen 10-SRNP-32223|658[0n]|BOLD:ABU5849
Triraphis camilocamargoi|[238]|DHJPAR0017285|Anteros formosus|660[0n]|BOLD:AAJ3968
Triraphis camilocamargoi|[239]|DHJPAR0049947|Anteros formosus|658[0n]|BOLD:AAJ3968
Triraphis camilocamargoi|[240]|DHJPAR0051362|Anteros formosus|658[0n]|BOLD:AAJ3968
Triraphis camilocamargoi|[241]|DHJPAR0049949|Anteros formosus|658[0n]|BOLD:AAJ3968
Triraphis camilocamargoi|[242]|DHJPAR0063461|Anteros formosus|658[0n]|BOLD:AAJ3968
Triraphis Janzen5131|[243]|DHJPAR0065131|Menander menander|631[0n]|BOLD:AEC2255
Triraphis Janzen5373|[244]|DHJPAR0029045|Cyanophrys fusius|652[0n]|BOLD:AAA5373
Triraphis danielhubi|[245]|BIOUG08905-F04|BSE Malaise Trap|658[0n]|BOLD:AAM9596
Triraphis danielhubi|[246]|BIOUG52036-E06|Malaise trap PL12-8B|653[0n]|BOLD:AAM9596
Triraphis danielhubi|[247]|BIOUG55245-G05|Malaise trap PL12-5C|654[0n]|BOLD:AAM9596
Triraphis danielhubi|[248]|BIOUG49950-A01|Malaise trap PL12-3B|653[0n]|BOLD:AAM9596
Triraphis danielhubi|[249]|BIOUG49810-D10|Malaise trap PL12-3B|653[0n]|BOLD:AAM9596
Triraphis danielhubi|[250]|BIOUG49945-D03|Malaise trap PL12-3B|654[0n]|BOLD:AAM9596
Triraphis danielhubi|[251]|BIOUG53747-E10|Malaise trap PL12-1D|655[0n]|BOLD:AAM9596
Triraphis danielhubi|[252]|BIOUG55309-E02|Malaise trap PL12-4D|653[0n]|BOLD:AAM9596
Triraphis danielhubi|[253]|BIOUG50934-G12|Malaise trap PL12-5B|654[0n]|BOLD:AAM9596
Triraphis danielhubi|[254]|BIOUG08911-B10|BSE Malaise Trap|658[0n]|BOLD:AAM9596
Triraphis danielhubi|[255]|BIOUG08911-B11|BSE Malaise Trap|658[0n]|BOLD:AAM9596
Triraphis danielhubi|[256]|BIOUG49430-C05|Malaise trap PL12-3B|646[0n]|BOLD:AAM9596
Triraphis danielhubi|[257]|BIOUG17468-D07|BSE Malaise Trap|591[0n]|BOLD:AAM9596
Triraphis danielhubi|[258]|BIOUG17494-D05|BSE Malaise Trap|603[0n]|BOLD:AAM9596
Triraphis bradzlotnicki|[259]|DHJPAR0035545|Podalia orsilocha|628[0n]|BOLD:AAD7334
Triraphis bradzlotnicki|[260]|DHJPAR0035251|Podalia orsilocha|617[0n]|BOLD:AAD7334
Triraphis bradzlotnicki|[261]|DHJPAR0035546|Podalia orsilocha|658[0n]|BOLD:AAD7334
Triraphis bradzlotnicki|[262]|DHJPAR0045688|Venadicodia caneti|658[0n]|BOLD:AAD7334
Triraphis bradzlotnicki|[263]|DHJPAR0035289|Podalia orsilocha|658[0n]|BOLD:AAD7334
Triraphis bradzlotnicki|[264]|DHJPAR0062195|Venadicodia caneti|658[0n]|BOLD:AAD7334
Triraphis bradzlotnicki|[265]|DHJPAR0035536|Podalia orsilocha|658[0n]|BOLD:AAD7334
Triraphis bradzlotnicki|[266]|DHJPAR0035258|Podalia orsilocha|658[0n]|BOLD:AAD7334
Triraphis matssegnestami|[267]|DHJPAR0045708|Venadicodia caneti|658[0n]|BOLD:ABU7454
Triraphis briannestjacquesae|[268]|DHJPAR0045388|Trosia nigropunctigera|658[1n]|BOLD:AAF7900
Triraphis briannestjacquesae|[269]|DHJPAR0055965|Trosia nigropunctigera|670[0n]|BOLD:AAF7900
Triraphis briannestjacquesae|[270]|DHJPAR0055968|Trosia nigropunctigera|670[0n]|BOLD:AAF7900
Triraphis briannestjacquesae|[271]|DHJPAR0055967|Trosia nigropunctigera|670[0n]|BOLD:AAF7900
Triraphis briannestjacquesae|[272]|DHJPAR0055966|Trosia nigropunctigera|670[0n]|BOLD:AAF7900
Triraphis ferrisjabri|[273]|BIOUG27980-B02|ESG Malaise Trap Year 2|585[0n]|BOLD:ADA2501
Triraphis federicomatarritai|[274]|BIOUG19932-A06|ESG Malaise Trap|591[0n]|BOLD:ACX5348
Triraphis carolinepalmerae|[275]|BIOUG27999-A10|ESG Malaise Trap|594[0n]|BOLD:ABA9319
Triraphis carolinepalmerae|[276]|DHJPAR0045383|Isochaetes dwagsi|658[2n]|BOLD:ABA9319
Triraphis Janzen3599|[277]|DHJPAR0048078|zygJanzen01 Janzen21|658[0n]|BOLD:ABY3599
Triraphis Janzen04|[278]|DHJPAR0017287|Megalopyge Janzen06|288[1n]
Triraphis mariobozai|[279]|DHJPAR0021186|Megalopyge Janzen06|660[0n]|BOLD:AAB1652
Triraphis mariobozai|[280]|DHJPAR0028756|Megalopyge Janzen06|657[0n]|BOLD:AAB1652
Triraphis mariobozai|[281]|DHJPAR0021177|Megalopyge Janzen06|657[0n]|BOLD:AAB1652
Triraphis mariobozai|[282]|DHJPAR0021189|Megalopyge Janzen06|657[0n]|BOLD:AAB1652
Triraphis mariobozai|[283]|DHJPAR0021176|Megalopyge Janzen06|657[0n]|BOLD:AAB1652
Triraphis mariobozai|[284]|DHJPAR0021178|Megalopyge Janzen06|657[0n]|BOLD:AAB1652
Triraphis mariobozai|[285]|DHJPAR0021172|Megalopyge Janzen06|657[0n]|BOLD:AAB1652
Triraphis mariobozai|[286]|DHJPAR0021173|Megalopyge Janzen06|657[0n]|BOLD:AAB1652
Triraphis mariobozai|[287]|DHJPAR0021174|Megalopyge Janzen06|657[0n]|BOLD:AAB1652
Triraphis mariobozai|[288]|DHJPAR0021179|Megalopyge Janzen06|657[0n]|BOLD:AAB1652
Triraphis mariobozai|[289]|DHJPAR0021175|Megalopyge Janzen06|657[0n]|BOLD:AAB1652
Triraphis billfreelandi|[290]|DHJPAR0029047|Vipsophobetron davisi|657[0n]|BOLD:AAA5375
Triraphis billfreelandi|[291]|DHJPAR0050910|Parasa sandrae|645[0n]|BOLD:AAA5375
Triraphis billfreelandi|[292]|DHJPAR0040078|Parasa sandrae|658[0n]|BOLD:AAA5375

Triraphis billfreelandi|[290]|DHJPAR0029047|Vipsophobetron davisi|657[0n]|BOLD:AAA5375
Triraphis billfreelandi|[291]|DHJPAR0050910|Parasa sandrae|645[0n]|BOLD:AAA5375
Triraphis billfreelandi|[292]|DHJPAR0040078|Parasa sandrae|658[0n]|BOLD:AAA5375
Triraphis Janzen8815|[293]|DHJPAR0038023|Euclea mesoamericana|658[0n]|BOLD:AAH8815
Triraphis billmclarneyi|[294]|DHJPAR0036311|zygjanzen01 Janzen23|658[0n]|BOLD:AAA7065
Triraphis billmclarneyi|[295]|DHJPAR0036310|zygjanzen01 Janzen23|658[0n]|BOLD:AAA7065
Triraphis billmclarneyi|[296]|DHJPAR0030595|zygjanzen01 Janzen23|630[1n]|BOLD:AAA7065
Triraphis billmclarneyi|[297]|DHJPAR0030597|zygjanzen01 Janzen23|658[0n]|BOLD:AAA7065
Triraphis billmclarneyi|[298]|DHJPAR0030594|zygjanzen01 Janzen23|658[0n]|BOLD:AAA7065
Triraphis billmclarneyi|[299]|DHJPAR0030592|zygjanzen01 Janzen23|658[1n]|BOLD:AAA7065
Triraphis billmclarneyi|[300]|DHJPAR0030596|zygjanzen01 Janzen23|658[0n]|BOLD:AAA7065
Triraphis billmclarneyi|[301]|DHJPAR0037873|zygjanzen01 Janzen23|658[0n]|BOLD:AAA7065
Triraphis billmclarneyi|[302]|DHJPAR0030598|zygjanzen01 Janzen23|633[0n]|BOLD:AAA7065
Triraphis billripplei|[303]|DHJPAR0021201|Norape Janzen03|660[0n]|BOLD:AAB1658
Triraphis billripplei|[304]|DHJPAR0028330|Megalopygidae 98-SRNP-3934|576[0n]|BOLD:AAB1658
Triraphis billripplei|[305]|DHJPAR0036305|Norape Janzend03|658[0n]|BOLD:AAB1658
Triraphis billripplei|[306]|DHJPAR0028329|Megalopygidae 98-SRNP-3934|654[0n]|BOLD:AAB1658
Triraphis billripplei|[307]|BIOUG28640-H06|Malaise trap PL12-7A|591[0n]|BOLD:AAB1658
Triraphis christerhanssoni|[308]|DHJPAR0058770|megaJanzen01 98-SRNP-3934|658[0n]|BOLD:ADB1219
Triraphis christerhanssoni|[309]|DHJPAR0058774|megaJanzen01 98-SRNP-3934|658[0n]|BOLD:ADB1219
Triraphis christerhanssoni|[310]|DHJPAR0058772|megaJanzen01 98-SRNP-3934|658[0n]|BOLD:ADB1219
Triraphis christerhanssoni|[311]|DHJPAR0058773|megaJanzen01 98-SRNP-3934|658[0n]|BOLD:ADB1219
Triraphis christerhanssoni|[312]|DHJPAR0058775|megaJanzen01 98-SRNP-3934|658[0n]|BOLD:ADB1219
Triraphis christerhanssoni|[313]|DHJPAR0058771|megaJanzen01 98-SRNP-3934|658[0n]|BOLD:ADB1219
Triraphis defectus|[314]|DHJPAR0029046|Vipsania rosabella|657[0n]|BOLD:AAA5374
Triraphis defectus|[315]|BIOUG05386-B06|BSE Malaise Trap|658[0n]|BOLD:AAA5374
Triraphis defectus|[316]|BIOUG05279-H01|BSE Malaise Trap|658[0n]|BOLD:AAA5374
Triraphis defectus|[317]|DHJPAR0021194|Parasa wellesca|657[0n]|BOLD:AAA5374
Triraphis christhompsoni|[318]|DHJPAR0029048|Pampa Janzen04|657[0n]|BOLD:ACK7801
Triraphis christhompsoni|[319]|DHJPAR0048107|Pampa Janzen04|658[0n]|BOLD:ACK7801
Triraphis christhompsoni|[320]|DHJPAR0064009|Pampa Janzen04|629[0n]|BOLD:ACK7801
Triraphis christhompsoni|[321]|DHJPAR0029064|Pampa Janzen04|657[0n]|BOLD:ACK7801
Triraphis christhompsoni|[322]|DHJPAR0065330|Harrisinia Janzen18|658[0n]|BOLD:ACK7801
Triraphis christhompsoni|[323]|DHJPAR0045818|Pampa Janzen04|658[0n]|BOLD:ACK7801
Triraphis christhompsoni|[324]|DHJPAR0059698|Pampa Janzen04|658[0n]|BOLD:ACK7801
Triraphis christhompsoni|[325]|DHJPAR0065339|Harrisinia Janzen18|647[0n]|BOLD:ACK7801
Triraphis christhompsoni|[326]|DHJPAR0050069|Pampa Janzen04|605[0n]|BOLD:ACK7801
Triraphis christhompsoni|[327]|DHJPAR0029059|Pampa Janzen04|351[8n]|BOLD:ACK7801
Triraphis bobandersoni|[328]|DHJPAR0064450|Epiperola vaferella|658[0n]|BOLD:AEB4766
Triraphis Janzen02|[329]|DHJPAR0063968|Epiperola vaferella|658[0n]|BOLD:AEB4766
Triraphis bobandersoni|[330]|DHJPAR0023701|Epiperola vaferella|645[0n]|BOLD:AAB8975
Triraphis bobandersoni|[331]|DHJPAR0023533|Epiperola vaferella|629[0n]|BOLD:AAB8975
Triraphis bobandersoni|[332]|DHJPAR0023698|Epiperola vaferella|629[0n]|BOLD:AAB8975
Triraphis bobandersoni|[333]|DHJPAR0023722|Epiperola vaferella|629[0n]|BOLD:AAB8975
Triraphis bobandersoni|[334]|DHJPAR0023694|Epiperola vaferella|629[0n]|BOLD:AAB8975
Triraphis bobandersoni|[335]|DHJPAR0023690|Epiperola vaferella|642[0n]|BOLD:AAB8975
Triraphis bobandersoni|[336]|DHJPAR0023704|Epiperola vaferella|645[0n]|BOLD:AAB8975
Triraphis bobandersoni|[337]|DHJPAR0023716|Epiperola vaferella|645[0n]|BOLD:AAB8975
Triraphis bobandersoni|[338]|DHJPAR0038384|Epiperola vaferella|658[0n]|BOLD:AAB8975
Triraphis bobandersoni|[339]|DHJPAR0023532|Epiperola vaferella|657[0n]|BOLD:AAB8975
Triraphis bobandersoni|[340]|DHJPAR0038385|Epiperola vaferella|658[0n]|BOLD:AAB8975
Triraphis bobandersoni|[341]|DHJPAR0051666|Epiperola vaferella|658[0n]|BOLD:AAB8975
Triraphis bobandersoni|[342]|DHJPAR0037964|Epiperola vaferella|658[0n]|BOLD:AAB8975
Triraphis bobandersoni|[343]|DHJPAR0023689|Epiperola vaferella|635[0n]|BOLD:AAB8975
Triraphis bobandersoni|[344]|DHJPAR0023519|Epiperola vaferella|635[0n]|BOLD:AAB8975
Triraphis bobandersoni|[345]|DHJPAR0030619|Epiperola vaferella|638[0n]|BOLD:AAB8975
Triraphis bobandersoni|[346]|DHJPAR0023702|Epiperola vaferella|630[0n]|BOLD:AAB8975
Triraphis bobandersoni|[347]|DHJPAR0037970|Epiperola vaferella|658[0n]|BOLD:AAB8975
Triraphis bobandersoni|[348]|DHJPAR0023527|Epiperola vaferella|657[0n]|BOLD:AAB8975
Triraphis bobandersoni|[349]|DHJPAR0037969|Epiperola vaferella|658[0n]|BOLD:AAB8975
Triraphis bobandersoni|[350]|DHJPAR0051315|Epiperola vaferella|658[0n]|BOLD:AAB8975
Triraphis bobandersoni|[351]|DHJPAR0037973|Epiperola vaferella|658[0n]|BOLD:AAB8975
Triraphis bobandersoni|[352]|DHJPAR0037972|Epiperola vaferella|658[0n]|BOLD:AAB8975
Triraphis bobandersoni|[353]|DHJPAR0038386|Epiperola vaferella|658[0n]|BOLD:AAB8975
Triraphis bobandersoni|[354]|DHJPAR0017286|Epiperola vaferella|657[0n]|BOLD:AAB8975
Triraphis bobandersoni|[355]|DHJPAR0023518|Epiperola vaferella|631[0n]|BOLD:AAB8975
Rogadinae|[356]|BIOUG56035-E07|Malaise trap PL12-3D|652[0n]|BOLD:AAH8697
Heterogamus donstonei|[357]|BIOUG51263-E01|Malaise trap PL12-8A|640[0n]|BOLD:AAH8697
Heterogamus donstonei|[358]|BIOUG50161-D02|Malaise trap PL12-5A|654[0n]|BOLD:AAH8697
Rogadinae|[359]|BIOUG52713-D09|Malaise trap PL12-9C|652[0n]|BOLD:AAH8697
Rogadinae|[360]|BIOUG53747-A04|Malaise trap PL12-1D|655[0n]|BOLD:AAH8697
Rogadinae|[361]|BIOUG55890-F12|Malaise trap PL12-1D|651[0n]|BOLD:AAH8697
Rogadinae|[362]|BIOUG55545-C11|Malaise trap PL12-6D|654[0n]|BOLD:AAH8697
Rogadinae|[363]|BIOUG55228-A09|Malaise trap PL12-4D|654[0n]|BOLD:AAH8697
Rogadinae|[364]|BIOUG55308-B04|Malaise trap PL12-4D|654[0n]|BOLD:AAH8697
Rogadinae|[365]|BIOUG53747-A03|Malaise trap PL12-1D|655[0n]|BOLD:AAH8697
Rogadinae|[366]|BIOUG55886-H10|Malaise trap PL12-1D|653[0n]|BOLD:AAH8697
Heterogamus donstonei|[367]|BIOUG50155-F08|Malaise trap PL12-5A|654[0n]|BOLD:AAH8697
Rogadinae|[368]|BIOUG55308-E08|Malaise trap PL12-4D|654[0n]|BOLD:AAH8697
Rogadinae|[369]|BIOUG54626-A01|Malaise trap PL12-4D|654[0n]|BOLD:AAH8697
Heterogamus donstonei|[370]|BIOUG50698-B07|Malaise trap PL12-5A|658[0n]|BOLD:AAH8697
Heterogamus donstonei|[371]|BIOUG29392-G08|Malaise trap PL12-9A|576[0n]|BOLD:AAH8697
Heterogamus donstonei|[372]|BIOUG30941-F01|Malaise trap PL12-2A|591[0n]|BOLD:AAH8697
Heterogamus donstonei|[373]|BIOUG29239-A07|Malaise trap PL12-4A|588[0n]|BOLD:AAH8697
Heterogamus donstonei|[374]|BIOUG29390-H04|Malaise trap PL12-9A|589[0n]|BOLD:AAH8697
Heterogamus donstonei|[375]|BIOUG29970-B01|Malaise trap PL12-3A|588[0n]|BOLD:AAH8697
Heterogamus donstonei|[376]|BIOUG29499-D11|Malaise trap PL12-6A|567[0n]|BOLD:AAH8697
Heterogamus donstonei|[377]|BIOUG29958-B04|Malaise trap PL12-3A|576[0n]|BOLD:AAH8697
Heterogamus donstonei|[378]|BIOUG28659-B04|Malaise trap PL12-1A|591[0n]|BOLD:AAH8697
Aleiodes alessandracallejae|[379]|BIOUG52931-E12|Malaise trap PL12-3C|651[0n]|BOLD:ACJ2417
Aleiodes alessandracallejae|[380]|BIOUG53027-C10|Malaise trap PL12-3C|640[0n]|BOLD:ACJ2417
Aleiodes alessandracallejae|[381]|BIOUG53172-A01|Malaise trap PL12-3C|647[0n]|BOLD:ACJ2417
Aleiodes alessandracallejae|[382]|BIOUG53336-B05|Malaise trap PL12-1C|650[0n]|BOLD:ACJ2417
Aleiodes alessandracallejae|[383]|BIOUG46743-F10|Malaise trap PL12-4B|655[0n]|BOLD:ACJ2417
Aleiodes alessandracallejae|[384]|BIOUG52065-H07|Malaise trap PL12-8B|650[0n]|BOLD:ACJ2417
Aleiodes alessandracallejae|[385]|BIOUG29728-B07|Malaise trap PL12-3A|555[2n]|BOLD:ACJ2417
Aleiodes alessandracallejae|[386]|BIOUG55809-H07|Malaise trap PL12-1D|640[0n]|BOLD:ACJ2417
Aleiodes alessandracallejae|[387]|BIOUG54606-D03|Malaise trap PL12-3D|644[0n]|BOLD:ACJ2417
Aleiodes alessandracallejae|[388]|BIOUG53265-D02|Malaise trap PL12-3C|652[0n]|BOLD:ACJ2417
Aleiodes alessandracallejae|[389]|BIOUG54079-F12|Malaise trap PL12-2C|640[0n]|BOLD:ACJ2417
Aleiodes alessandracallejae|[390]|BIOUG51524-G03|Malaise trap PL12-3C|647[0n]|BOLD:ACJ2417

[illegible]
Aleiodes alessandracallejae|[388]|BIOUG53265-D02|Malaise trap PL12-3C|652[0n]|BOLD:ACJ2417
Aleiodes alessandracallejae|[389]|BIOUG54079-F12|Malaise trap PL12-2C|640[0n]|BOLD:ACJ2417
Aleiodes alessandracallejae|[390]|BIOUG51524-G03|Malaise trap PL12-3C|647[0n]|BOLD:ACJ2417
Aleiodes alessandracallejae|[391]|BIOUG30963-C06|Malaise trap PL12-2A|555[2n]|BOLD:ACJ2417
Aleiodes alessandracallejae|[392]|BIOUG07616-F05|BSE Malaise Trap|565[0n]|BOLD:ACJ2417
Aleiodes alessandracallejae|[393]|BIOUG54771-F08|Malaise trap PL12-8C|640[0n]|BOLD:ACJ2417
Aleiodes alessandracallejae|[394]|BIOUG52292-B11|Malaise trap PL12-3C|653[0n]|BOLD:ACJ2417
Aleiodes alessandracallejae|[395]|BIOUG55013-F12|Malaise trap PL12-8C|653[0n]|BOLD:ACJ2417
Aleiodes alessandracallejae|[396]|BIOUG55653-B08|Malaise trap PL12-1D|652[0n]|BOLD:ACJ2417
Aleiodes alessandracallejae|[397]|BIOUG56134-D04|Malaise trap PL12-6D|654[0n]|BOLD:ACJ2417
Aleiodes alessandracallejae|[398]|BIOUG55656-C09|Malaise trap PL12-1D|654[0n]|BOLD:ACJ2417
Aleiodes alessandracallejae|[399]|BIOUG44108-C05|Malaise trap PL12-3B|654[0n]|BOLD:ACJ2417
Aleiodes alessandracallejae|[400]|BIOUG52308-C03|Malaise trap PL12-3C|653[0n]|BOLD:ACJ2417
Aleiodes alessandracallejae|[401]|BIOUG44876-F03|Malaise trap PL12-1B|653[0n]|BOLD:ACJ2417
Aleiodes alessandracallejae|[402]|BIOUG51467-D04|Malaise trap PL12-2B|653[0n]|BOLD:ACJ2417
Aleiodes alessandracallejae|[403]|BIOUG53030-H03|Malaise trap PL12-3C|655[0n]|BOLD:ACJ2417
Aleiodes alessandracallejae|[404]|BIOUG52598-G02|Malaise trap PL12-3C|652[0n]|BOLD:ACJ2417
Aleiodes alessandracallejae|[405]|BIOUG44782-D07|Malaise trap PL12-1B|653[0n]|BOLD:ACJ2417
Aleiodes alessandracallejae|[406]|BIOUG52070-F05|Malaise trap PL12-8B|651[0n]|BOLD:ACJ2417
Aleiodes alessandracallejae|[407]|BIOUG53600-B05|Malaise trap PL12-6C|651[0n]|BOLD:ACJ2417
Aleiodes alessandracallejae|[408]|BIOUG53172-C02|Malaise trap PL12-3C|652[0n]|BOLD:ACJ2417
Aleiodes alessandracallejae|[409]|BIOUG52564-H11|Malaise trap PL12-3C|655[0n]|BOLD:ACJ2417
Aleiodes alessandracallejae|[410]|BIOUG52400-G02|Malaise trap PL12-3C|653[0n]|BOLD:ACJ2417
Aleiodes alessandracallejae|[411]|BIOUG53172-H06|Malaise trap PL12-3C|652[0n]|BOLD:ACJ2417
Aleiodes alessandracallejae|[412]|BIOUG44782-C11|Malaise trap PL12-1B|654[0n]|BOLD:ACJ2417
Aleiodes alessandracallejae|[413]|BIOUG52291-C01|Malaise trap PL12-3C|655[0n]|BOLD:ACJ2417
Aleiodes alessandracallejae|[414]|BIOUG53245-A10|Malaise trap PL12-1C|651[0n]|BOLD:ACJ2417
Aleiodes alessandracallejae|[415]|BIOUG53143-F11|Malaise trap PL12-9C|651[0n]|BOLD:ACJ2417
Aleiodes alessandracallejae|[416]|BIOUG51936-C05|Malaise trap PL12-8B|651[0n]|BOLD:ACJ2417
Aleiodes alessandracallejae|[417]|BIOUG46567-G05|Malaise trap PL12-6B|653[0n]|BOLD:ACJ2417
Aleiodes alessandracallejae|[418]|BIOUG52291-F04|Malaise trap PL12-3C|652[0n]|BOLD:ACJ2417
Aleiodes alessandracallejae|[419]|BIOUG44686-F03|Malaise trap PL12-3B|654[0n]|BOLD:ACJ2417
Aleiodes alessandracallejae|[420]|BIOUG53172-B04|Malaise trap PL12-3C|656[0n]|BOLD:ACJ2417
Aleiodes alessandracallejae|[421]|BIOUG44838-E03|Malaise trap PL12-1B|653[0n]|BOLD:ACJ2417
Aleiodes alessandracallejae|[422]|BIOUG56102-A11|Malaise trap PL12-4D|654[0n]|BOLD:ACJ2417
Aleiodes alessandracallejae|[423]|BIOUG52338-H06|Malaise trap PL12-3C|652[0n]|BOLD:ACJ2417
Aleiodes alessandracallejae|[424]|BIOUG52437-G05|Malaise trap PL12-3C|653[0n]|BOLD:ACJ2417
Aleiodes alessandracallejae|[425]|BIOUG52468-B10|Malaise trap PL12-3C|653[0n]|BOLD:ACJ2417
Aleiodes alessandracallejae|[426]|BIOUG44027-C01|Malaise trap PL12-3B|652[0n]|BOLD:ACJ2417
Aleiodes alessandracallejae|[427]|BIOUG53029-D11|Malaise trap PL12-3C|651[0n]|BOLD:ACJ2417
Aleiodes alessandracallejae|[428]|BIOUG44173-C07|Malaise trap PL12-3B|654[0n]|BOLD:ACJ2417
Aleiodes alessandracallejae|[429]|BIOUG48852-E07|Malaise trap PL12-5A|654[0n]|BOLD:ACJ2417
Aleiodes alessandracallejae|[430]|BIOUG56122-C02|Malaise trap PL12-6D|654[0n]|BOLD:ACJ2417
Aleiodes alessandracallejae|[431]|BIOUG52403-A11|Malaise trap PL12-3C|653[0n]|BOLD:ACJ2417
Aleiodes alessandracallejae|[432]|BIOUG44762-E04|Malaise trap PL12-3B|654[0n]|BOLD:ACJ2417
Aleiodes alessandracallejae|[433]|BIOUG53172-B01|Malaise trap PL12-3C|652[0n]|BOLD:ACJ2417
Aleiodes alessandracallejae|[434]|BIOUG54503-A12|Malaise trap PL12-5C|653[0n]|BOLD:ACJ2417
Aleiodes alessandracallejae|[435]|BIOUG46641-H04|Malaise trap PL12-6B|654[0n]|BOLD:ACJ2417
Aleiodes alessandracallejae|[436]|BIOUG44775-D05|Malaise trap PL12-3B|654[0n]|BOLD:ACJ2417
Aleiodes alessandracallejae|[437]|BIOUG51829-H05|Malaise trap PL12-7B|653[0n]|BOLD:ACJ2417
Aleiodes alessandracallejae|[438]|BIOUG44761-E12|Malaise trap PL12-3B|654[0n]|BOLD:ACJ2417
Aleiodes alessandracallejae|[439]|BIOUG51525-A11|Malaise trap PL12-3C|652[0n]|BOLD:ACJ2417
Aleiodes alessandracallejae|[440]|BIOUG52309-D03|Malaise trap PL12-3C|654[0n]|BOLD:ACJ2417
Aleiodes alessandracallejae|[441]|BIOUG57205-F11|Malaise trap PL12-2D|654[0n]|BOLD:ACJ2417
Aleiodes alessandracallejae|[442]|BIOUG52931-G02|Malaise trap PL12-3C|651[0n]|BOLD:ACJ2417
Aleiodes alessandracallejae|[443]|BIOUG52992-D10|Malaise trap PL12-3C|651[0n]|BOLD:ACJ2417
Aleiodes alessandracallejae|[444]|BIOUG53264-D08|Malaise trap PL12-3C|652[0n]|BOLD:ACJ2417
Aleiodes alessandracallejae|[445]|BIOUG53600-C01|Malaise trap PL12-6C|654[0n]|BOLD:ACJ2417
Aleiodes alessandracallejae|[446]|BIOUG53616-E10|Malaise trap PL12-3C|653[0n]|BOLD:ACJ2417
Aleiodes alessandracallejae|[447]|BIOUG55508-E12|Malaise trap PL12-6D|653[0n]|BOLD:ACJ2417
Aleiodes alessandracallejae|[448]|BIOUG51829-G07|Malaise trap PL12-7B|652[0n]|BOLD:ACJ2417
Aleiodes alessandracallejae|[449]|BIOUG54606-C06|Malaise trap PL12-3D|651[0n]|BOLD:ACJ2417
Aleiodes alessandracallejae|[450]|BIOUG51525-F11|Malaise trap PL12-3C|652[0n]|BOLD:ACJ2417
Aleiodes alessandracallejae|[451]|BIOUG52292-H08|Malaise trap PL12-3C|653[0n]|BOLD:ACJ2417
Aleiodes alessandracallejae|[452]|BIOUG53616-H04|Malaise trap PL12-3C|652[0n]|BOLD:ACJ2417
Aleiodes alessandracallejae|[453]|BIOUG52406-D12|Malaise trap PL12-3C|652[0n]|BOLD:ACJ2417
Aleiodes alessandracallejae|[454]|BIOUG55809-H08|Malaise trap PL12-1D|672[0n]|BOLD:ACJ2417
Aleiodes alessandracallejae|[455]|BIOUG09740-D04|BSE Malaise Trap|658[0n]|BOLD:ACJ2417
Aleiodes alessandracallejae|[456]|BIOUG55653-A12|Malaise trap PL12-1D|663[0n]|BOLD:ACJ2417
Aleiodes alessandracallejae|[457]|BIOUG51631-H03|Malaise trap PL12-2B|643[0n]|BOLD:ACJ2417
Aleiodes alessandracallejae|[458]|BIOUG55013-G10|Malaise trap PL12-8C|655[0n]|BOLD:ACJ2417
Aleiodes alessandracallejae|[459]|BIOUG53265-C11|Malaise trap PL12-3C|641[0n]|BOLD:ACJ2417
Aleiodes alessandracallejae|[460]|BIOUG51523-H11|Malaise trap PL12-3C|640[0n]|BOLD:ACJ2417
Aleiodes alessandracallejae|[461]|BIOUG53264-D12|Malaise trap PL12-3C|654[0n]|BOLD:ACJ2417
Aleiodes alessandracallejae|[462]|BIOUG52602-G04|Malaise trap PL12-3C|655[0n]|BOLD:ACJ2417
Aleiodes alessandracallejae|[463]|BIOUG52337-C10|Malaise trap PL12-3C|655[0n]|BOLD:ACJ2417
Aleiodes alessandracallejae|[464]|BIOUG52066-H06|Malaise trap PL12-8B|654[0n]|BOLD:ACJ2417
Aleiodes alessandracallejae|[465]|BIOUG53435-D12|Malaise trap PL12-1C|652[0n]|BOLD:ACJ2417
Aleiodes alessandracallejae|[466]|BIOUG52457-C05|Malaise trap PL12-3C|653[0n]|BOLD:ACJ2417
Aleiodes alessandracallejae|[467]|BIOUG57205-B04|Malaise trap PL12-2D|654[0n]|BOLD:ACJ2417
Aleiodes alessandracallejae|[468]|BIOUG48852-E06|Malaise trap PL12-5A|652[0n]|BOLD:ACJ2417
Aleiodes alessandracallejae|[469]|BIOUG53029-C12|Malaise trap PL12-3C|652[0n]|BOLD:ACJ2417
Aleiodes alessandracallejae|[470]|BIOUG52368-A05|Malaise trap PL12-3C|654[0n]|BOLD:ACJ2417
Aleiodes alessandracallejae|[471]|BIOUG54960-C01|Malaise trap PL12-3D|657[0n]|BOLD:ACJ2417
Aleiodes alessandracallejae|[472]|BIOUG53435-A06|Malaise trap PL12-1C|650[0n]|BOLD:ACJ2417
Aleiodes alessandracallejae|[473]|BIOUG29883-H11|Malaise trap PL12-3A|534[0n]|BOLD:ACJ2417
Aleiodes alessandracallejae|[474]|BIOUG51524-G10|Malaise trap PL12-3C|640[0n]|BOLD:ACJ2417
Aleiodes alessandracallejae|[475]|BIOUG52536-H10|Malaise trap PL12-3C|645[0n]|BOLD:ACJ2417
Aleiodes alessandracallejae|[476]|BIOUG52367-C08|Malaise trap PL12-3C|655[0n]|BOLD:ACJ2417
Aleiodes alessandracallejae|[477]|BIOUG44872-E06|Malaise trap PL12-1B|654[0n]|BOLD:ACJ2417
Aleiodes alessandracallejae|[478]|BIOUG53811-A03|Malaise trap PL12-7C|652[0n]|BOLD:ACJ2417
Aleiodes alessandracallejae|[479]|BIOUG52308-G10|Malaise trap PL12-3C|654[0n]|BOLD:ACJ2417
Aleiodes alessandracallejae|[480]|BIOUG46562-B09|Malaise trap PL12-6B|654[0n]|BOLD:ACJ2417
Aleiodes alessandracallejae|[481]|BIOUG54387-C06|Malaise trap PL12-3D|653[0n]|BOLD:ACJ2417
Aleiodes alessandracallejae|[482]|BIOUG52597-E01|Malaise trap PL12-3C|657[0n]|BOLD:ACJ2417
Aleiodes alessandracallejae|[483]|BIOUG55084-G02|Malaise trap PL12-1D|658[0n]|BOLD:ACJ2417
Aleiodes alessandracallejae|[484]|BIOUG29862-A04|Malaise trap PL12-3A|576[0n]|BOLD:ACJ2417
Aleiodes alessandracallejae|[485]|BIOUG29863-G04|Malaise trap PL12-3A|585[0n]|BOLD:ACJ2417
Aleiodes alessandracallejae|[486]|BIOUG29836-H03|Malaise trap PL12-3A|588[0n]|BOLD:ACJ2417
Aleiodes alessandracallejae|[487]|BIOUG29879-F03|Malaise trap PL12-3A|585[0n]|BOLD:ACJ2417

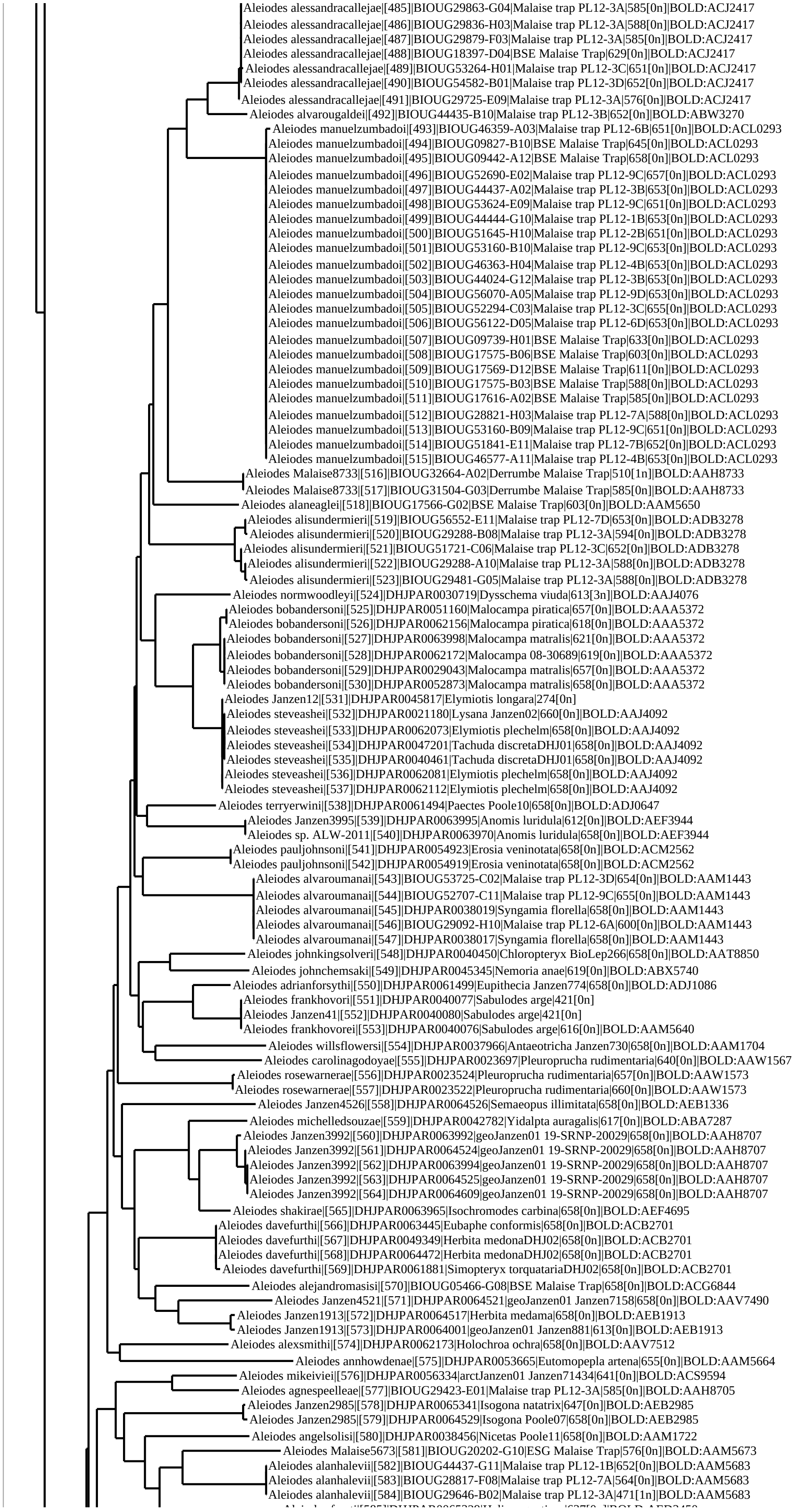
Aleiodes alessandracallejae|[485]|BIOUG29863-G04|Malaise trap PL12-3A|585[0n]|BOLD:ACJ2417
Aleiodes alessandracallejae|[486]|BIOUG29836-H03|Malaise trap PL12-3A|588[0n]|BOLD:ACJ2417
Aleiodes alessandracallejae|[487]|BIOUG29879-F03|Malaise trap PL12-3A|585[0n]|BOLD:ACJ2417
Aleiodes alessandracallejae|[488]|BIOUG18397-D04|BSE Malaise Trap|629[0n]|BOLD:ACJ2417
Aleiodes alessandracallejae|[489]|BIOUG53264-H01|Malaise trap PL12-3C|651[0n]|BOLD:ACJ2417
Aleiodes alessandracallejae|[490]|BIOUG54582-B01|Malaise trap PL12-3D|652[0n]|BOLD:ACJ2417
Aleiodes alessandracallejae|[491]|BIOUG29725-E09|Malaise trap PL12-3A|576[0n]|BOLD:ACJ2417
Aleiodes alvarougaldei|[492]|BIOUG44435-B10|Malaise trap PL12-3B|652[0n]|BOLD:ABW3270
Aleiodes manuelzumbadoi|[493]|BIOUG46359-A03|Malaise trap PL12-6B|651[0n]|BOLD:ACL0293
Aleiodes manuelzumbadoi|[494]|BIOUG09827-B10|BSE Malaise Trap|645[0n]|BOLD:ACL0293
Aleiodes manuelzumbadoi|[495]|BIOUG09442-A12|BSE Malaise Trap|658[0n]|BOLD:ACL0293
Aleiodes manuelzumbadoi|[496]|BIOUG52690-E02|Malaise trap PL12-9C|657[0n]|BOLD:ACL0293
Aleiodes manuelzumbadoi|[497]|BIOUG44437-A02|Malaise trap PL12-3B|653[0n]|BOLD:ACL0293
Aleiodes manuelzumbadoi|[498]|BIOUG53624-E09|Malaise trap PL12-9C|651[0n]|BOLD:ACL0293
Aleiodes manuelzumbadoi|[499]|BIOUG44444-G10|Malaise trap PL12-1B|653[0n]|BOLD:ACL0293
Aleiodes manuelzumbadoi|[500]|BIOUG51645-H10|Malaise trap PL12-2B|651[0n]|BOLD:ACL0293
Aleiodes manuelzumbadoi|[501]|BIOUG53160-B10|Malaise trap PL12-9C|653[0n]|BOLD:ACL0293
Aleiodes manuelzumbadoi|[502]|BIOUG46363-H04|Malaise trap PL12-4B|653[0n]|BOLD:ACL0293
Aleiodes manuelzumbadoi|[503]|BIOUG44024-G12|Malaise trap PL12-3B|653[0n]|BOLD:ACL0293
Aleiodes manuelzumbadoi|[504]|BIOUG56070-A05|Malaise trap PL12-9D|653[0n]|BOLD:ACL0293
Aleiodes manuelzumbadoi|[505]|BIOUG52294-C03|Malaise trap PL12-3C|655[0n]|BOLD:ACL0293
Aleiodes manuelzumbadoi|[506]|BIOUG56122-D05|Malaise trap PL12-6D|653[0n]|BOLD:ACL0293
Aleiodes manuelzumbadoi|[507]|BIOUG09739-H01|BSE Malaise Trap|633[0n]|BOLD:ACL0293
Aleiodes manuelzumbadoi|[508]|BIOUG17575-B06|BSE Malaise Trap|603[0n]|BOLD:ACL0293
Aleiodes manuelzumbadoi|[509]|BIOUG17569-D12|BSE Malaise Trap|611[0n]|BOLD:ACL0293
Aleiodes manuelzumbadoi|[510]|BIOUG17575-B03|BSE Malaise Trap|588[0n]|BOLD:ACL0293
Aleiodes manuelzumbadoi|[511]|BIOUG17616-A02|BSE Malaise Trap|585[0n]|BOLD:ACL0293
Aleiodes manuelzumbadoi|[512]|BIOUG28821-H03|Malaise trap PL12-7A|588[0n]|BOLD:ACL0293
Aleiodes manuelzumbadoi|[513]|BIOUG53160-B09|Malaise trap PL12-9C|651[0n]|BOLD:ACL0293
Aleiodes manuelzumbadoi|[514]|BIOUG51841-E11|Malaise trap PL12-7B|652[0n]|BOLD:ACL0293
Aleiodes manuelzumbadoi|[515]|BIOUG46577-A11|Malaise trap PL12-4B|653[0n]|BOLD:ACL0293
Aleiodes Malaise8733|[516]|BIOUG32664-A02|Derrumbe Malaise Trap|510[1n]|BOLD:AAH8733
Aleiodes Malaise8733|[517]|BIOUG31504-G03|Derrumbe Malaise Trap|585[0n]|BOLD:AAH8733
Aleiodes alaneaglei|[518]|BIOUG17566-G02|BSE Malaise Trap|603[0n]|BOLD:AAM5650
Aleiodes alisundermieri|[519]|BIOUG56552-E11|Malaise trap PL12-7D|653[0n]|BOLD:ADB3278
Aleiodes alisundermieri|[520]|BIOUG29288-B08|Malaise trap PL12-3A|594[0n]|BOLD:ADB3278
Aleiodes alisundermieri|[521]|BIOUG51721-C06|Malaise trap PL12-3C|652[0n]|BOLD:ADB3278
Aleiodes alisundermieri|[522]|BIOUG29288-A10|Malaise trap PL12-3A|588[0n]|BOLD:ADB3278
Aleiodes alisundermieri|[523]|BIOUG29481-G05|Malaise trap PL12-3A|588[0n]|BOLD:ADB3278
Aleiodes normwoodleyi|[524]|DHJPAR0030719|Dysschema viuda|613[3n]|BOLD:AAJ4076
Aleiodes bobandersoni|[525]|DHJPAR0051160|Malocampa piratica|657[0n]|BOLD:AAA5372
Aleiodes bobandersoni|[526]|DHJPAR0062156|Malocampa piratica|618[0n]|BOLD:AAA5372
Aleiodes bobandersoni|[527]|DHJPAR0063998|Malocampa matralis|621[0n]|BOLD:AAA5372
Aleiodes bobandersoni|[528]|DHJPAR0062172|Malocampa 08-30689|619[0n]|BOLD:AAA5372
Aleiodes bobandersoni|[529]|DHJPAR0029043|Malocampa matralis|657[0n]|BOLD:AAA5372
Aleiodes bobandersoni|[530]|DHJPAR0052873|Malocampa matralis|658[0n]|BOLD:AAA5372
Aleiodes Janzen12|[531]|DHJPAR0045817|Elymiotis longara|274[0n]
Aleiodes steveashei|[532]|DHJPAR0021180|Lysana Janzen02|660[0n]|BOLD:AAJ4092
Aleiodes steveashei|[533]|DHJPAR0062073|Elymiotis plechelm|658[0n]|BOLD:AAJ4092
Aleiodes steveashei|[534]|DHJPAR0047201|Tachuda discretaDHJ01|658[0n]|BOLD:AAJ4092
Aleiodes steveashei|[535]|DHJPAR0040461|Tachuda discretaDHJ01|658[0n]|BOLD:AAJ4092
Aleiodes steveashei|[536]|DHJPAR0062081|Elymiotis plechelm|658[0n]|BOLD:AAJ4092
Aleiodes steveashei|[537]|DHJPAR0062112|Elymiotis plechelm|658[0n]|BOLD:AAJ4092
Aleiodes terryerwini|[538]|DHJPAR0061494|Paectes Poole10|658[0n]|BOLD:ADJ0647
Aleiodes Janzen3995|[539]|DHJPAR0063995|Anomis luridula|612[0n]|BOLD:AEF3944
Aleiodes sp. ALW-2011|[540]|DHJPAR0063970|Anomis luridula|658[0n]|BOLD:AEF3944
Aleiodes pauljohnsoni|[541]|DHJPAR0054923|Erosia veninotata|658[0n]|BOLD:ACM2562
Aleiodes pauljohnsoni|[542]|DHJPAR0054919|Erosia veninotata|658[0n]|BOLD:ACM2562
Aleiodes alvaroumanai|[543]|BIOUG53725-C02|Malaise trap PL12-3D|654[0n]|BOLD:AAM1443
Aleiodes alvaroumanai|[544]|BIOUG52707-C11|Malaise trap PL12-9C|655[0n]|BOLD:AAM1443
Aleiodes alvaroumanai|[545]|DHJPAR0038019|Syngamia florella|658[0n]|BOLD:AAM1443
Aleiodes alvaroumanai|[546]|BIOUG29092-H10|Malaise trap PL12-6A|600[0n]|BOLD:AAM1443
Aleiodes alvaroumanai|[547]|DHJPAR0038017|Syngamia florella|658[0n]|BOLD:AAM1443
Aleiodes johnkingsolveri|[548]|DHJPAR0040450|Chloropteryx BioLep266|658[0n]|BOLD:AAT8850
Aleiodes johnchemsaki|[549]|DHJPAR0045345|Nemoria anae|619[0n]|BOLD:ABX5740
Aleiodes adrianforsythi|[550]|DHJPAR0061499|Eupithecia Janzen774|658[0n]|BOLD:ADJ1086
Aleiodes frankhovori|[551]|DHJPAR0040077|Sabulodes arge|421[0n]
Aleiodes Janzen41|[552]|DHJPAR0040080|Sabulodes arge|421[0n]
Aleiodes frankhovorei|[553]|DHJPAR0040076|Sabulodes arge|616[0n]|BOLD:AAM5640
Aleiodes willsflowersi|[554]|DHJPAR0037966|Antaeotricha Janzen730|658[0n]|BOLD:AAM1704
Aleiodes carolinagodoyae|[555]|DHJPAR0023697|Pleuroprucha rudimentaria|640[0n]|BOLD:AAW1567
Aleiodes rosewarnerae|[556]|DHJPAR0023524|Pleuroprucha rudimentaria|657[0n]|BOLD:AAW1573
Aleiodes rosewarnerae|[557]|DHJPAR0023522|Pleuroprucha rudimentaria|660[0n]|BOLD:AAW1573
Aleiodes Janzen4526|[558]|DHJPAR0064526|Semaeopus illimitata|658[0n]|BOLD:AEB1336
Aleiodes michelledsouzae|[559]|DHJPAR0042782|Yidalpta auragalis|617[0n]|BOLD:ABA7287
Aleiodes Janzen3992|[560]|DHJPAR0063992|geoJanzen01 19-SRNP-20029|658[0n]|BOLD:AAH8707
Aleiodes Janzen3992|[561]|DHJPAR0064524|geoJanzen01 19-SRNP-20029|658[0n]|BOLD:AAH8707
Aleiodes Janzen3992|[562]|DHJPAR0063994|geoJanzen01 19-SRNP-20029|658[0n]|BOLD:AAH8707
Aleiodes Janzen3992|[563]|DHJPAR0064525|geoJanzen01 19-SRNP-20029|658[0n]|BOLD:AAH8707
Aleiodes Janzen3992|[564]|DHJPAR0064609|geoJanzen01 19-SRNP-20029|658[0n]|BOLD:AAH8707
Aleiodes shakirae|[565]|DHJPAR0063965|Isochromodes carbina|658[0n]|BOLD:AEF4695
Aleiodes davefurthi|[566]|DHJPAR0063445|Eubaphe conformis|658[0n]|BOLD:ACB2701
Aleiodes davefurthi|[567]|DHJPAR0049349|Herbita medonaDHJ02|658[0n]|BOLD:ACB2701
Aleiodes davefurthi|[568]|DHJPAR0064472|Herbita medonaDHJ02|658[0n]|BOLD:ACB2701
Aleiodes davefurthi|[569]|DHJPAR0061881|Simopteryx torquatariaDHJ02|658[0n]|BOLD:ACB2701
Aleiodes alejandromasisi|[570]|BIOUG05466-G08|BSE Malaise Trap|658[0n]|BOLD:ACG6844
Aleiodes Janzen4521|[571]|DHJPAR0064521|geoJanzen01 Janzen7158|658[0n]|BOLD:AAV7490
Aleiodes Janzen1913|[572]|DHJPAR0064517|Herbita medama|658[0n]|BOLD:AEB1913
Aleiodes Janzen1913|[573]|DHJPAR0064001|geoJanzen01 Janzen881|613[0n]|BOLD:AEB1913
Aleiodes alexsmithi|[574]|DHJPAR0062173|Holochroa ochra|658[0n]|BOLD:AAV7512
Aleiodes annhowdenae|[575]|DHJPAR0053665|Eutomopepla artena|655[0n]|BOLD:AAM5664
Aleiodes mikeiviei|[576]|DHJPAR0056334|arctJanzen01 Janzen71434|641[0n]|BOLD:ACS9594
Aleiodes agnespeelleae|[577]|BIOUG29423-E01|Malaise trap PL12-3A|585[0n]|BOLD:AAH8705
Aleiodes Janzen2985|[578]|DHJPAR0065341|Isogona natatrix|647[0n]|BOLD:AEB2985
Aleiodes Janzen2985|[579]|DHJPAR0064529|Isogona Poole07|658[0n]|BOLD:AEB2985
Aleiodes angelsolisi|[580]|DHJPAR0038456|Nicetas Poole11|658[0n]|BOLD:AAM1722
Aleiodes Malaise5673|[581]|BIOUG20202-G10|ESG Malaise Trap|576[0n]|BOLD:AAM5673
Aleiodes alanhalevii|[582]|BIOUG44437-G11|Malaise trap PL12-1B|652[0n]|BOLD:AAM5683
Aleiodes alanhalevii|[583]|BIOUG28817-F08|Malaise trap PL12-7A|564[0n]|BOLD:AAM5683
Aleiodes alanhalevii|[584]|BIOUG29646-B02|Malaise trap PL12-3A|471[1n]|BOLD:AAM5683

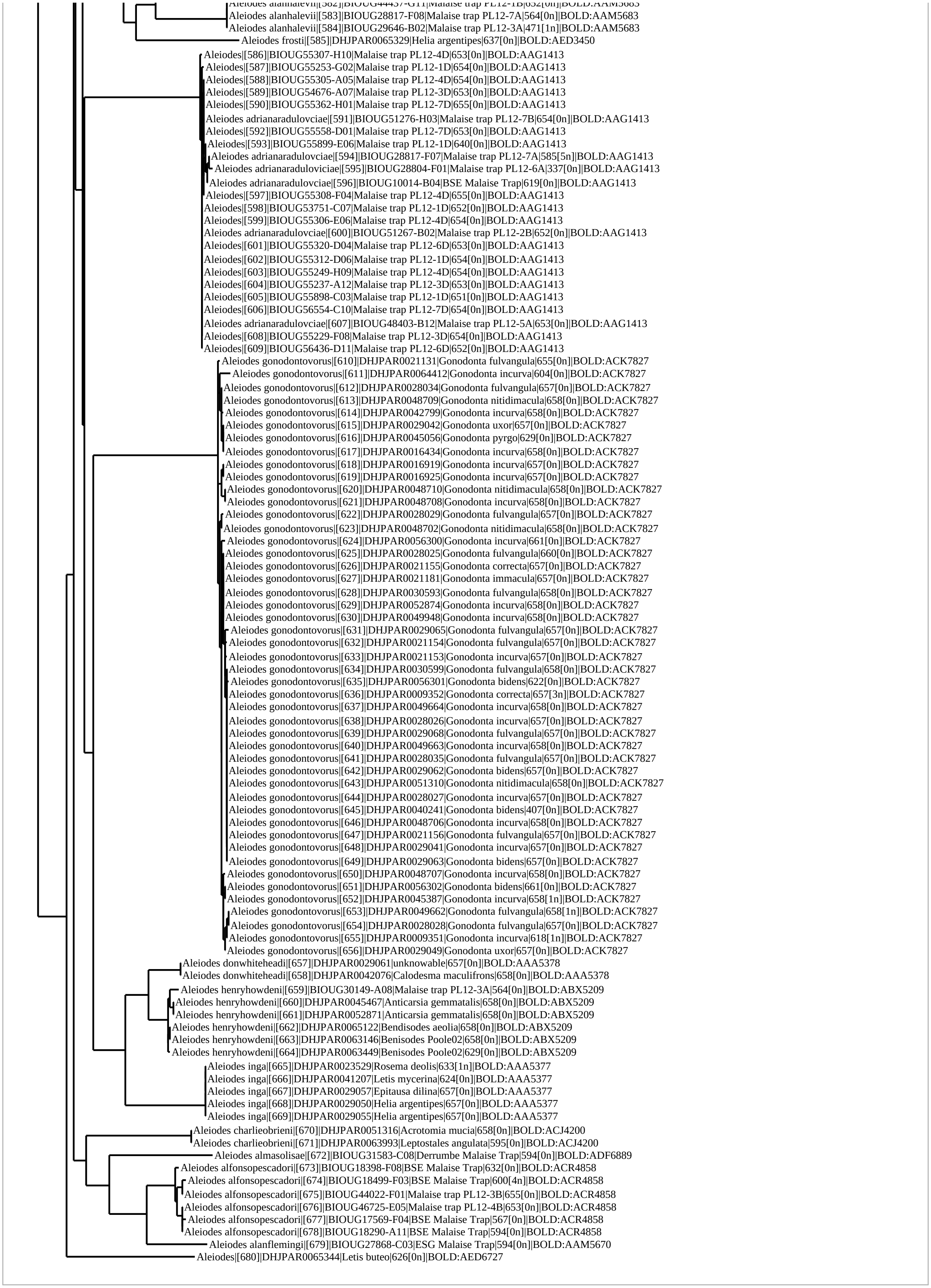
Aleiodes alanhalevii|[582]|BIOUG44437-G11|Malaise trap PL12-1B|652[0n]|BOLD:AAM5683
Aleiodes alanhalevii|[583]|BIOUG28817-F08|Malaise trap PL12-7A|564[0n]|BOLD:AAM5683
Aleiodes alanhalevii|[584]|BIOUG29646-B02|Malaise trap PL12-3A|471[1n]|BOLD:AAM5683
Aleiodes frosti|[585]|DHJPAR0065329|Helia argentipes|637[0n]|BOLD:AED3450
Aleiodes|[586]|BIOUG55307-H10|Malaise trap PL12-4D|653[0n]|BOLD:AAG1413
Aleiodes|[587]|BIOUG55253-G02|Malaise trap PL12-1D|654[0n]|BOLD:AAG1413
Aleiodes|[588]|BIOUG55305-A05|Malaise trap PL12-4D|654[0n]|BOLD:AAG1413
Aleiodes|[589]|BIOUG54676-A07|Malaise trap PL12-3D|653[0n]|BOLD:AAG1413
Aleiodes|[590]|BIOUG55362-H01|Malaise trap PL12-7D|655[0n]|BOLD:AAG1413
Aleiodes adrianaradulovciae|[591]|BIOUG51276-H03|Malaise trap PL12-7B|654[0n]|BOLD:AAG1413
Aleiodes|[592]|BIOUG55558-D01|Malaise trap PL12-7D|653[0n]|BOLD:AAG1413
Aleiodes|[593]|BIOUG55899-E06|Malaise trap PL12-1D|640[0n]|BOLD:AAG1413
Aleiodes adrianaradulovciae|[594]|BIOUG28817-F07|Malaise trap PL12-7A|585[5n]|BOLD:AAG1413
Aleiodes adrianaraduloviciae|[595]|BIOUG28804-F01|Malaise trap PL12-6A|337[0n]|BOLD:AAG1413
Aleiodes adrianaradulovciae|[596]|BIOUG10014-B04|BSE Malaise Trap|619[0n]|BOLD:AAG1413
Aleiodes|[597]|BIOUG55308-F04|Malaise trap PL12-4D|655[0n]|BOLD:AAG1413
Aleiodes|[598]|BIOUG53751-C07|Malaise trap PL12-1D|652[0n]|BOLD:AAG1413
Aleiodes|[599]|BIOUG55306-E06|Malaise trap PL12-4D|654[0n]|BOLD:AAG1413
Aleiodes adrianaradulovciae|[600]|BIOUG51267-B02|Malaise trap PL12-2B|652[0n]|BOLD:AAG1413
Aleiodes|[601]|BIOUG55320-D04|Malaise trap PL12-6D|653[0n]|BOLD:AAG1413
Aleiodes|[602]|BIOUG55312-D06|Malaise trap PL12-1D|654[0n]|BOLD:AAG1413
Aleiodes|[603]|BIOUG55249-H09|Malaise trap PL12-4D|654[0n]|BOLD:AAG1413
Aleiodes|[604]|BIOUG55237-A12|Malaise trap PL12-3D|653[0n]|BOLD:AAG1413
Aleiodes|[605]|BIOUG55898-C03|Malaise trap PL12-1D|651[0n]|BOLD:AAG1413
Aleiodes|[606]|BIOUG56554-C10|Malaise trap PL12-7D|654[0n]|BOLD:AAG1413
Aleiodes adrianaradulovciae|[607]|BIOUG48403-B12|Malaise trap PL12-5A|653[0n]|BOLD:AAG1413
Aleiodes|[608]|BIOUG55229-F08|Malaise trap PL12-3D|654[0n]|BOLD:AAG1413
Aleiodes|[609]|BIOUG56436-D11|Malaise trap PL12-6D|652[0n]|BOLD:AAG1413
Aleiodes gonodontovorus|[610]|DHJPAR0021131|Gonodonta fulvangula|655[0n]|BOLD:ACK7827
Aleiodes gonodontovorus|[611]|DHJPAR0064412|Gonodonta incurva|604[0n]|BOLD:ACK7827
Aleiodes gonodontovorus|[612]|DHJPAR0028034|Gonodonta fulvangula|657[0n]|BOLD:ACK7827
Aleiodes gonodontovorus|[613]|DHJPAR0048709|Gonodonta nitidimacula|658[0n]|BOLD:ACK7827
Aleiodes gonodontovorus|[614]|DHJPAR0042799|Gonodonta incurva|658[0n]|BOLD:ACK7827
Aleiodes gonodontovorus|[615]|DHJPAR0029042|Gonodonta uxor|657[0n]|BOLD:ACK7827
Aleiodes gonodontovorus|[616]|DHJPAR0045056|Gonodonta pyrgo|629[0n]|BOLD:ACK7827
Aleiodes gonodontovorus|[617]|DHJPAR0016434|Gonodonta incurva|658[0n]|BOLD:ACK7827
Aleiodes gonodontovorus|[618]|DHJPAR0016919|Gonodonta incurva|657[0n]|BOLD:ACK7827
Aleiodes gonodontovorus|[619]|DHJPAR0016925|Gonodonta incurva|657[0n]|BOLD:ACK7827
Aleiodes gonodontovorus|[620]|DHJPAR0048710|Gonodonta nitidimacula|658[0n]|BOLD:ACK7827
Aleiodes gonodontovorus|[621]|DHJPAR0048708|Gonodonta incurva|658[0n]|BOLD:ACK7827
Aleiodes gonodontovorus|[622]|DHJPAR0028029|Gonodonta fulvangula|657[0n]|BOLD:ACK7827
Aleiodes gonodontovorus|[623]|DHJPAR0048702|Gonodonta nitidimacula|658[0n]|BOLD:ACK7827
Aleiodes gonodontovorus|[624]|DHJPAR0056300|Gonodonta incurva|661[0n]|BOLD:ACK7827
Aleiodes gonodontovorus|[625]|DHJPAR0028025|Gonodonta fulvangula|660[0n]|BOLD:ACK7827
Aleiodes gonodontovorus|[626]|DHJPAR0021155|Gonodonta correcta|657[0n]|BOLD:ACK7827
Aleiodes gonodontovorus|[627]|DHJPAR0021181|Gonodonta immacula|657[0n]|BOLD:ACK7827
Aleiodes gonodontovorus|[628]|DHJPAR0030593|Gonodonta fulvangula|658[0n]|BOLD:ACK7827
Aleiodes gonodontovorus|[629]|DHJPAR0052874|Gonodonta incurva|658[0n]|BOLD:ACK7827
Aleiodes gonodontovorus|[630]|DHJPAR0049948|Gonodonta incurva|658[0n]|BOLD:ACK7827
Aleiodes gonodontovorus|[631]|DHJPAR0029065|Gonodonta fulvangula|657[0n]|BOLD:ACK7827
Aleiodes gonodontovorus|[632]|DHJPAR0021154|Gonodonta fulvangula|657[0n]|BOLD:ACK7827
Aleiodes gonodontovorus|[633]|DHJPAR0021153|Gonodonta incurva|657[0n]|BOLD:ACK7827
Aleiodes gonodontovorus|[634]|DHJPAR0030599|Gonodonta fulvangula|658[0n]|BOLD:ACK7827
Aleiodes gonodontovorus|[635]|DHJPAR0056301|Gonodonta bidens|622[0n]|BOLD:ACK7827
Aleiodes gonodontovorus|[636]|DHJPAR0009352|Gonodonta correcta|657[3n]|BOLD:ACK7827
Aleiodes gonodontovorus|[637]|DHJPAR0049664|Gonodonta incurva|658[0n]|BOLD:ACK7827
Aleiodes gonodontovorus|[638]|DHJPAR0028026|Gonodonta incurva|657[0n]|BOLD:ACK7827
Aleiodes gonodontovorus|[639]|DHJPAR0029068|Gonodonta fulvangula|657[0n]|BOLD:ACK7827
Aleiodes gonodontovorus|[640]|DHJPAR0049663|Gonodonta incurva|658[0n]|BOLD:ACK7827
Aleiodes gonodontovorus|[641]|DHJPAR0028035|Gonodonta fulvangula|657[0n]|BOLD:ACK7827
Aleiodes gonodontovorus|[642]|DHJPAR0029062|Gonodonta bidens|657[0n]|BOLD:ACK7827
Aleiodes gonodontovorus|[643]|DHJPAR0051310|Gonodonta nitidimacula|658[0n]|BOLD:ACK7827
Aleiodes gonodontovorus|[644]|DHJPAR0028027|Gonodonta incurva|657[0n]|BOLD:ACK7827
Aleiodes gonodontovorus|[645]|DHJPAR0040241|Gonodonta bidens|407[0n]|BOLD:ACK7827
Aleiodes gonodontovorus|[646]|DHJPAR0048706|Gonodonta incurva|658[0n]|BOLD:ACK7827
Aleiodes gonodontovorus|[647]|DHJPAR0021156|Gonodonta fulvangula|657[0n]|BOLD:ACK7827
Aleiodes gonodontovorus|[648]|DHJPAR0029041|Gonodonta incurva|657[0n]|BOLD:ACK7827
Aleiodes gonodontovorus|[649]|DHJPAR0029063|Gonodonta bidens|657[0n]|BOLD:ACK7827
Aleiodes gonodontovorus|[650]|DHJPAR0048707|Gonodonta incurva|658[0n]|BOLD:ACK7827
Aleiodes gonodontovorus|[651]|DHJPAR0056302|Gonodonta bidens|661[0n]|BOLD:ACK7827
Aleiodes gonodontovorus|[652]|DHJPAR0045387|Gonodonta incurva|658[1n]|BOLD:ACK7827
Aleiodes gonodontovorus|[653]|DHJPAR0049662|Gonodonta fulvangula|658[1n]|BOLD:ACK7827
Aleiodes gonodontovorus|[654]|DHJPAR0028028|Gonodonta fulvangula|657[0n]|BOLD:ACK7827
Aleiodes gonodontovorus|[655]|DHJPAR0009351|Gonodonta incurva|618[1n]|BOLD:ACK7827
Aleiodes gonodontovorus|[656]|DHJPAR0029049|Gonodonta uxor|657[0n]|BOLD:ACK7827
Aleiodes donwhiteheadi|[657]|DHJPAR0029061|unknowable|657[0n]|BOLD:AAA5378
Aleiodes donwhiteheadi|[658]|DHJPAR0042076|Calodesma maculifrons|658[0n]|BOLD:AAA5378
Aleiodes henryhowdeni|[659]|BIOUG30149-A08|Malaise trap PL12-3A|564[0n]|BOLD:ABX5209
Aleiodes henryhowdeni|[660]|DHJPAR0045467|Anticarsia gemmatalis|658[0n]|BOLD:ABX5209
Aleiodes henryhowdeni|[661]|DHJPAR0052871|Anticarsia gemmatalis|658[0n]|BOLD:ABX5209
Aleiodes henryhowdeni|[662]|DHJPAR0065122|Bendisodes aeolia|658[0n]|BOLD:ABX5209
Aleiodes henryhowdeni|[663]|DHJPAR0063146|Benisodes Poole02|658[0n]|BOLD:ABX5209
Aleiodes henryhowdeni|[664]|DHJPAR0063449|Benisodes Poole02|629[0n]|BOLD:ABX5209
Aleiodes inga|[665]|DHJPAR0023529|Rosema deolis|633[1n]|BOLD:AAA5377
Aleiodes inga|[666]|DHJPAR0041207|Letis mycerina|624[0n]|BOLD:AAA5377
Aleiodes inga|[667]|DHJPAR0029057|Epitausa dilina|657[0n]|BOLD:AAA5377
Aleiodes inga|[668]|DHJPAR0029050|Helia argentipes|657[0n]|BOLD:AAA5377
Aleiodes inga|[669]|DHJPAR0029055|Helia argentipes|657[0n]|BOLD:AAA5377
Aleiodes charlieobrieni|[670]|DHJPAR0051316|Acrotomia mucia|658[0n]|BOLD:ACJ4200
Aleiodes charlieobrieni|[671]|DHJPAR0063993|Leptostales angulata|595[0n]|BOLD:ACJ4200
Aleiodes almasolisae|[672]|BIOUG31583-C08|Derrumbe Malaise Trap|594[0n]|BOLD:ADF6889
Aleiodes alfonsopescadori|[673]|BIOUG18398-F08|BSE Malaise Trap|632[0n]|BOLD:ACR4858
Aleiodes alfonsopescadori|[674]|BIOUG18499-F03|BSE Malaise Trap|600[4n]|BOLD:ACR4858
Aleiodes alfonsopescadori|[675]|BIOUG44022-F01|Malaise trap PL12-3B|655[0n]|BOLD:ACR4858
Aleiodes alfonsopescadori|[676]|BIOUG46725-E05|Malaise trap PL12-4B|653[0n]|BOLD:ACR4858
Aleiodes alfonsopescadori|[677]|BIOUG17569-F04|BSE Malaise Trap|567[0n]|BOLD:ACR4858
Aleiodes alfonsopescadori|[678]|BIOUG18290-A11|BSE Malaise Trap|594[0n]|BOLD:ACR4858
Aleiodes alanflemingi|[679]|BIOUG27868-C03|ESG Malaise Trap|594[0n]|BOLD:AAM5670
Aleiodes|[680]|DHJPAR0065344|Letis buteo|626[0n]|BOLD:AED6727
